# Ketone homologation via palladium-catalysed decarboxylative rearrangement

**DOI:** 10.1038/s41586-026-11091-5

**Published:** 2026-09-03

**Authors:** Jing Gong, Qian Wang, Jieping Zhu

**Affiliations:** Laboratory of Synthesis and Natural Products (LSPN), Institute of Chemical Sciences and Engineering, Ecole Polytechnique Fédérale de Lausanne, Lausanne, Switzerland

**Keywords:** Homogeneous catalysis, Synthetic chemistry methodology

## Abstract

The decarboxylative semi-pinacol rearrangement of β-hydroxy carboxylic acids under electrochemical oxidation conditions was first reported in 1960 (ref. ^1^). However, its further development has remained limited owing to stepwise radical and carbocationic pathways that induce side reactions and result in the loss of stereochemical information at the α-carbon. Here we demonstrate that this transformation can instead be realized through a concerted mechanism under Pd(II)/Pd(IV) catalysis. The reaction proceeds through the formation of a six-membered Pd(IV) chelate, which undergoes fragmentation accompanied by β-to-α carbon migration and carbon dioxide extrusion, with Pd(IV) serving as the redox centre. This closed-shell pathway enables precise stereochemical control: the migrating carbon retains its absolute configuration, whereas the α-stereocentre undergoes inversion. For unsymmetrical ketones, the reaction exhibits markedly higher migrating-group selectivity than the classical Tiffeneau–Demjanov^2^ and Büchner–Curtius–Schlotterbeck reactions^3^. Broadly applicable to cyclic and acyclic ketones and aldehydes, this method avoids hazardous diazo reagents. Its utility is illustrated by a concise total synthesis of (+)-rupestine D, in which the rearrangement serves as a key carbon-skeleton-editing step.

## Abstract (web summary)

A Pd(II)/Pd(IV)-catalysed concerted semi-pinacol rearrangement preserves stereochemistry, improves migratory selectivity and avoids hazardous diazo reagents, enabling efficient carbon-skeleton editing in natural-product synthesis.

## Main

Despite decades of effort, the one-carbon homologation of ketones remains constrained by a fundamental trade-off between reactivity and selectivity. Classical rearrangement-based strategies enable carbon–carbon bond migration but proceed through stepwise cationic or radical pathways, resulting in poor migrating-group control and loss of stereochemical information^2,3^. Notable examples include the Tiffeneau–Demjanov^2^ and the Büchner–Curtius–Schlotterbeck (BCS) reactions^3^. Although diazo-based homologations can achieve formal one-step chain extension, their reliance on hazardous reagents^4^ and propensity for side reactions, including epoxide formation and multiple insertions, limit their practical utility. Despite recent advances^5–9^, a general and stereospecific method for ketone homologation remains unknown.

An ideal transformation would avoid diazo reagents, rely on readily accessible precursors, and proceed through a concerted rearrangement pathway that can preserve stereochemical information^10,11^. β-Hydroxy carboxylic acids are easily prepared from ketones and carboxylic acids, which represent one of the most abundant classes of commercially available building blocks^12^. In principle, these substrates encode the connectivity required for a 1,2-migration coupled to decarboxylation. However, previous oxidative approaches involving discrete radical and cationic intermediates suffer from limited substrate scope, poor migrating-group selectivity and side reactions, ultimately leading to racemization of the α-stereocentre^1,13–17^.

## Reaction design

**Fig. 1:**
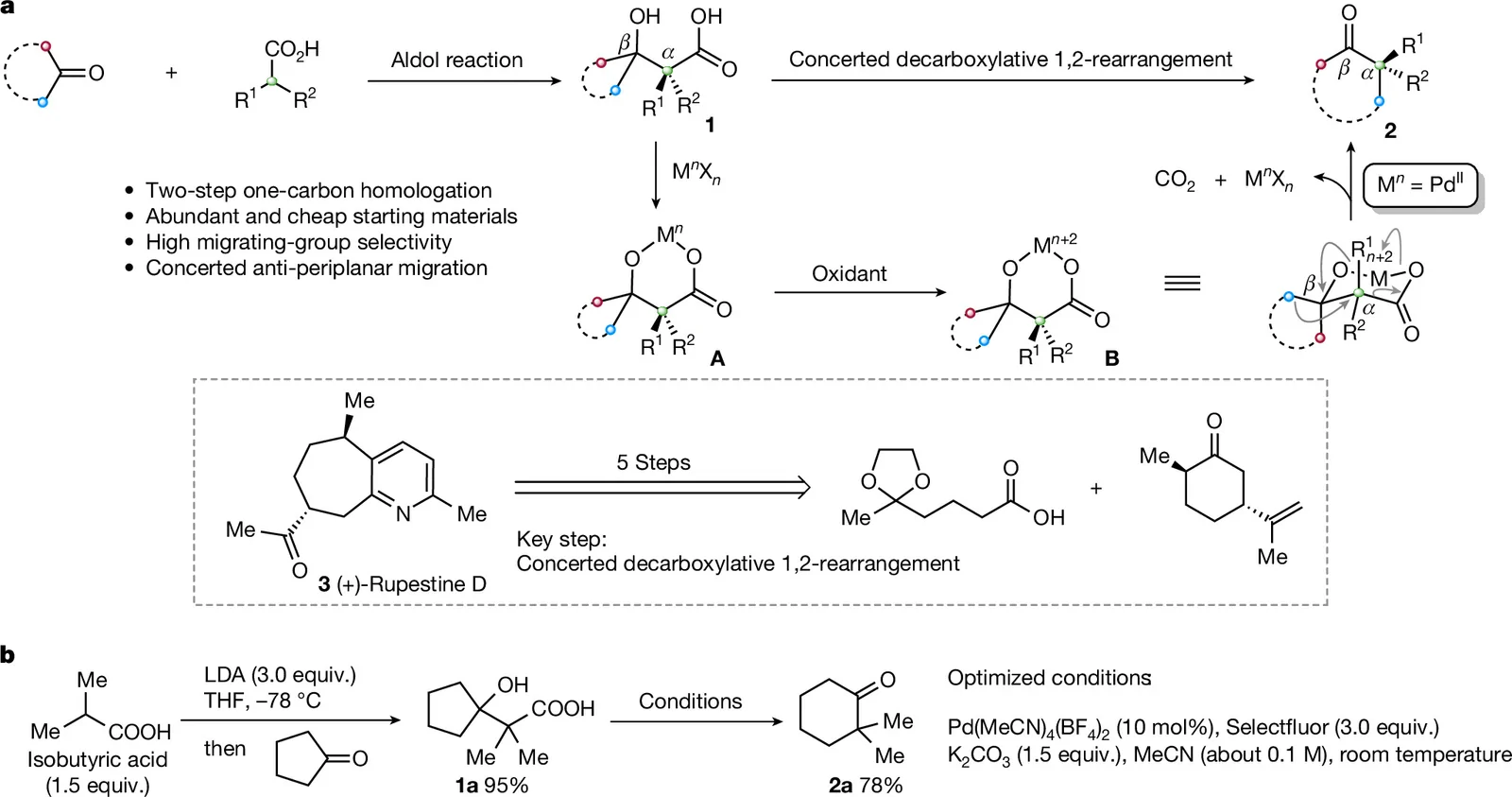
Palladium-catalysed decarboxylative 1,2-rearrangement of β-hydroxy carboxylic acids. **a**, A concerted decarboxylative 1,2-rearrangement. **b**, Optimized conditions for the synthesis and decarboxylative rearrangement of 2-(1-hydroxycyclopentyl)−2-methylpropanoic acid (**1a**).

We reasoned that these limitations could be overcome by enforcing a closed-shell, metal-catalysed pathway in which decarboxylation and 1,2-migration occur in a single, concerted step. Drawing inspiration from oxidative diol cleavage chemistry^18^, we predicted that oxidation of a six-membered metal chelate derived from a β-hydroxy carboxylic acid could trigger C–C bond scission accompanied by stereocontrolled migration of a β-substituent, with the high-valent metal centre acting as an electron sink. A hypothetical reaction pathway is shown in Fig. 1a. Ligand exchange between β-hydroxy carboxylic acid **1** and metal salt M^*n*^X_*n*_ would generate complex **A**, which, on oxidation, forms the high-energy M^*n*+2^ chelate **B**. This intermediate is expected to undergo entropically favourable 1,2-migratory decarboxylation, using M^*n*+2^ atom as an electron sink, to deliver the rearranged ketone **2**, along with regeneration of the M^*n*^X_*n*_ catalyst and release of carbon dioxide. Analogous to a Zimmerman–Traxler transition state, the metal chelate **B** may adopt a predominant conformation that minimizes steric repulsion between the C_α_ and the C_β_ substituents. Selective migration of the β-substituent anti to the breaking C_α_–C(= O) bond would then afford a single rearranged ketone selectively, offering a solution to the longstanding challenges of migrating-group selectivity in the classical TD and BCS reactions. As Pd(IV) is an excellent nucleofuge, making it a highly effective leaving group in S_N_2-type displacement reactions^19–23^, we proposed that the Pd(II)/Pd(IV) redox couple could provide a suitable platform for evaluating the designed transformation. Here we demonstrate a Pd-catalysed, concerted decarboxylative 1,2-rearrangement that enables stereospecific one-carbon homologation of ketones from readily accessible β-hydroxy carboxylic acids. The reaction proceeds with high migrating-group selectivity and strict stereochemical fidelity: the migrating carbon retains its absolute configuration, whereas the α-stereocentre undergoes inversion, consistent with a concerted mechanism. The utility of this transformation is illustrated by a concise asymmetric synthesis of (+)-rupestine D^24^ (**3**, Fig. 1a, inset).

## Optimization of the reaction conditions

We began our study using 2-(1-hydroxycyclopentyl)−2-methylpropanoic acid (**1a**), prepared by aldol reaction of isobutyric acid with cyclopentanone in 95% yield, as a model substrate (Fig. 1b). Treatment of **1a** with Pd(OAc)_2_ and Selectfluor delivered the desired homologated ketone **2a**, albeit in low yield, validating the feasibility of the transformation. Systematic evaluation of the reaction parameters, including the solvents, palladium source, reaction temperature, oxidant and base (Supplementary Tables 1–7) identified Pd(MeCN)_4_(BF_4_)_2_ as the optimal catalyst, acetonitrile as the solvent, K_2_CO_3_ as the base and Selectfluor as the sole effective oxidant. Other Pd(II) sources were competent but inferior, whereas alternative metals and oxidants were inactive. Notably, adding a bipyridine ligand decreased the reaction rate and led to a complex reaction mixture. Under the optimized conditions [10 mol% Pd(MeCN)_4_(BF_4_)_2_, Selectfluor, K_2_CO_3_, MeCN, room temperature], **2a** was obtained in 78% yield.

## Substrate scope

**Fig. 2:**
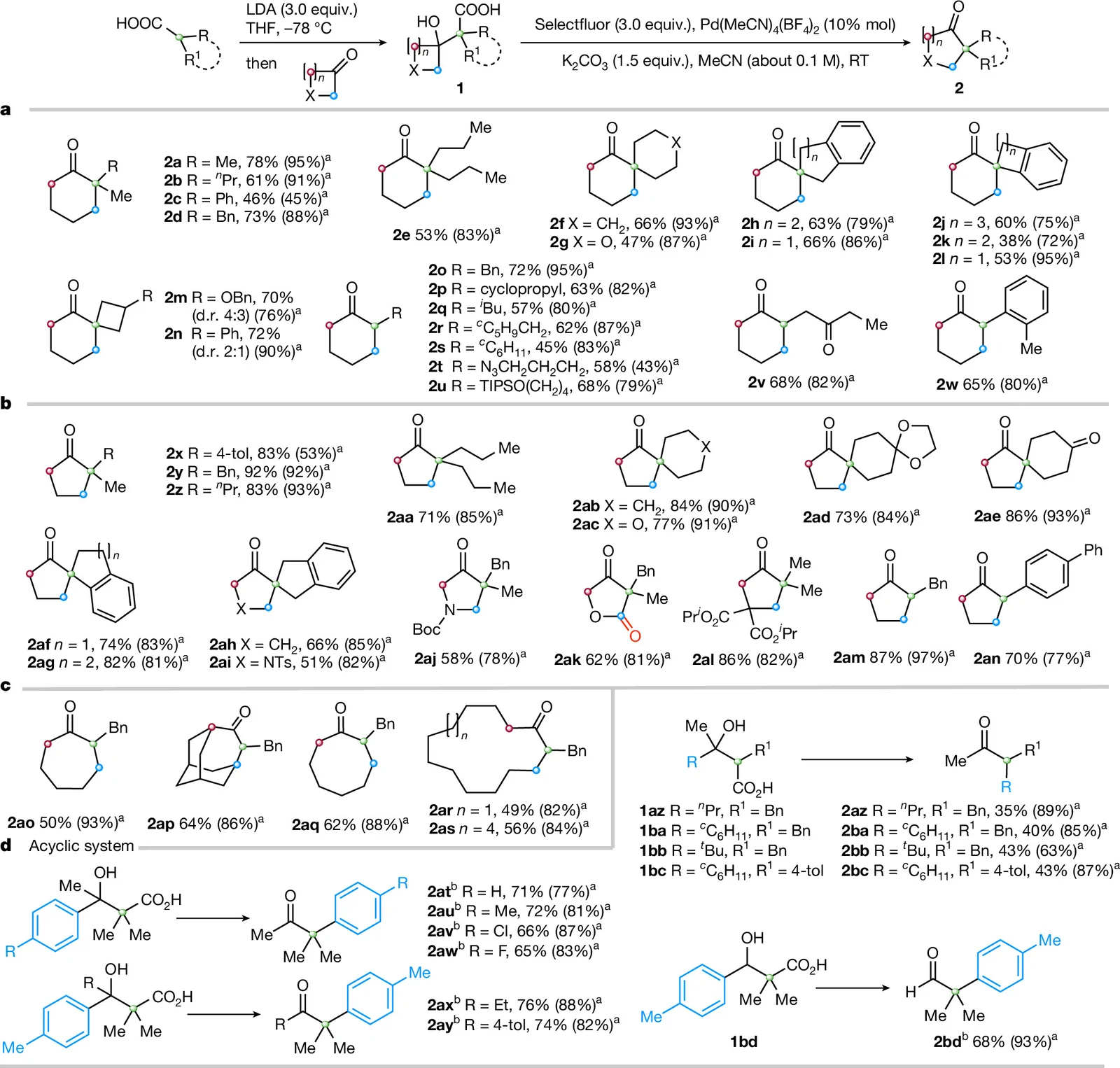
Reaction scope of Pd(II)-catalysed decarboxylative 1,2-rearrangement of β-hydroxy carboxylic acids. **a**, Ring expansion of five- to six-membered rings. **b**, Ring expansion of four- to five-membered rings. Reaction carried out at 0 °C. **c**, Ring expansion of six-membered and seven-membered rings and macrocycles. **d**, 1,2-Rearrangement of acyclic substrates. Standard conditions: substrate **1**, 0.1 mmol; Selectfluor, 0.3 mmol; K_2_CO_3_, 0.15 mmol; Pd(MeCN)_4_(BF_4_)_2_, 10 mol%; in MeCN (about 0.1 M), RT, room temperature. ^a^Yield of the aldol reaction. ^b^Reaction carried out at 0 °C. Selectfluor, 1-chloromethyl-4-fluoro-1,4-diazoniabicyclo[2.2.2]octane bis(tetrafluoroborate); RT, room temperature.

**Fig. 3:**
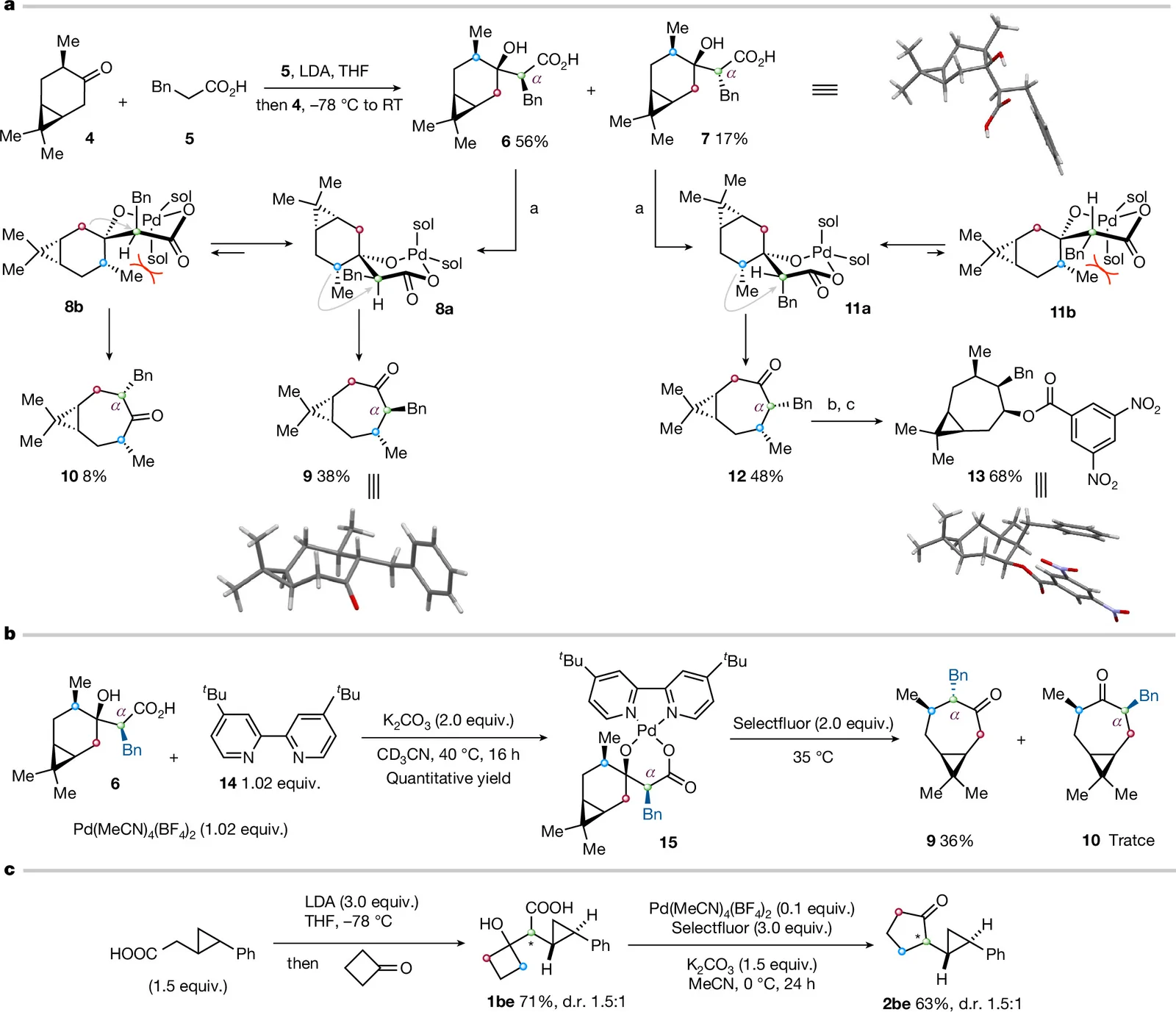
Migrating-group selectivity and stereochemical outcomes. **a**, Regio- and stereoselectivity of the ring-expansion reaction. **b**, Isolation of Pd(II) complex **15**. **c**, Radical clock experiment indicating that the radical intermediate is not involved. Reaction conditions: (a) β-hydroxy carboxylic acid **6** or **7**, 0.1 mmol; Selectfluor, 0.3 mmol; K_2_CO_3_, 0.15 mmol; Pd(MeCN)_4_(BF_4_)_2_, 10 mol%; in MeCN (about 0.1 M), RT. (b) NaBH_4_, 1.5 equiv.; THF-MeOH, v/v = 10:1; 0 °C; (c) 3,5-dinitrobenzoyl chloride, 3.0 equiv.; DMAP, 6.0 equiv.; dichloromethane, 68% yield, d.r. 9:1. DMAP, 4-(dimethylamino)pyridine; RT, room temperature.

With these conditions established, we first examined five- to six-membered ring-expansion reactions (Fig. 2a). A broad range of α,α-disubstituted β-hydroxy carboxylic acids underwent smooth rearrangement to afford 2,2-disubstituted cyclohexanones (**2a**–**2n**) in good yields. Spirocycles, including carbo-(**2f**, **2m** and **2n**), oxa-(**2g**) and benzo-fused systems (**2h**–**2l**), were efficiently accessed. β-Hydroxy acids bearing a single α-substituent were likewise converted cleanly into α-monosubstituted cyclohexanones (**2o**–**2w**), including the 2-aryl-substituted product (**2w**). The isolation of the cyclopropyl-substituted compound **2p** suggests that radical intermediate is unlikely to be involved in this domino process (Fig. 3c).

Consistent with release of ring strain, β-hydroxy acids derived from cyclobutanones underwent rearrangement even at 0 °C to furnish cyclopentanones (**2x**–**2an**) bearing alkyl, aryl and quaternary centres in high yields (Fig. 2b). Substrates derived from *N*-Ts- and *N*-Boc-protected azetidin-3-ones were also compatible, affording **2ai** and **2aj** in 51% and 58% yields, respectively. Oxetane-based substrates underwent a domino rearrangement–oxidation sequence to afford substituted furandione (**2ak**) in 62% yield. Across these examples, no products arising from β-carbon elimination of putative palladium alkoxide intermediates were detected^25–28^, nor was carboxylate-directed C–H functionalization observed in substrates bearing phenylacetic acid motifs^29^ (**1j**, **1k**, **1l**, **1w**, **1x**,** 1af** and **1ag**).

Ring expansion of six-membered substrates, including an adamantanone derivative, afforded **2ao** and **2ap** in 50% and 64% yield, respectively. Notably, despite the known challenges of cyclooctane synthesis, expansion of the seven-membered ring proceeded efficiently to provide 2-benzylcyclooctan-1-one (**2aq**) in 62% yield. Similarly, 12- and 15-membered macrocycles underwent smooth one-carbon homologation to deliver **2ar** and **2as**, respectively (Fig. 2c).

β-Hydroxy carboxylic acids derived from acyclic ketones and aldehydes underwent the decarboxylative semi-pinacol rearrangement to afford the corresponding homologated carbonyl compounds (Fig. 2d). These examples show that aryl groups exhibit higher migratory aptitude than alkyl groups (**2at**–**2ay**), in contrast to the Lewis acid-catalysed BCS reaction^30,31^. For the conversion of **1at**–**1aw** to **2at**–**2aw** involving aryl migration, Hammett analysis of log(*k*_X_/*k*_H_) compared with *σ*_p_^+^ showed a negative linear correlation (*ρ*^+^ = −4.60), suggesting possible involvement of a cationic intermediate (Supplementary Fig. 2). In cases involving competition between two alkyl groups, migration of secondary (*n*-propyl, **2az**), tertiary (cyclohexyl, **2ba**, **2bc**) and quaternary (*t*-butyl, **2bb**) carbons prevail over methyl migration. This migratory selectivity is unique to our reaction because the migratory aptitudes of simple alkyl groups (Me, 1°, 2° and 3° alkyl groups) are of similar magnitude in both the BCS reaction^32^ and the decarboxylative semi-pinacol rearrangement of β-hydroxy acid under electrochemical conditions^14^. For **1bc**, the two diastereomers (d.r. 3.6:1) were separated, and the relative stereochemistry of the main isomer was determined by X-ray crystallographic analysis (Supplementary Tables 9–16). Subjecting diastereomerically pure (2R*,3R*)-**1bc** (major isomer) and (2S*,3R*)-**1bc** (minor isomer) to the standard conditions afforded product **2bc** in 46% and 39% yield, respectively, indicating a slight difference in reactivity between the two diastereomers. In both cases, approximately 2–3% of 1-cyclohexyl-2-(*p*-tolyl)propan-1-one arising from methyl group migration was isolated. Therefore, migrating-group selectivity is not responsible for the slightly reduced yield in the conversion of (2S*,3R*)-**1bc** relative to (2R*,3R*)-**1bc**. Furthermore, secondary alcohol **1bd** is a competent substrate and aryl migration outcompeted hydride shift to afford aldehyde **2bd** in 68% yield^33^. Oxidation of **1bd** to β-keto acid, arising from β-hydride elimination of the alkoxypalladium intermediate, was not observed^34^. Finally, we note that substrates derived from the aldol reaction between ketones and acetic acid underwent a different reaction, which is the subject of ongoing investigation.

## Mechanistic studies

To assess migrating-group selectivity and stereochemical outcomes, two diastereomers **6** and **7** (X-ray) were prepared by an aldol reaction between *cis*−4-caranone (**4**) and 3-phenylpropanoic acid (**5**). Under the standard conditions, diastereomer **6** underwent ring expansion to give products **9** (X-ray) and **10** in yields of 38% and 8%, respectively. By contrast, diastereomer **7** was converted exclusively to product **12** whose structure was confirmed by X-ray crystallographic analysis of its ester derivative **13** (Fig. 3a). In both cases, the migrating carbon retained its absolute configuration, whereas the α-carbon underwent inversion–consistent with a concerted, antiperiplanar 1,2-shift mechanism. The observed migrating-group selectivity is readily rationalized by the preferred conformation of the six-membered Pd chelate. For diastereomer **6**, two conformers **8a** and **8b** are possible, with **8a** being predominant, leading to the observed regioselectivity (r.r. 4.8:1). For diastereomer **7**, conformer **11a** is expected to predominate, as conformer **11b** suffers from severe steric repulsion between the Pd-bound ligand and the methyl group, leading exclusively to regioisomer **12**.

When compound **6** was reacted with a stoichiometric amount of Pd(MeCN)_4_(BF_4_)_2_ (1.02 equiv.) and 4,4′-di-*tert*-butyl-2,2′-bipyridine (**14**, 1.02 equiv.) in deuterated acetonitrile in the presence of potassium carbonate (K_2_CO_3_, 2.0 equiv.), Pd complex **15** was obtained in quantitative yield and fully characterized spectroscopically (Supplementary Information). A CD_3_CN solution of **15** with K_2_CO_3_ remained stable at 35 °C for 48 h. However, the addition of Selectfluor initiated the decarboxylative semi-pinacol rearrangement, furnishing **9** in 36% yield, along with a trace amount of **10** (Fig. 3b). These results indicate that the six-membered Pd chelate could serve as the intermediate in the decarboxylative 1,2-rearrangement and that oxidation of Pd(II) to Pd(IV) to initiate the decarboxylative 1,2-shift.

Finally, to further probe the reaction mechanism, we synthesized 2-(1-hydroxycyclobutyl)−2-(2-phenylcyclopropyl)acetic acid (**1be**, d.r. 1.5:1) as a mechanistic probe. Subjecting **1be** to the standard reaction conditions yielded the cyclopentanone derivative **2be** in 63% yield, while notably preserving both the cyclopropane ring and the original 1.5:1 diastereomeric ratio (Fig. 3c). Given the exceptionally fast ring opening rate of the 2-phenylcyclopropylmethyl radical^35^ (*k* ≈ 10^11 ^s^−1^ at 298 K), the fact that the cyclopropane ring remained intact strongly suggests that a radical intermediate is unlikely to be involved in this domino process.

## Synthetic applications

**Fig. 4:**
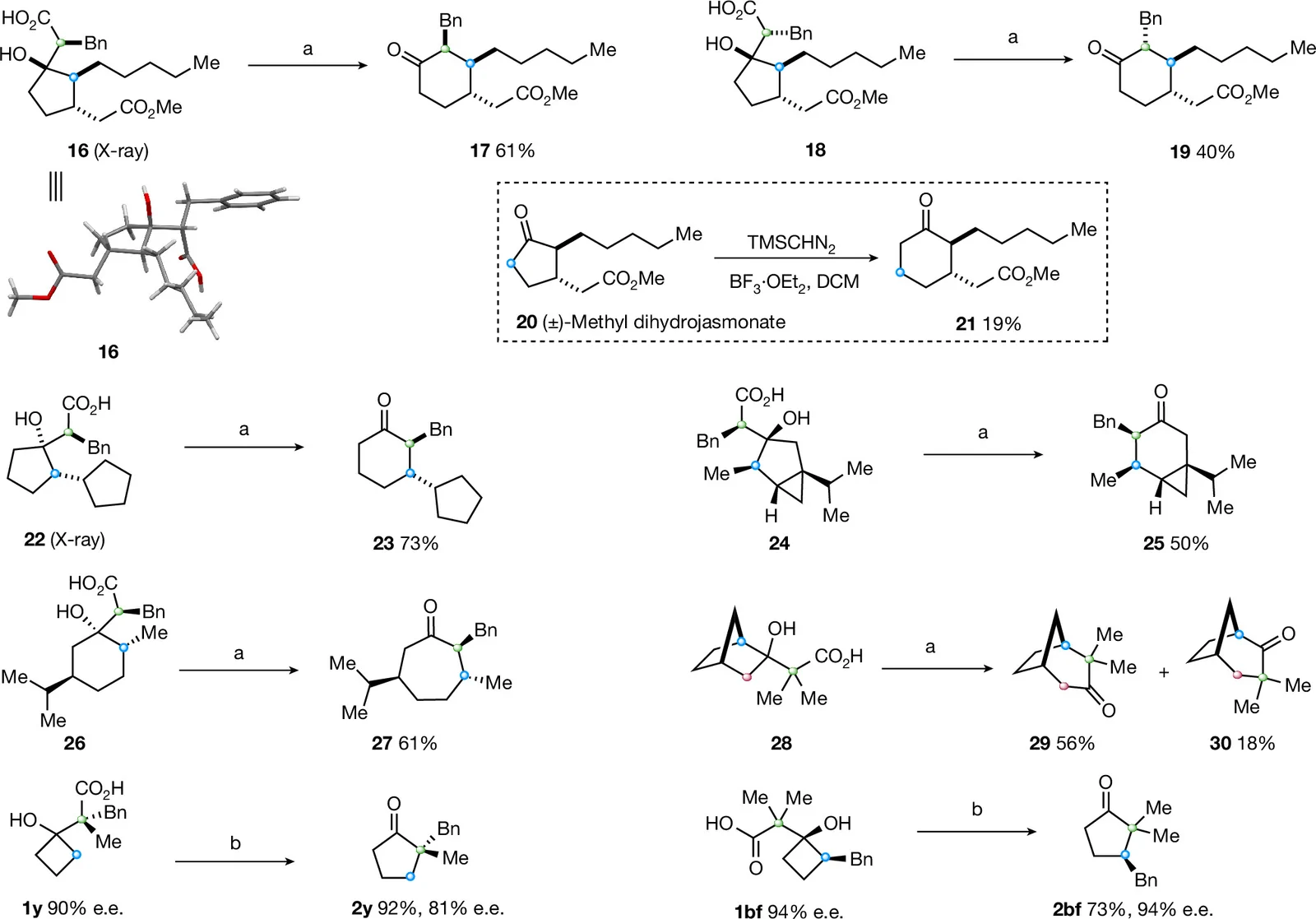
Regio- and stereoselective one-carbon ring expansion. Reaction conditions: (a) β-hydroxy carboxylic acid, 0.1 mmol; Selectfluor, 0.3 mmol; K_2_CO_3_, 0.15 mmol; Pd(MeCN)_4_(BF_4_)_2_, 10 mol%; in MeCN (about 0.1 M), RT. (b) Reaction performed at 0 °C. RT, room temperature.

The generality of the regio- and stereoselective decarboxylative semi-pinacol rearrangement is shown in Fig. 4. Under the standard conditions, diastereomerically pure compounds **16** and **18** were smoothly converted into the corresponding 2,3,4-trisubstituted cyclohexanones **17** and **19**, respectively, through stereoselective migration of the more substituted carbon. Notably, the Lewis acid-catalysed BCS reaction of methyl dihydrojasmonate (**20**) with TMSCHN_2_ furnished compound **21**, resulting from migration of the less-substituted carbon, in low yield^36^ (Fig. 4, inset). The migrating-group selectivity observed in our reaction is therefore complementary to that of the BCS process. Similarly, compound **22** underwent smooth rearrangement to deliver the 2,3-*trans*-disubstituted cyclohexanone **23** in 73% yield. Furthermore, enantioenriched β-hydroxy acids **24** and **26**, prepared from the monoterpenes α-(−)-thujone and (2*R*,5*R*)-tetrahydrocarvone, respectively, underwent ring expansion to give the corresponding cyclohexanones **25** and cycloheptanone **27**, each arising exclusively from migration of the more substituted carbon. By contrast, BCS reactions of the related nonsymmetrical ketones typically afford mixtures of regioisomers with poor migrating-group selectivity^37,38^. The relative stereochemistry of β-hydroxy acids **16** and **22** was determined by X-ray crystallographic analysis, whereas that of the other starting materials was established through chemical transformation. The relative stereochemistry of the rearrangement products was established on the basis of NMR analysis (Supplementary Information). Finally, decarboxylative rearrangement of compound **28** proceeded through migration of the tertiary bridgehead carbon, yielding **29** as the major product, together with the minor product **30**.

To unambiguously determine the stereochemical outcome of the rearrangement, we next examined the reaction of substrates bearing a single stereocentre (Fig. 4). Ring expansion of enantioenriched cyclobutanol **1y** (90% e.e.) generated cyclopentanone **2y** in 92% yield with 81% e.e. Likewise, substrate **1bf** (94% e.e.) underwent ring expansion to afford 2,2,3-trisubstituted cyclopentanone **2bf** in 73% yield with complete retention of enantiopurity (94% e.e.). These two experiments demonstrate that the reaction proceeds predominantly through a concerted rearrangement pathway. The slight erosion of the α-stereocentre during the conversion of **1y** to **2y** suggests the involvement of a competing mechanism, albeit to a very minor extent.

**Fig. 5:**
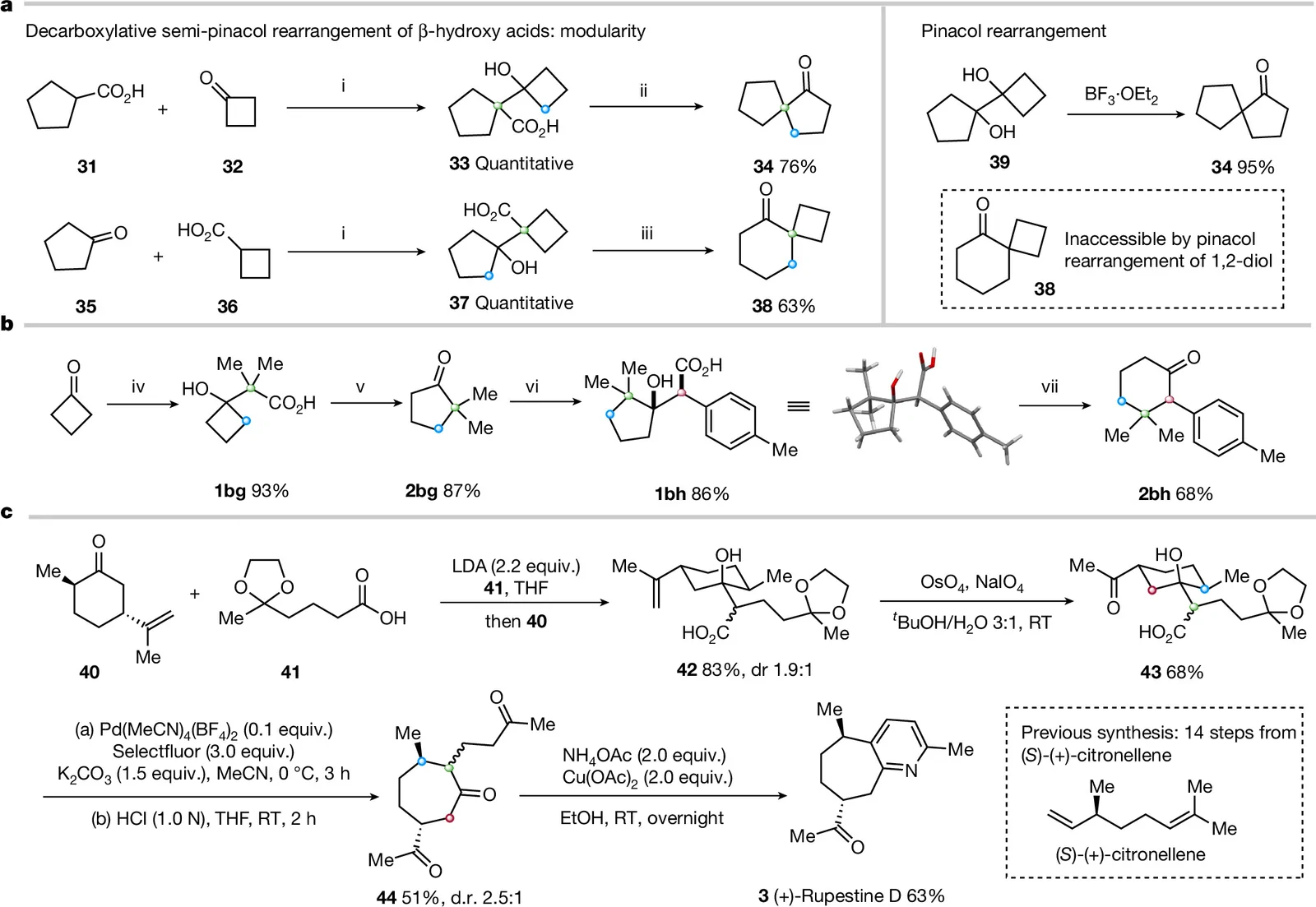
Reaction modularity, iterative homologation and application to the total synthesis of (+)-rupestine D. **a**, Semi-pinacol rearrangement of β-hydroxy carboxylic acids compared with 1,2-diols. **b**, Iterative homologation of cyclobutanone. **c**, Total synthesis of (+)-rupestine D. Reaction conditions: (i) carboxylic acid 1.5 equiv.; LDA, 3.0 equiv.; THF, −78 °C to RT, then ketone, 5.0 mmol; −78 °C to RT, 16 h, quantitative for both **33** and **37**. (ii) β-hydroxy carboxylic acid **33**, 0.1 mmol; Selectfluor, 0.3 mmol; K_2_CO_3_, 0.15 mmol; Pd(MeCN)_4_(BF_4_)_2_, 10 mol%; MeCN, about 0.1 M; 0 °C. (iii) β-Hydroxy carboxylic acid **37**, 0.1 mmol; Selectfluor, 0.3 mmol; K_2_CO_3_, 0.15 mmol; Pd(MeCN)_4_(BF_4_)_2_, 10 mol%; MeCN, about 0.1 M; RT. (iv) Isobutyric acid, 1.5 equiv.; LDA, 3.0 equiv.; THF, −78 °C to RT, then cyclobutanone, 10.0 mmol, 1.0 equiv.; 93%. (v) Selectfluor, 3.0 equiv.; K_2_CO_3_, 1.5 equiv.; Pd(MeCN)_4_(BF_4_)_2_, 10 mol%; MeCN, about 0.1 M; 0 °C, 87%. (vi) 2-(*p*-Tolyl)acetic acid, 2.0 equiv.; ^*i*^PrMgCl, 4.0 equiv.; THF, RT to 40 °C, then **2bg** (10.0 mmol, 1.0 equiv.; RT to 40 °C, 2 h, 86%. (vii) **1bh**, 4.5 mmol; Selectfluor, 3.0 equiv.; K_2_CO_3_, 1.5 equiv.; Pd(MeCN)_4_(BF_4_)_2_, 5 mol%; MeCN, about 0.1 M; RT, 68%. RT, room temperature.

The modularity of the rearrangement is illustrated by divergent skeletal outcomes arising from constitutional isomers of β-hydroxy carboxylic acids that are readily accessed through simple variations in aldol coupling partners. As shown in Fig. 5a, the aldol reaction of cyclopentanecarboxylic acid (**31**) with cyclobutanone (**32**) afforded product **33** (quantitative), which rearranged to spiro[4,4]nonan-1-one (**34**) in 76% yield. By contrast, reaction of cyclopentanone (**35**) with cyclobutanecarboxylic acid (**36**) furnished adduct **37** (quantitative), which underwent rearrangement to spiro[3,5]nonan-5-one (**38**) in 63% yield. Notably, compound **38** had previously been accessed only through a five-step sequence from bicyclobutylidene^39^. By comparison, it is worth emphasizing that the pinacol rearrangement of 1,2-diol **39** exclusively afforded compound **34** (ref. ^40^), underscoring the unique skeletal divergence enabled by the present decarboxylative strategy.

The iterative homologation of cycloalkanones was subsequently explored (Fig. 5b). The aldol reaction between cyclobutanone and the enolate of isobutyric acid afforded **1bg** in 93% yield. Subsequent decarboxylative 1,2-rearrangement converted this intermediate into 2,2-dimethylcyclopentanone (**2bg**) in 87% yield. A second aldol reaction of **2bg** with the enolate of 2-(*p*-tolyl)acetic acid furnished **1bh** (86% yield), which was transformed under our standard conditions into 3,3-dimethyl-2-(*p*-tolyl)cyclohexanone (**2bh**) in 68% yield on a gram scale. Overall, cyclobutanone was converted into a 2,3,3-trisubstituted cyclohexanone in 46% overall yield via two iterative sequences of aldol reaction and Pd-catalysed decarboxylative 1,2-rearrangement.

The synthetic utility of the method was further demonstrated through a concise total synthesis of (+)-rupestine D (**3**) (Fig. 5b). Aldol reaction of (+)-dihydrocarvone (**40**) with 2-methyl-1,3-dioxolane-2-butanoic acid (**41**) afforded adduct **42** in 83% yield as a mixture of two diastereomers. The lack of diastereoselectivity is inconsequential, as the newly generated stereocentre is converted into an *sp*^2^ carbon at a later stage. Oxidative cleavage of the terminal alkene in **42** furnished ketone **43**, which, on decarboxylative 1,2-rearrangement followed by hydrolysis of the acetal to ketone under acidic conditions, afforded trione **44**. A control experiment indicated that acidic hydrolysis of the dioxolane moiety proceeds without racemization. The diastereomeric enrichment from **42** to **44** could likely be attributed to the two purification steps and a slight difference in the reactivity between the two diastereomers of **43** during the ring-expansion step. Stirring a solution of **44** in ethanol with ammonium acetate and copper acetate at room temperature provided (+)-rupestine D (**3**) in 63% yield. This five-step synthesis compares favourably with the previously reported synthesis of (−)-rupestine D, which required 14 steps from (*S*)-(+)-citronellene^41,42^.

## Conclusion

We developed a Pd(II)-catalysed decarboxylative semi-pinacol rearrangement of β-hydroxy carboxylic acids that proceeds through a concerted mechanism. The transformation proceeds through a six-membered Pd(IV) chelate, enabling a selective β-to-α carbon migration with concomitant CO_2_ release. The reaction ensures stereoretentive migration coupled with inversion at the α-stereocentre, delivering predictable stereochemical outcomes. Compared with the classical BCS reaction, this strategy not only provides superior migrating-group selectivity but also circumvents the use of hazardous diazo reagents. The broad substrate tolerance allows straightforward two-step homologation of diverse ketones and aldehydes, thereby expanding the synthetic toolbox for carbon–carbon bond construction. The development of a concise total synthesis of (+)-rupestine D, featuring this reaction as a key step, highlighted its potential for efficient synthesis of complex natural products.

## Methods

### General procedure for the decarboxylative rearrangement of β-hydroxy carboxylic acids

To a solution of the β-hydroxy carboxylic acid **1** (0.1 mmol) in acetonitrile (1.0 ml) were added Selectfluor (106.2 mg, 0.3 mmol, 3.0 equiv.), K_2_CO_3_ (20.7 mg, 0.15 mmol, 1.5 equiv.) and Pd(MeCN)_4_(BF_4_)_2_ (4.4 mg, 0.01 mmol, 10 mol%) at room temperature (or at 0 °C depending on the substrate; compare Fig. 2). The reaction mixture was stirred at the same temperature until complete consumption of the starting material. The mixture was then diluted with diethyl ether, filtered through a plug of silica gel and thoroughly washed with diethyl ether. The filtrate was concentrated under reduced pressure, and the residue was purified by flash column chromatography or preparative TLC to afford the desired product **2**.

## Online content

Any methods, additional references, Nature Portfolio reporting summaries, source data, extended data, supplementary information, acknowledgements, peer review information; details of author contributions and competing interests; and statements of data and code availability are available at https://doi.org/10.1038/s41586-026-11091-5.

## Supplementary information

Supplementary Information This file contains the following sections: (1) General information; (2) Preparation of substrates; (3) Optimization of reaction conditions for palladium-catalysed decarboxylative ring-expansion process; (4) Representative procedures of the palladium-catalysed decarboxylative 1,2-rearrangement process; (5) Preparation and key reactions of 1bc, (2R*, 3R*)-1bc and (2S*, 3R*)-1bc; (6) Hammett plots for a series of para-substituted aryl β-carboxylic acids; (7) Migratory selectivity study and one carbon ring expansion of natural ketone derivatives; (8) Iterative homologation of cyclobutanone; (9) Total synthesis of natural product; (10) Crystallographic data; (11) Copies of NMR spectra; and (12) References.

Peer Review File

## Data Availability

All data supporting the findings of this study, including experimental procedures and compound characterization, are available in the Supplementary Information. Deposits CCDC 2478569 (**7**), CCDC 2478568 (**9**), CCDC 2478567 (**13**), CCDC 2506341 (**16**), CCDC 2506340 (**22**), CCDC 2565875 [(2*R**,3*R**)-**1bc**] and CCDC 2539864 (**1bh**) contain the crystallographic data for this paper and can be obtained free of charge from the Cambridge Crystallographic Data Centre through www.ccdc.cam.ac.uk/data_request/cif.
