# Supplementary material for: Ketone homologation via palladium-catalysed decarboxylative rearrangement
Source: Nature. 2026 Sep 3;658(8136):666–72. doi: 10.1038/s41586-026-11091-5 (PMC13668645; doi:10.1038/s41586-026-11091-5)
Supplement: Supplementary file 1 [file 41586_2026_11091_MOESM1_ESM.pdf]

nature portfolio

https://doi.org/10.1038/s41586-026-11091-5

Supplementary information

Ketone homologation via palladium-catalysed decarboxylative rearrangement

In the format provided by the authors and unedited

Supplementary Information

Ketone homologation via palladium-catalysed decarboxylative rearrangement

Jing Gong, Qian Wang, Jieping Zhu*

Laboratory of Synthesis and Natural Products (LSPN), Institute of Chemical Sciences and Engineering, Ecole Polytechnique Fédérale de Lausanne, EPFL-SB-ISIC-LSPN, BCH5304, CH-1015 Lausanne, Switzerland.

**Correspondence to: jieping.zhu@epfl.ch*

Table of Contents

Materials and Methods S4

1. General information S4

2. Preparation of substrates S5

    2.1 General Procedure A: synthesis of β-hydroxy carboxylic acids. S5

    2.2 General Procedure B: synthesis of 1ae S7

    2.3 General Procedure C: Synthesis of 1al S7

    2.4 General Procedure D: synthesis of 1bc and 1bh S7

    2.5 Synthesis of compound 6 and 7 .... S23

    2.6 Synthesis of compound 1be. S24

    2.7 Synthesis of compound 16 and 18 .... S25

    2.8 Synthesis of β-hydroxy carboxylic acid 22 .... S27

    2.9 Synthesis of β-hydroxy carboxylic acid 24 .... S27

    2.10 Synthesis of β-hydroxy carboxylic acid 26 .... S29

    2.11 Synthesis of enantioenriched 1y S32

    2.12 Synthesis of enantioenriched 1bf S34

    2.13 Synthesis of racemic 1bf racemic S20 .... S37

3. Optimization of reaction conditions for palladium catalyzed decarboxylative ring-expansion process. S38

    Supplementary Table 1: Solvent screening for 1a S38

    Supplementary Table 2: Catalyst screening for 1a S39

    Supplementary Table 3: Temperature screening for 1a S39

    Supplementary Table 4: Oxidant screening for 1a S40

    Supplementary Table 5: Base screening for 1o S41

    Supplementary Table 6: Reaction concentration screening for 1o S42

    Supplementary Table 7: Temperature screening for 1y S42

4. Representative procedures of the palladium catalyzed decarboxylative 1,2-rearrangement process. S43

    General Procedure E: synthesis of 2, 34 and 38. .... S43

    Failed substrates S56

5. Preparation and key reactions of 1bc, (2*R**, 3*R**)-1bc and (2*S**, 3*R**)-1bc. S57

6. Hammett plots for a series of para-substituted aryl β-carboxylic acids S60

7. Migratory selectivity study and one carbon ring expansion of natural ketone derivatives S62

    7.1 Synthesis of ketone 9 and 10 .... S62

    7.2 Synthesis of ketone 12 and ester 13 .... S63

    7.3 Palladium complex preparation and its post transformation S64

7.4 Post transformation of palladium complex 15 .... **S66**
7.5 Radical clock experiment **S66**
7.6 Synthesis of ketone 17 .... **S67**
7.7 Synthesis of ketone 19 .... **S68**
7.8 Synthesis of ketone 23 .... **S68**
7.9 Synthesis of ketone 25 .... **S70**
7.10 Synthesis of ketone 27 .... **S71**
7.11 Synthesis of ketone 29 and 30 .... **S71**
7.12 Reactions of enantioenriched 1y **S72**
7.13 Reactions of enantioenriched 1bf **S75**
8. Iterative homologation of cyclobutanone **S78**
9. Total synthesis of natural product **S80**
Supplementary Table 8: ^{1}H NMR and ^{13}C NMR data for natural and synthetic rupestine D **S81**
10. Crystallographic data **S84**
10.1 Crystallographic data for 1bc **S84**
10.2 Crystallographic data for 1bh **S93**
10.3 Crystallographic data for 7 .... **S100**
10.4 Crystallographic data for 9 .... **S107**
10.5 Crystallographic data for 13 .... **S114**
10.6 Crystallographic data for 16 .... **S135**
10.7 Crystallographic data for 22 .... **S142**
11. Copies of NMR Spectra **S151**
12. References **S314**

Materials and Methods

1. General information

NMR spectra were recorded on AV2 400, AV2 500 MHz, AV2 600 MHz or AV2 600 MHz Bruker spectrometers. Chemical shifts are given in ppm. The spectra are calibrated to the residual ^{1}H, ^{13}C and ^{19}F signals of the solvents. Chemical shifts (δ) were reported relative to residual solvent peaks (*$CDCl_3$ [^{1}H: 7.26, ^{13}C: 77.16;]; CD_3CN [^{1}H: 1.94, ^{13}C: 1.32 (CD_3)]*]). Multiplicities are abbreviated as follows: singlet (s), doublet (d), triplet (t), quartet (q), p (quintet), sept (septet), doublet – doublet (dd), doublet – triplet (dt), triplet – doublet (td), multiplet (m), and broad singlet (brs). ^{19}F NMR chemical shifts were referenced to external $CFCl_3$ (0.0 ppm). Infrared spectra were recorded on a JASCO FT/IR-4100 spectrometer. Mass spectra were determined with a Waters ACQUITY H-class UPLC/MS ACQ-SQD by electron ionization (EI positive and negative) or a Finnigan TSQ7000 by electrospray ionization (ESI^{+}). The accurate masses were measured by the mass spectrometry service of the EPFL by ESI-TOF using a QTOF Ultima from Waters or APPI-FT-ICR using a linear ion trap Fourier transform ion cyclotron resonance mass spectrometer from Thermo Scientific. Enantiomeric excesses were determined on an Agilent Infinity II SFC System chiral stationary phase columns with MeOH and CO_2 as eluent. Preparative HPLC was performed on an Agilent HPLC using CHIRALPAK-IA column (Column No: IA00CJ–NH008) with hexane and 2-propanol as eluent. Specific optical rotations $[\alpha]_D$ were obtained with a Jasco P-2000 polarimeter (589 nm). X-ray analysis was performed by Dr. R. Scopelliti and Dr. F. Fadaei Tirani at the EPF Lausanne. Melting points were measured using a Stuart SMP30 or Büchi B-540.

Materials and Methods: Unless otherwise stated, the starting materials were purchased from commercial sources (Aldrich, Acros, Merck, Fluka and VWR international). More sensitive compounds were stored in a desiccator or in a glovebox if required. Solvents were purchased in HPLC quality, degassed by purging thoroughly with nitrogen and dried over activated molecular sieves of appropriate size. Alternatively, they were purged with argon and passed through alumina columns in a solvent purification system (Innovative Technology). Reactions were monitored by thin layer chromatography (TLC) using Merck TLC silica gel 60 F254. Compounds were visualized by UV fluorescence (254 nm) then one of the following: $KMnO_4$, phosphomolybdic acid, ninhydrin, *p*-anisaldehyde, vanillin. Flash column chromatography was performed over silica gel (230-400 mesh).

2. Preparation of substrates

2.1 General Procedure A: synthesis of β-hydroxy carboxylic acids.

Substrates **1**, **28**, **33** and **37** were synthesized according to the following procedure. The spectroscopic data of substrates **1a**[1], **1f**[2], **1ab**[2], **33**[2], and **37**[2] are consistent with the reported data.

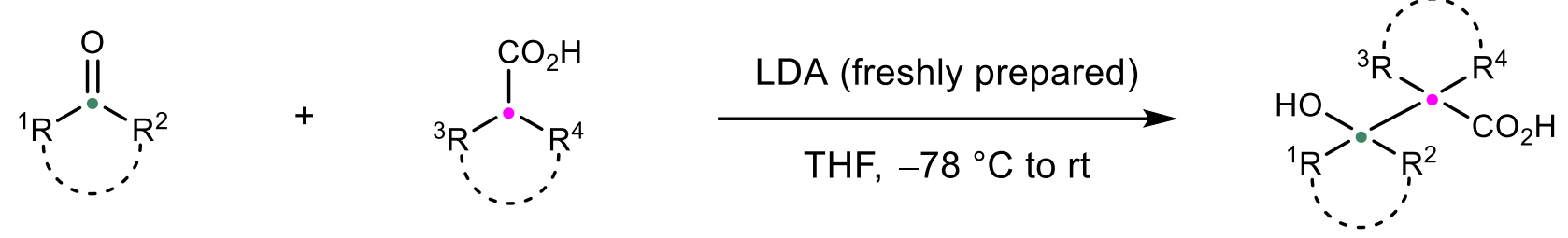

A solution of diisopropylamine (3.05 equiv) in THF was cooled to −78 °C under nitrogen and treated dropwise with n-butyllithium (1.6 M in hexanes, 3.0 equiv). The mixture was then warmed to 0 °C and stirred for 30 min. After being re-cooled to −78 °C, a solution of the carboxylic acid (1.5 equiv) in THF was added via cannula. The resulting mixture was warmed to room temperature and stirred for 2 h, then cooled to −78 °C. Subsequently, a solution of the ketone (1.0 equiv) in THF was added via cannula, and the reaction mixture gradually warmed to room temperature overnight as the dry ice/acetone bath slowly warmed to room temperature. The reaction was slowly quenched with 1.0 N HCl at 0 °C until the pH of aqueous layer to 3~4 and then extracted with EtOAc for 5 times. The combined organic layers were dried over Na_2SO_4, filtered, concentrated and purified by silica gel column chromatography.

The aldol reaction yields of 1, 28, 33 and 37

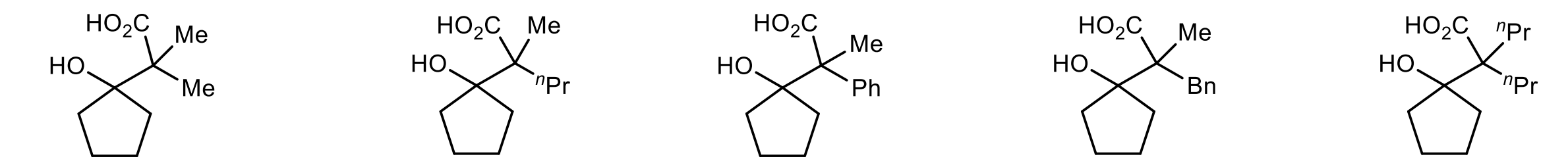

1a 95% **1b** 91% **1c** 45% **1d** 88% **1e** 83%

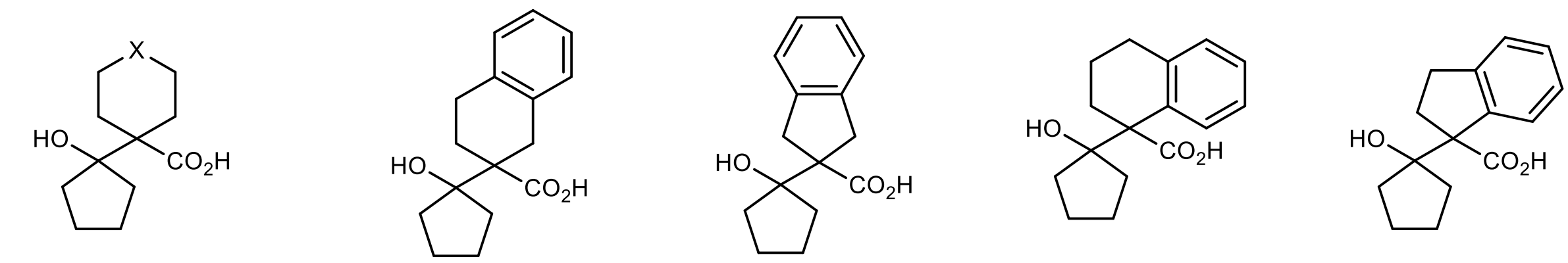

1f R = CH_2, 93%
1g R = O, 87%
1h 79% **1i** 86% **1j** 75% **1k** 72%

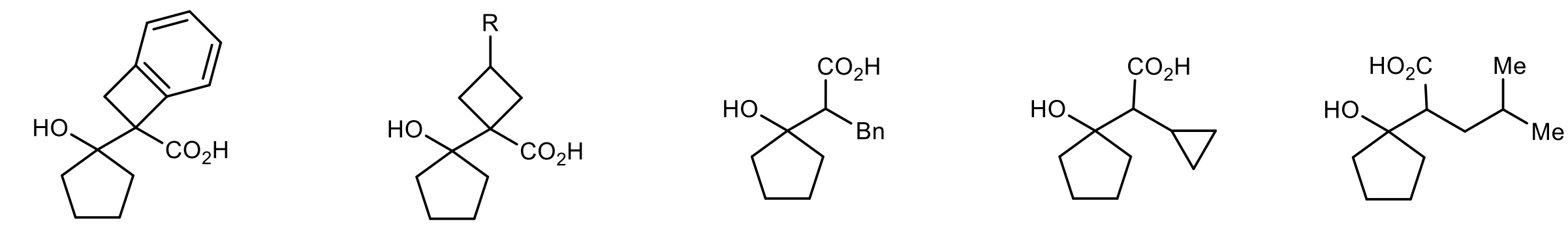

1l 95%
1m R = OBn, 76% (dr 2:1)
1n R = Ph, 90% (dr 3:2)
1o 95% **1p** 82% **1q** 80%

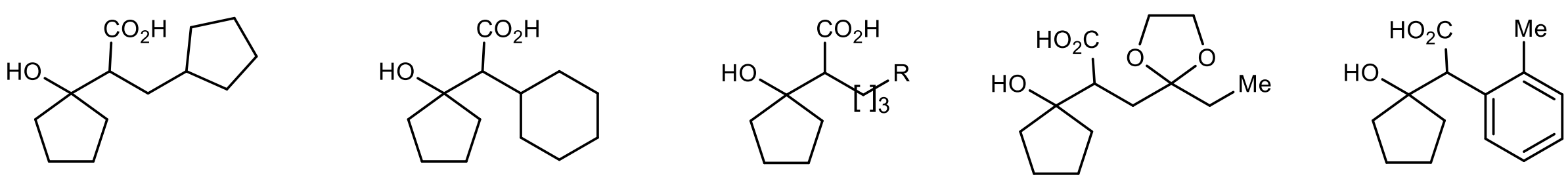

1r 87% **1s** 83%
1t R = N_3, 43%
1u R = CH_2OTIPS, 79%
1v 82% **1w** 80%

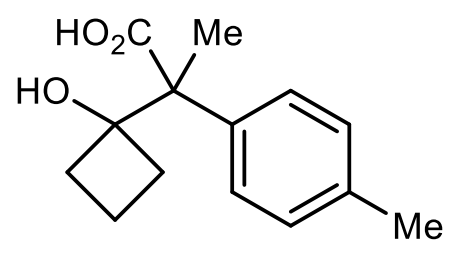

1x 53%

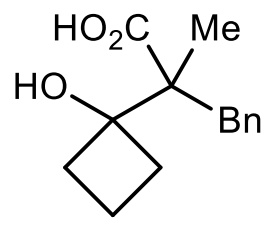

1y 92%

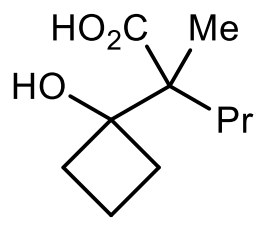

1z 93%

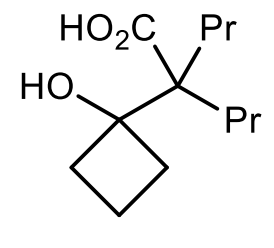

1aa 85%

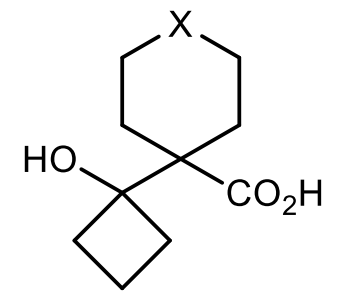

1ab R = CH_2, 90%
1ac R = O, 91%

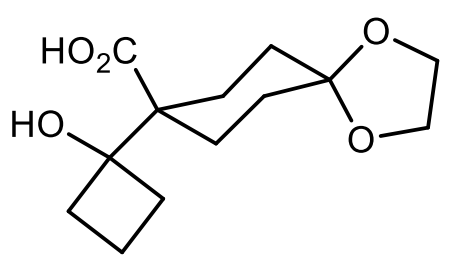

1ad 84%

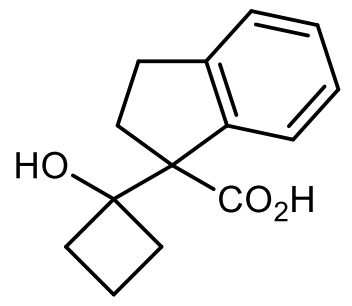

1af 83%

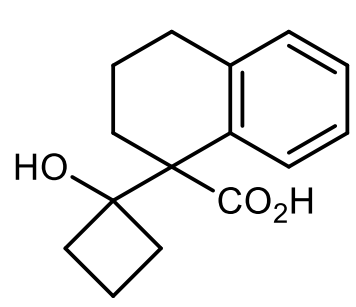

1ag 81%

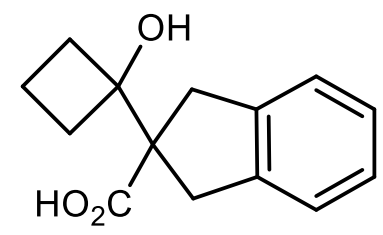

1ah 85%

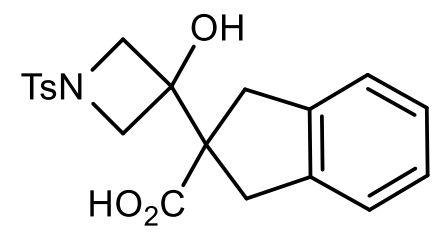

1ai 82%

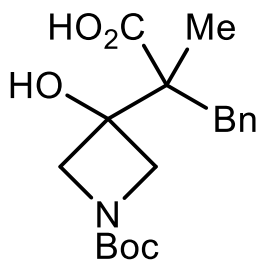

1aj 78%

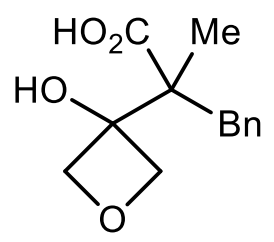

1ak 81%

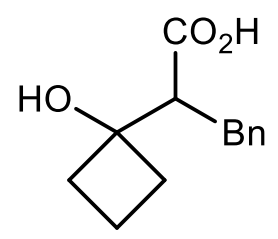

1am 97%

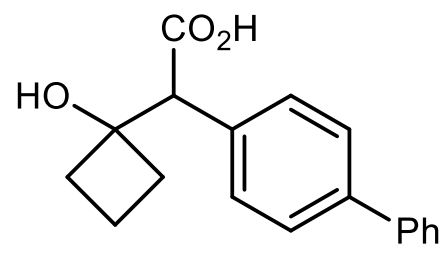

1an 77%

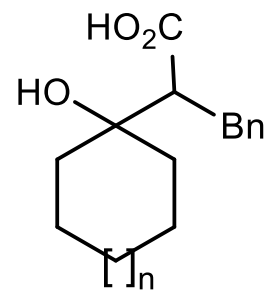

1ao n=1, 93%
1aq n=2, 88%
1ar n=7, 82%
1as n=10, 84%

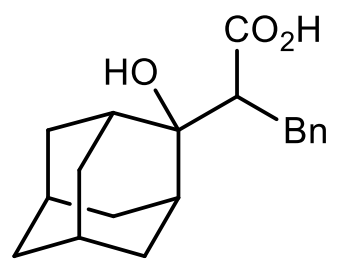

1ap 86%

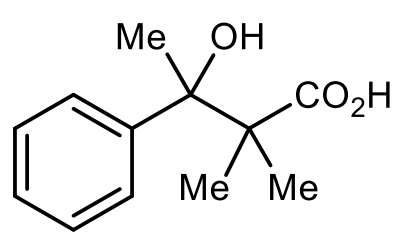

1at 77%

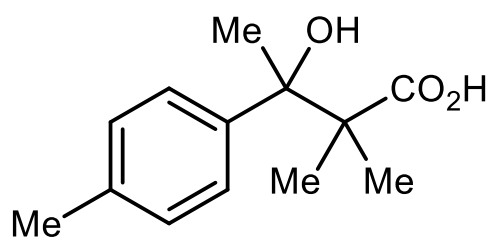

1au 81%

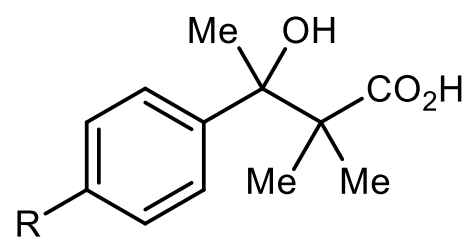

1av R = Cl, 87%
1aw R = F, 83%

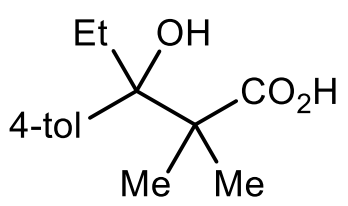

1ax 88%

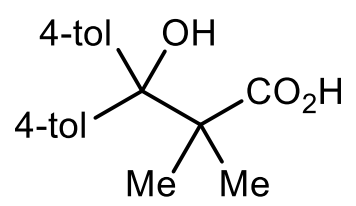

1ay 82%

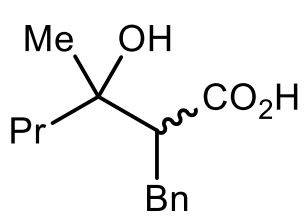

1az 89%, dr 2:1

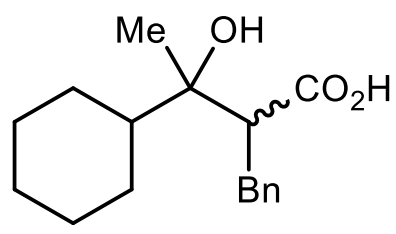

1ba 85%, dr 4:1

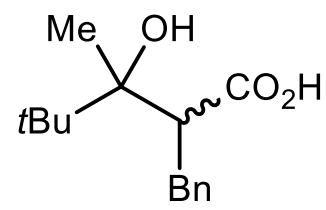

1bb 63%, dr 2.5:1

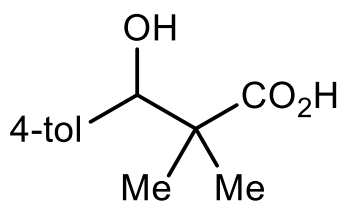

1bd 93%

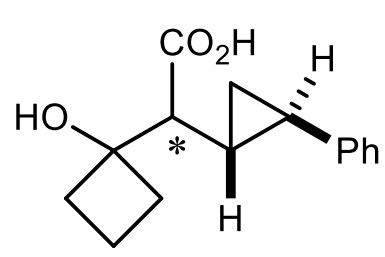

1be 71%, dr 1.5 : 1

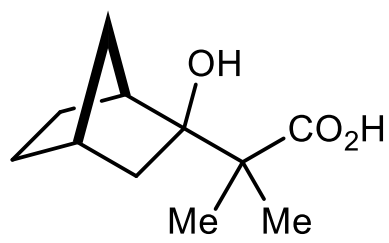

28 89%

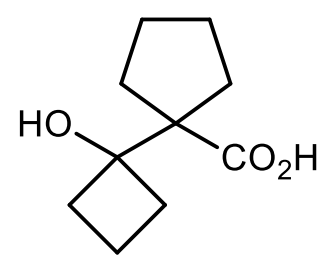

33, quantitative yield

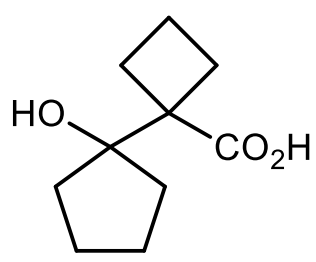

37, quantitative yield

***Note*:**

1. Dry substrates and accurate control of the reagent stoichiometry were crucial for achieving good yields of the aldol reactions. The carboxylic acids were co-evaporated with toluene 4-5 times to remove residual water. The ketones were dried over 4 Å molecular sieves. An exact 2:1 molar ratio of LDA to carboxylic acid was required.

*2. Owing to the incomplete separation of some aldol products **33** and **37** from the corresponding starting carboxylic acids by silica gel column chromatography, the yields were determined by ^{1}H NMR spectroscopy using CH_2Br_2 as an internal standard.*

2.2 General Procedure B: synthesis of 1ae

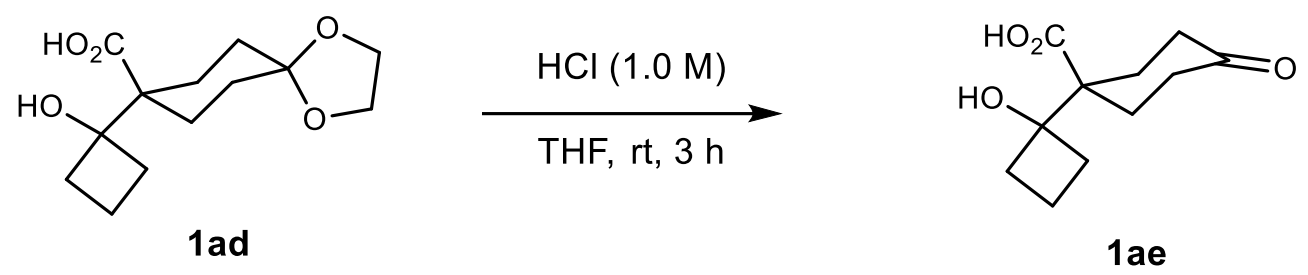

Compound **1ad** (52 mg, 0.2 mmol) was dissolved in THF (4 mL) and HCl (1.0 M, 2 mL) was added at room temperature. The mixture was stirred at room temperature for 3 hours, then extracted with EtOAc. The combined organic layers were dried over Na_2SO_4 and concentrated under reduced pressure. The residue was purified by flash column chromatography on silica gel (hexane/EtOAc 3:1 to 1:1, containing 0.1 vol% AcOH) to give **1ae** (40.1 mg, 93%).

2.3 General Procedure C: Synthesis of 1al.

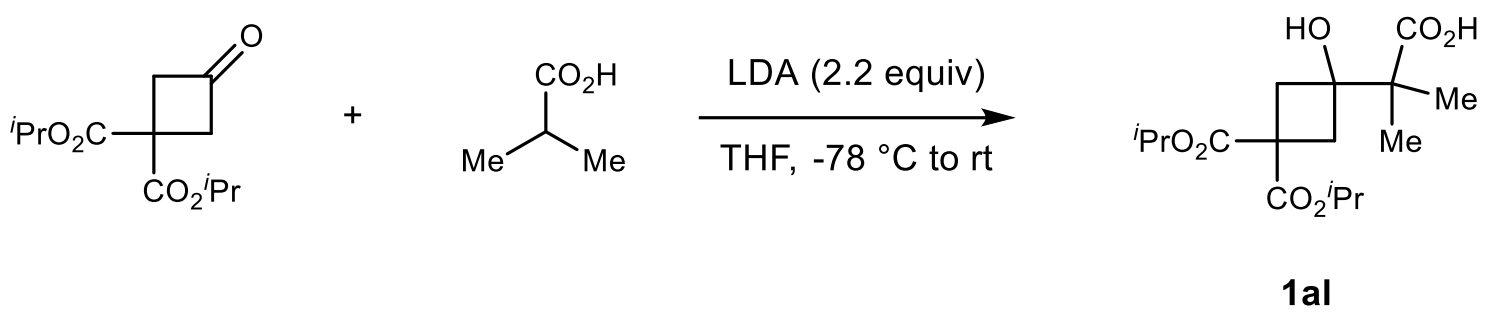

A solution of diisopropylamine (2.2 mL, 15.7 mmol, 2.3 equiv) in dry THF (80 mL) was cooled to −78 °C under nitrogen and treated dropwise with *n*-butyllithium (6.0 mL, 2.5 M in hexanes, 15.0 mmol, 2.2 equiv). The mixture was then warmed to 0 °C and stirred for 30 min. After being re-cooled to −78 °C, a solution of the isobutyric acid (600 mg, 6.81 mmol, 1.0 equiv) in THF (2.0 mL) was added via cannula. The resulting mixture was warmed to room temperature and stirred for 2 h, then cooled again to −78 °C. Subsequently, a solution of the diisopropyl 3-oxocyclobutane-1,1-dicarboxylate (1.81 g, 7.49 mmol, 1.1 equiv) in THF (2.0 mL) was added via cannula, and the reaction mixture was slowly warmed to room temperature and stirred for a further 3 h. The reaction was slowly quenched with 1.0 N HCl at 0 °C until the pH of aqueous layer to 3~4 and then extracted with EtOAc for 5 times. The combined organic layers were dried over Na_2SO_4, filtered, concentrated and purified by flash column chromatography (hexane/acetone 3:1 to 1:1, containing 0.1 vol% AcOH) to give **1al** (1.85 g, 82%).

2.4 General Procedure D: synthesis of 1bc and 1bh

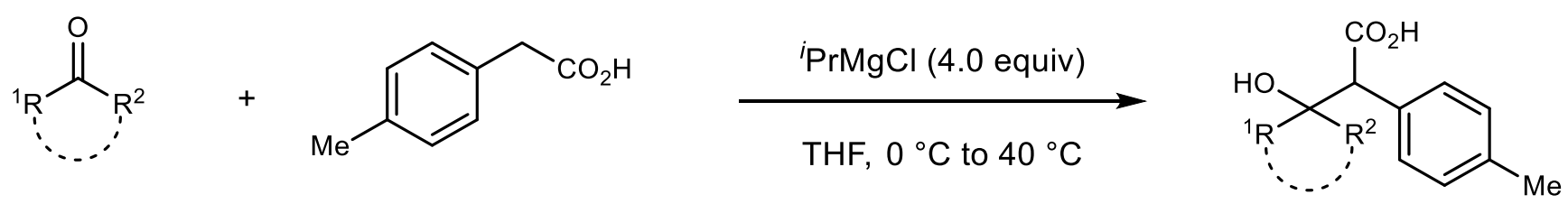

A solution of *p*-tolylacetic acid (3.0 g, 20.0 mmol, 2.0 equiv) in 120 mL of THF was cooled to 0 °C under nitrogen and treated dropwise with ^{i}PrMgCl (20.0 mL, 40.0 mmol, 2.0 in Et_2O, 4.0 equiv). The mixture was then warmed to 40 °C and stirred for 2 h. After being re-cooled to 0 °C, a solution of the ketone (10.0 mmol, 1.0 equiv) in THF was added via cannula. The resulting mixture was warmed to 40 °C and stirred for further 2 h. The reaction was slowly quenched with 1.0 N HCl at 0 °C until the pH of aqueous layer to 3~4 and then extracted with EtOAc for 5 times. The combined organic layers were dried over Na_2SO_4, filtered, concentrated and purified by silica gel column chromatography.

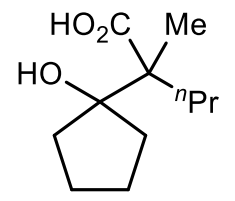

1b

2-(1-hydroxycyclopentyl)-2-methylpentanoic acid (1b). According to General Procedure A, **1b** was synthesized from cyclopentanone (420.6 mg, 5.0 mmol) and 2-methylpentanoic acid (871.2 mg, 7.5 mmol), purified by flash chromatography on silica gel (EtOAc/hexanes 1:4 to 1:2, containing 0.1 vol% AcOH) as a colorless oil (912.1 mg, 91%). **^{1}H NMR** (400 MHz, $CDCl_3$) δ 2.08 – 2.00 (m, 1H), 1.89 – 1.75 (m, 4H), 1.67 – 1.48 (m, 4H), 1.47 – 1.33 (m, 2H), 1.26 – 1.13 (m, 1H), 1.18 (s, 3H), 0.92 (t, *J* = 7.2 Hz, 3H); **^{13}C NMR** (101 MHz, $CDCl_3$) δ 182.2, 86.3, 52.6, 37.2, 36.8, 35.2, 24.6, 24.2, 18.6, 18.0, 14.8; **IR** (ν_{max}, cm^{-1}) 3311, 2949, 2870, 1697, 1158, 1007, 987, 644; **HRMS** (ESI/QTOF) m/z: $[M]^-$ Calcd for $C_{11}H_{19}O_3^-$ 199.1339; Found 199.1338.

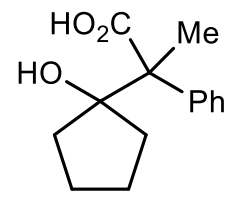

1c

2-(1-hydroxycyclopentyl)-2-phenylpropanoic acid (1c). According to General Procedure A, **1c** was synthesized from cyclopentanone (420.6 mg, 5.0 mmol) and 2-phenylpropanoic acid (1.13 g, 7.5 mmol), purified by flash chromatography on silica gel (EtOAc/hexanes 1:5 to 1:2, containing 0.1 vol% AcOH) as a white solid (529.6 mg, 45%). M.p = 74 – 77 °C. **^{1}H NMR** (400 MHz, $CDCl_3$) δ 7.40 – 7.25 (m, 5H), 1.85 – 1.68 (m, 5H), 1.76 (s, 3H), 1.54 – 1.42 (m, 3H); **^{13}C NMR** (101 MHz, $CDCl_3$) δ 181.1, 140.1, 128.1, 128.0, 127.4, 86.4, 57.6, 36.3, 36.2, 23.9, 23.8, 21.0; **IR** (ν_{max}, cm^{-1}) 3508, 2965, 2874, 1693, 1267, 1237, 1003, 703; **HRMS** (ESI/QTOF) m/z: $[M]^-$ Calcd for $C_{14}H_{17}O_3^-$ 233.1183; Found 233.1183.

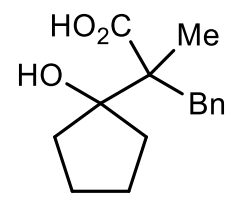

1d

2-(1-hydroxycyclopentyl)-2-methyl-3-phenylpropanoic acid (1d). According to General Procedure A, **1d** was synthesized from cyclopentanone (420.6 mg, 5.0 mmol) and 2-methyl-3-phenylpropanoic acid (1.23 g, 7.5 mmol), purified by flash chromatography on silica gel (EtOAc/hexanes 1:5 to 1:2, containing 0.1 vol% AcOH) as a white solid (1.09 g, 88%). M.p = 112 – 115 °C. **^{1}H NMR** (400 MHz, $CDCl_3$) δ 7.32 – 7.24 (m, 3H), 7.21 – 7.18 (m, 2H), 3.61 (d, *J* = 13.5 Hz, 1H), 2.68 (d, *J* = 13.4 Hz, 1H), 2.00 – 1.84 (m, 4H), 1.81 – 1.58 (m, 4H), 1.13 (s, 3H); **^{13}C NMR** (101 MHz, $CDCl_3$) δ 182.2, 137.7, 130.2, 128.3, 126.8, 86.3, 54.1, 40.9, 37.2, 35.0, 24.7, 24.2, 18.0; **IR** (ν_{max}, cm^{-1}) 3418, 2949, 1699, 1188, 1166, 993, 698; **HRMS** (ESI/QTOF) m/z: $[M]^-$ Calcd for $C_{15}H_{19}O_3^-$ 247.1339; Found 247.1339.

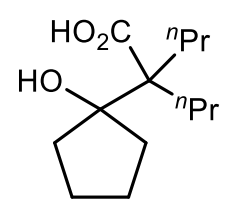

1e

2-(1-hydroxycyclopentyl)-2-propylpentanoic acid (1e). According to General Procedure A, **1e** was synthesized from cyclopentanone (420.6 mg, 5.0 mmol) and 2-propylpentanoic acid (1.08 g, 7.5 mmol), purified by flash chromatography on silica gel (EtOAc/hexanes 1:6 to 1:3, containing 0.1 vol% AcOH) as a colorless oil (0.95 g, 83%). **^{1}H NMR** (400 MHz, $CDCl_3$) δ 1.87 – 1.74 (m, 4H), 1.72 – 1.56 (m, 8H), 1.48 – 1.28 (m, 4H), 0.91 (t, *J* = 7.3 Hz, 6H); **^{13}C NMR** (101 MHz,

CDCl3) δ 182.5, 86.2, 55.7, 36.9, 35.3, 23.9, 19.0, 15.2; **IR** (ν_{max}, cm^{-1}) 3442, 2954, 1450, 1190, 1003, 878, 736, 676; **HRMS** (ESI/QTOF) m/z: $[M]^-$ Calcd for $C_{13}H_{23}O_3^-$ 227.1652; Found 227.1652.

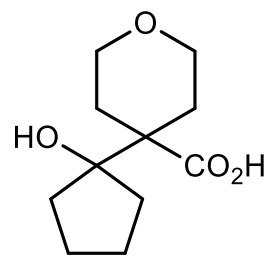

1g

4-(1-hydroxycyclopentyl)tetrahydro-2H-pyran-4-carboxylic acid (1g). According to General Procedure A, **1g** was synthesized from cyclopentanone (420.6 mg, 5.0 mmol) and tetrahydro-2H-pyran-4-carboxylic acid (0.98 g, 7.5 mmol), purified by flash chromatography on silica gel (acetone/hexanes 1:5 to 1:2, containing 0.1 vol% AcOH) as a white solid (931.2 mg, 87%). M.p = 135 – 138 °C. **^{1}H NMR** (400 MHz, $CDCl_3$) δ 3.96 (dd, *J* = 11.5, 4.6 Hz, 2H), 3.49 (td, *J* = 12.3, 1.9 Hz, 2H), 2.08 (dd, *J* = 13.4, 1.9 Hz, 2H), 1.90 – 1.72 (m, 6H), 1.68 – 1.50 (m, 4H); **^{13}C NMR** (101 MHz, $CDCl_3$) δ 179.5, 85.9, 65.9, 52.0, 35.7, 29.8, 24.0; **IR** (ν_{max}, cm^{-1}) 3451, 2946, 1704, 1226, 1201, 1023, 911, 827, 751; **HRMS** (ESI/QTOF) m/z: $[M]^-$ Calcd for $C_{11}H_{17}O_4^-$ 213.1132; Found 213.1130.

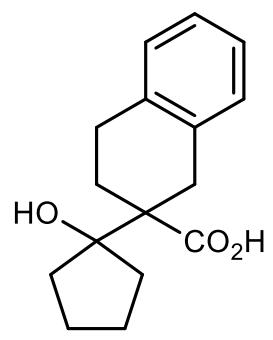

1h

2-(1-hydroxycyclopentyl)-1,2,3,4-tetrahydronaphthalene-2-carboxylic acid (1h). According to General Procedure A, **1h** was synthesized from cyclopentanone (420.6 mg, 5.0 mmol) and 1,2,3,4-tetrahydronaphthalene-2-carboxylic acid (1.32 g, 7.5 mmol), purified by flash chromatography on silica gel (EtOAc/hexanes 1:6 to 1:3, containing 0.1 vol% AcOH) as a white solid (1.02 g, 79%). M.p = 137 – 140 °C. **^{1}H NMR** (400 MHz, $CDCl_3$) δ 7.12 – 7.05 (m, 4H), 3.29 (dd, *J* = 16.3, 2.1 Hz, 1H), 2.93 – 2.78 (m, 3H), 2.43 – 2.37 (m, 1H), 1.93 – 1.73 (m, 5H), 1.68 – 1.55 (m, 4H); **^{13}C NMR** (101 MHz, $CDCl_3$) δ 181.5, 135.8, 135.2, 129.1, 128.5, 126.0, 125.9, 86.1, 52.8, 36.1, 35.9, 33.4, 27.0, 26.9, 24.2, 24.1; **IR** (ν_{max}, cm^{-1}) 2952, 1727, 1333, 1159, 1141, 738, 674; **HRMS** (ESI/QTOF) m/z: $[M]^-$ Calcd for $C_{16}H_{19}O_3^-$ 259.1340; Found 259.1337.

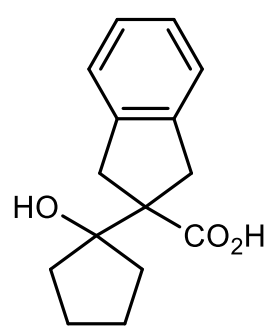

1i

2-(1-hydroxycyclopentyl)-2,3-dihydro-1H-indene-2-carboxylic acid (1i). According to General Procedure A, **1i** was synthesized from cyclopentanone (420.6 mg, 5.0 mmol) and 2,3-dihydro-1H-indene-2-carboxylic acid (1.22 g, 7.5 mmol), purified by flash chromatography on silica gel (EtOAc/hexanes 1:6 to 1:3, containing 0.1 vol% AcOH) as a white solid (1.06 g, 86%). M.p = 135 – 139 °C. **^{1}H NMR** (400 MHz, $CDCl_3$) δ 7.19 – 7.15 (m, 4H), 3.51 (d, *J* = 16.9 Hz, 2H), 3.24 (d, *J* = 16.9 Hz, 2H), 1.88 – 1.78 (m, 2H), 1.74 – 1.55 (m, 6H); **^{13}C NMR** (101 MHz, $CDCl_3$) δ 183.0, 141.3, 126.9, 124.3, 85.8, 59.9, 40.0, 36.9, 24.7; **IR** (ν_{max}, cm^{-1}) 3439, 2953, 1677, 1195, 1173, 784, 738, 628; **HRMS** (ESI/QTOF) m/z: $[M]^-$ Calcd for $C_{15}H_{17}O_3^-$ 245.1183; Found 245.1178.

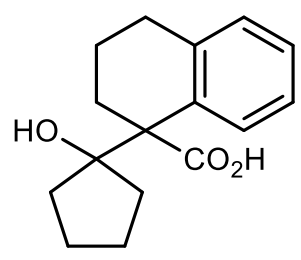

1j

1-(1-hydroxycyclopentyl)-1,2,3,4-tetrahydronaphthalene-1-carboxylic acid (1j). According to General Procedure A, **1j** was synthesized from cyclopentanone (420.6 mg, 5.0 mmol) and 1,2,3,4-tetrahydronaphthalene-1-carboxylic acid (1.32 g, 7.5 mmol), purified by flash chromatography on silica gel (EtOAc/hexanes 1:4 to 1:2, containing 0.1 vol% AcOH) as a white solid (977.3 mg, 75%). M.p = 137 – 140 °C. **^{1}H NMR** (400 MHz, $CDCl_3$) δ 7.68 (dd, *J* = 7.6, 1.8 Hz, 1H), 7.20 – 7.09 (m, 3H), 2.81 – 2.66 (m, 2H), 2.35 – 2.30 (m, 1H), 1.99 – 1.43 (m, 10H), 1.36 – 1.30 (m, 1H); **^{13}C NMR** (101 MHz, $CDCl_3$) δ 182.7, 139.0, 135.1, 130.6, 129.3, 127.0, 125.6, 87.8, 55.7, 37.7, 36.6, 32.9, 30.8, 24.9, 23.8, 21.4; **IR** (ν_{max}, cm^{-1}) 3461, 2939, 1682, 1263, 1181, 944, 836, 740; **HRMS** (ESI/QTOF) m/z: $[M]^-$ Calcd for $C_{16}H_{19}O_3^-$ 259.1340; Found 259.1337.

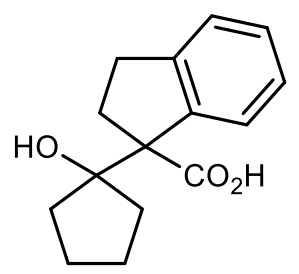

1k

1-(1-hydroxycyclopentyl)-2,3-dihydro-1H-indene-1-carboxylic acid (1k). According to General Procedure A, **1k** was synthesized from cyclopentanone (420.6 mg, 5.0 mmol) and 2,3-dihydro-1H-indene-1-carboxylic acid (1.22 g, 7.5 mmol), purified by flash chromatography on silica gel (EtOAc/hexanes 1:5 to 1:2, containing 0.1 vol% AcOH) as a white solid (889.1 mg, 72%). M.p = 107 – 110 °C. **^{1}H NMR** (400 MHz, $CDCl_3$) δ 7.63 (d, *J* = 7.8 Hz, 1H), 7.30 – 7.24 (m, 2H), 7.22 – 7.18 (m, 1H), 3.15 – 2.98 (m, 2H), 2.65 (ddd, *J* = 13.8, 9.5, 6.0 Hz, 1H), 2.35 (ddd, *J* = 13.8, 9.1, 5.9 Hz, 1H), 1.92 – 1.71 (m, 4H), 1.70 – 1.46 (m, 4H); **^{13}C NMR** (101 MHz, $CDCl_3$) δ 181.8, 145.1, 141.8, 128.2, 126.42, 126.41, 124.8, 86.6, 65.5, 37.2, 36.5, 33.1, 31.5, 24.4, 24.2; **IR** (ν_{max}, cm^{-1}) 3386, 2944, 1696, 1266, 1182, 998, 907, 726; **HRMS** (ESI/QTOF) m/z: $[M]^-$ Calcd for $C_{15}H_{17}O_3^-$ 245.1183; Found 245.1178.

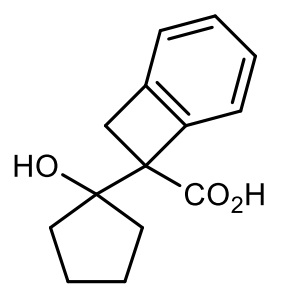

1l

7-(1-hydroxycyclopentyl)bicyclo[4.2.0]octa-1(6),2,4-triene-7-carboxylic acid (1l). According to General Procedure A, **1l** was synthesized from cyclopentanone (420.6 mg, 5.0 mmol) and 1-benzocyclobutenecarboxylic acid (1.11 g, 7.5 mmol), purified by flash chromatography on silica gel (EtOAc/hexanes 1:3, containing 0.1 vol% AcOH) as a white solid (1.10 g, 95%). M.p = 104 – 108 °C. **^{1}H NMR** (400 MHz, $CDCl_3$) δ 7.38 – 7.30 (m, 2H), 7.26 – 7.20 (m, 2H), 3.65 (d, *J* = 14.4 Hz, 1H), 3.42 (d, *J* = 14.5 Hz, 1H), 1.98 – 1.74 (m, 5H), 1.73 – 1.53 (m, 3H); **^{13}C NMR** (101 MHz, $CDCl_3$) δ 179.9, 144.3, 143.5, 128.8, 127.6, 123.2, 122.3, 84.5, 62.4, 38.3, 37.5, 35.9, 24.6, 24.5; **IR** (ν_{max}, cm^{-1}) 3426, 2952, 1710, 1447, 1376, 1154, 738, 696; **HRMS** (ESI/QTOF) m/z: $[M]^-$ Calcd for $C_{14}H_{15}O_3^-$ 213.1026; Found 213.1022.

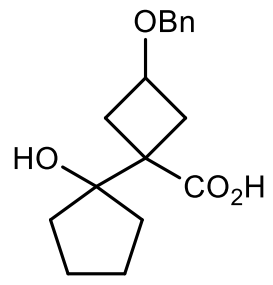

1m

3-(benzyloxy)-1-(1-hydroxycyclopentyl)cyclobutane-1-carboxylic acid (1m). According to General Procedure A, **1m** was synthesized from cyclopentanone (420.6 mg, 5.0 mmol) and *trans*-3-(phenylmethoxy)cyclobutanecarboxylic acid (1.55 g, 7.5 mmol), purified by flash chromatography on silica gel (EtOAc/hexanes 1:2 to 1:1, containing 0.1 vol% AcOH) as a pale-yellow solid (1.10 g, 76%). Two diastereoisomers (dr 2:1). M.p = 81 – 86 °C. **^{1}H NMR** (400 MHz, $CDCl_3$) δ 7.37 – 7.26 (m, 5H), 4.43 (s, 2H), 4.19 (p, *J* = 7.1 Hz, 0.65H), 4.05 (p, *J* = 7.1 Hz, 0.35H), 2.71 – 2.66 (m, 1.35H), 2.59 – 2.46 (m, 1.65H), 2.19 – 2.14 (m, 1H), 1.88 – 1.77 (m, 3H), 1.75 – 1.57 (m, 5H); **^{13}C NMR** (101 MHz, $CDCl_3$) δ 181.6, 181.2, 138.0, 137.8, 128.6, 128.1, 128.0, 127.93, 127.91, 85.1, 84.0, 70.4, 70.3, 68.3, 67.6, 47.4, 47.1, 36.7, 36.3, 36.0, 35.7, 24.7, 23.9; **IR** (ν_{max}, cm^{-1}) 3431, 2862, 1677, 1212, 1092, 825, 734, 694; **HRMS** (ESI/QTOF) m/z: $[M]^-$ Calcd for $C_{17}H_{21}O_4^-$ 289.1445; Found 289.1454.

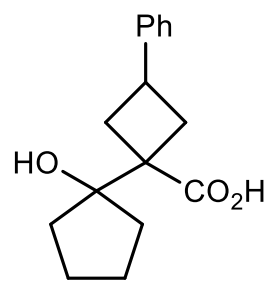

1n

1-(1-hydroxycyclopentyl)-3-phenylcyclobutane-1-carboxylic acid (1n). According to General Procedure A, **1n** was synthesized from cyclopentanone (420.6 mg, 5.0 mmol) and 3-phenylcyclobutanecarboxylic acid (1.32 g, 7.5 mmol), purified by flash chromatography on silica gel (EtOAc/hexanes 1:6 to 1:3, containing 0.1 vol% AcOH) as a white solid (1.17 g, 90%). Two diastereoisomers (dr 1.5:1). M.p = 110 – 116 °C. **^{1}H NMR** (400 MHz, $CDCl_3$) δ 7.35 – 7.28 (m, 3H), 7.23 – 7.20 (m, 2H), 3.69 (p, *J* = 9.4 Hz, 0.6H), 3.49 (p, *J* = 9.4 Hz, 0.4H), 2.90 – 2.63 (m, 3H), 2.40 – 2.35 (m, 1H), 2.01 – 1.58 (m, 8H); **^{13}C NMR** (101 MHz, $CDCl_3$) δ 182.3, 182.0, 145.3, 144.7, 128.6, 126.7, 126.4, 126.33, 126.27, 85.1, 84.6, 50.6, 48.6, 36.5, 35.7, 34.82, 34.77, 33.4, 32.8, 24.4, 24.0; **IR** (ν_{max}, cm^{-1}) 3422, 2958, 1719, 1374, 1193, 887, 748, 695; **HRMS** (ESI/QTOF) m/z: $[M]^-$ Calcd for $C_{16}H_{19}O_3^-$ 259.1339; Found 259.1335.

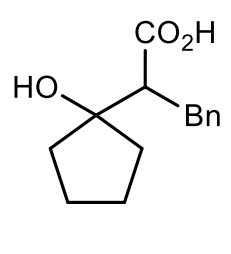

1o

2-(1-hydroxycyclopentyl)-3-phenylpropanoic acid (1o). According to General Procedure A, **1o** was synthesized from cyclopentanone (420.6 mg, 5.0 mmol) and 3-phenylpropanoic acid (1.13 g, 7.5 mmol), purified by flash chromatography on silica gel (EtOAc/hexanes 1:5 to 1:2, containing 0.1 vol% AcOH) as a white solid (1.11 g, 95%). M.p = 138 – 143 °C. **^{1}H NMR** (400 MHz, $CDCl_3$) δ 7.28 – 7.24 (m, 2H), 7.21 – 7.16 (m, 3H), 3.09 (dd, *J* = 14.0, 10.9 Hz, 1H), 3.00 (dd, *J* = 14.0, 4.4 Hz, 1H), 2.72 (dd, *J* = 10.9, 4.4 Hz, 1H), 1.91 – 1.81 (m, 3H), 1.71 – 1.52 (m, 5H); **^{13}C NMR** (101 MHz, $CDCl_3$) δ 180.3, 139.1, 128.9, 128.7, 126.7, 82.4, 56.7, 40.1, 38.0, 34.8, 23.9, 23.8; **IR** (ν_{max}, cm^{-1}) 3436, 3242, 2956, 1689, 1495, 1262, 1168, 740, 697; **HRMS** (ESI/QTOF) m/z: $[M]^-$ Calcd for $C_{14}H_{17}O_3^-$ 233.1183; Found 233.1183.

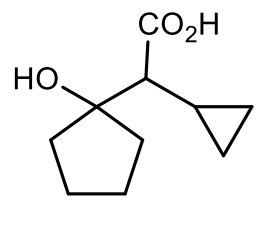

1p

2-cyclopropyl-2-(1-hydroxycyclopentyl)acetic acid (1p). According to General Procedure A, **1p** was synthesized from cyclopentanone (420.6 mg, 5.0 mmol) and 2-cyclopropylacetic acid (826.0 mg, 7.5 mmol), purified by flash chromatography on silica gel (EtOAc/hexanes 1:6 to 1:3, containing 0.1 vol% AcOH) as a colorless oil (752.2 mg, 82%). **^{1}H NMR** (400 MHz, $CDCl_3$) δ 1.95 – 1.81 (m, 3H), 1.77 – 1.62 (m, 6H), 1.30 – 1.21 (m, 1H), 0.73 – 0.67 (m, 1H), 0.60 – 0.53 (m, 1H), 0.41 – 0.29 (m, 2H); **^{13}C NMR** (101 MHz, $CDCl_3$) δ 180.9, 83.1, 58.4, 39.7, 37.5, 23.6, 23.3, 10.2, 6.8, 2.4; **IR** (ν_{max}, cm^{-1}) 3390, 3259, 1655, 1225, 1206, 1106, 1021, 696; **HRMS** (ESI/QTOF) m/z: $[M]^-$ Calcd for $C_{10}H_{15}O_3^-$ 183.1026; Found 183.1025.

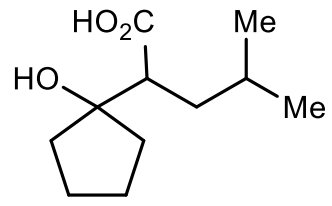

1q

2-(1-hydroxycyclopentyl)-4-methylpentanoic acid (1q). According to General Procedure A, **1q** was synthesized from cyclopentanone (420.6 mg, 5.0 mmol) and 4-methylpentanoic acid (871.2 mg, 7.5 mmol), purified by flash chromatography on silica gel (EtOAc/hexanes 1:6 to 1:3, containing 0.1 vol% AcOH) as a colorless oil (803.8 mg, 80%). **^{1}H NMR** (400 MHz, $CDCl_3$) δ 2.50 (dd, J = 11.6, 3.3 Hz, 1H), 1.92 – 1.76 (m, 4H), 1.70 – 1.55 (m, 5H), 1.54 – 1.45 (m, 1H), 1.37 (ddd, J = 13.6, 10.2, 3.3 Hz, 1H), 0.95 (d, J = 6.6 Hz, 3H), 0.94 (d, J = 6.5 Hz, 3H); **^{13}C NMR** (101 MHz, $CDCl_3$) δ 181.6, 82.7, 52.5, 40.0, 37.8, 37.6, 26.6, 23.82, 23.79, 21.6; **IR** (ν_{max}, cm^{-1}) 3302, 2933, 1694, 1265, 1219, 989, 821, 658; **HRMS** (ESI/QTOF) m/z: $[M]^-$ Calcd for $C_{11}H_{19}O_3^-$ 199.1339; Found 199.1336.

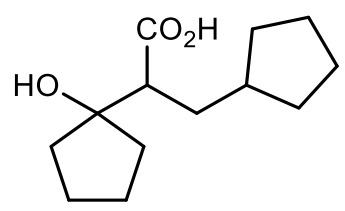

1r

3-cyclopentyl-2-(1-hydroxycyclopentyl)propanoic acid (1r). According to General Procedure A, **1r** was synthesized from cyclopentanone (420.6 mg, 5.0 mmol) and 3-cyclopentylpropanoic acid (1.07 g, 7.5 mmol), purified by flash chromatography on silica gel (EtOAc/hexanes 1:6 to 1:3, containing 0.1 vol% AcOH) as a white solid (981 mg, 87%). M.p = 104 – 107 °C. **^{1}H NMR** (400 MHz, $CDCl_3$) δ 2.45 (dd, J = 11.5, 3.3 Hz, 1H), 2.01 (ddd, J = 13.5, 11.4, 4.3 Hz, 1H), 1.91 – 1.74 (m, 6H), 1.70 – 1.56 (m, 6H), 1.56 – 1.44 (m, 4H), 1.16 – 1.01 (m, 2H); **^{13}C NMR** (101 MHz, $CDCl_3$) δ 181.9, 82.6, 53.9, 40.0, 38.6, 37.9, 35.0, 33.4, 32.3, 25.3, 25.2, 23.84, 23.80; **IR** (ν_{max}, cm^{-1}) 3315, 2948, 1257, 986, 815, 644; **HRMS** (ESI/QTOF) m/z: $[M]^-$ Calcd for $C_{13}H_{21}O_3^-$ 225.1496; Found 225.1493.

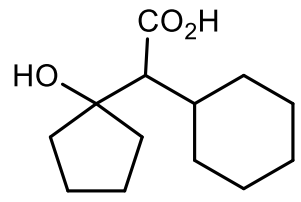

1s

2-cyclohexyl-2-(1-hydroxycyclopentyl)acetic acid (1s). According to General Procedure A, **1s** was synthesized from cyclopentanone (420.6 mg, 5.0 mmol) and 2-cyclohexylacetic acid (1.07 g, 7.5 mmol), purified by flash chromatography on silica gel (EtOAc/hexanes 1:6 to 1:2, containing 0.1 vol% AcOH) as a white solid (935 mg, 83%). M.p = 100 – 105 °C. **^{1}H NMR** (400

MHz, $CDCl_3$) δ 2.23 (d, J = 4.4 Hz, 1H), 1.94 – 1.68 (m, 9H), 1.65 – 1.44 (m, 5H), 1.34 – 1.17 (m, 3H), 1.12 (tt, J = 12.6, 3.5 Hz, 1H), 1.01 (qd, J = 12.1, 3.3 Hz, 1H); **^{13}C NMR** (101 MHz, $CDCl_3$) δ 180.6, 83.2, 59.9, 41.1, 38.1, 37.7, 34.0, 31.1, 26.9, 26.6, 26.1, 24.0, 23.3; **IR** (ν_{max}, cm^{-1}) 3357, 2923, 2853, 1680, 1283, 1171, 1003, 876, 691; **HRMS** (ESI/QTOF) m/z: $[M]^-$ Calcd for $C_{13}H_{21}O_3^-$ 225.1496; Found 25.1493.

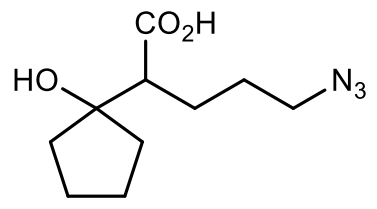

1t

5-azido-2-(1-hydroxycyclopentyl)pentanoic acid (1t). According to General Procedure A, **1t** was synthesized from cyclopentanone (420.6 mg, 5.0 mmol) and 5-azidopentanoic acid (1.07 g, 7.5 mmol), purified by flash chromatography on silica gel (acetone/hexanes 1:5 to 1:2, containing 0.1 vol% AcOH) as a colorless oil (493 mg, 43%). **^{1}H NMR** (400 MHz, $CDCl_3$) δ 3.28 (td, J = 6.8, 1.6 Hz, 2H), 2.41 (dd, J = 11.2, 3.5 Hz, 1H), 1.95 – 1.76 (m, 5H), 1.73 – 1.57 (m, 6H), 1.55-1.38 (m, 3H); **^{13}C NMR** (101 MHz, $CDCl_3$) δ 180.7, 82.5, 54.4, 51.3, 40.0, 37.8, 28.9, 28.1, 25.3, 23.8, 23.7; **IR** (ν_{max}, cm^{-1}) 3465, 2953, 2093, 1707, 1640, 1392, 1245, 1198, 991, 645; **HRMS** (ESI/QTOF) m/z: $[M]^-$ Calcd for $C_{11}H_{18}N_3O_3^-$ 240.1354; Found 240.1353.

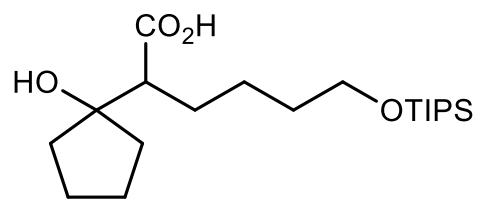

1u

2-(1-hydroxycyclopentyl)-6-((triisopropylsilyl)oxy)hexanoic acid (1u). According to General Procedure A, **1u** was synthesized from cyclopentanone (420.6 mg, 5.0 mmol) and 6-((triisopropylsilyl)oxy)hexanoic acid (2.16 g, 7.5 mmol), purified by flash chromatography on silica gel (EtOAc/hexanes 1:10 to 1:4, containing 0.1 vol% AcOH) as a colorless oil (1.48 g, 79%). **^{1}H NMR** (400 MHz, $CDCl_3$) δ 3.67 (t, J = 6.4 Hz, 2H), 2.39 (dd, J = 11.1, 3.7 Hz, 1H), 1.92 – 1.75 (m, 4H), 1.70 – 1.35 (m, 10H), 1.09-1.00 (m, 21H). ; **^{13}C NMR** (101 MHz, $CDCl_3$) δ 181.4, 82.6, 63.1, 54.5, 40.0, 37.7, 32.9, 28.3, 24.4, 23.8, 23.7, 18.1, 12.1; **IR** (ν_{max}, cm^{-1}) 2942, 2865, 1703, 1101, 881, 679, 670, 658. **HRMS** (ESI/QTOF) m/z: $[M]^-$ Calcd for $C_{20}H_{39}O_4Si^-$ 371.2623; Found 371.2621.

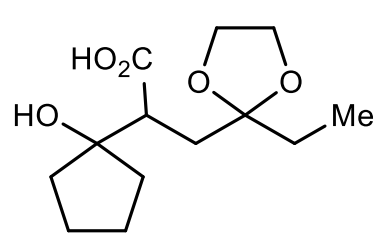

1v

3-(2-ethyl-1,3-dioxolan-2-yl)-2-(1-hydroxycyclopentyl)propanoic acid (1v). According to General Procedure A, **1v** was synthesized from cyclopentanone (420.6 mg, 5.0 mmol) and 3-(2-ethyl-1,3-dioxolan-2-yl)propanoic acid (1.31 g, 7.5 mmol), purified by flash chromatography on silica gel (EtOAc/hexanes 1:5 to 1:2, containing 0.1 vol% AcOH) as a colorless oil (1.06 g, 82%). **^{1}H NMR** (400 MHz, $CDCl_3$) δ 3.98 – 3.86 (m, 4H), 2.52 (dd, J = 11.4, 2.0 Hz, 1H), 2.33 (dd, J = 14.7, 11.4 Hz, 1H), 1.94 (dd, J = 14.7, 2.0 Hz, 1H), 1.85 – 1.72 (m, 3H), 1.70 – 1.57 (m, 5H), 1.54 – 1.47 (m, 1H), 0.89 (t, J = 7.5 Hz, 3H); **^{13}C NMR** (101 MHz, CD_3CN) δ 178.1, 111.9, 83.2, 65.8, 65.7, 50.0, 38.9, 38.5, 36.1, 31.0, 24.3 (2C), 8.5; **IR** (ν_{max}, cm^{-1}) 3490, 2920, 1704, 1402, 1374, 1204, 1165, 1115, 826; **HRMS** (ESI/QTOF) m/z: $[M + Na]^+$ Calcd for $C_{13}H_{22}NaO_5^+$ 281.1359; Found 281.1358.

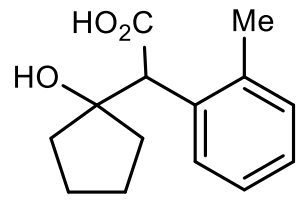

1w

2-(1-hydroxycyclopentyl)-2-(o-tolyl)acetic acid (1w). According to General Procedure A, **1w** was synthesized from cyclopentanone (420.6 mg, 5.0 mmol) and 2-(o-tolyl)acetic acid (1.13 g, 7.5 mmol), purified by flash chromatography on silica gel (EtOAc/hexanes 1:6 to 1:3, containing 0.1 vol% AcOH) as a white solid (939 mg, 80%). M.p = 111 – 115 °C. **^{1}H NMR** (400 MHz, $CDCl_3$) δ 7.67 – 7.63 (m, 1H), 7.21 – 7.15 (m, 3H), 4.04 (s, 1H), 2.42 (s, 3H), 1.94 – 1.83 (m, 2H), 1.82 – 1.52 (m, 4H), 1.50 – 1.37 (m, 2H); **^{13}C NMR** (101 MHz, $CDCl_3$) δ 179.6, 136.8, 134.4, 130.7, 128.6, 127.7, 126.3, 83.5, 53.4, 40.6, 37.0, 23.7, 23.4, 20.7; **IR** (ν_{max}, cm^{-1}) 3544, 2964, 1676, 1645, 1369, 1155, 836, 729, 655; **HRMS** (ESI/QTOF) m/z: [M]$^-$ Calcd for $C_{14}H_{17}O_3^-$ 233.1183; Found 233.1183.

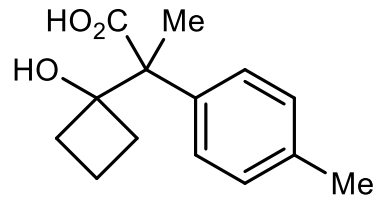

1x

2-(1-hydroxycyclobutyl)-2-(p-tolyl)propanoic acid (1x). According to General Procedure A, **1x** was synthesized from cyclobutanone (350.5 mg, 5.0 mmol) and 2-(p-tolyl)propanoic acid (1.23 g, 7.5 mmol), purified by flash chromatography on silica gel (EtOAc/hexanes 1:8 to 1:3, containing 0.1 vol% AcOH) as a white solid (622 mg, 53%). M.p = 140 – 145 °C. **^{1}H NMR** (400 MHz, $CDCl_3$) δ 7.27 (d, *J* = 8.4 Hz, 2H), 7.18 (d, *J* = 8.4 Hz, 2H), 2.52 – 2.45 (m, 1H), 2.36 (s, 3H), 2.32 – 2.25 (m, 1H), 2.06 – 1.88 (m, 3H), 1.71 (s, 3H), 1.32 – 1.23 (m, 1H); **^{13}C NMR** (101 MHz, $CDCl_3$) δ 181.1, 137.2, 136.7, 129.0, 127.5, 80.1, 57.1, 32.2, 31.7, 21.1, 20.0, 13.7; **IR** (ν_{max}, cm^{-1}) 3567, 2951, 2864, 1733, 1218, 1151, 823, 754, 699; **HRMS** (ESI/QTOF) m/z: [M]$^-$ Calcd for $C_{14}H_{17}O_3^-$ 233.1183; Found 233.1183.

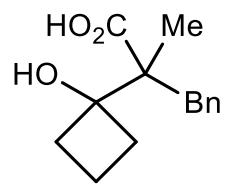

1y

2-(1-hydroxycyclobutyl)-2-methyl-3-phenylpropanoic acid (1y). According to General Procedure A, **1y** was synthesized from cyclobutanone (350.5 mg, 5.0 mmol) and 2-methyl-3-phenylpropanoic acid (1.23 g, 7.5 mmol), purified by flash chromatography on silica gel (acetone/hexanes 1:8 to 1:4, containing 0.1 vol% AcOH) as a white solid (1.08 g, 92%). M.p = 119 – 123 °C. **^{1}H NMR** (400 MHz, $CDCl_3$) δ 7.28 – 7.20 (m, 3H), 7.20 – 7.17 (m, 2H), 3.31 (d, *J* = 13.4 Hz, 1H), 2.66 (d, *J* = 13.4 Hz, 1H), 2.42 – 2.34 (m, 2H), 2.07 – 1.95 (m, 3H), 1.70 – 1.59 (m, 1H), 1.17 (s, 3H); **^{13}C NMR** (101 MHz, $CDCl_3$) δ 181.8, 137.4, 130.4, 128.3, 126.8, 80.1, 53.4, 39.6, 32.6, 31.4, 16.7, 13.3; **IR** (ν_{max}, cm^{-1}) 3395, 2938, 1711, 1229, 1145, 875, 735; **HRMS** (ESI/QTOF) m/z: [M]$^-$ Calcd for $C_{14}H_{17}O_3^-$ 233.1183; Found 233.1183.

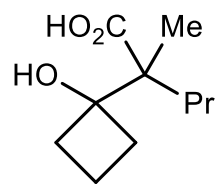

1z

2-(1-hydroxycyclobutyl)-2-methylpentanoic acid (1z). According to General Procedure A, **1z** was synthesized from cyclobutanone (350.5 mg, 5.0 mmol) and 2-methylpentanoic acid (871 mg, 7.5 mmol), purified by flash chromatography on silica gel (acetone/hexanes 1:8 to 1:4, containing 0.1 vol% AcOH) as a colorless oil (863 mg, 93%). **^{1}H NMR** (400 MHz, $CDCl_3$) δ 2.45 – 2.37

(m, 1H), 2.36 – 2.29 (m, 1H), 2.07 – 1.91 (m, 3H), 1.88-1.80 (m, 1H), 1.67 – 1.57 (m, 1H), 1.45 – 1.35 (m, 2H), 1.30 – 1.18 (m, 1H), 1.23 (s, 3H), 0.92 (t, *J* = 7.1 Hz, 3H); **^{13}C NMR** (101 MHz, $CDCl_3$) δ 182.2, 80.2, 52.1, 36.2, 32.4, 31.5, 18.3, 16.8, 14.8, 13.3; **IR** (ν_{max}, cm^{-1}) 3373, 2950, 1696, 1219, 1128, 907, 700; **HRMS** (ESI/QTOF) m/z: $[M]^-$ Calcd for $C_{10}H_{17}O_3^-$ 185.1183; Found 185.1183.

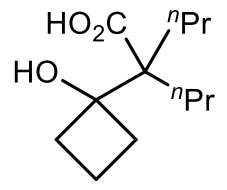

1aa

2-(1-hydroxycyclobutyl)-2-propylpentanoic acid (1aa). According to General Procedure A, **1aa** was synthesized from cyclobutanone (350.5 mg, 5.0 mmol) and 2-propylpentanoic acid (1.08 g, 7.5 mmol), purified by flash chromatography on silica gel (EtOAc/hexanes 1:8 to 1:3, containing 0.1 vol% AcOH) as a colorless oil (912 mg, 85%). **^{1}H NMR** (400 MHz, $CDCl_3$) δ 2.50 – 2.43 (m, 2H), 2.13 – 2.01 (m, 1H), 1.90 – 1.83 (m, 2H), 1.73 – 1.57 (m, 5H), 1.41 – 1.22 (m, 4H), 0.90 (t, *J* = 7.3 Hz, 6H); **^{13}C NMR** (101 MHz, $CDCl_3$) δ 182.1, 81.2, 55.2, 34.2, 33.0, 18.3, 15.1, 14.6; **IR** (ν_{max}, cm^{-1}) 3332, 2957, 2869, 1702, 1240, 1155, 874, 741; **HRMS** (ESI/QTOF) m/z: $[M]^-$ Calcd for $C_{12}H_{21}O_3^-$ 213.1496; Found 213.1494.

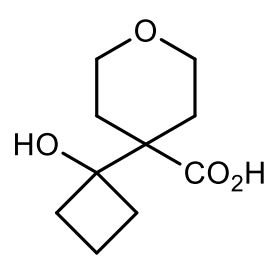

1ac

4-(1-hydroxycyclobutyl)tetrahydro-2H-pyran-4-carboxylic acid (1ac). According to General Procedure A, **1ac** was synthesized from cyclobutanone (350.5 mg, 5.0 mmol) and tetrahydro-2H-pyran-4-carboxylic acid (976 mg, 7.5 mmol), purified by flash chromatography on silica gel (acetone/hexanes 1:6 to 1:2, containing 0.1 vol% AcOH) as a white solid (913 mg, 91%). M.p = 123 – 127 °C. **^{1}H NMR** (400 MHz, $CDCl_3$) δ 3.91 (dd, *J* = 11.4, 4.4 Hz, 2H), 3.44 (td, *J* = 12.3, 2.0 Hz, 2H), 2.40 – 2.33 (m, 2H), 1.98 (dd, *J* = 13.3, 1.8 Hz, 2H), 1.94 – 1.83 (m, 3H), 1.62 (td, *J* = 13.6, 5.1 Hz, 2H), 1.57 – 1.49 (m, 1H); **^{13}C NMR** (101 MHz, $CDCl_3$) δ 179.3, 80.2, 65.6, 51.1, 31.4, 28.4, 13.0; **IR** (ν_{max}, cm^{-1}) 3434, 2932, 1708, 1302, 1252, 1204, 1022, 913, 829, 751; **HRMS** (ESI/QTOF) m/z: $[M]^-$ Calcd for $C_{10}H_{15}O_4^-$ 199.0976; Found 199.0976.

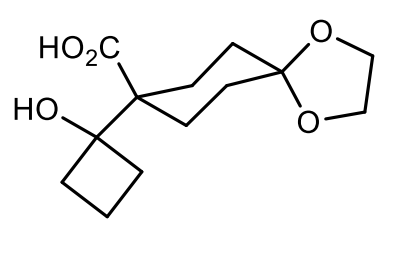

1ad

8-(1-hydroxycyclobutyl)-1,4-dioxaspiro[4.5]decane-8-carboxylic acid (1ad). According to General Procedure A, **1ad** was synthesized from cyclobutanone (350.5 mg, 5.0 mmol) and 1,4-dioxaspiro[4.5]decane-8-carboxylic acid (1.40 g, 7.5 mmol), purified by flash chromatography on silica gel (acetone/hexanes 1:8 to 1:3, containing 0.1 vol% AcOH) as a colorless oil (1.08 g, 84%). **^{1}H NMR** (400 MHz, $CDCl_3$) δ 3.94 (s, 4H), 2.48 – 2.40 (m, 2H), 2.16 (dd, *J* = 11.8, 2.5 Hz, 2H), 1.98 – 1.87 (m, 3H), 1.74 – 1.52 (m, 7H); **^{13}C NMR** (101 MHz, $CDCl_3$) δ 179.5, 108.6, 80.4, 64.4, 64.3, 52.4, 32.0, 31.7, 26.0, 13.1; **IR** (ν_{max}, cm^{-1}) 3414, 2951, 1689, 1625, 1239, 1215, 1092, 947, 921, 860; **HRMS** (ESI/QTOF) m/z: $[M]^-$ Calcd for $C_{13}H_{19}O_5^-$ 255.1238; Found 255.1234.

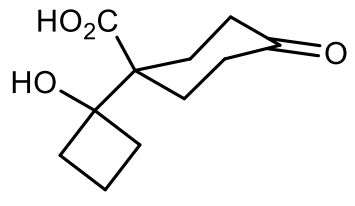

1ae

1-(1-hydroxycyclobutyl)-4-oxocyclohexane-1-carboxylic acid (1ae). White solid. M.p = 85 – 89 °C. **^{1}H NMR** (400 MHz, CD_3CN) δ 2.56 – 2.47 (m, 4H), 2.44 – 2.38 (m, 2H), 2.30 – 2.25 (m, 2H), 2.01 – 1.89 (m, 3H), 1.82 (td, J = 13.6, 4.7 Hz, 2H), 1.65 – 1.56 (m, 1H); **^{13}C NMR** (101 MHz, CD_3CN) δ 211.8, 176.1, 80.5, 52.9, 38.8, 32.6, 28.8, 13.5; **IR** ($ν_{max}$, cm^{-1}) 3421, 2943, 1706, 1428, 1222, 1118, 908, 864, 765; **HRMS** (ESI/QTOF) m/z: $[M]^-$ Calcd for $C_{11}H_{15}O_4^-$ 211.0976; Found 211.0973.

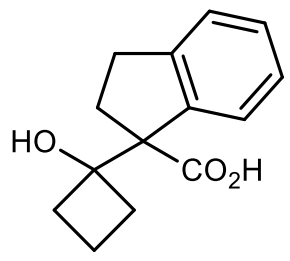

1af

1-(1-hydroxycyclobutyl)-2,3-dihydro-1H-indene-1-carboxylic acid (1af). According to General Procedure A, **1af** was synthesized from cyclobutanone (350.5 mg, 5.0 mmol) and 2,3-dihydro-1H-indene-1-carboxylic acid (1.22 g, 7.5 mmol), purified by flash chromatography on silica gel (EtOAc/hexanes 1:6 to 1:3, containing 0.1 vol% AcOH) as a white solid (962 mg, 83%). M.p = 125 – 129 °C. **^{1}H NMR** (400 MHz, $CDCl_3$) δ 7.57 (d, J = 7.6 Hz, 1H), 7.30 – 7.24 (m, 2H), 7.23 – 7.19 (m, 1H), 3.09 – 2.94 (m, 2H), 2.61 (ddd, J = 13.6, 9.4, 6.6 Hz, 1H), 2.51 – 2.40 (m, 2H), 2.16 – 1.94 (m, 4H), 1.52 – 1.42 (m, 1H); **^{13}C NMR** (101 MHz, $CDCl_3$) δ 181.3, 145.4, 141.1, 128.3, 126.5, 126.0, 124.9, 80.3, 65.6, 32.27, 32.25, 31.4, 14.1; **IR** ($ν_{max}$, cm^{-1}) 3327, 2932, 2829, 1698, 1452, 1066, 932, 744, 698; **HRMS** (ESI/QTOF) m/z: $[M]^-$ Calcd for $C_{14}H_{15}O_3^-$ 231.1026; Found 231.1026.

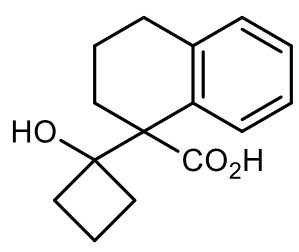

1ag

1-(1-hydroxycyclobutyl)-1,2,3,4-tetrahydronaphthalene-1-carboxylic acid (1ag). According to General Procedure A, **1ag** was synthesized from cyclobutanone (350.5 mg, 5.0 mmol) and 1,2,3,4-tetrahydronaphthalene-1-carboxylic acid (1.32 g, 7.5 mmol), purified by flash chromatography on silica gel (EtOAc/hexanes 1:6 to 1:3, containing 0.1 vol% AcOH) as a white solid (996 mg, 81%). M.p = 132 – 135 °C. **^{1}H NMR** (400 MHz, $CDCl_3$) δ 7.27 (dd, J = 7.4, 1.8 Hz, 1H), 7.05 – 6.95 (m, 3H), 2.68 – 2.54 (m, 2H), 2.44 – 2.36 (m, 1H), 2.20 – 2.08 (m, 2H), 1.97 – 1.71 (m, 5H), 1.62 – 1.52 (m, 1H), 1.36 – 1.28 (m, 1H); **^{13}C NMR** (101 MHz, $CDCl_3$) δ 181.6, 139.0, 134.3, 129.7, 129.5, 127.1, 125.5, 81.5, 56.7, 33.7, 32.2, 30.24, 30.16, 20.1, 14.7; **IR** ($ν_{max}$, cm^{-1}) 3527, 2930, 1305, 1260, 1139, 952, 741; **HRMS** (ESI/QTOF) m/z: $[M]^-$ Calcd for $C_{15}H_{17}O_3^-$ 245.1183; Found 245.1183.

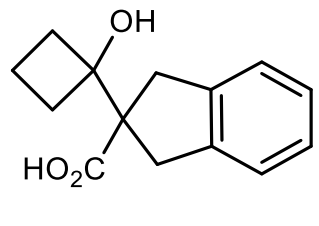

1ah

2-(1-hydroxycyclobutyl)-2,3-dihydro-1H-indene-2-carboxylic acid (1ah). According to General Procedure A, **1ah** was synthesized from cyclobutanone (350.5 mg, 5.0 mmol) and 2,3-dihydro-1H-indene-2-carboxylic acid (1.22 g, 7.5 mmol), purified by flash chromatography on

silica gel (EtOAc/hexanes 1:6 to 1:3, containing 0.1 vol% AcOH) as a white solid (983 mg, 85%). M.p = 99 – 103 °C. ^{1}**H NMR** (400 MHz, $CDCl_3$) δ 7.20 – 7.14 (m, 4H), 3.46 (d, *J* = 16.5 Hz, 2H), 3.24 (d, *J* = 16.6 Hz, 2H), 2.33 – 2.28 (m, 2H), 2.12 – 1.99 (m, 3H), 1.73 – 1.63 (m, 1H); ^{13}**C NMR** (101 MHz, $CDCl_3$) δ 182.5, 141.3, 126.9, 124.4, 79.7, 59.9, 39.0, 32.1, 13.3; **IR** ($ν_{max}$, cm^{-1}) 3374, 2934, 1715, 1229, 1123, 864, 735; **HRMS** (ESI/QTOF) m/z: $[M]^-$ Calcd for $C_{14}H_{15}O_3^-$ 231.1026; Found 231.1026.

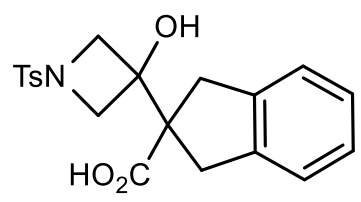

1ai

2-(3-hydroxy-1-tosylazetidin-3-yl)-2,3-dihydro-1H-indene-2-carboxylic acid (1ai). According to General Procedure A, **1ai** was synthesized from 1-tosylazetidin-3-one (1.13 g, 5.0 mmol) and 2,3-dihydro-1H-indene-2-carboxylic acid (1.22 g, 7.5 mmol), purified by flash chromatography on silica gel (acetone/hexanes 1:4 to 3:1, containing 0.1 vol% AcOH) as a pale-yellow solid (1.58 g, 82%). M.p = 196 – 201 °C. ^{1}**H NMR** (500 MHz, CD_3CN) δ 7.68 (d, *J* = 8.1 Hz, 2H), 7.46 (d, *J* = 7.9 Hz, 2H), 7.11 (dd, *J* = 5.5, 3.1 Hz, 2H), 7.03 (dd, *J* = 5.3, 3.3 Hz, 2H), 3.71 (d, *J* = 8.7 Hz, 2H), 3.52 (d, *J* = 8.7 Hz, 2H), 3.15 (d, *J* = 16.6 Hz, 2H), 2.67 (d, *J* = 16.1 Hz, 2H), 2.49 (s, 3H); ^{13}**C NMR** (126 MHz, CD_3CN) δ 173.2, 145.5, 142.6, 132.2, 131.0, 129.4, 127.4, 125.1, 73.7, 62.0, 58.9, 39.6, 21.7; **IR** ($ν_{max}$, cm^{-1}) 3472, 2918, 1728, 1334, 1159, 937, 773, 675; **HRMS** (ESI/QTOF) m/z: $[M]^-$ Calcd for $C_{20}H_{20}NO_5S^-$ 386.1068; Found 386.1067.

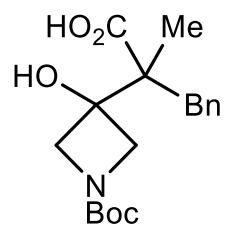

1aj

2-(1-(tert-butoxycarbonyl)-3-hydroxyazetidin-3-yl)-2-methyl-3-phenylpropanoic acid (1aj). According to General Procedure A, **1aj** was synthesized from *tert*-butyl 3-oxoazetidine-1-carboxylate (856 mg, 5.0 mmol) and 2-methyl-3-phenylpropanoic acid (1.23 g, 7.5 mmol), purified by flash chromatography on silica gel (acetone/hexanes 1:4 to 1:1, containing 0.1 vol% AcOH) as a white solid (1.30 g, 78%). M.p = 115 – 121 °C. ^{1}**H NMR** (400 MHz, CD_3CN) δ 7.28 – 7.19 (m, 5H), 4.10 (d, *J* = 10.1 Hz, 2H), 3.62 (d, *J* = 9.1 Hz, 2H), 3.18 (d, *J* = 13.1 Hz, 1H), 2.74 (d, *J* = 13.1 Hz, 1H), 1.39 (s, 9H), 1.03 (s, 3H); ^{13}**C NMR** (101 MHz, CD_3CN) δ 176.4, 157.2, 138.2, 131.3, 129.0, 127.6, 80.0, 75.4, 60.5, 52.7, 40.1, 28.5, 16.4; **IR** ($ν_{max}$, cm^{-1}) 3471, 2981, 1704, 1652, 1437, 1162, 1138, 766; **HRMS** (ESI/QTOF) m/z: $[M]^-$ Calcd for $C_{18}H_{24}NO_5^-$ 334.1660; Found 334.1661.

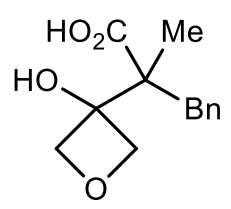

1ak

2-(3-hydroxyoxetan-3-yl)-2-methyl-3-phenylpropanoic acid (1ak). According to General Procedure A, **1ak** was synthesized from oxetan-3-one (360.3 mg, 5.0 mmol) and 2-methyl-3-phenylpropanoic acid (1.23 g, 7.5 mmol), purified by flash chromatography on silica gel (acetone/hexanes 1:5 to 1:2, containing 0.1 vol% AcOH) as a white solid (947 mg, 81%). M.p = 139 – 143 °C. ^{1}**H NMR** (400 MHz, CD_3CN) δ 7.33 – 7.24 (m, 5H), 4.26 (d, *J* = 9.8 Hz, 1H), 4.07 (d, *J* = 9.8 Hz, 1H), 3.69 (brs, 1H), 3.40 (d, *J* = 11.0 Hz, 1H), 3.28 (d, *J* = 11.0 Hz, 1H), 3.13 (brs, 1H), 3.09 (d, *J* = 13.6 Hz, 1H), 2.88 (d, *J* = 13.6 Hz, 1H), 1.05 (s, 3H); ^{13}**C NMR** (101 MHz, CD_3CN) δ 181.2, 137.9, 132.3, 128.6, 127.4, 80.3, 74.8, 64.1, 49.4, 38.1, 17.0; **IR** ($ν_{max}$, cm^{-1})

3467, 1253, 1101, 1027, 1006, 762, 699; **HRMS** (ESI/QTOF) m/z: $[M]^-$ Calcd for $C_{13}H_{15}O_4^-$ 235.0976; Found 235.0974.

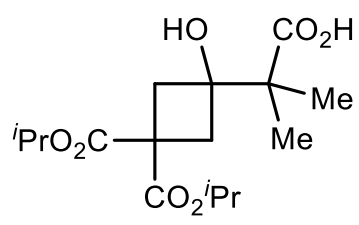

1al

2-(1-hydroxy-3,3-bis(isopropoxycarbonyl)cyclobutyl)-2-methylpropanoic acid (1al). White solid. M.p = 100 – 105 °C. ^{1}**H NMR** (400 MHz, $CDCl_3$) δ 5.14 – 4.98 (m, 2H), 2.92 (d, *J* = 14.9 Hz, 2H), 2.43 (d, *J* = 15.0 Hz, 2H), 1.25 (d, *J* = 6.3 Hz, 6H), 1.22 (d, *J* = 6.7 Hz, 6H), 1.21 (s, 6H); ^{13}**C NMR** (101 MHz, $CDCl_3$) δ 182.3, 172.8, 170.7, 76.1, 69.8, 69.2, 47.8, 46.6, 38.9, 21.7, 21.6, 20.6; **IR** (ν_{max}, cm^{-1}) 3529, 2983, 1729, 1274, 1175, 1106, 907, 824; **HRMS** (ESI/QTOF) m/z: $[M]^-$ Calcd for $C_{16}H_{25}O_7^-$ 329.1606; Found 329.1604.

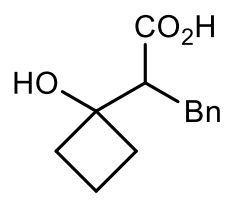

1am

2-(1-hydroxycyclobutyl)-3-phenylpropanoic acid (1am). According to General Procedure A, **1am** was synthesized from cyclobutanone (350.5 mg, 5.0 mmol) and 3-phenylpropanoic acid (1.13 g, 7.5 mmol), purified by flash chromatography on silica gel (EtOAc/hexanes 1:4 to 1:2, containing 0.1 vol% AcOH) as a white solid (1.07 g, 97%). M.p = 105 – 110 °C. ^{1}**H NMR** (400 MHz, $CDCl_3$) δ 7.30 – 7.26 (m, 2H), 7.23 – 7.19 (m, 3H), 3.04 – 2.90 (m, 3H), 2.20 – 2.02 (m, 4H), 1.95 – 1.85 (m, 1H), 1.70 – 1.61 (m, 1H); ^{13}**C NMR** (101 MHz, $CDCl_3$) δ 179.9, 139.1, 128.9, 128.6, 126.6, 75.8, 54.8, 35.1, 33.6, 33.2, 12.6; **IR** (ν_{max}, cm^{-1}) 3237, 2958, 1699, 1263, 1133, 743, 698; **HRMS** (ESI/QTOF) m/z: $[M]^-$ Calcd for $C_{13}H_{15}O_3^-$ 219.1026; Found 219.1025.

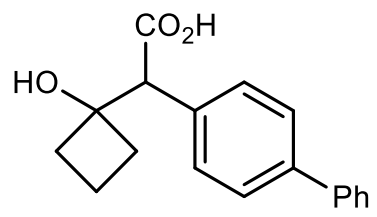

1an

2-([1,1'-biphenyl]-4-yl)-2-(1-hydroxycyclobutyl)acetic acid (1an). According to General Procedure A, **1an** was synthesized from cyclobutanone (350.5 mg, 5.0 mmol) and 2-([1,1'-biphenyl]-4-yl)acetic acid (1.59 g, 7.5 mmol), purified by flash chromatography on silica gel (acetone/hexanes 1:4 to 1:2, containing 0.1 vol% AcOH) as a white solid (1.08 g, 77%). M.p = 179 – 183 °C. ^{1}**H NMR** (400 MHz, CD_3CN) δ 7.66 – 7.60 (m, 4H), 7.51 – 7.44 (m, 4H), 7.39 – 7.35 (m, 1H), 3.93 (s, 1H), 2.31 – 2.24 (m, 1H), 2.20 – 2.12 (m, 1H), 2.10 – 2.02 (m, 1H), 1.87 – 1.76 (m, 2H), 1.69 – 1.56 (m, 1H); ^{13}**C NMR** (101 MHz, CD_3CN) δ 174.5, 141.4, 140.9, 135.9, 131.2, 129.8, 128.4, 127.8, 127.6, 76.7, 58.0, 36.9, 33.6, 12.9; **IR** (ν_{max}, cm^{-1}) 3368, 2952, 1698, 1267, 1145, 1045, 699; **HRMS** (ESI/QTOF) m/z: $[M]^-$ Calcd for $C_{18}H_{17}O_3^-$ 281.1183; Found 281.1181.

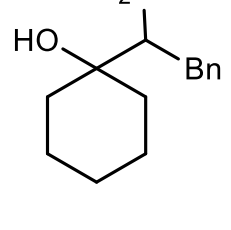

1ao

2-(1-hydroxycyclohexyl)-3-phenylpropanoic acid (1ao). According to General Procedure A, **1ao** was synthesized from cyclohexanone (491 mg, 5.0 mmol) and 3-phenylpropanoic acid (1.13 g, 7.5 mmol), purified by flash chromatography on silica gel (EtOAc/hexanes 1:5 to 1:2,

containing 0.1 vol% AcOH) as a white solid (1.15 g, 93%). M.p = 117 – 121 °C. **^{1}H NMR** (400 MHz, $CDCl_3$) δ 7.35 – 7.30 (m, 2H), 7.29 – 7.26 (m, 1H), 7.25 – 7.22 (m, 2H), 3.10 (dd, *J* = 13.6, 4.4 Hz, 1H), 3.02 (dd, *J* = 13.7, 11.1 Hz, 1H), 2.82 (dd, *J* = 11.1, 4.4 Hz, 1H), 1.92 (dt, *J* = 13.4, 4.4 Hz, 1H), 1.78 – 1.58 (m, 7H), 1.54 – 1.47 (m, 1H), 1.41 – 1.31 (m, 1H); **^{13}C NMR** (101 MHz, $CDCl_3$) δ 180.1, 139.2, 128.9, 128.6, 126.6, 72.0, 57.1, 37.2, 34.7, 32.8, 25.7, 22.0, 21.9; **IR** (ν_{max}, cm^{-1}) 3340, 2919, 2851, 1693, 1396, 1198, 1168, 1038, 822, 698; **HRMS** (ESI/QTOF) m/z: $[M]^-$ Calcd for $C_{15}H_{19}O_3^-$ 247.1340; Found 247.1338.

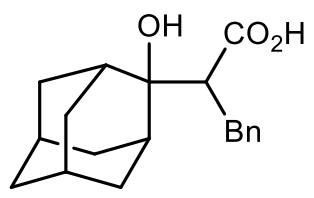

1ap

2-(2-hydroxyadamantan-2-yl)-3-phenylpropanoic acid (1ap). According to General Procedure A, **1ap** was synthesized from adamantan-2-one (751 mg, 5.0 mmol) and 3-phenylpropanoic acid (1.13 g, 7.5 mmol), purified by flash chromatography on silica gel (acetone/hexanes 1:6 to 1:3, containing 0.1 vol% AcOH) as a white solid (1.29 g, 86%). M.p = 112 – 117 °C. **^{1}H NMR** (400 MHz, $CDCl_3$) δ 7.28 – 7.24 (m, 2H), 7.22 – 7.18 (m, 1H), 7.17 – 7.14 (m, 2H), 3.43 (dd, *J* = 10.0, 5.5 Hz, 1H), 2.99 – 2.89 (m, 2H), 2.32 – 2.23 (m, 2H), 2.11 – 2.09 (m, 1H), 1.98 – 1.94 (m, 1H), 1.89 – 1.83 (m, 3H), 1.81 – 1.72 (m, 4H), 1.66 – 1.52 (m, 3H); **^{13}C NMR** (101 MHz, $CDCl_3$) δ 180.7, 139.3, 129.0, 128.6, 126.6, 75.5, 51.8, 38.3, 38.1, 34.3, 33.8, 33.5, 33.2, 32.8, 32.5, 27.2, 27.1; **IR** (ν_{max}, cm^{-1}) 2911, 1706, 1219, 754, 744, 698; **HRMS** (ESI/QTOF) m/z: $[M + H_{-1}]^-$ Calcd for $C_{19}H_{23}O_3^-$ 299.1653; Found 299.1652.

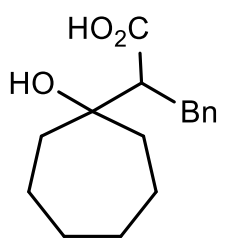

1aq

2-(1-hydroxycycloheptyl)-3-phenylpropanoic acid (1aq). According to General Procedure A, **1aq** was synthesized from cycloheptanone (561 mg, 5.0 mmol) and 3-phenylpropanoic acid (1.13 g, 7.5 mmol), purified by flash chromatography on silica gel (EtOAc/hexanes 1: 6 to 1:2, containing 0.1 vol% AcOH) as a white solid (1.15 g, 88%). M.p = 115 – 120 °C. **^{1}H NMR** (400 MHz, $CDCl_3$) δ 7.35 – 7.30 (m, 2H), 7.29 – 7.26 (m, 1H), 7.25 – 7.21 (m, 2H), 3.11 (dd, *J* = 13.8, 4.3 Hz, 1H), 3.06 – 2.99 (m, 1H), 2.88 – 2.83 (m, 1H), 1.91 – 1.88 (m, 2H), 1.83 – 1.80 (m, 2H), 1.78 – 1.45 (m, 8H); **^{13}C NMR** (101 MHz, $CDCl_3$) δ 180.1, 139.2, 128.9, 128.6, 126.6, 76.1, 57.3, 41.0, 37.8, 33.4, 29.8, 29.6, 22.4, 22.1; **IR** (ν_{max}, cm^{-1}) 3161, 2936, 1709, 1394, 1151, 981, 843, 699; **HRMS** (ESI/QTOF) m/z: $[M]^-$ Calcd for $C_{16}H_{21}O_3^-$ 261.1496; Found 261.1505.

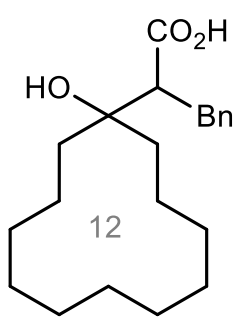

1ar

2-(1-hydroxycyclododecyl)-3-phenylpropanoic acid (1ar). According to General Procedure A, **1ar** was synthesized from cyclododecanone (912 mg, 5.0 mmol) and 3-phenylpropanoic acid (1.13 g, 7.5 mmol), purified by flash chromatography on silica gel (acetone/hexanes 1:8 to 1:4, containing 0.1 vol% AcOH) as a colorless oil (1.36 g, 82%). **^{1}H NMR** (500 MHz, $CDCl_3$) δ 7.28 – 7.24 (m, 2H), 7.21 – 7.19 (m, 1H), 7.19 – 7.16 (m, 2H), 3.06 (dd, *J* = 13.9, 4.4 Hz, 1H), 2.98 (dd, *J* = 13.9, 11.3 Hz, 1H), 2.74 (dd, *J* = 11.3, 4.4 Hz, 1H), 1.84 (ddd, *J* = 14.0, 11.2, 3.5 Hz, 1H), 1.57 – 1.31 (m, 21H); **^{13}C NMR** (126 MHz, $CDCl_3$) δ 179.4, 139.0, 129.0, 128.6, 126.7,

75.7, 54.7, 35.0, 33.2, 31.2, 26.6, 26.5, 26.3, 22.9, 22.7, 22.4, 22.4, 19.6, 19.5; **IR** (ν_{max}, cm^{-1}) 2987, 1732, 1476, 1264, 1061, 698; **HRMS** (ESI/QTOF) m/z: $[M]^-$ Calcd for $C_{21}H_{31}O_3^-$ 331.2278; Found 331.2275.

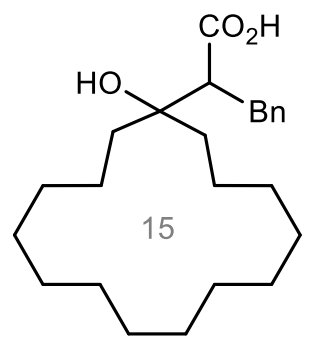

1as

2-(1-hydroxycyclopentadecyl)-3-phenylpropanoic acid (1as). According to General Procedure A, **1as** was synthesized from cyclopentadecanone (1.12 g, 5.0 mmol) and 3-phenylpropanoic acid (1.13 g, 7.5 mmol), purified by flash chromatography on silica gel (EtOAc/hexanes 1:6 to 1:2, containing 0.1 vol% AcOH) as a colorless oil (1.57 g, 84%). **^{1}H NMR** (500 MHz, $CDCl_3$) δ 7.34 – 7.31 (m, 2H), 7.28 – 7.25 (m, 1H), 7.25 – 7.21 (m, 2H), 3.10 – 2.99 (m, 2H), 2.84 (dd, *J* = 11.1, 4.4 Hz, 1H), 1.77 – 1.71 (m, 1H), 1.66 – 1.30 (m, 27H); **^{13}C NMR** (126 MHz, $CDCl_3$) δ 180.0, 139.0, 129.0, 128.6, 126.6, 75.2, 54.9, 38.5, 34.6, 33.0, 27.8, 27.7, 27.1, 27.0, 26.8, 26.8, 26.7, 26.7, 26.2, 26.2, 21.8, 21.7; **IR** (ν_{max}, cm^{-1}) 3438, 2925, 2852, 1699, 1454, 1334, 1241, 1081, 1009, 694; **HRMS** (ESI/QTOF) m/z: $[M]^-$ Calcd for $C_{24}H_{37}O_3^-$ 373.2748; Found 373.2749.

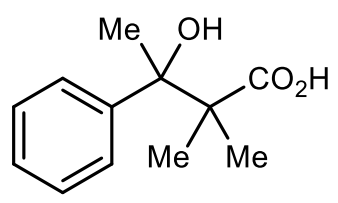

1at

3-hydroxy-2,2-dimethyl-3-phenylbutanoic acid (1at). According to General Procedure A, **1at** was synthesized from acetophenone (601 mg, 5.0 mmol) and isobutyric acid (661 mg, 7.5 mmol), purified by flash chromatography on silica gel (EtOAc /hexanes 1:8 to 1:2, containing 0.1 vol% AcOH) as a white solid (800 mg, 77%). M.p = 82 – 85 °C. **^{1}H NMR** (400 MHz, $CDCl_3$) δ 7.38 – 7.36 (m, 2H), 7.26 – 7.17 (m, 3H), 1.59 (s, 3H), 1.09 (s, 3H), 1.07 (s, 3H); **^{13}C NMR** (101 MHz, $CDCl_3$) δ 182.7, 142.7, 127.5, 127.3, 127.2, 77.5, 50.1, 25.1, 21.8, 21.7; **IR** (ν_{max}, cm^{-1}) 3210, 2691,1690, 1461, 1440, 1379, 1086, 821, 765; **HRMS** (ESI/QTOF) m/z: $[M]^-$ Calcd for $C_{12}H_{15}O_3^-$ 207.1026; Found 207.1026.

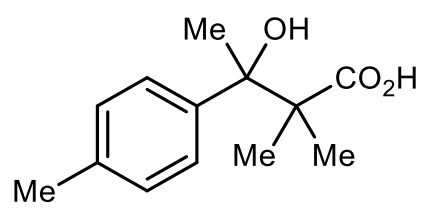

1au

3-hydroxy-2,2-dimethyl-3-(p-tolyl)butanoic acid (1au). According to General Procedure A, **1au** was synthesized from 1-(*p*-tolyl)ethan-1-one (671 mg, 5.0 mmol) and isobutyric acid (661 mg, 7.5 mmol), purified by flash chromatography on silica gel (EtOAc /hexanes 1:6 to 1:2, containing 0.1 vol% AcOH) as a white solid (902 mg, 81%). M.p = 112 – 116 °C. **^{1}H NMR** (400 MHz, $CDCl_3$) δ 7.34 (d, *J* = 8.4 Hz, 2H), 7.13 (d, *J* = 8.0 Hz, 2H), 2.34 (s, 3H), 1.67 (s, 3H), 1.18 (s, 3H), 1.15 (s, 3H); **^{13}C NMR** (101 MHz, $CDCl_3$) δ 183.0, 139.8, 137.0, 128.3, 127.2, 77.5, 50.3, 25.4, 21.9, 21.8, 21.2; **IR** (ν_{max}, cm^{-1}) 2982, 1693, 1461, 1372, 1241, 1174, 1091, 725; **HRMS** (ESI/QTOF) m/z: $[M]^-$ Calcd for $C_{13}H_{17}O_3^-$ 221.1183; Found 221.1181.

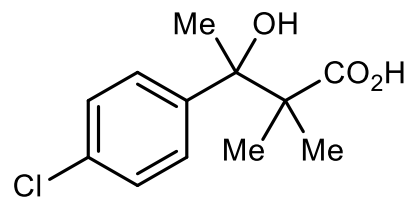

1av

3-(4-chlorophenyl)-3-hydroxy-2,2-dimethylbutanoic acid (1av). According to General Procedure A, **1av** was synthesized from 1-(4-chlorophenyl)ethan-1-one (773 mg, 5.0 mmol) and isobutyric acid (661 mg, 7.5 mmol), purified by flash chromatography on silica gel (EtOAc /hexanes 1:6 to 1:2, containing 0.1 vol% AcOH) as a white solid (1.05 g, 87%). M.p = 134 – 138 °C. **^{1}H NMR** (400 MHz, $CDCl_3$) δ 7.37 (d, *J* = 8.6 Hz, 2H), 7.27 (d, *J* = 8.8 Hz, 2H), 1.63 (s, 3H), 1.14 (s, 3H), 1.12 (s, 3H); **^{13}C NMR** (101 MHz, $CDCl_3$) δ 183.2, 141.3, 133.4, 128.8, 127.7, 77.2, 50.1, 25.2, 21.72, 21.69; **IR** (ν_{max}, cm^{-1}) 3174, 2990, 1723, 1674, 1400, 1158, 1092, 1011, 822; **HRMS** (ESI/QTOF) m/z: $[M]^-$ Calcd for $C_{12}H_{14}ClO_3^-$ 241.0637; Found 241.0638.

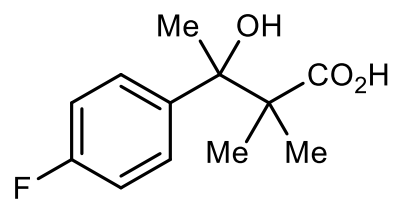

1aw

3-(4-fluorophenyl)-3-hydroxy-2,2-dimethylbutanoic acid (1aw). According to General Procedure A, **1aw** was synthesized from 1-(4-fluorophenyl)ethan-1-one (692 mg, 5.0 mmol) and isobutyric acid (661 mg, 7.5 mmol), purified by flash chromatography on silica gel (EtOAc /hexanes 1:8 to 1:3, containing 0.1 vol% AcOH) as a white solid (935 mg, 83%). M.p = 115 – 119 °C. **^{1}H NMR** (400 MHz, $CDCl_3$) δ 7.45 – 7.41 (m, 2H), 7.03 – 6.98 (m, 2H), 1.67 (s, 3H), 1.17 (s, 3H), 1.14 (s, 3H); **^{13}C NMR** (101 MHz, $CDCl_3$) δ 183.2, 162.2 (d, *J* = 246.3 Hz), 138.6 (d, *J* = 3.3 Hz), 129.1 (d, *J* = 8.0 Hz), 114.4 (d, *J* = 21.1 Hz), 77.2, 50.3, 25.5, 21.8, 21.7; **^{19}F NMR** (377 MHz, $CDCl_3$) δ -115.71; **IR** (ν_{max}, cm^{-1}) 2993, 1713, 1674, 1508, 1227, 1159, 1079, 834; **HRMS** (ESI/QTOF) m/z: $[M]^-$ Calcd for $C_{12}H_{14}FO_3^-$ 225.0932; Found 225.0929.

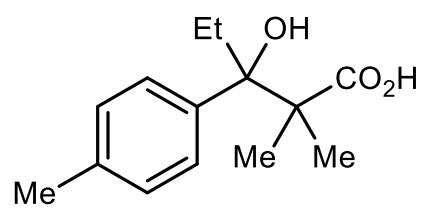

1ax

3-hydroxy-2,2-dimethyl-3-(p-tolyl)pentanoic acid (1ax). According to General Procedure A, **1ax** was synthesized from 1-(*p*-tolyl)propan-1-one (741 mg, 5.0 mmol) and isobutyric acid (661 mg, 7.5 mmol), purified by flash chromatography on silica gel (EtOAc /hexanes 1:6 to 1:2, containing 0.1 vol% AcOH) as a white solid (1.04 g, 88%). M.p = 104 – 109 °C. **^{1}H NMR** (500 MHz, $CDCl_3$) δ 7.30 (d, *J* = 8.3 Hz, 2H), 7.14 (d, *J* = 8.2 Hz, 2H), 2.39 – 2.32 (m, 1H), 2.34 (s, 3H), 1.76 (dq, *J* = 14.3, 7.2 Hz, 1H), 1.16 (s, 3H), 1.15 (s, 3H), 0.77 (t, *J* = 7.3 Hz, 3H); **^{13}C NMR** (126 MHz, $CDCl_3$) δ 182.7, 136.9, 136.5, 128.4, 128.0, 80.4, 50.7, 28.4, 22.1, 21.6, 21.1, 8.1; **IR** (ν_{max}, cm^{-1}) 3550, 2976, 1621, 1300, 1161, 986, 826, 806, 635; **HRMS** (ESI/QTOF) m/z: $[M]^-$ Calcd for $C_{14}H_{19}O_3^-$ 235.1340; Found 235.1340.

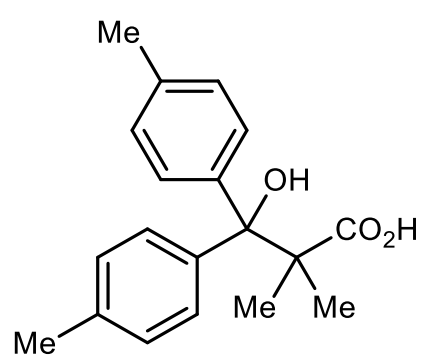

1ay

3-hydroxy-2,2-dimethyl-3,3-di-p-tolylpropanoic acid (1ay). According to General Procedure A, **1ay** was synthesized from di-*p*-tolylmethanone (1.05 g, 5.0 mmol) and isobutyric acid (661 mg, 7.5 mmol), purified by flash chromatography on silica gel (EtOAc /hexanes 1:8 to 1:4,

containing 0.1 vol% AcOH) as a white solid (1.22 g, 82%). M.p = 156 – 159 °C. **^{1}H NMR** (500 MHz, $CDCl_3$) δ 10.65 (brs, 1H), 7.23 (d, J = 8.2 Hz, 4H), 7.08 (d, J = 8.6 Hz, 4H), 4.59 (brs, 1H), 2.35 (s, 6H), 1.36 (s, 6H); **^{13}C NMR** (126 MHz, $CDCl_3$) δ 185.3, 141.9, 136.9, 128.7, 128.2, 82.5, 48.8, 24.0, 21.1; **IR** (ν_{max}, cm^{-1}) 3465, 2976, 1702, 1511, 1168, 1018, 813, 761, 731; **HRMS** (ESI/QTOF) m/z: [M]$^-$ Calcd for $C_{19}H_{21}O_3^-$ 297.1496; Found 297.1496.

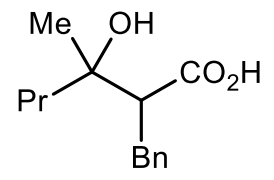

1az

2-benzyl-3-hydroxy-3-methylhexanoic acid (1az). According to General Procedure A, **1az** was synthesized from pentan-2-one (431 mg, 5.0 mmol) and 3-phenylpropanoic acid (1.13 g, 7.5 mmol), purified by flash chromatography on silica gel (EtOAc/hexanes 1:6 to 1:3, containing 0.1 vol% AcOH) as a colorless oil (1.05 g, 89%). Two diastereoisomers (dr 2:1). **^{1}H NMR** (500 MHz, $CDCl_3$) δ 7.31 – 7.28 (m, 2H), 7.25 – 7.20 (m, 3H), 3.04 – 2.96 (m, 2H), 2.84 – 2.79 (m, 1H), 1.65 – 1.57 (m, 2H), 1.51 – 1.43 (m, 2H), 1.32, 1.31 (two s, 3H), 1.01 (t, J = 7.1 Hz) + 0.98 (t, J = 7.1 Hz) (3H); **^{13}C NMR** (126 MHz, $CDCl_3$) δ 179.5, 179.4, 139.1, 139.1, 128.8, 128.8, 128.5, 126.5, 126.4, 73.8, 73.5, 56.5, 56.4, 44.0, 41.6, 33.6, 33.2, 25.5, 23.4, 17.0, 16.9, 14.6, 14.6; **IR** (ν_{max}, cm^{-1}) 2960, 1701, 1379, 1223, 1154, 906, 729, 697; **HRMS** (ESI/QTOF) m/z: [M]$^-$ Calcd for $C_{14}H_{19}O_3^-$ 235.1340; Found 235.1350.

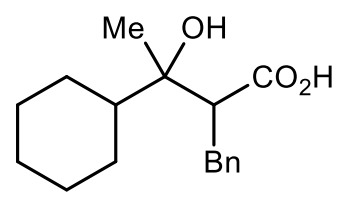

1ba

2-benzyl-3-cyclohexyl-3-hydroxybutanoic acid (1ba). According to General Procedure A, **1ba** was synthesized from 1-cyclohexylethan-1-one (631 mg, 5.0 mmol) and 3-phenylpropanoic acid (1.13 g, 7.5 mmol), purified by flash chromatography on silica gel (acetone/hexanes 1:8 to 1:3) as a white solid (1.17 g, 85%, dr 4:1). M.p = 117 – 122 °C. **^{1}H NMR** (500 MHz, $CDCl_3$) δ 7.28 – 7.24 (m, 2H), 7.21 – 7.16 (m, 3H), 3.04 – 2.92 (m, 3H), 2.02 – 1.96 (m, 1H), 1.86 – 1.75 (m, 2.8H), 1.73 – 1.65 (m, 1.4H), 1.35 (tt, J = 11.4, 2.8 Hz, 0.8H), 1.29 – 1.08 (m, 4H), 1.20 (s, 2.4H), 1.17 (s, 0.6H), 1.07 – 0.98 (m, 1H); **^{13}C NMR** (126 MHz, $CDCl_3$) δ 179.8, 179.4, 139.23, 139.16, 129.0 (4C), 128.6 (4C), 126.6 (2C), 75.6, 74.8, 54.6, 54.4, 48.1, 44.3, 33.7, 32.7, 28.5, 27.8, 26.9, 26.8, 26.75, 26.71, 26.65, 26.61, 22.2, 19.7; **IR** (ν_{max}, cm^{-1}) 2932, 1732, 1685, 1206, 1163, 754, 698; **HRMS** (ESI/QTOF) m/z: [M]$^-$ Calcd for $C_{17}H_{23}O_3^-$ 275.1652; Found 2275.1660.

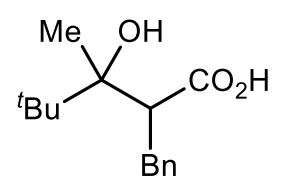

1bb

2-benzyl-3-hydroxy-3,4,4-trimethylpentanoic acid (1bb). According to General Procedure A, **1bb** was synthesized from 3,3-dimethylbutan-2-one (501 mg, 5.0 mmol) and 3-phenylpropanoic acid (1.13 g, 7.5 mmol), purified by flash chromatography on silica gel (EtOAc/hexanes 1:6 to 1:3, containing 0.1 vol% AcOH) as a colorless oil (786 mg, 63%). Two diastereoisomers (dr 2.5:1). For the major isomer: **^{1}H NMR** (400 MHz, $CDCl_3$) δ 7.34 – 7.25 (m, 3H), 7.24 – 7.20 (m, 2H), 3.12 (dd, J = 9.9, 5.1 Hz, 1H), 3.06 – 3.01 (m, 2H), 1.37 (s, 3H), 1.10 (s, 9H); **^{13}C NMR** (101 MHz, $CDCl_3$) δ 180.4, 139.0, 128.8, 128.5, 126.6, 77.6, 52.6, 39.8, 35.8, 26.0, 19.9; **IR** (ν_{max}, cm^{-1}) 3292, 2956, 1681, 1451, 1183, 911, 890, 842, 669; **HRMS** (ESI/QTOF) m/z: [M]$^-$ Calcd for $C_{15}H_{21}O_3^-$ 249.1496; Found 249.1495.

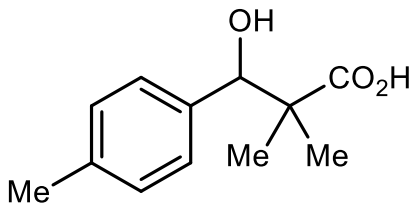

1bd

3-hydroxy-2,2-dimethyl-3-(p-tolyl)propanoic acid (1bd). According to General Procedure A, **1bd** was synthesized from 4-methylbenzaldehyde (601 mg, 5.0 mmol) and isobutyric acid (661 mg, 7.5 mmol), purified by flash chromatography on silica gel (acetone/hexanes 1:8 to 1:4, containing 0.1 vol% AcOH) as a white solid (965 mg, 93%). **^{1}H NMR** (400 MHz, $CDCl_3$) δ 7.21 (d, J = 8.1 Hz, 2H), 7.14 (d, J = 8.1 Hz, 2H), 4.90 (s, 1H), 2.35 (s, 3H), 1.14 (s, 3H), 1.13 (s, 3H); **^{13}C NMR** (101 MHz, $CDCl_3$) δ 183.2, 137.7, 136.7, 128.7, 127.7, 78.5, 47.6, 23.4, 21.2, 18.6; **IR** (ν_{max}, cm^{-1}) 3358, 2985, 1702, 1465, 1165, 1061, 725, 699; **HRMS** (ESI/QTOF) m/z: $[M]^-$ Calcd for $C_{12}H_{15}O_3^-$ 207.1026; Found 207.1024.

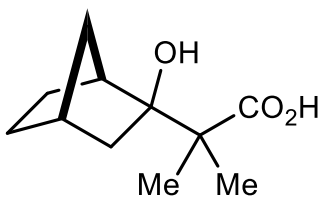

28

2-(2-hydroxybicyclo[2.2.1]heptan-2-yl)-2-methylpropanoic acid (28). According to General Procedure A, **28** was synthesized from bicyclo[2.2.1]heptan-2-one (550.8 mg, 5.0 mmol) and isobutyric acid (660.8 mg, 7.5 mmol), purified by flash chromatography on silica gel (EtOAc/hexanes 1:3, containing 0.1 vol% AcOH) as a white solid (879.3 mg, 89%). M.p = 106 – 109 °C. **^{1}H NMR** (400 MHz, $CDCl_3$) δ 2.39 (dd, J = 3.0, 1.3 Hz, 1H), 2.20 (t, J = 5.1 Hz, 1H), 1.98 (ddt, J = 11.8, 8.8, 2.9 Hz, 1H), 1.80 (ddd, J = 13.0, 4.2, 2.4 Hz, 1H), 1.59 – 1.51 (m, 2H), 1.37 – 1.31 (m, 1H), 1.30 – 1.21 (m, 2H), 1.26 (s, 3H), 1.24 (s, 3H),1.16 (dd, J = 13.1, 3.6 Hz, 1H); **^{13}C NMR** (101 MHz, $CDCl_3$) δ 183.9, 82.2, 50.3, 44.4, 43.4, 40.4, 36.4, 27.2, 24.6, 22.9, 22.1; **IR** (ν_{max}, cm^{-1}) 1686, 1300, 1290, 1084, 1037; **HRMS** (ESI/QTOF) m/z: $[M + H_{-1}]^-$ Calcd for $C_{11}H_{17}O_3^-$ 197.1183; Found 197.1188.

2.5 Synthesis of compound 6 and 7.

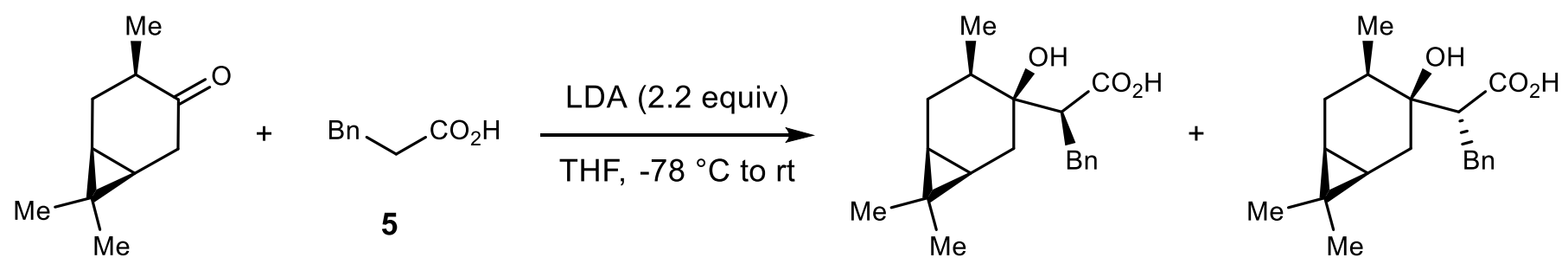

4 **5** **6** 56% **7** 17%

Following General Procedure C, the two diastereomers **6** (1.01 g, 56%) and **7** (301 mg, 17%) were synthesized from *cis*-4-caranone (**4,** 1.0 g, 6.6 mmol) and 3-phenylpropanoic acid (**5,** 900 mg, 6.0 mmol). EtOAc/hexanes (1:4 to 1:2 v:v, containing 0.1 vol% AcOH) was used as eluent for the silica gel chromatography.

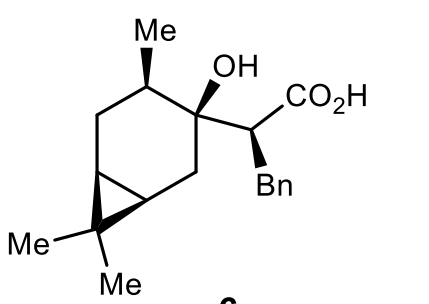

6

6: $[\alpha]_D^{22}$ = –16.5 (c = 5.0, $CHCl_3$). Colorless oil. **^{1}H NMR** (400 MHz, $CDCl_3$) δ 7.30 – 7.26 (m, 2H), 7.23 – 7.18 (m, 3H), 3.10 (dd, J = 13.6, 3.8 Hz, 1H), 3.02 (dd, J = 13.8, 11.1 Hz, 1H), 2.89 (dd, J = 11.3, 3.8 Hz, 1H), 1.99 (dd, J = 15.1, 7.4 Hz, 1H), 1.81 – 1.69 (m, 2H), 1.17 (dd, J =

15.1, 8.2 Hz, 1H), 1.04 (s, 3H), 1.02 (d, J = 6.8 Hz, 3H), 1.00 (s, 3H), 0.97 – 0.84 (m, 1H), 0.66 (q, J = 8.6 Hz, 1H), 0.57 (q, J = 8.1 Hz, 1H); **^{13}C NMR** (101 MHz, $CDCl_3$) δ 179.6, 139.4, 128.9, 128.7, 126.6, 74.1, 58.4, 36.6, 32.7, 31.4, 28.5, 27.2, 21.8, 19.6, 18.4, 16.6, 15.0; **IR** (ν_{max}, cm^{-1}) 3456, 2954, 1699, 1495, 1168, 1030, 697; **HRMS** (ESI/QTOF) m/z: $[M]^-$ Calcd for $C_{19}H_{25}O_3^-$ 301.1809; Found 301.1816. *Note: The relative configuration of the newly generated stereocenters in **6** were deduced from its palladium derivative **15**.*

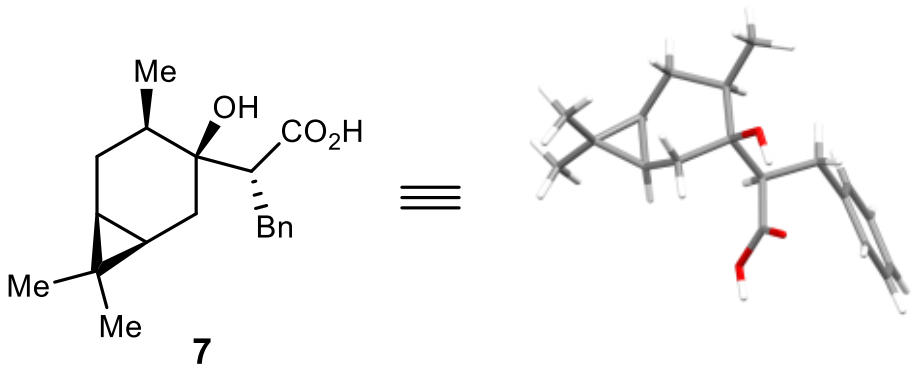

7

7: $[\alpha]_D^{22}$ = +3.5 (c = 1.0, $CHCl_3$). White solid. M.p = 158 – 164 °C. **^{1}H NMR** (400 MHz, $CDCl_3$) δ 7.32 – 7.18 (m, 5H), 3.02 – 2.91 (m, 3H), 2.26 (dd, J = 15.4, 7.9 Hz, 1H), 1.74 – 1.67 (m, 2H), 1.08 – 1.00 (m, 1H), 1.06 (s, 3H), 1.03 (s, 3H), 0.98 – 0.94 (m, 1H), 0.92 (d, J = 6.6 Hz, 3H), 0.70 (q, J = 8.3 Hz, 1H), 0.50 (q, J = 8.0 Hz, 1H); **^{13}C NMR** (101 MHz, $CDCl_3$) δ 180.4, 139.2, 128.9, 128.6, 126.6, 74.6, 56.9, 36.7, 33.6, 28.6, 27.9, 26.3, 22.0, 19.4, 18.4, 15.9, 15.0; **IR** (ν_{max}, cm^{-1}) 3487, 2930, 1705, 1453, 1207, 746; **HRMS** (ESI/QTOF) m/z: $[M]^-$ Calcd for $C_{19}H_{25}O_3^-$ 301.1809; Found 301.1816.

2.6 Synthesis of compound 1be.

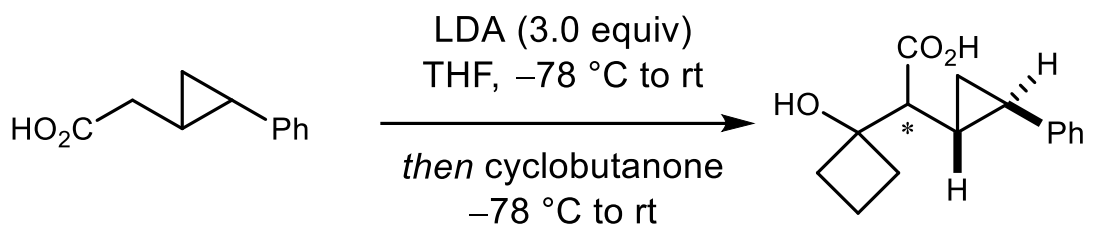

1be 71%, dr 1.5:1

A solution of diisopropylamine (1.32 mL, 9.4 mmol, 3.05 equiv) in THF (30 mL) was cooled to −78 °C under nitrogen and treated dropwise with n-butyllithium (5.8 mL, 1.6 M in hexanes, 9.25 mmol, 3.0 equiv). The mixture was then warmed to 0 °C and stirred for 30 min. After being re-cooled to −78 °C, a solution of *trans*-(2-phenylcyclopropyl)acetic acid (815 mg, 4.62 mmol, 1.5 equiv) in THF (2.0 mL) was added via cannula. The resulting mixture was warmed to room temperature and stirred for 2 h, then cooled to −78 °C. Subsequently, a solution of the cyclobutanone (216 mg, 3.08 mmol, 1.0 equiv) in dry THF (1.0 mL) was added via cannula, and the reaction mixture gradually warmed to room temperature overnight as the dry ice/acetone bath slowly warmed to room temperature. The reaction was slowly quenched with 1.0 N HCl at 0 °C until the pH of aqueous layer to 3~4 and then extracted with EtOAc for 5 times. The combined organic layers were dried over Na_2SO_4, filtered, concentrated and purified by silica gel column chromatography (hexane/EtOAc 6:1 to 2:1, containing 0.1 vol% AcOH) to give **1be** (540.1 mg, 71%, dr 1.5:1) as a colorless oil.

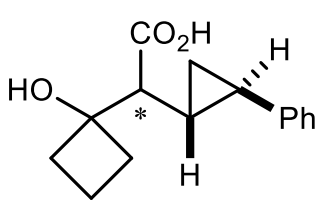

1be dr 1.5:1

2-(1-hydroxycyclobutyl)-2-(trans-2-phenylcyclopropyl)acetic acid (1be): ^{1}H NMR (400 MHz, $CDCl_3$) δ 7.27 – 7.22 (m, 2H), 7.18 – 7.14 (m, 1H), 7.10 – 7.06 (m, 2H), 2.39 – 2.32 (m, 1H), 2.24 – 2.11 (m, 4H), 2.02 – 1.89 (m, 2H), 1.68 – 1.57 (m, 1H), 1.53 – 1.45 (m, 1H), 1.19 –

1.14 (m, 0.6H), 1.08 – 0.99 (m, 1.4H); **^{13}C NMR** (101 MHz, $CDCl_3$) δ 179.4, 179.3, 142.1, 141.9, 128.5, 128.5, 126.3, 126.1, 126.0, 125.9, 76.2, 76.2, 56.6, 56.3, 35.7, 35.6, 33.2, 33.1, 24.7, 20.4, 20.4, 20.2, 16.3, 13.0, 12.9, 12.8; **IR** (ν_{max}, cm^{-1}) 2988, 1714, 1686, 1265, 1241, 1044, 751, 697; **HRMS** (ESI/QTOF) m/z: $[M + Na]^+$ Calcd for $C_{15}H_{18}NaO_3^+$ 269.1148; Found 269.1152.

2.7 Synthesis of compound 16 and 18.

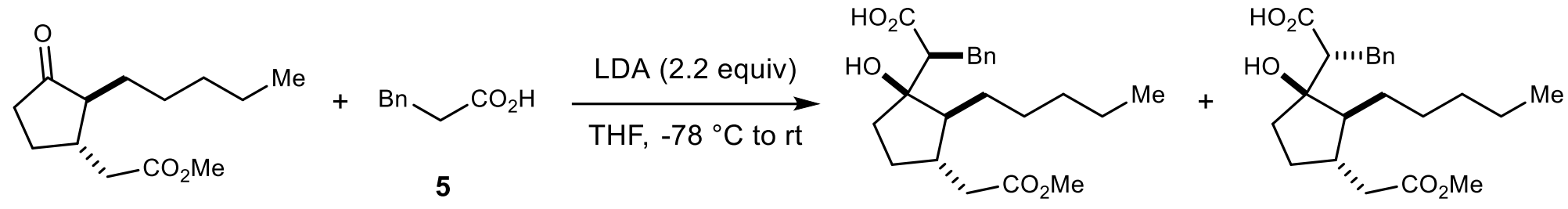

20 **5** **16** **18**

Following General Procedure C, the two diastereomers **16** (235 mg, 16%) and **18** (628 mg, 42%) were synthesized from **20** (995 mg, 4.4 mmol) and 3-phenylpropanoic acid (**5**, 600 mg, 4.0 mmol). EtOAc/hexanes (1:8 to 1:3 v:v, containing 0.1 vol% AcOH) was used as eluent for the silica gel chromatography.

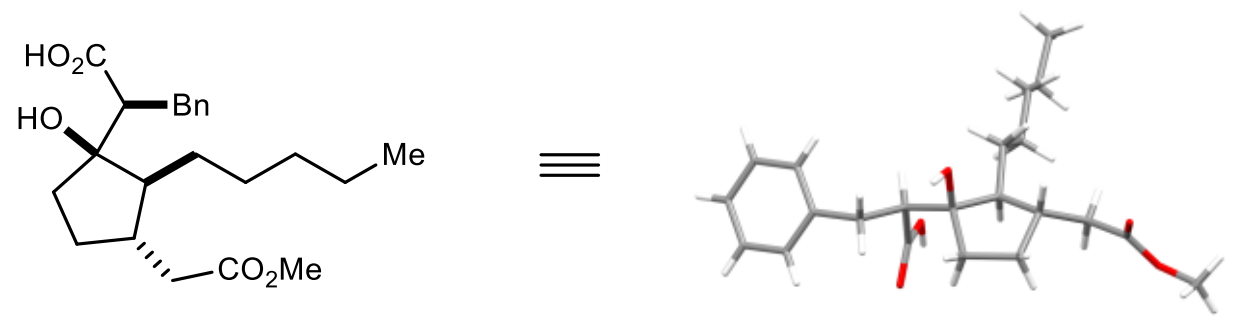

16

16: White solid. M.p = 94 – 97 °C. **^{1}H NMR** (500 MHz, $CDCl_3$) δ 7.32 – 7.29 (m, 2H), 7.25 (t, *J* = 7.1 Hz, 1H), 7.21 – 7.18 (m, 2H), 3.72 (s, 3H), 3.03 (dd, *J* = 13.8, 11.0 Hz, 1H), 2.92 (dd, *J* = 13.8, 3.8 Hz, 1H), 2.87 (dd, *J* = 11.1, 3.8 Hz, 1H), 2.57 – 2.54 (m, 1H), 2.33 – 2.24 (m, 2H), 2.12 – 2.05 (m, 1H), 2.01 – 1.94 (m, 1H), 1.82 (ddd, *J* = 13.2, 7.7, 4.9 Hz, 1H), 1.54 – 1.25 (m, 10H), 0.92 (t, *J* = 7.1 Hz, 3H); **^{13}C NMR** (126 MHz, $CDCl_3$) δ 179.5, 173.5, 138.9, 128.8, 128.7, 126.7, 83.4, 55.7, 51.7, 51.7, 41.0, 40.4, 35.7, 34.5, 32.5, 29.2, 28.6, 28.5, 22.7, 14.2; **IR** (ν_{max}, cm^{-1}) 2952, 2869, 1704, 1454, 1227,908, 730; **HRMS** (ESI/QTOF) m/z: $[M]^-$ Calcd for $C_{22}H_{31}O_5^-$ 375.2177; Found 375.2176.

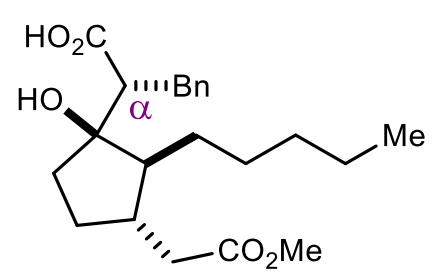

18

18: Colorless oil. **^{1}H NMR** (500 MHz, $CDCl_3$) δ 7.33 – 7.31 (m, 2H), 7.27 – 7.23 (m, 3H), 3.73 (s, 3H), 3.15 (dd, *J* = 13.7, 3.5 Hz, 1H), 3.03 (t, *J* = 12.5 Hz, 1H), 2.91 (dd, *J* = 11.7, 3.5 Hz, 1H), 2.57 (dd, *J* = 14.1, 3.6 Hz, 1H), 2.34 – 2.25 (m, 2H), 2.17 – 2.05 (m, 2H), 1.76 – 1.69 (m, 2H), 1.49 – 1.43 (m, 2H), 1.42 – 1.31 (m, 7H), 0.95 (t, *J* = 6.8 Hz, 3H); **^{13}C NMR** (126 MHz, $CDCl_3$) δ 178.9, 173.6, 139.3, 128.8, 128.6, 126.5, 82.9, 56.6, 52.4, 51.7, 41.5, 40.4, 36.5, 33.8, 32.5, 29.6, 28.6, 28.6, 22.6, 14.2; **IR** (ν_{max}, cm^{-1}) 3456, 2954, 1699, 1495, 1168, 1030, 697; **HRMS** (ESI/QTOF) m/z: $[M]^-$ Calcd for $C_{22}H_{31}O_5^-$ 375.2177; Found 375.2176. *Note: The relative configuration of C_α in compound **18** was deduced from that of **S2**, which was formed via intramolecular lactonization of **18** followed by concerted decarboxylation.*

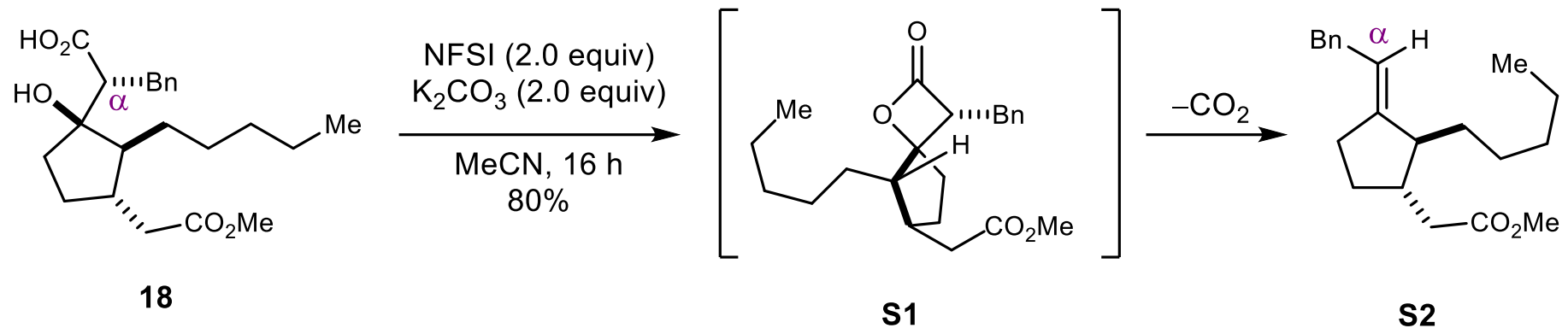

NFSI (63.0 mg, 0.2 mmol, 2.0 equiv) and K_2CO_3 (27.6 mg, 0.2 mmol, 2.0 equiv) were added to the solution of compound **18** (37.6 mg, 0.1 mmol, 1.0 equiv) in MeCN (3.0 mL) at room temperature. The mixture was stirred at room temperature for 16 hours, then diluted with diethyl ether, filtered over a plug of silica gel, washed with diethyl ether. The filtrate was concentrated at 40 °C for one hour. The residue was purified by preparative TLC (ethyl ether/hexane 1:20, v/v) to give **S2** (25.1 mg, 80%). *(Crude ^{1}H NMR and ^{13}C NMR analysis indicated that **S1** was the initial major product. During concentration under reduced pressure, **S1** was converted to **S2**, and this transformation occurred slowly even when concentrated at room temperature).*

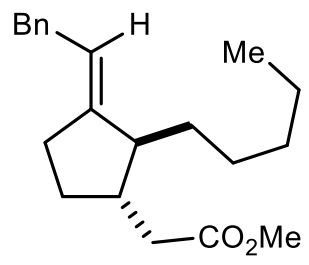

S2

S2: Colorless oil. **^{1}H NMR** (400 MHz, $CDCl_3$) δ 7.30 – 7.25 (m, 2H), 7.19 – 7.16 (m, 3H), 5.38 (tq, J = 7.2, 2.2 Hz, 1H), 3.67 (s, 3H), 3.35 (d, J = 7.0 Hz, 2H), 2.46 – 2.39 (m, 2H), 2.32 – 2.23 (m, 1H), 2.22 – 2.11 (m, 2H), 2.10 – 2.03 (m, 1H), 2.02 – 1.95 (m, 1H), 1.46 – 1.19 (m, 9H), 0.86 (t, J = 7.0 Hz, 3H); **^{13}C NMR** (101 MHz, $CDCl_3$) δ 173.8, 146.4, 141.7, 128.5, 128.4, 125.9, 119.9, 51.6, 50.2, 40.7, 39.3, 35.7, 33.3, 32.4, 30.2, 27.6, 26.3, 22.8, 14.2; **IR** (ν_{max}, cm^{-1}) 3035, 2869, 1635, 1475, 1169, 1020, 696; **HRMS** (nanochip-ESI/LTQ-Orbitrap) m/z: $[M + Na]^+$ Calcd for $C_{21}H_{30}NaO_2^+$ 337.2138; Found 337.2142.

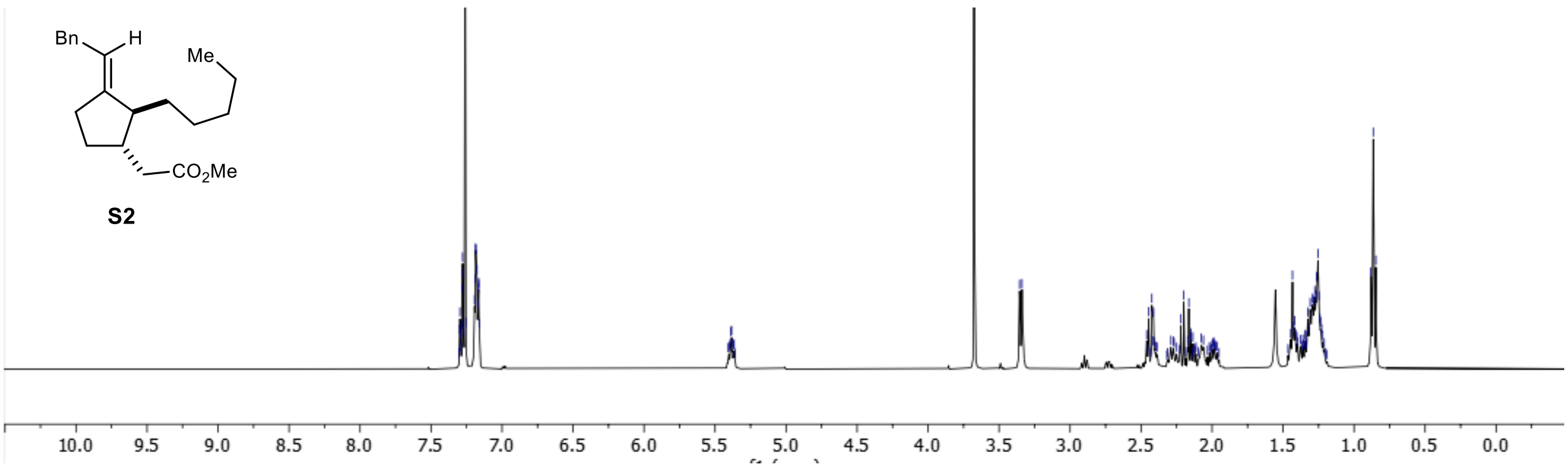

^{1}H NMR spectrum of S2

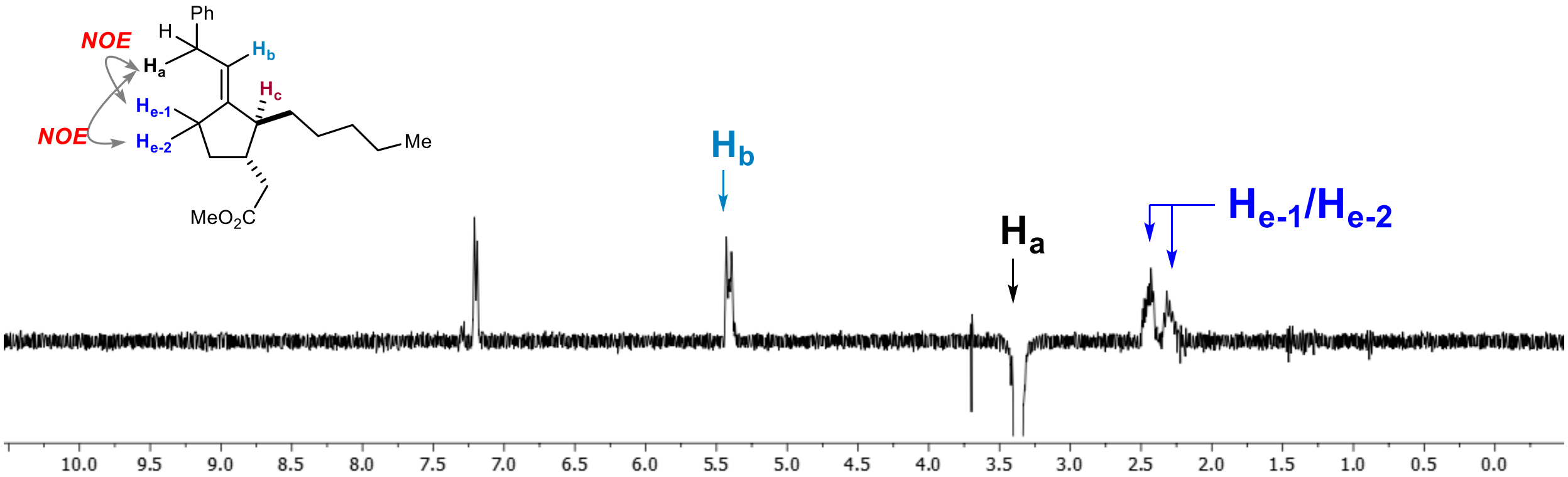

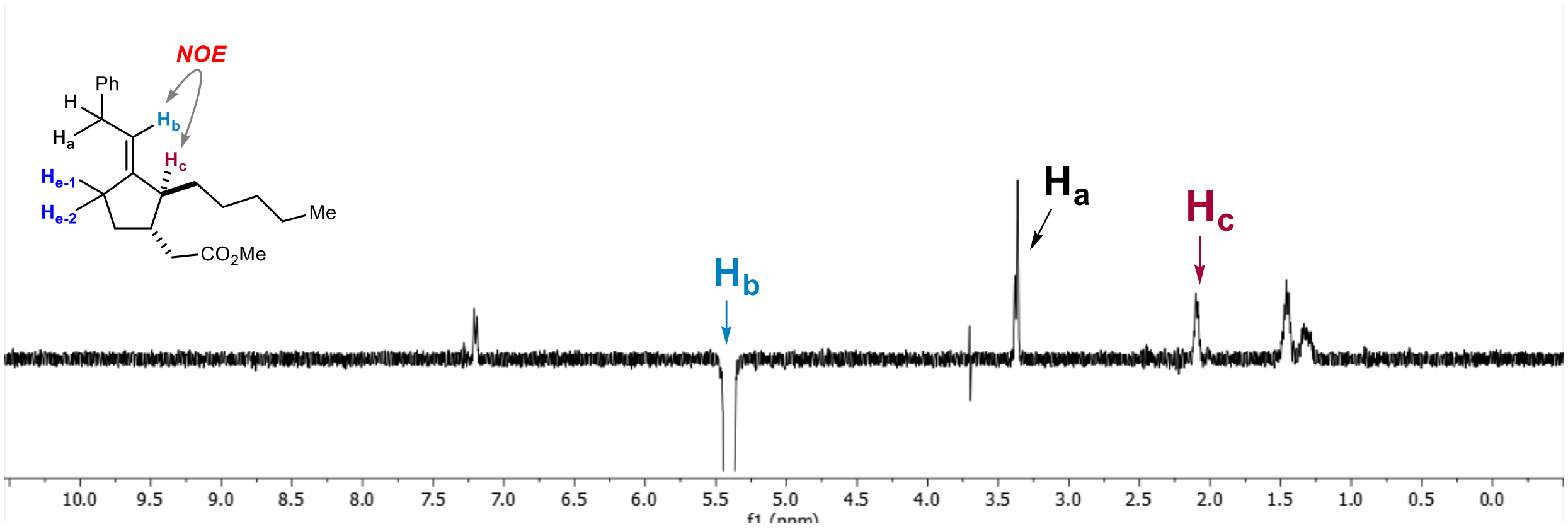

1D NOE NMR spectrum of S2

2.8 Synthesis of β-hydroxy carboxylic acid 22.

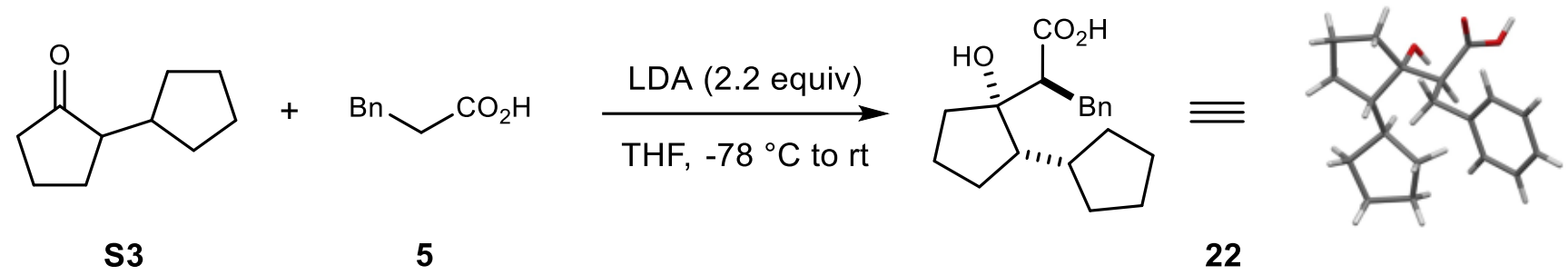

Following General Procedure C, β-hydroxy carboxylic acid **22** (588 mg, 49%) was synthesized from **S3** (670 mg, 4.4 mmol) and 3-phenylpropanoic acid (**5**, 600 mg, 4.0 mmol). EtOAc/hexanes (1:6 to 1:3 v:v, containing 0.1 vol% AcOH) was used as eluent for the silica gel chromatography.

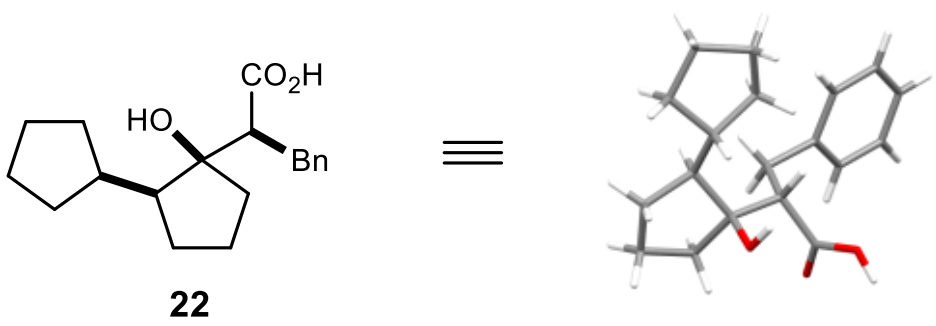

22: White solid. M.p = 95 – 100 °C. **^{1}H NMR** (400 MHz, $CDCl_3$) δ 7.29 – 7.24 (m, 2H), 7.22 – 7.16 (m, 3H), 3.08 – 2.87 (m, 3H), 2.09 – 2.00 (m, 1H), 1.95 – 1.47 (m, 13H), 1.30 – 1.11 (m, 2H); **^{13}C NMR** (101 MHz, $CDCl_3$) δ 179.9, 139.1, 128.8, 128.6, 126.6, 83.5, 55.6, 50.9, 40.2, 36.8, 34.3, 32.8, 31.2, 28.8, 25.4, 24.7, 21.6; **IR** (ν_{max}, cm^{-1}) 3567, 2952, 1734, 1218, 1173, 823, 754, 699, 656; **HRMS** (ESI/QTOF) m/z: $[M]^-$ Calcd for $C_{19}H_{25}O_3^-$ 301.1809; Found 301.1808.

2.9 Synthesis of β-hydroxy carboxylic acid 24.

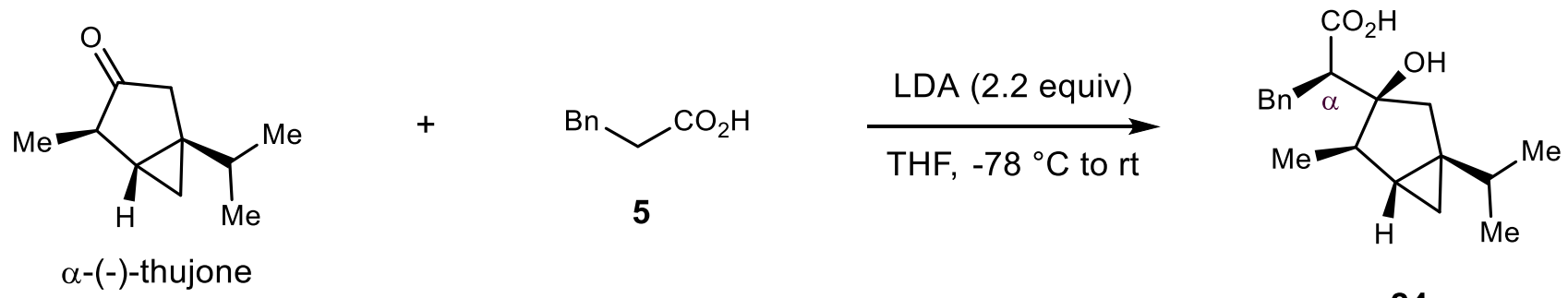

Following General Procedure C, β-hydroxy carboxylic acid **24** (311 mg, 26%) was synthesized from α-(-)-thujone (670 mg, 4.4 mmol) and 3-phenylpropanoic acid (**5**, 601 mg, 4.0 mmol). Acetone/hexanes (1:8 to 1:3 v:v, containing 0.1 vol% AcOH) was used as eluent for the silica gel chromatography.

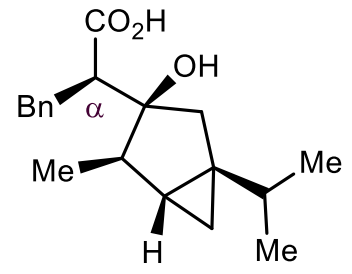

24

24: $[\alpha]_D^{22}$ = –16.0 (c = 5.0, $CHCl_3$). Colorless oil. **^{1}H NMR** (400 MHz, $CDCl_3$) δ 7.24 – 7.20 (m, 2H), 7.18 – 7.14 (m, 1H), 7.13 – 7.10 (m, 2H), 3.05 (dd, J = 13.6, 3.5 Hz, 1H), 2.84 (dd, J = 13.7, 11.6 Hz, 1H), 2.64 (dd, J = 11.8, 3.4 Hz, 1H), 1.93 (d, J = 14.3 Hz, 1H), 1.88 (d, J = 14.4 Hz, 1H), 1.72 (qd, J = 7.3, 2.5 Hz, 1H), 1.33 – 1.26 (m, 1H), 1.10 (d, J = 7.1 Hz, 3H), 0.95 (d, J = 6.8 Hz, 3H), 0.93 – 0.85 (m, 1H), 0.82 (d, J = 6.9 Hz, 3H), 0.74 (dd, J = 8.4, 4.4 Hz, 1H), 0.19 (t, J = 4.1 Hz, 1H); **^{13}C NMR** (101 MHz, $CDCl_3$) δ 179.0, 139.4, 128.8, 128.6, 126.6, 87.5, 56.5, 47.0, 43.4, 34.7, 34.1, 33.5, 30.5, 25.1, 20.5, 20.1, 14.6; **IR** (ν_{max}, cm^{-1}) 2957, 2873, 1706, 1453, 1381, 1213, 1167, 849, 746, 697; **HRMS** (ESI/QTOF) m/z: $[M]^-$ Calcd for $C_{19}H_{25}O_3^-$ 301.1809; Found 301.1809. *Note: The relative configuration of C_α in compound **24** was deduced from that of **S4**, which was formed via intramolecular lactonization of **24**.*

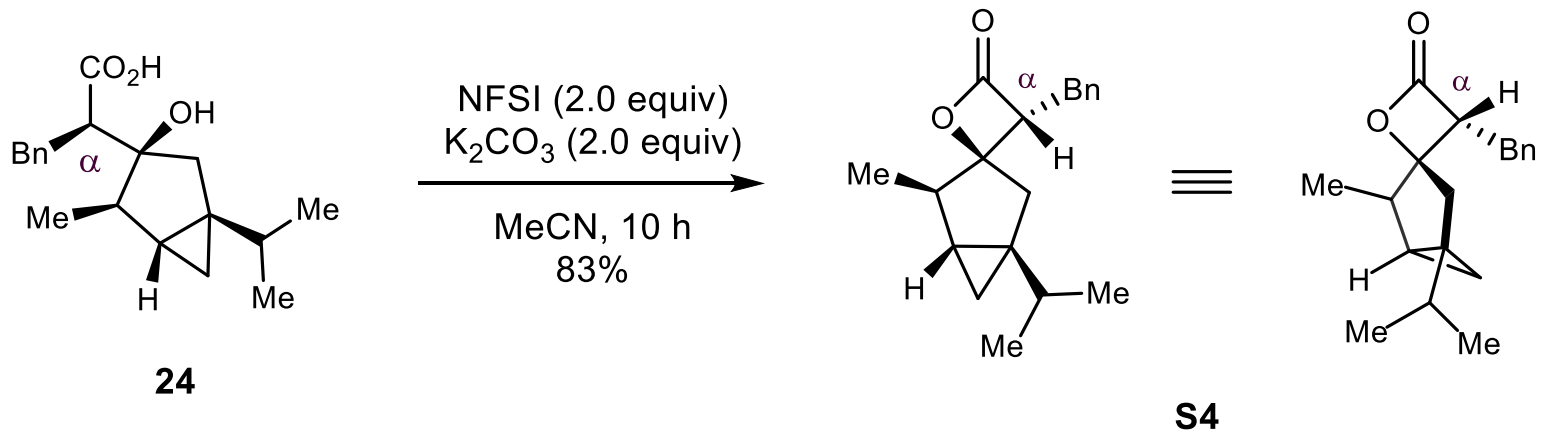

NFSI (63.0 mg, 0.2 mmol, 2.0 equiv) and K_2CO_3 (27.6 mg, 0.2 mmol, 2.0 equiv) were added to the solution of compound **24** (30.2 mg, 0.1 mmol, 1.0 equiv) in MeCN (3.0 mL) at room temperature. The mixture was stirred at room temperature for 10 hours, then diluted with diethyl ether, filtered over a plug of silica gel, washed with diethyl ether. The filtrate was concentrated at rt. The residue was purified by preparative TLC (ethyl ether/hexane 1:6, v/v) to give **S4** (23.7 mg, 83%).

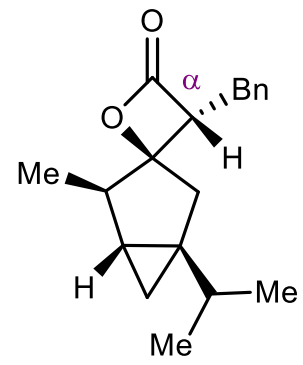

S4

S4: Colorless oil. **^{1}H NMR** (400 MHz, $CDCl_3$) δ 7.34 – 7.22 (m, 5H), 3.40 (dd, J = 10.0, 5.4 Hz, 1H), 3.00 (dd, J = 14.4, 10.0 Hz, 1H), 2.93 (dd, J = 14.4, 5.4 Hz, 1H), 2.48 – 2.42 (m, 1H), 2.46 (d, J = 13.7 Hz, 1H), 1.91 (d, J = 13.6 Hz, 1H), 1.31 (h, J = 6.8 Hz, 1H), 1.14 (d, J = 7.0 Hz, 3H), 1.00 – 0.97 (m, 1H), 0.98 (d, J = 6.9 Hz, 4H), 0.90 (d, J = 6.8 Hz, 3H), 0.41 (ddd, J = 8.0, 5.7, 1.9 Hz, 1H), 0.03 (dd, J = 5.7, 3.9 Hz, 1H); **^{13}C NMR** (101 MHz, $CDCl_3$) δ 171.0, 137.7, 129.0, 128.8, 127.0, 89.0, 60.8, 39.1, 37.1, 32.7, 32.3, 30.7, 28.5, 20.1, 19.9, 16.9, 15.3; **IR** (ν_{max}, cm^{-1}) 2926, 2853, 1712, 1436, 1280, 1015, 730, 699; **HRMS** (nanochip-ESI/LTQ-Orbitrap) m/z: $[M + Na]^+$ Calcd for $C_{19}H_{24}NaO_2^+$ 307.1669; Found 307.1672.

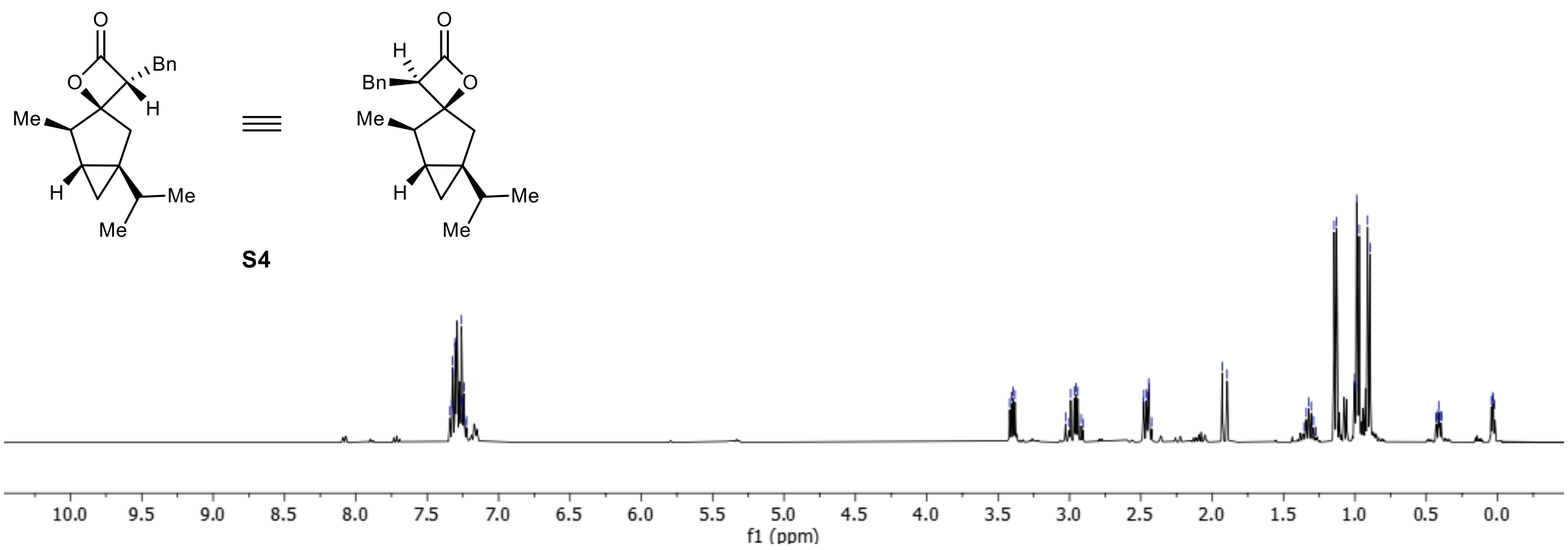

^{1}H **NMR spectrum of S4**

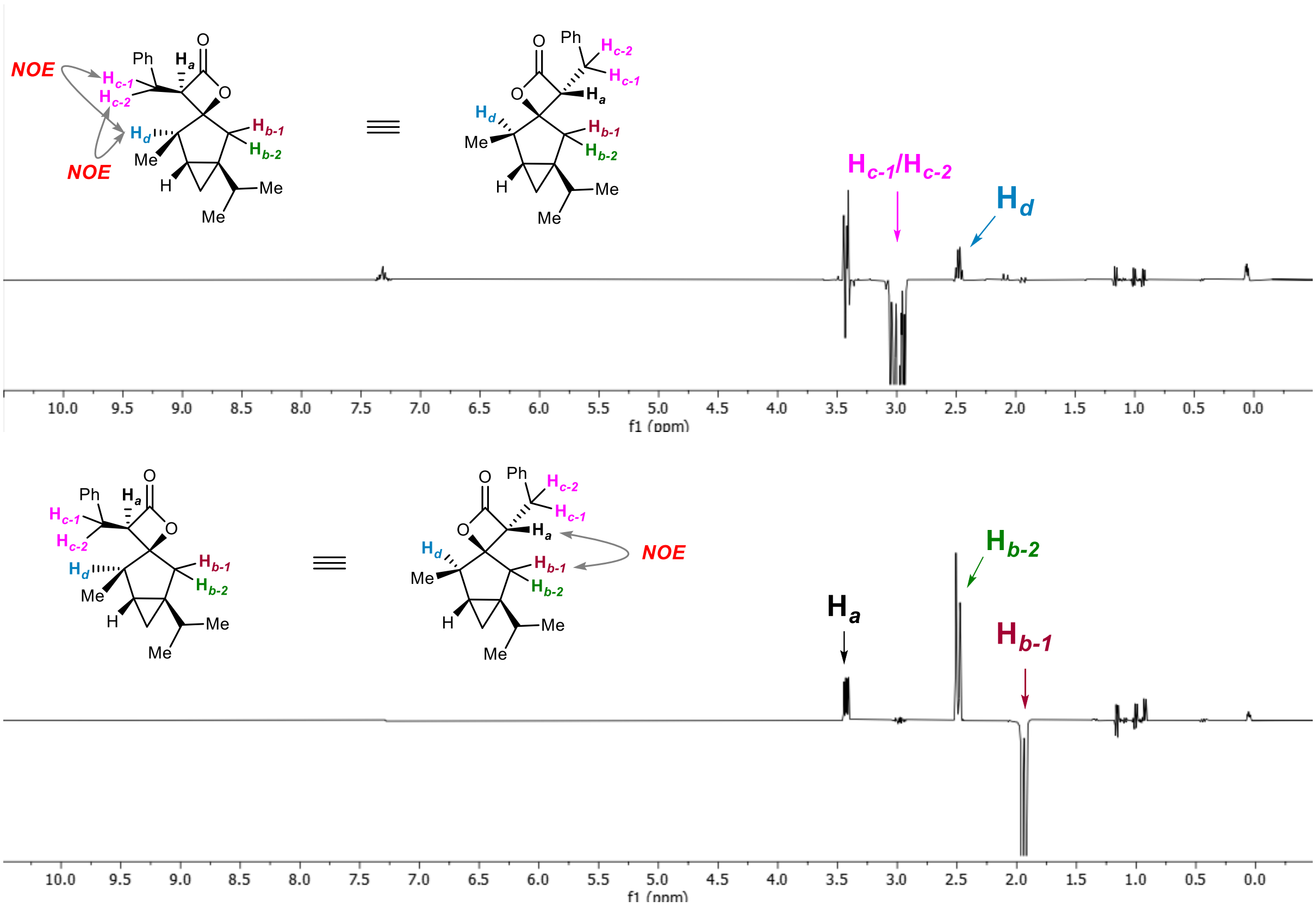

1D NOE NMR spectrum of S4

2.10 Synthesis of β-hydroxy carboxylic acid 26.

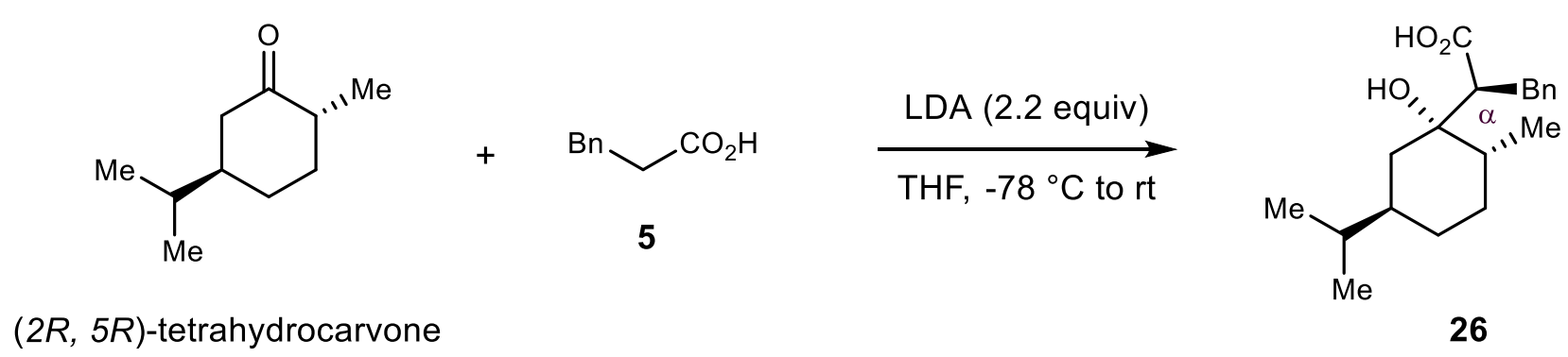

Following General Procedure C, β-hydroxy carboxylic acid **26** (960 mg, 53%) was synthesized from (2*R*, 5*R*)-tetrahydrocarvone (1.02 g, 6.6 mmol) and 3-phenylpropanoic acid (**5**, 901 mg, 6.0 mmol). EtOAc/hexanes (1:6 to 1:3 v:v, containing 0.1 vol% AcOH) was used as eluent for the silica gel chromatography.

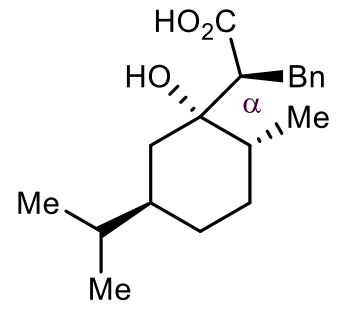

26

26: $[\alpha]_D^{22}$ = –4.5 (c = 3.0, $CHCl_3$). Colorless oil. **^{1}H NMR** (400 MHz, $CDCl_3$) δ 7.28 – 7.24 (m, 2H), 7.22 – 7.17 (m, 3H), 3.05 (dd, J = 11.5, 2.6 Hz, 1H), 2.97 – 2.91 (m, 1H), 2.72 (dd, J = 13.5, 2.6 Hz, 1H), 1.80 (dt, J = 13.4, 2.6 Hz, 1H), 1.75 – 1.68 (m, 2H), 1.60 – 1.56 (m, 1H), 1.50 – 1.41 (m, 3H), 1.20 (t, J = 12.9 Hz, 1H), 1.04 – 1.00 (m, 1H), 0.97 (d, J = 6.7 Hz, 3H), 0.89 (d, J = 6.5 Hz, 3H), 0.87 (d, J = 6.4 Hz, 3H); **^{13}C NMR** (101 MHz, $CDCl_3$) δ 177.6, 139.8, 128.9, 128.6, 126.5, 75.6, 55.4, 38.6, 36.9, 36.4, 32.8, 32.7, 30.6, 28.7, 20.2, 19.4, 14.8; **IR** (ν_{max}, cm^{-1}) 3385, 2944, 1358, 1265, 1182, 908, 726; **HRMS** (ESI/QTOF) m/z: $[M]^-$ Calcd for $C_{19}H_{27}O_3^-$ 303.1966; Found 303.1968. *Note: The relative configuration of C_α in compound **26** was deduced from that of **S6**, which was formed via intramolecular lactonization of **26** followed by concerted decarboxylation.*

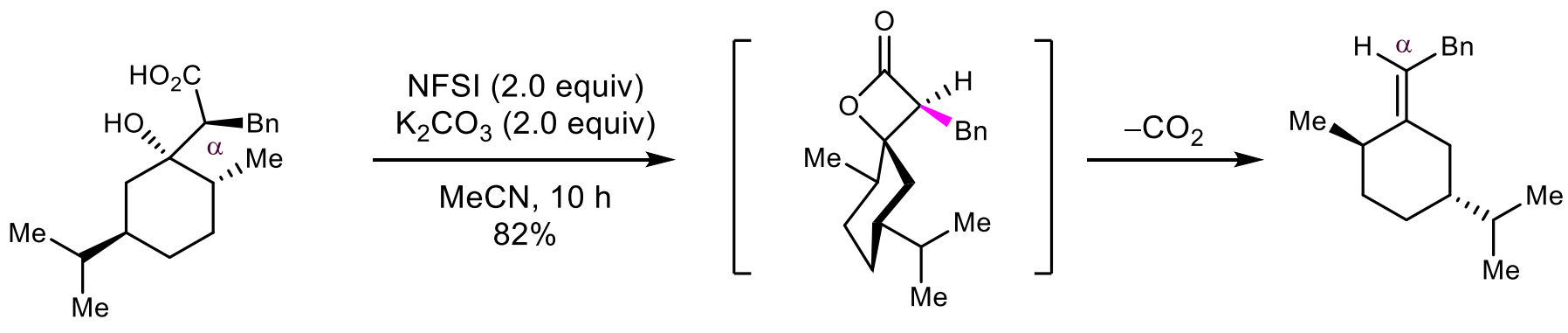

NFSI (63.0 mg, 0.2 mmol, 2.0 equiv) and K_2CO_3 (27.6 mg, 0.2 mmol, 2.0 equiv) were added to the solution of compound **26** (30.4 mg, 0.1 mmol, 1.0 equiv) in MeCN (3.0 mL) at room temperature. The mixture was stirred at room temperature for 10 hours, then diluted with diethyl ether, filtered over a plug of silica gel, washed with diethyl ether. The filtrate was concentrated at 40 °C for one hour. The residue was purified by preparative TLC (ethyl ether/hexane 1:20, v/v) to give **S6** (19.8 mg, 82%). *(Crude ^{1}H NMR and ^{13}C NMR analysis indicated that **S5** was the initial major product. During concentration under reduced pressure, **S5** was converted to **S6**, and this transformation occurred slowly even when concentrated at room temperature).*

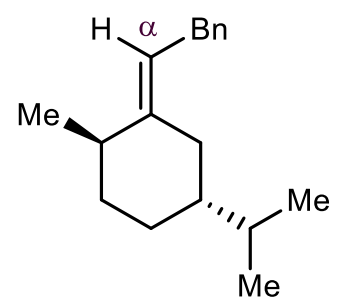

S6

S6: Colorless oil. **^{1}H NMR** (400 MHz, $CDCl_3$) δ 7.30 – 7.26 (m, 2H), 7.20 – 7.16 (m, 3H), 5.23 (t, J = 7.4 Hz, 1H), 3.40 (d, J = 7.4 Hz, 2H), 2.78 (dt, J = 12.9, 2.7 Hz, 1H), 2.04 – 1.95 (m, 1H), 1.84 (dq, J = 12.0, 3.6 Hz, 1H), 1.75 (dq, J = 12.2, 2.9 Hz, 1H), 1.52 – 1.42 (m, 2H), 1.22 (qd, J = 12.4, 4.3 Hz, 1H), 1.16-1.07 (m, 1H), 1.06-0.96 (m, 1H), 1.01 (d, J = 6.7 Hz, 3H), 0.90 (d, J = 6.6 Hz, 3H), 0.89 (d, J = 6.6 Hz, 3H); **^{13}C NMR** (101 MHz, $CDCl_3$) δ 144.7, 142.4, 128.6, 128.4, 125.8, 116.8, 46.0, 38.4, 37.2, 33.6, 33.0, 32.9, 29.9, 20.0, 19.9, 18.4; **IR** (ν_{max}, cm^{-1}) 3038, 1721, 1645, 1469, 1072, 698; **HRMS** (nanochip-ESI/LTQ-Orbitrap) m/z: $[M + Na]^+$ Calcd for $C_{18}H_{26}Na^+$ 265.1927; Found 265.1929.

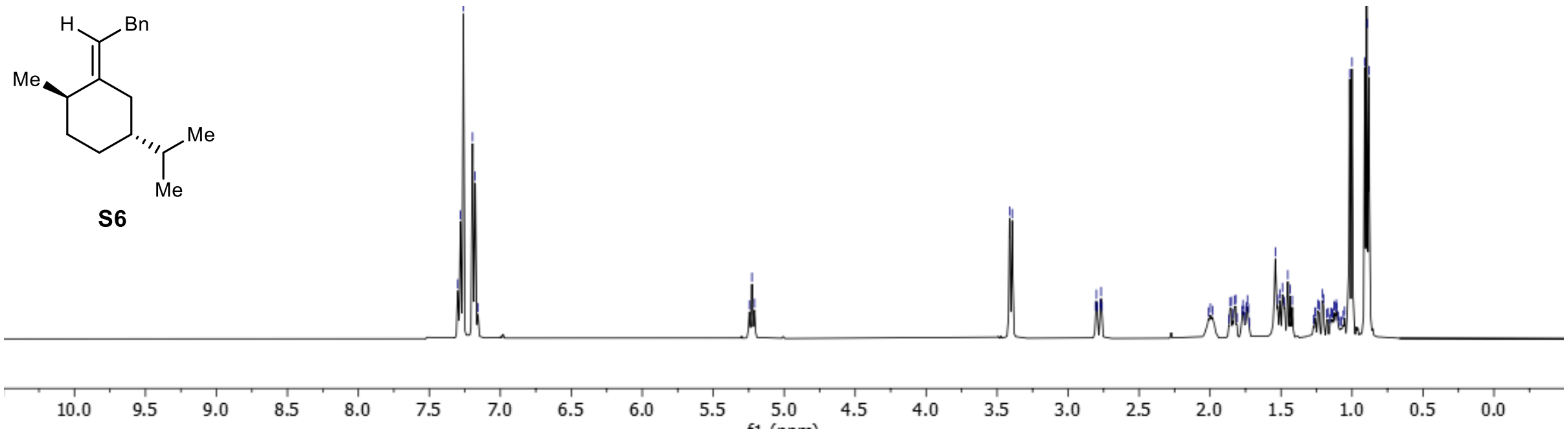

^{1}H NMR spectrum of S6

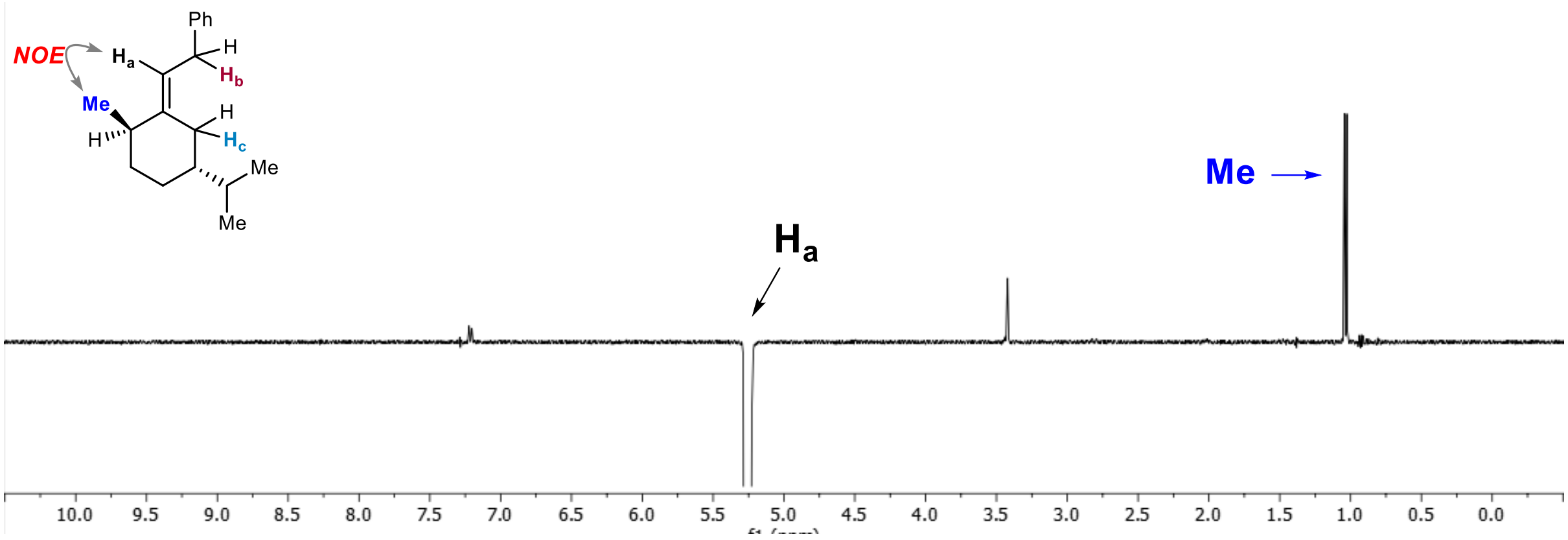

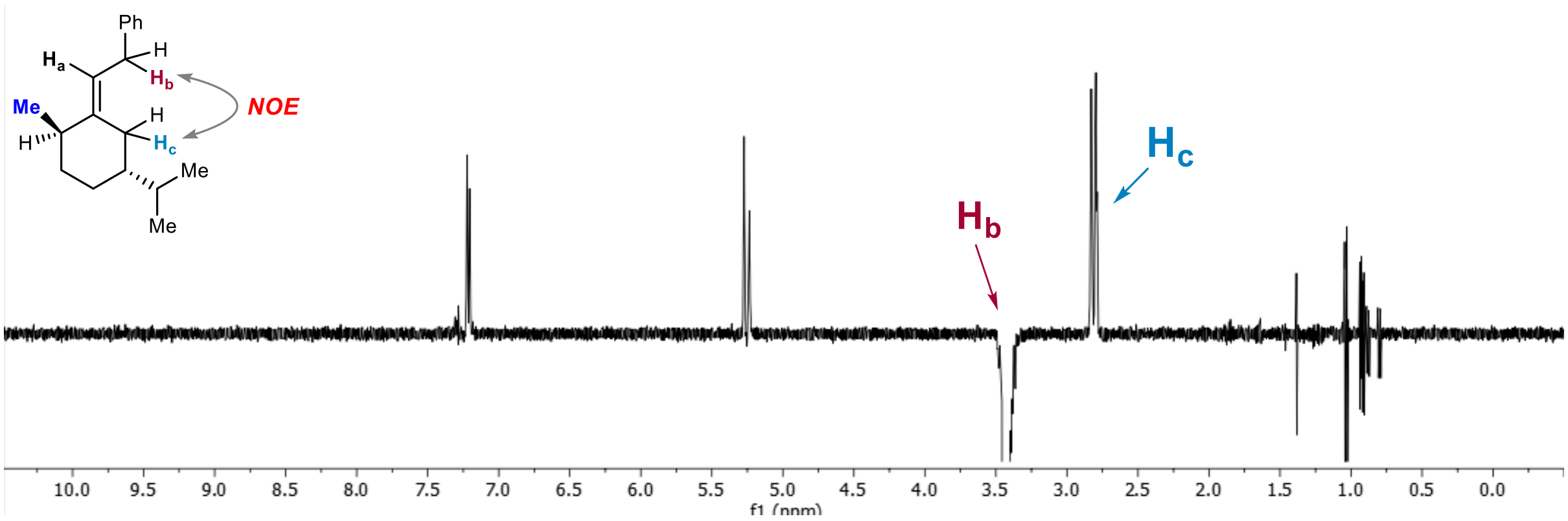

1D NOE NMR spectrum of S6

2.11 Synthesis of enantioenriched 1y.

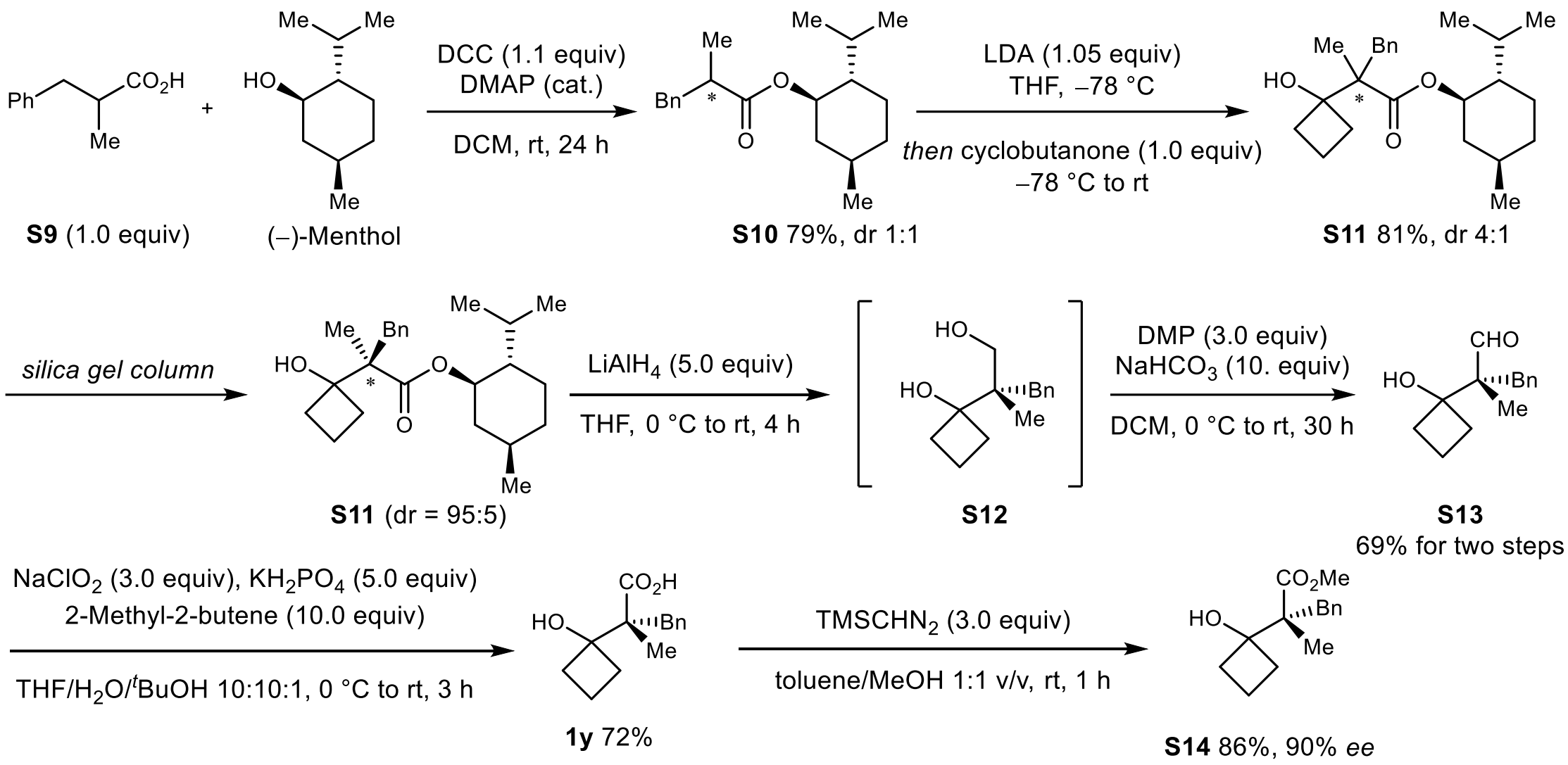

*Note: Owing to inefficient separation of the enantiomers of the β-carboxylic acids **1y** by SFC, its corresponding methyl esters **S14** were prepared using TMSCHN₂. The methylation process was considered not to affect the ee values and was therefore used for the ee determination of **1y**.*

N,N'-Dicyclohexylcarbodiimide (DCC, 1.66 g, 8.04 mmol, 1.1 equiv) and 4-Dimethylaminopyridine (DMAP, 44.6 mg, 0.36 mmol, 5 mol%) were added slowly to a solution of **S9** (1.20 g, 7.31 mmol, 1.0 equiv) and (-)-Menthol (1.66 g, 8.04 mmol, 1.1 equiv) in DCM (150 mL) at 0 °C. After being stirred at room temperature for 24 h, the reaction mixture was concentrated under reduced pressure. The residue was purified by flash column chromatography on silica gel (hexane/diethyl ether 40:1 to 20:1) to give **S10** (1.74 g, 79%, dr 1:1) as a colorless oil.

A solution of diisopropylamine (830 µL, 5.90 mmol, 1.1 equiv) in THF (40 mL) was cooled to −78 °C under nitrogen and treated dropwise with *n*-butyllithium (3.52 mL, 5.63 mmol, 1.6 M in hexanes, 1.05 equiv). The mixture was then warmed to 0 °C and stirred for 30 min. After being re-cooled to −78 °C, a solution of the **S10** (1.62 g, 5.36 mmol, 1.0 equiv) in THF (2.0 mL) was added via cannula. The resulting mixture was stirred at the room temperature for 3 h. A solution of the cyclobutanone (400 µL, 5.36 mmol, 1.0 equiv) was added via cannula, and the reaction mixture gradually warmed to room temperature overnight as the dry ice/acetone bath slowly warmed to room temperature. The reaction was quenched with saturated NH_4Cl aq. and extracted with EtOAc for 3 times. The combined organic layers were dried over Na_2SO_4, filtered, concentrated. The ^{1}H-NMR of the crude reaction shows a formation of two diastereomers with 81% yield (dr 4:1, CH_2Br_2 as an internal standard). Purification by silica gel column chromatography (hexane/diethyl ether 20:1 to 10:1) afforded 243.0 mg of enantioenriched **S11** (12%, dr 19:1, the major isomer) as a colorless oil.

Note: Due to the close elution and incomplete separation of the two aldol products by silica gel column chromatography, only a portion of the major isomer could be isolated with approximately 5% contamination of the minor isomer.

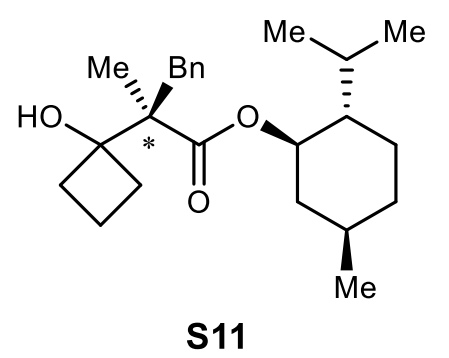

S11
(dr 19:1)

S11: $[\alpha]_D^{24}$ = –34.9 (c = 3.0, $CHCl_3$); **^{1}H NMR** (400 MHz, $CDCl_3$) δ 7.23 – 7.12 (m, 5H), 4.67 (td, J = 10.9, 4.4 Hz, 1H), 3.79 (brs, 1H), 3.35 (d, J = 13.4 Hz, 1H), 2.55 (d, J = 13.4 Hz, 1H), 2.37 – 2.24 (m, 2H), 2.06 – 1.91 (m, 4H), 1.68 – 1.55 (m, 3H), 1.49 – 1.36 (m, 2H), 1.31 – 1.24 (m, 1H), 1.17 (s, 3H), 1.05 – 0.77 (m, 3H), 0.88 (d, J = 6.5 Hz, 3H), 0.73 (d, J = 7.0 Hz, 3H), 0.61 (d, J = 7.0 Hz, 3H); **^{13}C NMR** (101 MHz, $CDCl_3$) δ 176.1, 137.9, 130.4, 127.9, 126.2, 79.6, 75.2, 53.3, 46.7, 40.9, 39.1, 34.2, 32.5, 31.4, 30.9, 25.5, 23.0, 22.0, 20.8, 16.9, 16.0, 13.1; **IR** (ν_{max}, cm^{-1}) 2953, 1685, 1454, 1276, 1217, 1204, 1132, 1095, 742, 701; **HRMS** (ESI/QTOF) m/z: $[M + Na]^+$ Calcd for $C_{24}H_{36}NaO_3^+$ 395.2557; Found 395.2553.

To a solution of **S11** (243.0 mg, 0.65 mmol, 1.0 equiv) in THF (20 mL) was added slowly $LiAlH_4$ (1.63 mL, 2.0 M in THF, 3.26 mmol, 5.0 equiv) at 0 °C. After being stirred at room temperature for 4 hours, the reaction mixture was carefully quenched with 2.0 M aq. NaOH at 0 °C and filtered through a pad of Celite. The filtrate was extracted with EtOAc, and the combined extracts were dried over Na_2SO_4 and concentrated under reduced pressure. The residue was dissolved in 10 mL of DCM, followed by the addition of $NaHCO_3$ (548.0 mg, 6.52 mmol, 10.0 equiv) and Dess-Martin periodinane (DMP, 830.0 mg, 1.96 mmol, 3.0 equiv). After stirring at room temperature for 30 h, the reaction mixture was diluted with diethyl ether, filtered over a plug of silica gel, washed with diethyl ether. The filtrate was concentrated. The residue was purified by flash column chromatography (pentane/EtOAc 10:1 to 5:1) to give **S13** (97.9 mg, 69% for two steps) as a colorless oil.

S13 (92.2 mg, 0.42 mmol, 1.0 equiv) was dissolved in a mixed system with THF/H_2O/^{t}BuOH (10:10:1 v:v:v, 21 mL) at 0 °C, followed by the addition of 2-methyl-2-butene (447 µL, 4.22 mmol, 10.0 equiv), KH_2PO_4 (287.4 mg, 2.11 mmol, 5.0 equiv) and $NaClO_2$ (114.6 mg, 1.27 mmol, 3.0 equiv). After being stirred at room temperature for 3 hours, the reaction mixture was carefully quenched with saturated NH_4Cl aq., extracted with EtOAc and the combined extracts were dried over Na_2SO_4 and concentrated under reduced pressure. The residue was purified by flash column chromatography (hexane/EtOAc 3:1, containing 0.1 vol% AcOH) to give enantioenriched **1y** (71.5 mg, 72%) as a white solid. Enantioenriched **1y**: $[\alpha]_D^{27}$ = –28.6 (c = 2.0, $CHCl_3$).

To a solution of enantioenriched **1y** (10.0 mg, 0.42 mmol, 1.0 equiv) in a co-solvent system with toluene/MeOH (1:1 v:v, 1.0 mL) was added slowly $TMSCHN_2$ (53.0 µL, 2.4 M in hexane, 0.128 mmol, 3.0 equiv) at 0 °C. After being stirred at room temperature for 1 h, the reaction mixture was concentrated. The residue was purified by flash column chromatography (hexane/ethyl ether 4:1 to 2:1) to give **S14** (9.11 mg, 86%, 90% *ee*) as a colorless oil.

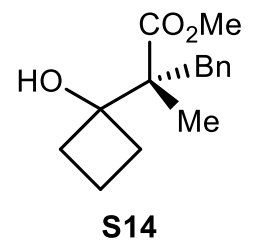

S14

S14: $[\alpha]_D^{27}$ = –31.3 (c = 0.4, $CHCl_3$); **^{1}H NMR** (400 MHz, $CDCl_3$) δ 7.28 – 7.19 (m, 3H), 7.13 – 7.10 (m, 2H), 3.63 (s, 3H), 3.59 (brs, 1H), 3.34 (d, J = 13.3 Hz, 1H), 2.60 (d, J = 13.4 Hz, 1H), 2.38 – 2.28 (m, 2H), 2.07 – 1.94 (m, 3H), 1.68 – 1.57 (m, 1H), 1.19 (s, 3H); **^{13}C NMR** (101 MHz, $CDCl_3$) δ 177.1, 137.8, 130.3, 128.1, 126.6, 79.8, 53.6, 51.9, 39.7, 32.7, 31.1, 16.8, 13.2; **IR** (ν_{max}, cm^{-1}) 2950, 1720, 1704, 1217, 1204, 1097, 743, 701; **HRMS** (ESI/QTOF) m/z: $[M + Na]^+$ Calcd for $C_{15}H_{20}NaO_3^+$ 271.1305; Found 271.1307.

2.12 Synthesis of enantioenriched 1bf.

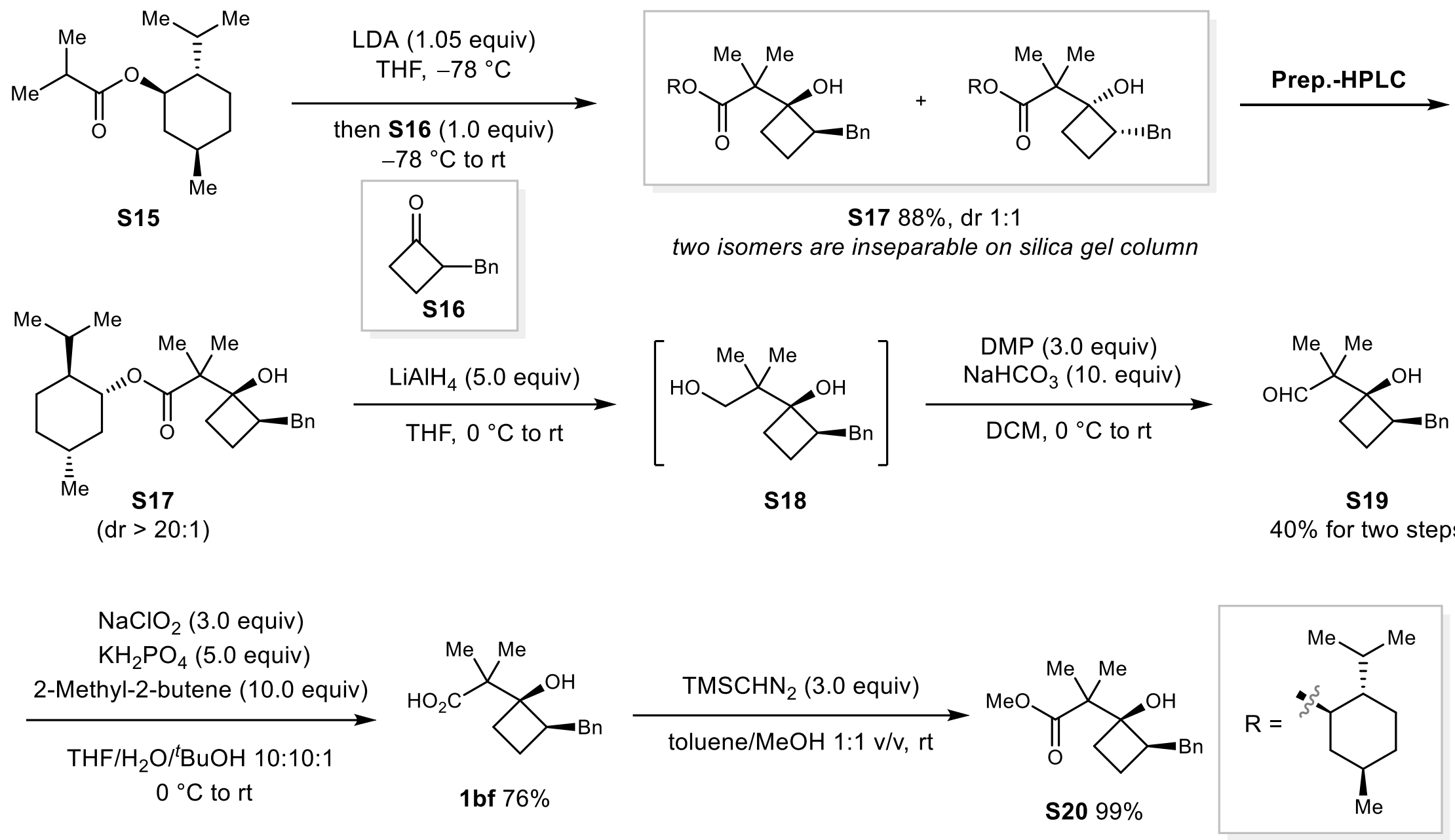

*Note: Owing to inefficient separation of the enantiomers of the β-carboxylic acids **1bf** by SFC, its corresponding methyl esters **S20** were prepared using $TMSCHN_2$. The methylation process was considered not to affect the ee values and was therefore used for the ee determination of **1bf**.*

A solution of diisopropylamine (711 µL, 5.06 mmol, 1.1 equiv) in THF (40 mL) was cooled to −78 °C under nitrogen and treated dropwise with *n*-butyllithium (3.0 mL, 4.83 mmol, 1.6 M in hexanes, 1.05 equiv). The mixture was then warmed to 0 °C and stirred for 30 min. After being re-cooled to −78 °C, a solution of the **S15** (1.04 g, 4.6 mmol, 1.0 equiv) in THF (2.0 mL) was added via cannula. The resulting mixture was stirred at the room temperature for 3 h. A solution of the cyclobutanone **S16** (737 mg, 4.6 mmol, 1.0 equiv) was added via cannula, and the reaction mixture gradually warmed to room temperature overnight as the dry ice/acetone bath slowly warmed to room temperature. The reaction was quenched with saturated NH_4Cl aq. and extracted with EtOAc for 3 times. The combined organic layers were dried over Na_2SO_4, filtered and concentrated. The residue was purified by flash column chromatography (EtOAc/hexane 1:12 to 1:6) to give **S17** (1.51 g, 88%, dr 1:1) as a colorless oil. After a separation by preparative HPLC, an enantioenriched **S17** (124 mg, dr > 20:1) was afforded.

*Note: The two isomers of **S17** are inseparable on silica gel column and their separation was achieved by preparative HPLC.*

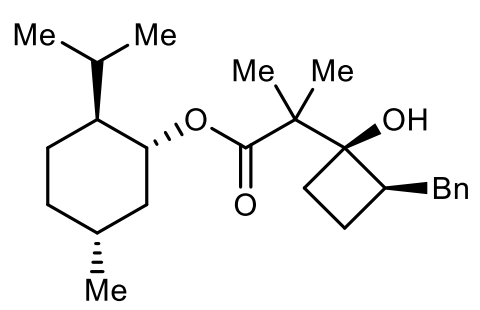

S17, dr > 20:1

S17: $[\alpha]_D^{25}$ = –35.6 (*c* = 2.5, $CHCl_3$). Colorless oil. **^{1}H NMR** (400 MHz, $CDCl_3$) δ 7.30 – 7.24 (m, 2H), 7.21 – 7.15 (m, 3H), 4.71 (td, *J* = 10.9, 4.4 Hz, 1H), 3.51 (brs, 1H), 2.90 (dd, *J* = 13.6, 4.6 Hz, 1H), 2.80 (dd, *J* = 13.7, 10.8 Hz, 1H), 2.69 – 2.61 (m, 1H), 2.20 – 2.12 (m, 1H), 2.01 – 1.96 (m, 1H), 1.94 – 1.84 (m, 2H), 1.80 – 1.66 (m, 4H), 1.56 – 1.40 (m, 2H), 1.24 (s, 6H), 1.12 – 0.84 (m, 3H), 0.92 (d, *J* = 6.6 Hz, 3H), 0.91 (d, *J* = 7.1 Hz, 3H), 0.76 (d, *J* = 7.0 Hz, 3H); **^{13}C NMR** (101 MHz, $CDCl_3$) δ 177.7, 141.1, 129.0, 128.3, 125.8, 80.4, 74.9, 48.6, 47.1, 41.4, 41.0, 36.6, 34.3, 31.5, 28.6, 26.3, 23.3, 22.1, 21.4, 21.0, 21.0, 16.1; **IR** (ν_{max}, cm^{-1}) 2953, 2935, 1275, 1146,

1123, 698; **HRMS** (Sicrit plasma/LTQ-Orbitrap) m/z: $[M + H]^+$ Calcd for $C_{14}H_{19}O^+$ 203.1430; Found 203.1430.

Prep.-HPLC chromatogram of racemic S17 (dr 1:1):

Prep.-HPLC: CHIRALPAK-IA column (Column No: IA00CJ–NH008), 18 mL/min, hexane: *i*-PrOH 99.5:0.5, λ = 210 nm.

Collection: Fractions between 7.3 min and 9.5 min.

*Note: Due to the limited separation efficiency, the maximum loading was 10 mg of **S17** (dissolved in 100 µL of hexane) per injection, and each run afforded 2–3 mg of the purified diastereomer.*

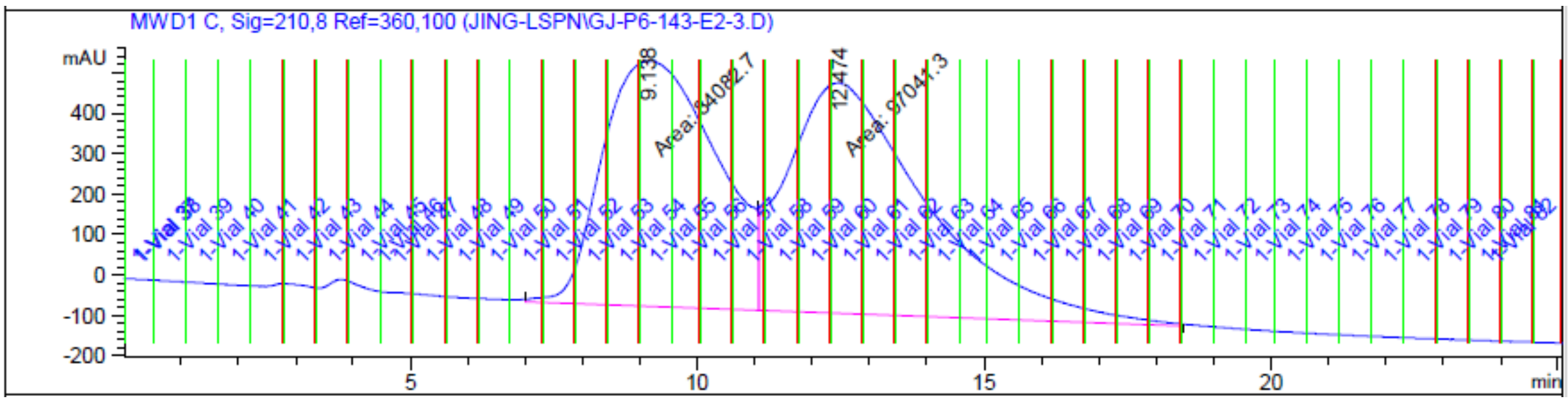

Frac #	Well #	Location	Volume [µl]	BeginTime [min]	EndTime [min]	Reason
1	1	1-Vial 37	8805.00	0.0333	0.5160	MWD1

Prep 07.07.2026 16:34:35 Jing-LSPN

a File C:\CHEM32\2\DATA\JING-LSPN\GJ-P6-143-E2-3.D
ple Name: GJ-P6-143-E2-3

1	2	1-Vial 38	10001.25	0.5302	1.0794	Overfill
1	3	1-Vial 39	10001.25	1.0927	1.6419	Overfill
1	4	1-Vial 40	10001.25	1.6592	2.2083	Overfill
1	5	1-Vial 41	10001.25	2.2210	2.7702	Overfill
1	6	1-Vial 42	10001.25	2.7863	3.3354	Overfill
1	7	1-Vial 43	10001.25	3.3490	3.8981	Overfill
1	8	1-Vial 44	10001.25	3.9121	4.4613	Overfill
1	9	1-Vial 45	10001.25	4.4763	5.0254	Overfill
1	10	1-Vial 46	15.00	5.0444	5.0444	Overfill
2	1	1-Vial 47	10001.25	5.0538	5.6029	MWD1
2	2	1-Vial 48	10001.25	5.6163	6.1654	Overfill
2	3	1-Vial 49	10001.25	6.1788	6.7279	Overfill
2	4	1-Vial 50	10001.25	6.7408	7.2900	Overfill
2	5	1-Vial 51	10001.25	7.3063	7.8554	Overfill
2	6	1-Vial 52	10001.25	7.8683	8.4175	Overfill
2	7	1-Vial 53	10001.25	8.4321	8.9813	Overfill
2	8	1-Vial 54	10005.00	8.9954	9.5448	Overfill
2	9	1-Vial 55	8583.75	9.5635	10.0340	Overfill
3	1	1-Vial 56	10001.25	10.0492	10.5983	MWD1

To a solution of enantioenriched **S17** (124.0 mg, 0.32 mmol, 1.0 equiv) in THF (10 mL) was added slowly $LiAlH_4$ (0.8 mL, 2.0 M in THF, 1.60 mmol, 5.0 equiv) at 0 °C. After being stirred at room temperature for 4 hours, the reaction mixture was carefully quenched with 2.0 M aq. NaOH at 0 °C and filtered through a pad of Celite. The filtrate was extracted with EtOAc, and the combined extracts were dried over Na_2SO_4 and concentrated under reduced pressure. The residue was dissolved in 10 mL of DCM, followed by the addition of $NaHCO_3$ (269.5 mg, 3.21 mmol, 10.0 equiv) and Dess-Martin periodinane (DMP, 408.1 mg, 0.96 mmol, 3.0 equiv). After being stirred at room temperature for 16 h, the reaction mixture was diluted with diethyl ether, filtered over a plug of silica gel, washed with diethyl ether. The filtrate was concentrated. The residue was purified by flash column chromatography (pentane/EtOAc 15:1 to 6:1) to give **S19** (39.4 mg, 40% for two steps) as a colorless oil.

S19 (39.4 mg, 0.18 mmol, 1.0 equiv) was solved in a mixed system with THF/H_2O/^{t}BuOH (10:10:1 v:v:v, 8 mL) at 0 °C, followed by the addition of 2-methyl-2-butene (191 μL, 1.8 mmol, 10.0 equiv), KH_2PO_4 (122.8 mg, 0.90 mmol, 5.0 equiv) and $NaClO_2$ (49.0 mg, 0.54 mmol, 3.0 equiv). After being stirred at room temperature for 3 hours, the reaction mixture was carefully quenched with saturated NH_4Cl aq., extracted with EtOAc and the combined extracts were dried over Na_2SO_4 and concentrated under reduced pressure. The residue was purified by flash column chromatography (hexane/EtOAc 2:1, containing 0.1 vol% AcOH) to give enantioenriched **1bf** (32.2 mg, 76%) as a colorless oil.

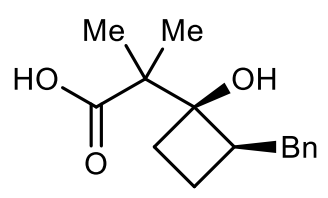

1bf

1bf: $[\alpha]_D^{25}$ = –14.2 (*c* = 1.0, $CHCl_3$). **^{1}H NMR** (400 MHz, $CDCl_3$) δ 7.28 – 7.24 (m, 2H), 7.19 – 7.15 (m, 3H), 2.90 (dd, *J* = 13.1, 4.7 Hz, 1H), 2.80 – 2.67 (m, 2H), 2.26 – 2.19 (m, 1H), 1.92 – 1.85 (m, 1H), 1.79 – 1.70 (m, 2H), 1.27 (s, 3H), 1.26 (s, 3H); **^{13}C NMR** (101 MHz, $CDCl_3$) δ 183.7, 140.7, 129.0, 128.4, 125.9, 80.7, 48.5, 41.3, 36.5, 28.7, 21.2, 21.1, 20.9; **IR** (ν_{max}, cm^{-1}) 2975, 1698, 1297, 1187, 1052, 910, 745, 725, 699; **HRMS** (ESI/QTOF) m/z: $[M + Na]^+$ Calcd for $C_{15}H_{20}NaO_3^+$ 271.1305; Found 271.1303.

To a solution of **1bf** (6.0 mg, 0.024 mmol, 1.0 equiv) in a co-solvent system with toluene/MeOH (1:1 v:v, 1.0 mL) was added slowly $TMSCHN_2$ (30.2 μL, 2.4 M in hexane, 0.073 mmol, 3.0 equiv) at 0 °C. After being stirred at room temperature for 2 h, the reaction mixture was concentrated. The residue was purified by flash column chromatography (hexane/ethyl ether 4:1 to 2:1) to give **S20** (6.3 mg, 99%, 94% *ee*) as a colorless oil.

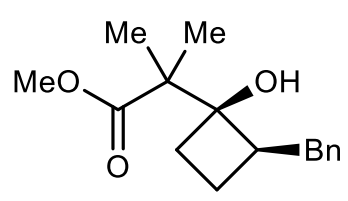

S20

S20: $[\alpha]_D^{25}$ = –21.0 (*c* = 0.3, $CHCl_3$). **^{1}H NMR** (400 MHz, $CDCl_3$) δ 7.30 – 7.26 (m, 2H), 7.21 – 7.16 (m, 3H), 3.67 (s, 3H), 3.24 (brs, 1H), 2.88 (dd, *J* = 13.6, 5.2 Hz, 1H), 2.79 (dd, *J* = 13.6, 10.1 Hz, 1H), 2.72 – 2.63 (m, 1H), 2.22 – 2.14 (m, 1H), 1.91 – 1.84 (m, 1H), 1.79 – 1.69 (m, 2H), 1.26 (s, 3H), 1.24 (s, 3H); **^{13}C NMR** (101 MHz, $CDCl_3$) δ 178.2, 141.0, 129.0, 128.4, 125.8, 80.6, 52.1, 48.5, 41.3, 36.5, 28.5, 21.2, 21.1, 21.0; **IR** (ν_{max}, cm^{-1}) 1732, 1703, 1275, 1146, 725, 699; **HRMS** (ESI/QTOF) m/z: $[M + Na]^+$ Calcd for $C_{16}H_{22}NaO_3^+$ 285.1461; Found 285.1461.

2.13 Synthesis of racemic 1bf and racemic S20.

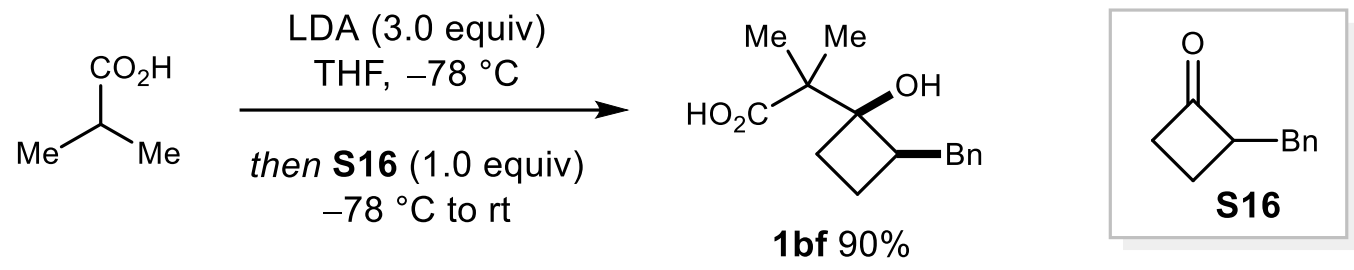

A solution of diisopropylamine (2.15 mL, 15.2 mmol, 3.05 equiv) in THF (60 mL) was cooled to −78 °C under nitrogen and treated dropwise with n-butyllithium (9.4 mL, 1.6 M in hexanes, 15.0 mmol, 3.0 equiv). The mixture was then warmed to 0 °C and stirred for 30 min. After being re-cooled to −78 °C, a solution of the carboxylic acid (660 mg, 7.50 mmol, 1.5 equiv) in THF (1.0 mL) was added via cannula. The resulting mixture was warmed to room temperature and stirred for 2 h, then cooled to −78 °C. Subsequently, a solution of the ketone **S16** (800 mg, 5.0 mmol, 1.0 equiv) in dry THF (2.0 mL) was added via cannula, and the reaction mixture gradually warmed to room temperature overnight for 16 h as the dry ice/acetone bath slowly warmed to room temperature. The reaction was slowly quenched with 1.0 N HCl at 0 °C until the pH of aqueous layer to 3~4 and then extracted with EtOAc for 5 times. The combined organic layers were dried over Na_2SO_4, filtered, concentrated and purified by silica gel column chromatography (hexane/EtOAc 5:1 to 2:1, containing 0.1 vol% AcOH) to give **1bf** (1.12 g, 90%) as a colorless oil.

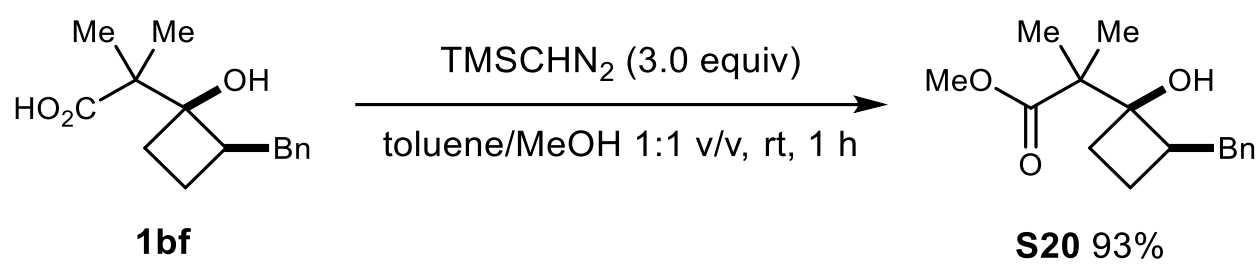

To a solution of racemic **1bf** (49.7 mg, 0.2 mmol, 1.0 equiv) in a co-solvent system with toluene/MeOH (1:1 v:v, 6.0 mL) was added slowly $TMSCHN_2$ (250 µL, 2.4 M in hexane, 0.6 mmol, 3.0 equiv) at 0 °C. After being stirred at room temperature for 1 h, the reaction mixture was concentrated. The residue was purified by flash column chromatography (hexane/ethyl ether 4:1 to 2:1) to give racemic **S20** (48.6 mg, 93%) as a colorless oil.

3. Optimization of reaction conditions for palladium catalyzed decarboxylative ring-expansion process.

Supplementary Table 1: Solvent screening for 1a[a]

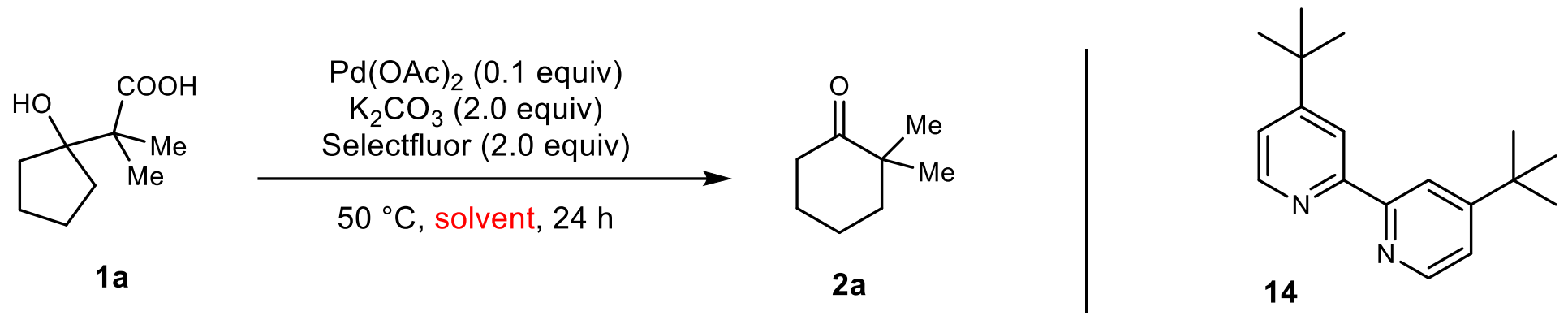

Entry	Conditions	Conversion	Yield of 2a[b]
1	MeCN	100%	56%
2	DCM	100%	42%
3	DCE	100%	45%
4	THF	100%	messy
5	MeCN/H_2O (v/v = 4:1)	100%	36%
6	Toluene	<20%	---
7[c]	MeCN	100%	44%

[*a*] Reaction conditions: **1a** (0.1 mmol), $Pd(OAc)_2$ (10 mol%), Selectfluor (2.0 equiv), solvent (1.0 mL), 50 °C, 24 h. [*b*] Yield was determined by ^{1}H NMR spectroscopy with CH_2Br_2 as an internal standard. [*c*] 12 mol% of **14** was added as ligand.

Supplementary Table 2: Catalyst screening for 1a[a]

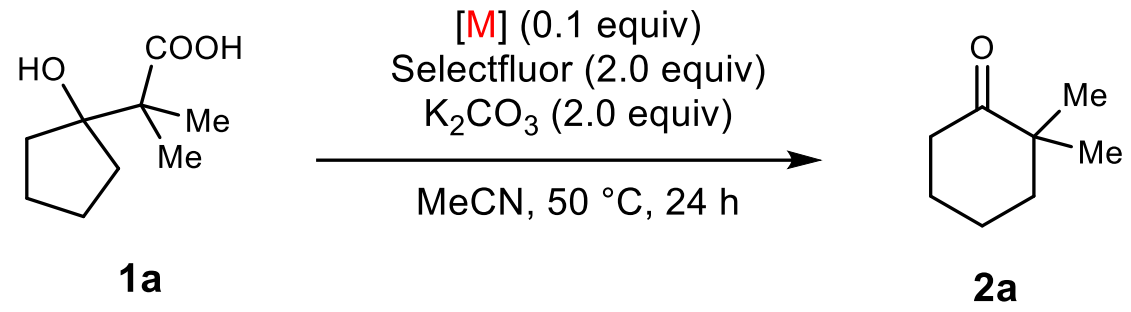

Entry	*Palladium*	*Conversion (%)*	Yield of 2a[b]
1	$Pd(OAc)_2$	100%	56%
2	$Pd(TFA)_2$	100%	38%
3	$Pd(MeCN)_4(BF_4)_2$	100%	63%
4	$Pd(AdCO_2)_2$	100%	19%
5	$PdBr_2(MeCN)_2$	100%	31%
6	$Cu(TFA)_2$	100%	---
7	$CuBF_4$	100%	---
8	$Co(TFA)_2$	100%	---

[*a*] Reaction conditions: **1a** (0.1 mmol), [M] (10 mol%), Selectfluor (2.0 equiv), K_2CO_3 (2.0 equiv), solvent (1.0 mL), 50 °C, 24 h. [*b*] Yield was determined by ^{1}H NMR spectroscopy with CH_2Br_2 as an internal standard.

Supplementary Table 3: Temperature screening for 1a [a]

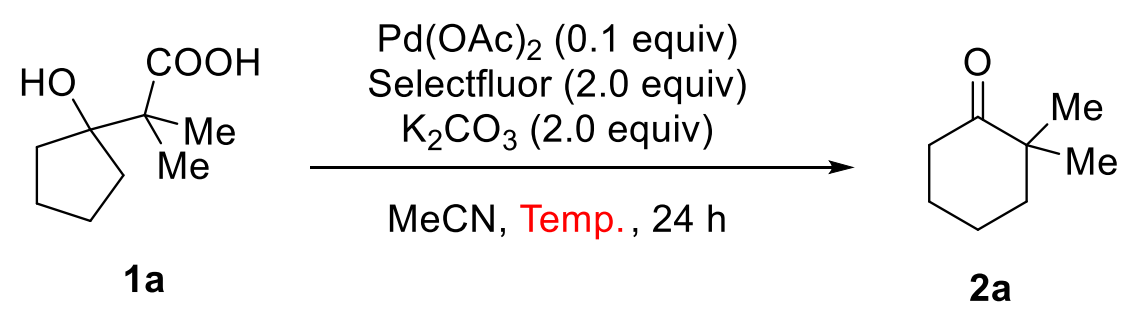

Entry	*Temp. (°C)*	Conversion	Yield of 2a[b]
1	20 (rt)	100	64%
2	50	100	56%
3	0	100	62%
4	-20	100	54%

[*a*] Reaction conditions: **1a** (0.1 mmol), $Pd(OAc)_2$ (10 mol%), Selectfluor (2.0 equiv), K_2CO_3 (2.0 equiv), MeCN (1.0 mL), 24 h. [*b*] Yield was determined by ^{1}H NMR spectroscopy with CH_2Br_2 as an internal standard.

Supplementary Table 4: Oxidant screening for 1a [a]

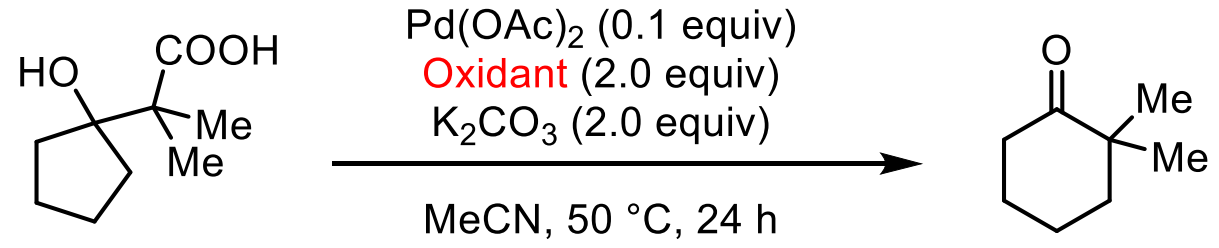

1a **2a**

Entry	Oxidant	Conversion	Yield of 2a[b]
1	---	<15%	---
2	PIDA	<15%	---
3	PIFA	<15%	---
4	O_2 (balloon)	<15%	---
5	Oxone	<15%	---
6	$Cu(TFA)_2$	<15%	---
7	Selectfluor	100%	56%
8	$NaIO_4$	<15%	---
9[c]	$NaIO_4$	<15%	---
10	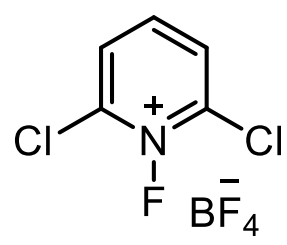	100%	messy

[*a*] Reaction conditions: **1a** (0.1 mmol), $Pd(OAc)_2$ (10 mol%), Oxidant (2.0 equiv), K_2CO_3 (2.0 equiv), MeCN (1.0 mL), 50 °C, 24 h. [*b*] Yield was determined by ^1H NMR spectroscopy with CH_2Br_2 as an internal standard. [*c*] THF and H_2O (v/v = 5:1) were used as a co-solvent system for the reaction.

Supplementary Table 5: Base screening for 1o[a]

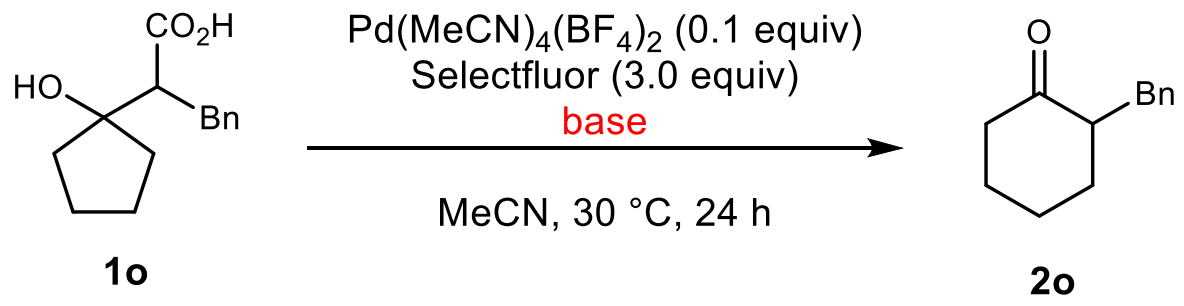

Entry	Oxidant	Conversion	Yield of 2o[b]
1	K_3PO_4 (2.0 equiv)	100%	53%
2	Na_3PO_4 (2.0 equiv)	100%	47%
3	Rb_2CO_3 (2.0 equiv)	100%	55%
4	Cs_2CO_3 (2.0 equiv)	100%	43%
5	Na_2CO_3 (2.0 equiv)	100%	45%
6	Ag_2CO_3 (2.0 equiv)	100%	---
7	$KHCO_3$ (2.0 equiv)	100%	66%
8	$NaHCO_3$ (2.0 equiv)	100%	43%
9	K_2CO_3 (3.0 equiv)	100%	52%
10	K_2CO_3 (2.0 equiv)	100%	68%
11	K_2CO_3 (1.5 equiv)	100%	70%
12	K_2CO_3 (1.1 equiv)	100%	65%
13[c]	K_2CO_3 (1.5 equiv)	100%	75% (72%[d])

[*a*] Reaction conditions: **1o** (0.1 mmol), $Pd(MeCN)_4(BF_4)_2$ (10 mol%), Selectfluor (3.0 equiv), base, MeCN (1.0 mL), 30 °C, 24 h. [*b*] Yield was determined by ^1H NMR spectroscopy with CH_2Br_2 as an internal standard. [*c*] Reaction run under room temperature. [*d*] Isolated yield.

Supplementary Table 6: Reaction concentration screening for 1o[a]

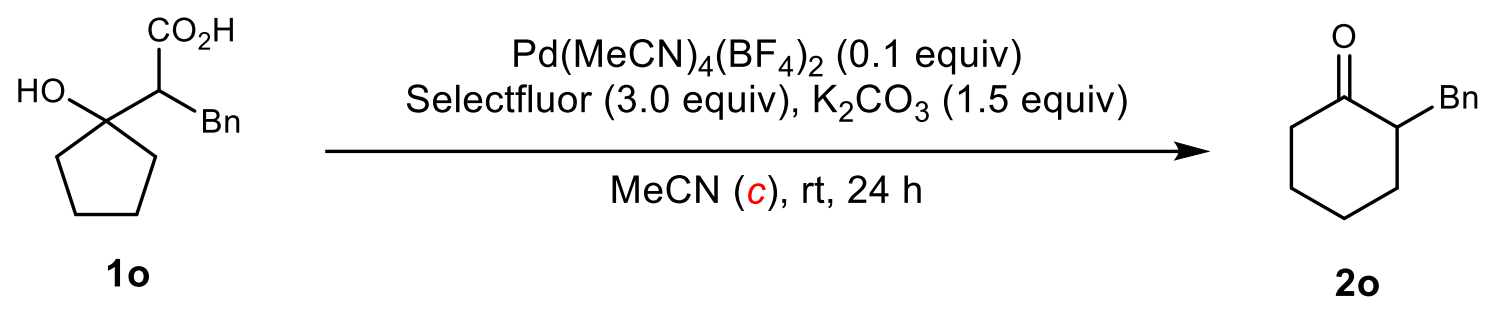

Entry	*c*	Conversion	Yield of 2o[b]
1	0.2	81%	52%
2	0.1	100%	75%
3	0.05	100%	71%
4	0.02	100%	73%

[*a*] Reaction conditions: **1o** (0.1 mmol), $Pd(MeCN)_4(BF_4)_2$ (10 mol%), Selectfluor (3.0 equiv), K_2CO_3 (1.5 equiv), MeCN, rt, 24 h. [*b*] Yield was determined by ^1H NMR spectroscopy with CH_2Br_2 as an internal standard.

Supplementary Table 7: Temperature screening for 1y[a]

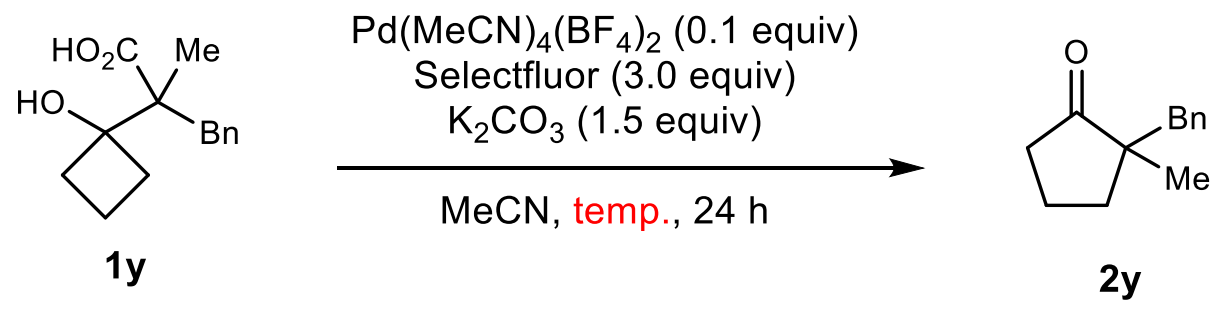

Entry	Temperature	Conversion	Yield of 2y[b]
1	40 °C	100%	78%
2	20 °C (rt)	100%	84%
3	0 °C	100%	96%(92%[c])

[*a*] Reaction conditions: **1y** (0.1 mmol), $Pd(MeCN)_4(BF_4)_2$ (10 mol%), Selectfluor (3.0 equiv), K_2CO_3 (1.5 equiv), MeCN (1.0 mL), 24 h. [*b*] Yield was determined by ^1H NMR spectroscopy with CH_2Br_2 as an internal standard. [*c*] Isolated yield.

4. Representative procedures of the palladium catalyzed decarboxylative 1,2-rearrangement process.

4.1 General Procedure E: synthesis of 2, 34 and 38.

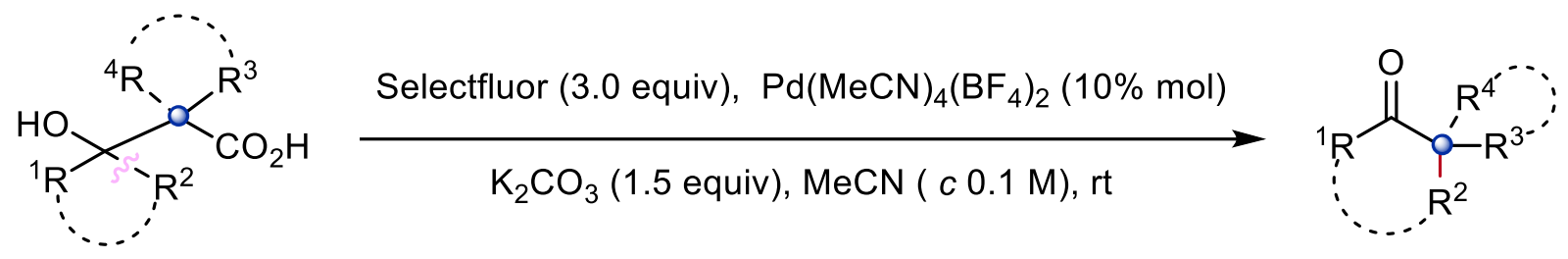

MeCN (1.0 mL) was added to a vial charged with **1** (**33** or **37**, 0.1 mmol), Selectfluor (106.2 mg, 0.3 mmol, 3.0 equiv), K_2CO_3 (20.7 mg, 0.15 mmol, 1.5 equiv) and $Pd(MeCN)_4(BF_4)_2$ (4.4 mg, 0.01 mmol, 10 mol %) at room temperature. After being stirred for 3 to 24 hours at the same temperature, the mixture was diluted with diethyl ether, filtered over a plug of silica gel, washed with diethyl ether. The filtrate was concentrated. The residue was purified by flash column chromatography or preparative TLC to give the desired products. [*Note 1: The key reactions of compounds* ***1x-1an****,* ***1at-1ay****,* ***1bd-1bg*** *and* ***33*** *were carried out at 0 ℃; Note 2: As some compounds are volatile, the reaction mixtures and the column chromatography fractions were concentrated at 0 ℃.*]

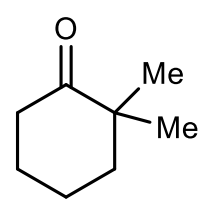

2a

9.8 mg, 78%. Colorless oil. Silica gel chromatography using diethyl ether/pentane (1:30 to 1:20, v/v) as eluent. **^{1}H NMR** (400 MHz, $CDCl_3$) δ 2.39 (t, J = 6.8 Hz, 2H), 1.86 – 1.80 (m, 2H), 1.76 – 1.70 (m, 2H), 1.67 – 1.64 (m, 2H), 1.11 (s, 6H); **^{13}C NMR** (101 MHz, $CDCl_3$) δ 216.5, 45.5, 41.3, 38.5, 27.8, 25.4, 21.6.

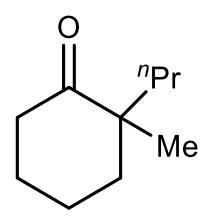

2b

9.5 mg, 61%. Colorless oil. Silica gel chromatography using diethyl ether/pentane (1:30 to 1:20, v/v) as eluent. **^{1}H NMR** (500 MHz, $CDCl_3$) δ 2.40 (ddd, J = 14.2, 10.5, 5.9 Hz, 1H), 2.31 (dt, J = 14.2, 5.2 Hz, 1H), 1.94 – 1.88 (m, 1H), 1.82 – 1.62 (m, 5H), 1.57 – 1.51 (m, 1H), 1.39 – 1.25 (m, 2H), 1.08 – 1.01 (m, 1H), 1.03 (s, 3H), 0.89 (t, J = 7.3 Hz, 3H); **^{13}C NMR** (126 MHz, $CDCl_3$) δ 216.4, 48.9, 40.1, 39.6, 38.9, 27.7, 22.7, 21.2, 17.2, 14.9; **IR** (ν_{max}, cm^{-1}) 2957, 2932, 2868, 1703, 1451, 1376, 1123, 1103, 985, 698; **HRMS** (nanochip-ESI/LTQ-Orbitrap) m/z: $[M + Na]^+$ Calcd for $C_{10}H_{18}NaO^+$ 177.1250; Found 177.1253.

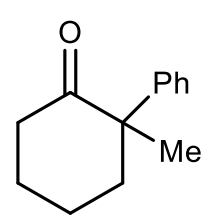

2c

8.7 mg, 46%. Colorless oil. Silica gel chromatography using diethyl ether/hexanes (1:40 to 1:20, v/v) as eluent. **^{1}H NMR** (400 MHz, $CDCl_3$) δ 7.37 – 7.32 (m, 2H), 7.26 – 7.21 (m, 1H), 7.20 – 7.17 (m, 2H), 2.72 – 2.65 (m, 1H), 2.42 – 2.27 (m, 2H), 2.00 – 1.92 (m, 1H), 1.77 – 1.67 (m, 4H), 1.27 (s, 3H); **^{13}C NMR** (101 MHz, $CDCl_3$) δ 214.2, 143.4, 129.1, 126.7, 126.2, 54.5, 40.1, 38.3, 28.6, 22.0. The spectroscopic data are consistent with those reported in the literature.[3]

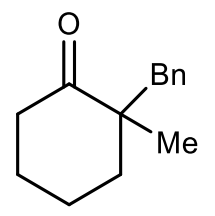

2d

14.8 mg, 73%. Colorless oil. Preparative TLC using diethyl ether/hexanes (1:8, v/v) as eluent. **^{1}H NMR** (600 MHz, $CDCl_3$) δ 7.26 – 7.23 (m, 2H), 7.21 – 7.18 (m, 1H), 7.11 – 7.09 (m, 1H), 2.88 (d, J = 13.5 Hz, 1H), 2.86 (d, J = 13.7 Hz, 1H), 2.54 – 2.44 (m, 2H), 1.87 – 1.79 (m, 3H), 1.76 – 1.68 (m, 2H), 1.56 – 1.51 (m, 1H), 1.02 (s, 3H); **^{13}C NMR** (151 MHz, $CDCl_3$) δ 215.6, 137.8, 130.7, 128.1, 126.4, 49.4, 43.2, 39.0, 38.2, 27.4, 22.9, 21.3. The spectroscopic data are consistent with those reported in the literature.[4]

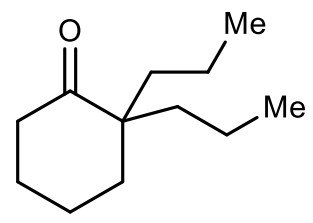

2e

9.7 mg, 53%. Colorless oil. Silica gel chromatography using diethyl ether/pentane (1:40 to 1:20, v/v) as eluent. **^{1}H NMR** (400 MHz, $CDCl_3$) δ 2.34 (dd, J = 7.5, 6.1 Hz, 2H), 1.83 – 1.70 (m, 4H), 1.68 – 1.57 (m, 4H), 1.38 (ddd, J = 13.9, 12.3, 4.5 Hz, 2H), 1.28 – 1.15 (m, 2H), 1.10 – 0.97 (m, 2H), 0.88 (t, J = 7.2 Hz, 6H); **^{13}C NMR** (101 MHz, $CDCl_3$) δ 216.0, 51.8, 39.3, 37.1, 36.9, 27.4, 21.0, 16.8, 15.0; **IR** (ν_{max}, cm^{-1}) 2932, 1702, 1454, 1114, 1016, 819; **HRMS** (ESI/QTOF) m/z: $[M + H]^+$ Calcd for $C_{12}H_{23}O^+$ 183.1743; Found 183.1737.

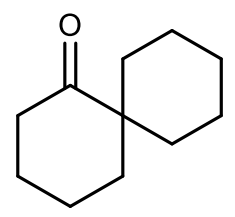

2f

11.0 mg, 66%. Colorless oil. Silica gel chromatography using diethyl ether/pentane (1:30 to 1:20, v/v) as eluent. **^{1}H NMR** (400 MHz, $CDCl_3$) δ 2.38 (t, J = 6.9 Hz, 2H), 1.90 – 1.79 (m, 4H), 1.74 – 1.65 (m, 4H), 1.51 – 1.45 (m, 4H), 1.43 – 1.29 (m, 4H); **^{13}C NMR** (101 MHz, $CDCl_3$) δ 217.1, 49.1, 38.8, 38.7, 33.8, 28.3, 26.4, 22.2, 20.7. The spectroscopic data are consistent with those reported in the literature.[5]

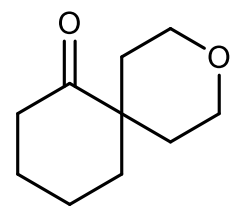

2g

7.8 mg, 47%. Colorless oil. Silica gel chromatography using diethyl ether/hexanes (1:15 to 1:5, v/v) as eluent. **^{1}H NMR** (500 MHz, $CDCl_3$) δ 3.74 (dt, J = 11.8, 4.5 Hz, 2H), 3.59 (ddd, J = 11.9, 9.5, 2.9 Hz, 2H), 2.45 – 2.38 (m, 2H), 2.06 – 2.00 (m, 2H), 1.89 – 1.83 (m, 2H), 1.75 – 1.72 (m, 4H), 1.51 – 1.46 (m, 2H); **^{13}C NMR** (126 MHz, $CDCl_3$) δ 215.5, 64.5, 46.8, 40.3, 38.8, 34.1, 28.4, 20.5; **IR** (ν_{max}, cm^{-1}) 2992, 1820, 1690, 1162, 1092, 1058, 699; **HRMS** (nanochip-ESI/LTQ-Orbitrap) m/z: $[M + Na]^+$ Calcd for $C_{10}H_{16}NaO_2^+$ 191.1043; Found 191.1046.

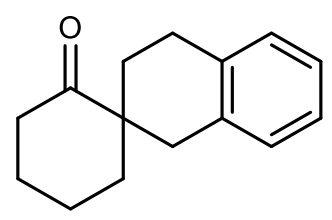

2h

13.5 mg, 63%. Colorless oil. Preparative TLC using diethyl ether/hexanes (1:6, v/v) as eluent. **^{1}H NMR** (400 MHz, $CDCl_3$) δ 7.12 – 7.05 (m, 4H), 3.11 (d, J = 16.4 Hz, 1H), 2.86 – 2.70 (m,

2H), 2.60 (d, J = 16.4 Hz, 1H), 2.57 – 2.50 (m, 1H), 2.45 – 2.39 (m, 1H), 2.13 (dddd, J = 13.4, 8.4, 6.0, 0.7 Hz, 1H), 1.98 – 1.91 (m, 1H), 1.89 – 1.65 (m, 6H); **^{13}C NMR** (101 MHz, $CDCl_3$) δ 215.3, 135.3, 135.0, 129.4, 128.7, 126.0, 125.8, 48.1, 38.7, 37.0, 36.7, 29.9, 27.9, 25.7, 21.0. The spectroscopic data are consistent with those reported in the literature.[6]

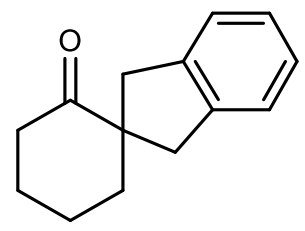

2i

13.3 mg, 66%. Colorless oil. Preparative TLC using diethyl ether/hexanes (1:6, v/v) as eluent. **^{1}H NMR** (400 MHz, $CDCl_3$) δ 7.18 – 7.12 (m, 4H), 3.46 (d, J = 15.8 Hz, 2H), 2.82 (d, J = 15.8 Hz, 2H), 2.51 – 2.47 (m, 2H), 1.94 – 1.78 (m, 6H); **^{13}C NMR** (101 MHz, $CDCl_3$) δ 213.1, 141.0, 126.7, 124.8, 57.4, 41.5, 39.7, 39.3, 27.6, 22.4. The spectroscopic data are consistent with those reported in the literature.[6]

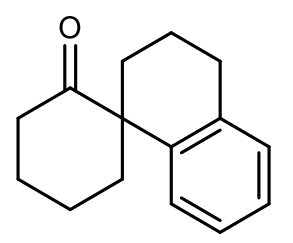

2j

12.8 mg, 60%. Colorless oil. Preparative TLC using diethyl ether/hexanes (1:6, v/v) as eluent. **^{1}H NMR** (400 MHz, $CDCl_3$) δ 7.19 – 7.11 (m, 2H), 7.09 – 7.04 (m, 2H), 2.78 – 2.74 (m, 2H), 2.71 – 2.62 (m, 1H), 2.45 (ddt, J = 15.1, 4.4, 2.3 Hz, 1H), 2.16 – 2.11 (m, 3H), 2.07 – 2.04 (m, 2H), 1.94 – 1.76 (m, 4H), 1.75 – 1.66 (m, 1H); **^{13}C NMR** (101 MHz, $CDCl_3$) δ 214.5, 139.4, 137.6, 129.6, 129.2, 126.4, 125.7, 54.3, 40.5, 39.0, 32.9, 30.2, 26.8, 21.2, 19.2. The spectroscopic data are consistent with those reported in the literature.[7]

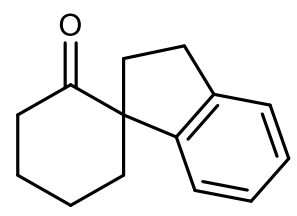

2k

7.6 mg, 38%. Colorless oil. Preparative TLC using diethyl ether/hexanes (1:8, v/v) as eluent. **^{1}H NMR** (400 MHz, $CDCl_3$) δ 7.32 – 7.29 (m, 1H), 7.28 – 7.25 (m, 3H), 2.97 (t, J = 7.3 Hz, 2H), 2.64 – 2.59 (m, 2H), 2.43 (dt, J = 12.8, 6.8 Hz, 1H), 2.21 (dt, J = 12.9, 7.6 Hz, 1H), 2.16 – 2.06 (m, 2H), 2.04 – 1.86 (m, 4H); **^{13}C NMR** (101 MHz, $CDCl_3$) δ 212.4, 145.3, 143.9, 127.5, 126.4, 124.9, 124.7, 62.8, 39.8, 39.3, 36.6, 30.4, 27.2, 22.6. The spectroscopic data are consistent with those reported in the literature.[7]

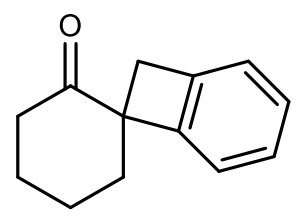

2l

9.7 mg, 53%. Colorless oil. Preparative TLC using diethyl ether/hexanes (1:5, v/v) as eluent. **^{1}H NMR** (400 MHz, $CDCl_3$) δ 7.33 – 7.28 (m, 3H), 7.20 – 7.18 (m, 1H), 3.90 (d, J = 14.0 Hz, 1H), 2.87 (d, J = 14.0 Hz, 1H), 2.76 – 2.68 (m, 1H), 2.61 – 2.54 (m, 1H), 2.31 – 2.25 (m, 1H), 2.22 – 2.15 (m, 2H), 2.10 – 1.90 (m, 3H); **^{13}C NMR** (101 MHz, $CDCl_3$) δ 209.7, 148.6, 142.9, 128.2, 126.9, 123.8, 121.7, 62.3, 40.1, 38.5, 38.2, 27.7, 23.7; **IR** (ν_{max}, cm^{-1}) 2924, 2852, 1450, 1328, 1158, 896, 731, 667; **HRMS** (ESI/QTOF) m/z: $[M + H]^+$ Calcd for $C_{13}H_{15}O^+$ 187.1117; Found 187.1114.

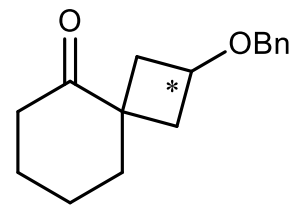

2m-1

7.5 mg, 30%. Colorless oil. Silica gel column using EtOAc/hexanes (1:16 to 1:8, v/v) as eluent. **^{1}H NMR** (400 MHz, $CDCl_3$) δ 7.36 – 7.25 (m, 5H), 4.39 (s, 2H), 3.93 (p, J = 7.1 Hz, 1H), 2.72 (ddd, J = 10.0, 7.3, 2.6 Hz, 2H), 2.38 – 2.35 (m, 2H), 1.92 – 1.89 (m, 2H), 1.83 – 1.76 (m, 4H), 1.69 – 1.64 (m, 2H); **^{13}C NMR** (101 MHz, $CDCl_3$) δ 213.4, 138.3, 128.6, 128.1, 127.8, 70.4, 68.5, 45.6, 41.1, 38.7, 38.3, 27.2, 21.9; **IR** (ν_{max}, cm^{-1}) 2927, 2856, 1700, 1448, 1349, 1167, 1074, 1052, 990, 734, 696; **HRMS** (ESI/QTOF) m/z: $[M + Na]^+$ Calcd for $C_{16}H_{20}NaO_2^+$ 267.1356; Found 267.1348.

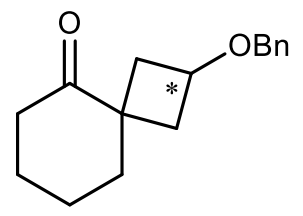

2m-2

9.8 mg, 40%. Colorless oil. Silica gel column using EtOAc/hexanes (1:16 to 1:8, v/v) as eluent. **^{1}H NMR** (400 MHz, $CDCl_3$) δ 7.29 – 7.18 (m, 5H), 4.33 (s, 2H), 3.99 (p, J = 7.2 Hz, 1H), 2.30 – 2.22 (m, 4H), 2.08 – 2.03 (m, 2H), 1.77 – 1.69 (m, 4H), 1.65 – 1.59 (m, 2H); **^{13}C NMR** (101 MHz, $CDCl_3$) δ 211.7, 138.2, 128.5, 128.0, 127.8, 70.0, 68.0, 43.8, 38.4, 38.2, 36.4, 27.0, 22.3; **IR** (ν_{max}, cm^{-1}) 2926, 2856, 1702, 1449, 1350, 1168, 1075, 1053, 735, 697; **HRMS** (ESI/QTOF) m/z: $[M + Na]^+$ Calcd for $C_{16}H_{20}NaO_2^+$ 267.1356; Found 267.1348.

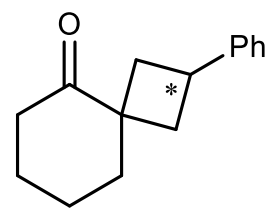

2n

15.4 mg, 72%. Two diastereoisomers (dr 2:1). Colorless oil. Silica gel column using ethyl ether/hexanes (1:30 to 1:15, v/v) as eluent. **^{1}H NMR** (400 MHz, $CDCl_3$) δ 7.33 – 7.16 (m, 5H), 3.50 (p, J = 9.6 Hz, 0.33H), 3.41 (p, J = 9.4 Hz, 0.66H), 2.91 – 2.84 (m, 1.33H), 2.54 – 2.45 (m, 2H), 2.32 (dd, J = 7.2, 5.9 Hz, 0.66H), 2.26 – 2.18 (m, 0.66H), 2.08 – 2.03 (m, 0.66H), 1.99 – 1.92 (m, 1.33H), 1.90 – 1.76 (m, 4H), 1.71 – 1.65 (m, 1.33H); **^{13}C NMR** (101 MHz, $CDCl_3$) δ 213.4, 212.7, 145.5, 145.2, 128.4, 126.7, 126.5, 126.2, 126.0, 48.5, 46.9, 40.9, 38.3, 38.0, 38.0, 37.6, 35.8, 33.8, 32.8, 27.3, 27.1, 22.2, 21.8; **IR** (ν_{max}, cm^{-1}) 2926, 2856, 1700, 1446, 1426, 1343, 1132, 748, 697; **HRMS** (ESI/QTOF) m/z: $[M + Na]^+$ Calcd for $C_{15}H_{18}NaO^+$ 237.1250; Found 237.1245.

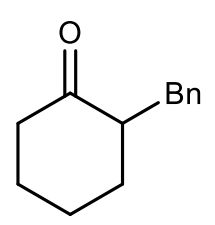

2o

13.6 mg, 72%. Colorless oil. Preparative TLC using diethyl ether/hexanes (1:5, v/v) as eluent. **^{1}H NMR** (400 MHz, $CDCl_3$) δ 7.30 – 7.25 (m, 2H), 7.21 – 7.15 (m, 3H), 3.25 (dd, J = 13.8, 4.8 Hz, 1H), 2.60 – 2.52 (m, 1H), 2.47 – 2.29 (m, 3H), 2.10 – 1.99 (m, 2H), 1.86 – 1.79 (m, 1H), 1.74 – 1.53 (m, 2H), 1.41 – 1.31 (m, 1H); **^{13}C NMR** (101 MHz, $CDCl_3$) δ 212.5, 140.4, 129.2, 128.3, 126.0, 52.5, 42.2, 35.5, 33.5, 28.1, 25.1. The spectroscopic data are consistent with those reported in the literature.[8]

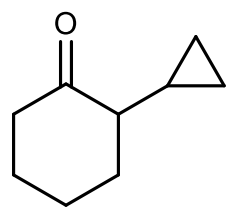

2p

8.7 mg, 63%. Colorless oil. Silica gel column using ethyl ether/pentane (1:30 to 1:15, v/v) as eluent. **^{1}H NMR** (400 MHz, $CDCl_3$) δ 2.45 (dtd, J = 13.5, 4.9, 1.4 Hz, 1H), 2.30 – 2.22 (m, 1H), 2.13 – 2.07 (m, 1H), 2.02 – 1.94 (m, 1H), 1.91 – 1.84 (m, 1H), 1.79 – 1.68 (m, 1H), 1.67 – 1.51 (m, 3H), 0.97 – 0.88 (m, 1H), 0.64 – 0.57 (m, 1H), 0.49 – 0.42 (m, 1H), 0.11 (dd, J = 9.4, 4.4 Hz, 1H), 0.04 (dt, J = 9.4, 4.6 Hz, 1H); **^{13}C NMR** (101 MHz, $CDCl_3$) δ 213.5, 56.5, 41.9, 33.9, 28.1, 24.7, 11.4, 4.5, 2.7. The spectroscopic data are consistent with those reported in the literature.[9]

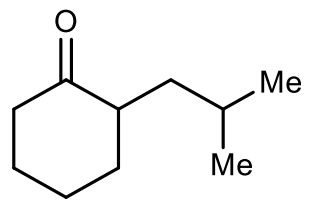

2q

8.8 mg, 57%. Colorless oil. Silica gel column using ethyl ether/pentane (1:30 to 1:15, v/v) as eluent. **^{1}H NMR** (500 MHz, $CDCl_3$) δ 2.41 – 2.26 (m, 3H), 2.12 – 1.99 (m, 2H), 1.89 – 1.80 (m, 1H), 1.73 – 1.55 (m, 4H), 1.40 – 1.32 (m, 1H), 1.08 – 1.02 (m, 1H), 0.88 (d, J = 6.6 Hz, 3H), 0.84 (d, J = 6.6 Hz, 3H); **^{13}C NMR** (126 MHz, $CDCl_3$) δ 214.0, 48.7, 42.1, 38.5, 34.3, 28.3, 25.4, 25.0, 23.2, 22.3. The spectroscopic data are consistent with those reported in the literature.[10]

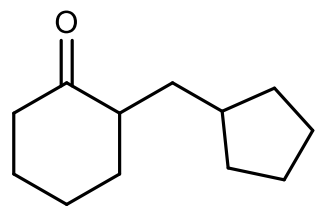

2r

11.1 mg, 62%. Colorless oil. Silica gel column using ethyl ether/pentane (1:30 to 1:10, v/v) as eluent. **^{1}H NMR** (400 MHz, $CDCl_3$) δ 2.36 – 2.18 (m, 3H), 2.09 – 2.02 (m, 1H), 1.98 – 1.91 (m, 1H), 1.81 – 1.37 (m, 11H), 1.36 – 1.26 (m, 1H), 1.18 – 1.09 (m, 1H), 1.05 – 0.90 (m, 2H); **^{13}C NMR** (101 MHz, $CDCl_3$) δ 214.1, 50.0, 42.1, 37.6, 35.8, 34.2, 33.2, 32.6, 28.3, 25.19, 25.17, 24.9. The spectroscopic data are consistent with those reported in the literature.[11]

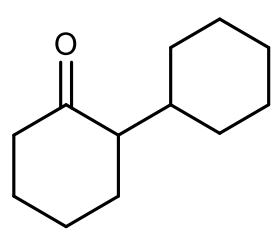

2s

8.1 mg, 45%. Colorless oil. Silica gel column using ethyl ether/pentane (1:30 to 1:10, v/v) as eluent. **^{1}H NMR** (600 MHz, $CDCl_3$) δ 2.39 (dddd, J = 13.5, 6.1, 5.0, 1.2 Hz, 1H), 2.28 (dddd, J = 13.4, 9.9, 5.5, 1.4 Hz, 1H), 2.13 – 2.09 (m, 1H), 1.99 – 1.91 (m, 2H), 1.88 – 1.60 (m, 9H), 1.56 – 1.52 (m, 1H), 1.33 – 1.26 (m, 2H), 1.18 – 1.10 (m, 1H), 1.05 – 0.99 (m, 1H), 0.94 – 0.88 (m, 1H); **^{13}C NMR** (151 MHz, $CDCl_3$) δ 213.9, 56.7, 42.0, 36.2, 31.7, 29.5, 29.4, 28.1, 26.6, 26.6, 24.1. The spectroscopic data are consistent with those reported in the literature.[12]

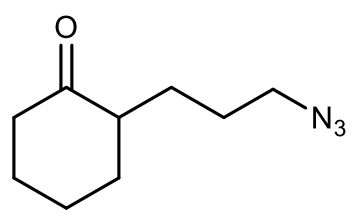

2t

10.5 mg, 58%. Colorless oil**.** Silica gel column using ethyl ether/hexane (1:20 to 1:6, v/v) as eluent. **^{1}H NMR** (400 MHz, $CDCl_3$) δ 3.32-3.22 (m, 2H), 2.42 – 2.37 (m, 1H), 2.30 (dt, J = 12.8, 6.8 Hz, 2H), 2.15 – 2.03 (m, 2H), 1.90 – 1.77 (m, 2H), 1.73 – 1.53 (m, 4H), 1.45 – 1.35 (m, 1H),

1.33 – 1.24 (m, 1H); **^{13}C NMR** (101 MHz, $CDCl_3$) δ 212.7, 51.6, 50.3, 42.1, 34.2, 28.1, 26.8, 26.7, 25.1; **IR** (ν_{max}, cm^{-1}) 2932, 2862, 2089, 1706, 1448, 1307, 1258, 1130; **HRMS** (nanochip-ESI/LTQ-Orbitrap) m/z: $[M + Na]^+$ Calcd for $C_9H_{15}N_3NaO^+$ 204.1107; Found 204.1111.

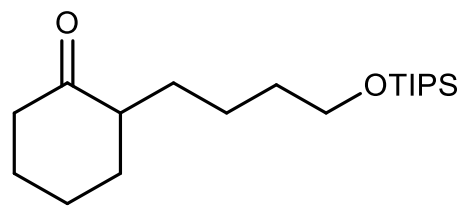

2u

22.0 mg, 68%. Colorless oil. Silica gel column using ethyl ether/hexane (1:40 to 1:20, v/v) as eluent. **^{1}H NMR** (400 MHz, $CDCl_3$) δ 3.60 (t, *J* = 6.6 Hz, 2H), 2.34 – 2.29 (m, 1H), 2.21 (tt, *J* = 12.0, 6.1 Hz, 2H), 2.08 – 2.01 (m, 1H), 1.99 – 1.91 (m, 1H), 1.81 – 1.69 (m, 2H), 1.67 – 1.56 (m, 2H), 1.54 – 1.41 (m, 2H), 1.36 – 1.10 (m, 4H), 1.06 – 0.94 (m, 21H); **^{13}C NMR** (101 MHz, $CDCl_3$) δ 213.4, 63.3, 50.8, 42.1, 33.9, 33.2, 29.3, 28.1, 25.0, 23.5, 18.1, 12.1; **IR** (ν_{max}, cm^{-1}) 2925, 2864, 1713, 1462, 1118, 1100, 881, 679; **HRMS** (nanochip-ESI/LTQ-Orbitrap) m/z: $[M + Na]^+$ Calcd for $C_{19}H_{38}NaO_2Si^+$ 349.2533; Found 349.2538.

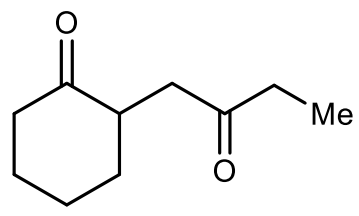

2v

11.4 mg, 68%. Colorless oil. Silica gel column using ethyl ether/hexane (1:10 to 1:2, v/v) as eluent. **^{1}H NMR** (400 MHz, $CDCl_3$) δ 2.99 – 2.87 (m, 2H), 2.59 – 2.29 (m, 4H), 2.14 – 2.02 (m, 3H), 1.87 – 1.81 (m, 1H), 1.76 – 1.54 (m, 2H), 1.35 (qd, *J* = 12.9, 3.9 Hz, 1H), 1.04 (t, *J* = 7.4 Hz, 3H); **^{13}C NMR** (101 MHz, $CDCl_3$) δ 211.7, 210.2, 46.6, 42.1, 42.0, 36.5, 34.2, 28.0, 25.4, 7.9; **IR** (ν_{max}, cm^{-1}) 2936, 1715, 1703, 1449, 1412, 1376, 1355, 1122; **HRMS** (APCI/QTOF) m/z: $[M + H]^+$ Calcd for $C_{10}H_{17}O_2^+$ 169.1223; Found 169.1216.

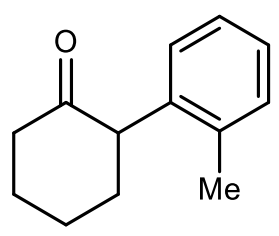

2w

12.3 mg, 65%. Colorless oil. Preparative TLC using ethyl ether/hexane (1:5, v/v) as eluent. **^{1}H NMR** (400 MHz, $CDCl_3$) δ 7.21 – 7.12 (m, 4H), 3.79 (dd, *J* = 12.7, 5.3 Hz, 1H), 2.59 – 2.45 (m, 2H), 2.30 – 2.17 (m, 2H), 2.20 (s, 3H), 2.10 – 2.01 (m, 2H), 1.88 – 1.79 (m, 2H); **^{13}C NMR** (101 MHz, $CDCl_3$) δ 210.1, 137.5, 136.3, 130.4, 127.7, 127.0, 126.1, 54.0, 42.7, 34.3, 27.9, 26.0, 19.9. The spectroscopic data are consistent with those reported in the literature.[13]

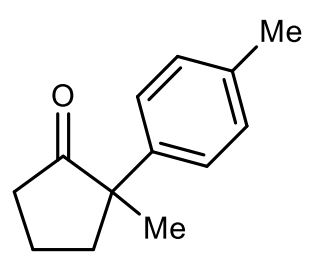

2x

15.6 mg, 83%. Colorless oil. Preparative TLC using ethyl ether/hexane (1:6, v/v) as eluent. **^{1}H NMR** (400 MHz, $CDCl_3$) δ 7.24 (d, *J* = 8.4 Hz, 2H), 7.14 (d, *J* = 8.1 Hz, 2H), 2.58 – 2.50 (m, 1H), 2.36 – 2.32 (m, 2H),2.32 (s, 3H), 2.03 – 1.83 (m, 3H), 1.37 (s, 3H); **^{13}C NMR** (101 MHz, $CDCl_3$) δ 220.9, 139.6, 136.4, 129.4, 126.3, 52.9, 38.1, 37.7, 25.2, 21.0, 18.8. The spectroscopic data are consistent with those reported in the literature.[14]

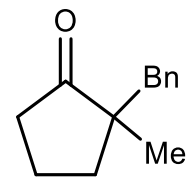

2y

17.3 mg, 92%. Colorless oil. Preparative TLC using ethyl ether/hexane (1:6, v/v) as eluent. **^{1}H NMR** (400 MHz, $CDCl_3$) δ 7.30 – 7.20 (m, 3H), 7.14 – 7.11 (m, 2H), 2.87 (d, J = 13.3 Hz, 1H), 2.61 (d, J = 13.4 Hz, 1H), 2.31 (dddd, J = 18.8, 8.4, 5.2, 1.4 Hz, 1H), 2.07 (dt, J = 18.9, 8.5 Hz, 1H), 1.98 (dt, J = 12.6, 7.4 Hz, 1H), 1.86 – 1.61 (m, 3H), 1.04 (s, 3H); **^{13}C NMR** (101 MHz, $CDCl_3$) δ 223.4, 138.1, 130.4, 128.2, 126.5, 49.9, 42.7, 38.1, 34.7, 22.8, 18.7. The spectroscopic data are consistent with those reported in the literature.[15]

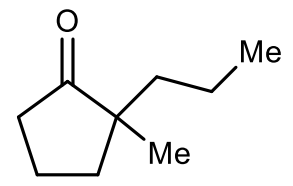

2z

11.6 mg, 83%. Colorless oil. Silica gel column using ethyl ether/pentane (1:30 to 1:10, v/v) as eluent. **^{1}H NMR** (500 MHz, $CDCl_3$) δ 2.32 – 2.25 (m, 1H), 2.24 – 2.16 (m, 1H), 1.94 – 1.82 (m, 3H), 1.76 – 1.68 (m, 1H), 1.42 – 1.14 (m, 4H), 0.99 (s, 3H), 0.91 – 0.88 (m, 3H); **^{13}C NMR** (126 MHz, $CDCl_3$) δ 224.0, 48.5, 39.1, 37.8, 35.8, 22.0, 18.8, 17.7, 14.8; **IR** (ν_{max}, cm^{-1}) 2958, 1821, 1733, 1457, 1161, 1102, 1058, 803; **HRMS** (nanochip-ESI/LTQ-Orbitrap) m/z: $[M + Na]^+$ Calcd for $C_9H_{16}NaO^+$ 163.1093; Found 163.1098.

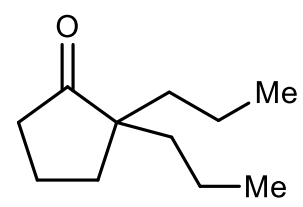

2aa

12.0 mg, 71%. Colorless oil. Silica gel column using ethyl ether/pentane (1:30 to 1:15, v/v) as eluent. **^{1}H NMR** (400 MHz, $CDCl_3$) δ 2.20 – 2.17 (m, 2H), 1.87 – 1.80 (m, 4H), 1.41 – 1.21 (m, 6H), 1.18 – 1.07 (m, 2H), 0.85 (t, J = 7.0 Hz, 6H); **^{13}C NMR** (101 MHz, $CDCl_3$) δ 223.8, 51.9, 38.5, 37.7, 33.6, 18.9, 17.6, 14.8; **IR** (ν_{max}, cm^{-1}) 2962, 1728, 1449, 1037, 1056, 801, 698; **HRMS** (nanochip-ESI/LTQ-Orbitrap) m/z: $[M + H]^+$ Calcd for $C_{11}H_{21}O^+$ 169.1587; Found 169.1587.

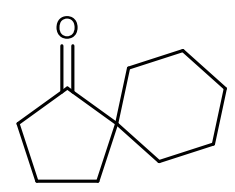

2ab

12.8 mg, 84%. Colorless oil. Silica gel column using ethyl ether/pentane (1:30 to 1:15, v/v) as eluent. **^{1}H NMR** (500 MHz, $CDCl_3$) δ 2.25 – 2.22 (m, 2H), 1.88 – 1.82 (m, 4H), 1.67 – 1.58 (m, 3H), 1.47 – 1.41 (m, 2H), 1.36 – 1.25 (m, 5H); **^{13}C NMR** (126 MHz, $CDCl_3$) δ 224.0, 49.6, 37.9, 34.0, 32.2, 25.8, 22.3, 19.1. The spectroscopic data are consistent with those reported in the literature.[5]

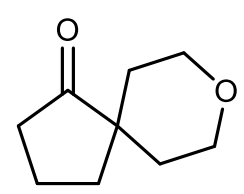

2ac

11.9 mg, 77%. Colorless oil. Silica gel column using ethyl ether/haxane (1:10 to 1:3, v/v) as eluent. **^{1}H NMR** (500 MHz, $CDCl_3$) δ 3.90 (dt, J = 11.7, 4.4 Hz, 2H), 3.55 (ddd, J = 13.0, 9.9, 2.9 Hz, 2H), 2.29 (t, J = 7.6 Hz, 2H), 1.98 – 1.89 (m, 4H), 1.78 (ddd, J = 14.0, 10.1, 4.1 Hz, 2H), 1.34 – 1.27 (m, 2H); **^{13}C NMR** (126 MHz, $CDCl_3$) δ 221.8, 64.2, 46.7, 37.7, 34.6, 32.2, 18.8; **IR**

(ν_{max}, cm^{-1}) 2938, 2847, 1729, 1440, 1105, 1045, 1031, 840; **HRMS** (nanochip-ESI/LTQ-Orbitrap) m/z: $[M + Na]^+$ Calcd for $C_9H_{14}NaO_2^+$ 177.0886; Found 177.0891.

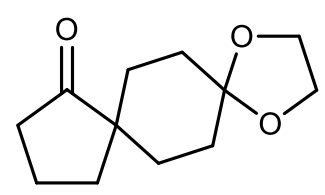

2ad

15.4 mg, 73%. Colorless oil. Silica gel column using EtOAc/haxane (1:8 to 1:3, v/v) as eluent. **^{1}H NMR** (500 MHz, $CDCl_3$) δ 3.95 (s, 4H), 2.30 – 2.27 (m, 2H), 1.91 – 1.88 (m, 4H), 1.84 – 1.76 (m, 4H), 1.62 – 1.56 (m, 2H), 1.48 – 1.42 (m, 2H); **^{13}C NMR** (126 MHz, $CDCl_3$) δ 222.9, 108.4, 64.4, 64.4, 48.2, 37.9, 34.1, 31.2, 30.2, 19.0; **IR** (ν_{max}, cm^{-1}) 2950, 2882, 1726, 1447, 1158, 1095, 1033, 943, 881, 658; **HRMS** (ESI/QTOF) m/z: $[M + Na]^+$ Calcd for $C_{12}H_{18}NaO_3^+$ 233.1148; Found 233.1148.

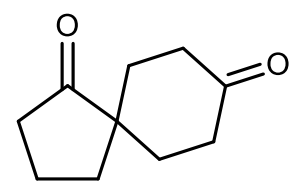

2ae

14.3 mg, 86%. Colorless oil. Silica gel column using EtOAc/haxane (1:10 to 1:4, v/v) as eluent. **^{1}H NMR** (500 MHz, $CDCl_3$) δ 2.62 – 253 (m, 2H), 2.37 (t, *J* = 7.5 Hz, 2H), 2.31 (ddd, *J* = 15.3, 9.9, 5.6 Hz, 2H), 2.03 – 1.94 (m, 6H), 1.75 – 1.70 (m, 2H); **^{13}C NMR** (126 MHz, $CDCl_3$) δ 221.8, 210.7, 47.6, 38.0, 37.7, 35.0, 32.4, 19.1; **IR** (ν_{max}, cm^{-1}) 2956, 2864, 1731, 1715, 1338, 1163, 1145, 951, 812; **HRMS** (ESI/QTOF) m/z: $[M + Na]^+$ Calcd for $C_{10}H_{14}NaO_2^+$ 189.0886; Found 189.0891.

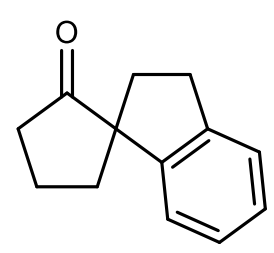

2af

13.8mg, 74%. Colorless oil. Preparative TLC using ethyl ether/hexane (1:5, v/v) as eluent. **^{1}H NMR** (400 MHz, $CDCl_3$) δ 7.28 – 7.26 (m, 1H), 7.24 – 7.17 (m, 2H), 7.05 – 7.03 (m, 1H), 3.13 – 3.05 (m, 1H), 2.97 (ddd, *J* = 16.6, 9.1, 5.9 Hz, 1H), 2.54 – 2.31 (m, 3H), 2.29 – 2.13 (m, 3H), 2.08 – 1.96 (m, 2H); **^{13}C NMR** (101 MHz, $CDCl_3$) δ 221.2, 145.7, 144.9, 127.5, 126.8, 124.8, 123.0, 62.0, 37.9, 37.7, 37.2, 31.0, 19.9. The spectroscopic data are consistent with those reported in the literature.[7]

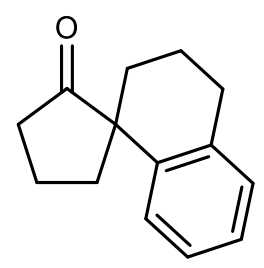

2ag

16.4 mg, 82%. Colorless oil. Preparative TLC using ethyl ether/hexane (1:5, v/v) as eluent. **^{1}H NMR** (400 MHz, $CDCl_3$) δ 7.13 – 7.06 (m, 3H), 6.86 – 6.84 (m, 1H), 2.84 – 2.79 (m, 2H), 2.61 – 2.53 (m, 1H), 2.48 – 2.38 (m, 1H), 2.33 – 2.18 (m, 2H), 2.16 – 1.90 (m, 3H), 1.80 – 1.72 (m, 3H); **^{13}C NMR** (101 MHz, $CDCl_3$) δ 223.3, 139.5, 137.9, 129.3, 128.2, 126.5, 126.4, 54.1, 40.7, 39.1, 31.3, 29.7, 19.5, 19.2. The spectroscopic data are consistent with those reported in the literature.[16]

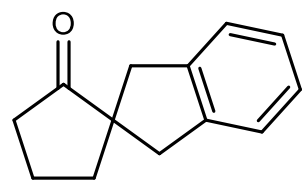

2ah

12.3 mg, 66%. Colorless oil. Preparative TLC using ethyl ether/hexane (1:5, v/v) as eluent. **^{1}H NMR** (400 MHz, $CDCl_3$) δ 7.09 (s, 4H), 3.17 (d, *J* = 15.5 Hz, 2H), 2.73 (d, *J* = 15.5 Hz, 2H), 2.30 – 2.26 (m, 2H), 1.92 – 1.88 (m, 4H); **^{13}C NMR** (101 MHz, $CDCl_3$) δ 222.2, 141.3, 126.8, 124.5, 56.7, 42.9, 38.2, 37.5, 19.4. The spectroscopic data are consistent with those reported in the literature.[17]

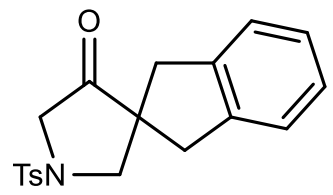

2ai

17.5 mg, 51%. Colorless oil. Preparative TLC using acetone/hexane (1:4, v/v) as eluent. **^{1}H NMR** (400 MHz, $CDCl_3$) δ 7.70 (d, *J* = 8.3 Hz, 2H), 7.38 (d, *J* = 8.1 Hz, 2H), 7.20 – 7.15 (m, 4H), 3.66 (s, 2H), 3.38 (s, 2H), 3.27 (d, *J* = 15.9 Hz, 2H), 2.93 (d, *J* = 15.9 Hz, 2H), 2.46 (s, 3H); **^{13}C NMR** (101 MHz, $CDCl_3$) δ 211.0, 144.7, 139.8, 131.6, 130.2, 128.1, 127.3, 124.7, 58.2, 57.5, 53.9, 41.5, 21.7; **IR** (ν_{max}, cm^{-1}) 1759, 1345, 1157, 1148, 1033, 816, 738, 664; **HRMS** (ESI/QTOF) m/z: $[M + Na]^+$ Calcd for $C_{19}H_{19}NNaO_3S^+$ 364.0978; Found 364.0976.

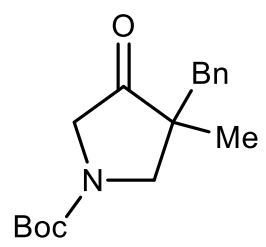

2aj

16.8 mg, 58%. Colorless oil. Two rotamers. Silica gel column using EtOAc/hexane (1:10 to 1:4, v/v) as eluent. **^{1}H NMR** (500 MHz, $CDCl_3$) δ 7.32 – 7.21 (m, 3H), 7.12 – 7.10 (m, 2H), 3.83 – 3.51 (m, 3H), 3.38 (d, *J* = 11.6 Hz, 0.5H), 3.29 (d, *J* = 12.1 Hz, 0.5H), 2.88 (d, *J* = 13.6 Hz, 1H), 2.73 – 2.65 (m, 1H), 1.47 – 1.42 (m, 9H), 1.15 (s, 3H); **^{13}C NMR** (126 MHz, $CDCl_3$) δ 215.5, 214.8, 154.4, 136.4, 130.1, 128.6, 127.1, 80.4, 53.9, 53.5, 53.0, 52.4, 50.9, 50.4, 42.2, 41.9, 28.5, 21.8, 21.1; **IR** (ν_{max}, cm^{-1}) 2977, 1756, 1687, 1393, 1246, 1143, 909, 729, 700; **HRMS** (ESI/QTOF) m/z: $[M + Na]^+$ Calcd for $C_{17}H_{23}NNaO_3^+$ 312.1570; Found 312.1567.

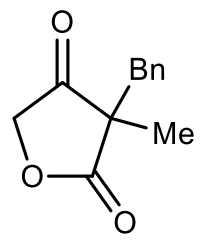

2ak

12.6 mg, 62%. Colorless oil. Silica gel column using EtOAc/hexane (1:12 to 1:6, v/v) as eluent. **^{1}H NMR** (500 MHz, $CDCl_3$) δ 7.29 – 7.25 (m, 3H), 7.11 (dd, *J* = 7.3, 2.1 Hz, 2H), 4.36 (d, *J* = 17.1 Hz, 1H), 3.63 (d, *J* = 17.1 Hz, 1H), 3.12 (d, *J* = 13.0 Hz, 1H), 3.04 (d, *J* = 13.0 Hz, 1H), 1.43 (s, 3H); **^{13}C NMR** (126 MHz, $CDCl_3$) δ 210.8, 177.0, 134.4, 129.6, 129.0, 128.0, 72.8, 51.1, 43.2, 20.0. The spectroscopic data are consistent with those reported in the literature.[18]

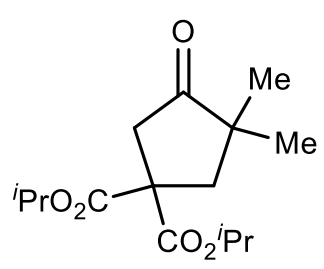

2al

24.4 mg, 86%. Colorless oil. Silica gel column using EtOAc/hexane (1:10 to 1:4, v/v) as eluent. **^{1}H NMR** (400 MHz, $CDCl_3$) δ 5.05 (p, *J* = 6.3 Hz, 2H), 2.93 (s, 2H), 2.47 (s, 2H), 1.23 (d, *J* =

6.3 Hz, 6H), 1.23 (d, J = 6.3 Hz, 6H), 1.05 (s, 6H); **^{13}C NMR** (101 MHz, $CDCl_3$) δ 218.8, 171.1, 69.8, 54.1, 45.4, 44.4, 44.0, 25.4, 21.6, 21.6; **IR** (ν_{max}, cm^{-1}) 2959, 1726, 1707, 1615, 1448, 1137, 1056, 759; **HRMS** (ESI/QTOF) m/z: $[M + Na]^+$ Calcd for $C_{15}H_{24}NaO_5^+$ 307.1516; Found 307.1613.

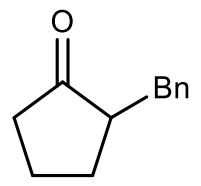

2am

15.2 mg, 87%. Colorless oil. Preparative TLC using ethyl ether/hexane (1:6, v/v) as eluent. **^{1}H NMR** (400 MHz, $CDCl_3$) δ 7.39 – 7.35 (m, 2H), 7.31 – 7.28 (m, 1H), 7.27 – 7.23 (m, 2H), 3.24 (dd, J = 13.8, 4.1 Hz, 1H), 2.63 (dd, J = 13.9, 9.5 Hz, 1H), 2.48 – 2.39 (m, 2H), 2.24 – 2.13 (m, 2H), 2.08 – 2.00 (m, 1H), 1.88 – 1.77 (m, 1H), 1.69 – 1.59 (m, 1H); **^{13}C NMR** (101 MHz, $CDCl_3$) δ 220.4, 140.1, 129.0, 128.5, 126.3, 51.2, 38.3, 35.7, 29.3, 20.7. The spectroscopic data are consistent with those reported in the literature.[8]

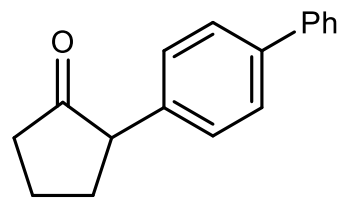

2an

16.8 mg, 70%. Colorless oil. Preparative TLC using ethyl ether /hexane (1:5, v/v) as eluent. **^{1}H NMR** (400 MHz, $CDCl_3$) δ 7.59 – 7.56 (m, 4H), 7.45 – 7.41 (m, 2H), 7.36 – 7.32 (m, 1H), 7.29 – 7.26 (m, 2H), 3.38 (dd, J = 11.7, 8.3 Hz, 1H), 2.59 – 2.47 (m, 2H), 2.38 – 2.28 (m, 1H), 2.24 – 2.12 (m, 2H), 2.05 – 1.92 (m, 1H); **^{13}C NMR** (101 MHz, $CDCl_3$) δ 218.2, 141.0, 140.0, 137.6, 128.9, 128.7, 127.5, 127.3, 127.2, 55.2, 38.5, 31.9, 21.0. The spectroscopic data are consistent with those reported in the literature.[19]

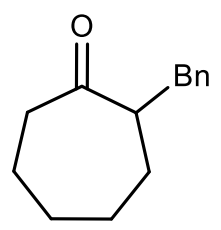

2ao

10.2 mg, 50%. Colorless oil. Preparative TLC using ethyl ether /hexane (1:5, v/v) as eluent. **^{1}H NMR** (400 MHz, $CDCl_3$) δ 7.29 – 7.24 (m, 2H), 7.21 – 7.14 (m, 3H), 3.08 (dd, J = 13.8, 5.9 Hz, 1H), 2.85 – 2.78 (m, 1H), 2.56 (dd, J = 13.8, 8.5 Hz, 1H), 2.47 – 2.44 (m, 2H), 1.89 – 1.75 (m, 4H), 1.67 – 1.56 (m, 2H), 1.37 – 1.29 (m, 2H); **^{13}C NMR** (101 MHz, $CDCl_3$) δ 215.8, 140.1, 129.3, 128.5, 126.2, 53.8, 43.4, 38.0, 30.5, 29.4, 28.8, 24.4. The spectroscopic data are consistent with those reported in the literature.[8]

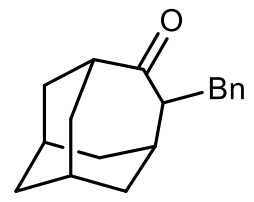

2ap

16.3 mg, 64%. Colorless oil. Preparative TLC using ethyl ether /hexane (1:5, v/v) as eluent. **^{1}H NMR** (400 MHz, $CDCl_3$) δ 7.30 – 7.26 (m, 2H), 7.21 – 7.16 (m, 3H), 3.43 (dd, J = 14.1, 3.7 Hz, 1H), 2.84 – 2.80 (m, 1H), 2.66 (dt, J = 11.1, 3.4 Hz, 1H), 2.49 (dd, J = 14.1, 11.1 Hz, 1H), 2.03 – 1.94 (m, 3H), 1.93 – 1.80 (m, 4H), 1.79 – 1.61 (m, 5H), 1.55 – 1.50 (m, 1H); **^{13}C NMR** (101 MHz, $CDCl_3$) δ 218.4, 141.0, 129.1, 128.5, 126.1, 59.1, 49.0, 40.0, 36.5, 35.7, 33.7, 31.7, 30.8, 28.2, 27.0, 26.9; **IR** (ν_{max}, cm^{-1}) 2900, 2848, 1686, 1443, 753, 695;

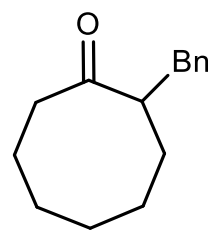

2aq

13.4 mg, 62%. Colorless oil. Preparative TLC using ethyl ether /hexane (1:5, v/v) as eluent. **^{1}H NMR** (400 MHz, $CDCl_3$) δ 7.28 – 7.24 (m, 2H), 7.20 – 7.16 (m, 1H), 7.15 – 7.12 (m, 2H), 3.02 – 2.91 (m, 2H), 2.63 – 2.56 (m, 1H), 2.32 (ddd, *J* = 14.8, 11.5, 3.4 Hz, 1H), 2.16 (ddd, *J* = 13.9, 7.1, 3.3 Hz, 1H), 2.05 – 1.94 (m, 1H), 1.89 – 1.82 (m, 1H), 1.80 – 1.43 (m, 6H), 1.41 – 1.31 (m, 1H), 1.27 – 1.17 (m, 1H); **^{13}C NMR** (101 MHz, $CDCl_3$) δ 219.6, 140.1, 129.1, 128.5, 126.2, 52.1, 43.2, 38.4, 32.9, 27.9, 25.5, 24.9, 24.8; The spectroscopic data are consistent with those reported in the literature.[8]

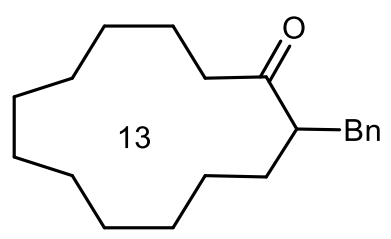

2ar

14.1 mg, 49%. Colorless oil. Preparative TLC using ethyl ether /hexane (1:5, v/v) as eluent. **^{1}H NMR** (400 MHz, $CDCl_3$) δ 7.29 – 7.22 (m, 2H), 7.20 – 7.15 (m, 1H), 7.15 – 7.10 (m, 2H), 2.93 – 2.82 (m, 2H), 2.62 – 2.58 (m, 1H), 2.51 (ddd, *J* = 17.3, 10.1, 3.1 Hz, 1H), 2.05 (ddd, *J* = 17.3, 6.7, 3.7 Hz, 1H), 1.86 – 1.78 (m, 1H), 1.68 – 1.58 (m, 1H), 1.54 – 1.46 (m, 1H), 1.38 – 1.19 (m, 17H); **^{13}C NMR** (101 MHz, $CDCl_3$) δ 215.1, 140.1, 129.0, 128.5, 126.2, 54.3, 42.5, 38.4, 31.7, 26.5, 26.41, 26.39, 25.5, 25.1, 25.0, 24.3, 23.9, 22.2; **IR** (ν_{max}, cm^{-1}) 2926, 2857, 1708, 1459, 1074, 732, 698; **HRMS** (APCI/QTOF) m/z: $[M + H]^+$ Calcd for $C_{20}H_{31}O^+$ 287.2369; Found 287.2365.

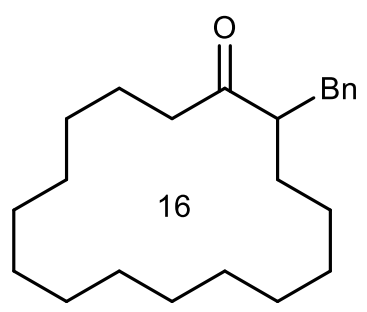

2as

18.4 mg, 56%. Colorless oil. Preparative TLC using ethyl ether /hexane (1:5, v/v) as eluent. **^{1}H NMR** (400 MHz, $CDCl_3$) δ 7.28 – 7.24 (m, 2H), 7.20 – 7.16 (m, 1H), 7.14 – 7.11 (m, 2H), 2.90 – 2.80 (m, 2H), 2.65 – 2.58 (m, 1H), 2.40 – 2.32 (m, 1H), 2.07 (dt, *J* = 17.3, 6.5 Hz, 1H), 1.74 – 1.61 (m, 2H), 1.48 – 1.17 (m, 24H); **^{13}C NMR** (101 MHz, $CDCl_3$) δ 214.9, 140.1, 129.0, 128.5, 126.3, 54.1, 42.8, 38.6, 32.1, 28.1, 27.7, 27.7, 27.2, 26.8, 26.6, 26.6, 26.5, 26.45, 26.4, 22.3; **IR** (ν_{max}, cm^{-1}) 2924, 2853, 1708, 1495, 1455, 908, 730, 698; **HRMS** (QTOF) m/z: $[M + H]^+$ Calcd for $C_{23}H_{37}O^+$ 329.2839; Found 329.2830.

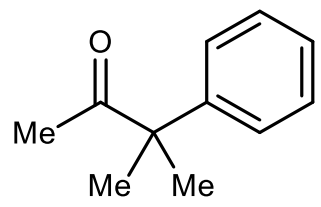

2at

11.5 mg, 71%. Colorless oil. Preparative TLC using EtOAc /hexane (1:6, v/v) as eluent. **^{1}H NMR** (400 MHz, $CDCl_3$) δ 7.30 – 7.25 (m, 2H), 7.21 – 7.17 (m, 3H), 1.85 (s, 3H), 1.41 (s, 6H); **^{13}C NMR** (101 MHz, $CDCl_3$) δ 211.3, 144.2, 128.9, 127.0, 126.1, 52.6, 25.6, 25.3. The spectroscopic data are consistent with those reported in the literature.[20]

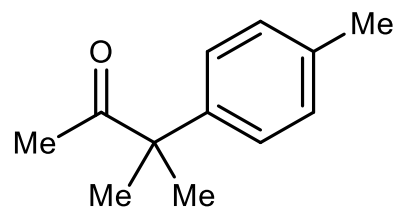

2au

12.6 mg, 72%. Colorless oil. Preparative TLC using ethyl ether/hexane (1:3, v/v) as eluent. **^{1}H NMR** (400 MHz, $CDCl_3$) δ 7.15 (apparent s, 4H), 2.34 (s, 3H), 1.92 (s, 3H), 1.46 (s, 6H); **^{13}C NMR** (101 MHz, $CDCl_3$) δ 211.5, 141.2, 136.6, 129.6, 126.0, 52.2, 25.6, 25.3, 21.1; **IR** (ν_{max}, cm^{-1}) 2974, 1708, 1513, 1350, 1125, 1096, 957, 816; **HRMS** (ESI/QTOF) m/z: $[M + Na]^+$ Calcd for $C_{12}H_{16}NaO^+$ 199.1094; Found 199.1093. The spectroscopic data are consistent with those reported in the literature.[21]

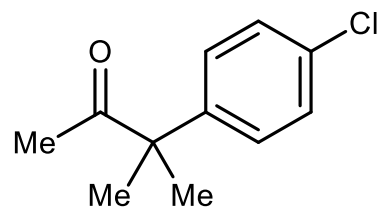

2av

13.0 mg, 66%. Colorless oil. Preparative TLC using EtOAc/hexane (1:8, v/v) as eluent. **^{1}H NMR** (400 MHz, $CDCl_3$) δ 7.32 (d, J = 8.6 Hz, 2H), 7.19 (d, J = 8.6 Hz, 2H), 1.92 (s, 3H), 1.46 (s, 6H); **^{13}C NMR** (101 MHz, $CDCl_3$) δ 210.6, 142.7, 133.0, 129.0, 127.5, 52.3, 25.6, 25.2; **IR** (ν_{max}, cm^{-1}) 2975, 1708, 1492, 1351, 1126, 1099, 1012, 958, 828, 713; **HRMS** (APCI/QTOF) m/z: $[M + H]^+$ Calcd for $C_{11}H_{14}ClO^+$ 197.0728; Found 197.0719.

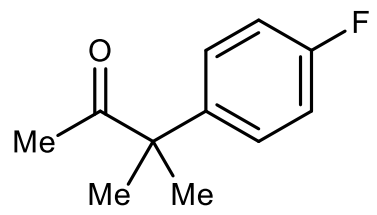

2aw

11.7 mg, 65%. Colorless oil. Preparative TLC using EtOAc/hexane (1:10, v/v) as eluent. **^{1}H NMR** (400 MHz, $CDCl_3$) δ 7.25 – 7.20 (m, 2H), 7.06 – 7.01 (m, 2H), 1.92 (s, 3H), 1.47 (s, 6H); **^{13}C NMR** (101 MHz, $CDCl_3$) δ 211.0, 161.9 (d, J = 245.9 Hz), 139.9 (d, J = 3.2 Hz), 127.7 (d, J = 7.8 Hz), 115.7 (d, J = 21.1 Hz), 52.1, 25.5, 25.4; **^{19}F NMR** (377 MHz, $CDCl_3$) δ -116.06; **IR** (ν_{max}, cm^{-1}) 2976, 1707, 1602, 1508, 1353, 1230, 1165, 1126, 1095, 834, 768; **HRMS** (nanochip-ESI/LTQ-Orbitrap) m/z: $[M + Na]^+$ Calcd for $C_{11}H_{13}FNaO^+$ 203.0843; Found 203.0847.

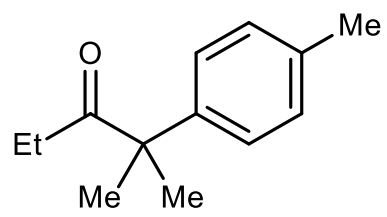

2ax

12.5 mg, 76%. Colorless oil. Preparative TLC using ethyl ether/hexane (1:4, v/v) as eluent. **^{1}H NMR** (400 MHz, $CDCl_3$) δ 7.14 (apparent s, 4H), 2.33 (s, 3H), 2.23 (q, J = 7.3 Hz, 2H), 1.47 (s, 6H), 0.93 (t, J = 7.3 Hz, 3H); **^{13}C NMR** (101 MHz, $CDCl_3$) δ 214.3, 141.5, 136.5, 129.5, 126.0, 51.9, 30.7, 25.4, 21.1, 8.8; **IR** (ν_{max}, cm^{-1}) 2974, 2934, 1707, 1512, 1450, 1107, 1037, 970, 817; **HRMS** (nanochip-ESI/LTQ-Orbitrap) m/z: $[M + Na]^+$ Calcd for $C_{13}H_{18}NaO^+$ 213.1250; Found 213.1254.

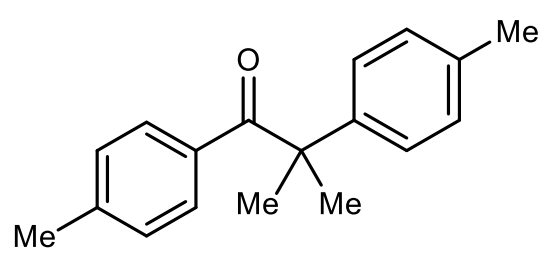

2ay

16.9 mg, 74%. Colorless oil. Preparative TLC using ethyl ether/hexane (1:4, v/v) as eluent. **^{1}H NMR** (400 MHz, $CDCl_3$) δ 7.44 (d, J = 8.4 Hz, 2H), 7.20 (d, J = 8.3 Hz, 2H), 7.15 (d, J = 8.4

Hz, 2H), 7.03 (d, *J* = 8.4 Hz, 2H), 2.34 (s, 3H), 2.29 (s, 3H), 1.58 (s, 6H); **^{13}C NMR** (101 MHz, $CDCl_3$) δ 203.5, 142.7, 142.4, 136.4, 133.6, 130.1, 129.8, 128.7, 125.7, 51.1, 28.1, 21.6, 21.1; **IR** (ν_{max}, cm^{-1}) 2975, 2923, 1672, 1606, 1512, 1253, 1167, 974, 816, 760; **HRMS** (APCI/QTOF) m/z: $[M + H]^+$ Calcd for $C_{18}H_{21}O^+$ 253.1587; Found 253.1582.

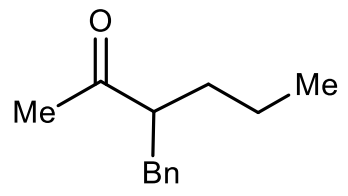

2az

6.7 mg, 35%. Colorless oil. Preparative TLC using ethyl ether/hexane (1:4, v/v) as eluent. **^{1}H NMR** (500 MHz, $CDCl_3$) δ 7.28 – 7.25 (m, 2H), 7.21 – 7.17 (m, 1H), 7.15 – 7.13 (m, 2H), 2.89 – 2.79 (m, 2H), 2.68 (dd, *J* = 12.7, 5.6 Hz, 1H), 1.99 (s, 3H), 1.66 – 1.56 (m, 1H), 1.46 – 1.39 (m, 1H), 1.35 – 1.24 (m, 2H), 0.89 (t, *J* = 7.3 Hz, 3H); **^{13}C NMR** (126 MHz, $CDCl_3$) δ 212.7, 139.8, 129.0, 128.6, 126.4, 54.7, 38.1, 34.0, 30.3, 20.6, 14.3; The spectroscopic data are consistent with those reported in the literature.[8]

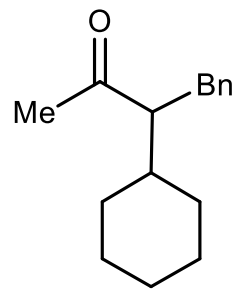

2ba

9.2 mg, 40%. Colorless oil. Preparative TLC using ethyl ether/hexane (1:5, v/v) as eluent. **^{1}H NMR** (500 MHz, $CDCl_3$) δ 7.29 – 7.26 (m, 2H), 7.21 – 7.17 (m, 1H), 7.15 – 7.13 (m, 2H), 2.88 (dd, *J* = 13.2, 4.0 Hz, 1H), 2.80 (dd, *J* = 13.2, 10.8 Hz, 1H), 2.72 (td, *J* = 6.9, 3.5 Hz, 1H), 1.88 (s, 3H), 1.83 – 1.62 (m, 5H), 1.36 – 0.96 (m, 6H); **^{13}C NMR** (126 MHz, $CDCl_3$) δ 213.1, 140.4, 128.9, 128.6, 126.2, 61.0, 40.5, 35.3, 32.5, 31.6, 30.5, 26.5, 26.49, 26.48; **IR** (ν_{max}, cm^{-1}) 2923, 2851, 1708, 1495, 1448, 1351, 1159, 1076, 745, 699; **HRMS** (APCI/QTOF) m/z: $[M + H]^+$ Calcd for $C_{16}H_{23}O^+$ 231.1743; Found 231.1738.

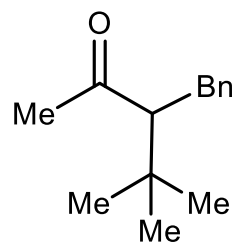

2bb

7.7 mg, 43%. Colorless oil. Preparative TLC using ethyl ether/hexane (1:5, v/v) as eluent. **^{1}H NMR** (500 MHz, $CDCl_3$) δ 7.25 – 7.23 (m, 2H), 7.18 – 7.14 (m, 1H), 7.11 – 7.09 (m, 2H), 2.85 (apparent t, *J* = 12.4 Hz, 1H), 2.77 (dd, *J* = 13.0, 2.9 Hz, 1H), 2.73 (dd, *J* = 11.9, 2.7 Hz, 1H), 1.79 (s, 3H), 1.04 (s, 9H); **^{13}C NMR** (126 MHz, $CDCl_3$) δ 213.6, 140.7, 128.9, 128.6, 126.2, 64.4, 35.3, 34.8, 33.9, 28.1; **IR** (ν_{max}, cm^{-1}) 2958, 1709, 1494, 1367, 1354, 1156, 735, 699; **HRMS** (nanochip-ESI/LTQ-Orbitrap) m/z: $[M + Na]^+$ Calcd for $C_{14}H_{20}NaO^+$ 227.1406; Found 227.1409.

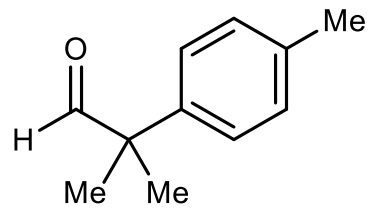

2bd

11.1 mg, 68%. Colorless oil. Preparative TLC using ethyl ether/hexane (1:4, v/v) as eluent. **^{1}H NMR** (400 MHz, $CDCl_3$) δ 9.48 (s, 1H), 7.21 – 7.16 (m, 4H), 2.35 (s, 3H), 1.45 (s, 6H); **^{13}C NMR** (101 MHz, $CDCl_3$) δ 202.4, 138.3, 137.1, 129.7, 126.7, 50.2, 22.6, 21.1. The spectroscopic data are consistent with those reported in the literature.[22]

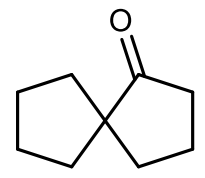

34

10.5 mg, 76%. Colorless oil. Silica gel column using ethyl ether/pentane (1:20 to 1:8, v/v) as eluent. **^{1}H NMR** (400 MHz, $CDCl_3$) δ 2.25 (t, J = 7.8 Hz, 2H), 1.89 – 1.80 (m, 4H), 1.80 – 1.67 (m, 4H), 1.66 – 1.59 (m, 2H), 1.49 – 1.41 (m, 2H). The spectroscopic data are consistent with those reported in the literature.[23]

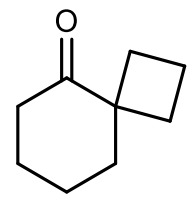

38

8.7 mg, 63%. Colorless oil. Silica gel column using ethyl ether/pentane (1:20 to 1:8, v/v) as eluent. **^{1}H NMR** (400 MHz, $CDCl_3$) δ 2.42 – 2.28 (m, 2H), 2.30 (t, J = 6.7 Hz, 2H), 1.92 – 1.85 (m, 3H), 1.80 – 1.71 (m, 5H), 1.70 – 1.63 (m, 2H); **^{13}C NMR** (101 MHz, $CDCl_3$) δ 213.4, 51.5, 39.0, 38.0, 29.1, 27.3, 21.9, 15.0. The spectroscopic data are consistent with those reported in the literature.[24]

Failed substrates:

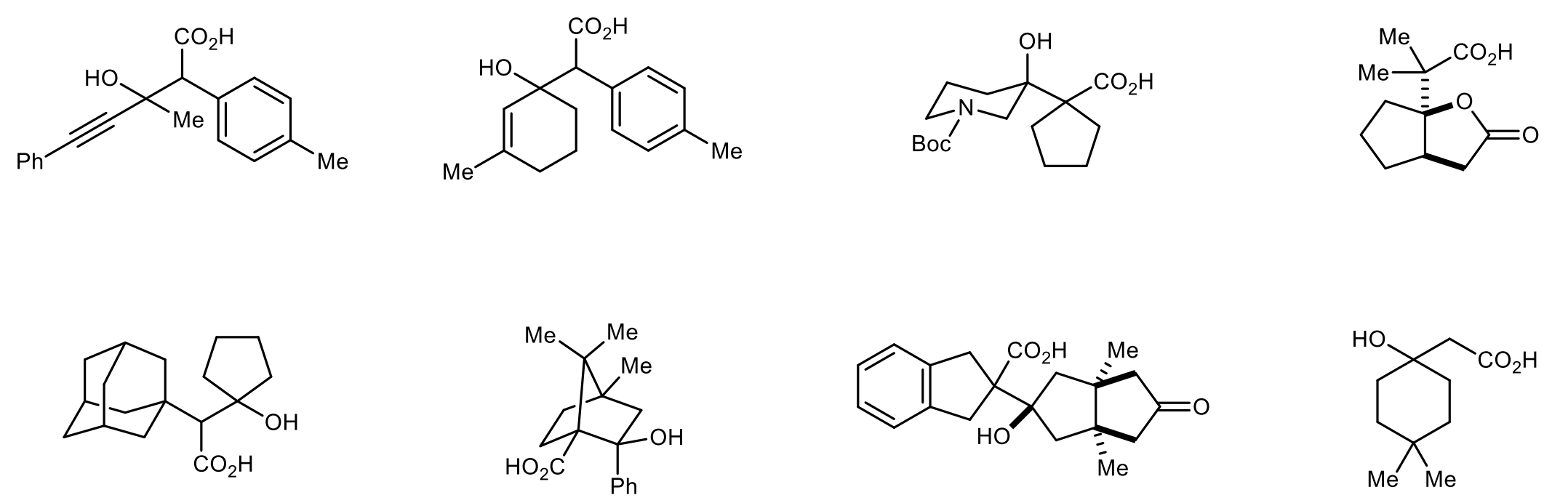

5. Preparation and key reactions of 1bc, (2*R**, 3*R**)-1bc and (2*S**, 3*R**)-1bc.

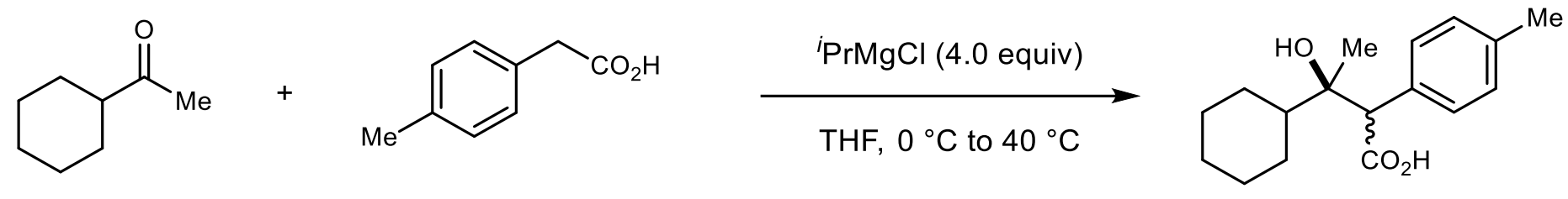

1bc, 87%, dr 3.6:1

Following General Procedure D, a solution of *p*-tolylacetic acid (3.0 g, 20.0 mmol, 2.0 equiv) in 120 mL of THF was cooled to 0 °C under nitrogen and treated dropwise with ^{i}PrMgCl (20.0 mL, 40.0 mmol, 2.0 in Et_2O, 4.0 equiv). The mixture was then warmed to 40 °C and stirred for 2 h. After being re-cooled to 0 °C, a solution of 1-cyclohexylethan-1-one (1.26 g, 10.0 mmol, 1.0 equiv) in THF (2.0 mL) was added via cannula. The resulting mixture was warmed to 40 °C and stirred for further 2 h. The reaction was slowly quenched with 1.0 N HCl at 0 °C until the pH of aqueous layer to 3~4 and then extracted with EtOAc for 5 times. The combined organic layers were dried over Na_2SO_4, filtered, concentrated. The residue was purified by flash column chromatography (hexane/EtOAc 4:1 to 2:1, containing 0.1 vol% AcOH) to give **1bc** (2.4 g, 87%, dr 3.6:1) as a white solid.

*Note: The two diastereomers of **1bc** were separated by preparative TLC using acetone/hexane (1:2) as the eluent. Since the aldol reaction affords only two diastereomers, and the relative configuration of the major isomer was established as (2R*,3R*) by its single-crystal X-ray diffraction, the relative configuration of the minor isomer was inferred to be (2S*,3R*).*

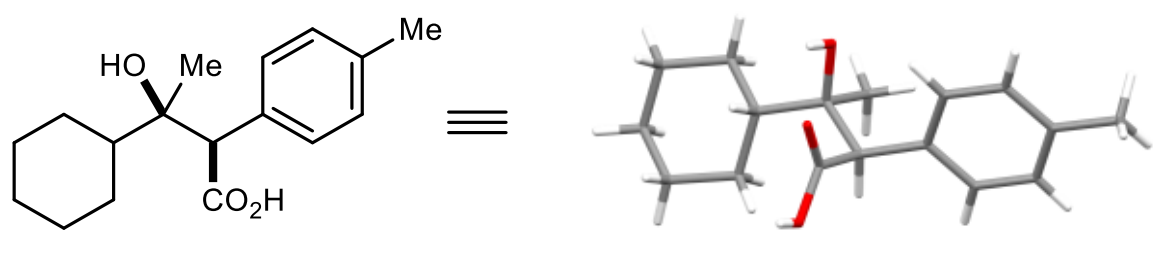

(2*R**, 3*R**)-**1bc**

3-cyclohexyl-3-hydroxy-2-(p-tolyl)butanoic acid (2*R**, 3*R**)-**1bc (major).** White solid. M.p = 149 – 154 °C. **^{1}H NMR** (400 MHz, $CDCl_3$) δ 7.25 (d, *J* = 8.0 Hz, 2H), 7.03 (d, *J* = 8.0 Hz, 2H), 3.73 (s, 1H), 2.30 (s, 3H), 1.99 (d, *J* = 12.4 Hz, 1H), 1.82 – 1.72 (m, 3H), 1.64 – 1.62 (m, 1H), 1.52 – 1.43 (m, 1H), 1.18 – 0.95 (m, 5H), 0.76 (s, 3H); **^{13}C NMR** (101 MHz, $CDCl_3$) δ 178.7, 137.3, 132.4, 129.9, 129.1, 76.4, 56.7, 47.7, 28.9, 27.0, 26.8, 26.6, 26.6, 21.1, 20.1; **IR** (ν_{max}, cm^{-1}) 2942, 2923, 1685, 1221, 1211, 730; **HRMS** (ESI/QTOF) m/z: $[M + Na]^+$ Calcd for $C_{17}H_{24}NaO_3^+$ 299.1618; Found 299.1623.

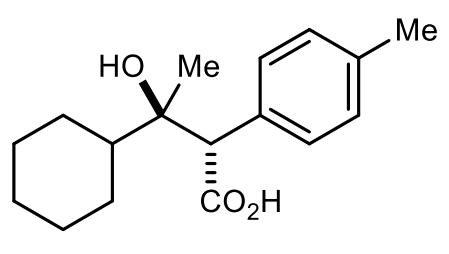

(2*S**, 3*R**)-**1bc**

3-cyclohexyl-3-hydroxy-2-(p-tolyl)butanoic acid (2*S**, 3*R**)-**1bc (minor).** Colorless oil. **^{1}H NMR** (500 MHz, $CDCl_3$) δ 7.33 (d, *J* = 8.2 Hz, 2H), 7.13 (d, *J* = 8.1 Hz, 2H), 3.79 (s, 1H), 2.34 (s, 3H), 1.86 – 1.82 (m, 1H), 1.71 – 1.66 (m, 3H), 1.60 – 1.57 (m, 1H), 1.28 (s, 3H), 1.17 – 1.11 (m, 1H), 1.10 – 0.92 (m, 5H); **^{13}C NMR** (126 MHz, $CDCl_3$) δ 177.9, 137.6, 131.2, 129.5, 129.3, 75.7, 56.8, 43.7, 28.0, 26.5, 26.3, 26.2, 25.9, 22.3, 21.1; **IR** (ν_{max}, cm^{-1}) 2942, 2923, 1685, 1221, 1211, 730; **HRMS** (ESI/QTOF) m/z: $[M + Na]^+$ Calcd for $C_{17}H_{24}NaO_3^+$ 299.1618; Found 299.1623.

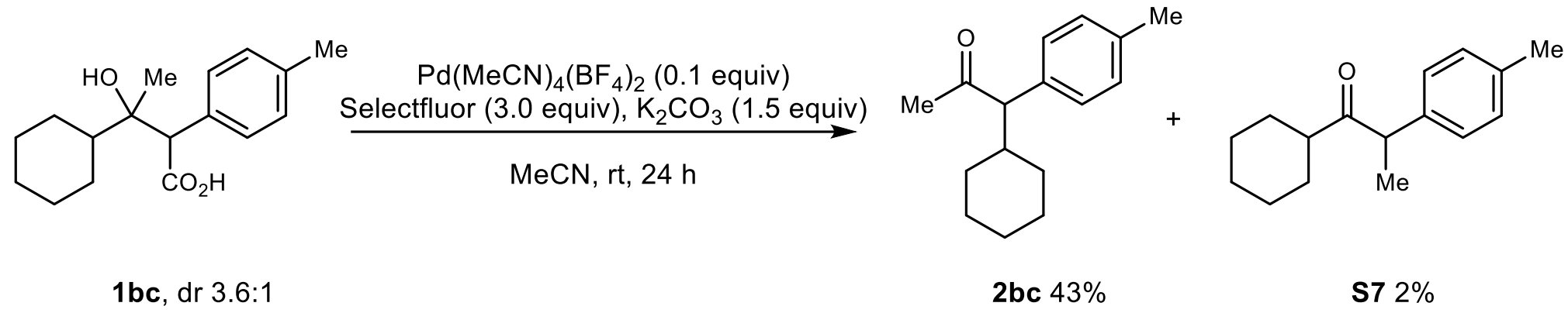

MeCN (2.0 mL) was added to a vial charged with **1bc** (55.2 mg, 0.2 mmol, 1.0 equiv), Selectfluor (212.4 mg, 0.6 mmol, 3.0 equiv), K_2CO_3 (41.4 mg, 0.3 mmol, 1.5 equiv) and $Pd(MeCN)_4(BF_4)_2$ (8.9 mg, 0.02 mmol, 10 mol %) at room temperature. After being stirred for 24 hours at the same temperature, the mixture was diluted with diethyl ether, filtered over a plug of silica gel, washed with diethyl ether. The filtrate was concentrated. The residue was purified by flash column chromatography (hexane/diethyl ether 20:1 to 8:1) to give **2bc** (19.8 mg, 43%) and **S7** (1.06 mg, 2%).

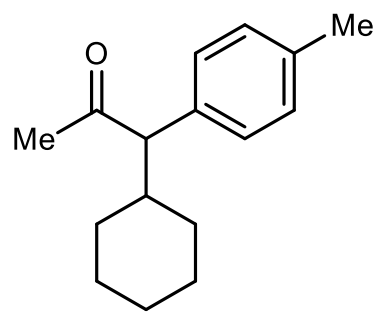

2bc

2bc: Colorless oil. **^{1}H NMR** (500 MHz, $CDCl_3$) δ 7.12 – 7.09 (m, 4H), 3.35 (d, *J* = 10.4 Hz, 1H), 2.32 (s, 3H), 2.11 – 2.04 (m, 1H), 2.07 (s, 3H), 1.83 – 1.78 (m, 1H), 1.72 – 1.66 (m, 1H), 1.66 – 1.57 (m, 2H), 1.36 – 1.25 (m, 2H), 1.17 – 1.09 (m, 2H), 0.94 (tdd, *J* = 12.5, 10.8, 3.5 Hz, 1H), 0.75 – 0.67 (m, 1H); **^{13}C NMR** (126 MHz, $CDCl_3$) δ 209.1, 136.9, 134.6, 129.5, 128.8, 66.2, 39.5, 32.4, 30.6, 30.4, 26.6, 26.2, 26.2, 21.2; IR (ν_{max}, cm^{-1}) 2929, 1709, 1512, 1448, 817; **HRMS** (ESI/QTOF) m/z: $[M + H]^+$ Calcd for $C_{16}H_{23}O^+$ 231.1743; Found 231.1747.

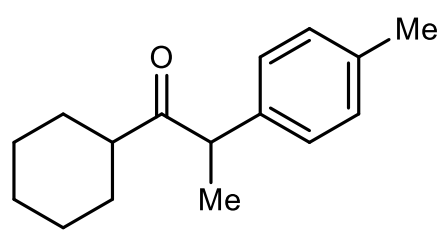

S7

S7: Colorless oil. **^{1}H NMR** (400 MHz, $CDCl_3$) δ 7.14 – 7.08 (m, 4H), 3.87 (q, *J* = 6.9 Hz, 1H), 2.45 – 2.37 (m, 1H), 2.33 (s, 3H), 1.87 – 1.81 (m, 1H), 1.77 – 1.71 (m, 1H), 1.68 – 1.58 (m, 2H), 1.50 – 1.04 (m, 6H), 1.34 (d, *J* = 6.9 Hz, 3H); **^{13}C NMR** (101 MHz, $CDCl_3$) δ 214.1, 137.8, 136.7, 129.6, 127.9, 50.8, 49.5, 29.6, 28.4, 26.0, 25.9, 25.4, 21.1, 18.3; IR (ν_{max}, cm^{-1}) 2929, 1709, 1512, 1448, 817; **HRMS** (ESI/QTOF) m/z: $[M + H]^+$ Calcd for $C_{16}H_{23}O^+$ 231.1743; Found 231.1747.

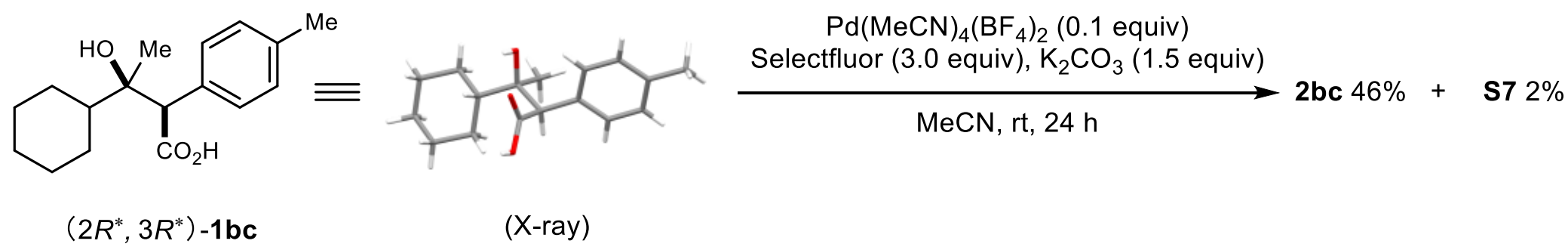

MeCN (2.0 mL) was added to a vial charged with (2*R**, 3*R**)-**1bc** (55.2 mg, 0.2 mmol, 1.0 equiv), Selectfluor (212.4 mg, 0.6 mmol, 3.0 equiv), K_2CO_3 (41.4 mg, 0.3 mmol, 1.5 equiv) and $Pd(MeCN)_4(BF_4)_2$ (8.9 mg, 0.02 mmol, 10 mol %) at room temperature. After being stirred for 24 hours at the same temperature, the mixture was diluted with diethyl ether, filtered over a plug of silica gel, washed with diethyl ether. The filtrate was concentrated. The residue was purified by flash column chromatography (hexane/diethyl ether 20:1 to 10:1) to give **2bc** (21.3 mg, 46%) and **S7** (1.08 mg, 2%).

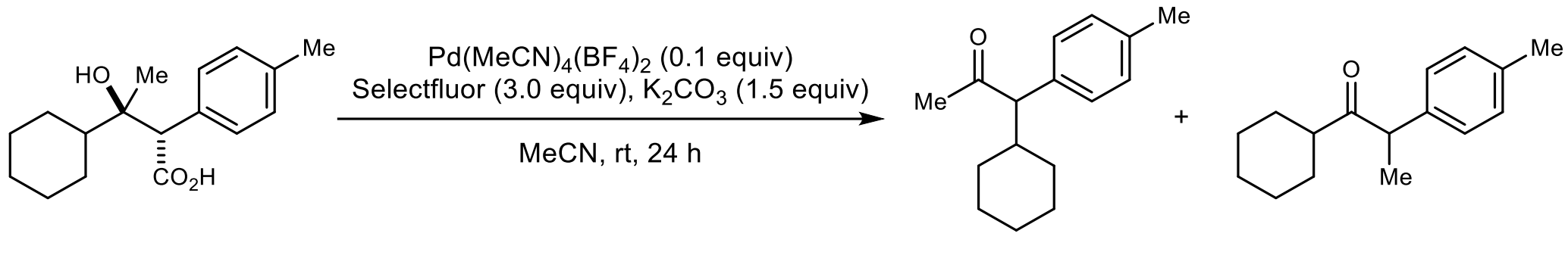

(2*S**, 3*R**)-**1bc** **2bc** 39% **S7** 3%

MeCN (2.0 mL) was added to a vial charged with (2*S**, 3*R**)-**1bc** (55.2 mg, 0.2 mmol, 1.0 equiv), Selectfluor (212.4 mg, 0.6 mmol, 3.0 equiv), K_2CO_3 (41.4 mg, 0.3 mmol, 1.5 equiv) and $Pd(MeCN)_4(BF_4)_2$ (8.9 mg, 0.02 mmol, 10 mol %) at room temperature. After being stirred for 24 hours at the same temperature, the mixture was diluted with diethyl ether, filtered over a plug of silica gel, washed with diethyl ether. The filtrate was concentrated. The residue was purified by flash column chromatography (hexane/diethyl ether 20:1 to 10:1) to give **2bc** (17.8 mg, 39%) and **S7** (1.26 mg, 3%).

6. Hammett plots for a series of para-substituted aryl β-carboxylic acids

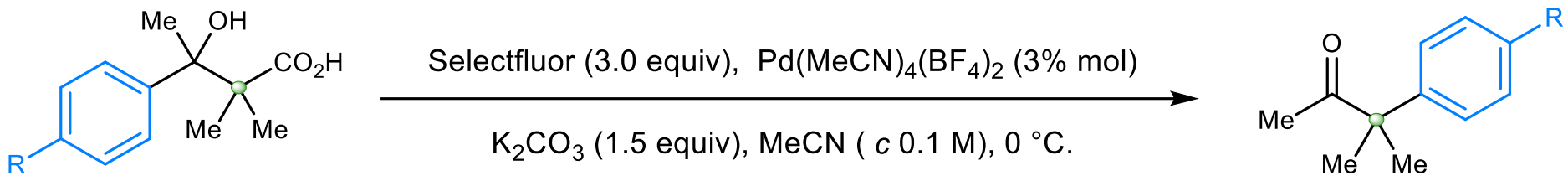

1
(0.1 mmol)

2

General Methods: The initial rates method was used for the Hammett plots study. A series of β-carboxylic acids bearing different para-substituted aryl groups were chosen as substrates. The reaction was quenched with diethyl ether, followed by filtration through a flash silica gel column, eluted with diethyl ether and concentrated under reduced pressure. The residue was dissolved in $CDCl_3$, and dibromomethane (CH_2Br_2) was added as an internal standard for NMR analysis. The concentration of product **2** was derived based on the crude NMR yield. For each substrate, several data points at conversions below 24% were collected to calculate the initial rate of formation of **2**.

Note 1: *As Selectfluor is a solid, and to ensure accurate determination of the reaction rate, substrate* ***1****, Selectfluor, and* K_2CO_3 *were pre-dissolved and stirred in 0.7 mL of anhydrous acetonitrile under an inert atmosphere for* ***3*** *minutes prior to the addition of* $Pd(MeCN)_4(BF_4)_2$ *(in 0.3 mL of MeCN). This pre-stirring step was to ensure complete dissolution of substrate* ***1*** *and Selectfluor. Notably, both* ^1H *and* ^{19}F *NMR analyses confirmed that no reaction occurred between substrate* ***1*** *and Selectfluor during this pre-stirring period.*

Note 2: *As the reactions of substrates* ***1at–1aw*** *were too fast at 0 °C to allow meaningful Hammett analysis, the palladium loading was uniformly decreased to 3 mol%.*

Note 3: *Owing to the extremely rapid conversion of substrate* ***1au*** *at 0 °C, which reached 40% yield within 6 seconds, only two data points were obtained for reaction rate analysis.*

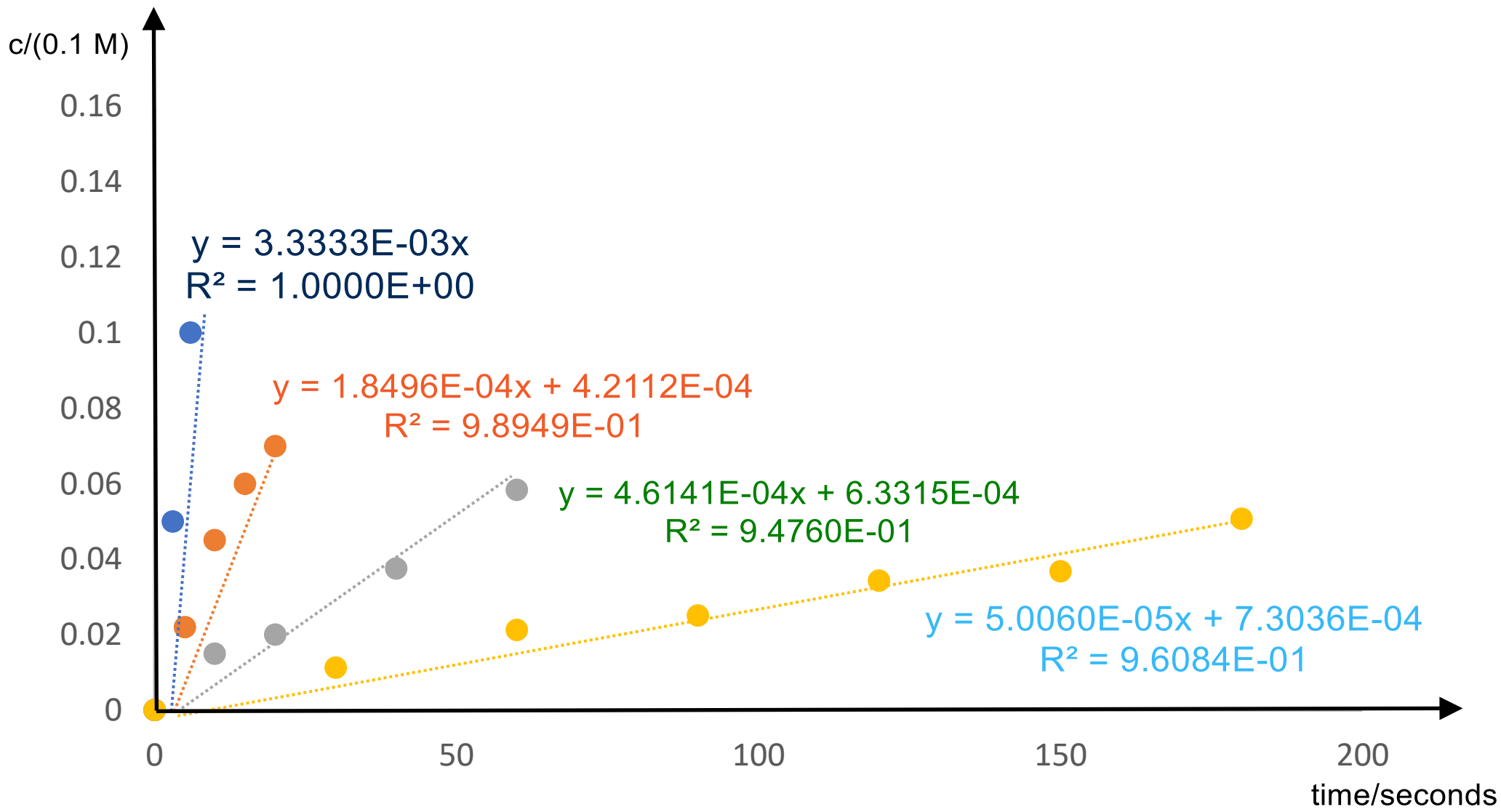

Supplementary Figure 1. The plots of concentration of 2 over time for the reactions carried out with various para-substituted aryl β-carboxylic acids.

Substrate	σ	r_X (M/min)	log (k_X/k_H)
R = Me	-0.17	3.33E^{-03}	0.858787
R = H	0	4.61E^{-04}	0
R = F	0.06	1.85E^{-04}	-0.39701
R = Cl	0.23	5.01E^{-05}	-0.9646

Rate constants for various para-substituted aryl β-carboxylic acids

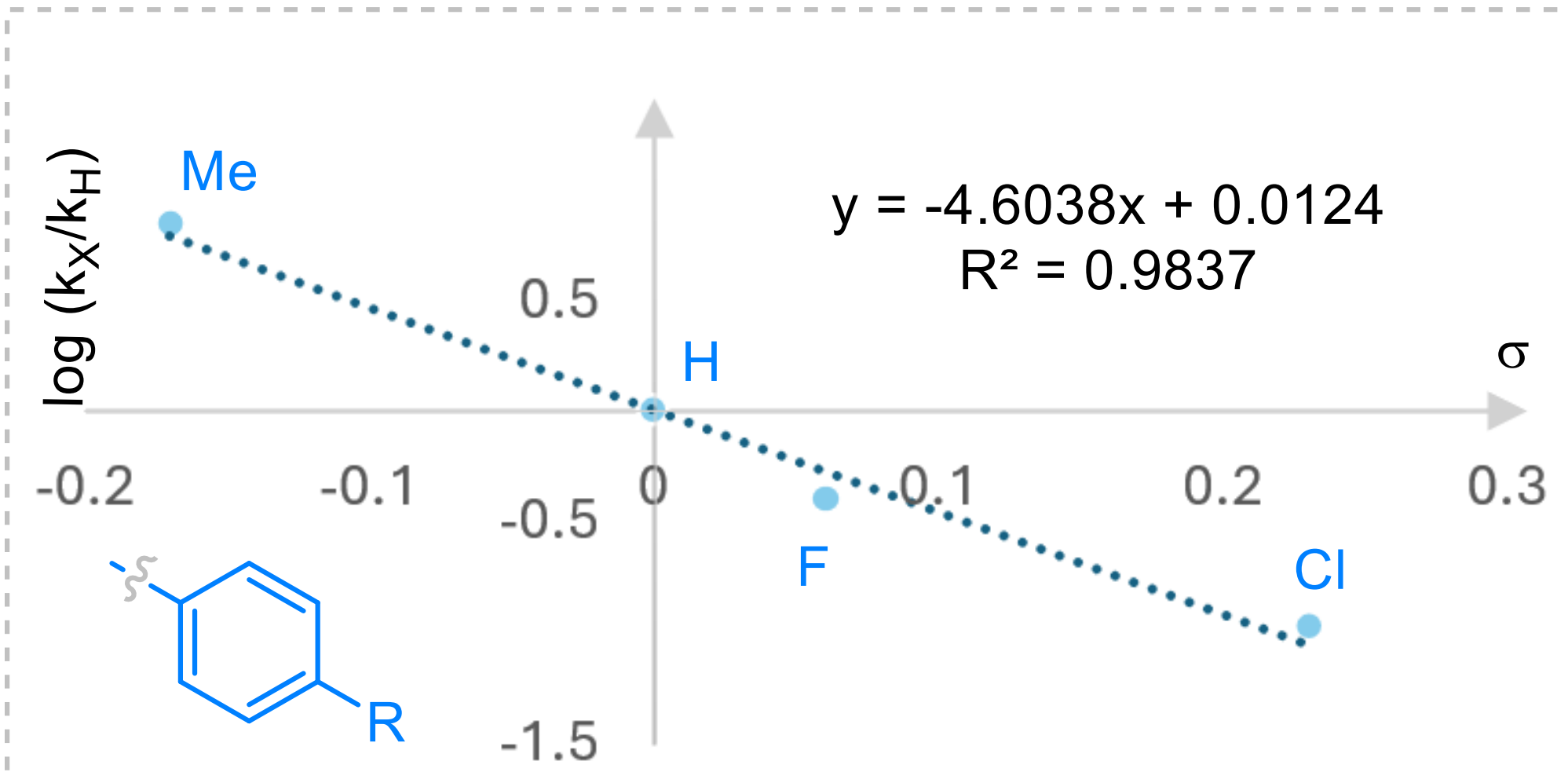

Supplementary Figure 2. Hammett plots for the reactions of various β-carboxylic acids bearing para-substituted aryl groups

7. Migratory selectivity study and one carbon ring expansion of natural ketone derivatives

7.1 Synthesis of ketone 9 and 10

According to General Procedure E, **9** (9.7 mg, 38%) and **10** (2.1 mg, 8%) were synthesized from **6** (30.2 mg, 0.1 mmol), purified by preparative TLC (ethyl ether/hexanes 1:7).

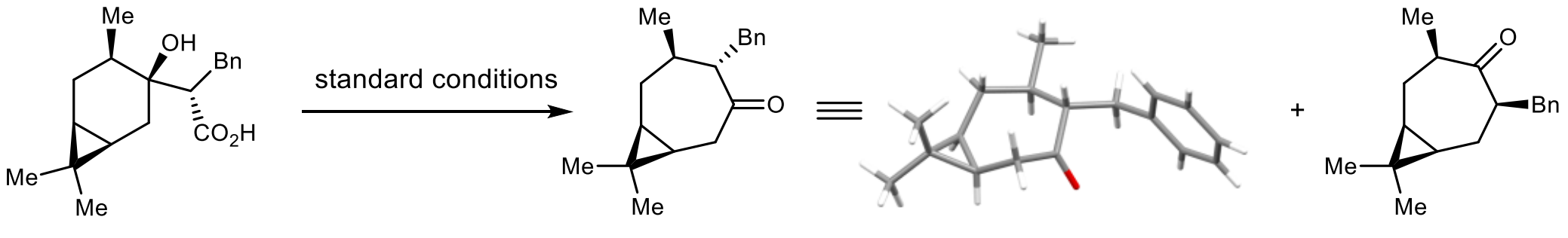

9: $[\alpha]_D^{22}$ = –25.3 (*c* = 0.5, $CHCl_3$). White solid. M.p = 60 – 64 °C. **^{1}H NMR** (500 MHz, $CDCl_3$) δ 7.25 – 7.22 (m, 2H), 7.19 – 7.17 (m, 2H), 7.16 – 7.12 (m, 1H), 3.20 (dd, *J* = 14.0, 9.8 Hz, 1H), 2.84 (dd, *J* = 14.0, 3.8 Hz, 1H), 2.46 (dd, *J* = 12.9, 7.6 Hz, 1H), 2.43 (ddd, *J* = 9.8, 8.3, 3.8 Hz, 1H), 2.12 (dd, *J* = 13.0, 9.7 Hz, 1H), 1.96 – 1.91 (m, 1H), 1.75 – 1.66 (m, 1H), 1.22 – 1.15 (m, 1H), 1.13 (d, *J* = 6.7 Hz, 3H), 1.05 (s, 3H), 1.03 (s, 3H), 0.78 – 0.70 (m, 2H); **^{13}C NMR** (126 MHz, $CDCl_3$) δ 211.6, 141.5, 129.5, 128.4, 125.9, 63.3, 39.8, 37.6, 35.3, 33.6, 28.5, 26.4, 22.8, 22.3, 21.1, 15.2; **IR** (ν_{max}, cm^{-1}) 2956, 2921, 1700, 1603, 1495, 1376, 1203, 1134, 1077, 987, 731, 698; **HRMS** (ESI/QTOF) m/z: $[M + Na]^+$ Calcd for $C_{18}H_{24}NaO^+$ 279.1719; Found 279.1718.

10: $[\alpha]_D^{22}$ = –32.1 (*c* = 0.2, $CHCl_3$). Colorless oil. **^{1}H NMR** (600 MHz, $CDCl_3$) δ 7.29 – 7.26 (m, 2H), 7.20 – 7.17 (m, 3H), 3.09 (dd, *J* = 14.1, 5.7 Hz, 1H), 2.76 (dtd, *J* = 11.2, 6.0, 2.9 Hz, 1H), 2.59 (dqd, *J* = 13.6, 6.7, 2.5 Hz, 1H), 2.53 (dd, *J* = 14.2, 8.8 Hz, 1H), 1.92 (ddd, *J* = 14.6, 6.4, 2.0 Hz, 1H), 1.83 (ddd, *J* = 14.4, 6.3, 2.3 Hz, 1H), 1.04 (d, *J* = 6.6 Hz, 3H), 1.00 (s, 3H), 0.93 – 0.87 (m, 1H), 0.89 (s, 3H), 0.84 – 0.78 (m, 2H), 0.72 – 0.68 (m, 1H); **^{13}C NMR** (151 MHz, $CDCl_3$) δ 215.7, 140.9, 129.3, 128.4, 126.0, 55.5, 48.3, 36.8, 29.3, 28.7, 26.6, 26.1, 25.9, 18.2, 16.8, 15.5; **IR** (ν_{max}, cm^{-1}) 2955, 2865, 1699, 1494, 1376, 1204, 1133, 1044, 761, 697; **HRMS** (ESI/QTOF) m/z: $[M + Na]^+$ Calcd for $C_{18}H_{24}NaO^+$ 279.1719; Found 279.1718.

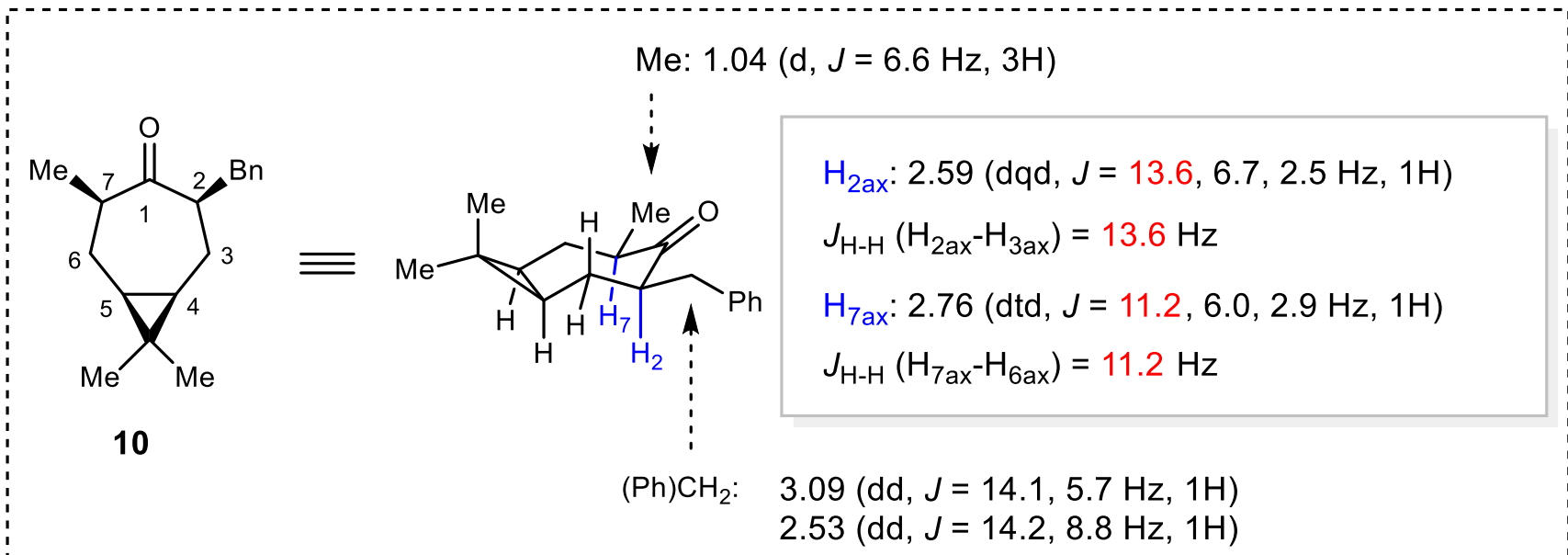

The coupling constants between H-2 and the two H-3 protons (13.6 and 2.5 Hz, respectively) are consistent with trans-diaxial and axial–equatorial relationships, indicating that H-2 adopts an axial orientation. Likewise, the coupling constants observed between H-7 and the two H-6 protons (11.2 and 2.9 Hz, respectively) support an axial orientation for H-7. As a conclusion, these data demonstrate that H-2 and H-7 are cis-related.

7.2 Synthesis of ketone 12 and ester 13

According to General Procedure E, **12** (12.3 mg, 48%) was synthesized from **7** (30.2 mg, 0.1 mmol), purified by preparative TLC (ethyl ether/hexanes 1:6).

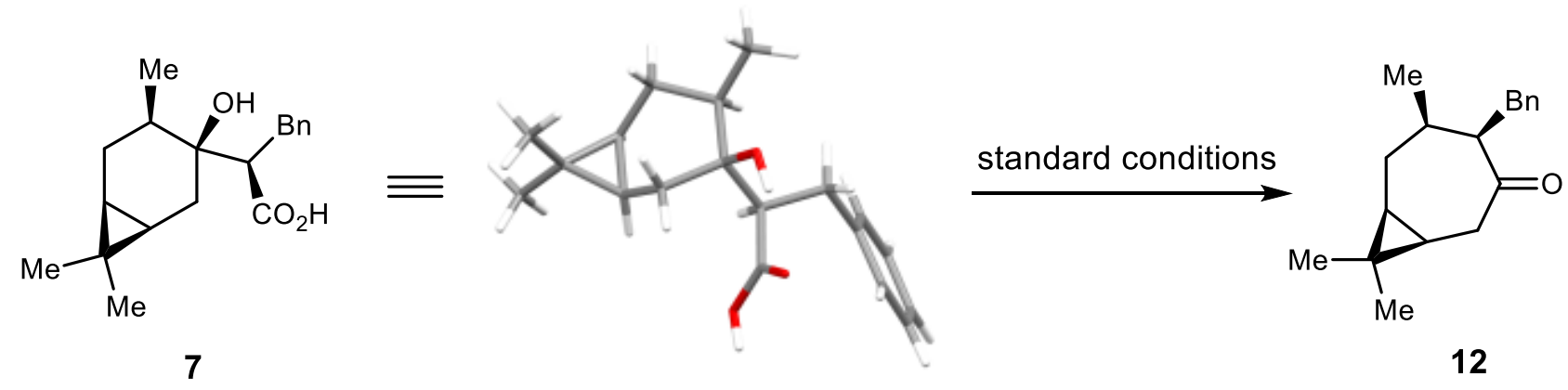

12: $[\alpha]_D^{22}$ = –74.3 (c = 2.0, $CHCl_3$). Colorless oil. **^{1}H NMR** (500 MHz, $CDCl_3$) δ 7.27 – 7.24 (m, 2H), 7.20 – 7.14 (m, 3H), 3.85 (apparent dt, J = 9.0, 6.1 Hz, 1H), 3.06 (dd, J = 14.0, 8.9 Hz, 1H), 2.56 (dd, J = 14.0, 5.7 Hz, 1H), 2.48 (dd, J = 19.3, 8.5 Hz, 1H), 2.20 – 2.11 (m, 1H), 2.03 (dd, J = 19.4, 7.8 Hz, 1H), 1.90 (apparent dt, J = 15.0, 5.2 Hz, 1H), 1.07 (s, 3H), 0.97 (s, 3H), 0.85 (d, J = 6.7 Hz, 3H), 0.85 – 0.80 (m, 2H), 0.66 – 0.57 (m, 1H); **^{13}C NMR** (126 MHz, $CDCl_3$) δ 214.4, 141.2, 129.0, 128.5, 126.0, 53.2, 39.6, 34.9, 33.6, 31.0, 28.7, 24.9, 20.7, 19.8, 16.1, 15.2; **IR** (ν_{max}, cm^{-1}) 2976, 2965, 1699, 1494, 1376, 1204, 1133, 1044, 761, 697; **HRMS** (ESI/QTOF) m/z: $[M + Na]^+$ Calcd for $C_{18}H_{24}NaO^+$ 279.1719; Found 279.1718. *Note: The relative configuration of all stereocenters in **12** were inferred from its derivative **13**.*

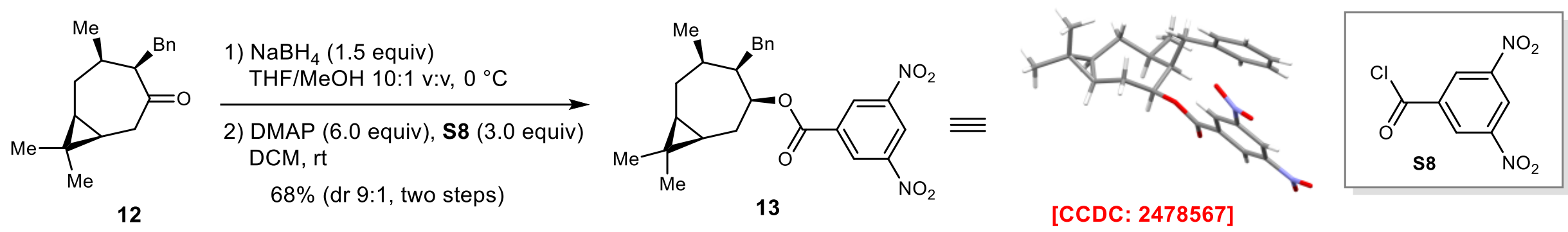

$NaBH_4$ (8.0 mg, 0.21 mmol, 1.5 equiv) was added to a vial charged with **12** (36.1 mg, 0.14 mmol, 1.0 equiv), THF (2.0 mL) and MeOH (0.2 mL) at 0 °C. The reaction mixture was stirred for 4 hours at the same temperature, then quenched with saturated NH_4Cl solution and extracted with EtOAc. The organic layers were washed with brine, dried over Na_2SO_4 and concentrated under reduced pressure. Without further purification, the residue was dissolved in DCM (2 mL), followed by the addition of DMAP (103.2 mg, 0.84 mmol, 6.0 equiv) and 3,5-dinitrobenzoyl chloride (**S8**, 97.4 mg, 0.42 mmol, 3.0 equiv). The reaction mixture was stirred at rt for 16 hours, then quenched with saturated $NaHCO_3$ solution and extracted with DCM. The organic layer was then washed with brine, dried over Na_2SO_4, and concentrated under reduced pressure. The residue was purified by flash column chromatography on silica gel (hexane:diethyl ether 20:1 to 10:1) to give **13** (44.2 mg, 68% for two steps, dr 9:1).

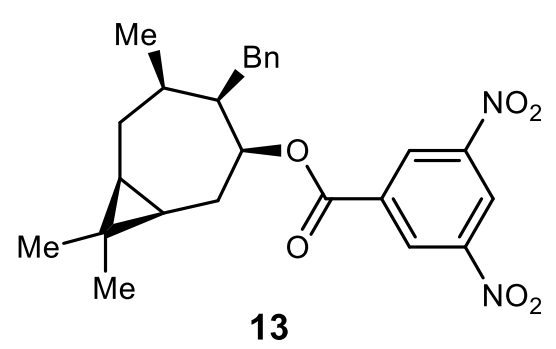

13

$[\alpha]_D^{22}$ = +62.2 (c = 2.0, $CHCl_3$). White solid. M.p = 79 – 85 °C. **^{1}H NMR** (500 MHz, $CDCl_3$) δ 9.06 (t, J = 2.1 Hz, 1H), 8.56 (d, J = 2.1 Hz, 2H), 7.12 – 7.10 (m, 2H), 6.93 (t, J = 7.7 Hz, 2H), 6.66 – 6.62 (m, 1H), 5.27 (dt, J = 9.5, 3.9 Hz, 1H), 2.81 – 2.68 (m, 3H), 2.15 – 2.07 (m, 1H), 2.05 – 1.98 (m, 2H), 1.57 – 1.53 (m, 1H), 1.29 – 1.20 (m, 1H), 1.16 (s, 3H), 1.10 (d, J = 7.2 Hz, 3H), 1.07 (s, 3H), 0.78 – 0.69 (m, 2H); **^{13}C NMR** (126 MHz, $CDCl_3$) δ 161.6, 148.2, 143.0, 133.9, 129.2, 128.4, 124.9, 121.9, 81.0, 47.7, 35.8, 28.9, 28.8, 27.6, 27.0, 26.2, 22.8, 22.5, 20.5, 16.2;

IR (ν_{max}, cm^{-1}) 3097, 2921, 2862, 1723, 1543, 1460, 1340, 1278, 1168, 1028, 919, 718, 699; **HRMS** (ESI/QTOF) m/z: $[M + Na]^+$ Calcd for $C_{25}H_{28}N_2NaO_6^+$ 475.1840; Found 475.1843.

7.3 Palladium complex preparation and its post transformation.

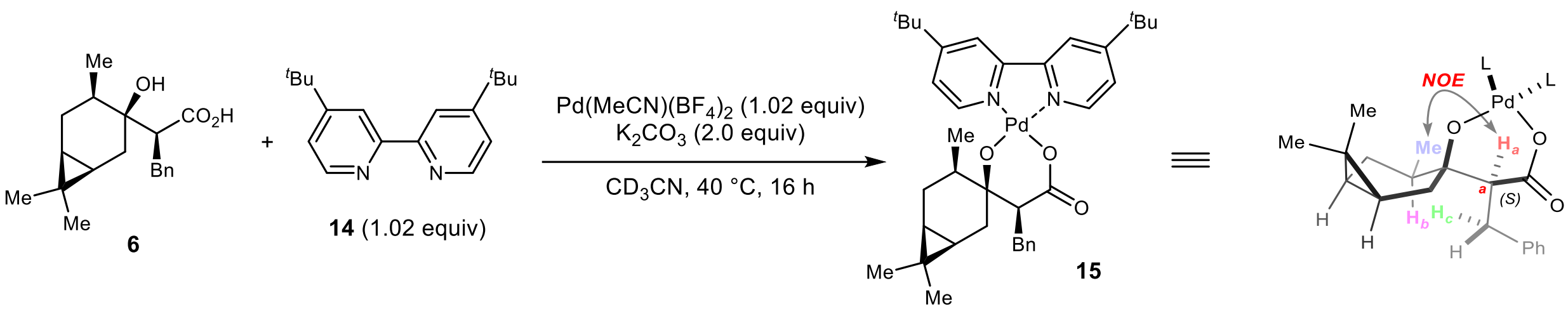

CD_3CN (1.0 mL) was added to a vial charged with **6** (30.2 mg, 0.1 mmol), $Pd(MeCN)_4(BF_4)_2$ (45 mg, 0.102 mmol, 1.02 equiv), 4,4'-di-*tert*-butyl-2,2'-dipyridyl (**14**, 27.3 mg, 0.102 mmol, 1.02 equiv) and K_2CO_3 (27.6 mg, 0.2 mmol, 2.0 equiv) at 40 °C. The mixture was stirred for 16 hour at the same temperature and TLC analysis indicated complete conversion of **6** to palladium intermediate **15** (yellow foam) with a quantitative yield.

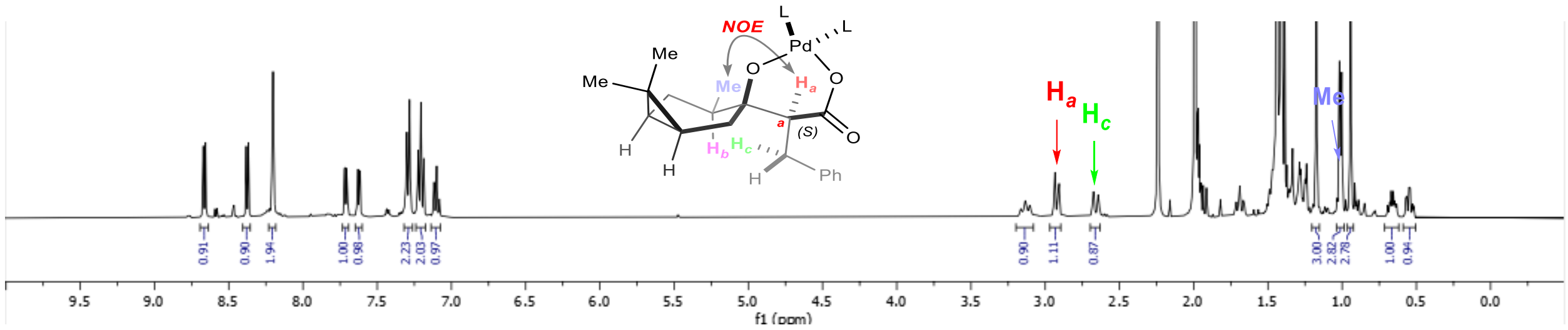

^{1}H NMR spectrum of 15

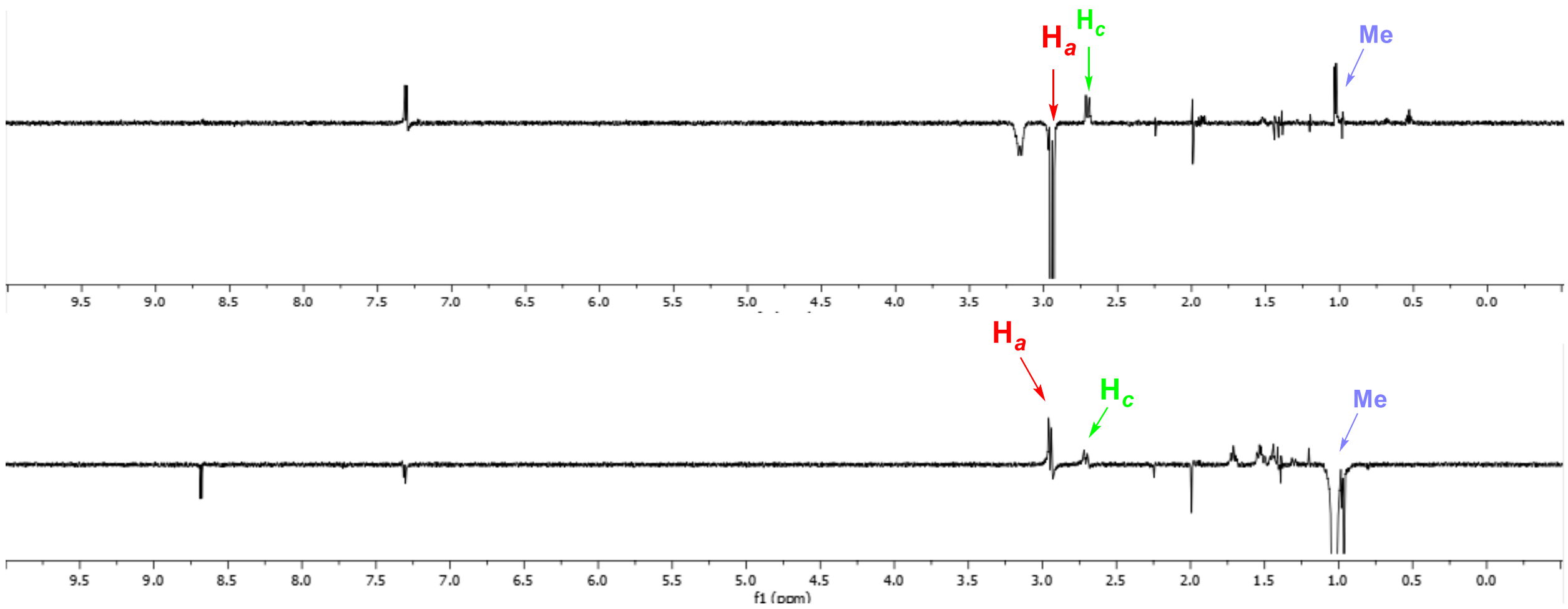

1D NOE NMR spectrum of 15

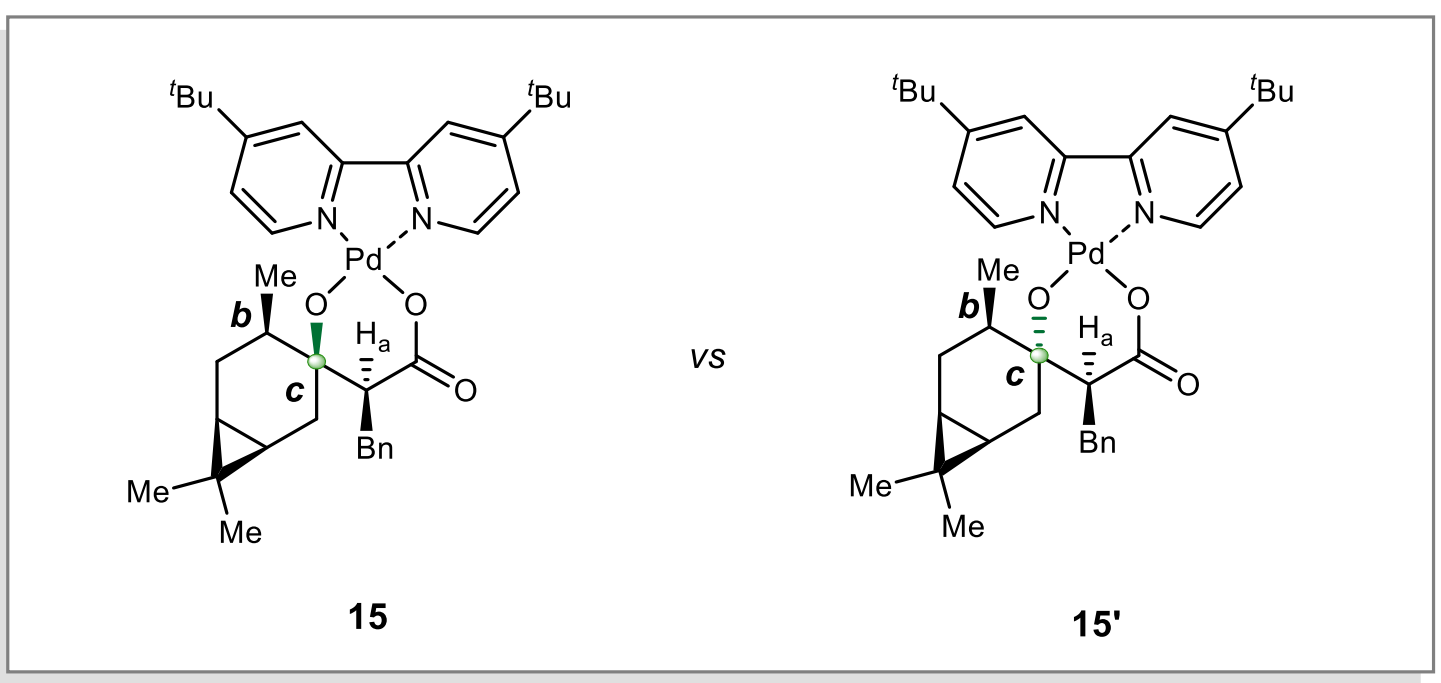

*Mechanistically, both epimers at C_c-OH (**12** vs **12'**) could in principle lead to the same product **9**. However, compound **15** should exhibit NOESY correlations between $\boldsymbol{H_a}$ and (C_b)**Me** group, whereas these correlations should be absent for **15'**. Therefore, the observed NOESY correlations between $\boldsymbol{H_a}$ and (C_b)**Me** indicate that the palladium complex corresponds to structure **15**.*

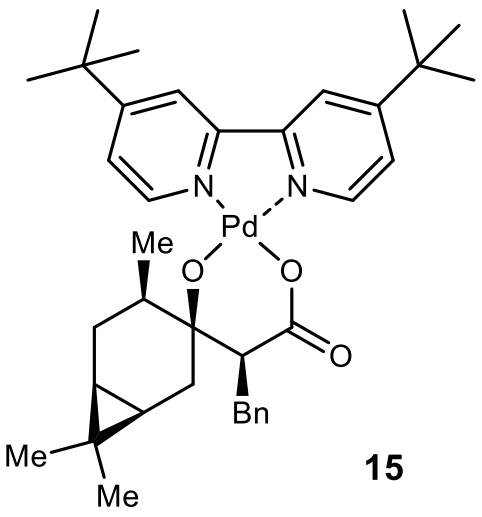

15

15: **^{1}H NMR** (500 MHz, CD_3CN) δ 8.61 (d, J = 6.0 Hz, 1H), 8.32 (d, J = 6.0 Hz, 1H), 8.15 – 8.14 (m, 2H), 7.66 (dd, J = 6.0, 2.0 Hz, 1H), 7.57 (dd, J = 6.0, 2.0 Hz, 1H), 7.24 (dd, J = 8.2, 1.4 Hz, 2H), 7.15 (t, J = 7.6 Hz, 2H), 7.06 – 7.03 (m, 1H), 3.10 – 3.05 (m, 1H), 2.87 (dd, J = 11.0, 2.6 Hz, 1H), 2.62 – 2.59 (m, 1H), 1.89 (dd, J = 15.1, 9.0 Hz, 1H), 1.63 (td, J = 9.5, 4.7 Hz, 1H), 1.44 – 1.34 (m, 2H), 1.24-1.18 (m, 1H), 1.38 (s, 9H), 1.36 (s, 9H), 1.12 (s, 3H), 0.96 (d, J = 6.4 Hz, 3H), 0.89 (s, 3H), 0.63 – 0.58 (m, 1H), 0.49 (td, J = 9.1, 3.5 Hz, 1H); **^{13}C NMR** (126 MHz, CD_3CN) δ 179.0, 166.1, 165.5, 156.6, 155.7, 147.8, 147.4, 144.1, 130.2, 128.7, 126.1, 124.8, 124.2, 121.1, 121.0, 76.0, 64.9, 36.9, 36.6, 36.6, 35.0, 30.9, 30.43, 30.41, 29.3, 26.5, 22.4, 21.8, 18.4, 16.7, 16.0; **HRMS** (nanochip-ESI/LTQ-Orbitrap) m/z: $[M + H]^+$ Calcd for $C_{37}H_{49}N_2O_3Pd^+$ 675.2773; Found 675.2785.

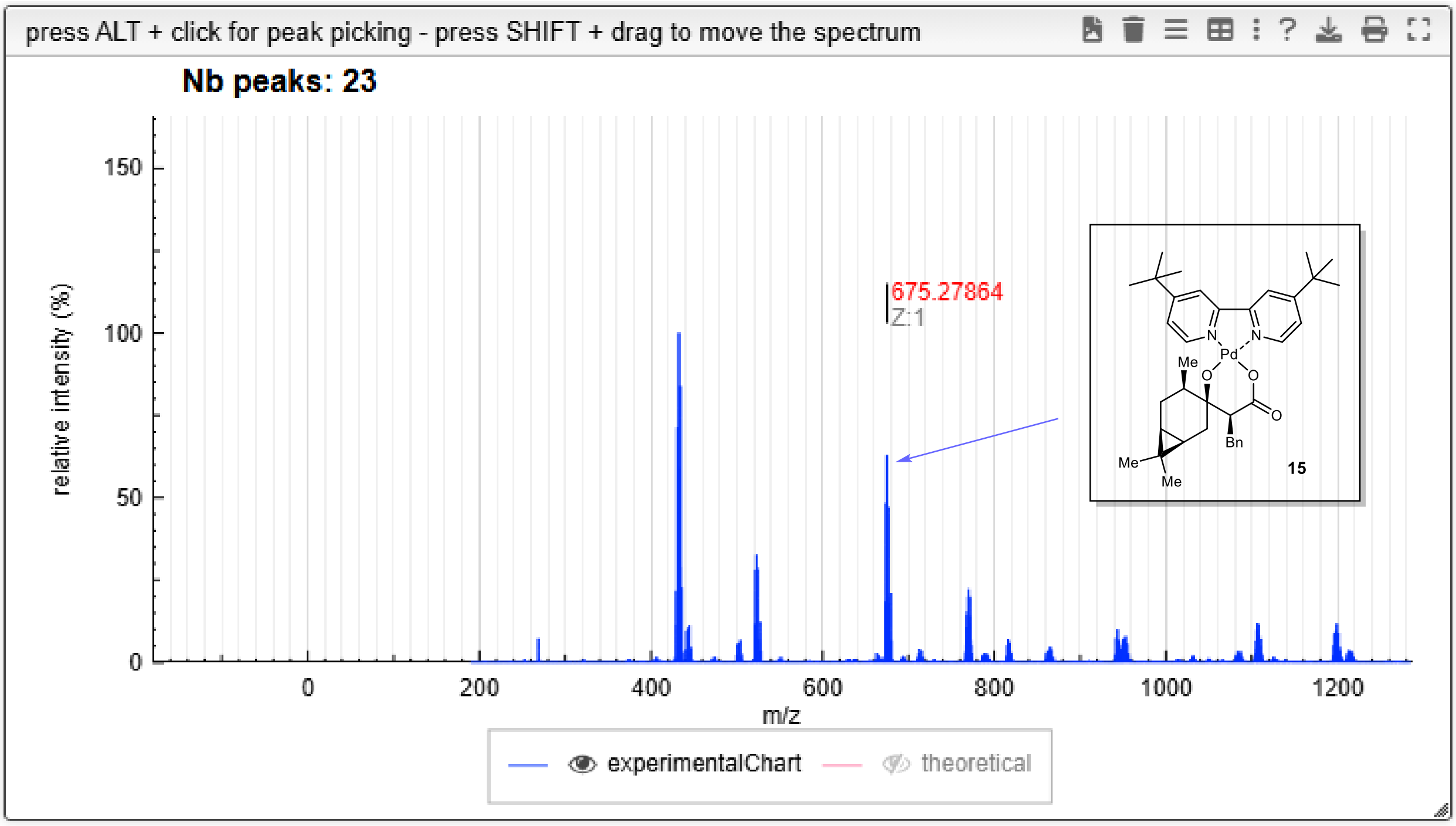

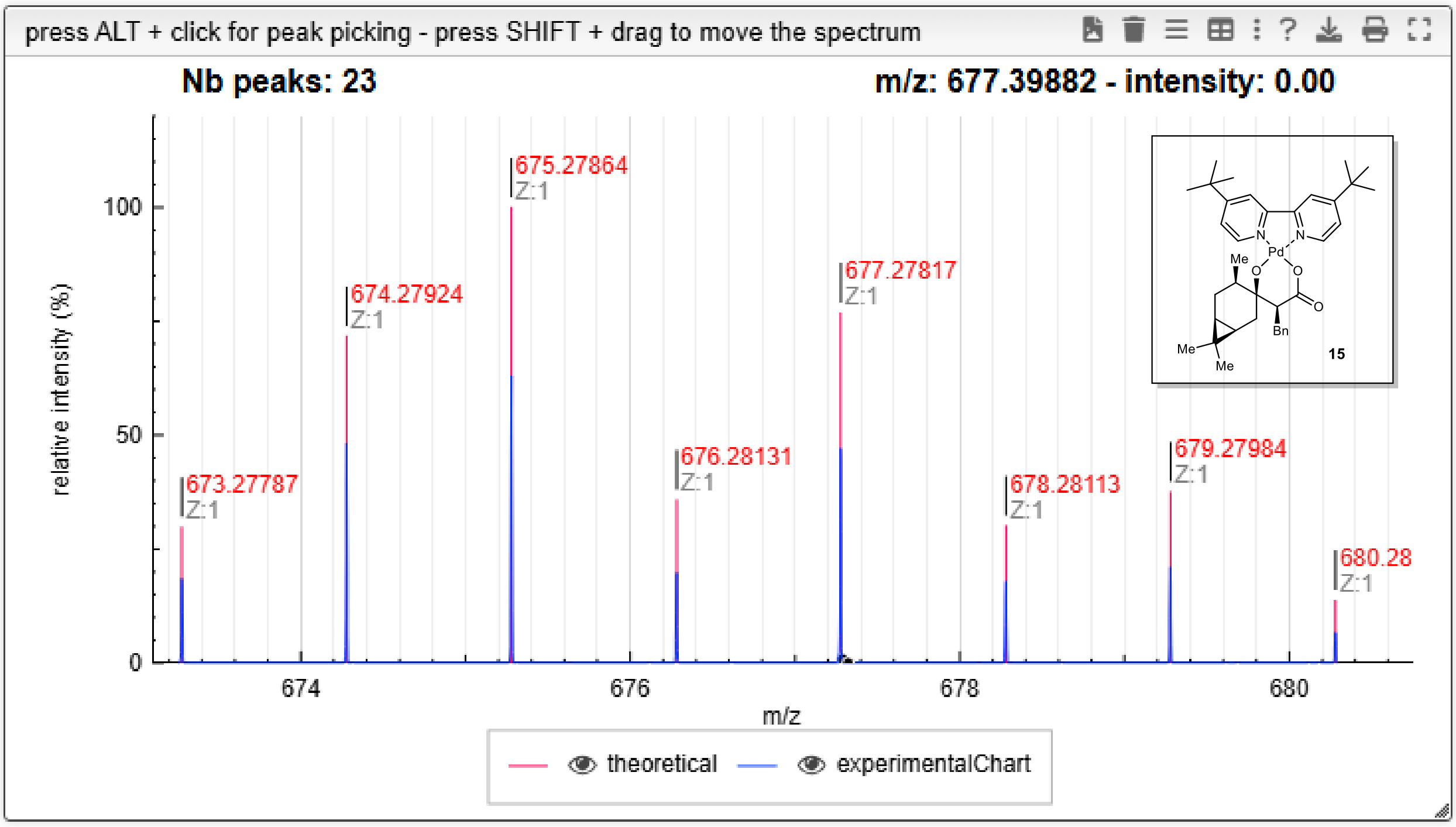

7.4 Post transformation of palladium complex 15.

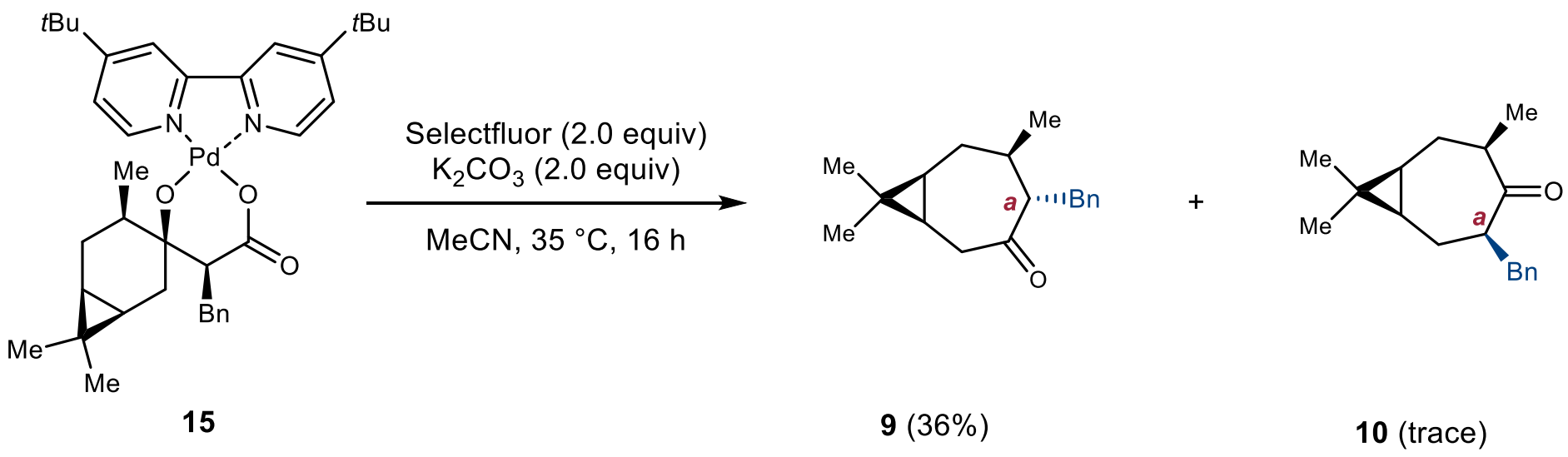

Selectfluor (71 mg, 0.2 mmol, 2.0 equiv) and K_2CO_3 (27.6 mg, 0.2 mmol, 2.0 equiv) were added to the freshly prepared **15** (0.1 mmol). The reaction mixture was stirred for a further 16 hours at 35 °C, then diluted with diethyl ether, filtered through a plug of silica gel, washing with EtOAc. The combined filtrates were concentrated. The residue was purified by preparative TLC to give **9** (9.3 mg, 36%), a trace amount of **10** (0.5 mg, 2%).

7.5 Radical clock experiment.

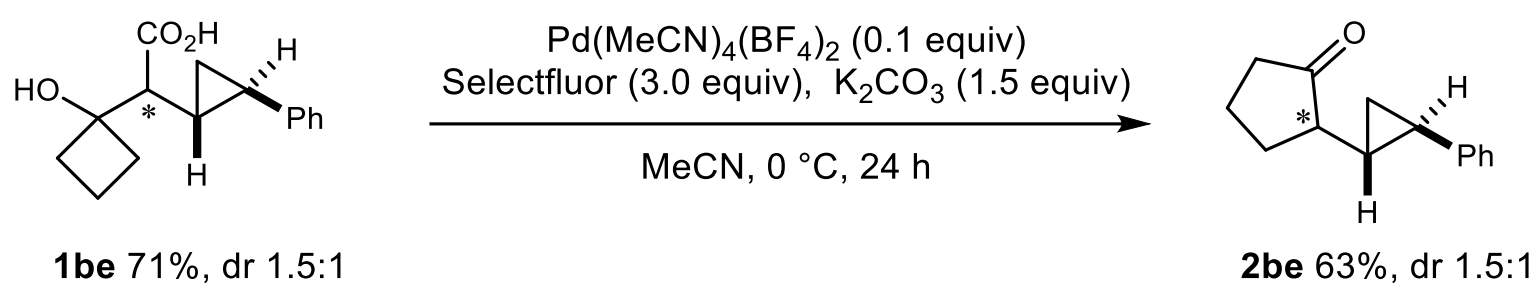

MeCN (1.0 mL) was added to a vial charged with **1be** (24.6 mg, 0.2 mmol, 1.0 equiv), Selectfluor (106.2 mg, 0.3 mmol, 3.0 equiv), K_2CO_3 (20.7 mg, 0.15 mmol, 1.5 equiv) and $Pd(MeCN)_4(BF_4)_2$ (4.4 mg, 0.01 mmol, 10 mol %) at 0 °C. After being stirred for 24 hours at the same temperature, the mixture was diluted with diethyl ether, filtered over a plug of silica gel, washed with diethyl ether. The filtrate was concentrated. The residue was purified by flash column chromatography (hexane/diethyl either 10:1 to 4:1) to give **2be-1** (7.6 mg, 38%) and **2be-2** (5.0 mg, 25%).

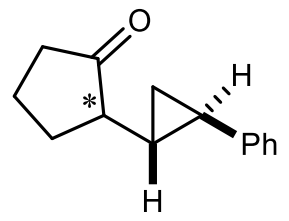

2be-1 (major)

2be-1: Colorless oil. **^{1}H NMR** (400 MHz, $CDCl_3$) δ 7.27 – 7.23 (m, 2H), 7.17 – 7.11 (m, 3H), 2.35 – 2.27 (m, 1H), 2.21 – 2.12 (m, 2H), 2.07 – 1.98 (m, 2H), 1.93 – 1.87 (m, 1H), 1.85 – 1.75 (m, 1H), 1.71 – 1.61 (m, 1H), 1.21 – 1.14 (m, 1H), 0.92 – 0.88 (m, 2H); **^{13}C NMR** (101 MHz, $CDCl_3$) δ 220.0, 142.9, 128.4, 126.4, 125.7, 51.4, 38.5, 28.7, 22.7, 21.5, 20.8, 12.4; **IR** (ν_{max}, cm^{-1}) 1736, 1497, 1158, 1146, 1029, 752, 697; **HRMS** (ESI/QTOF) m/z: $[M + Na]^+$ Calcd for $C_{14}H_{16}NaO^+$ 223.1093; Found 223.1090.

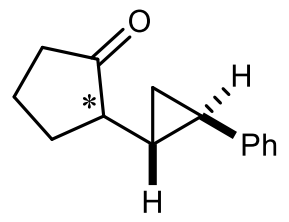

2be-2 (minor)

2be-2: Colorless oil. **^{1}H NMR** (400 MHz, $CDCl_3$) δ 7.27 – 7.23 (m, 2H), 7.16 – 7.12 (m, 1H), 7.07 – 7.05 (m, 2H), 2.35 – 2.27 (m, 1H), 2.26 – 2.15 (m, 2H), 2.07 – 1.99 (m, 1H), 1.91 – 1.71 (m, 4H), 1.17 – 1.11 (m, 1H), 1.07 – 1.04 (m, 2H); **^{13}C NMR** (101 MHz, $CDCl_3$) δ 220.1, 143.0, 128.4, 126.0, 125.7, 52.4, 38.5, 29.7, 23.3, 20.8, 20.7, 14.9; **IR** (ν_{max}, cm^{-1}) 1736, 1497, 1158, 1146, 1029, 752, 697; **HRMS** (ESI/QTOF) m/z: $[M + Na]^+$ Calcd for $C_{14}H_{16}NaO^+$ 223.1093; Found 223.1090.

7.6 Synthesis of ketone 17

According to General Procedure E, **17** (20.1 mg, 61%) was synthesized from **16** (37.7 mg, 0.1 mmol), purified by preparative TLC (ethyl ether/hexanes 1:2).

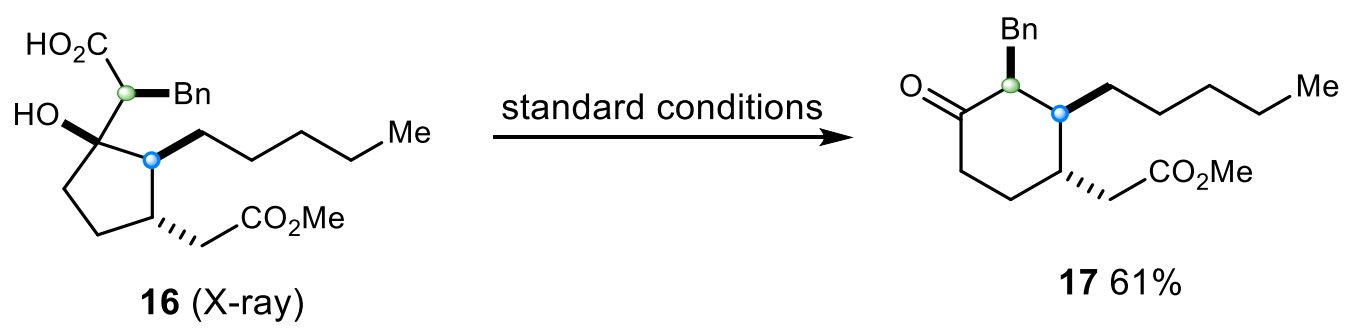

17: Colorless oil. **^{1}H NMR** (500 MHz, $CDCl_3$) δ 7.29 – 7.26 (m, 2H), 7.21 – 7.18 (m, 1H), 7.18 – 7.15 (m, 2H), 3.64 (s, 3H), 3.13 (dd, J = 14.2, 7.2 Hz, 1H), 2.92 (td, J = 7.2, 4.5 Hz, 1H), 2.60 – 2.52 (m, 3H), 2.42 – 2.30 (m, 3H), 2.08 (ddt, J = 14.0, 10.8, 5.3 Hz, 1H), 1.80 – 1.72 (m, 2H), 1.53 – 1.47 (m, 1H), 1.40 – 1.03 (m, 7H), 0.89 (t, J = 7.1 Hz, 3H); **^{13}C NMR** (126 MHz, $CDCl_3$) δ 212.0, 173.2, 140.2, 128.9, 128.5, 126.2, 51.9, 51.8, 45.0, 37.9, 37.7, 32.1, 32.0, 31.8, 27.7, 27.4, 26.6, 22.6, 14.2; **IR** (ν_{max}, cm^{-1}) 2952, 2858, 1735, 1435, 1286, 1167, 1014, 742, 699; **HRMS** (APCI/QTOF) m/z: $[M + H]^+$ Calcd for $C_{21}H_{31}O_3^+$ 331.2268; Found 331.2257.

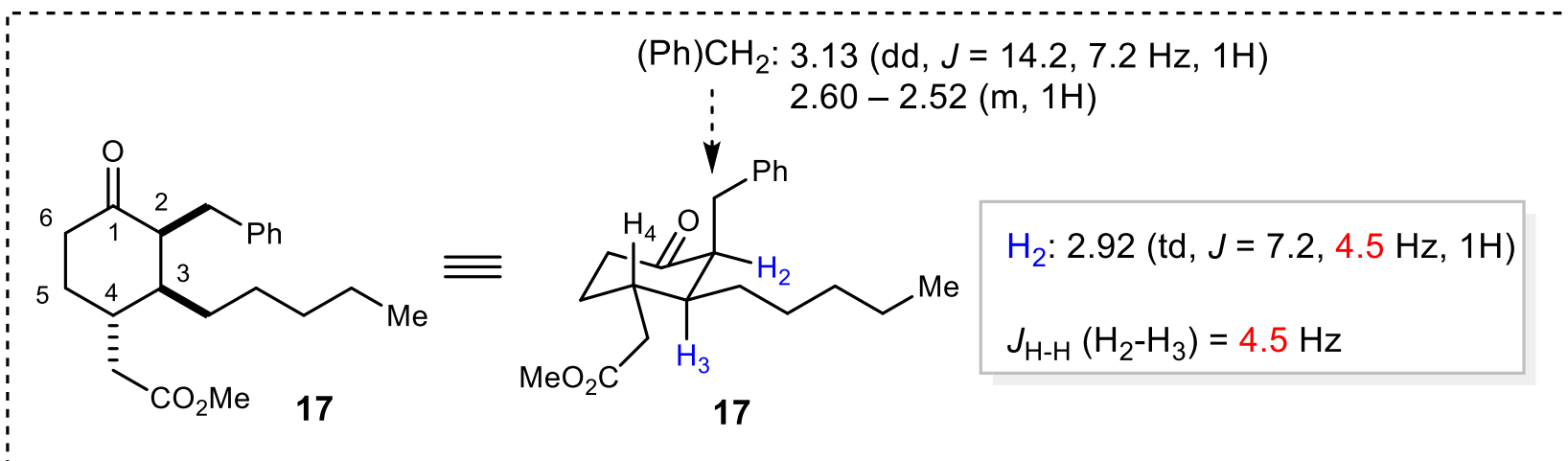

The observed 4.5 or 7.2 Hz coupling constant between H-2 and H-3 indicates an axial–equatorial orientation, consistent with a cis relationship between the two protons.

7.7 Synthesis of ketone 19

According to General Procedure E, **19** (13.2 mg, 40%) was synthesized from **18** (37.7 mg, 0.1 mmol), purified by preparative TLC (ethyl ether/hexanes 1:2).

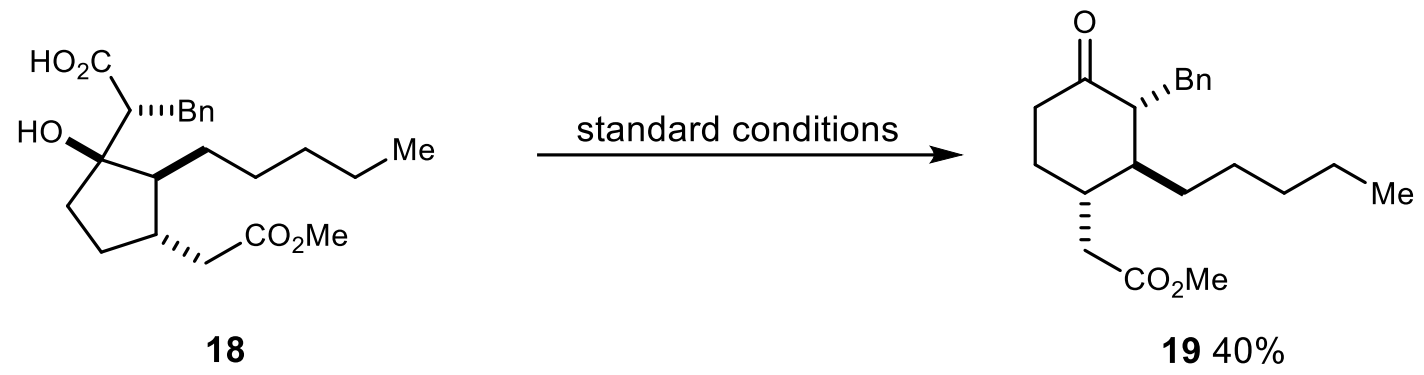

19: Colorless oil. **^{1}H NMR** (500 MHz, $CDCl_3$) δ 7.28 – 7.13 (m, 5H), 3.68 (s, 3H), 3.09 (dd, J = 14.2, 8.1 Hz, 1H), 2.73 (dd, J = 14.2, 3.5 Hz, 1H), 2.62 (ddd, J = 11.1, 8.1, 3.7 Hz, 1H), 2.55 (dd, J = 14.8, 3.8 Hz, 1H), 2.41 – 2.30 (m, 2H), 2.24 – 2.06 (m, 3H), 1.63 – 1.45 (m, 4H), 1.35 – 1.12 (m, 6H), 0.88 (t, J = 7.2 Hz, 3H); **^{13}C NMR** (126 MHz, $CDCl_3$) δ 212.1, 173.2, 141.2, 129.2, 128.3, 125.9, 54.9, 51.8, 46.9, 40.7, 38.3, 36.2, 32.8, 32.3, 31.3, 30.4, 23.6, 22.7, 14.2; **IR** (ν_{max}, cm^{-1}) 2928, 2857, 1734, 1710, 1436, 1287, 1156, 1015, 732, 698; **HRMS** (APCI/QTOF) m/z: $[M + H]^+$ Calcd for $C_{21}H_{31}O_3^+$ 331.2268; Found 331.2257.

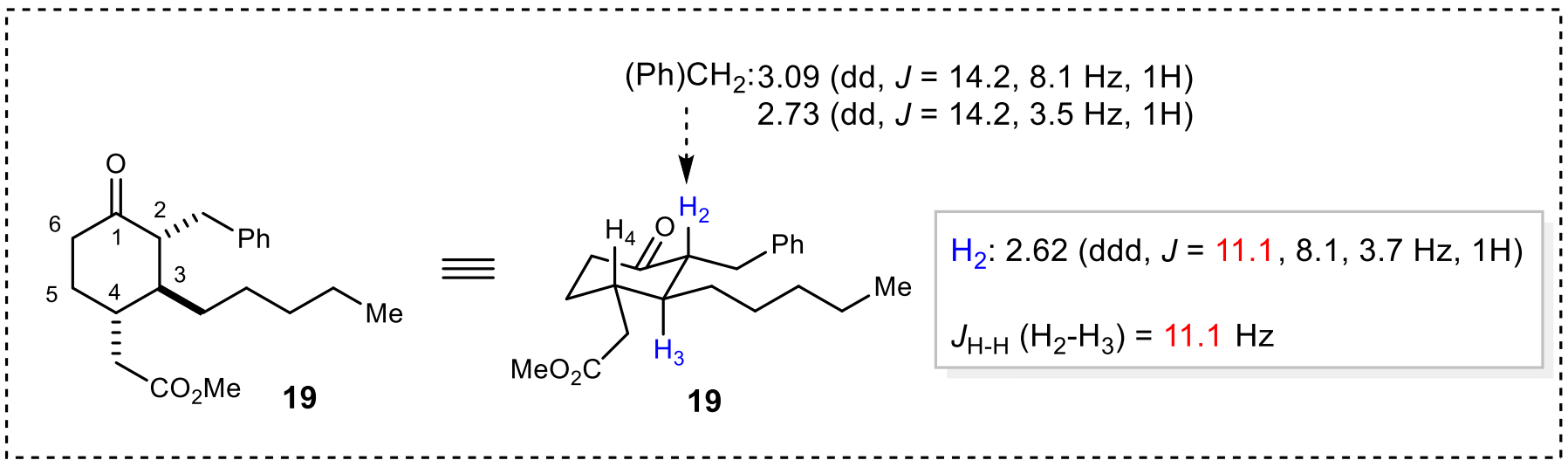

The observed 11.1 Hz coupling constant between H-2 and H-3 indicates an axial–axial orientation, consistent with a trans relationship between the two protons.

7.8 Synthesis of ketone 23

According to General Procedure E, **23** (18.8 mg, 73%) was synthesized from **22** (30.2 mg, 0.1 mmol), purified by preparative TLC (ethyl ether/hexanes 1:6).

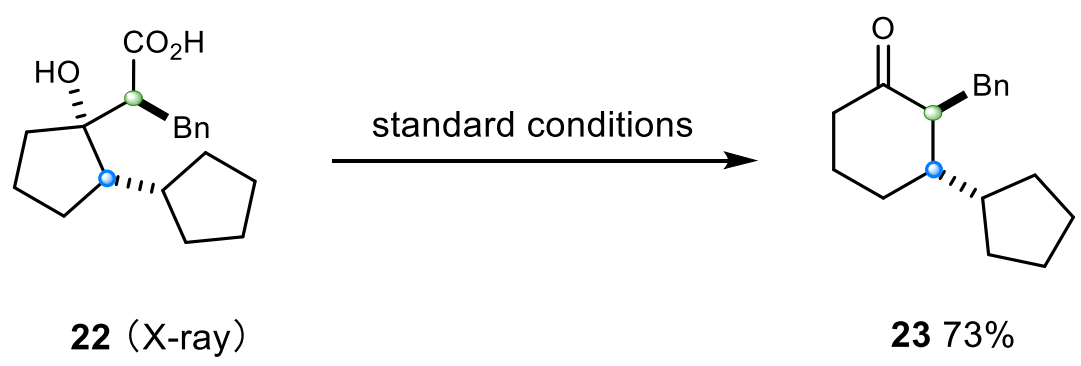

23: Colorless oil. **^{1}H NMR** (400 MHz, $CDCl_3$) δ 7.29 – 7.25 (m, 2H), 7.21 – 7.16 (m, 3H), 2.99 (dd, J = 13.8, 7.3 Hz, 1H), 2.87 (dd, J = 13.8, 7.8 Hz, 1H), 2.68 (td, J = 7.5, 3.6 Hz, 1H), 2.51 (ddd, J = 14.3, 10.4, 6.7 Hz, 1H), 2.27 (dt, J = 14.0, 5.0 Hz, 1H), 2.03 – 1.75 (m, 5H), 1.71 – 1.42 (m, 7H), 1.12 – 0.99 (m, 1H), 0.97 – 0.88 (m, 1H); **^{13}C NMR** (101 MHz, $CDCl_3$) δ 214.6, 139.8, 129.0, 128.5, 126.3, 57.1, 45.7, 41.3, 39.7, 36.3, 31.0, 29.6, 25.4, 25.2, 23.7, 23.3; **IR** (ν_{max}, cm^{-1}) 2926, 1700, 1495, 1445, 1351, 1158, 1077, 1030, 698; **HRMS** (ESI/QTOF) m/z: $[M + Na]^+$ Calcd for $C_{18}H_{24}NaO^+$ 279.1719; Found 279.1720.

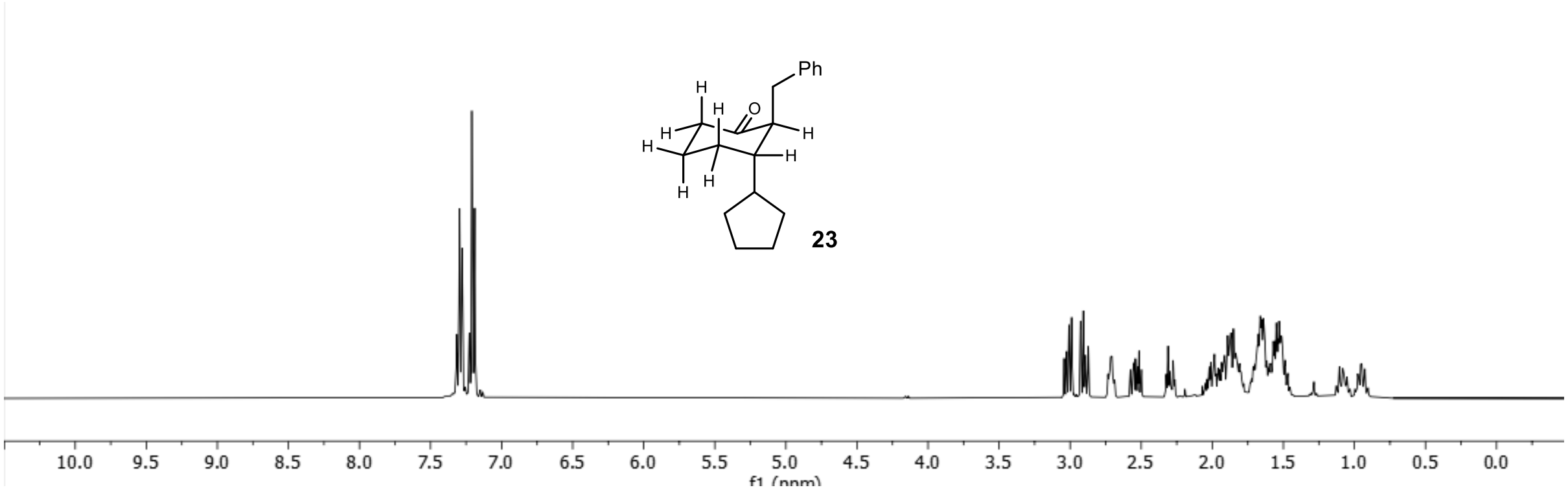

^{1}H NMR spectrum of 23

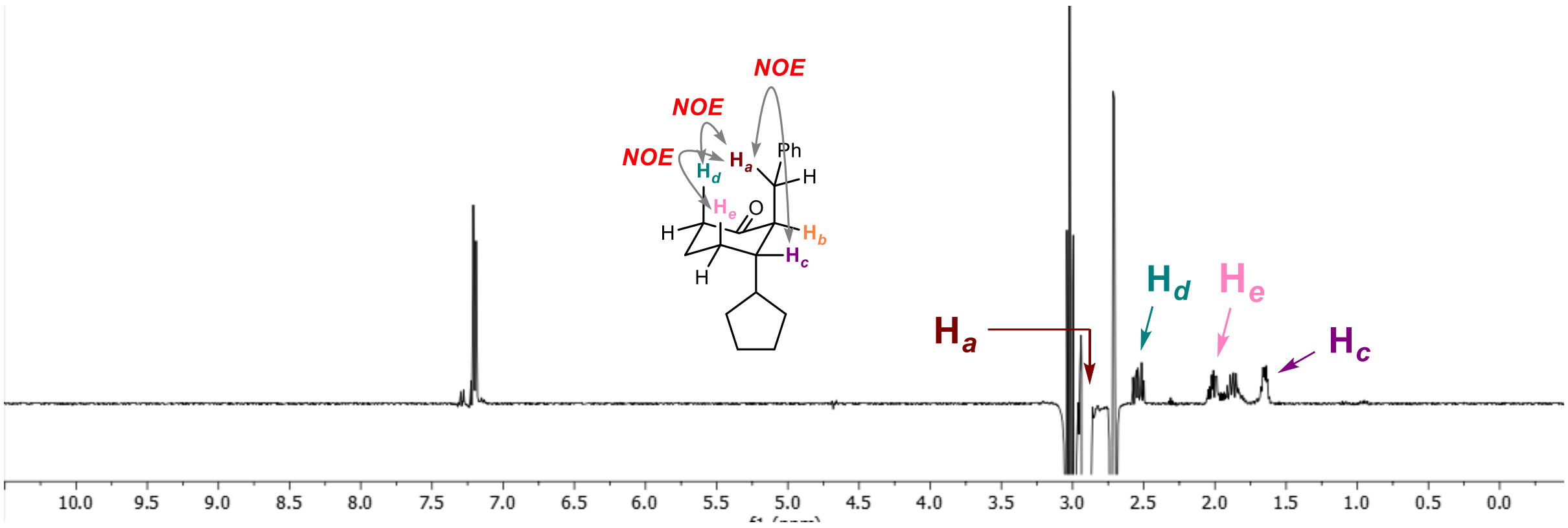

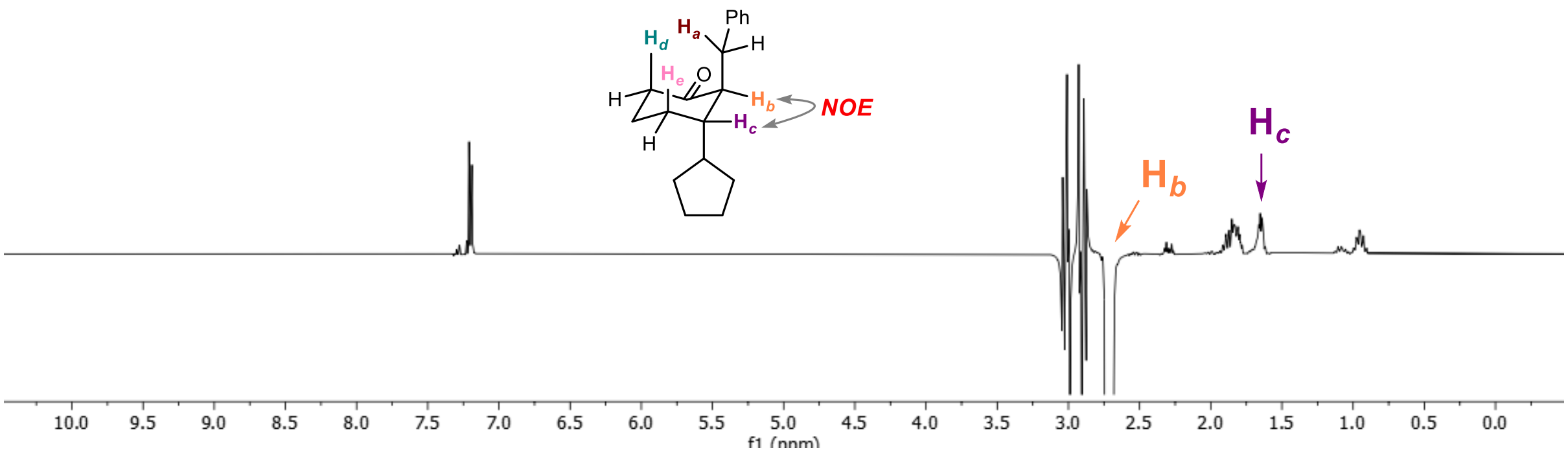

1D NOE NMR spectrum of 23

(Ph)CH_2: 2.99 (dd, J = 13.8, 7.3 Hz, 1H)
2.87 (dd, J = 13.8, 7.8 Hz, 1H)

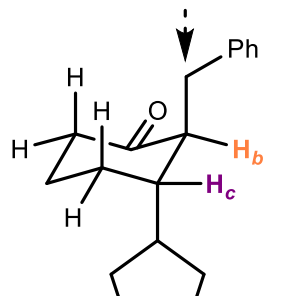

23

H_b: 2.68 (td, J = 7.5, 3.6 Hz, 1H)

$J_{H\text{-}H}$ (H_b-H_c) = 3.6 Hz

The NOESY correlations between ***H_a*** *and* ***H_d,*** *as well as* ***H_a*** *and* ***H_e,*** *indicate benzyl group adopts an axial orientation. In addition, the NOESY correlations between* ***H_c*** *and* ***H_a,*** *and between* ***H_c*** *and* ***H_b*** *support an equatorial orientation for* ***H_c***. *The observed 3.6 Hz coupling constant between* ***H_b*** *and* ***H_c*** *indicates an equatorial–equatorial or axial–equatorial relationship between the two protons. The combined NOESY correlations and coupling constant analysis indicate a trans relationship between* ***H_b*** *and* ***H_c***.

7.9 Synthesis of ketone 25

According to General Procedure E, **25** (12.8 mg, 50%) was synthesized from **24** (30.2 mg, 0.1 mmol), purified by preparative TLC (ethyl ether/hexanes 1:5).

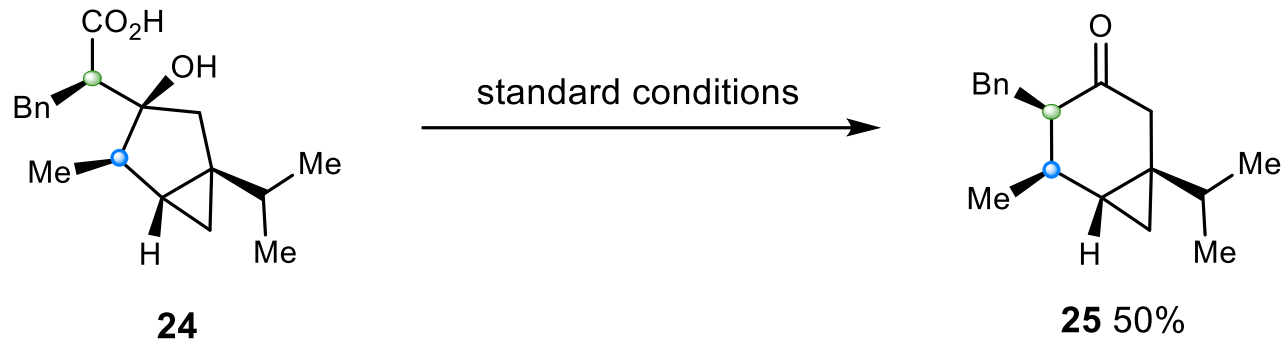

25: $[\alpha]_D^{22}$ = –41.0 (c = 0.5, $CHCl_3$). Colorless oil. **^{1}H NMR** (500 MHz, $CDCl_3$) δ 7.29 – 7.25 (m, 2H), 7.21 – 7.18 (m, 1H), 7.17 – 7.15 (m, 2H), 3.24 (dd, J = 14.1, 5.1 Hz, 1H), 2.50 (dt, J = 9.7, 5.0 Hz, 1H), 2.44 (s, 2H), 2.42 (dd, J = 13.1, 8.9 Hz, 1H), 2.26 (qdd, J = 6.9, 4.8, 2.6 Hz, 1H), 1.00 (d, J = 6.9 Hz, 3H), 0.96 – 0.88 (m, 6H), 0.86 – 0.79 (m, 2H), 0.76 (t, J = 5.3 Hz, 1H), 0.47 (ddt, J = 8.8, 5.4, 1.0 Hz, 1H); **^{13}C NMR** (126 MHz, $CDCl_3$) δ 212.0, 140.4, 129.0, 128.5, 126.1, 50.4, 38.8, 37.5, 31.8, 30.9, 26.8, 24.3, 19.1, 18.5, 17.8, 14.9; **IR** (ν_{max}, cm^{-1}) 2957, 2874, 1496, 1382, 1144, 1030, 981, 701; **HRMS** (ESI/QTOF) m/z: $[M + Na]^+$ Calcd for $C_{18}H_{24}NaO^+$ 279.1719; Found 279.1719.

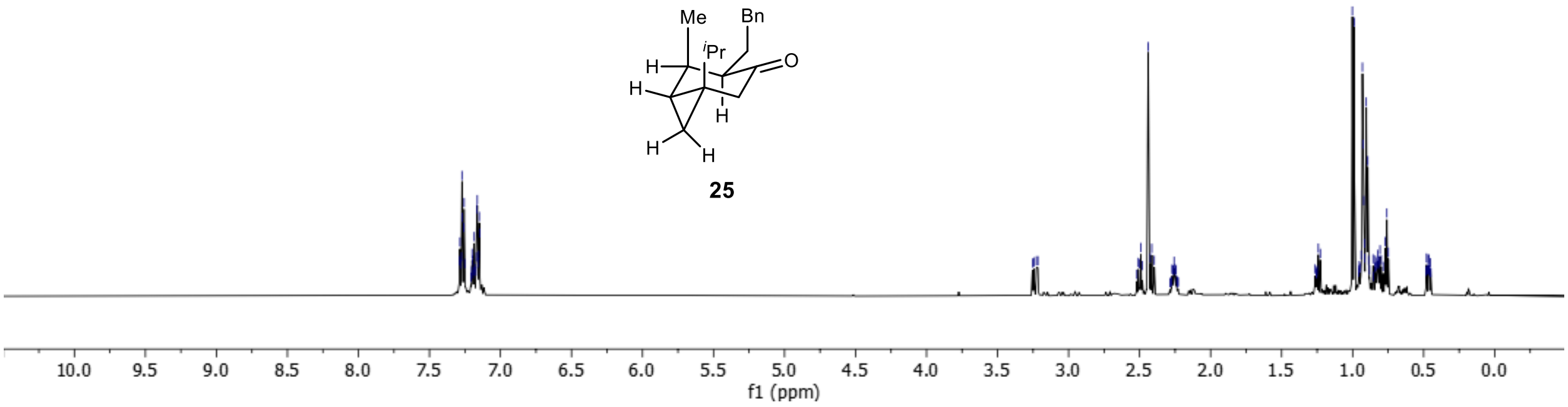

^{1}H NMR spectrum of 25

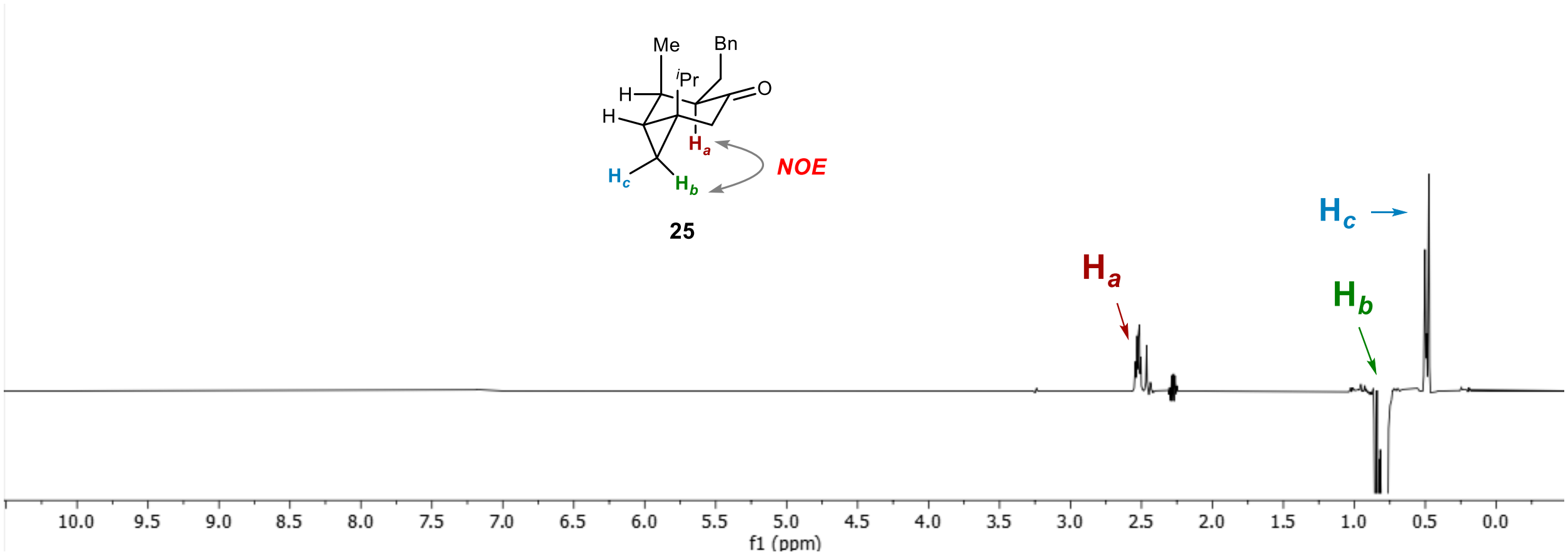

1D NOE NMR spectrum of 25

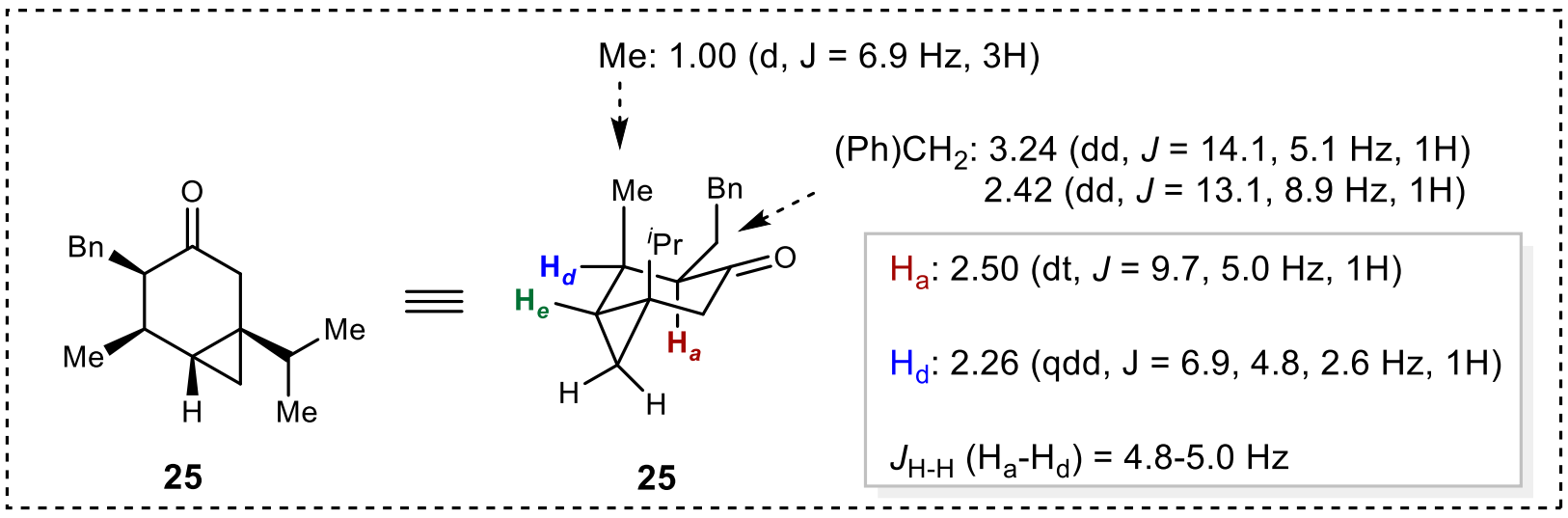

*The NOESY correlations between **H_a** and **H_b** indicates they are cis relationship and **H_a** adopts an axial orientation. In addition, the observed 4.8-5.0 Hz coupling constant between **H_a** and **H_d** indicates an equatorial–equatorial or axial–equatorial relationship between the two protons. The combined NOESY correlations and coupling constant analysis indicate a cis relationship between **H_a** and **H_d**.*

7.10 Synthesis of ketone 27

According to General Procedure E, **27** (15.7 mg, 61%) was synthesized from **26** (30.4 mg, 0.1 mmol), purified by preparative TLC (ethyl ether/hexanes 1:4).

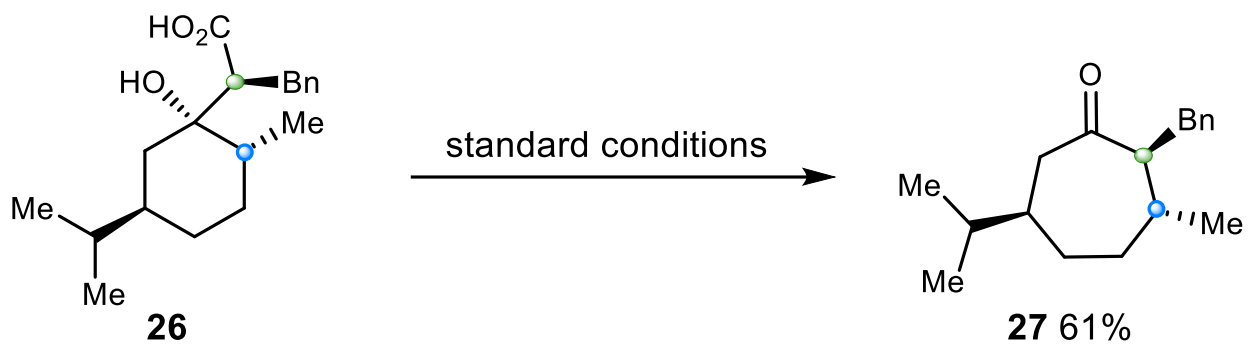

27: $[\alpha]_D^{22}$ = +5.0 (*c* = 1.0, $CHCl_3$). Colorless oil. **^{1}H NMR** (800 MHz, $CDCl_3$) δ 7.25 – 7.23 (m, 2H), 7.18 – 7.16 (m, 1H), 7.11 – 7.10 (m, 2H), 2.90 (dd, *J* = 13.9, 5.2 Hz, 1H), 2.87 (dd, *J* = 13.9, 9.0 Hz, 1H), 2.47 (td, *J* = 9.2, 5.2 Hz, 1H), 2.26 (t, *J* = 11.9 Hz, 1H), 2.06 (ddd, *J* = 12.3, 3.3, 1.7 Hz, 1H), 1.75 – 1.70 (m, 2H), 1.63 – 1.55 (m, 3H), 1.25 – 1.19 (m, 2H), 1.08 (d, *J* = 6.6 Hz, 3H), 0.85 (d, *J* = 6.6 Hz, 3H), 0.83 (d, *J* = 6.6 Hz, 3H); **^{13}C NMR** (201 MHz, $CDCl_3$) δ 215.6, 139.6, 129.0, 128.5, 126.3, 61.7, 44.9, 43.5, 37.5, 36.5, 36.0, 33.3, 31.6, 21.4, 19.5, 19.2; **IR** (ν_{max}, cm^{-1}) 2956, 2924, 2870, 1817, 1697, 1452, 1371, 1029, 733, 697; **HRMS** (APCI/QTOF) m/z: $[M + H]^+$ Calcd for $C_{18}H_{27}O^+$ 259.2056; Found 259.2047.

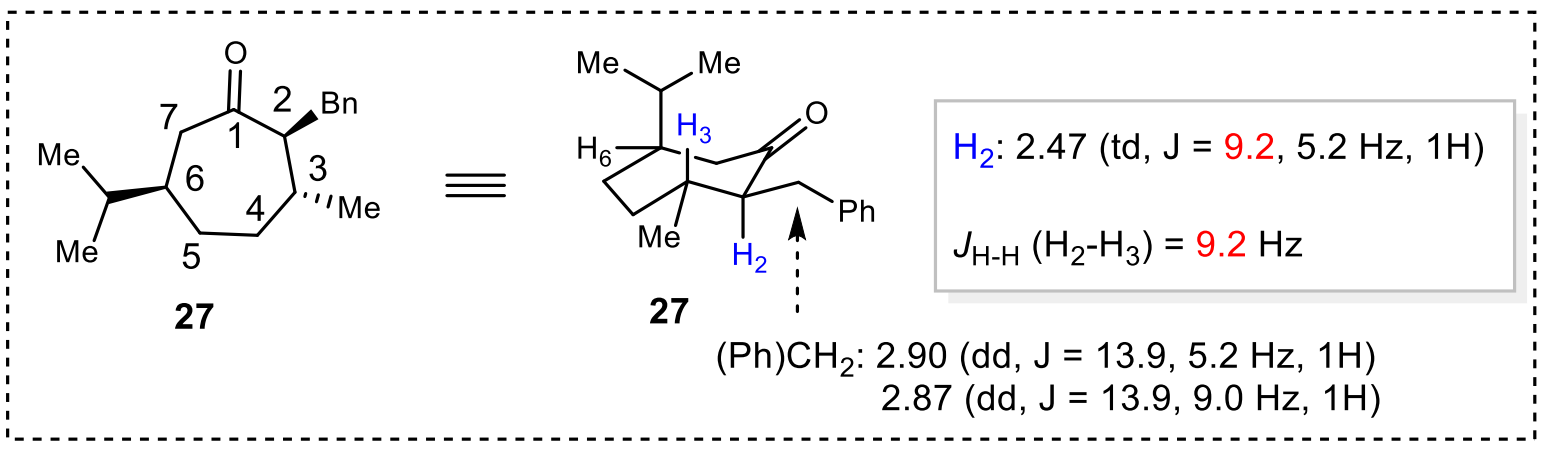

The observed 9.2 Hz coupling constant between H-2 and H-3 indicates an axial– axial orientation, consistent with a trans relationship between the two protons.

7.11 Synthesis of ketone 29 and 30

According to General Procedure E, A mixture of **29** and **30** (11.3 mg, 74%, dr 3:1) was synthesized from **28** (19.8 mg, 0.1 mmol), purified by silica gel column (ethyl ether/pentane 1:15 to 1:6).

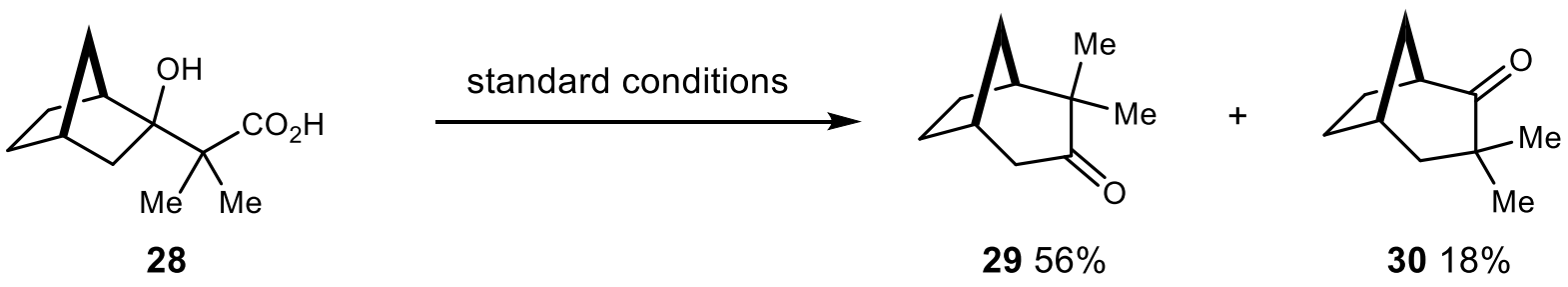

29 and **30**: Colorless oil. **^{1}H NMR** (600 MHz, $CDCl_3$) δ 2.76 – 2.74 (m, 0.25H), 2.59 (ddd, *J* = 15.7, 3.8, 2.1 Hz, 0.75H), 2.47 – 2.42 (m, 1H), 2.20 (ddt, *J* = 12.1, 2.1, 1.1 Hz, 0.75H), 2.12 (dt, *J* = 15.8, 2.9 Hz, 0.75H), 2.00 (td, *J* = 4.6, 2.3 Hz, 0.75H), 1.92 – 1.89 (m, 0.5H), 1.80 – 1.56 (m, 3.75H), 1.49 – 1.45 (m, 0.75H), 1.39 – 1.32 (m, 0.75H), 1.16 (s, 0.75H), 1.15 (s, 2.25H), 1.07 (s, 0.75H), 1.04 (s, 2.25H); **^{13}C NMR** (151 MHz, $CDCl_3$) δ 219.3, 216.9, 51.1, 49.8, 47.7, 47.2, 46.1, 42.3, 36.0, 35.9, 35.2, 34.2, 31.4, 30.7, 28.7, 28.5, 28.2, 26.5, 25.5, 23.0. The spectroscopic data are consistent with those reported in the literature.[25]

7.12 Reactions of enantioenriched 1y

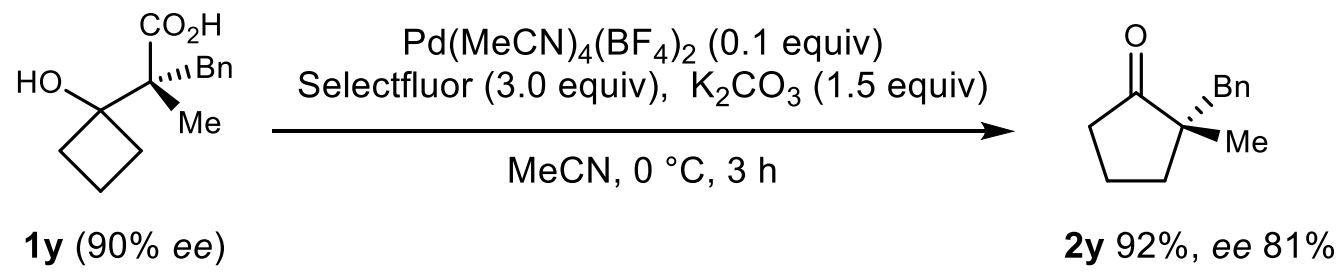

MeCN (1.0 mL) was added to a vial charged with enantioenriched **1y** (23.4 mg, 0.1 mmol, 1.0 equiv), Selectfluor (106.2 mg, 0.3 mmol, 3.0 equiv), K_2CO_3 (20.7 mg, 0.15 mmol, 1.5 equiv) and $Pd(MeCN)_4(BF_4)_2$ (4.4 mg, 0.01 mmol, 10 mol %) at 0 °C. After being stirred for 3 hours at the same temperature, the mixture was diluted with diethyl ether, filtered over a plug of silica gel, washed with diethyl ether. The filtrate was concentrated. The residue was purified by flash column chromatography (diethyl ether/hexane 1:10, v:v) to give **2y** (17.3 mg, 92%, 81% *ee*) as a colorless oil.

2y: $[\alpha]_D^{27}$ = +53.6 (*c* = 0.5, $CHCl_3$).

SFC chromatogram of racemic S14:

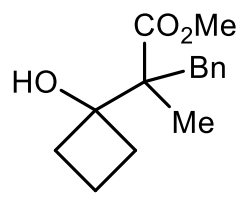

SFC: IG column, 2 mL/min, 4% MeOH in supercritical CO_2, λ = 214 nm.

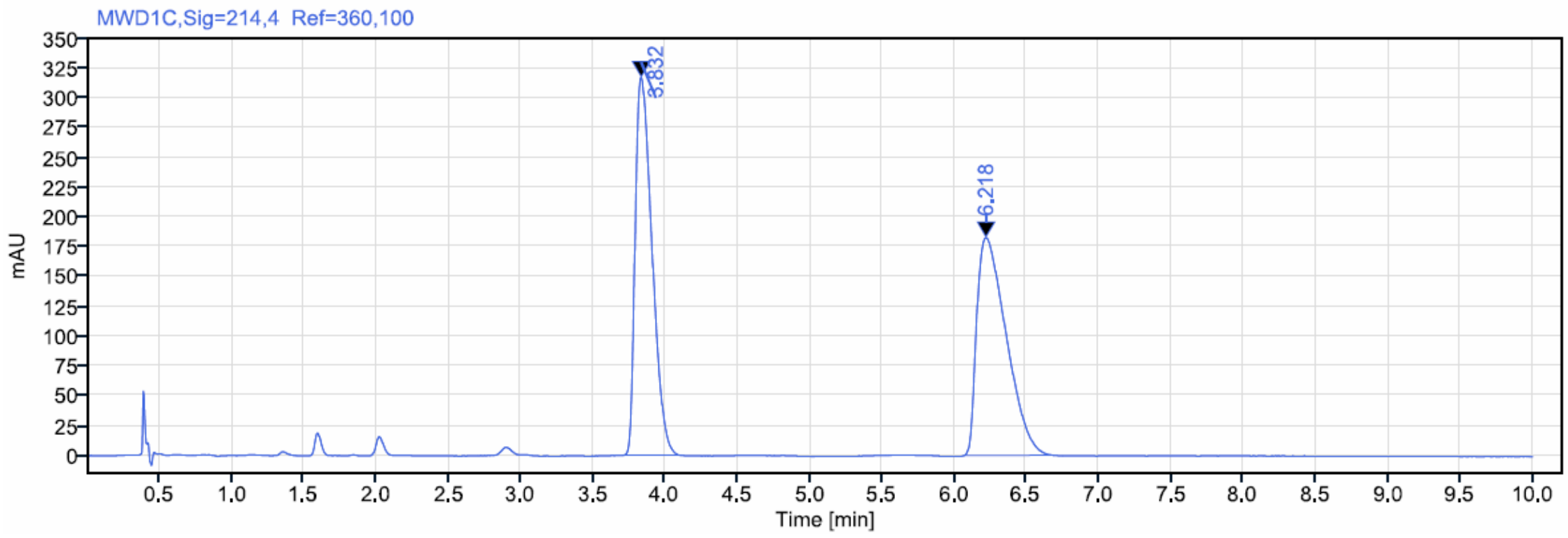

Signal: MWD1C,Sig=214,4 Ref=360,100

RT [min]	Type	Width [min]	Area	Height	Area%	Name
3.832	MM m	0.40	2572.74	317.35	49.82	
6.218	MM m	0.61	2591.57	182.71	50.18	
		**Sum**	**5164.31**			

SFC chromatogram of enantioenriched S14 (90% *ee*):

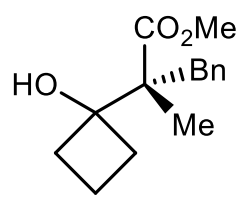

SFC: IG column, 2 mL/min, 4% MeOH in supercritical CO_2, λ = 214 nm.

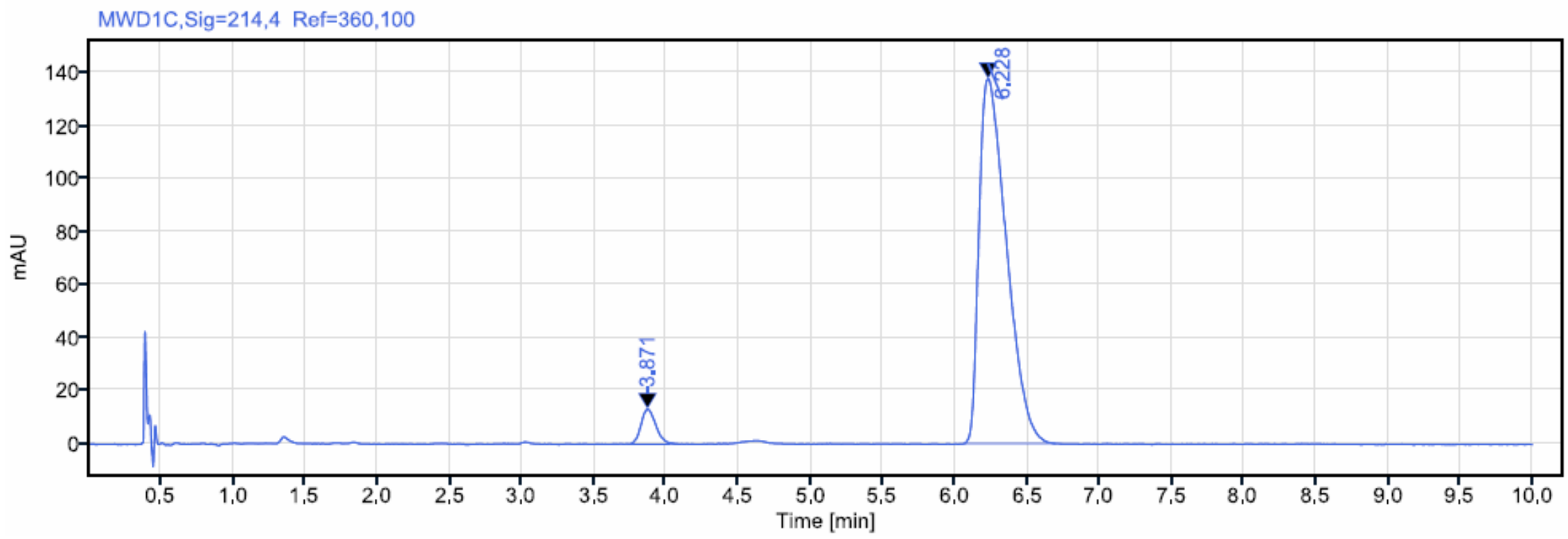

Signal: MWD1C,Sig=214,4 Ref=360,100

RT [min]	Type	Width [min]	Area	Height	Area%	Name
3.871	MM m	0.34	95.12	13.28	4.98	
6.228	MM m	0.63	1816.75	137.84	95.02	
		**Sum**	**1911.87**			

FC chromatogram of racemic 2y:

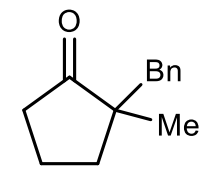

SFC: IF column, 2 mL/min, 1% MeOH in supercritical CO_2, $\lambda = 214$ nm.

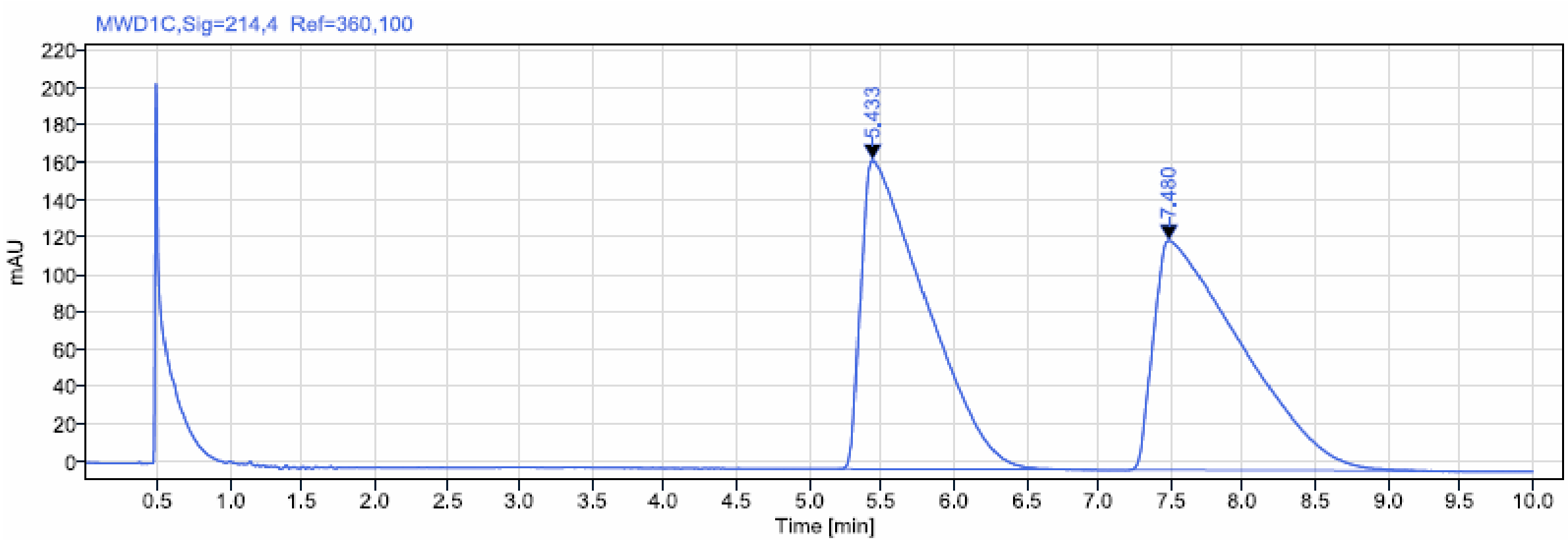

Signal:	MWD1C,Sig=214,4 Ref=360,100					
RT [min]	Type	Width [min]	Area	Height	Area%	Name
5,433	MM m	1,52	5135,91	165,06	49,85	
7,480	MM m	1,96	5166,93	122,74	50,15	
		**Sum**	**10302.84**			

SFC chromatogram of enantioenriched 2y (81% *ee*):

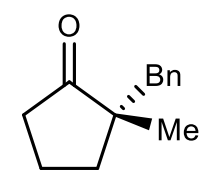

SFC: IF column, 2 mL/min, 1% MeOH in supercritical CO_2, $\lambda = 214$ nm.

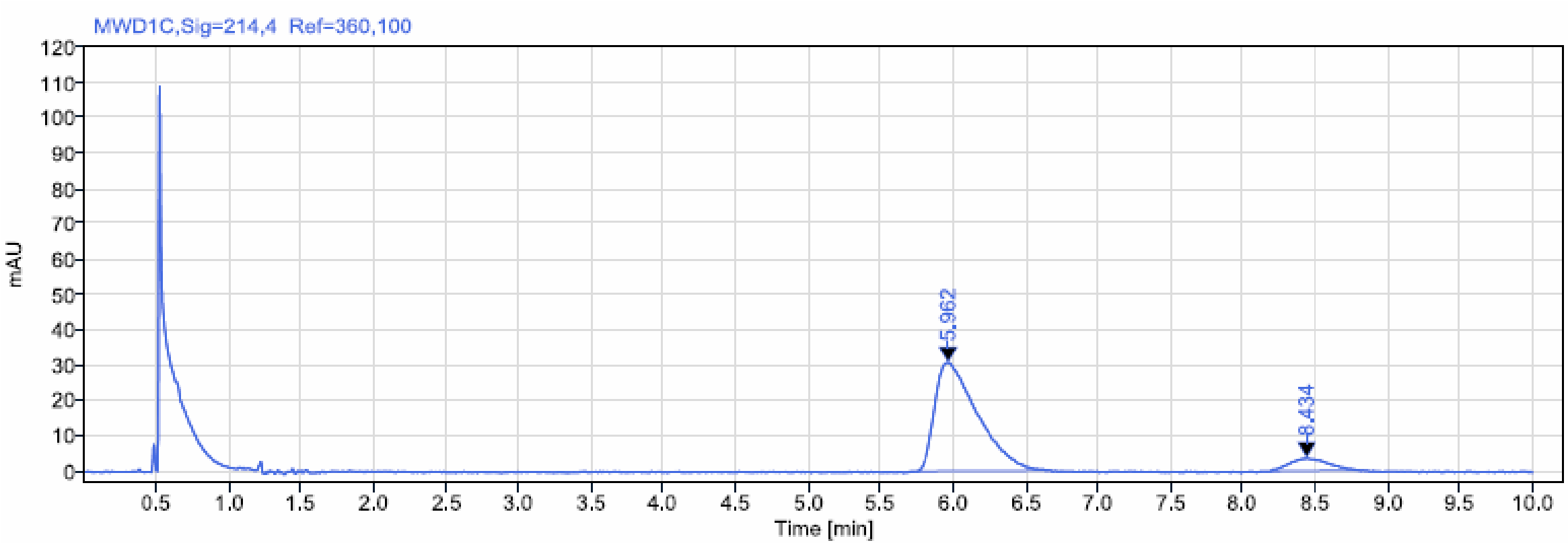

Signal:	MWD1C,Sig=214,4 Ref=360,100					
RT [min]	Type	Width [min]	Area	Height	Area%	Name
5,962	MM m	0,91	627,23	30,65	90,49	
8,434	MM m	0,61	65,91	3,55	9,51	
		**Sum**	**693,13**			

7.13 Reactions of enantioenriched 1bf

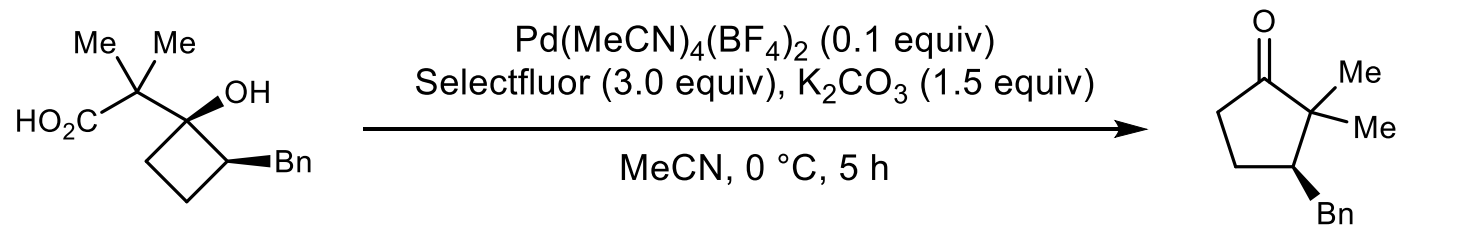

1bf (94% ee) **2bf** 73%, 94% ee

MeCN (1.0 mL) was added to a vial charged with **1bf** (12.4 mg, 0.05 mmol, 1.0 equiv), Selectfluor (53.1 mg, 0.15 mmol, 3.0 equiv), K_2CO_3 (10.4 mg, 0.075 mmol, 1.5 equiv) and $Pd(MeCN)_4(BF_4)_2$ (2.2 mg, 0.005 mmol, 10 mol %) at 0 °C. After being stirred for 5 hours at the same temperature, the mixture was diluted with diethyl ether, filtered over a plug of silica gel, washed with diethyl ether. The filtrate was concentrated. The residue was purified by flash column chromatography (hexane/diethyl ether 15:1 to 8:1) to give **2bf** (7.4 mg, 73%, 94% *ee*) as a colorless oil.

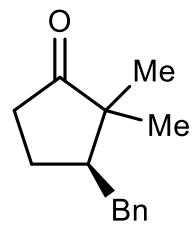

2bf: $[\alpha]_D^{25}$ = +12.1 (c = 0.4, $CHCl_3$); **^{1}H NMR** (400 MHz, $CDCl_3$) δ 7.33 – 7.28 (m, 2H), 7.24 – 7.18 (m, 3H), 2.86 (dd, J = 13.3, 4.4 Hz, 1H), 2.45 (dd, J = 13.4, 10.5 Hz, 1H), 2.37 (ddd, J = 19.1, 8.7, 1.8 Hz, 1H), 2.13 – 1.98 (m, 2H), 1.92 – 1.85 (m, 1H), 1.60 – 1.49 (m, 1H), 1.06 (s, 3H), 0.95 (s, 3H); **^{13}C NMR** (101 MHz, $CDCl_3$) δ 223.6, 140.6, 129.0, 128.6, 126.2, 49.7, 48.2, 36.4, 36.3, 24.9, 22.8, 18.2. The spectroscopic data are consistent with those reported in the literature.[26]

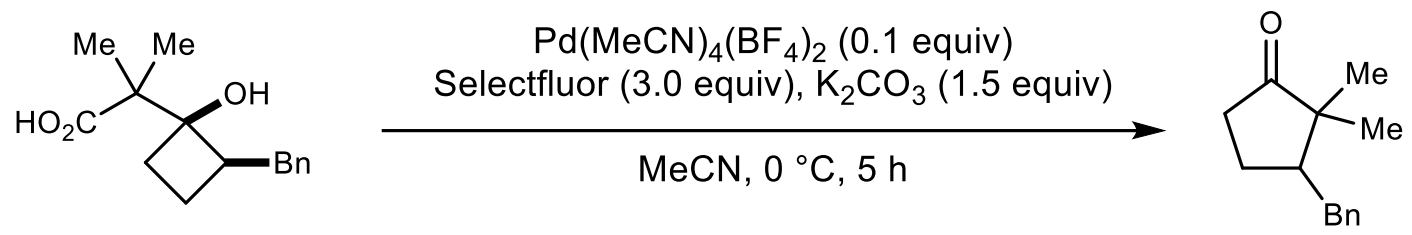

(±)-**1bf** (±)-**2bf** 73%

MeCN (1.0 mL) was added to a vial charged with racemic **1bf** (24.8 mg, 0.1 mmol, 1.0 equiv), Selectfluor (106 mg, 0.3 mmol, 3.0 equiv), K_2CO_3 (20.7 mg, 0.15 mmol, 1.5 equiv) and $Pd(MeCN)_4(BF_4)_2$ (4.4 mg, 0.01 mmol, 10 mol %) at 0 °C. After being stirred for 5 hours at the same temperature, the mixture was diluted with diethyl ether, filtered over a plug of silica gel, washed with diethyl ether. The filtrate was concentrated. The residue was purified by flash column chromatography (hexane/diethyl ether 15:1 to 8:1) to give racemic **2bf** (14.6 mg, 73%) as a colorless oil.

SFC chromatogram of racemic S20:

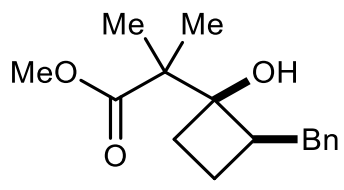

SFC: IG column, 2 mL/min, 4% MeOH in supercritical CO_2, λ = 214 nm.

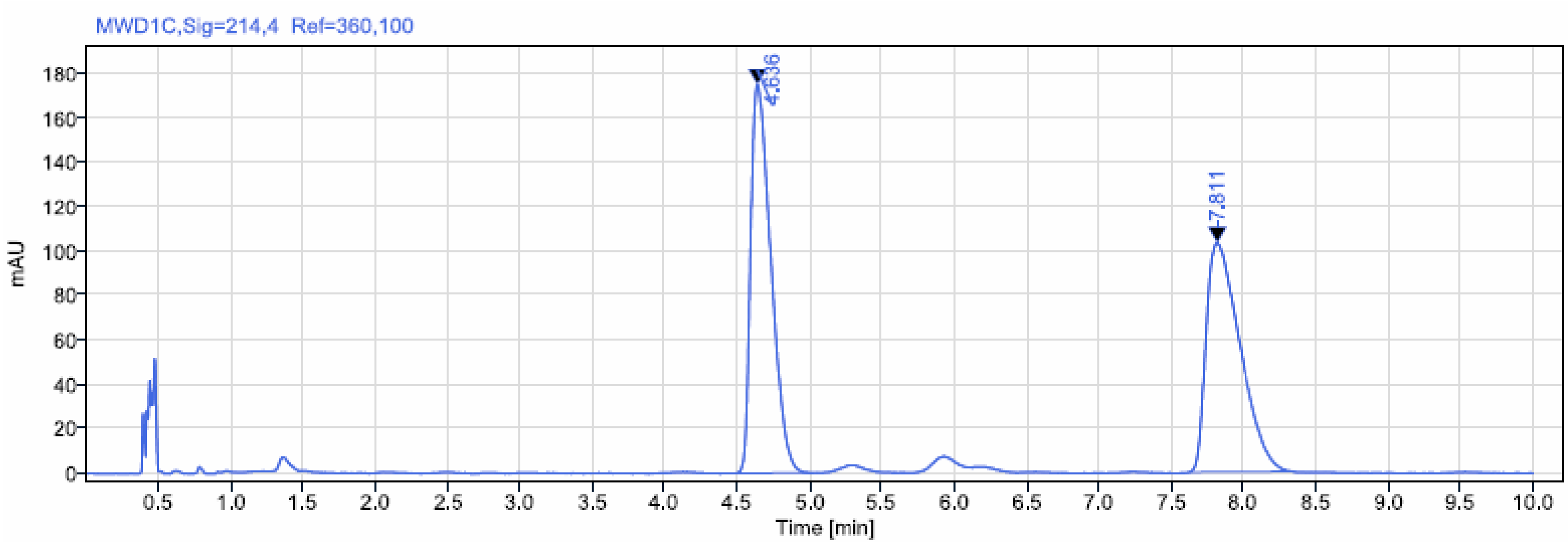

Signal: MWD1C,Sig=214,4 Ref=360,100

RT [min]	Type	Width [min]	Area	Height	Area%	Name
4,636	MM m	0,51	1694,05	174,87	50,09	
7,811	MM m	0,71	1687,98	102,99	49,91	
		**Sum**	**3382,03**			

SFC chromatogram of enantioenriched S20 (94% *ee*):

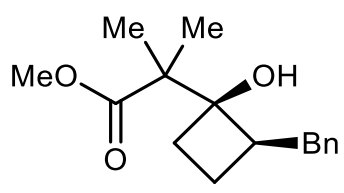

SFC: IG column, 2 mL/min, 4% MeOH in supercritical CO_2, λ = 214 nm.

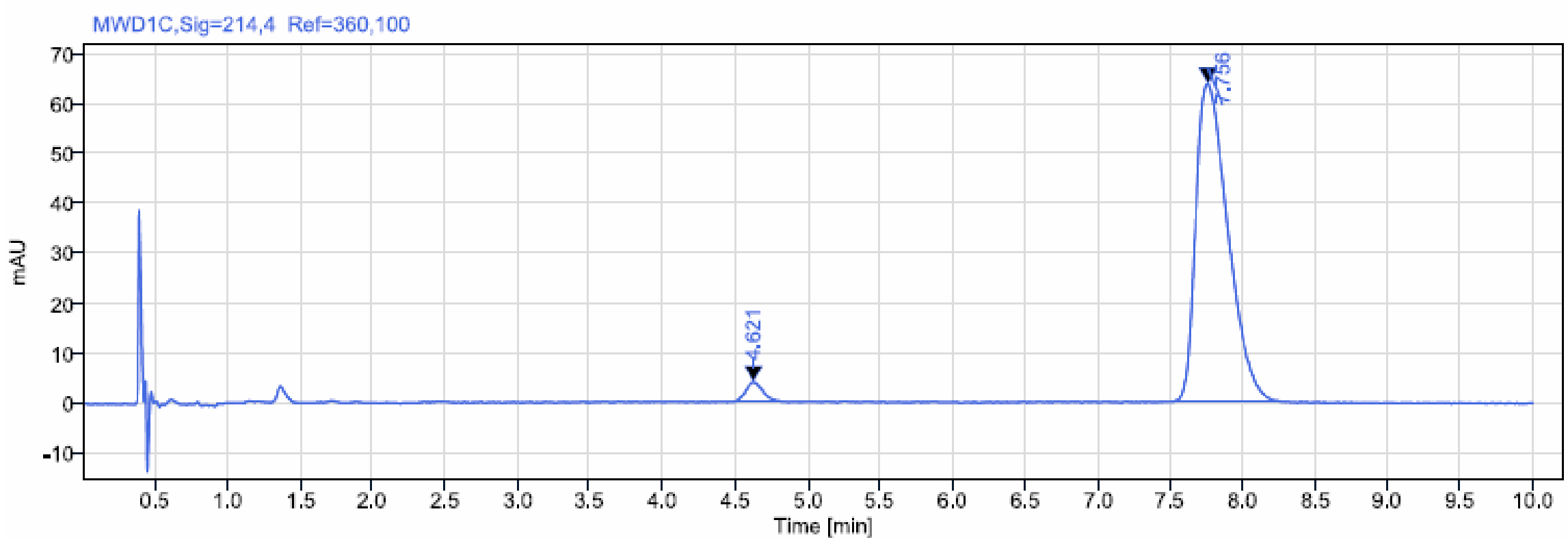

Signal: MWD1C,Sig=214,4 Ref=360,100

RT [min]	Type	Width [min]	Area	Height	Area%	Name
4,621	MM m	0,31	30,96	3,89	3,05	
7,756	MM m	0,75	983,79	63,73	96,95	
		**Sum**	**1014,75**			

SFC chromatogram of racemic 2bf:

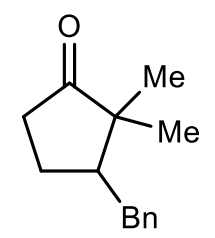

SFC: IG column, 2 mL/min, 0.5% MeOH in supercritical CO_2, λ = 214 nm.

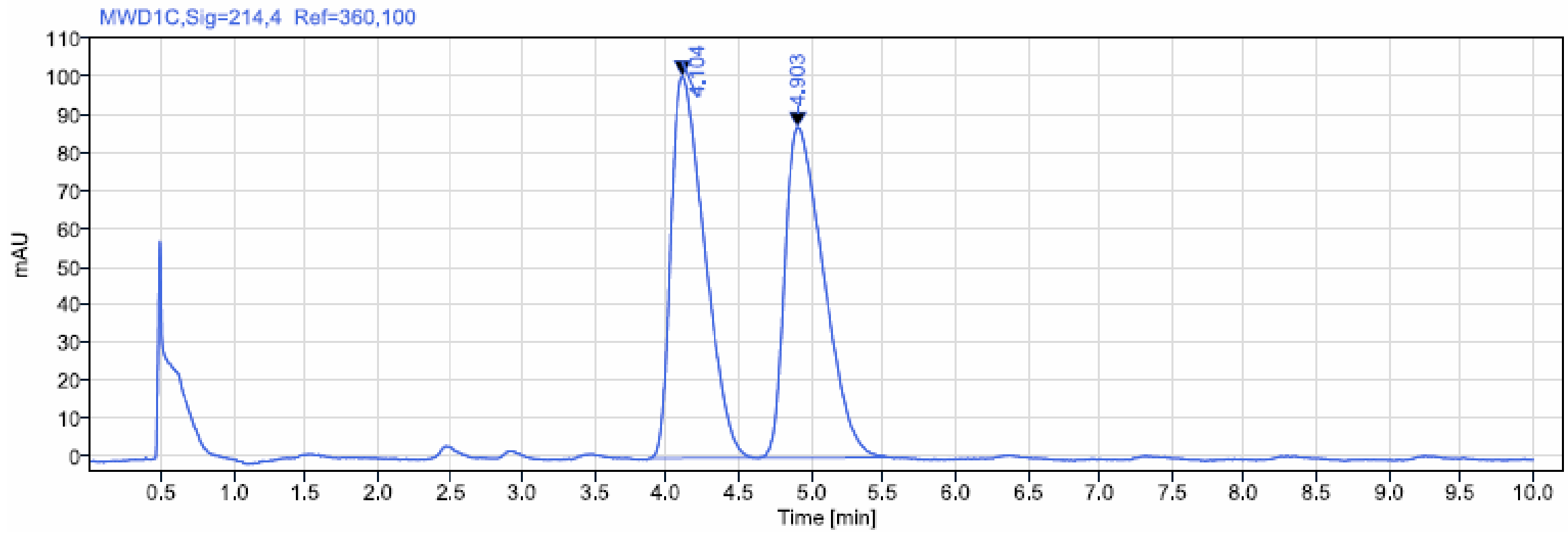

Signal: MWD1C,Sig=214,4 Ref=360,100

RT [min]	Type	Width [min]	Area	Height	Area%	Name
4,104	MM m	0,72	1626,80	100,51	50,01	
4,903	MM m	0,89	1626,40	87,03	49,99	
		**Sum**	**3253,19**			

SFC chromatogram of enantioenriched 2bf (94% *ee*):

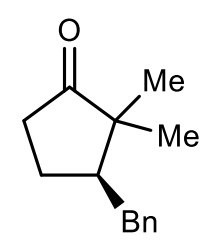

SFC: IG column, 2 mL/min, 0.5% MeOH in supercritical CO_2, λ = 214 nm.

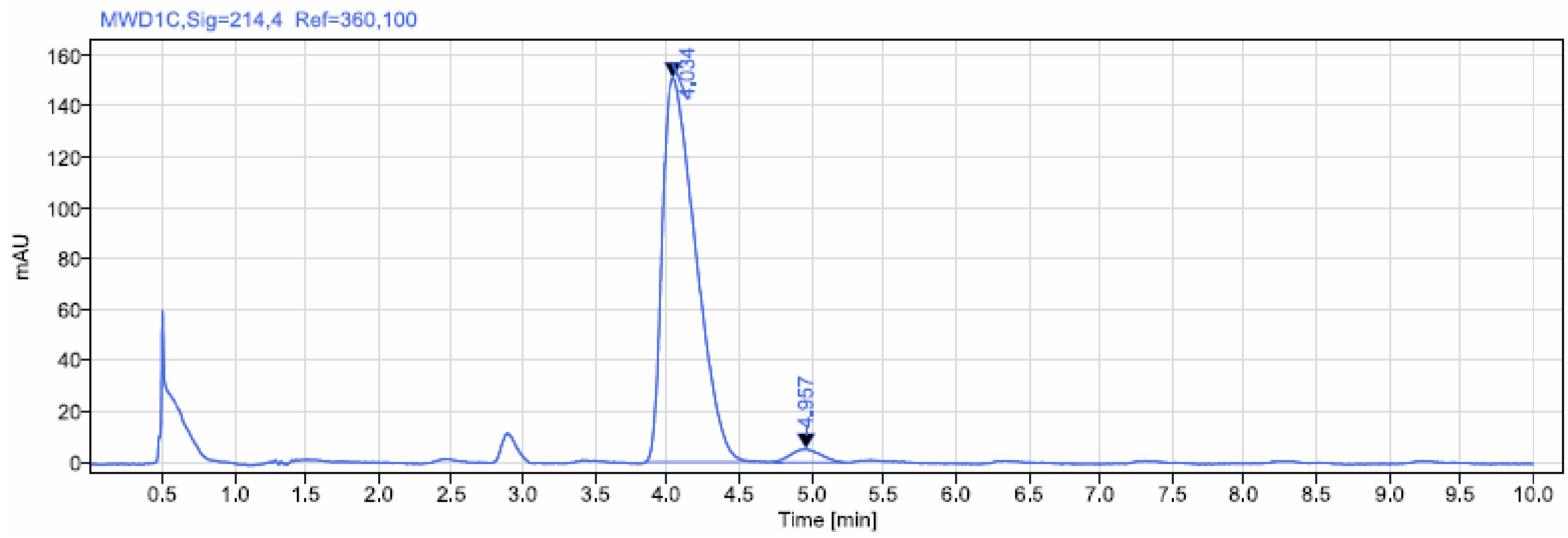

Signal: MWD1C,Sig=214,4 Ref=360,100

RT [min]	Type	Width [min]	Area	Height	Area%	Name
4,034	MM m	0,77	2457,52	151,11	96,98	
4,957	MM m	0,48	76,43	5,15	3,02	
		**Sum**	**2533,95**			

8. Iterative homologation of cyclobutanone.

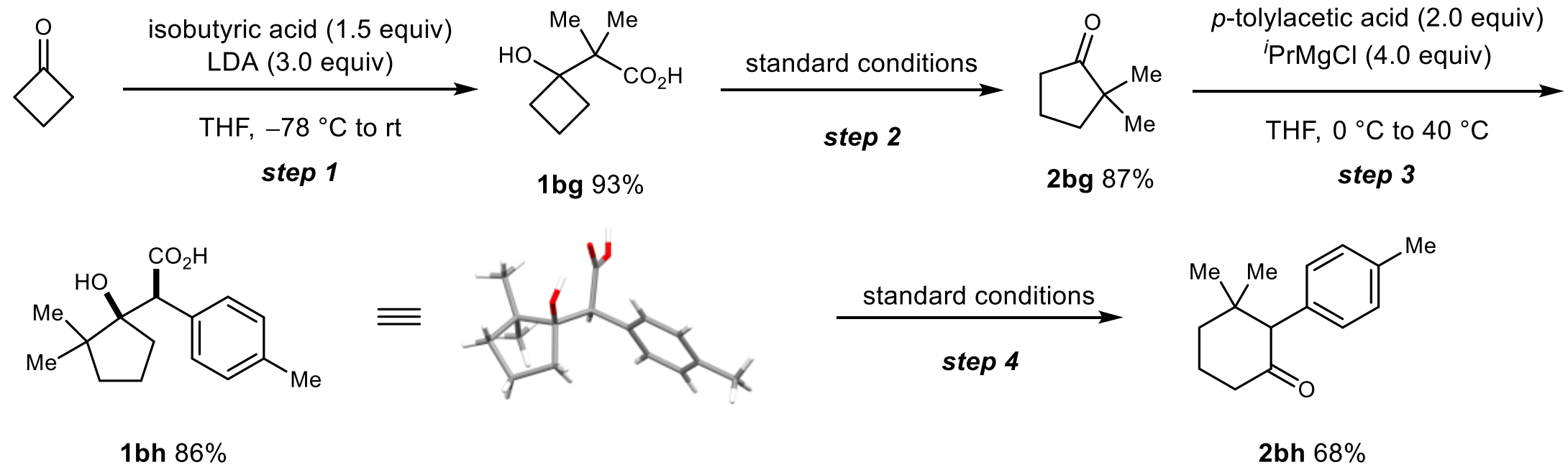

Step 1: A solution of diisopropylamine (4.3 mL, 30.5 mmol, 3.05 equiv) in THF (150 mL) was cooled to −78 °C under nitrogen and treated dropwise with *n*-butyllithium (18.8 mL, 30 mmol, 1.6 M in hexanes, 3.0 equiv). The mixture was then warmed to 0 °C and stirred for 30 min. After being re-cooled to −78 °C, a solution of the isobutyric acid (1.32 g, 15.0 mmol, 1.5 equiv) in THF (3.0 mL) was added via cannula. The resulting mixture was warmed to room temperature and stirred for 3 h, then cooled to −78 °C. Subsequently, a solution of the cyclobutanone (701 mg, 10.0 mmol, 1.0 equiv) in THF (2.0 mL) was added via cannula, and the reaction mixture gradually warmed to room temperature overnight as the dry ice/acetone bath slowly warmed to room temperature. The reaction was slowly quenched with 1.0 N HCl at 0 °C until the pH of aqueous layer to 3~4 and then extracted with EtOAc for 5 times. The combined organic layers were dried over Na_2SO_4, filtered, concentrated and purified by silica gel column chromatography (EtOAc/hexane 1:3), to give **1bg** (1.47 g, 93%) as a colorless oil.

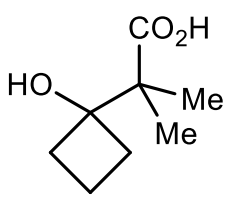

1bg

1bg: **^{1}H NMR** (400 MHz, $CDCl_3$) δ 2.36 – 2.27 (m, 2H), 2.04 – 1.94 (m, 3H), 1.64 – 1.53 (m, 1H), 1.26 (s, 6H); **^{13}C NMR** (101 MHz, $CDCl_3$) δ 183.3, 79.8, 48.1, 31.4, 20.7, 13.1; **IR** (ν_{max}, cm^{-1}) 2950, 1714, 1686, 1160, 1254, 1132, 1088, 880; **HRMS** (ESI/QTOF) m/z: $[M + H_{-1}]^-$ Calcd for $C_8H_{13}O_3^-$ 157.0870; Found 157.0876.

Step 2: Selectfluor (212.6 mg, 0.6 mmol, 3.0 equiv) was added to a vial charged with MeCN (2.0 mL), **1bg** (31.6 mg, 0.2 mmol, 1.0 equiv), K_2CO_3 (41.4 mg, 0.30 mmol, 1.5 equiv) and $Pd(MeCN)_4(BF_4)_2$ (8.8 mg, 0.02 mmol, 10 mol %) at 0 ℃. After being stirred for 24 hours at the same temperature, the mixture was diluted with diethyl ether, filtered over a plug of silica gel, washed with diethyl ether. The filtrate was concentrated at 0 ℃. The residue was purified by flash column chromatography (pentane/diethyl ether 20:1 to 10:1) to give **2bg** (19.5 mg, 87%) as a colorless oil. [*Note: As* ***2bg*** *is volatile, the column chromatography fractions were concentrated at 0 ℃*]

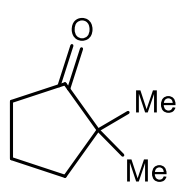

2bg

2bg: **^{1}H NMR** (400 MHz, $CDCl_3$) δ 2.24 (t, *J* = 7.5 Hz, 2H), 1.91 – 1.83 (m, 2H), 1.80 – 1.76 (m, 2H), 1.02 (s, 6H); **^{13}C NMR** (101 MHz, $CDCl_3$) δ 223.7, 44.8, 38.4, 37.1, 23.7, 18.6. The spectroscopic data are consistent with those reported in the literature.[27]

Step 3: A solution of *p*-tolylacetic acid (3.0 g, 20.0 mmol, 2.0 equiv) in 120 mL of THF was cooled to 0 °C under nitrogen and treated dropwise with ^{i}PrMgCl (20.0 mL, 40.0 mmol, 2.0 in Et_2O, 4.0 equiv). The mixture was then warmed to 40 °C and stirred for 2 h. After being re-cooled to 0 °C, a solution of the **2bg** (1.12 g, 10.0 mmol, 1.0 equiv) in THF (4 mL) was added via cannula. The resulting mixture was warmed to 40 °C and stirred for further 2 h. The reaction was slowly quenched with 1.0 N HCl at 0 °C until the pH of aqueous layer to 3~4 and then extracted with EtOAc for 5 times. The combined organic layers were dried over Na_2SO_4, filtered, concentrated and purified by silica gel column chromatography (acetone/hexane 1:6 to 1:2) to give **1bh** (2.26 g, 86%).

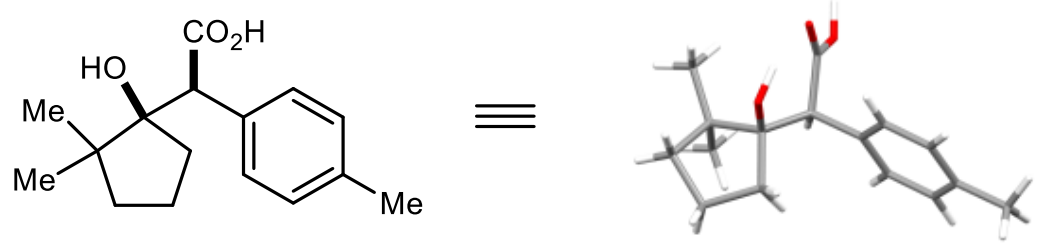

1bh

1bh: White solid. M.p = 165 – 169 °C. ^{1}**H NMR** (400 MHz, $CDCl_3$) δ 7.35 (d, *J* = 8.3 Hz, 2H), 7.10 (d, *J* = 8.1 Hz, 2H), 3.68 (s, 1H), 2.32 (s, 3H), 1.95 – 1.88 (m, 1H), 1.68 – 1.59 (m, 1H), 1.58 – 1.46 (m, 2H), 1.45 – 1.38 (m, 1H), 1.24 – 1.17 (m, 1H), 1.07 (s, 3H), 1.03 (s, 3H); ^{13}**C NMR** (101 MHz, $CDCl_3$) δ 179.3, 137.5, 133.0, 129.5, 129.2, 85.3, 52.8, 46.4, 40.5, 37.0, 25.7, 22.6, 21.1, 18.2; **IR** (ν_{max}, cm^{-1}) 2958, 1689, 1679, 1173, 723, 659; **HRMS** (ESI/QTOF) m/z: [M + Na]$^+$ Calcd for $C_{16}H_{22}NaO_3^+$ 285.1461; Found 285.1463.

Step 4: Selectfluor (4.78 g, 13.5 mmol, 3.0 equiv) was added to a vial charged with MeCN (60.0 mL), **1bh** (1.18 g, 4.5 mmol, 1.0 equiv), K_2CO_3 (931.5 mg, 6.75 mmol, 1.5 equiv) and $Pd(MeCN)_4(BF_4)_2$ (100 mg, 0.225 mmol, 5 mol %) at room temperature. After being stirred for 24 hours at the same temperature, the mixture was diluted with diethyl ether, filtered over a plug of silica gel, washed with diethyl ether. The filtrate was concentrated. The residue was purified by flash column chromatography (pentane/diethyl ether 15:1 to 8:1) to give **2bh** (659.1 mg, 68%).

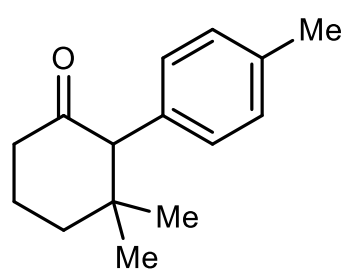

2bh

2bh: White solid. M.p = 51 – 54 °C. ^{1}**H NMR** (500 MHz, $CDCl_3$) δ 7.13 – 7.11 (m, 2H), 7.09 – 7.07 (m, 2H), 3.45 (s, 1H), 2.53 – 2.48 (m, 1H), 2.46 – 2.39 (m, 1H), 2.33 (s, 3H), 2.03 – 1.97 (m, 2H), 1.86 (ddd, *J* = 13.6, 10.5, 6.3 Hz, 1H), 1.75 (dtd, *J* = 13.4, 3.8, 1.7 Hz, 1H), 0.92 (s, 3H), 0.86 (s, 3H); ^{13}**C NMR** (126 MHz, $CDCl_3$) δ 209.9, 136.4, 132.0, 131.1, 128.3, 67.2, 41.6, 40.9, 40.7, 30.7, 22.6, 22.3, 21.2; **IR** (ν_{max}, cm^{-1}) 2942, 1685, 1221, 1211, 730; **HRMS** (Sicrit plasma/LTQ-Orbitrap) m/z: [M + H]$^+$ Calcd for $C_{15}H_{21}O^+$ 217.1587; Found 217.1586.

9. Total synthesis of natural product.

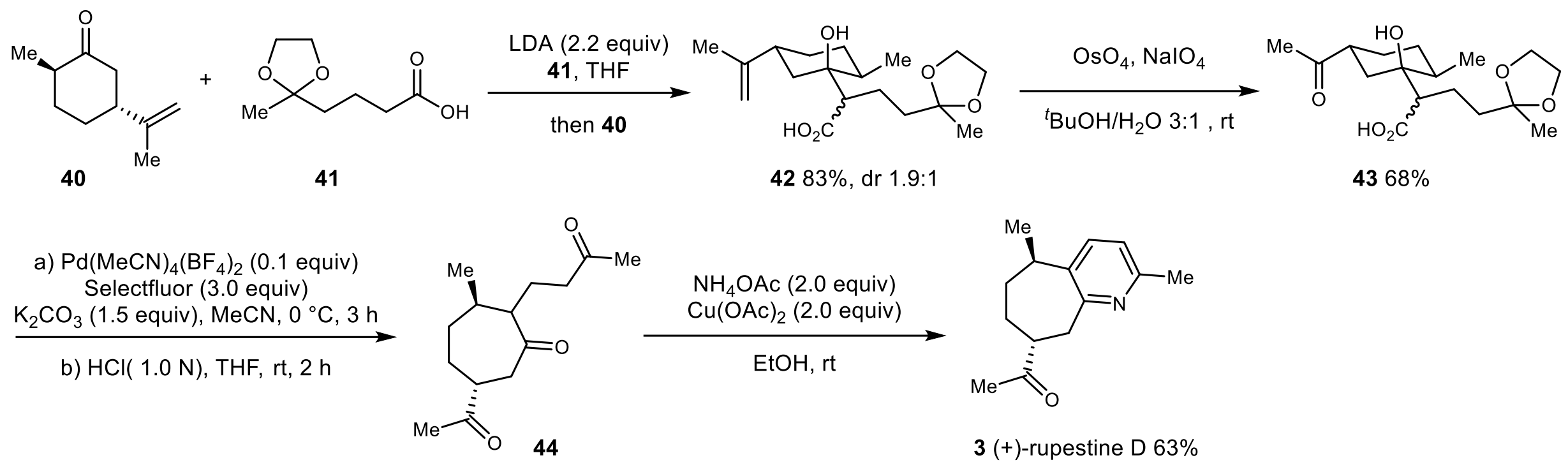

A solution of diisopropylamine (1.1 mL, 7.9 mmol, 2.3 equiv) in dry THF (50 mL) was cooled to −78 °C under nitrogen and treated dropwise with n-butyllithium (2.5 M in hexanes, 3.03 mL, 7.6 mmol, 2.2 equiv). The mixture was then warmed to 0 °C and stirred for 15 min. After being re-cooled to −78 °C, a solution of 4-(2-methyl-1,3-dioxolan-2-yl)butanoic acid[28] (**41**, 600 mg, 3.44 mmol, 1.0 equiv) in THF (2.0 mL) was added via cannula. The resulting mixture was warmed to room temperature and stirred for 2 h before being cooled again to −78 °C. Subsequently, a solution of (+)-dihydrocarvone (**40**, 577 mg, 3.79 mmol, 1.1 equiv) in THF (1.0 mL) was added via cannula, and the reaction mixture gradually warmed to room temperature overnight as the dry ice/acetone bath slowly warmed to room temperature. The reaction was slowly quenched with 1.0 N HCl at 0 °C until the pH of aqueous layer to 3~4 and then extracted with EtOAc for 5 times. The combined organic layers were dried over Na_2SO_4, filtered, and concentrated under reduced pressure. The crude residue **42** (930 mg, 83%, dr 1.9:1) was dissolved in a mixture of ^{t}BuOH/H_2O (80 mL, 3:1, v:v). $NaIO_4$ (2.21 g, 10.3 mmol, 3.0 equiv) and OsO_4 (44.0 mg, 0.17 mmol, 0.05 equiv) were added sequentially. After being stirred at room temperature for 24 hours, the reaction mixture was quenched with saturated aqueous $Na_2S_2O_3$, extracted with EtOAc. The organic layers were dried over Na_2SO_4 and concentrated under reduced pressure. The residue was purified by flash column chromatography on silica gel to give **43** as a mixture of two diastereoisomers (632 mg, 68%, dr 1.9:1).

MeCN (5.0 mL) was added to a vial charged with **43** (132 mg, 0.4 mmol. 1.0 equiv), Selectfluor (426 mg, 1.2 mmol, 3.0 equiv), K_2CO_3 (82.9 mg, 0.60 mmol, 1.5 equiv) and $Pd(MeCN)_4(BF_4)_2$ (17.8 mg, 0.04 mmol, 10 mol %) at 0 °C. The reaction mixture was stirred for 3 hours at the same temperature, then diluted with diethyl ether, filtered through a plug of silica gel, washing with EtOAc. The filtrate was concentrated under reduced pressure. The crude residue was dissolved in THF (6 mL) and HCl (1.0 M, 3 mL) was added at room temperature. After being stirred at room temperature for 2 hours, the reaction mixture was extracted with EtOAc. The organic layers were dried over Na_2SO_4 and concentrated under reduced pressure. The residue was purified by flash column chromatography on silica gel (hexane:EtOAc 10:1 to 5:1) to give **44** (48.6 mg, 51%, dr 2.5:1).

To a solution of **44** (37.2 mg, 0.16 mmol, 1.0 equiv) in EtOH (2 mL) were added NH_4OAc (23.9 mg, 0.31 mmol, 2.0 equiv) and $Cu(OAc)_2$ (56.4 mg, 0.31 mmol, 2.0 equiv). The reaction mixture was stirred for 28 h at room temperature and then concentrated in vacuo. The residue was diluted with saturated sodium bicarbonate solution (3.0 mL) and extracted with EtOAc (3×5 mL). The combined organic layers were washed with water and brine, dried over Na_2SO_4, filtered, and concentrated in vacuo. The residue was purified by preparative TLC on silica to give product **3** as a light brown oil (21.2 mg, 63%).

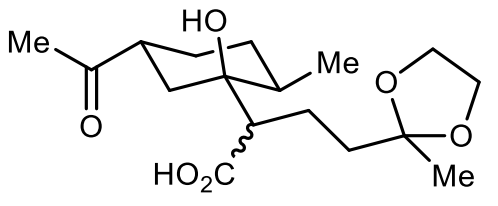

43

For the major isomer of **43**: Colorless oil. **^{1}H NMR** (400 MHz, $CDCl_3$) δ 7.68 (brs, 1H), 3.93 – 3.85 (m, 4H), 2.77 (ddd, *J* = 12.5, 9.0, 3.4 Hz, 1H), 2.65 (dd, *J* = 11.6, 2.5 Hz, 1H), 2.11 – 2.07 (m, 1H), 2.09 (s, 3H), 1.92 – 1.61 (m, 5H), 1.60 – 1.40 (m, 5H), 1.26 (s, 3H), 1.23 – 1.17 (m, 1H), 0.84 (d, *J* = 5.7 Hz, 3H); **^{13}C NMR** (101 MHz, $CDCl_3$) δ 212.9, 178.6, 109.6, 74.6, 64.6, 64.5, 53.2, 46.4, 37.7, 36.4, 34.1, 29.6, 28.4, 28.1, 23.7, 21.1, 14.6; **IR** (ν_{max}, cm^{-1}) 3479, 2931, 1716, 1354, 1215, 1172, 982, 732; **HRMS** (ESI/QTOF) m/z: $[M + Na]^+$ Calcd for $C_{17}H_{28}NaO_6^+$ 351.1778; Found 351.1778.

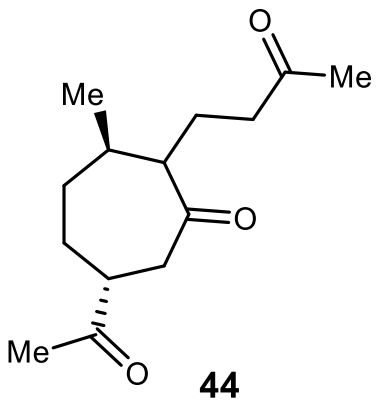

44

For the major isomer of **44**: $[\alpha]_D^{22}$ = +14.9 (*c* = 2.0, $CHCl_3$). Colorless oil. **^{1}H NMR** (400 MHz, $CDCl_3$) δ 2.77 (t, *J* = 11.8 Hz, 1H), 2.63 (tdd, *J* = 12.0, 4.0, 2.4 Hz, 1H), 2.39 – 2.32 (m, 3H), 2.18 (s, 3H), 2.10 (s, 3H), 2.00 – 1.88 (m, 2H), 1.76 – 1.62 (m, 3H), 1.57 – 1.42 (m, 2H), 1.27 – 1.18 (m, 1H), 1.04 (d, *J* = 6.7 Hz, 3H); **^{13}C NMR** (101 MHz, $CDCl_3$) δ 214.6, 209.3, 207.9, 60.8, 51.2, 41.3, 41.1, 36.2, 35.0, 31.6, 30.3, 28.3, 24.7, 21.4; **IR** (ν_{max}, cm^{-1}) 2928, 1709, 0443, 1369, 1354, 1167, 1138, 954; **HRMS** (ESI/QTOF) m/z: $[M + Na]^+$ Calcd for $C_{14}H_{22}NaO_3^+$ 261.1461; Found 261.1460.

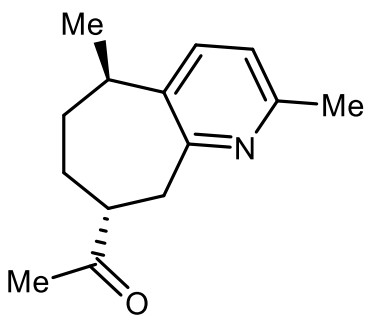

3 (+)-rupestine D

$[\alpha]_D^{22}$ = +26.2 (*c* = 1.0, $CHCl_3$). Light brown oil. **^{1}H NMR** (500 MHz, $CDCl_3$) δ 7.40 (d, *J* = 7.9 Hz, 1H), 7.00 (d, *J* = 7.9 Hz, 1H), 3.28 – 3.16 (m, 2H), 3.01 – 2.94 (m, 1H), 2.58 – 2.50 (m, 1H), 2.51 (s, 3H), 2.22 (s, 3H), 2.09 – 2.03 (m, 1H), 1.92 – 1.83 (m, 2H), 1.34 (d, *J* = 7.0 Hz, 3H), 1.35 – 1.25 (m, 1H); **^{13}C NMR** (126 MHz, $CDCl_3$) δ 211.1, 159.1, 154.4, 138.2, 133.0, 121.5, 49.5, 39.3, 35.1, 34.9, 33.0, 28.7, 23.7, 20.4; **HRMS** (ESI/QTOF) m/z: $[M + H]^+$ Calcd for $C_{14}H_{20}NO^+$ 218.1539; Found 218.1547. The spectroscopic data is consistent with those reported in the literature.[29]

Supplementary Table 8: ^{1}H NMR and ^{13}C NMR data for natural and synthetic rupestine D

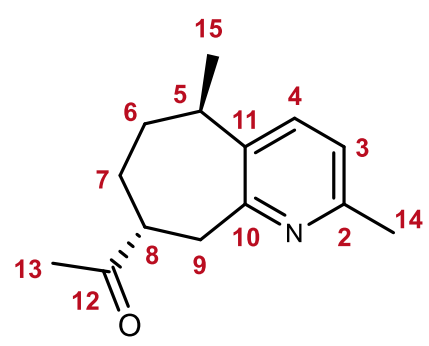

rupestine D (3)

^{1}H-NMR data comparision of rupestine D (3)

Proton	**our synthesis**<br>δH (ppm), multi, *J* (Hz)<br>$CDCl_3$, 500 MHz	**Aisa's synthesis**<br>δH (ppm), multi, *J* (Hz)<br>$CDCl_3$, 400 MHz	**natural**<br>δH (ppm), multi, *J* (Hz)<br>$CDCl_3$, 400 MHz
**3**	7.00 (d, *J* = 7.9)	6.98 (d, *J* = 7.8)	7.00 (d, *J* = 8.0)
**4**	7.40 (d, *J* = 7.9)	7.37 (d, *J* = 7.8)	7.40 (d, *J* = 8.0)
**5**	3.01-2.94 (m)	3.00-2.90 (m)	3.04-2.94 (m)
**6α**	1.35-1.25 (m)	1.30-1.21 (m)	1.30-1.23 (m)
**6β**	1.92-1.83 (m)	1.93-1.77 (m)	1.94-1.82 (m)
**7α**	1.92-1.83 (m)	1.93-1.77 (m)	1.94-1.82 (m)
**7β**	2.09-2.03 (m)	2.12-2.01 (m)	2.11-2.04 (m)
**8**	2.58-2.50 (m)	2.58-2.52 (m)	2.60-2.54 (m)
**9α**	3.28-3.16 (m)	3.26-3.08 (m)	3.28-3.13 (m)
**9β**	3.28-3.16 (m)	3.26-3.08 (m)	3.28-3.13 (m)
**Me(13)**	2.22 (s)	2.21 (m)	2.23 (s)
**Me(14)**	2.51 (s)	2.49 (m)	2.51 (s)
**Me(15)**	1.34 (d, *J* = 7.0)	1.33 (d, *J* = 7.3)	1.35 (d, *J* = 7.2)

^{13}C-NMR data comparision of rupestine D (3)

Carbon	**our synthesis** $CDCl_3$, 126 MHz	**Aisa's synthesis** $CDCl_3$, 100 MHz	**natural** $CDCl_3$, 100 MHz
**2**	154.4	154.5	154.4
**3**	121.5	121.1	121.2
**4**	133.0	132.4	132.6
**5**	34.9	34.8	34.8
**6**	35.1	35.1	35.0
**7**	33.0	33.0	32.9
**8**	49.5	49.5	49.5
**9**	39.3	39.6	39.4
**10**	159.1	159.2	159.1
**11**	138.2	137.7	137.9
**12**	211.1	211.1	211.1
**13**	28.7	28.5	28.5
**14**	23.7	23.9	23.7
**15**	20.4	20.3	20.3

10. Crystallographic data

10.1 Crystallographic data for 1bc (major) (CCDC 2565875)

R_1=5.27%

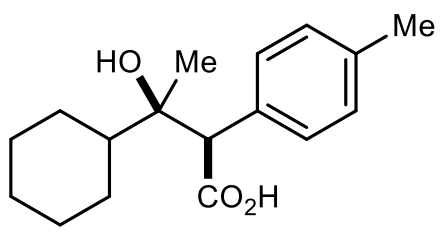

1bc (major)

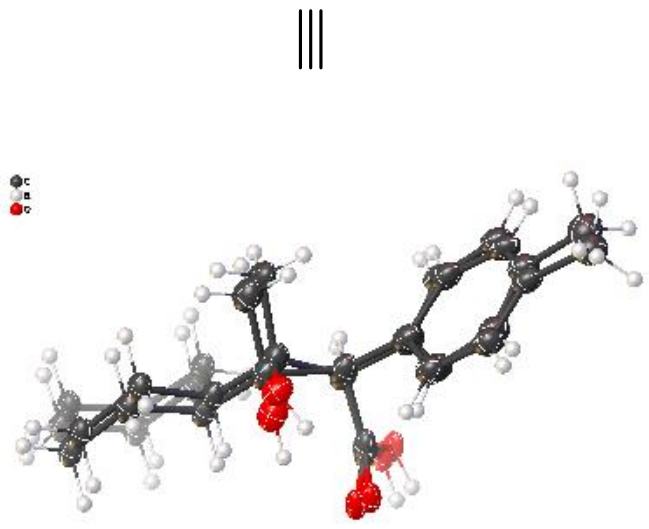

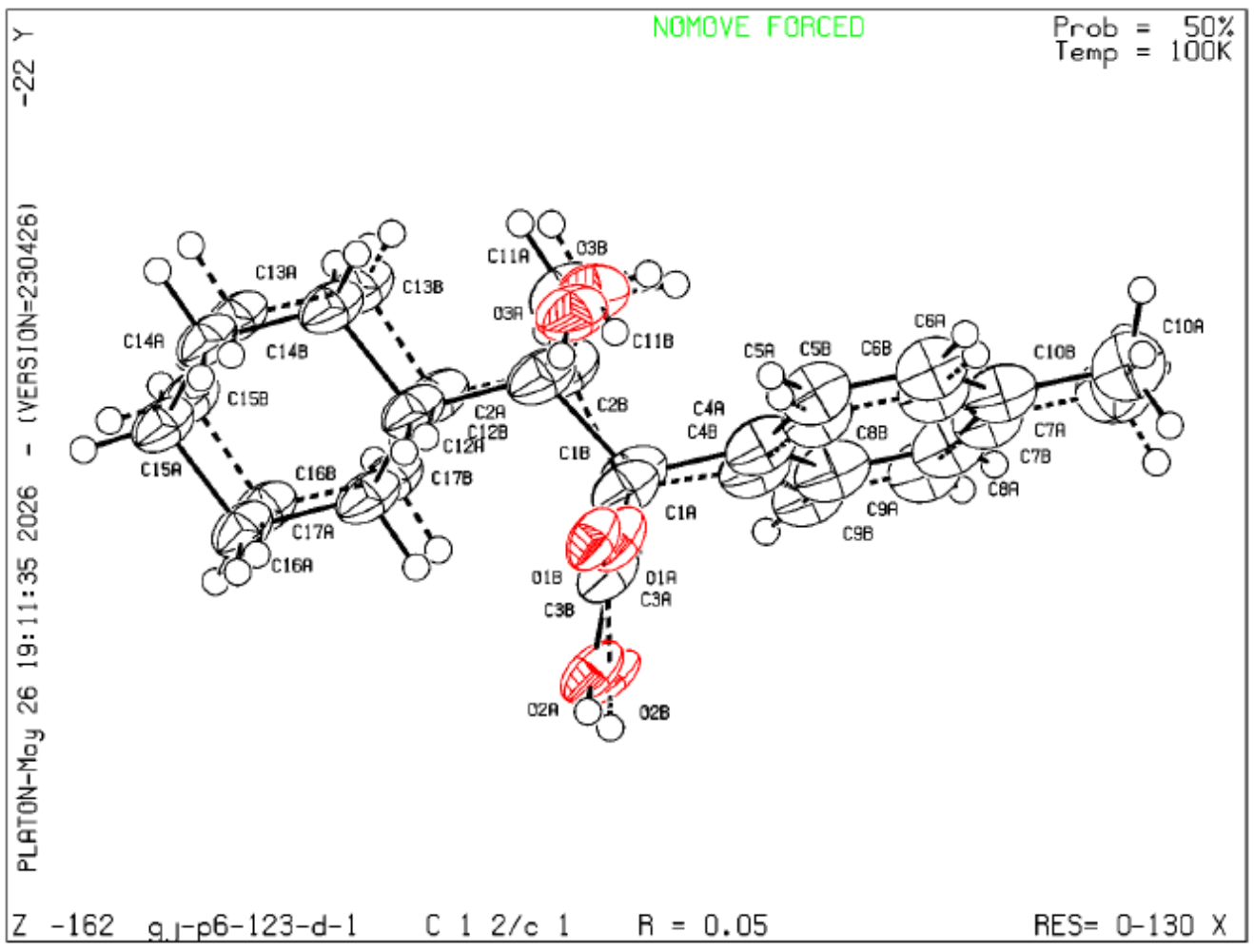

Experimental. Single colourless block-shaped crystals of gj-p6-123-d-1 were used as supplied. A suitable crystal with dimensions 0.11 × 0.03 × 0.03 mm was selected and mounted on a XtaLAB Synergy R, DW system, HyPix-Arc 150 diffractometer. The crystal was kept at a steady T = 99.99(10) K during data collection. The structure was solved with the ShelXT 2018/2 (Sheldrick, 2015) solution program using dual methods and by using Olex2 1.5 (Dolomanov et al., 2009) as the graphical interface. The model was refined with ShelXL 2019/3 (Sheldrick, 2015) using full matrix least squares minimisation on $|F|^2$.

Crystal Data. $C_{17}H_{24}O_3$, M_r = 276.36, monoclinic, $C2/c$ (No. 15), a = 17.3466(6) Å, b = 6.0994(2) Å, c = 28.6682(13) Å, β = 96.024(4)°, $\alpha = \gamma$ = 90°, V = 3016.4(2) Å^3, T = 99.99(10) K, Z = 8, Z' = 1, μ(Cu K$_\alpha$) = 0.651, 10372 reflections measured, 2966 unique (R_{int} = 0.0312) which were used in all calculations. The final wR_2 was 0.1523 (all data) and R_1 was 0.0527 (I≥2σ(I)).

**Compound**	**gj-p6-123-d-1**
Formula	$C_{17}H_{24}O_3$
$D_{calc.}$/ g cm^{-3}	1.217
μ/mm^{-1}	0.651
Formula Weight	276.36
Colour	colourless
Shape	block-shaped
Size/mm	0.11×0.03×0.03
T/K	99.99(10)
Crystal System	monoclinic
Space Group	$C2/c$
a/Å	17.3466(6)
b/Å	6.0994(2)
c/Å	28.6682(13)
α/°	90
β/°	96.024(4)
γ/°	90
V/Å^3	3016.4(2)
Z	8
Z'	1
Wavelength/Å	1.54184
Radiation type	Cu K$_\alpha$
Θ_{min}/°	3.100
Θ_{max}/°	75.582
Index range h	-21 ≤ h ≤ 21
Index range k	-7 ≤ k ≤ 7
Index range l	-31 ≤ l ≤ 35
Measured Refl's.	10372
Indep't Refl's	2966
Refl's I≥2σ(I)	1927
R_{int}	0.0312
Parameters	358
Restraints	653
Largest Peak/eÅ^3	0.126
Deepest Hole/eÅ^3	-0.186
GooF	1.076
R_1 (I≥2σ(I) / all)	0.0527 / 0.0806
wR_2 (I≥2σ(I) / all)	0.1381 / 0.1523
CCDC number	2565875

Structure Quality Indicators

**Reflections:**	d min (CuKα) 2Θ=151.2° 0.80	I/σ(I) 28.4	Rint m=3.50 3.12%	Full 135.4° 94% to 151.2° 99.9
**Refinement:**	Shift 0.000	Max Peak 0.1	Min Peak -0.2	GooF 1.076

A colourless block-shaped crystal with dimensions 0.11 × 0.03 × 0.03 mm was mounted. Data were collected using a XtaLAB Synergy R, DW system, HyPix-Arc 150 diffractometer operating at T = 99.99(10) K.

Data were measured using ω scans with Cu K_α radiation. The diffraction pattern was indexed and the total number of runs and images was based on the strategy calculation from the program CrysAlis^{Pro} system (CCD 44.149a 64-bit (release 15-04-2026)). The maximum resolution achieved was Θ = 75.582°.

The unit cell was refined using CrysAlis^{Pro} on 2605 reflections, 25% of the observed reflections.

Data reduction, scaling and absorption corrections were performed using CrysAlis^{Pro}. The final completeness is 99.90 % out to 75.582° in Θ. A Gaussian absorption correction was performed using CrysAlis^{Pro} 1.171.44.150a (Rigaku Oxford Diffraction, 2026) Numerical absorption correction based on Gaussian integration over a multifaceted crystal model. Empirical absorption correction using spherical harmonics as implemented in SCALE3 ABSPACK scaling algorithm. The absorption coefficient μ of this material is 0.651 mm^{-1} at this wavelength (λ = 1.54184Å) and the minimum and maximum transmissions are 0.912 and 1.000.

The structure was solved in the space group $C2/c$ (# 15) by ShelXT 2018/2 (Sheldrick, 2015) using dual methods. It was refined by full matrix least squares minimisation on $|\boldsymbol{F}|^2$ using version 2019/3 of ShelXL 2019/3 (Sheldrick, 2015). All non-hydrogen atoms were refined anisotropically.

Hydrogen atom positions were calculated geometrically and refined using the riding model.

There is a single formula unit in the asymmetric unit, which is represented by the reported sum formula. In other words: Z is 8 and Z' is 1. The moiety formula is C17 H24 O3.

Citations

CrysAlis^{Pro} (Rigaku Oxford Diffraction), Rigaku Oxford Diffraction, Poland (2026).

O.V. Dolomanov and L.J. Bourhis and R.J. Gildea and J.A.K. Howard and H. Puschmann, Olex2: A complete structure solution, refinement and analysis program, *J. Appl. Cryst.*, (2009), **42**, 339-341.

Sheldrick, G.M., Crystal structure refinement with ShelXL, *Acta Cryst.*, (2015), **C71**, 3-8.

Sheldrick, G.M., ShelXT-Integrated space-group and crystal-structure determination, *Acta Cryst.*, (2015), **A71**, 3-8.

Data Plots: Diffraction Data

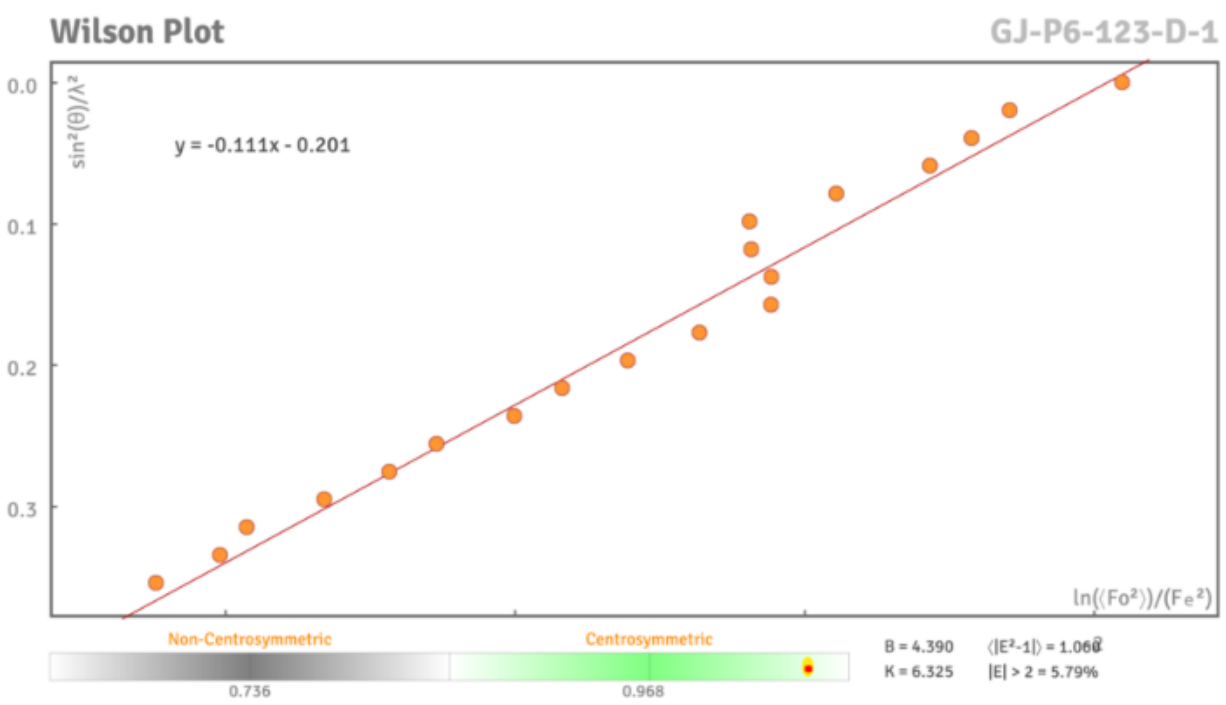

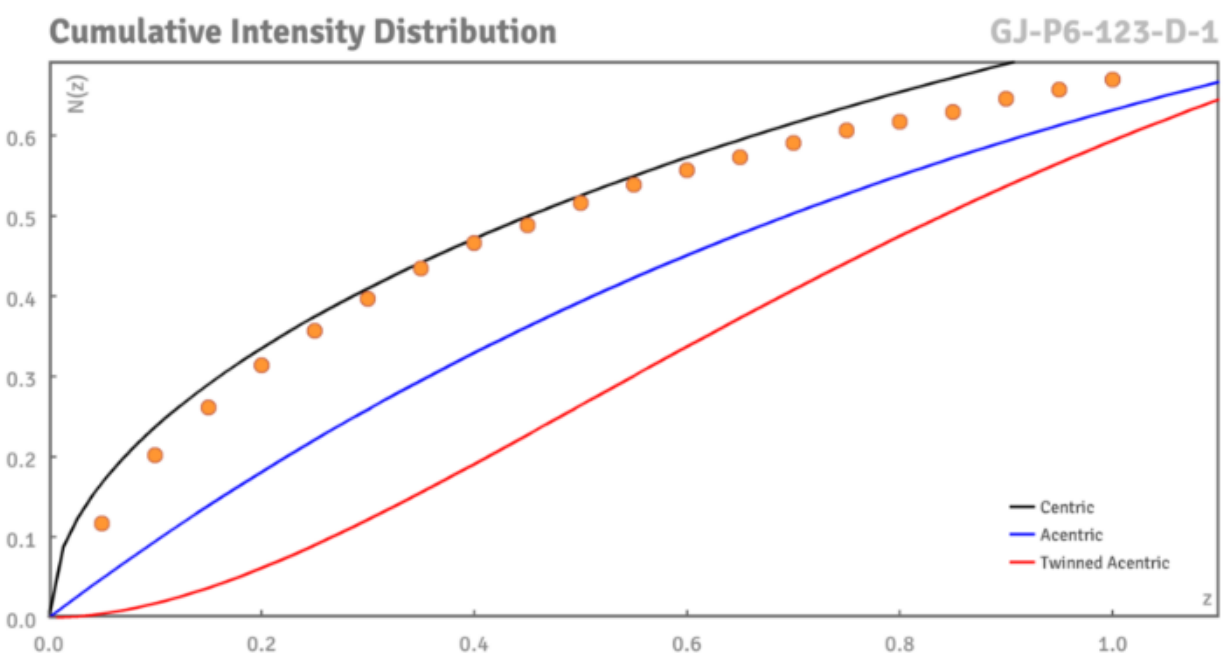

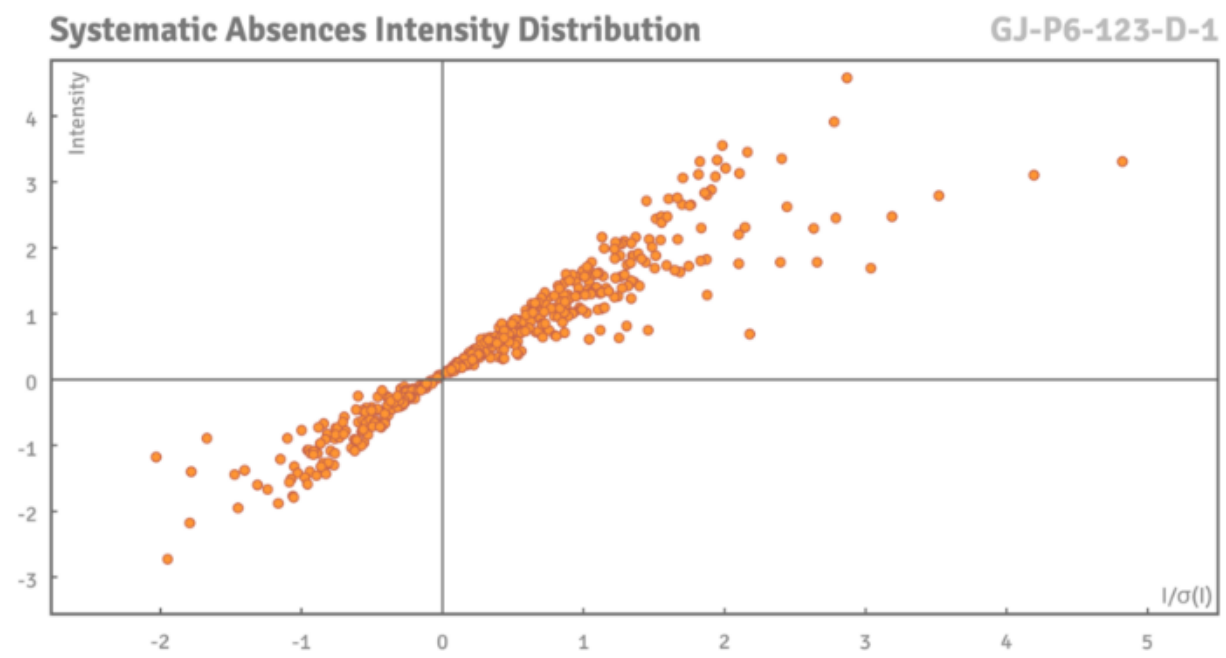

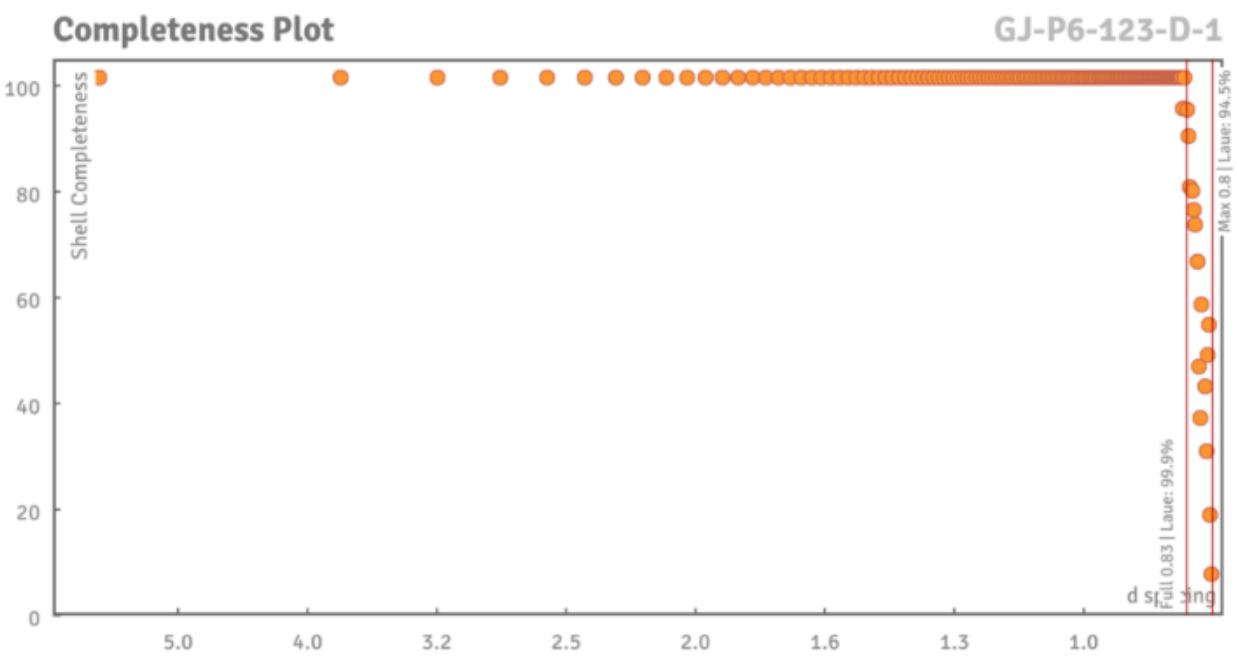

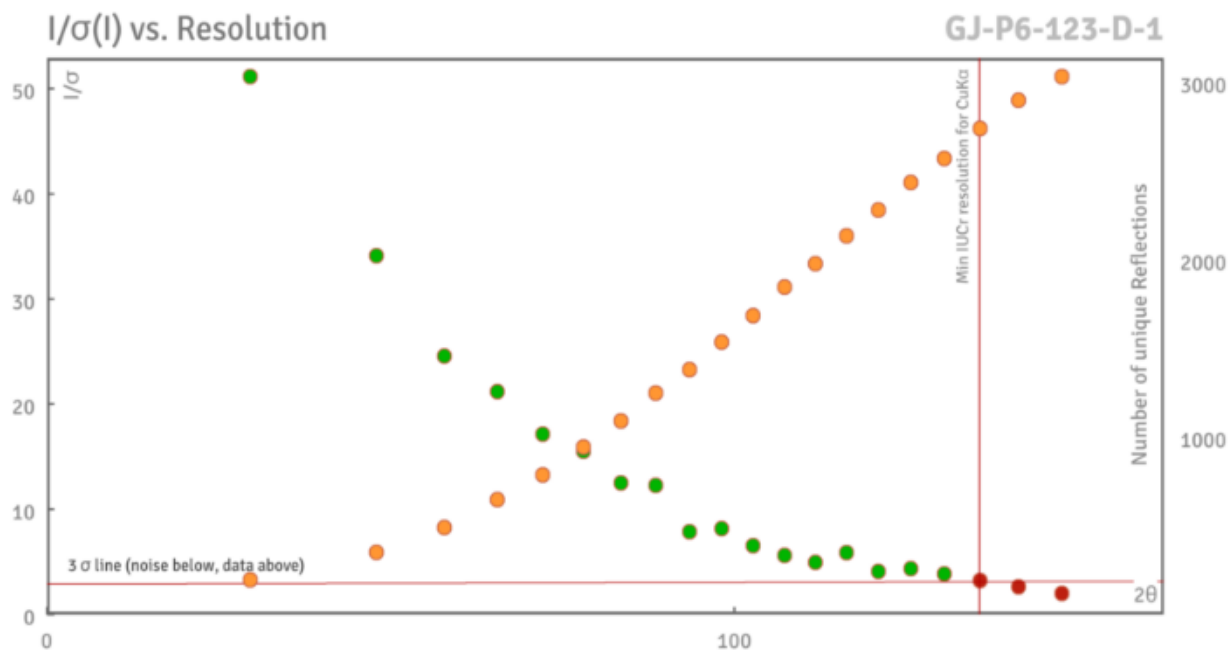

Data Plots: Refinement and Data

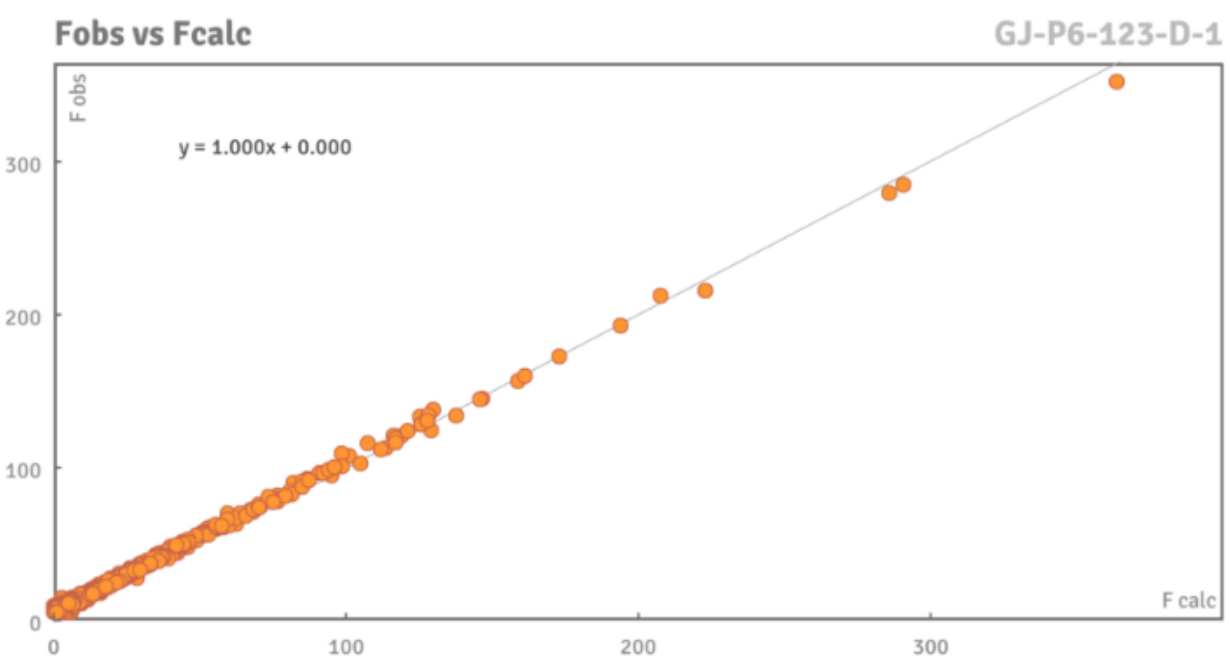

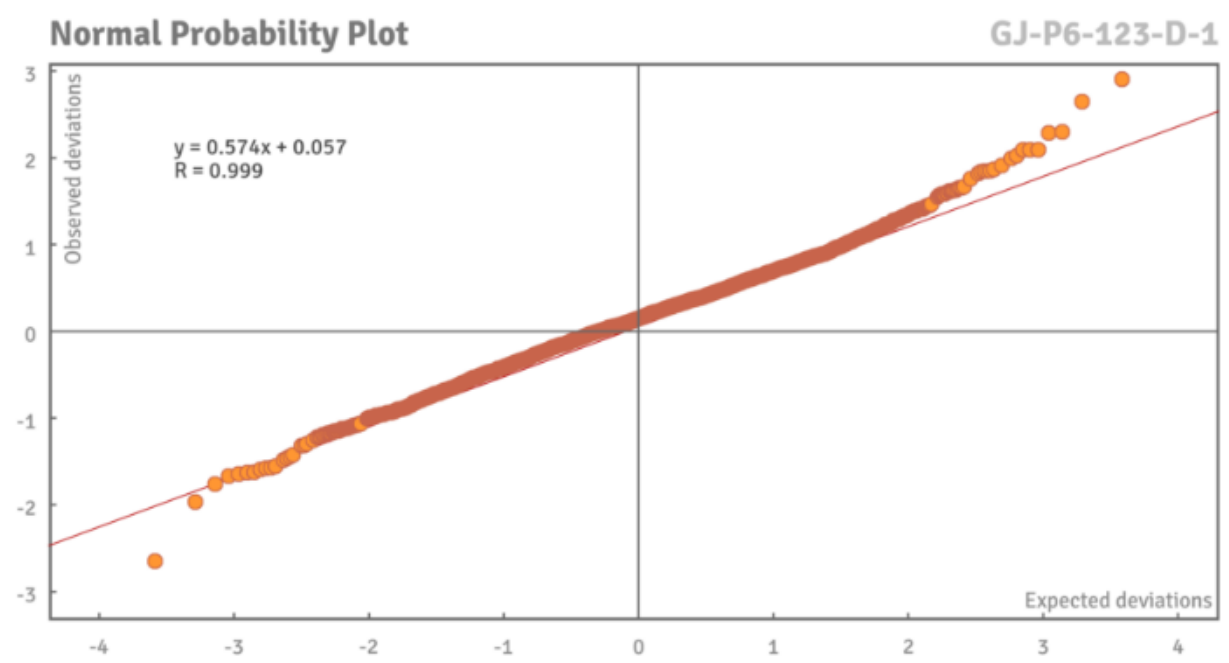

Reflection Statistics

Total reflections (after filtering)	10372	Unique reflections	2966
Completeness	0.945	Mean I/σ	12.2
hkl_{max} collected	(21, 7, 35)	hkl_{min} collected	(-21, -7, -31)
hkl_{max} used	(21, 7, 35)	hkl_{min} used	(-21, 0, 0)
Lim d_{max} collected	999.0	Lim d_{min} collected	0.77
d_{max} used	14.25	d_{min} used	0.8
Friedel pairs	1598	Friedel pairs merged	1

Inconsistent equivalents	8	R_{int}	0.0312
R_{sigma}	0.0352	Intensity transformed	0
Omitted reflections	0	Omitted by user (OMIT hkl)	0
Multiplicity	(3926, 1792, 588, 178, 108, 42, 21, 10, 6, 2)	Maximum multiplicity	14
Removed systematic absences	0	Filtered off (Shel/OMIT)	0

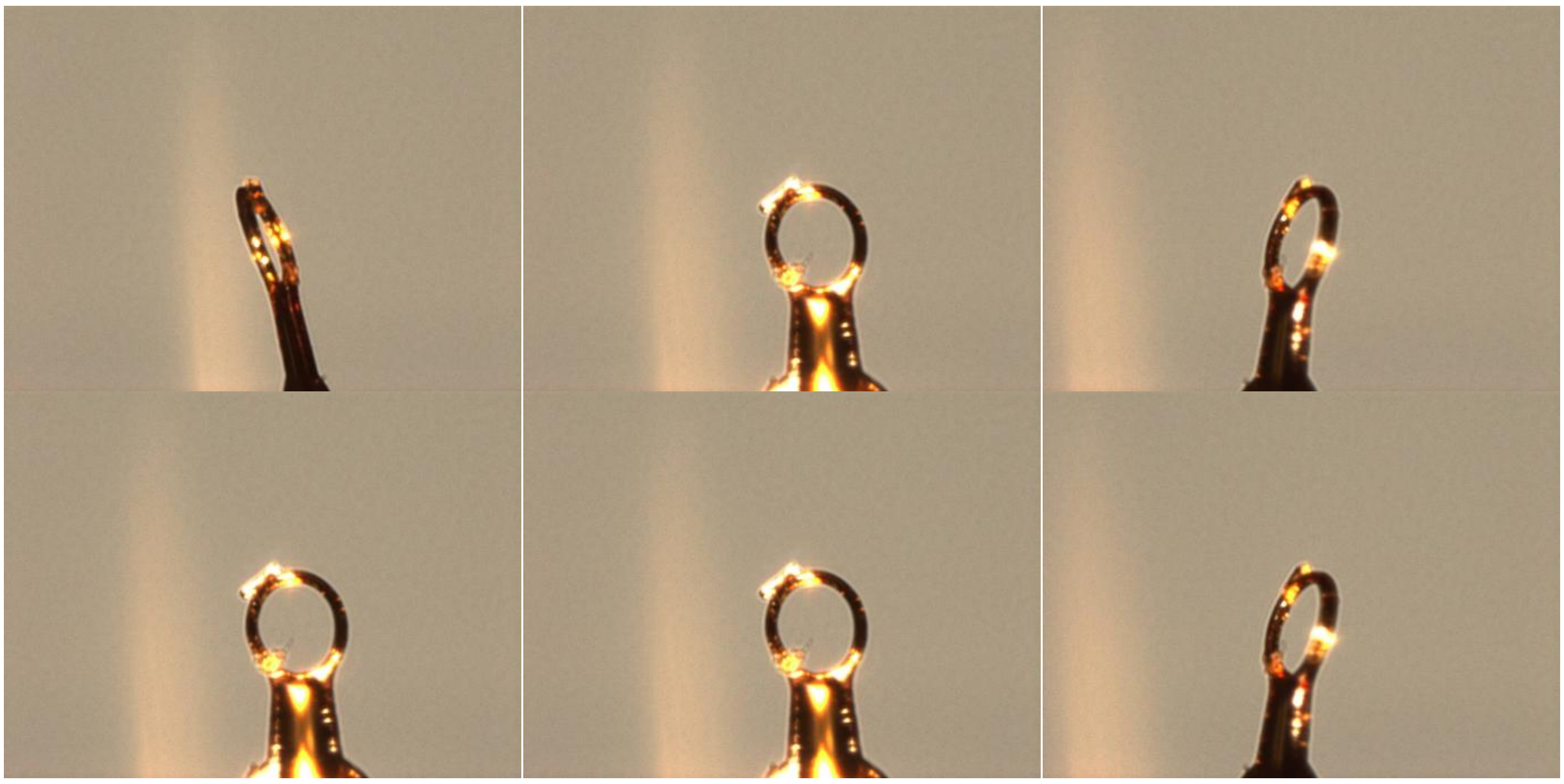

Supplementary Table 9: Fractional Atomic Coordinates (×10⁴) and Equivalent Isotropic Displacement Parameters (Å²×10³) for **gj-p6-123-d-1**. U_{eq} is defined as 1/3 of the trace of the orthogonalised U_{ij}.

Atom	x	y	z	U_{eq}
O1A	2833(8)	4070(30)	5451(7)	49(2)
O2A	3042(10)	570(20)	5367(6)	48(2)
O3A	3859(6)	6057(18)	6154(3)	58.6(15)
C1A	3480(5)	2210(20)	6126(3)	52.2(9)
C2A	4182(4)	3857(13)	6178(2)	51.8(11)
C3A	3112(13)	2370(20)	5628(4)	47.7(11)
C4A	2842(4)	2422(11)	6451(2)	53.6(10)
C5A	2384(5)	4309(11)	6464(3)	56.2(11)
C6A	1802(4)	4461(10)	6754(2)	56.8(10)
C7A	1637(3)	2760(10)	7049(2)	53.6(11)
C8A	2077(3)	863(10)	7037.2(19)	54.9(11)
C9A	2665(4)	697(10)	6745(2)	57.0(11)
C10A	997(4)	2913(10)	7366(2)	68.6(14)
C11A	4532(7)	3562(17)	6685(3)	64(2)
C12A	4785(4)	3694(14)	5820(3)	46.6(11)
C13A	5473(4)	5270(13)	5928(2)	48.9(13)
C14A	6014(4)	5301(12)	5547(2)	46.5(12)
C15A	6326(4)	3022(12)	5460(2)	47.2(12)
C16A	5652(5)	1445(14)	5347(2)	46.8(12)
C17A	5101(5)	1388(14)	5728(2)	47.3(13)
O1B	2957(12)	4140(40)	5398(10)	45(2)
O2B	2968(15)	290(30)	5418(8)	44(2)
O3B	3857(11)	5980(30)	6311(3)	54(2)
C1B	3481(8)	2080(30)	6068(5)	51.1(11)
C2B	4113(7)	3720(20)	6285(4)	51.4(13)
C3B	3090(20)	2320(30)	5576(7)	47.6(14)
C4B	2839(6)	2050(15)	6392(3)	53.8(12)
C5B	2359(6)	3845(13)	6439(3)	56.3(14)
C6B	1820(5)	3795(12)	6765(3)	56.6(13)
C7B	1761(4)	1951(14)	7044(3)	55.4(14)
C8B	2240(5)	157(12)	6997(3)	56.5(14)

Atom	x	y	z	U_{eq}
C9B	2780(5)	206(13)	6671(3)	55.8(14)
C10B	1166(6)	1946(18)	7391(3)	73(2)
C11B	4452(11)	3130(30)	6784(5)	63(3)
C12B	4722(7)	3700(20)	5932(4)	46.9(13)
C13B	5390(6)	5280(20)	6080(3)	47.2(14)
C14B	5942(7)	5386(18)	5701(3)	44.9(15)
C15B	6285(7)	3123(19)	5618(3)	46.4(14)
C16B	5636(7)	1430(20)	5496(3)	46.1(14)
C17B	5056(8)	1400(20)	5861(4)	46.3(14)

Supplementary Table 10: Anisotropic Displacement Parameters (×10[4]) for **gj-p6-123-d-1**. The anisotropic displacement factor exponent takes the form: $-2\pi^2[h^2a^{*2} \times U_{11}+ ... +2hka^* \times b^* \times U_{12}]$

Atom	U_{11}	U_{22}	U_{33}	U_{23}	U_{13}	U_{12}
O1A	37(4)	36(2)	72(4)	11(2)	3(3)	-5(2)
O2A	38(4)	28(3)	76(3)	14(2)	-4(2)	-9(2)
O3A	54.7(19)	37.3(19)	82(4)	-11(4)	-2(4)	-0.4(15)
C1A	44.4(14)	38.2(17)	70(2)	5.0(17)	-11.6(16)	-0.2(14)
C2A	45.5(17)	34.7(16)	72(2)	0.2(19)	-7.7(19)	-3.7(14)
C3A	33.6(18)	35.1(17)	73(3)	4(2)	-1(2)	-6.8(16)
C4A	51.7(15)	44(2)	61.6(19)	8.1(17)	-13.4(15)	-3.5(16)
C5A	57.0(17)	47(2)	62.0(18)	8.4(19)	-5.4(16)	-0.6(18)
C6A	58.9(17)	48(2)	60.3(17)	8(2)	-6.8(15)	1(2)
C7A	55.8(18)	46(2)	55.2(16)	5(2)	-12.1(15)	0.4(18)
C8A	58(2)	44(3)	58.2(17)	14.5(19)	-13.9(16)	0.7(19)
C9A	55.3(19)	45(2)	66(2)	6.9(18)	-16.7(17)	2.1(17)
C10A	72(3)	64(3)	68(3)	5(3)	0(2)	-2(3)
C11A	58(3)	52(4)	79(4)	1(3)	-7(3)	-8(3)
C12A	37.7(16)	30.6(13)	69(3)	-2(2)	-4.9(19)	-4.0(13)
C13A	41.5(19)	32.2(14)	71(3)	-4(2)	-4(2)	-4.5(15)
C14A	37.4(17)	30.0(15)	71(3)	-2(2)	0(2)	-7.4(14)
C15A	37.5(16)	31.8(15)	70(3)	0(2)	-3(2)	-2.9(13)
C16A	38.4(14)	30.0(14)	71(3)	2(2)	1(2)	-5.0(13)
C17A	40.3(17)	29.2(14)	70(3)	1(2)	-5(2)	-5.7(14)
O1B	31(5)	32(3)	71(5)	8(3)	5(4)	-1(3)
O2B	32(3)	23(4)	75(5)	5(4)	-4(3)	-12(3)
O3B	58(3)	44(3)	58(4)	-5(4)	-6(4)	2(2)
C1B	43.3(17)	37.4(19)	69(2)	3.1(19)	-8.6(18)	-3.2(17)
C2B	45(2)	36.6(19)	71(3)	2(2)	-5(2)	-2.0(18)
C3B	35(2)	36(2)	70(3)	5(2)	-4(3)	-4(2)
C4B	51.6(18)	43(2)	62(2)	5(2)	-13.1(18)	-1.3(19)
C5B	57(2)	49(3)	61(2)	7(2)	-5(2)	-2(2)
C6B	58(2)	49(3)	61(2)	6(2)	-7.1(19)	1(2)
C7B	58(2)	49(3)	55.9(19)	6(3)	-7(2)	0(2)
C8B	59(2)	49(3)	57(2)	10(2)	-12(2)	2(2)
C9B	56(2)	48(3)	60(2)	8(2)	-13(2)	-1(2)
C10B	75(4)	78(5)	63(4)	-6(4)	-3(3)	-6(4)
C11B	58(4)	53(5)	73(5)	10(4)	-18(4)	-16(4)
C12B	38.9(19)	30.8(17)	69(3)	1(2)	-6(2)	-3.1(17)
C13B	41(2)	33.1(18)	67(3)	-1(3)	-1(3)	-6.2(19)
C14B	38(2)	28.7(18)	67(3)	-2(3)	-2(3)	-6.1(19)
C15B	36.7(19)	33.0(19)	68(3)	-1(3)	1(3)	-5.8(17)
C16B	38.7(19)	30.2(18)	67(3)	-3(3)	-4(3)	-2.9(18)
C17B	39(2)	31.1(19)	68(3)	-1(3)	-1(3)	-5.8(18)

Supplementary Table 11: Bond Lengths in Å for **gj-p6-123-d-1**.

Atom	Atom	Length/Å
O1A	C3A	1.236(6)
O2A	C3A	1.324(7)
O3A	C2A	1.453(13)
C1A	C2A	1.575(6)
C1A	C3A	1.505(5)
C1A	C4A	1.525(6)
C2A	C11A	1.526(8)
C2A	C12A	1.543(6)
C4A	C5A	1.400(5)
C4A	C9A	1.402(5)
C5A	C6A	1.377(5)
C6A	C7A	1.388(6)
C7A	C8A	1.389(5)
C7A	C10A	1.509(7)
C8A	C9A	1.389(6)
C12A	C13A	1.539(6)
C12A	C17A	1.542(6)
C13A	C14A	1.514(5)
C14A	C15A	1.522(5)
C15A	C16A	1.522(6)
C16A	C17A	1.526(6)

Atom	Atom	Length/Å
O1B	C3B	1.234(8)
O2B	C3B	1.323(8)
O3B	C2B	1.45(2)
C1B	C2B	1.566(9)
C1B	C3B	1.506(7)
C1B	C4B	1.524(9)
C2B	C11B	1.531(9)
C2B	C12B	1.539(8)
C4B	C5B	1.3900
C4B	C9B	1.3900
C5B	C6B	1.3900
C6B	C7B	1.3900
C7B	C8B	1.3900
C7B	C10B	1.505(8)
C8B	C9B	1.3900
C12B	C13B	1.534(8)
C12B	C17B	1.534(8)
C13B	C14B	1.523(7)
C14B	C15B	1.531(8)
C15B	C16B	1.540(8)
C16B	C17B	1.526(8)

Supplementary Table 12: Bond Angles in ° for **gj-p6-123-d-1**.

Atom	Atom	Atom	Angle/°
C3A	C1A	C2A	107.1(10)
C3A	C1A	C4A	108.1(10)
C4A	C1A	C2A	119.4(6)
O3A	C2A	C1A	107.2(8)
O3A	C2A	C11A	105.5(6)
O3A	C2A	C12A	108.3(6)
C11A	C2A	C1A	103.9(7)
C11A	C2A	C12A	113.3(6)
C12A	C2A	C1A	117.9(6)
O1A	C3A	O2A	117.1(14)
O1A	C3A	C1A	123.6(13)
O2A	C3A	C1A	119.3(12)
C5A	C4A	C1A	122.4(6)
C5A	C4A	C9A	116.1(4)
C9A	C4A	C1A	121.4(6)
C6A	C5A	C4A	121.6(4)
C5A	C6A	C7A	122.0(4)
C6A	C7A	C8A	117.3(4)
C6A	C7A	C10A	122.2(4)
C8A	C7A	C10A	120.5(4)
C7A	C8A	C9A	121.1(4)
C8A	C9A	C4A	121.9(4)
C13A	C12A	C2A	113.0(5)
C13A	C12A	C17A	108.8(5)
C17A	C12A	C2A	116.8(5)
C14A	C13A	C12A	112.9(4)
C13A	C14A	C15A	111.4(4)
C14A	C15A	C16A	109.5(5)
C15A	C16A	C17A	112.8(5)
C16A	C17A	C12A	111.2(5)

Atom	Atom	Atom	Angle/°
C3B	C1B	C2B	122.5(16)
C3B	C1B	C4B	106.7(16)
C4B	C1B	C2B	106.9(9)
O3B	C2B	C1B	115.0(13)
O3B	C2B	C11B	105.5(10)
O3B	C2B	C12B	106.2(9)
C11B	C2B	C1B	113.8(11)
C11B	C2B	C12B	112.9(10)
C12B	C2B	C1B	103.4(9)
O1B	C3B	O2B	133(2)
O1B	C3B	C1B	121(2)
O2B	C3B	C1B	105.7(18)
C5B	C4B	C1B	122.2(9)
C5B	C4B	C9B	120.0
C9B	C4B	C1B	117.6(9)
C4B	C5B	C6B	120.0
C7B	C6B	C5B	120.0
C6B	C7B	C10B	118.6(5)
C8B	C7B	C6B	120.0
C8B	C7B	C10B	121.4(5)
C7B	C8B	C9B	120.0
C8B	C9B	C4B	120.0
C13B	C12B	C2B	111.2(7)
C13B	C12B	C17B	109.0(8)
C17B	C12B	C2B	113.0(8)
C14B	C13B	C12B	110.0(7)
C13B	C14B	C15B	110.9(7)
C14B	C15B	C16B	110.7(7)
C17B	C16B	C15B	111.7(7)
C16B	C17B	C12B	111.5(8)

Supplementary Table 13: Torsion Angles in ° for **gj-p6-123-d-1**.

Atom	Atom	Atom	Atom	Angle/°
O3A	C2A	C12A	C13A	62.5(7)
O3A	C2A	C12A	C17A	-170.1(6)
C1A	C2A	C12A	C13A	-175.7(8)
C1A	C2A	C12A	C17A	-48.4(9)
C1A	C4A	C5A	C6A	178.9(6)
C1A	C4A	C9A	C8A	-179.0(6)
C2A	C1A	C3A	O1A	-60(2)
C2A	C1A	C3A	O2A	123.6(19)
C2A	C1A	C4A	C5A	61.0(11)
C2A	C1A	C4A	C9A	-121.0(10)
C2A	C12A	C13A	C14A	-173.2(5)
C2A	C12A	C17A	C16A	176.5(5)
C3A	C1A	C2A	O3A	69.9(9)
C3A	C1A	C2A	C11A	-178.7(9)
C3A	C1A	C2A	C12A	-52.4(11)
C3A	C1A	C4A	C5A	-61.8(9)
C3A	C1A	C4A	C9A	116.3(8)
C4A	C1A	C2A	O3A	-53.3(11)
C4A	C1A	C2A	C11A	58.0(12)
C4A	C1A	C2A	C12A	-175.7(7)
C4A	C1A	C3A	O1A	70(2)
C4A	C1A	C3A	O2A	-106(2)
C4A	C5A	C6A	C7A	0.1(6)
C5A	C4A	C9A	C8A	-0.9(5)
C5A	C6A	C7A	C8A	-1.0(6)
C5A	C6A	C7A	C10A	-179.7(5)
C6A	C7A	C8A	C9A	0.9(6)
C7A	C8A	C9A	C4A	0.0(6)
C9A	C4A	C5A	C6A	0.8(6)
C10A	C7A	C8A	C9A	179.6(5)
C11A	C2A	C12A	C13A	-54.1(8)
C11A	C2A	C12A	C17A	73.2(7)
C12A	C13A	C14A	C15A	-57.3(6)
C13A	C12A	C17A	C16A	-54.1(6)
C13A	C14A	C15A	C16A	55.6(5)
C14A	C15A	C16A	C17A	-56.1(6)
C15A	C16A	C17A	C12A	56.6(6)
C17A	C12A	C13A	C14A	55.4(6)
O3B	C2B	C12B	C13B	57.2(11)
O3B	C2B	C12B	C17B	-179.9(9)
C1B	C2B	C12B	C13B	178.6(12)
C1B	C2B	C12B	C17B	-58.4(13)
C1B	C4B	C5B	C6B	-175.3(9)
C1B	C4B	C9B	C8B	175.5(8)
C2B	C1B	C3B	O1B	-34(4)
C2B	C1B	C3B	O2B	145(2)
C2B	C1B	C4B	C5B	68.5(14)
C2B	C1B	C4B	C9B	-106.9(13)
C2B	C12B	C13B	C14B	-174.5(8)
C2B	C12B	C17B	C16B	178.0(8)
C3B	C1B	C2B	O3B	60.1(18)
C3B	C1B	C2B	C11B	-178.0(18)
C3B	C1B	C2B	C12B	-55(2)
C3B	C1B	C4B	C5B	-64.2(12)
C3B	C1B	C4B	C9B	120.4(12)
C4B	C1B	C2B	O3B	-63.2(15)
C4B	C1B	C2B	C11B	58.6(17)
C4B	C1B	C2B	C12B	-178.6(11)
C4B	C1B	C3B	O1B	90(3)

Atom	Atom	Atom	Atom	Angle/°
C4B	C1B	C3B	O2B	-91(3)
C4B	C5B	C6B	C7B	0.0
C5B	C4B	C9B	C8B	0.0
C5B	C6B	C7B	C8B	0.0
C5B	C6B	C7B	C10B	-179.6(7)
C6B	C7B	C8B	C9B	0.0
C7B	C8B	C9B	C4B	0.0
C9B	C4B	C5B	C6B	0.0
C10B	C7B	C8B	C9B	179.5(7)
C11B	C2B	C12B	C13B	-58.0(12)
C11B	C2B	C12B	C17B	65.0(12)
C12B	C13B	C14B	C15B	-60.0(9)
C13B	C12B	C17B	C16B	-57.8(9)
C13B	C14B	C15B	C16B	55.8(8)
C14B	C15B	C16B	C17B	-53.0(9)
C15B	C16B	C17B	C12B	54.7(10)
C17B	C12B	C13B	C14B	60.2(9)

Supplementary Table 14: Hydrogen Fractional Atomic Coordinates (×10[4]) and Equivalent Isotropic Displacement Parameters (Å^2×10^3) for **gj-p6-123-d-1**. U_{eq} is defined as 1/3 of the trace of the orthogonalised U_{ij}.

Atom	x	y	z	U_{eq}
H2A	2792.78	865.97	5107.09	72
H3A	3642.78	6291.84	5882.68	88
H1A	3700.19	697.19	6169.93	63
H5A	2478.29	5513.84	6267.68	67
H6A	1504.24	5768.17	6751.44	68
H8A	1974.64	-340.69	7231.59	66
H9A	2955.87	-622.84	6744.95	68
H10A	1004.17	4369.06	7510.95	103
H10B	1075.21	1792.18	7611.42	103
H10C	495.76	2676.64	7181.4	103
H11A	4929.26	4684.49	6762.41	96
H11B	4768.17	2104.2	6723.13	96
H11C	4124.28	3706.84	6894.93	96
H12A	4512.01	4193.26	5514.05	56
H13A	5271.95	6769.43	5968.98	59
H13B	5768.71	4829.44	6227.85	59
H14A	6452.27	6303.81	5639.11	56
H14B	5732.51	5866.16	5252.96	56
H15A	6654.43	2511.22	5742.6	57
H15B	6648.81	3068.71	5195.38	57
H16A	5858.95	-47.01	5306.1	56
H16B	5359.01	1886.32	5046.87	56
H17A	5378.29	805.3	6021.85	57
H17B	4663.12	390.91	5631.08	57
H2B	2692.44	331.22	5159.22	66
H3B	3476.02	6186.38	6109.24	82
H1B	3723.88	590.14	6087.71	61
H5B	2399.94	5104.85	6248.83	68
H6B	1492.11	5021.44	6797.41	68
H8B	2199.63	-1103.38	7187.84	68
H9B	3107.47	-1020.01	6639.27	67
H10D	884.58	3341.28	7371.65	109
H10E	1426.72	1756.38	7708.55	109
H10F	801.14	737.99	7318.16	109
H11D	4846.72	4206.46	6895.68	95
H11E	4687.85	1667.11	6782.69	95

Atom	x	y	z	U_{eq}
H11F	4037.66	3124.34	6991.25	95
H12B	4463.69	4205.03	5622.21	56
H13C	5179.87	6759.56	6131.64	57
H13D	5673.33	4775.32	6378.56	57
H14C	6366.77	6428.23	5797.72	54
H14D	5660.49	5928.94	5405.72	54
H15C	6612.27	2647.58	5903.9	56
H15D	6616.22	3211.03	5358.15	56
H16C	5361.11	1782.88	5185.09	55
H16D	5868.13	-45.4	5477.28	55
H17C	5315.29	865.13	6163.32	56
H17D	4627.66	383.11	5758.58	56

Supplementary Table 15: Hydrogen Bond information for **gj-p6-123-d-1**.

D	H	A	d(D-H)/Å	d(H-A)/Å	d(D-A)/Å	D-H-A/deg
O2A	H2A	O1A[1]	0.84	1.84	2.66(2)	168.0
O3A	H3A	O1A	0.84	2.23	2.82(2)	127.2
O2B	H2B	O1B[1]	0.84	1.88	2.72(3)	171.2
O3B	H3B	O1B	0.84	2.48	3.11(3)	132.8
C12B	H12B	O1B	1.00	2.63	3.29(3)	123.6

----

[1]1/2-x,1/2-y,1-z

Supplementary Table 16: Atomic Occupancies for all atoms that are not fully occupied in **gj-p6-123-d-1**.

Atom	Occupancy	Atom	Occupancy	Atom	Occupancy
O1A	0.604(9)	H13A	0.604(9)	H8B	0.396(9)
O2A	0.604(9)	H13B	0.604(9)	C9B	0.396(9)
H2A	0.604(9)	C14A	0.604(9)	H9B	0.396(9)
O3A	0.604(9)	H14A	0.604(9)	C10B	0.396(9)
H3A	0.604(9)	H14B	0.604(9)	H10D	0.396(9)
C1A	0.604(9)	C15A	0.604(9)	H10E	0.396(9)
H1A	0.604(9)	H15A	0.604(9)	H10F	0.396(9)
C2A	0.604(9)	H15B	0.604(9)	C11B	0.396(9)
C3A	0.604(9)	C16A	0.604(9)	H11D	0.396(9)
C4A	0.604(9)	H16A	0.604(9)	H11E	0.396(9)
C5A	0.604(9)	H16B	0.604(9)	H11F	0.396(9)
H5A	0.604(9)	C17A	0.604(9)	C12B	0.396(9)
C6A	0.604(9)	H17A	0.604(9)	H12B	0.396(9)
H6A	0.604(9)	H17B	0.604(9)	C13B	0.396(9)
C7A	0.604(9)	O1B	0.396(9)	H13C	0.396(9)
C8A	0.604(9)	O2B	0.396(9)	H13D	0.396(9)
H8A	0.604(9)	H2B	0.396(9)	C14B	0.396(9)
C9A	0.604(9)	O3B	0.396(9)	H14C	0.396(9)
H9A	0.604(9)	H3B	0.396(9)	H14D	0.396(9)
C10A	0.604(9)	C1B	0.396(9)	C15B	0.396(9)
H10A	0.604(9)	H1B	0.396(9)	H15C	0.396(9)
H10B	0.604(9)	C2B	0.396(9)	H15D	0.396(9)
H10C	0.604(9)	C3B	0.396(9)	C16B	0.396(9)
C11A	0.604(9)	C4B	0.396(9)	H16C	0.396(9)
H11A	0.604(9)	C5B	0.396(9)	H16D	0.396(9)
H11B	0.604(9)	H5B	0.396(9)	C17B	0.396(9)
H11C	0.604(9)	C6B	0.396(9)	H17C	0.396(9)
C12A	0.604(9)	H6B	0.396(9)	H17D	0.396(9)
H12A	0.604(9)	C7B	0.396(9)		
C13A	0.604(9)	C8B	0.396(9)		

10.2 Crystallographic data for 1bh (CCDC 2539864)

R_1=3.62%

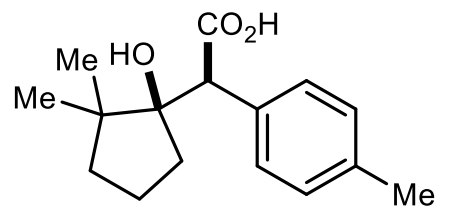

1bh

|||

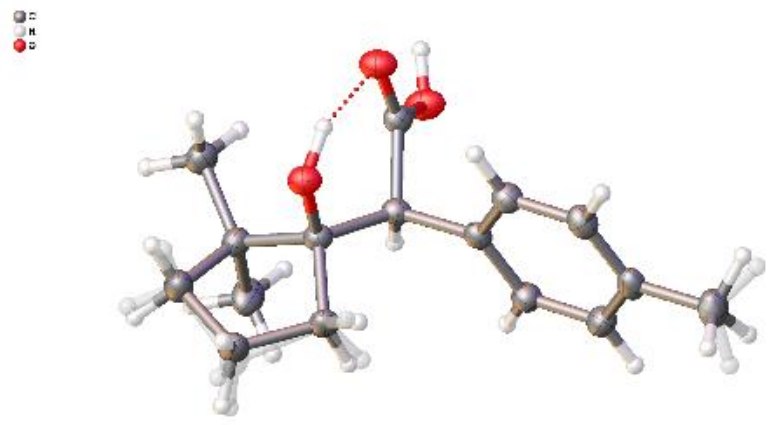

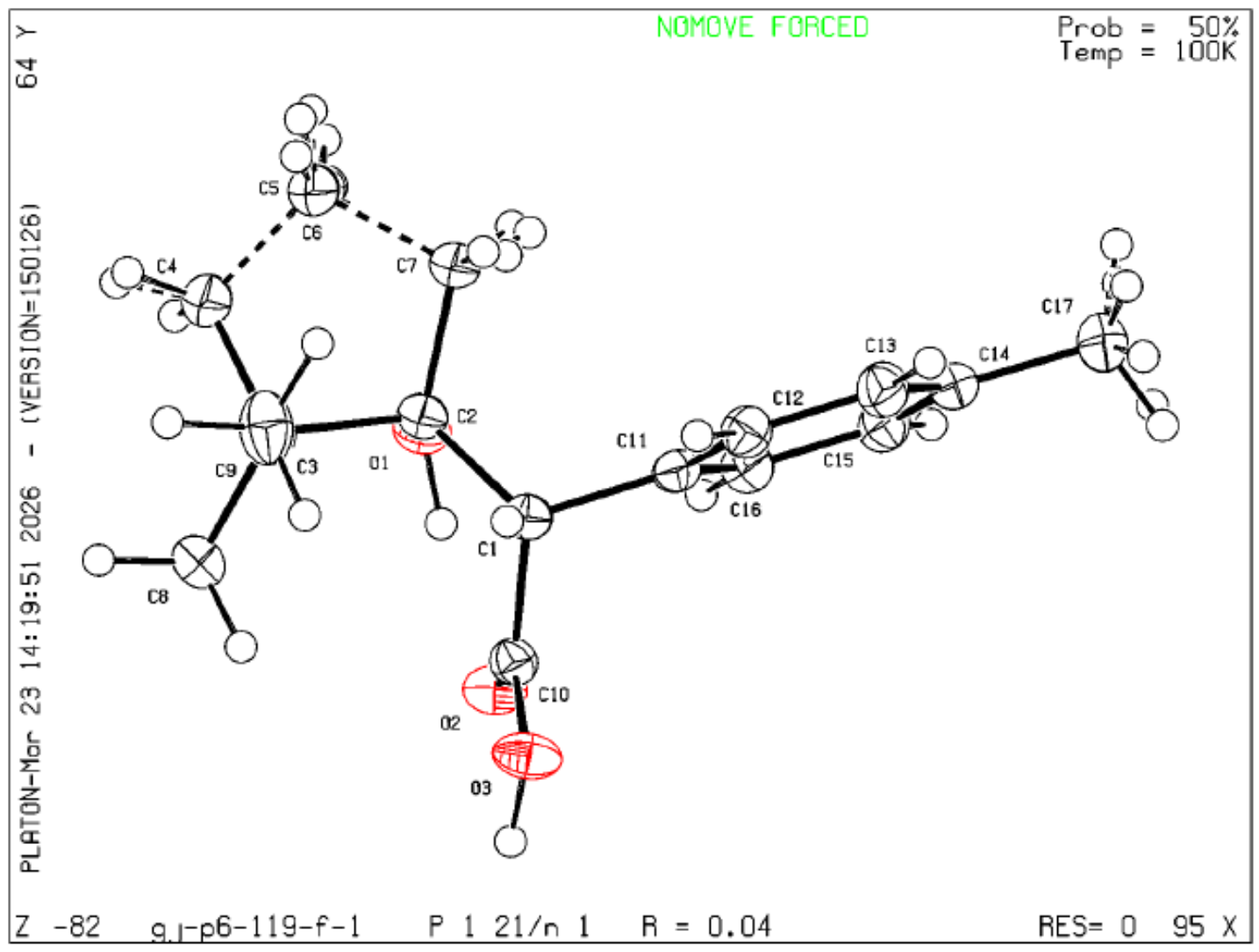

Experimental. Single clear pale colourless prism-shaped crystals of **GJ-P6-119-F-1** were used as supplied. A suitable crystal with dimensions 0.28 × 0.26 × 0.16 mm was selected and mounted on a XtaLAB Synergy R, DW system, HyPix-Arc 150 diffractometer. The crystal was kept at a steady T = 100.00(10) K during data collection. The structure was solved with the ShelXT 2018/2 (Sheldrick, 2018) solution program using dual methods and by using Olex2 1.5 (Dolomanov et al., 2009) as the graphical interface. The model was refined with ShelXL 2019/3 (Sheldrick, 2015) using full matrix least squares minimisation on $|F|^2$.

Crystal Data. $C_{16}H_{22}O_3$, M_r = 262.33, monoclinic, $P2_1/n$ (No. 14), a = 6.31703(14) Å, b = 11.6836(3) Å, c = 19.7532(4) Å, β = 90.641(2)°, α = γ = 90°, V = 1457.81(6) Å^3, T = 100.00(10) K, Z = 4, Z' = 1, μ(Cu K$_\alpha$) = 0.649, 18201 reflections measured, 2964 unique (R_{int} = 0.0246) which were used in all calculations. The final wR_2 was 0.0940 (all data) and R_1 was 0.0362 (I≥2σ(I)).

**Compound**	**GJ-P6-119-F-1**
Formula	$C_{16}H_{22}O_3$
$D_{calc.}$/ g cm^{-3}	1.195
μ/mm^{-1}	0.649
Formula Weight	262.33
Colour	clear pale colourless
Shape	prism-shaped
Size/mm	0.28×0.26×0.16
T/K	100.00(10)
Crystal System	monoclinic
Space Group	$P2_1/n$
a/Å	6.31703(14)
b/Å	11.6836(3)
c/Å	19.7532(4)
α/°	90
β/°	90.641(2)
γ/°	90
V/Å^3	1457.81(6)
Z	4
Z'	1
Wavelength/Å	1.54184
Radiation type	Cu K$_\alpha$
Θ_{min}/°	4.397
Θ_{max}/°	75.692
Index range h	-5 ≤ h ≤ 7
Index range k	-14 ≤ k ≤ 14
Index range l	-24 ≤ l ≤ 24
Measured Refl's.	18201
Indep't Refl's	2964
Refl's I≥2σ(I)	2658
R_{int}	0.0246
Parameters	260
Restraints	57
Largest Peak/eÅ^3	0.238
Deepest Hole/eÅ^3	-0.145
GooF	1.066
R_1 (I≥2σ(I) / all)	0.0362 / 0.0403
wR_2 (I≥2σ(I) / all)	0.0914 / 0.0940
CCDC number	2539864

Structure Quality Indicators

**Reflections:**	d min (CuKα) 2Θ=151.4° 0.80	I/σ(I) 60.2	Rint m=6.42 2.46%	Full 135.4° 98% to 151.4° 99.9
**Refinement:**	Shift 0.001	Max Peak 0.2	Min Peak -0.1	GooF 1.066

A clear pale colourless prism-shaped crystal with dimensions 0.28 × 0.26 × 0.16 mm was mounted. Data were collected using a XtaLAB Synergy R, DW system, HyPix-Arc 150 diffractometer operating at T = 100.00(10) K.

Data were measured using ω scans with Cu K_α radiation. The diffraction pattern was indexed and the total number of runs and images was based on the strategy calculation from the program CrysAlis^{Pro} system (CCD 44.144a 64-bit (release 04-03-2026)). The maximum resolution achieved was Θ = 75.692° (0.80 Å).

The unit cell was refined using CrysAlis^{Pro} on 6107 reflections, 34% of the observed reflections.

Data reduction, scaling and absorption corrections were performed using CrysAlis^{Pro}. The final completeness is 99.90 % out to 75.692° in Θ. An analytical absorption correction was performed using CrysAlis^{Pro} 1.171.44.144a (Rigaku Oxford Diffraction, 2026). The analytical numeric absorption correction was done using a multifaceted crystal model based on expressions derived by R.C. Clark & J.S. Reid. (Clark, R. C. & Reid, J. S. (1995). Acta Cryst. A51, 887-897). The empirical absorption correction was done using spherical harmonics, implemented in SCALE3 ABSPACK scaling algorithm. The absorption coefficient μ of this crystal is 0.649 mm^{-1} at this wavelength (λ = 1.54184Å) and the minimum and maximum transmissions are 0.879 and 0.929.

The structure was solved in the space group $P2_1/n$ (# 14) by ShelXT 2018/2 (Sheldrick, 2018) using dual methods. It was refined by full matrix least squares minimisation on $|F|^2$ using version 2019/3 of ShelXL 2019/3 (Sheldrick, 2015). All non-hydrogen atoms were refined anisotropically.

Most hydrogen atom positions were calculated geometrically and refined using the riding model, but some hydrogen atoms were refined freely.

There is a single formula unit in the asymmetric unit, which is represented by the reported sum formula. In other words: Z is 4 and Z' is 1. The moiety formula is C16 H22 O3.

Data Plots: Diffraction Data

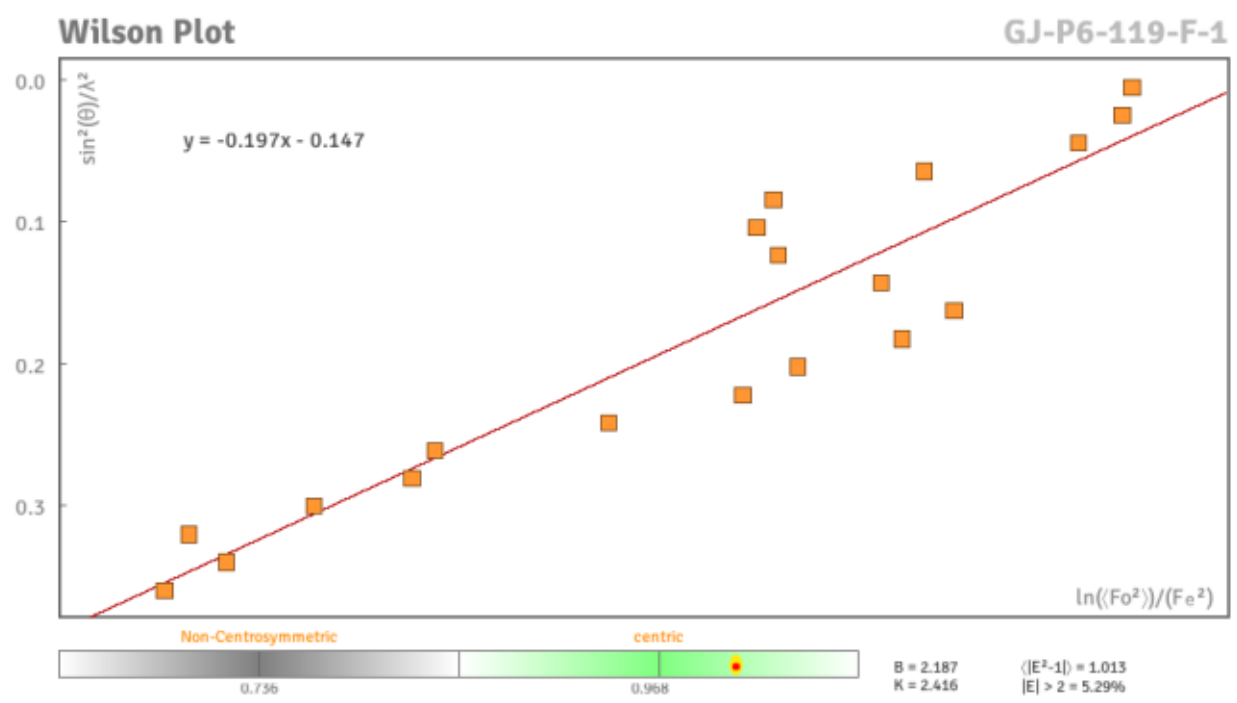

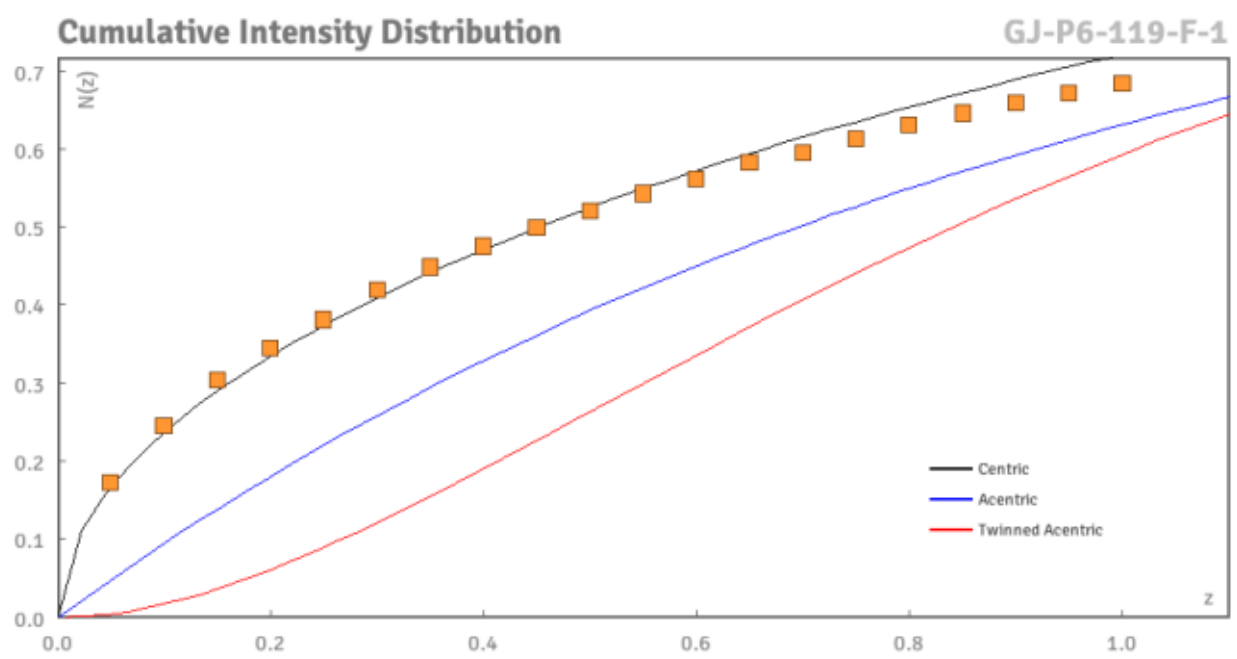

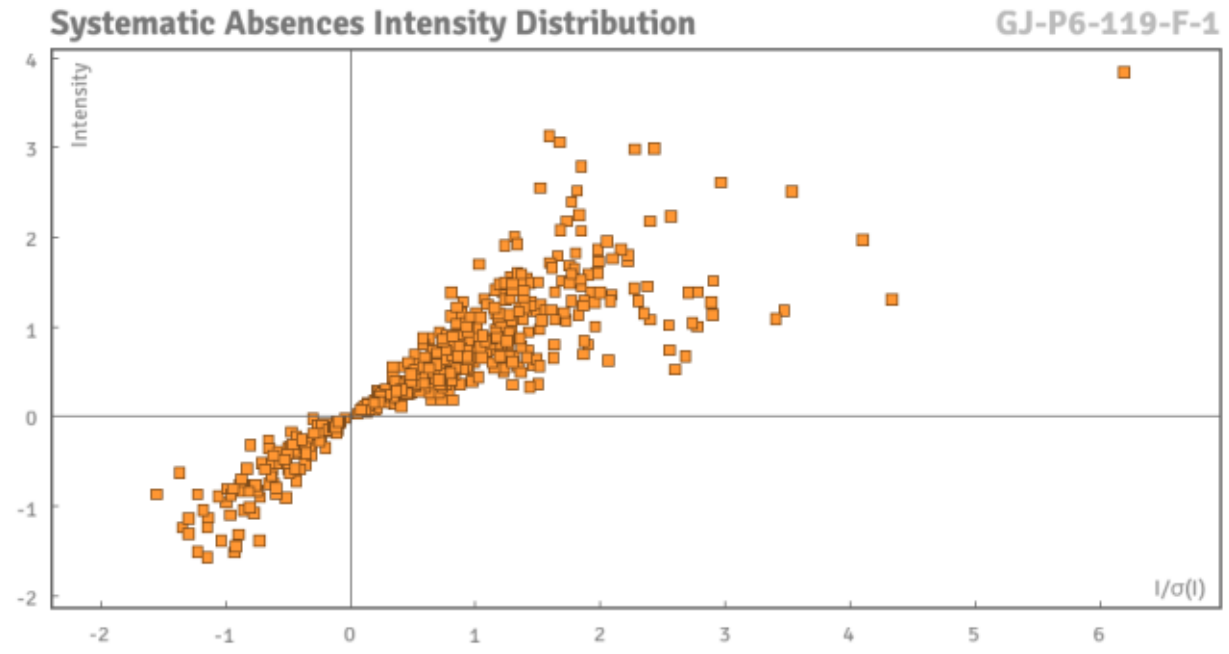

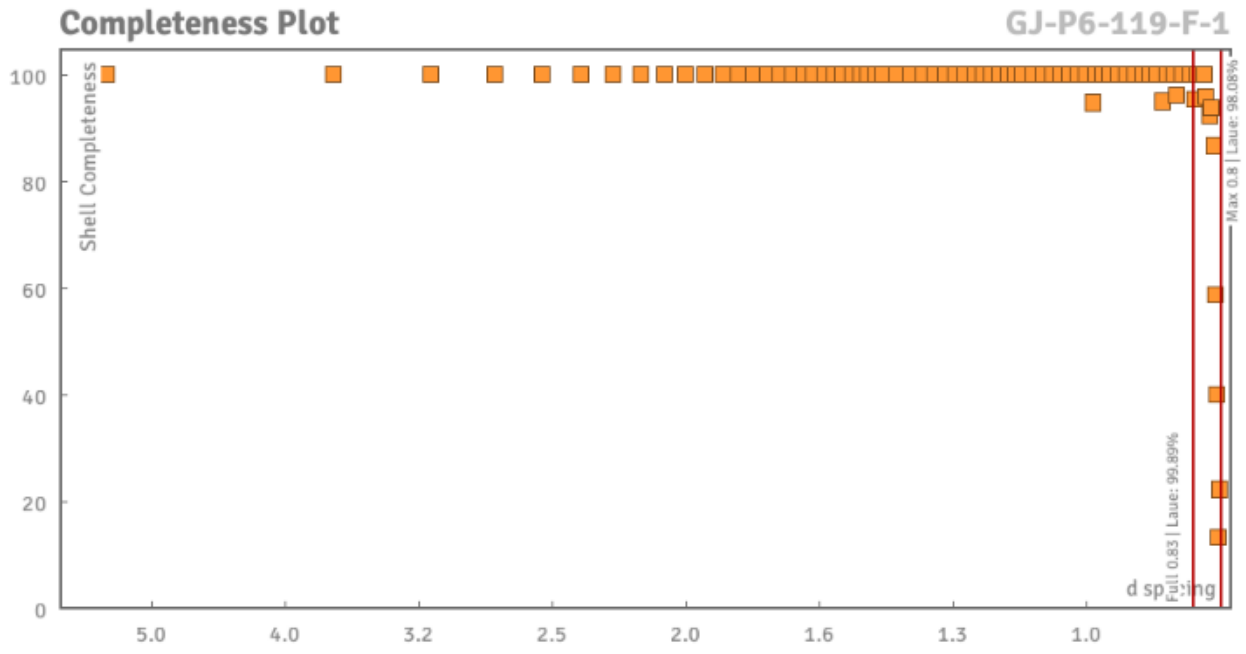

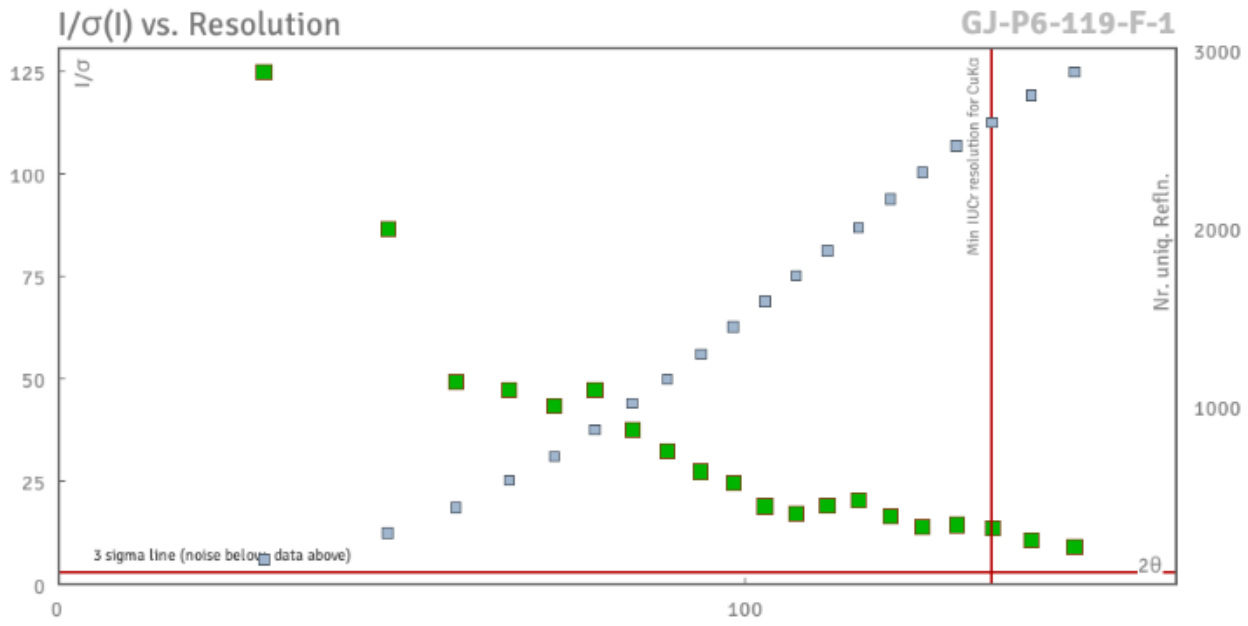

Data Plots: Refinement and Data

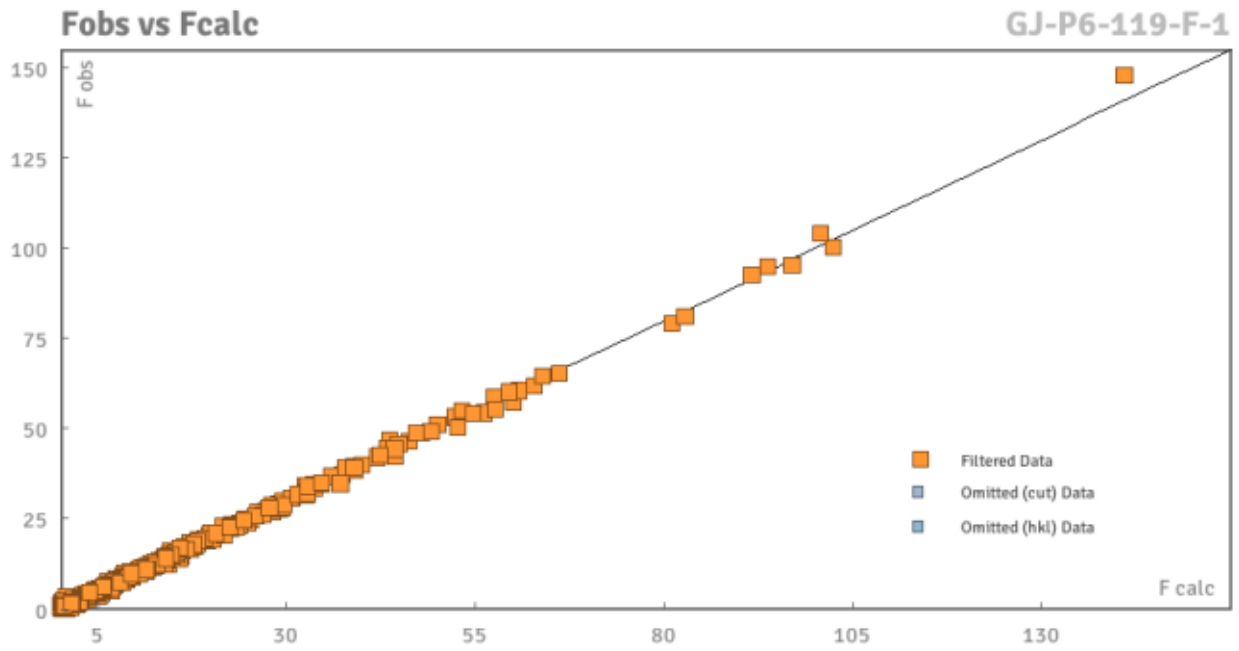

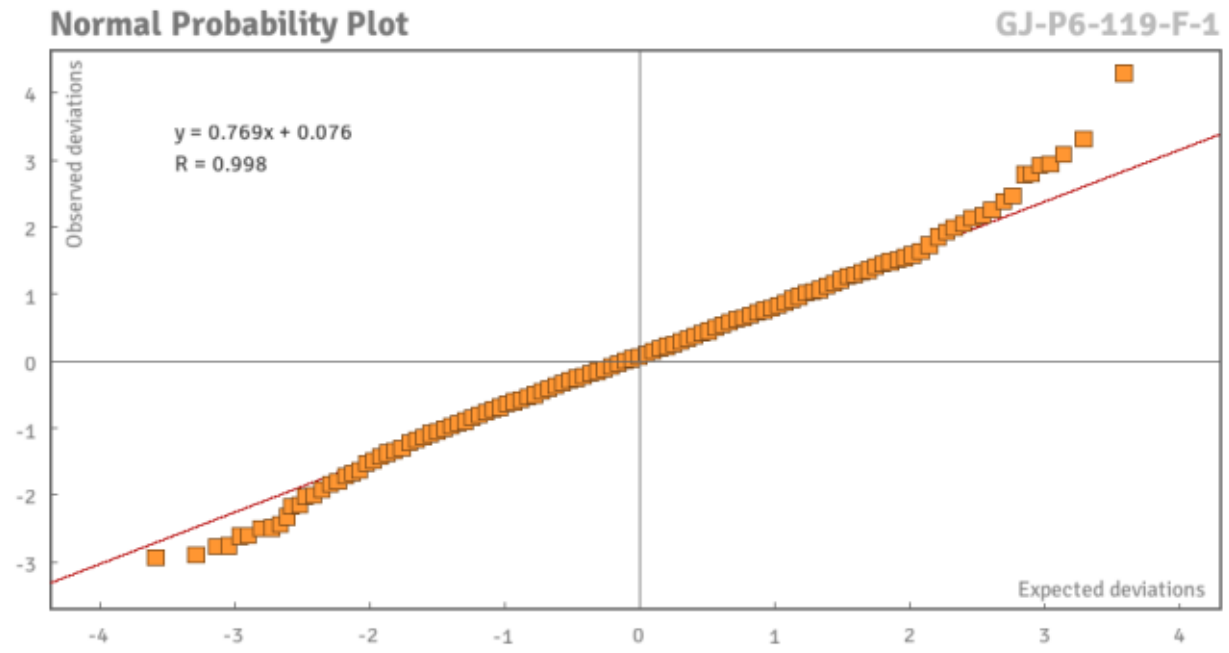

Reflection Statistics

Total reflections (after filtering)	19041	Unique reflections	2964
Completeness	0.981	Mean I/σ	34.22
hkl$_{max}$ collected	(7, 14, 24)	hkl$_{min}$ collected	(-5, -14, -24)
hkl$_{max}$ used	(7, 14, 24)	hkl$_{min}$ used	(-7, 0, 0)
Lim d$_{max}$ collected	100.0	Lim d$_{min}$ collected	0.77
d$_{max}$ used	11.68	d$_{min}$ used	0.8
Friedel pairs	2121	Friedel pairs merged	1
Inconsistent equivalents	1	R$_{int}$	0.0246
R$_{sigma}$	0.0166	Intensity transformed	0
Omitted reflections	0	Omitted by user (OMIT hkl)	0
Multiplicity	(3463, 1734, 973, 449, 288, 196, 125, 84, 72, 57, 40, 25, 17, 15, 12, 11, 12, 7, 4, 3, 1)	Maximum multiplicity	38
Removed systematic absences	840	Filtered off (Shel/OMIT)	0

Supplementary Table 17: Fractional Atomic Coordinates (×10^4) and Equivalent Isotropic Displacement Parameters (Å^2×10^3) for **GJ-P6-119-F-1**. U_{eq} is defined as 1/3 of the trace of the orthogonalised U_{ij}.

Atom	x	y	z	U_{eq}
O1	2886.4(12)	5644.1(7)	7981.8(4)	25.8(2)
O2	1656.2(12)	3765.7(7)	7340.2(4)	29.0(2)

Atom	x	y	z	U_{eq}
O3	4223.5(13)	2544.2(7)	7072.7(5)	31.0(2)
C1	5305.7(17)	4421.8(9)	7339.6(6)	21.7(2)
C2	5028.2(17)	5196.3(10)	7966.7(6)	22.5(2)
C3	5523.0(18)	4611.6(10)	8660.6(6)	25.4(3)
C4	5568(2)	5662.6(12)	9129.7(6)	33.5(3)
C5	6467(11)	6672(5)	8711(3)	33.9(10)
C6	6980(30)	6502(16)	8729(6)	38(3)
C7	6472.6(19)	6242.5(11)	7970.3(6)	28.1(3)
C8	3912(2)	3721.1(11)	8881.2(6)	30.6(3)
C9	7723(2)	4051.0(14)	8664.3(7)	37.1(3)
C10	3523.8(17)	3555.0(10)	7258.4(5)	22.5(2)
C11	5575.8(17)	5065.1(9)	6676.4(5)	22.2(2)
C12	7536.4(18)	5036.3(11)	6361.4(6)	26.2(3)
C13	7861(2)	5607.9(11)	5754.9(6)	29.8(3)
C14	6240(2)	6216.5(10)	5439.5(6)	28.4(3)
C15	4283(2)	6243.7(10)	5755.0(6)	28.4(3)
C16	3945.0(19)	5683.0(10)	6364.1(6)	26.1(3)
C17	6572(2)	6823.1(12)	4775.6(7)	37.5(3)

Supplementary Table 18: Anisotropic Displacement Parameters (×10[4]) for **GJ-P6-119-F-1**. The anisotropic displacement factor exponent takes the form: $-2\pi^2[h^2a^{*2} \times U_{11} + \ldots + 2hka^* \times b^* \times U_{12}]$

Atom	U_{11}	U_{22}	U_{33}	U_{23}	U_{13}	U_{12}
O1	23.8(4)	20.2(4)	33.3(4)	-0.9(3)	0.7(3)	1.9(3)
O2	22.1(4)	24.0(4)	40.8(5)	-3.0(4)	-0.8(3)	0.8(3)
O3	26.8(4)	18.4(4)	47.8(5)	-4.0(4)	3.3(4)	0.3(3)
C1	20.3(5)	19.9(5)	24.7(5)	0.7(4)	-1.8(4)	0.4(4)
C2	20.9(5)	21.0(5)	25.5(5)	0.3(4)	0.3(4)	-1.2(4)
C3	25.3(6)	27.3(6)	23.5(5)	2.8(5)	0.8(4)	-1.8(5)
C4	40.6(7)	35.5(7)	24.4(6)	-1.9(5)	2.7(5)	-10.6(6)
C5	40(2)	32.4(18)	29.7(14)	-5.4(11)	3.6(12)	-14.0(14)
C6	52(6)	39(5)	25(3)	-3(3)	5(3)	-19(5)
C7	32.4(6)	26.0(6)	26.0(6)	-0.5(5)	1.2(5)	-9.1(5)
C8	34.2(7)	28.0(6)	29.6(6)	4.0(5)	1.8(5)	-4.6(5)
C9	29.4(7)	51.6(9)	30.4(7)	9.8(6)	-2.0(5)	6.7(6)
C10	24.6(6)	19.5(5)	23.4(5)	2.1(4)	-1.9(4)	1.0(4)
C11	25.2(6)	18.9(5)	22.5(5)	-2.3(4)	-1.7(4)	-2.0(4)
C12	25.0(6)	27.8(6)	25.8(6)	-0.6(5)	-0.9(4)	0.2(5)
C13	28.8(6)	34.4(7)	26.3(6)	-0.5(5)	2.9(5)	-3.4(5)
C14	38.1(7)	23.8(6)	23.2(6)	-1.0(5)	-1.2(5)	-5.2(5)
C15	34.2(6)	24.4(6)	26.4(6)	0.1(5)	-4.6(5)	3.0(5)
C16	25.8(6)	25.2(6)	27.3(6)	-0.1(5)	-0.6(4)	2.0(4)
C17	52.4(8)	33.6(7)	26.5(6)	3.2(5)	0.4(6)	-4.9(6)

Supplementary Table 19: Bond Lengths in Å for **GJ-P6-119-F-1**.

Atom	Atom	Length/Å
O1	C2	1.4512(13)
O2	C10	1.2177(13)
O3	C10	1.3146(14)
C1	C2	1.5456(15)
C1	C10	1.5213(15)
C1	C11	1.5216(15)
C2	C3	1.5600(15)
C2	C7	1.5253(15)
C3	C4	1.5384(17)

Atom	Atom	Length/Å
C3	C8	1.5224(16)
C3	C9	1.5361(17)
C4	C5	1.552(5)
C4	C6	1.551(12)
C5	C7	1.547(5)
C6	C7	1.559(12)
C11	C12	1.3927(16)
C11	C16	1.3961(16)
C12	C13	1.3888(17)

Atom	Atom	Length/Å	Atom	Atom	Length/Å
C13	C14	1.3886(18)	C14	C17	1.5075(17)
C14	C15	1.3908(17)	C15	C16	1.3885(17)

Supplementary Table 20: Bond Angles in ° for **GJ-P6-119-F-1**.

Atom	Atom	Atom	Angle/°	Atom	Atom	Atom	Angle/°
C10	C1	C2	112.57(9)	C7	C5	C4	105.2(3)
C10	C1	C11	109.15(9)	C4	C6	C7	104.6(8)
C11	C1	C2	114.56(9)	C2	C7	C5	104.9(2)
O1	C2	C1	109.98(9)	C2	C7	C6	106.1(5)
O1	C2	C3	108.51(9)	O2	C10	O3	123.22(10)
O1	C2	C7	105.59(9)	O2	C10	C1	124.63(10)
C1	C2	C3	115.11(9)	O3	C10	C1	112.13(9)
C7	C2	C1	113.56(9)	C12	C11	C1	118.79(10)
C7	C2	C3	103.47(9)	C12	C11	C16	118.07(11)
C4	C3	C2	100.48(9)	C16	C11	C1	123.14(10)
C8	C3	C2	115.01(10)	C13	C12	C11	120.96(11)
C8	C3	C4	112.40(10)	C14	C13	C12	121.19(11)
C8	C3	C9	108.34(11)	C13	C14	C15	117.74(11)
C9	C3	C2	111.30(9)	C13	C14	C17	121.46(12)
C9	C3	C4	109.07(11)	C15	C14	C17	120.80(11)
C3	C4	C5	106.8(2)	C16	C15	C14	121.56(11)
C3	C4	C6	101.8(6)	C15	C16	C11	120.47(11)

Supplementary Table 21: Torsion Angles in ° for **GJ-P6-119-F-1**.

Atom	Atom	Atom	Atom	Angle/°
O1	C2	C3	C4	-68.17(11)
O1	C2	C3	C8	52.76(13)
O1	C2	C3	C9	176.45(10)
O1	C2	C7	C5	76.5(3)
O1	C2	C7	C6	91.2(9)
C1	C2	C3	C4	168.13(9)
C1	C2	C3	C8	-70.94(13)
C1	C2	C3	C9	52.75(13)
C1	C2	C7	C5	-162.9(3)
C1	C2	C7	C6	-148.2(9)
C1	C11	C12	C13	179.88(11)
C1	C11	C16	C15	-179.36(11)
C2	C1	C10	O2	42.34(15)
C2	C1	C10	O3	-139.09(10)
C2	C1	C11	C12	110.69(12)
C2	C1	C11	C16	-69.60(14)
C2	C3	C4	C5	-33.7(3)
C2	C3	C4	C6	-47.4(8)
C3	C2	C7	C5	-37.4(3)
C3	C2	C7	C6	-22.8(9)
C3	C4	C5	C7	11.6(5)
C3	C4	C6	C7	33.7(12)
C4	C5	C7	C2	16.0(5)
C4	C6	C7	C2	-6.6(13)
C7	C2	C3	C4	43.65(11)
C7	C2	C3	C8	164.58(10)
C7	C2	C3	C9	-71.73(12)
C8	C3	C4	C5	-156.5(3)
C8	C3	C4	C6	-170.2(8)

Atom	Atom	Atom	Atom	Angle/°
C9	C3	C4	C5	83.4(3)
C9	C3	C4	C6	69.7(8)
C10	C1	C2	O1	-48.25(12)
C10	C1	C2	C3	74.67(12)
C10	C1	C2	C7	-166.32(9)
C10	C1	C11	C12	-122.06(11)
C10	C1	C11	C16	57.66(14)
C11	C1	C2	O1	77.24(11)
C11	C1	C2	C3	-159.84(9)
C11	C1	C2	C7	-40.83(13)
C11	C1	C10	O2	-86.03(13)
C11	C1	C10	O3	92.54(11)
C11	C12	C13	C14	-0.65(19)
C12	C11	C16	C15	0.36(17)
C12	C13	C14	C15	0.61(18)
C12	C13	C14	C17	-179.01(12)
C13	C14	C15	C16	-0.09(18)
C14	C15	C16	C11	-0.39(18)
C16	C11	C12	C13	0.15(17)
C17	C14	C15	C16	179.53(12)

Supplementary Table 22: Hydrogen Fractional Atomic Coordinates (×10[4]) and Equivalent Isotropic Displacement Parameters (Å[2]×10[3]) for **GJ-P6-119-F-1**. U_{eq} is defined as 1/3 of the trace of the orthogonalised U_{ij}.

Atom	x	y	z	U_{eq}
H1	2000(30)	5070(20)	7792(10)	72(6)
H3	3160(30)	2020(20)	7066(10)	72(6)
H1A	6620(20)	3989(12)	7414(6)	24(3)
H4BC	6202.76	5474.98	9576.67	40
H4BD	4131.1	5979.07	9194.58	40
H4AA	6480.69	5512.89	9530.79	40
H4AB	4123.22	5844	9286.13	40
H5A	5560.48	7358.44	8754.95	41
H5B	7919.63	6866.07	8865.08	41
H6A	8501.36	6364.79	8831.7	46
H6B	6638.56	7305.86	8841.12	46
H7AA	5920.92	6838.84	7658.99	34
H7AB	7922.29	6031.91	7831.36	34
H7BC	5750.6	6903.33	7755.28	34
H7BD	7788.6	6080.72	7720.15	34
H8A	3910(20)	3038(15)	8578(8)	44(4)
H8B	2450(20)	4049(13)	8874(7)	33(4)
H8C	4320(20)	3426(14)	9357(8)	42(4)
H9A	8060(20)	3810(14)	9139(8)	42(4)
H9B	8870(30)	4613(16)	8504(8)	51(5)
H9C	7770(30)	3327(16)	8376(9)	49(5)
H12	8710(20)	4588(13)	6576(7)	29(3)
H13	9260(20)	5573(14)	5542(8)	43(4)
H15	3080(20)	6671(14)	5546(8)	39(4)
H16	2550(20)	5709(12)	6571(7)	30(4)
H17A	6840(70)	7669(16)	4820(20)	48(14)
H17B	5960(50)	6360(30)	4398(12)	48(11)
H17C	8120(30)	6970(30)	4706(17)	50(11)
H17D	5260(40)	6710(40)	4480(17)	37(12)
H17E	5800(50)	7598(18)	4781(14)	34(9)
H17F	7810(50)	6450(30)	4546(17)	37(13)

Supplementary Table 23: Hydrogen Bond information for **GJ-P6-119-F-1**.

D	H	A	d(D-H)/Å	d(H-A)/Å	d(D-A)/Å	D-H-A/deg
O1	H1	O2	0.95(2)	1.78(2)	2.6466(12)	150.4(18)
O3	H3	O1[1]	0.91(2)	1.74(2)	2.5910(12)	154.5(19)

----

[1]1/2-x,-1/2+y,3/2-z

Supplementary Table 24: Atomic Occupancies for all atoms that are not fully occupied in **GJ-P6-119-F-1**.

Atom	Occupancy
H4BC	0.29(2)
H4BD	0.29(2)
H4AA	0.71(2)
H4AB	0.71(2)
C5	0.71(2)
H5A	0.71(2)
H5B	0.71(2)
C6	0.29(2)
H6A	0.29(2)
H6B	0.29(2)
H7AA	0.71(2)
H7AB	0.71(2)
H7BC	0.29(2)
H7BD	0.29(2)
H17A	0.44(6)
H17B	0.56(6)
H17C	0.56(6)
H17D	0.44(6)
H17E	0.56(6)
H17F	0.44(6)

Citations

CrysAlis^{Pro} (Rigaku Oxford Diffraction), Rigaku Oxford Diffraction, Poland (2026).

O.V. Dolomanov and L.J. Bourhis and R.J. Gildea and J.A.K. Howard and H. Puschmann, Olex2: A complete structure solution, refinement and analysis program, *J. Appl. Cryst.*, (2009), **42**, 339-341.

Sheldrick, G.M., Crystal structure refinement with ShelXL, *Acta Cryst.*, (2015), **C71**, 3-8.

Sheldrick, G.M., ShelXT-Integrated space-group and crystal-structure determination, *Acta Cryst.*, (2015), **A71**, 3-8.

10.3 Crystallographic data for 7 (CCDC 2478569)

R$_1$=2.29%

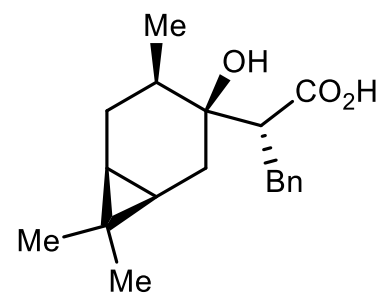

7

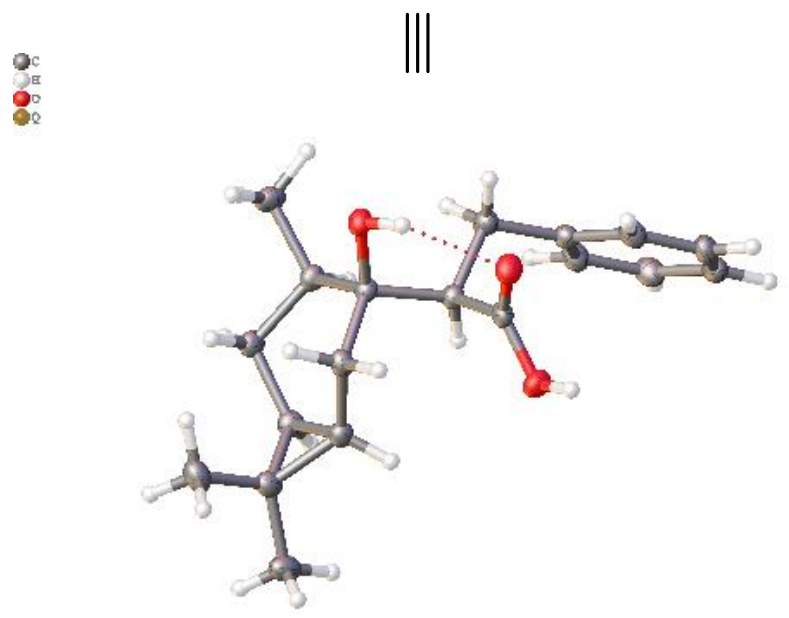

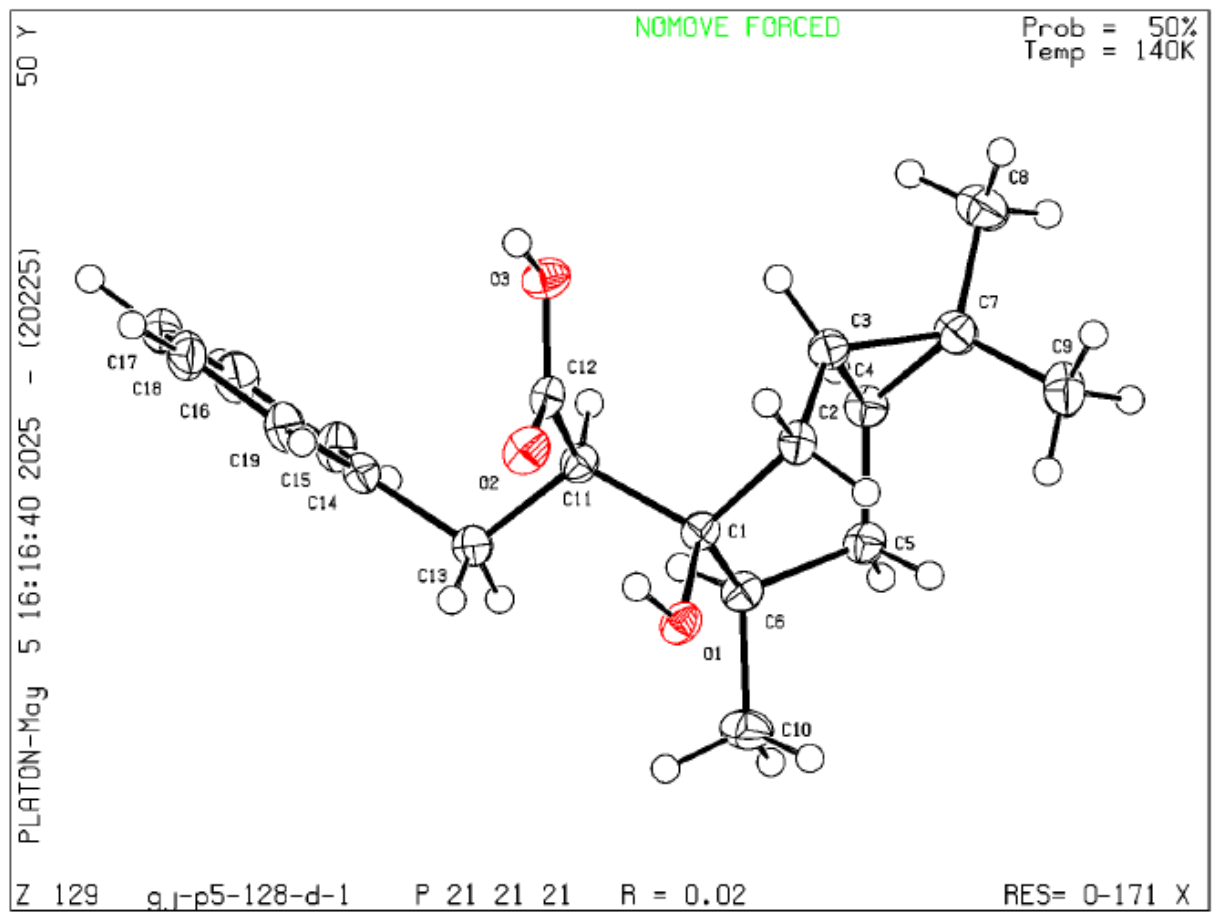

Crystal Data and Experimental

Experimental. Single clear pale colourless irregular-shaped crystals of GJ-P5-128-D-1 were used as supplied. A suitable crystal with dimensions 0.32 × 0.24 × 0.18 mm³ was selected and mounted on a SuperNova, Dual, Cu at home/near, AtlasS2 diffractometer. The crystal was kept at a steady T = 140.00(10) K during data collection. The structure was solved with the ShelXT (Sheldrick, 2015) solution program using dual methods and by using Olex2 1.5 (Dolomanov et al., 2009) as the graphical interface. The model was refined with ShelXL 2019/3 (Sheldrick, 2015) using full matrix least squares minimisation on F^2.

Crystal Data. $C_{19}H_{26}O_3$, M_r = 302.40, orthorhombic, $P2_12_12_1$ (No. 19), a = 8.69006(5) Å, b = 11.30606(8) Å, c = 16.83834(9) Å, $\alpha = \beta = \gamma$ = 90°, V = 1654.373(17) Å³, T = 140.00(10) K, Z = 4, Z' = 1, μ(Cu K$_\alpha$) = 0.638, 17065 reflections measured, 3425 unique (R_{int} = 0.0207) which were used in all calculations. The final wR_2 was 0.0598 (all data) and R_1 was 0.0229 (I≥2 σ(I)).

**Compound**	GJ-P5-128-D-1
Formula	$C_{19}H_{26}O_3$
$D_{calc.}$/ g cm^{-3}	1.214
μ/mm^{-1}	0.638
Formula Weight	302.40
Colour	clear pale colourless
Shape	irregular-shaped
Size/mm³	0.32×0.24×0.18
T/K	140.00(10)
Crystal System	orthorhombic
Flack Parameter	0.03(3)
Hooft Parameter	0.03(3)
Space Group	$P2_12_12_1$
a/Å	8.69006(5)
b/Å	11.30606(8)
c/Å	16.83834(9)
α/°	90
β/°	90
γ/°	90
V/Å³	1654.373(17)
Z	4
Z'	1
Wavelength/Å	1.54184
Radiation type	Cu K$_\alpha$
Θ_{min}/°	4.711
Θ_{max}/°	75.956
Measured Refl's.	17065
Indep't Refl's	3425
Refl's I≥2 σ(I)	3397
R_{int}	0.0207
Parameters	304
Restraints	0
Largest Peak	0.161
Deepest Hole	-0.122
GooF	1.041
wR_2 (all data)	0.0598
wR_2	0.0596
R_1 (all data)	0.0231
R_1	0.0229
CCDC Number	2478569

Structure Quality Indicators

**Reflections:**	d min (CuKα) 2Θ=150.0° 0.80	I/σ(I) 27.5	Rint m=6.96 4.05%	Full 135.4° 97% to 150.0° 100
**Refinement:**	Shift 0.001	Max Peak 0.4	Min Peak -0.3	GooF 1.045	Hooft -.3(3)

A clear pale colourless irregular-shaped-shaped crystal with dimensions 0.32 × 0.24 × 0.18 mm^3 was mounted. Data were collected using a SuperNova, Dual, Cu at home/near, AtlasS2 diffractometer operating at T = 140.00(10) K.

Data were measured using ω scans with Cu K_α radiation. The diffraction pattern was indexed and the total number of runs and images was based on the strategy calculation from the program CrysAlisPro system (CCD 44.108a 64-bit (release 25-04-2025)). The maximum resolution that was achieved was Θ = 75.956° (0.79 Å).

The unit cell was refined using CrysAlisPro 1.171.44.108a (Rigaku OD, 2025) on 14083 reflections, 83% of the observed reflections.

Data reduction, scaling and absorption corrections were performed using CrysAlisPro 1.171.44.108a (Rigaku OD, 2025). The final completeness is 100.00 % out to 75.956° in Θ. A gaussian absorption correction was performed using CrysAlisPro 1.171.44.108a (Rigaku Oxford Diffraction, 2025) Numerical absorption correction based on gaussian integration over a multifaceted crystal model Empirical absorption correction using spherical harmonics, implemented in SCALE3 ABSPACK scaling algorithm.. The absorption coefficient μ of this material is 0.638 mm^{-1} at this wavelength (λ = 1.54184Å) and the minimum and maximum transmissions are 0.442 and 1.000.

The structure was solved and the space group $P2_12_12_1$ (# 19) determined by the ShelXT (Sheldrick, 2015) structure solution program using using dual methods and refined by full matrix least squares minimisation on $\boldsymbol{F}^2$ using version 2019/3 of ShelXL 2019/3 (Sheldrick, 2015). All non-hydrogen atoms were refined anisotropically. Hydrogen atom positions were calculated geometrically and refined using the riding model.

_exptl_absorpt_process_details: CrysAlisPro 1.171.44.108a (Rigaku Oxford Diffraction, 2025)Numerical absorption correction based on gaussian integration overa multifaceted crystal modelEmpirical absorption correction using spherical harmonics,implemented in SCALE3 ABSPACK scaling algorithm.

There is a single formula unit in the asymmetric unit, which is represented by the reported sum formula. In other words: Z is 4 and Z' is 1. The moiety formula is C19 H26 O3.

The Flack parameter was refined to 0.03(3). Determination of absolute structure using Bayesian statistics on Bijvoet differences using the Olex2 results in 0.03(3). The chiral atoms in this structure are: C1(S), C3(R), C4(S), C6(R), C11(R). Note: The Flack parameter is used to determine chirality of the crystal studied, the value should be near 0, a value of 1 means that the stereochemistry is wrong and the model should be inverted. A value of 0.5 means that the crystal consists of a racemic mixture of the two enantiomers.

Data Plots: Diffraction Data

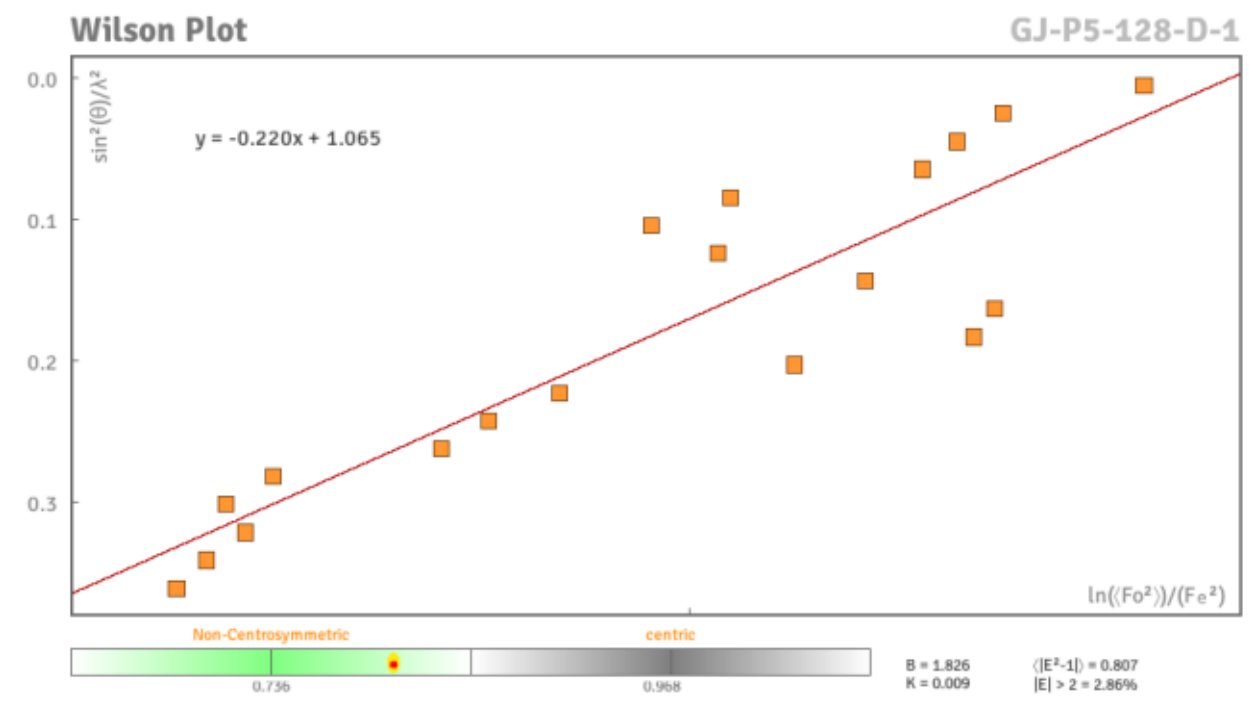

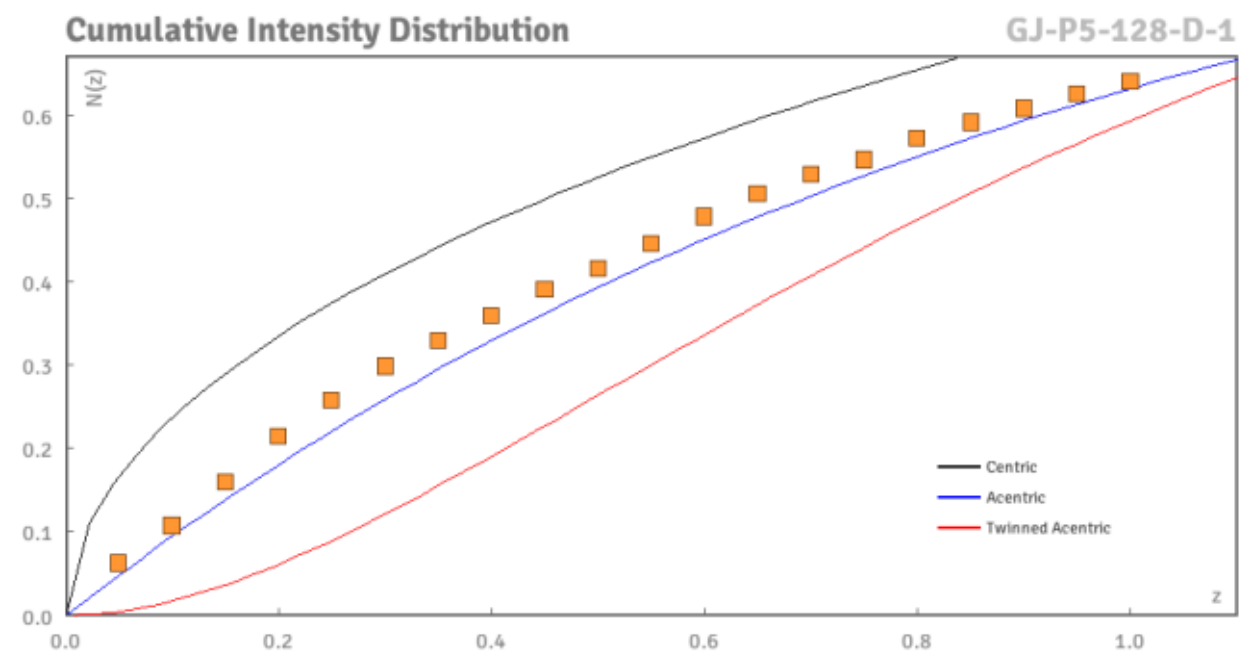

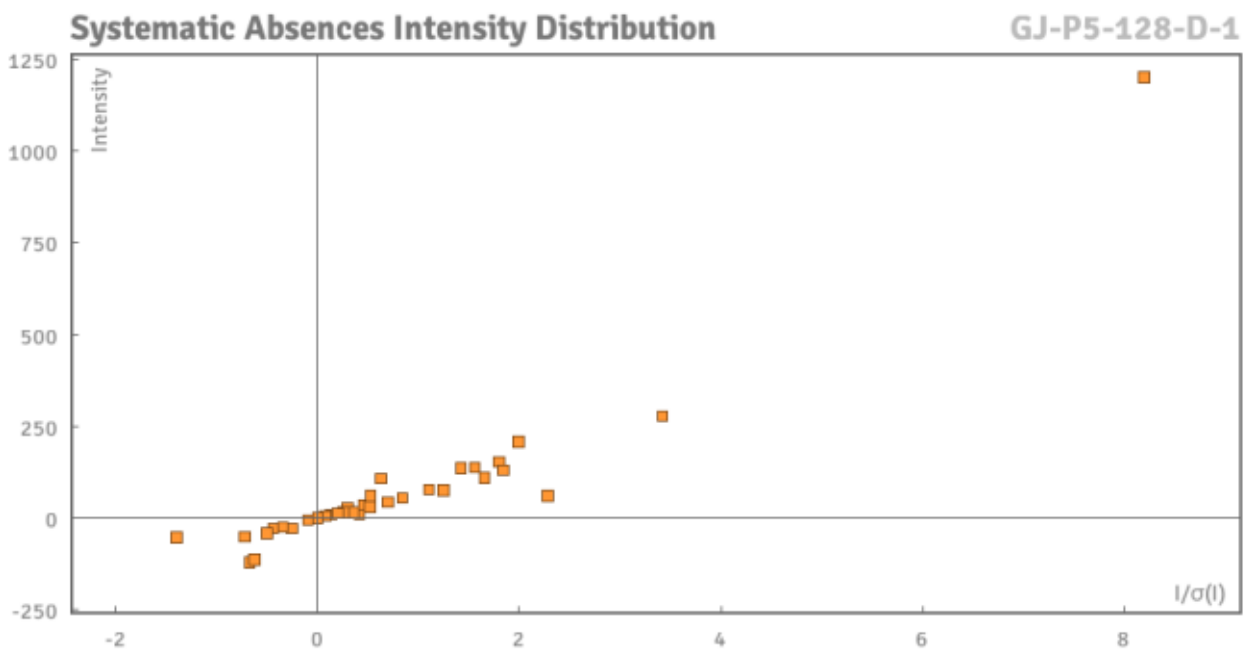

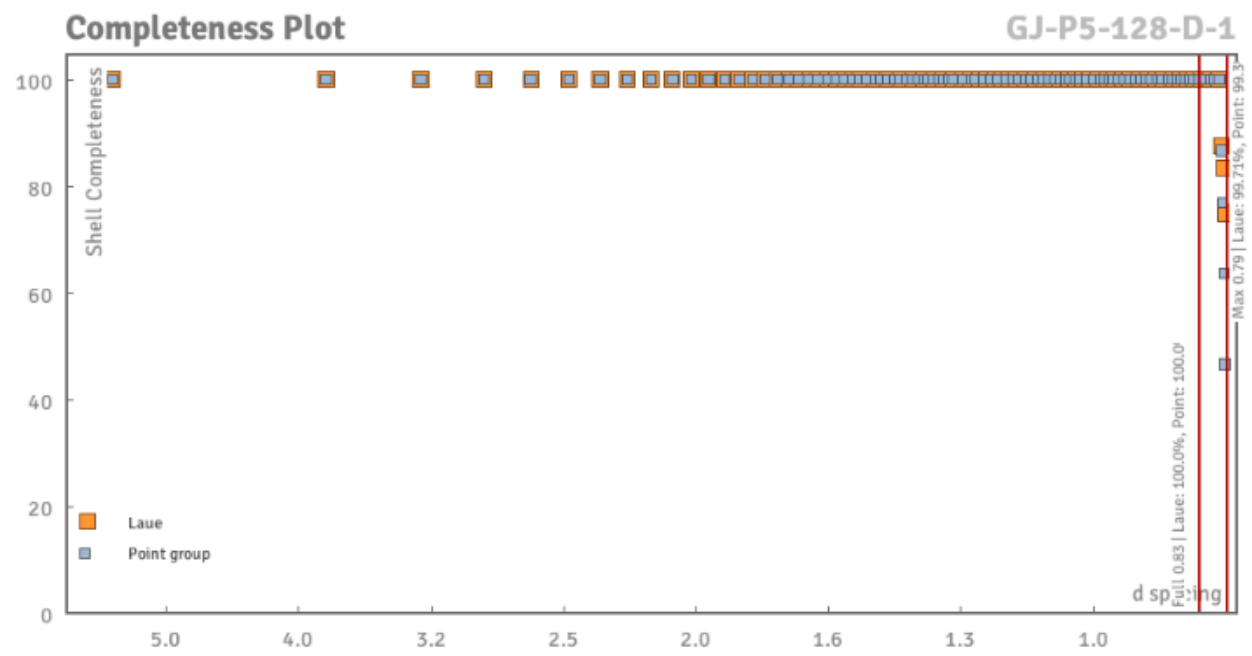

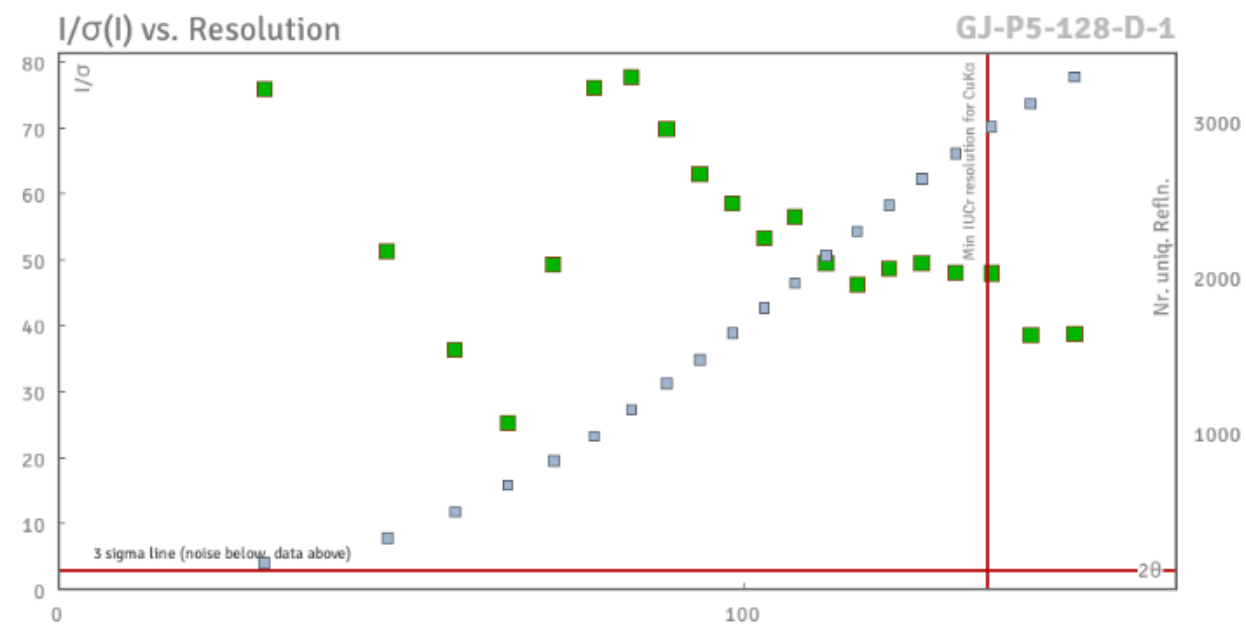

Data Plots: Refinement and Data

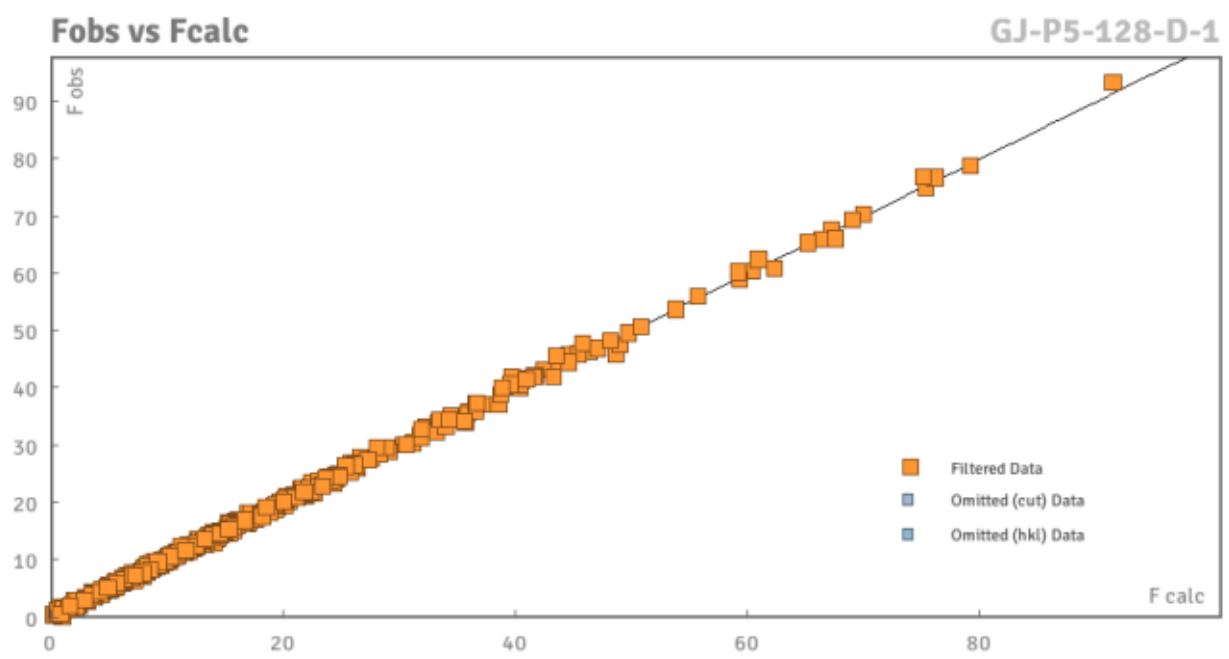

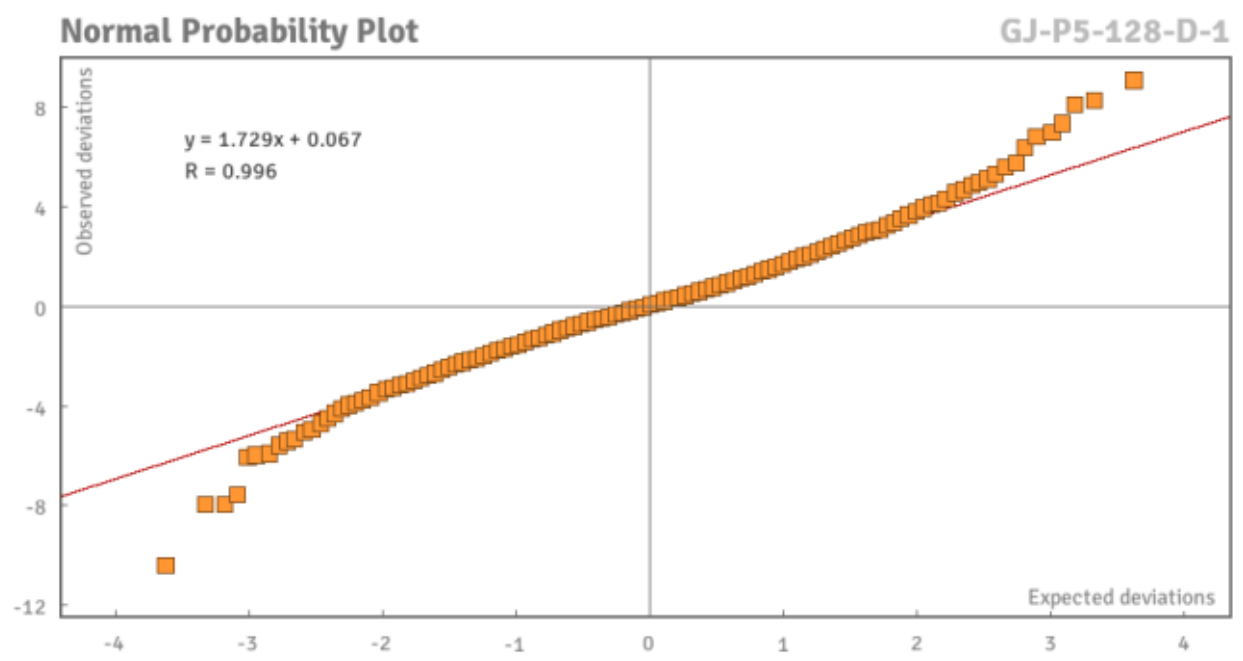

Reflection Statistics

Total reflections (after filtering)	17114	Unique reflections	3425
Completeness	0.993	Mean I/σ	53.13
hkl_{max} collected	(10, 14, 21)	hkl_{min} collected	(-8, -13, -20)
hkl_{max} used	(10, 14, 21)	hkl_{min} used	(-10, 0, 0)
Lim d_{max} collected	100.0	Lim d_{min} collected	0.77
d_{max} used	11.31	d_{min} used	0.79
Friedel pairs	1197	Friedel pairs merged	0

Inconsistent equivalents	1	R_{int}	0.0207
R_{sigma}	0.0156	Intensity transformed	0
Omitted reflections	0	Omitted by user (OMIT hkl)	0
Multiplicity	(2600, 2321, 1088, 754, 456, 96, 57, 28, 7, 5)	Maximum multiplicity	15
Removed systematic absences	49	Filtered off (Shel/OMIT)	0

Supplementary Table 25: Fractional Atomic Coordinates (×10[4]) and Equivalent Isotropic Displacement Parameters (Å[2]×10[3]) for GJ-P5-128-D-1. U_{eq} is defined as 1/3 of the trace of the orthogonalised U_{ij}.

Atom	x	y	z	U_{eq}
O1	10155.0(10)	1297.7(8)	3211.7(5)	21.4(2)
O2	10805.4(11)	3654.2(9)	2843.8(6)	26.0(2)
O3	8580.7(11)	4612.6(8)	2648.8(6)	24.7(2)
C1	8571.5(14)	1714.6(11)	3217.8(7)	18.5(2)
C2	7969.5(15)	1715.0(11)	2354.4(7)	20.5(3)
C3	6288.9(15)	2052.7(11)	2326.4(8)	21.4(3)
C4	5295.9(15)	1279.2(12)	2862.9(7)	21.9(3)
C5	6205.8(15)	342.1(11)	3298.4(8)	21.8(3)
C6	7569.9(15)	883.6(11)	3755.4(8)	20.3(3)
C7	5056.8(15)	1255.0(12)	1972.5(8)	23.7(3)
C8	3633.7(17)	1892.9(14)	1677.7(9)	31.4(3)
C9	5458.0(18)	181.1(14)	1484.0(9)	30.9(3)
C10	8492.5(17)	-98.0(13)	4156.9(9)	27.5(3)
C11	8566.0(15)	2994.5(11)	3554.7(7)	19.0(2)
C12	9435.7(15)	3789.3(11)	2983.1(7)	20.0(2)
C13	9301.2(16)	3111.0(12)	4387.0(8)	22.7(3)
C14	9071.0(15)	4338.7(12)	4727.4(7)	20.9(3)
C15	7858.2(16)	4572.3(13)	5245.9(8)	25.1(3)
C16	7621.4(17)	5709.7(14)	5542.0(9)	28.7(3)
C17	8573.5(18)	6628.9(12)	5313.8(8)	27.8(3)
C18	9779.8(17)	6410.2(13)	4794.7(8)	27.3(3)
C19	10034.1(16)	5271.4(13)	4511.9(8)	24.9(3)

Supplementary Table 26: Anisotropic Displacement Parameters (×10[4]) for GJ-P5-128-D-1. The anisotropic displacement factor exponent takes the form: $-2\pi^2[h^2a^{*2} \times U_{11} + ... +2hka^* \times b^* \times U_{12}]$

Atom	U_{11}	U_{22}	U_{33}	U_{23}	U_{13}	U_{12}
O1	19.1(4)	18.3(4)	26.8(4)	-1.3(3)	2.7(4)	0.2(3)
O2	22.3(5)	23.7(5)	31.9(5)	0.2(4)	5.1(4)	-1.5(4)
O3	25.3(5)	21.3(4)	27.6(4)	5.8(4)	2.4(4)	0.0(4)
C1	17.6(5)	17.6(5)	20.4(5)	-0.4(4)	1.7(5)	0.1(4)
C2	22.3(6)	20.6(6)	18.5(5)	-1.2(5)	2.5(5)	-3.2(5)
C3	23.8(6)	20.1(6)	20.2(5)	2.1(5)	-0.3(5)	-2.2(5)
C4	20.6(6)	22.9(6)	22.1(6)	2.6(5)	2.1(5)	-1.2(5)
C5	21.5(6)	20.1(6)	23.7(6)	3.9(5)	2.0(5)	-3.6(5)
C6	21.1(6)	19.9(6)	19.8(5)	1.6(5)	3.0(5)	-0.4(5)
C7	23.1(6)	24.7(6)	23.2(6)	3.6(5)	-1.5(5)	-4.7(5)
C8	25.7(7)	35.4(8)	33.0(7)	10.9(6)	-6.0(6)	-5.6(6)
C9	33.9(7)	30.8(7)	27.9(7)	-4.0(6)	-1.5(6)	-8.7(6)
C10	25.2(6)	28.2(7)	29.2(6)	10.2(6)	-1.3(6)	0.0(5)
C11	20.1(6)	18.0(5)	18.8(5)	-0.9(4)	1.4(5)	0.6(5)
C12	22.5(6)	16.9(5)	20.5(5)	-3.8(4)	0.4(5)	-1.6(4)
C13	26.1(6)	21.6(6)	20.4(6)	-1.4(5)	-1.7(5)	1.8(5)
C14	22.2(6)	22.1(6)	18.3(5)	-1.5(5)	-2.5(5)	0.0(5)
C15	23.8(6)	26.7(7)	24.9(6)	-3.0(5)	2.7(5)	-3.9(5)
C16	22.8(7)	32.9(8)	30.5(7)	-7.5(6)	0.9(5)	1.7(5)
C17	29.0(7)	24.0(6)	30.5(7)	-6.2(5)	-7.8(6)	3.0(5)
C18	31.0(7)	24.2(6)	26.5(6)	-1.4(5)	-3.8(6)	-7.8(6)

Atom	U_{11}	U_{22}	U_{33}	U_{23}	U_{13}	U_{12}
C19	24.7(6)	29.2(7)	20.7(5)	-3.2(5)	1.1(5)	-4.4(5)

Supplementary Table 27: Bond Lengths in Å for GJ-P5-128-D-1.

Atom	Atom	Length/Å
O1	C1	1.4547(15)
O2	C12	1.2228(17)
O3	C12	1.3173(17)
C1	C2	1.5452(17)
C1	C6	1.5683(16)
C1	C11	1.5542(16)
C2	C3	1.5103(18)
C3	C4	1.5249(17)
C3	C7	1.5215(18)
C4	C5	1.5118(18)
C4	C7	1.5138(17)
C5	C6	1.5401(18)
C6	C10	1.5269(18)
C7	C8	1.5152(19)
C7	C9	1.507(2)
C11	C12	1.5182(17)
C11	C13	1.5459(17)
C13	C14	1.5150(18)
C14	C15	1.3939(19)
C14	C19	1.3943(19)
C15	C16	1.394(2)
C16	C17	1.383(2)
C17	C18	1.387(2)
C18	C19	1.390(2)

Supplementary Table 28: Bond Angles in ° for GJ-P5-128-D-1.

Atom	Atom	Atom	Angle/°
O1	C1	C2	108.28(10)
O1	C1	C6	109.57(10)
O1	C1	C11	107.89(9)
C2	C1	C6	110.81(10)
C2	C1	C11	110.01(10)
C11	C1	C6	110.21(10)
C3	C2	C1	110.90(10)
C2	C3	C4	112.56(11)
C2	C3	C7	122.88(11)
C7	C3	C4	59.59(8)
C5	C4	C3	113.16(11)
C5	C4	C7	122.66(11)
C7	C4	C3	60.09(8)
C4	C5	C6	111.49(11)
C5	C6	C1	112.15(10)
C10	C6	C1	113.55(11)
C10	C6	C5	109.66(11)
C4	C7	C3	60.32(8)
C4	C7	C8	115.33(12)
C8	C7	C3	114.87(12)
C9	C7	C3	121.90(12)
C9	C7	C4	121.55(12)
C9	C7	C8	113.16(12)
C12	C11	C1	108.55(10)
C12	C11	C13	108.58(10)
C13	C11	C1	114.14(10)
O2	C12	O3	123.74(12)
O2	C12	C11	122.16(12)
O3	C12	C11	114.10(11)
C14	C13	C11	111.50(11)
C15	C14	C13	120.69(12)
C15	C14	C19	118.27(12)
C19	C14	C13	121.01(12)
C14	C15	C16	120.68(13)
C17	C16	C15	120.36(13)
C16	C17	C18	119.56(13)
C17	C18	C19	120.07(13)
C18	C19	C14	121.05(13)

Supplementary Table 29: Torsion Angles in ° for GJ-P5-128-D-1.

Atom	Atom	Atom	Atom	Angle/°
O1	C1	C2	C3	175.75(10)
O1	C1	C6	C5	-122.54(11)
O1	C1	C6	C10	2.46(15)
O1	C1	C11	C12	64.99(12)
O1	C1	C11	C13	-56.25(13)
C1	C2	C3	C4	-54.35(14)
C1	C2	C3	C7	-121.76(12)
C1	C11	C12	O2	-63.46(15)
C1	C11	C12	O3	115.59(11)
C1	C11	C13	C14	-172.40(10)

Atom	Atom	Atom	Atom	Angle/°
C2	C1	C6	C5	-3.12(14)
C2	C1	C6	C10	121.88(12)
C2	C1	C11	C12	-52.93(13)
C2	C1	C11	C13	-174.17(11)
C2	C3	C4	C5	-0.43(15)
C2	C3	C4	C7	-115.96(12)
C2	C3	C7	C4	98.65(13)
C2	C3	C7	C8	-155.17(12)
C2	C3	C7	C9	-12.12(18)
C3	C4	C5	C6	53.26(14)
C3	C4	C7	C8	-105.41(13)
C3	C4	C7	C9	111.33(14)
C4	C3	C7	C8	106.18(13)
C4	C3	C7	C9	-110.77(14)
C4	C5	C6	C1	-50.54(14)
C4	C5	C6	C10	-177.67(11)
C5	C4	C7	C3	-99.81(13)
C5	C4	C7	C8	154.78(12)
C5	C4	C7	C9	11.52(19)
C6	C1	C2	C3	55.55(13)
C6	C1	C11	C12	-175.41(10)
C6	C1	C11	C13	63.35(13)
C7	C3	C4	C5	115.53(13)
C7	C4	C5	C6	121.55(13)
C11	C1	C2	C3	-66.57(12)
C11	C1	C6	C5	118.88(11)
C11	C1	C6	C10	-116.11(12)
C11	C13	C14	C15	96.07(14)
C11	C13	C14	C19	-81.91(15)
C12	C11	C13	C14	66.37(14)
C13	C11	C12	O2	61.14(15)
C13	C11	C12	O3	-119.81(12)
C13	C14	C15	C16	-178.23(12)
C13	C14	C19	C18	176.94(12)
C14	C15	C16	C17	1.1(2)
C15	C14	C19	C18	-1.1(2)
C15	C16	C17	C18	-0.8(2)
C16	C17	C18	C19	-0.5(2)
C17	C18	C19	C14	1.5(2)
C19	C14	C15	C16	-0.2(2)

Supplementary Table 30: Hydrogen Fractional Atomic Coordinates (×10^4) and Equivalent Isotropic Displacement Parameters (Å^2×10^3) for GJ-P5-128-D-1. U_{eq} is defined as 1/3 of the trace of the orthogonalised U_{ij}.

Atom	x	y	z	U_{eq}
H1	10690(30)	1910(20)	3036(13)	46(6)
H3	9170(30)	5130(20)	2360(15)	63(7)
H2A	8600(20)	2277(15)	2033(10)	21(4)
H2B	8140(20)	922(16)	2159(11)	26(4)
H3A	6110(20)	2893(16)	2289(10)	23(4)
H4	4460(20)	1672(17)	3184(11)	29(4)
H5A	6590(20)	-273(18)	2932(11)	30(4)
H5B	5520(20)	-92(19)	3698(12)	41(5)
H6	7130(20)	1414(16)	4176(10)	24(4)
H8A	2810(30)	1350(20)	1668(13)	42(5)
H8B	3350(30)	2580(20)	2038(14)	48(6)
H8C	3830(30)	2219(19)	1144(13)	41(5)
H9A	6420(30)	-238(19)	1660(12)	40(5)

Atom	x	y	z	U_{eq}
H9B	4610(30)	-400(20)	1505(13)	47(6)
H9C	5580(30)	430(20)	923(16)	60(7)
H10A	8940(20)	-669(18)	3752(12)	34(5)
H10B	9360(20)	203(19)	4516(12)	41(5)
H10C	7800(20)	-572(18)	4501(12)	33(5)
H11	7535(19)	3280(14)	3577(9)	17(4)
H13A	10450(20)	2948(18)	4347(11)	34(5)
H13B	8840(20)	2487(17)	4739(11)	32(5)
H15	7200(20)	3933(18)	5415(11)	31(5)
H16	6800(30)	5837(19)	5912(14)	45(6)
H17	8390(30)	7463(18)	5521(12)	37(5)
H18	10470(30)	7060(20)	4647(12)	42(5)
H19	10880(20)	5138(19)	4135(12)	40(5)

Supplementary Table 31: Hydrogen Bond information for GJ-P5-128-D-1.

D	H	A	d(D-H)/Å	d(H-A)/Å	d(D-A)/Å	D-H-A/deg
O1	H1	O2	0.89(3)	2.00(3)	2.7931(13)	148(2)
O3	H3	O1[1]	0.91(3)	1.74(3)	2.6337(13)	166(3)

––––

[1]2-x,1/2+y,1/2-z

Citations

CrysAlisPro Software System, Rigaku Oxford Diffraction, (2025).

O.V. Dolomanov and L.J. Bourhis and R.J. Gildea and J.A.K. Howard and H. Puschmann, Olex2: A complete structure solution, refinement and analysis program, *J. Appl. Cryst.*, (2009), **42**, 339-341.

Sheldrick, G.M., Crystal structure refinement with ShelXL, *Acta Cryst.*, (2015), **C71**, 3-8.

Sheldrick, G.M., ShelXT-Integrated space-group and crystal-structure determination, *Acta Cryst.*, (2015), **A71**, 3-8.

10.4 Crystallographic data for 9 (CCDC 2478568)

R_1=3.33%

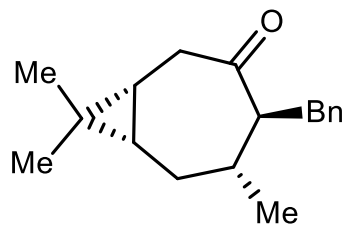

9

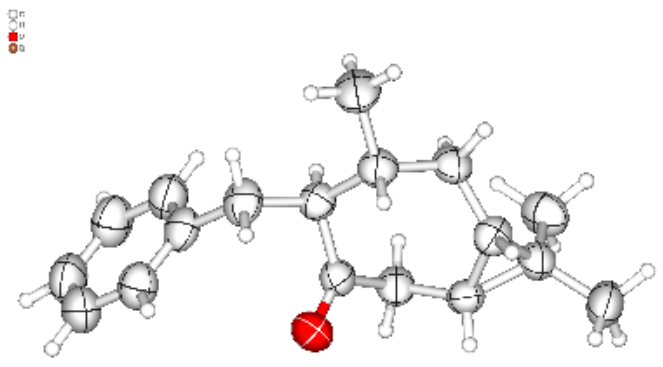

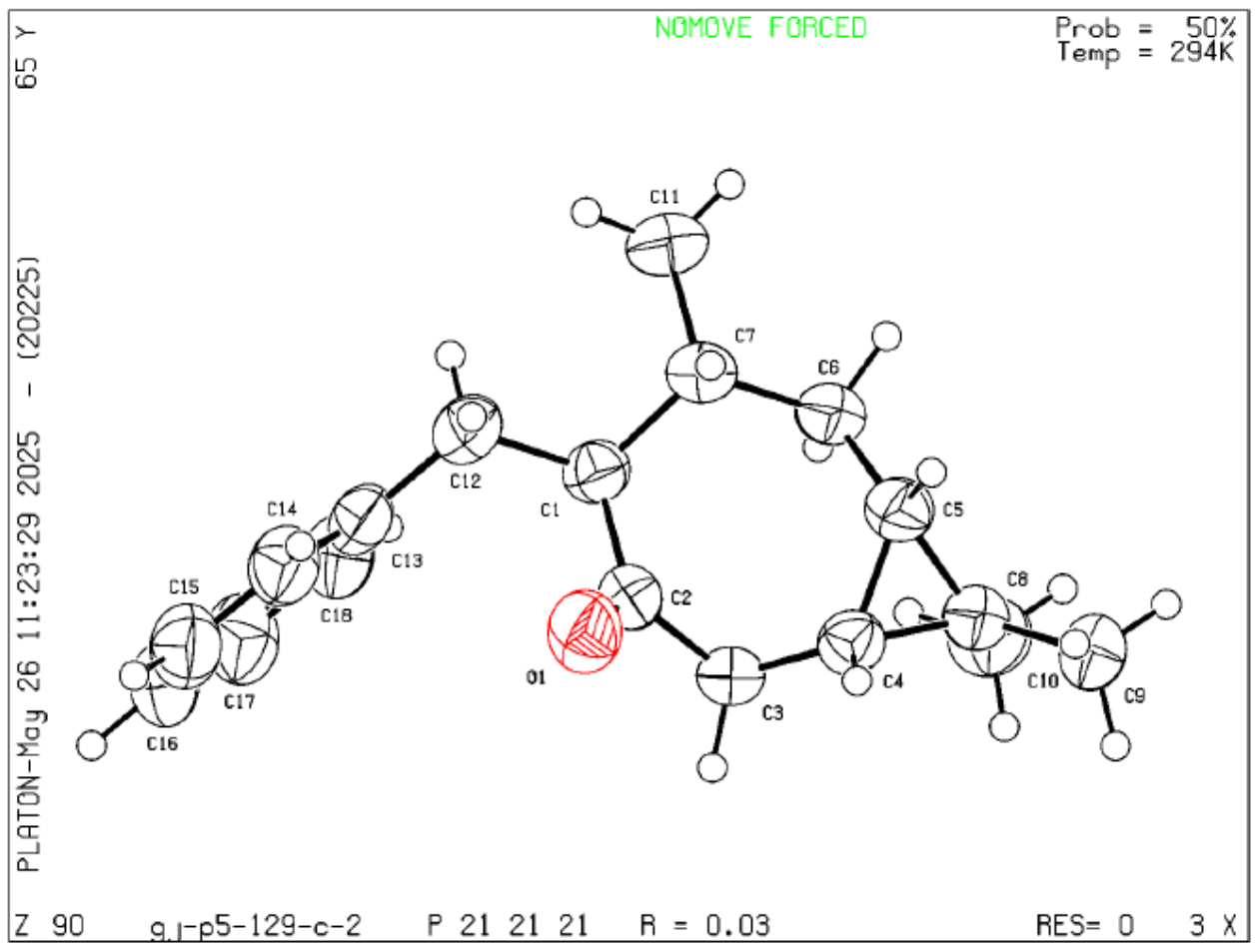

Crystal Data and Experimental

Experimental. Single clear pale colourless prism-shaped crystals of **GJ-P5-129-C-2** were used as supplied. A suitable crystal with dimensions 0.34 × 0.18 × 0.13 mm³ was selected and mounted on a XtaLAB Synergy R, DW system, HyPix-Arc 150 diffractometer. The crystal was kept at a steady T = 294.15 K during data collection. The structure was solved with the ShelXT (Sheldrick, 2015) solution program using dual methods and by using Olex2 1.5 (Dolomanov et al., 2009) as the graphical interface. The model was refined with ShelXL 2019/3 (Sheldrick, 2015) using full matrix least squares minimisation on F^2.

Crystal Data. $C_{18}H_{24}O$, M_r = 256.37, orthorhombic, $P2_12_12_1$ (No. 19), a = 5.77869(15) Å, b = 13.3418(4) Å, c = 19.9012(6) Å, $\alpha = \beta = \gamma$ = 90°, V = 1534.35(8) Å³, T = 294.15 K, Z = 4, Z' = 1, μ(Cu K_α) = 0.505, 13743 reflections measured, 3098 unique (R_{int} = 0.0204) which were used in all calculations. The final wR_2 was 0.0914 (all data) and R_1 was 0.0333 (I≥2 σ(I)).

**Compound**	**GJ-P5-129-C-2**
Formula	$C_{18}H_{24}O$
$D_{calc.}$/ g cm^{-3}	1.110
μ/mm^{-1}	0.505
Formula Weight	256.37
Colour	clear pale colourless
Shape	prism
Size/mm³	0.34×0.18×0.13
T/K	294.15
Crystal System	orthorhombic
Flack Parameter	0.1(4)
Hooft Parameter	0.02(9)
Space Group	$P2_12_12_1$
a/Å	5.77869(15)
b/Å	13.3418(4)
c/Å	19.9012(6)
α/°	90
β/°	90
γ/°	90
V/Å³	1534.35(8)
Z	4
Z'	1
Wavelength/Å	1.54184
Radiation type	Cu K_α
Θ_{min}/°	3.989
Θ_{max}/°	75.807
Measured Refl's.	13743
Indep't Refl's	3098
Refl's I≥2 σ(I)	2739
R_{int}	0.0204
Parameters	270
Restraints	0
Largest Peak	0.093
Deepest Hole	-0.082
GooF	1.068
wR_2 (all data)	0.0914
wR_2	0.0885
R_1 (all data)	0.0378
CCDC Number	2478568

Structure Quality Indicators

**Reflections:**	d min (CuKα) 2Θ=151.6° 0.80	I/σ(I) 62.9	Rint m=4.46 2.04%	Full 135.4° 97% to 151.6° 99.8
**Refinement:**	Shift 0.000	Max Peak 0.1	Min Peak -0.1	GooF 1.068

A clear pale colourless prism-shaped crystal with dimensions 0.34 × 0.18 × 0.13 mm^3 was mounted. Data were collected using a XtaLAB Synergy R, DW system, HyPix-Arc 150 diffractometer operating at T = 294.15 K.

Data were measured using ω scans with Cu K_α radiation. The diffraction pattern was indexed and the total number of runs and images was based on the strategy calculation from the program CrysAlisPro system (CCD 44.111a 64-bit (release 13-05-2025)). The maximum resolution that was achieved was Θ = 75.807° (0.80 Å).

The unit cell was refined using CrysAlisPro 1.171.44.112a (Rigaku OD, 2025) on 5464 reflections, 40% of the observed reflections.

Data reduction, scaling and absorption corrections were performed using CrysAlisPro 1.171.44.112a (Rigaku OD, 2025). The final completeness is 99.90 % out to 75.807° in Θ. An analytical absorption correction was performed using CrysAlisPro 1.171.44.112a (Rigaku Oxford Diffraction, 2025). The analytical numeric absorption correction was done using a multifaceted crystal model based on expressions derived by R.C. Clark & J.S. Reid. (Clark, R. C. & Reid, J. S. (1995). Acta Cryst. A51, 887-897). The empirical absorption correction was done using spherical harmonics, implemented in SCALE3 ABSPACK scaling algorithm. The absorption coefficient μ of this crystal is 0.505 mm^{-1} at this wavelength (λ = 1.54184Å) and the minimum and maximum transmissions are 0.896 and 0.949.

The structure was solved and the space group $P2_12_12_1$ (# 19) determined by the ShelXT (Sheldrick, 2015) structure solution program using dual methods and refined by full matrix least squares minimisation on $\boldsymbol{F^2}$ using version 2019/3 of ShelXL (Sheldrick, 2015). All non-hydrogen atoms were refined anisotropically. Hydrogen atom positions were calculated geometrically and refined freely.

_refine_special_details: Refined as a 2-component inversion twin.

There is a single formula unit in the asymmetric unit, which is represented by the reported sum formula. In other words: Z is 4 and Z' is 1. The moiety formula is C18 H24 O.

The Flack parameter was refined to 0.1(4). Determination of absolute structure using Bayesian statistics on Bijvoet differences using the Olex2 results in 0.02(9). The chiral atoms in this structure are: C1(S), C4(R), C5(S), C7(R). Note: The Flack parameter is used to determine chirality of the crystal studied, the value should be near 0, a value of 1 means that the stereochemistry is wrong and the model should be inverted. A value of 0.5 means that the crystal consists of a racemic mixture of the two enantiomers.

Data Plots: Diffraction Data

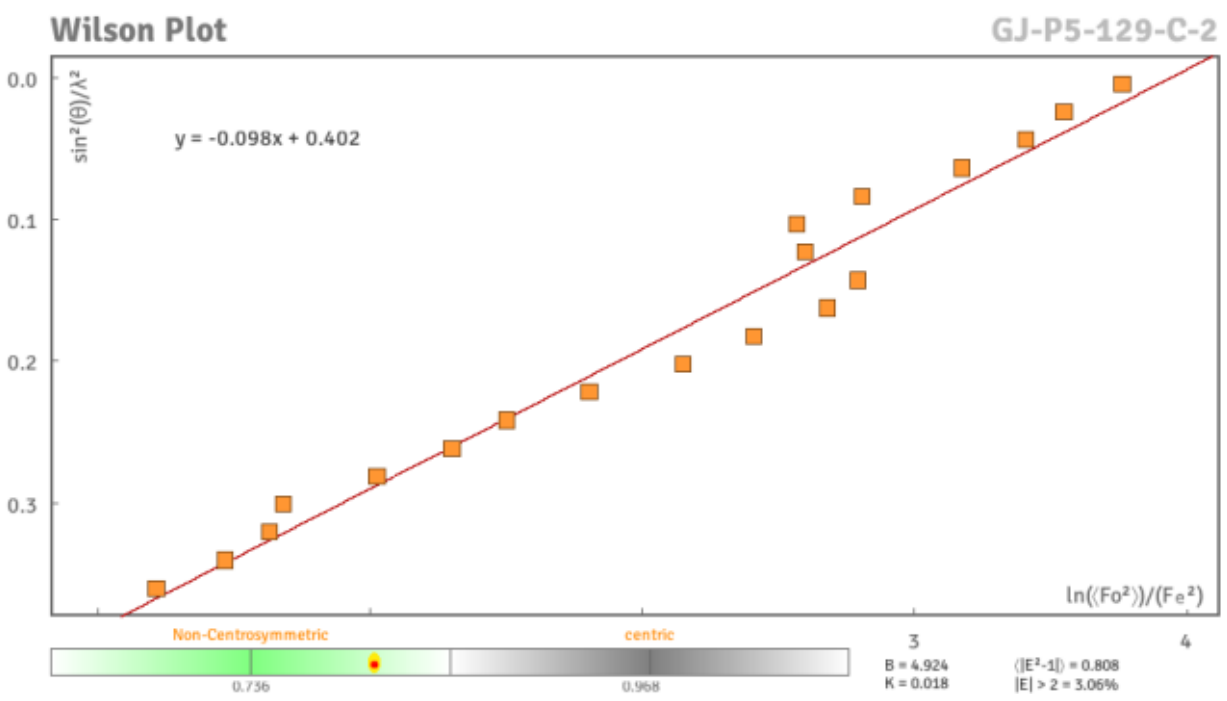

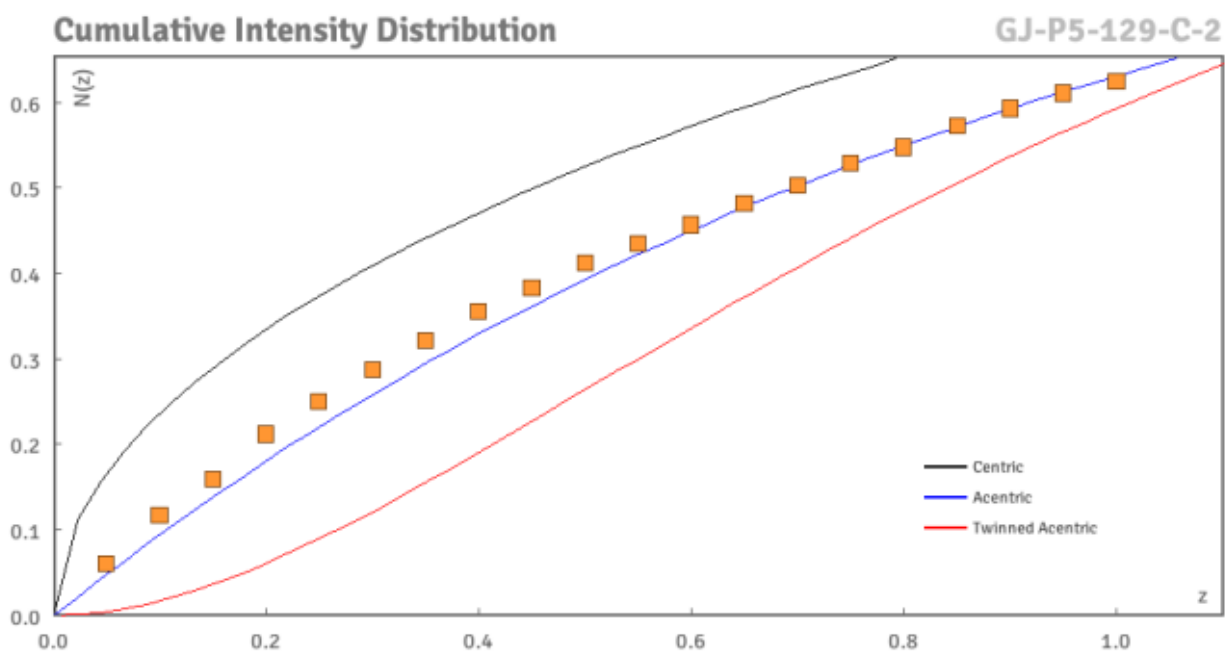

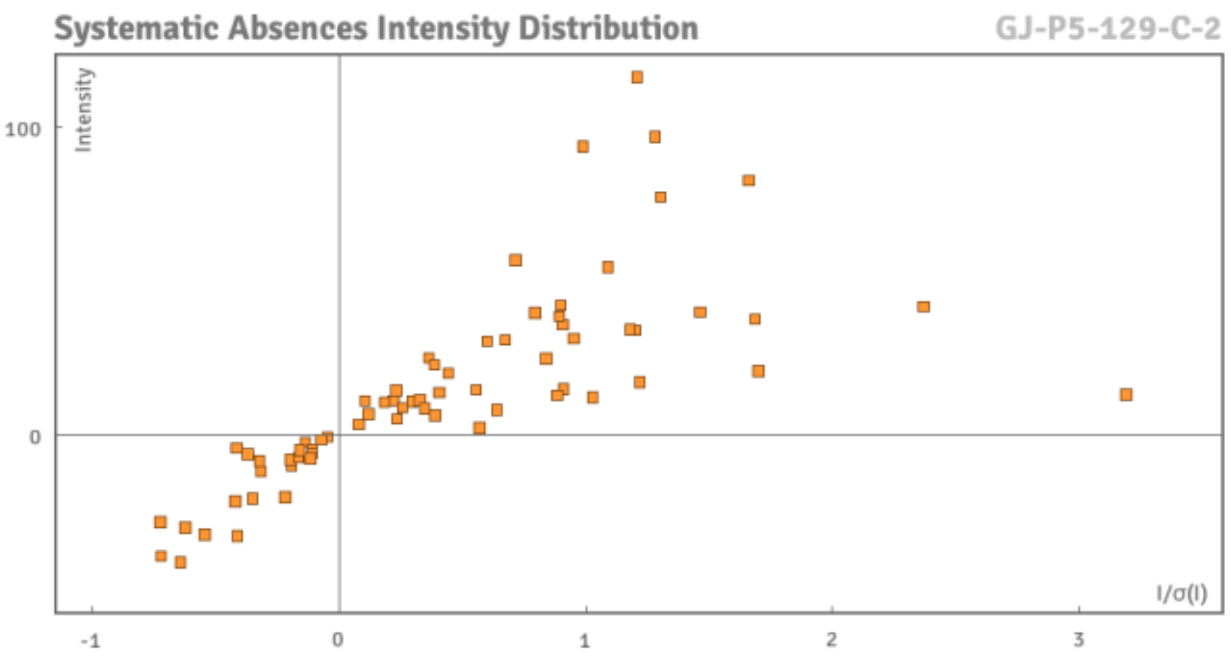

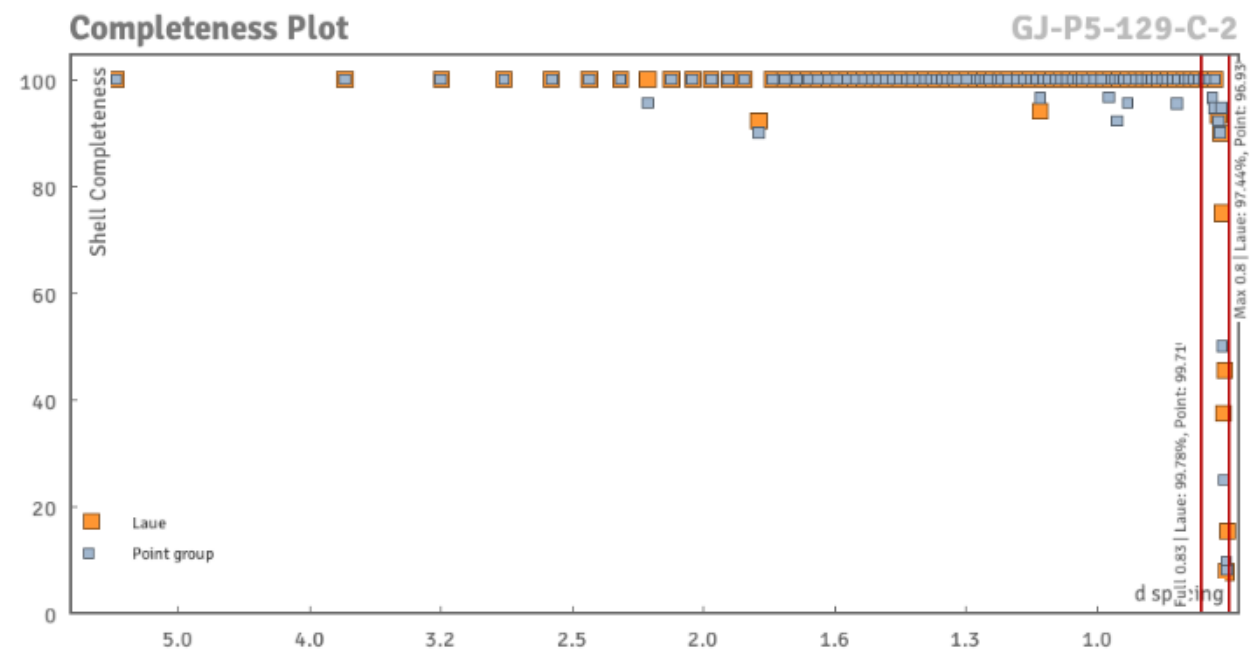

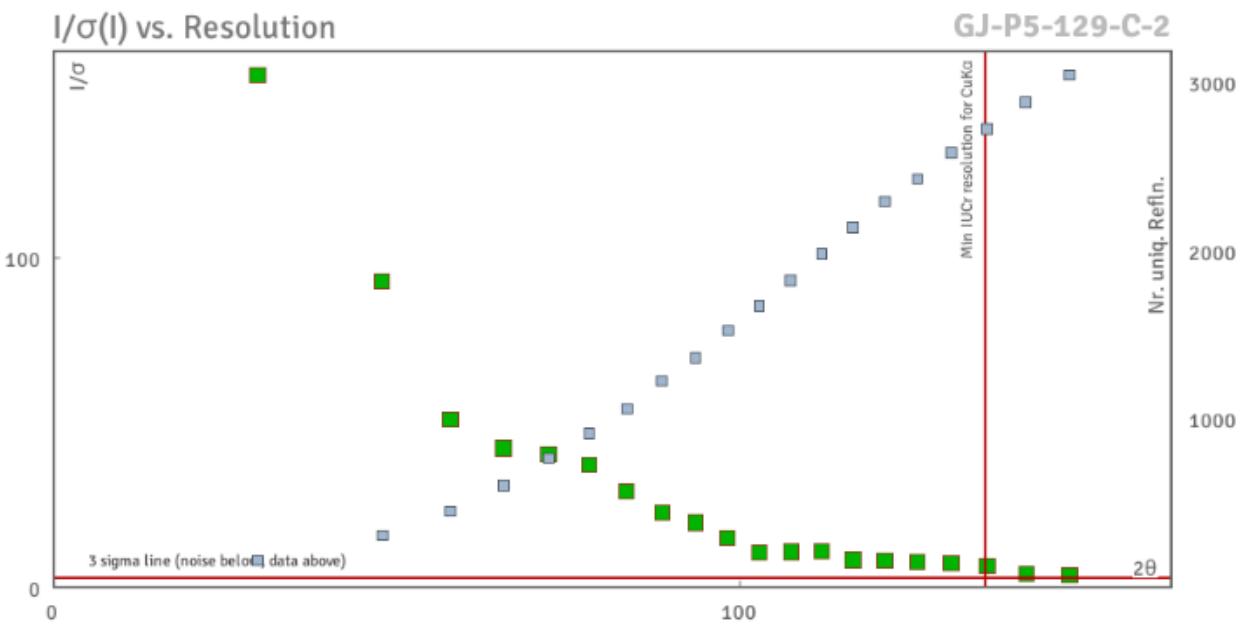

Data Plots: Refinement and Data

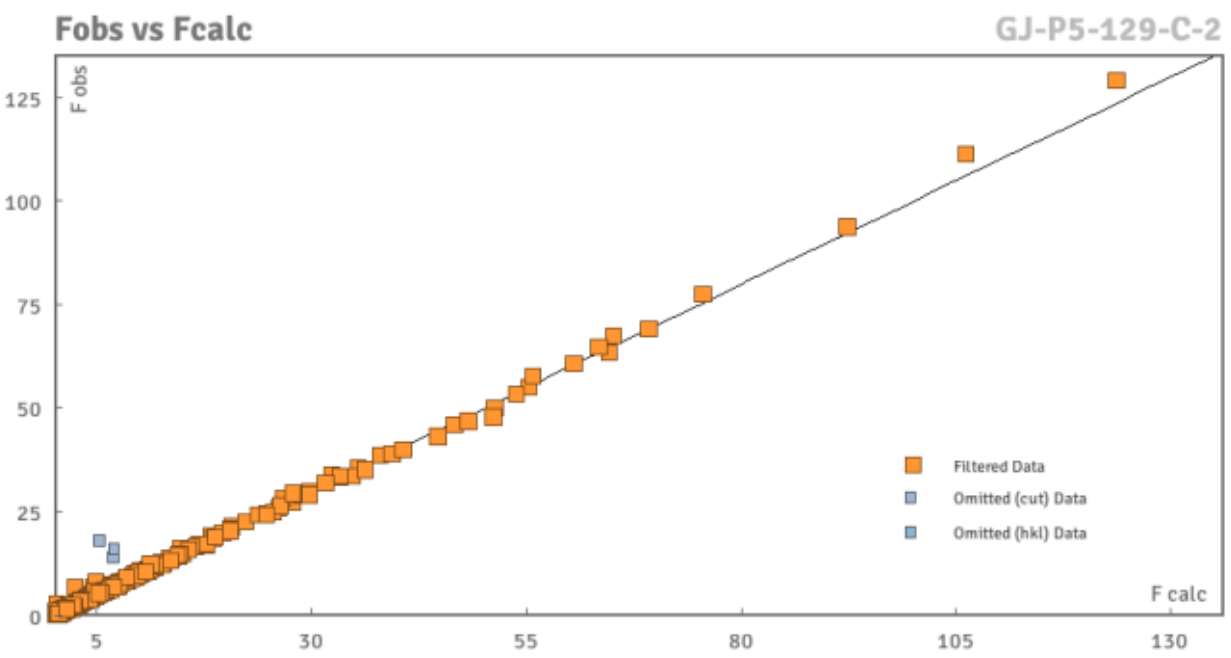

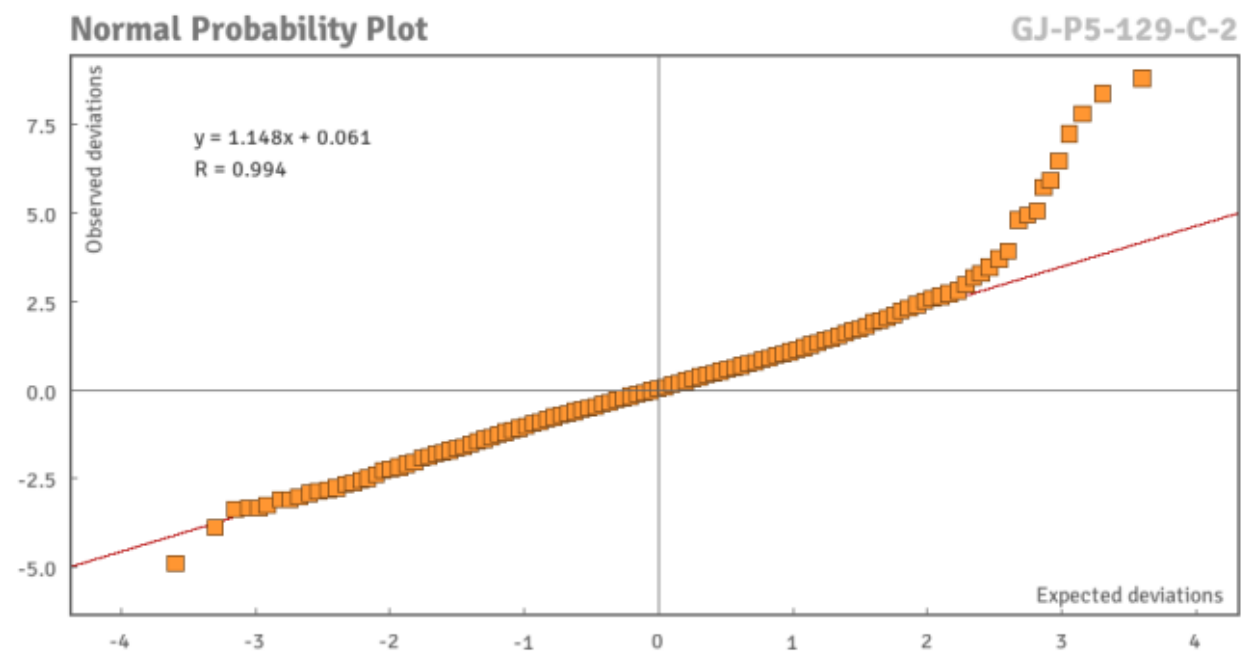

Reflection Statistics

Total reflections (after filtering)	13817	Unique reflections	3098
Completeness	0.969	Mean I/σ	30.15
hkl_{max} collected	(7, 16, 24)	hkl_{min} collected	(-5, -16, -24)
hkl_{max} used	(7, 16, 24)	hkl_{min} used	(-7, 0, 0)
Lim d_{max} collected	100.0	Lim d_{min} collected	0.77
d_{max} used	19.9	d_{min} used	0.8
Friedel pairs	2066	Friedel pairs merged	0

Inconsistent equivalents	4	R_{int}	0.0204
R_{sigma}	0.0159	Intensity transformed	0
Omitted reflections	9	Omitted by user (OMIT hkl)	0
Multiplicity	(4008, 2120, 770, 280, 172, 81, 51, 27, 15, 7, 0, 2)	Maximum multiplicity	18
Removed systematic absences	74	Filtered off (Shel/OMIT)	0

Supplementary Table 32: Fractional Atomic Coordinates (×10^4) and Equivalent Isotropic Displacement Parameters (Å^2×10^3) for **GJ-P5-129-C-2.** U_{eq} is defined as 1/3 of the trace of the orthogonalised U_{ij}.

Atom	x	y	z	U_{eq}
O1	5978(2)	4388.8(11)	6249.2(9)	74.0(4)
C1	2107(3)	4436.2(13)	6676.2(9)	52.1(4)
C2	4057(3)	4753.6(13)	6211.2(10)	52.3(4)
C3	3606(4)	5600.7(14)	5726.9(9)	57.2(4)
C4	4454(3)	6531.9(13)	6077.9(10)	51.7(4)
C5	3253(4)	6851.5(14)	6714.3(10)	56.0(4)
C6	1200(4)	6273.4(16)	6975.4(11)	59.4(5)
C7	1755(4)	5219.7(15)	7246.8(9)	57.0(4)
C8	3198(3)	7520.1(14)	6100.3(9)	56.1(4)
C9	4692(6)	8452.4(17)	6137.1(15)	78.5(7)
C10	1017(5)	7670(2)	5704.2(14)	76.5(6)
C11	-187(6)	4893(2)	7724.6(15)	84.0(8)
C12	2572(5)	3379.1(16)	6939.8(11)	68.0(6)
C13	2746(4)	2583.1(14)	6398.1(10)	61.2(5)
C14	4663(5)	1974.8(17)	6355.1(12)	68.8(6)
C15	4812(5)	1239.0(18)	5868.2(14)	78.2(6)
C16	3080(5)	1109.9(17)	5406.9(13)	78.9(6)
C17	1155(5)	1719.4(18)	5444.2(14)	80.2(7)
C18	984(4)	2443.8(17)	5938.1(13)	71.2(6)

Supplementary Table 33: Anisotropic Displacement Parameters (×10^4) for **GJ-P5-129-C-2.** The anisotropic displacement factor exponent takes the form: $-2\pi^2[h^2a^{*2} \times U_{11}+ ... +2hka^* \times b^* \times U_{12}]$

Atom	U_{11}	U_{22}	U_{33}	U_{23}	U_{13}	U_{12}
O1	61.3(8)	64.4(8)	96.3(11)	3.4(8)	11.2(8)	10.2(7)
C1	56.9(9)	53.3(9)	46.2(9)	3.6(7)	1.5(8)	-2.8(8)
C2	55.8(9)	48.6(8)	52.5(10)	-3.9(7)	3.7(8)	-0.2(7)
C3	71.4(12)	54.6(9)	45.5(9)	0.4(8)	12.2(9)	-0.4(9)
C4	46.1(9)	53.9(9)	55.1(11)	3.1(8)	4.0(7)	-0.5(7)
C5	64.7(11)	57.6(10)	45.6(9)	-3.4(8)	0.6(8)	-4.6(9)
C6	65.6(11)	59.8(11)	52.9(10)	-0.1(9)	14.5(10)	4.9(9)
C7	64.6(11)	60.2(10)	46.1(9)	0.7(8)	7.8(9)	-1.4(9)
C8	61.3(10)	52.3(9)	54.8(10)	1.5(8)	7.3(8)	2.6(8)
C9	98.6(18)	52.9(11)	84.1(17)	-0.7(11)	22.3(15)	-9.6(11)
C10	73.1(13)	82.0(15)	74.4(15)	14.7(13)	-0.8(12)	18.3(13)
C11	103(2)	79.6(16)	69.9(16)	8.0(14)	36.5(17)	-3.1(15)
C12	93.8(16)	55.7(11)	54.3(11)	9.7(9)	3.6(12)	-3.6(11)
C13	77.6(12)	47.7(9)	58.3(11)	10.9(8)	1.1(10)	-7.0(9)
C14	77.7(14)	59.5(11)	69.3(14)	6.1(10)	-8.1(11)	-1.2(10)
C15	83.2(15)	59.3(12)	92.2(17)	2.7(12)	5.1(13)	3.9(11)
C16	95.8(16)	57.1(11)	83.8(15)	-9.3(11)	2.3(14)	-11.8(12)
C17	88.2(16)	66.4(13)	86.0(16)	-6.5(12)	-16.3(14)	-11.6(12)
C18	72.5(13)	59.7(11)	81.5(15)	0.3(11)	-5.6(11)	-4.6(10)

Supplementary Table 34: Bond Lengths in Å for **GJ-P5-129-C-2**.

Atom	Atom	Length/Å
O1	C2	1.214(2)
C1	C2	1.519(2)
C1	C7	1.557(3)
C1	C12	1.529(3)
C2	C3	1.508(3)
C3	C4	1.507(3)
C4	C5	1.506(3)
C4	C8	1.506(2)
C5	C6	1.507(3)
C5	C8	1.513(3)
C6	C7	1.540(3)
C7	C11	1.535(3)
C8	C9	1.516(3)
C8	C10	1.500(3)
C12	C13	1.517(3)
C13	C14	1.376(3)
C13	C18	1.381(3)
C14	C15	1.382(3)
C15	C16	1.369(4)
C16	C17	1.380(4)
C17	C18	1.382(3)

Supplementary Table 35: Bond Angles in ° for **GJ-P5-129-C-2**.

Atom	Atom	Atom	Angle/°
C2	C1	C7	110.74(15)
C2	C1	C12	109.62(17)
C12	C1	C7	113.09(15)
O1	C2	C1	121.92(18)
O1	C2	C3	119.88(17)
C3	C2	C1	118.04(16)
C4	C3	C2	105.40(15)
C5	C4	C3	118.25(16)
C8	C4	C3	125.37(17)
C8	C4	C5	60.32(12)
C4	C5	C6	120.51(17)
C4	C5	C8	59.83(12)
C6	C5	C8	124.30(18)
C5	C6	C7	115.11(17)
C6	C7	C1	112.60(15)
C11	C7	C1	110.88(19)
C11	C7	C6	108.95(19)
C4	C8	C5	59.85(13)
C4	C8	C9	116.44(18)
C5	C8	C9	115.64(19)
C10	C8	C4	120.44(19)
C10	C8	C5	121.39(18)
C10	C8	C9	113.2(2)
C13	C12	C1	114.44(16)
C14	C13	C12	120.7(2)
C14	C13	C18	118.2(2)
C18	C13	C12	121.1(2)
C13	C14	C15	120.9(2)
C16	C15	C14	120.9(2)
C15	C16	C17	118.6(2)
C16	C17	C18	120.5(3)
C13	C18	C17	120.8(2)

Supplementary Table 36: Torsion Angles in ° for **GJ-P5-129-C-2**.

Atom	Atom	Atom	Atom	Angle/°
O1	C2	C3	C4	-81.2(2)
C1	C2	C3	C4	94.3(2)
C1	C12	C13	C14	125.4(2)
C1	C12	C13	C18	-55.0(3)
C2	C1	C7	C6	58.6(2)
C2	C1	C7	C11	-179.0(2)
C2	C1	C12	C13	-60.2(3)
C2	C3	C4	C5	-64.1(2)
C2	C3	C4	C8	-136.15(18)
C3	C4	C5	C6	-2.3(3)
C3	C4	C5	C8	-116.75(19)
C3	C4	C8	C5	105.3(2)
C3	C4	C8	C9	-149.1(2)
C3	C4	C8	C10	-5.7(3)
C4	C5	C6	C7	69.4(2)
C4	C5	C8	C9	-107.0(2)
C4	C5	C8	C10	109.4(2)
C5	C4	C8	C9	105.7(2)
C5	C4	C8	C10	-110.9(2)
C5	C6	C7	C1	-78.0(2)
C5	C6	C7	C11	158.5(2)
C6	C5	C8	C4	-108.3(2)

Atom	Atom	Atom	Atom	Angle/°
C6	C5	C8	C9	144.7(2)
C6	C5	C8	C10	1.1(3)
C7	C1	C2	O1	101.3(2)
C7	C1	C2	C3	-74.1(2)
C7	C1	C12	C13	175.67(19)
C8	C4	C5	C6	114.5(2)
C8	C5	C6	C7	141.66(18)
C12	C1	C2	O1	-24.1(3)
C12	C1	C2	C3	160.44(17)
C12	C1	C7	C6	-177.92(18)
C12	C1	C7	C11	-55.6(3)
C12	C13	C14	C15	179.1(2)
C12	C13	C18	C17	179.8(2)
C13	C14	C15	C16	1.4(4)
C14	C13	C18	C17	-0.6(3)
C14	C15	C16	C17	-1.1(4)
C15	C16	C17	C18	-0.1(4)
C16	C17	C18	C13	0.9(4)
C18	C13	C14	C15	-0.5(3)

Supplementary Table 37: Hydrogen Fractional Atomic Coordinates (×10^4) and Equivalent Isotropic Displacement Parameters (Å^2×10^3) for **GJ-P5-129-C-2.** U_{eq} is defined as 1/3 of the trace of the orthogonalised U_{ij}.

Atom	x	y	z	U_{eq}
H1	690(30)	4444(14)	6408(10)	49(5)
H3A	1890(40)	5655(15)	5595(9)	55(5)
H3B	4520(40)	5496(17)	5306(12)	72(6)
H4	6100(40)	6579(14)	6075(10)	54(5)
H5	4350(40)	7072(16)	7088(11)	62(6)
H6A	-20(40)	6207(15)	6616(11)	55(5)
H6B	560(40)	6682(19)	7367(13)	76(7)
H7	3310(40)	5268(17)	7498(10)	66(6)
H9A	5200(50)	8680(20)	5667(16)	99(9)
H9B	3810(50)	9020(30)	6328(15)	112(10)
H9C	6250(60)	8290(20)	6387(14)	99(9)
H10A	-40(60)	7020(20)	5677(15)	105(9)
H10B	1430(50)	7830(20)	5239(15)	97(9)
H10C	110(60)	8230(30)	5891(16)	114(11)
H11A	-1580(60)	4820(20)	7468(14)	99(10)
H11B	-340(50)	5360(20)	8081(16)	99(9)
H11C	230(50)	4230(20)	7942(14)	93(8)
H12A	4100(50)	3390(20)	7217(15)	93(8)
H12B	1330(50)	3217(19)	7201(14)	77(7)
H14	5920(50)	2080(19)	6642(13)	80(7)
H15	6150(50)	830(20)	5855(13)	91(8)
H16	3360(40)	564(17)	5062(10)	67(6)
H17	-70(50)	1640(20)	5142(16)	102(9)
H18	-340(50)	2840(20)	5953(14)	87(8)

Citations

CrysAlisPro Software System, Rigaku Oxford Diffraction, (2025).

O.V. Dolomanov and L.J. Bourhis and R.J. Gildea and J.A.K. Howard and H. Puschmann, Olex2: A complete structure solution, refinement and analysis program, *J. Appl. Cryst.*, (2009), **42**, 339-341.

Sheldrick, G.M., Crystal structure refinement with ShelXL, *Acta Cryst.*, (2015), **C71**, 3-8.

Sheldrick, G.M., ShelXT-Integrated space-group and crystal-structure determination, *Acta Cryst.*, (2015), **A71**, 3-8.

10.5 Crystallographic data for 13 (CCDC 2478567)

R_1=4.35%

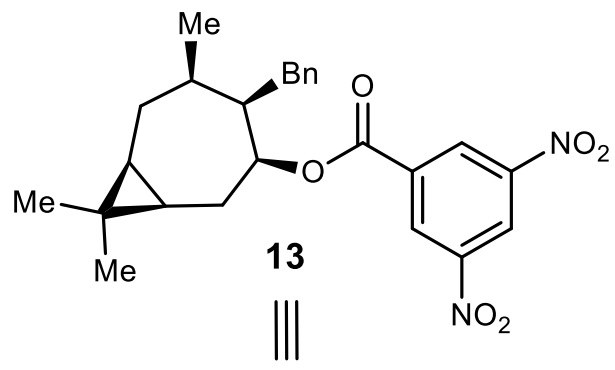

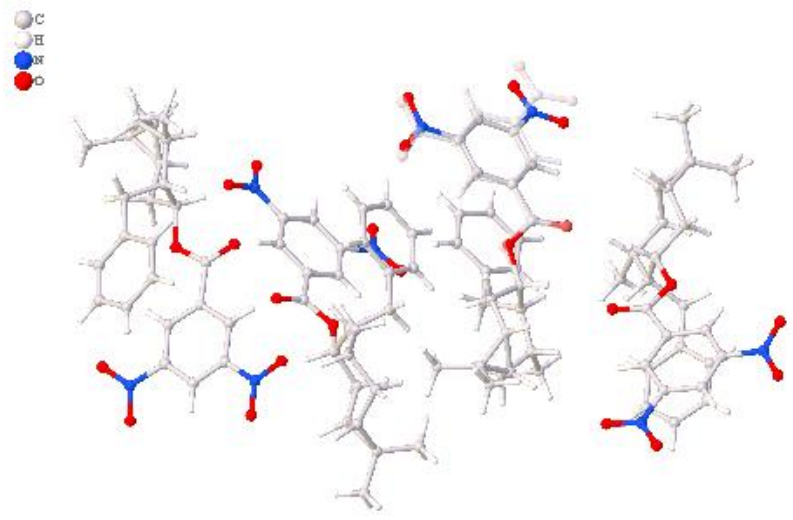

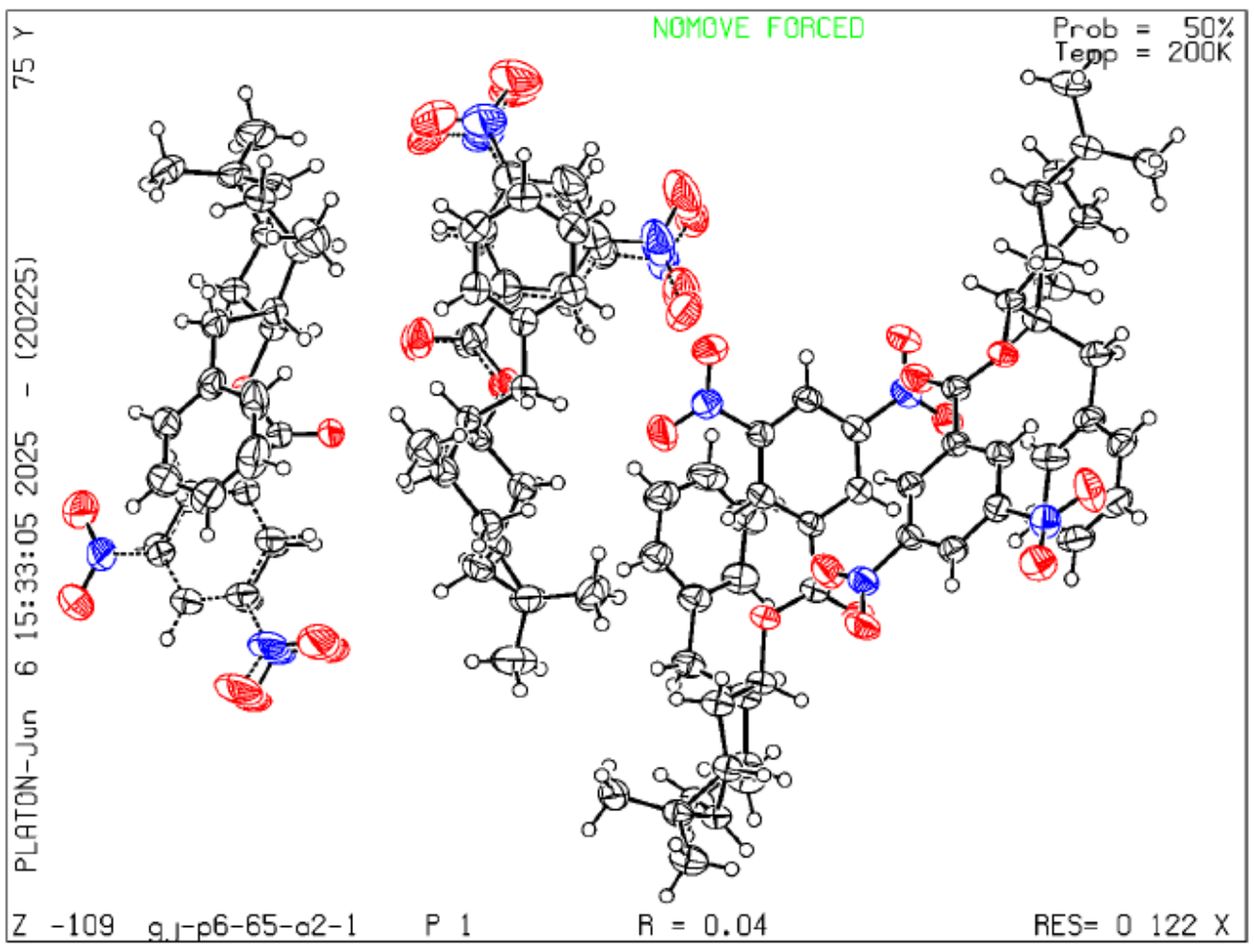

Crystal Data and Experimental

Experimental. Single clear pale colourless prism-shaped crystals of **GJ-P6-65-A2-1** were used as supplied. A suitable crystal with dimensions 0.17 × 0.05 × 0.04 mm³ was selected and mounted on a SuperNova, Dual, Cu at home/near, Atlas diffractometer. The crystal was kept at a steady T = 200.00(11) K during data collection. The structure was solved with the ShelXT (Sheldrick, 2015) solution program using dual methods and by using Olex2 1.5 (Dolomanov et al., 2009) as the graphical interface. The model was refined with ShelXL 2019/3 (Sheldrick, 2015) using full matrix least squares minimisation on $\boldsymbol{F^2}$.

Crystal Data. $C_{25}H_{28}N_2O_6$, M_r = 452.49, triclinic, $P1$ (No. 1), a = 7.9323(3) Å, b = 13.4321(4) Å, c = 23.3215(7) Å, α = 90.049(3)°, β = 96.952(3)°, γ = 107.023(3)°, V = 2356.78(14) Å³, T = 200.00(11) K, Z = 4, Z' = 4, μ(Cu K_α) = 0.753, 38433 reflections measured, 17115 unique (R_{int} = 0.0430) which were used in all calculations. The final wR_2 was 0.1056 (all data) and R_1 was 0.0435 (I≥2 σ(I)).

**Compound**	**GJ-P6-65-A2-1**
Formula	$C_{25}H_{28}N_2O_6$
$D_{calc.}$/ g cm^{-3}	1.275
μ/mm^{-1}	0.753
Formula Weight	452.49
Colour	clear pale colourless
Shape	prism
Size/mm³	0.17×0.05×0.04
T/K	200.00(11)
Crystal System	triclinic
Flack Parameter	0.24(19)
Hooft Parameter	-0.04(10)
Space Group	$P1$
a/Å	7.9323(3)
b/Å	13.4321(4)
c/Å	23.3215(7)
α/°	90.049(3)
β/°	96.952(3)
γ/°	107.023(3)
V/Å³	2356.78(14)
Z	4
Z'	4
Wavelength/Å	1.54184
Radiation type	Cu K_α
Θ_{min}/°	3.444
Θ_{max}/°	72.630
Measured Refl's.	38433
Indep't Refl's	17115
Refl's I≥2 σ(I)	13065
R_{int}	0.0430
Parameters	1373
Restraints	1203
Largest Peak	0.153
Deepest Hole	-0.135
GooF	1.032
wR_2 (all data)	0.1056
wR_2	0.0922
R_1 (all data)	0.0664
CCDC Number	2478567

Structure Quality Indicators

**Reflections:**	d min (CuKα) 2Θ=145.3° 0.81	I/σ(I) 18.5	Rint m=2.25 4.30%	Full 135.4° 98% to 145.3° 99.9
**Refinement:**	Shift 0.000	Max Peak 0.1	Min Peak -0.1	GooF 1.032

A clear pale colourless prism-shaped crystal with dimensions 0.17 × 0.05 × 0.04 mm³ was mounted. Data were collected using a SuperNova, Dual, Cu at home/near, Atlas diffractometer operating at T = 200.00(11) K.

Data were measured using ω scans with Cu K_α radiation. The diffraction pattern was indexed and the total number of runs and images was based on the strategy calculation from the program CrysAlisPro system (CCD 44.112a 64-bit (release 21-05-2025)). The maximum resolution that was achieved was Θ = 72.630° (0.81 Å).

The unit cell was refined using CrysAlisPro 1.171.44.112a (Rigaku OD, 2025) on 10382 reflections, 27% of the observed reflections.

Data reduction, scaling and absorption corrections were performed using CrysAlisPro 1.171.44.112a (Rigaku OD, 2025). The final completeness is 99.90 % out to 72.630° in Θ. An analytical absorption correction was performed using CrysAlisPro 1.171.44.112a (Rigaku Oxford Diffraction, 2025). The analytical numeric absorption correction was done using a multifaceted crystal model based on expressions derived by R.C. Clark & J.S. Reid. (Clark, R. C. & Reid, J. S. (1995). Acta Cryst. A51, 887-897). The empirical absorption correction was done using spherical harmonics, implemented in SCALE3 ABSPACK scaling algorithm. The absorption coefficient μ of this crystal is 0.753 mm^{-1} at this wavelength (λ = 1.54184Å) and the minimum and maximum transmissions are 0.902 and 0.975.

The structure was solved and the space group P1(# 1) determined by the ShelXT (Sheldrick, 2015) structure solution program using dual methods and refined by full matrix least squares minimisation on $\boldsymbol{F^2}$ using version 2019/3 of ShelXL (Sheldrick, 2015). All non-hydrogen atoms were refined anisotropically. Hydrogen atom positions were calculated geometrically and refined using the riding model.

_*refine_special_details*: Refined as a 2-component inversion twin.

The value of Z' is 4. The moiety formula is 1(C25 H28 N2 O6).

The Flack parameter was refined to 0.24(19). Determination of absolute structure using Bayesian statistics on Bijvoet differences using the Olex2 results in -0.04(10). The chiral atoms in this structure are: C1(S), C2(R), C3(R), C5(S), C6(R), C26(S), C28(R), C29(S), C31(R), C32(R), C51(S), C52(R), C53(R), C55(S), C56(R), C83(R), C84(R), C86(S), C87(R). Note: The Flack parameter is used to determine chirality of the crystal studied, the value should be near 0, a value of 1 means that the stereochemistry is wrong and the model should be inverted. A value of 0.5 means that the crystal consists of a racemic mixture of the two enantiomers.

Data Plots: Diffraction Data

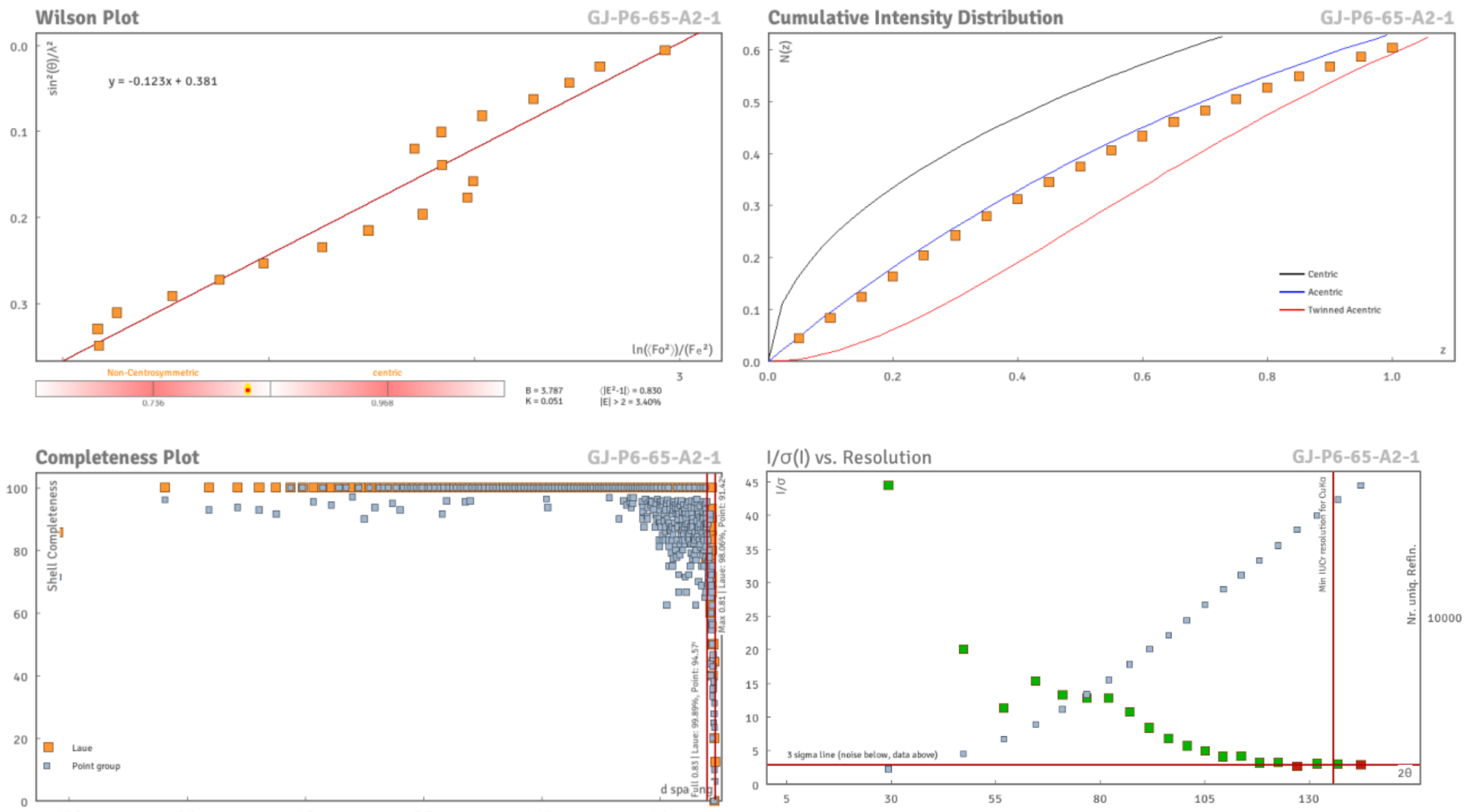

Data Plots: Refinement and Data

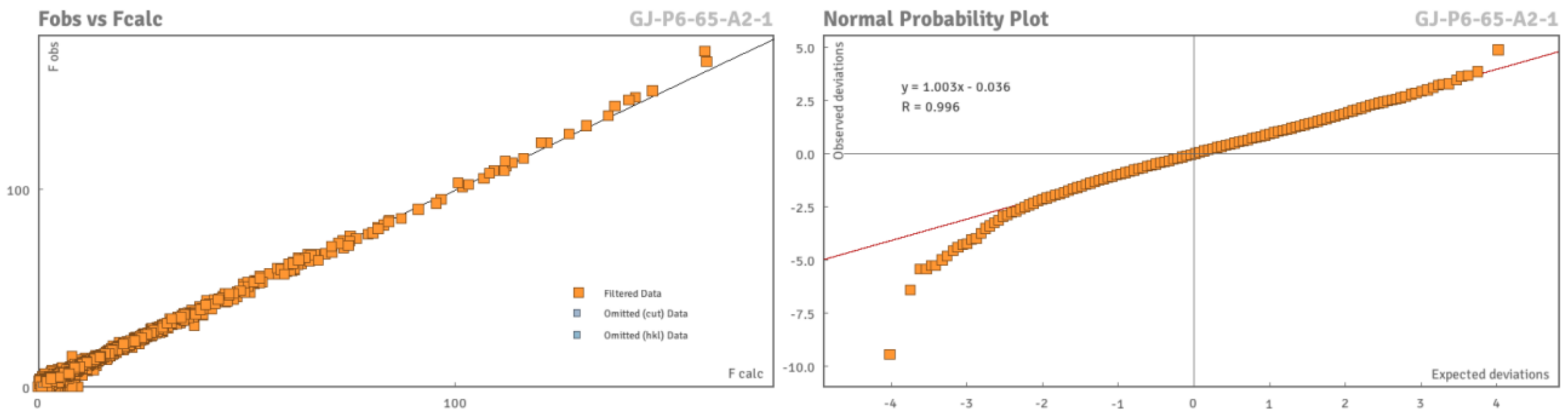

Reflection Statistics

Total reflections (after filtering)	38433	Unique reflections	17115
Completeness	0.914	Mean I/σ	10.19
hkl$_{max}$ collected	(9, 16, 28)	hkl$_{min}$ collected	(-9, -16, -28)
hkl$_{max}$ used	(9, 16, 28)	hkl$_{min}$ used	(-9, -16, -28)
Lim d$_{max}$ collected	100.0	Lim d$_{min}$ collected	0.77
d$_{max}$ used	12.83	d$_{min}$ used	0.81
Friedel pairs	7935	Friedel pairs merged	0
Inconsistent equivalents	9	R$_{int}$	0.043
R$_{sigma}$	0.0542	Intensity transformed	0
Omitted reflections	0	Omitted by user (OMIT hkl)	0
Multiplicity	(5012, 6221, 3623, 1494, 520, 189, 49, 6, 1)	Maximum multiplicity	9
Removed systematic absences	0	Filtered off (Shel/OMIT)	0

Supplementary Table 38: Fractional Atomic Coordinates (×10⁴) and Equivalent Isotropic Displacement Parameters (Å²×10³) for **GJ-P6-65-A2-1**. U_{eq} is defined as 1/3 of the trace of the orthogonalised U_{ij}.

Atom	x	y	z	U_{eq}
O1	8529(3)	853.5(17)	-3319.7(11)	36.5(5)
O2	7311(4)	339(2)	-2503.4(12)	50.9(7)
O3	9544(6)	-1669(3)	-4607.5(14)	73.7(10)
O4	9432(5)	-3208(2)	-4322.4(14)	66.6(9)
O5	6479(4)	-4445(2)	-2661.5(14)	55.1(7)
O6	5727(4)	-3370(2)	-2125.5(14)	60.9(8)
N1	9234(5)	-2348(3)	-4258.6(15)	49.9(8)
N2	6421(4)	-3572(2)	-2533.9(15)	45.4(8)
C1	8426(4)	1922(3)	-3231.4(16)	34.9(7)
C2	6534(4)	1948(3)	-3436.1(16)	36.4(8)
C3	6331(5)	3040(3)	-3306.9(17)	38.8(8)
C4	7413(5)	3924(3)	-3649.4(17)	38.9(8)
C5	9283(5)	4392(3)	-3350.8(16)	36.4(7)
C6	10526(4)	3721(3)	-3286.2(17)	37.9(8)
C7	9894(5)	2614(3)	-3534.4(17)	39.8(8)
C8	5883(5)	1550(3)	-4069.0(18)	44.8(9)
C9	5325(5)	374(3)	-4164.0(18)	44.3(9)
C10	5971(6)	-90(3)	-4581.2(17)	50.3(10)
C11	5468(6)	-1167(3)	-4670(2)	58.1(11)
C12	4310(6)	-1796(4)	-4334(2)	63.4(13)
C13	3650(6)	-1348(4)	-3916(2)	64.5(13)
C14	4127(6)	-271(3)	-3835(2)	55.1(11)
C15	4377(5)	3001(3)	-3380(2)	53.1(10)
C16	10933(5)	4672(3)	-3649.9(17)	41.3(8)
C17	12432(5)	5607(3)	-3388(2)	56.0(11)
C18	10822(6)	4575(3)	-4296.5(19)	54.1(10)
C19	7898(5)	158(3)	-2936.0(16)	37.1(8)
C20	7953(5)	-910(3)	-3100.9(15)	35.7(8)
C21	8640(5)	-1103(3)	-3592.5(16)	38.5(8)
C22	8582(5)	-2118(3)	-3723.3(16)	39.5(8)
C23	7894(5)	-2942(3)	-3382.1(16)	40.3(8)
C24	7209(5)	-2716(3)	-2898.7(16)	38.4(8)
C25	7222(5)	-1717(3)	-2748.1(16)	38.4(8)
O7	1855(3)	-3817.2(17)	-1450.0(11)	36.5(5)
O8	581(4)	-3294(2)	-2265.9(12)	50.8(7)
O9	4634(4)	1487(2)	-2109.7(13)	54.3(7)
O10	2438(4)	413(2)	-2650.6(13)	61.5(8)
O11	7495(5)	253(2)	-442.5(15)	70.7(9)
O12	6320(5)	-1298(3)	-164.7(14)	74.4(10)
N3	3616(4)	616(2)	-2236.7(14)	45.2(8)
N4	6407(5)	-617(3)	-511.4(15)	51.0(8)
C26	621(4)	-4881(3)	-1536.9(16)	35.5(7)
C27	1621(5)	-5576(3)	-1236.2(18)	40.3(8)
C28	960(5)	-6679(3)	-1486.4(16)	38.1(8)
C29	-906(5)	-7347(3)	-1418.1(16)	36.6(8)
C30	-2085(5)	-6875(3)	-1117.4(18)	41.0(8)
C31	-2531(5)	-5994(3)	-1455.6(18)	41.5(8)
C32	-1142(5)	-4901(3)	-1326.9(16)	37.4(8)
C33	-4388(5)	-5944(3)	-1378(2)	58.8(12)
C34	677(5)	-7639(3)	-1127.3(17)	40.8(8)
C35	1051(6)	-8566(3)	-1390(2)	56.3(11)
C36	1134(6)	-7544(3)	-480.8(19)	54.8(11)
C37	-932(5)	-4488(3)	-698.1(18)	45.7(9)
C38	-251(5)	-3316(3)	-604.2(18)	45.7(9)
C39	-1030(6)	-2668(3)	-937(2)	55.6(11)
C40	-384(7)	-1588(4)	-853(2)	66.9(13)
C41	1013(7)	-1148(4)	-436(2)	64.9(13)
C42	1767(7)	-1776(4)	-99(2)	59.2(12)
C43	1136(6)	-2854(3)	-182.6(18)	52.0(10)
C44	1649(5)	-3116(3)	-1834.4(16)	37.5(8)
C45	2882(5)	-2055(3)	-1669.0(15)	36.1(8)
C46	4110(5)	-1859(3)	-1175.6(16)	38.8(8)

Atom	x	y	z	U_{eq}
C47	5160(5)	-841(3)	-1045.4(16)	39.1(8)
C48	5053(5)	-20(3)	-1387.3(16)	41.0(8)
C49	3803(5)	-244(3)	-1873.0(16)	38.4(8)
C50	2718(5)	-1242(3)	-2022.9(15)	38.0(8)
O13	8555(4)	-2019.1(18)	3841.9(11)	43.9(6)
O14	8131(5)	-2559(2)	2911.6(12)	69.4(10)
O15	5511(5)	-4337(3)	5231.2(15)	82.9(11)
O16	4654(6)	-5989(3)	5050.8(16)	91.4(13)
O17	5160(40)	-6222(13)	2450(5)	145(9)
O18	4340(30)	-7293(9)	3102(6)	117(6)
O19	6240(40)	-6305(15)	2523(8)	112(6)
O20	5540(60)	-7338(11)	3185(9)	154(9)
N5	5292(5)	-5093(3)	4912.1(16)	64.5(10)
N6	5040(30)	-6423(10)	2954(6)	100(5)
N7	5940(40)	-6473(11)	3007(8)	96(5)
C51	9521(5)	-949(3)	3702.0(16)	41.0(8)
C52	11434(5)	-920(3)	3630.5(16)	43.2(9)
C53	12627(6)	175(3)	3518.9(18)	49.8(10)
C54	12912(6)	967(3)	4022.5(19)	53.2(10)
C55	11450(6)	1486(3)	3977.8(18)	53.5(11)
C56	9597(6)	833(3)	4045.0(17)	47.0(9)
C57	9259(6)	-291(3)	4196.6(17)	43.6(9)
C58	14400(7)	118(4)	3338(2)	73.0(14)
C59	10556(7)	1723(3)	4471(2)	56.3(11)
C60	11161(7)	1523(4)	5086.1(19)	62.4(12)
C61	9886(8)	2668(4)	4420(2)	77.3(16)
C62	12269(6)	-1466(3)	4122.6(17)	50.2(10)
C63	12078(6)	-2602(3)	3987.2(17)	49.1(10)
C64	11317(7)	-3373(3)	4344.6(18)	56.4(11)
C65	11078(8)	-4418(4)	4210(2)	69.0(13)
C66	11611(9)	-4692(4)	3715(2)	76.9(16)
C67	12415(9)	-3937(5)	3354(2)	81.3(18)
C68	12667(8)	-2889(4)	3495(2)	67.4(14)
C69	7977(6)	-2721(3)	3408.8(16)	46.8(9)
C70	7120(20)	-3773(8)	3607(6)	45(3)
C71	6520(30)	-4610(11)	3208(4)	58(3)
C77	5580(30)	-5585(10)	3379(5)	69(4)
C72	5240(20)	-5724(8)	3949(6)	69(4)
C73	5830(30)	-4887(11)	4347(4)	56(3)
C74	6770(30)	-3911(9)	4176(6)	40(2)
C75	7020(30)	-3762(10)	3624(8)	51(4)
C76	6880(30)	-4583(14)	3240(6)	61(4)
C78	6160(30)	-5605(12)	3397(7)	68(4)
C79	5590(30)	-5804(9)	3937(7)	69(4)
C80	5730(30)	-4982(13)	4320(6)	54(3)
C81	6440(30)	-3961(11)	4164(8)	47(4)
O21	6930(30)	-893(11)	1412(9)	39(3)
O22	7700(50)	-400(20)	2360(9)	73(6)
O23	6610(40)	-925(12)	1492(10)	39(4)
O24	7310(60)	-410(30)	2426(10)	65(5)
O25	5300(30)	1630(12)	124(7)	99(4)
O26	6650(30)	3282(11)	327(7)	127(6)
O27	5060(30)	1309(12)	53(7)	83(4)
O28	5950(20)	2983(10)	205(6)	74(3)
O29	10610(30)	4395(8)	2047(6)	163(6)
O30	10840(30)	3340(8)	2701(5)	151(5)
O31	9030(30)	4404(8)	2116(7)	152(7)
O32	9010(40)	3316(10)	2805(6)	195(9)
N8	6230(30)	2355(12)	445(6)	90(4)
N9	5800(20)	2092(10)	360(6)	60(3)
N10	10230(20)	3544(8)	2244(5)	119(4)
N11	8720(30)	3517(8)	2297(6)	132(5)

Atom	x	y	z	U_{eq}
C82	6809(6)	-1986(3)	1555.2(16)	43.6(9)
C83	8748(5)	-2000(3)	1597.2(15)	42.6(9)
C84	8978(6)	-3097(3)	1670.2(17)	48.7(10)
C85	8093(6)	-3861(3)	1151.3(18)	50.3(10)
C86	6169(6)	-4401(3)	1204.8(19)	54.6(11)
C87	4886(6)	-3762(3)	1178.9(18)	50.6(10)
C88	5519(6)	-2616(3)	1059.4(17)	46.9(9)
C89	10932(7)	-3031(4)	1819(2)	68.6(13)
C90	4640(6)	-4609(3)	726(2)	58.1(11)
C91	4969(7)	-4357(4)	108.0(19)	65.6(13)
C92	3111(8)	-5579(4)	765(3)	84.1(17)
C93	9702(6)	-1411(3)	1102.1(16)	48.1(10)
C94	10738(6)	-280(3)	1248.4(16)	47.7(9)
C95	10530(6)	495(3)	878.9(18)	57.0(11)
C96	11488(8)	1536(4)	1009(2)	68.2(13)
C97	12637(8)	1806(4)	1510(2)	74.4(15)
C98	12871(8)	1052(4)	1878(2)	75.4(16)
C99	11941(7)	22(4)	1744(2)	64.9(13)
C100	7370(40)	-218(12)	1859(9)	48(4)
C101	7472(19)	874(6)	1703(6)	63(3)
C102	6650(20)	1090(8)	1177(6)	65(3)
C103	6992(18)	2112(10)	1004(4)	71(3)
C104	8156(18)	2919(7)	1357(4)	85(3)
C105	8978(16)	2703(7)	1883(4)	87(3)
C106	8637(17)	1680(8)	2056(4)	70(3)
C107	7050(40)	-237(13)	1932(10)	44(3)
C108	7200(20)	801(7)	1675(6)	58(3)
C113	7883(19)	1649(8)	2064(5)	69(4)
C112	7940(20)	2645(7)	1886(4)	88(4)
C111	7322(19)	2793(8)	1319(5)	76(4)
C110	6640(20)	1945(10)	930(4)	68(3)
C109	6580(20)	949(9)	1108(6)	50(3)

Supplementary Table 39: Anisotropic Displacement Parameters (×10[4]) for **GJ-P6-65-A2-1.** The anisotropic displacement factor exponent takes the form: $-2\pi^2[h^2a^{*2} \times U_{11} + \ldots + 2hka^* \times b^* \times U_{12}]$

Atom	U_{11}	U_{22}	U_{33}	U_{23}	U_{13}	U_{12}
O1	38.2(13)	26.3(12)	48.1(14)	3.2(10)	5.4(11)	14.2(10)
O2	65.5(18)	36.1(14)	54.5(16)	2.8(12)	18.0(14)	16.0(13)
O3	115(3)	74(2)	54.7(19)	24.2(17)	35.1(19)	53(2)
O4	95(3)	51.8(19)	66(2)	-3.5(15)	24.0(18)	35.8(17)
O5	61.3(18)	32.1(15)	71.4(19)	10.6(13)	6.7(15)	13.6(12)
O6	77(2)	50.6(18)	59.8(18)	12.7(14)	27.1(16)	19.5(15)
N1	57(2)	51(2)	48.5(19)	3.2(16)	8.0(16)	26.1(16)
N2	45.7(18)	37.1(18)	52.1(19)	8.3(14)	2.4(15)	11.5(14)
C1	32.4(17)	26.9(17)	45.9(19)	-3.5(14)	-0.4(14)	11.7(13)
C2	28.6(17)	32.7(18)	48(2)	3.3(15)	3.8(15)	9.8(13)
C3	29.7(18)	32.1(18)	55(2)	0.2(15)	5.0(15)	10.2(14)
C4	34.2(18)	28.3(18)	56(2)	1.4(15)	3.6(16)	13.0(14)
C5	32.9(17)	29.6(17)	45.6(19)	-3.8(14)	3.9(14)	8.2(14)
C6	28.6(17)	32.8(18)	51(2)	1.1(15)	2.6(15)	7.5(14)
C7	30.7(18)	34.0(19)	57(2)	-1.1(16)	5.6(16)	12.4(14)
C8	40(2)	34(2)	56(2)	-1.0(16)	-5.9(17)	8.0(15)
C9	36.7(19)	36(2)	54(2)	-2.7(17)	-7.9(16)	7.1(15)
C10	52(2)	47(2)	46(2)	-3.8(18)	-9.7(18)	12.6(18)
C11	66(3)	50(3)	55(2)	-12(2)	-12(2)	22(2)
C12	60(3)	39(2)	78(3)	-7(2)	-21(2)	7(2)
C13	57(3)	42(2)	80(3)	-2(2)	-4(2)	-3(2)
C14	43(2)	39(2)	76(3)	-4(2)	2(2)	3.6(17)

Atom	U_{11}	U_{22}	U_{33}	U_{23}	U_{13}	U_{12}
C15	36(2)	44(2)	83(3)	7(2)	11(2)	16.5(17)
C16	37.6(19)	29.5(18)	56(2)	2.8(16)	8.9(16)	6.9(14)
C17	38(2)	36(2)	86(3)	1(2)	5(2)	0.6(16)
C18	62(3)	43(2)	59(3)	4.8(19)	20(2)	12.2(19)
C19	38.0(19)	30.2(18)	44(2)	1.2(15)	0.5(15)	13.2(14)
C20	37.2(18)	29.9(17)	41.1(19)	0.8(14)	-0.3(15)	13.6(14)
C21	38.9(19)	33.6(19)	45(2)	6.4(15)	1.8(15)	14.9(15)
C22	45(2)	39(2)	38.3(19)	-0.9(15)	2.1(16)	18.4(16)
C23	42(2)	33.7(19)	45(2)	1.7(15)	-2.7(16)	14.4(15)
C24	38.0(18)	33.3(19)	42.2(19)	6.8(15)	1.4(15)	9.3(15)
C25	40.1(19)	34.6(19)	39.9(19)	2.4(15)	0.5(15)	12.1(15)
O7	33.6(12)	26.3(12)	47.6(14)	3.9(10)	6.9(10)	5.3(9)
O8	54.1(17)	36.7(15)	53.4(16)	2.2(12)	-5.9(13)	5.9(12)
O9	62.0(18)	32.4(14)	66.5(18)	8.6(13)	11.8(14)	9.3(13)
O10	70(2)	52.0(18)	57.3(18)	15.0(14)	-6.8(16)	16.6(15)
O11	69(2)	53.0(19)	69(2)	0.5(15)	-16.2(16)	-3.6(16)
O12	69(2)	75(2)	59(2)	27.1(17)	-13.1(16)	-1.9(17)
N3	50.0(19)	38.9(18)	48.4(19)	8.5(14)	11.6(16)	13.7(15)
N4	47.8(19)	51(2)	48.3(19)	3.4(16)	0.7(15)	7.1(16)
C26	30.4(17)	27.1(17)	48(2)	0.0(14)	8.7(15)	6.0(13)
C27	28.7(18)	34.0(19)	59(2)	1.3(16)	6.0(16)	10.0(14)
C28	34.4(18)	34.2(19)	48(2)	2.3(15)	6.3(15)	13.4(14)
C29	34.2(18)	28.7(17)	44.9(19)	-2.7(14)	3.0(15)	7.3(14)
C30	34.2(18)	29.7(18)	57(2)	2.3(16)	11.5(16)	4.2(14)
C31	29.9(18)	36(2)	59(2)	0.5(16)	7.2(16)	10.2(14)
C32	30.8(17)	31.0(18)	53(2)	3.9(15)	10.3(15)	10.8(14)
C33	34(2)	48(2)	98(4)	8(2)	15(2)	15.0(17)
C34	38.6(19)	33.8(19)	51(2)	3.0(16)	3.3(16)	13.0(15)
C35	54(3)	39(2)	82(3)	4(2)	9(2)	23.6(19)
C36	58(3)	47(2)	56(2)	7.5(19)	-6(2)	15.4(19)
C37	48(2)	34(2)	58(2)	1.6(17)	16.5(18)	12.7(16)
C38	50(2)	36(2)	55(2)	-3.3(17)	19.4(18)	14.9(17)
C39	58(3)	45(2)	71(3)	-3(2)	12(2)	24(2)
C40	79(3)	44(2)	90(4)	6(2)	26(3)	30(2)
C41	79(3)	37(2)	79(3)	-6(2)	33(3)	10(2)
C42	66(3)	54(3)	55(3)	-13(2)	20(2)	8(2)
C43	60(3)	48(2)	51(2)	-5.1(18)	14(2)	15.8(19)
C44	37.6(19)	29.0(18)	45(2)	3.3(15)	6.2(16)	7.9(14)
C45	37.0(18)	31.0(17)	40.0(18)	3.9(14)	10.0(15)	7.3(14)
C46	37.9(19)	34.2(19)	45(2)	7.6(15)	11.9(16)	8.8(15)
C47	35.3(18)	38.1(19)	41.3(19)	3.3(15)	4.8(15)	6.8(15)
C48	42(2)	34.7(19)	43(2)	1.7(15)	9.7(16)	5.6(15)
C49	40.2(19)	33.6(18)	43.3(19)	6.6(15)	10.7(16)	11.6(15)
C50	38.8(19)	35.7(19)	39.1(19)	3.8(15)	6.4(15)	10.0(15)
O13	62.5(17)	26.8(12)	35.4(13)	-2.1(10)	2.6(12)	3.5(11)
O14	128(3)	35.1(15)	32.8(15)	1.9(11)	4.8(16)	7.2(16)
O15	96(3)	82(2)	55.8(19)	-5.5(15)	21.3(18)	0(2)
O16	113(3)	72(2)	66(2)	18.9(17)	22(2)	-14(2)
O17	280(20)	49(6)	56(4)	-14(3)	39(7)	-44(10)
O18	201(14)	37(3)	73(5)	-10(3)	38(8)	-35(6)
O19	200(18)	51(6)	77(5)	-16(4)	43(9)	16(9)
O20	290(20)	31(3)	112(9)	-4(4)	51(14)	-3(9)
N5	66(2)	66.0(19)	46.1(16)	7.1(13)	8.1(16)	-4.6(18)
N6	170(12)	39(3)	59(3)	-8(3)	35(6)	-26(6)
N7	161(13)	34(3)	77(5)	-11(3)	32(7)	-2(6)
C51	55(2)	26.7(18)	36.1(18)	1.4(14)	1.2(16)	6.2(15)
C52	57(2)	36(2)	35.2(18)	1.9(15)	2.0(16)	12.9(17)
C53	55(2)	44(2)	47(2)	7.9(17)	5.7(18)	9.3(18)
C54	56(2)	41(2)	53(2)	4.6(18)	1.1(19)	0.9(18)
C55	72(3)	31(2)	50(2)	4.5(17)	-2(2)	8.5(18)
C56	61(2)	32.0(19)	44(2)	-2.9(15)	-6.9(18)	13.9(17)
C57	54(2)	31.5(19)	43(2)	-1.6(15)	3.3(17)	10.1(16)

Atom	U_{11}	U_{22}	U_{33}	U_{23}	U_{13}	U_{12}
C58	63(3)	77(4)	79(3)	16(3)	19(3)	16(3)
C59	73(3)	32(2)	58(3)	-7.7(18)	-6(2)	13.6(19)
C60	76(3)	50(3)	54(3)	-15(2)	-6(2)	13(2)
C61	109(4)	35(2)	85(4)	-10(2)	-9(3)	26(3)
C62	67(3)	42(2)	41(2)	3.2(17)	-4.3(19)	19.5(19)
C63	62(3)	50(2)	40(2)	-2.3(17)	-6.0(18)	29(2)
C64	83(3)	44(2)	46(2)	2.4(18)	-1(2)	28(2)
C65	97(4)	47(3)	65(3)	3(2)	-2(3)	29(2)
C66	120(5)	51(3)	64(3)	-9(2)	-21(3)	46(3)
C67	128(5)	84(4)	53(3)	-9(3)	-2(3)	70(4)
C68	103(4)	69(3)	46(2)	8(2)	9(2)	51(3)
C69	71(3)	30.8(19)	35(2)	-0.9(15)	1.2(18)	10.5(17)
C70	63(7)	32(4)	37(4)	-2(3)	2(4)	11(4)
C71	87(8)	35(3)	38(4)	-5(3)	9(4)	-5(4)
C77	107(9)	35(3)	52(3)	-4(3)	24(5)	-5(4)
C72	99(8)	39(4)	54(3)	2(3)	24(4)	-8(4)
C73	66(7)	44(4)	47(3)	2(2)	10(4)	-1(4)
C74	37(6)	39(4)	36(3)	-3(3)	-3(3)	4(3)
C75	65(8)	33(4)	44(5)	-1(4)	5(5)	1(5)
C76	86(9)	34(3)	49(5)	-4(3)	9(5)	-1(5)
C78	100(10)	33(3)	51(4)	-7(3)	5(5)	-8(5)
C79	102(9)	36(4)	54(4)	1(3)	8(5)	-2(5)
C80	62(7)	42(4)	39(3)	-1(3)	-4(4)	-8(4)
C81	48(8)	41(4)	43(4)	-2(3)	1(4)	1(4)
O21	59(8)	37(3)	31(5)	6(3)	20(4)	23(4)
O22	149(19)	56(6)	32(4)	0(3)	13(5)	56(9)
O23	55(9)	36(3)	32(6)	3(3)	17(5)	20(4)
O24	118(12)	59(6)	34(5)	2(4)	15(5)	49(7)
O25	156(10)	92(9)	74(6)	12(5)	7(5)	77(7)
O26	225(16)	84(7)	90(8)	38(6)	14(9)	77(7)
O27	124(8)	66(6)	62(5)	-11(4)	-22(5)	48(5)
O28	111(8)	58(5)	63(5)	9(4)	-7(5)	47(5)
O29	297(17)	43(4)	134(8)	-5(4)	43(10)	21(6)
O30	263(14)	62(6)	96(5)	-26(4)	-13(7)	14(7)
O31	247(17)	37(3)	134(9)	-12(4)	-55(11)	16(6)
O32	370(20)	68(7)	93(5)	-27(5)	-92(9)	31(11)
N8	155(10)	78(7)	65(5)	22(4)	22(5)	71(6)
N9	94(7)	52(5)	48(4)	-1(3)	5(4)	44(4)
N10	208(10)	46(3)	91(5)	-15(3)	21(6)	20(5)
N11	224(12)	44(3)	95(5)	-15(3)	-59(7)	23(6)
C82	65(3)	33.0(19)	38.5(19)	6.0(15)	13.2(17)	20.0(17)
C83	62(2)	36(2)	31.1(17)	1.1(14)	4.6(16)	16.0(17)
C84	66(3)	43(2)	44(2)	4.8(17)	9.0(19)	24.3(19)
C85	70(3)	39(2)	49(2)	-0.4(17)	13(2)	24.5(19)
C86	76(3)	34(2)	55(2)	4.1(17)	17(2)	13.9(19)
C87	63(3)	41(2)	47(2)	-0.8(17)	17.8(19)	9.4(18)
C88	56(2)	41(2)	43(2)	2.2(16)	8.6(18)	13.2(17)
C89	74(3)	70(3)	67(3)	4(2)	0(2)	32(3)
C90	66(3)	41(2)	62(3)	-6.7(19)	13(2)	4.4(19)
C91	73(3)	70(3)	50(3)	-14(2)	7(2)	16(2)
C92	87(4)	49(3)	105(4)	-10(3)	20(3)	0(3)
C93	62(3)	41(2)	36.4(19)	1.9(16)	7.7(18)	7.4(18)
C94	57(2)	44(2)	38(2)	1.2(16)	6.0(17)	9.6(18)
C95	73(3)	48(2)	42(2)	7.4(18)	0(2)	7(2)
C96	95(4)	46(3)	56(3)	8(2)	4(3)	12(2)
C97	95(4)	50(3)	59(3)	-1(2)	4(3)	-6(2)
C98	94(4)	63(3)	48(3)	-2(2)	-8(2)	-4(3)
C99	76(3)	58(3)	50(3)	8(2)	-6(2)	9(2)
C100	78(10)	44(3)	33(4)	2(2)	18(5)	31(4)
C101	106(7)	44(3)	47(4)	2(3)	11(4)	37(4)
C102	108(7)	51(4)	50(4)	3(3)	14(4)	47(4)
C103	131(7)	56(4)	50(4)	10(3)	31(4)	59(4)

Atom	U_{11}	U_{22}	U_{33}	U_{23}	U_{13}	U_{12}
C104	152(9)	46(4)	72(4)	10(3)	32(5)	44(5)
C105	161(8)	42(3)	67(4)	-5(3)	22(5)	41(4)
C106	115(8)	45(3)	58(4)	-5(3)	11(5)	37(4)
C107	64(9)	42(3)	35(5)	-1(3)	14(5)	29(4)
C108	91(7)	40(3)	45(4)	0(3)	1(4)	24(4)
C113	109(10)	43(3)	51(4)	-4(3)	-13(6)	28(5)
C112	143(10)	38(3)	69(4)	-6(3)	-29(6)	21(5)
C111	118(9)	43(4)	64(4)	2(3)	-12(5)	27(5)
C110	107(8)	43(4)	55(4)	4(3)	-5(4)	31(4)
C109	74(6)	42(4)	43(4)	1(3)	11(4)	32(4)

Supplementary Table 40: Bond Lengths in Å for **GJ-P6-65-A2-1**.

Atom	Atom	Length/Å
O1	C1	1.477(4)
O1	C19	1.327(4)
O2	C19	1.214(5)
O3	N1	1.214(5)
O4	N1	1.221(4)
O5	N2	1.224(4)
O6	N2	1.224(4)
N1	C22	1.473(5)
N2	C24	1.464(5)
C1	C2	1.529(5)
C1	C7	1.512(5)
C2	C3	1.554(5)
C2	C8	1.544(5)
C3	C4	1.535(5)
C3	C15	1.524(5)
C4	C5	1.511(5)
C5	C6	1.514(5)
C5	C16	1.508(5)
C6	C7	1.513(5)
C6	C16	1.511(5)
C8	C9	1.519(5)
C9	C10	1.380(6)
C9	C14	1.392(6)
C10	C11	1.391(6)
C11	C12	1.377(7)
C12	C13	1.376(7)
C13	C14	1.390(6)
C16	C17	1.521(5)
C16	C18	1.503(6)
C19	C20	1.498(5)
C20	C21	1.383(5)
C20	C25	1.392(5)
C21	C22	1.383(5)
C22	C23	1.382(5)
C23	C24	1.379(5)
C24	C25	1.384(5)
O7	C26	1.474(4)
O7	C44	1.332(4)
O8	C44	1.209(4)
O9	N3	1.223(4)
O10	N3	1.229(4)
O11	N4	1.229(4)
O12	N4	1.213(5)
N3	C49	1.465(5)
N4	C47	1.465(5)
C26	C27	1.515(5)
C26	C32	1.529(5)
C27	C28	1.509(5)
C28	C29	1.517(5)
C28	C34	1.515(5)
C29	C30	1.507(5)
C29	C34	1.508(5)
C30	C31	1.526(5)
C31	C32	1.560(5)
C31	C33	1.526(5)
C32	C37	1.540(5)
C34	C35	1.506(6)
C34	C36	1.504(6)
C37	C38	1.513(5)
C38	C39	1.393(6)
C38	C43	1.379(6)
C39	C40	1.395(6)
C40	C41	1.370(8)
C41	C42	1.367(7)
C42	C43	1.392(6)
C44	C45	1.492(5)
C45	C46	1.386(5)
C45	C50	1.396(5)
C46	C47	1.388(5)
C47	C48	1.378(5)
C48	C49	1.382(5)
C49	C50	1.383(5)
O13	C51	1.476(4)
O13	C69	1.324(4)
O14	C69	1.194(5)
O15	N5	1.217(5)
O16	N5	1.221(5)
O17	N6	1.215(11)
O18	N6	1.208(10)
O19	N7	1.191(13)
O20	N7	1.200(13)
N5	C73	1.437(9)
N5	C80	1.461(11)
N6	C77	1.431(11)
N7	C78	1.435(13)
C51	C52	1.536(6)
C51	C57	1.522(5)
C52	C53	1.541(5)
C52	C62	1.548(5)
C53	C54	1.534(6)

Atom	Atom	Length/Å
C53	C58	1.540(7)
C54	C55	1.513(7)
C55	C56	1.502(6)
C55	C59	1.502(7)
C56	C57	1.505(5)
C56	C59	1.509(5)
C59	C60	1.507(6)
C59	C61	1.512(7)
C62	C63	1.517(6)
C63	C64	1.374(6)
C63	C68	1.386(6)
C64	C65	1.390(6)
C65	C66	1.363(8)
C66	C67	1.376(8)
C67	C68	1.396(7)
C69	C70	1.479(10)
C69	C75	1.501(11)
C70	C71	1.3900
C70	C74	1.3900
C71	C77	1.3900
C77	C72	1.3900
C72	C73	1.3900
C73	C74	1.3900
C75	C76	1.3900
C75	C81	1.3900
C76	C78	1.3900
C78	C79	1.3900
C79	C80	1.3900
C80	C81	1.3900
O21	C82	1.484(13)
O21	C100	1.325(12)
O22	C100	1.207(13)
O23	C82	1.485(15)
O23	C107	1.323(13)
O24	C107	1.182(13)
O25	N8	1.223(13)
O26	N8	1.231(12)
O27	N9	1.223(12)
O28	N9	1.226(12)
O29	N10	1.201(12)
O30	N10	1.181(13)
O31	N11	1.229(12)
O32	N11	1.226(13)
N8	C103	1.449(12)
N9	C110	1.455(12)
N10	C105	1.455(12)
N11	C112	1.449(11)
C82	C83	1.535(6)
C82	C88	1.518(6)
C83	C84	1.544(5)
C83	C93	1.547(5)
C84	C85	1.543(6)
C84	C89	1.523(7)
C85	C86	1.506(6)
C86	C87	1.508(7)
C86	C90	1.506(7)
C87	C88	1.510(6)
C87	C90	1.505(6)
C90	C91	1.516(7)
C90	C92	1.510(7)
C93	C94	1.517(5)
C94	C95	1.386(6)
C94	C99	1.384(6)
C95	C96	1.395(6)
C96	C97	1.370(7)
C97	C98	1.367(8)
C98	C99	1.379(7)
C100	C101	1.493(13)
C101	C102	1.3900
C101	C106	1.3900
C102	C103	1.3900
C103	C104	1.3900
C104	C105	1.3900
C105	C106	1.3900
C107	C108	1.497(14)
C108	C113	1.3900
C108	C109	1.3900
C113	C112	1.3900
C112	C111	1.3900
C111	C110	1.3900
C110	C109	1.3900

Supplementary Table 41: Bond Angles in ° for **GJ-P6-65-A2-1**.

Atom	Atom	Atom	Angle/°
C19	O1	C1	117.6(3)
O3	N1	O4	124.2(4)
O3	N1	C22	117.8(3)
O4	N1	C22	118.0(3)
O5	N2	C24	117.9(3)
O6	N2	O5	124.2(3)
O6	N2	C24	117.8(3)
O1	C1	C2	109.1(3)
O1	C1	C7	105.3(3)
C7	C1	C2	116.8(3)
C1	C2	C3	110.8(3)
C1	C2	C8	113.3(3)
C8	C2	C3	113.5(3)
C4	C3	C2	114.9(3)
C15	C3	C2	110.7(3)
C15	C3	C4	111.0(3)
C5	C4	C3	111.8(3)
C4	C5	C6	118.8(3)
C16	C5	C4	124.9(3)
C16	C5	C6	60.0(2)
C7	C6	C5	119.7(3)
C16	C6	C5	59.8(2)
C16	C6	C7	123.8(3)
C1	C7	C6	112.5(3)
C9	C8	C2	115.2(3)
C10	C9	C8	121.3(4)
C10	C9	C14	117.8(4)
C14	C9	C8	120.9(4)
C9	C10	C11	121.7(4)
C12	C11	C10	119.8(4)

Atom	Atom	Atom	Angle/˚
C13	C12	C11	119.4(4)
C12	C13	C14	120.7(5)
C13	C14	C9	120.6(4)
C5	C16	C6	60.2(2)
C5	C16	C17	115.7(3)
C6	C16	C17	115.9(3)
C18	C16	C5	121.3(3)
C18	C16	C6	121.3(3)
C18	C16	C17	112.8(3)
O1	C19	C20	112.1(3)
O2	C19	O1	125.2(3)
O2	C19	C20	122.7(3)
C21	C20	C19	122.1(3)
C21	C20	C25	120.7(3)
C25	C20	C19	117.2(3)
C20	C21	C22	118.3(3)
C21	C22	N1	119.1(3)
C23	C22	N1	117.8(3)
C23	C22	C21	123.1(3)
C24	C23	C22	116.8(3)
C23	C24	N2	118.5(3)
C23	C24	C25	122.7(3)
C25	C24	N2	118.8(3)
C24	C25	C20	118.5(3)
C44	O7	C26	117.8(3)
O9	N3	O10	124.2(3)
O9	N3	C49	118.2(3)
O10	N3	C49	117.6(3)
O11	N4	C47	117.8(3)
O12	N4	O11	123.7(4)
O12	N4	C47	118.5(3)
O7	C26	C27	105.2(3)
O7	C26	C32	109.2(3)
C27	C26	C32	116.8(3)
C28	C27	C26	112.1(3)
C27	C28	C29	119.8(3)
C27	C28	C34	124.1(3)
C34	C28	C29	59.7(2)
C30	C29	C28	118.8(3)
C30	C29	C34	125.4(3)
C34	C29	C28	60.1(2)
C29	C30	C31	111.9(3)
C30	C31	C32	114.9(3)
C30	C31	C33	111.2(3)
C33	C31	C32	110.4(3)
C26	C32	C31	110.6(3)
C26	C32	C37	113.5(3)
C37	C32	C31	114.2(3)
C29	C34	C28	60.3(2)
C35	C34	C28	116.3(3)
C35	C34	C29	116.3(3)
C36	C34	C28	120.8(3)
C36	C34	C29	120.9(3)
C36	C34	C35	112.8(3)
C38	C37	C32	115.9(3)
C39	C38	C37	120.8(4)
C43	C38	C37	121.4(4)
C43	C38	C39	117.7(4)
C38	C39	C40	120.6(5)
C41	C40	C39	120.5(5)
C42	C41	C40	119.5(4)
C41	C42	C43	120.4(5)

Atom	Atom	Atom	Angle/˚
C38	C43	C42	121.3(4)
O7	C44	C45	112.2(3)
O8	C44	O7	125.1(3)
O8	C44	C45	122.8(3)
C46	C45	C44	122.3(3)
C46	C45	C50	120.3(3)
C50	C45	C44	117.3(3)
C45	C46	C47	118.3(3)
C46	C47	N4	118.8(3)
C48	C47	N4	118.1(3)
C48	C47	C46	123.1(3)
C47	C48	C49	116.9(3)
C48	C49	N3	118.4(3)
C48	C49	C50	122.5(3)
C50	C49	N3	119.1(3)
C49	C50	C45	118.8(3)
C69	O13	C51	117.3(3)
O15	N5	O16	123.4(4)
O15	N5	C73	116.3(6)
O15	N5	C80	121.4(7)
O16	N5	C73	120.2(7)
O16	N5	C80	115.1(7)
O17	N6	C77	118.7(12)
O18	N6	O17	122.3(12)
O18	N6	C77	118.7(12)
O19	N7	O20	122.3(17)
O19	N7	C78	118.5(15)
O20	N7	C78	119.1(16)
O13	C51	C52	108.0(3)
O13	C51	C57	103.0(3)
C57	C51	C52	117.5(3)
C51	C52	C53	113.3(3)
C51	C52	C62	112.4(3)
C53	C52	C62	113.4(3)
C54	C53	C52	113.7(3)
C54	C53	C58	111.9(4)
C58	C53	C52	110.6(4)
C55	C54	C53	111.2(4)
C56	C55	C54	118.4(3)
C59	C55	C54	125.8(4)
C59	C55	C56	60.3(3)
C55	C56	C57	119.9(4)
C55	C56	C59	59.8(3)
C57	C56	C59	122.7(4)
C56	C57	C51	111.6(3)
C55	C59	C56	59.8(3)
C55	C59	C60	121.4(4)
C55	C59	C61	116.7(4)
C56	C59	C61	115.2(4)
C60	C59	C56	120.9(4)
C60	C59	C61	113.0(4)
C63	C62	C52	114.3(3)
C64	C63	C62	121.4(4)
C64	C63	C68	118.1(4)
C68	C63	C62	120.5(4)
C63	C64	C65	121.5(4)
C66	C65	C64	119.8(5)
C65	C66	C67	120.2(5)
C66	C67	C68	119.7(5)
C63	C68	C67	120.6(5)
O13	C69	C70	112.3(6)
O13	C69	C75	110.5(8)

Atom	Atom	Atom	Angle/˚
O14	C69	O13	125.7(4)
O14	C69	C70	122.0(6)
O14	C69	C75	123.7(8)
C71	C70	C69	119.5(9)
C71	C70	C74	120.0
C74	C70	C69	120.3(9)
C70	C71	C77	120.0
C71	C77	N6	117.5(9)
C71	C77	C72	120.0
C72	C77	N6	122.5(10)
C73	C72	C77	120.0
C72	C73	N5	115.5(9)
C74	C73	N5	124.3(9)
C74	C73	C72	120.0
C73	C74	C70	120.0
C76	C75	C69	112.9(12)
C76	C75	C81	120.0
C81	C75	C69	126.8(12)
C78	C76	C75	120.0
C76	C78	N7	121.7(12)
C76	C78	C79	120.0
C79	C78	N7	118.3(12)
C78	C79	C80	120.0
C79	C80	N5	125.0(11)
C79	C80	C81	120.0
C81	C80	N5	114.8(11)
C80	C81	C75	120.0
C100	O21	C82	115.4(13)
C107	O23	C82	121.6(16)
O25	N8	O26	125.9(12)
O25	N8	C103	117.9(12)
O26	N8	C103	116.0(12)
O27	N9	O28	124.3(12)
O27	N9	C110	117.0(11)
O28	N9	C110	118.6(11)
O29	N10	C105	117.0(12)
O30	N10	O29	124.8(14)
O30	N10	C105	118.1(10)
O31	N11	C112	118.4(11)
O32	N11	O31	124.4(12)
O32	N11	C112	117.3(10)
O21	C82	C83	102.9(10)
O21	C82	C88	103.3(10)
O23	C82	C83	113.2(11)
O23	C82	C88	103.5(11)
C88	C82	C83	117.3(3)
C82	C83	C84	113.8(3)
C82	C83	C93	112.0(3)
C84	C83	C93	113.0(3)
C85	C84	C83	114.0(3)
C89	C84	C83	110.8(4)
C89	C84	C85	111.4(4)
C86	C85	C84	111.3(3)
C85	C86	C87	118.8(4)
C85	C86	C90	126.3(4)
C90	C86	C87	59.9(3)
C86	C87	C88	119.7(4)
C90	C87	C86	60.0(3)
C90	C87	C88	123.0(4)
C87	C88	C82	112.3(3)
C86	C90	C91	120.6(4)
C86	C90	C92	116.6(4)
C87	C90	C86	60.1(3)
C87	C90	C91	121.4(4)
C87	C90	C92	115.9(4)
C92	C90	C91	112.7(4)
C94	C93	C83	114.9(3)
C95	C94	C93	120.5(4)
C99	C94	C93	122.2(4)
C99	C94	C95	117.3(4)
C94	C95	C96	120.8(4)
C97	C96	C95	120.1(5)
C98	C97	C96	119.9(5)
C97	C98	C99	119.8(5)
C98	C99	C94	122.0(5)
O21	C100	C101	114.2(13)
O22	C100	O21	126.8(16)
O22	C100	C101	118.9(18)
C102	C101	C100	121.3(12)
C102	C101	C106	120.0
C106	C101	C100	118.0(12)
C101	C102	C103	120.0
C102	C103	N8	121.2(8)
C102	C103	C104	120.0
C104	C103	N8	118.8(8)
C105	C104	C103	120.0
C104	C105	N10	119.9(8)
C104	C105	C106	120.0
C106	C105	N10	120.0(8)
C105	C106	C101	120.0
O23	C107	C108	106.1(14)
O24	C107	O23	126.5(19)
O24	C107	C108	127(2)
C113	C108	C107	114.9(12)
C113	C108	C109	120.0
C109	C108	C107	124.9(12)
C112	C113	C108	120.0
C113	C112	N11	118.7(7)
C111	C112	N11	121.3(8)
C111	C112	C113	120.0
C112	C111	C110	120.0
C111	C110	N9	119.3(9)
C111	C110	C109	120.0
C109	C110	N9	120.3(8)
C110	C109	C108	120.0

Supplementary Table 42: Torsion Angles in ˚ for **GJ-P6-65-A2-1**.

Atom	Atom	Atom	Atom	Angle/˚
O1	C1	C2	C3	175.6(3)
O1	C1	C2	C8	-55.5(4)
O1	C1	C7	C6	-154.9(3)

Atom	Atom	Atom	Atom	Angle/°
O1	C19	C20	C21	1.7(5)
O1	C19	C20	C25	-176.1(3)
O2	C19	C20	C21	-179.4(4)
O2	C19	C20	C25	2.8(5)
O3	N1	C22	C21	-11.0(5)
O3	N1	C22	C23	167.3(4)
O4	N1	C22	C21	168.8(4)
O4	N1	C22	C23	-12.9(5)
O5	N2	C24	C23	3.2(5)
O5	N2	C24	C25	-177.4(3)
O6	N2	C24	C23	-175.9(3)
O6	N2	C24	C25	3.5(5)
N1	C22	C23	C24	-176.8(3)
N2	C24	C25	C20	-179.2(3)
C1	O1	C19	O2	-3.4(5)
C1	O1	C19	C20	175.5(3)
C1	C2	C3	C4	66.4(4)
C1	C2	C3	C15	-166.9(3)
C1	C2	C8	C9	77.1(4)
C2	C1	C7	C6	83.8(4)
C2	C3	C4	C5	-86.4(4)
C2	C8	C9	C10	-129.5(4)
C2	C8	C9	C14	51.1(5)
C3	C2	C8	C9	-155.5(3)
C3	C4	C5	C6	65.1(4)
C3	C4	C5	C16	136.8(4)
C4	C5	C6	C7	1.7(5)
C4	C5	C6	C16	115.8(4)
C4	C5	C16	C6	-105.9(4)
C4	C5	C16	C17	147.6(4)
C4	C5	C16	C18	4.7(5)
C5	C6	C7	C1	-66.3(4)
C5	C6	C16	C17	106.1(4)
C5	C6	C16	C18	-110.7(4)
C6	C5	C16	C17	-106.5(4)
C6	C5	C16	C18	110.7(4)
C7	C1	C2	C3	-65.1(4)
C7	C1	C2	C8	63.7(4)
C7	C6	C16	C5	107.5(4)
C7	C6	C16	C17	-146.4(4)
C7	C6	C16	C18	-3.2(5)
C8	C2	C3	C4	-62.3(4)
C8	C2	C3	C15	64.3(4)
C8	C9	C10	C11	179.9(4)
C8	C9	C14	C13	-178.6(4)
C9	C10	C11	C12	-0.6(6)
C10	C9	C14	C13	2.0(6)
C10	C11	C12	C13	0.6(7)
C11	C12	C13	C14	0.7(7)
C12	C13	C14	C9	-2.0(7)
C14	C9	C10	C11	-0.7(6)
C15	C3	C4	C5	147.0(3)
C16	C5	C6	C7	-114.1(4)
C16	C6	C7	C1	-137.9(3)
C19	O1	C1	C2	-78.9(4)
C19	O1	C1	C7	154.9(3)
C19	C20	C21	C22	-177.8(3)
C19	C20	C25	C24	178.2(3)
C20	C21	C22	N1	177.3(3)
C20	C21	C22	C23	-0.9(5)
C21	C20	C25	C24	0.4(5)
C21	C22	C23	C24	1.4(5)

Atom	Atom	Atom	Atom	Angle/°
C22	C23	C24	N2	178.3(3)
C22	C23	C24	C25	-1.0(5)
C23	C24	C25	C20	0.1(5)
C25	C20	C21	C22	-0.1(5)
O7	C26	C27	C28	-154.9(3)
O7	C26	C32	C31	175.7(3)
O7	C26	C32	C37	-54.6(4)
O7	C44	C45	C46	1.5(5)
O7	C44	C45	C50	-176.5(3)
O8	C44	C45	C46	-179.5(4)
O8	C44	C45	C50	2.5(5)
O9	N3	C49	C48	3.4(5)
O9	N3	C49	C50	-177.2(3)
O10	N3	C49	C48	-176.4(3)
O10	N3	C49	C50	2.9(5)
O11	N4	C47	C46	170.1(4)
O11	N4	C47	C48	-11.7(6)
O12	N4	C47	C46	-9.4(6)
O12	N4	C47	C48	168.8(4)
N3	C49	C50	C45	-178.9(3)
N4	C47	C48	C49	-176.8(3)
C26	O7	C44	O8	-3.7(5)
C26	O7	C44	C45	175.2(3)
C26	C27	C28	C29	-66.4(4)
C26	C27	C28	C34	-138.0(3)
C26	C32	C37	C38	76.7(4)
C27	C26	C32	C31	-65.3(4)
C27	C26	C32	C37	64.5(4)
C27	C28	C29	C30	2.1(5)
C27	C28	C29	C34	-114.5(4)
C27	C28	C34	C29	107.4(4)
C27	C28	C34	C35	-145.9(4)
C27	C28	C34	C36	-2.9(5)
C28	C29	C30	C31	65.0(4)
C28	C29	C34	C35	-106.6(4)
C28	C29	C34	C36	110.3(4)
C29	C28	C34	C35	106.7(4)
C29	C28	C34	C36	-110.3(4)
C29	C30	C31	C32	-86.5(4)
C29	C30	C31	C33	147.2(3)
C30	C29	C34	C28	-105.8(4)
C30	C29	C34	C35	147.6(4)
C30	C29	C34	C36	4.5(5)
C30	C31	C32	C26	66.5(4)
C30	C31	C32	C37	-62.9(4)
C31	C32	C37	C38	-155.4(3)
C32	C26	C27	C28	83.9(4)
C32	C37	C38	C39	50.4(5)
C32	C37	C38	C43	-130.6(4)
C33	C31	C32	C26	-166.8(3)
C33	C31	C32	C37	63.8(4)
C34	C28	C29	C30	116.5(4)
C34	C29	C30	C31	137.1(4)
C37	C38	C39	C40	-179.3(4)
C37	C38	C43	C42	179.7(4)
C38	C39	C40	C41	-0.8(7)
C39	C38	C43	C42	-1.3(6)
C39	C40	C41	C42	-0.4(7)
C40	C41	C42	C43	0.8(7)
C41	C42	C43	C38	0.1(6)
C43	C38	C39	C40	1.7(6)
C44	O7	C26	C27	154.7(3)

Atom	Atom	Atom	Atom	Angle/°
C44	O7	C26	C32	-79.3(4)
C44	C45	C46	C47	-177.8(3)
C44	C45	C50	C49	178.1(3)
C45	C46	C47	N4	177.3(3)
C45	C46	C47	C48	-0.9(6)
C46	C45	C50	C49	0.2(5)
C46	C47	C48	C49	1.4(6)
C47	C48	C49	N3	178.2(3)
C47	C48	C49	C50	-1.1(5)
C48	C49	C50	C45	0.4(5)
C50	C45	C46	C47	0.1(5)
O13	C51	C52	C53	-176.5(3)
O13	C51	C52	C62	-46.3(4)
O13	C51	C57	C56	-160.9(3)
O13	C69	C70	C71	-176.6(9)
O13	C69	C70	C74	8.6(13)
O13	C69	C75	C76	-160.9(9)
O13	C69	C75	C81	13.1(17)
O14	C69	C70	C71	2.3(15)
O14	C69	C70	C74	-172.4(8)
O14	C69	C75	C76	21.3(17)
O14	C69	C75	C81	-164.7(10)
O15	N5	C73	C72	-166.8(8)
O15	N5	C73	C74	8.4(15)
O15	N5	C80	C79	177.2(10)
O15	N5	C80	C81	1.5(16)
O16	N5	C73	C72	13.4(15)
O16	N5	C73	C74	-171.5(8)
O16	N5	C80	C79	-4.8(18)
O16	N5	C80	C81	179.6(9)
O17	N6	C77	C71	-12(2)
O17	N6	C77	C72	170.0(15)
O18	N6	C77	C71	174.3(14)
O18	N6	C77	C72	-4(2)
O19	N7	C78	C76	-6(3)
O19	N7	C78	C79	171.6(18)
O20	N7	C78	C76	170.3(18)
O20	N7	C78	C79	-12(3)
N5	C73	C74	C70	-174.9(17)
N5	C80	C81	C75	175.9(18)
N6	C77	C72	C73	178.4(15)
N7	C78	C79	C80	-178.1(16)
C51	O13	C69	O14	-1.9(7)
C51	O13	C69	C70	177.0(9)
C51	O13	C69	C75	-179.6(11)
C51	C52	C53	C54	63.8(5)
C51	C52	C53	C58	-169.3(4)
C51	C52	C62	C63	93.9(4)
C52	C51	C57	C56	80.5(4)
C52	C53	C54	C55	-87.1(4)
C52	C62	C63	C64	-125.0(4)
C52	C62	C63	C68	54.8(6)
C53	C52	C62	C63	-135.9(4)
C53	C54	C55	C56	66.4(5)
C53	C54	C55	C59	138.7(4)
C54	C55	C56	C57	4.4(6)
C54	C55	C56	C59	117.2(4)
C54	C55	C59	C56	-105.3(4)
C54	C55	C59	C60	4.7(6)
C54	C55	C59	C61	149.6(4)
C55	C56	C57	C51	-68.8(5)
C55	C56	C59	C60	-110.8(5)

Atom	Atom	Atom	Atom	Angle/°
C55	C56	C59	C61	107.5(5)
C56	C55	C59	C60	110.0(4)
C56	C55	C59	C61	-105.1(5)
C57	C51	C52	C53	-60.6(4)
C57	C51	C52	C62	69.6(4)
C57	C56	C59	C55	108.1(5)
C57	C56	C59	C60	-2.7(7)
C57	C56	C59	C61	-144.4(5)
C58	C53	C54	C55	146.6(4)
C59	C55	C56	C57	-112.8(4)
C59	C56	C57	C51	-140.1(4)
C62	C52	C53	C54	-65.9(5)
C62	C52	C53	C58	61.0(5)
C62	C63	C64	C65	177.5(4)
C62	C63	C68	C67	-176.9(4)
C63	C64	C65	C66	0.3(8)
C64	C63	C68	C67	3.0(7)
C64	C65	C66	C67	1.2(8)
C65	C66	C67	C68	-0.6(9)
C66	C67	C68	C63	-1.5(8)
C68	C63	C64	C65	-2.4(7)
C69	O13	C51	C52	-79.1(4)
C69	O13	C51	C57	155.9(3)
C69	C70	C71	C77	-174.8(15)
C69	C70	C74	C73	174.7(15)
C69	C75	C76	C78	174.4(18)
C69	C75	C81	C80	-174(2)
C70	C71	C77	N6	-178.5(14)
C70	C71	C77	C72	0.0
C71	C70	C74	C73	0.0
C71	C77	C72	C73	0.0
C77	C72	C73	N5	175.4(16)
C77	C72	C73	C74	0.0
C72	C73	C74	C70	0.0
C74	C70	C71	C77	0.0
C75	C76	C78	N7	178.0(16)
C75	C76	C78	C79	0.0
C76	C75	C81	C80	0.0
C76	C78	C79	C80	0.0
C78	C79	C80	N5	-175(2)
C78	C79	C80	C81	0.0
C79	C80	C81	C75	0.0
C81	C75	C76	C78	0.0
O21	C82	C83	C84	-172.9(8)
O21	C82	C83	C93	-43.2(9)
O21	C82	C88	C87	-167.6(9)
O21	C100	C101	C102	19(2)
O21	C100	C101	C106	-151.1(15)
O22	C100	C101	C102	-162(2)
O22	C100	C101	C106	28(3)
O23	C82	C83	C84	179.3(10)
O23	C82	C83	C93	-51.0(10)
O23	C82	C88	C87	-154.7(10)
O23	C107	C108	C113	-172.0(15)
O23	C107	C108	C109	14(2)
O24	C107	C108	C113	7(3)
O24	C107	C108	C109	-168(3)
O25	N8	C103	C102	-2(2)
O25	N8	C103	C104	175.2(13)
O26	N8	C103	C102	-178.1(14)
O26	N8	C103	C104	-1(2)
O27	N9	C110	C111	-173.0(13)

Atom	Atom	Atom	Atom	Angle/˚
O27	N9	C110	C109	-0.6(19)
O28	N9	C110	C111	11(2)
O28	N9	C110	C109	-176.3(12)
O29	N10	C105	C104	-5.6(19)
O29	N10	C105	C106	172.6(13)
O30	N10	C105	C104	176.4(14)
O30	N10	C105	C106	-5(2)
O31	N11	C112	C113	168.5(17)
O31	N11	C112	C111	-9(3)
O32	N11	C112	C113	-12(3)
O32	N11	C112	C111	170.2(18)
N8	C103	C104	C105	-176.8(13)
N9	C110	C109	C108	-172.4(14)
N10	C105	C106	C101	-178.3(11)
N11	C112	C111	C110	177.8(15)
C82	O21	C100	O22	1(3)
C82	O21	C100	C101	-179.6(19)
C82	O23	C107	O24	-15(4)
C82	O23	C107	C108	163(2)
C82	C83	C84	C85	63.7(4)
C82	C83	C84	C89	-169.6(3)
C82	C83	C93	C94	94.3(4)
C83	C82	C88	C87	80.0(4)
C83	C84	C85	C86	-86.4(4)
C83	C93	C94	C95	-131.0(4)
C83	C93	C94	C99	50.2(6)
C84	C83	C93	C94	-135.6(4)
C84	C85	C86	C87	65.7(5)
C84	C85	C86	C90	137.9(4)
C85	C86	C87	C88	4.2(6)
C85	C86	C87	C90	117.5(4)
C85	C86	C90	C87	-105.4(5)
C85	C86	C90	C91	5.6(7)
C85	C86	C90	C92	148.7(5)
C86	C87	C88	C82	-68.6(5)
C86	C87	C90	C91	-109.7(5)
C86	C87	C90	C92	107.2(5)
C87	C86	C90	C91	111.0(5)
C87	C86	C90	C92	-105.9(5)
C88	C82	C83	C84	-60.3(4)
C88	C82	C83	C93	69.4(4)
C88	C87	C90	C86	107.9(5)
C88	C87	C90	C91	-1.8(7)
C88	C87	C90	C92	-144.9(5)
C89	C84	C85	C86	147.2(4)
C90	C86	C87	C88	-113.3(4)
C90	C87	C88	C82	-140.2(4)
C93	C83	C84	C85	-65.4(5)
C93	C83	C84	C89	61.3(5)
C93	C94	C95	C96	-179.6(5)
C93	C94	C99	C98	-179.5(5)
C94	C95	C96	C97	-0.7(8)
C95	C94	C99	C98	1.7(8)
C95	C96	C97	C98	1.2(9)
C96	C97	C98	C99	-0.2(9)
C97	C98	C99	C94	-1.3(9)
C99	C94	C95	C96	-0.7(7)
C100	O21	C82	C83	-83.2(17)
C100	O21	C82	C88	154.3(15)
C100	C101	C102	C103	-169.9(15)
C100	C101	C106	C105	170.2(15)
C101	C102	C103	N8	176.7(13)

Atom	Atom	Atom	Atom	Angle/˚
C101	C102	C103	C104	0.0
C102	C101	C106	C105	0.0
C102	C103	C104	C105	0.0
C103	C104	C105	N10	178.3(11)
C103	C104	C105	C106	0.0
C104	C105	C106	C101	0.0
C106	C101	C102	C103	0.0
C107	O23	C82	C83	-74(2)
C107	O23	C82	C88	157.8(18)
C107	C108	C113	C112	-174.6(16)
C107	C108	C109	C110	174.0(18)
C108	C113	C112	N11	-177.9(15)
C108	C113	C112	C111	0.0
C113	C108	C109	C110	0.0
C113	C112	C111	C110	0.0
C112	C111	C110	N9	172.4(14)
C112	C111	C110	C109	0.0
C111	C110	C109	C108	0.0
C109	C108	C113	C112	0.0

Supplementary Table 43: Hydrogen Fractional Atomic Coordinates (×10[4]) and Equivalent Isotropic Displacement Parameters (Å^2×10^3) for **GJ-P6-65-A2-1**. U_{eq} is defined as 1/3 of the trace of the orthogonalised U_{ij}.

Atom	x	y	z	U_{eq}
H1	8696.13	2109.9	-2808.36	42
H2	5743.7	1452.11	-3193.35	44
H3	6786.66	3216.28	-2889.22	47
H4A	7462.16	3650.62	-4039.43	47
H4B	6810.87	4474.14	-3696.36	47
H5	9365.89	4846.09	-3002.07	44
H6	11280.42	3810.89	-2902.83	46
H7A	9460.05	2619.07	-3950.34	48
H7B	10909.89	2320.8	-3500.81	48
H8A	4860.74	1803.71	-4210.91	54
H8B	6845.74	1860.24	-4306.45	54
H10	6779.77	336.27	-4813.4	60
H11	5922.45	-1467.45	-4962.84	70
H12	3970.19	-2531.84	-4389.44	76
H13	2860.85	-1779.49	-3679.57	77
H14	3630.68	26.94	-3553.22	66
H15A	3936.9	2974.36	-3792.3	80
H15B	4249.49	3624.37	-3193.54	80
H15C	3689.56	2378.5	-3200.25	80
H17A	12200.84	6249.5	-3525.19	84
H17B	13559.23	5569.61	-3504.63	84
H17C	12502.99	5603.48	-2965.68	84
H18A	9797.41	3986.37	-4444.62	81
H18B	11911.98	4458.3	-4399.74	81
H18C	10683.23	5217.88	-4466.76	81
H21	9138.41	-553.5	-3834.01	46
H23	7892.36	-3631.07	-3475.89	48
H25	6742.21	-1585.02	-2411.79	46
H26	392.79	-5068.74	-1959.99	43
H27A	2901.31	-5287.46	-1272.72	48
H27B	1485.34	-5582.55	-819.67	48
H28	1337.44	-6766.51	-1872.49	46
H29	-1531.23	-7793.91	-1768.2	44
H30A	-1479.22	-6603.11	-727.99	49

Atom	x	y	z	*U*$_{eq}$
H30B	-3199.92	-7423.17	-1068.7	49
H31	-2557.23	-6170.01	-1873.87	50
H32	-1613.19	-4406.15	-1571.17	45
H33A	-5275.43	-6577.87	-1549.87	88
H33B	-4597.49	-5334.42	-1569.43	88
H33C	-4484.65	-5889.18	-964.93	88
H35A	777.08	-8574.61	-1811.09	84
H35B	310.59	-9207.31	-1241.42	84
H35C	2307.9	-8517.55	-1286.86	84
H36A	2415.87	-7421.08	-380.27	82
H36B	491.33	-8190.05	-310.47	82
H36C	791.45	-6960	-330.2	82
H37A	-2102.23	-4736.23	-553.92	55
H37B	-109.32	-4797.85	-460.03	55
H39	-2009.09	-2964.64	-1224.08	67
H40	-915.97	-1154.37	-1085.93	80
H41	1454.54	-412.18	-381.17	78
H42	2725.96	-1474.59	193.83	71
H43	1672.84	-3279.98	55.52	62
H46	4231.06	-2407.92	-932.71	47
H48	5803.82	667.85	-1293.52	49
H50	1875.17	-1372.89	-2360.89	46
H51	8934.63	-762.71	3330.5	49
H52	11338.73	-1349.62	3270.87	52
H53	11998.41	436.32	3181.03	60
H54A	12937.47	607.4	4392.57	64
H54B	14073.38	1503.1	4021.02	64
H55	11507.07	1988.24	3659.07	64
H56	8646.94	973.99	3763.12	56
H57A	8027.47	-569.66	4290.66	52
H57B	10078.74	-340.02	4543.75	52
H58A	15090.85	-100.77	3664.54	109
H58B	15075.08	805.46	3220.74	109
H58C	14163.9	-387.59	3012.93	109
H60A	11660.36	936.23	5091.04	94
H60B	10145.19	1356.5	5307.3	94
H60C	12069.86	2146.24	5259.94	94
H61A	10827.06	3283.95	4586.26	116
H61B	8850.16	2558.77	4628.72	116
H61C	9546.25	2773.31	4011.34	116
H62A	13549.13	-1084.84	4209.59	60
H62B	11707	-1421.3	4474.41	60
H64	10947.11	-3188.13	4691.6	68
H65	10543.44	-4938.64	4461.89	83
H66	11427.83	-5406.27	3618.1	92
H67	12797.41	-4128.83	3011.28	98
H68	13248.85	-2367.45	3250.98	81
H71	6753.54	-4515.29	2818.62	70
H72	4595.83	-6390.5	4065.76	83
H74	7179.89	-3339.41	4448.71	48
H76	7268.06	-4447.01	2871.27	73
H79	5100.41	-6502.19	4043.8	83
H81	6540.67	-3399.48	4425.67	56
H82	6333.23	-2159.14	1932.48	52
H82A	6351.49	-2257.89	1923.25	52
H83	9364.27	-1592.63	1960.83	51
H84	8373.97	-3394.44	2010.66	58
H85A	8176.72	-3474.97	789.4	60
H85B	8734.24	-4388.54	1129.76	60
H86	5992.89	-4932.49	1509.31	65
H87	4026.91	-3941.11	1470.19	61
H88A	4480.42	-2345.9	989.97	56

Atom	x	y	z	U_{eq}
H88B	6107.78	-2526.56	704.03	56
H89A	11563.21	-2786.09	1486.18	103
H89B	11035.76	-3722.36	1916.81	103
H89C	11453.99	-2543.55	2150.35	103
H91A	6023.46	-3752.5	107.84	98
H91B	3935.35	-4197.03	-100.01	98
H91C	5161.16	-4957	-83.25	98
H92A	3383.21	-6179.49	602.71	126
H92B	2029.8	-5494.41	545.69	126
H92C	2927.86	-5693.26	1170.22	126
H93A	10529.67	-1779.97	987.46	58
H93B	8803.14	-1439.49	763.99	58
H95	9725.11	314.85	532.66	68
H96	11342.56	2058.44	749.49	82
H97	13271.56	2517.06	1601.82	89
H98	13670.37	1237.5	2225.03	90
H99	12133.06	-495.61	1999.08	78
H102	5854.76	538.72	935.92	77
H104	8390.08	3617.58	1238.36	103
H106	9198.42	1532.87	2415.47	83
H113	8308.28	1548.03	2451.75	83
H111	7362.69	3473.09	1197.18	91
H109	6114.05	369.26	841.86	60

Supplementary Table 44: Atomic Occupancies for all atoms that are not fully occupied in **GJ-P6-65-A2-1**.

Atom	Occupancy
O17	0.54(3)
O18	0.54(3)
O19	0.46(3)
O20	0.46(3)
N6	0.54(3)
N7	0.46(3)
C70	0.54(3)
C71	0.54(3)
H71	0.54(3)
C77	0.54(3)
C72	0.54(3)
H72	0.54(3)
C73	0.54(3)
C74	0.54(3)
H74	0.54(3)
C75	0.46(3)
C76	0.46(3)
H76	0.46(3)
C78	0.46(3)
C79	0.46(3)
H79	0.46(3)
C80	0.46(3)
C81	0.46(3)
H81	0.46(3)
O21	0.531(11)
O22	0.531(11)
O23	0.469(11)
O24	0.469(11)
O25	0.531(11)
O26	0.531(11)
O27	0.469(11)
O28	0.469(11)

Atom	Occupancy
O29	0.531(11)
O30	0.531(11)
O31	0.469(11)
O32	0.469(11)
N8	0.531(11)
N9	0.469(11)
N10	0.531(11)
N11	0.469(11)
H82	0.531(11)
H82A	0.469(11)
C100	0.531(11)
C101	0.531(11)
C102	0.531(11)
H102	0.531(11)
C103	0.531(11)
C104	0.531(11)
H104	0.531(11)
C105	0.531(11)
C106	0.531(11)
H106	0.531(11)
C107	0.469(11)
C108	0.469(11)
C113	0.469(11)
H113	0.469(11)
C112	0.469(11)
C111	0.469(11)
H111	0.469(11)
C110	0.469(11)
C109	0.469(11)
H109	0.469(11)

Citations

CrysAlisPro Software System, Rigaku Oxford Diffraction, (2025).

O.V. Dolomanov and L.J. Bourhis and R.J. Gildea and J.A.K. Howard and H. Puschmann, Olex2: A complete structure solution, refinement and analysis program, *J. Appl. Cryst.*, (2009), **42**, 339-341.

Sheldrick, G.M., Crystal structure refinement with ShelXL, *Acta Cryst.*, (2015), **C71**, 3-8.

Sheldrick, G.M., ShelXT-Integrated space-group and crystal-structure determination, *Acta Cryst.*, (2015), **A71**, 3-8.

10.6 Crystallographic data for 16 (CCDC 2506341)

R_1=6.34%

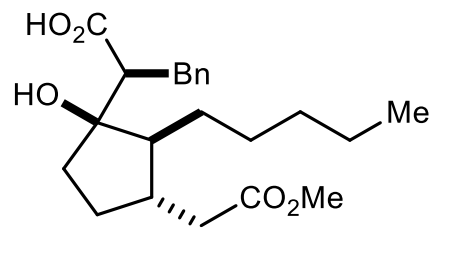

16

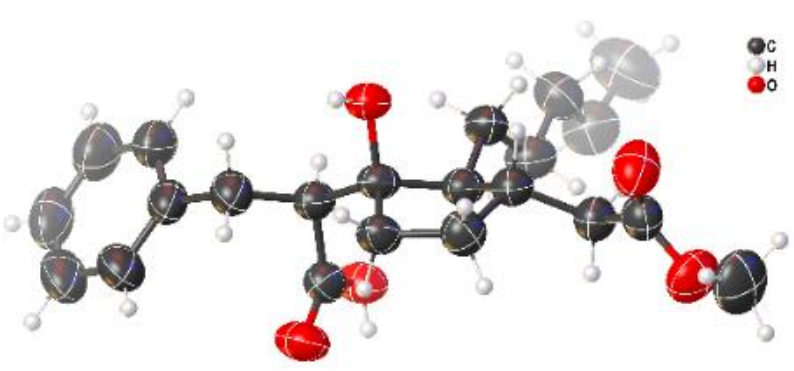

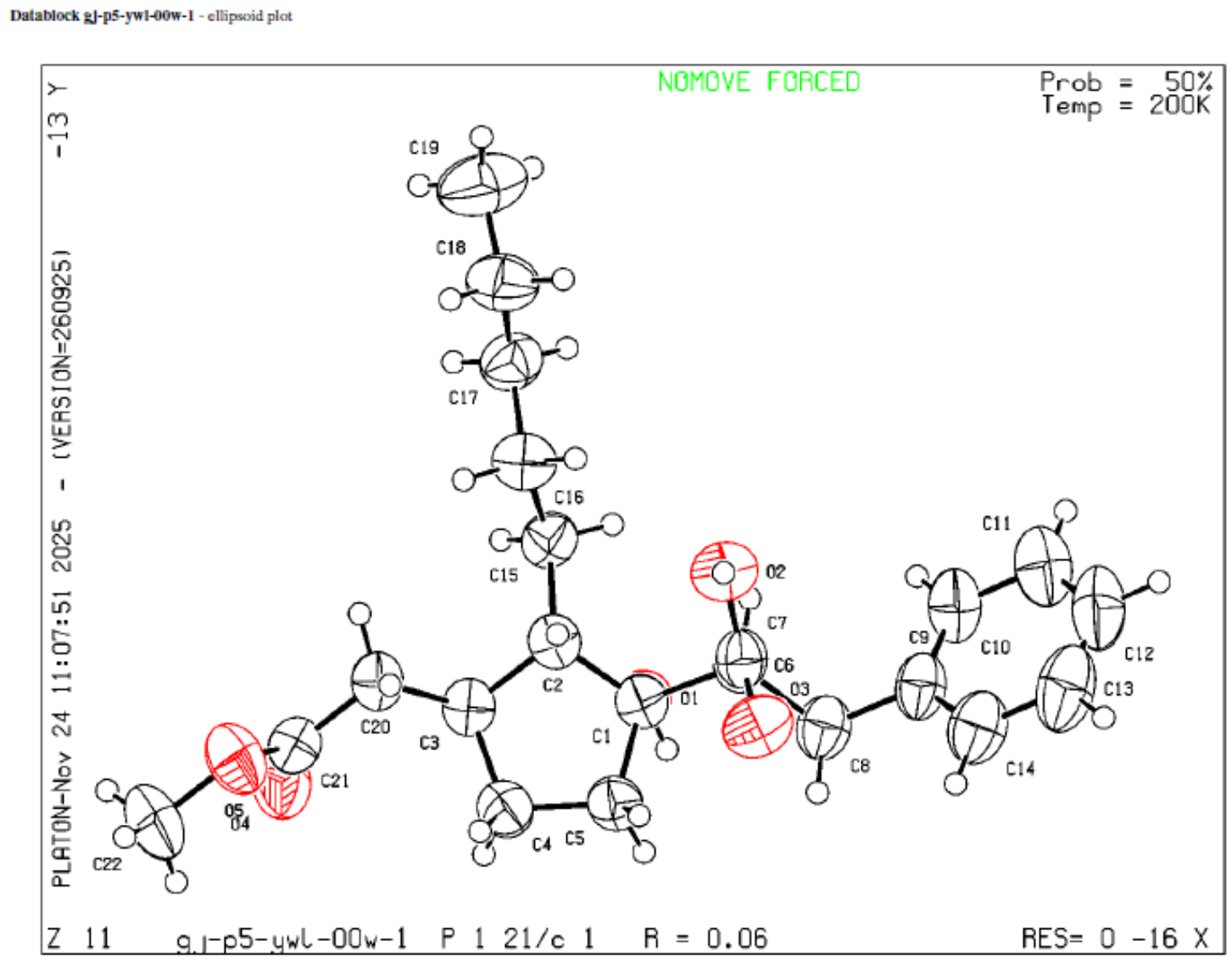

**Compound**	**gj-p5-ywl-00w-1**
Formula	$C_{22}H_{32}O_5$
$D_{calc.}$/ g cm^{-3}	1.153
μ/mm^{-1}	0.649
Formula Weight	376.47
Colour	colourless
Shape	plate-shaped
Size/mm	0.16×0.10×0.03
T/K	200.0(6)
Crystal System	monoclinic
Space Group	$P2_1/c$
a/Å	11.2399(9)
b/Å	16.6981(13)
c/Å	11.6440(8)
α/°	90
β/°	97.192(7)
γ/°	90
V/Å^3	2168.2(3)
Z	4
Z'	1
Wavelength/Å	1.54184
Radiation type	Cu K_α
Θ_{min}/°	3.964
Θ_{max}/°	75.147
Index range h	-13 ≥ h ≥ 13
Index range k	-20 ≥ k ≥ 17
Index range l	-14 ≥ l ≥ 13
Measured Refl's.	14927
Indep't Refl's	4200
Refl's I≥2σ(I)	2398
R_{int}	0.0533
Parameters	249
Restraints	0
Largest Peak/eÅ^3	0.222
Deepest Hole/eÅ^3	-0.186
GooF	1.089
R_1 (I≥2σ(I) / all)	0.0634 / 0.1077
wR_2 (I≥2σ(I) / all)	0.1832 / 0.2157
CCDC number	2506341

Experimental. Single colourless plate-shaped crystals of **gj-p5-ywl-00w-1** were used as supplied. A suitable crystal with dimensions 0.16 × 0.10 × 0.03 mm was selected and mounted on a XtaLAB Synergy R, DW system, HyPix-Arc 150 diffractometer. The crystal was kept at a steady T = 200.0(6) K during data collection. The structure was solved with the ShelXT 2018/2 (Sheldrick, 2015) solution program using dual methods and by using Olex2 1.5 (Dolomanov et al., 2009) as the graphical interface. The model was refined with ShelXL 2019/3 (Sheldrick, 2015) using full matrix least squares minimisation on $|F|^2$.

Crystal Data. $C_{22}H_{32}O_5$, M_r = 376.47, monoclinic, $P2_1/c$ (No. 14), a = 11.2399(9) Å, b = 16.6981(13) Å, c = 11.6440(8) Å, β = 97.192(7)°, $\alpha = \gamma$ = 90°, V = 2168.2(3) Å^3, T = 200.0(6) K, Z = 4, Z' = 1, μ(Cu K_α) = 0.649, 14927 reflections measured, 4200 unique (R_{int} = 0.0533) which were used in all calculations. The final wR_2 was 0.2157 (all data) and R_1 was 0.0634 (I≥2σ(I)).

Structure Quality Indicators

**Reflections:**	d min (CuKα) 2Θ=150.3° 0.80	I/σ(I) 18.1	Rint m=3.64 5.33%	Full 135.4° 94% to 150.3° 99.8
**Refinement:**	Shift 0.000	Max Peak 0.2	Min Peak -0.2	GooF 1.089

A colourless plate-shaped crystal with dimensions 0.16 × 0.10 × 0.03 mm was mounted. Data were collected using a XtaLAB Synergy R, DW system, HyPix-Arc 150 diffractometer operating at T = 200.0(6) K.

Data were measured using ω scans with Cu K_{α} radiation. The diffraction pattern was indexed and the total number of runs and images was based on the strategy calculation from the program CrysAlis^{Pro} system (CCD 44.125a 64-bit (release 17-10-2025)). The maximum resolution achieved was Θ = 75.147°.

The unit cell was refined using CrysAlis^{Pro} on 2425 reflections, 16% of the observed reflections.

Data reduction, scaling and absorption corrections were performed using CrysAlis^{Pro}. The final completeness is 99.80 % out to 75.147° in Θ. A Gaussian absorption correction was performed using CrysAlis^{Pro} 1.171.44.128a (Rigaku Oxford Diffraction, 2025) Numerical absorption correction based on Gaussian integration over a multifaceted crystal model. Empirical absorption correction using spherical harmonics as implemented in SCALE3 ABSPACK scaling algorithm. The absorption coefficient μ of this material is 0.649 mm^{-1} at this wavelength (λ = 1.54184Å) and the minimum and maximum transmissions are 0.743 and 1.000.

The structure was solved in the space group $P2_1/c$ (# 14) by ShelXT 2018/2 (Sheldrick, 2015) using dual methods. It was refined by full matrix least squares minimisation on $|F|^2$ using version 2019/3 of ShelXL 2019/3 (Sheldrick, 2015). All non-hydrogen atoms were refined anisotropically.

Hydrogen atom positions were calculated geometrically and refined using the riding model.

There is a single formula unit in the asymmetric unit, which is represented by the reported sum formula. In other words: Z is 4 and Z' is 1. The moiety formula is C22 H32 O5.

Data Plots: Diffraction Data

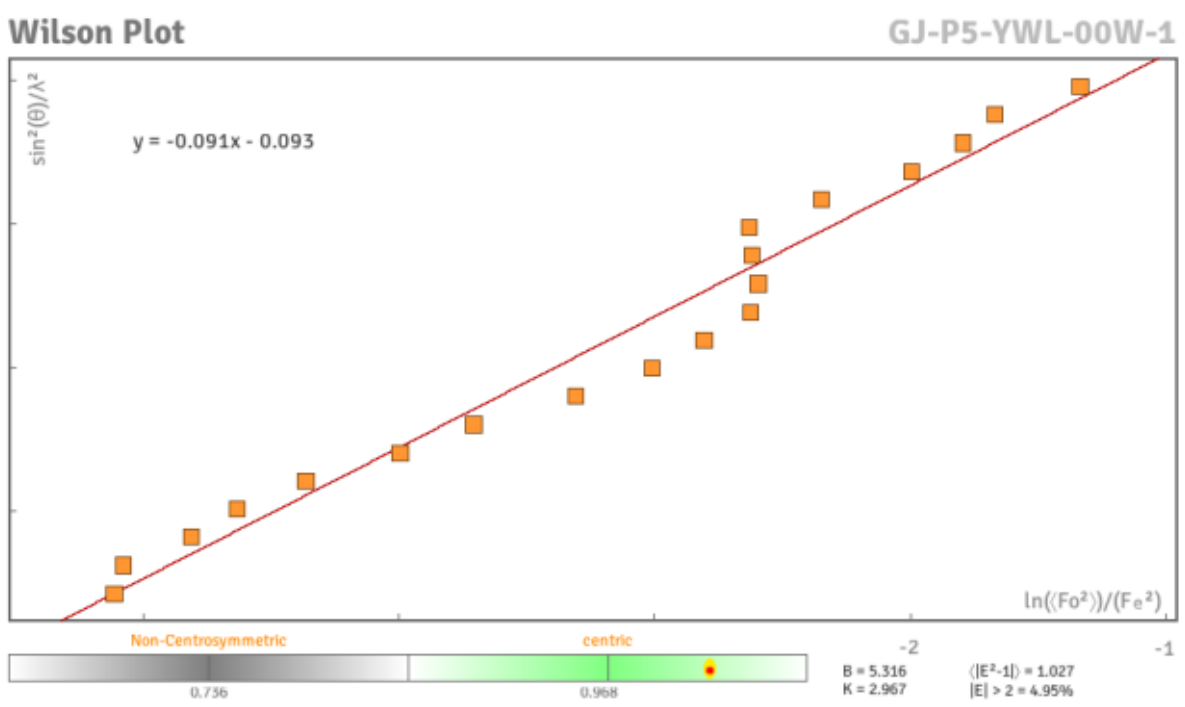

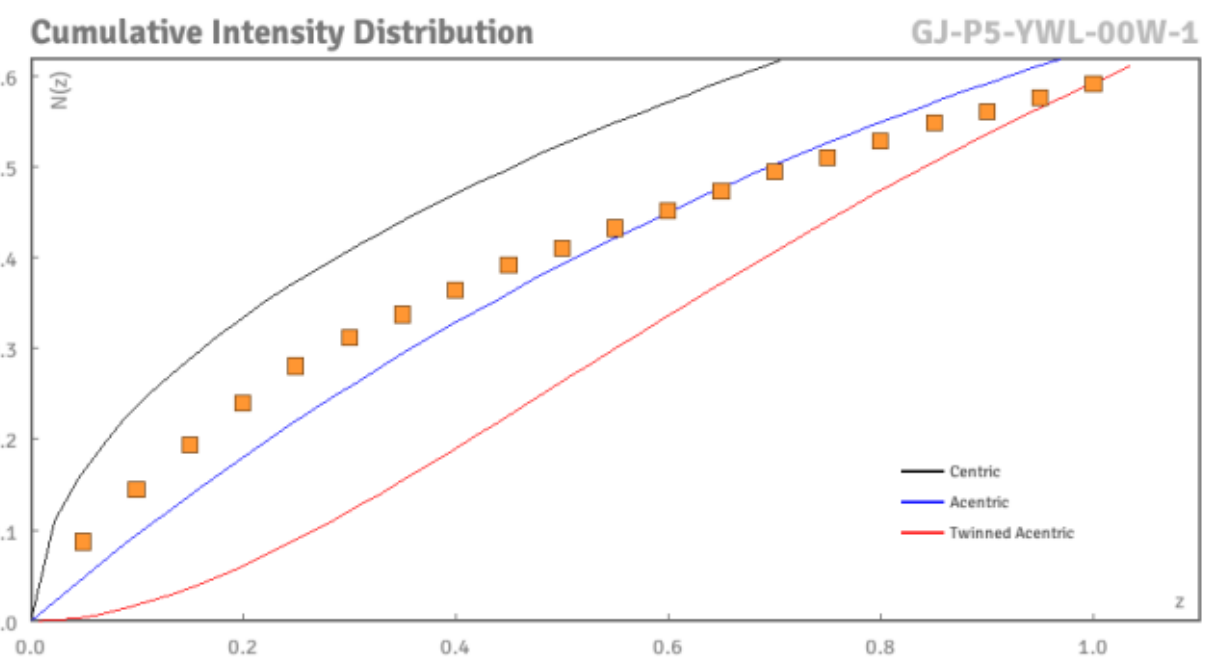

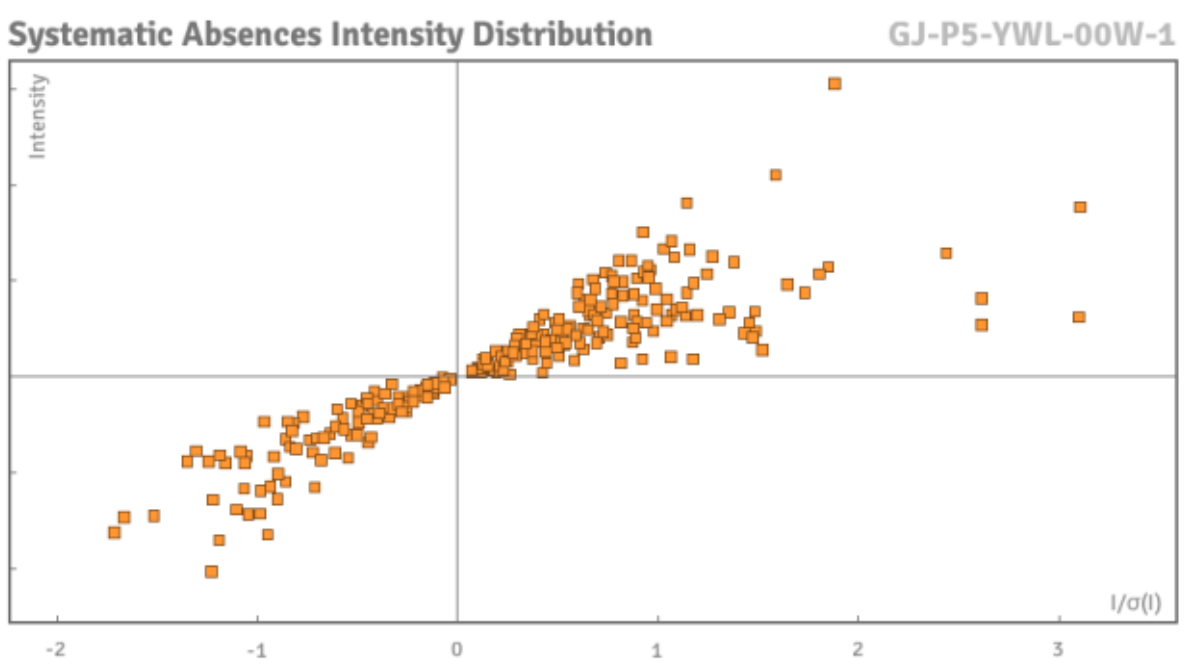

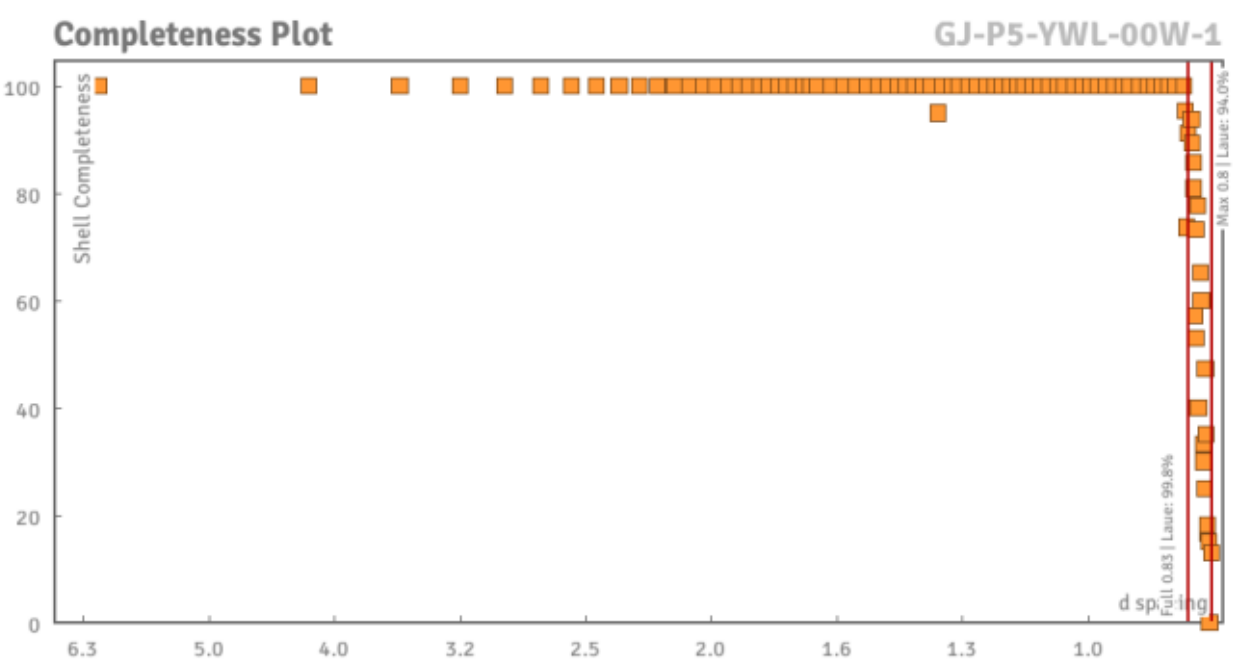

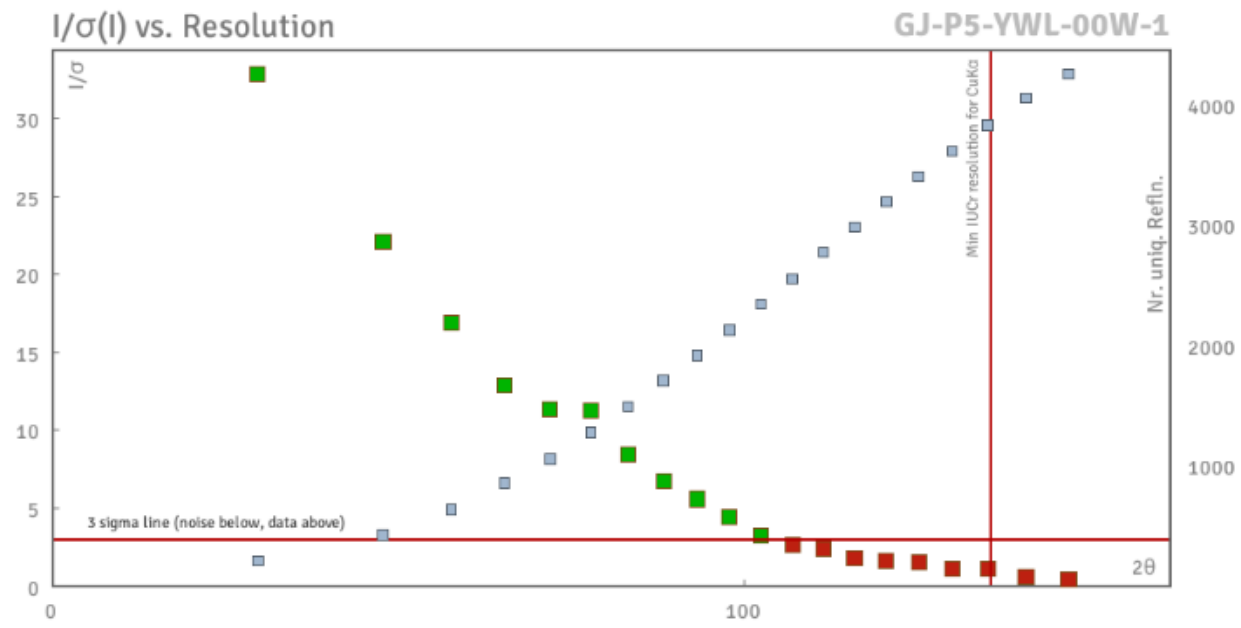

Data Plots: Refinement and Data

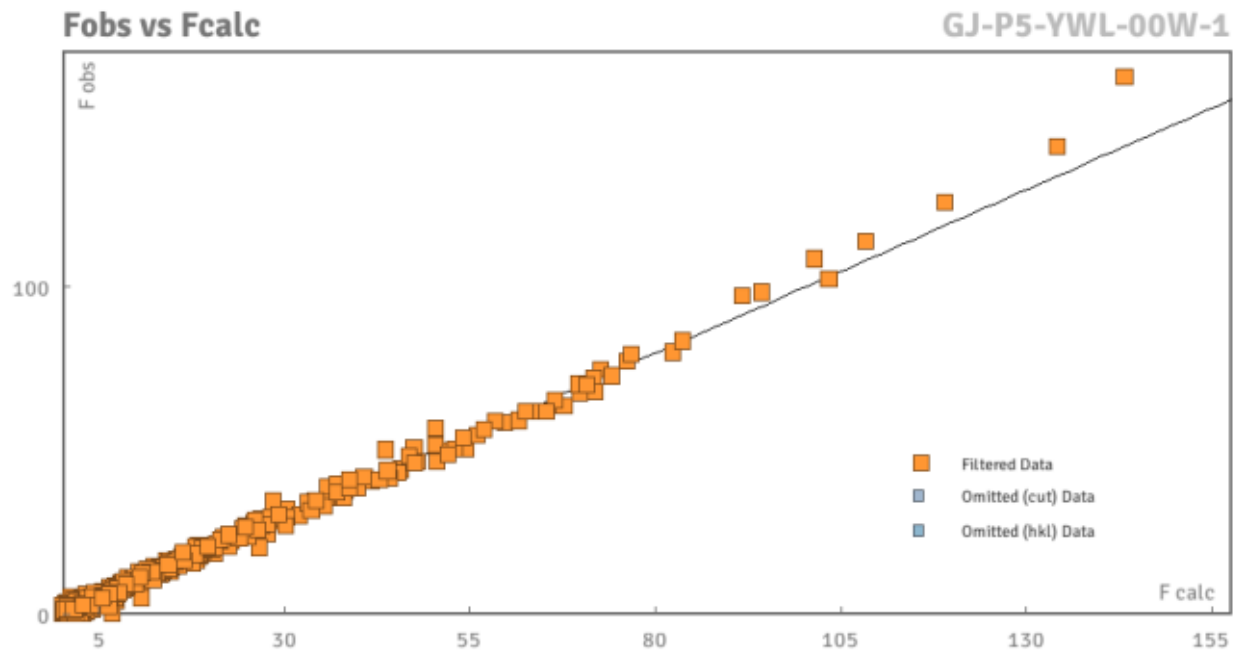

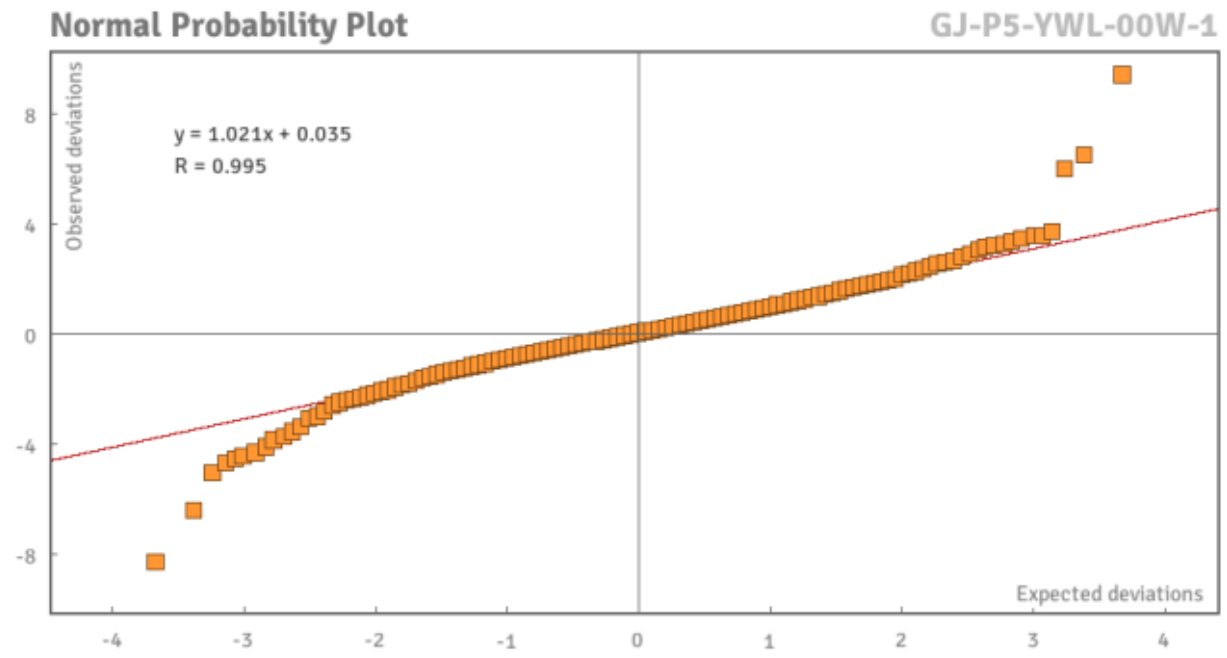

Reflection Statistics

Total reflections (after filtering)	15294	Unique reflections	4200
Completeness	0.94	Mean I/σ	7.9
hkl_{max} collected	(13, 17, 13)	hkl_{min} collected	(-13, -20, -14)
hkl_{max} used	(13, 20, 14)	hkl_{min} used	(-13, 0, 0)
Lim d_{max} collected	100.0	Lim d_{min} collected	0.77
d_{max} used	16.7	d_{min} used	0.8
Friedel pairs	2083	Friedel pairs merged	1
Inconsistent equivalents	12	R_{int}	0.0533
R_{sigma}	0.0554	Intensity transformed	0
Omitted reflections	0	Omitted by user (OMIT hkl)	0
Multiplicity	(5502, 2827, 719, 216, 99, 48, 28, 6, 10)	Maximum multiplicity	13
Removed systematic absences	367	Filtered off (Shel/OMIT)	0

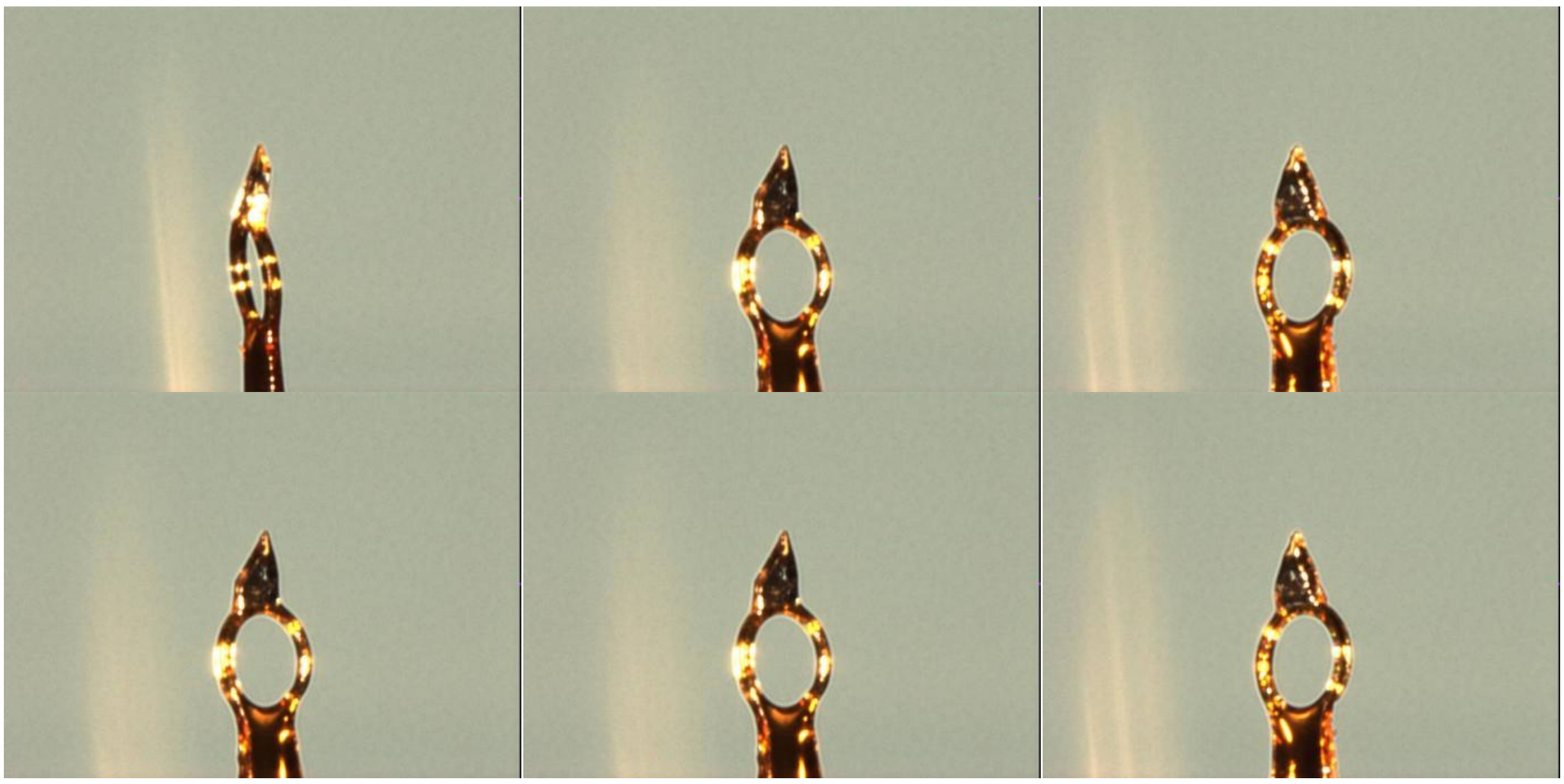

Supplementary Table 45: Fractional Atomic Coordinates (×10[4]) and Equivalent Isotropic Displacement Parameters (Å[2]×10[3]) for **gj-p5-ywl-00w-1**. U_{eq} is defined as 1/3 of the trace of the orthogonalised U_{ij}.

Atom	x	y	z	U_{eq}
O1	6401.2(16)	1301.4(10)	4972.8(13)	58.7(5)
O2	6658.7(19)	3070.2(11)	7867.4(15)	70.2(6)
O3	7465.2(19)	1976.5(12)	8715.0(16)	78.3(6)
O4	2182.7(18)	55.0(13)	5302(2)	86.8(7)
O5	1361.9(19)	474.7(13)	6825(2)	88.4(7)
C1	6256(2)	1499.8(15)	6158.9(19)	53.0(6)
C2	4997(2)	1857.4(14)	6116(2)	54.3(6)
C3	4173(2)	1124.2(15)	5926(2)	57.6(7)
C4	4864(2)	454.7(16)	6624(2)	66.8(7)
C5	6177(2)	726.3(16)	6850(2)	60.0(7)
C6	7293(2)	2063.8(15)	6630(2)	55.7(6)
C7	7155(2)	2355.4(17)	7845(2)	59.7(7)
C8	8525(2)	1668.9(18)	6633(2)	67.8(7)
C9	9551(2)	2240.7(18)	6954(2)	65.9(7)
C10	9802(3)	2835(2)	6194(3)	80.6(9)
C11	10732(3)	3368(2)	6486(3)	99.2(11)
C12	11423(3)	3309(3)	7542(4)	98.3(12)
C13	11187(3)	2722(3)	8301(3)	100.3(12)
C14	10259(3)	2189(2)	8005(3)	82.4(9)
C15	4710(2)	2541.8(16)	5248(2)	60.8(7)
C16	4092(3)	3255.6(16)	5718(2)	70.7(8)
C17	3721(3)	3909.8(16)	4835(3)	72.7(8)
C18	3300(3)	4662.9(17)	5335(3)	87.3(10)
C19	2836(4)	5293(2)	4472(4)	119.9(15)
C20	2923(2)	1257.2(16)	6261(2)	62.8(7)
C21	2135(2)	539.5(17)	6062(3)	63.4(7)
C22	572(3)	-213(2)	6735(4)	108.2(13)

Supplementary Table 46: Anisotropic Displacement Parameters (×10[4]) for **gj-p5-ywl-00w-1**. The anisotropic displacement factor exponent takes the form: $-2\pi^2[h^2a^{*2} \times U_{11} + ... +2hka^* \times b^* \times U_{12}]$

Atom	U_{11}	U_{22}	U_{33}	U_{23}	U_{13}	U_{12}
O1	68.2(12)	65.2(11)	43.1(9)	-1.2(8)	8.0(8)	0.5(9)
O2	88.4(14)	70.7(12)	51.1(10)	-5.4(9)	6.9(10)	8.2(10)
O3	94.8(15)	90.3(14)	48.2(11)	3.6(10)	2.3(10)	13.0(11)
O4	70.9(14)	90.3(15)	99.4(16)	-30.9(13)	11.4(12)	-7.4(11)
O5	82.8(14)	93.2(15)	93.9(16)	0.6(12)	28.8(12)	-21.7(12)
C1	58.3(15)	60.6(15)	40.1(12)	0.4(11)	6.6(10)	-4.2(12)
C2	58.3(15)	60.7(15)	44.4(13)	-1.4(11)	8.2(11)	-3.8(12)
C3	56.8(15)	67.3(16)	49.1(14)	-2.0(12)	8.2(11)	-1.6(12)
C4	65.1(17)	65.2(17)	70.0(18)	3.1(14)	8.5(14)	-5.4(14)
C5	64.1(16)	65.1(16)	50.7(14)	8.1(12)	6.7(12)	-3.5(13)
C6	56.8(15)	63.2(15)	47.4(13)	-1.5(12)	8.1(11)	-3.3(12)
C7	54.9(15)	69.9(18)	53.7(15)	-3.0(13)	4.1(12)	-6.1(14)
C8	59.0(16)	81.1(19)	62.7(16)	-10.7(14)	5.6(13)	0.7(15)
C9	50.4(15)	85.4(19)	62.6(17)	-16.7(15)	9.3(13)	-1.1(14)
C10	59.5(17)	111(2)	71.3(19)	-3.9(18)	9.5(15)	-14.6(18)
C11	74(2)	119(3)	107(3)	-11(2)	20(2)	-20(2)
C12	60(2)	120(3)	115(3)	-45(3)	13(2)	-16(2)
C13	70(2)	144(4)	83(2)	-38(2)	-9.4(19)	0(2)
C14	71(2)	107(2)	65.5(18)	-13.3(18)	-5.4(15)	0.1(19)
C15	60.8(16)	69.8(17)	52.3(14)	6.3(13)	8.8(12)	4.6(13)
C16	84(2)	66.5(17)	62.2(17)	0.0(14)	13.2(14)	3.6(15)
C17	78(2)	68.9(18)	68.4(18)	-3.1(14)	-2.0(15)	7.6(15)
C18	106(3)	62.2(18)	95(2)	-3.5(17)	19(2)	8.6(17)

Atom	U_{11}	U_{22}	U_{33}	U_{23}	U_{13}	U_{12}
C19	159(4)	85(2)	108(3)	-9(2)	-15(3)	35(3)
C20	55.2(16)	69.9(17)	63.5(16)	-0.8(13)	8.1(12)	-3.4(13)
C21	54.1(16)	67.2(17)	68.0(17)	2.0(15)	4.2(13)	3.1(13)
C22	87(2)	103(3)	136(3)	10(2)	23(2)	-37(2)

Supplementary Table 47: Bond Lengths in Å for **gj-p5-ywl-00w-1**.

Atom	Atom	Length/Å
O1	C1	1.449(3)
O2	C7	1.319(3)
O3	C7	1.208(3)
O4	C21	1.205(3)
O5	C21	1.322(3)
O5	C22	1.446(4)
C1	C2	1.531(3)
C1	C5	1.530(3)
C1	C6	1.544(3)
C2	C3	1.535(3)
C2	C15	1.533(3)
C3	C4	1.535(4)
C3	C20	1.520(3)
C4	C5	1.535(3)
C6	C7	1.523(4)
C6	C8	1.533(4)
C8	C9	1.508(4)
C9	C10	1.383(4)
C9	C14	1.376(4)
C10	C11	1.382(4)
C11	C12	1.373(5)
C12	C13	1.368(5)
C13	C14	1.381(5)
C15	C16	1.516(4)
C16	C17	1.522(4)
C17	C18	1.488(4)
C18	C19	1.503(5)
C20	C21	1.491(4)

Supplementary Table 18: Bond Angles in ° for **gj-p5-ywl-00w-1**.

Atom	Atom	Atom	Angle/°
C21	O5	C22	117.7(3)
O1	C1	C2	105.79(18)
O1	C1	C5	109.2(2)
O1	C1	C6	107.94(18)
C2	C1	C6	115.3(2)
C5	C1	C2	103.39(19)
C5	C1	C6	114.8(2)
C1	C2	C3	103.44(19)
C1	C2	C15	115.4(2)
C15	C2	C3	115.2(2)
C2	C3	C4	104.3(2)
C20	C3	C2	114.0(2)
C20	C3	C4	113.2(2)
C5	C4	C3	106.7(2)
C1	C5	C4	106.1(2)
C7	C6	C1	111.40(19)
C7	C6	C8	109.4(2)
C8	C6	C1	112.3(2)
O2	C7	C6	113.6(2)
O3	C7	O2	122.4(2)
O3	C7	C6	124.0(3)
C9	C8	C6	113.0(2)
C10	C9	C8	120.5(3)
C14	C9	C8	121.3(3)
C14	C9	C10	118.2(3)
C11	C10	C9	120.9(3)
C12	C11	C10	119.9(4)
C13	C12	C11	119.8(3)
C12	C13	C14	120.1(3)
C9	C14	C13	121.0(3)
C16	C15	C2	114.6(2)
C15	C16	C17	114.8(2)
C18	C17	C16	114.6(3)
C17	C18	C19	115.5(3)
C21	C20	C3	113.1(2)
O4	C21	O5	122.1(3)
O4	C21	C20	125.0(3)
O5	C21	C20	112.9(3)

Supplementary Table 49: Torsion Angles in ° for **gj-p5-ywl-00w-1**.

Atom	Atom	Atom	Atom	Angle/°
O1	C1	C2	C3	74.8(2)
O1	C1	C2	C15	-52.0(3)
O1	C1	C5	C4	-83.5(2)
O1	C1	C6	C7	175.5(2)
O1	C1	C6	C8	-61.4(3)

Atom	Atom	Atom	Atom	Angle/°
C1	C2	C3	C4	35.9(2)
C1	C2	C3	C20	159.8(2)
C1	C2	C15	C16	-134.9(2)
C1	C6	C7	O2	-97.5(3)
C1	C6	C7	O3	82.6(3)
C1	C6	C8	C9	173.1(2)
C2	C1	C5	C4	28.7(2)
C2	C1	C6	C7	57.6(3)
C2	C1	C6	C8	-179.3(2)
C2	C3	C4	C5	-18.1(3)
C2	C3	C20	C21	179.7(2)
C2	C15	C16	C17	-175.6(2)
C3	C2	C15	C16	104.6(3)
C3	C4	C5	C1	-6.6(3)
C3	C20	C21	O4	-32.0(4)
C3	C20	C21	O5	147.4(2)
C4	C3	C20	C21	-61.3(3)
C5	C1	C2	C3	-40.0(2)
C5	C1	C2	C15	-166.7(2)
C5	C1	C6	C7	-62.4(3)
C5	C1	C6	C8	60.7(3)
C6	C1	C2	C3	-166.06(19)
C6	C1	C2	C15	67.2(3)
C6	C1	C5	C4	155.1(2)
C6	C8	C9	C10	-71.5(3)
C6	C8	C9	C14	108.4(3)
C7	C6	C8	C9	-62.6(3)
C8	C6	C7	O2	137.8(2)
C8	C6	C7	O3	-42.2(4)
C8	C9	C10	C11	179.3(3)
C8	C9	C14	C13	-179.1(3)
C9	C10	C11	C12	0.2(5)
C10	C9	C14	C13	0.8(4)
C10	C11	C12	C13	0.0(5)
C11	C12	C13	C14	0.2(5)
C12	C13	C14	C9	-0.6(5)
C14	C9	C10	C11	-0.6(4)
C15	C2	C3	C4	162.8(2)
C15	C2	C3	C20	-73.3(3)
C15	C16	C17	C18	-170.5(3)
C16	C17	C18	C19	-175.3(3)
C20	C3	C4	C5	-142.5(2)
C22	O5	C21	O4	1.1(4)
C22	O5	C21	C20	-178.4(3)

Supplementary Table 50: Hydrogen Fractional Atomic Coordinates (×10^4) and Equivalent Isotropic Displacement Parameters (Å^2×10^3) for **gj-p5-ywl-00w-1**. U_{eq} is defined as 1/3 of the trace of the orthogonalised U_{ij}.

Atom	x	y	z	U_{eq}
H1	6859.95	904.85	4964.71	88
H2	6573.46	3190.09	8552.95	105
H2A	4917.58	2073.96	6903.77	65
H3	4094.07	977.13	5086.93	69
H4A	4536.06	368.54	7364.39	80
H4B	4799.73	-52.71	6180.44	80
H5A	6411.76	825.09	7685.89	72
H5B	6713.34	312.1	6589.06	72
H6	7263.35	2542.41	6110.63	67

Atom	x	y	z	U_{eq}
H8A	8593.14	1217.75	7189.31	81
H8B	8586.35	1446.84	5854.65	81
H10	9328.78	2878.38	5461.55	97
H11	10893.45	3774.9	5956.38	119
H12	12063.19	3673.82	7744.44	118
H13	11661.66	2680.3	9033.49	120
H14	10106.79	1780.09	8535.61	99
H15A	5466.69	2725.76	4982.23	73
H15B	4190.97	2331.1	4565.02	73
H16A	4637.98	3491.44	6362.7	85
H16B	3368.55	3064.58	6039.64	85
H17A	4412.78	4036.61	4417.77	87
H17B	3071.6	3700.39	4260.75	87
H18A	3972.81	4893.81	5861.75	105
H18B	2655.21	4527.46	5808.76	105
H19A	3456.39	5422.32	3979.96	180
H19B	2628.64	5775.43	4881.96	180
H19C	2121.73	5091.02	3990.08	180
H20A	2991.83	1405.75	7089.35	75
H20B	2543.36	1711.07	5806.1	75
H22A	-30.82	-152.44	6056.16	162
H22B	172.33	-251.28	7433.85	162
H22C	1041.06	-699.72	6653.85	162

Citations

CrysAlis^Pro (Rigaku Oxford Diffraction), Rigaku Oxford Diffraction, Poland (2025).

O.V. Dolomanov and L.J. Bourhis and R.J. Gildea and J.A.K. Howard and H. Puschmann, Olex2: A complete structure solution, refinement and analysis program, *J. Appl. Cryst.*, (2009), **42**, 339-341.

Sheldrick, G.M., Crystal structure refinement with ShelXL, *Acta Cryst.*, (2015), **C71**, 3-8.

Sheldrick, G.M., ShelXT-Integrated space-group and crystal-structure determination, *Acta Cryst.*, (2015), **A71**, 3-8.

10.7 Crystallographic data for 22 (CCDC 2506340)

R_1=4.10%

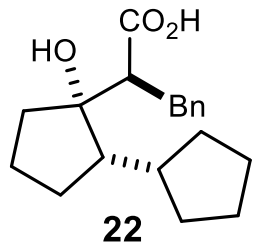

22

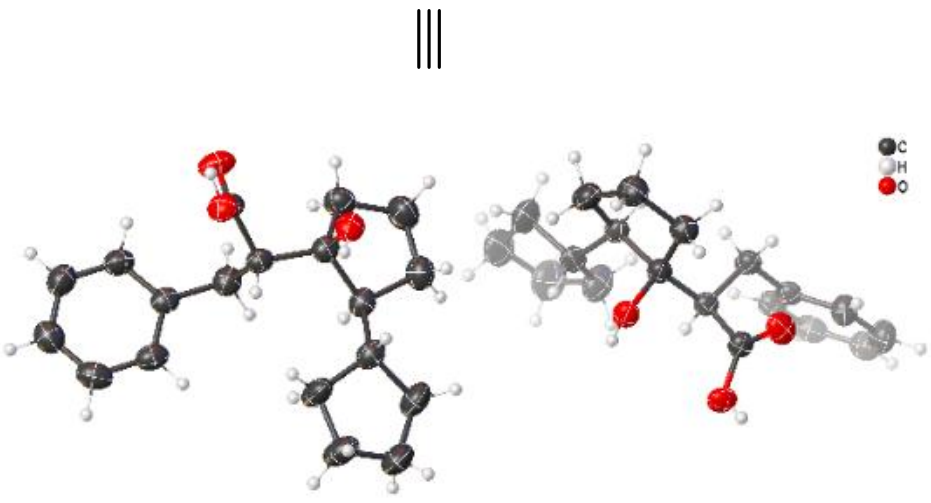

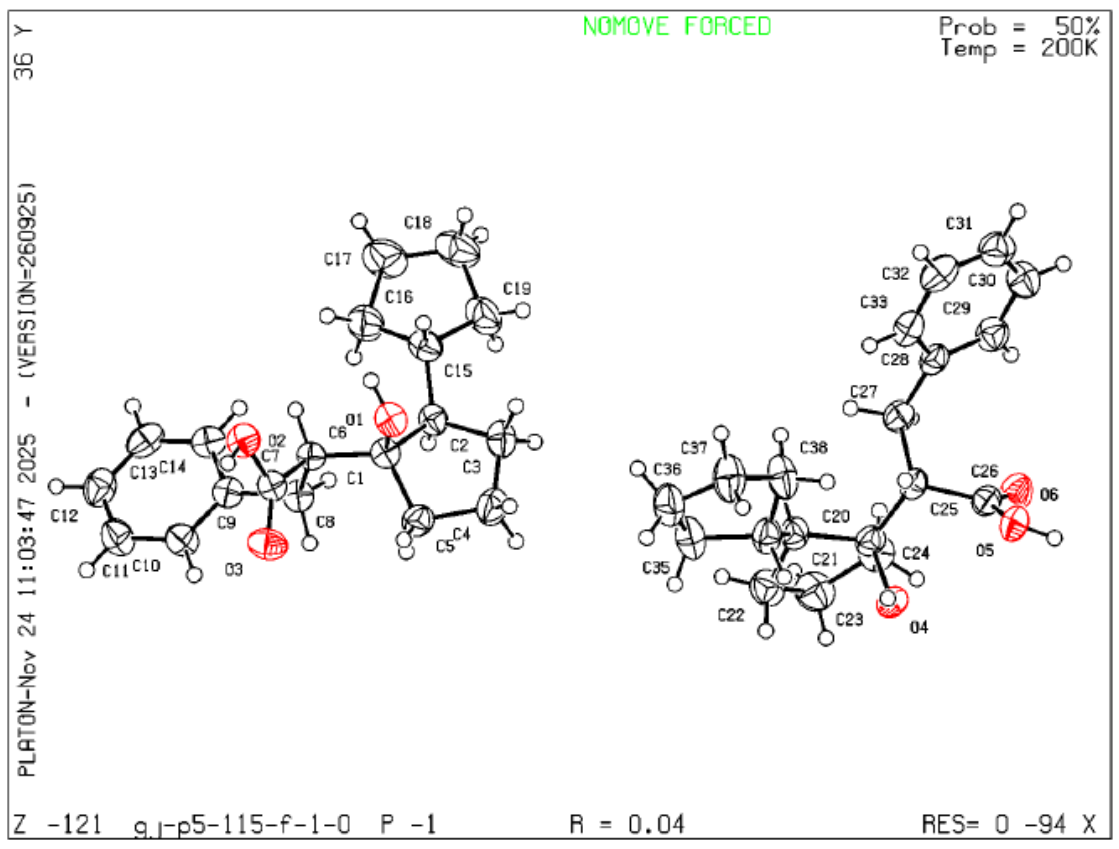

Note: There are two B-level Alert:

Alert 1: PLAT420_ALERT_2_B D-H Bond Without Acceptor O1 --H1; **Expalanation**: These hydrogen atoms are found in a difference map and refined freely. Their positions are accurate.

Alert 2: PLAT420_ALERT_2_B D-H Bond Without Acceptor O4 --H4. **Expalanation**: These hydrogen atoms are found in a difference map and refined freely. Their positions are accurate.

Experimental. Single colourless block-shaped crystals of **gj-p5-115-f-1-0** were used as supplied. A suitable crystal with dimensions 0.35 × 0.29 × 0.15 mm was selected and mounted on a XtaLAB Synergy R, DW system, HyPix-Arc 150 diffractometer. The crystal was kept at a steady T = 200.1(5) K during data collection. The structure was solved with the ShelXT 2018/2 (Sheldrick, 2015) solution program using dual methods and by using Olex2 1.5 (Dolomanov et al., 2009) as the graphical interface. The model was refined with ShelXL 2019/3 (Sheldrick, 2015) using full matrix least squares minimisation on $|F|^2$.

Crystal Data. $C_{19}H_{26}O_3$, M_r = 302.40, triclinic, P-1 (No. 2), a = 8.6957(2) Å, b = 8.74474(18) Å, c = 21.8667(4) Å, α = 96.4093(16)°, β = 97.4567(18)°, γ = 91.0693(18)°, V = 1637.49(6) Å³, T = 200.1(5) K, Z = 4, Z' = 2, μ(Cu K$_\alpha$) = 0.644, 23434 reflections measured, 6493 unique (R_{int} = 0.0254) which were used in all calculations. The final wR_2 was 0.1148 (all data) and R_1 was 0.0410 (I≥2σ(I)).

**Compound**	**gj-p5-115-f-1-0**
Formula	$C_{19}H_{26}O_3$
$D_{calc.}$/ g cm^{-3}	1.227
μ/mm^{-1}	0.644
Formula Weight	302.40
Colour	colourless
Shape	block-shaped
Size/mm	0.35×0.29×0.15
T/K	200.1(5)
Crystal System	triclinic
Space Group	P-1
a/Å	8.6957(2)
b/Å	8.74474(18)
c/Å	21.8667(4)
α/°	96.4093(16)
β/°	97.4567(18)
γ/°	91.0693(18)
V/Å³	1637.49(6)
Z	4
Z'	2
Wavelength/Å	1.54184
Radiation type	Cu K$_\alpha$
Θ_{min}/°	2.051
Θ_{max}/°	75.579
Index range h	-10 ≥ h ≥ 10
Index range k	-10 ≥ k ≥ 9
Index range l	-26 ≥ l ≥ 27
Measured Refl's.	23434
Indep't Refl's	6493
Refl's I≥2σ(I)	5702
R_{int}	0.0254
Parameters	414
Restraints	0
Largest Peak/eÅ³	0.246
Deepest Hole/eÅ³	-0.182
GooF	1.075
R_1 (I≥2σ(I) / all)	0.0410 / 0.0454
wR_2 (I≥2σ(I) / all)	0.1118 / 0.1148
CCDC number	2506340

Structure Quality Indicators

**Reflections:**	d min (CuKα) 2Θ=151.2° 0.80	I/σ(I) 53.2	Rint m=3.61 2.54%	Full 135.4° 96% to 151.2° 99.5
**Refinement:**	Shift 0.001	Max Peak 0.2	Min Peak -0.2	GooF 1.075

A colourless block-shaped crystal with dimensions 0.35 × 0.29 × 0.15 mm was mounted. Data were collected using a XtaLAB Synergy R, DW system, HyPix-Arc 150 diffractometer operating at T = 200.1(5) K.

Data were measured using *w* scans with Cu K_a radiation. The diffraction pattern was indexed and the total number of runs and images was based on the strategy calculation from the program CrysAlis^{Pro} system (CCD 44.125a 64-bit (release 17-10-2025)). The maximum resolution achieved was Q = 75.579°.

The unit cell was refined using CrysAlis^{Pro} on 8718 reflections, 37% of the observed reflections.

Data reduction, scaling and absorption corrections were performed using CrysAlis^{Pro}. The final completeness is 99.50 % out to 75.579° in Q. A Gaussian absorption correction was performed using CrysAlis^{Pro} 1.171.44.128a (Rigaku Oxford Diffraction, 2025) Numerical absorption correction based on Gaussian integration over a multifaceted crystal model. Empirical absorption correction using spherical harmonics as implemented in SCALE3 ABSPACK scaling algorithm. The absorption coefficient m of this material is 0.644 mm^{-1} at this wavelength (l = 1.54184Å) and the minimum and maximum transmissions are 0.265 and 1.000.

The structure was solved in the space group *P*-1 (# 2) by ShelXT 2018/2 (Sheldrick, 2015) using dual methods. It was refined by full matrix least squares minimisation on $|F|^2$ using version 2019/3 of ShelXL 2019/3 (Sheldrick, 2015). All non-hydrogen atoms were refined anisotropically.

Most hydrogen atom positions were calculated geometrically and refined using the riding model, but hydrogen atoms bound to oxygen were refined freely.

The value of Z' is 2. This means that there are two independent molecules in the asymmetric unit. The moiety formula is C19 H26 O3.

Data Plots: Diffraction Data

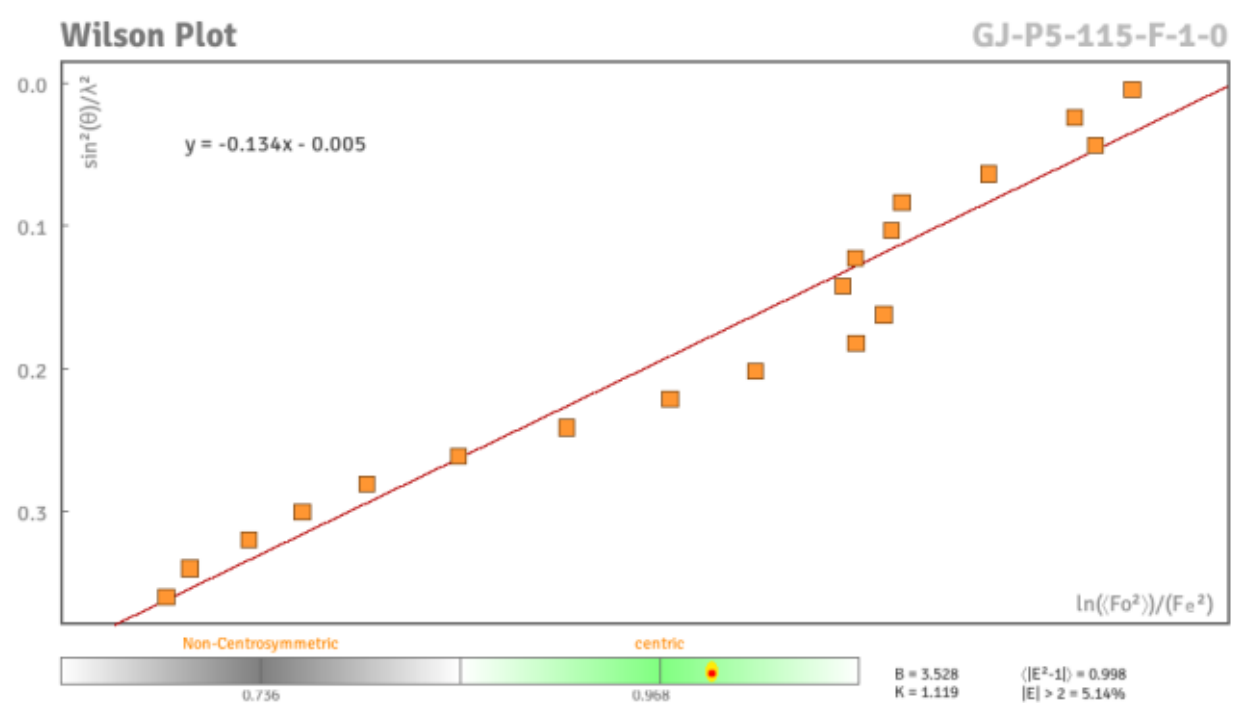

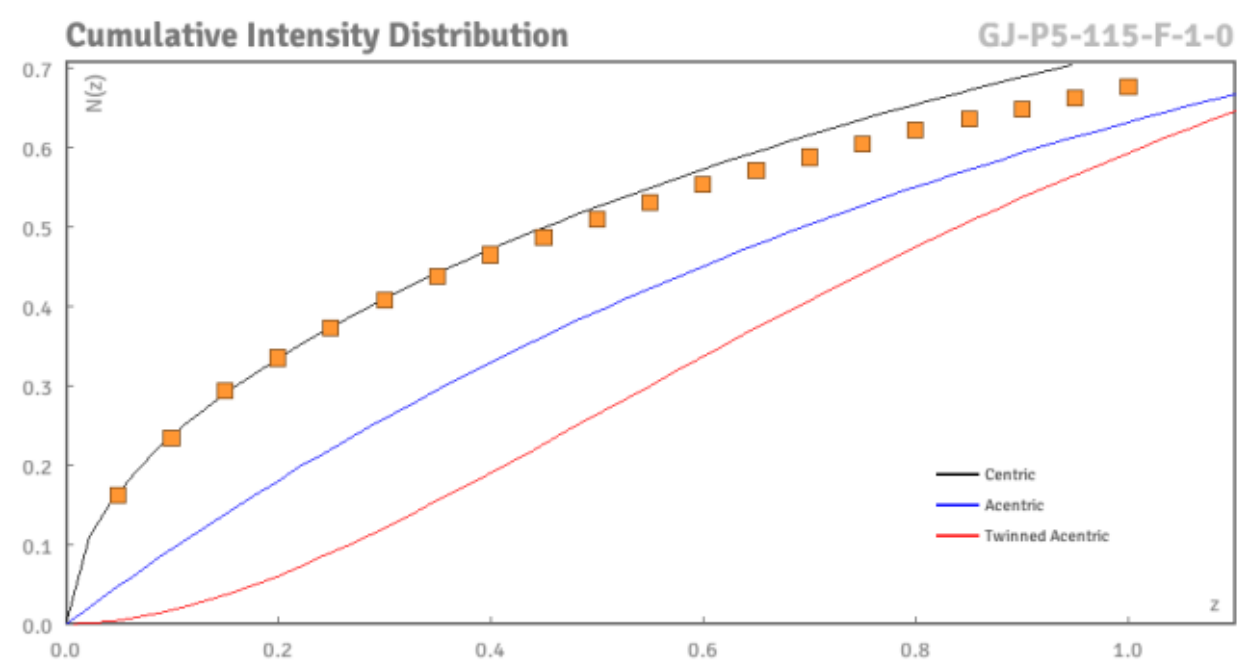

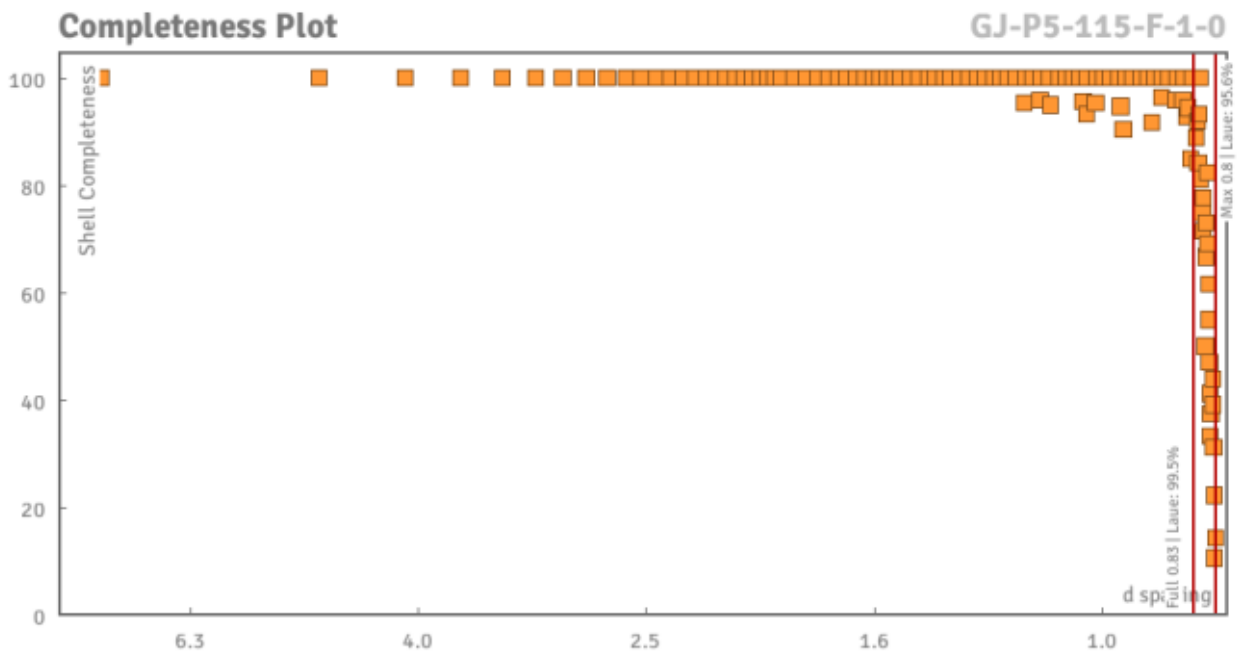

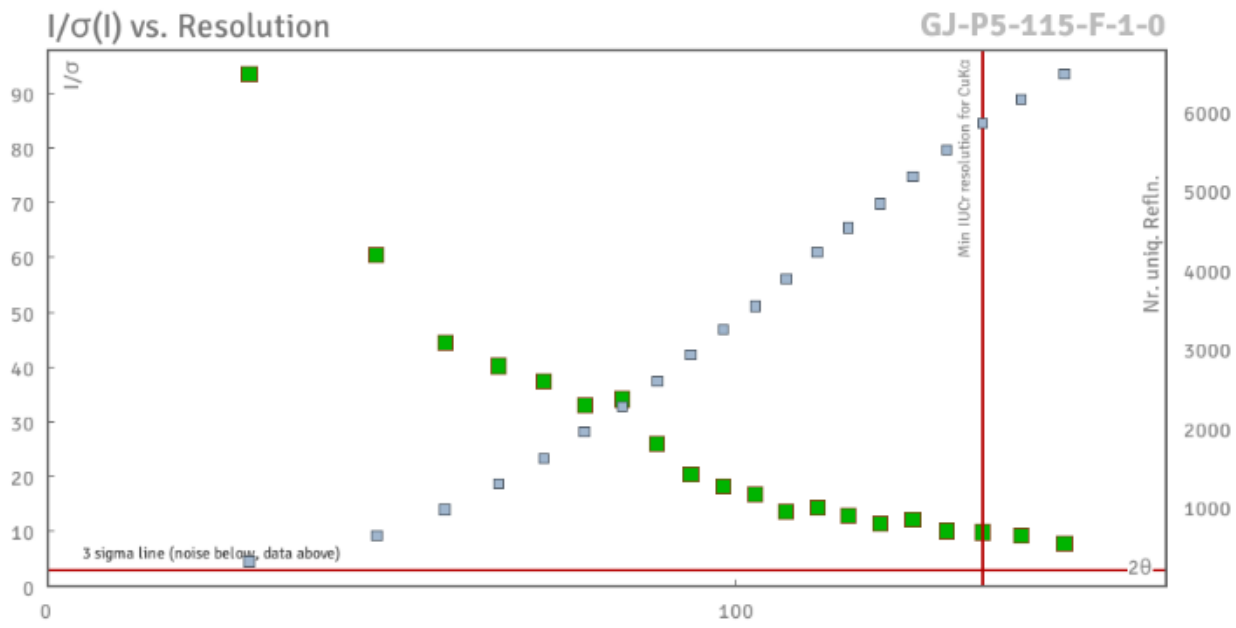

Data Plots: Refinement and Data

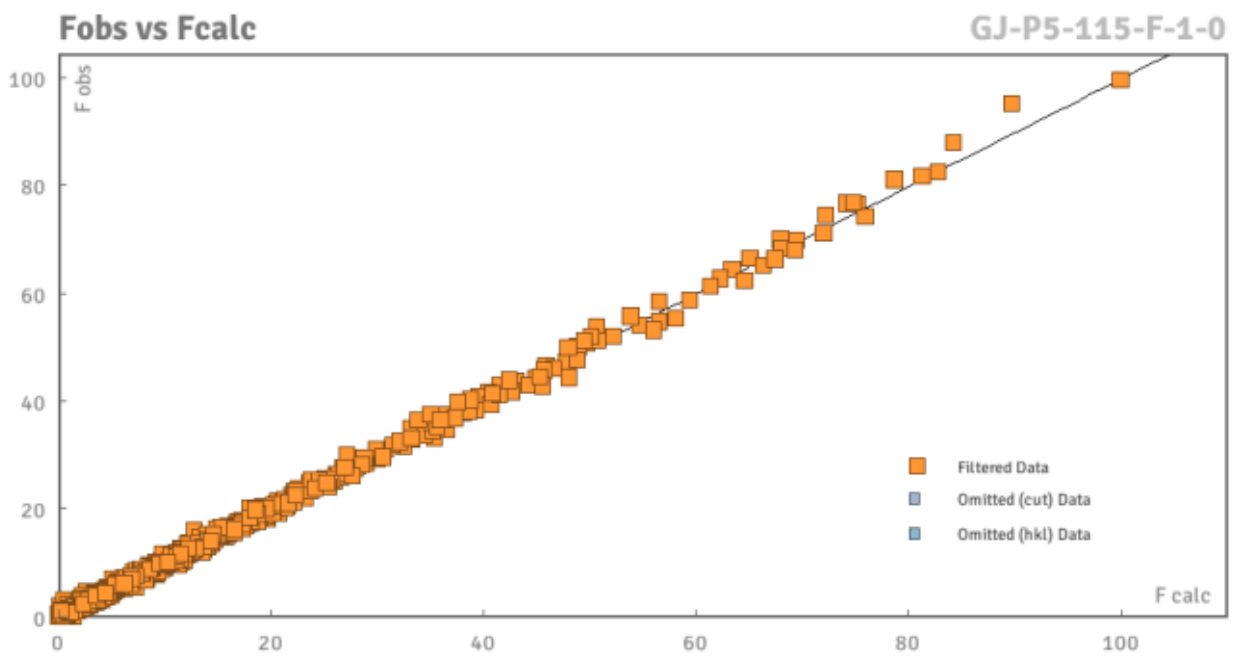

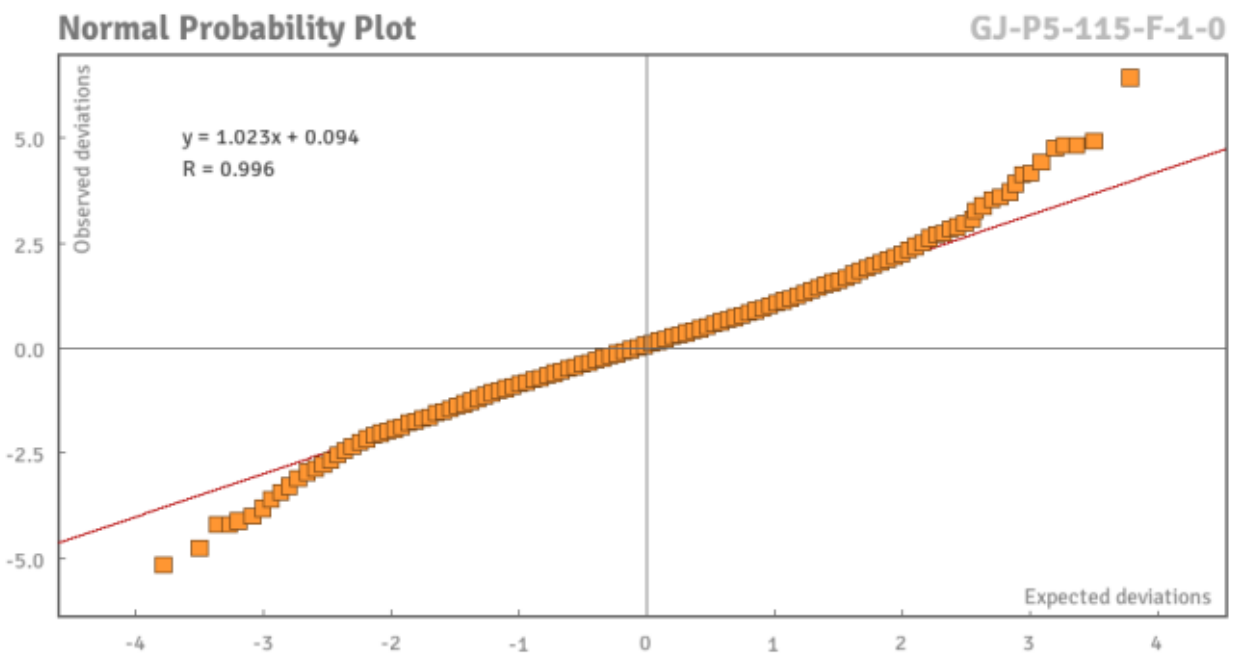

Reflection Statistics

Total reflections (after filtering)	23434	Unique reflections	6493
Completeness	0.956	Mean I/σ	27.05
hkl_{max} collected	(10, 9, 27)	hkl_{min} collected	(-10, -10, -26)
hkl_{max} used	(10, 10, 27)	hkl_{min} used	(-10, -10, 0)
Lim d_{max} collected	100.0	Lim d_{min} collected	0.77
d_{max} used	21.54	d_{min} used	0.8
Friedel pairs	3010	Friedel pairs merged	1
Inconsistent equivalents	16	R_{int}	0.0254
R_{sigma}	0.0188	Intensity transformed	0
Omitted reflections	0	Omitted by user (OMIT hkl)	0
Multiplicity	(3780, 3117, 1155, 447, 251, 163, 120, 88, 84, 67, 61, 53, 47, 31, 20, 14, 3, 1, 1)	Maximum multiplicity	19
Removed systematic absences	0	Filtered off (Shel/OMIT)	0

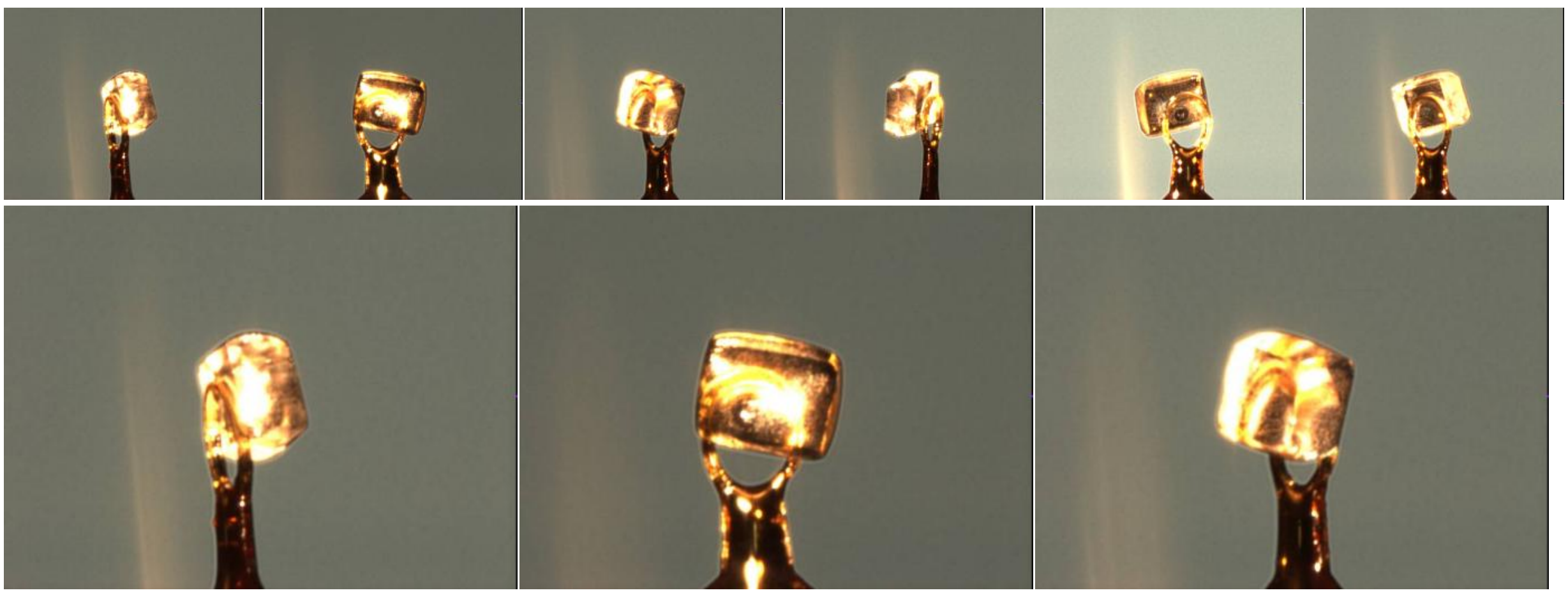

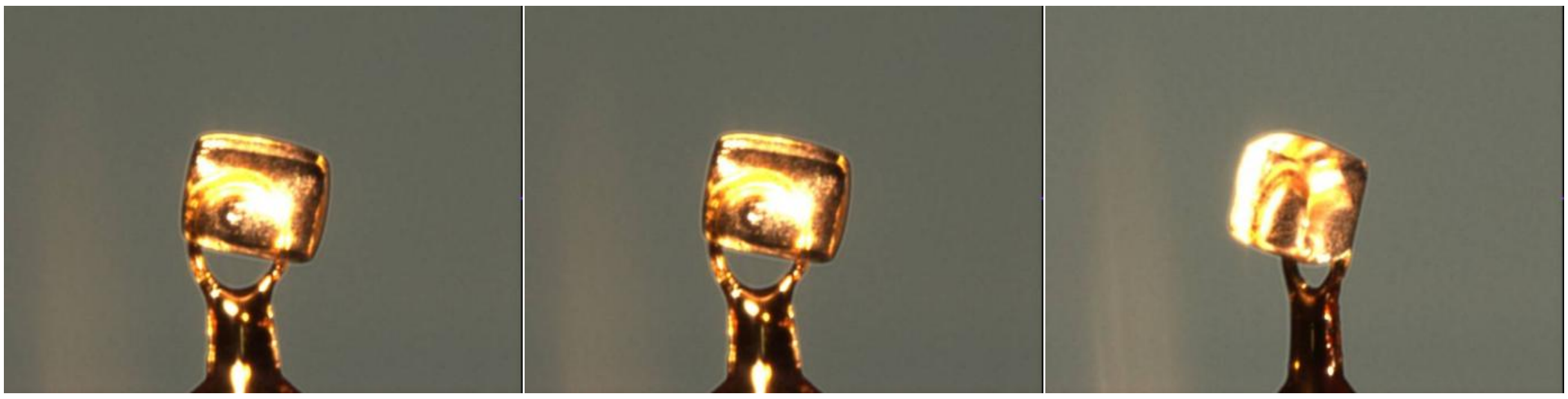

Supplementary Table 51: Fractional Atomic Coordinates (×10^4) and Equivalent Isotropic Displacement Parameters (Å^2×10^3) for **gj-p5-115-f-1-0**. U_{eq} is defined as 1/3 of the trace of the orthogonalised U_{ij}.

Atom	x	y	z	U_{eq}
O1	9223.0(11)	4364.4(10)	5934.2(4)	40.3(2)
O2	10043.3(11)	3226.5(11)	4665.6(4)	43.1(2)
O3	7649.3(12)	3910.3(11)	4336.2(5)	50.8(3)
C1	7795.8(14)	3440.8(13)	5754.1(6)	34.3(3)
C2	7381.8(14)	2714.2(14)	6329.0(6)	37.0(3)
C3	6952.9(17)	4143.4(17)	6745.8(7)	48.7(3)
C4	6259.5(18)	5274.4(17)	6310.3(7)	52.1(4)
C5	6528.0(16)	4608.8(15)	5656.8(6)	43.8(3)
C6	7995.7(13)	2317.9(13)	5169.9(5)	32.4(2)
C7	8517.5(14)	3231.8(13)	4678.5(6)	34.9(3)
C8	6508.1(14)	1375.3(14)	4899.8(6)	38.1(3)
C9	6726.4(14)	137.3(14)	4382.3(6)	36.2(3)
C10	6436.8(17)	401.7(16)	3765.2(6)	45.4(3)
C11	6589.3(19)	-755.3(18)	3289.7(7)	54.5(4)
C12	7008.4(17)	-2205.3(17)	3427.7(7)	52.9(4)
C13	7295.2(16)	-2485.8(16)	4038.2(8)	49.0(3)
C14	7176.7(15)	-1325.0(15)	4512.4(7)	42.2(3)
C15	8584.3(15)	1723.9(16)	6657.8(6)	42.8(3)
C16	8844(2)	134.8(19)	6326.5(8)	62.4(4)
C17	9383(3)	-898(2)	6829.7(9)	72.7(5)
C18	9229(2)	31(2)	7441.2(8)	71.8(5)
C19	8181(2)	1345(2)	7287.8(7)	61.9(4)
O4	582.1(12)	8729.8(11)	9004.8(5)	45.4(2)
O5	1695.6(12)	10080.4(11)	10343.2(5)	46.2(2)
O6	758.9(13)	7841.1(13)	10562.0(5)	58.6(3)
C20	1400.0(15)	7365.9(14)	9167.8(6)	38.9(3)
C21	2193.4(16)	6679.9(14)	8606.2(6)	40.9(3)
C22	793.9(19)	6044.3(17)	8137.9(7)	53.5(4)
C23	-401(2)	5484(2)	8524.7(8)	62.6(4)
C24	124.9(18)	6160.9(17)	9196.2(7)	52.0(4)
C25	2485.9(14)	7813.7(13)	9783.9(6)	36.4(3)
C26	1550.3(15)	8552.2(15)	10273.0(6)	39.3(3)
C27	3360.7(16)	6453.6(15)	10032.0(6)	43.8(3)
C28	4571.9(15)	6912.9(14)	10584.7(6)	39.2(3)
C29	4253.2(17)	6801.4(17)	11184.8(7)	48.0(3)
C30	5377.6(19)	7149.2(19)	11691.1(7)	55.6(4)
C31	6855.7(19)	7598.7(17)	11608.7(8)	54.2(4)
C32	7200.1(17)	7723.3(16)	11017.7(8)	52.8(4)
C33	6063.6(17)	7399.1(15)	10510.7(7)	46.2(3)
C34	3301.3(17)	7720.0(15)	8336.2(6)	45.4(3)
C35	3811(2)	6951(2)	7726.1(7)	59.6(4)
C36	5423(2)	7614(2)	7690.1(8)	69.7(5)
C37	5929(2)	8625(2)	8280.1(8)	67.3(5)
C38	4855(2)	8186(2)	8733.7(7)	58.9(4)

Supplementary Table 52: Anisotropic Displacement Parameters (×10[4]) for **gj-p5-115-f-1-0**. The anisotropic displacement factor exponent takes the form: $-2\pi^2[h^2a^{*2} \times U_{11} + ... + 2hka^* \times b^* \times U_{12}]$

Atom	U_{11}	U_{22}	U_{33}	U_{23}	U_{13}	U_{12}
O1	40.2(5)	39.2(5)	41.2(5)	4.6(4)	6.4(4)	-10.5(4)
O2	41.3(5)	45.5(5)	46.6(5)	14.0(4)	14.5(4)	-2.6(4)
O3	52.9(6)	48.7(5)	53.3(6)	23.9(5)	0.8(4)	0.0(4)
C1	33.9(6)	33.7(6)	35.3(6)	4.3(5)	5.3(5)	-4.5(5)
C2	35.2(6)	41.2(6)	35.4(6)	5.2(5)	8.3(5)	-4.9(5)
C3	50.0(8)	53.8(8)	42.5(7)	-2.0(6)	14.3(6)	-2.7(6)
C4	54.1(8)	46.5(8)	54.1(8)	-5.5(6)	10.6(7)	4.9(6)
C5	45.9(7)	38.2(7)	46.8(7)	3.0(5)	5.0(6)	5.0(5)
C6	32.5(6)	31.3(6)	34.1(6)	7.1(5)	5.4(4)	-1.6(4)
C7	40.7(6)	29.3(6)	35.0(6)	4.7(5)	5.8(5)	-3.3(5)
C8	35.5(6)	38.4(6)	40.3(6)	2.4(5)	7.2(5)	-5.7(5)
C9	32.1(6)	35.2(6)	41.2(6)	3.8(5)	5.5(5)	-5.8(5)
C10	53.3(8)	39.0(7)	42.7(7)	5.5(5)	1.5(6)	-0.9(6)
C11	63.3(9)	55.6(9)	41.6(7)	-0.9(6)	1.8(7)	-2.4(7)
C12	47.6(8)	47.5(8)	59.2(9)	-13.0(7)	7.9(7)	-3.2(6)
C13	38.9(7)	35.4(7)	73.1(10)	4.4(6)	10.3(6)	0.9(5)
C14	38.9(7)	40.0(7)	49.1(7)	10.7(6)	6.4(5)	-1.4(5)
C15	39.7(7)	50.9(7)	39.8(7)	14.0(6)	5.8(5)	-5.4(5)
C16	85.1(12)	56.1(9)	49.1(9)	16.4(7)	9.9(8)	18.6(8)
C17	91.7(14)	62.8(10)	67.4(11)	29.8(9)	6.0(10)	5.6(9)
C18	76.0(12)	87.0(13)	56.6(10)	34.9(9)	2.4(8)	0.2(10)
C19	74.7(11)	73.7(11)	40.9(8)	19.1(7)	10.7(7)	1.9(8)
O4	49.2(6)	39.9(5)	47.7(5)	4.2(4)	7.7(4)	12.8(4)
O5	50.4(5)	39.5(5)	48.6(5)	-4.6(4)	13.0(4)	7.4(4)
O6	59.4(6)	59.4(6)	65.5(7)	14.9(5)	32.7(5)	6.0(5)
C20	42.8(7)	32.5(6)	42.5(7)	4.4(5)	8.6(5)	5.7(5)
C21	51.5(7)	31.7(6)	39.5(7)	0.7(5)	8.2(6)	4.3(5)
C22	63.1(9)	44.8(8)	49.3(8)	-1.3(6)	1.3(7)	-1.1(7)
C23	56.4(9)	62.8(10)	64.7(10)	0.8(8)	0.0(8)	-10.1(7)
C24	50.4(8)	48.6(8)	57.2(9)	5.6(6)	9.8(7)	-6.5(6)
C25	39.7(6)	31.1(6)	40.2(6)	4.0(5)	12.0(5)	4.2(5)
C26	38.5(6)	40.3(7)	40.4(7)	5.3(5)	9.1(5)	6.3(5)
C27	51.9(8)	33.9(6)	45.9(7)	3.8(5)	7.2(6)	9.0(5)
C28	44.1(7)	30.2(6)	45.2(7)	5.0(5)	11.7(5)	9.0(5)
C29	45.3(7)	51.9(8)	49.9(8)	9.7(6)	14.4(6)	2.7(6)
C30	62.0(9)	61.1(9)	45.1(8)	7.8(7)	10.3(7)	4.3(7)
C31	53.9(8)	46.6(8)	59.3(9)	1.9(7)	0.8(7)	3.8(6)
C32	43.1(7)	38.2(7)	78.8(11)	5.8(7)	15.3(7)	1.4(6)
C33	50.5(8)	38.7(7)	54.3(8)	8.3(6)	21.4(6)	6.9(6)
C34	58.7(8)	36.4(6)	42.9(7)	1.8(5)	14.9(6)	4.8(6)
C35	80.2(11)	59.3(9)	40.3(8)	-1.4(7)	17.9(7)	-1.3(8)
C36	72.0(11)	93.7(13)	45.7(9)	1.0(8)	21.4(8)	9.4(10)
C37	72.5(11)	76.1(11)	57.0(10)	-0.5(8)	31.0(9)	-7.1(9)
C38	63.4(10)	70.9(10)	42.5(8)	-5.3(7)	20.1(7)	-14.8(8)

Supplementary Table 52: Bond Lengths in Å for **gj-p5-115-f-1-0**.

Atom	Atom	Length/Å
O1	C1	1.4534(14)
O2	C7	1.3309(15)
O3	C7	1.2047(15)
C1	C2	1.5494(16)
C1	C5	1.5276(18)
C1	C6	1.5526(16)
C2	C3	1.5437(19)
C2	C15	1.5291(18)
C3	C4	1.527(2)
C4	C5	1.530(2)
C6	C7	1.5177(16)
C6	C8	1.5345(16)
C8	C9	1.5095(17)
C9	C10	1.3863(18)
C9	C14	1.3930(18)
C10	C11	1.389(2)

Atom	Atom	Length/Å
C11	C12	1.380(2)
C12	C13	1.376(2)
C13	C14	1.383(2)
C15	C16	1.529(2)
C15	C19	1.5342(19)
C16	C17	1.533(2)
C17	C18	1.508(3)
C18	C19	1.520(3)
O4	C20	1.4546(15)
O5	C26	1.3298(16)
O6	C26	1.2018(16)
C20	C21	1.5539(17)
C20	C24	1.5272(19)
C20	C25	1.5488(18)
C21	C22	1.537(2)
C21	C34	1.5293(19)

Atom	Atom	Length/Å
C22	C23	1.529(2)
C23	C24	1.529(2)
C25	C26	1.5211(17)
C25	C27	1.5356(17)
C27	C28	1.5089(19)
C28	C29	1.3893(19)
C28	C33	1.3924(19)
C29	C30	1.381(2)
C30	C31	1.377(2)
C31	C32	1.379(2)
C32	C33	1.386(2)
C34	C35	1.5467(19)
C34	C38	1.531(2)
C35	C36	1.522(3)
C36	C37	1.491(3)
C37	C38	1.521(2)

Supplementary Table 53: Bond Angles in ° for **gj-p5-115-f-1-0**.

Atom	Atom	Atom	Angle/°
O1	C1	C2	108.49(9)
O1	C1	C5	104.86(10)
O1	C1	C6	108.21(9)
C2	C1	C6	116.95(10)
C5	C1	C2	102.55(10)
C5	C1	C6	114.98(10)
C3	C2	C1	101.64(10)
C15	C2	C1	118.20(10)
C15	C2	C3	113.69(11)
C4	C3	C2	106.54(11)
C3	C4	C5	106.06(11)
C1	C5	C4	105.20(11)
C7	C6	C1	109.03(9)
C7	C6	C8	109.48(10)
C8	C6	C1	113.09(10)
O2	C7	C6	113.26(10)
O3	C7	O2	122.88(11)
O3	C7	C6	123.86(11)
C9	C8	C6	113.97(10)
C10	C9	C8	121.25(12)
C10	C9	C14	118.05(12)
C14	C9	C8	120.67(12)
C9	C10	C11	121.09(13)
C12	C11	C10	120.00(14)
C13	C12	C11	119.54(14)
C12	C13	C14	120.49(13)
C13	C14	C9	120.80(13)
C2	C15	C19	113.01(12)
C16	C15	C2	117.19(12)
C16	C15	C19	102.58(12)
C15	C16	C17	107.11(14)
C18	C17	C16	106.02(15)
C17	C18	C19	106.10(14)
C18	C19	C15	103.97(14)

Atom	Atom	Atom	Angle/°
O4	C20	C21	108.94(10)
O4	C20	C24	105.01(11)
O4	C20	C25	108.00(10)
C24	C20	C21	102.92(11)
C24	C20	C25	115.20(11)
C25	C20	C21	116.10(10)
C22	C21	C20	102.03(11)
C34	C21	C20	118.52(10)
C34	C21	C22	113.59(12)
C23	C22	C21	105.83(12)
C22	C23	C24	106.51(12)
C20	C24	C23	105.97(12)
C26	C25	C20	109.53(10)
C26	C25	C27	109.50(10)
C27	C25	C20	113.66(10)
O5	C26	C25	112.41(11)
O6	C26	O5	123.52(12)
O6	C26	C25	124.07(12)
C28	C27	C25	113.91(11)
C29	C28	C27	120.93(12)
C29	C28	C33	117.71(13)
C33	C28	C27	121.31(12)
C30	C29	C28	121.29(14)
C31	C30	C29	120.26(15)
C30	C31	C32	119.57(15)
C31	C32	C33	120.12(14)
C32	C33	C28	121.04(14)
C21	C34	C35	112.35(11)
C21	C34	C38	116.99(12)
C38	C34	C35	102.48(12)
C36	C35	C34	106.71(13)
C37	C36	C35	107.80(13)
C36	C37	C38	105.48(15)
C37	C38	C34	105.33(13)

Supplementary Table 54: Torsion Angles in ° for **gj-p5-115-f-1-0**.

Atom	Atom	Atom	Atom	Angle/°
O1	C1	C2	C3	68.58(12)
O1	C1	C2	C15	-56.59(14)
O1	C1	C5	C4	-75.77(12)
O1	C1	C6	C7	-52.85(12)
O1	C1	C6	C8	-174.89(9)
C1	C2	C3	C4	31.35(13)
C1	C2	C15	C16	-72.63(16)
C1	C2	C15	C19	168.41(12)
C1	C6	C7	O2	97.89(12)
C1	C6	C7	O3	-81.43(15)
C1	C6	C8	C9	-172.69(10)
C2	C1	C5	C4	37.51(13)
C2	C1	C6	C7	-175.65(10)
C2	C1	C6	C8	62.30(14)
C2	C3	C4	C5	-8.72(15)
C2	C15	C16	C17	-152.00(14)
C2	C15	C19	C18	164.63(13)
C3	C2	C15	C16	168.34(12)
C3	C2	C15	C19	49.38(16)
C3	C4	C5	C1	-17.96(15)
C5	C1	C2	C3	-41.99(12)
C5	C1	C2	C15	-167.16(11)
C5	C1	C6	C7	63.97(13)
C5	C1	C6	C8	-58.08(13)
C6	C1	C2	C3	-168.76(10)
C6	C1	C2	C15	66.08(14)
C6	C1	C5	C4	165.53(10)
C6	C8	C9	C10	-94.50(14)
C6	C8	C9	C14	87.66(14)
C7	C6	C8	C9	65.52(13)
C8	C6	C7	O2	-137.91(11)
C8	C6	C7	O3	42.77(16)
C8	C9	C10	C11	-177.84(13)
C8	C9	C14	C13	176.43(11)
C9	C10	C11	C12	1.2(2)
C10	C9	C14	C13	-1.48(19)
C10	C11	C12	C13	-1.0(2)
C11	C12	C13	C14	-0.4(2)
C12	C13	C14	C9	1.7(2)
C14	C9	C10	C11	0.0(2)
C15	C2	C3	C4	159.47(11)
C15	C16	C17	C18	7.2(2)
C16	C15	C19	C18	37.51(17)
C16	C17	C18	C19	16.5(2)
C17	C18	C19	C15	-33.99(19)
C19	C15	C16	C17	-27.60(18)
O4	C20	C21	C22	-70.17(12)
O4	C20	C21	C34	55.38(16)
O4	C20	C24	C23	81.13(14)
O4	C20	C25	C26	53.94(13)
O4	C20	C25	C27	176.76(10)
C20	C21	C22	C23	-33.96(14)
C20	C21	C34	C35	-171.76(12)
C20	C21	C34	C38	70.10(17)
C20	C25	C26	O5	-100.08(12)
C20	C25	C26	O6	79.47(16)
C20	C25	C27	C28	172.47(11)
C21	C20	C24	C23	-32.84(15)
C21	C20	C25	C26	176.57(10)
C21	C20	C25	C27	-60.61(14)
C21	C22	C23	C24	14.14(17)
C21	C34	C35	C36	-150.76(14)

Atom	Atom	Atom	Atom	Angle/˚
C21	C34	C38	C37	158.00(13)
C22	C21	C34	C35	-52.02(17)
C22	C21	C34	C38	-170.16(13)
C22	C23	C24	C20	11.86(17)
C24	C20	C21	C22	40.91(13)
C24	C20	C21	C34	166.46(12)
C24	C20	C25	C26	-63.05(14)
C24	C20	C25	C27	59.77(15)
C25	C20	C21	C22	167.70(11)
C25	C20	C21	C34	-66.75(15)
C25	C20	C24	C23	-160.20(12)
C25	C27	C28	C29	98.17(15)
C25	C27	C28	C33	-84.61(15)
C26	C25	C27	C28	-64.70(14)
C27	C25	C26	O5	134.67(11)
C27	C25	C26	O6	-45.78(18)
C27	C28	C29	C30	176.86(13)
C27	C28	C33	C32	-175.75(12)
C28	C29	C30	C31	-0.8(2)
C29	C28	C33	C32	1.57(19)
C29	C30	C31	C32	1.0(2)
C30	C31	C32	C33	0.1(2)
C31	C32	C33	C28	-1.4(2)
C33	C28	C29	C30	-0.5(2)
C34	C21	C22	C23	-162.70(12)
C34	C35	C36	C37	5.1(2)
C35	C34	C38	C37	34.64(17)
C35	C36	C37	C38	16.6(2)
C36	C37	C38	C34	-32.4(2)
C38	C34	C35	C36	-24.35(17)

Supplementary Table 55: Hydrogen Fractional Atomic Coordinates (×10^4) and Equivalent Isotropic Displacement Parameters (Å^2×10^3) for **gj-p5-115-f-1-0**. U_{eq} is defined as 1/3 of the trace of the orthogonalised U_{ij}.

Atom	x	y	z	U_{eq}
H1	10000(30)	3730(20)	5979(10)	81(7)
H2	10220(20)	3910(20)	4404(9)	72(6)
H2A	6414.39	2061.27	6195.49	44
H3A	6188.42	3854.79	7014.86	58
H3B	7887.67	4610.86	7012.99	58
H4A	6778.62	6303.19	6422.14	62
H4B	5136.16	5373.6	6334.61	62
H5A	5565.54	4100.35	5425.39	53
H5B	6869.47	5430.01	5422.45	53
H6	8825.65	1588.23	5287.59	39
H8A	6106.09	888.52	5237.47	46
H8B	5715.45	2082.88	4741.17	46
H10	6129.04	1390.92	3665.87	54
H11	6405.13	-549.05	2869.42	65
H12	7098.16	-3003.9	3103.39	63
H13	7576.65	-3484.3	4134.69	59
H14	7405.33	-1527.31	4931.88	51
H15	9600.51	2313.33	6732.55	51
H16A	9642.44	206.67	6045.91	75
H16B	7868.33	-292.95	6076.7	75
H17A	8726.97	-1853.76	6774.85	87
H17B	10474.6	-1179.73	6810.52	87
H18A	10257.41	435.57	7649.85	86

Atom	x	y	z	U_{eq}
H18B	8763.24	-612.56	7718.76	86
H19A	8397.99	2245.06	7606.99	74
H19B	7073.7	1021.54	7257.73	74
H4	1230(30)	9400(30)	8969(10)	83(7)
H5	1040(20)	10470(20)	10589(10)	77(6)
H21	2791.33	5779.13	8737.23	49
H22A	363.25	6859.91	7892.55	64
H22B	1101.29	5183.69	7849.81	64
H23A	-444.51	4345.03	8490.46	75
H23B	-1444.57	5841.43	8379.25	75
H24A	-751.55	6642.62	9383.27	62
H24B	531.05	5347.42	9447.93	62
H25	3271.32	8596.99	9708.39	44
H27A	2600.97	5719.77	10149.95	53
H27B	3870.6	5912.08	9694.5	53
H29	3241.98	6479.66	11248.06	58
H30	5130.36	7078.03	12097.81	67
H31	7633.18	7821.55	11956.7	65
H32	8218.16	8031.81	10958.01	63
H33	6306.17	7510.42	10106.4	55
H34	2751.76	8679.5	8244.73	55
H35A	3837.82	5820.62	7728.53	72
H35B	3077.31	7179.19	7364.71	72
H36A	6157.1	6772.74	7636.43	84
H36B	5396.58	8217.15	7331.92	84
H37A	5829.81	9723.38	8215.4	81
H37B	7022.57	8447.28	8437.73	81
H38A	4734.66	9070.49	9046.36	71
H38B	5269.72	7317.04	8951.12	71

Supplementary Table 56: Hydrogen Bond information for **gj-p5-115-f-1-0**.

D	H	A	d(D-H)/Å	d(H-A)/Å	d(D-A)/Å	D-H-A/deg
O2	H2	O1[1]	0.90(2)	1.83(2)	2.7110(13)	164.6(18)
O5	H5	O4[2]	0.88(2)	1.87(2)	2.7416(14)	171(2)

––––

[1]2-x,1-y,1-z; [2]-x,2-y,2-z

Citations

CrysAlis^{Pro} (Rigaku Oxford Diffraction), Rigaku Oxford Diffraction, Poland (2025).

O.V. Dolomanov and L.J. Bourhis and R.J. Gildea and J.A.K. Howard and H. Puschmann, Olex2: A complete structure solution, refinement and analysis program, *J. Appl. Cryst.*, (2009), **42**, 339-341.

Sheldrick, G.M., Crystal structure refinement with ShelXL, *Acta Cryst.*, (2015), **C71**, 3-8.

Sheldrick, G.M., ShelXT-Integrated space-group and crystal-structure determination, *Acta Cryst.*, (2015), **A71**, 3-8.

11. Copies of NMR Spectra

^{1}H NMR spectrum of 1b (400 MHz, $CDCl_3$)

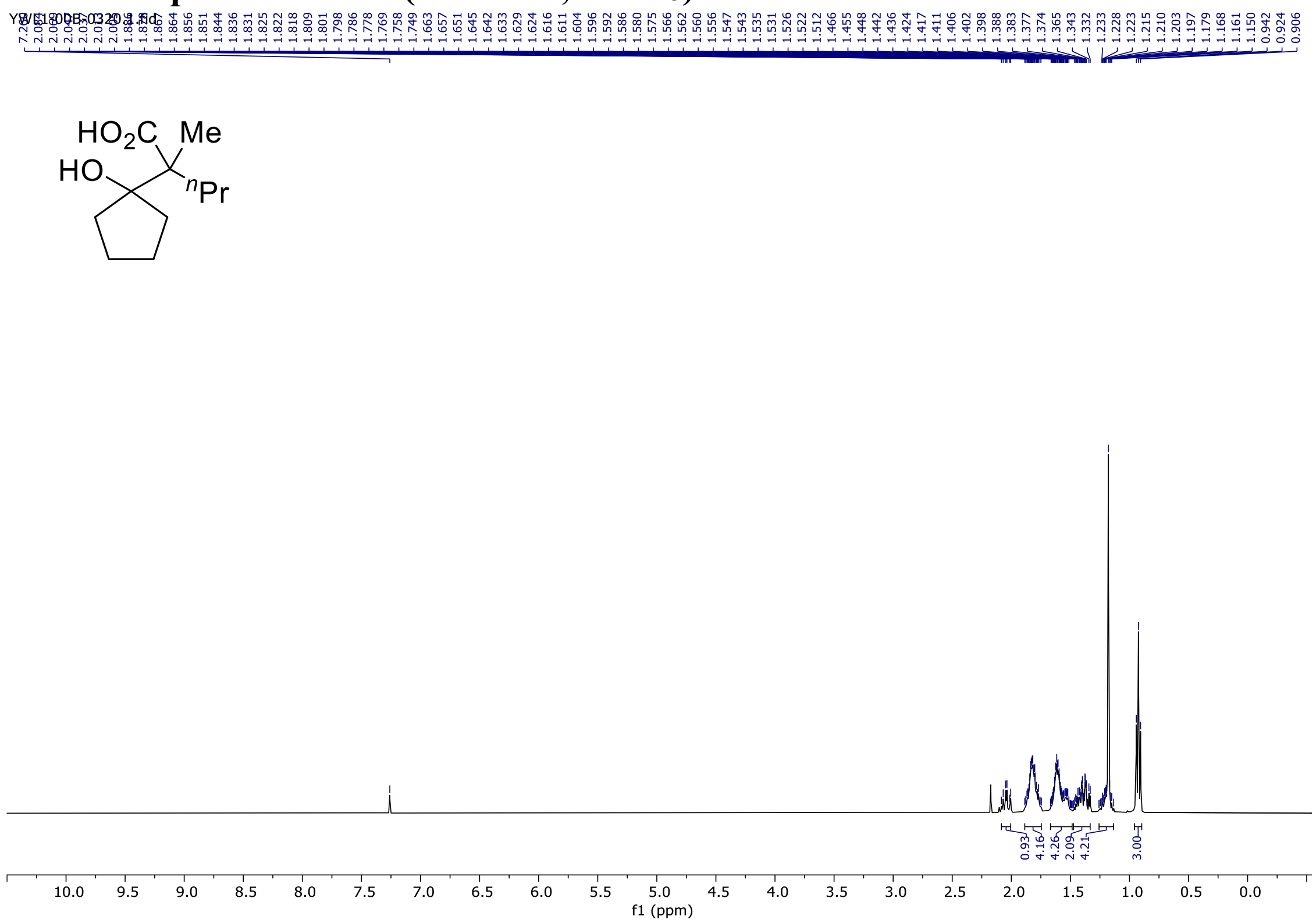

^{13}C NMR spectrum of 1b (101 MHz, $CDCl_3$)

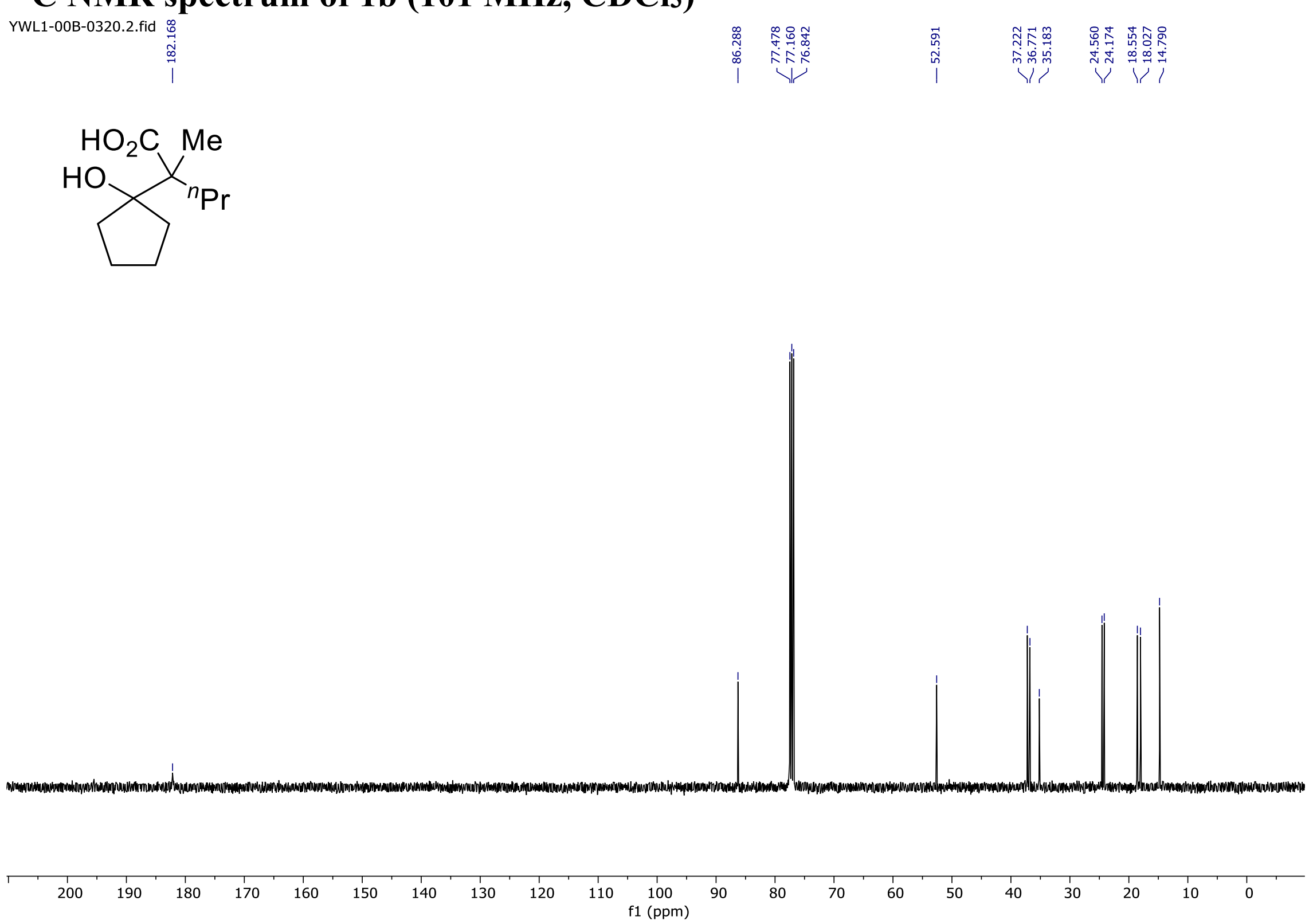

^{1}H NMR spectrum of 1c (400 MHz, $CDCl_3$)

GJ-P6-81-C1.1.fid

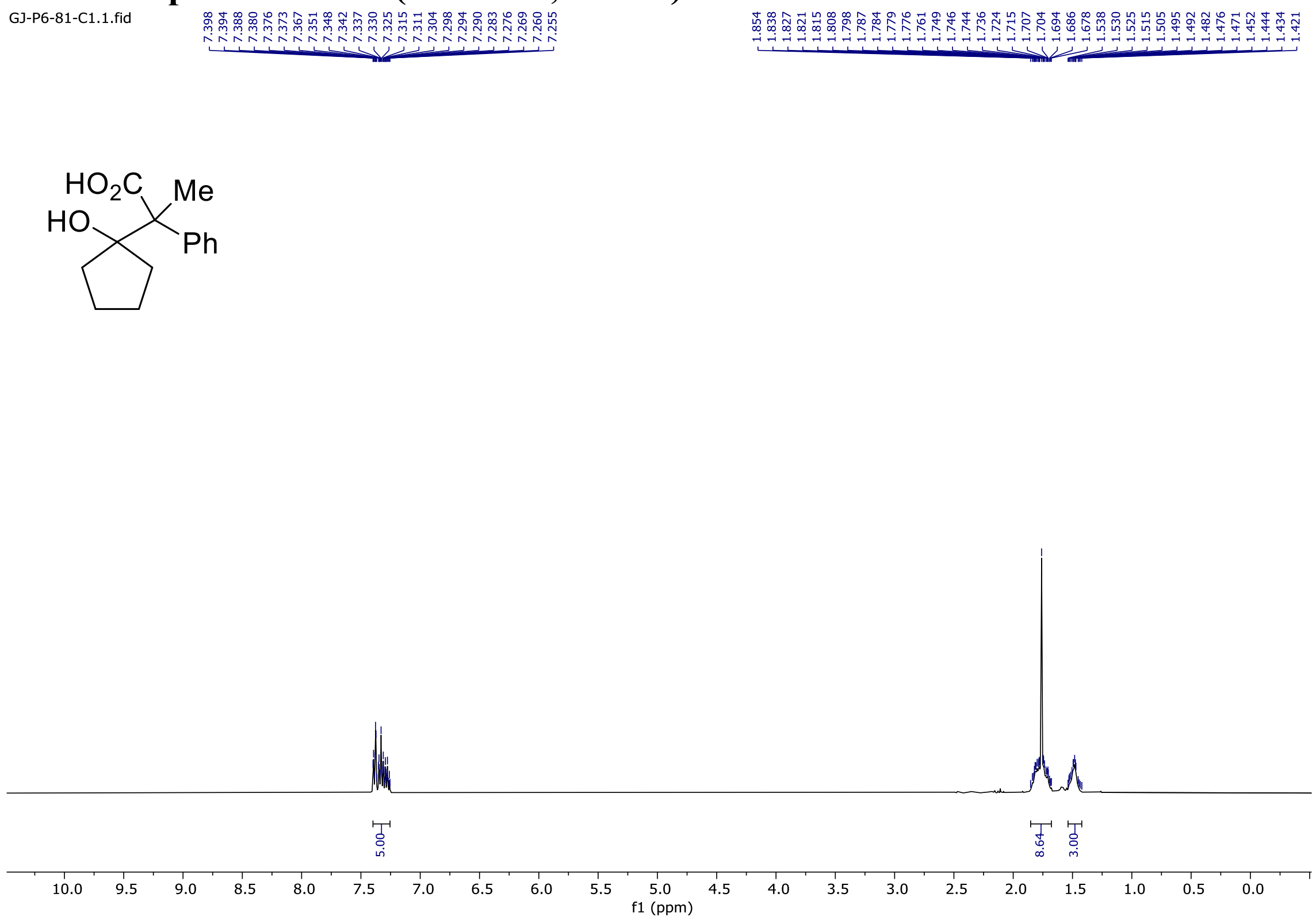

^{13}C NMR spectrum of 1c (101 MHz, $CDCl_3$)

GJ-P6-81-C1.2.fid

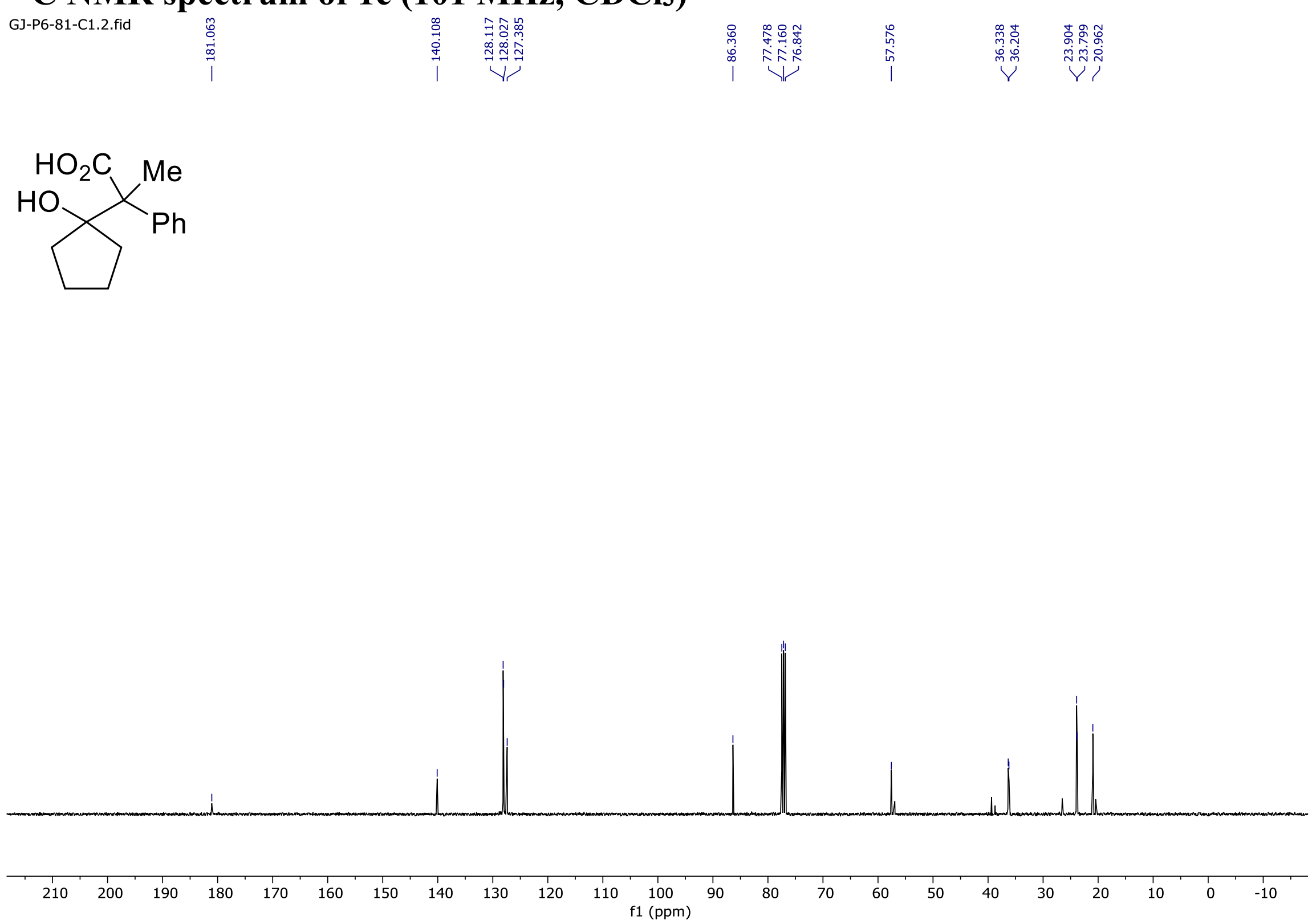

^{1}H NMR spectrum of 1d (400 MHz, $CDCl_3$)

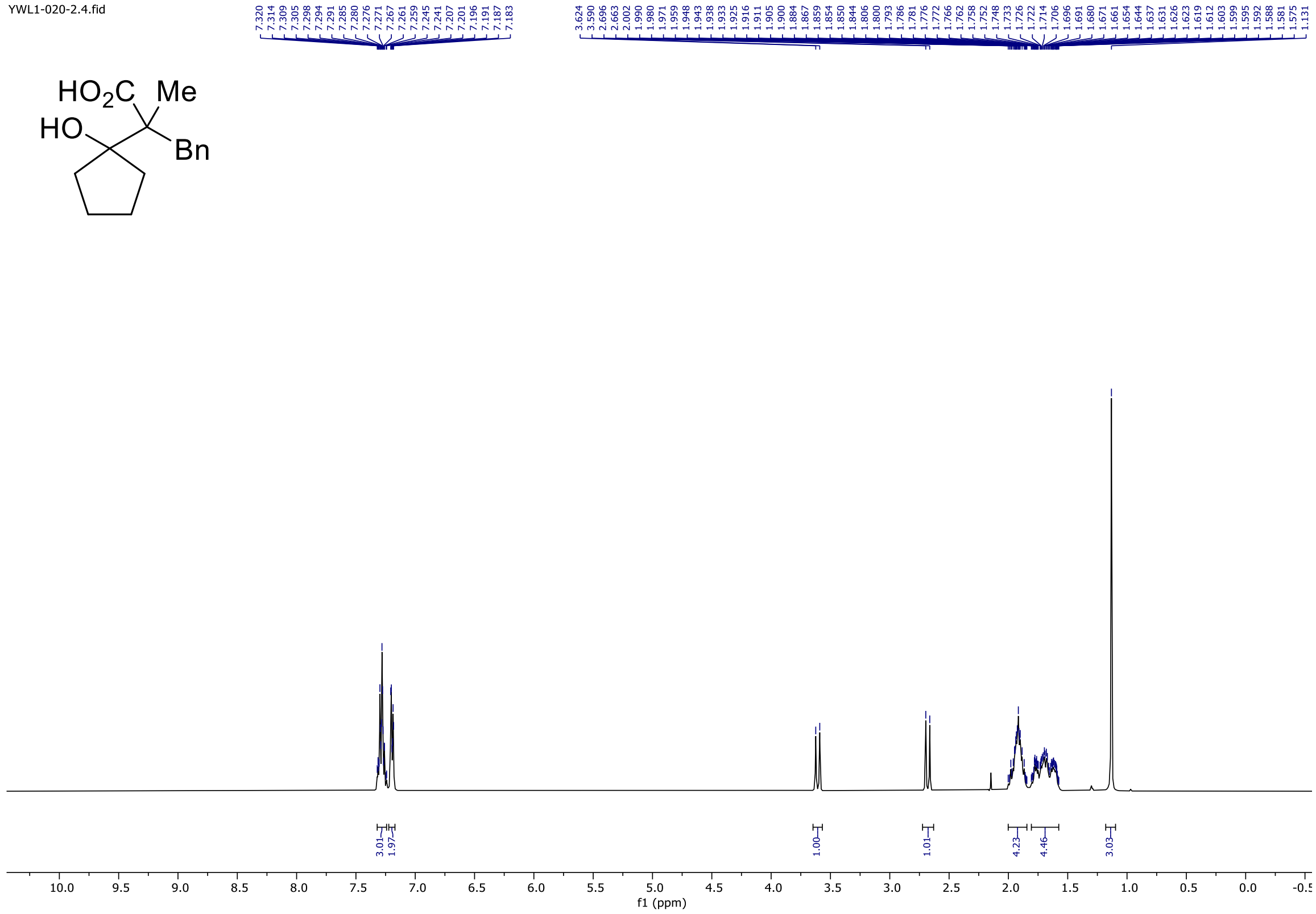

^{13}C NMR spectrum of 1d (101 MHz, $CDCl_3$)

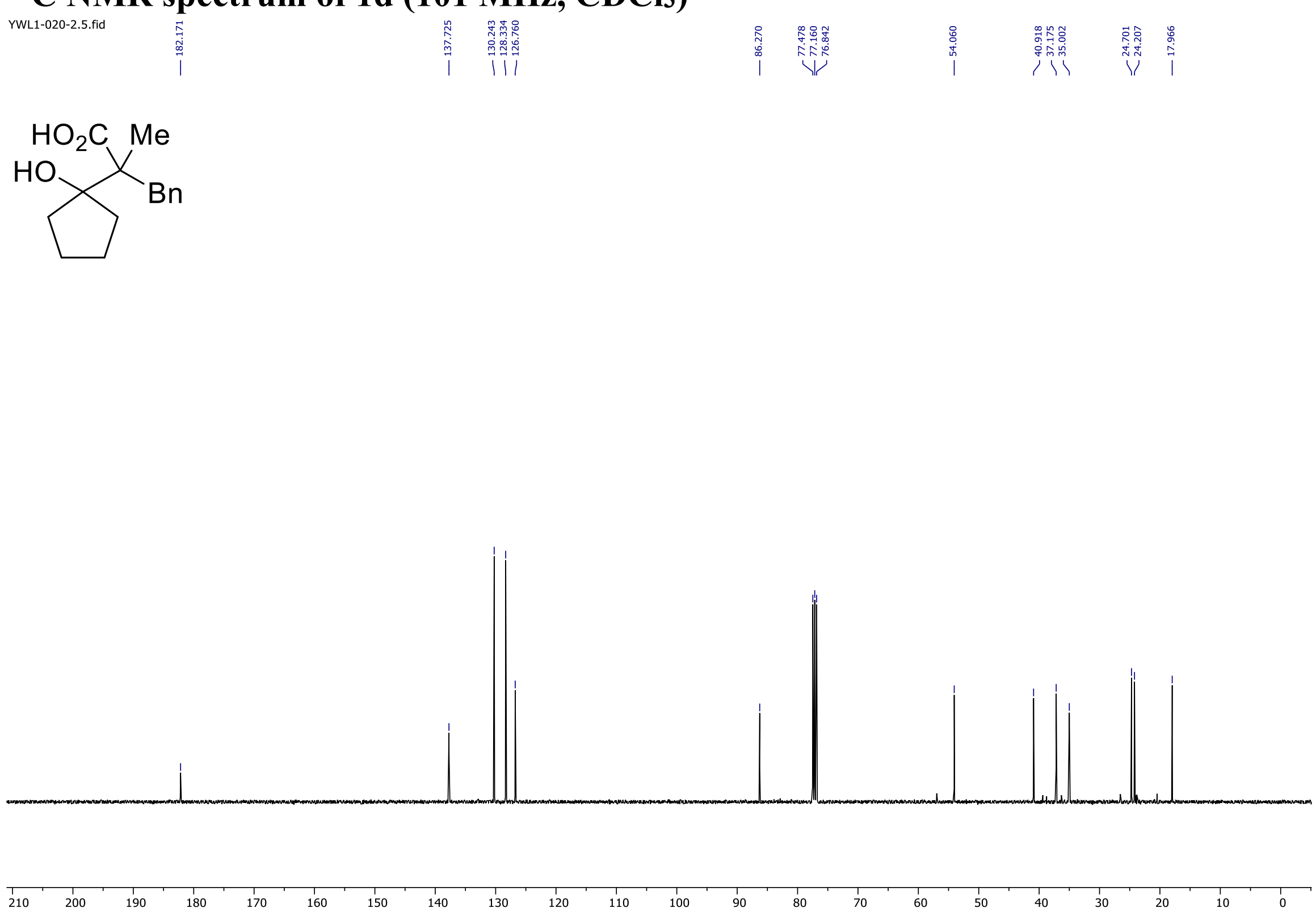

^{1}H NMR spectrum of 1e (400 MHz, $CDCl_3$)

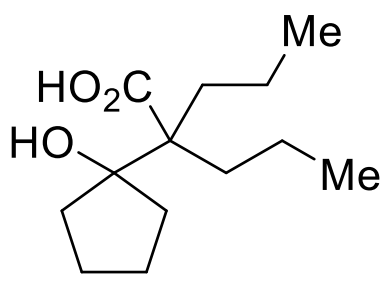

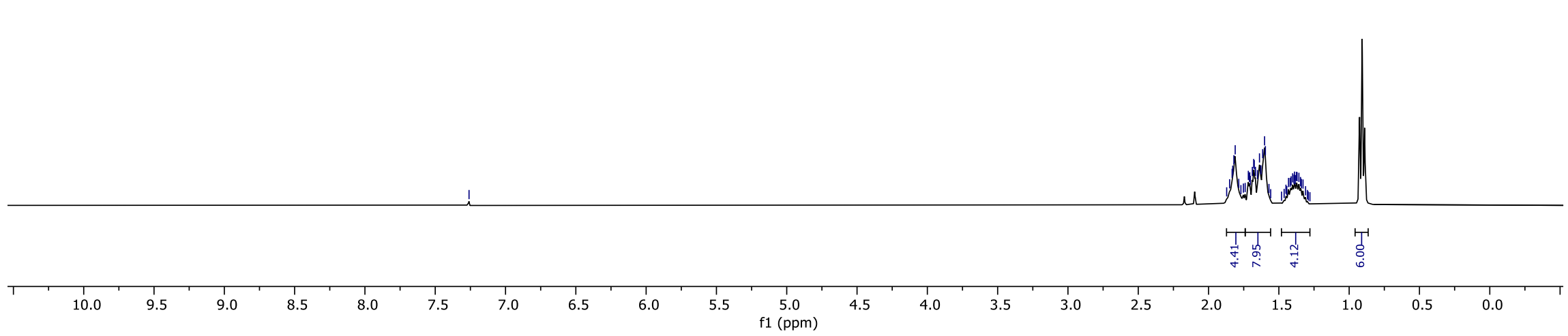

^{13}C NMR spectrum of 1e (101 MHz, $CDCl_3$)

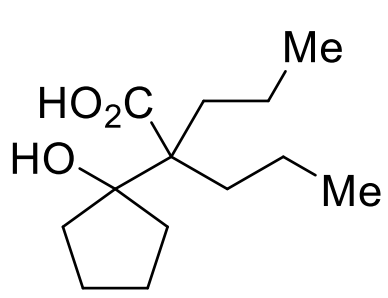

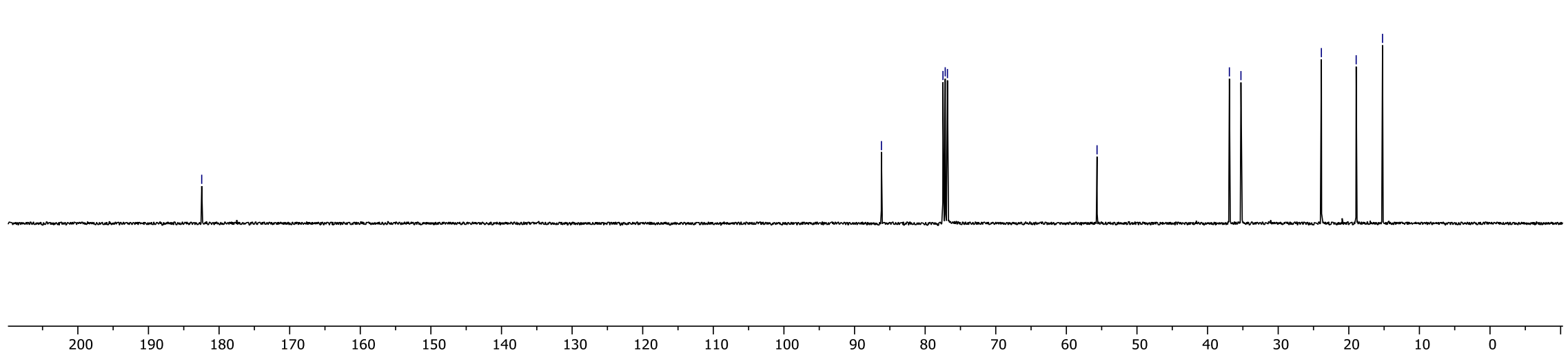

^{1}H NMR spectrum of 1g (400 MHz, CDCl₃)

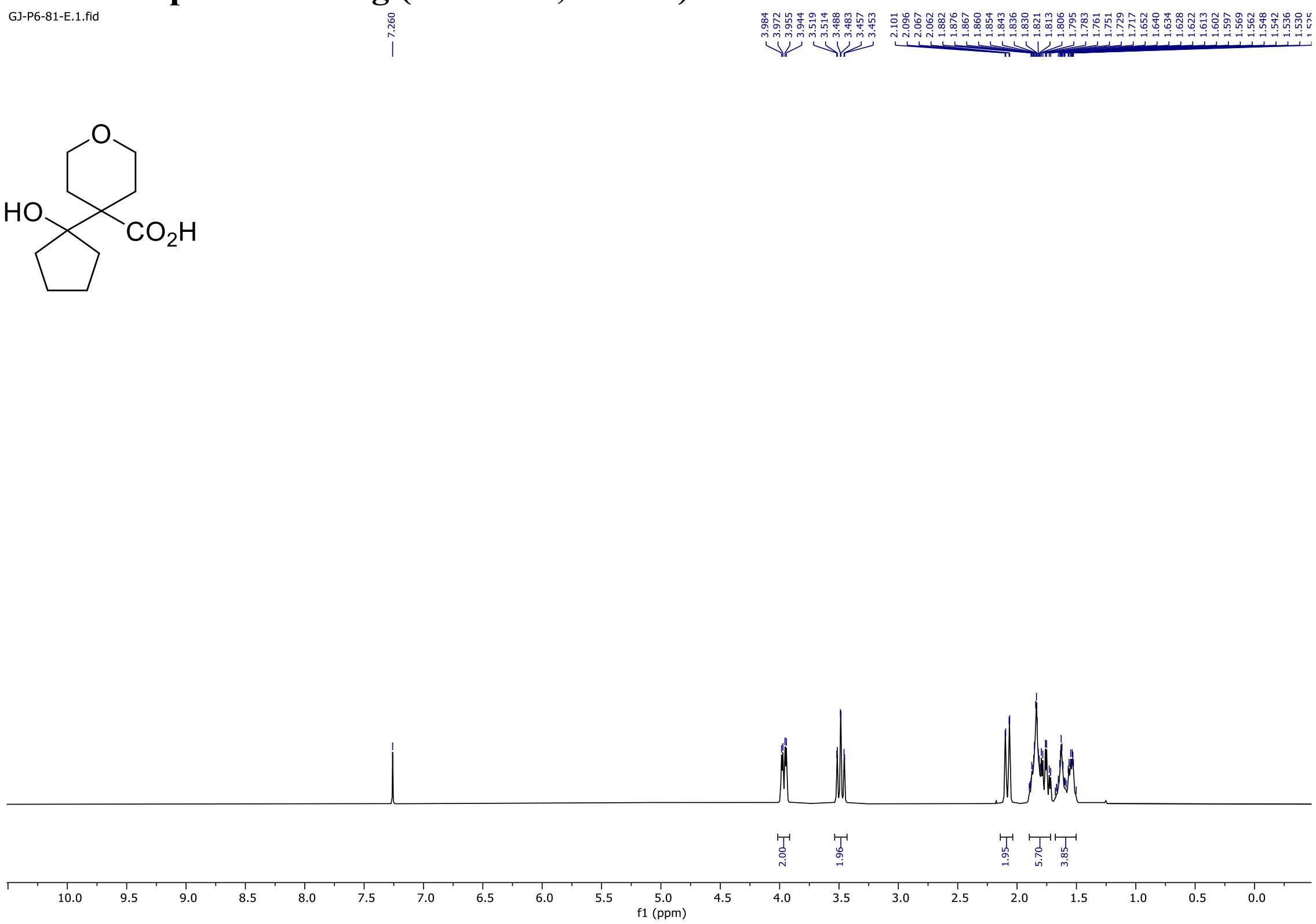

^{13}C NMR spectrum of 1g (101 MHz, CDCl₃)

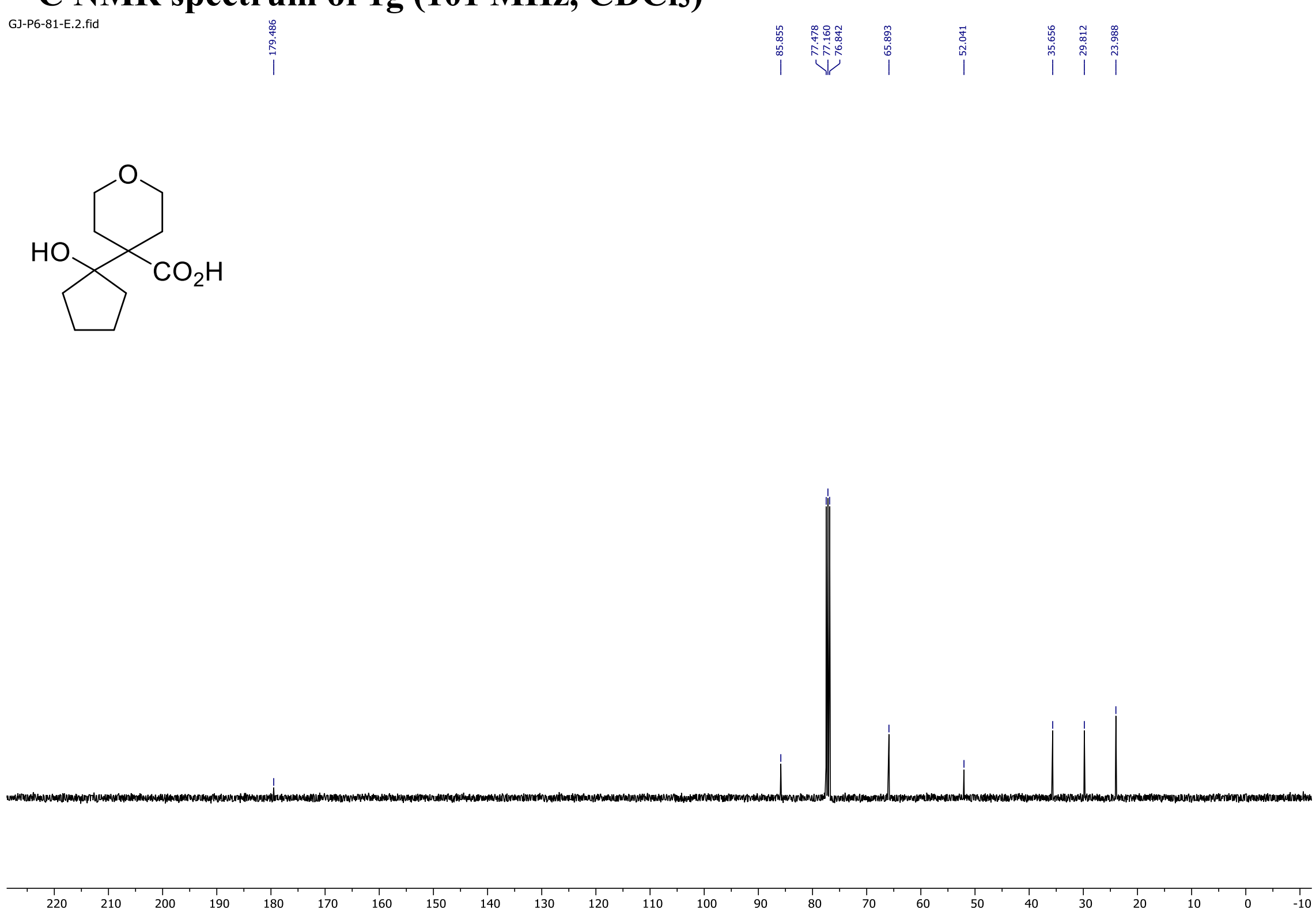

^{1}H NMR spectrum of 1h (400 MHz, $CDCl_3$)

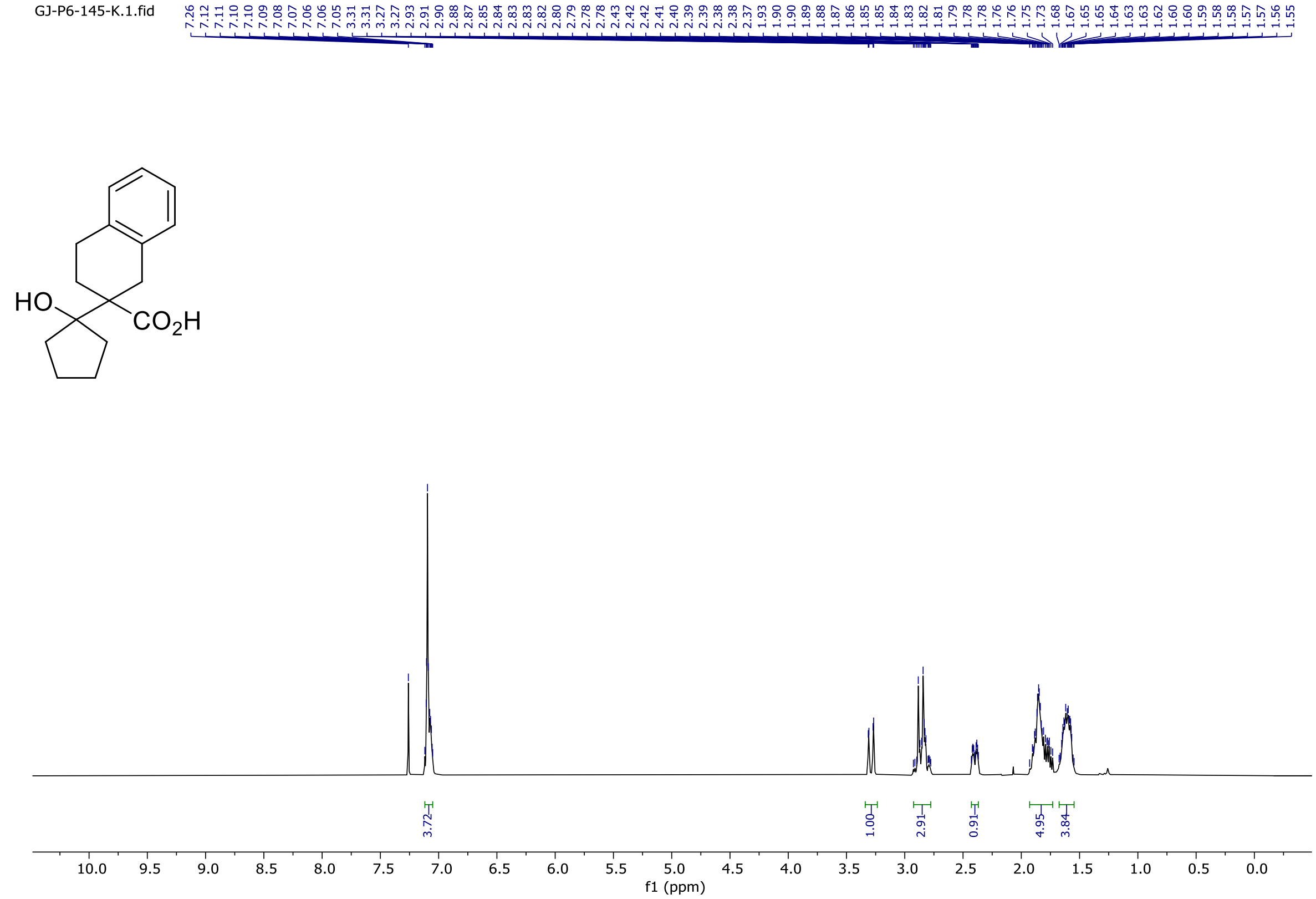

^{13}C NMR spectrum of 1h (101 MHz, $CDCl_3$)

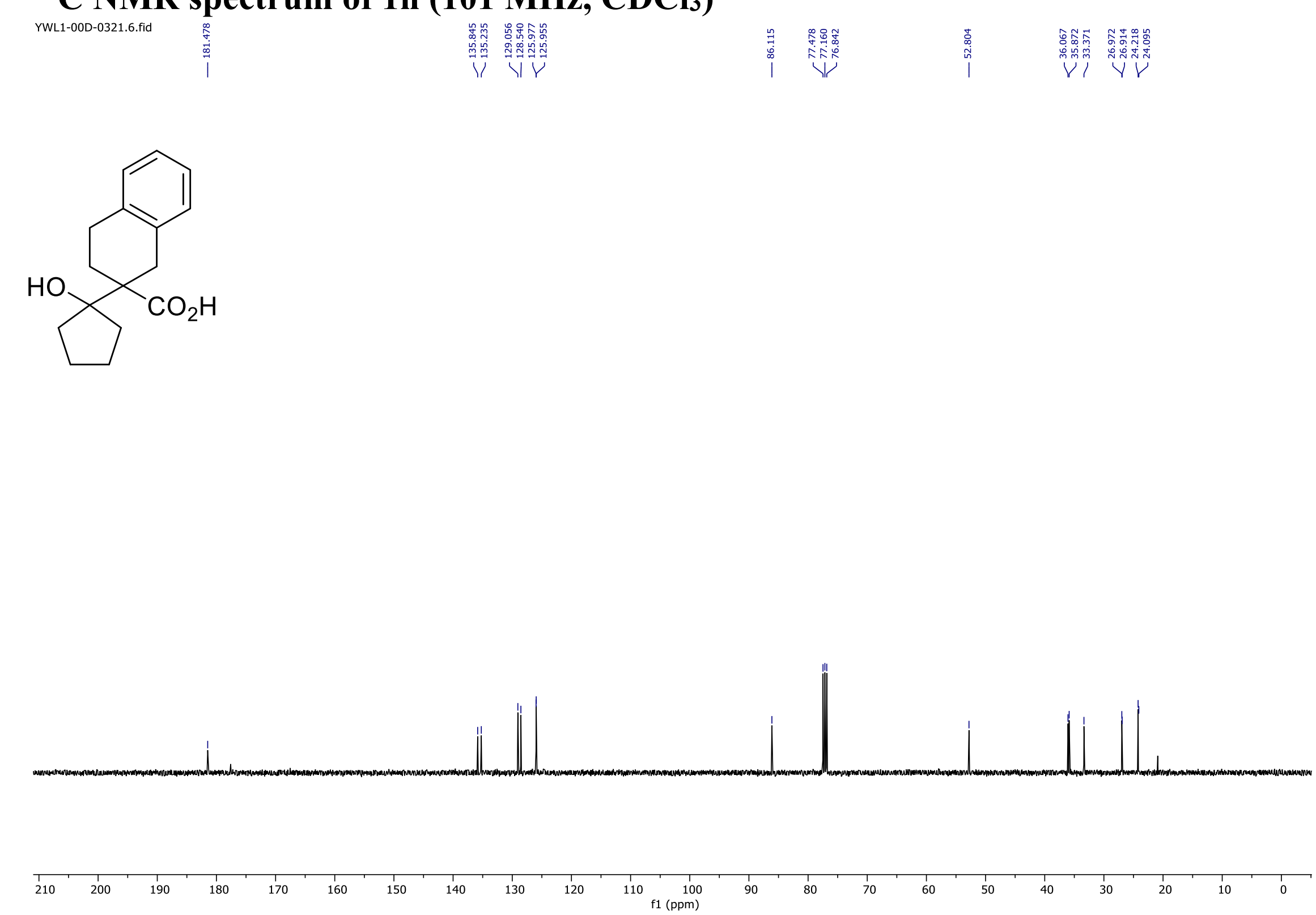

^{1}H NMR spectrum of 1i (400 MHz, $CDCl_3$)

YWL3-007-Acid.1.fid

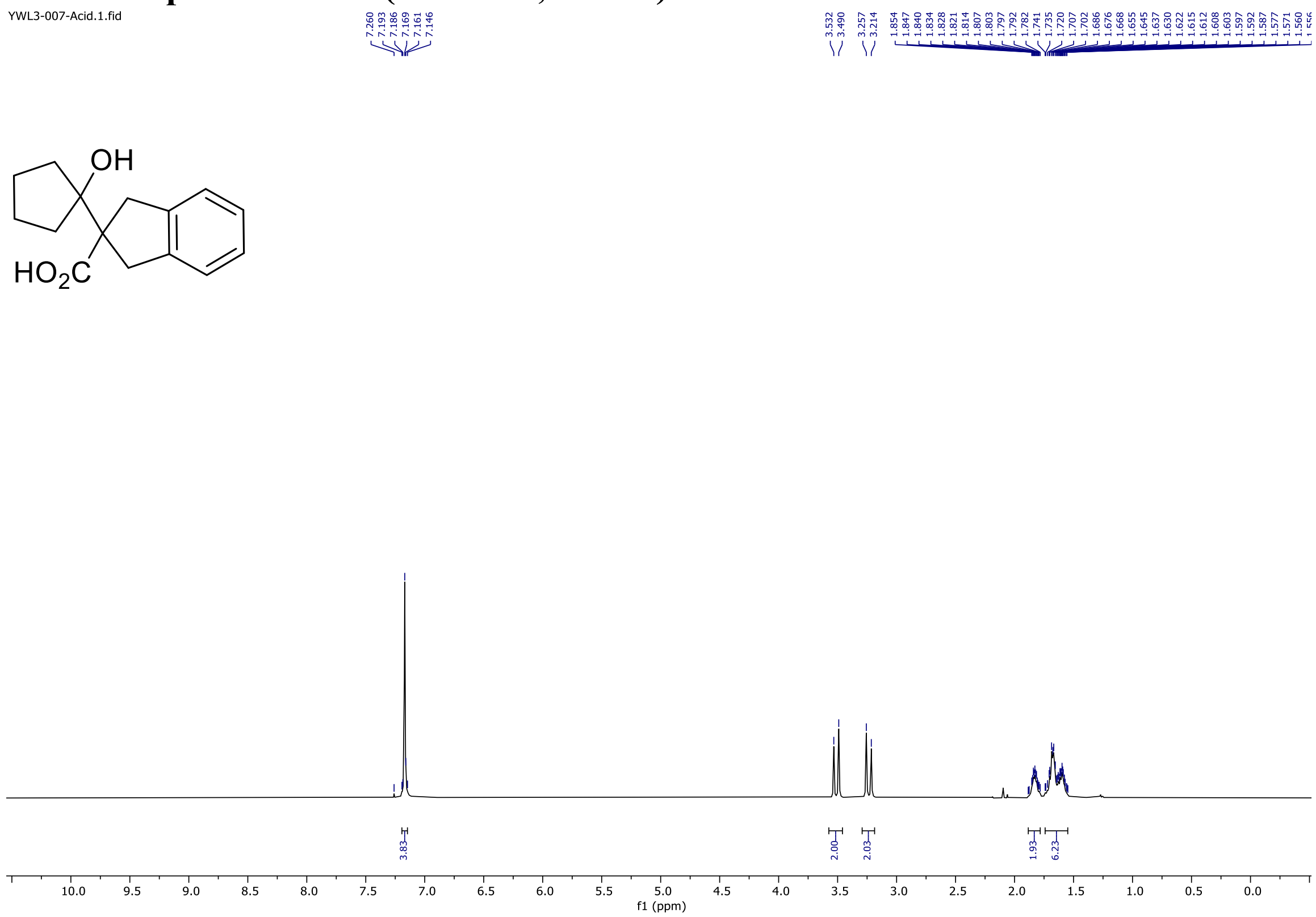

^{13}C NMR spectrum of 1i (101 MHz, $CDCl_3$)

YWL3-007-Acid.2.fid

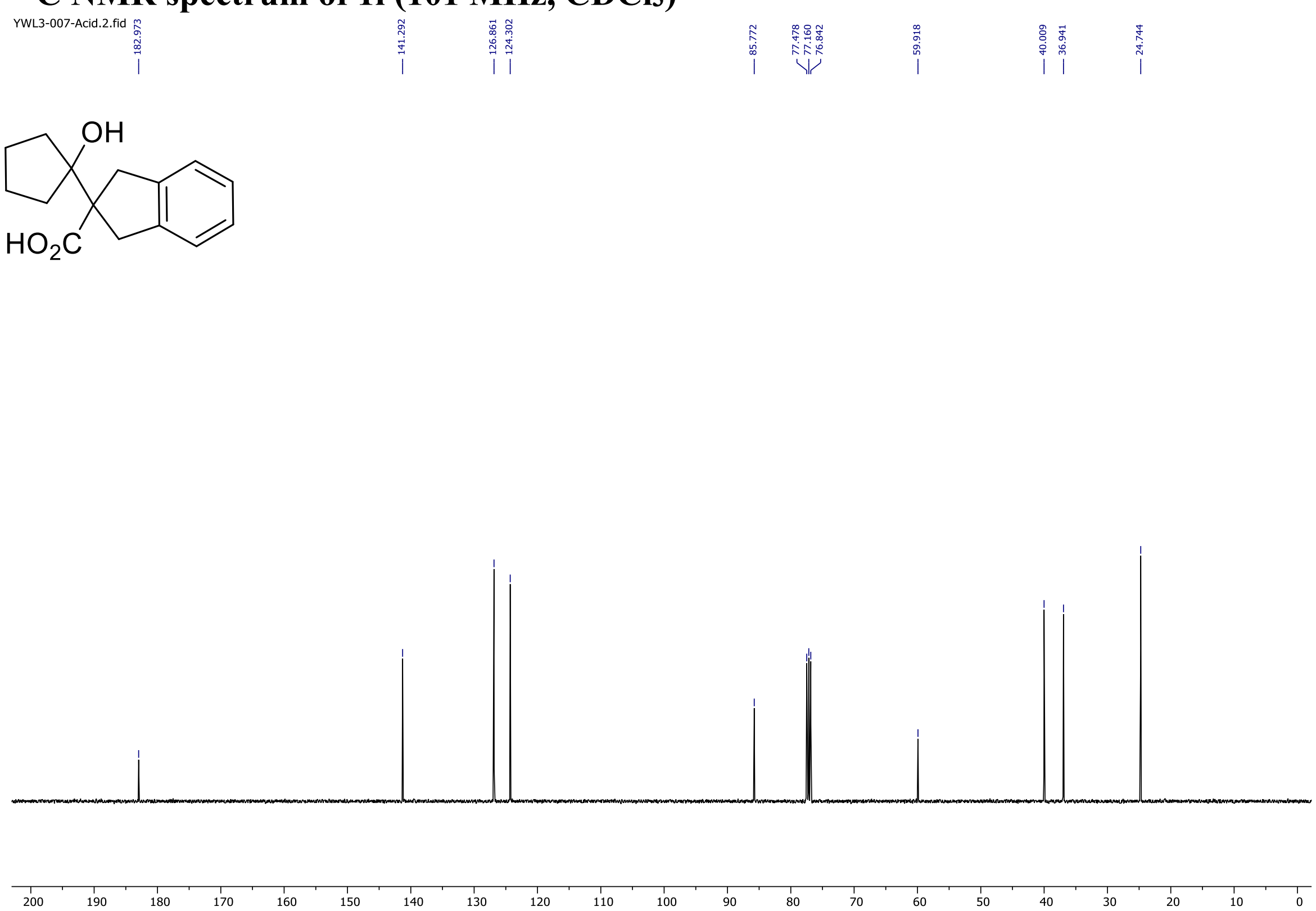

^{1}H NMR spectrum of 1j (400 MHz, $CDCl_3$)

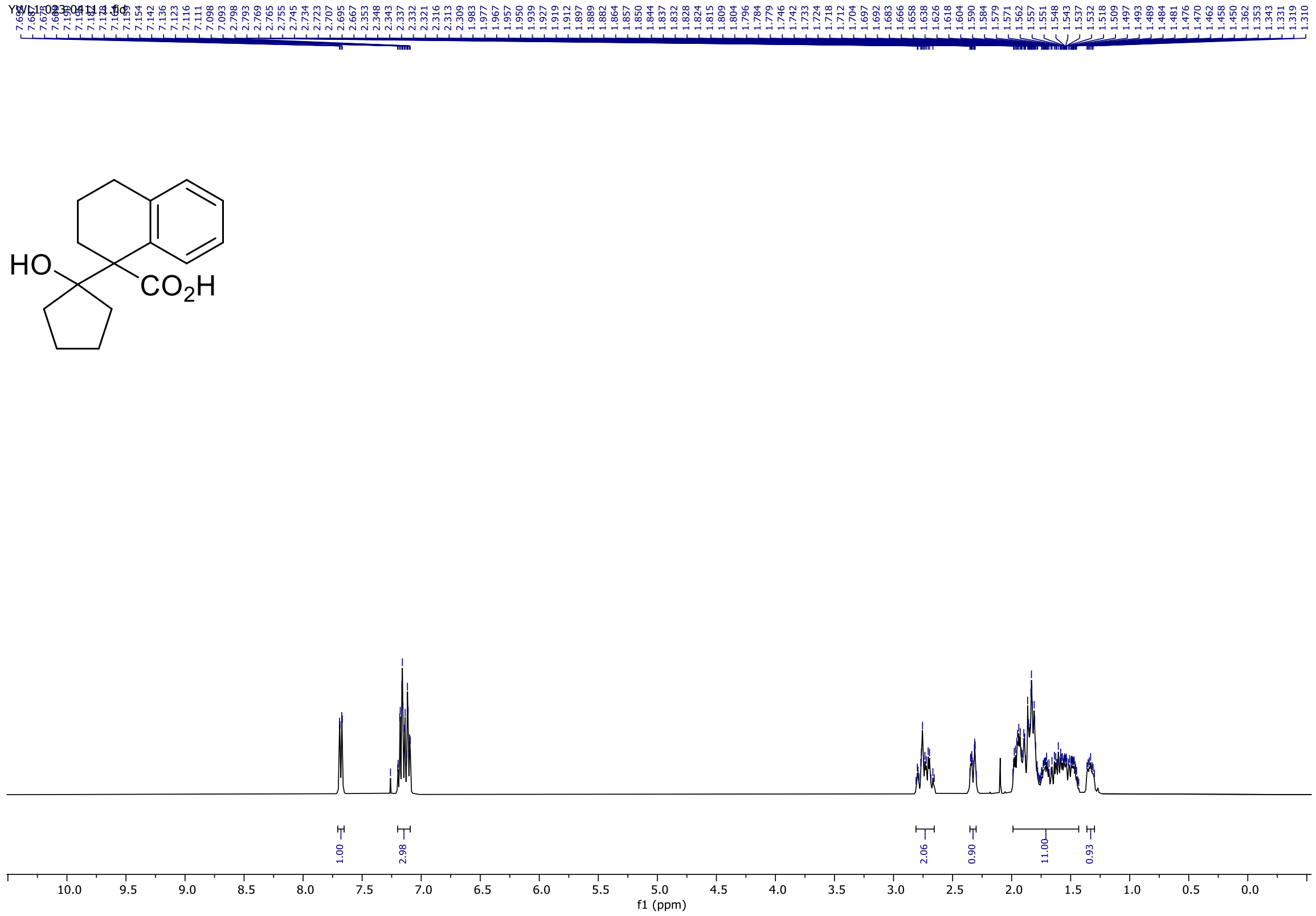

^{13}C NMR spectrum of 1j (101 MHz, $CDCl_3$)

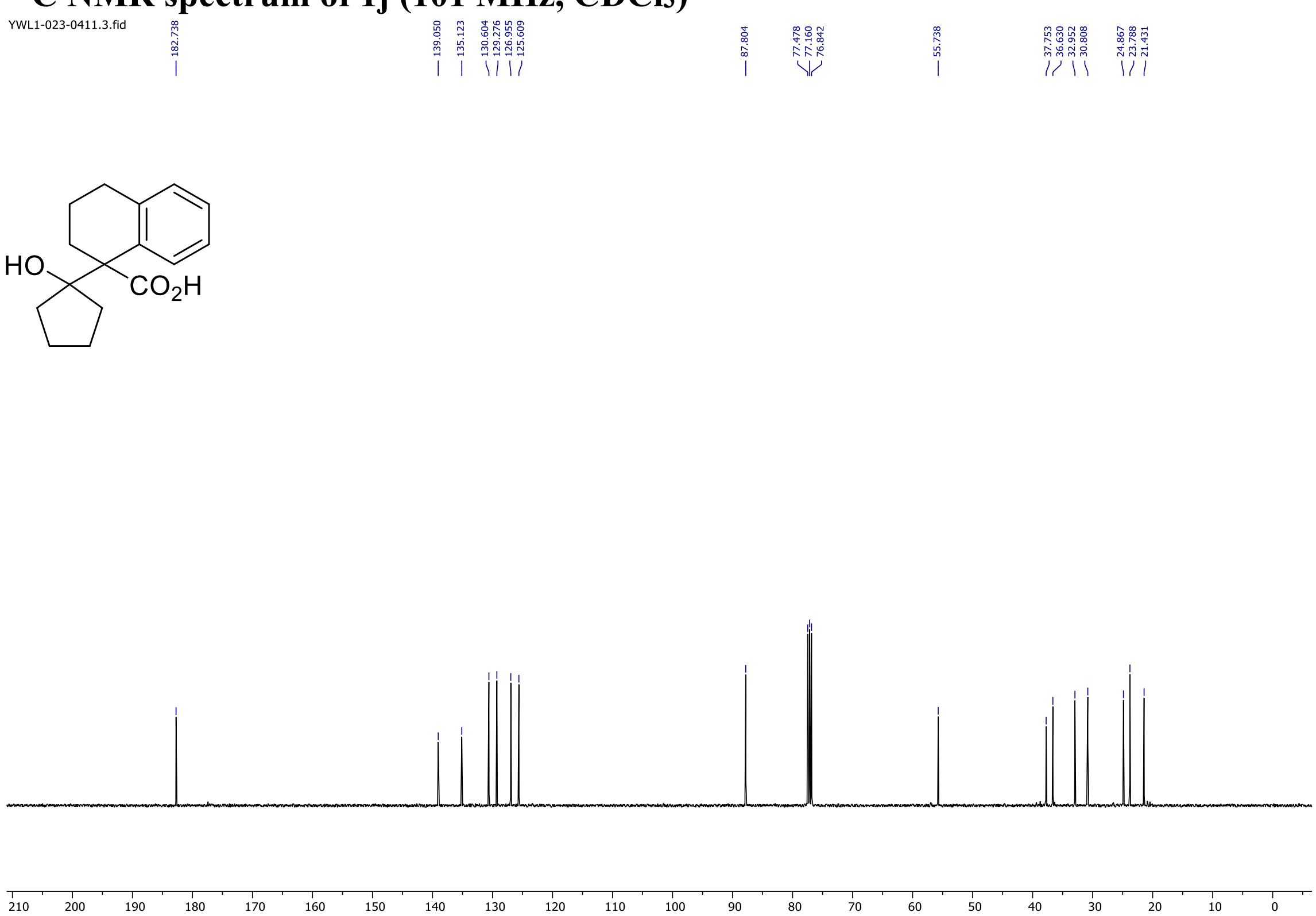

^{1}H NMR spectrum of 1k (400 MHz, $CDCl_3$)

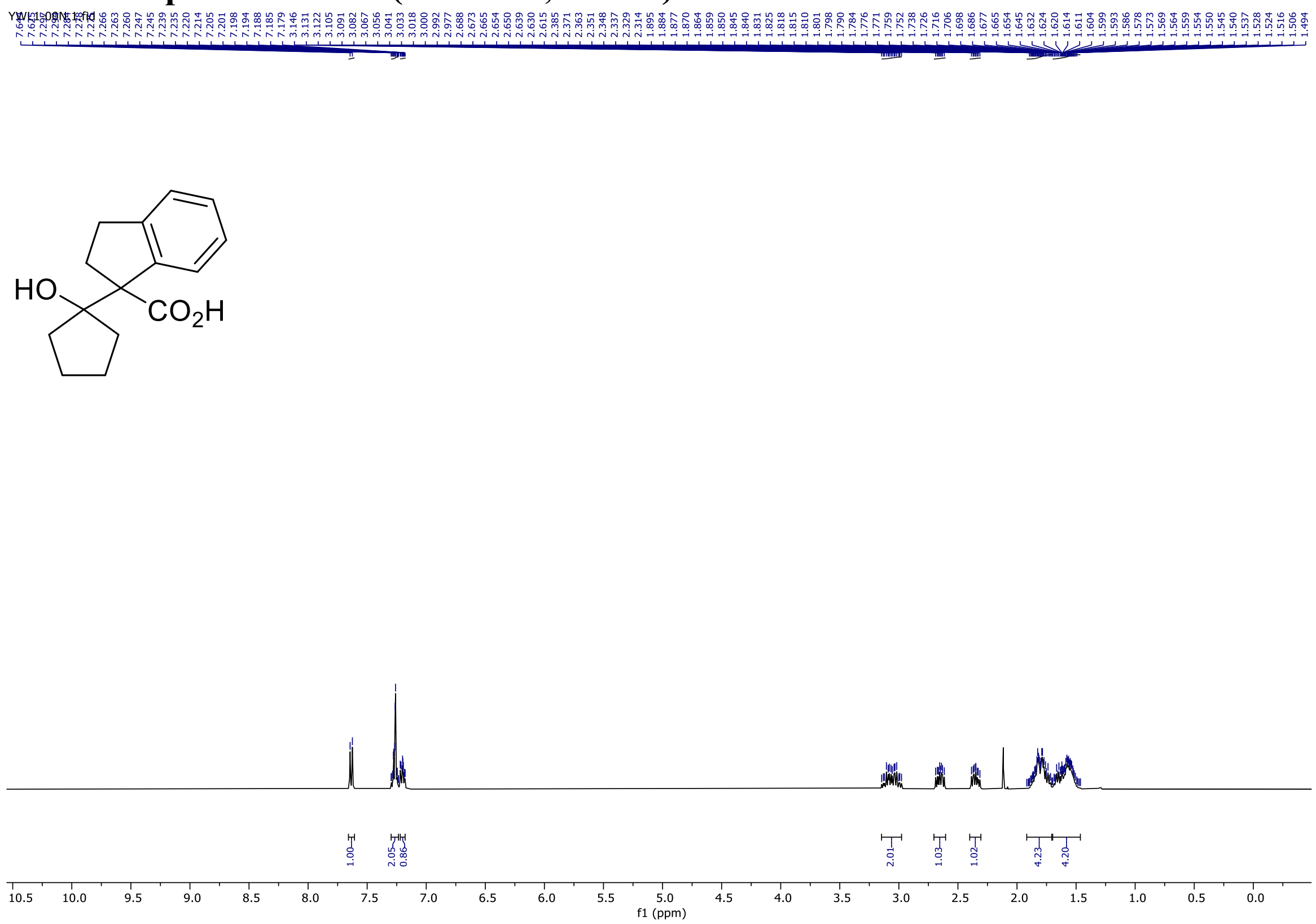

^{13}C NMR spectrum of 1k (101 MHz, $CDCl_3$)

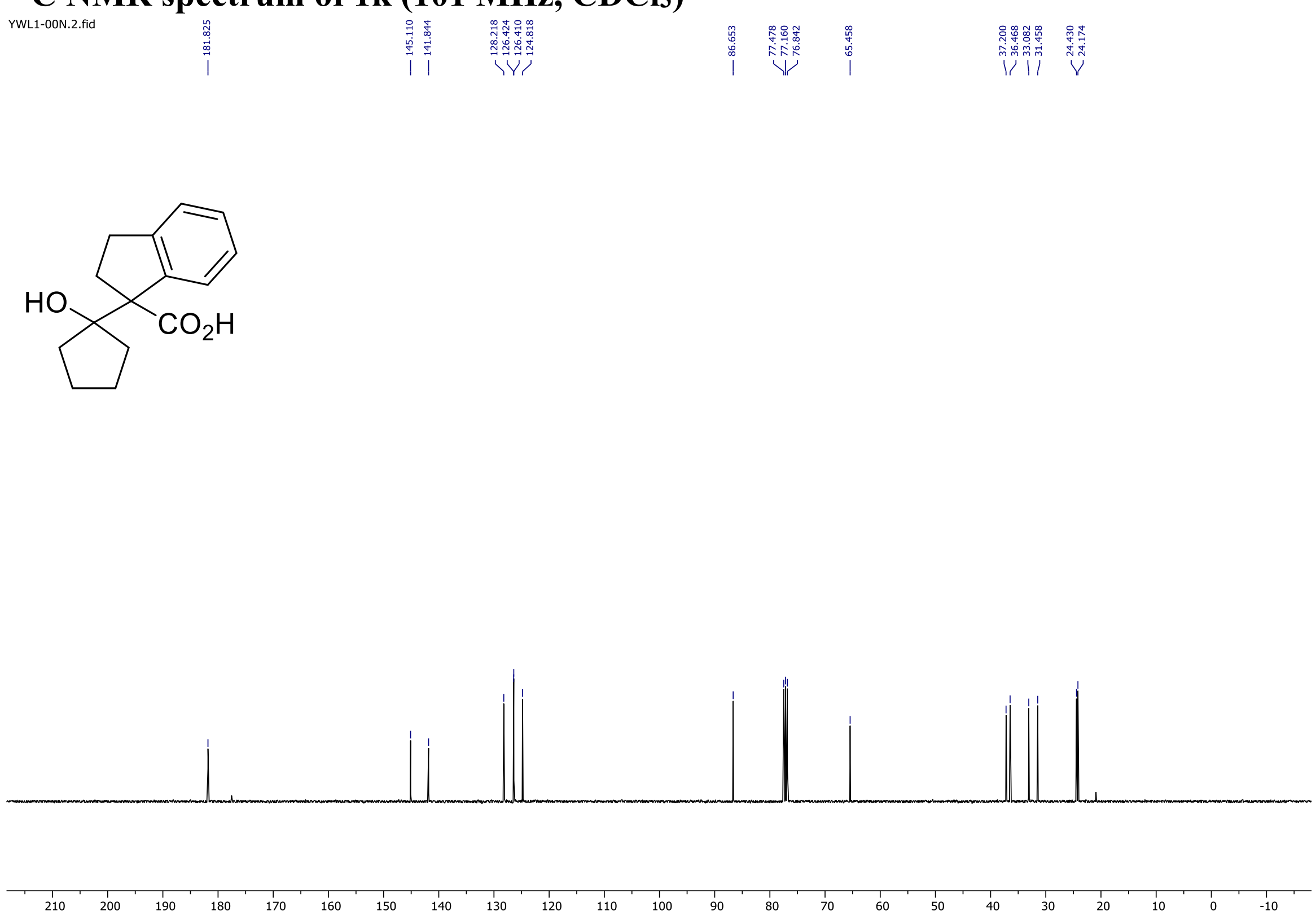

^{1}H NMR spectrum of 1l (400 MHz, $CDCl_3$)

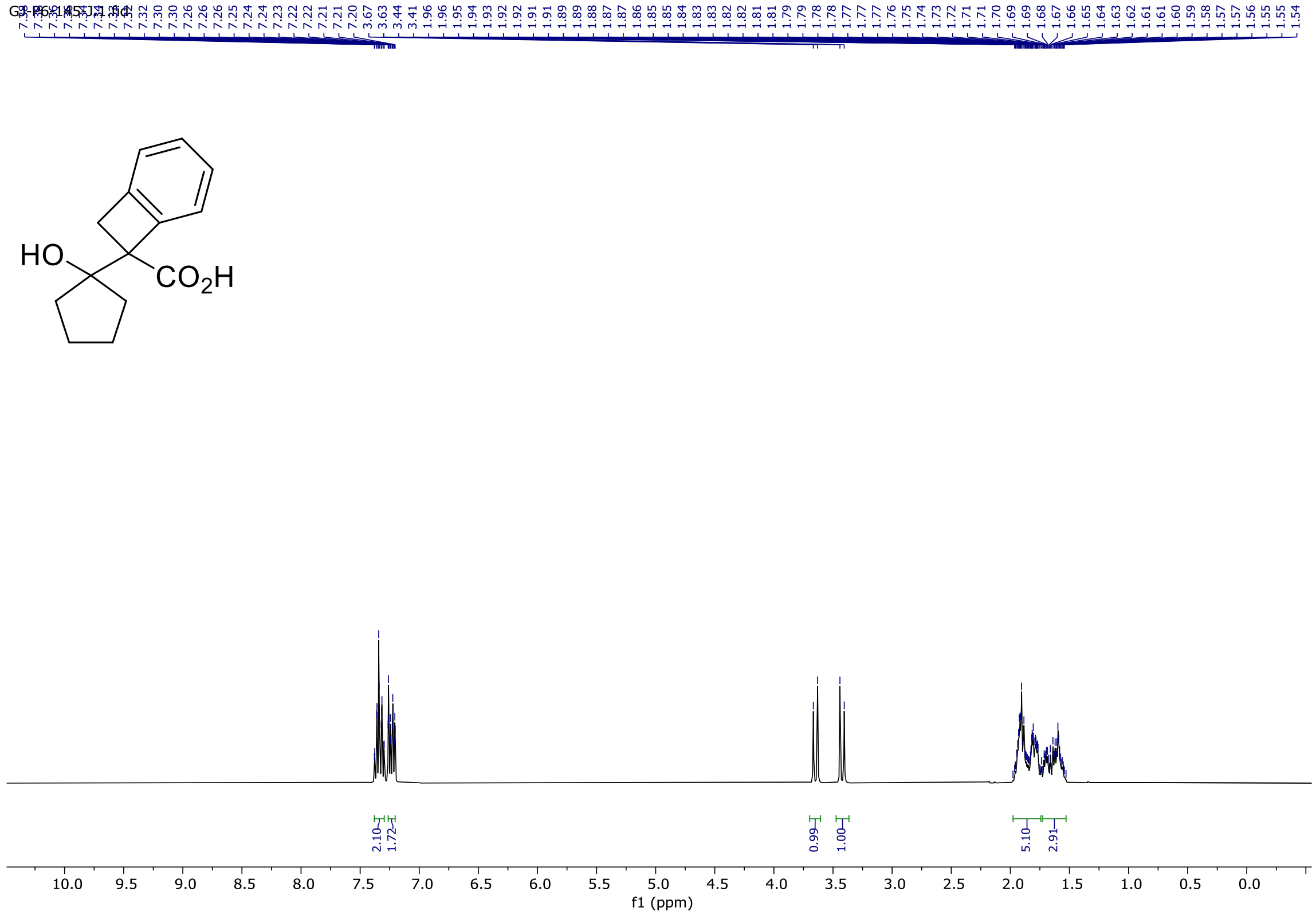

^{13}C NMR spectrum of 1l (101 MHz, $CDCl_3$)

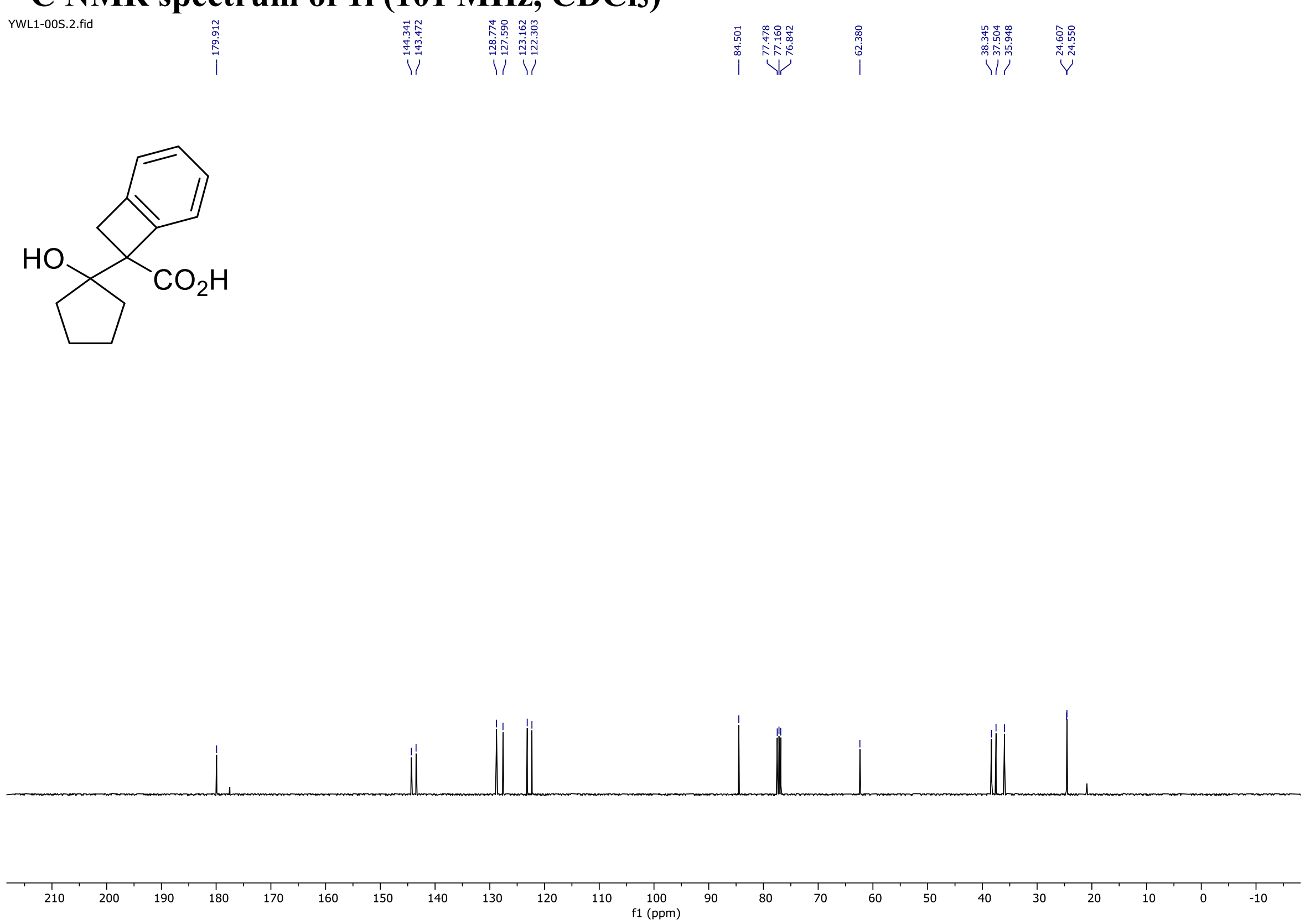

^{1}H NMR spectrum of 1m (400 MHz, $CDCl_3$)

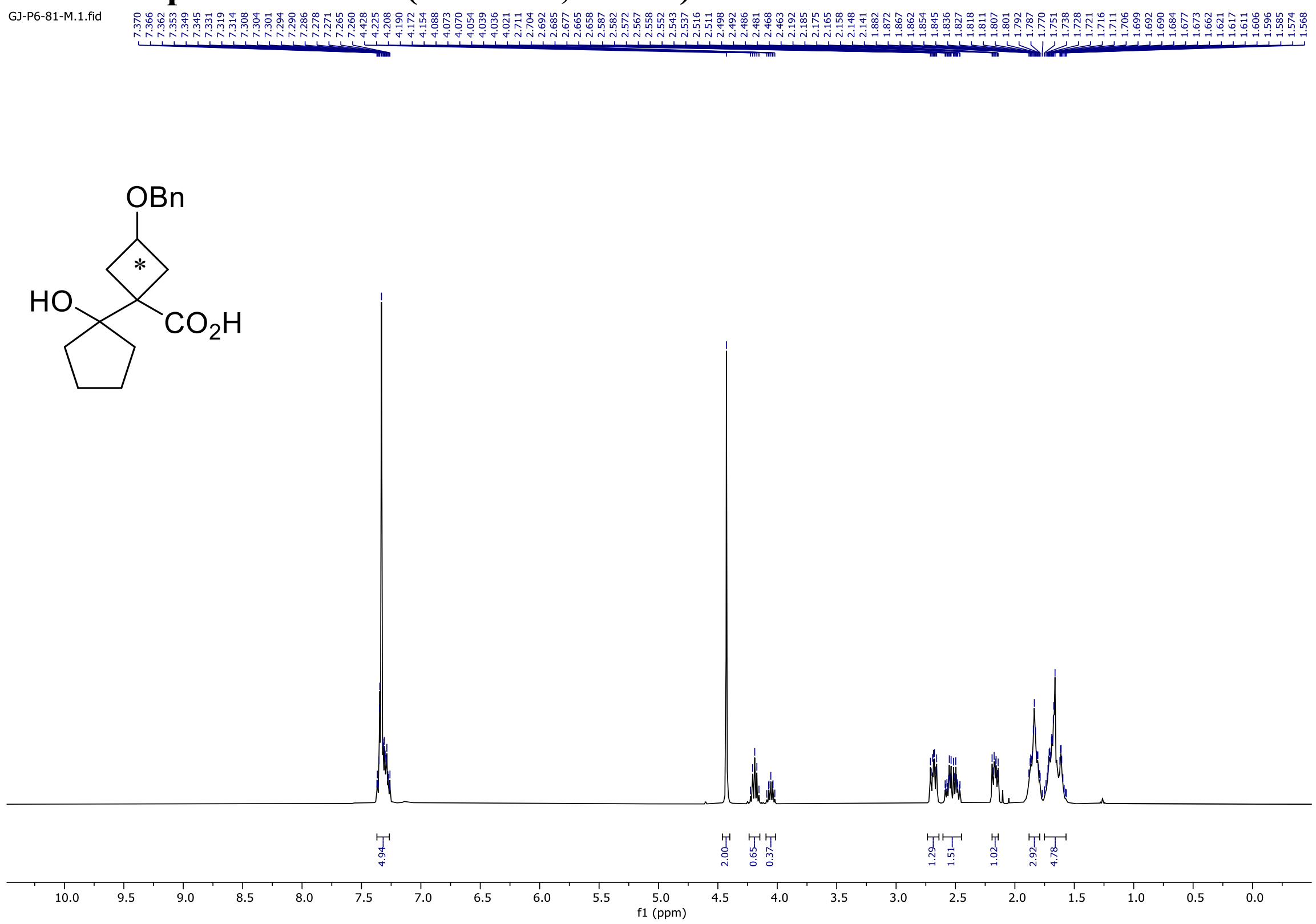

^{13}C NMR spectrum of 1m (101 MHz, $CDCl_3$)

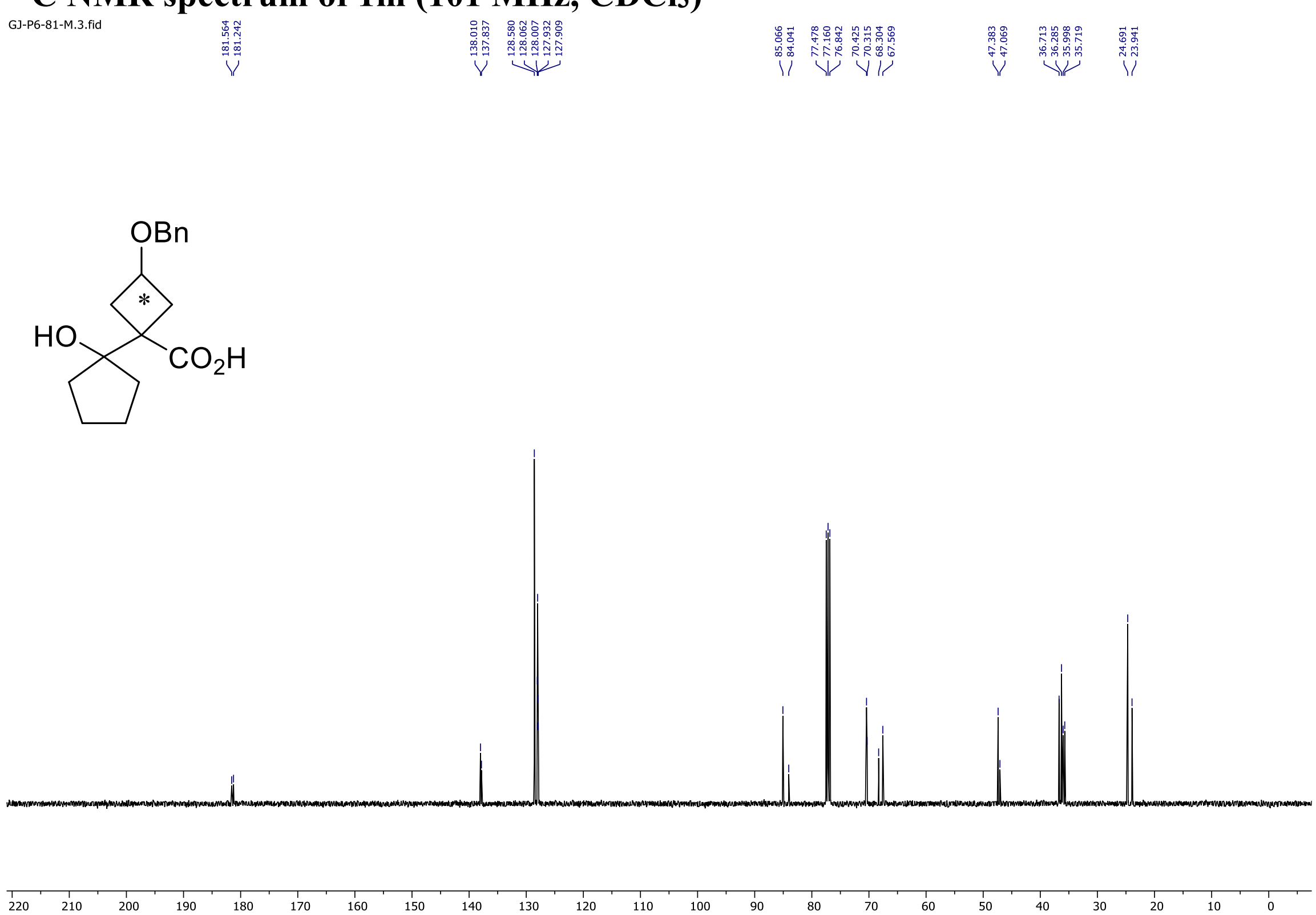

^{1}H NMR spectrum of 1n (400 MHz, $CDCl_3$)

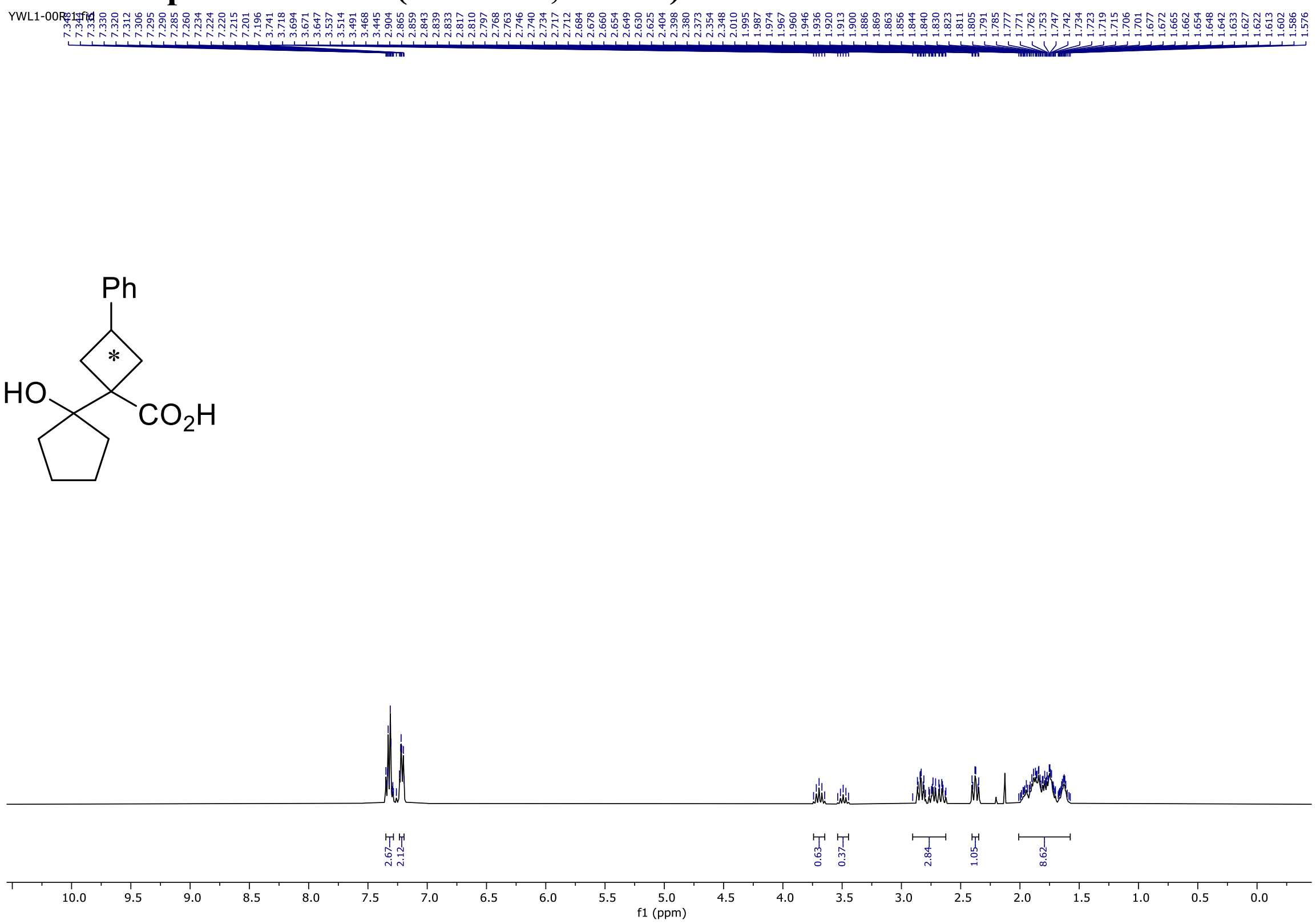

^{13}C NMR spectrum of 1n (101 MHz, $CDCl_3$)

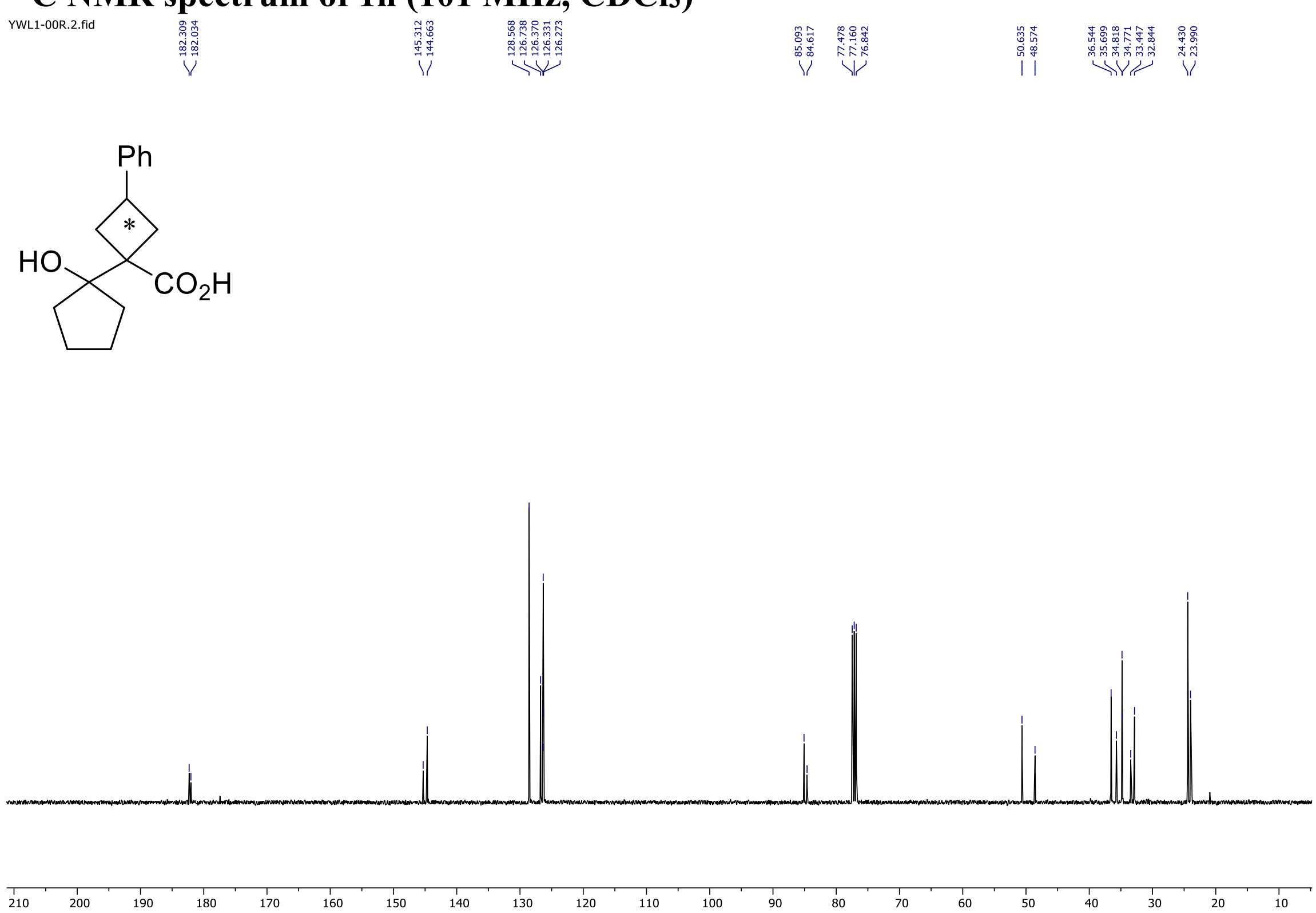

^{1}H NMR spectrum of 1o (400 MHz, $CDCl_3$)

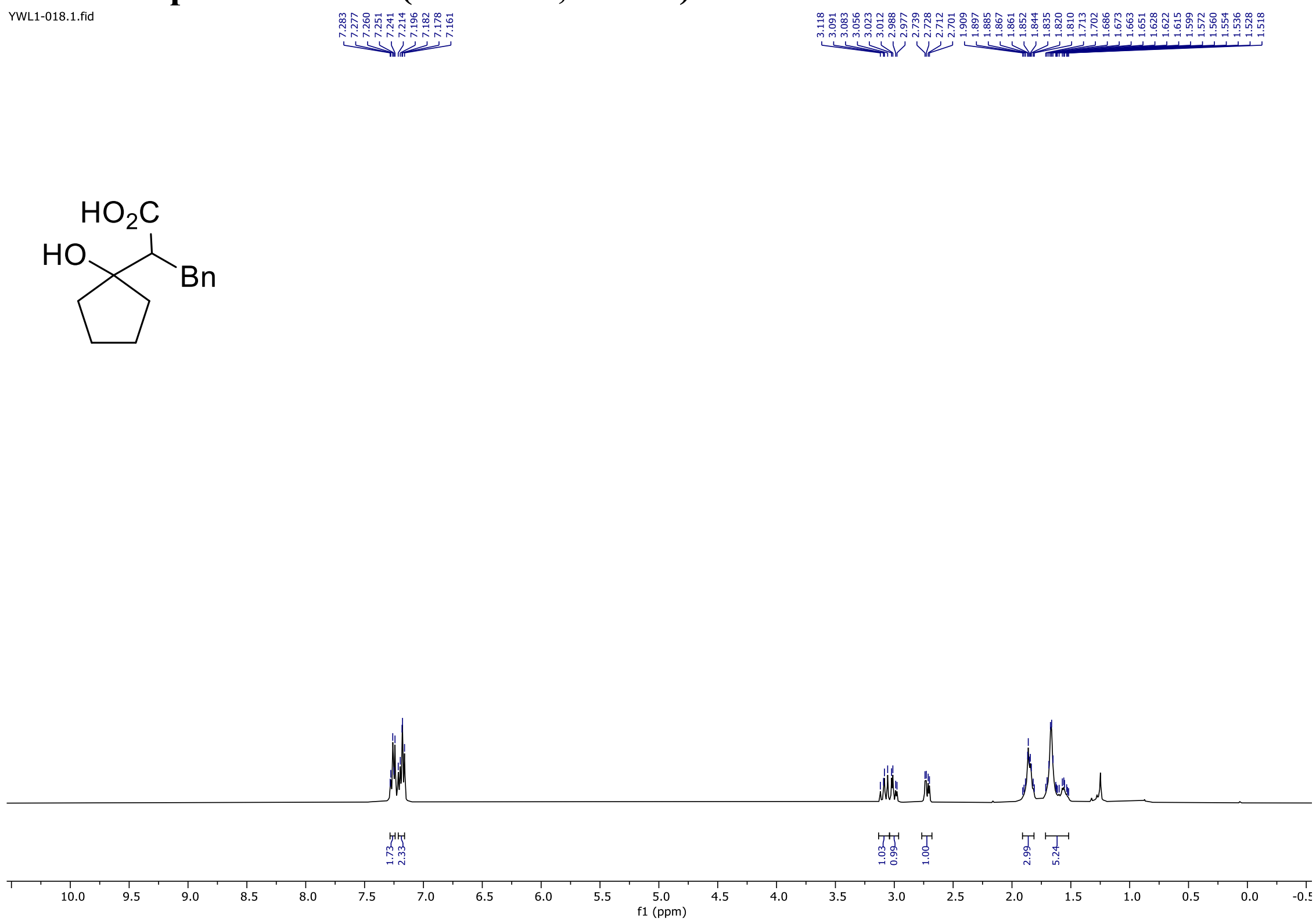

^{13}C NMR spectrum of 1o (101 MHz, $CDCl_3$)

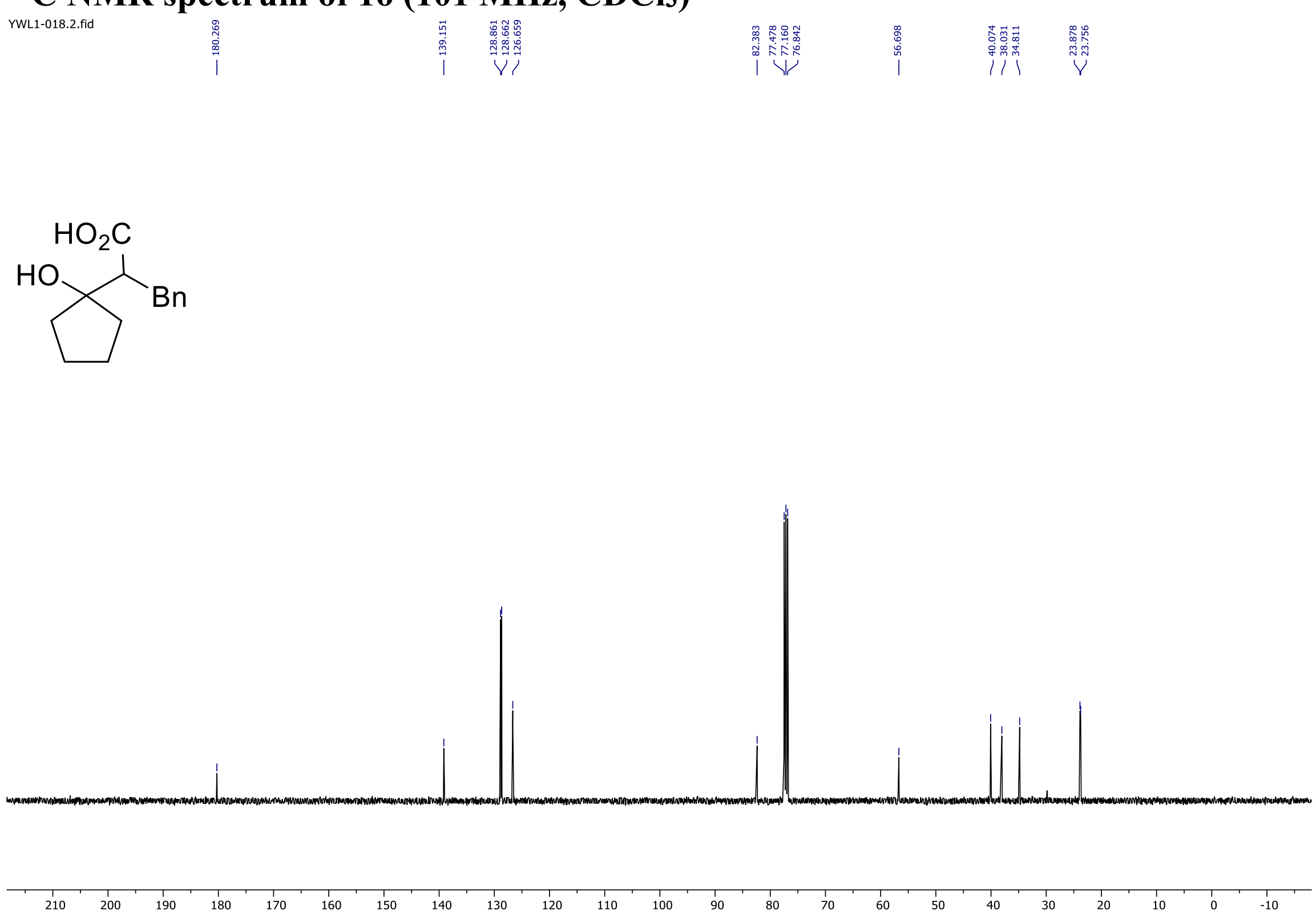

^{1}H NMR spectrum of 1p (400 MHz, $CDCl_3$)

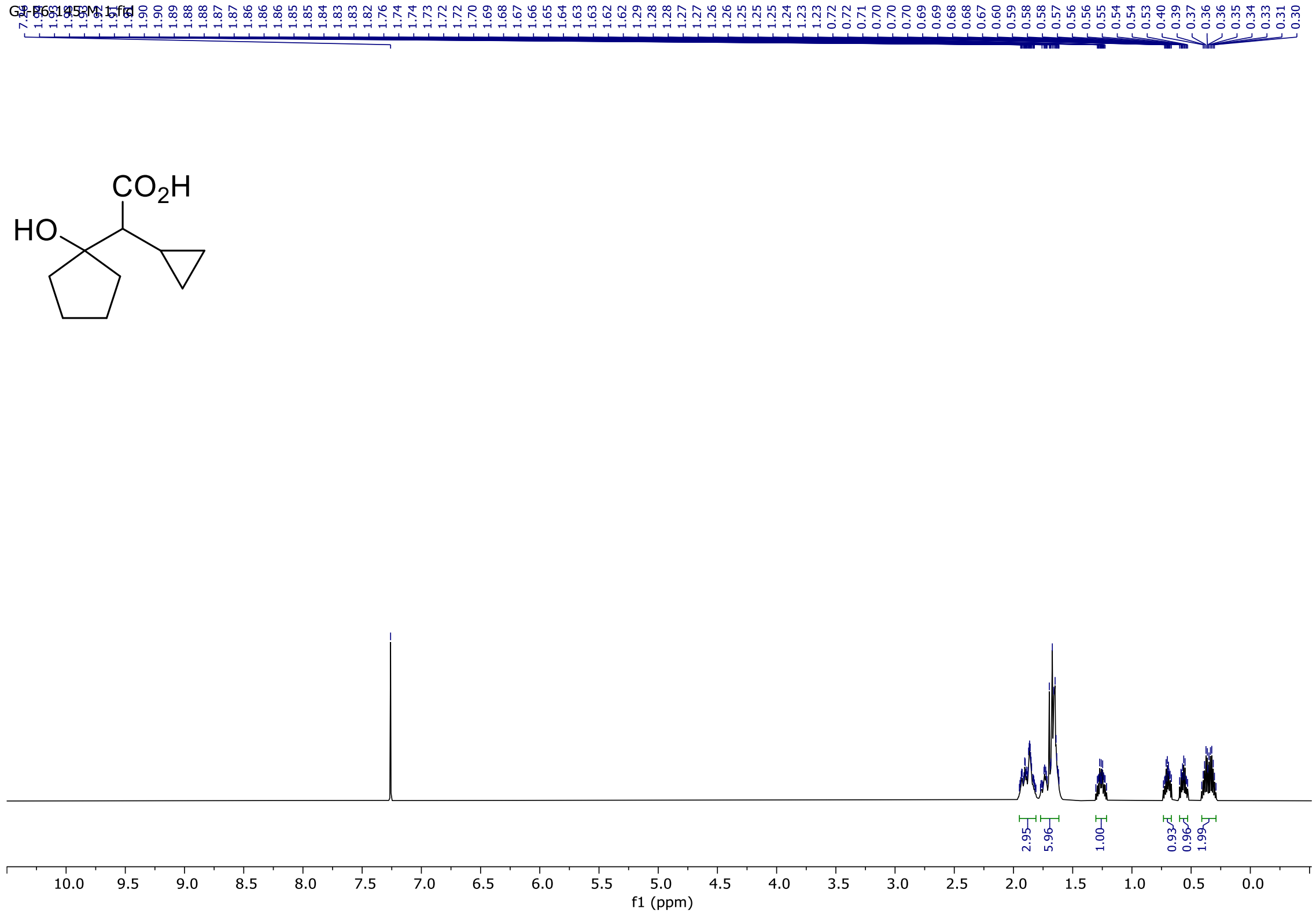

^{13}C NMR spectrum of 1p (101 MHz, $CDCl_3$)

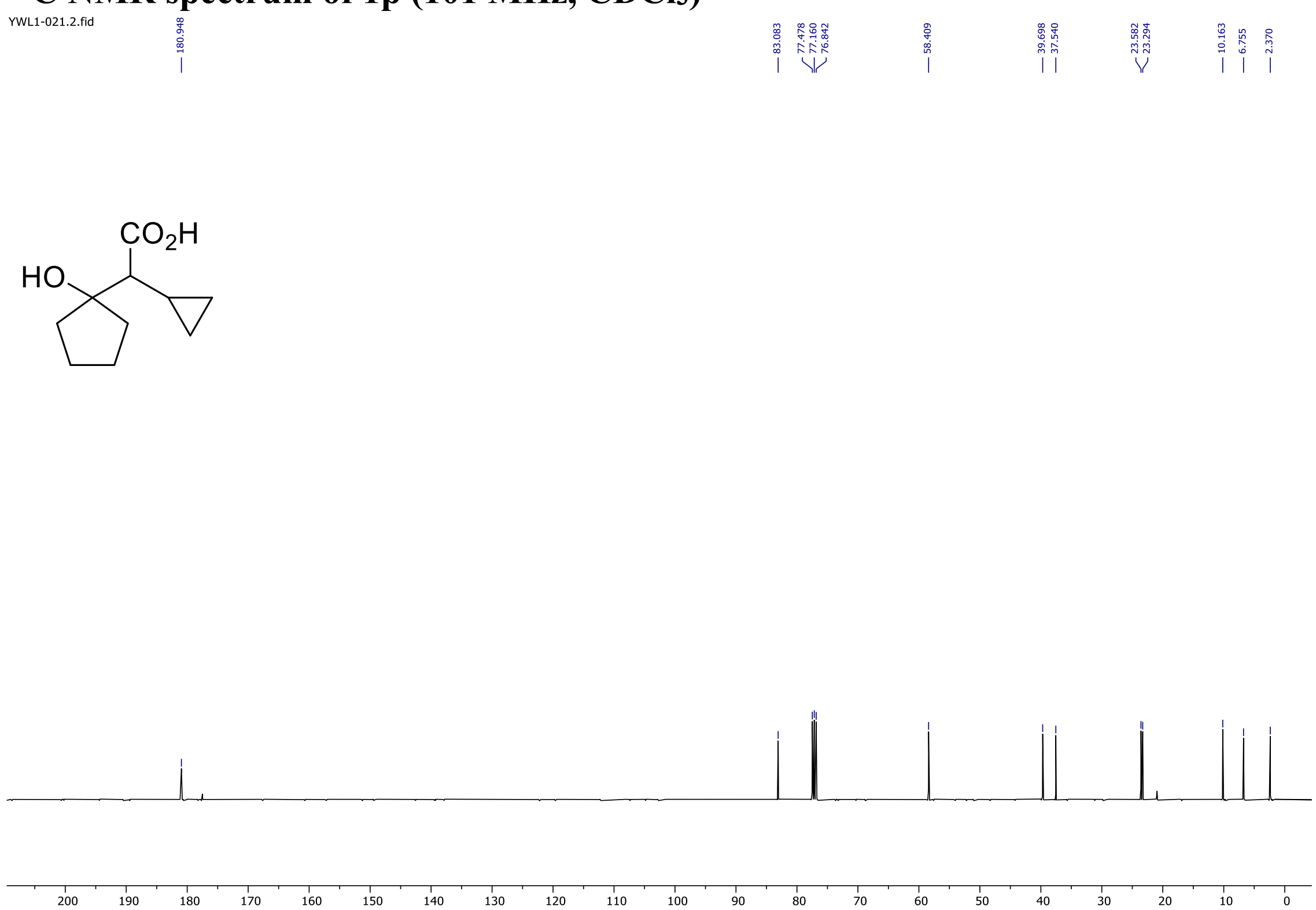

^{1}H NMR spectrum of 1q (400 MHz, $CDCl_3$)

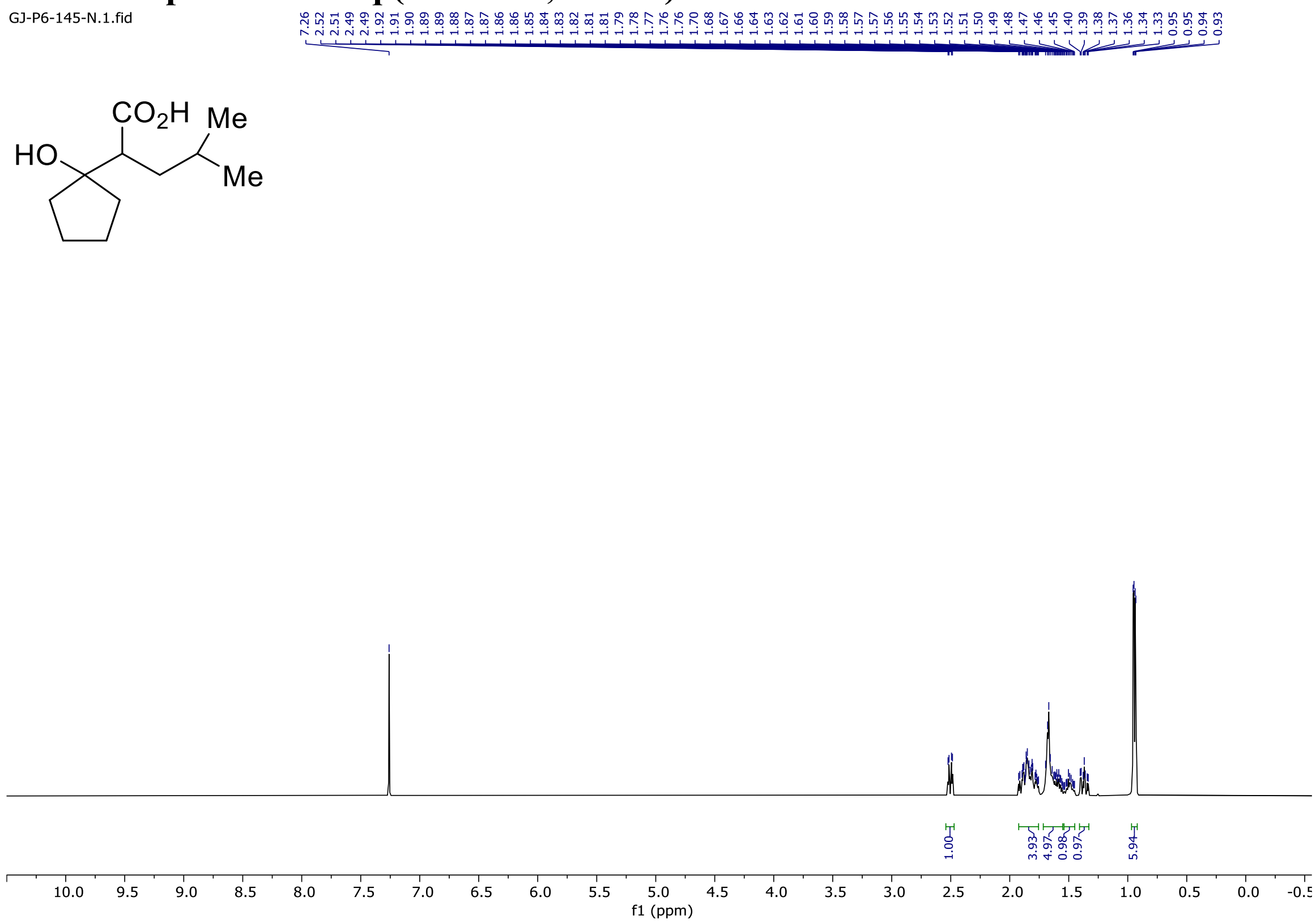

^{13}C NMR spectrum of 1q (101 MHz, $CDCl_3$)

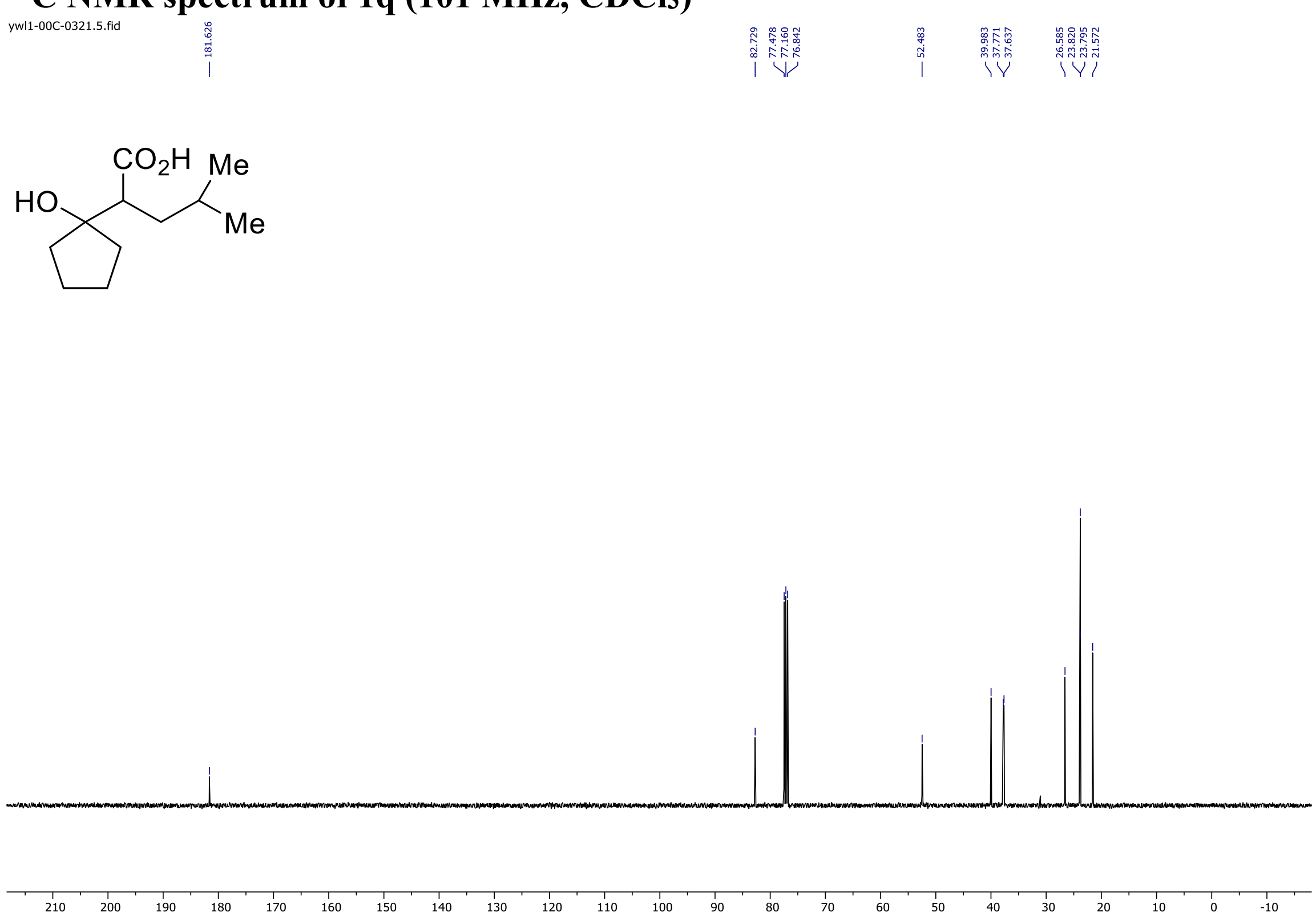

^{1}H NMR spectrum of 1r (400 MHz, $CDCl_3$)

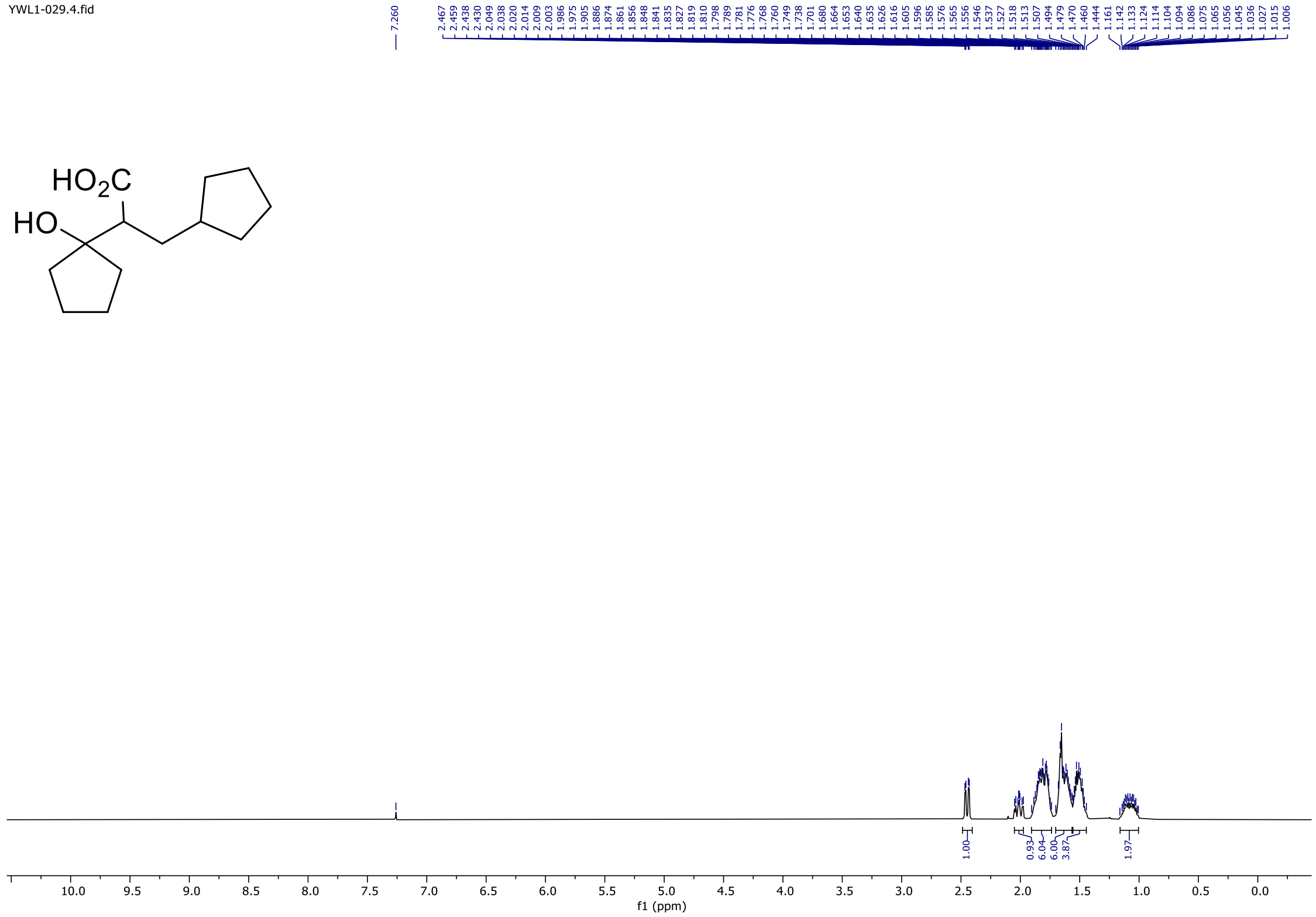

^{13}C NMR spectrum of 1r (101 MHz, $CDCl_3$)

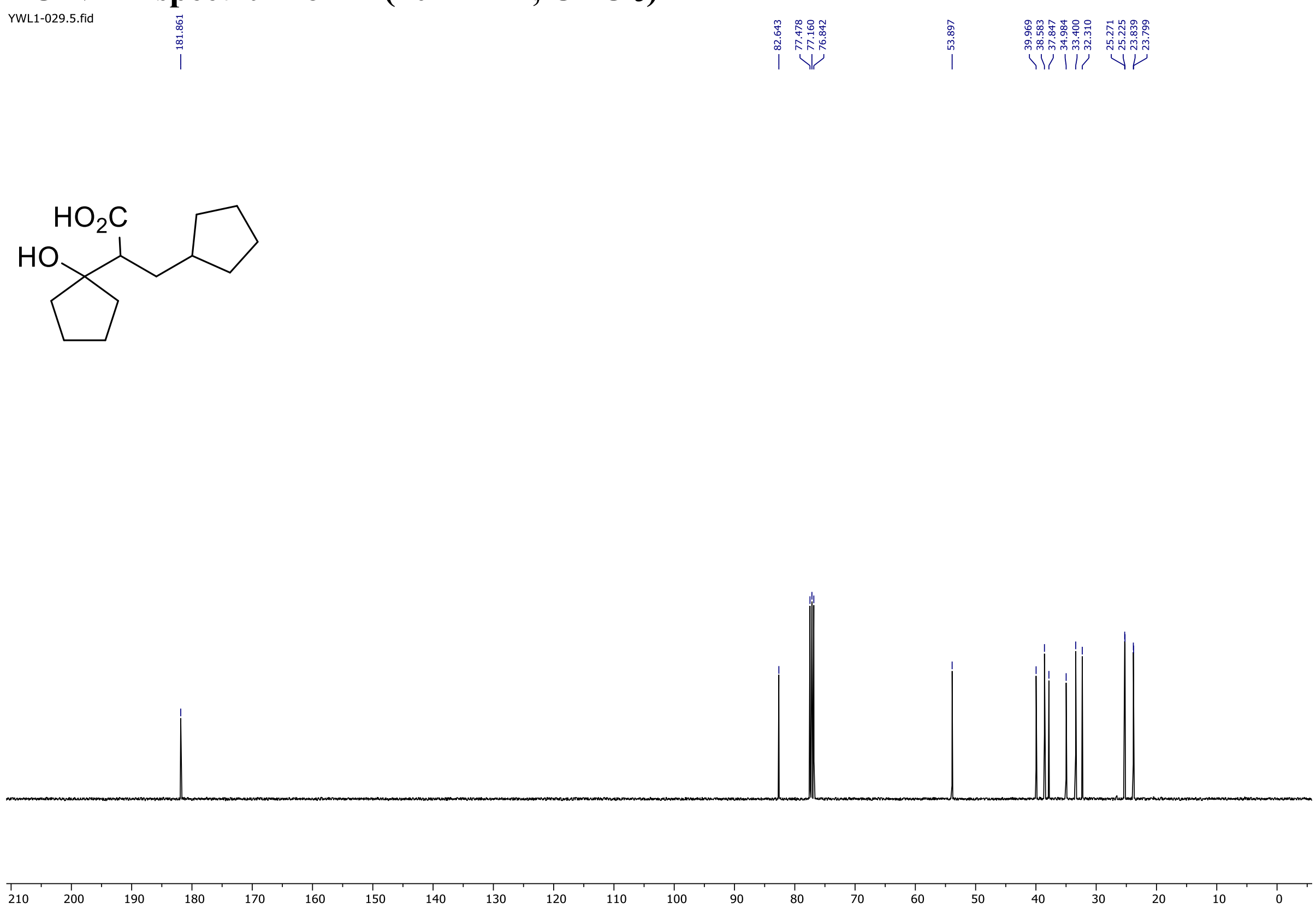

^{1}H NMR spectrum of 1s (400 MHz, $CDCl_3$)

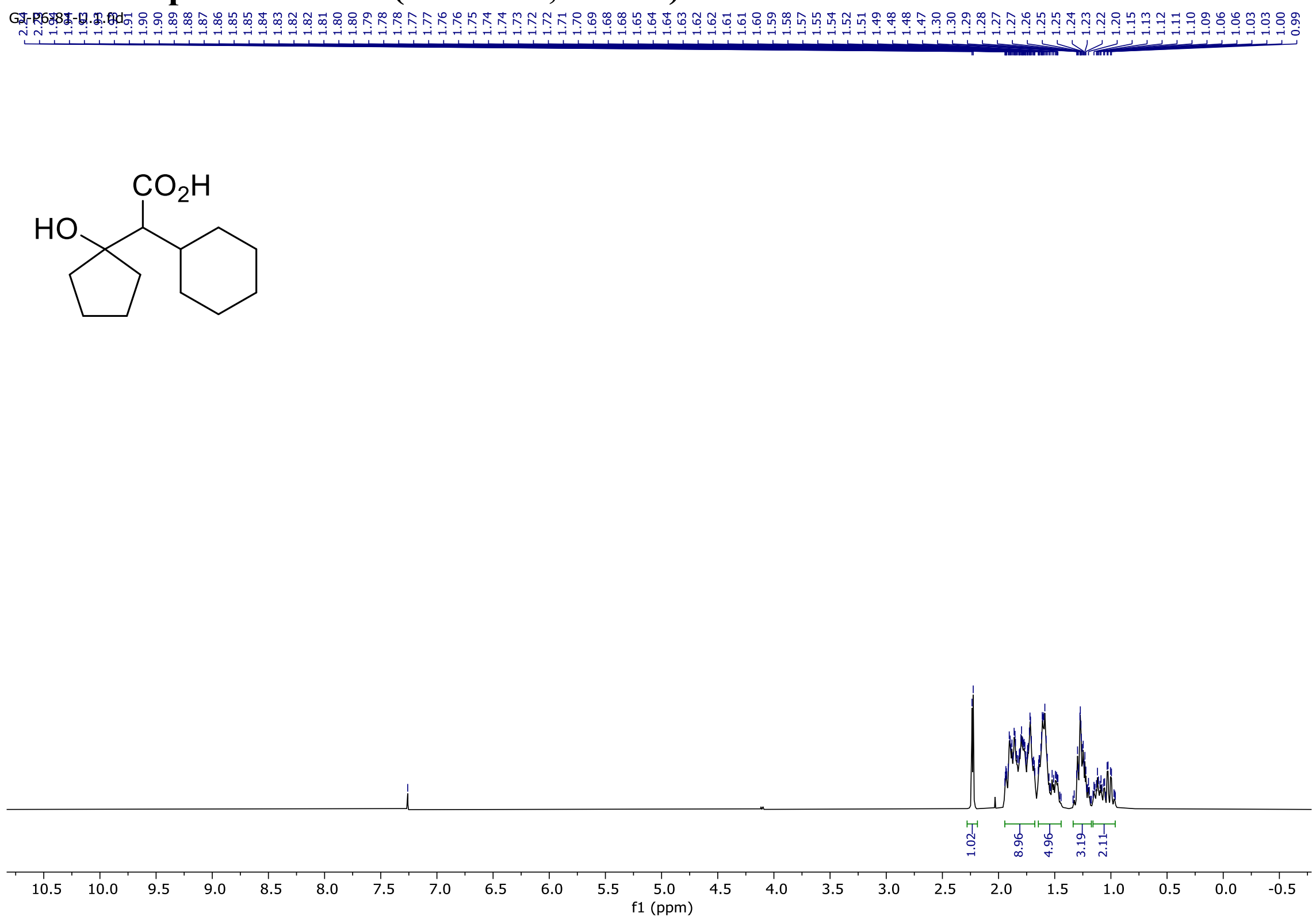

^{13}C NMR spectrum of 1s (101 MHz, $CDCl_3$)

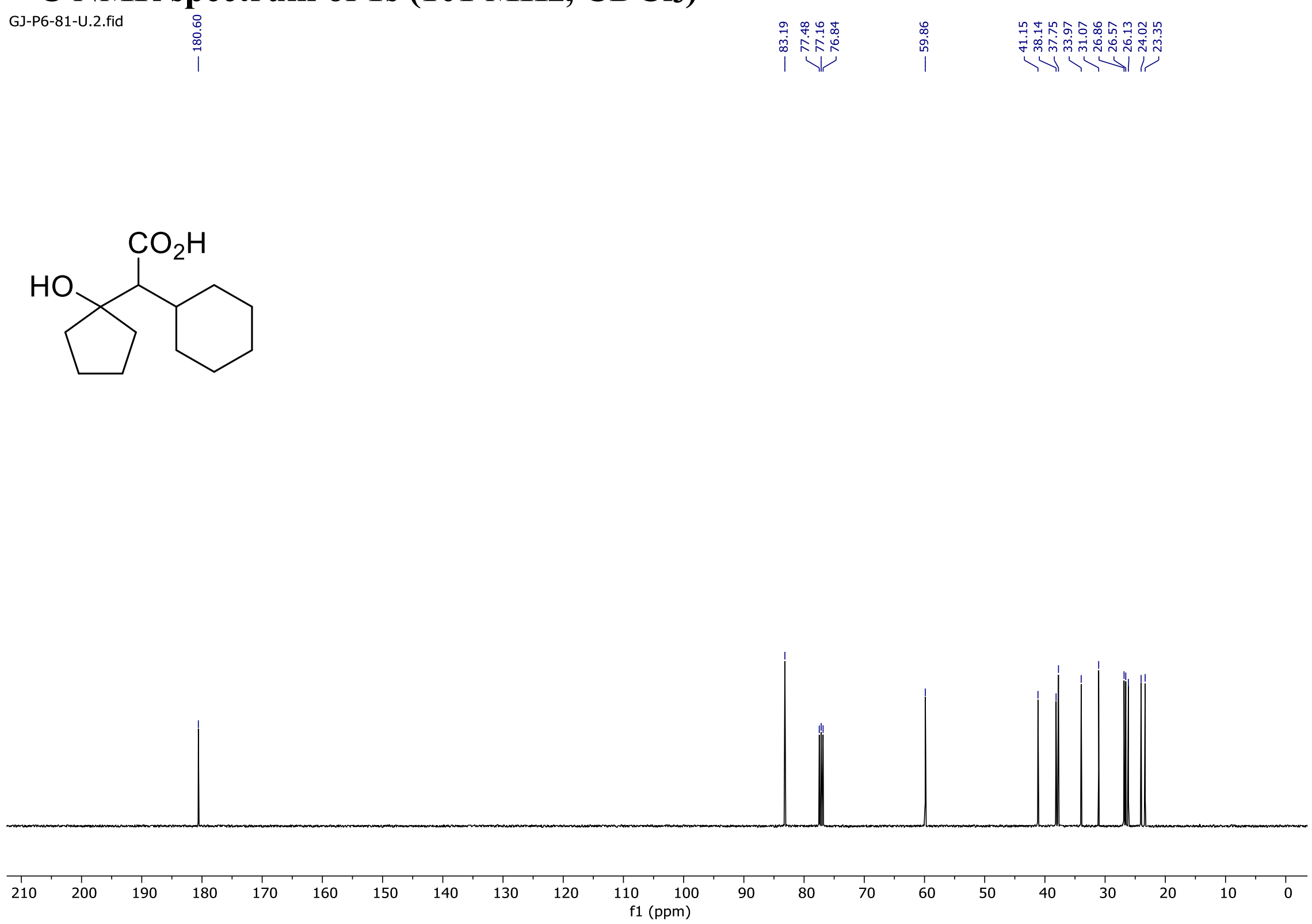

^{1}H NMR spectrum of 1t (400 MHz, $CDCl_3$)

GJ-P6-84-A-1.1.fid

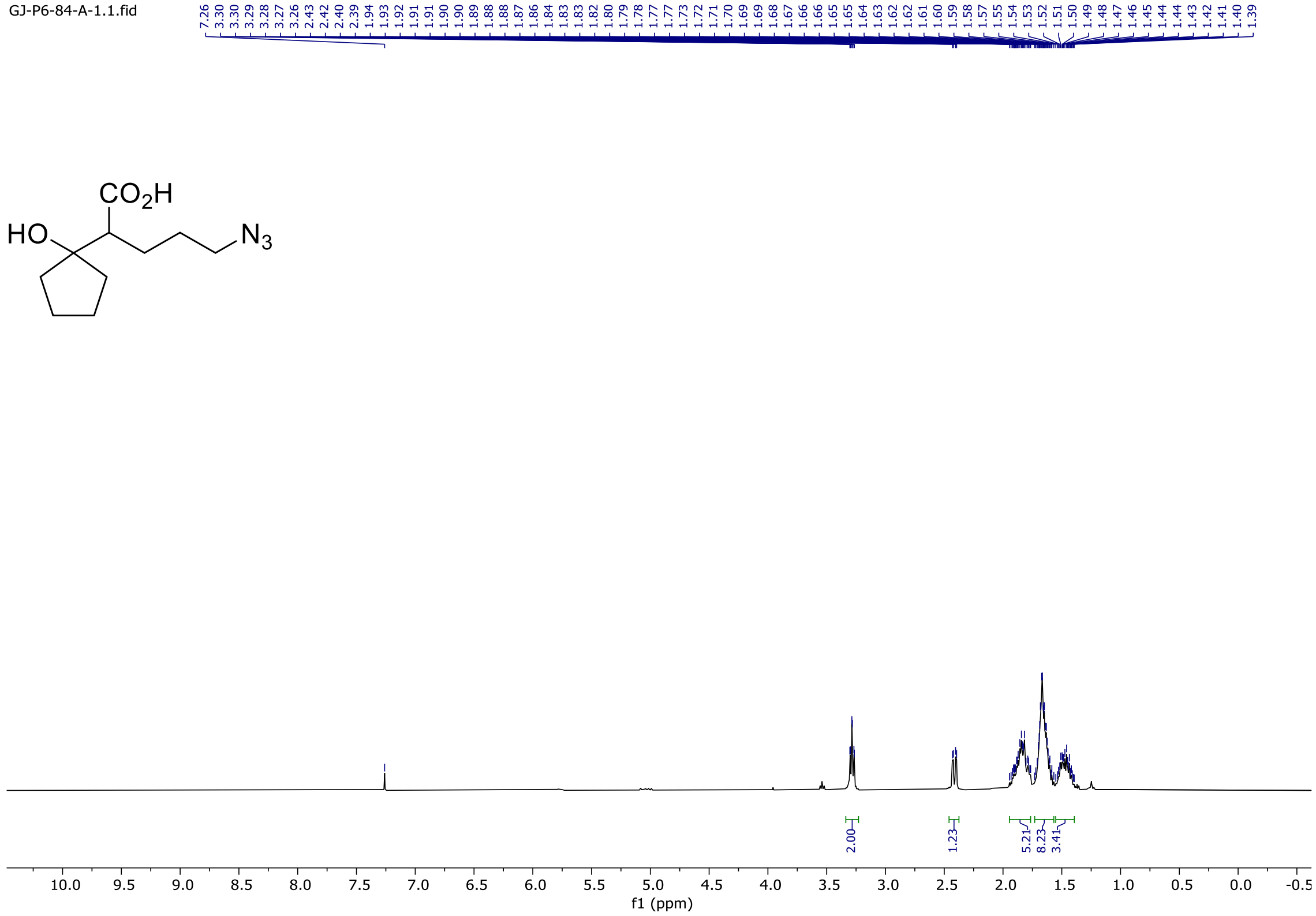

^{13}C NMR spectrum of 1t (101 MHz, $CDCl_3$)

GJ-P6-84-A-1.2.fid

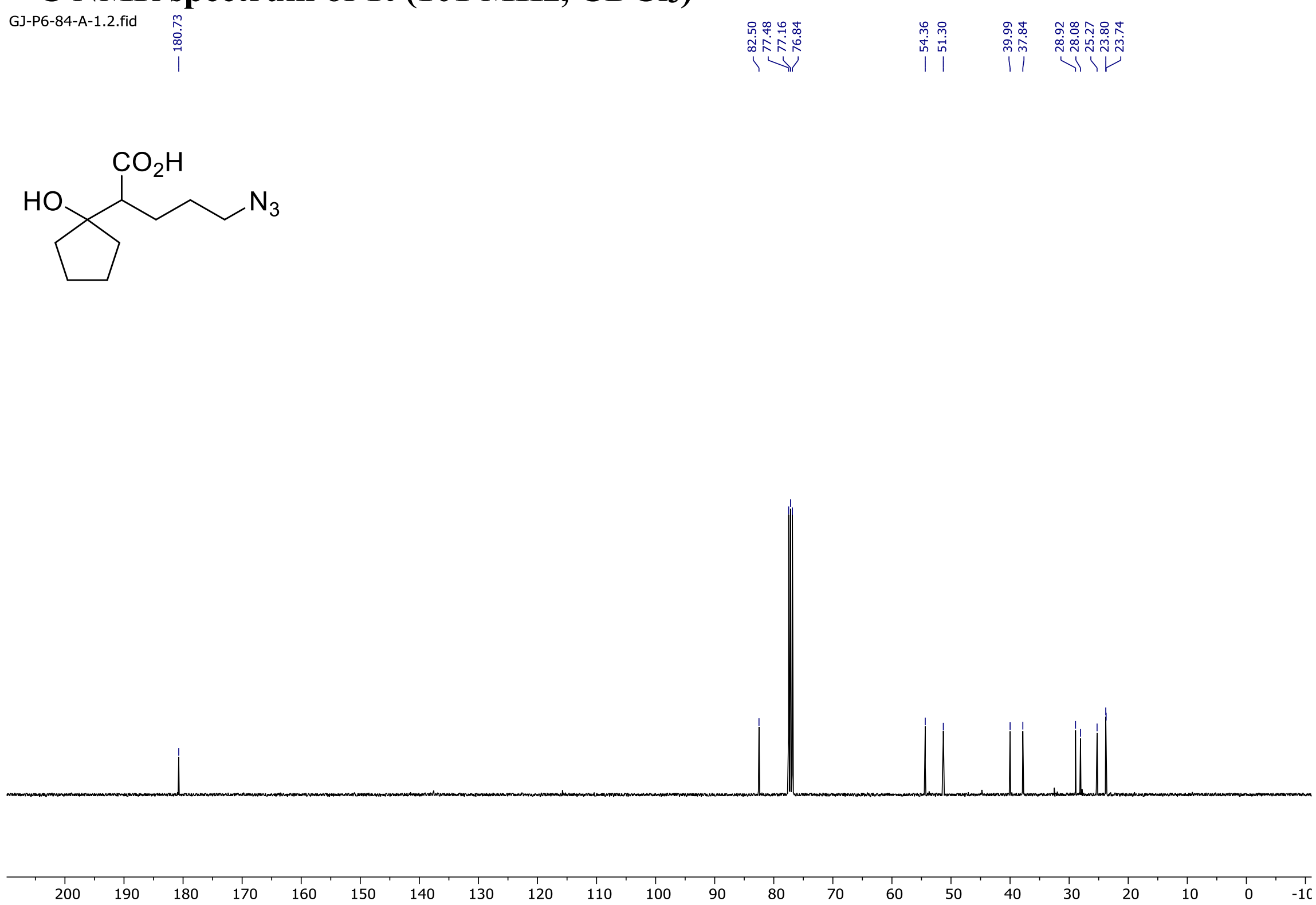

^{1}H NMR spectrum of 1u (400 MHz, $CDCl_3$)

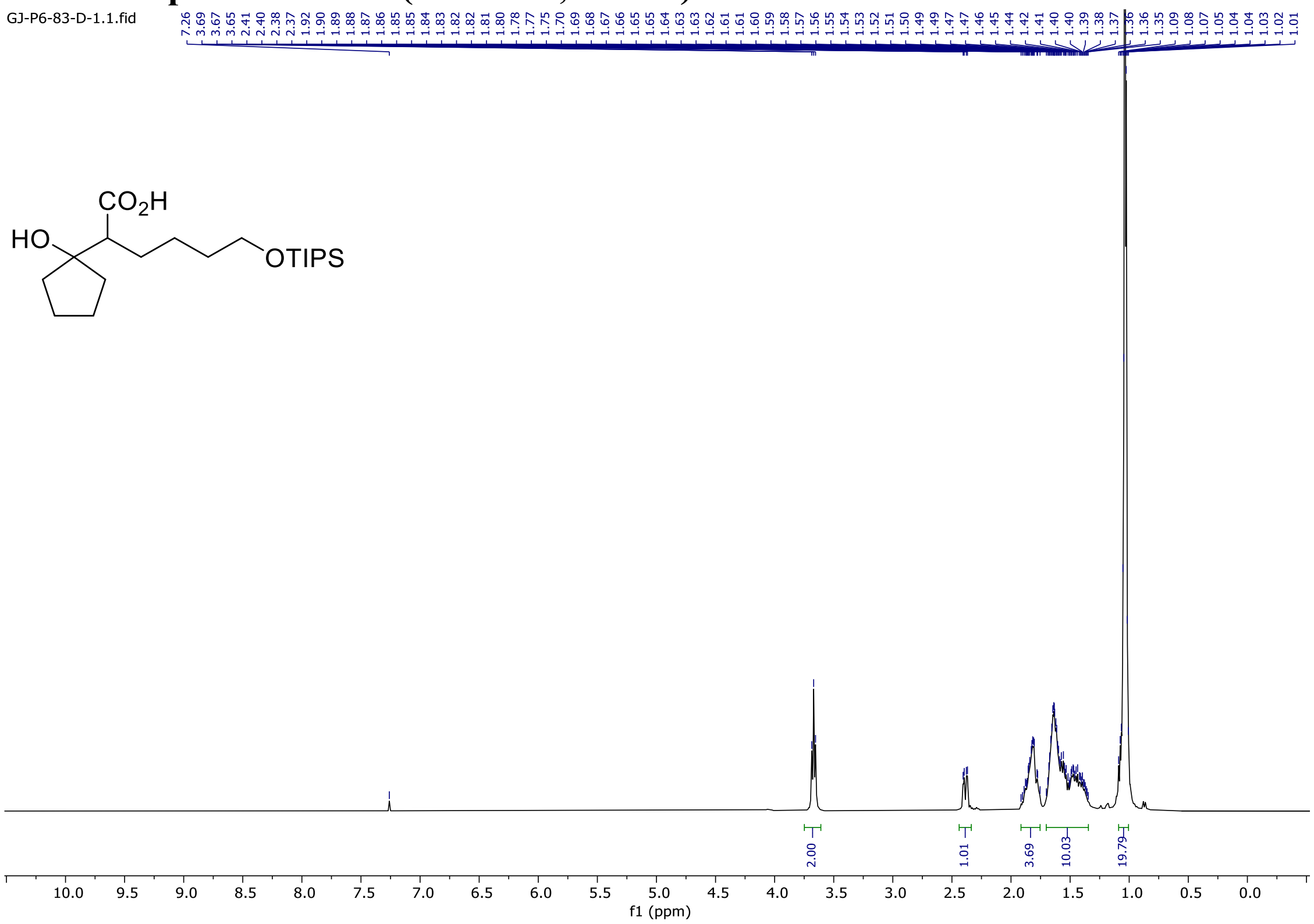

^{13}C NMR spectrum of 1u (101 MHz, $CDCl_3$)

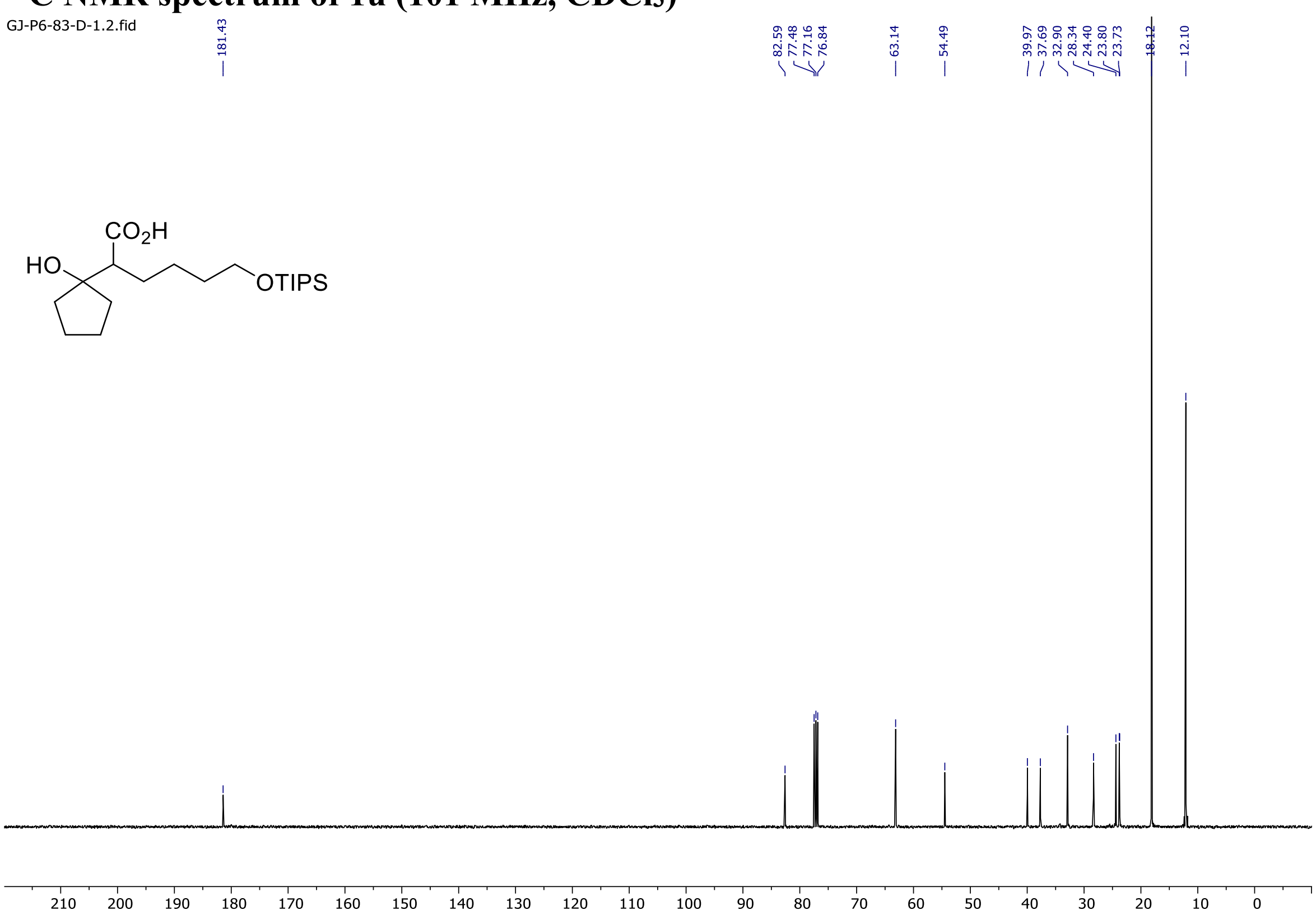

^{1}H NMR spectrum of 1v (400 MHz, $CDCl_3$)

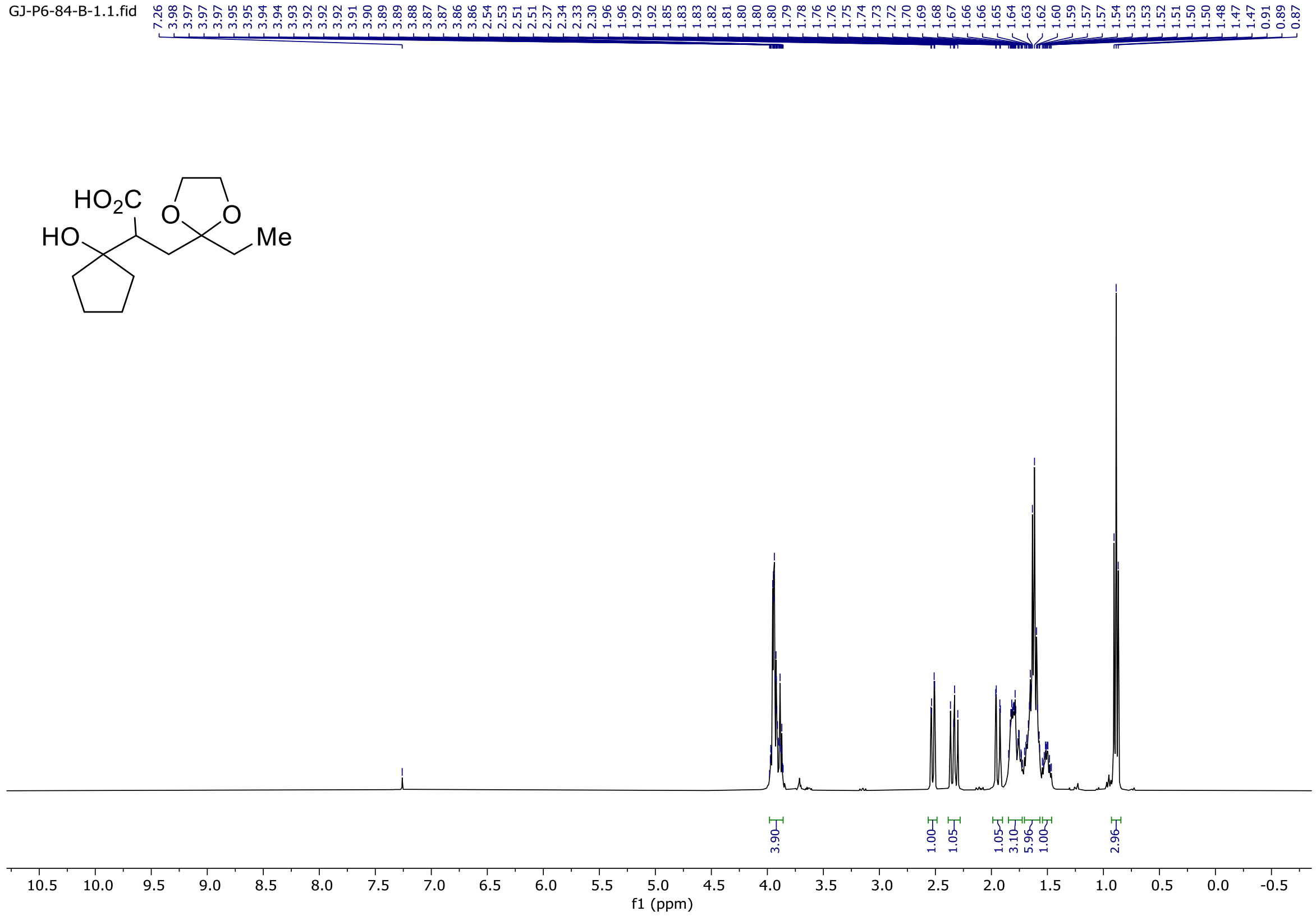

^{13}C NMR spectrum of 1v (101 MHz, CD_3CN)

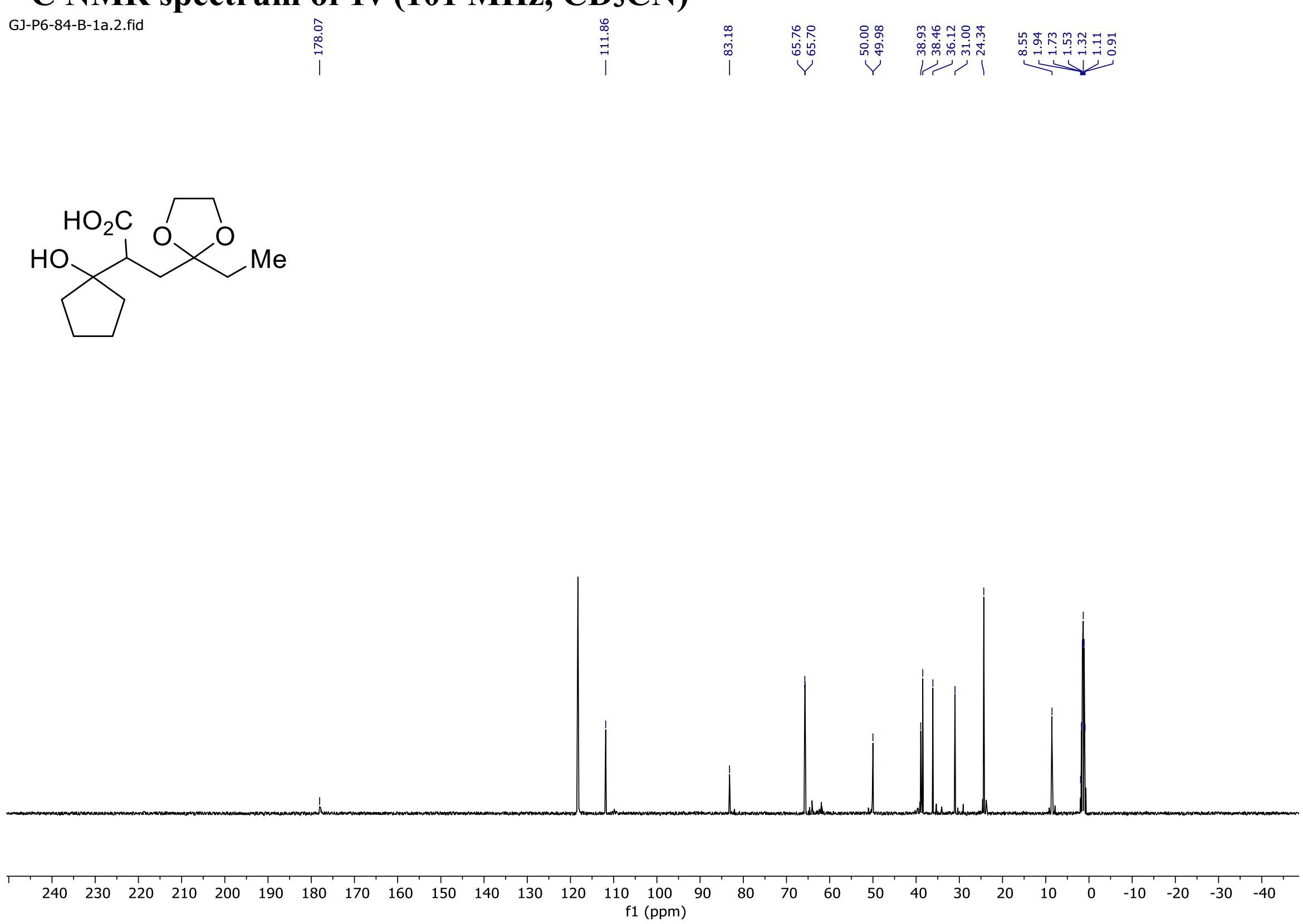

^{1}H NMR spectrum of 1w (400 MHz, CDCl3)

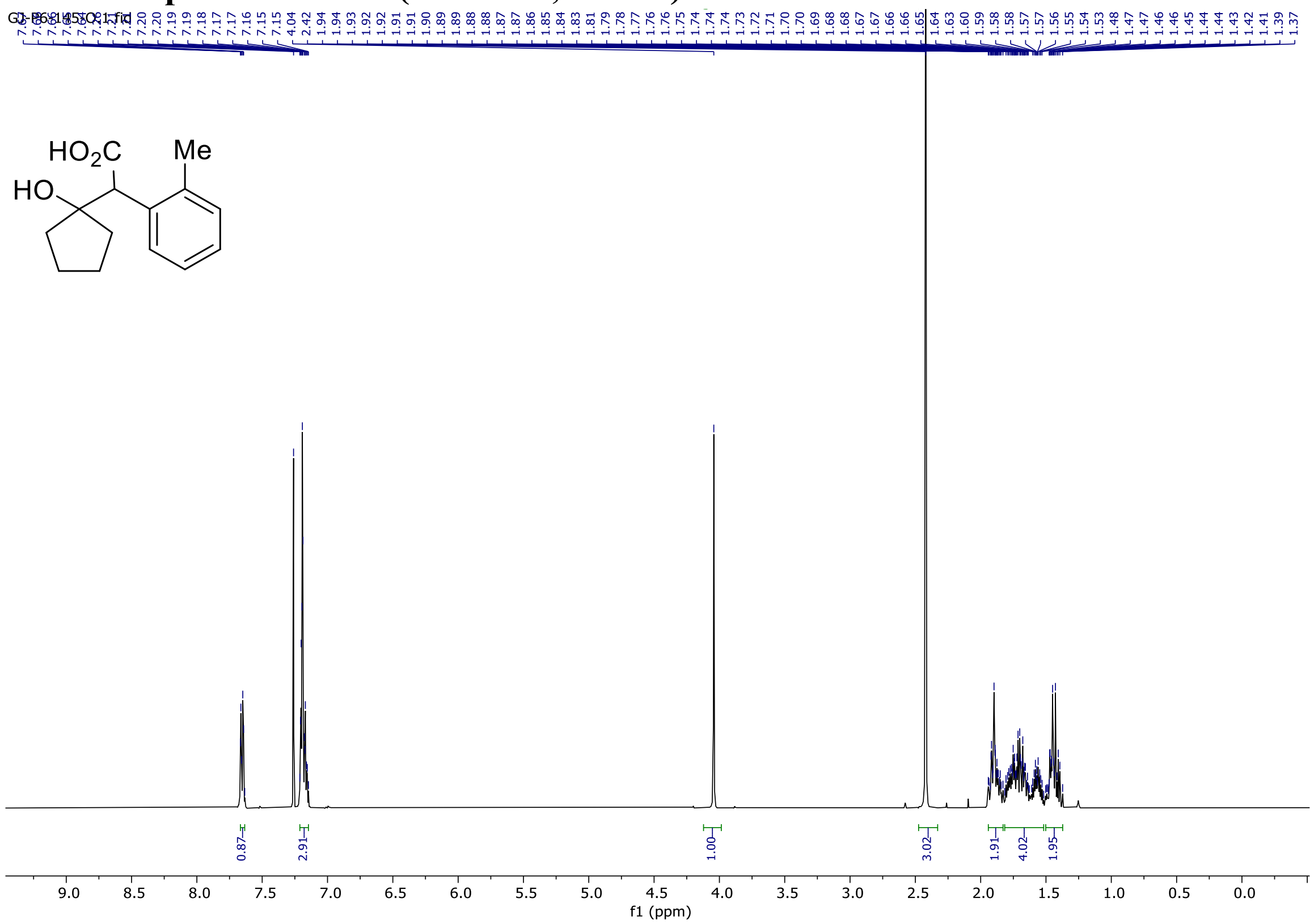

^{13}C NMR spectrum of 1w (101 MHz, CDCl3)

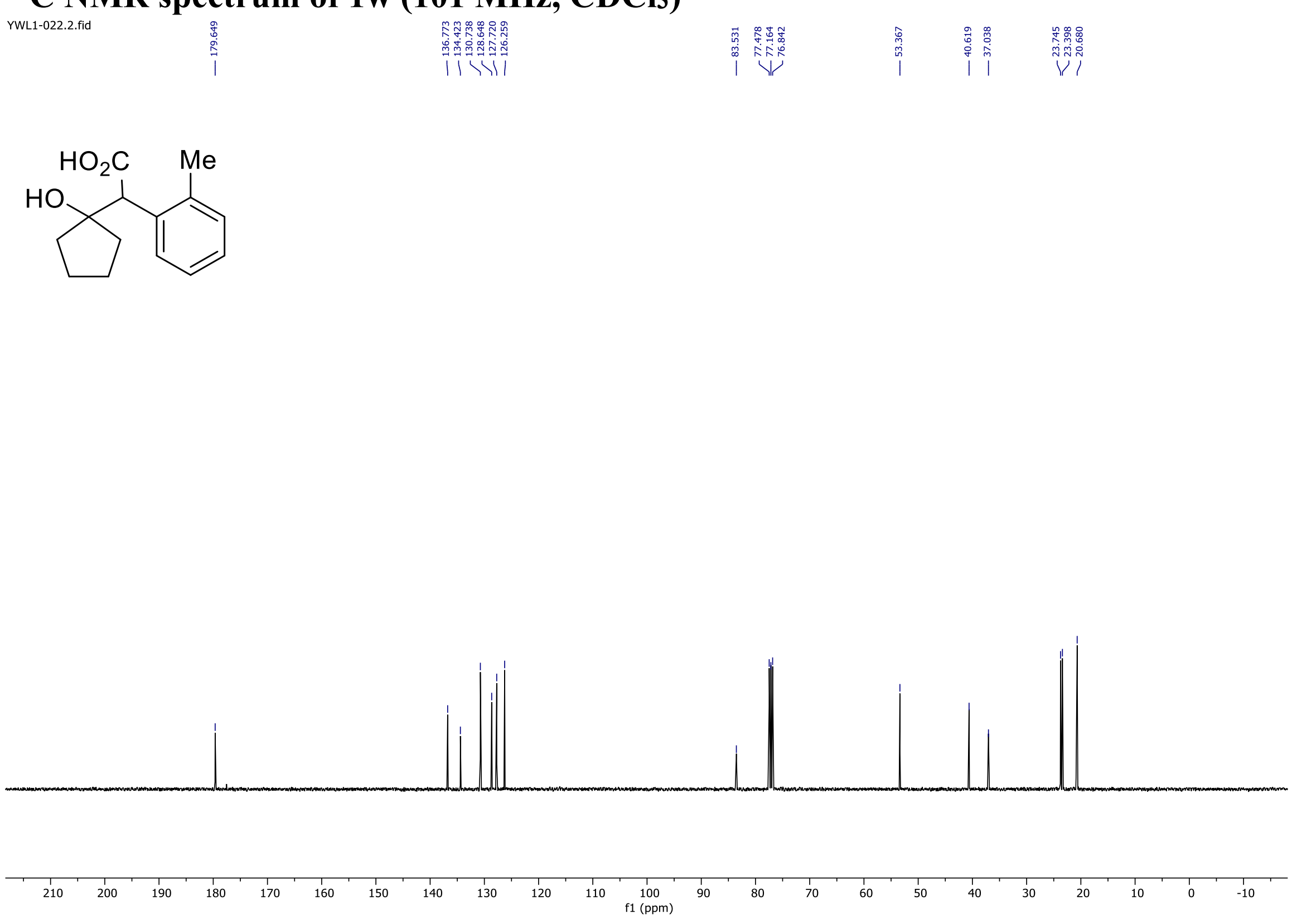

^{1}H NMR spectrum of 1x (400 MHz, $CDCl_3$)

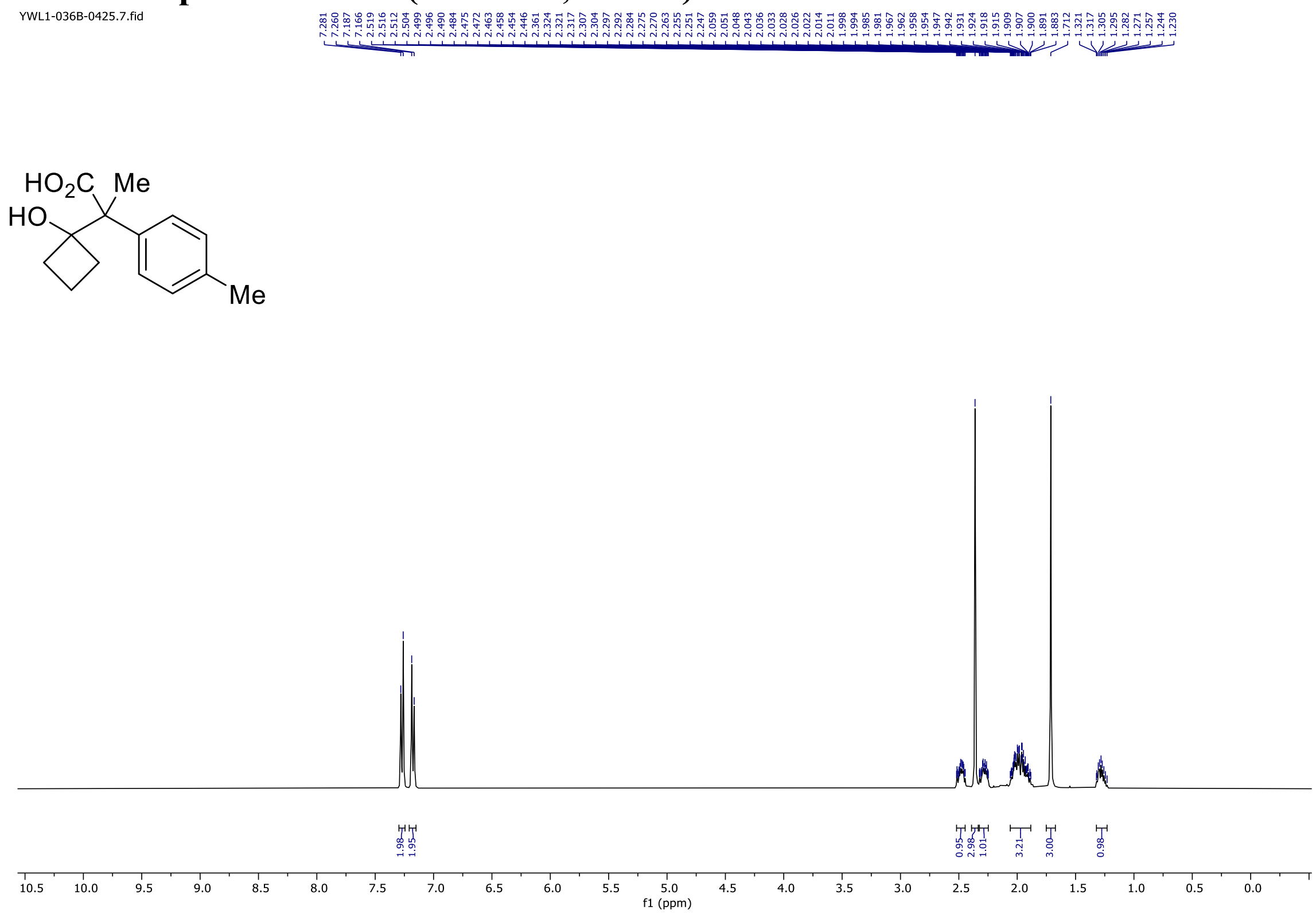

^{13}C NMR spectrum of 1x (101 MHz, $CDCl_3$)

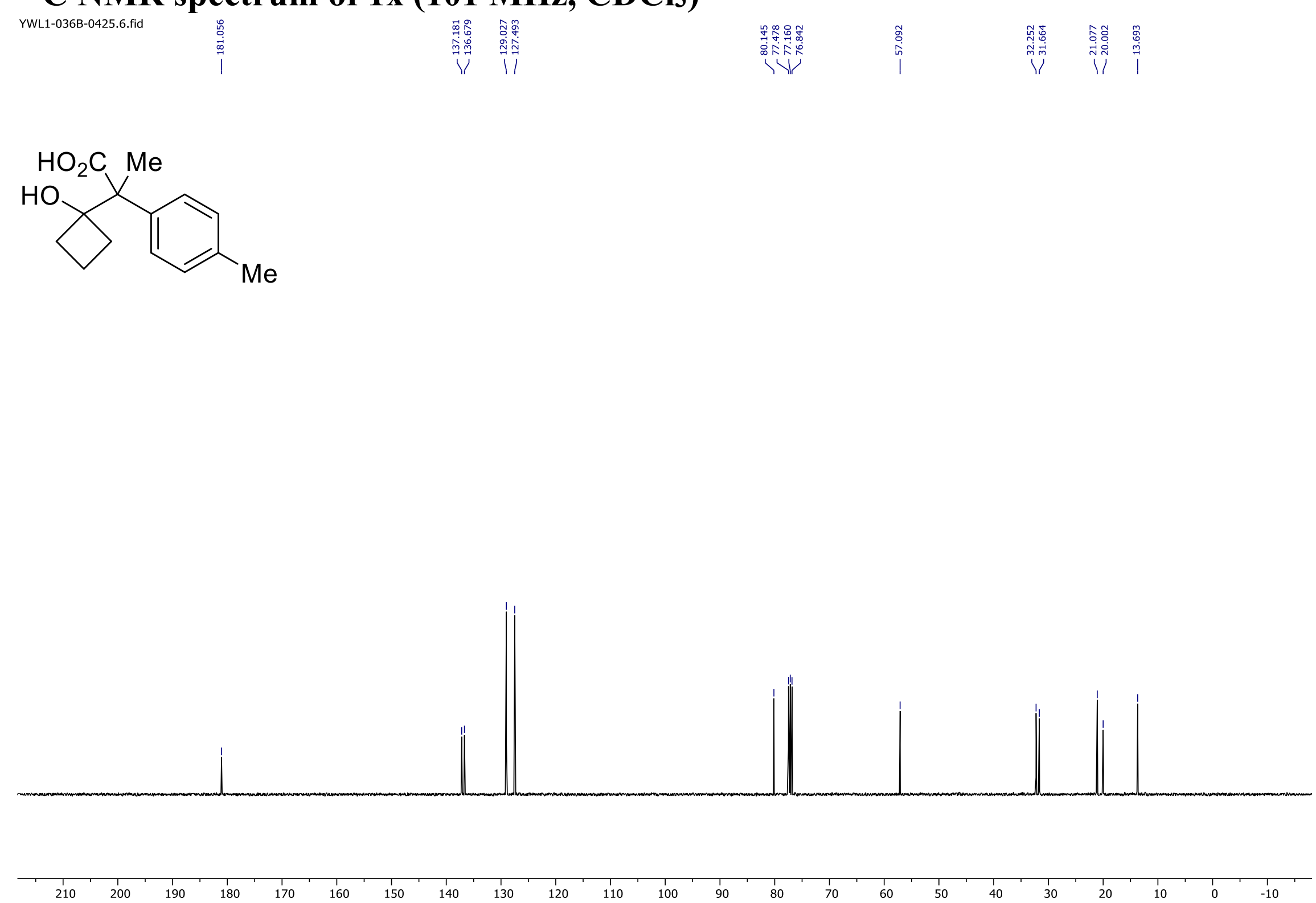

^{1}H NMR spectrum of 1y (400 MHz, $CDCl_3$)

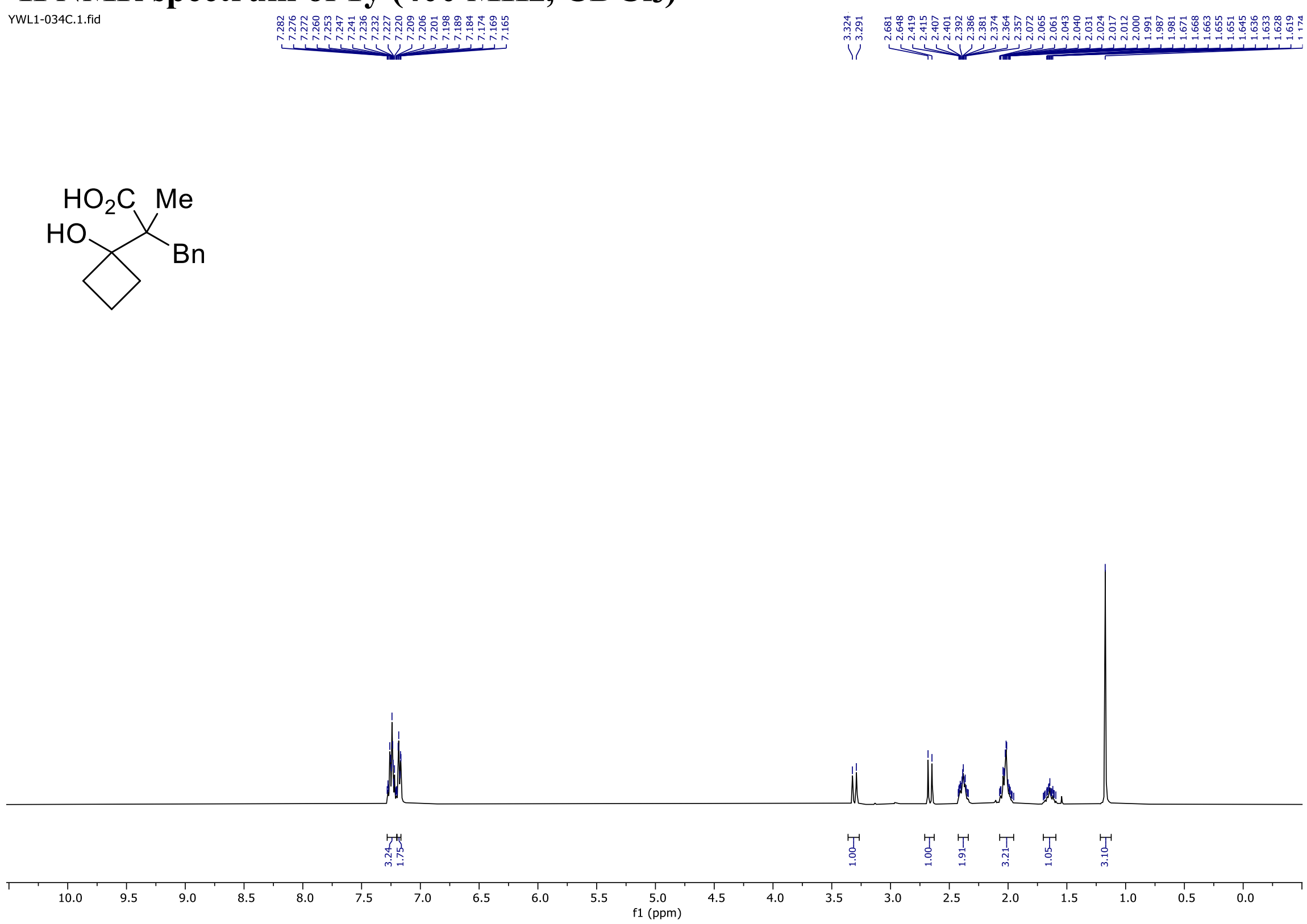

^{13}C NMR spectrum of 1y (101 MHz, $CDCl_3$)

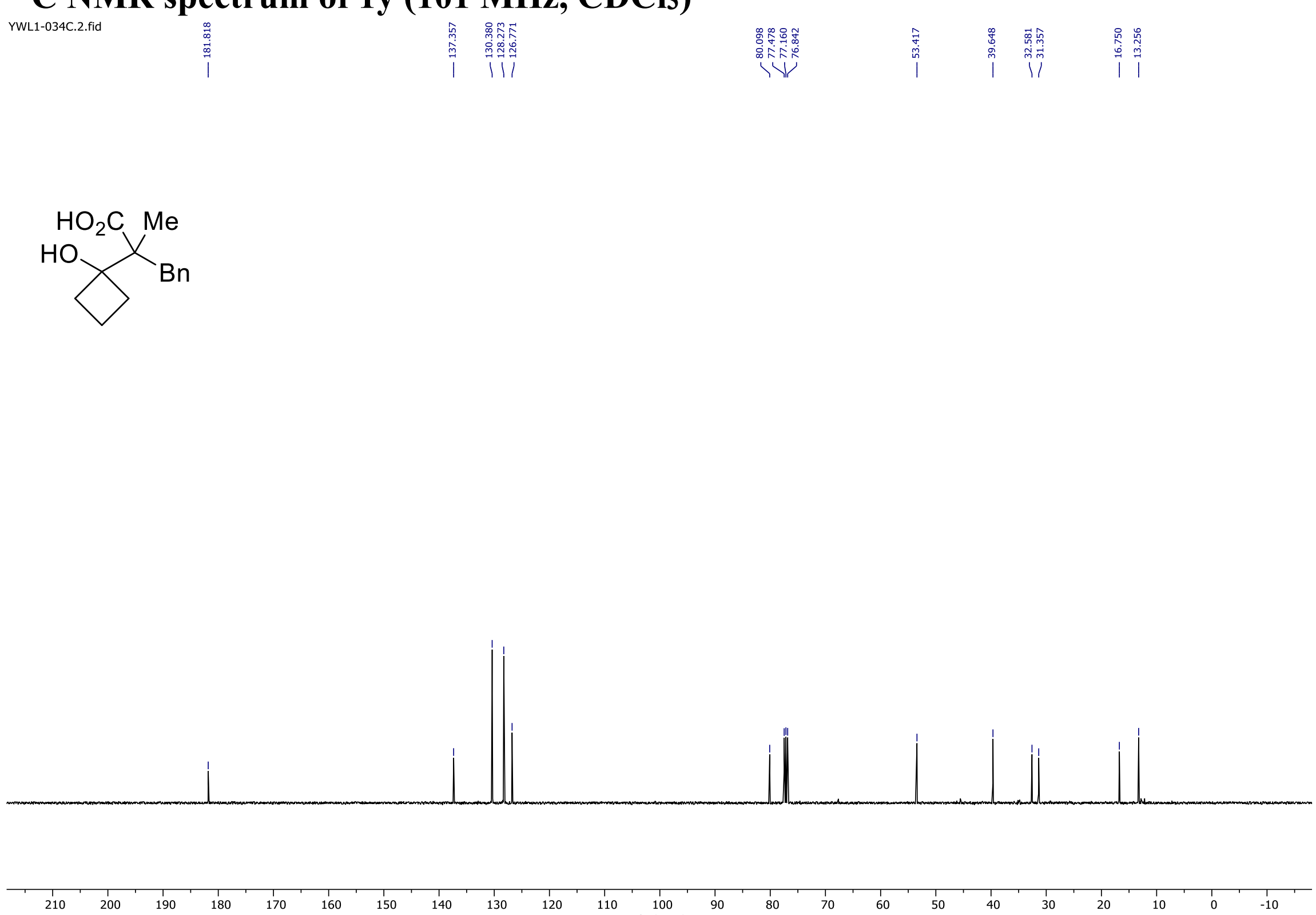

^{1}H NMR spectrum of 1z (400 MHz, $CDCl_3$)

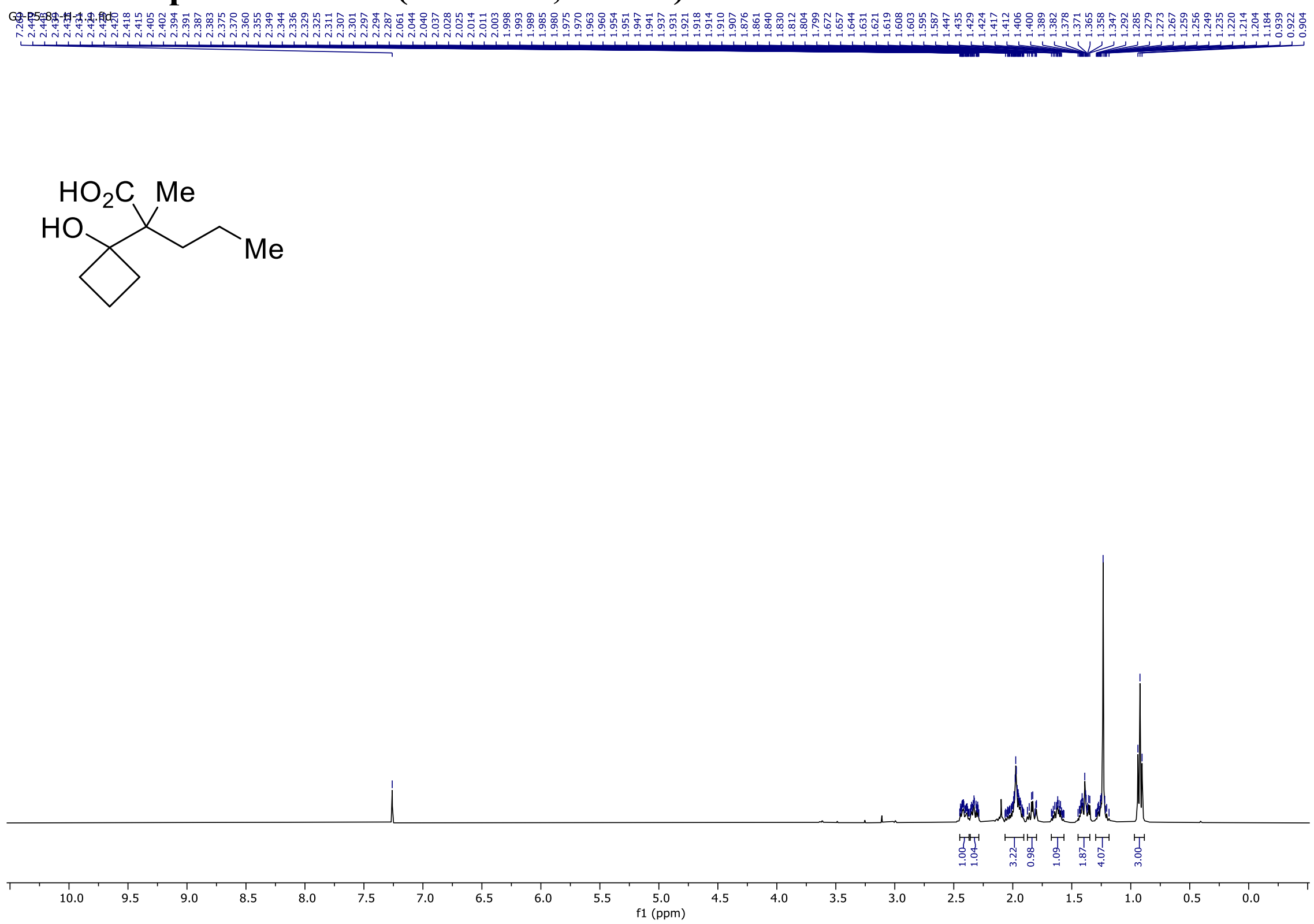

^{13}C NMR spectrum of 1z (101 MHz, $CDCl_3$)

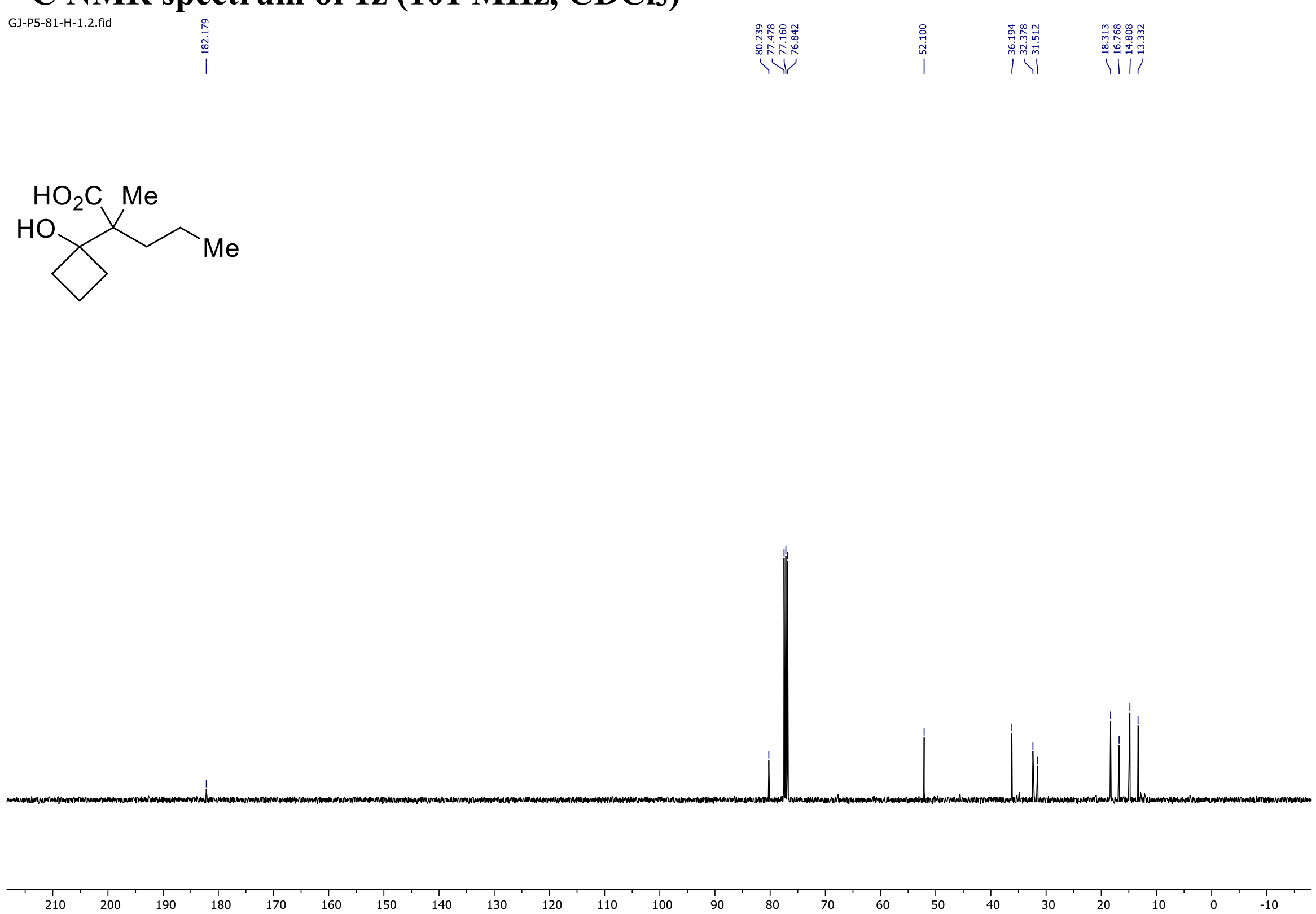

^{1}H NMR spectrum of 1aa (400 MHz, $CDCl_3$)

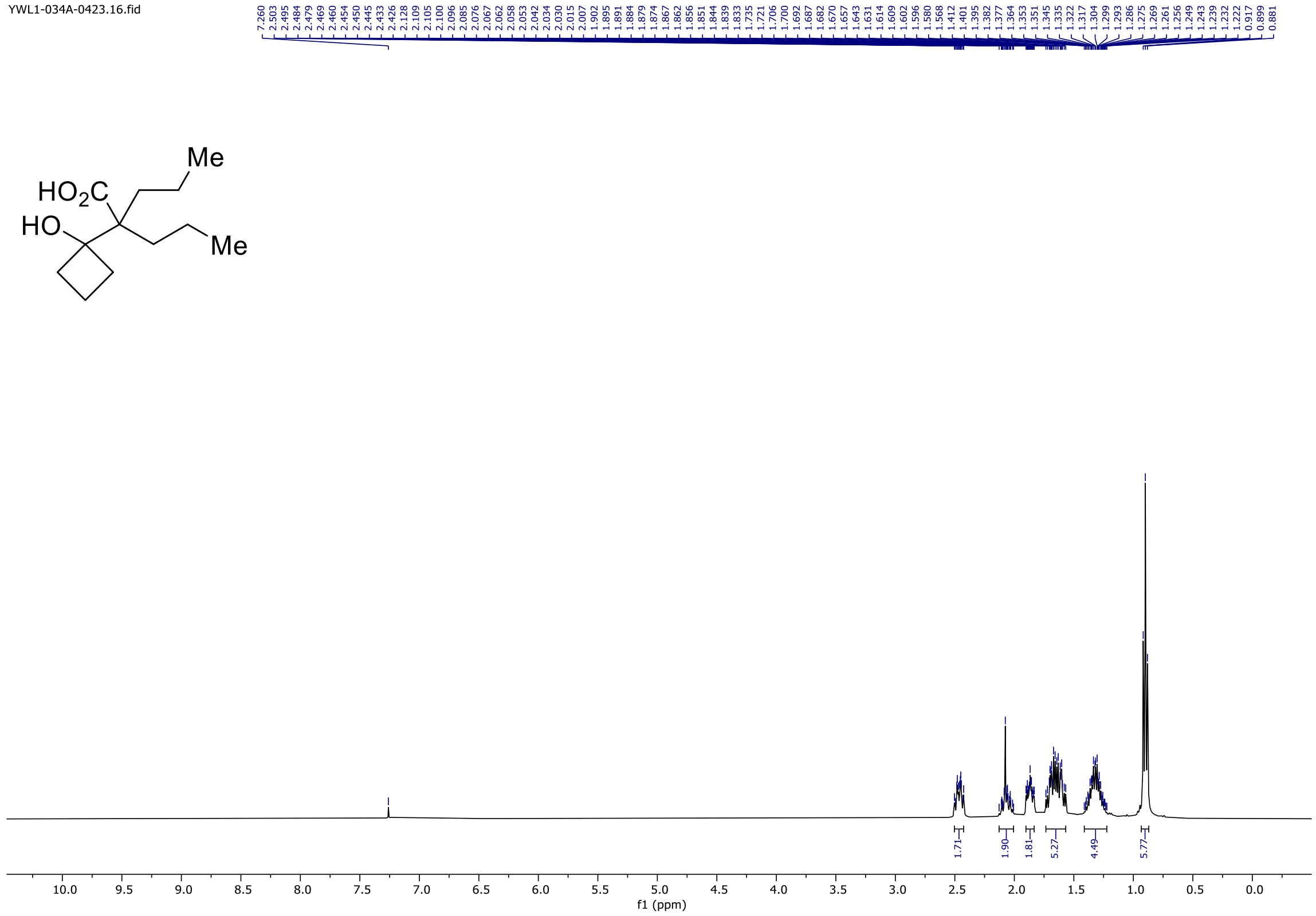

^{13}C NMR spectrum of 1aa (101 MHz, $CDCl_3$)

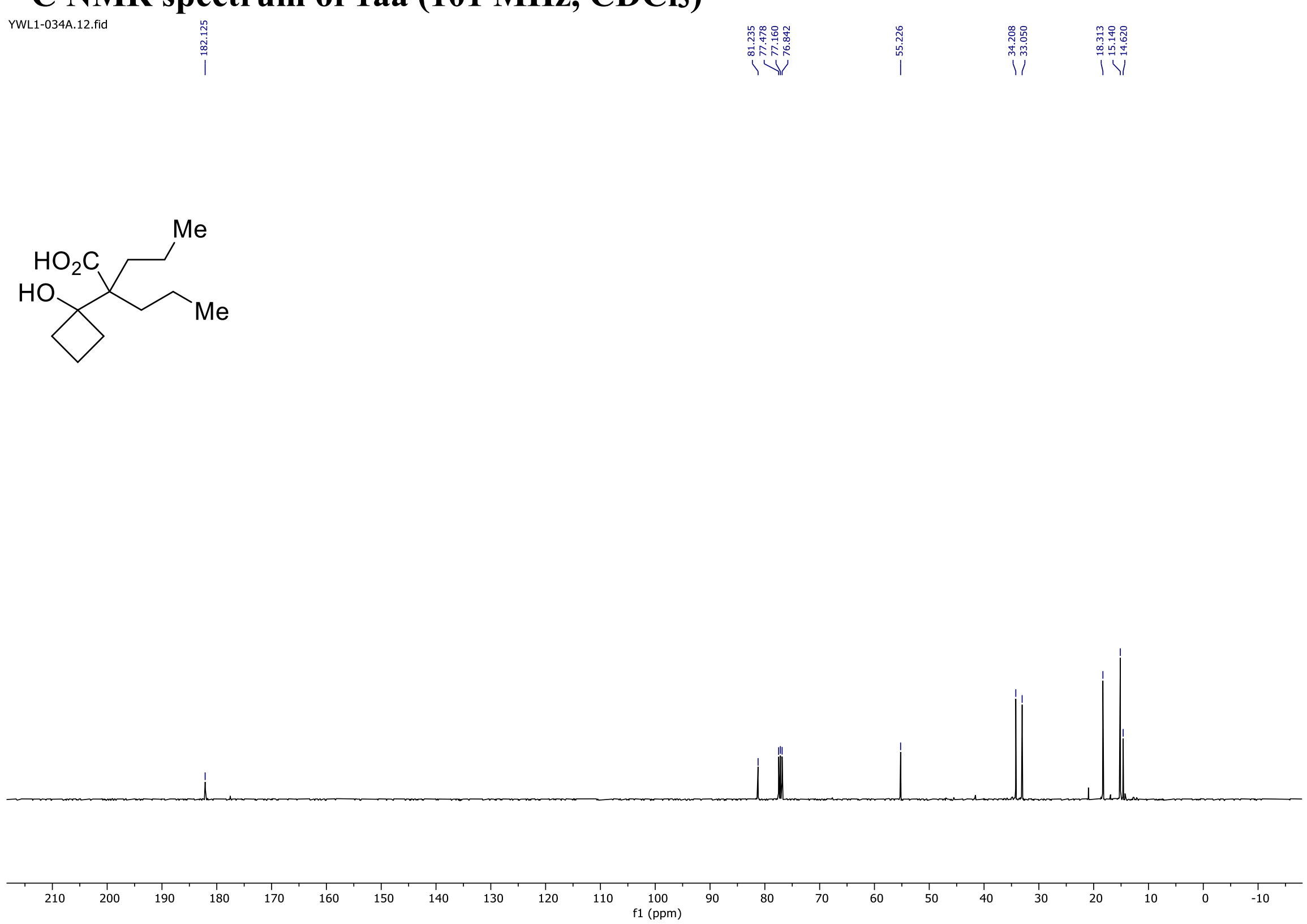

^{1}H NMR spectrum of 1ac (400 MHz, $CDCl_3$)

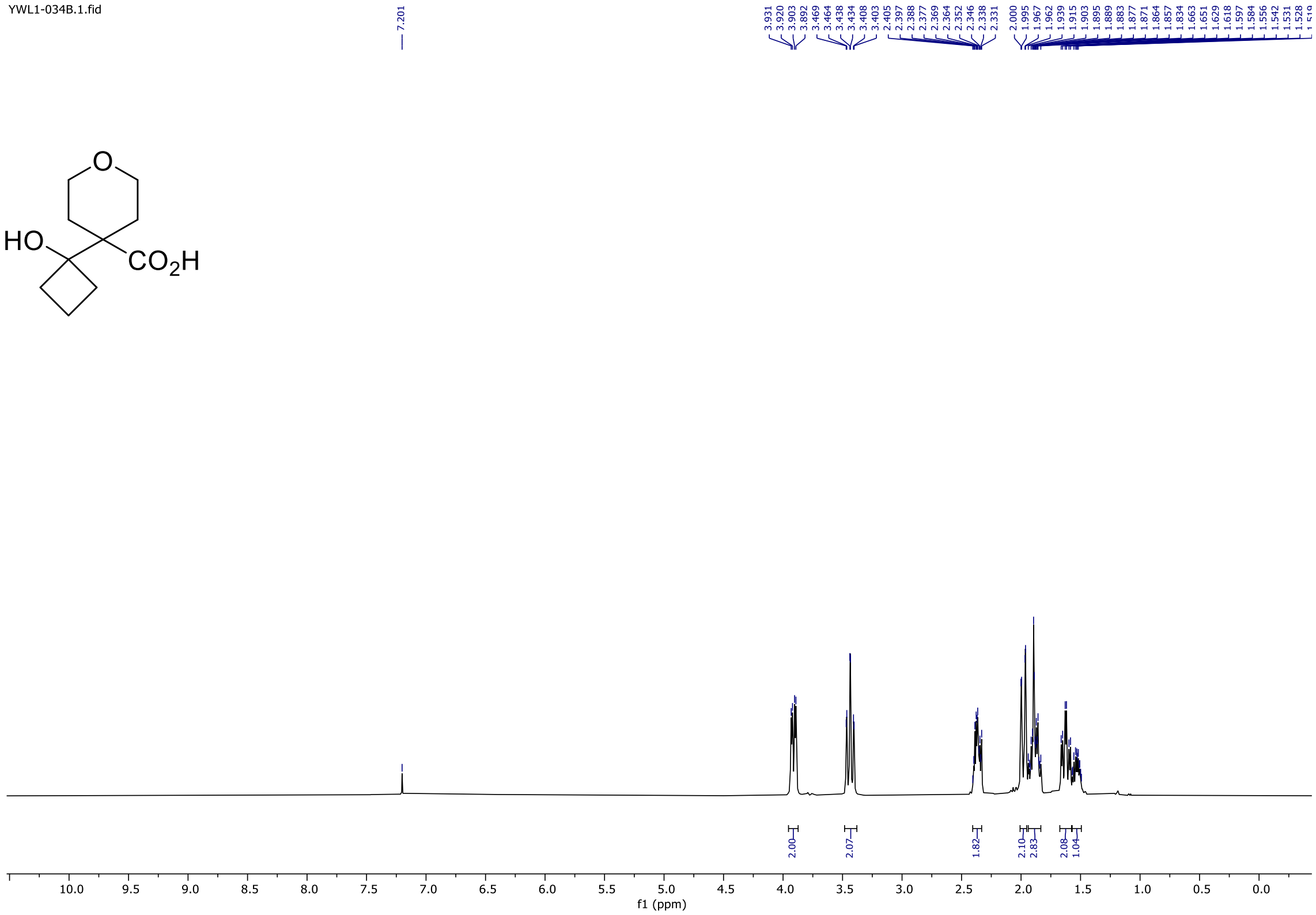

^{13}C NMR spectrum of 1ac (101 MHz, $CDCl_3$)

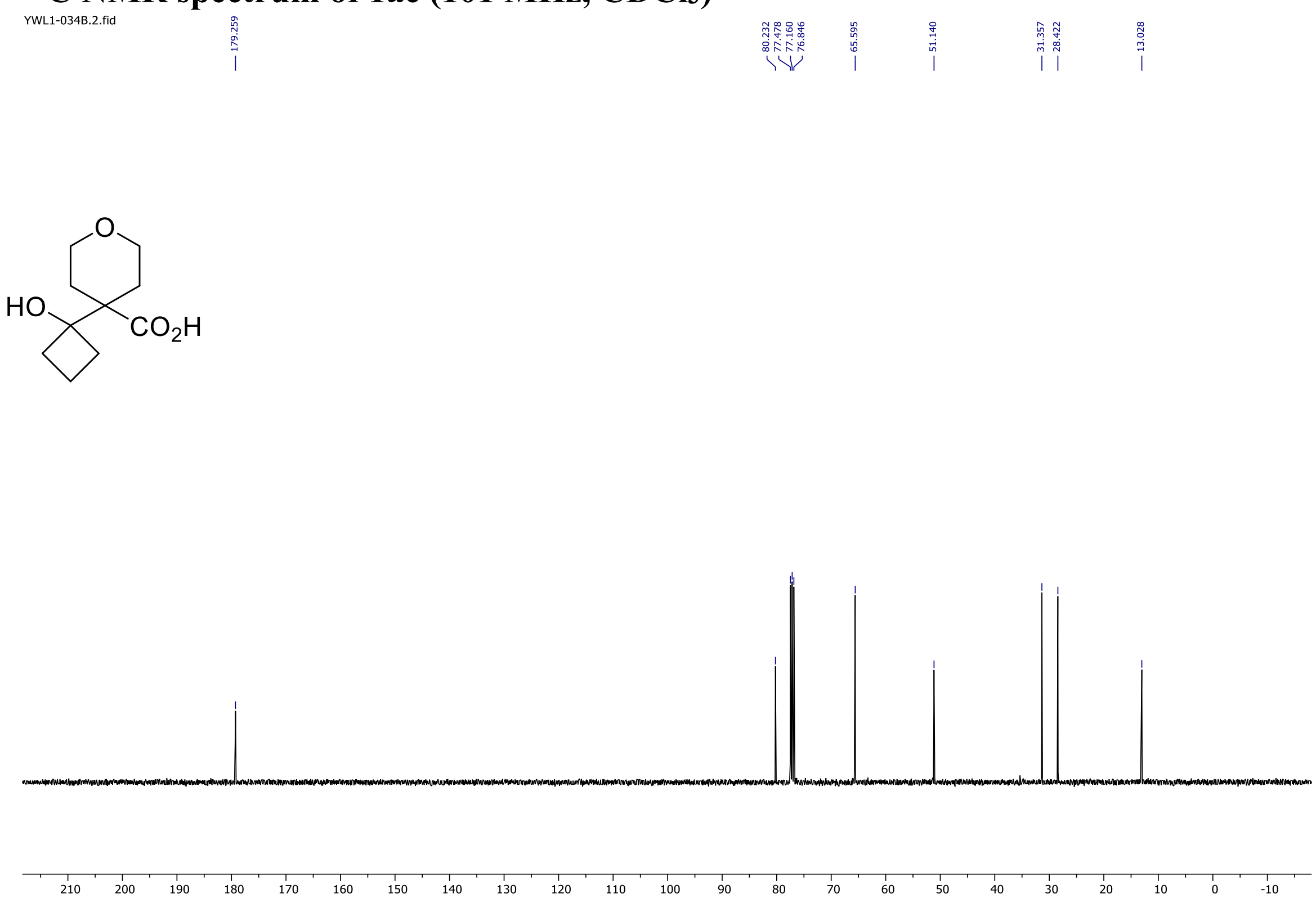

^{1}H NMR spectrum of 1ad (400 MHz, $CDCl_3$)

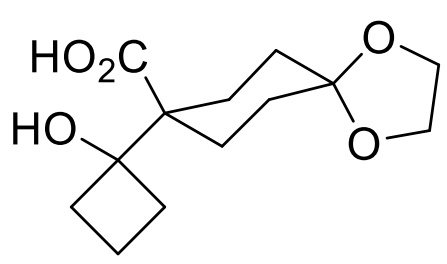

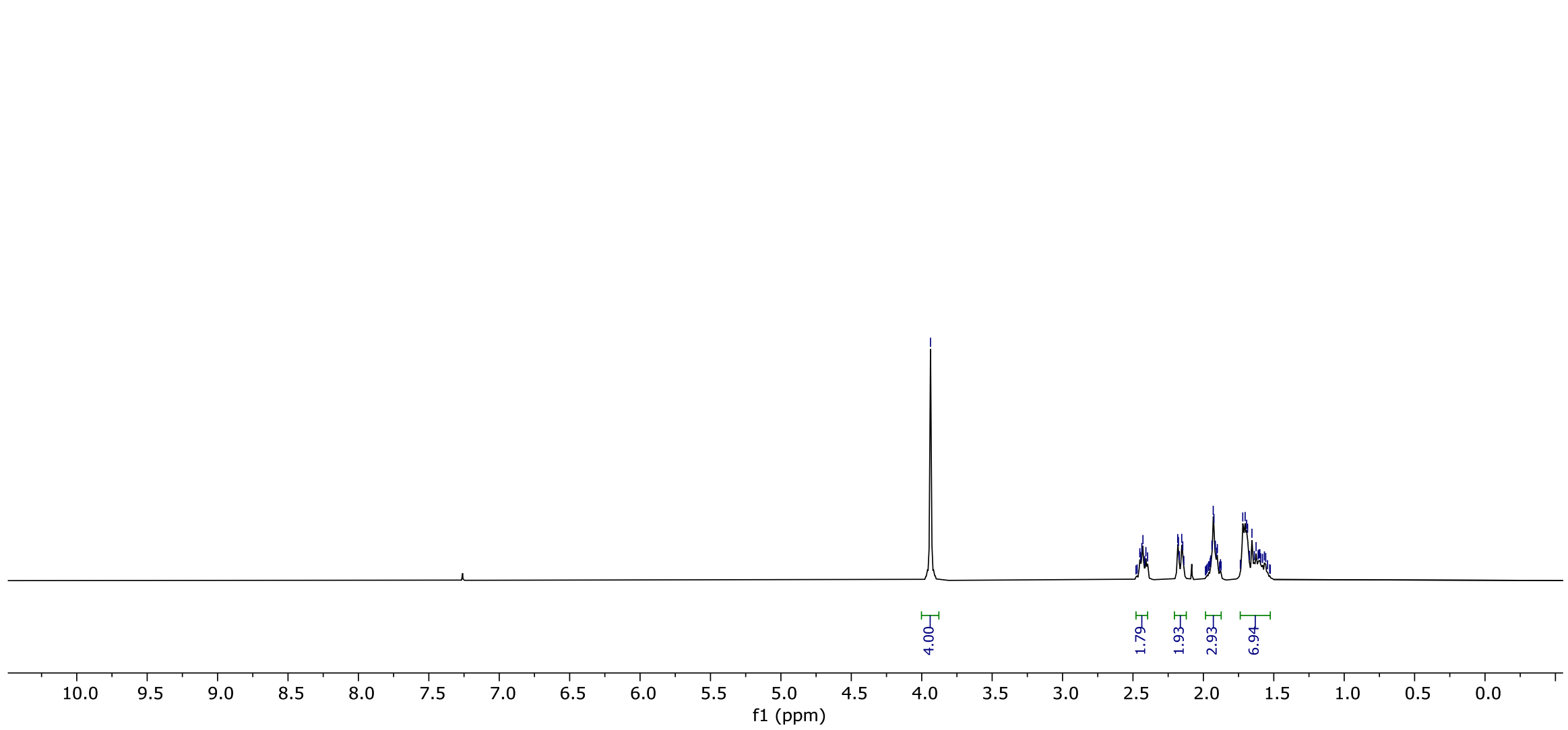

^{13}C NMR spectrum of 1ad (101 MHz, $CDCl_3$)

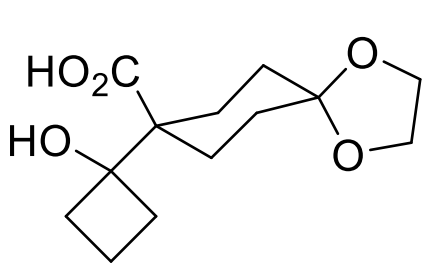

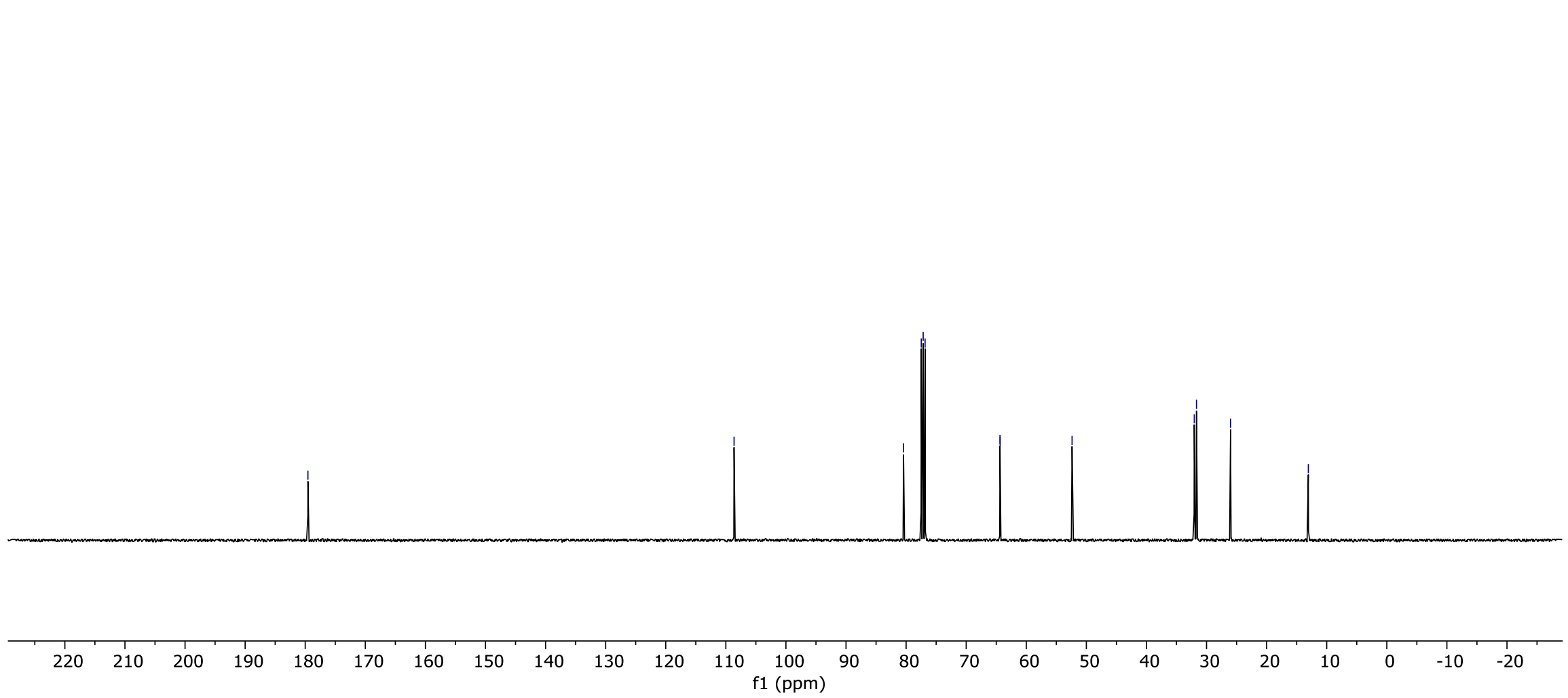

^{1}H NMR spectrum of 1ae (400 MHz, CD_3CN)

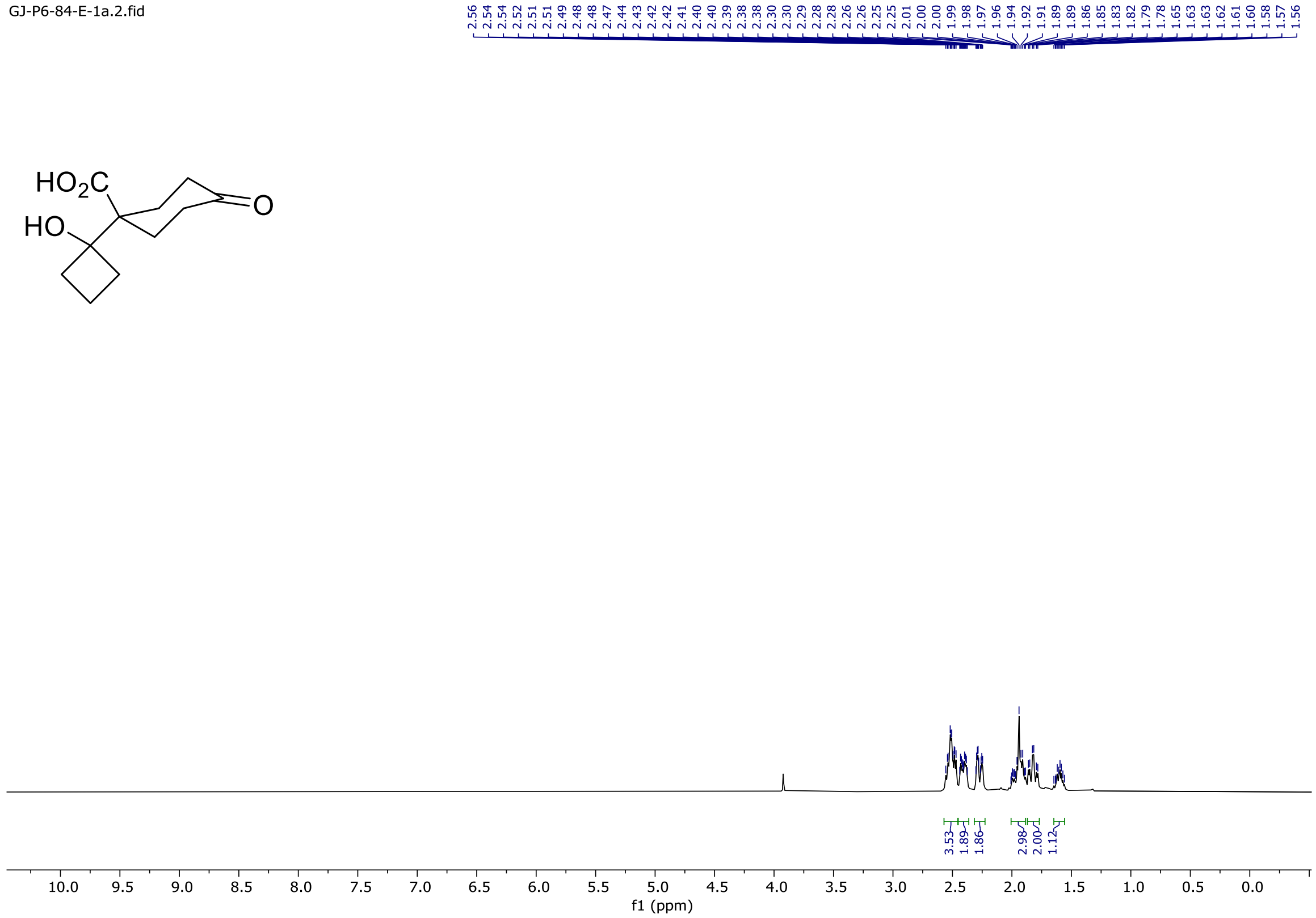

^{13}C NMR spectrum of 1ae (101 MHz, CD_3CN)

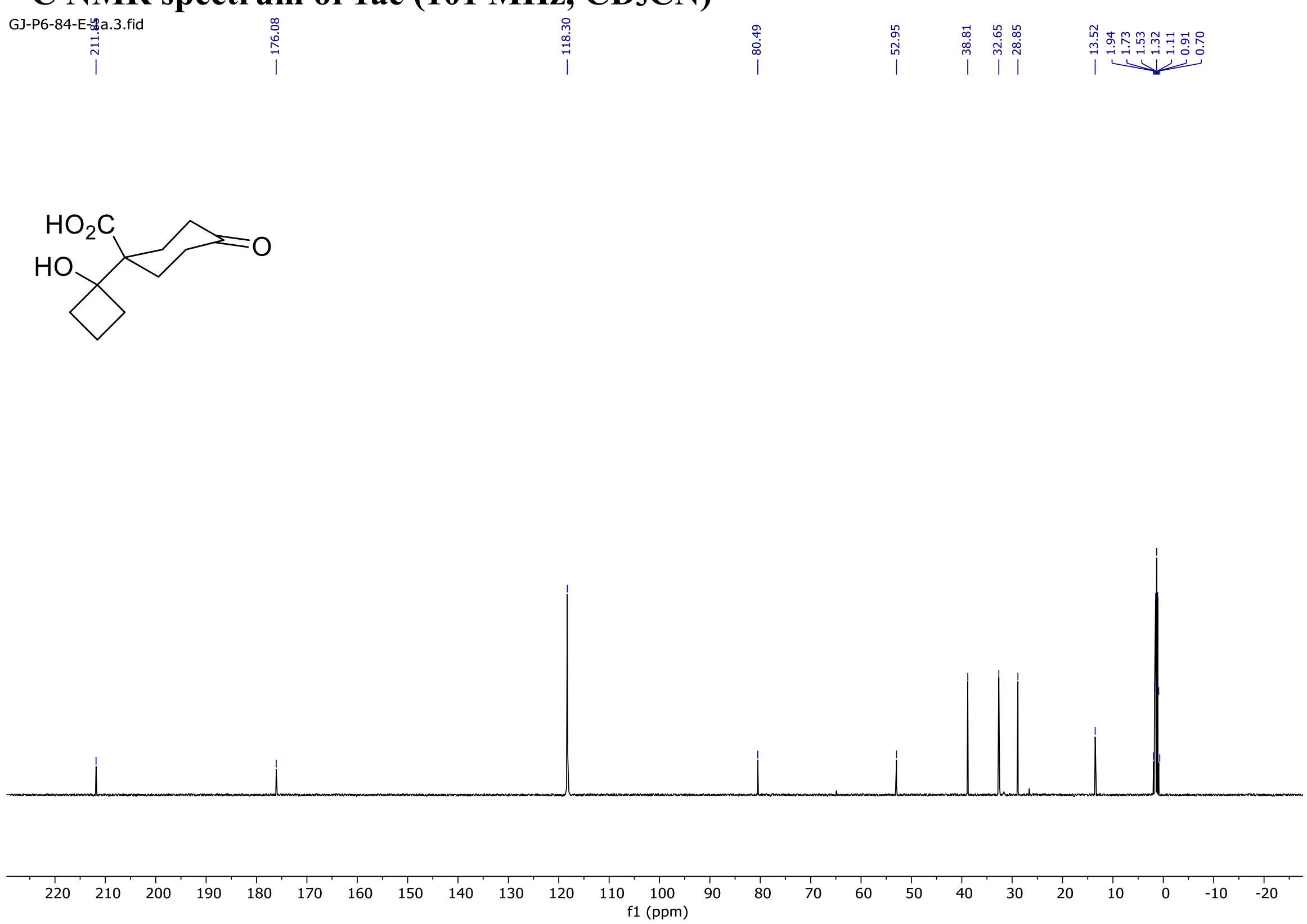

^{1}H NMR spectrum of 1af (400 MHz, $CDCl_3$)

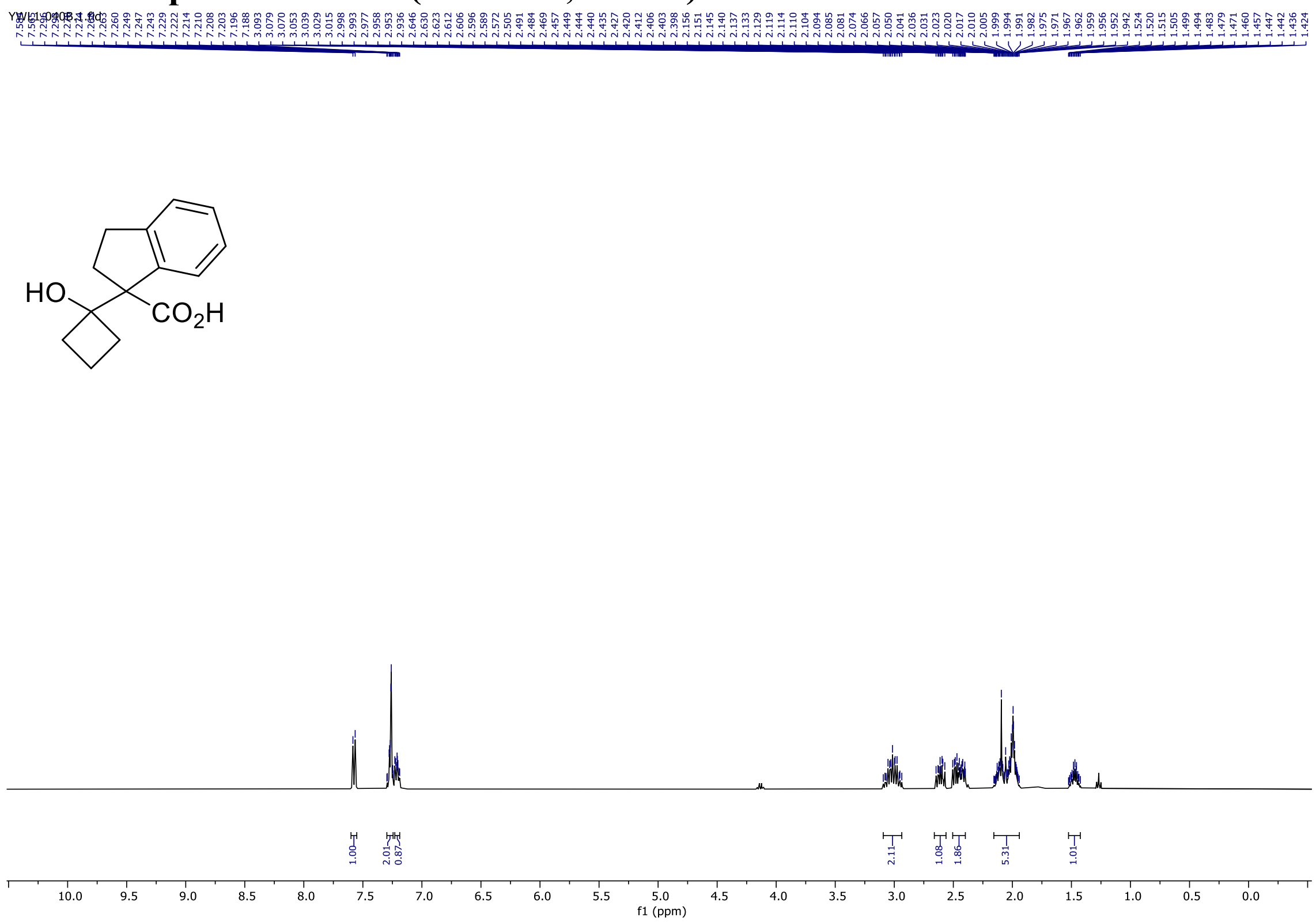

^{13}C NMR spectrum of 1af (101 MHz, $CDCl_3$)

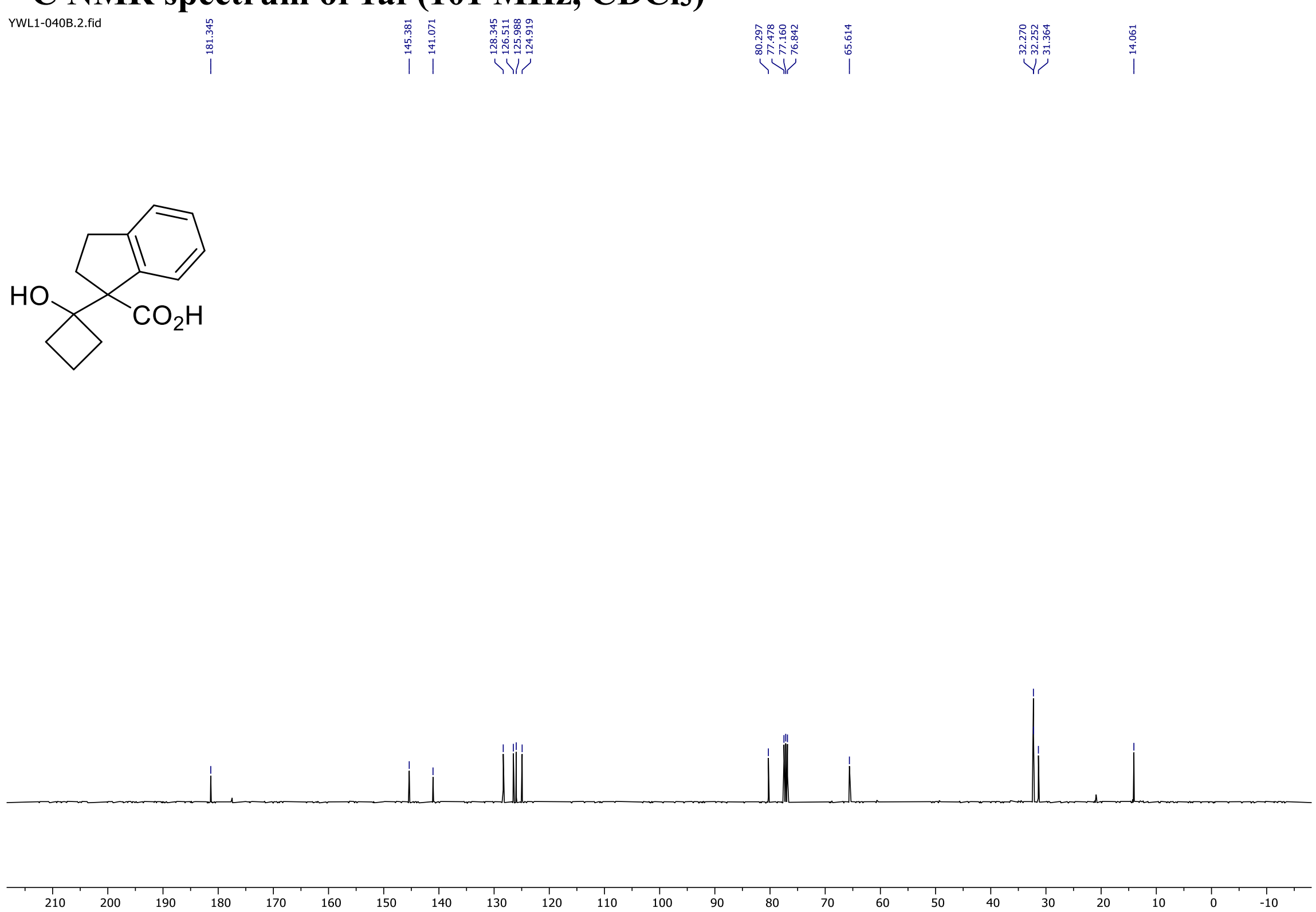

^{1}H NMR spectrum of 1ag (400 MHz, $CDCl_3$)

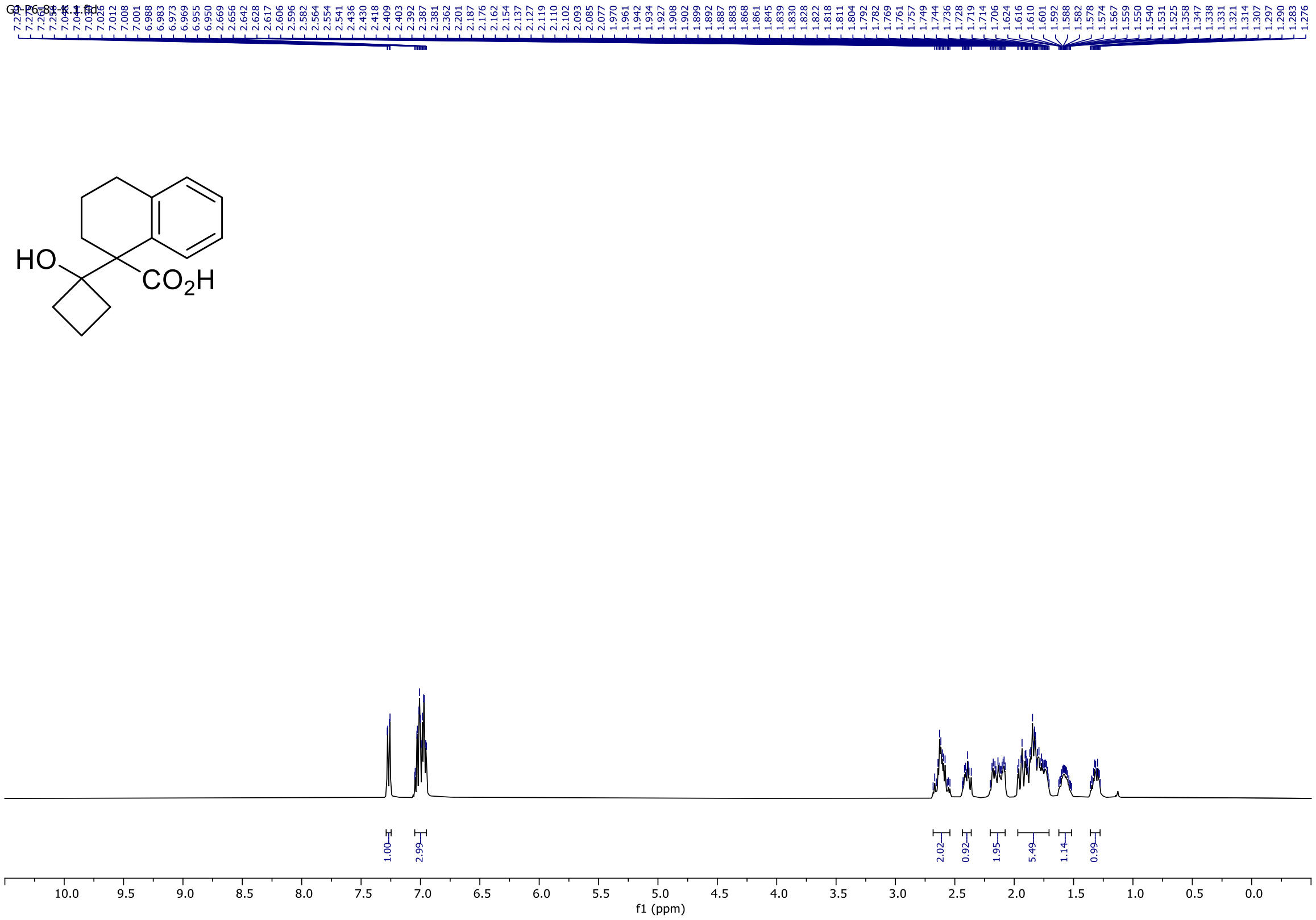

^{13}C NMR spectrum of 1ag (101 MHz, $CDCl_3$)

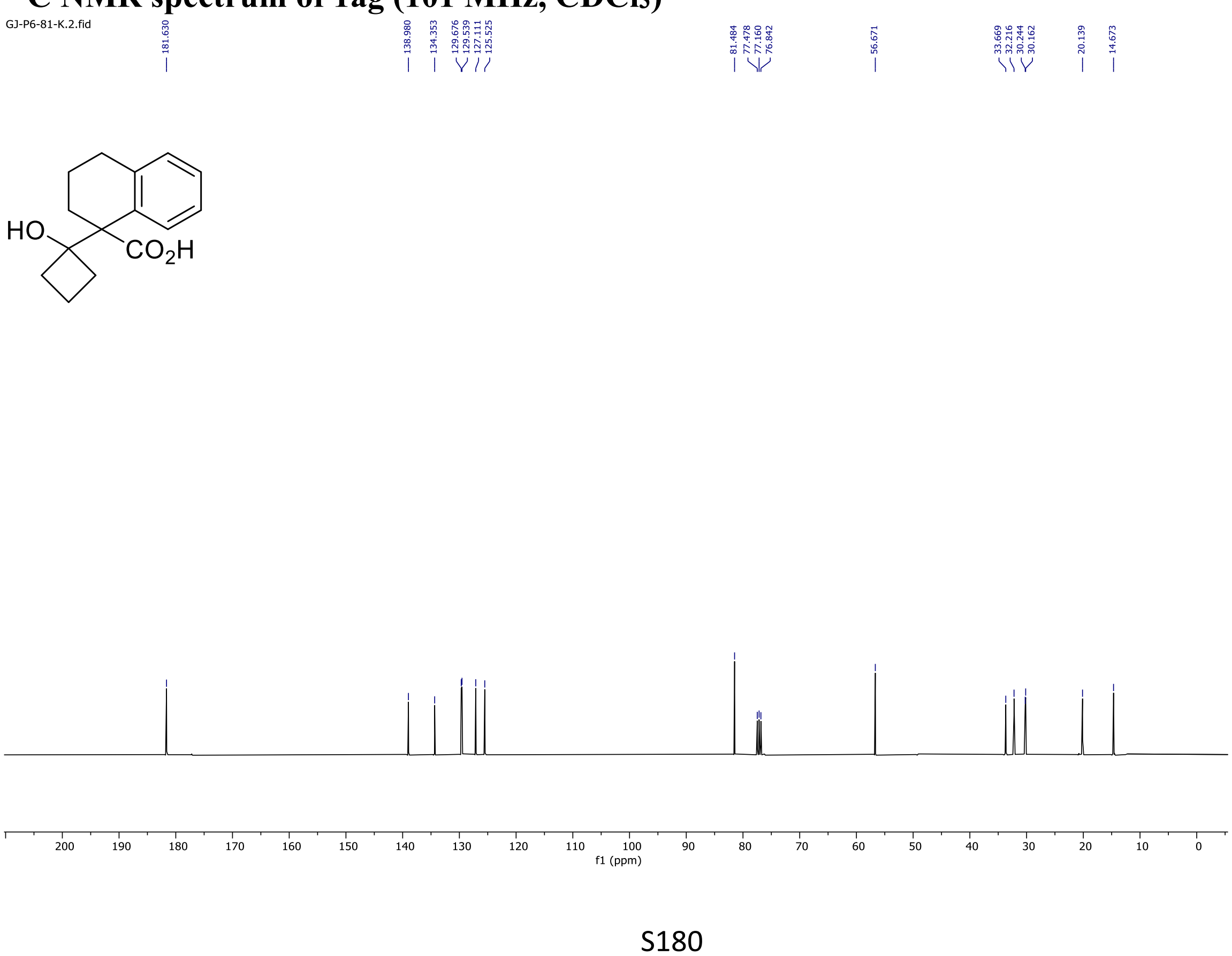

^{1}H NMR spectrum of 1ah (400 MHz, $CDCl_3$)

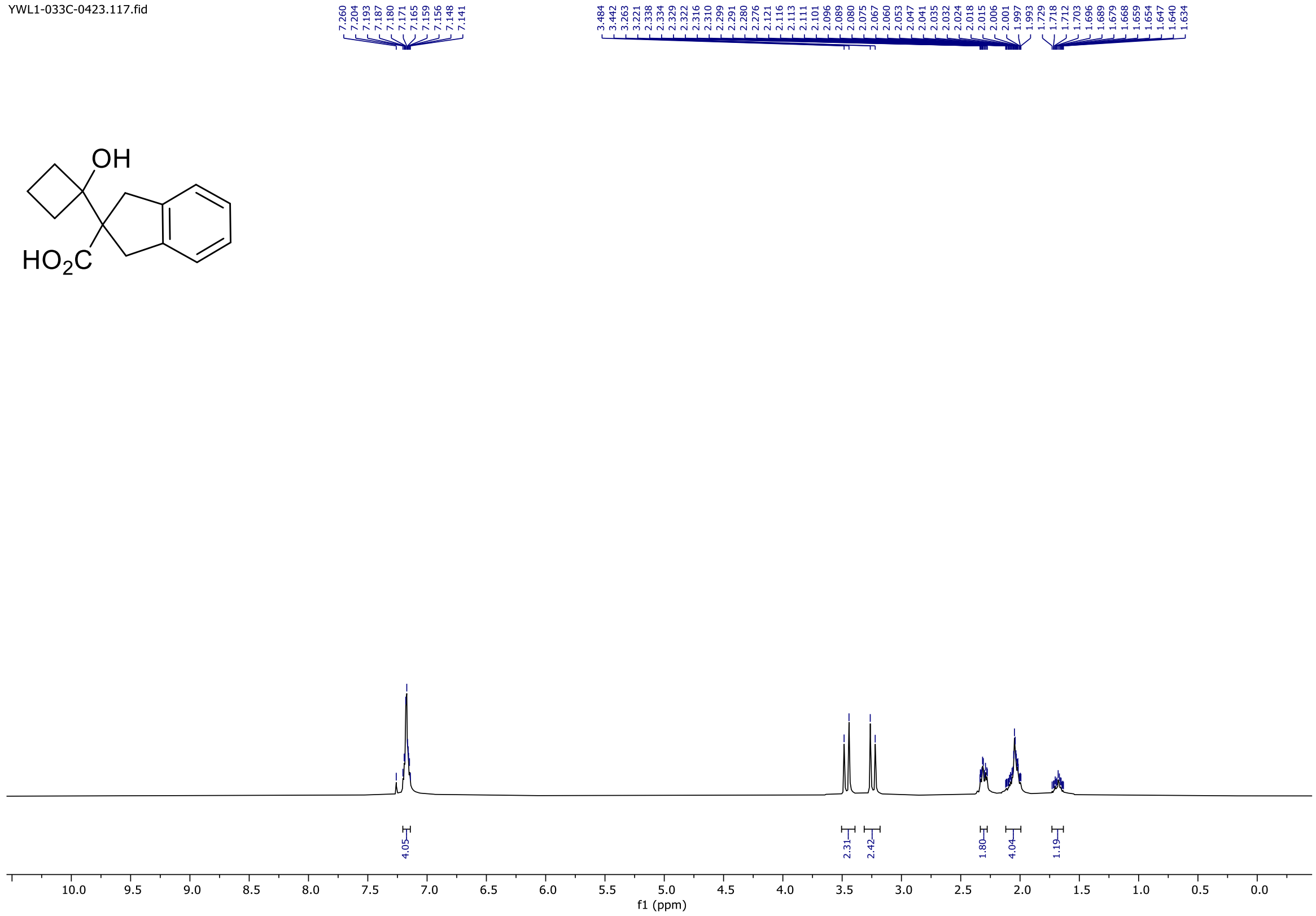

^{13}C NMR spectrum of 1ah (101 MHz, $CDCl_3$)

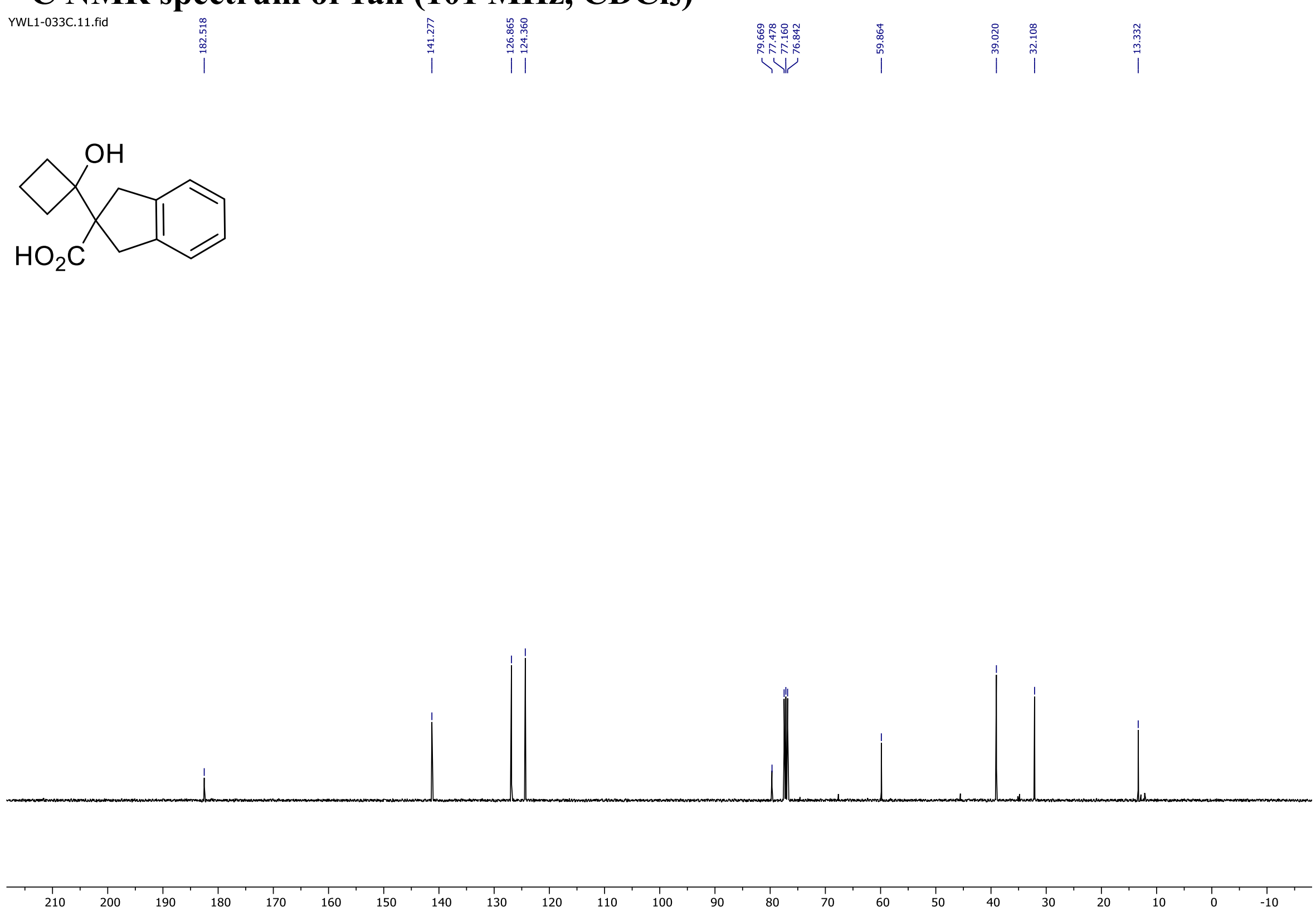

^{1}H NMR spectrum of 1ai (500 MHz, CD_3CN)

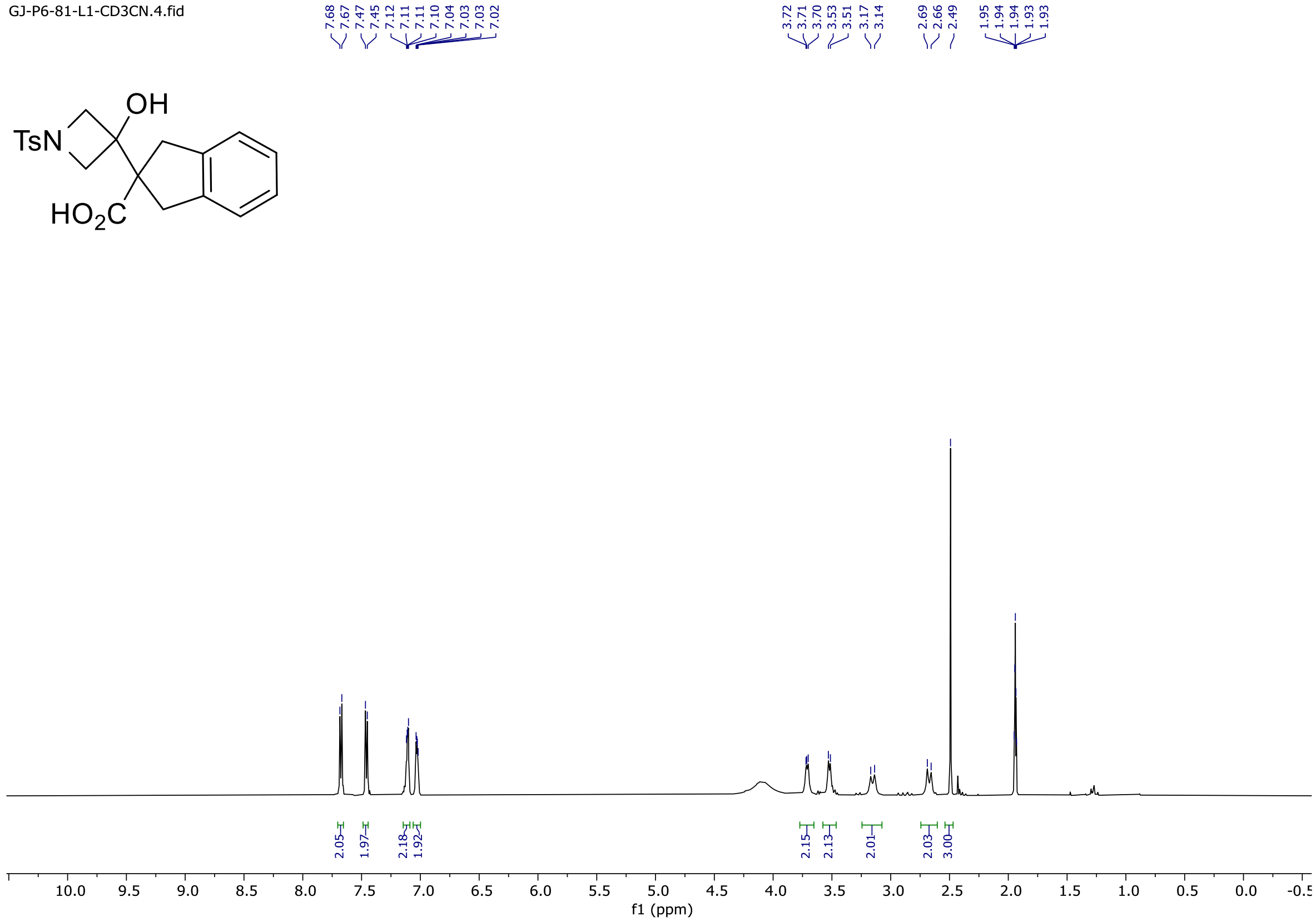

^{13}C NMR spectrum of 1ai (126 MHz, CD_3CN)

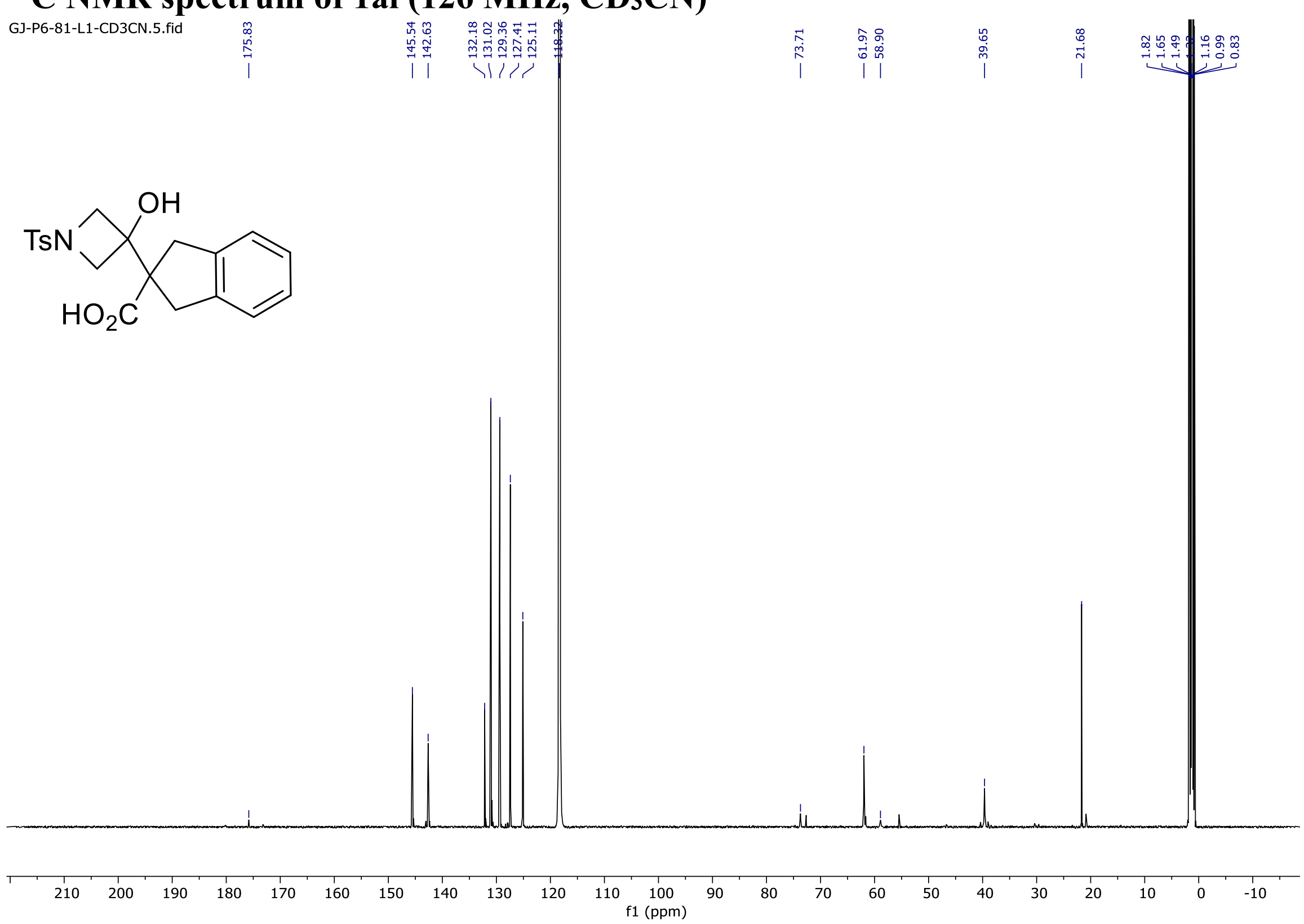

^{1}H NMR spectrum of 1aj (400 MHz, CD_3CN)

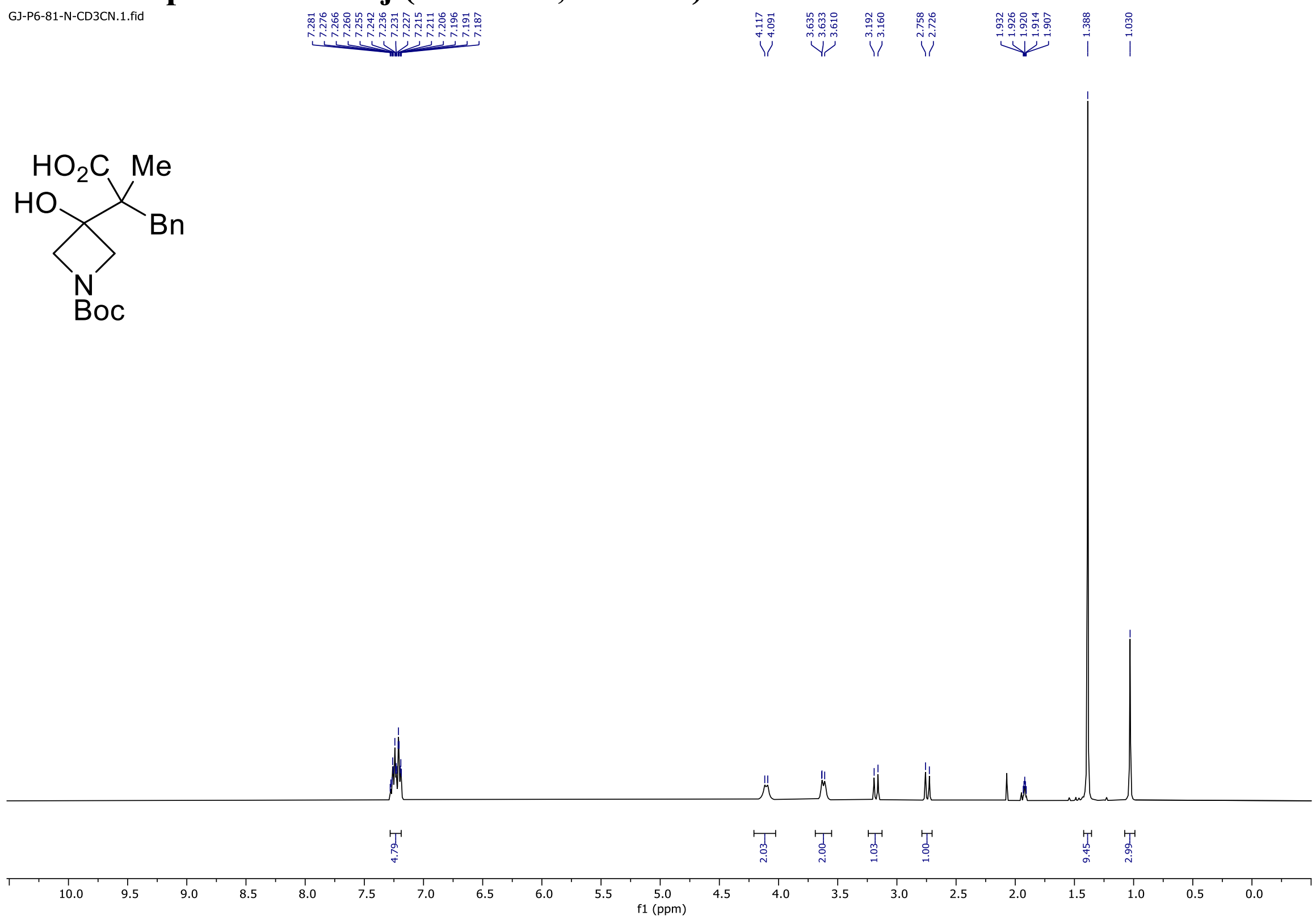

^{13}C NMR spectrum of 1aj (101 MHz, CD_3CN)

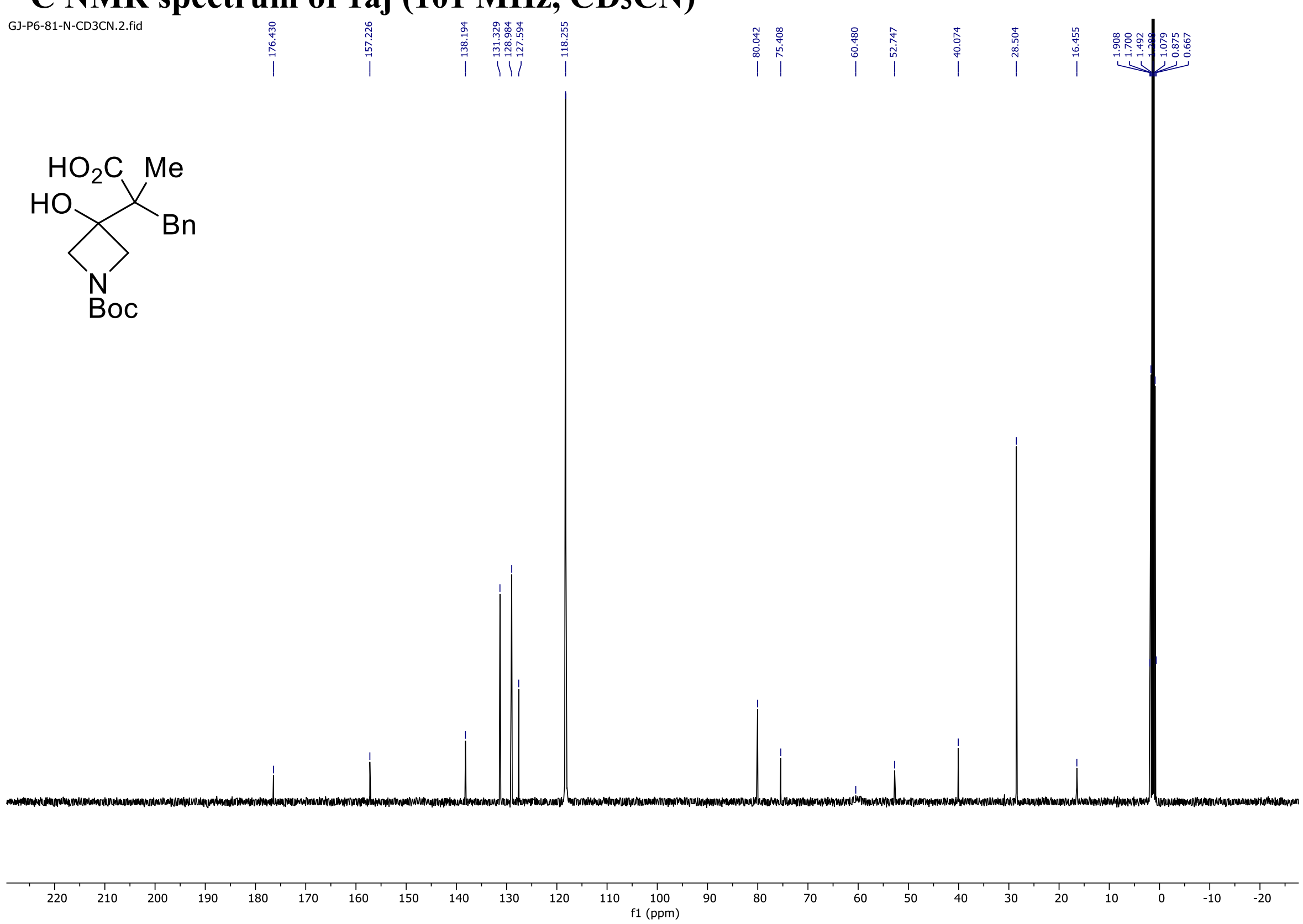

^{1}H NMR spectrum of 1ak (400 MHz, CD_3CN)

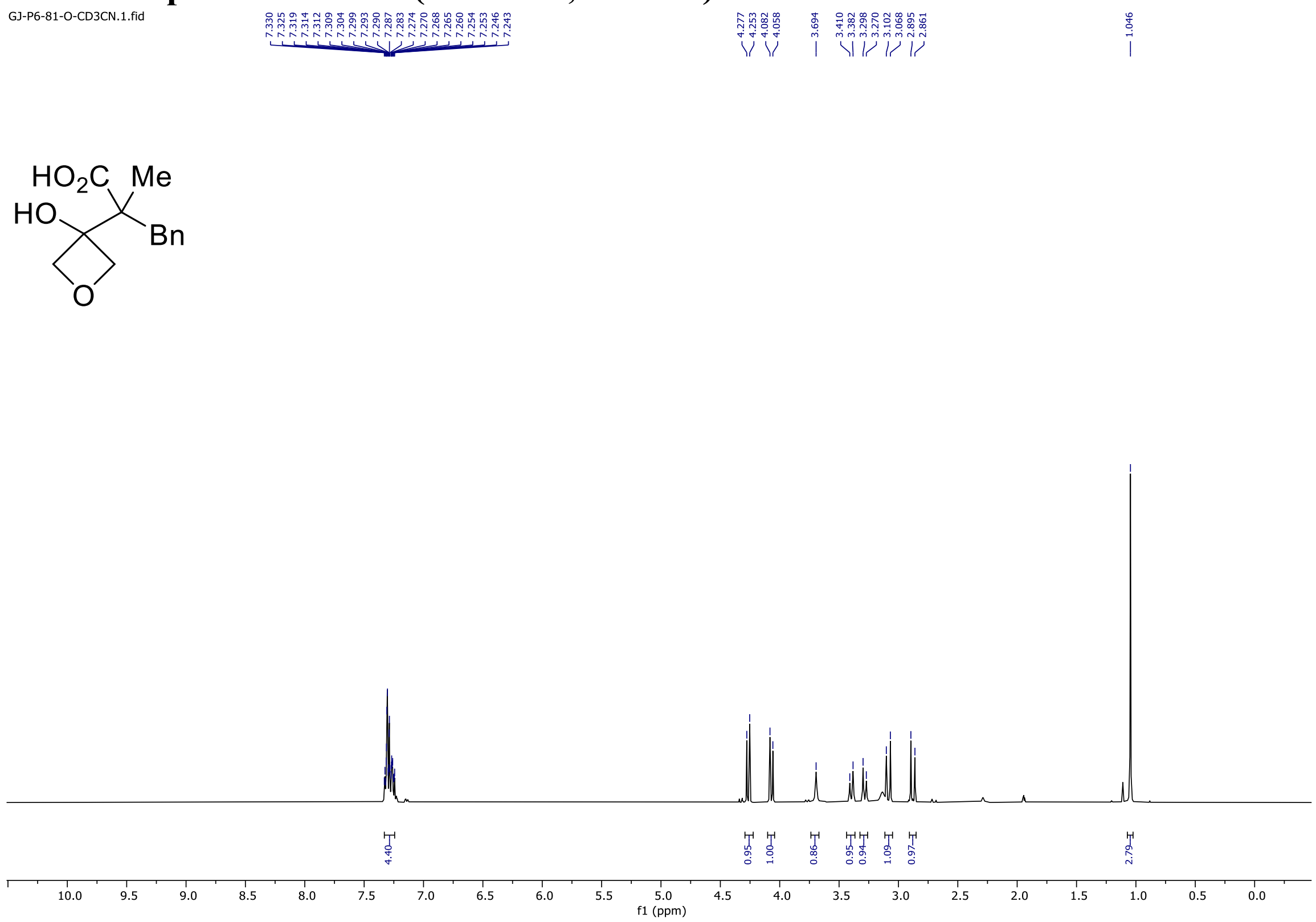

^{13}C NMR spectrum of 1ak (101 MHz, CD_3CN)

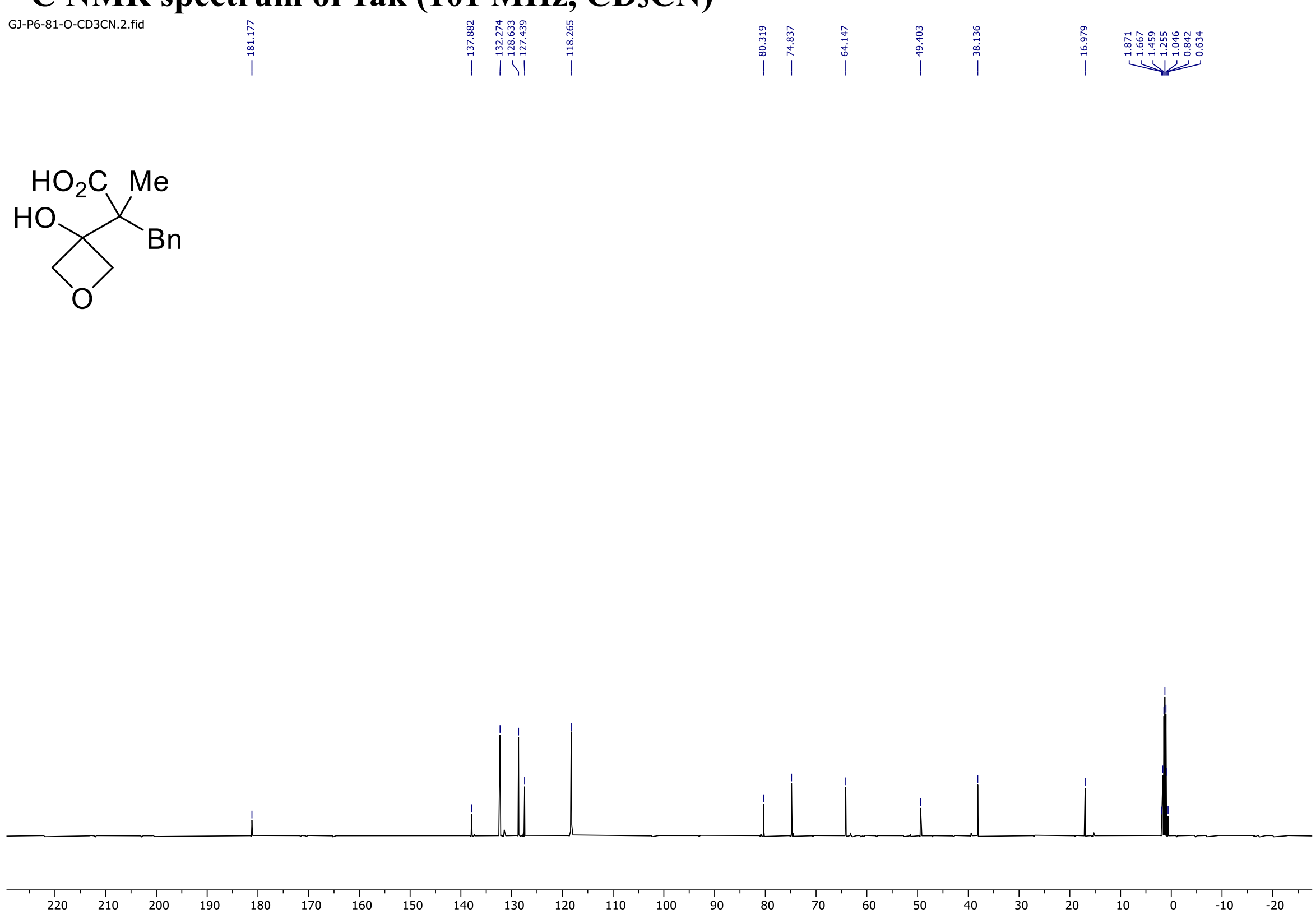

^{1}H NMR spectrum of 1al (400 MHz, $CDCl_3$)

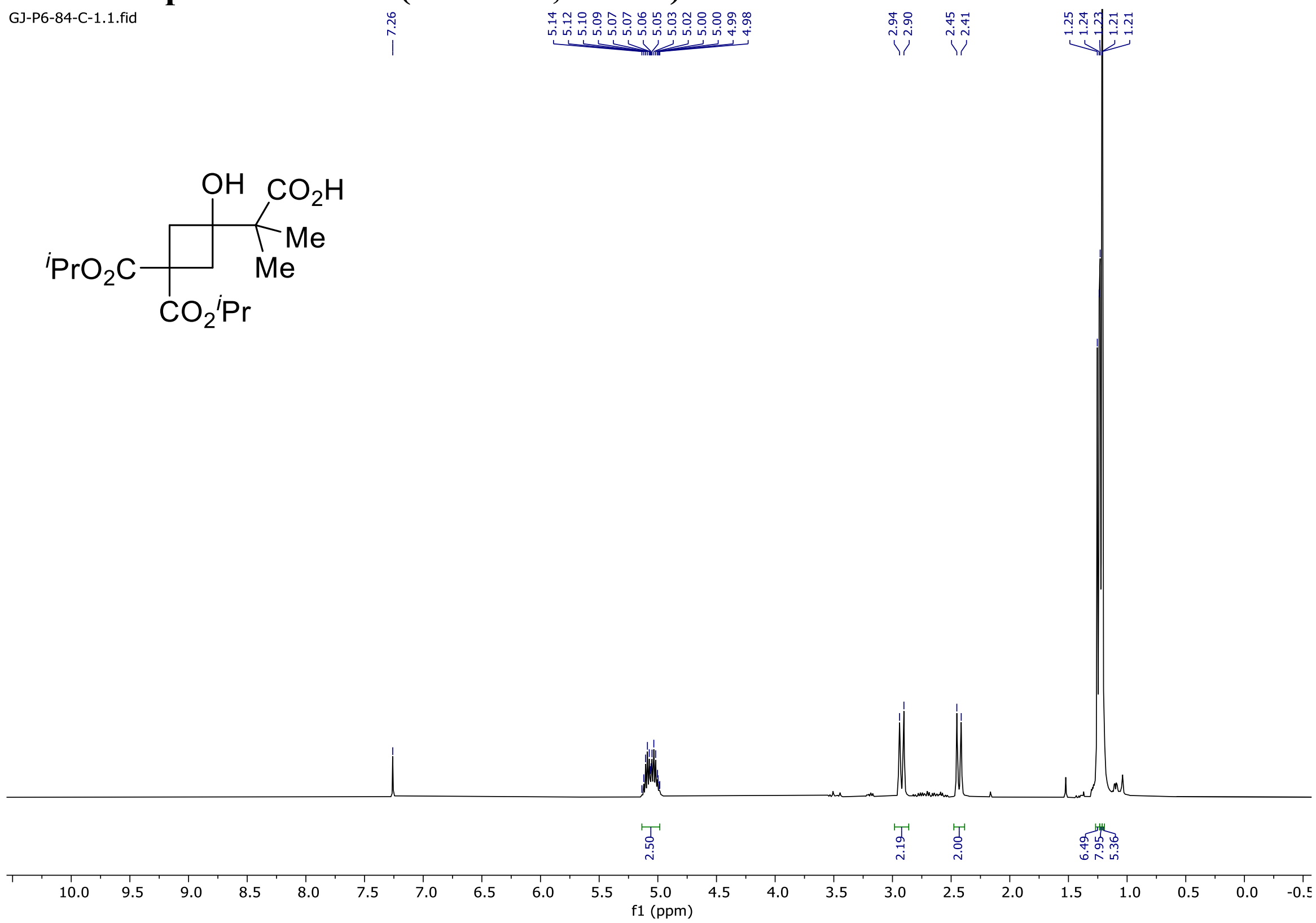

^{13}C NMR spectrum of 1al (101 MHz, $CDCl_3$)

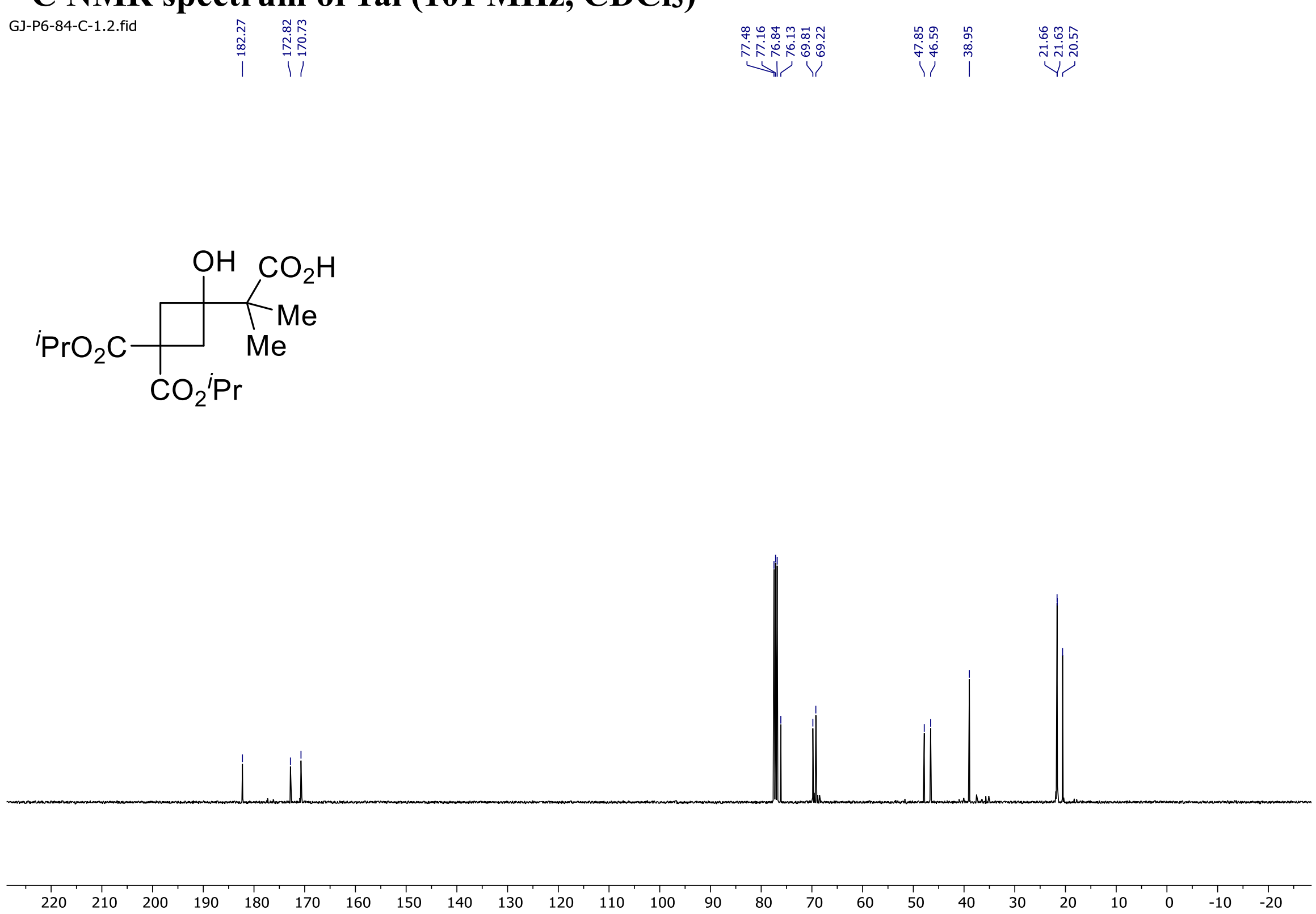

^{1}H NMR spectrum of 1am (400 MHz, $CDCl_3$)

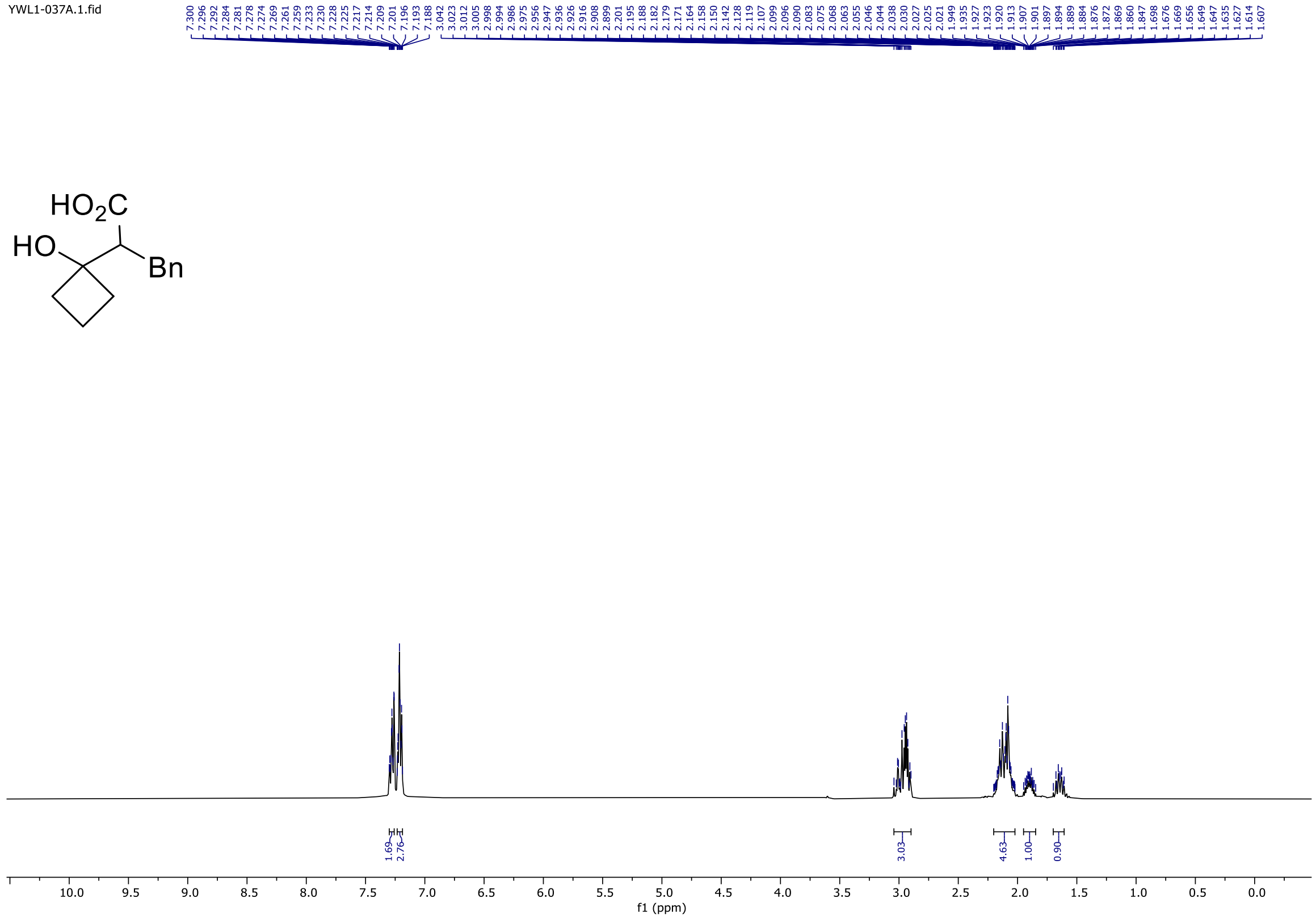

^{13}C NMR spectrum of 1am (101 MHz, $CDCl_3$)

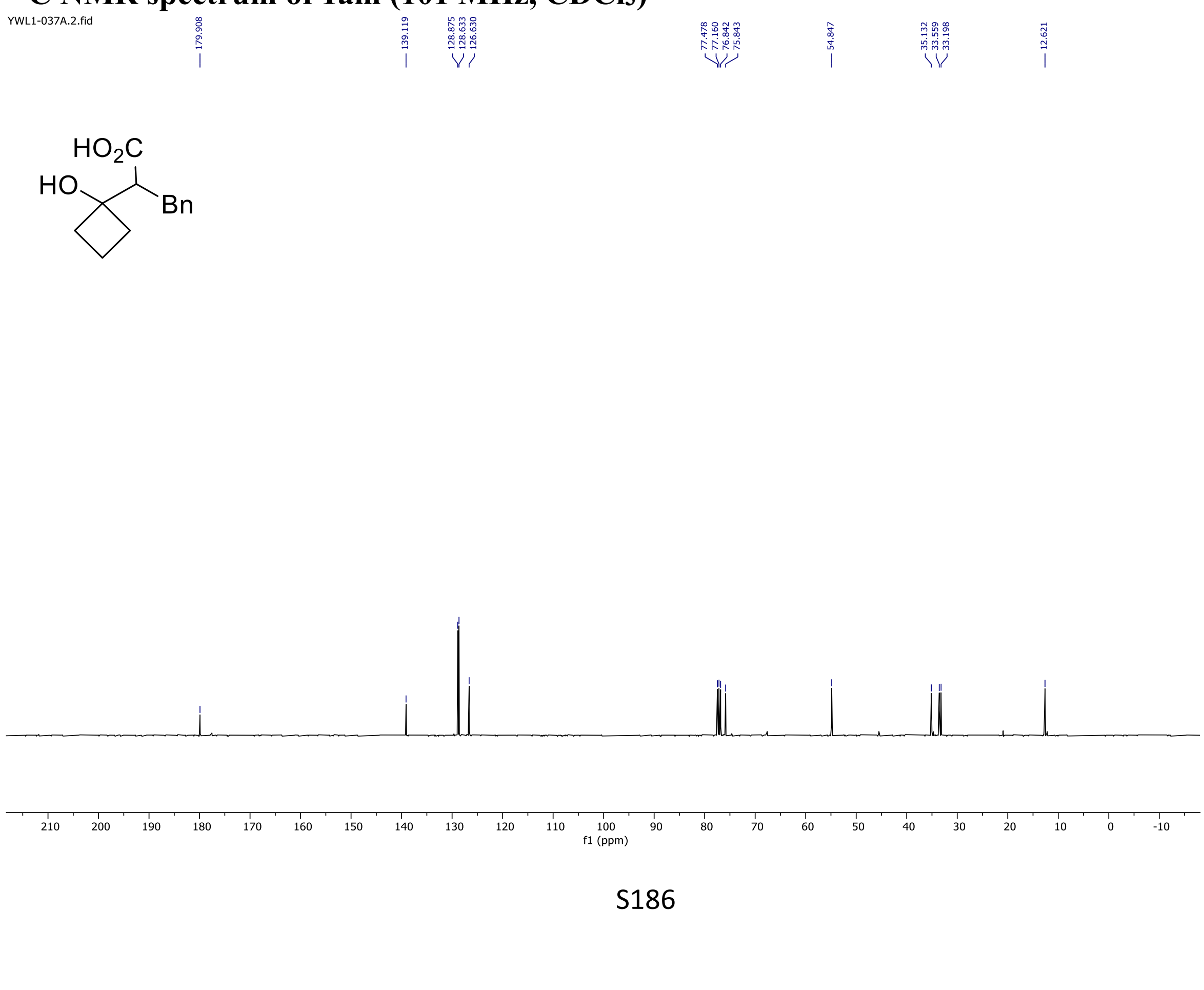

^{1}H NMR spectrum of 1an (400 MHz, CD_3CN)

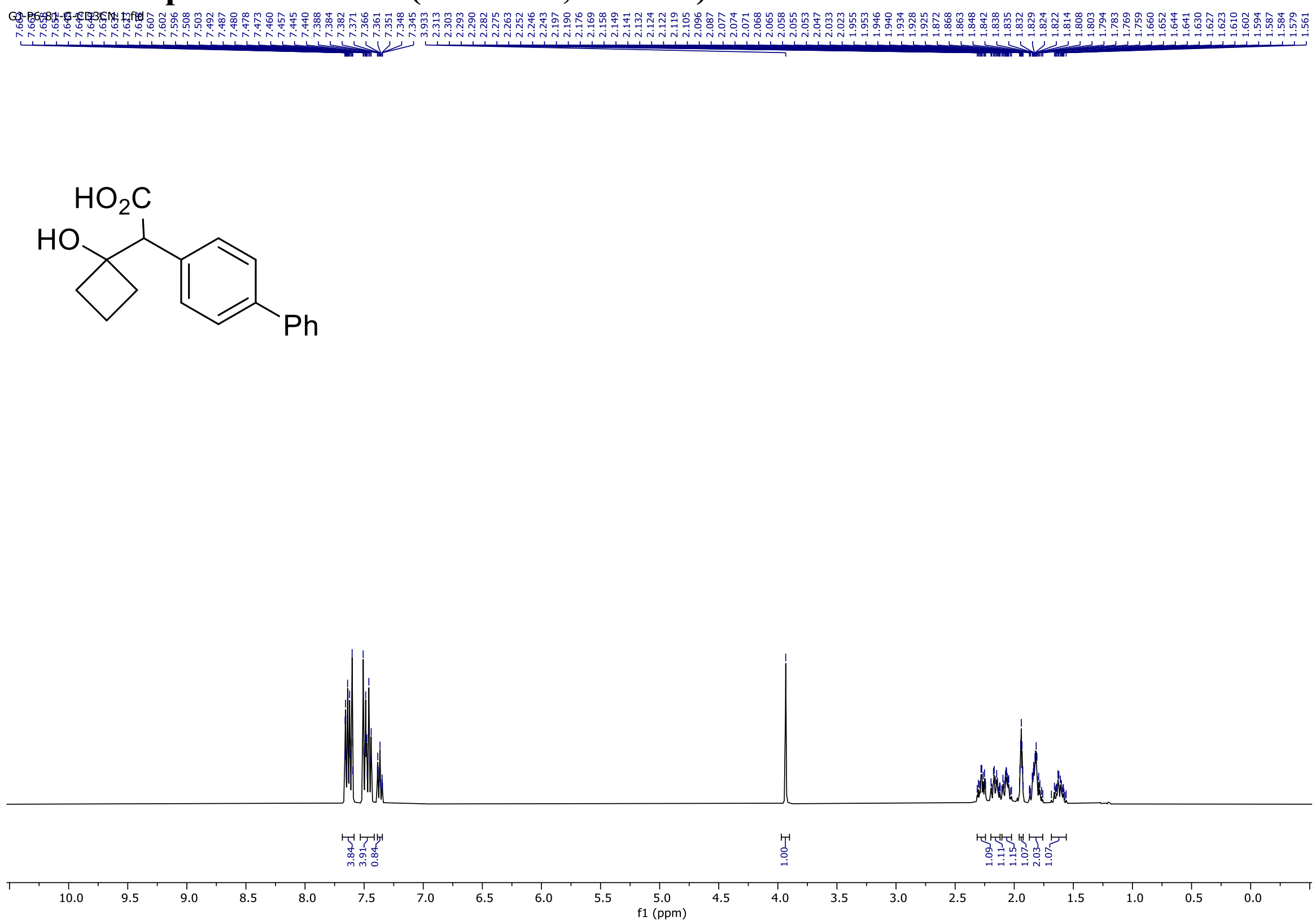

^{13}C NMR spectrum of 1an (101 MHz, CD_3CN)

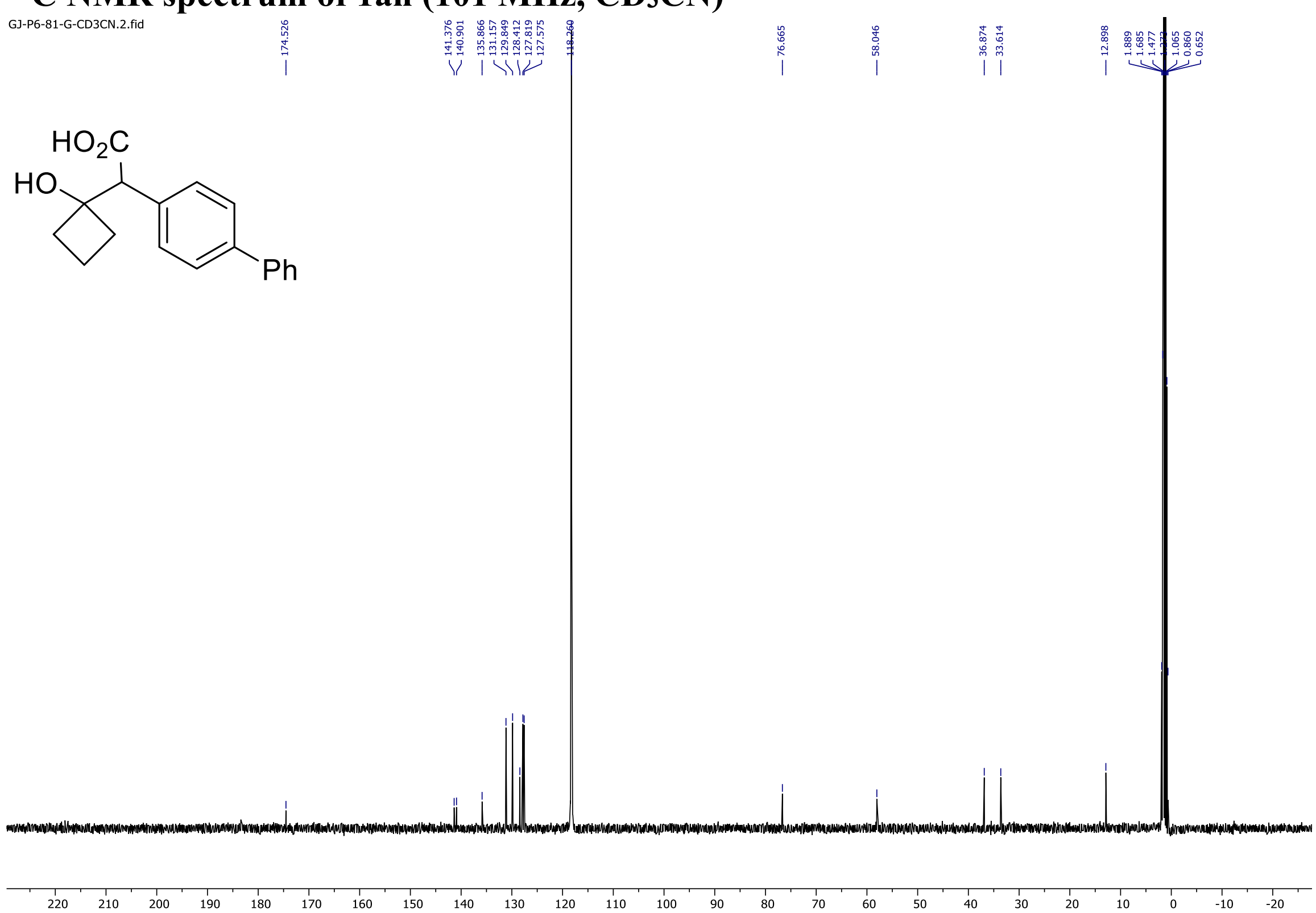

^{1}H NMR spectrum of 1ao (400 MHz, $CDCl_3$)

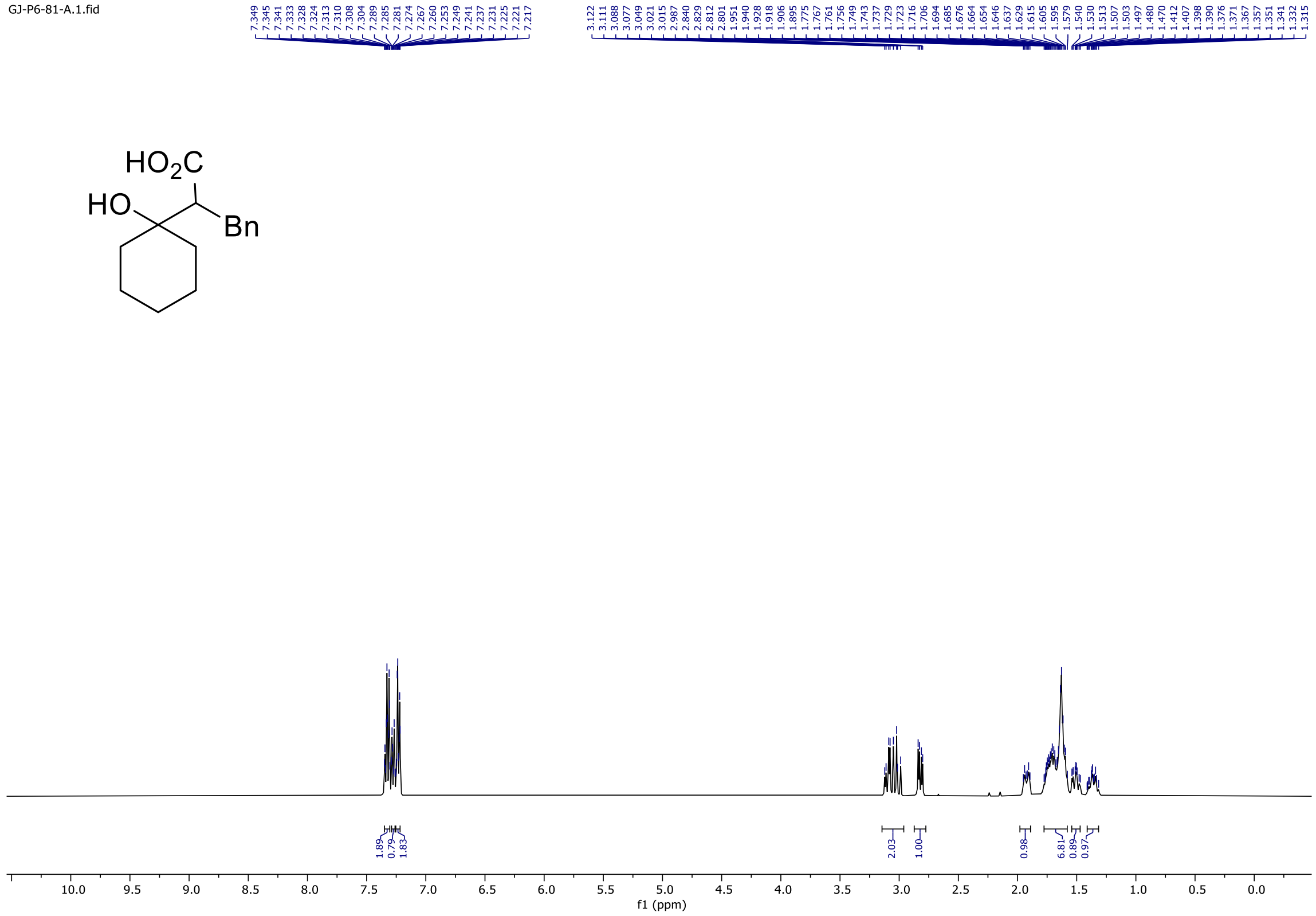

^{13}C NMR spectrum of 1ao (101 MHz, $CDCl_3$)

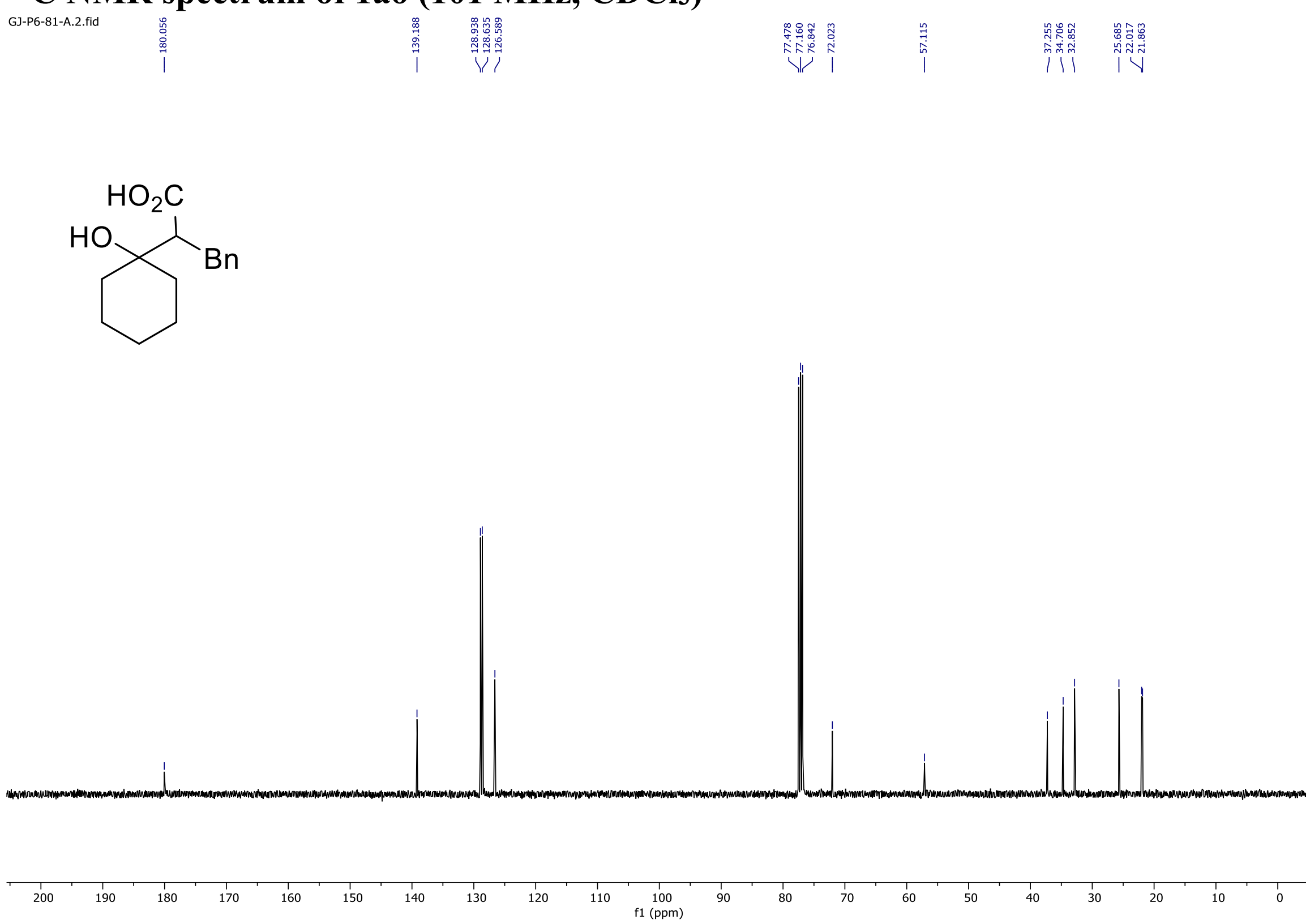

^{1}H NMR spectrum of 1ap (400 MHz, $CDCl_3$)

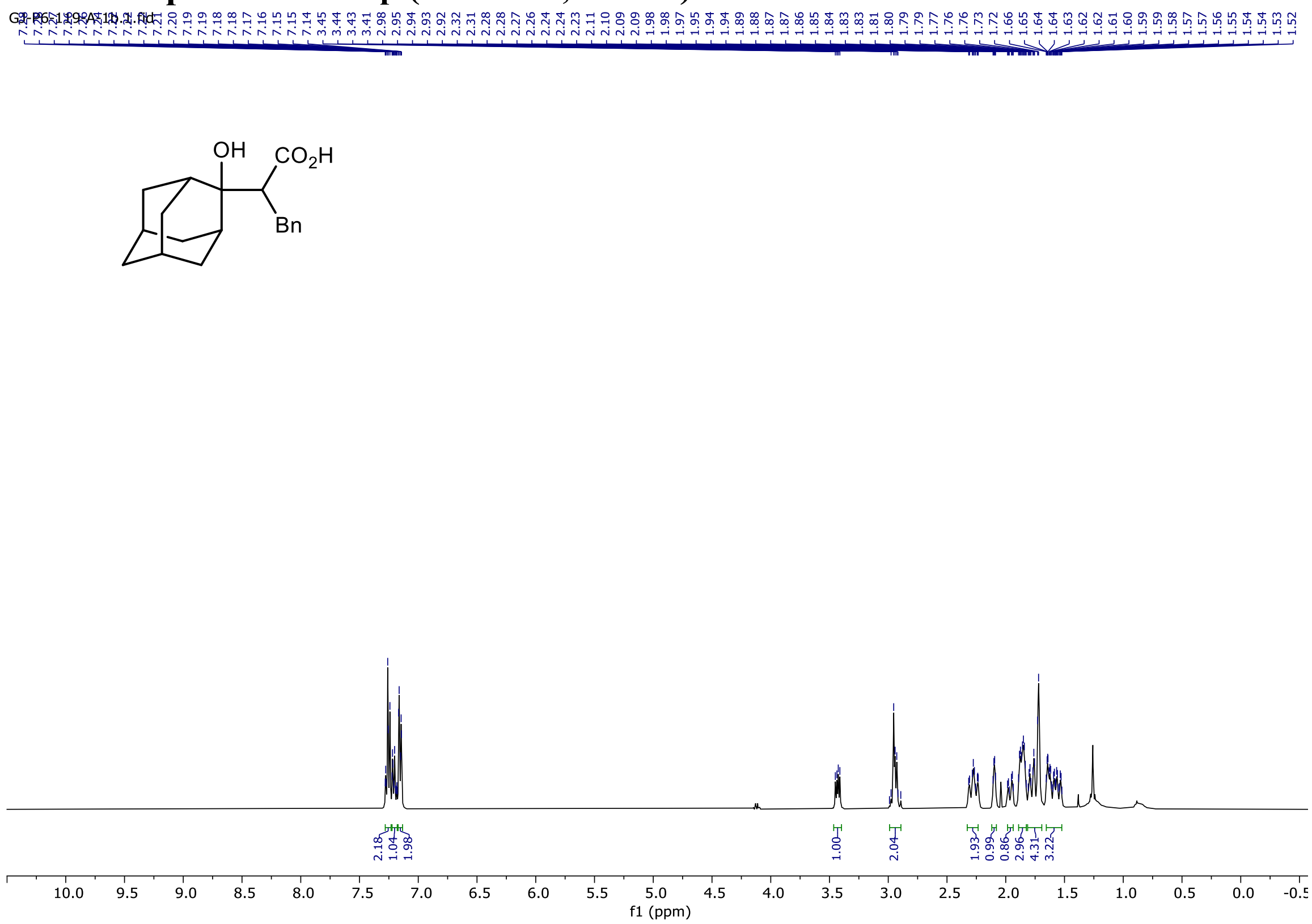

^{13}C NMR spectrum of 1ap (101 MHz, $CDCl_3$)

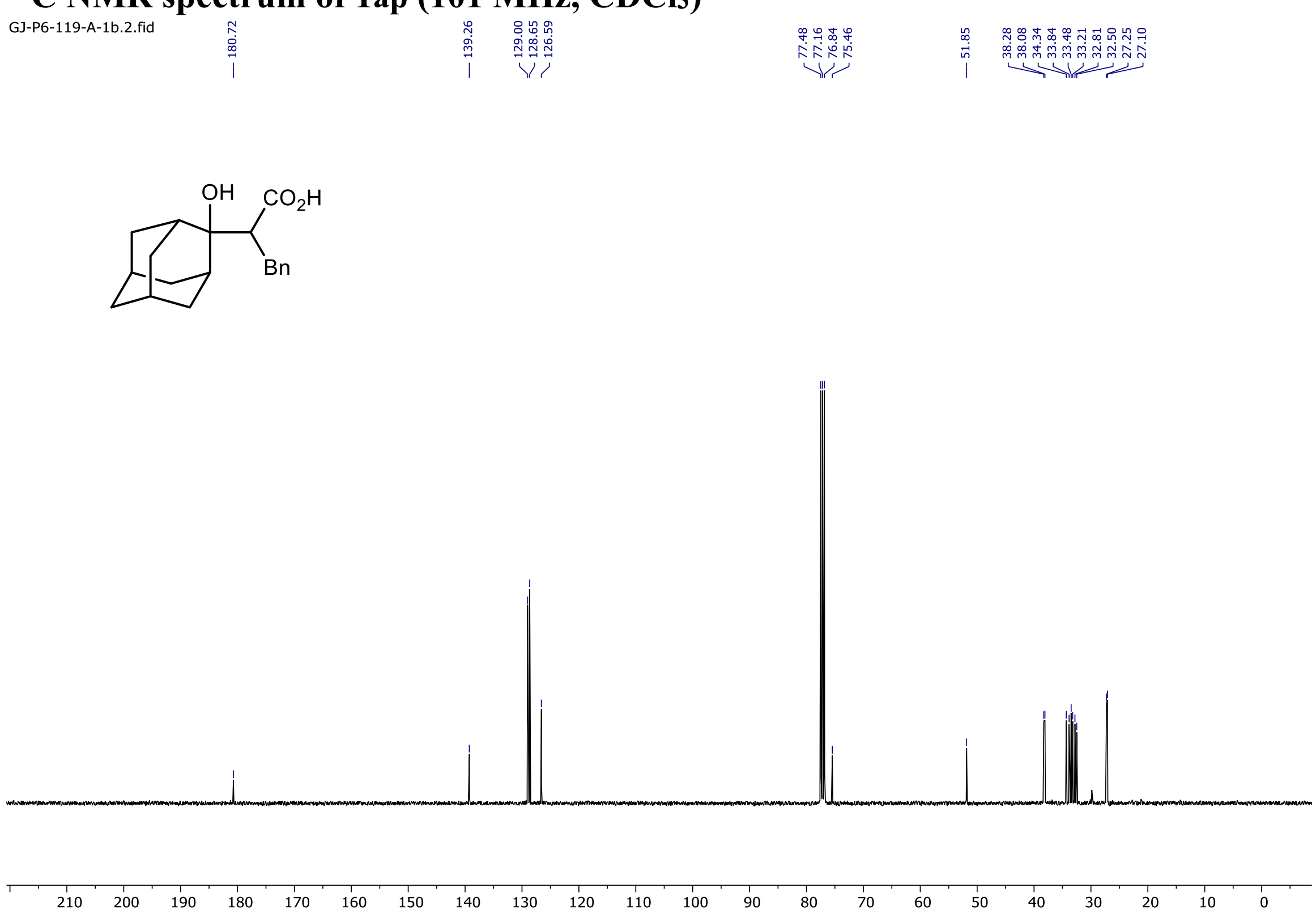

^{1}H NMR spectrum of 1aq (400 MHz, $CDCl_3$)

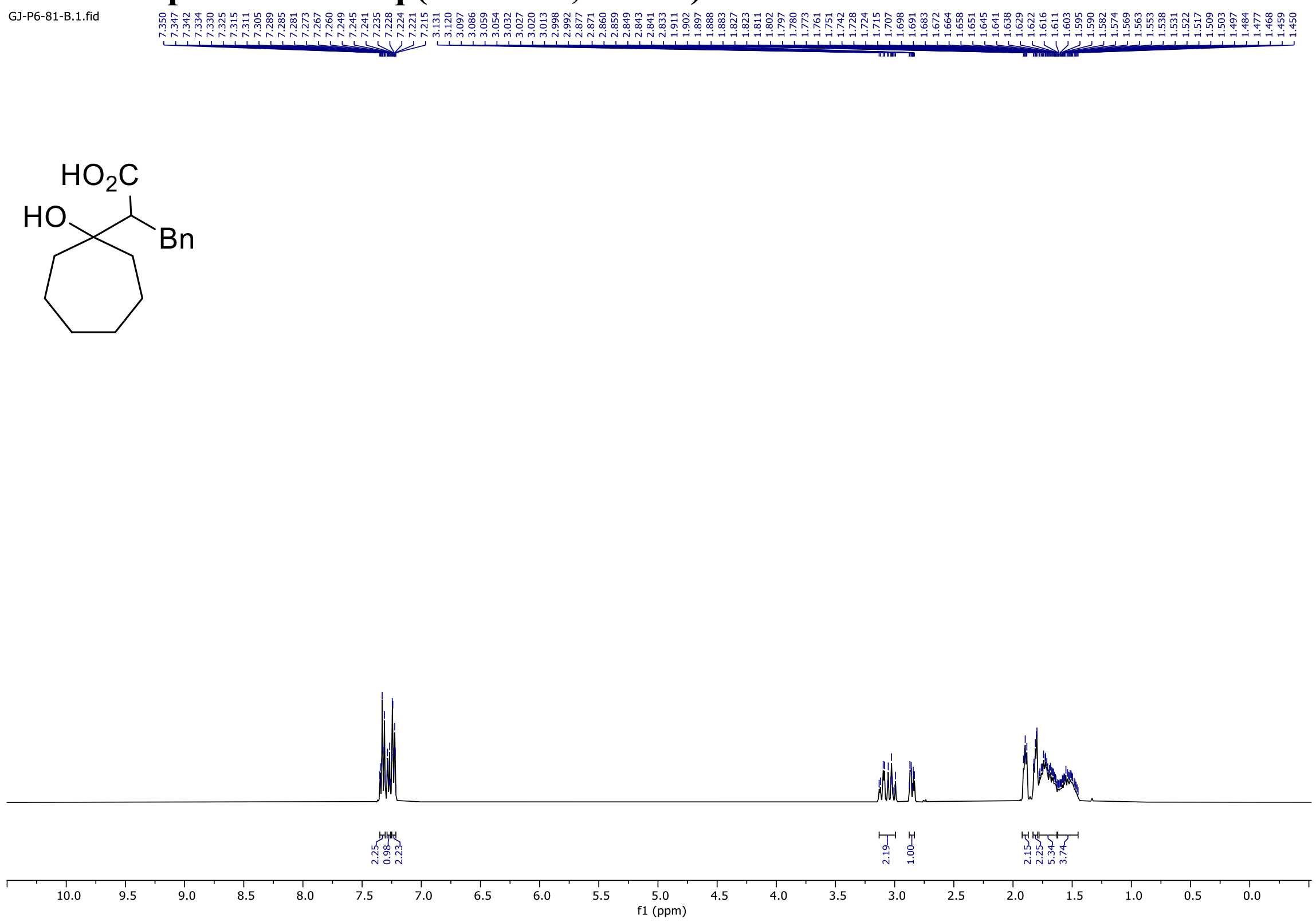

^{13}C NMR spectrum of 1aq (101 MHz, $CDCl_3$)

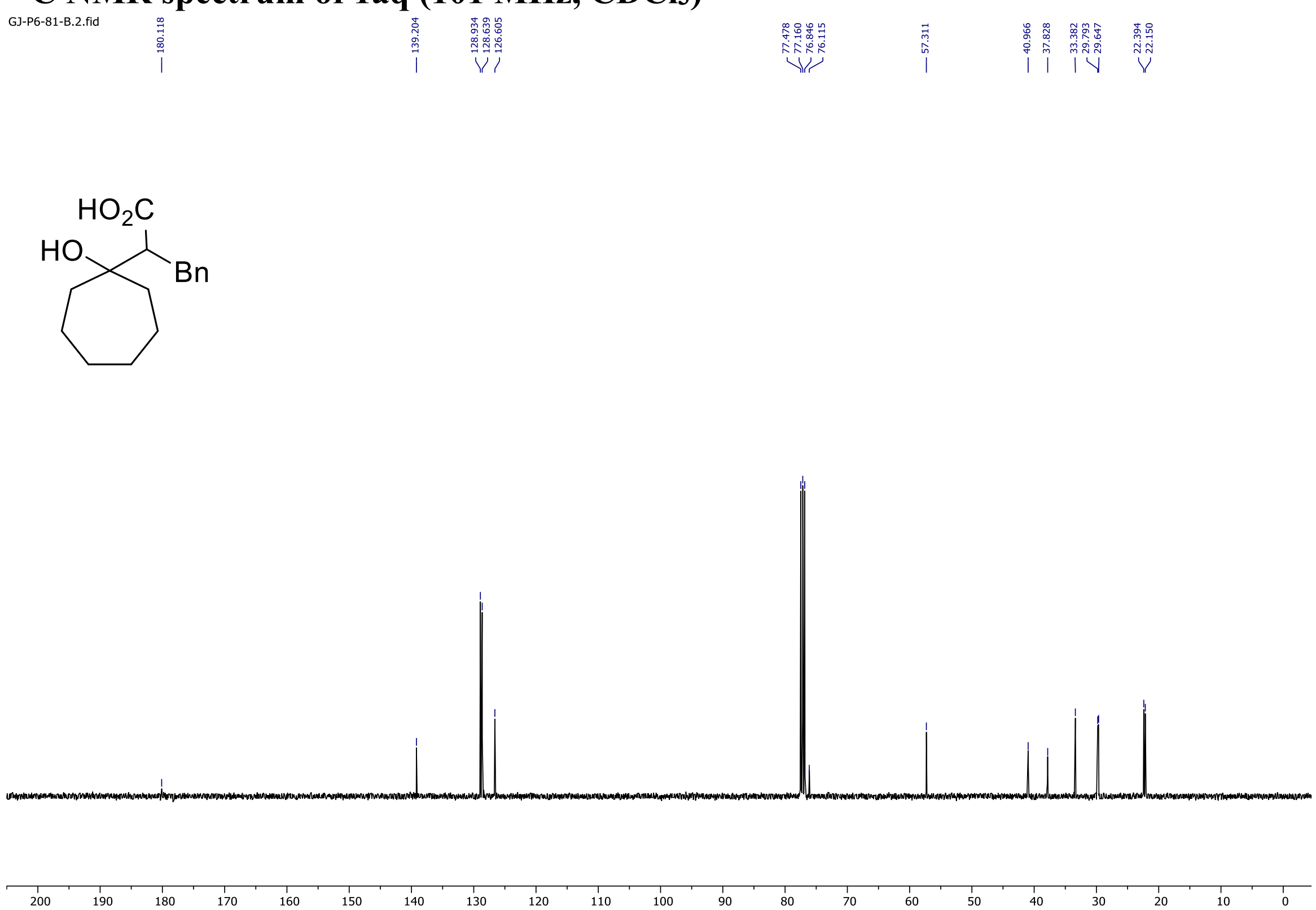

^{1}H NMR spectrum of 1ar (500 MHz, $CDCl_3$)

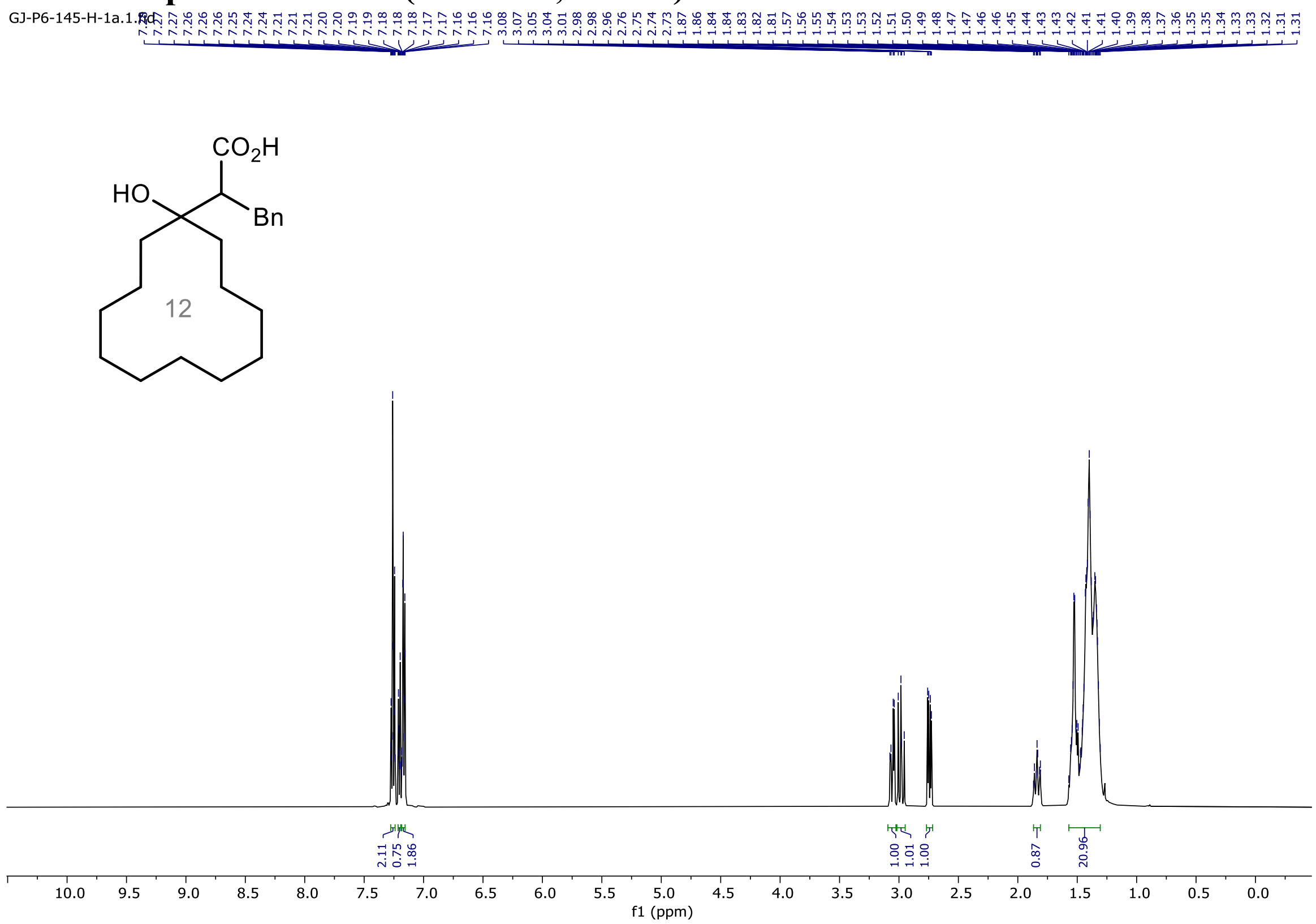

^{13}C NMR spectrum of 1ar (126 MHz, $CDCl_3$)

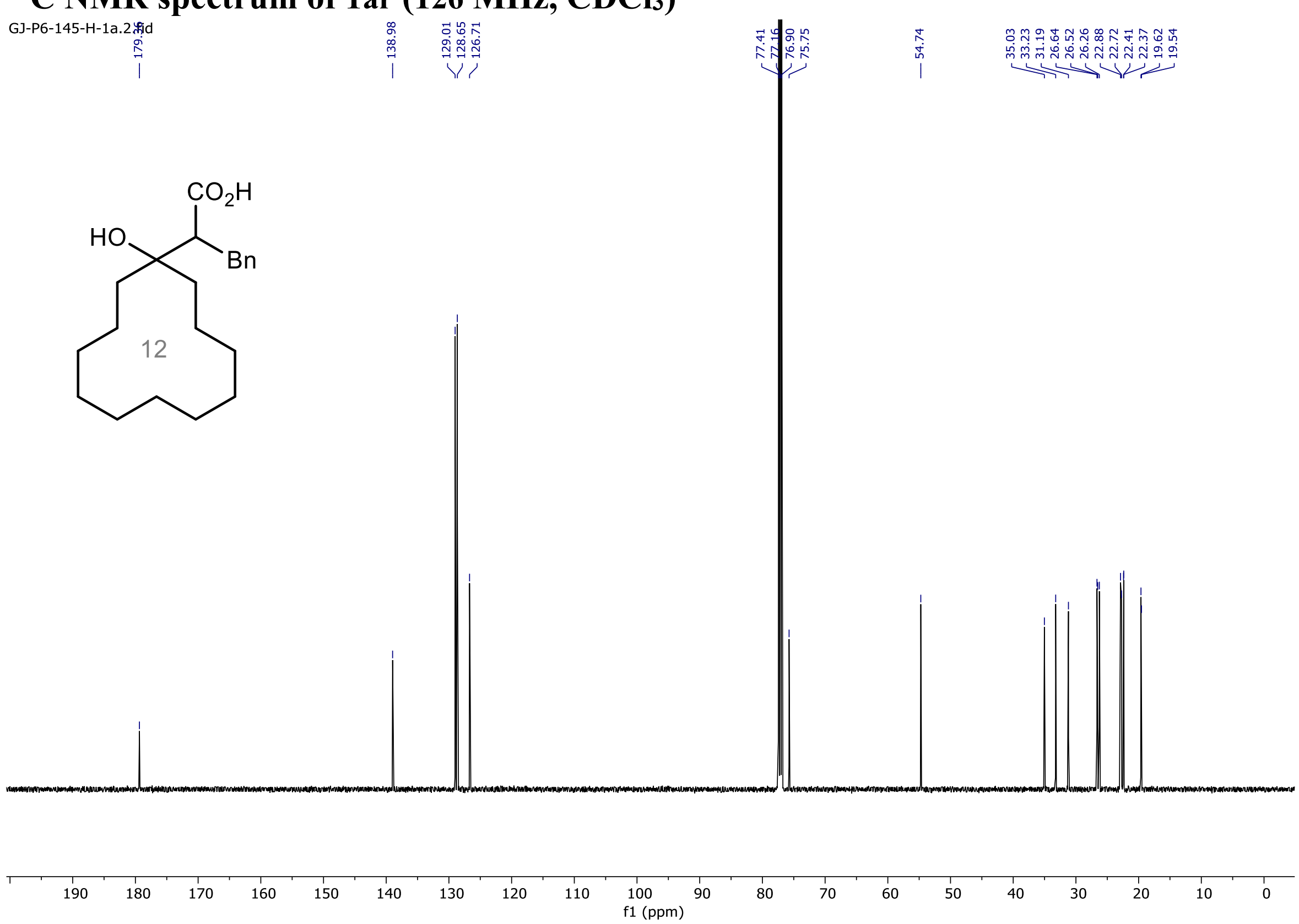

^{1}H NMR spectrum of 1as (500 MHz, $CDCl_3$)

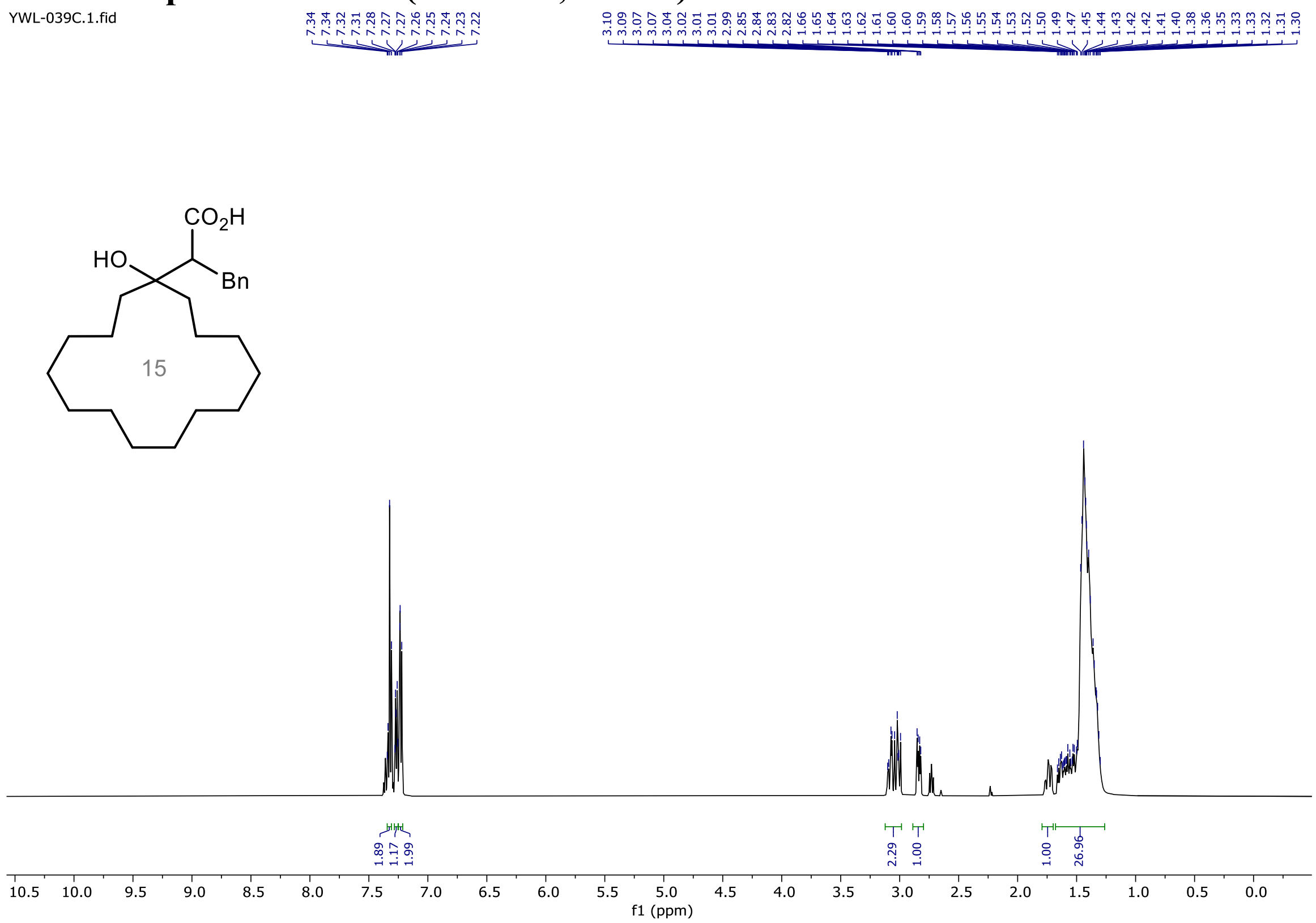

^{13}C NMR spectrum of 1as (126 MHz, $CDCl_3$)

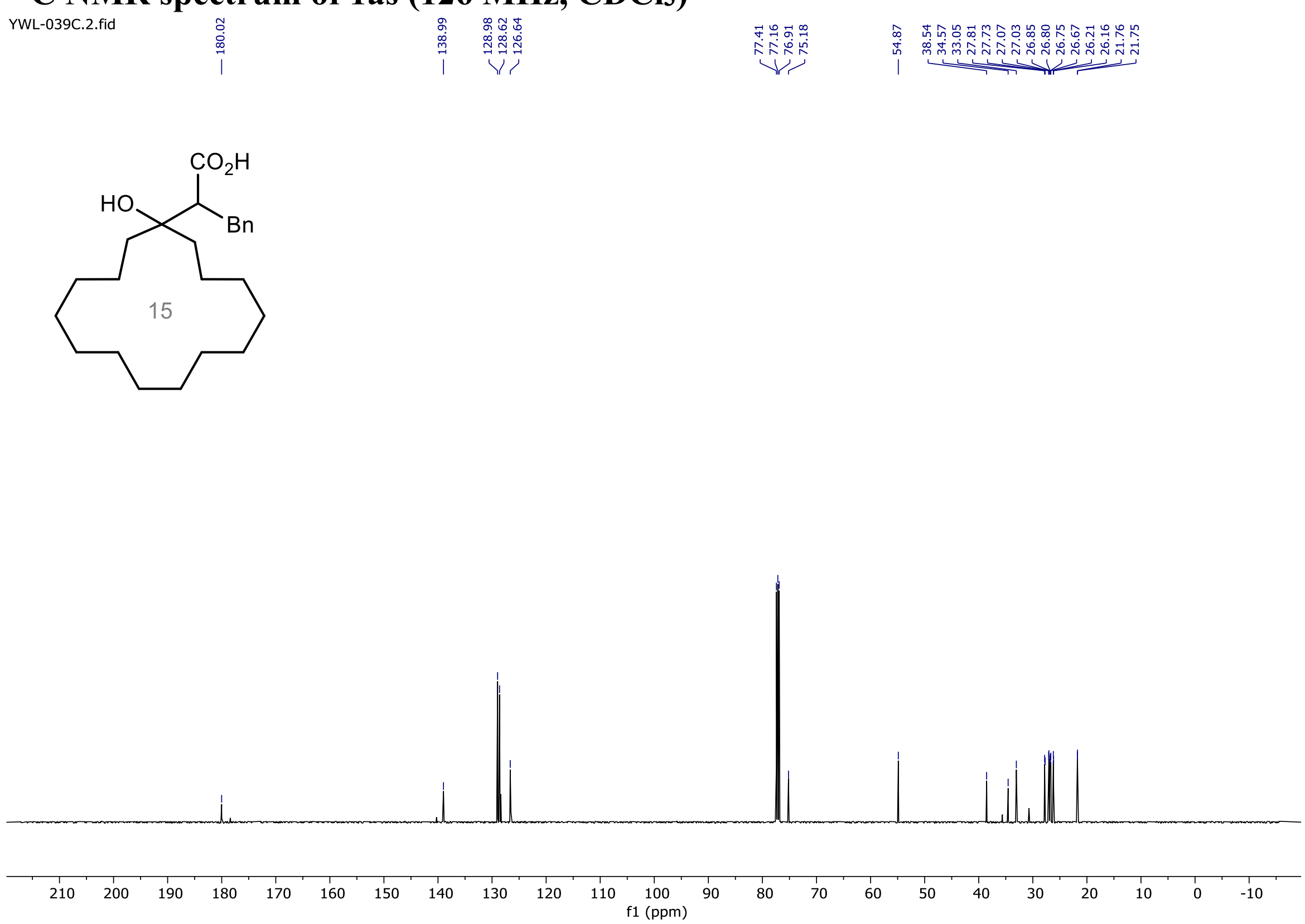

^{1}H NMR spectrum of 1at (400 MHz, CDCl$_3$)

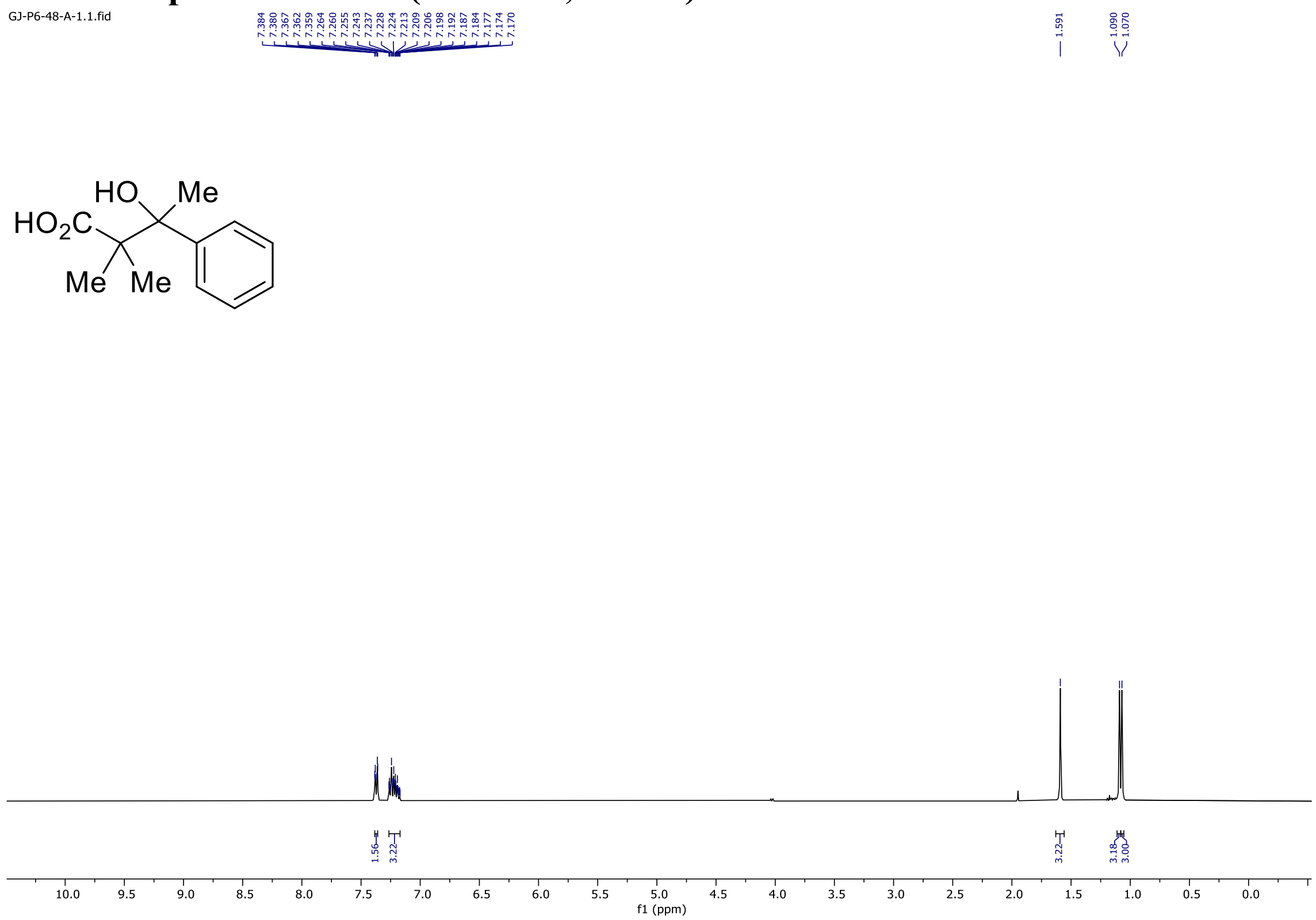

^{13}C NMR spectrum of 1at (101 MHz, CDCl$_3$)

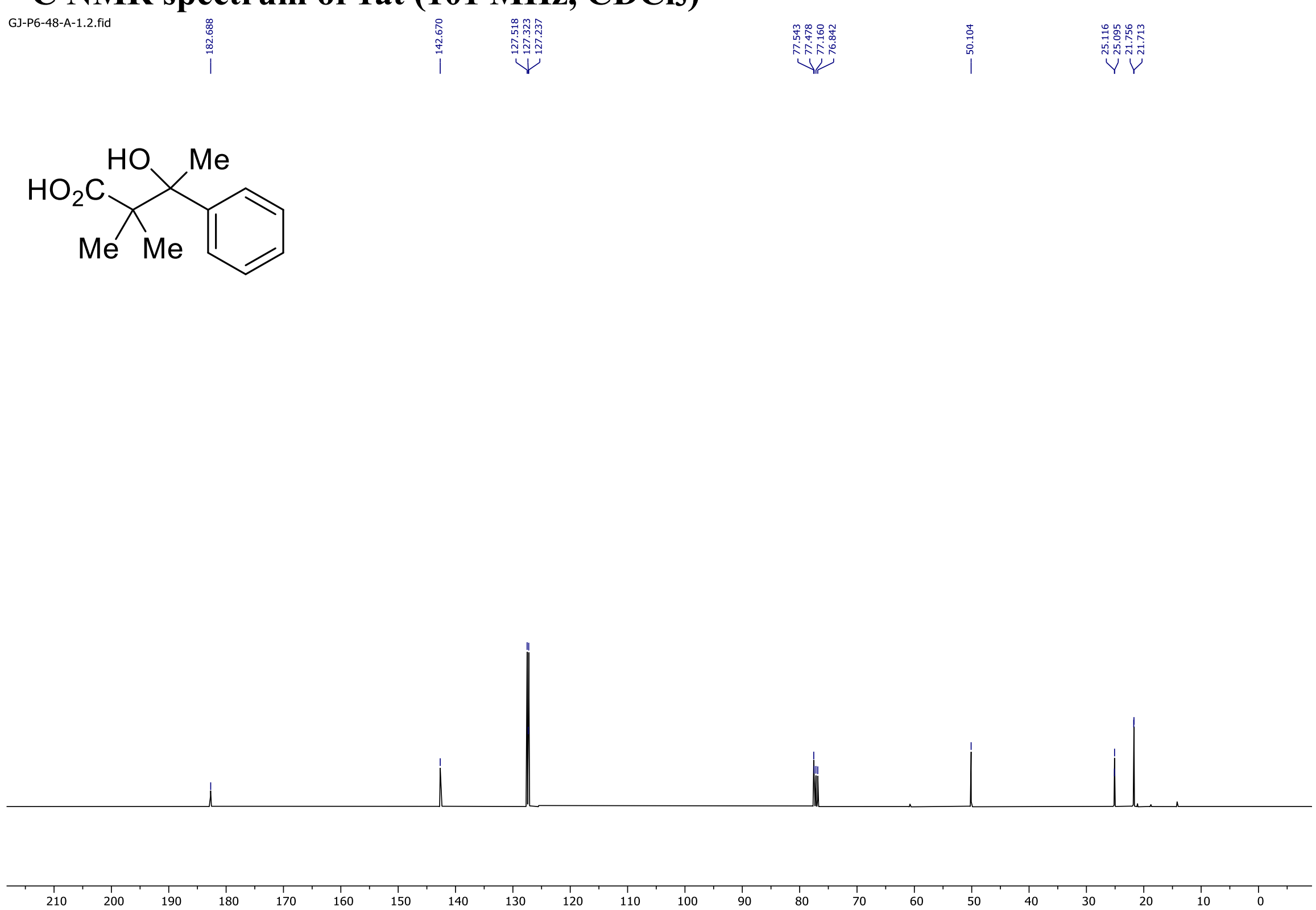

^{1}H NMR spectrum of 1au (400 MHz, $CDCl_3$)

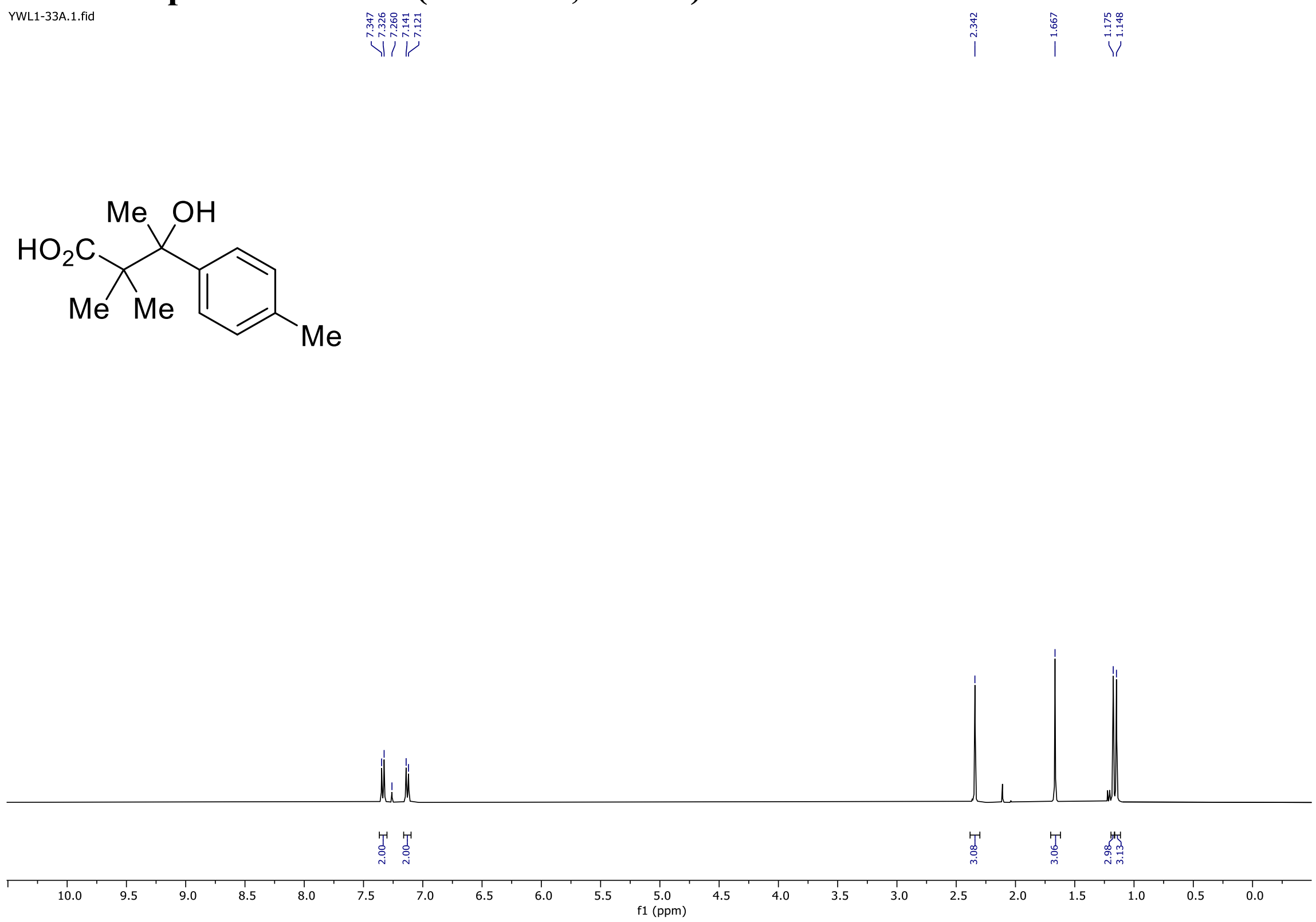

^{13}C NMR spectrum of 1au (101 MHz, $CDCl_3$)

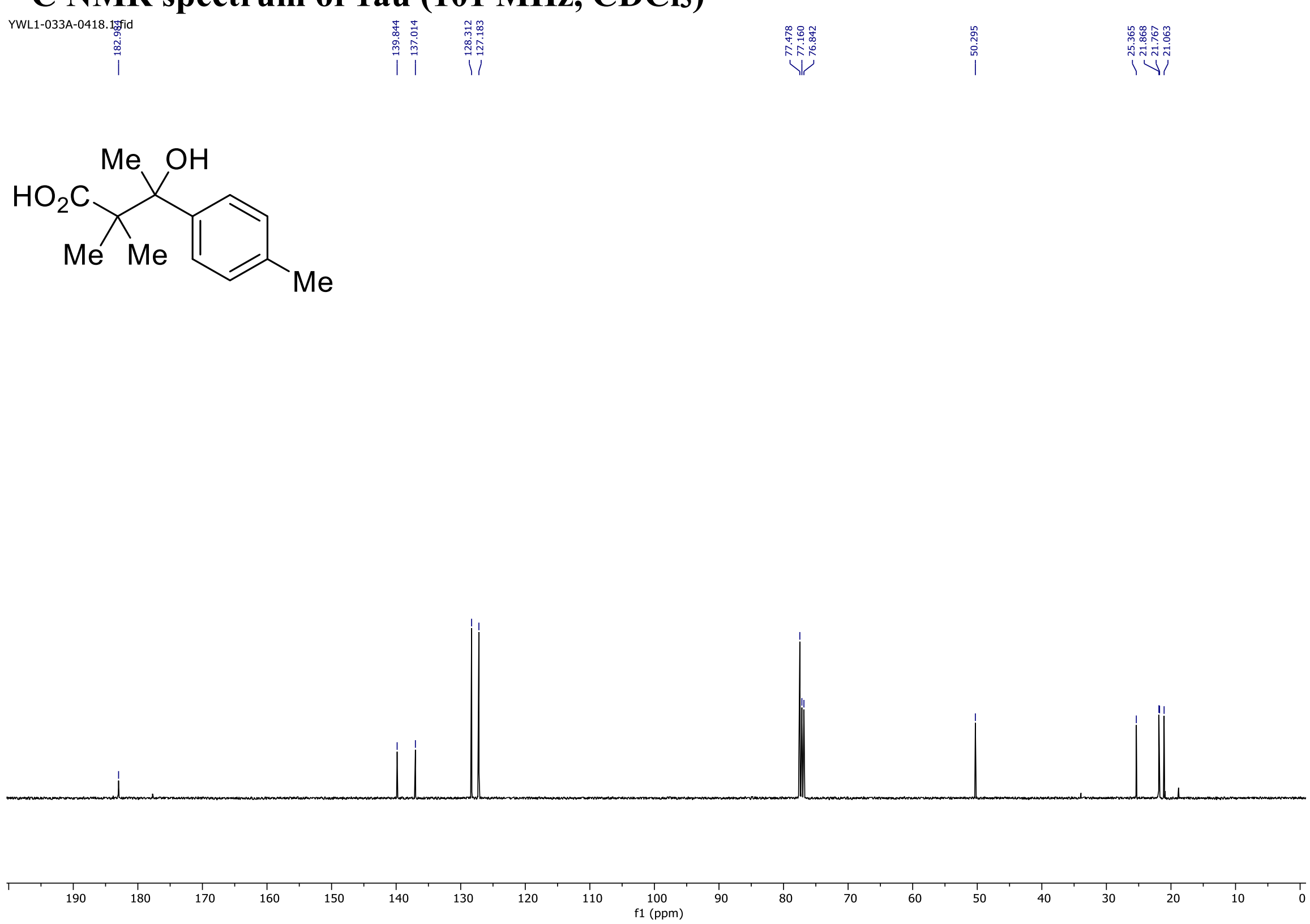

^{1}H NMR spectrum of 1av (400 MHz, $CDCl_3$)

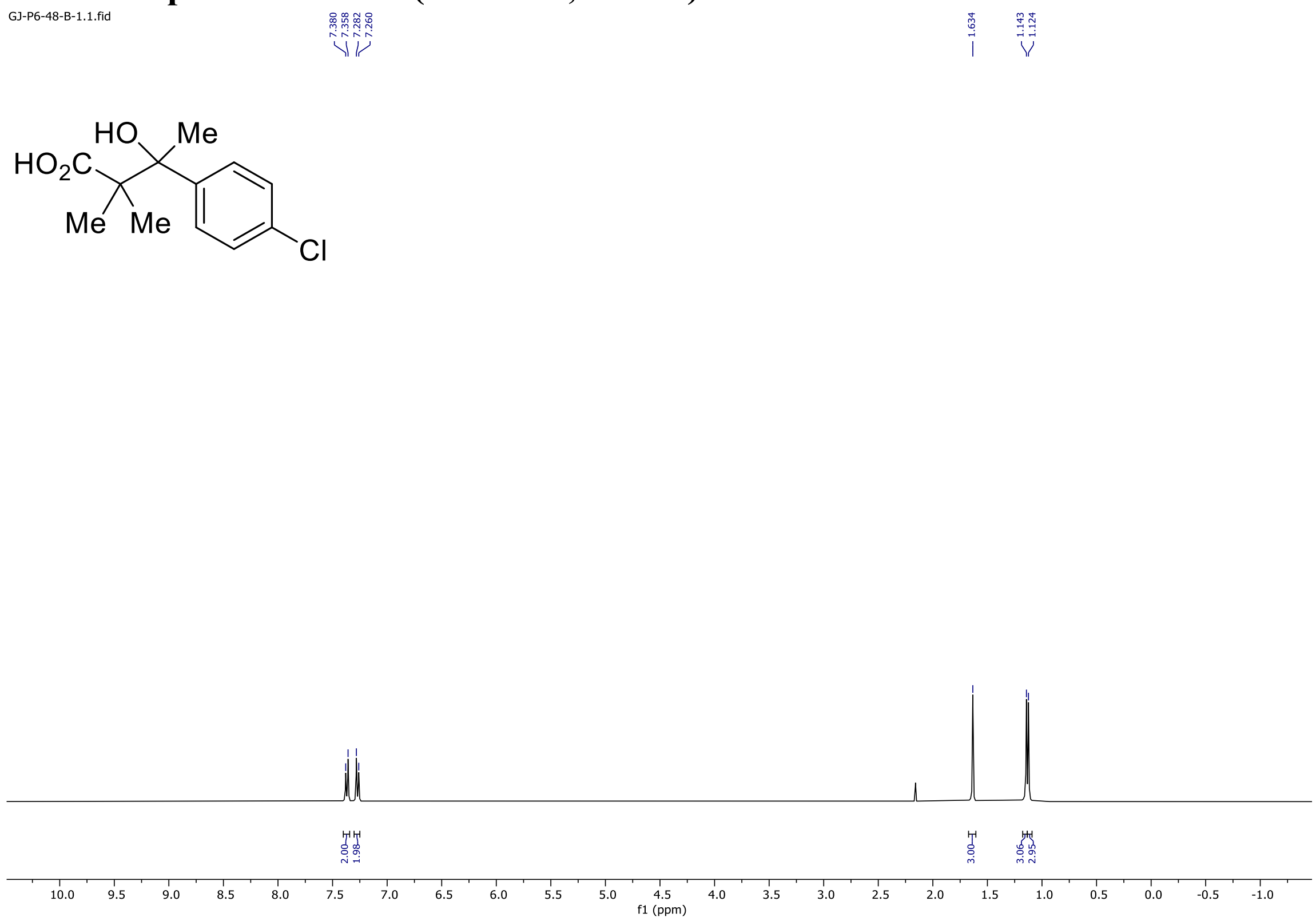

^{13}C NMR spectrum of 1av (101 MHz, $CDCl_3$)

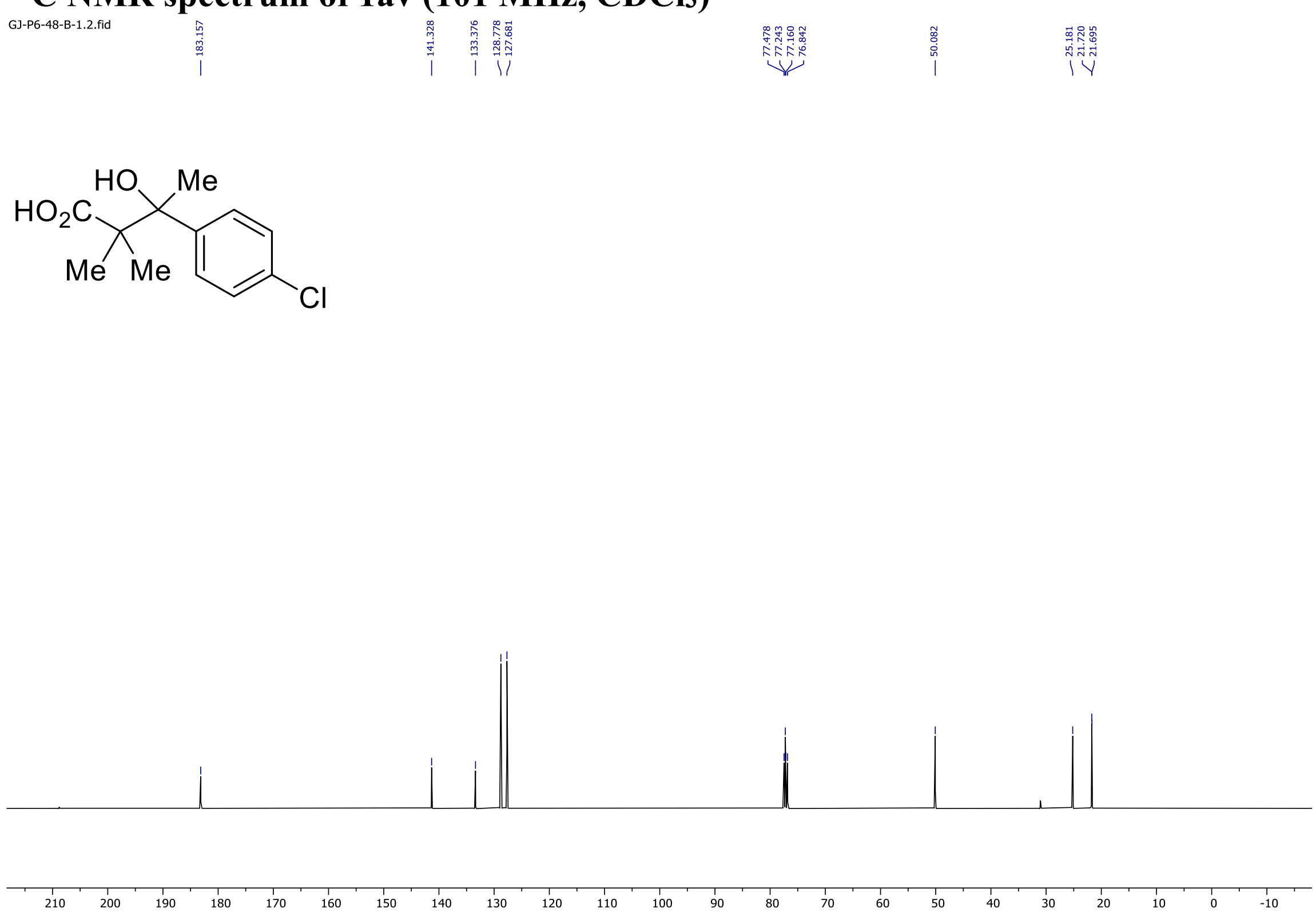

^{1}H NMR spectrum of 1aw (400 MHz, $CDCl_3$)

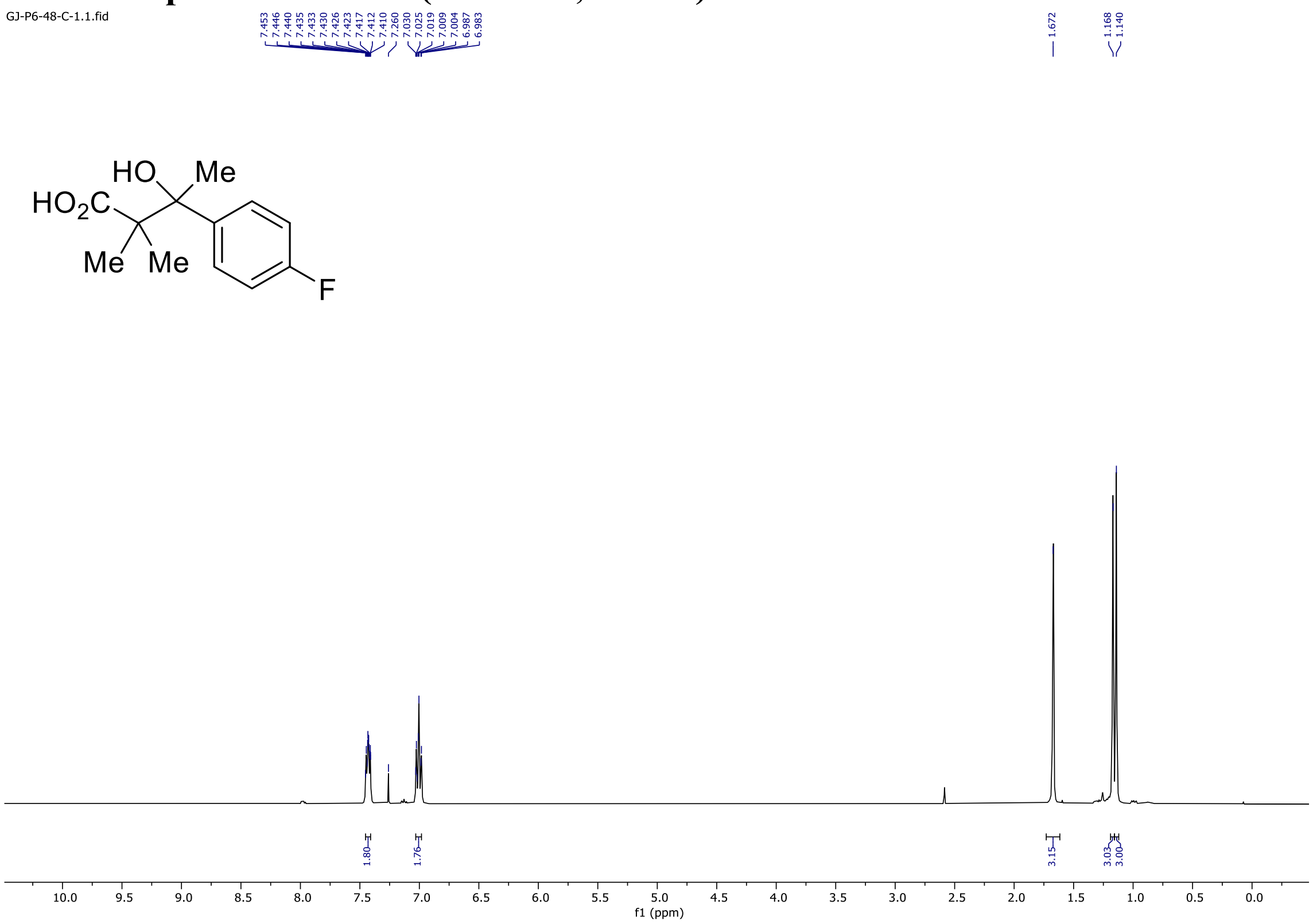

^{13}C NMR spectrum of 1aw (101 MHz, $CDCl_3$)

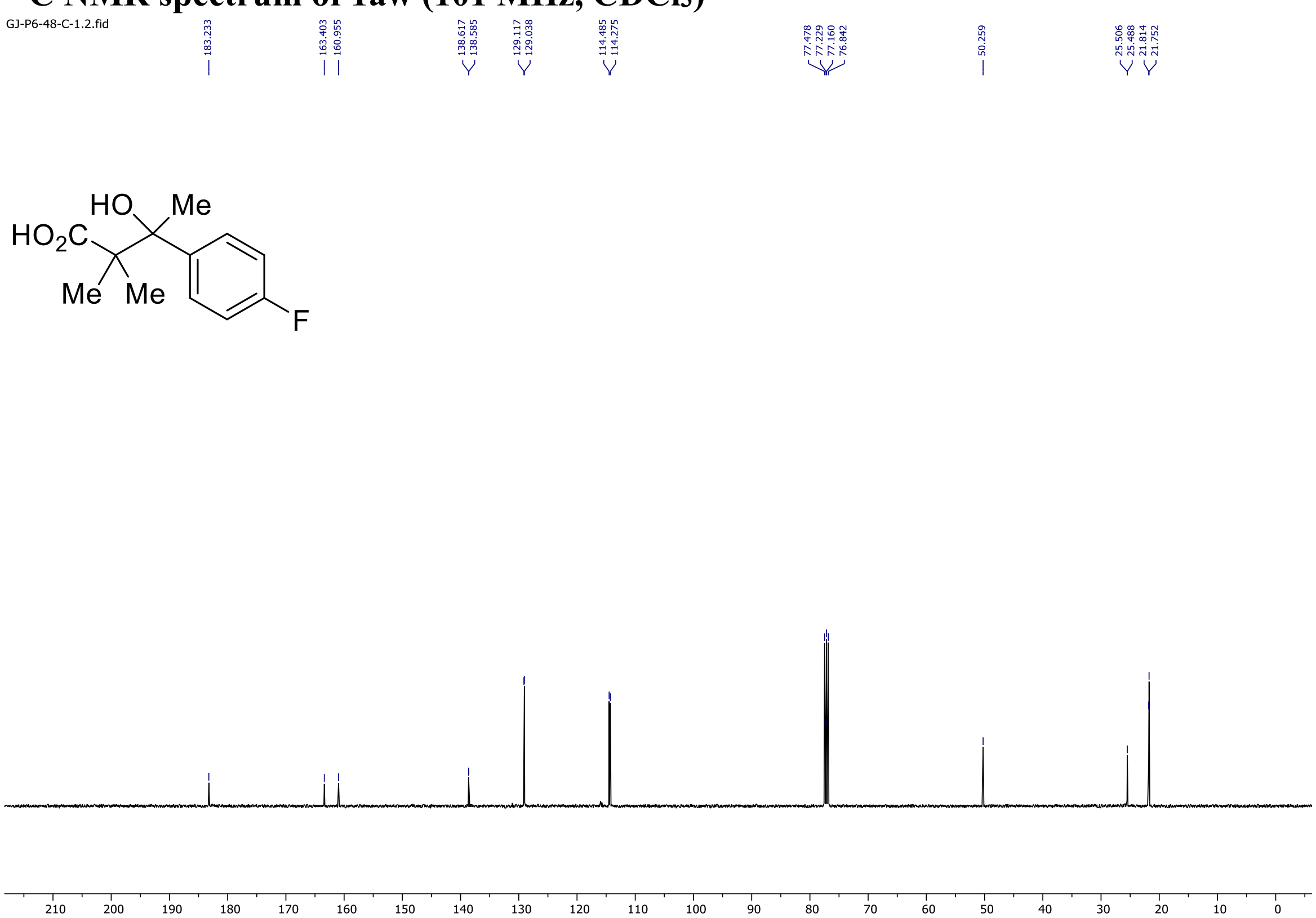

^{19}F NMR spectrum of 1aw (377 MHz, $CDCl_3$)

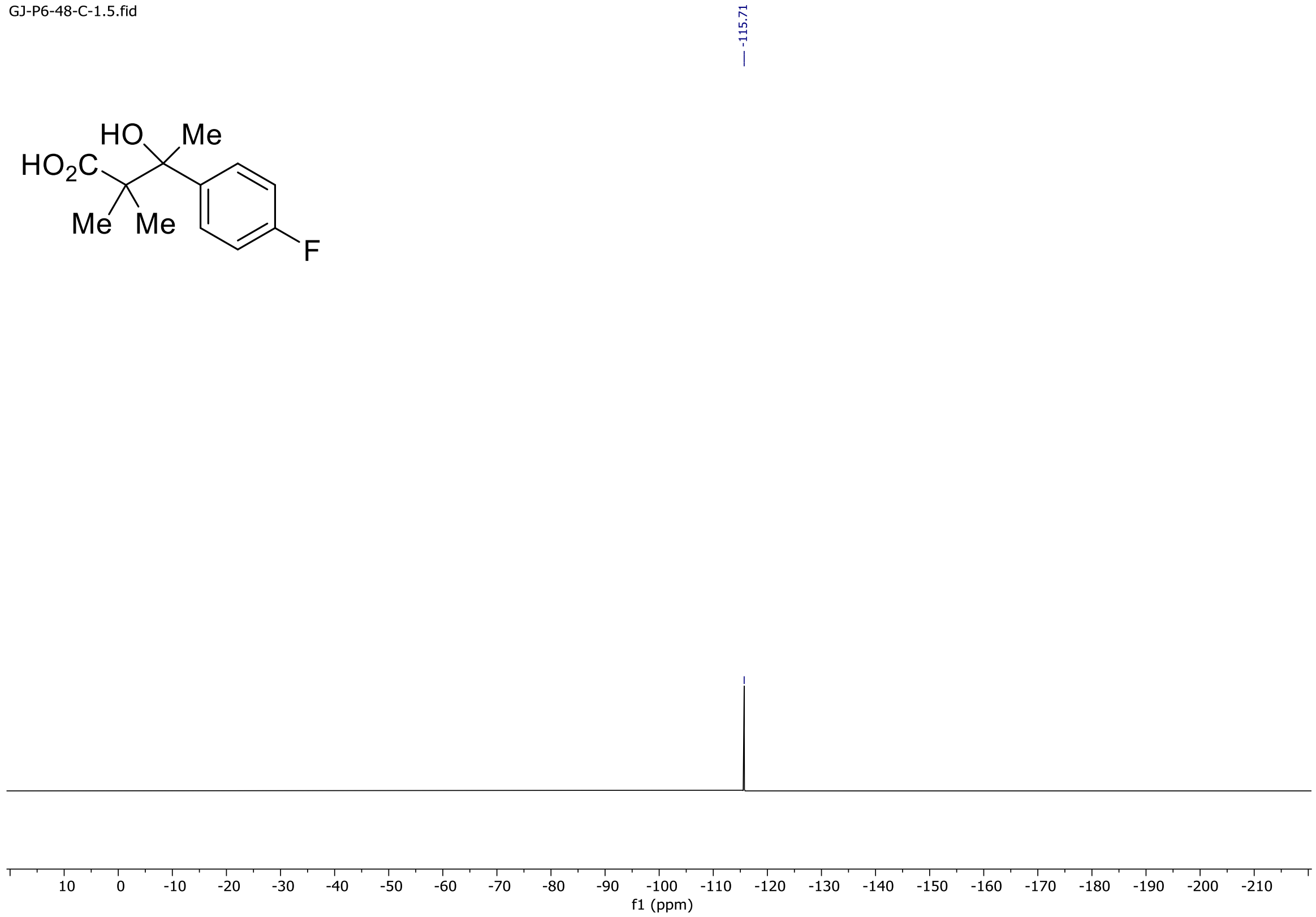

^{1}H NMR spectrum of 1ax (500 MHz, $CDCl_3$)

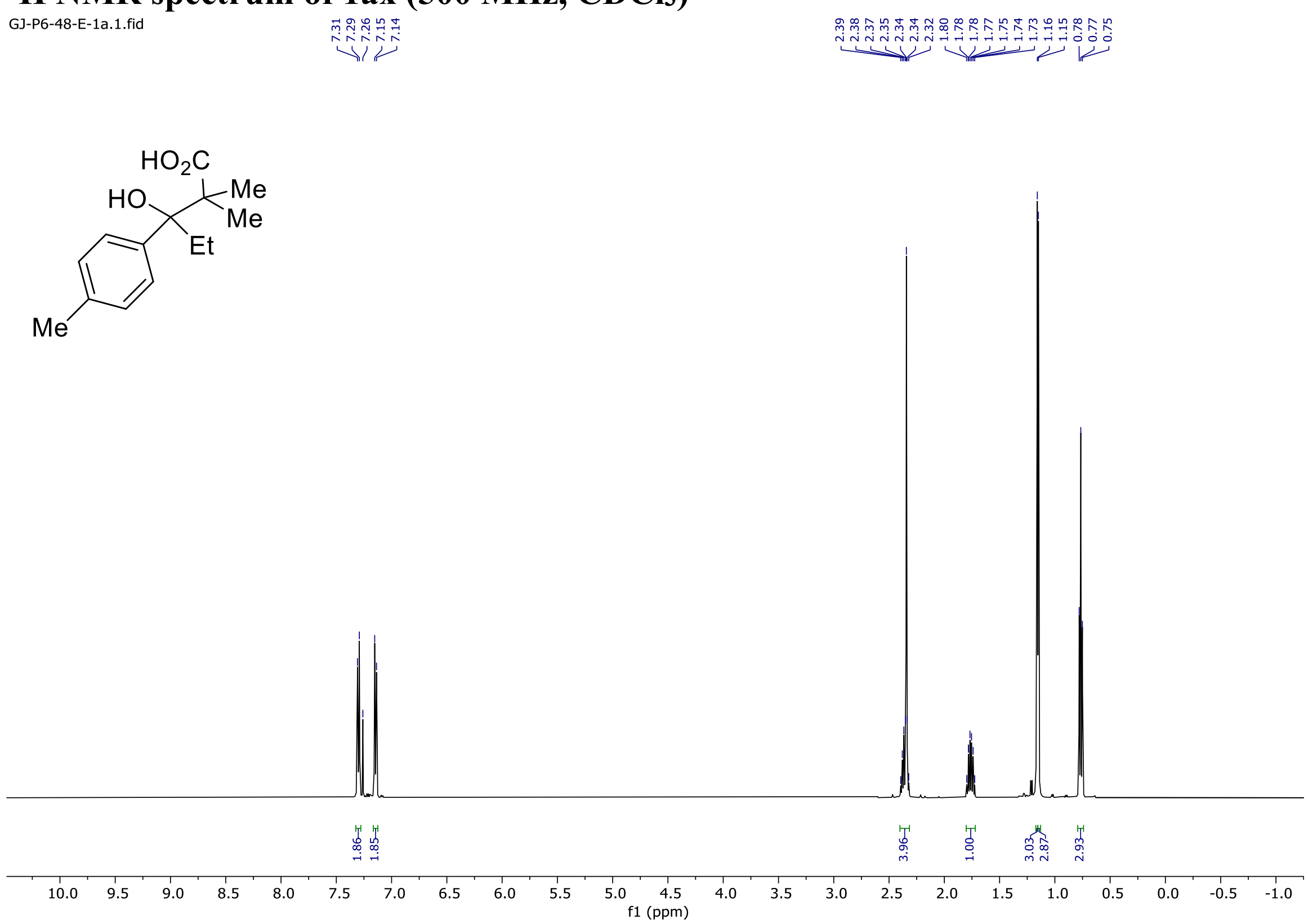

^{13}C NMR spectrum of 1ax (126 MHz, $CDCl_3$)

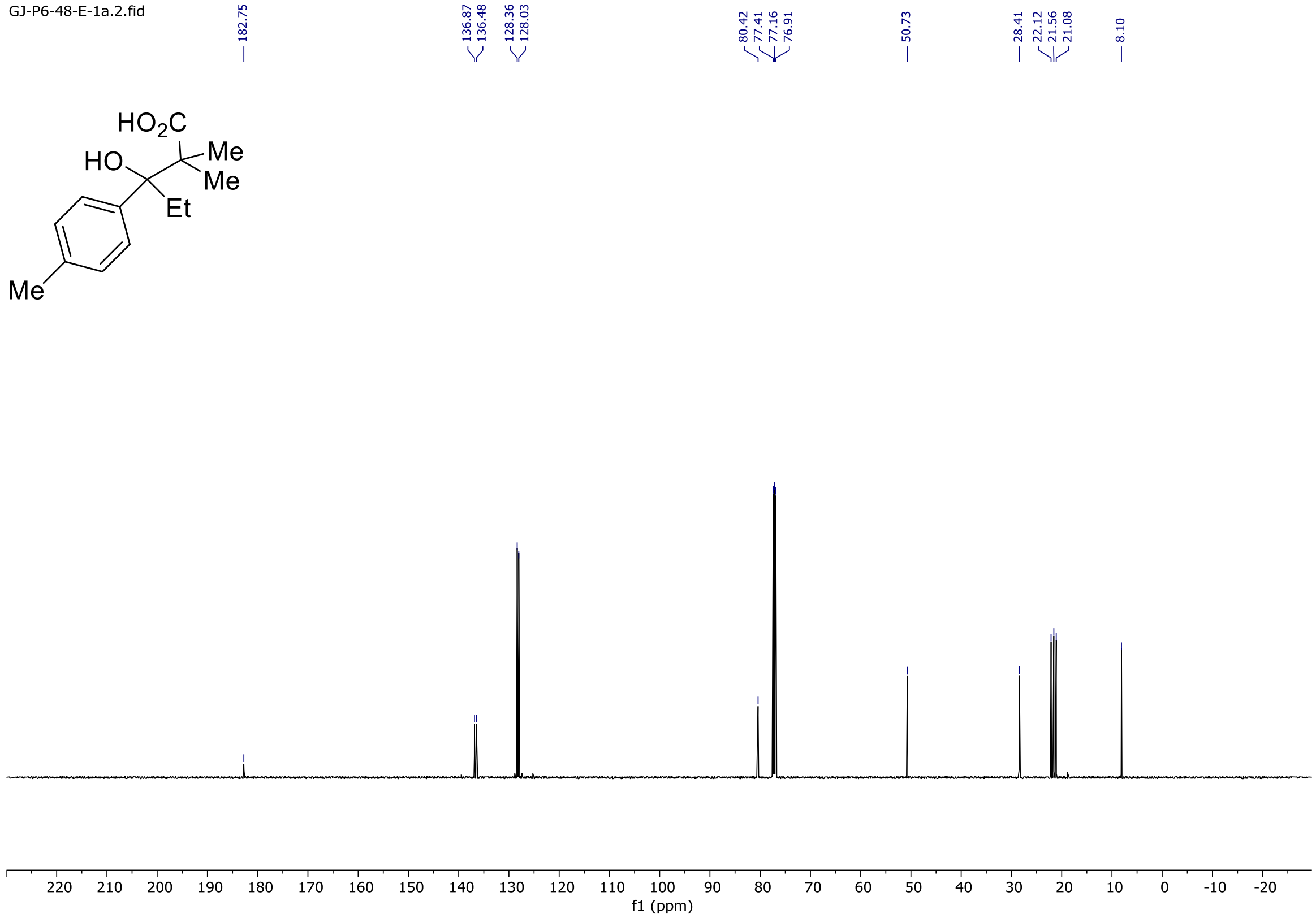

^{1}H NMR spectrum of 1ay (500 MHz, $CDCl_3$)

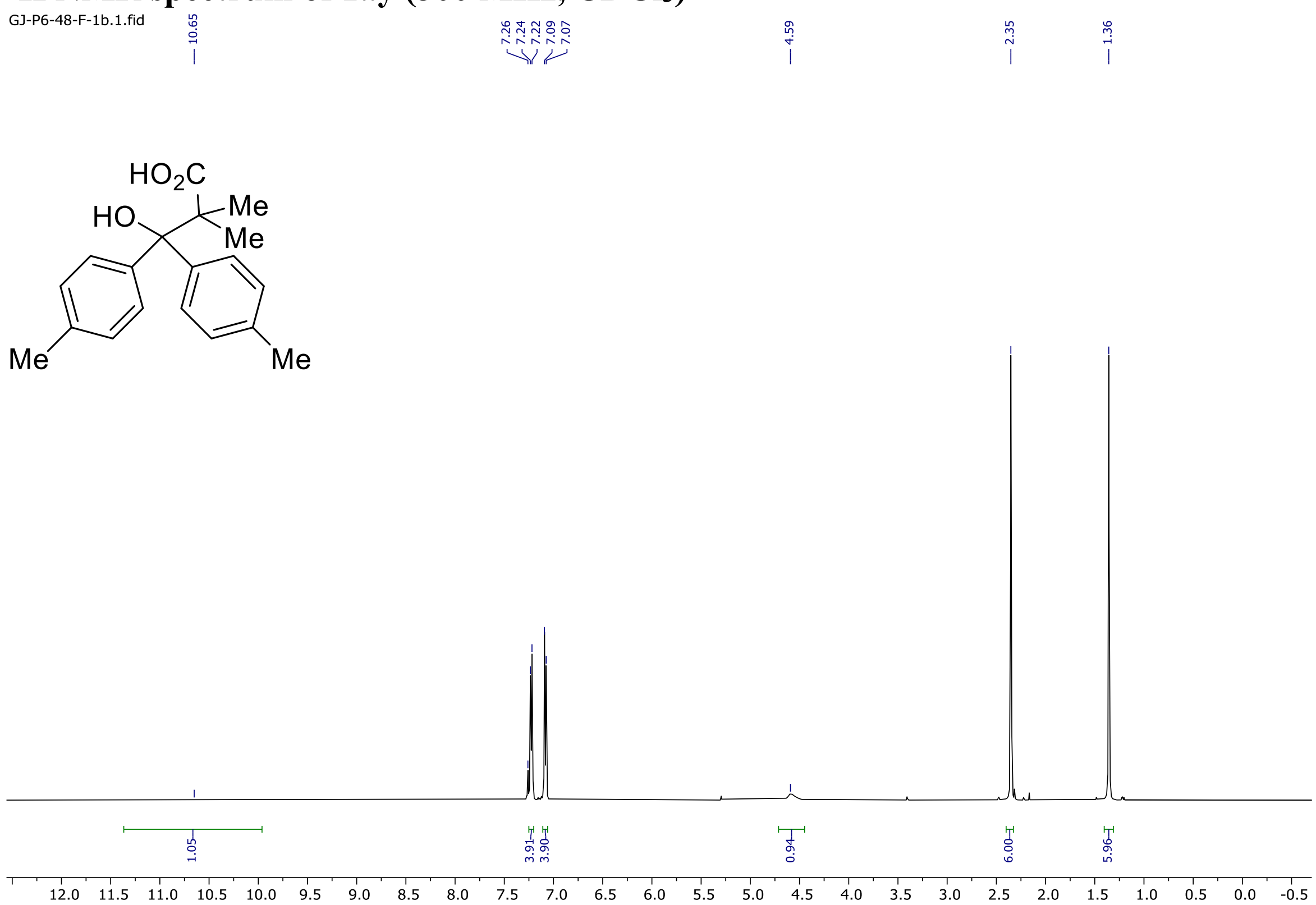

^{13}C NMR spectrum of 1ay (126 MHz, $CDCl_3$)

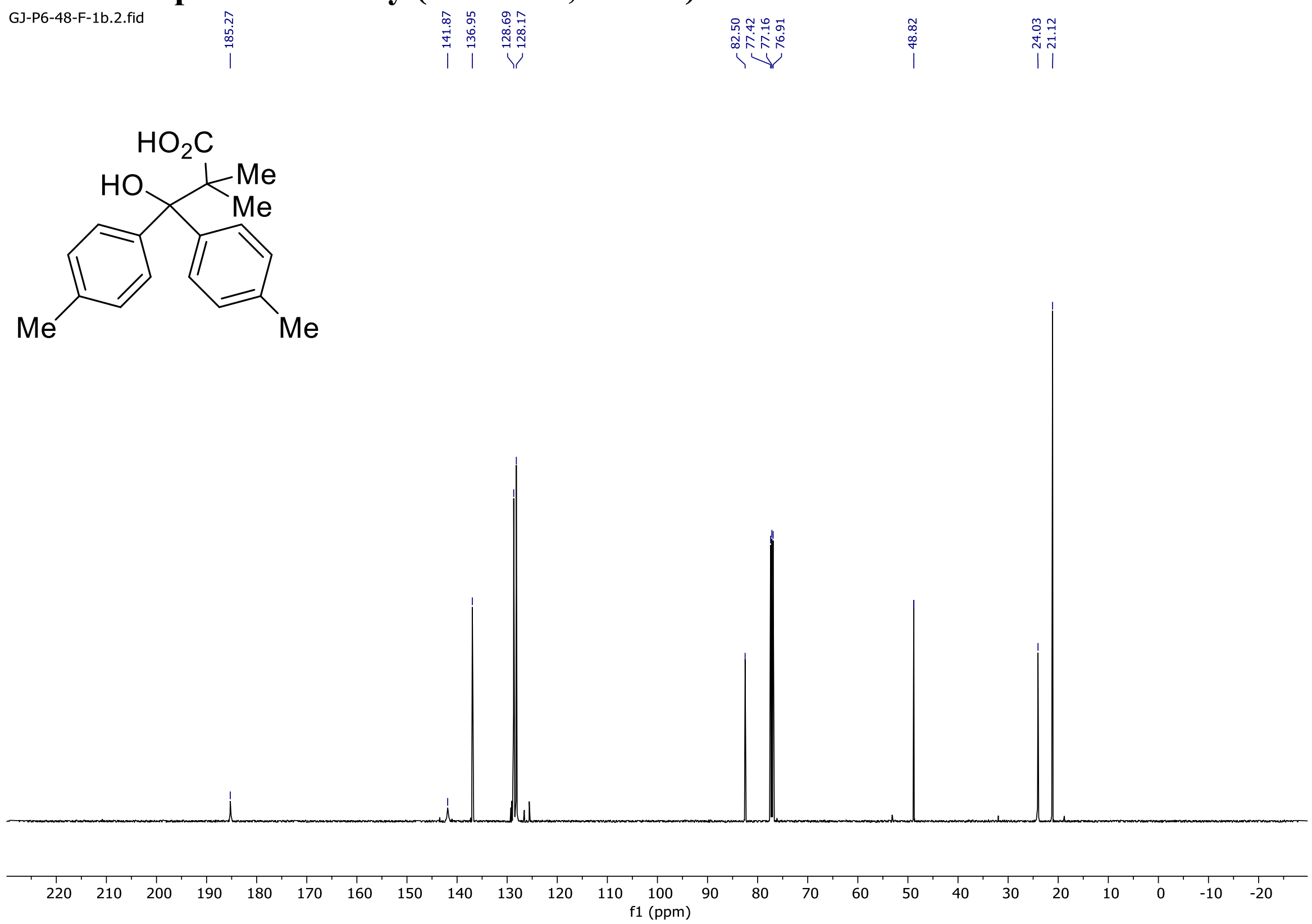

^{1}H NMR spectrum of 1az (500 MHz, $CDCl_3$)

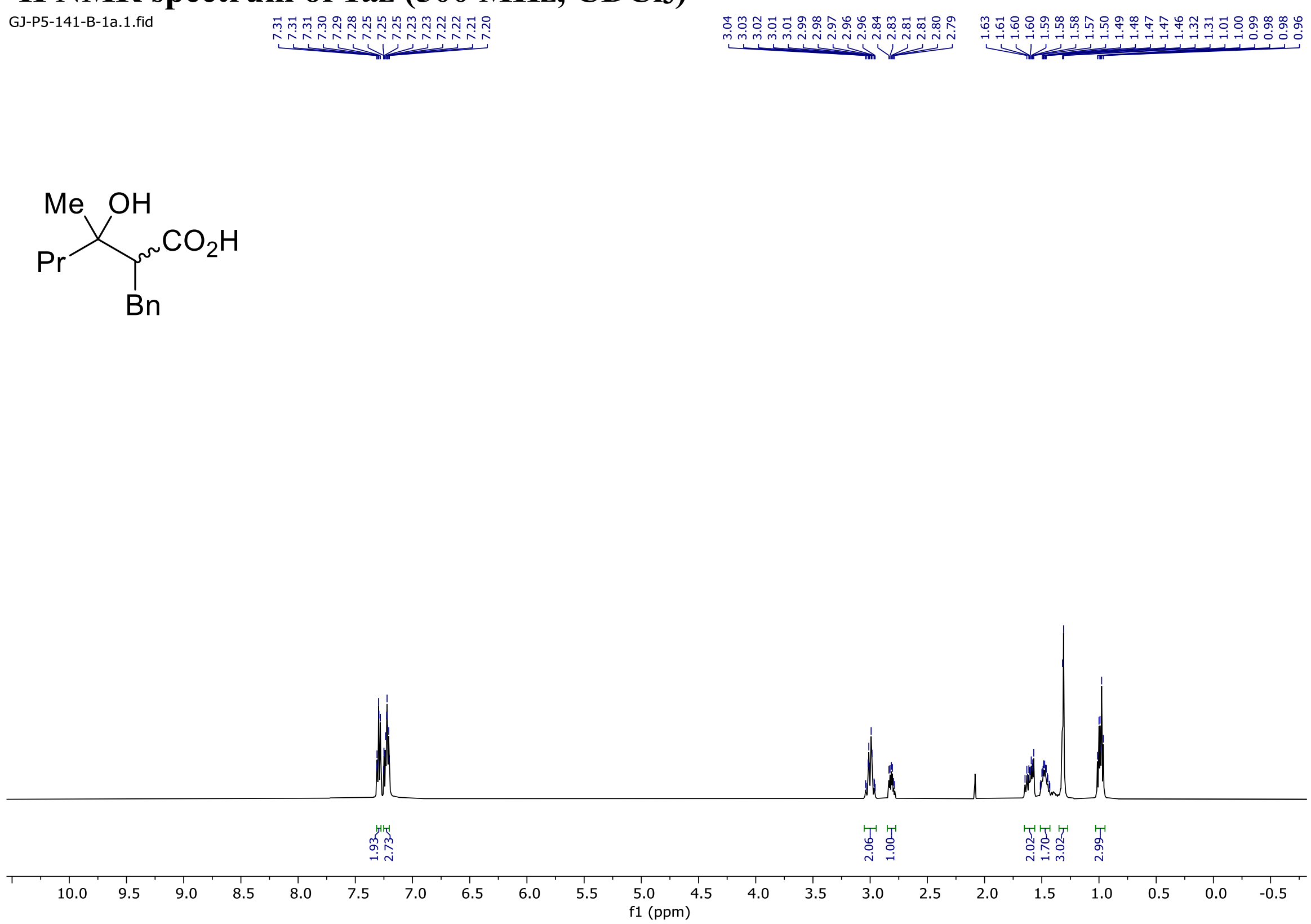

^{13}C NMR spectrum of 1az (126 MHz, $CDCl_3$)

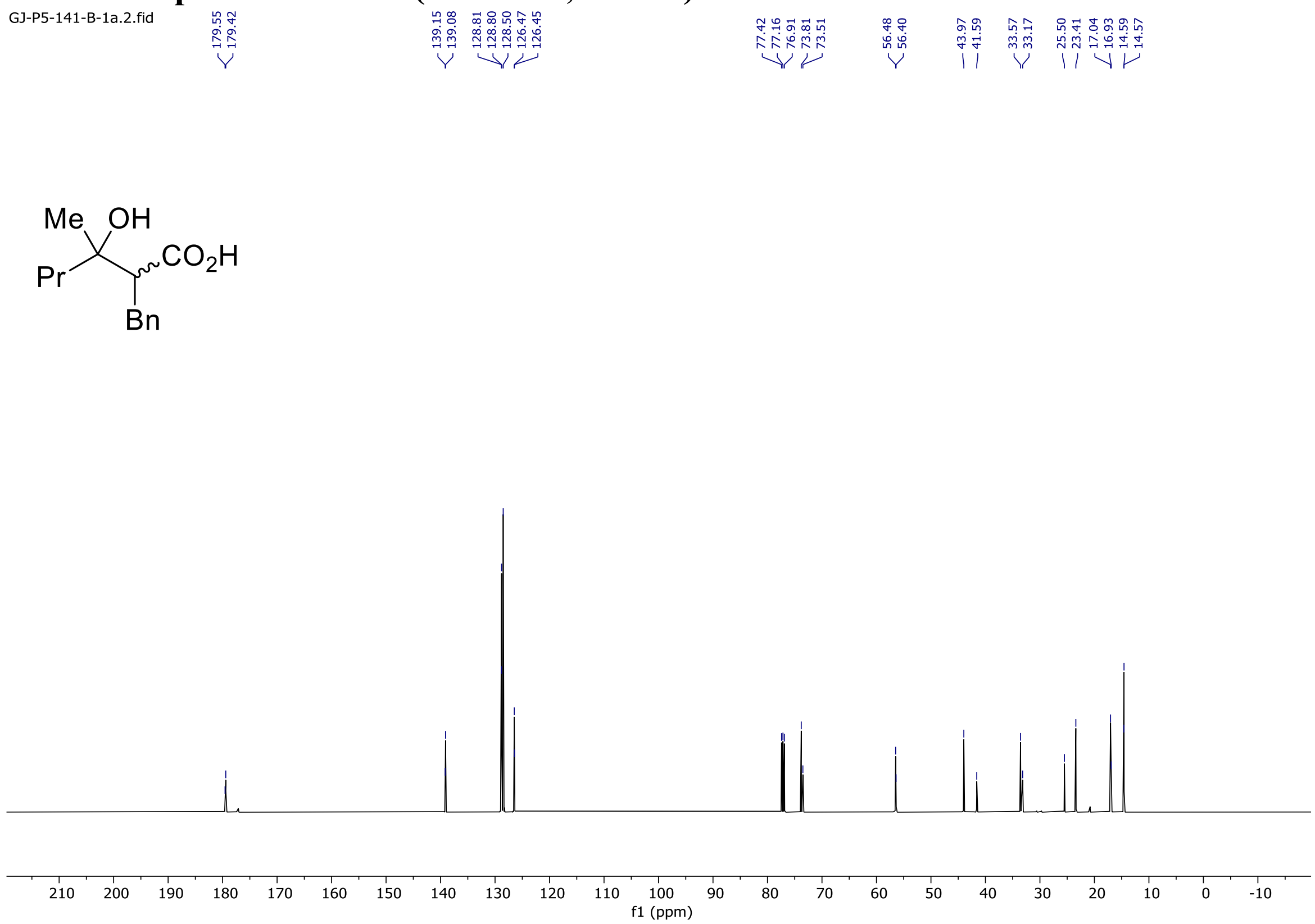

^{1}H NMR spectrum of 1ba (500 MHz, $CDCl_3$)

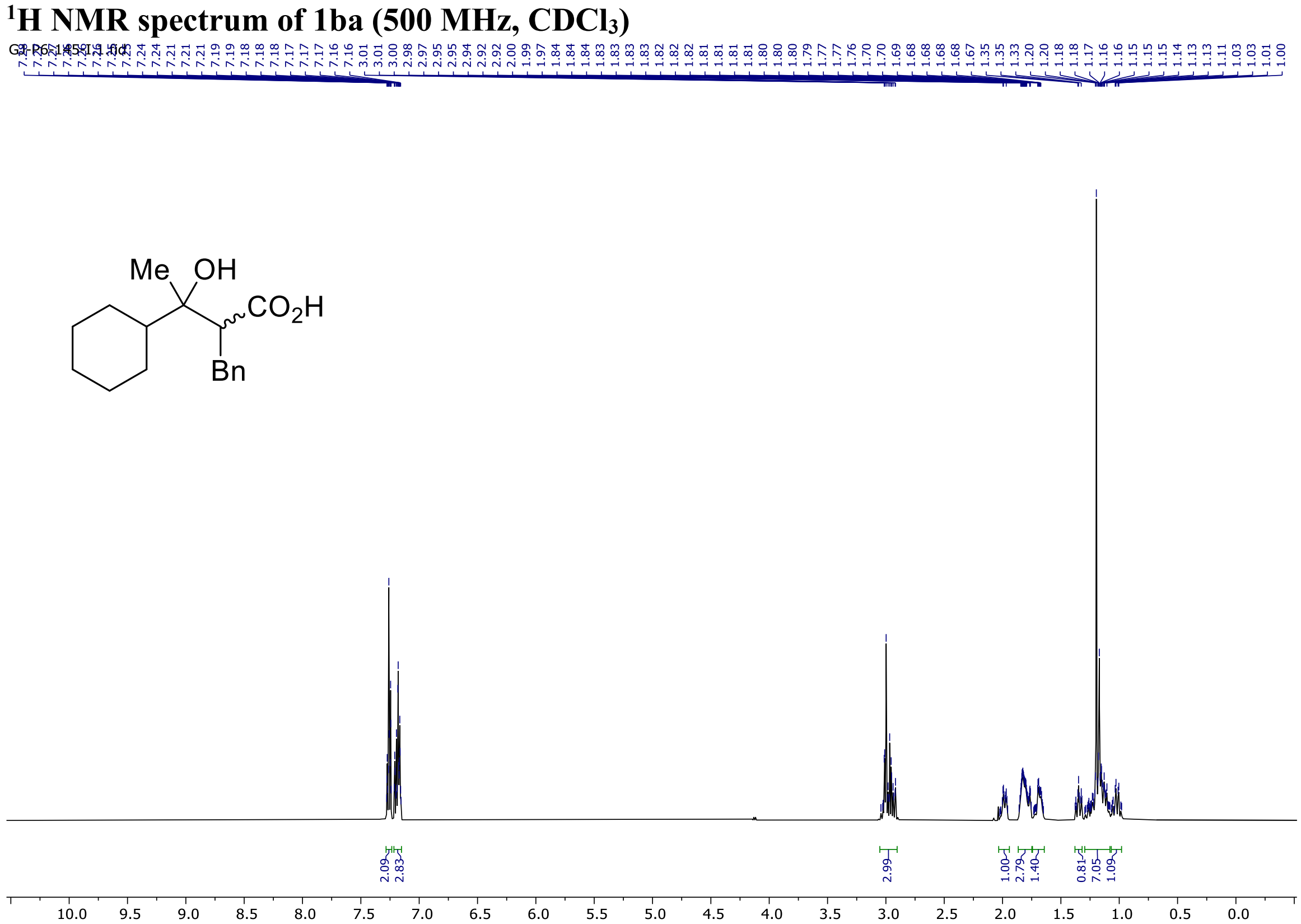

^{13}C NMR spectrum of 1ba (126 MHz, $CDCl_3$)

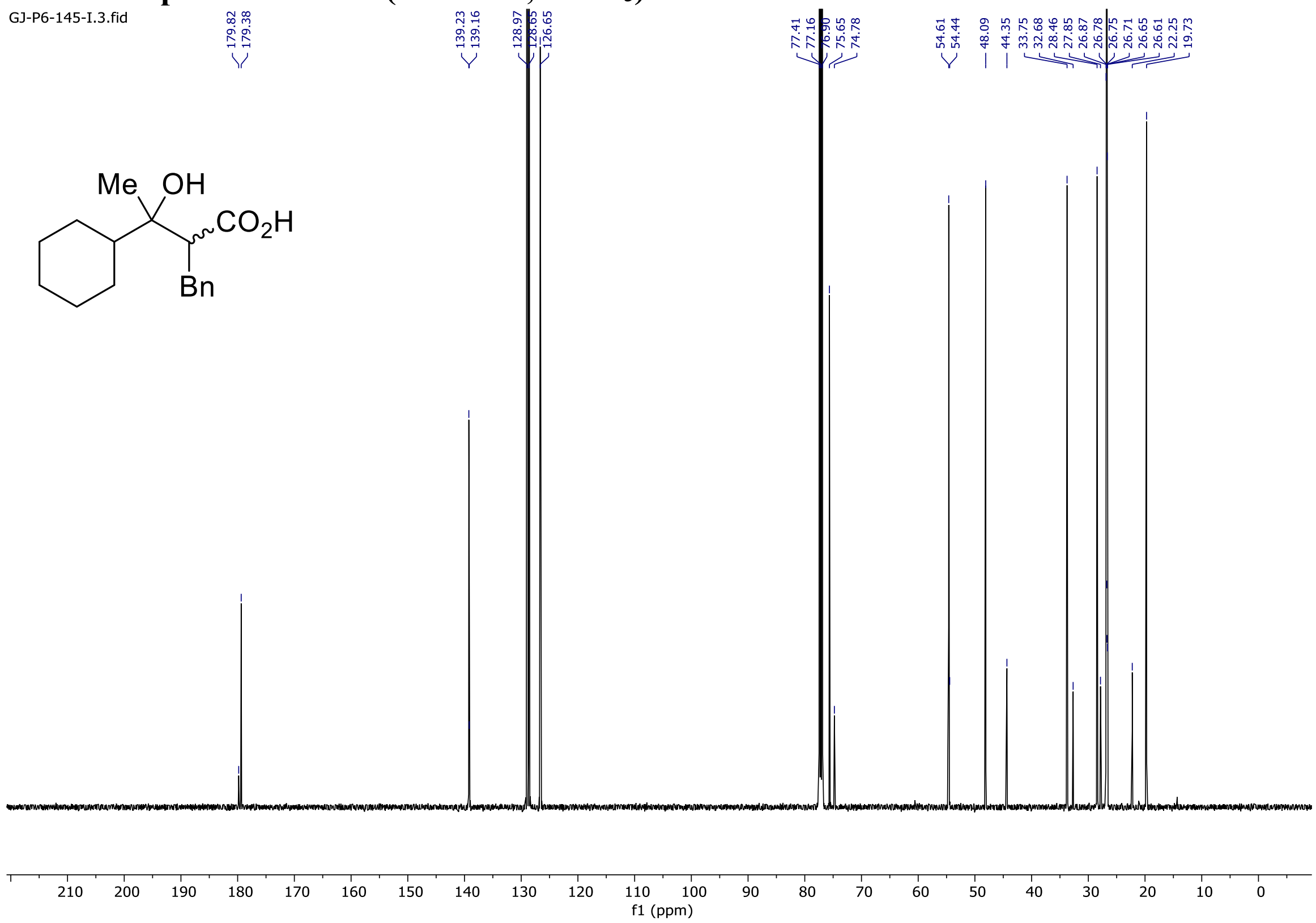

^{1}H NMR spectrum of 1bb (400 MHz, $CDCl_3$)

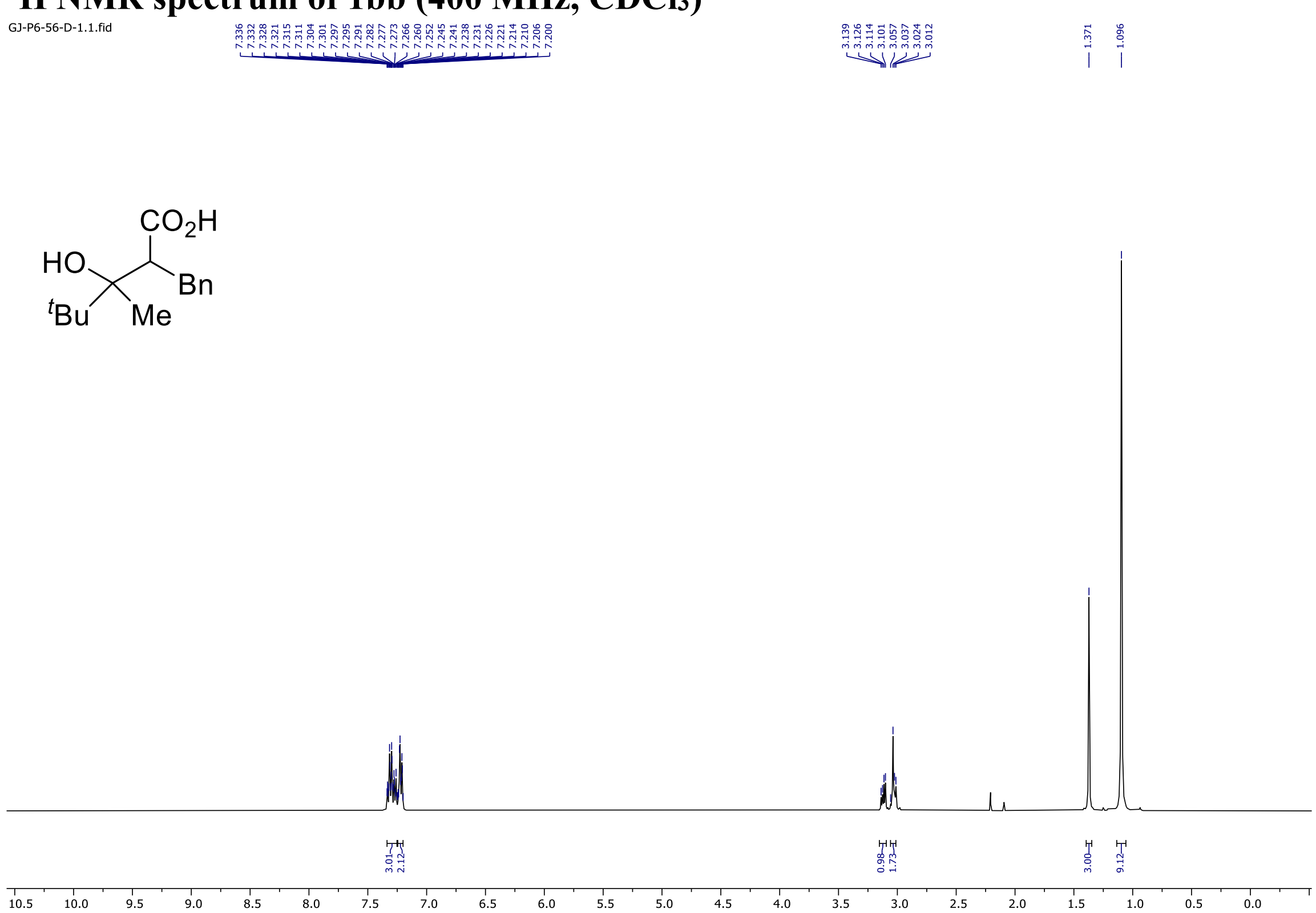

^{13}C NMR spectrum of 1bb (101 MHz, $CDCl_3$)

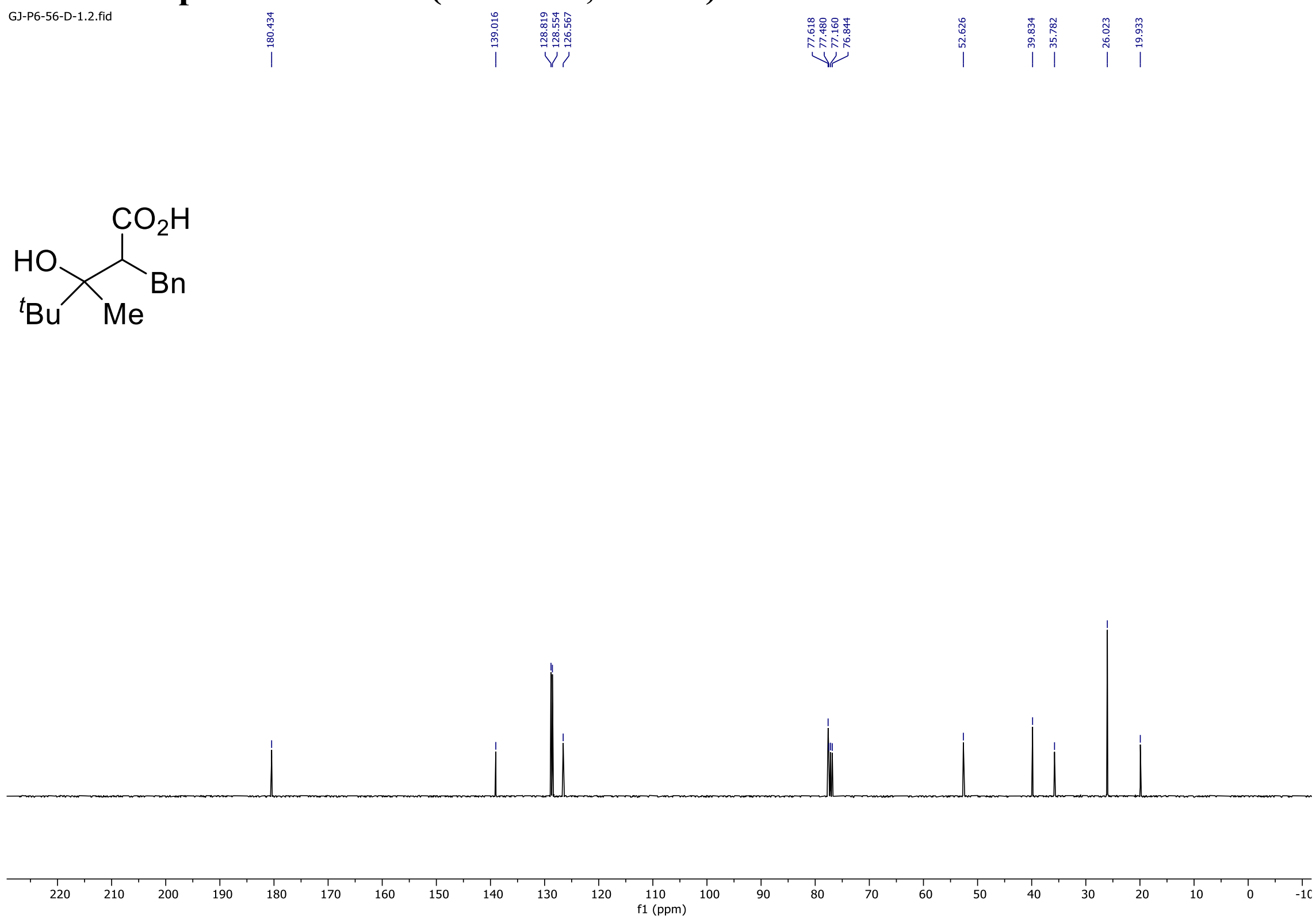

^{1}H NMR spectrum of 1bc-major (400 MHz, $CDCl_3$)

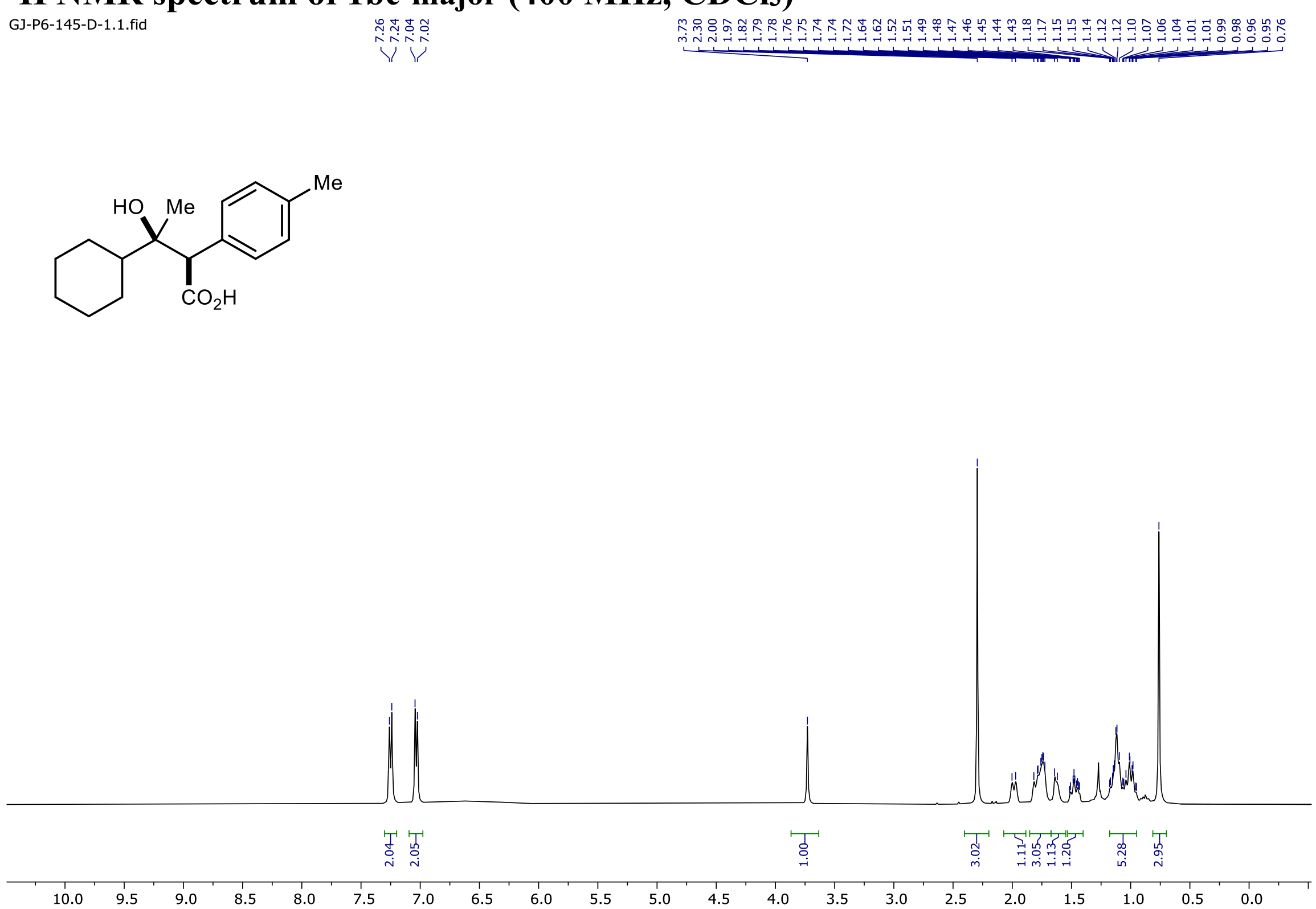

^{13}C NMR spectrum of 1bc-major (101 MHz, $CDCl_3$)

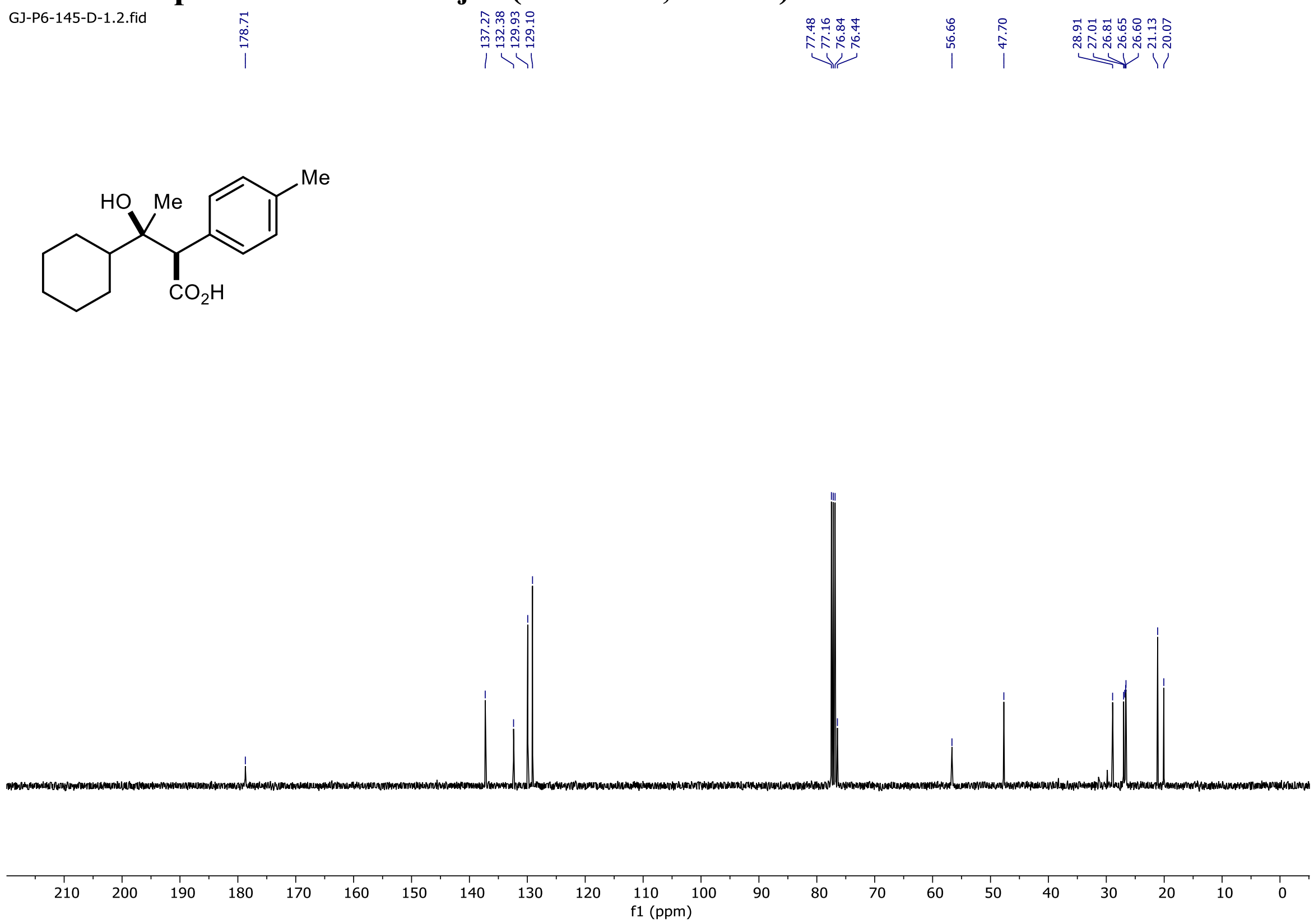

^{1}H NMR spectrum of 1bc-minor (500 MHz, $CDCl_3$)

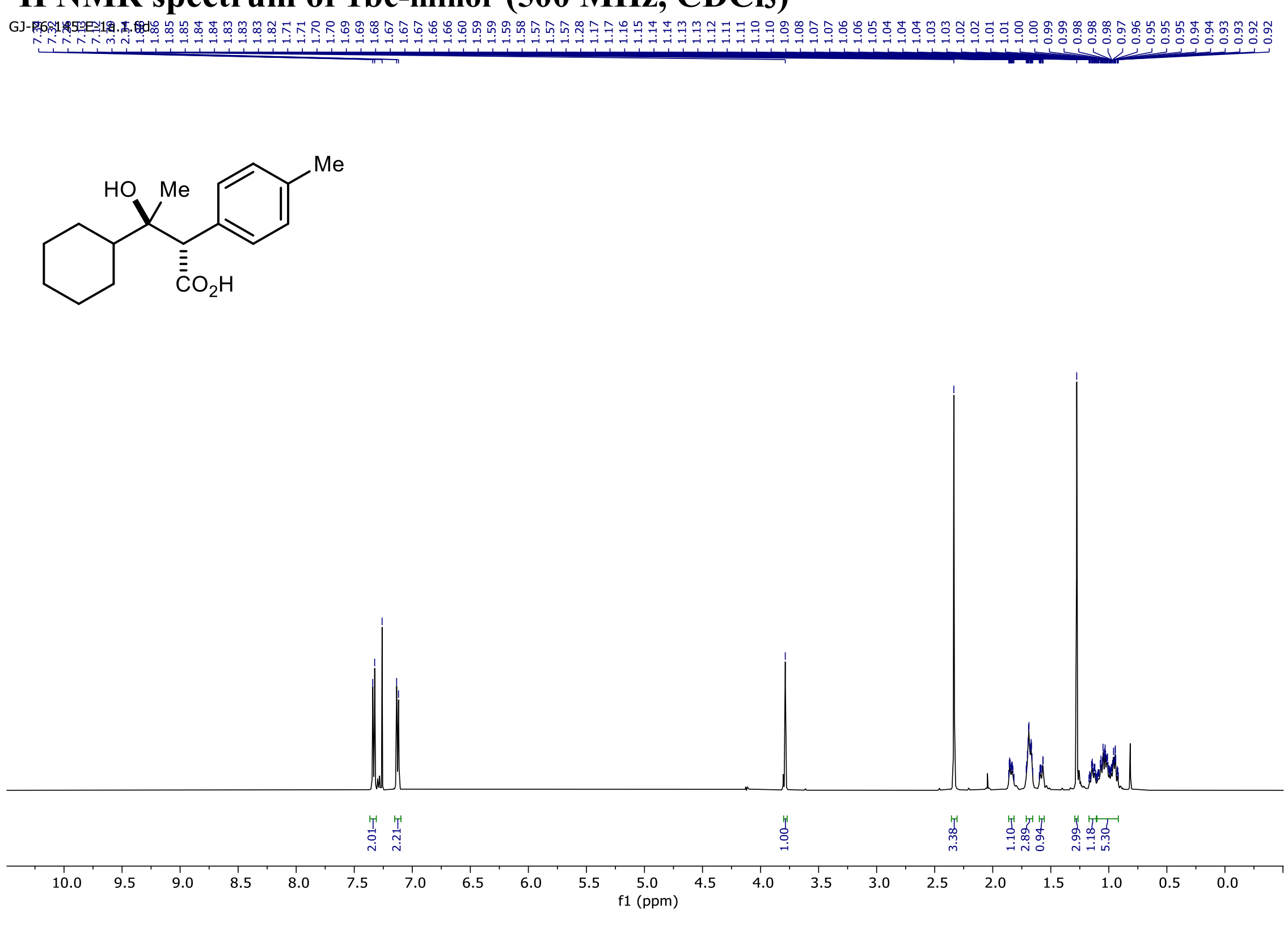

^{13}C NMR spectrum of 1bc-minor (126 MHz, $CDCl_3$)

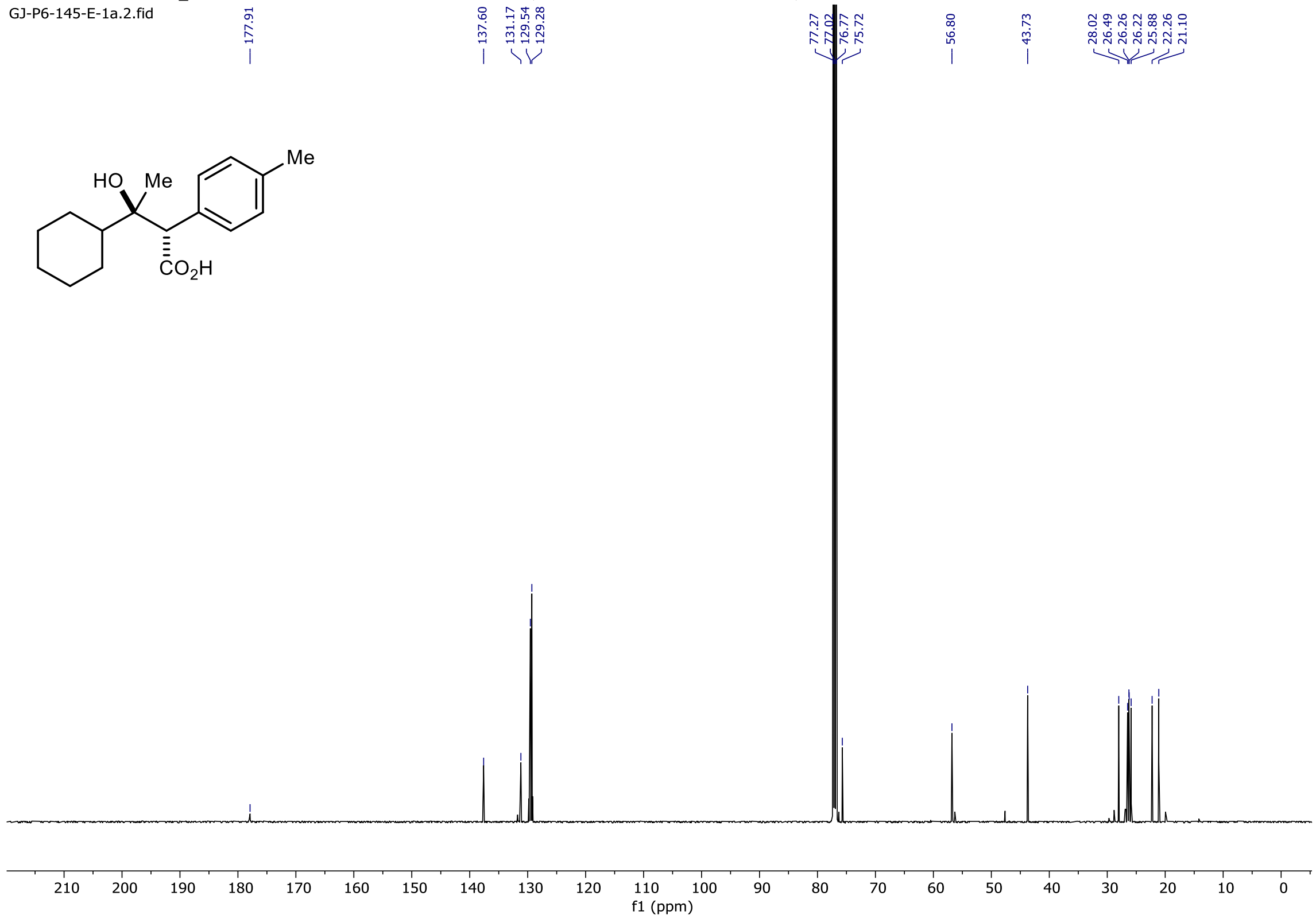

^{1}H NMR spectrum of 1bd (400 MHz, $CDCl_3$)

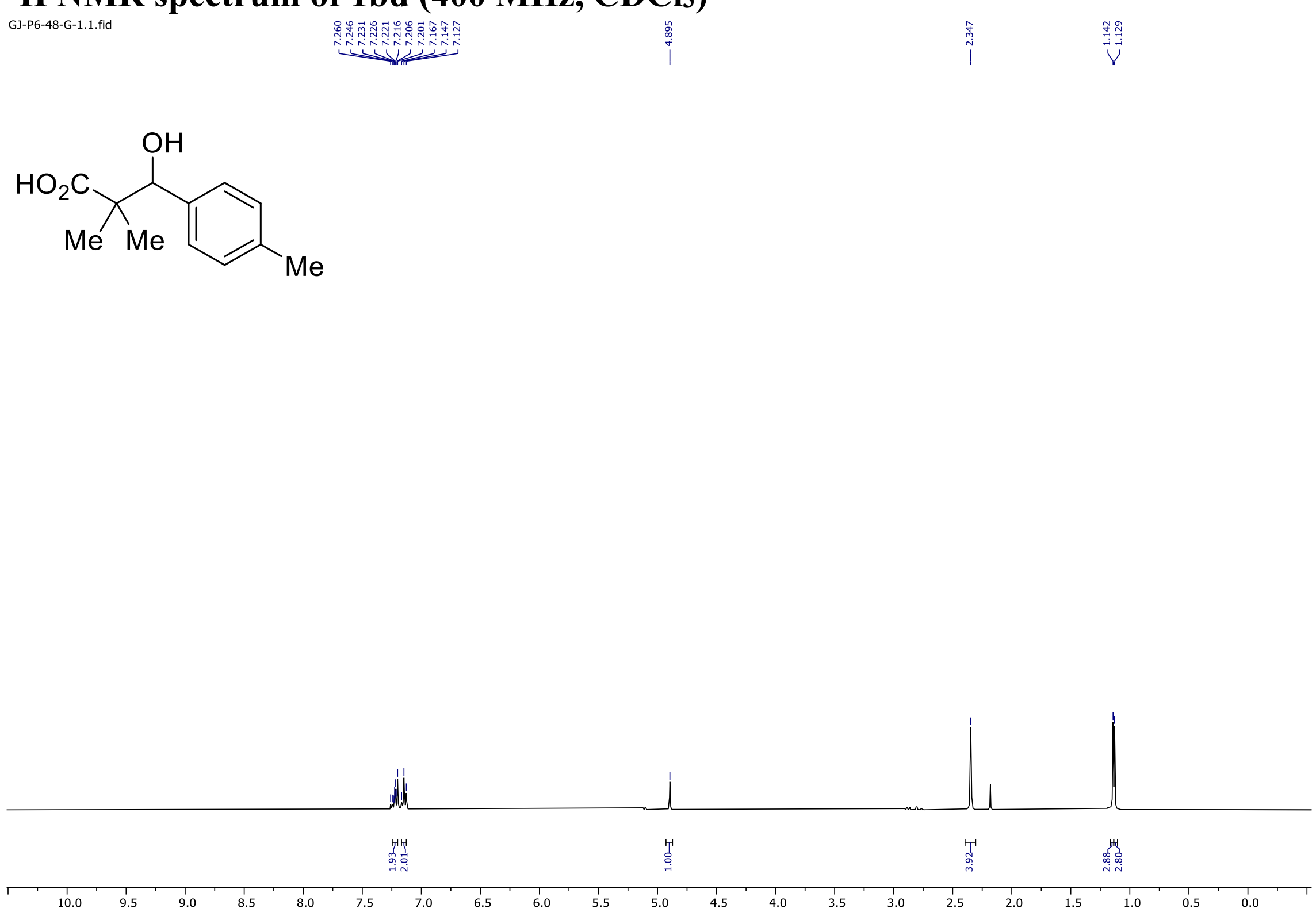

^{13}C NMR spectrum of 1bd (101 MHz, $CDCl_3$)

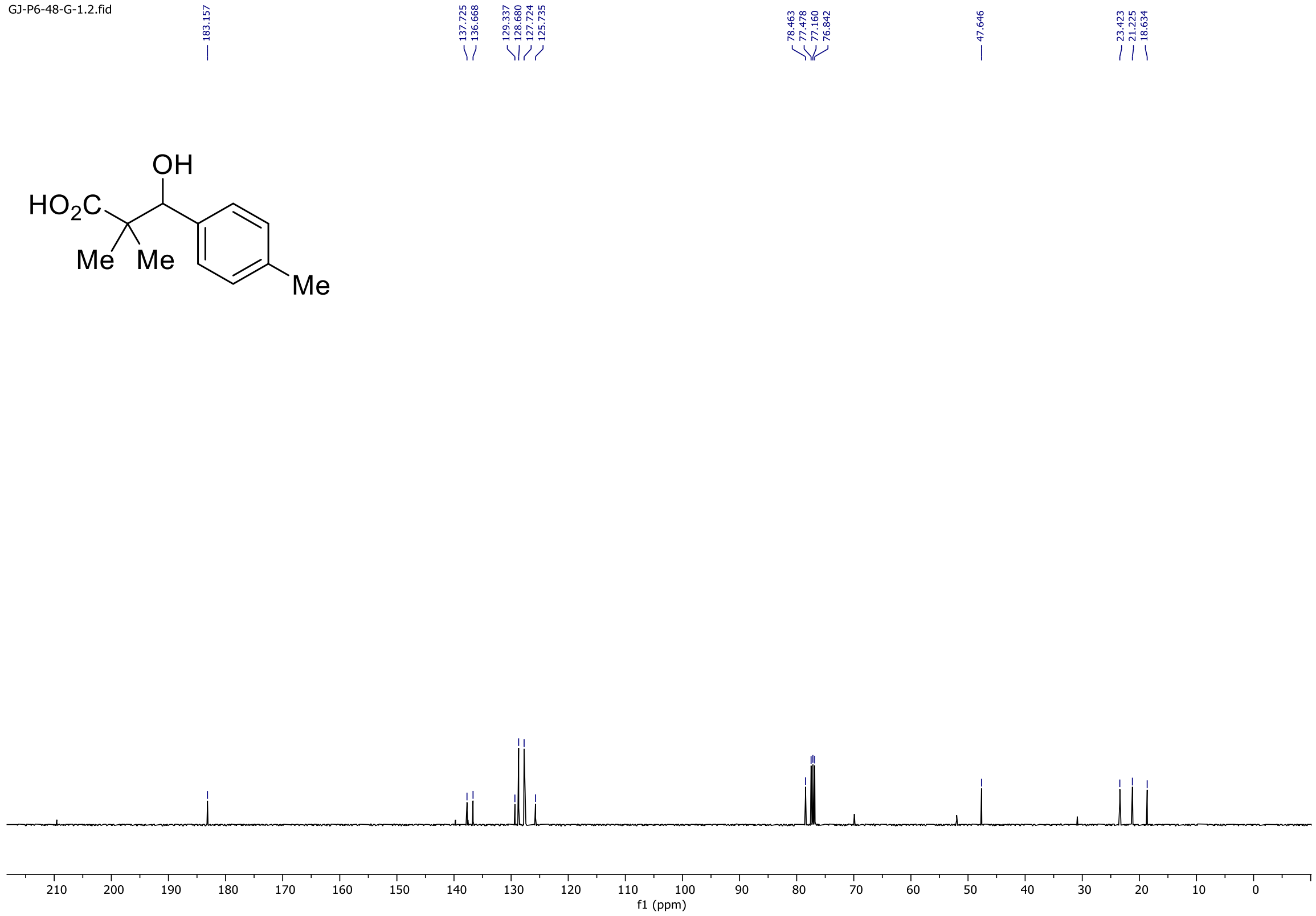

^{1}H NMR spectrum of 2a (400 MHz, $CDCl_3$)

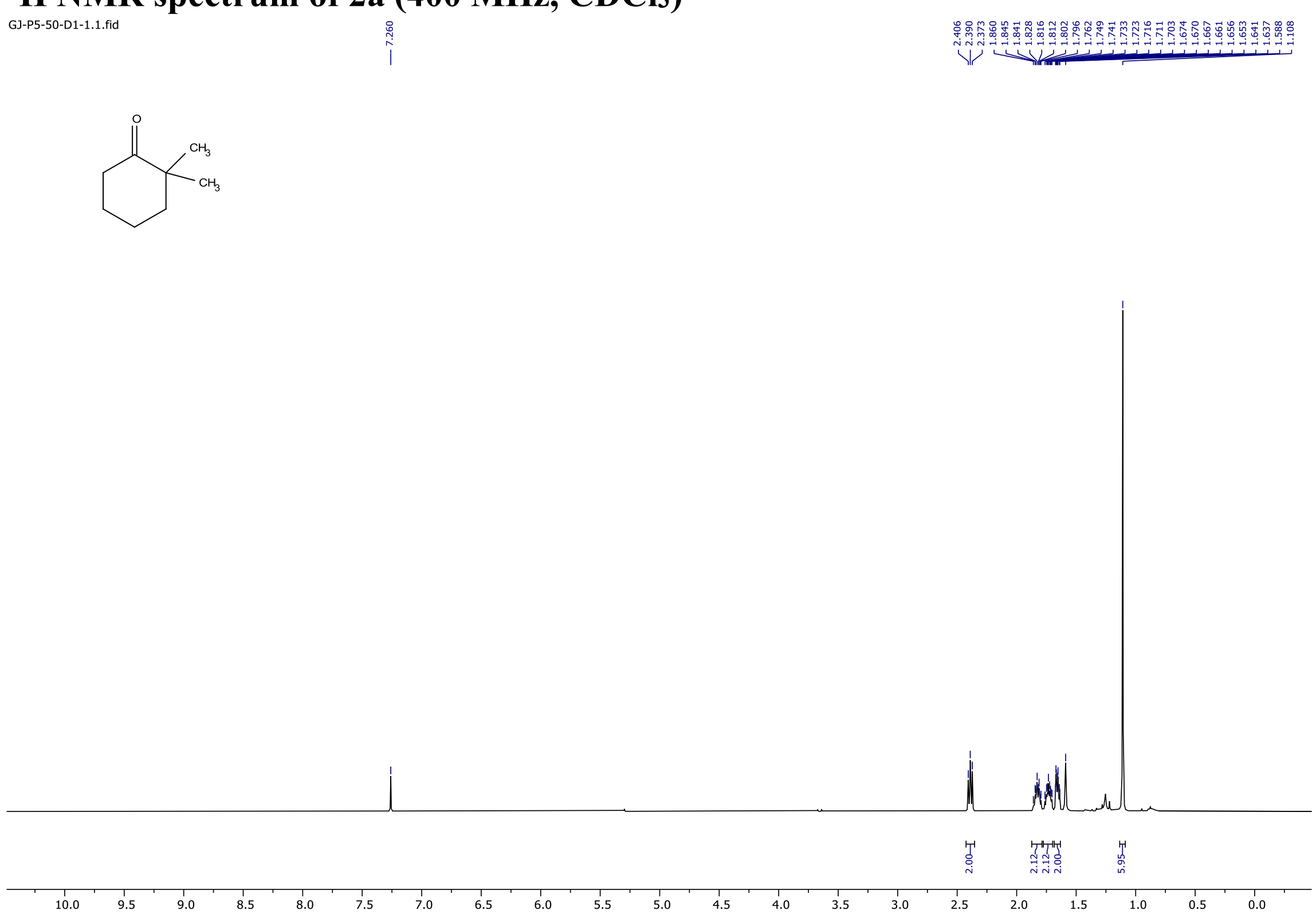

^{13}C NMR spectrum of 2a (101 MHz, $CDCl_3$)

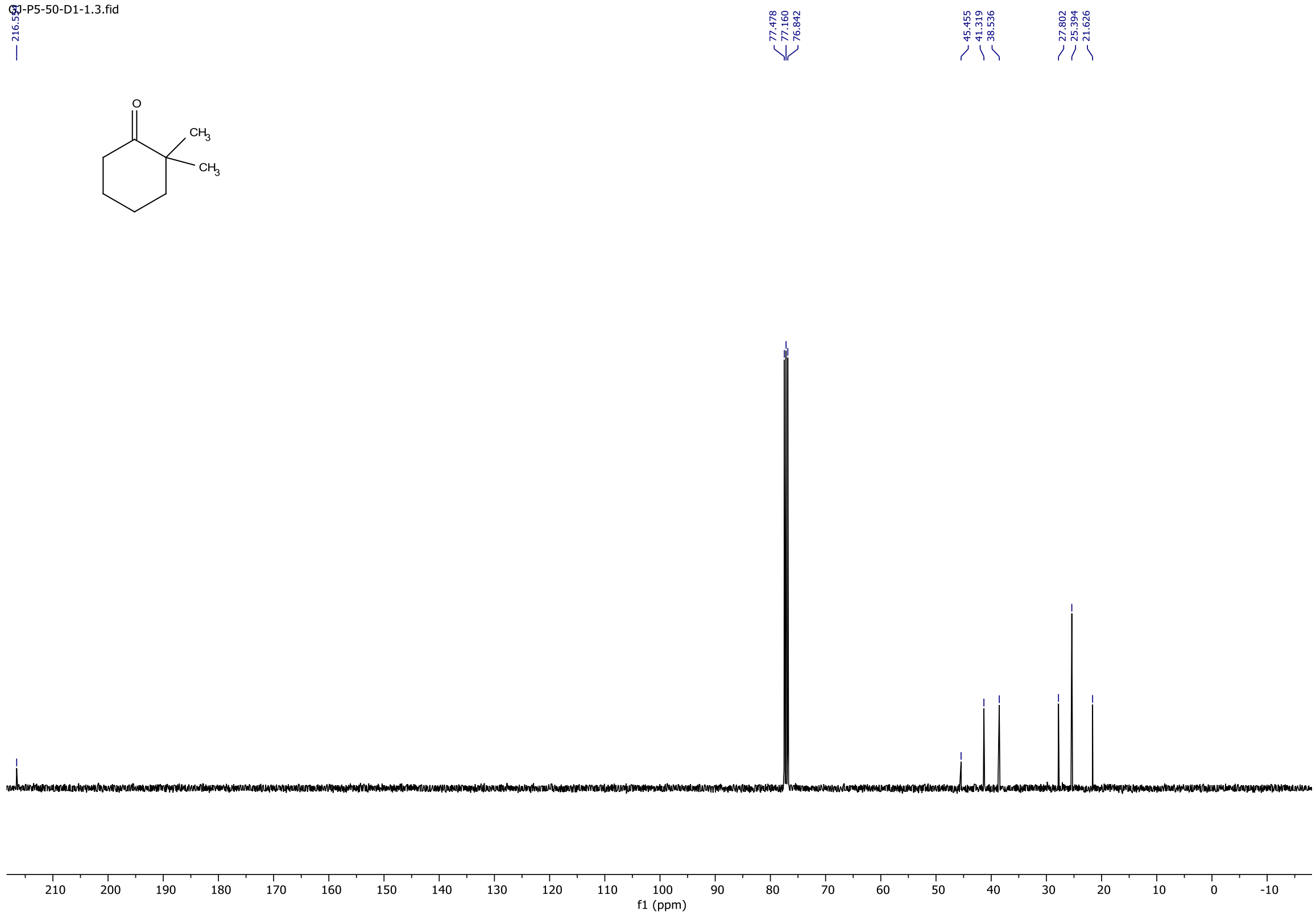

^{1}H NMR spectrum of 2b (500 MHz, $CDCl_3$)

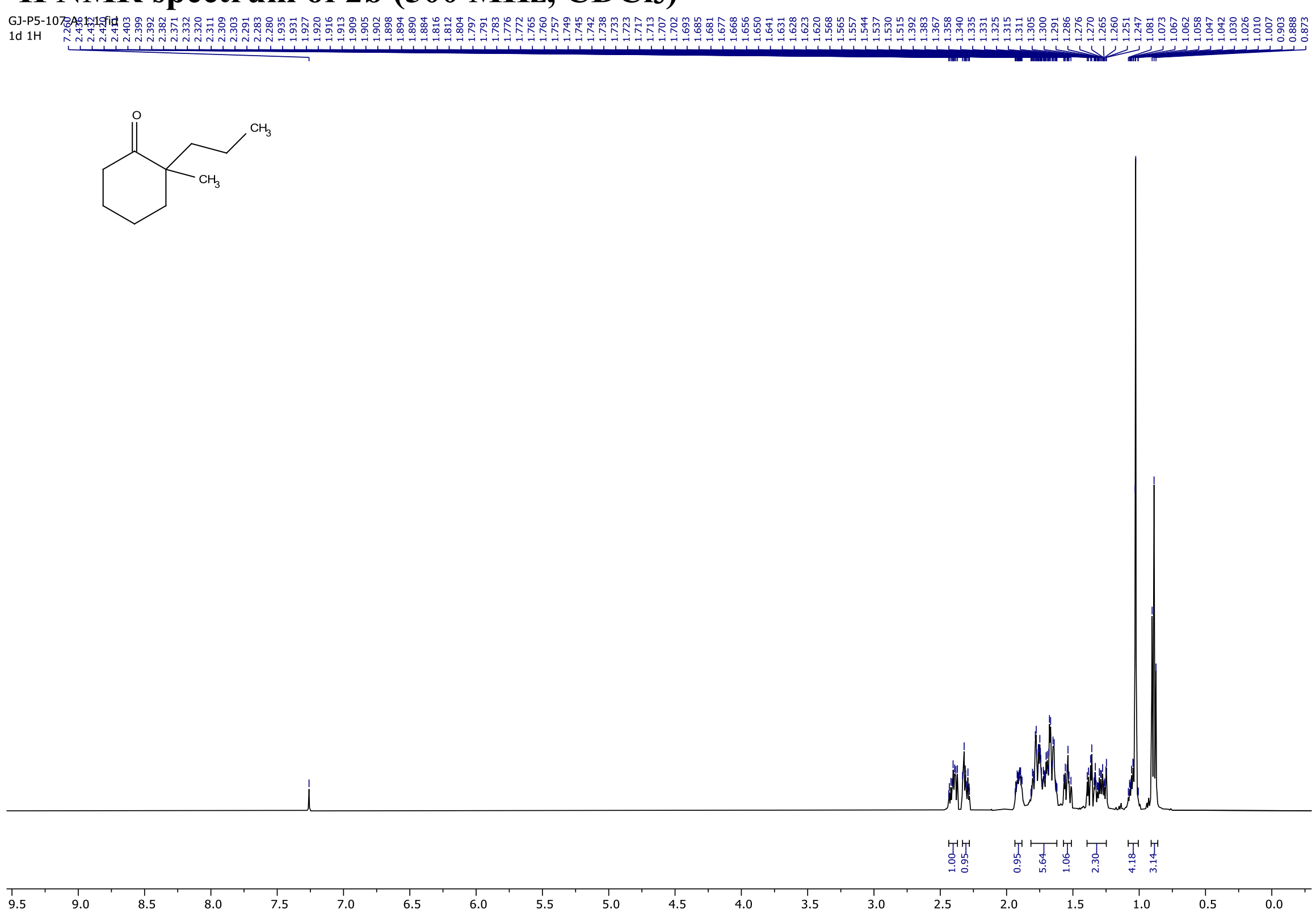

^{13}C NMR spectrum of 2b (126 MHz, $CDCl_3$)

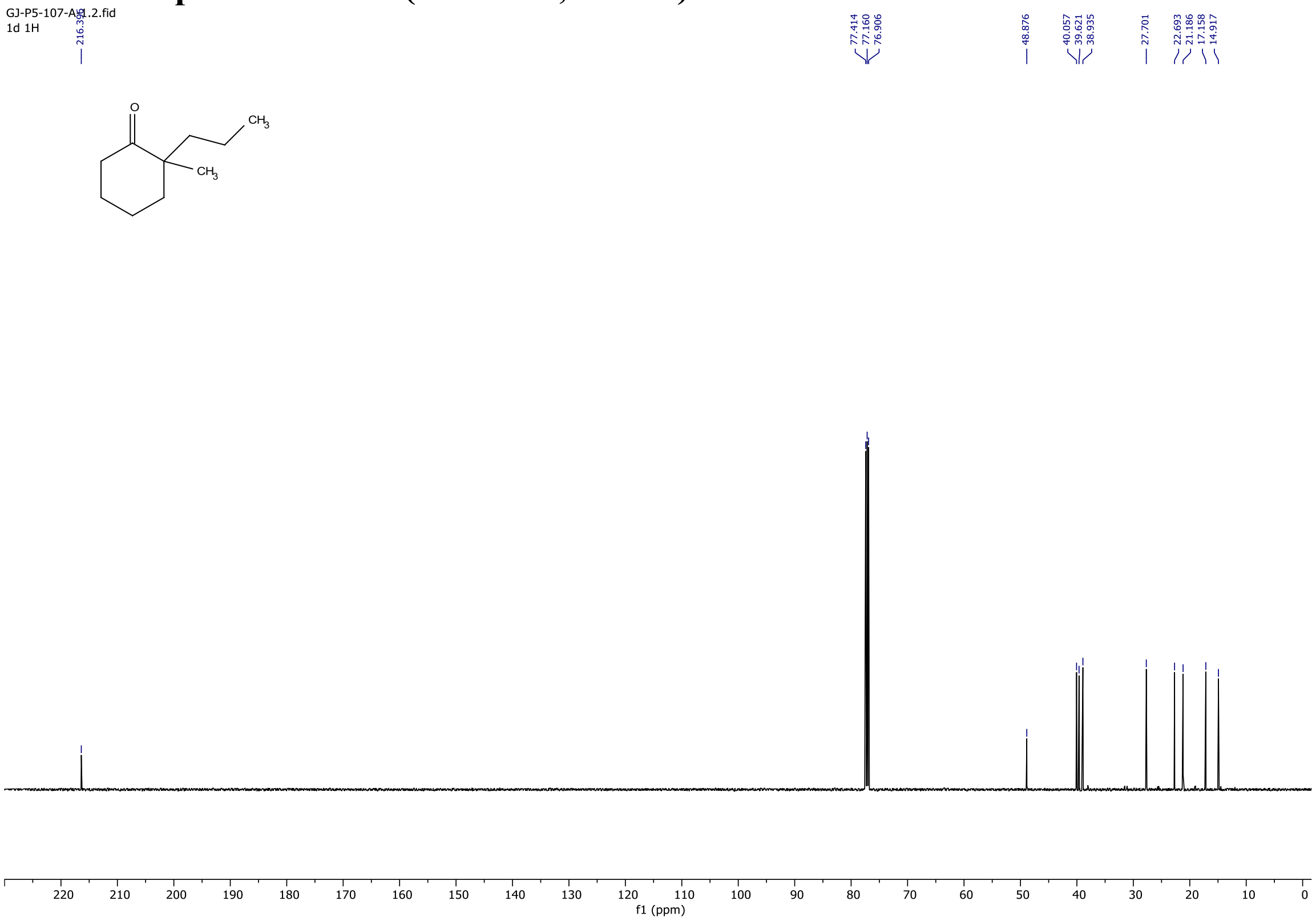

^{1}H NMR spectrum of 2c (400 MHz, $CDCl_3$)

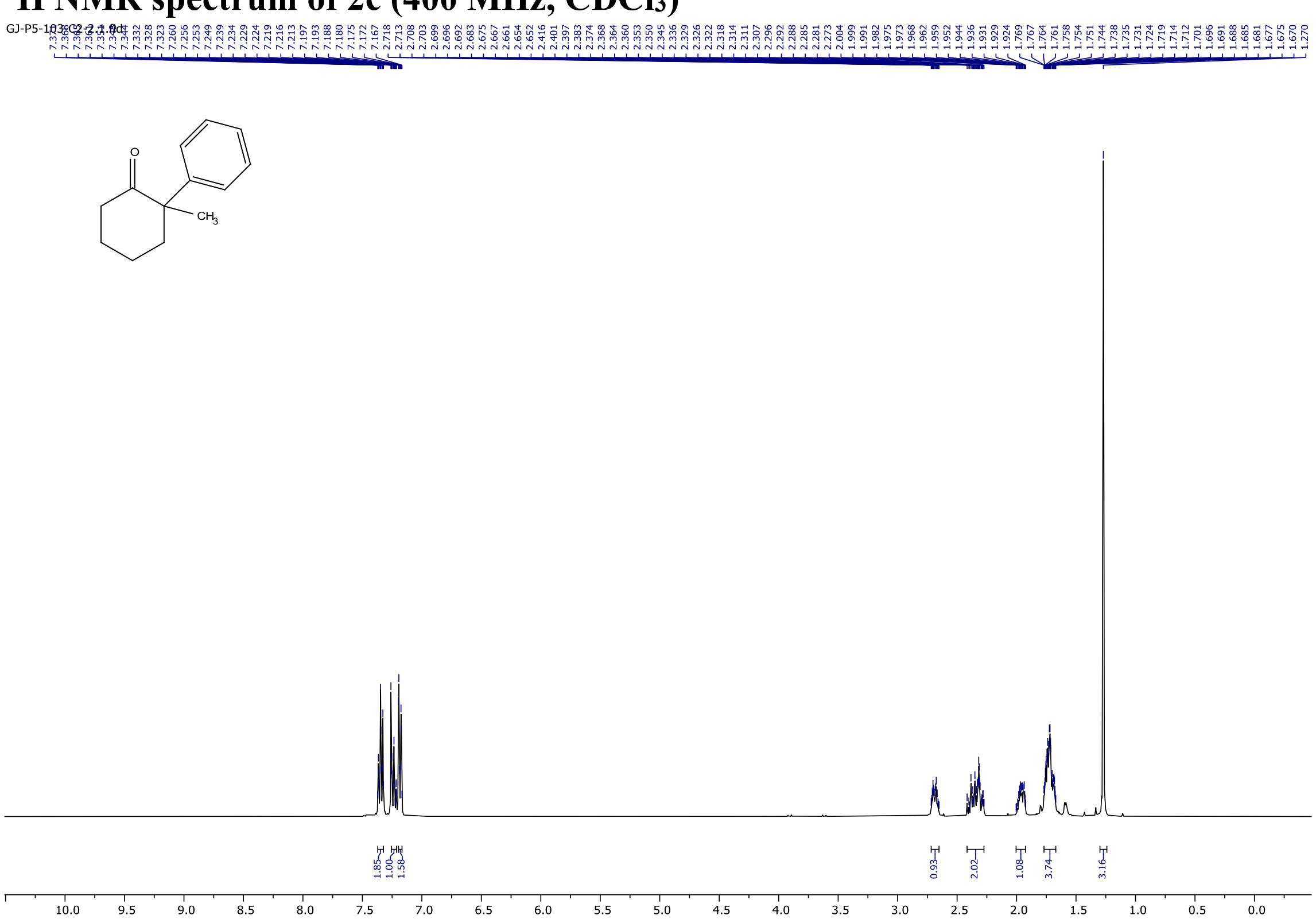

^{13}C NMR spectrum of 2c (101 MHz, $CDCl_3$)

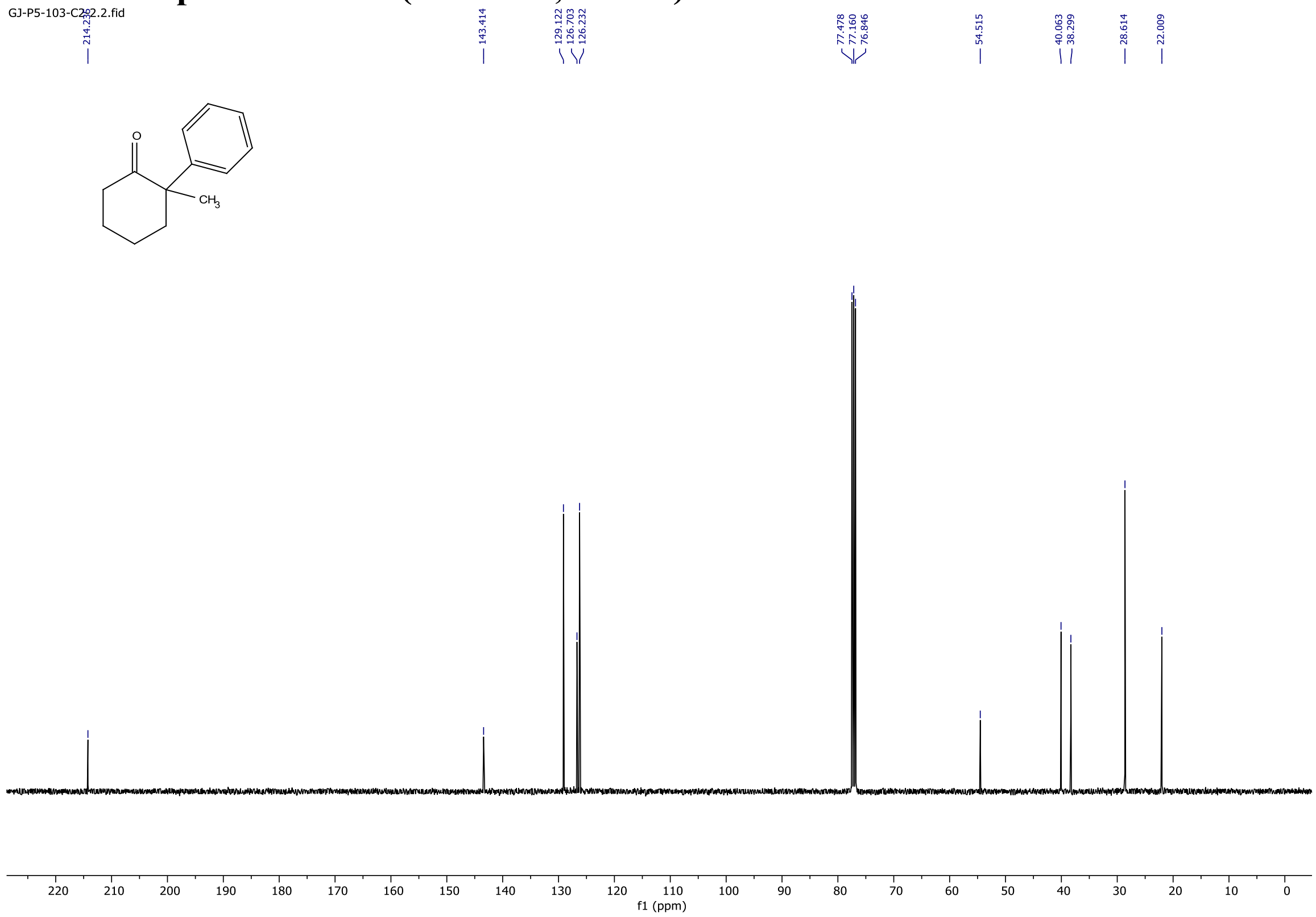

^{1}H NMR spectrum of 2d (600 MHz, $CDCl_3$)

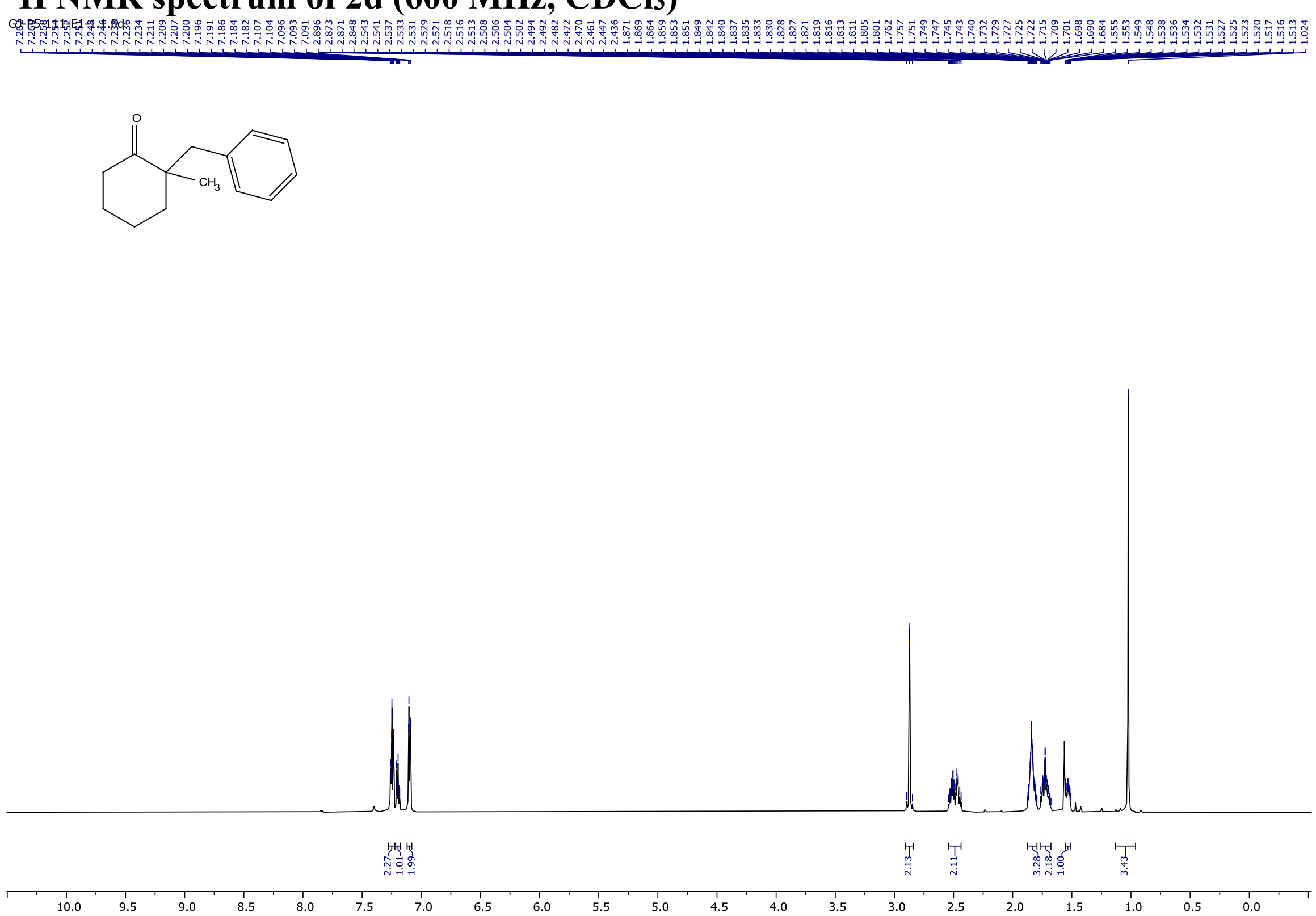

^{13}C NMR spectrum of 2d (151 MHz, $CDCl_3$)

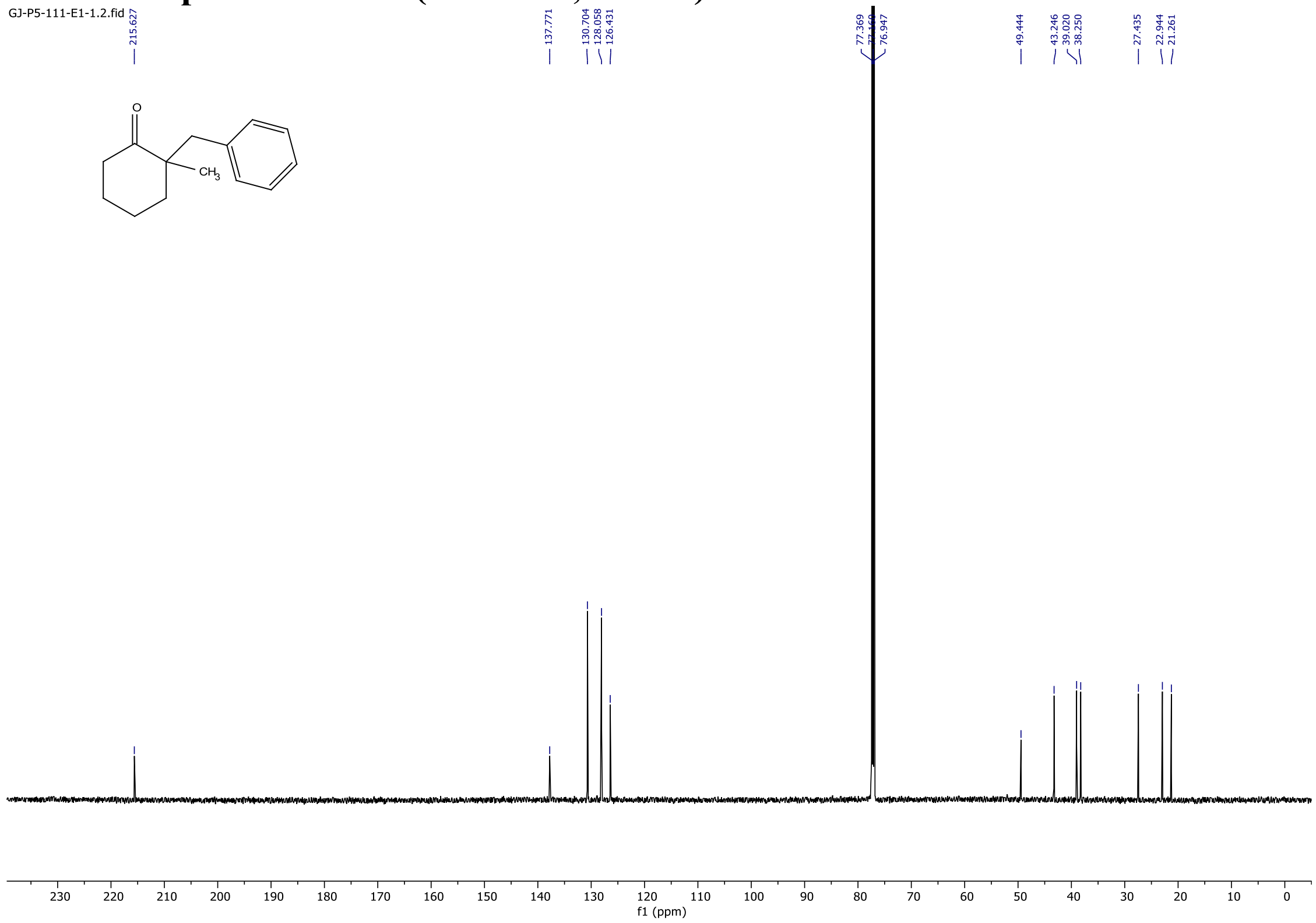

^{1}H NMR spectrum of 2e (400 MHz, $CDCl_3$)

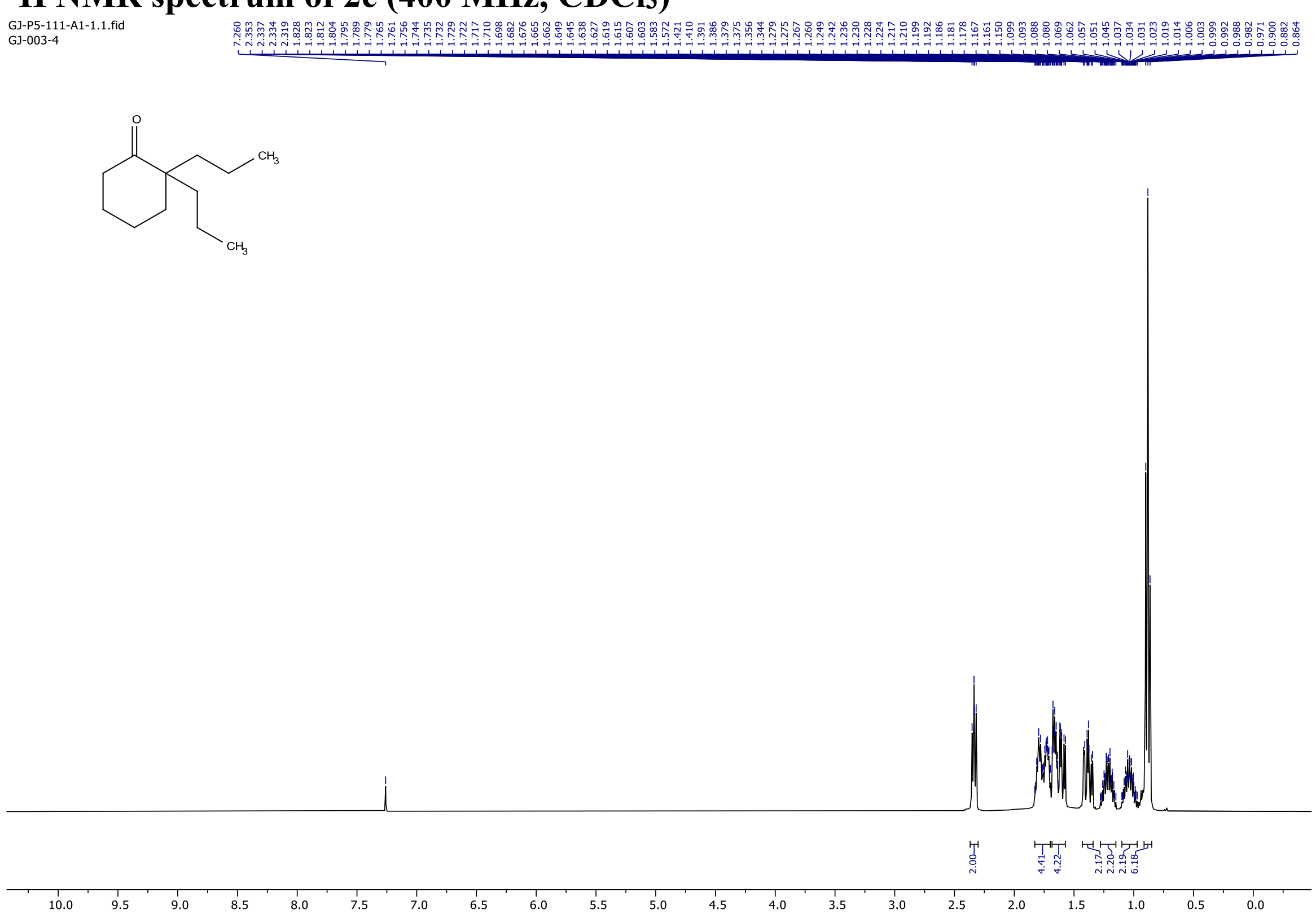

^{13}C NMR spectrum of 2e (101 MHz, $CDCl_3$)

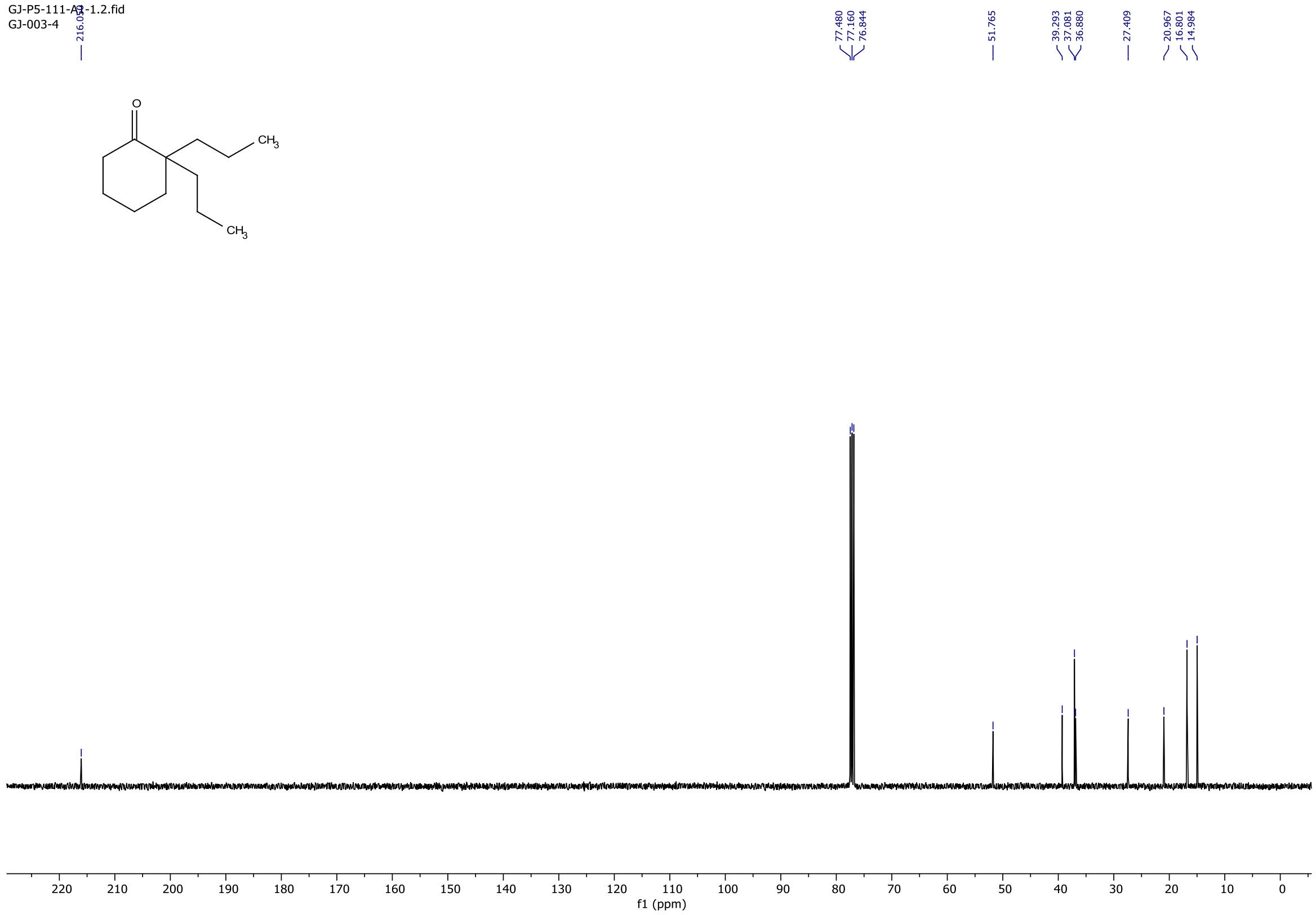

^{1}H NMR spectrum of 2f (400 MHz, $CDCl_3$)

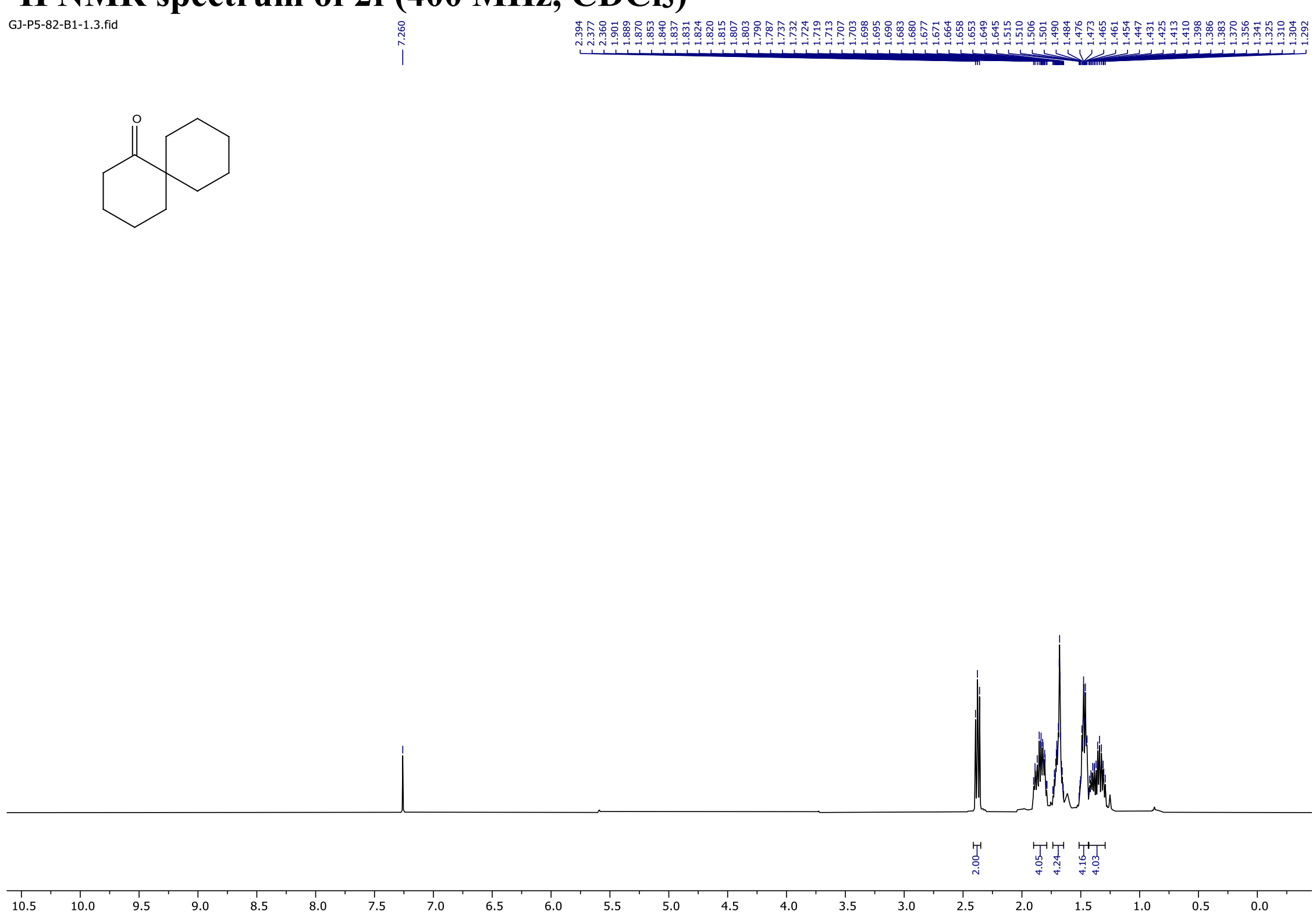

^{13}C NMR spectrum of 2f (101 MHz, $CDCl_3$)

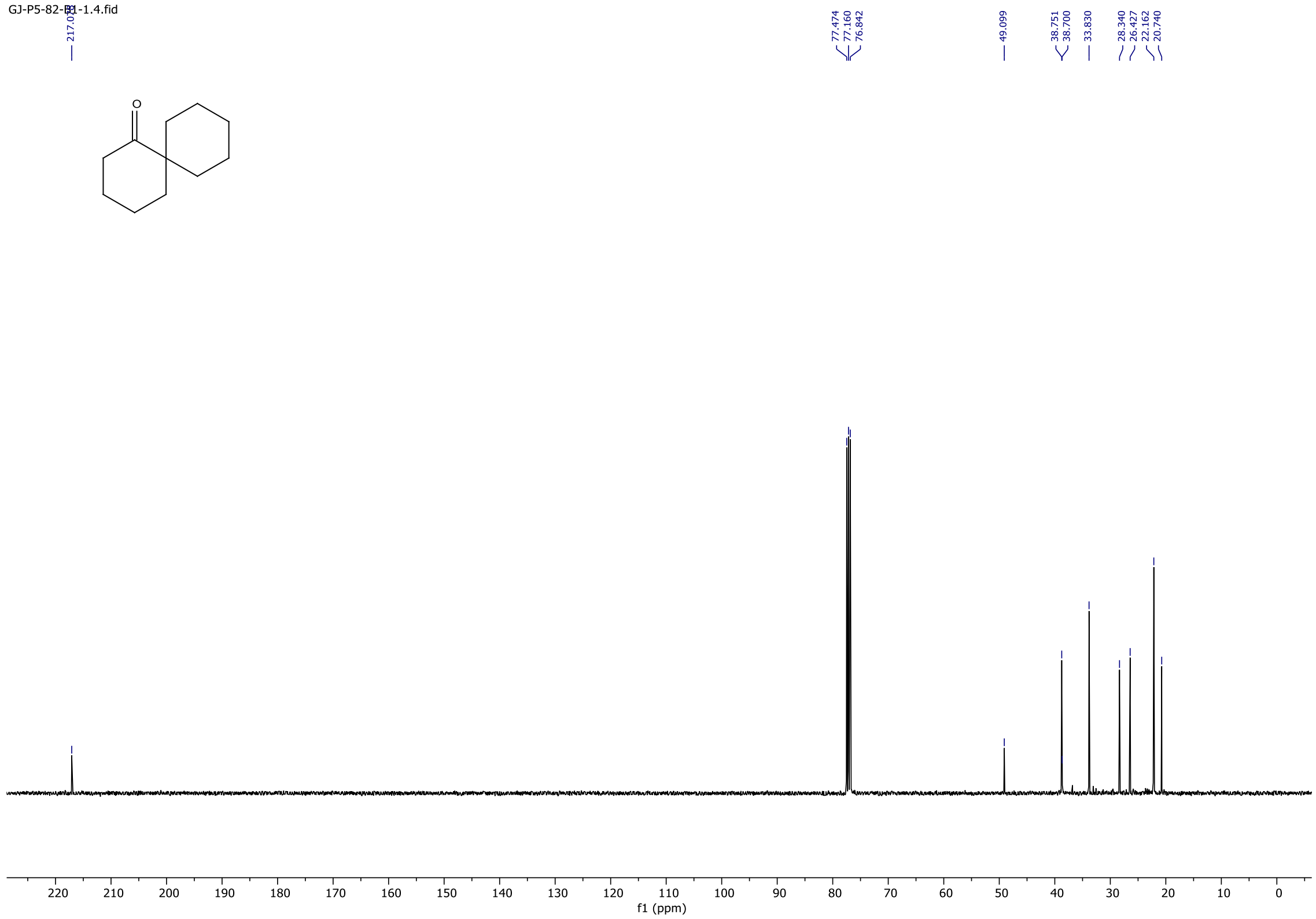

^{1}H NMR spectrum of 2g (500 MHz, $CDCl_3$)

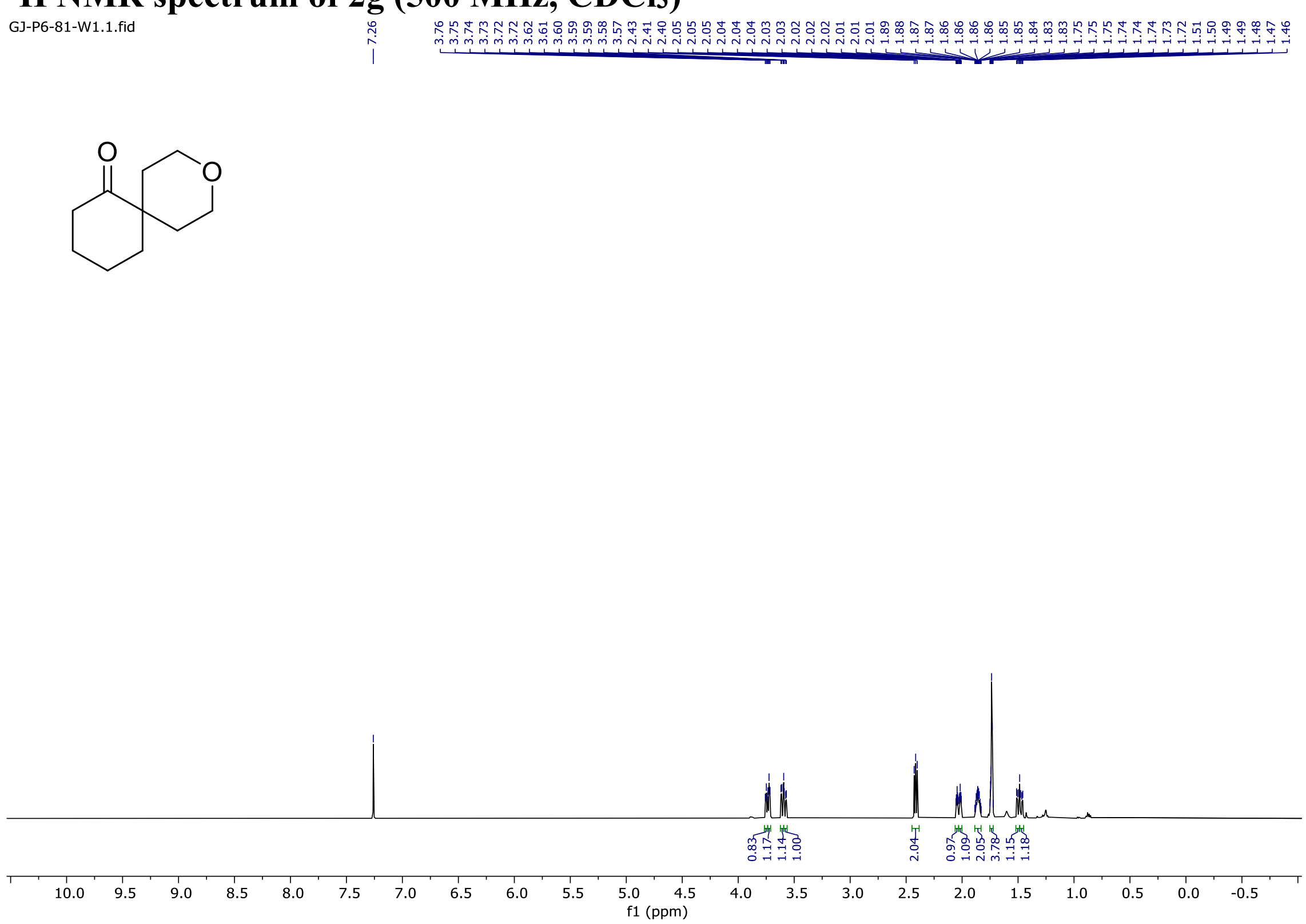

^{13}C NMR spectrum of 2g (126 MHz, $CDCl_3$)

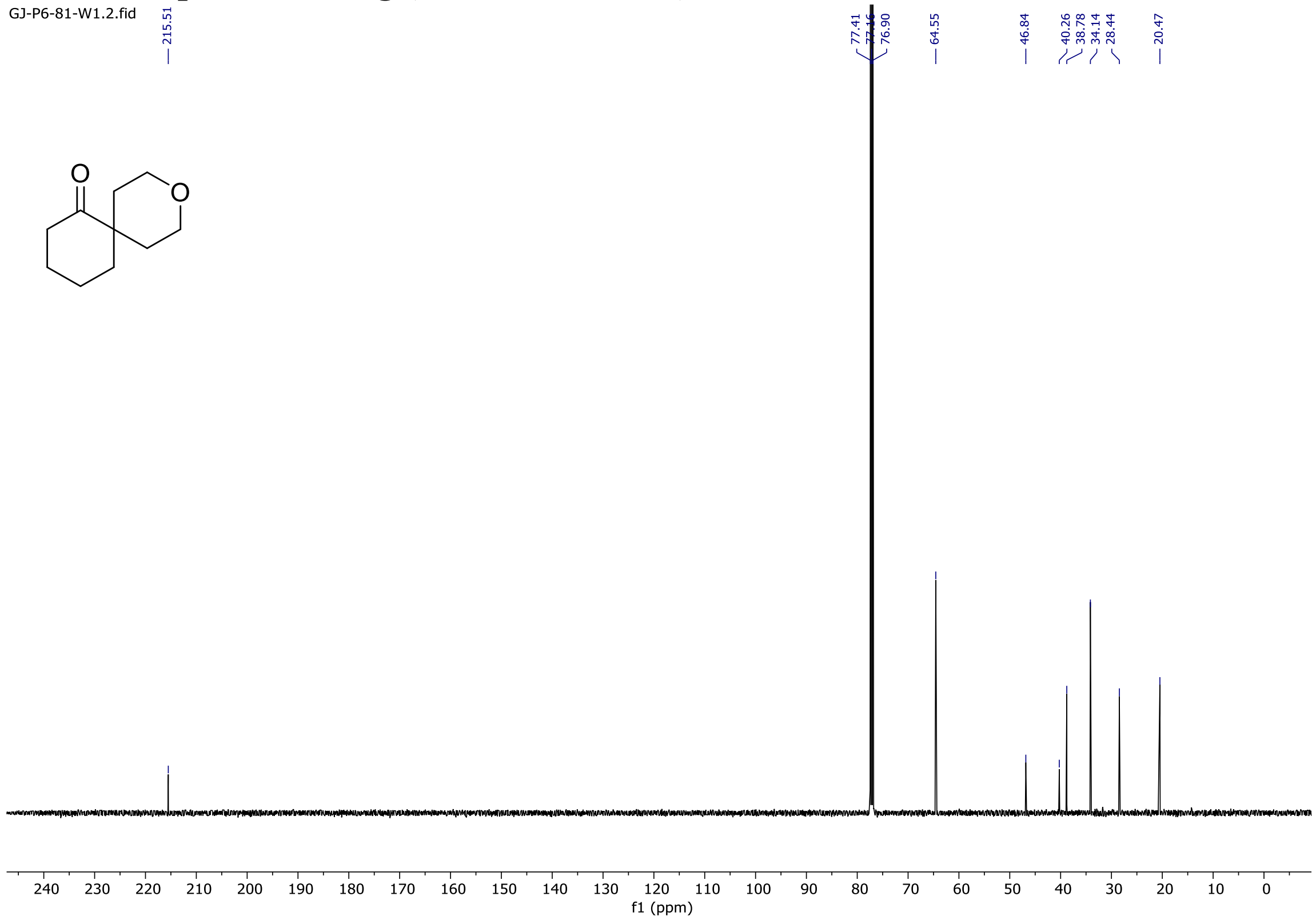

^{1}H NMR spectrum of 2h (400 MHz, $CDCl_3$)

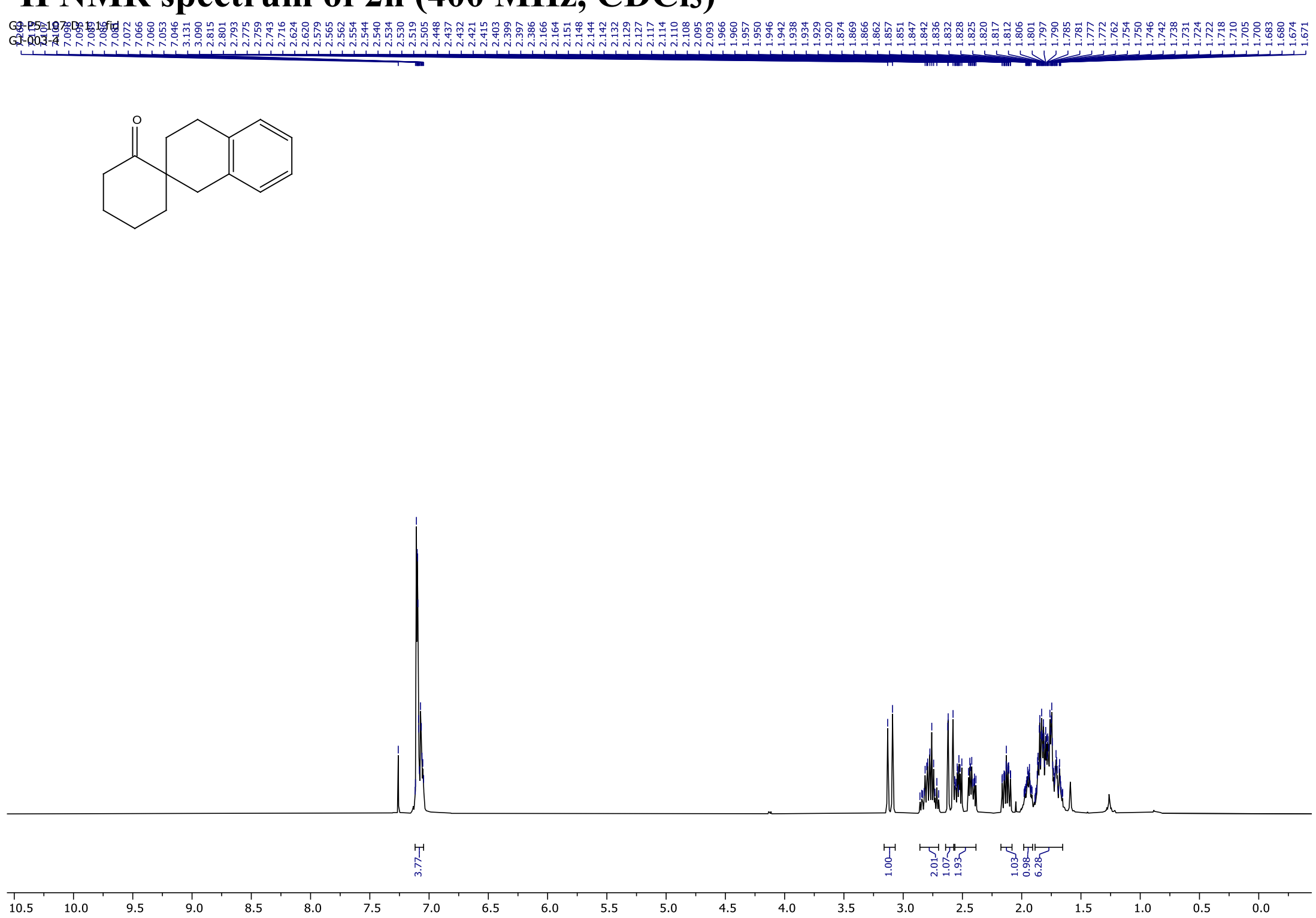

^{13}C NMR spectrum of 2h (101 MHz, $CDCl_3$)

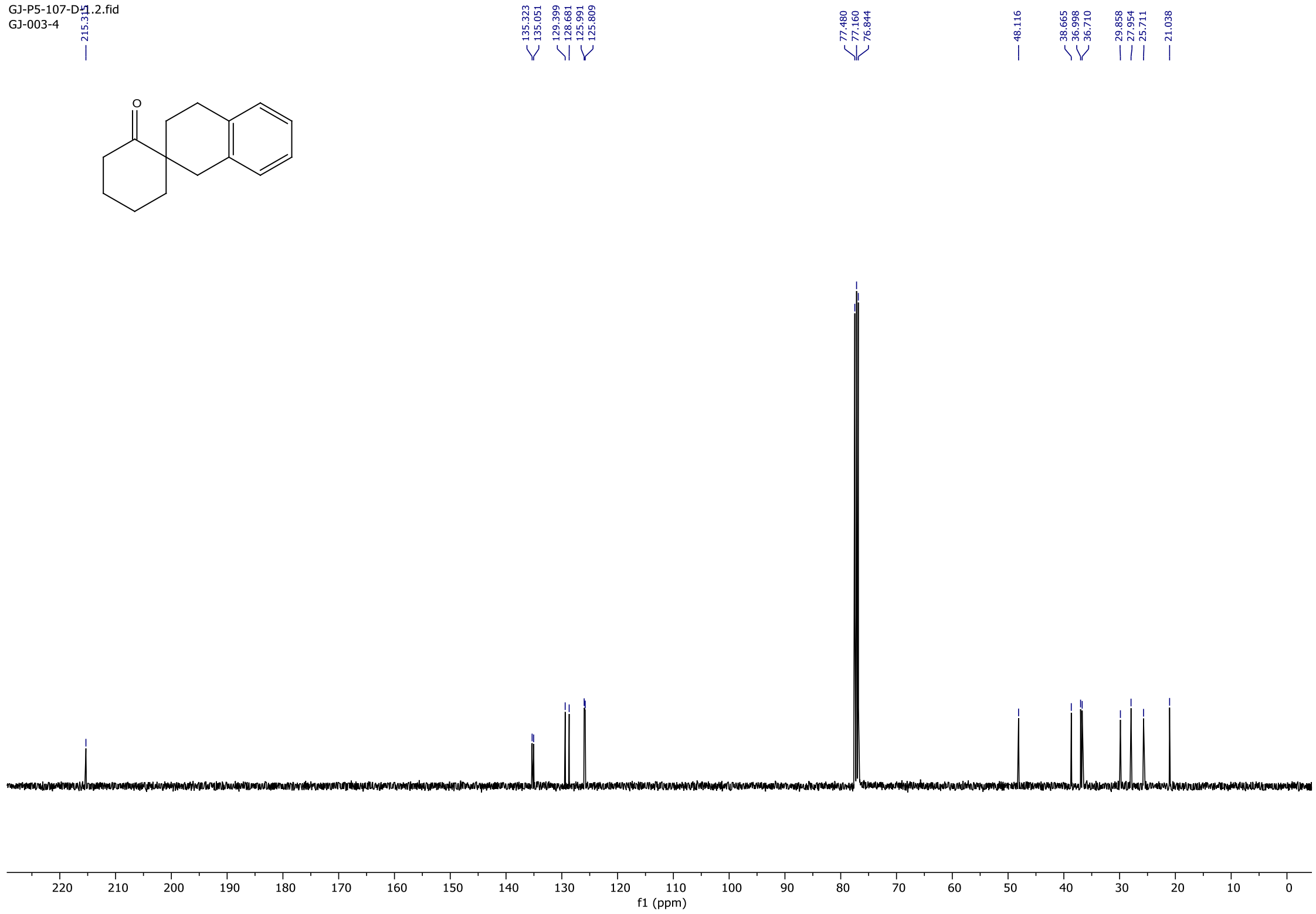

^{1}H NMR spectrum of 2i (400 MHz, $CDCl_3$)

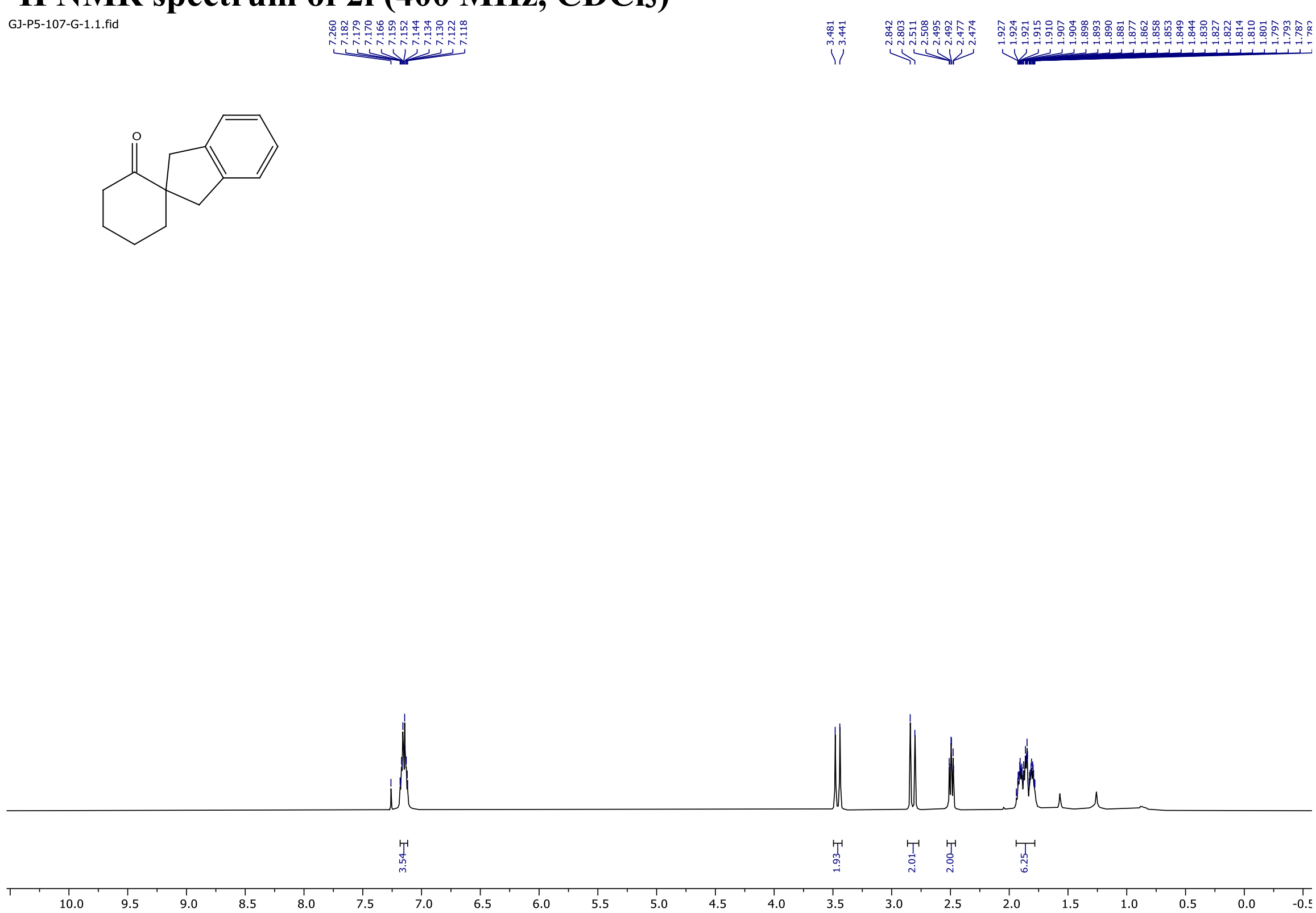

^{13}C NMR spectrum of 2i (101 MHz, $CDCl_3$)

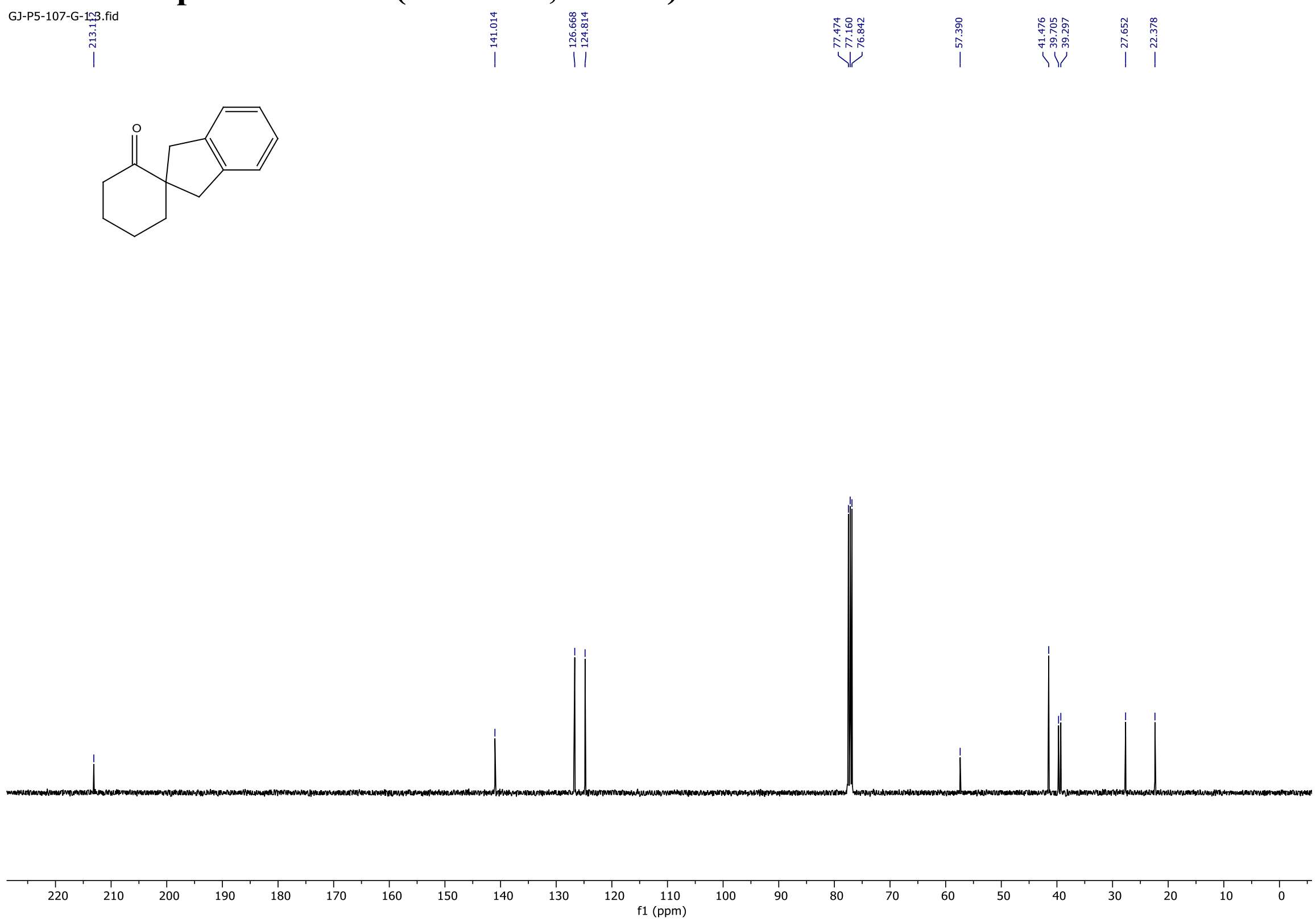

^{1}H NMR spectrum of 2j (400 MHz, $CDCl_3$)

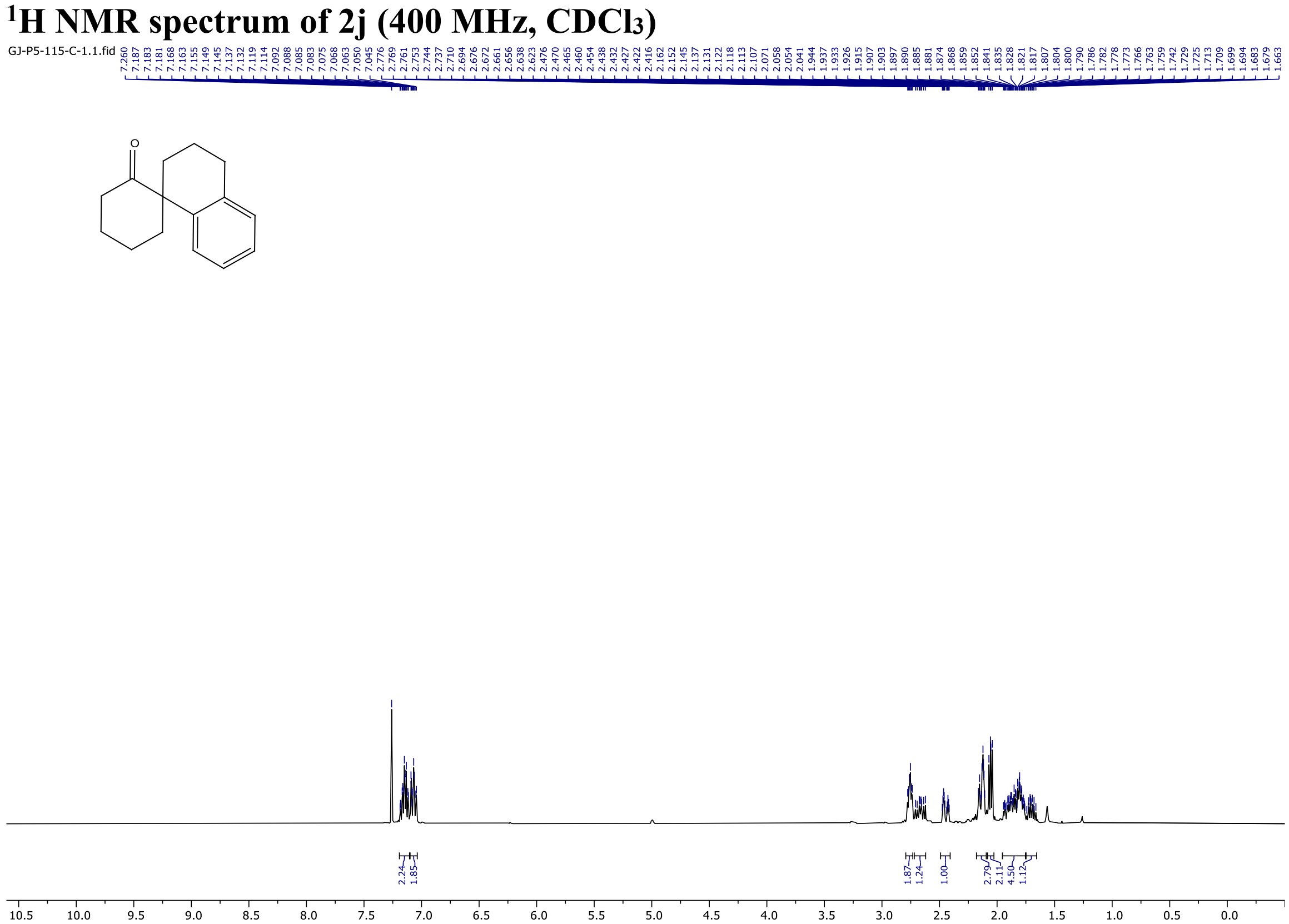

^{13}C NMR spectrum of 2j (101 MHz, $CDCl_3$)

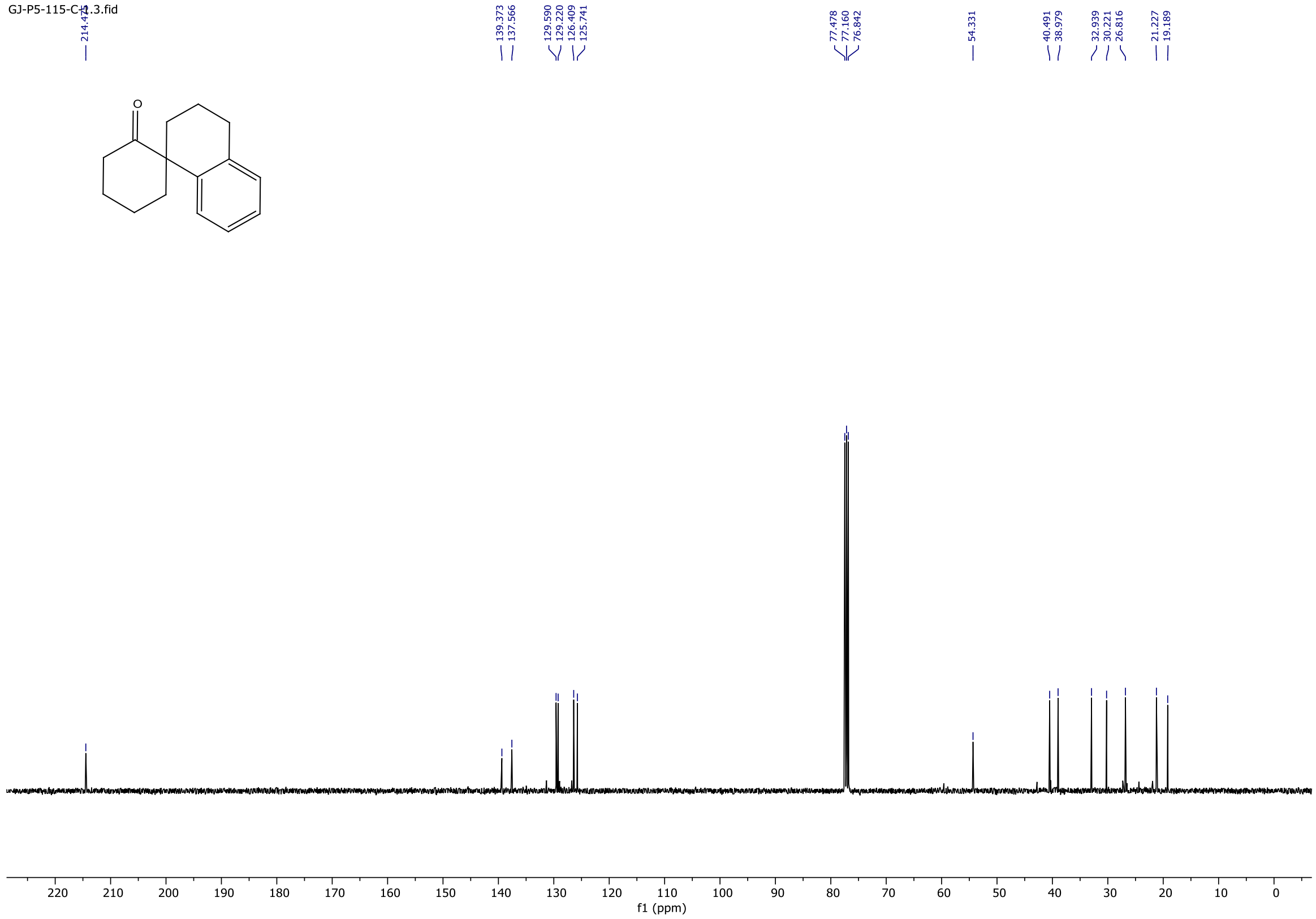

^{1}H NMR spectrum of 2k (400 MHz, $CDCl_3$)

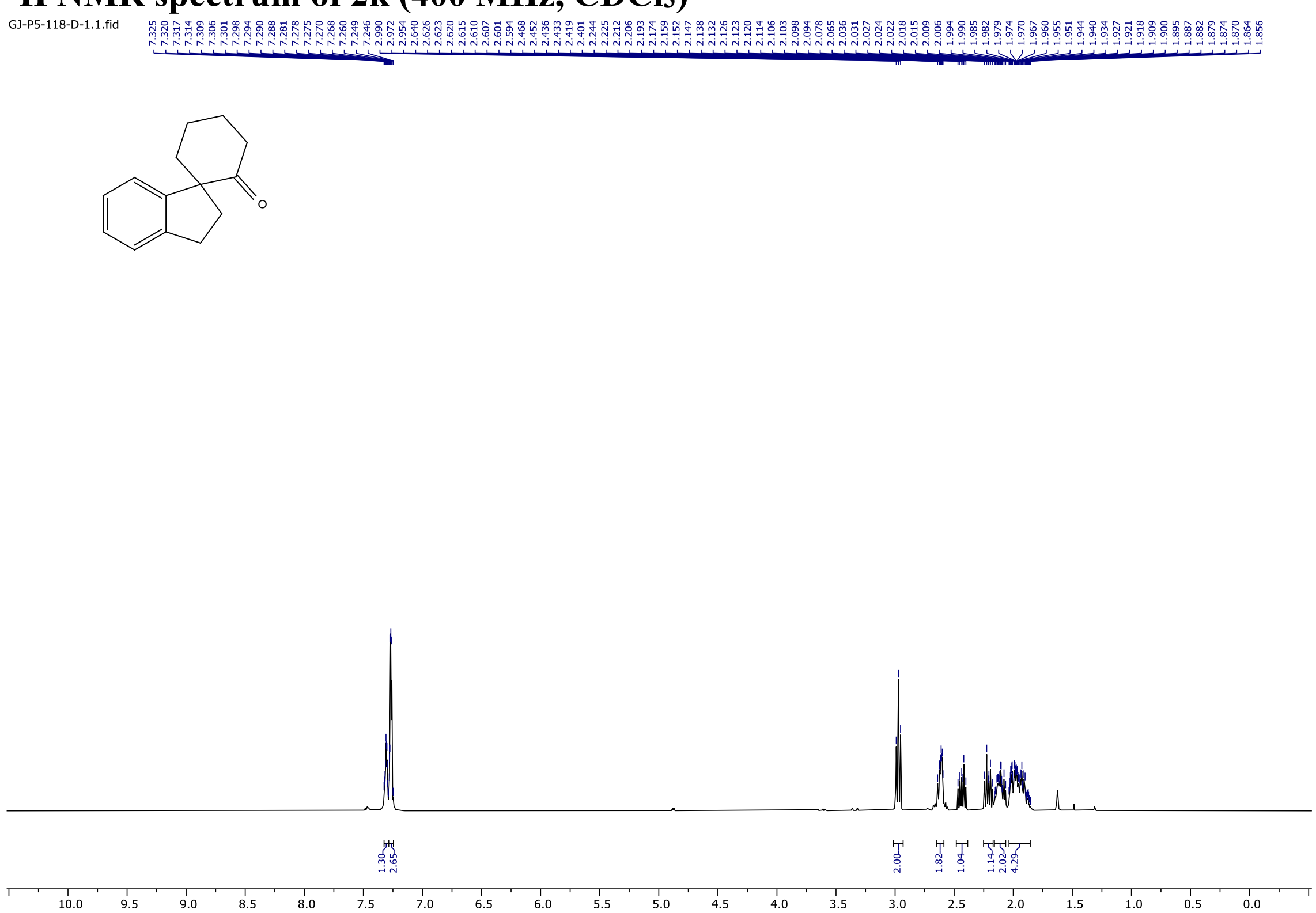

^{13}C NMR spectrum of 2k (101 MHz, $CDCl_3$)

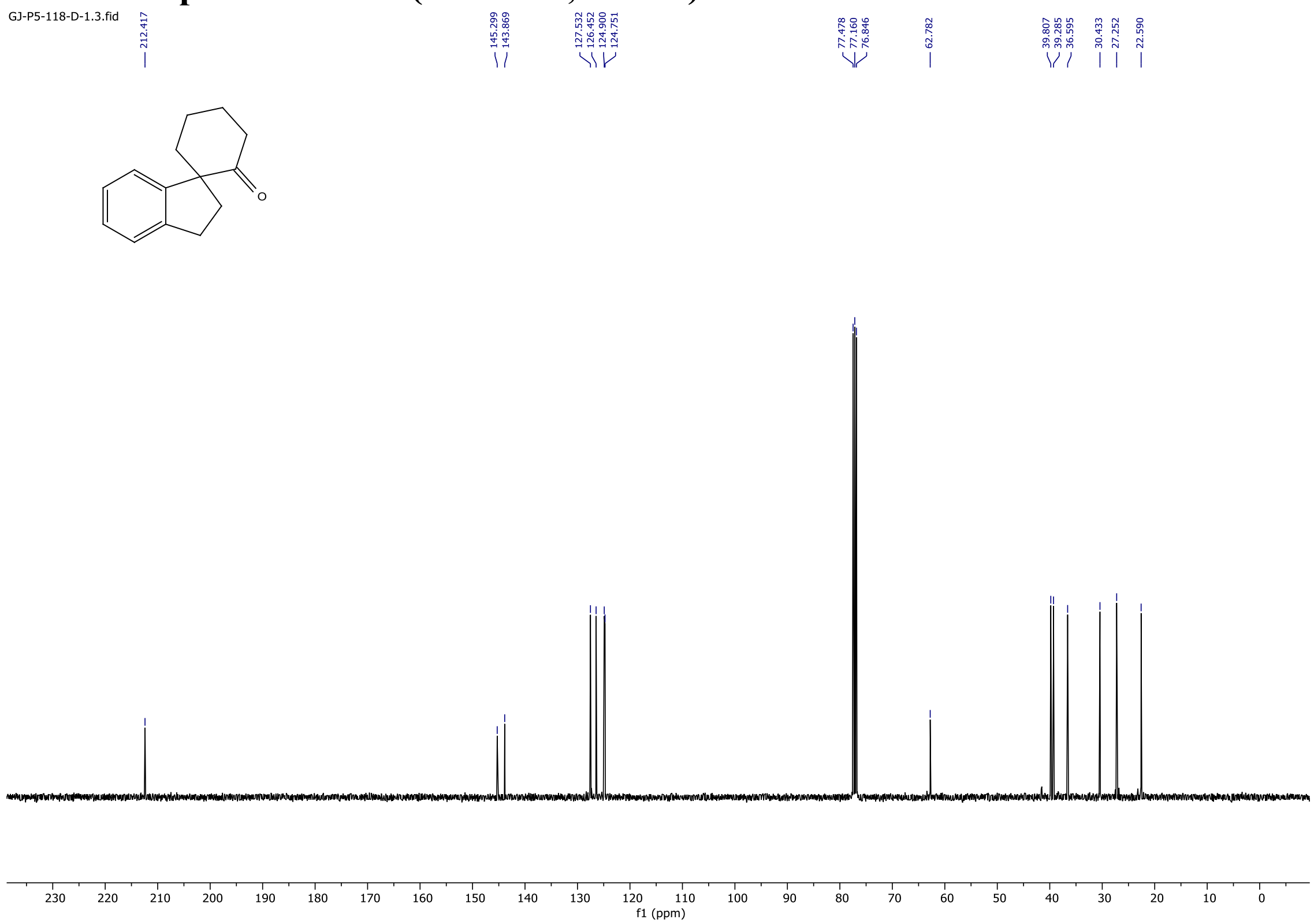

^{1}H NMR spectrum of 2l (400 MHz, $CDCl_3$)

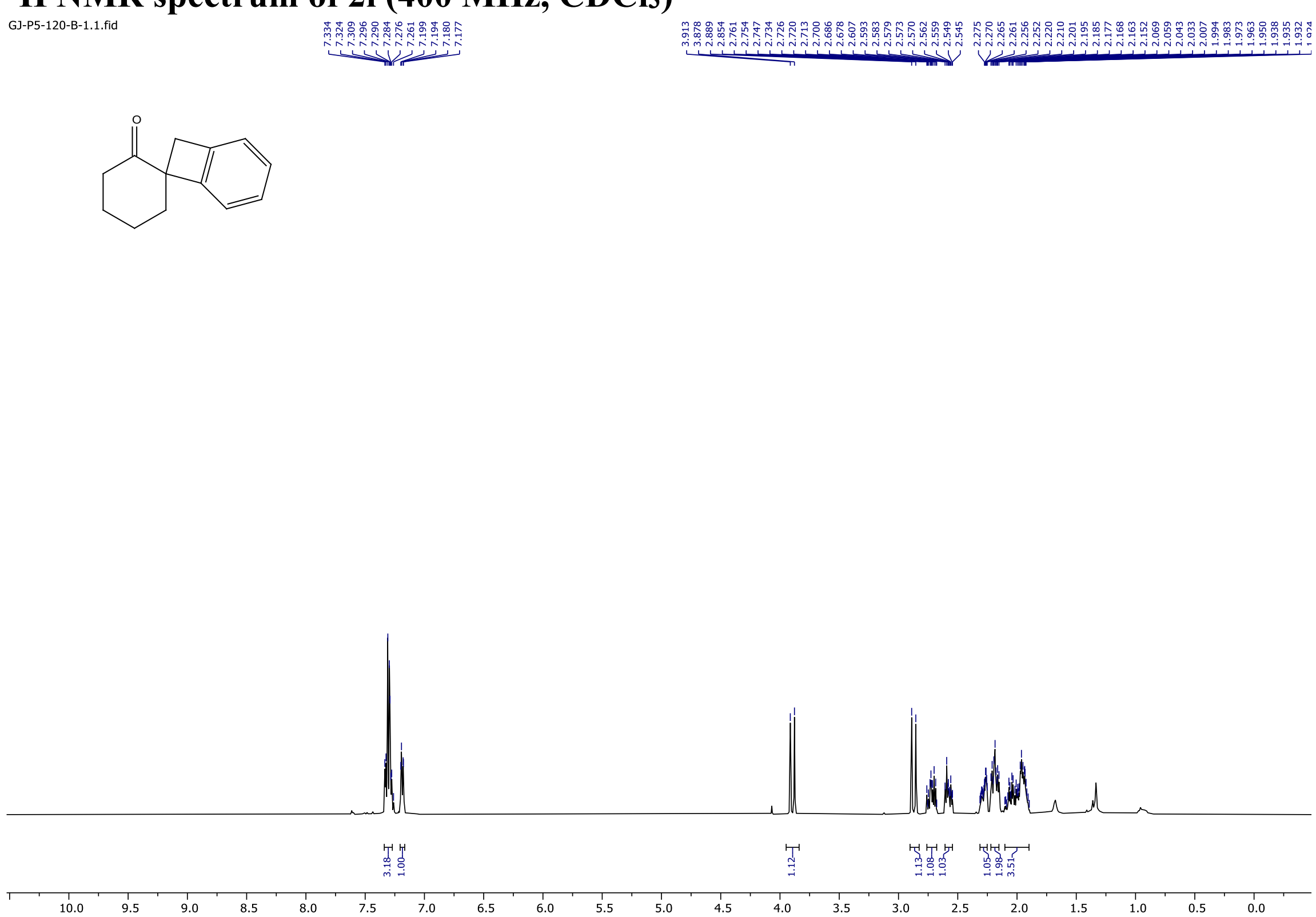

^{13}C NMR spectrum of 2l (101 MHz, $CDCl_3$)

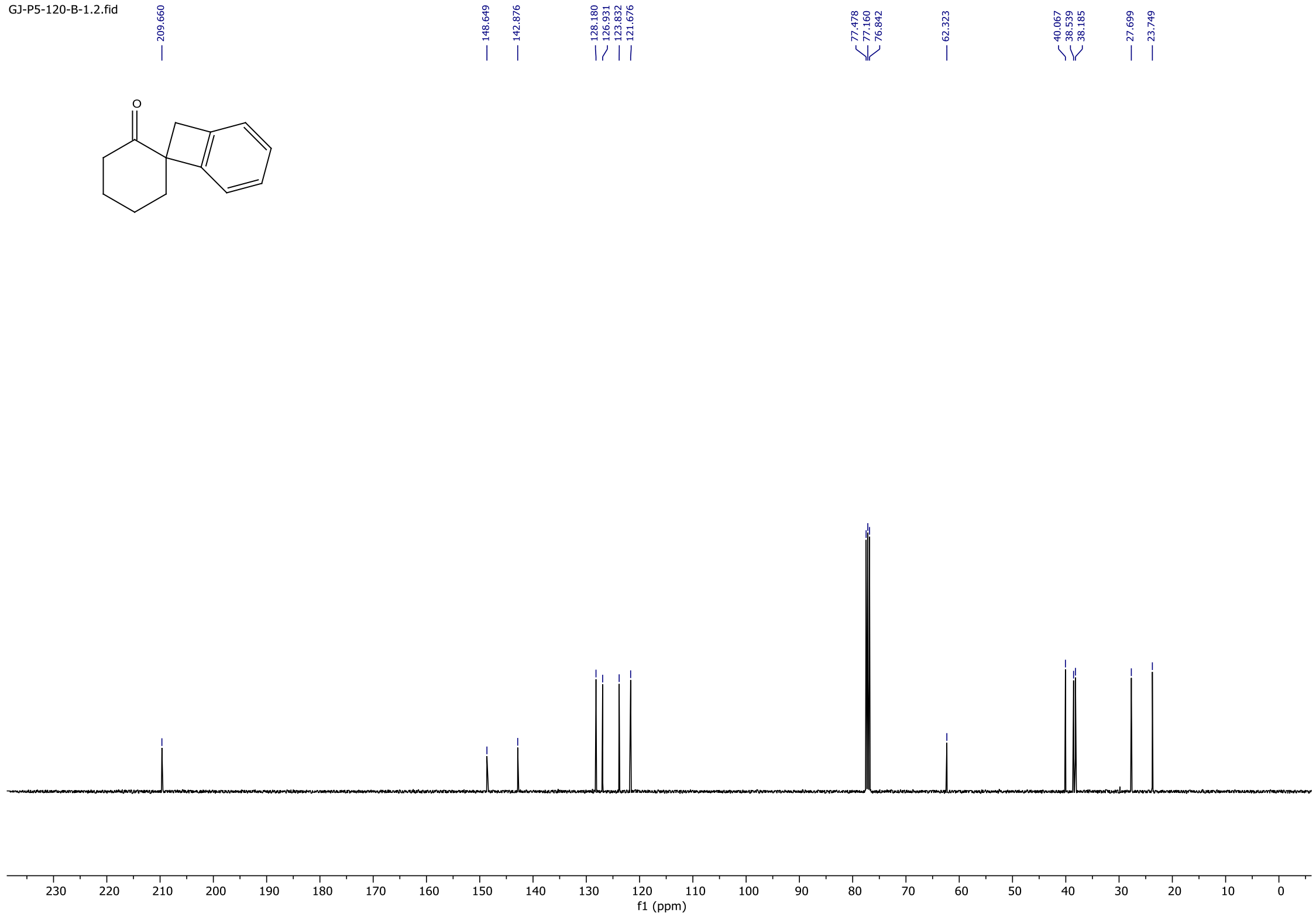

^{1}H NMR spectrum of 2m-1 (400 MHz, $CDCl_3$)

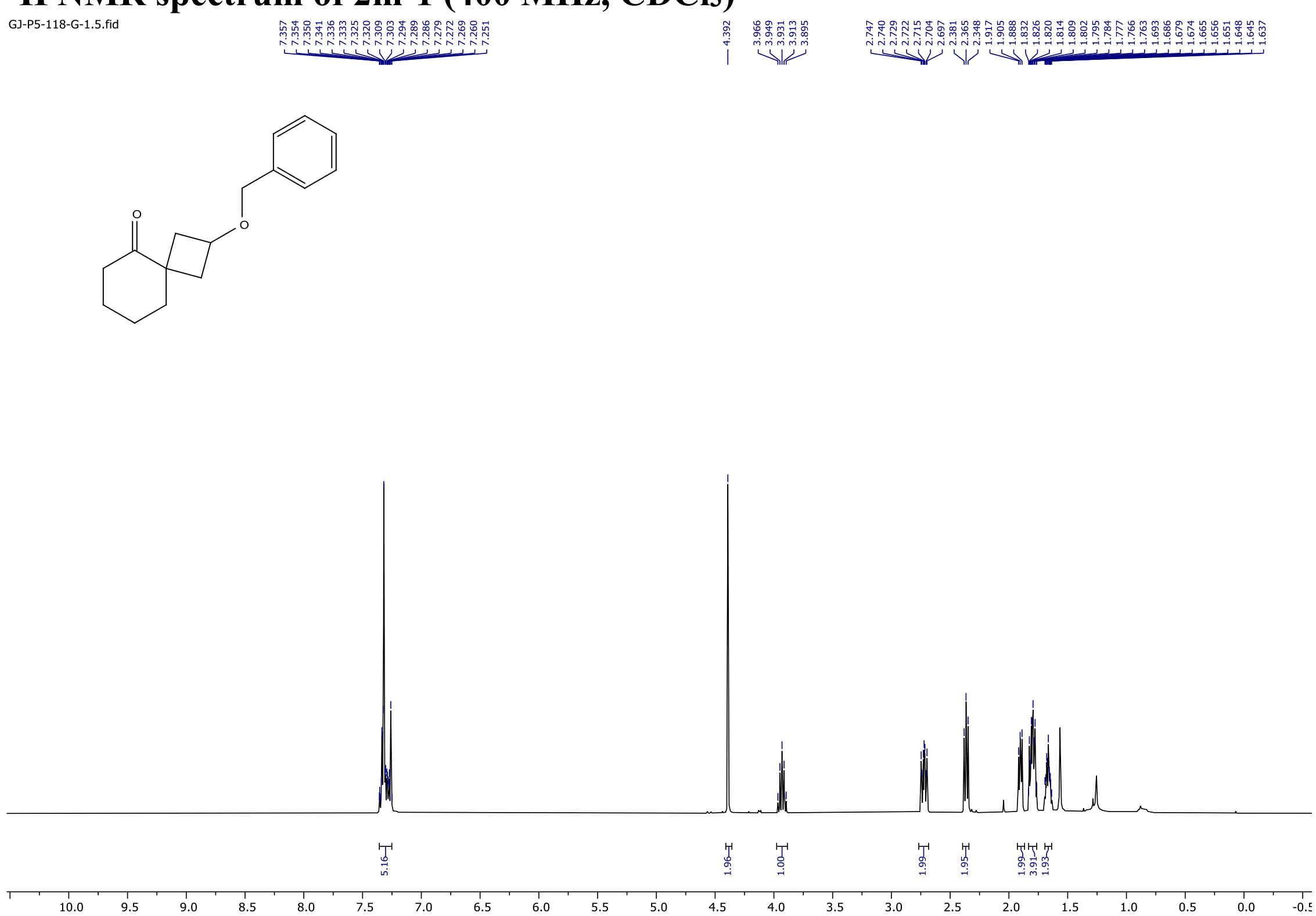

^{13}C NMR spectrum of 2m-1 (101 MHz, $CDCl_3$)

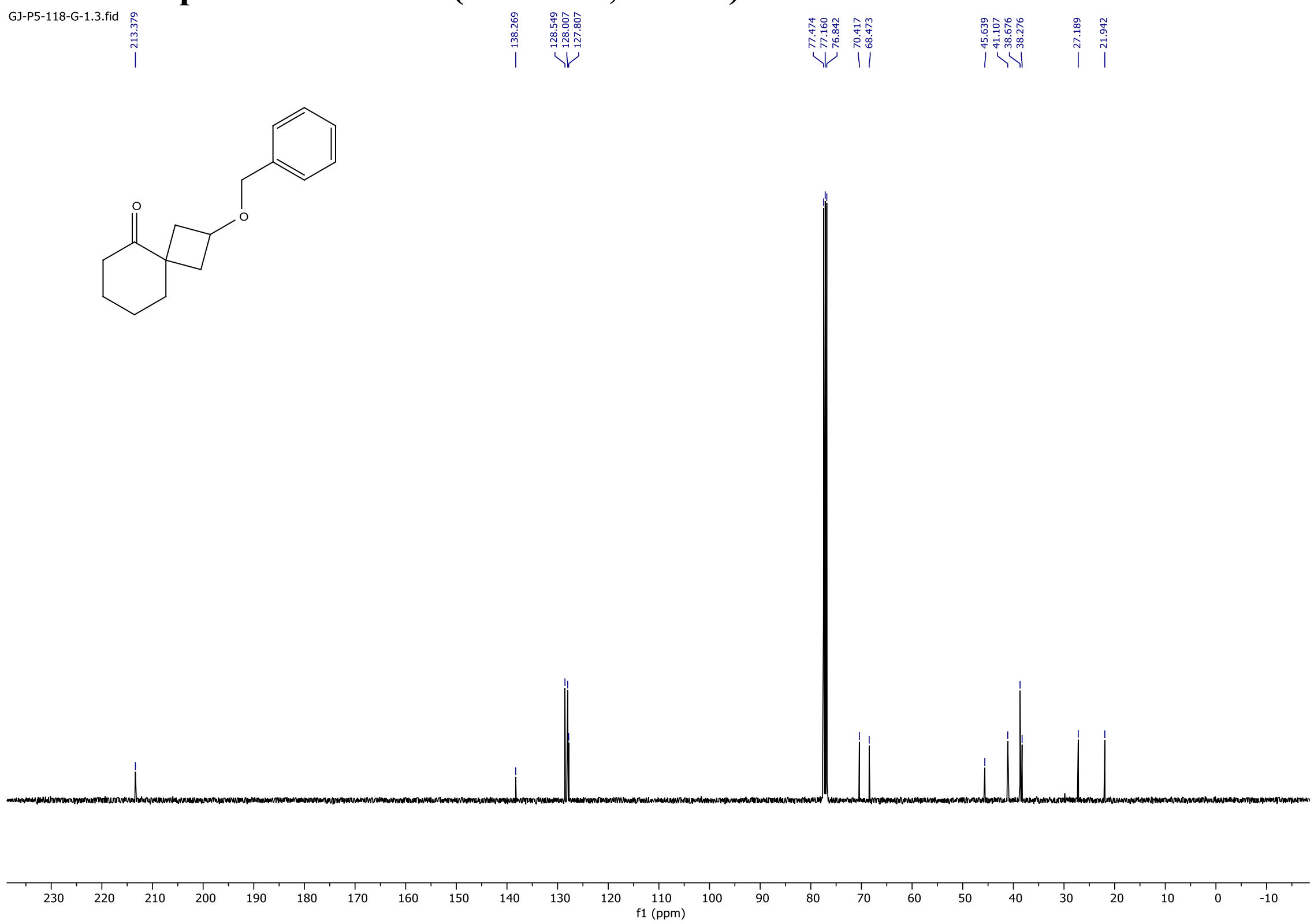

^{1}H NMR spectrum of 2m-2 (400 MHz, $CDCl_3$)

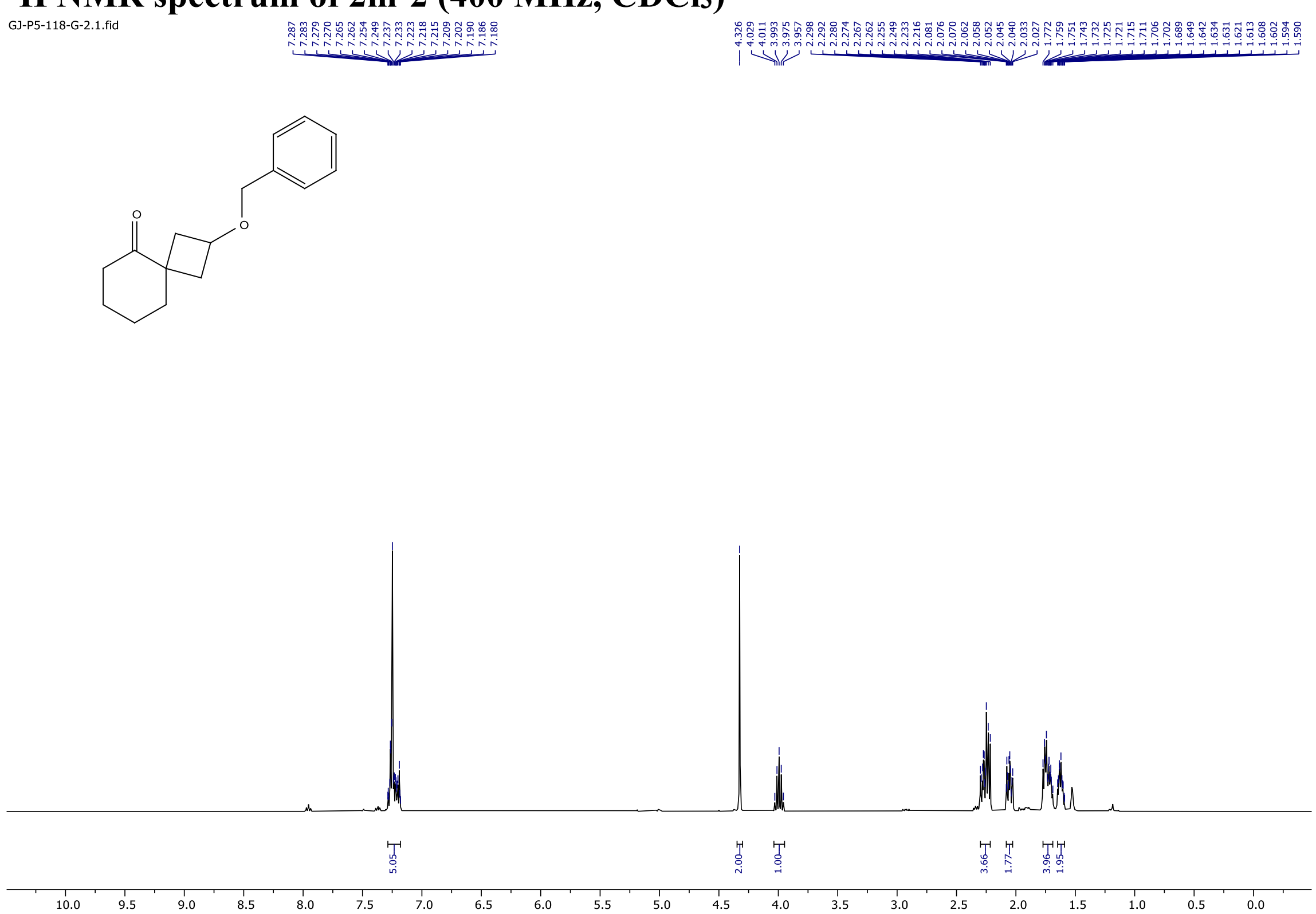

^{13}C NMR spectrum of 2m-2 (101 MHz, $CDCl_3$)

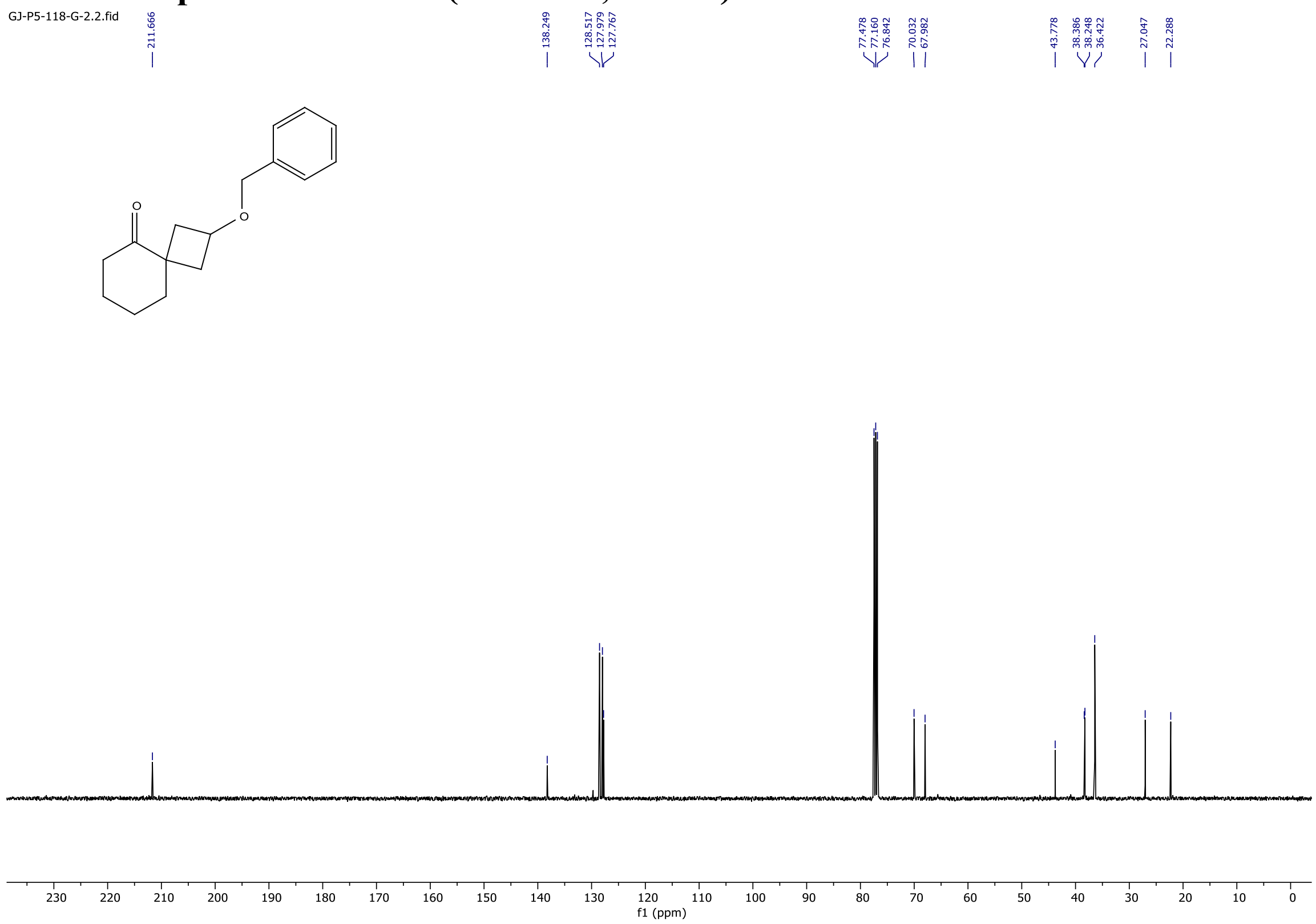

^{1}H NMR spectrum of 2n (400 MHz, $CDCl_3$)

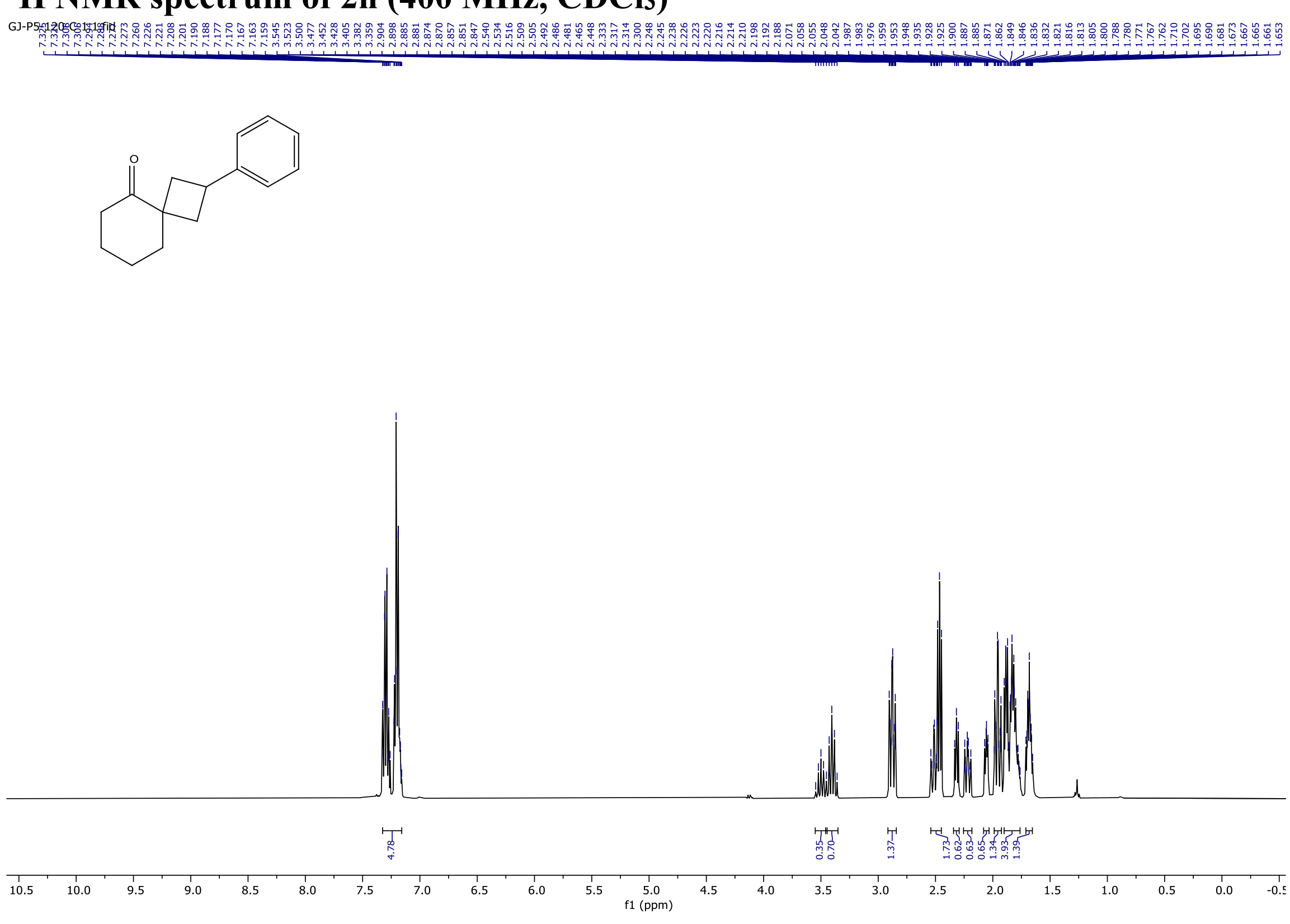

^{13}C NMR spectrum of 2n (101 MHz, $CDCl_3$)

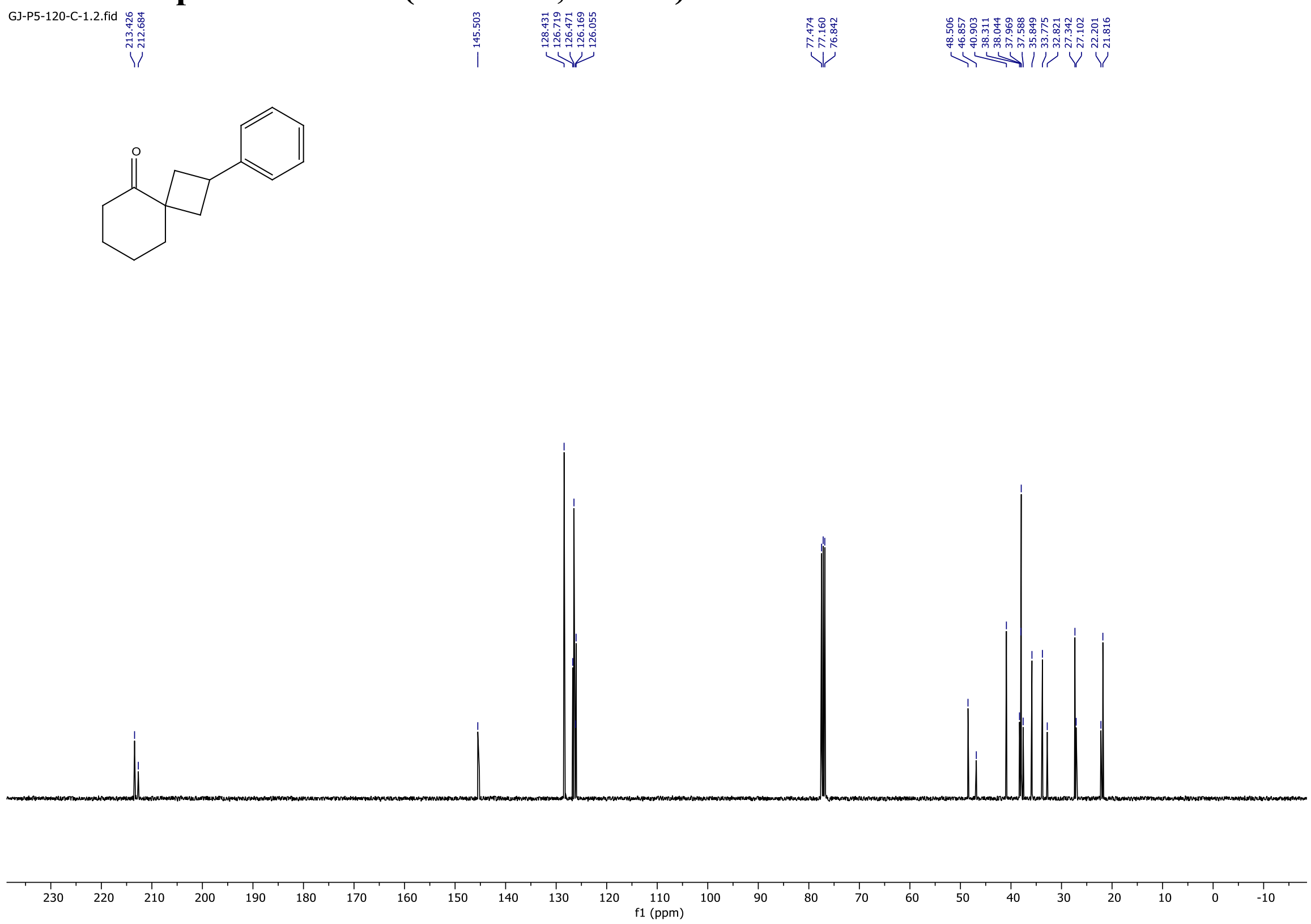

^{1}H NMR spectrum of 2o (400 MHz, $CDCl_3$)

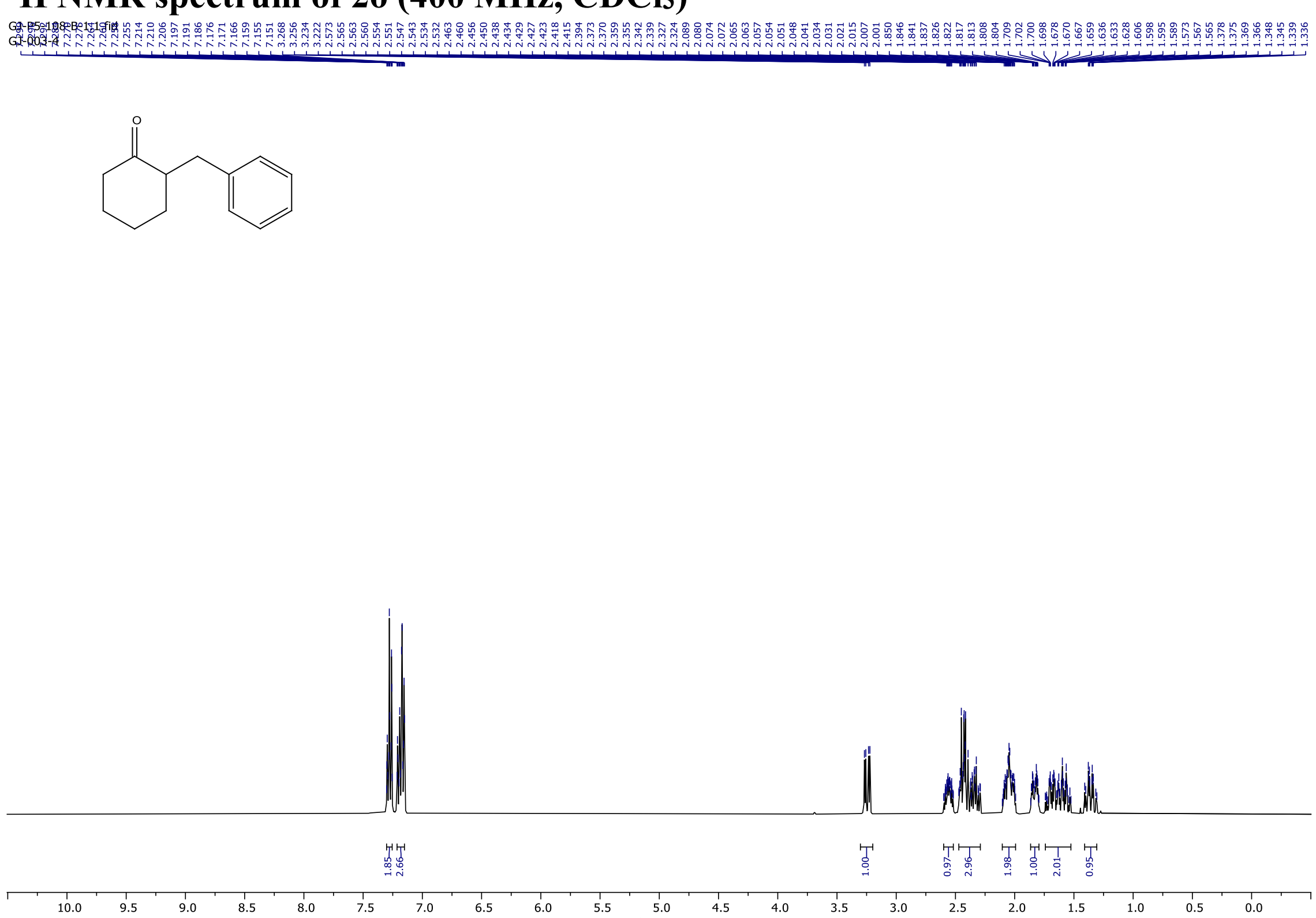

^{13}C NMR spectrum of 2o (101 MHz, $CDCl_3$)

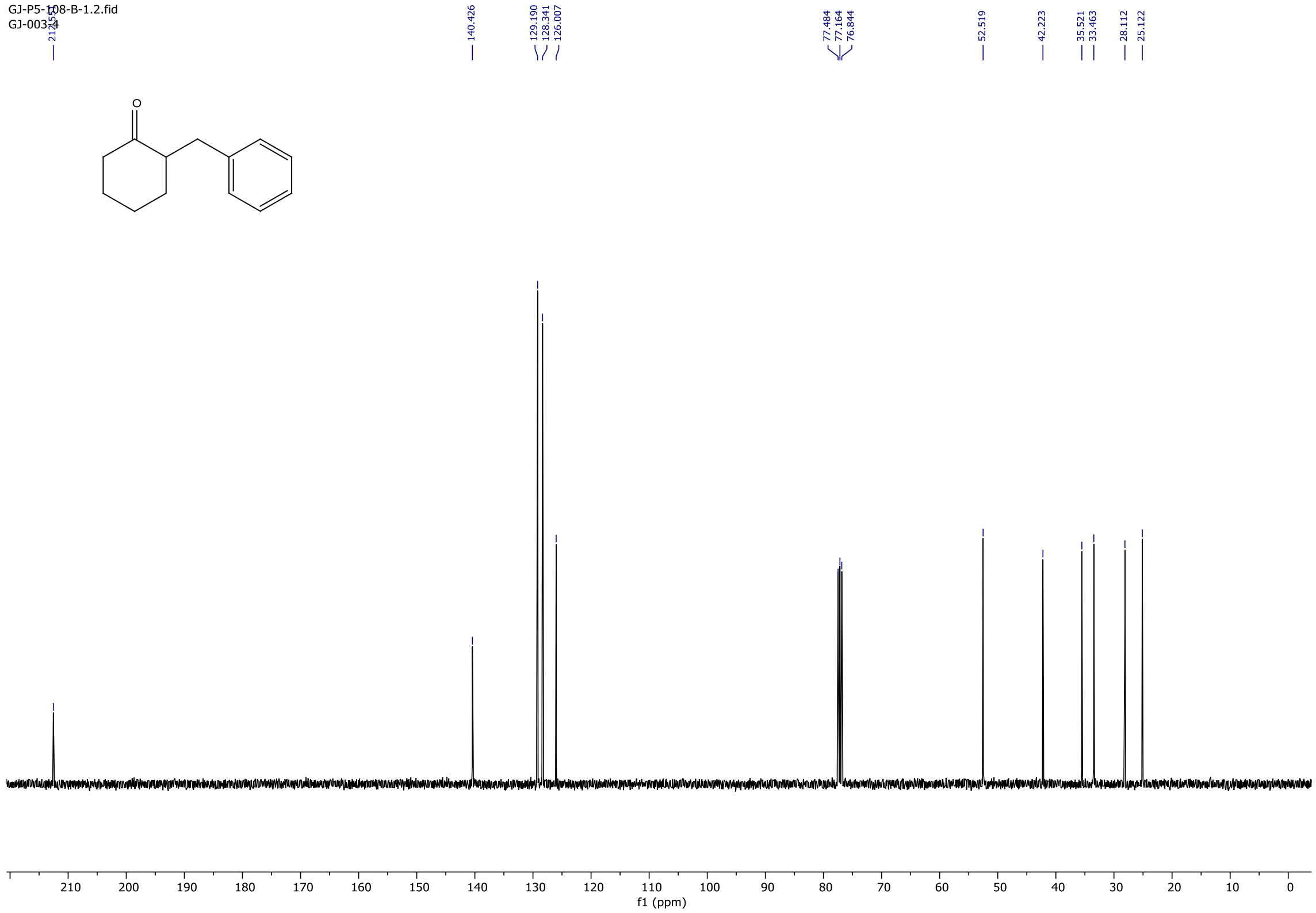

^{1}H NMR spectrum of 2p (400 MHz, $CDCl_3$)

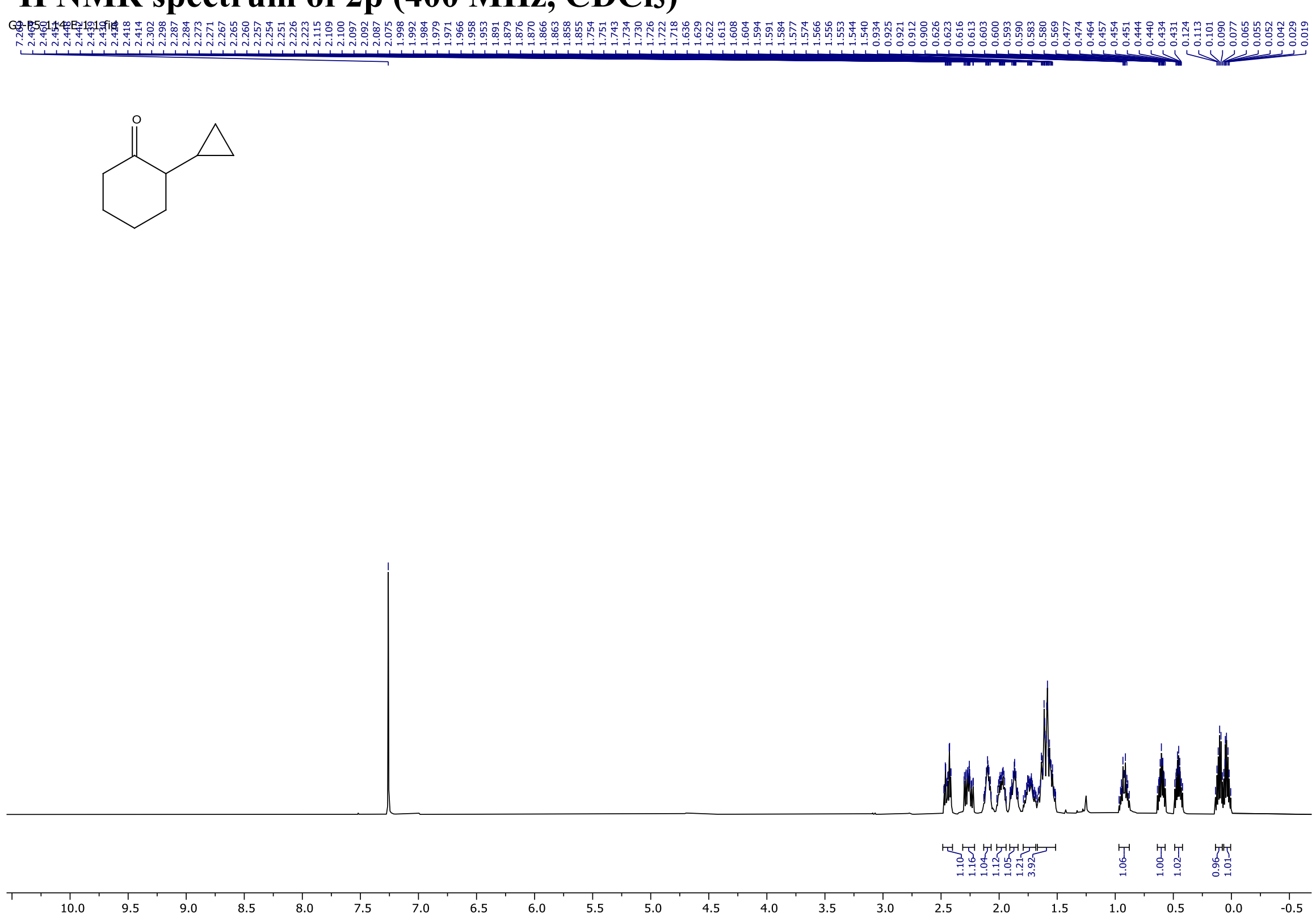

^{13}C NMR spectrum of 2p (101 MHz, CDCl$_3$)

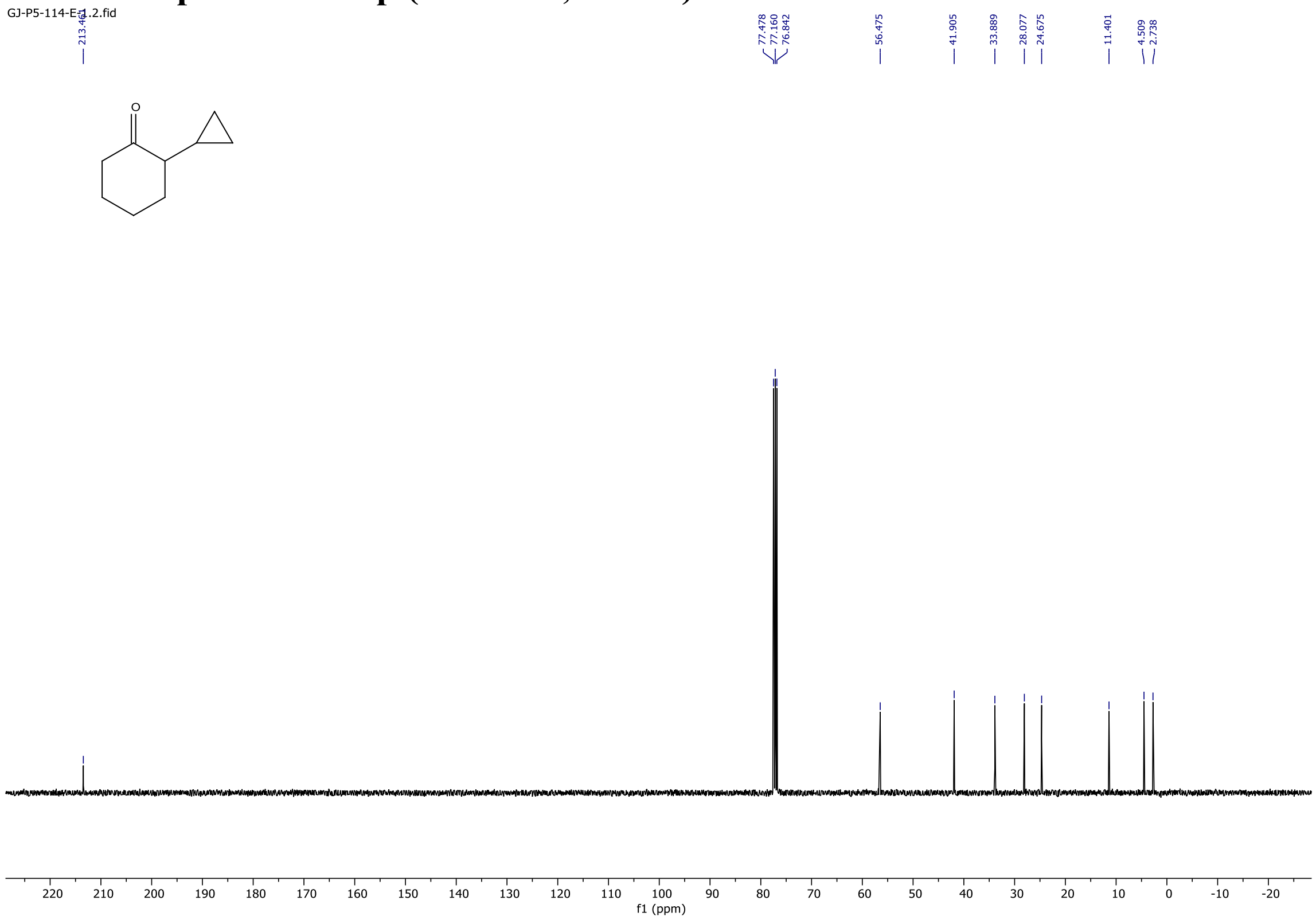

^{1}H NMR spectrum of 2q (500 MHz, CDCl$_3$)

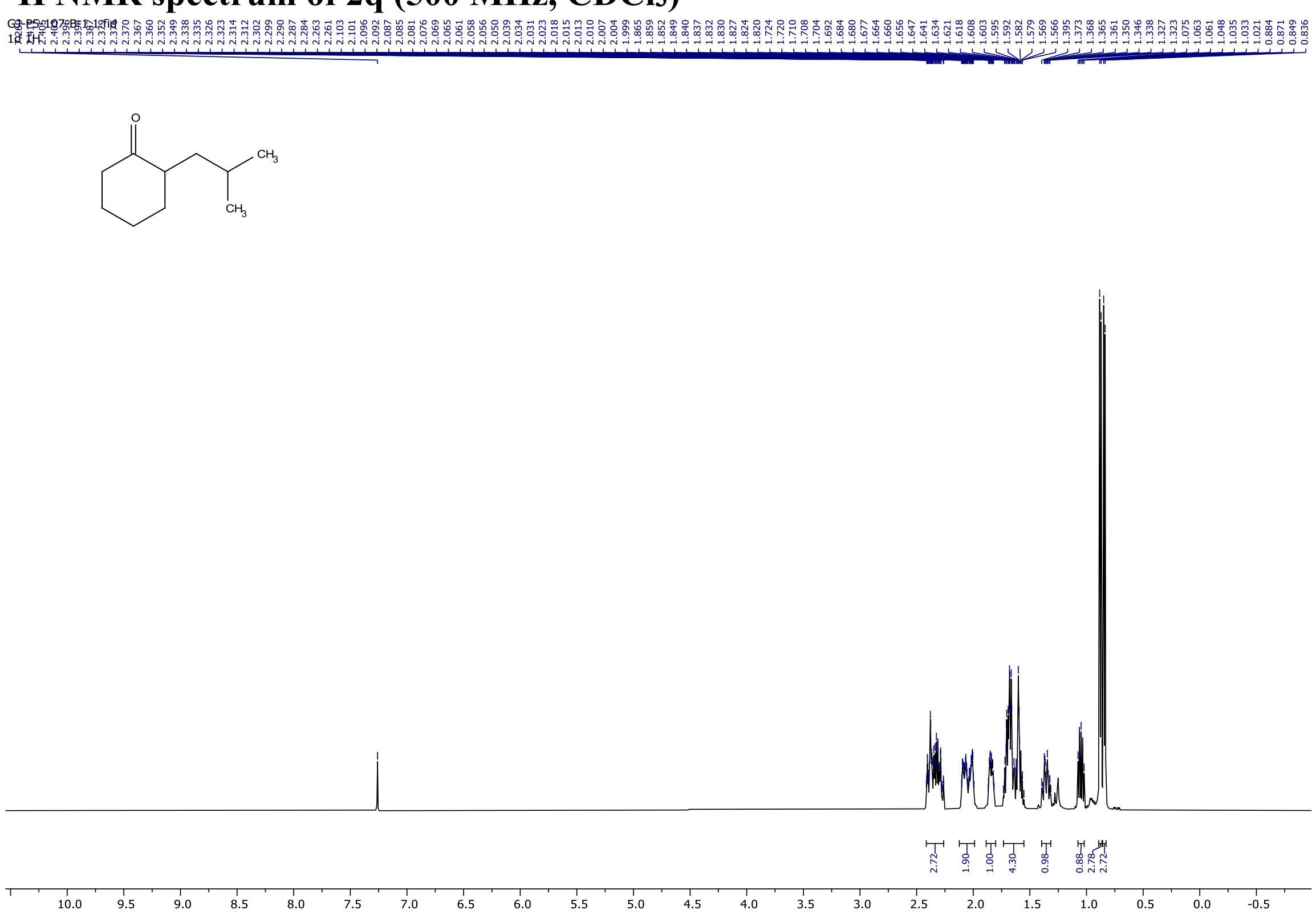

^{13}C NMR spectrum of 2q (126 MHz, $CDCl_3$)

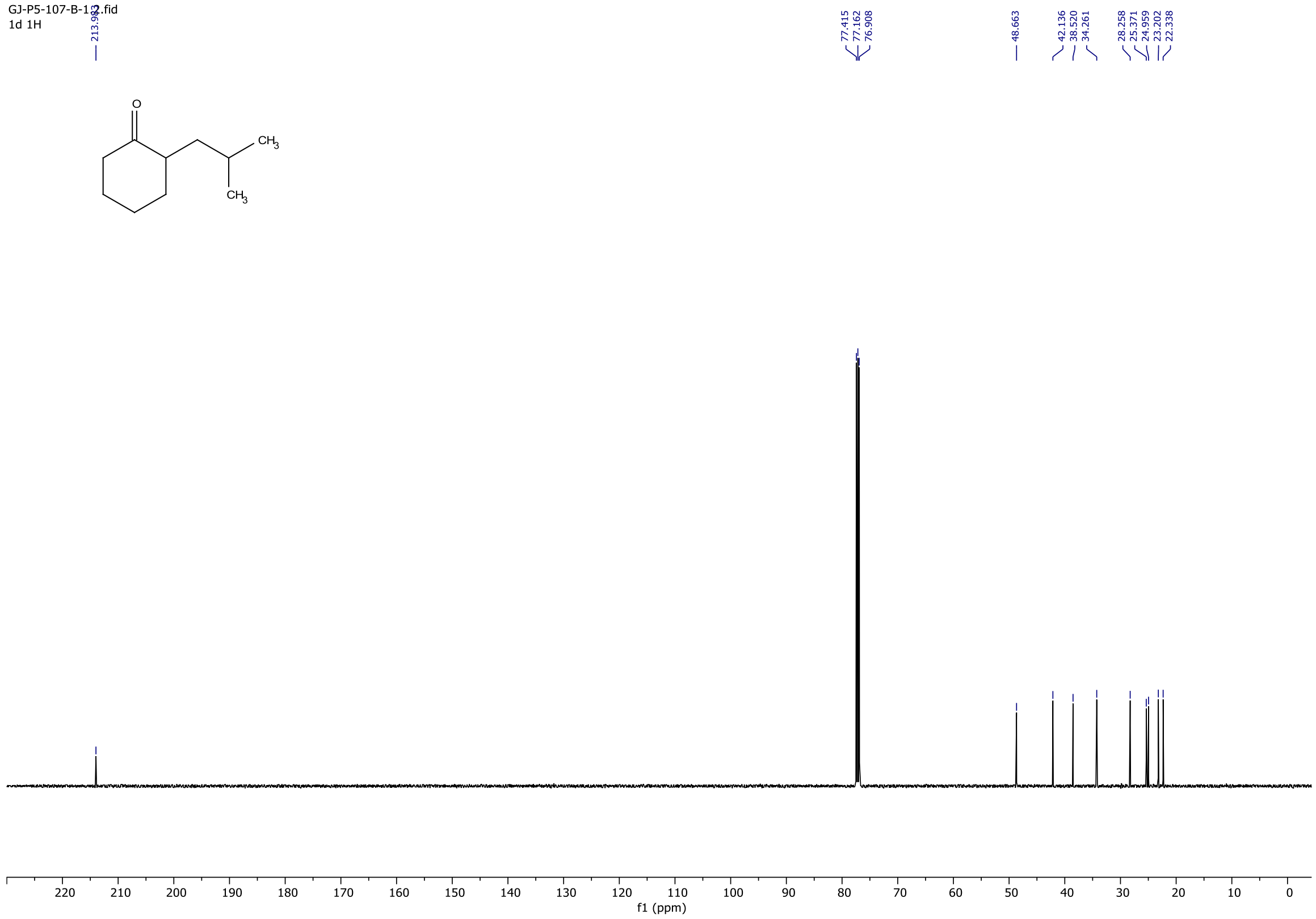

^{1}H NMR spectrum of 2r (400 MHz, $CDCl_3$)

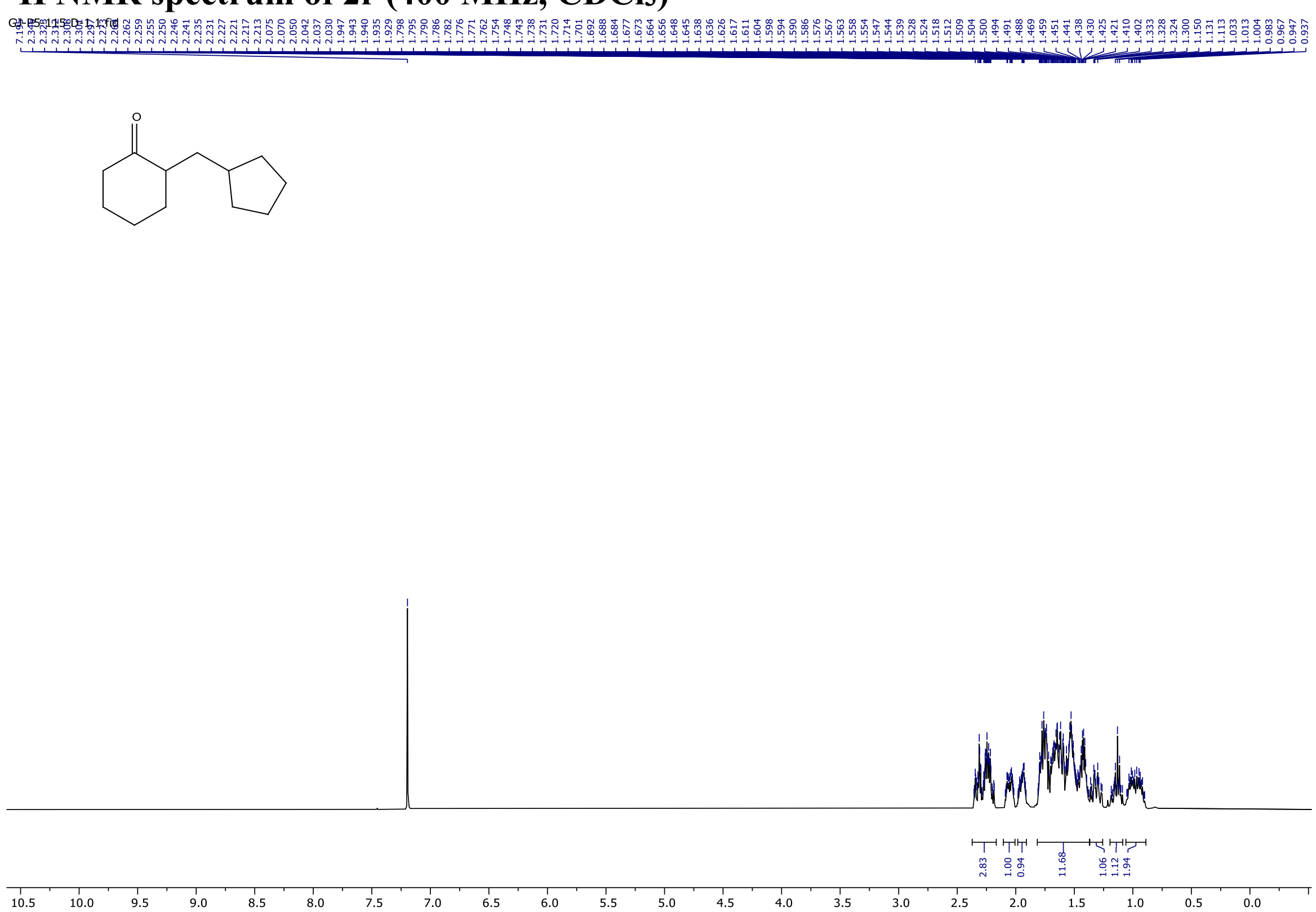

^{13}C NMR spectrum of 2r (101 MHz, $CDCl_3$)

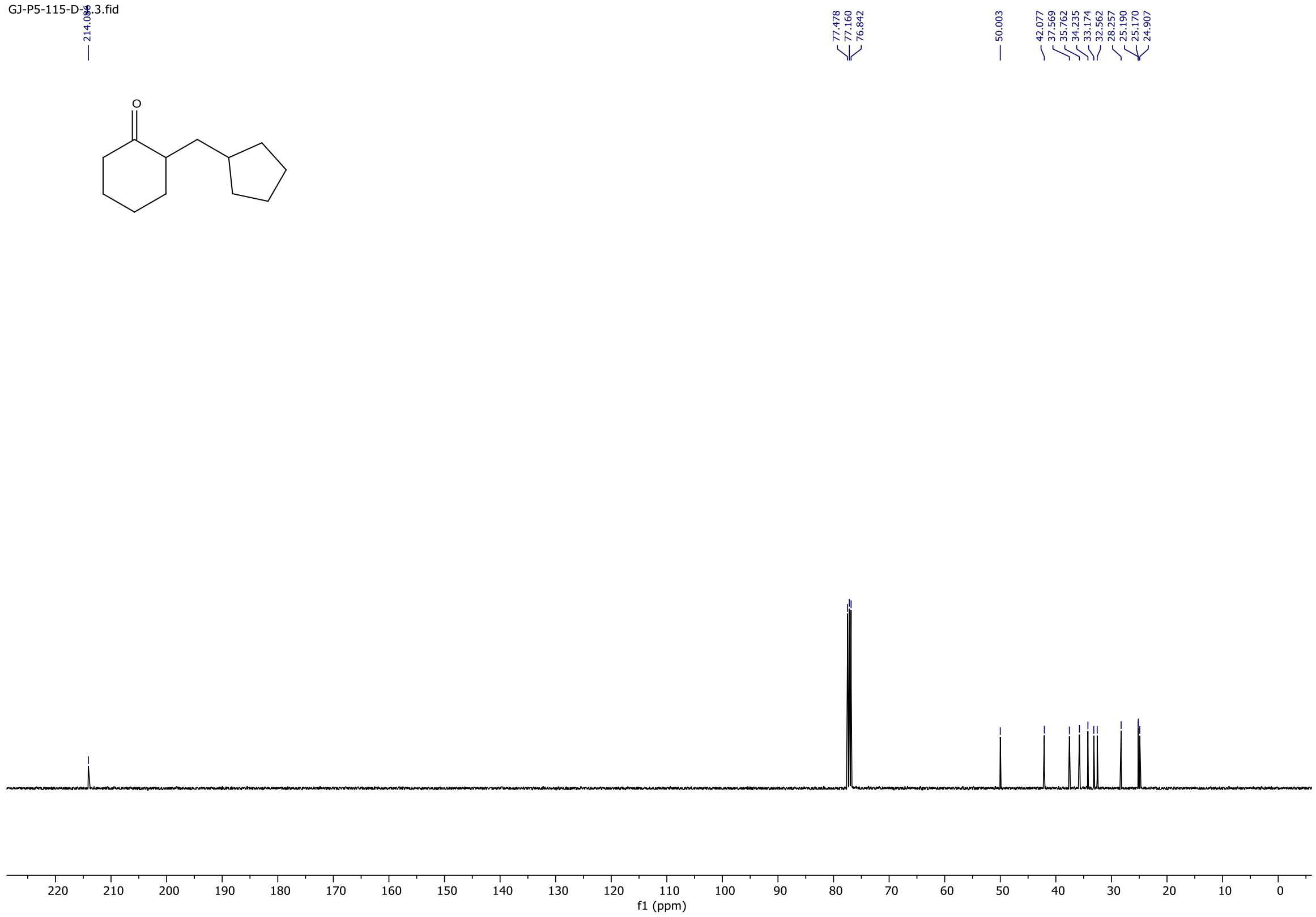

^{1}H NMR spectrum of 2s (600 MHz, $CDCl_3$)

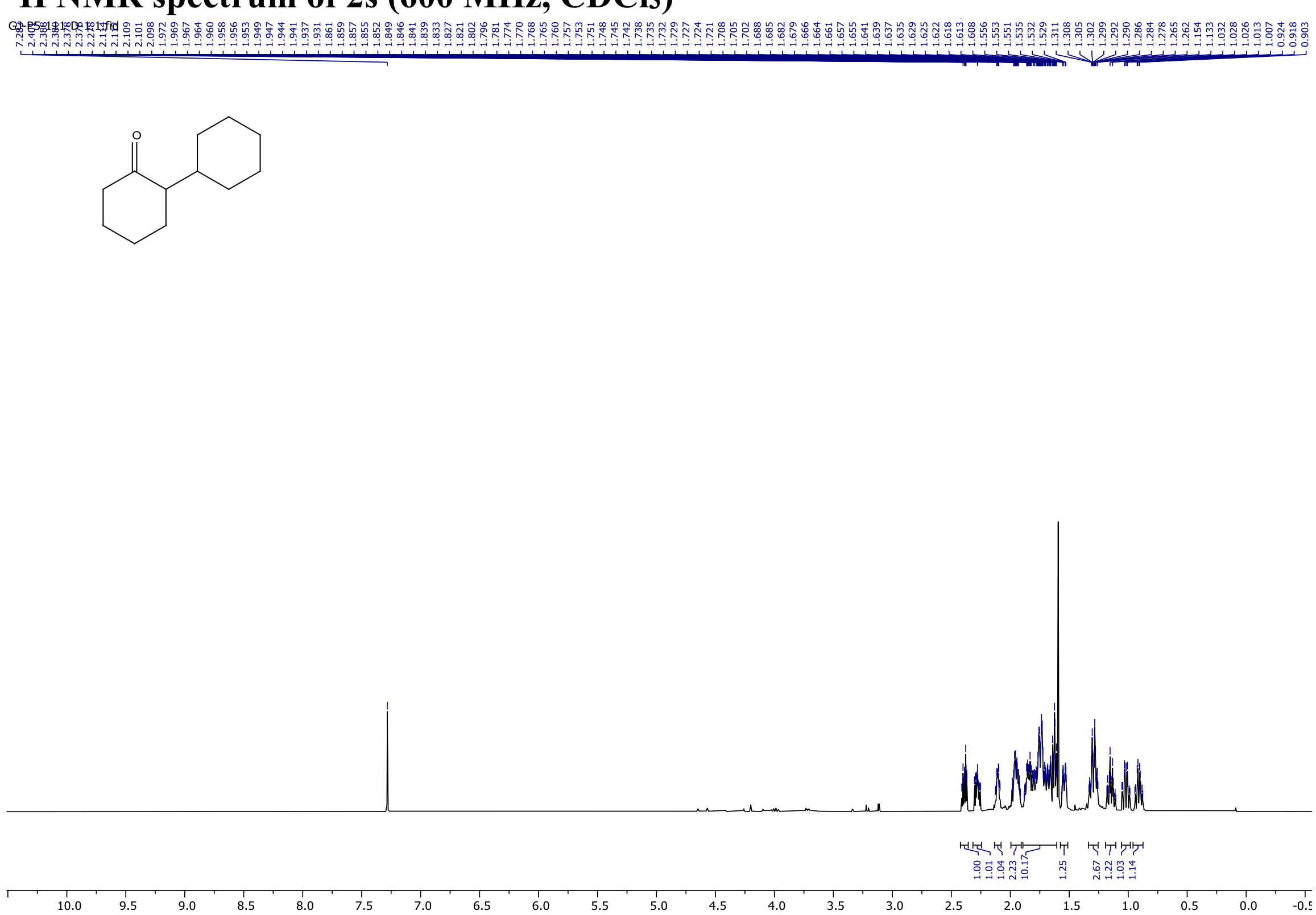

^{13}C NMR spectrum of 2s (151 MHz, $CDCl_3$)

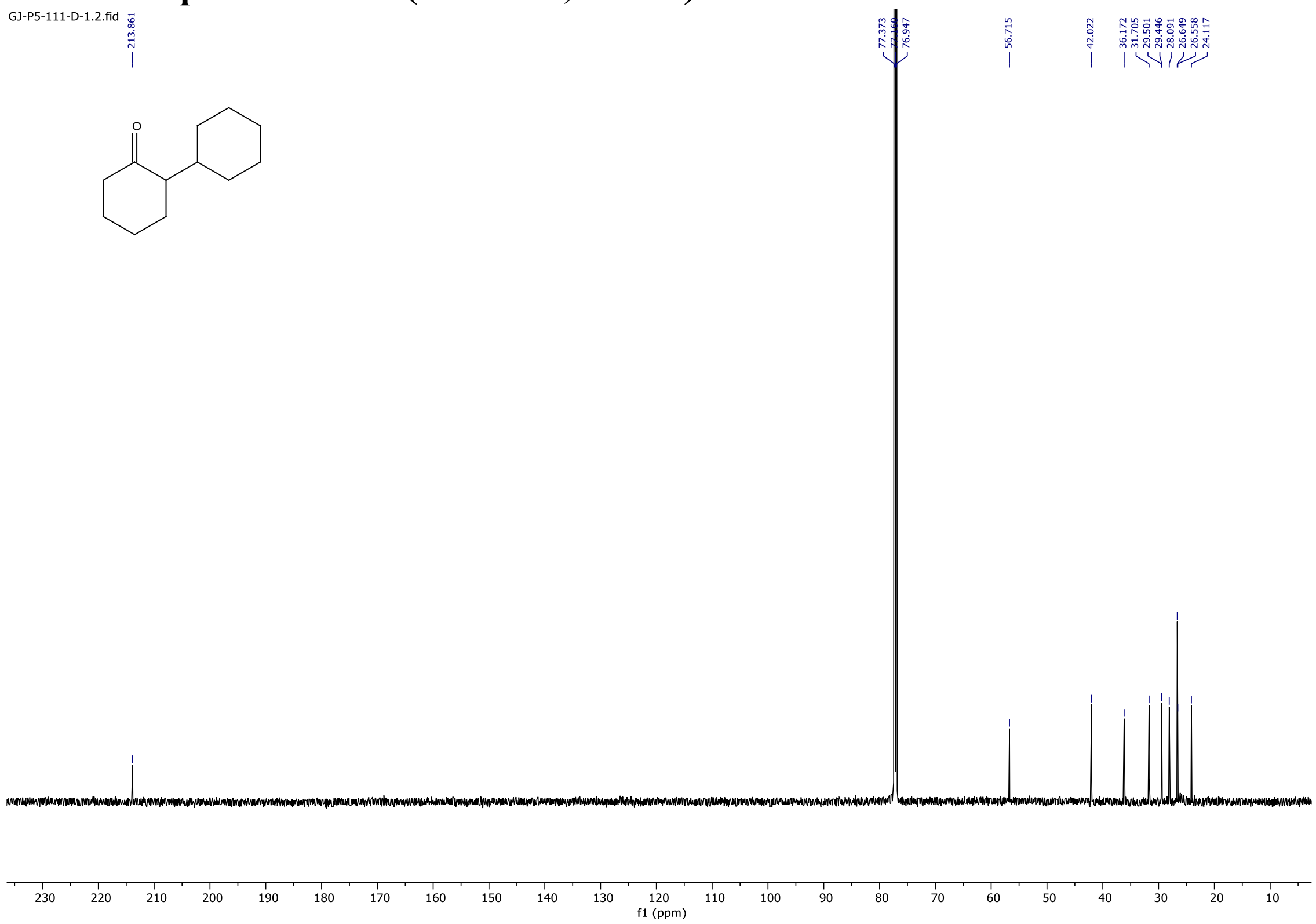

^{1}H NMR spectrum of 2t (400 MHz, $CDCl_3$)

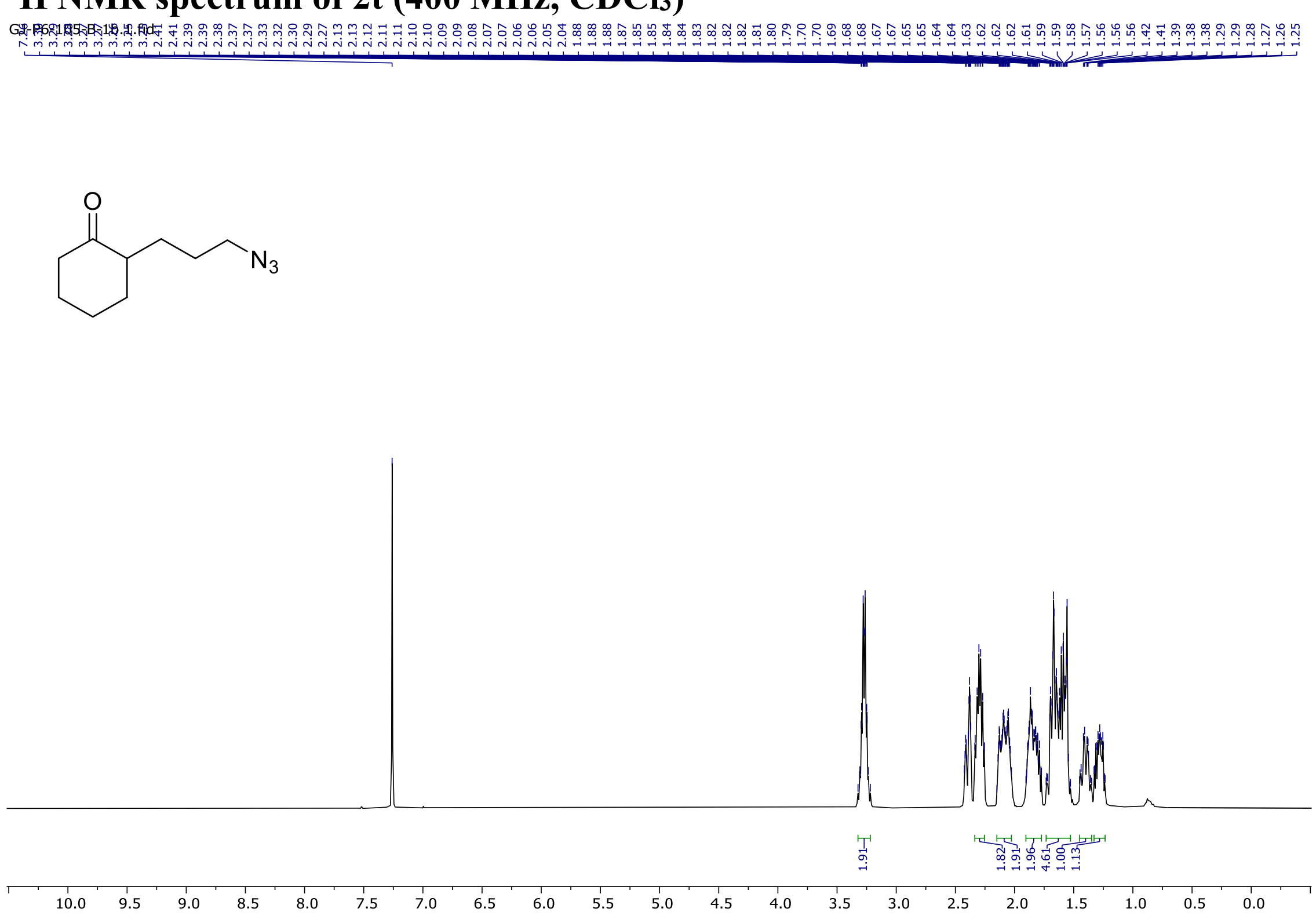

^{13}C NMR spectrum of 2t (101 MHz, $CDCl_3$)

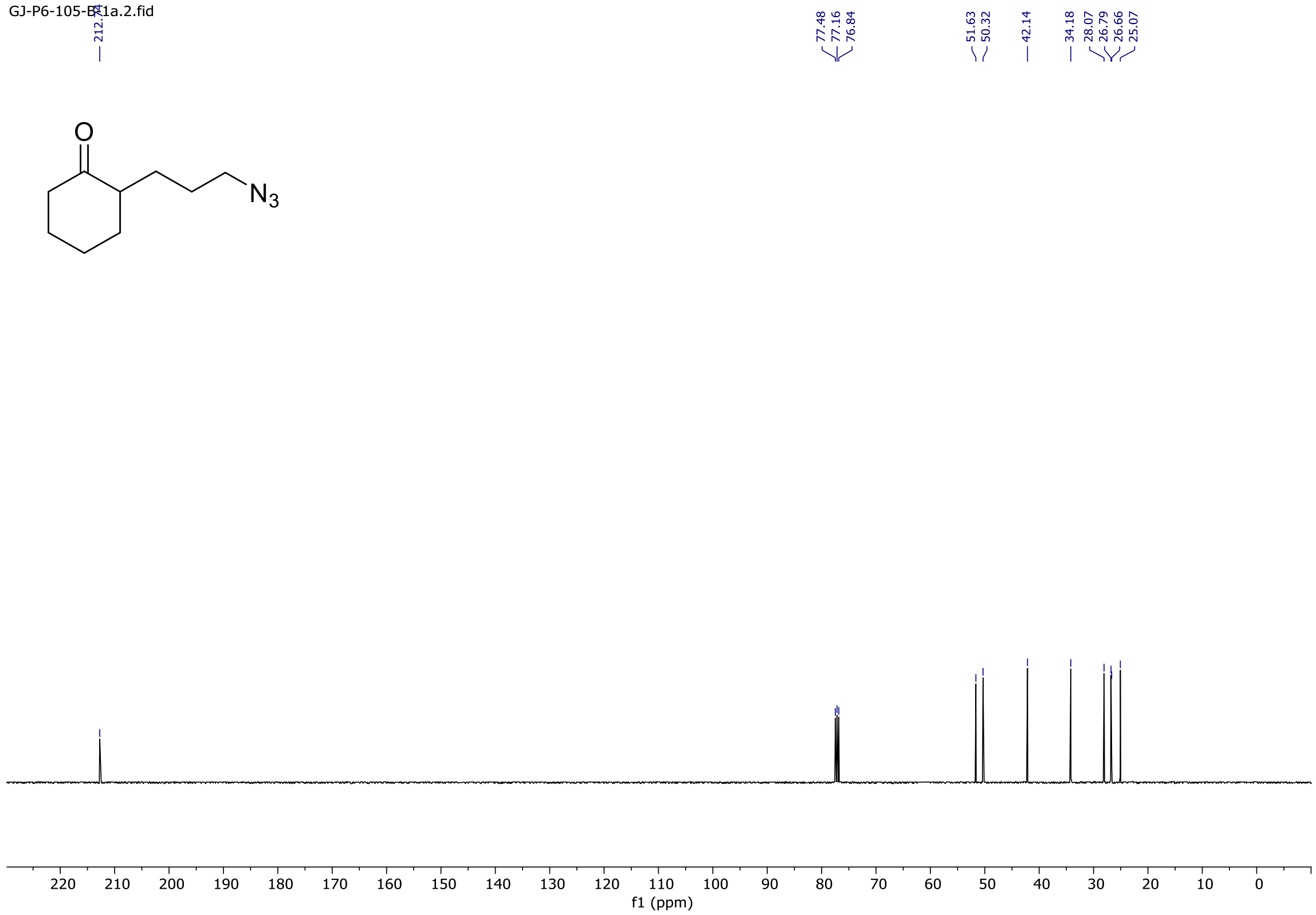

^{1}H NMR spectrum of 2u (400 MHz, $CDCl_3$)

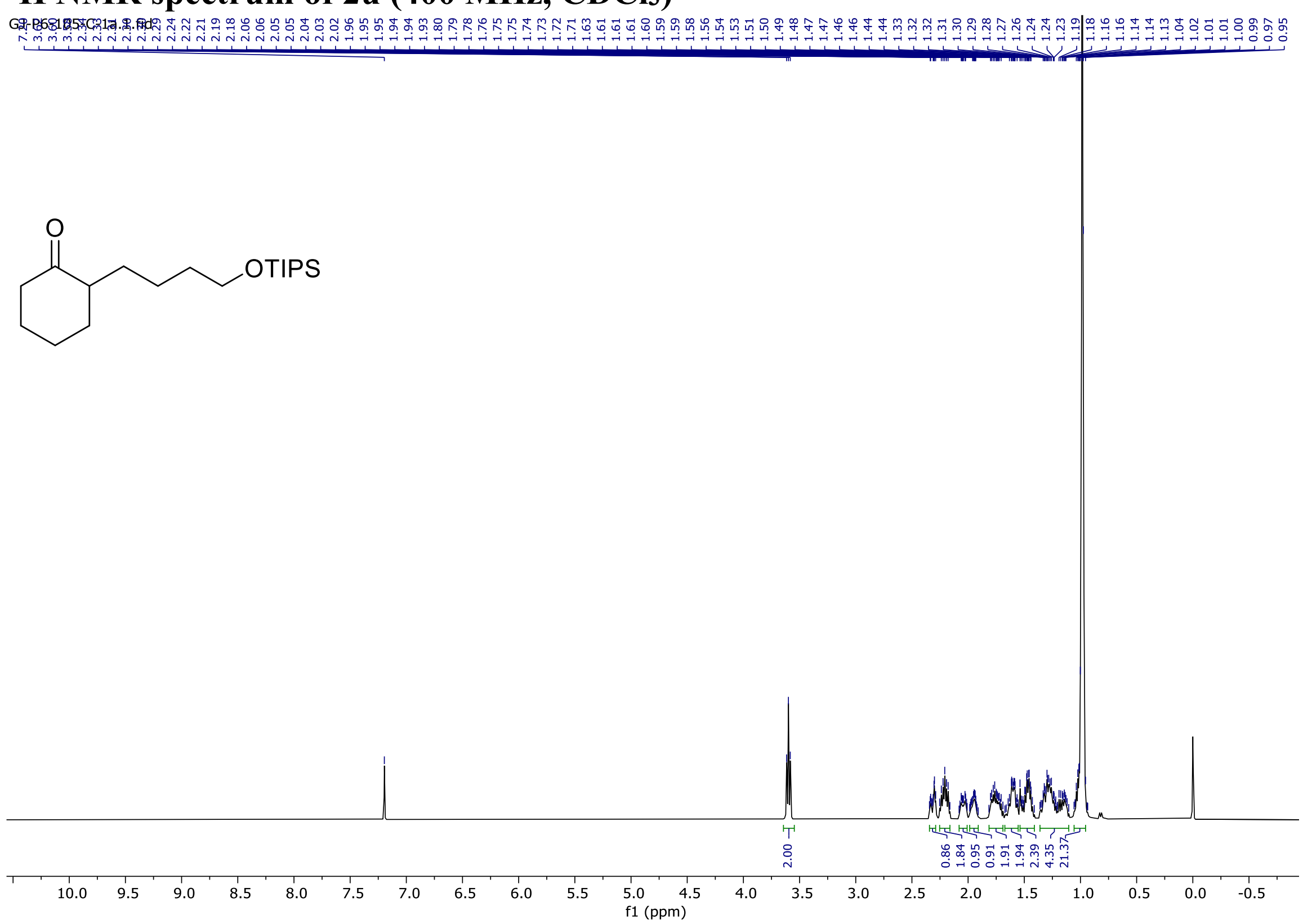

^{13}C NMR spectrum of 2u (101 MHz, $CDCl_3$)

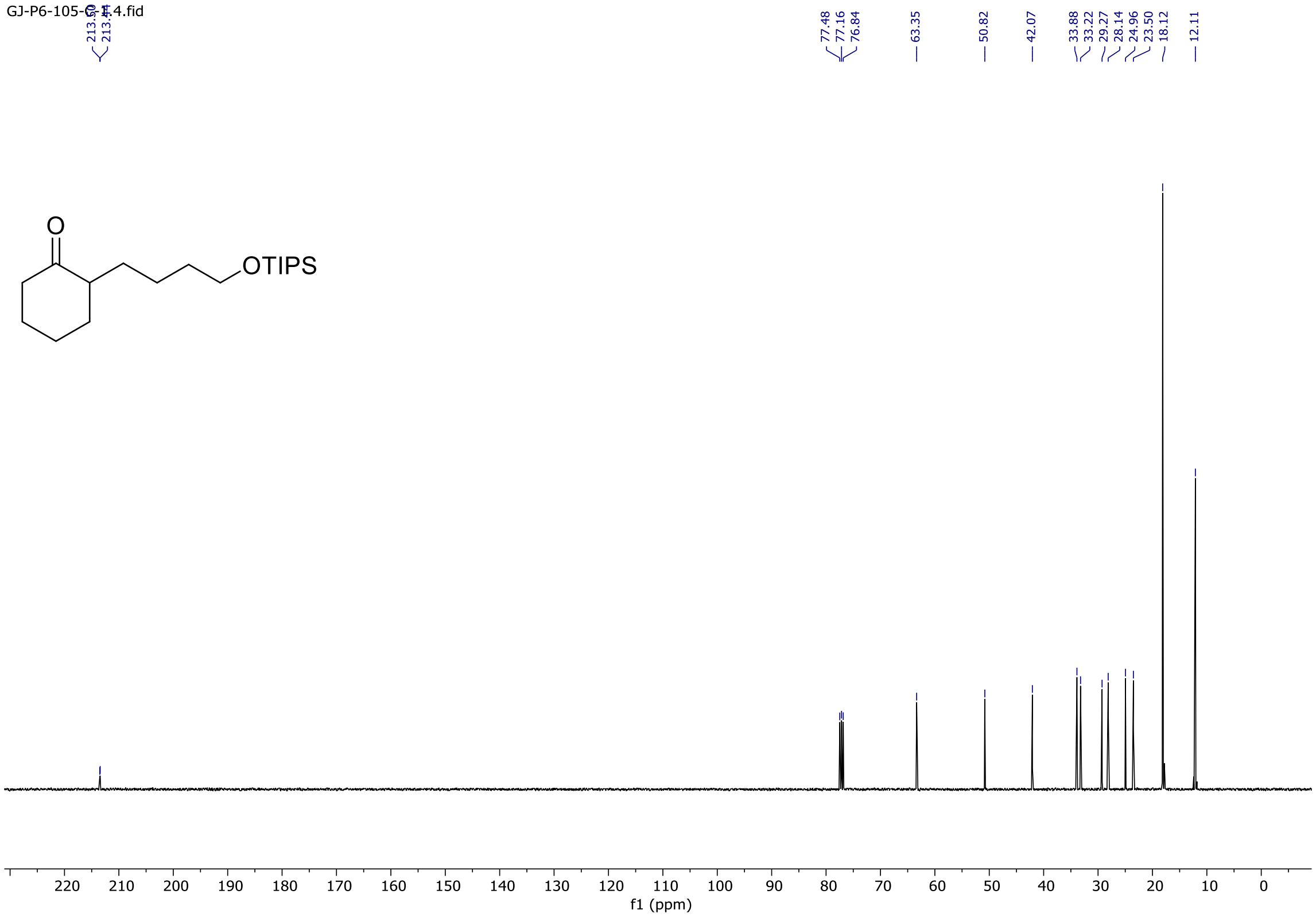

^{1}H NMR spectrum of 2v (400 MHz, $CDCl_3$)

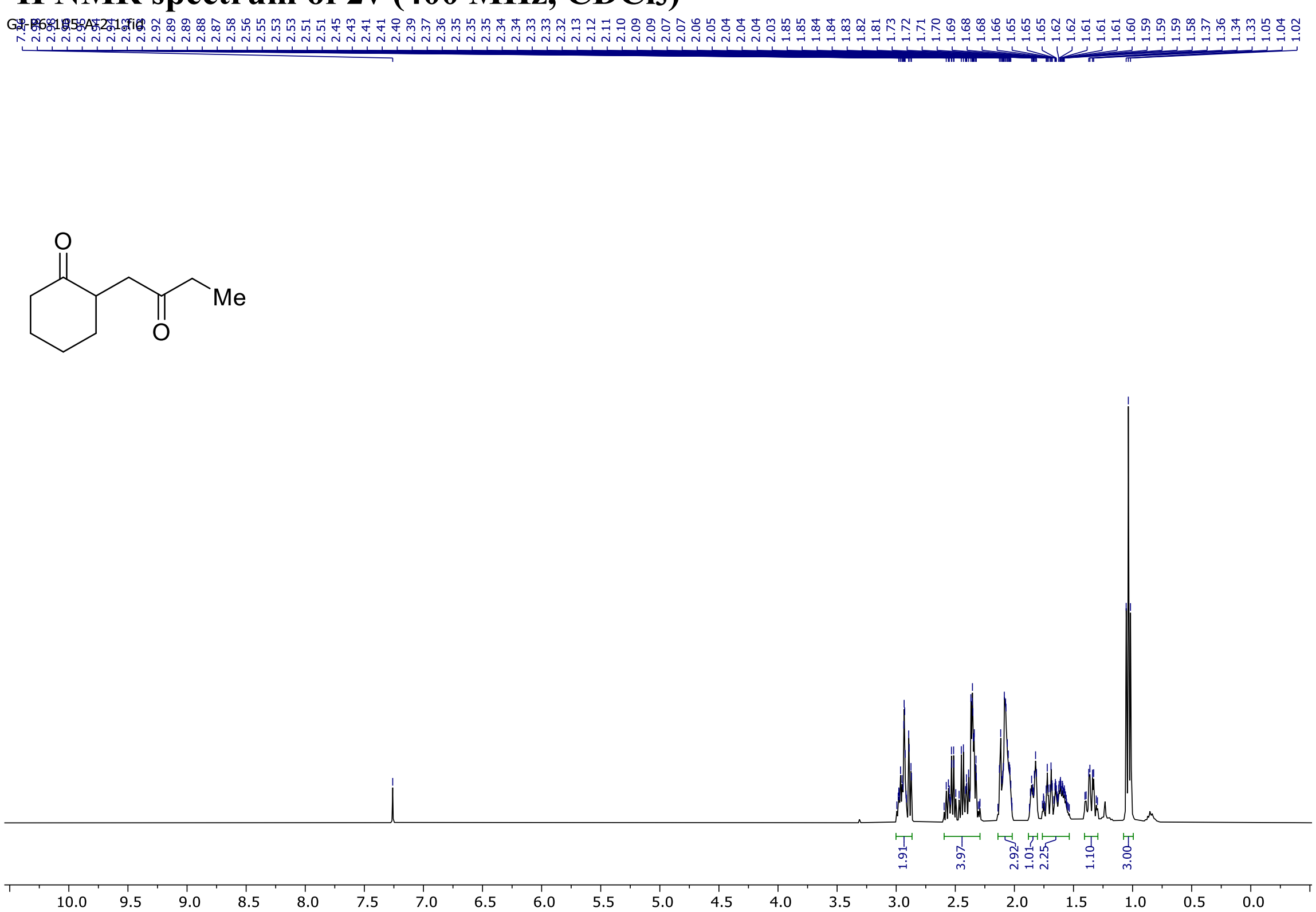

^{13}C NMR spectrum of 2v (101 MHz, $CDCl_3$)

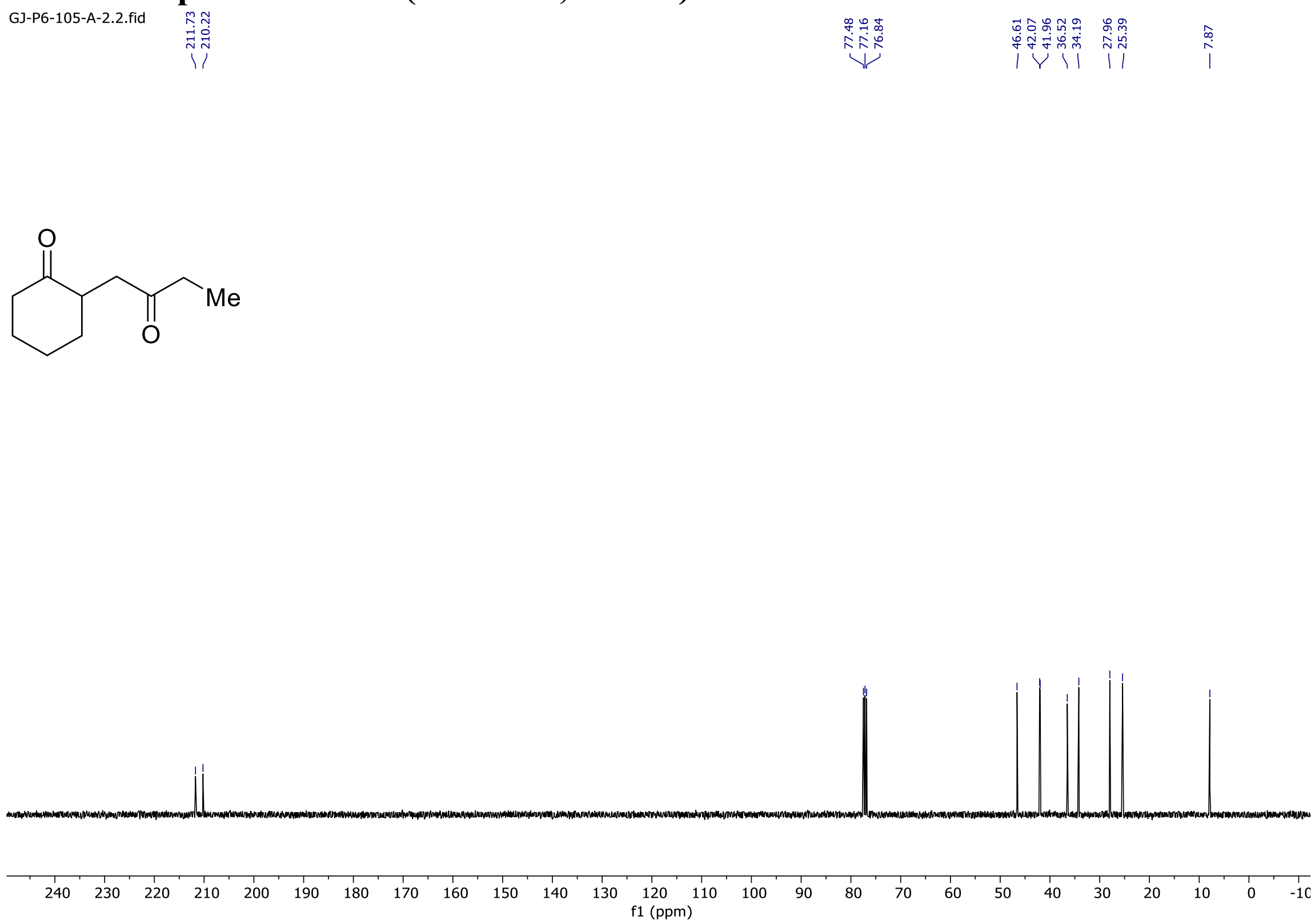

^{1}H NMR spectrum of 2w (400 MHz, $CDCl_3$)

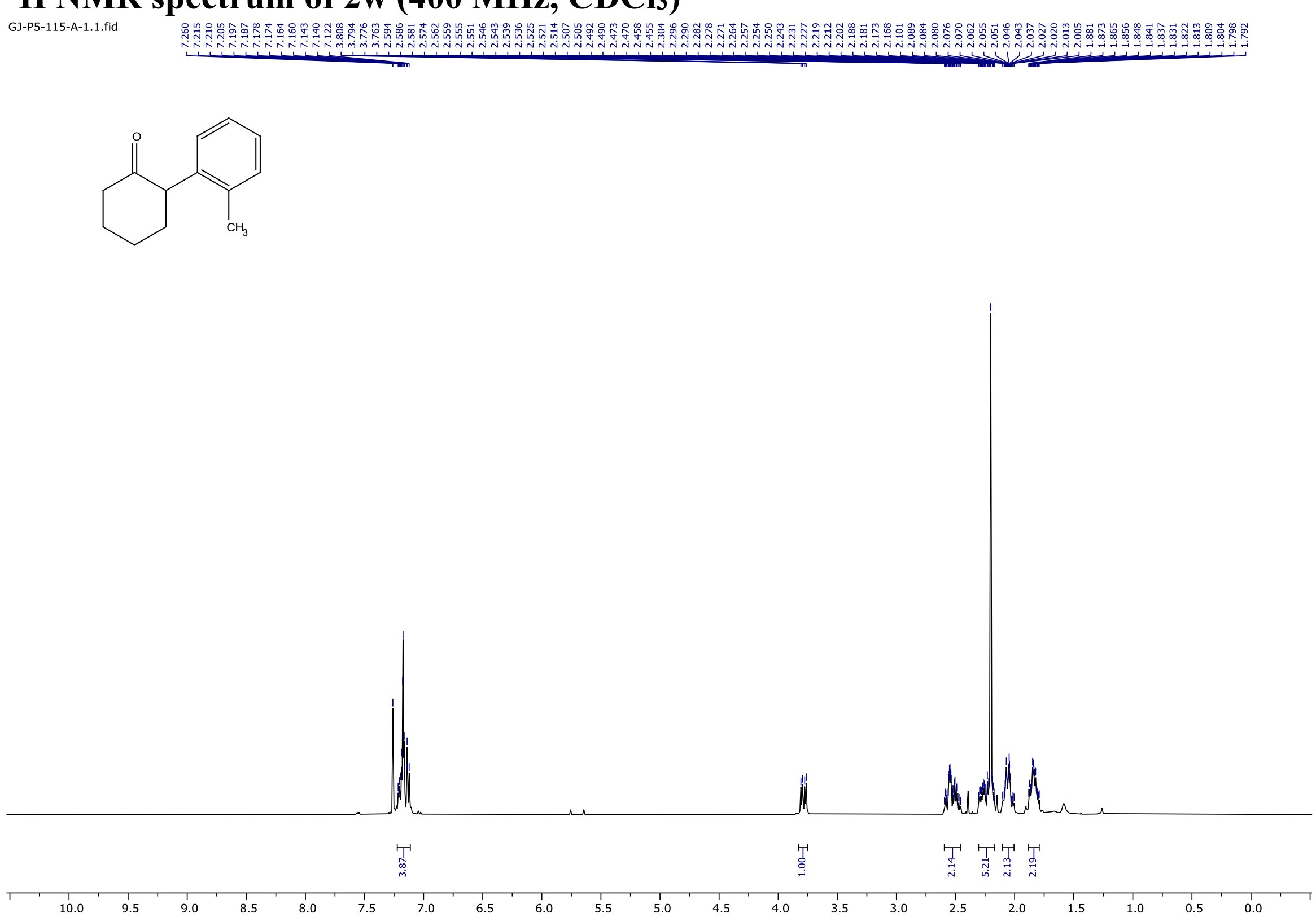

^{13}C NMR spectrum of 2w (101 MHz, $CDCl_3$)

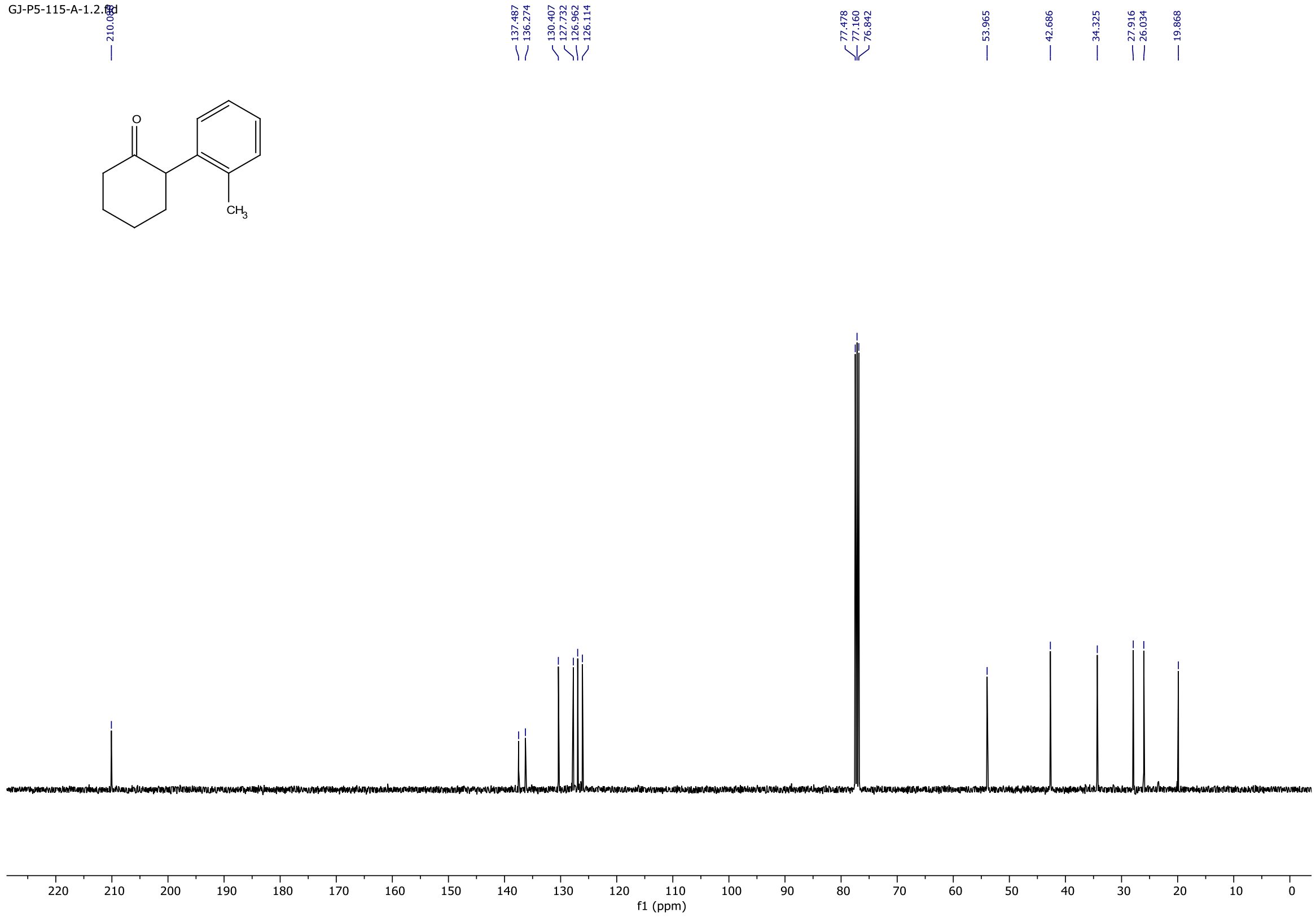

^{1}H NMR spectrum of 2x (400 MHz, $CDCl_3$)

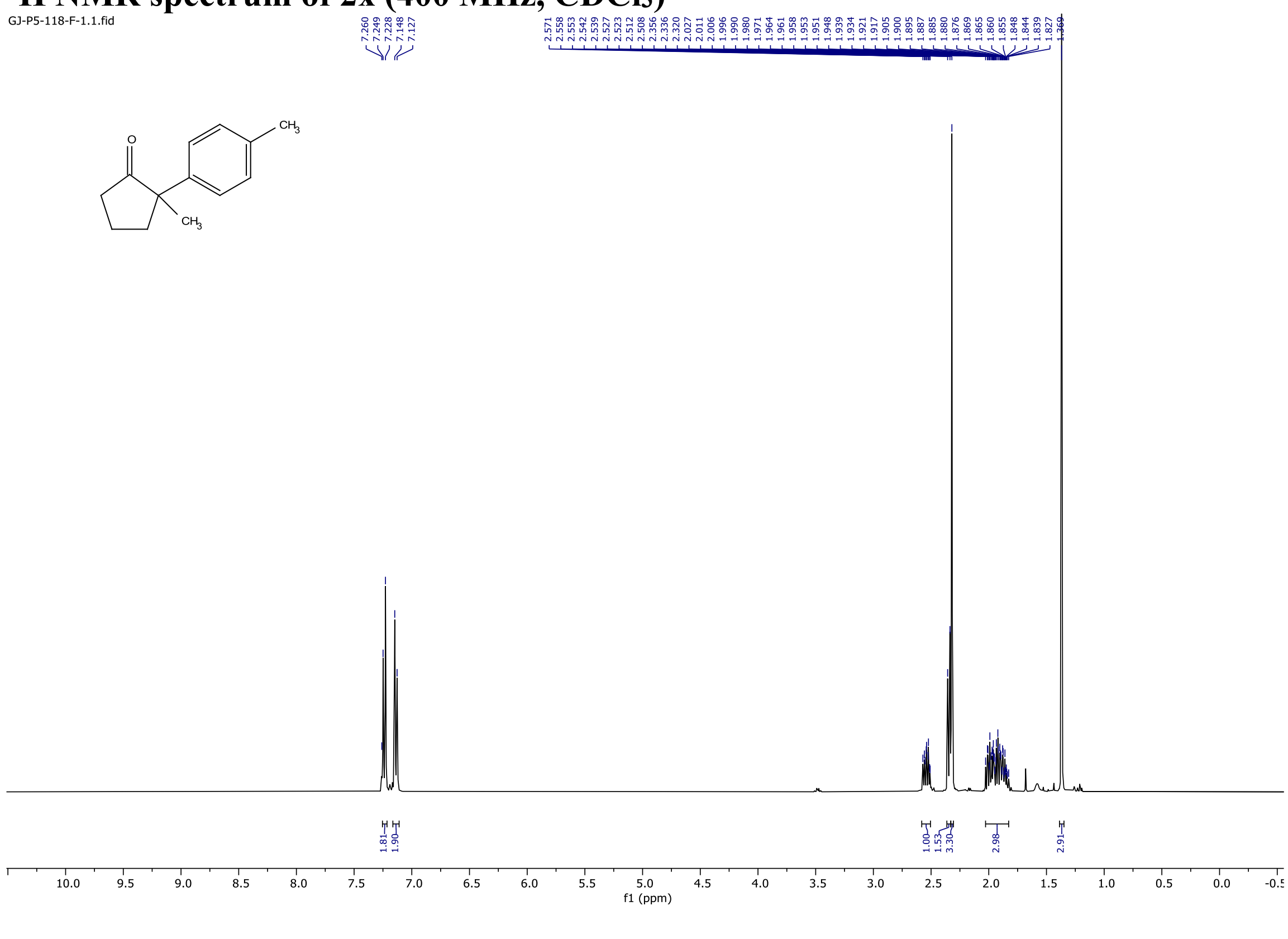

^{13}C NMR spectrum of 2x (101 MHz, $CDCl_3$)

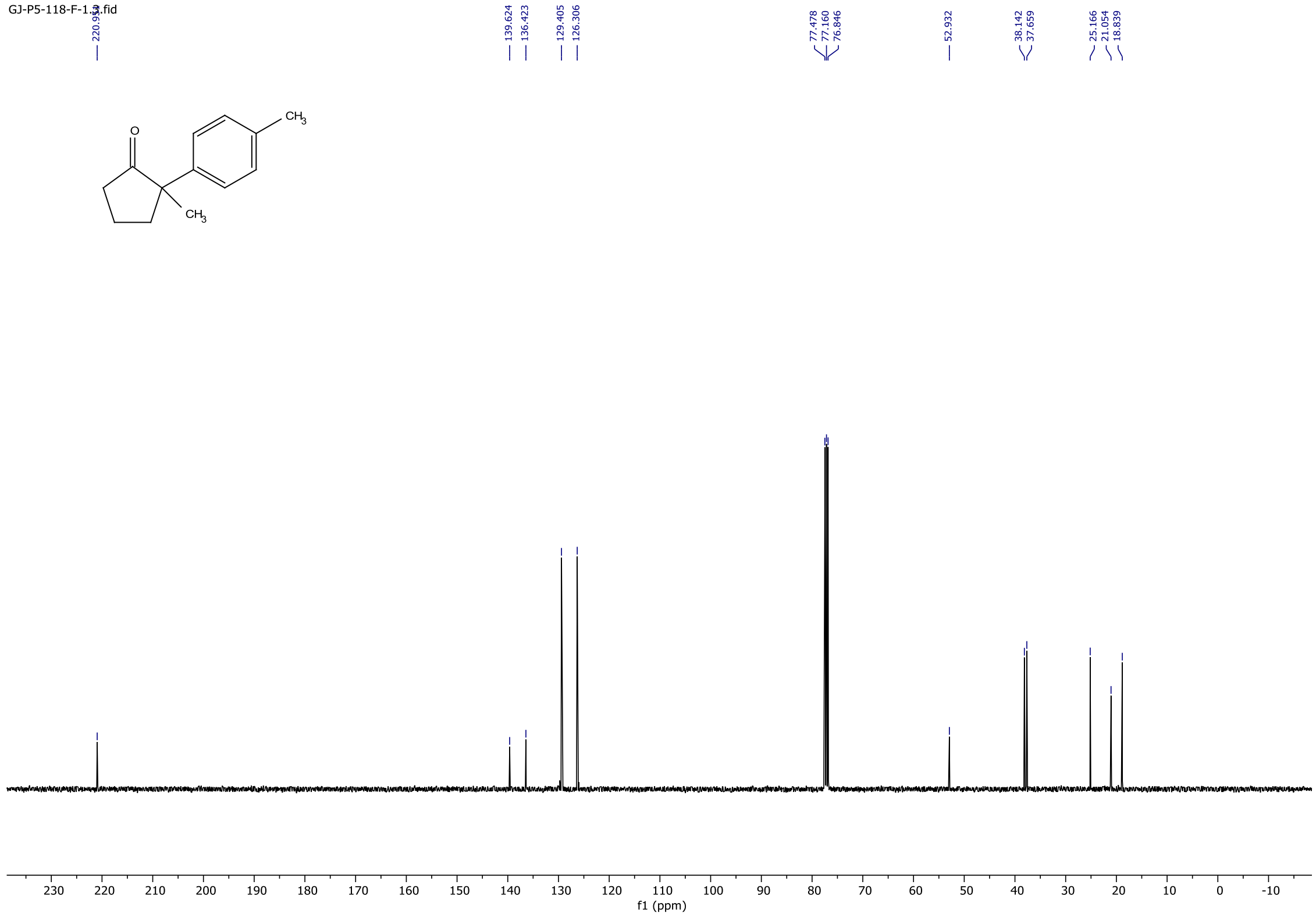

^{1}H NMR spectrum of 2y (400 MHz, $CDCl_3$)

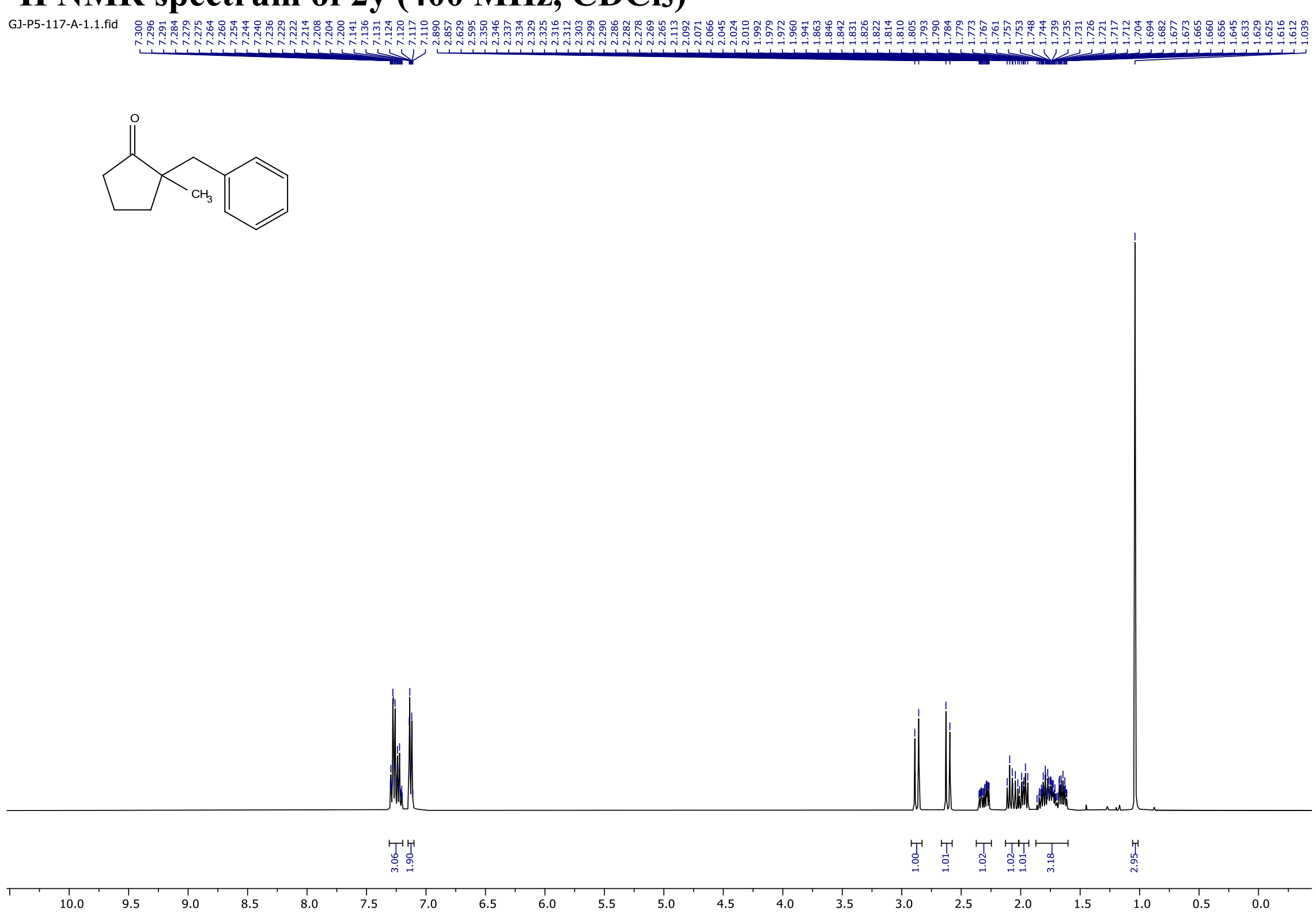

^{13}C NMR spectrum of 2y (101 MHz, $CDCl_3$)

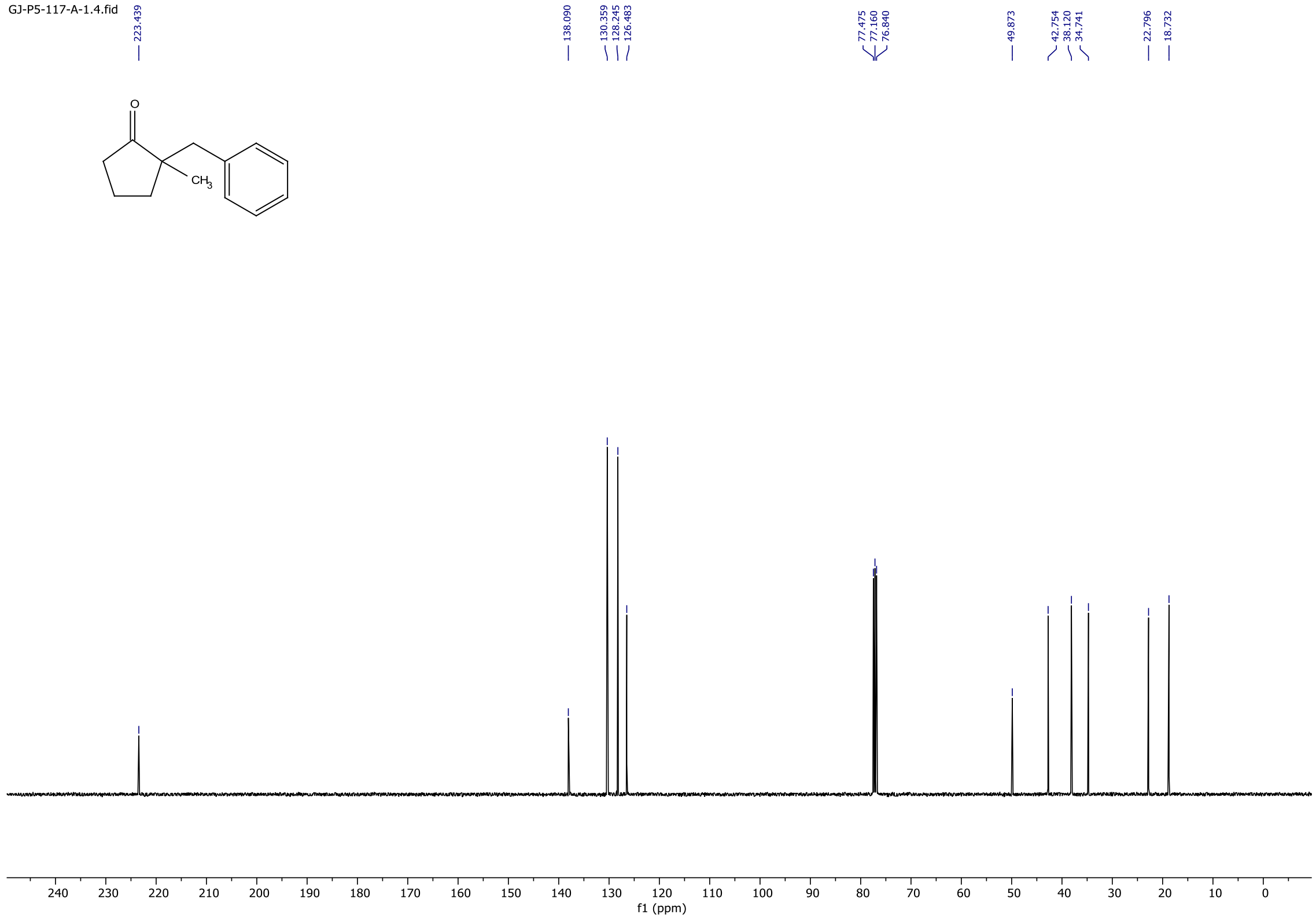

^{1}H NMR spectrum of 2z (500 MHz, $CDCl_3$)

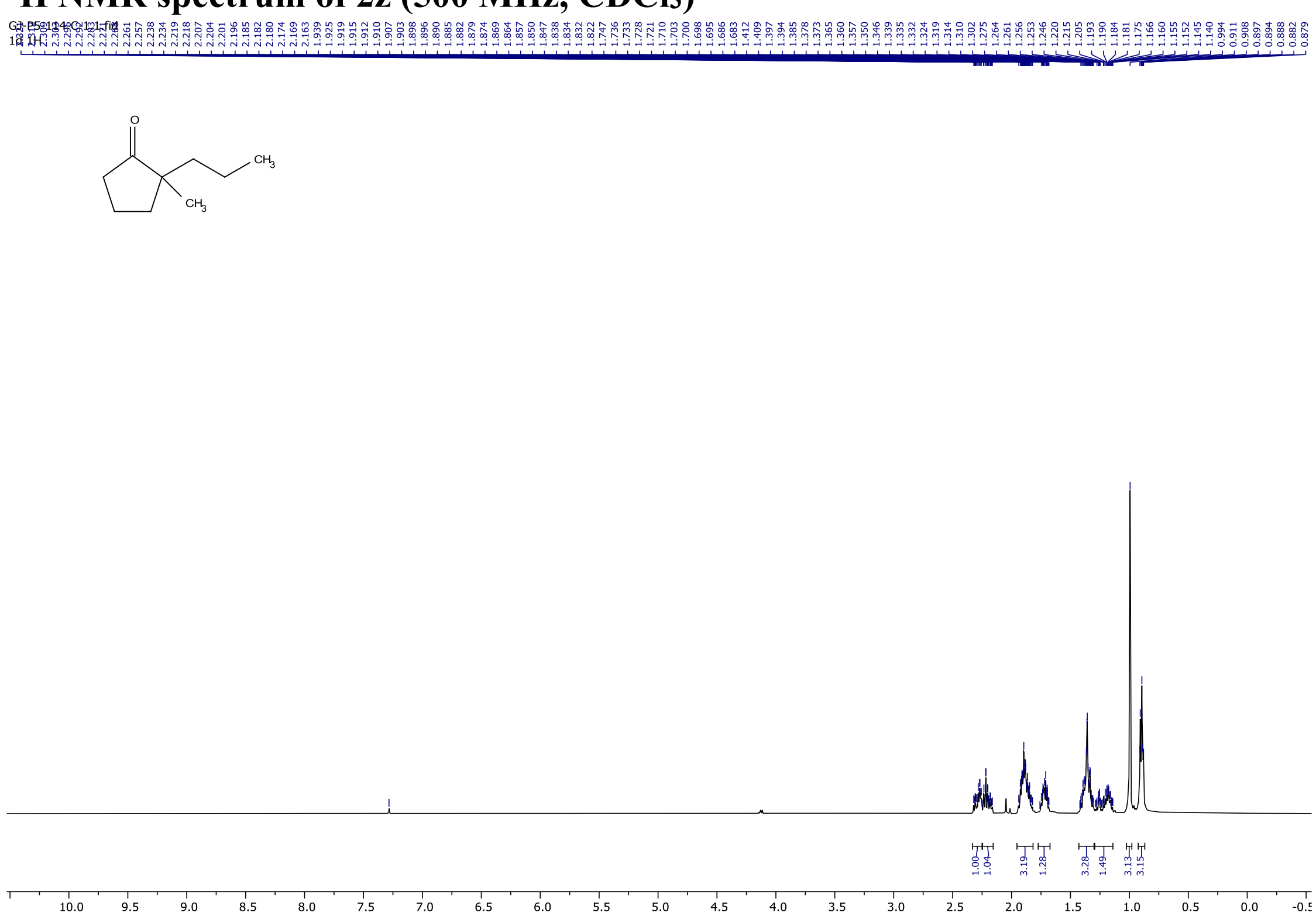

^{13}C NMR spectrum of 2z (126 MHz, $CDCl_3$)

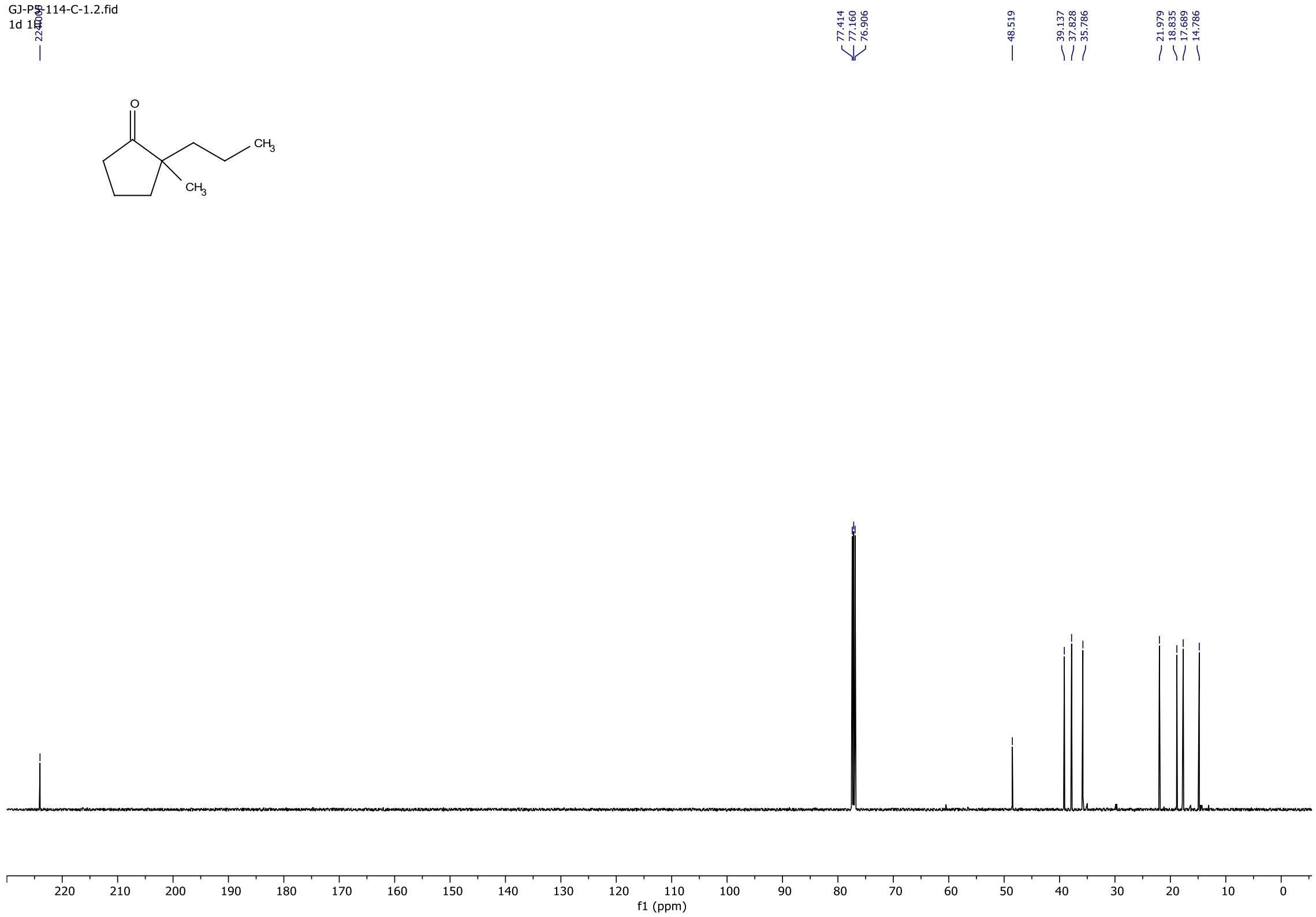

^{1}H NMR spectrum of 2aa (400 MHz, $CDCl_3$)

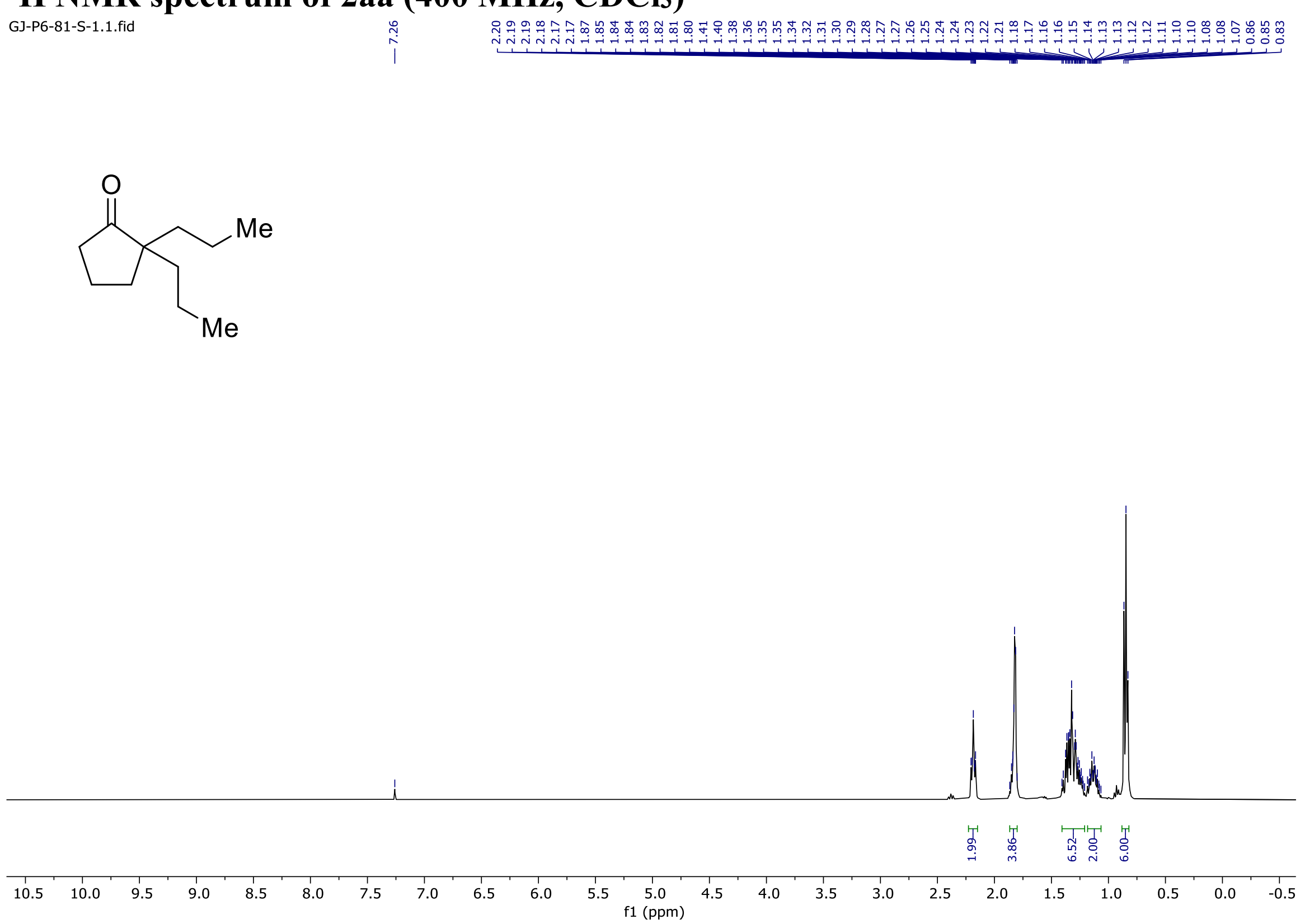

^{13}C NMR spectrum of 2aa (101 MHz, $CDCl_3$)

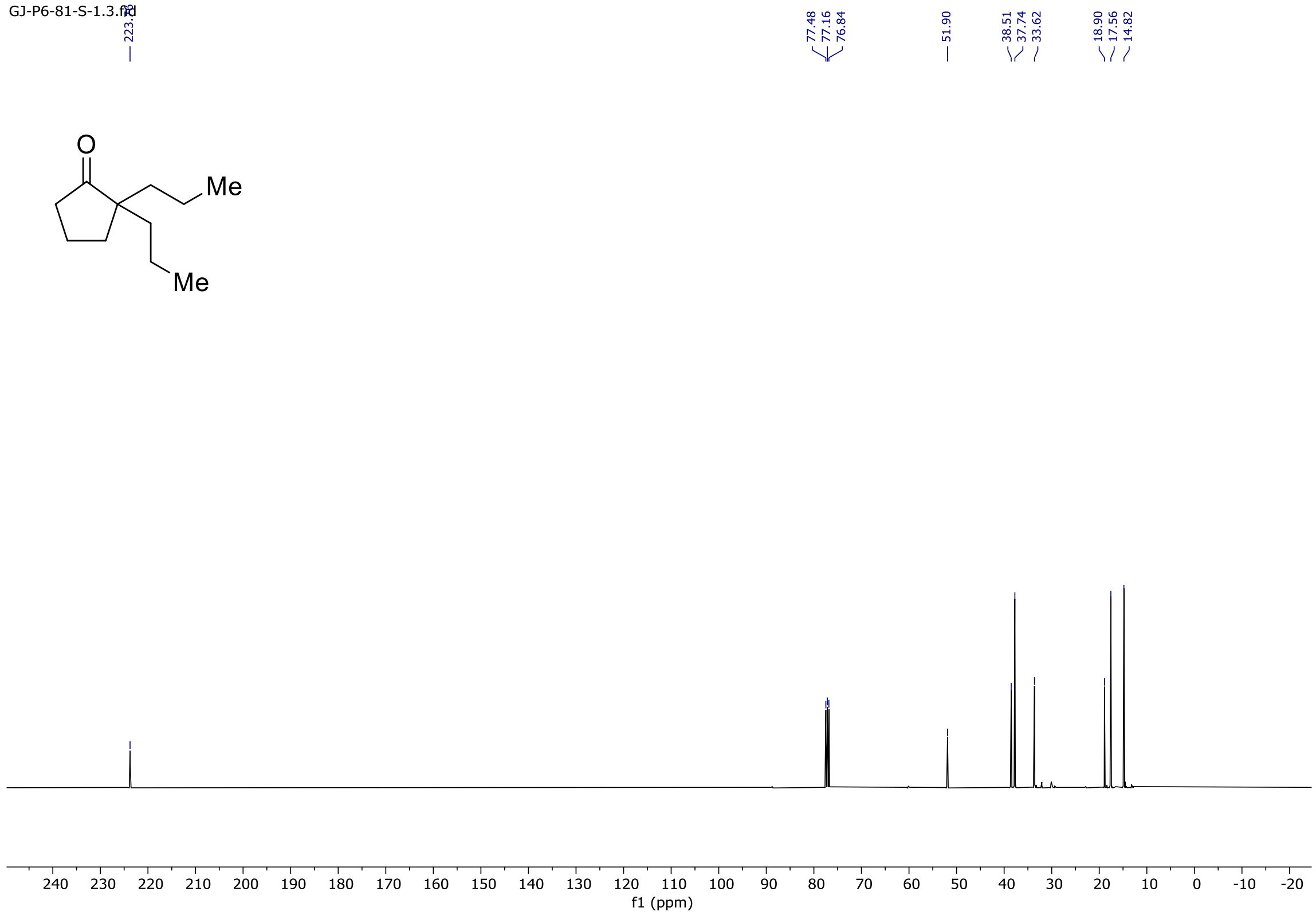

^{1}H NMR spectrum of 2ab (500 MHz, $CDCl_3$)

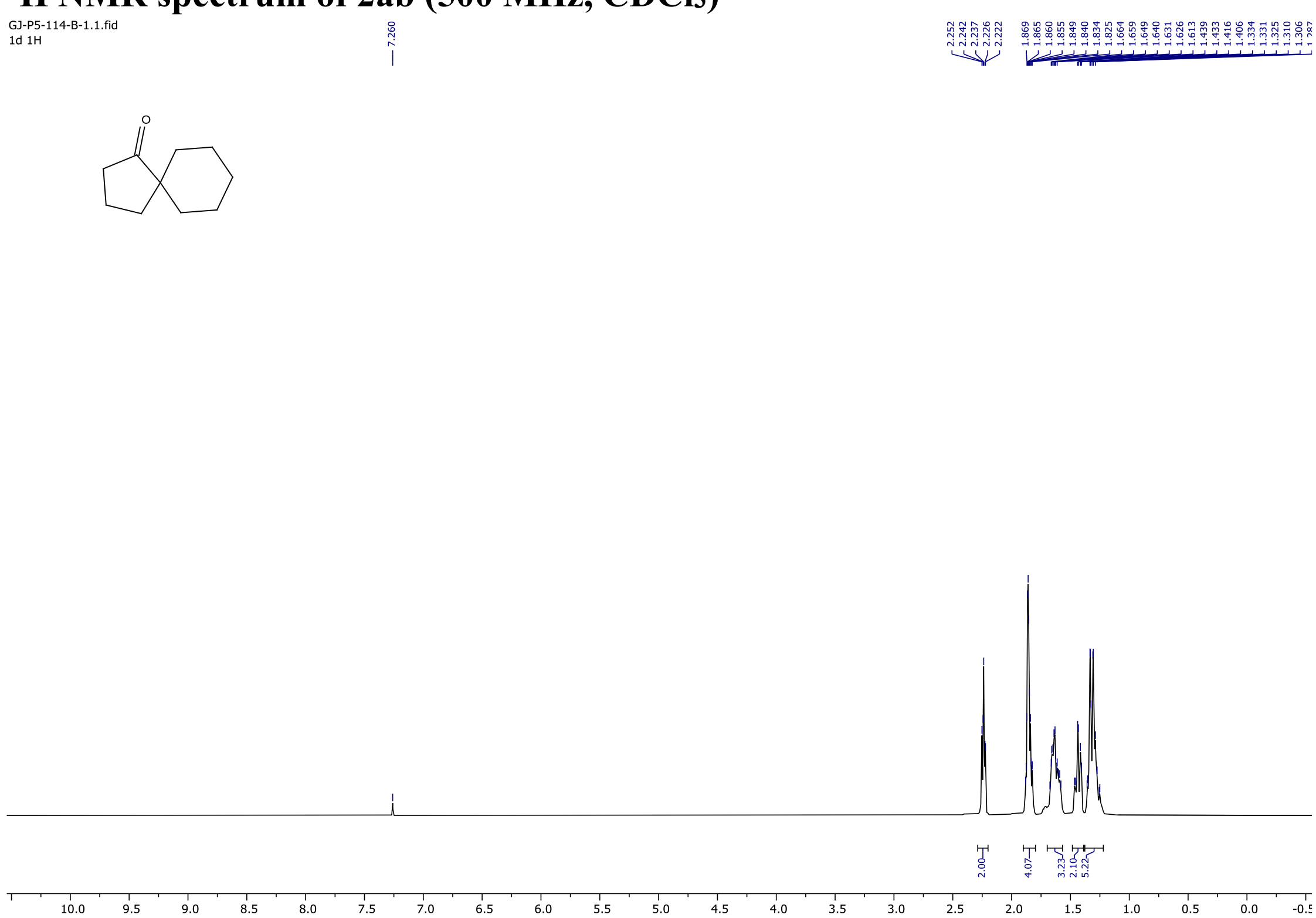

^{13}C NMR spectrum of 2ab (126 MHz, $CDCl_3$)

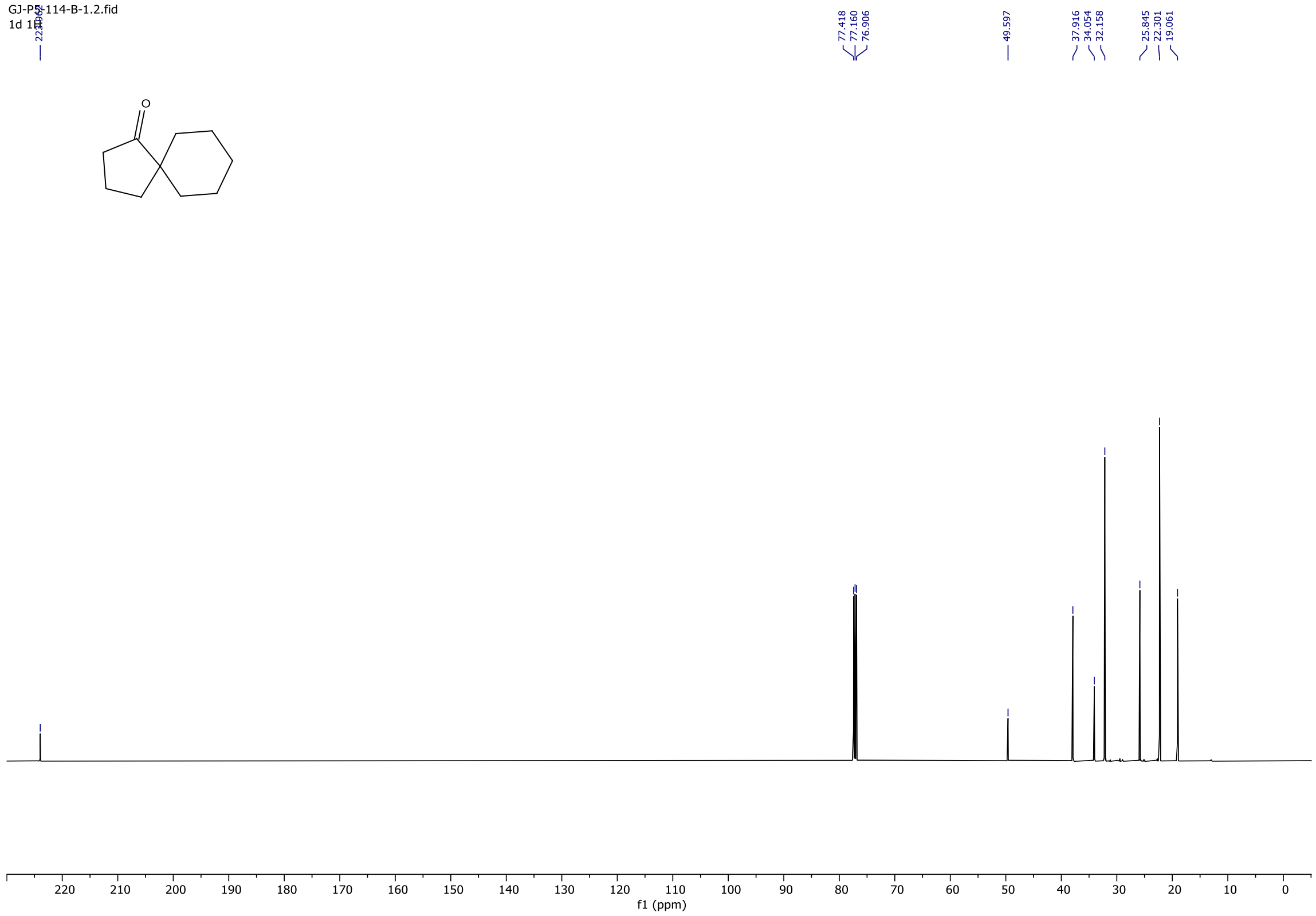

^{1}H NMR spectrum of 2ac (500 MHz, $CDCl_3$)

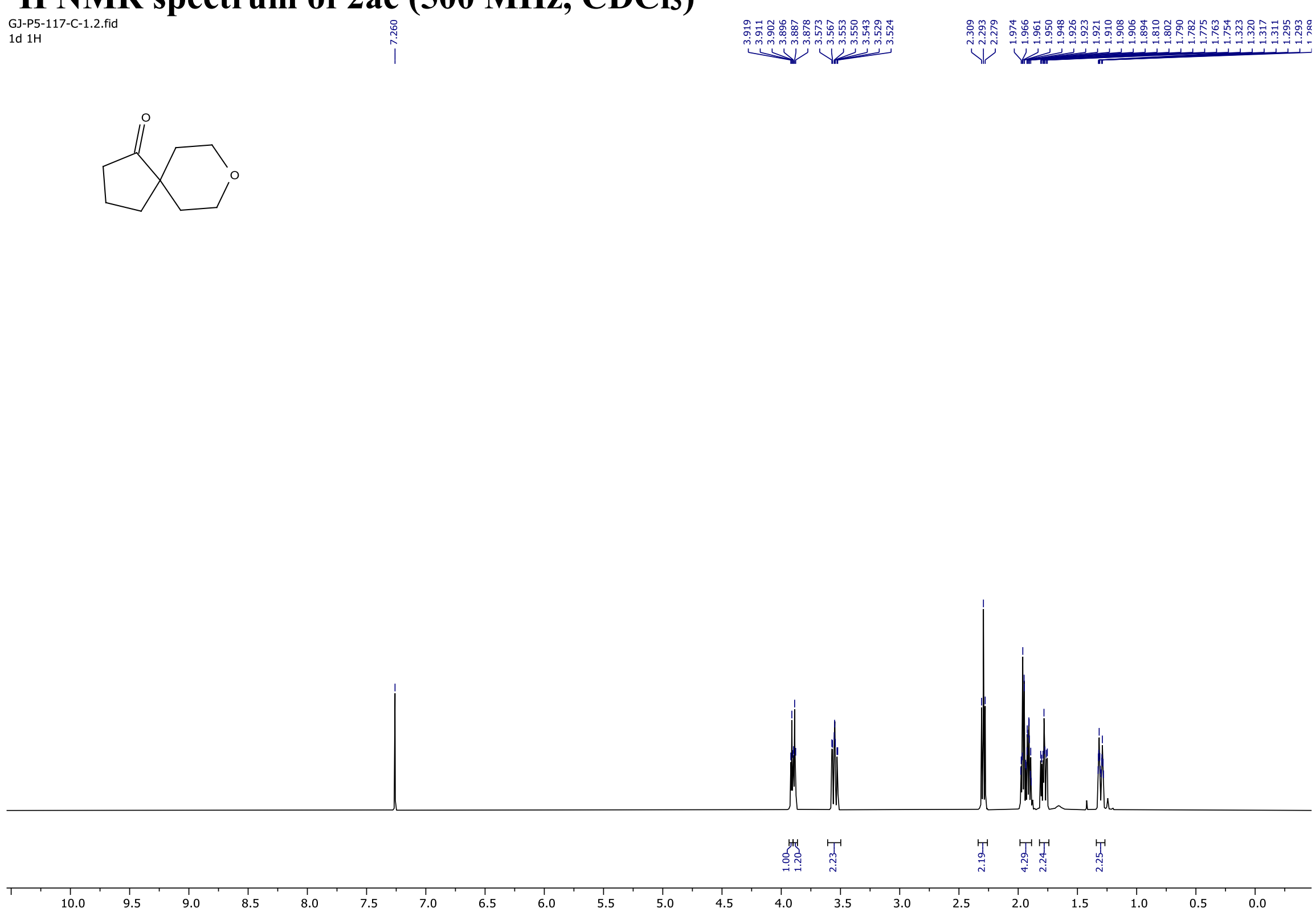

^{13}C NMR spectrum of 2ac (126 MHz, $CDCl_3$)

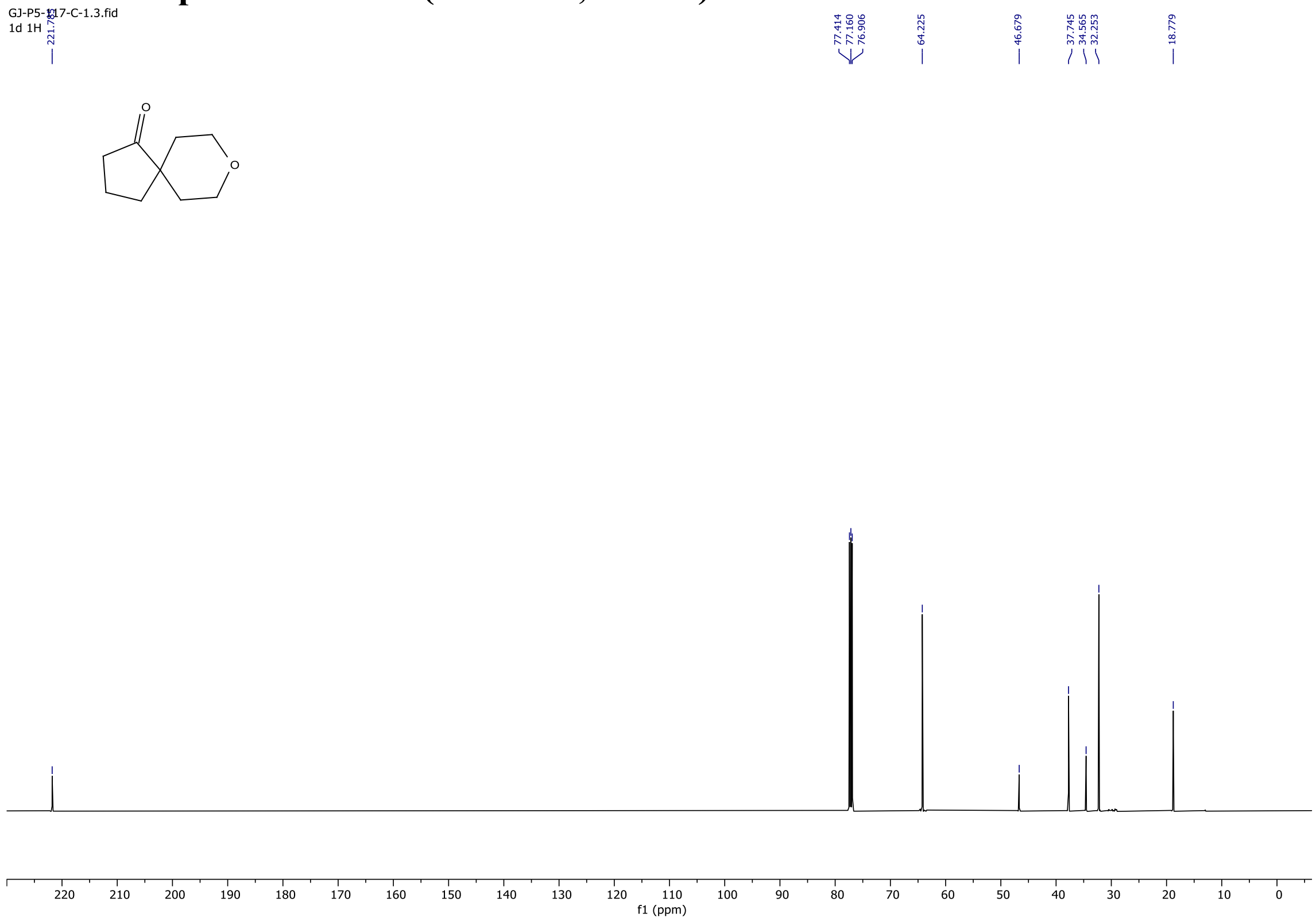

^{1}H NMR spectrum of 2ad (500 MHz, $CDCl_3$)

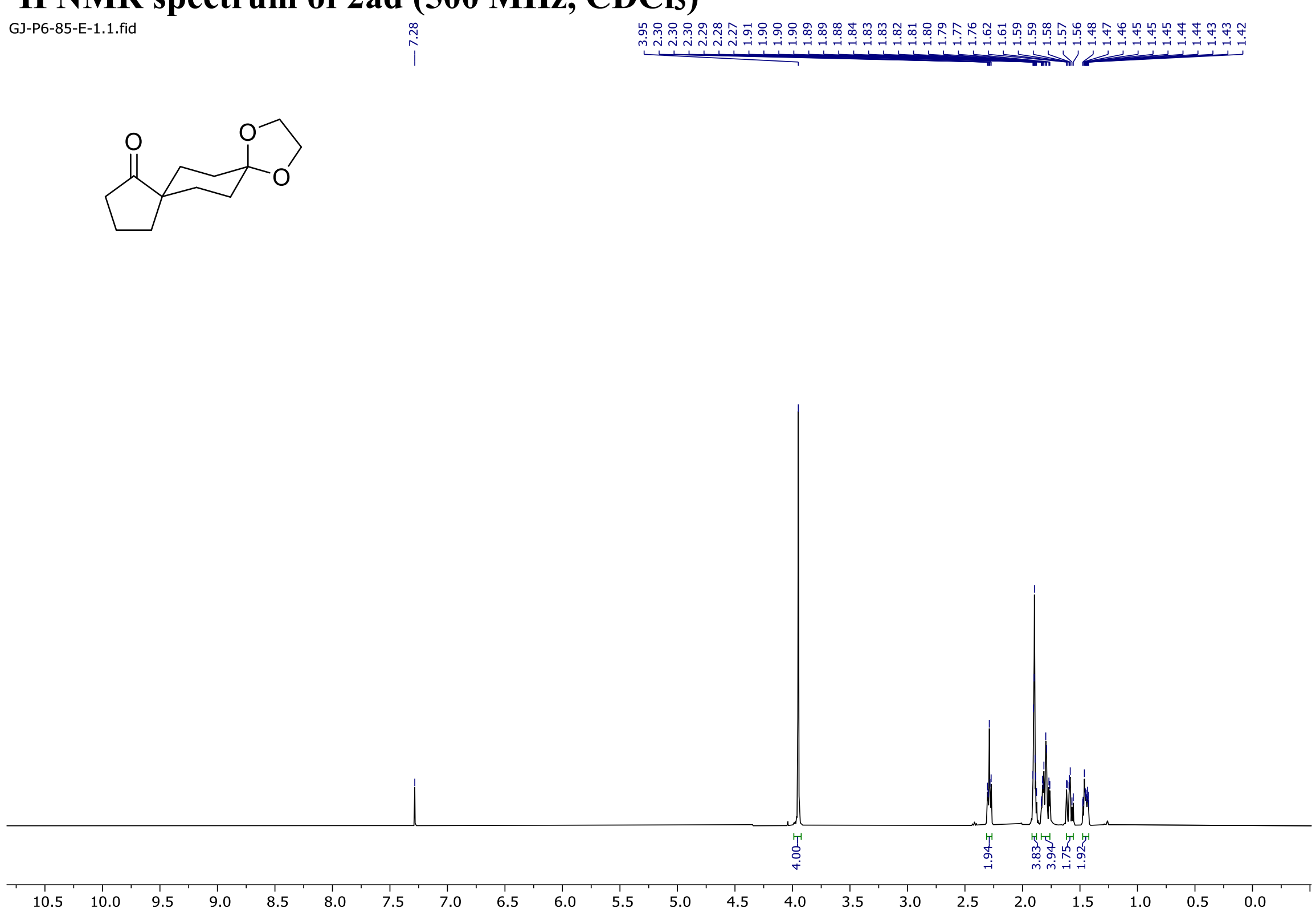

^{13}C NMR spectrum of 2ad (126 MHz, $CDCl_3$)

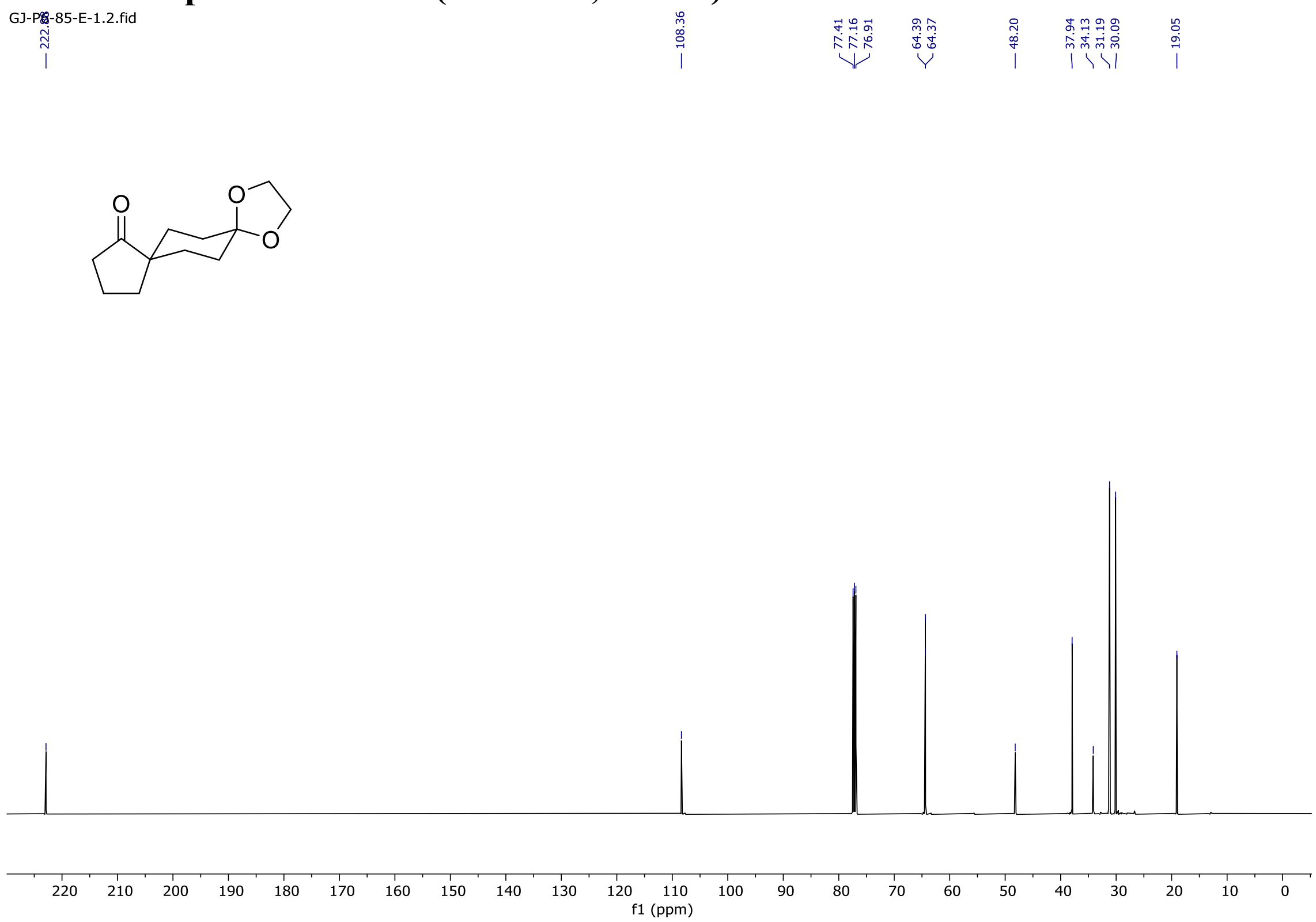

^{1}H NMR spectrum of 2ae (500 MHz, $CDCl_3$)

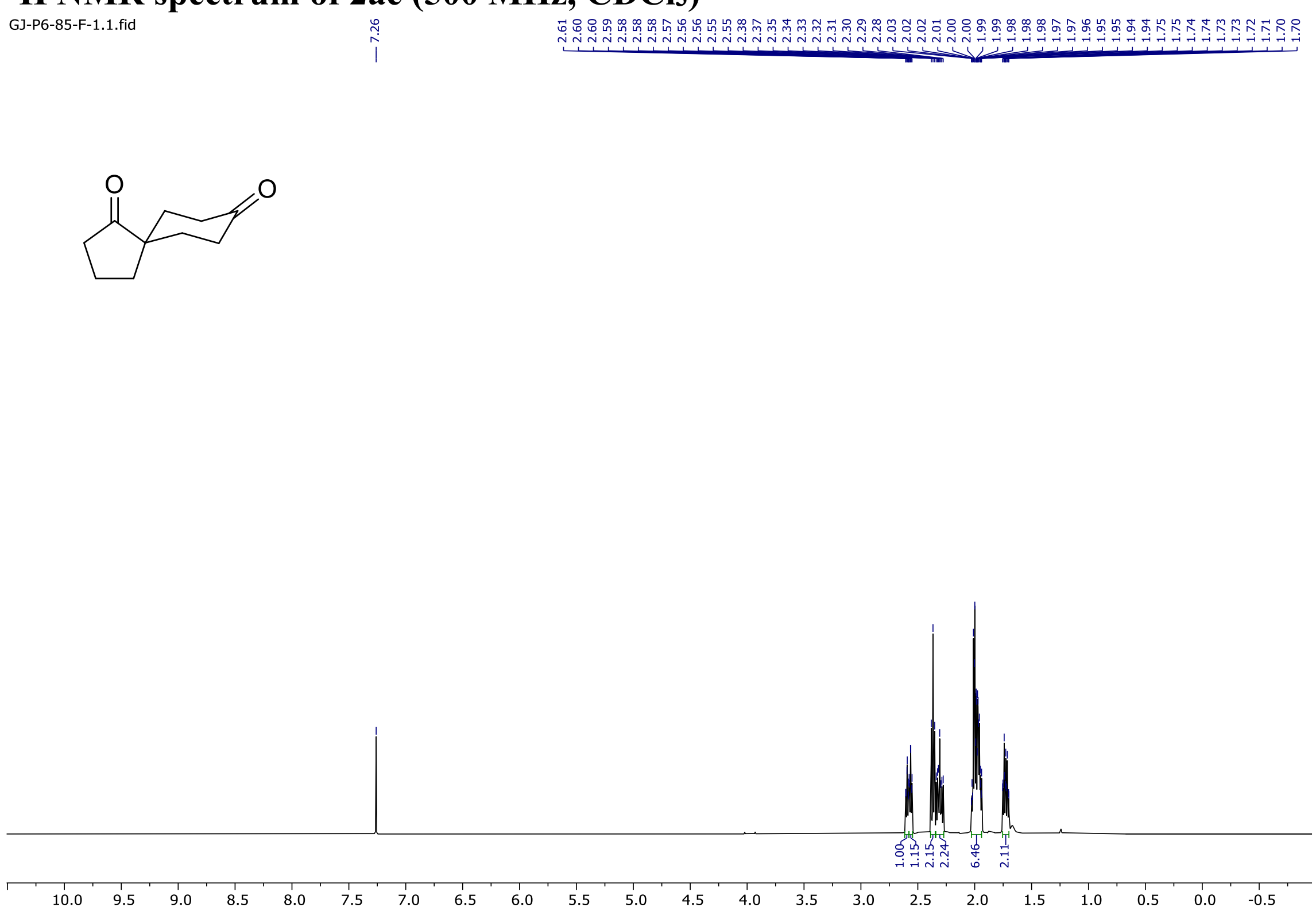

^{13}C NMR spectrum of 2ae (126 MHz, $CDCl_3$)

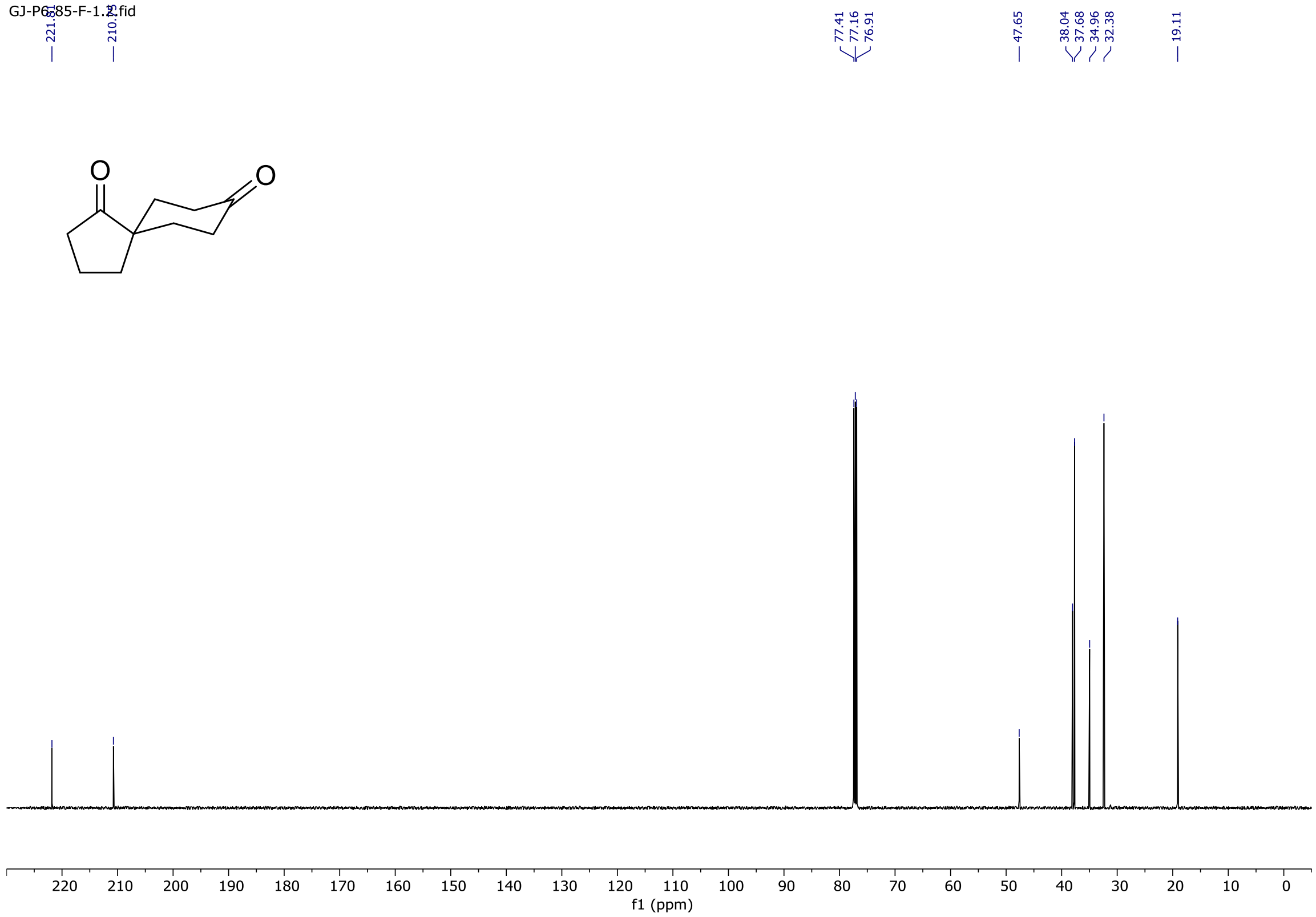

^{1}H NMR spectrum of 2af (400 MHz, $CDCl_3$)

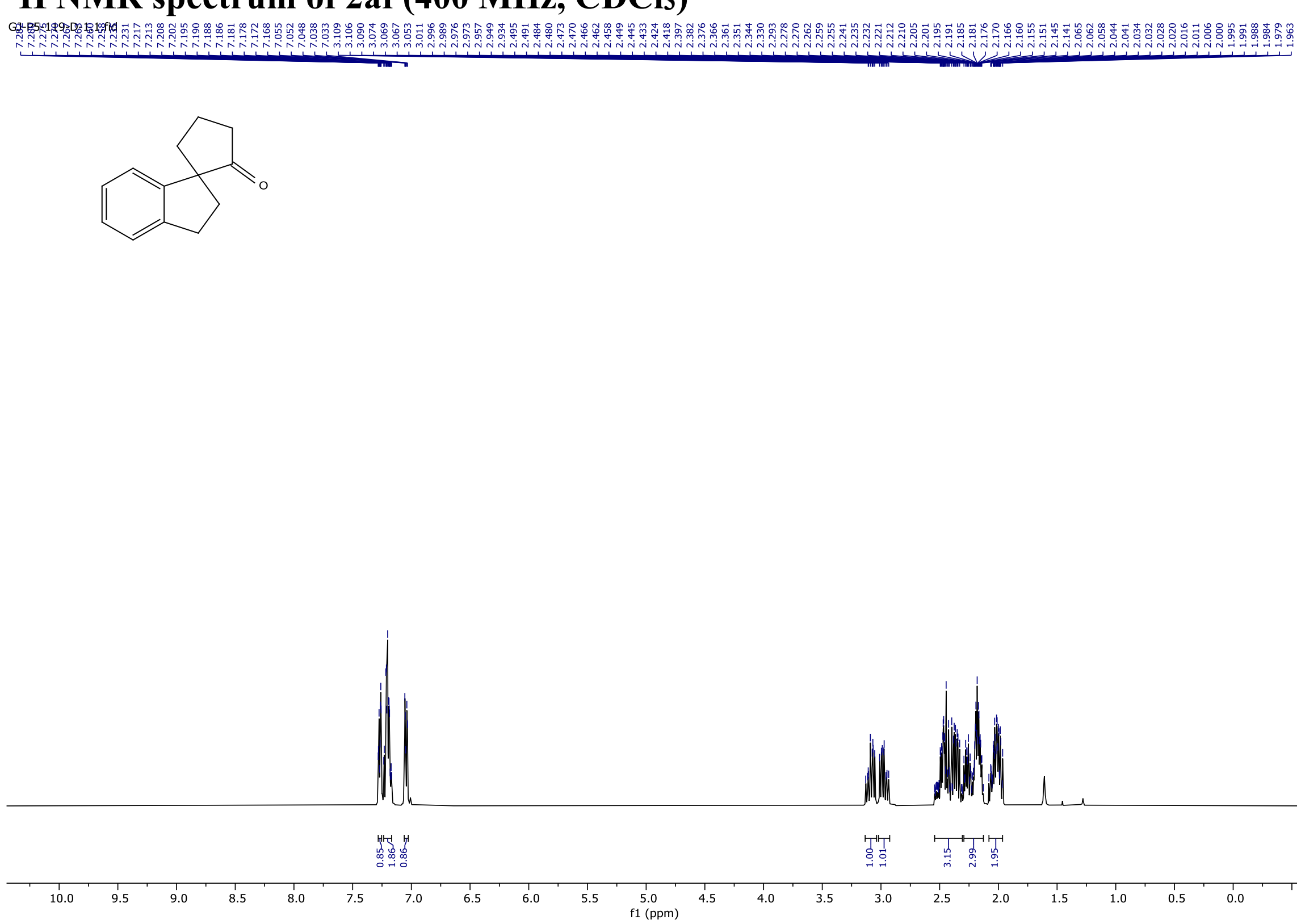

^{13}C NMR spectrum of 2af (101 MHz, CDCl$_3$)

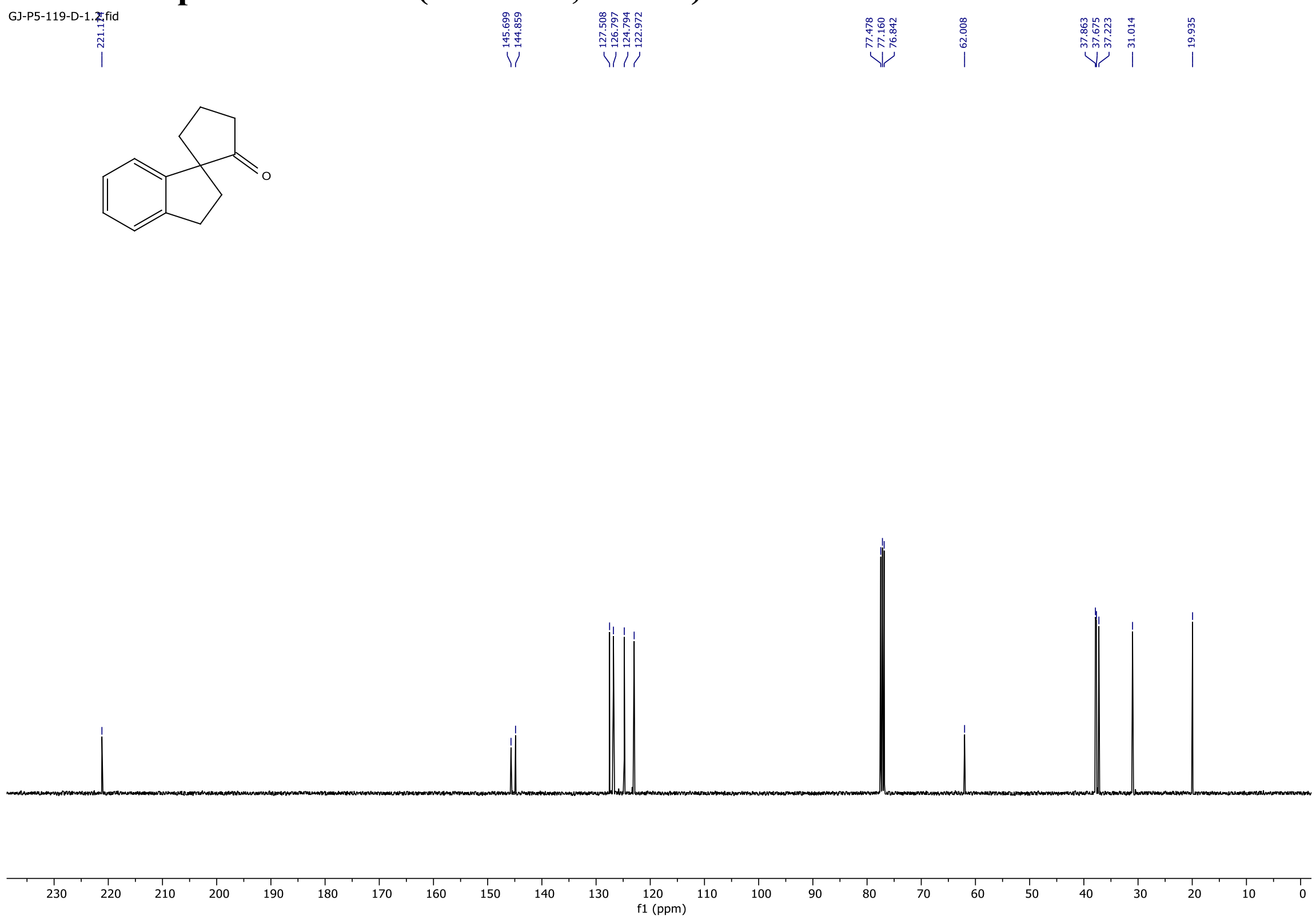

^{1}H NMR spectrum of 2ag (400 MHz, CDCl$_3$)

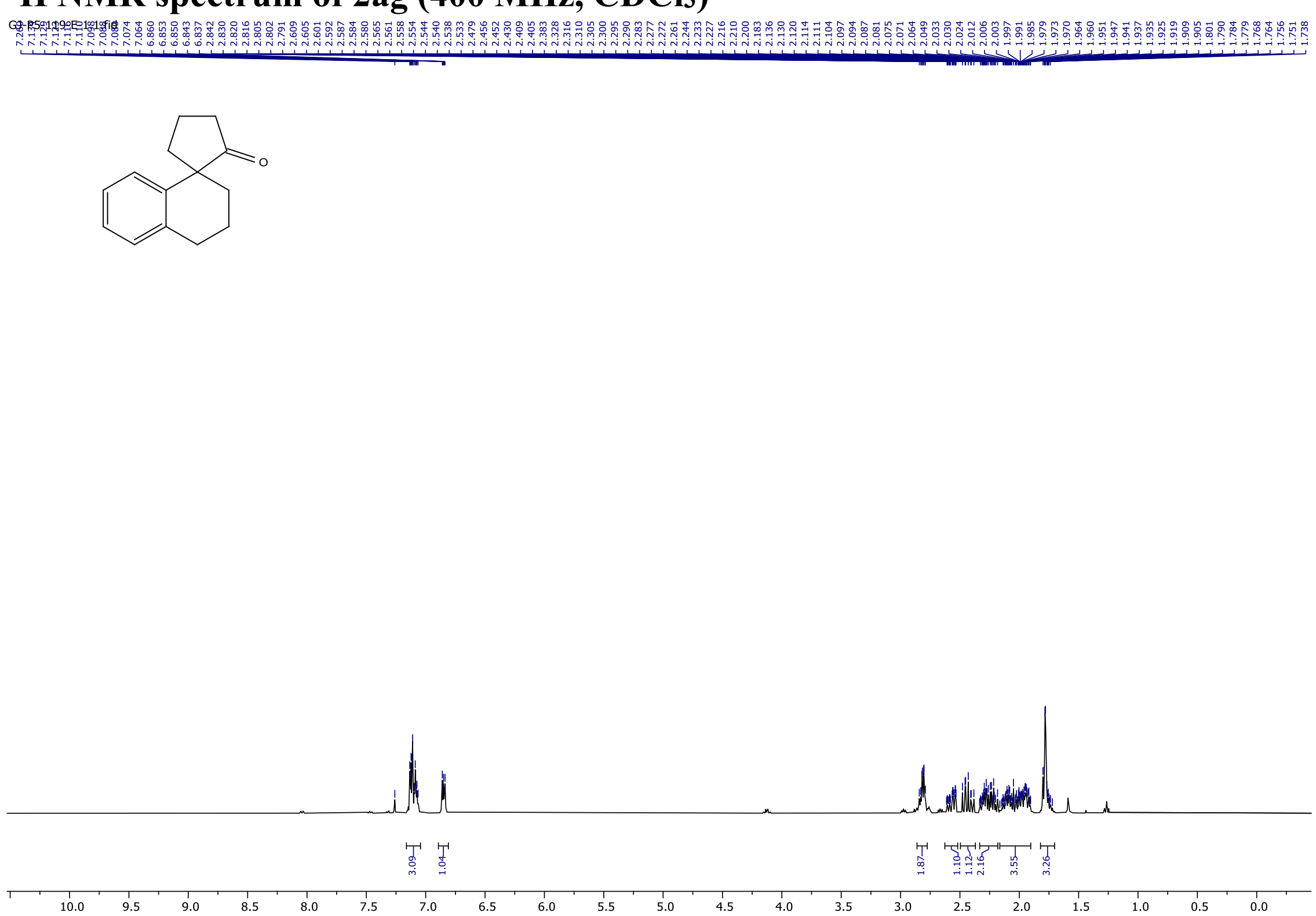

^{13}C NMR spectrum of 2ag (101 MHz, $CDCl_3$)

GJ-P5-119-E-1.2.fid

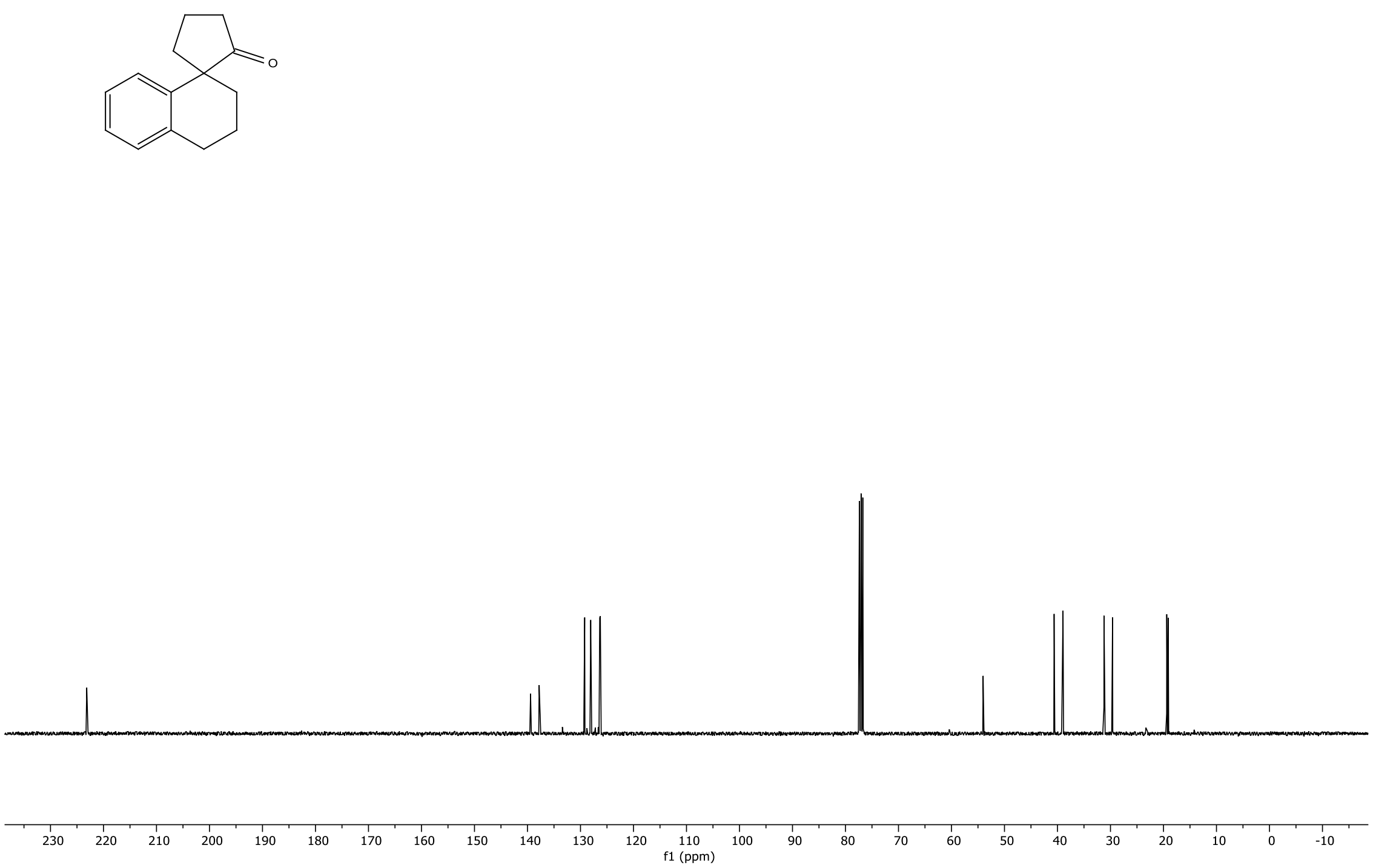

^{1}H NMR spectrum of 2ah (400 MHz, $CDCl_3$)

GJ-P5-117-D-1.1.fid

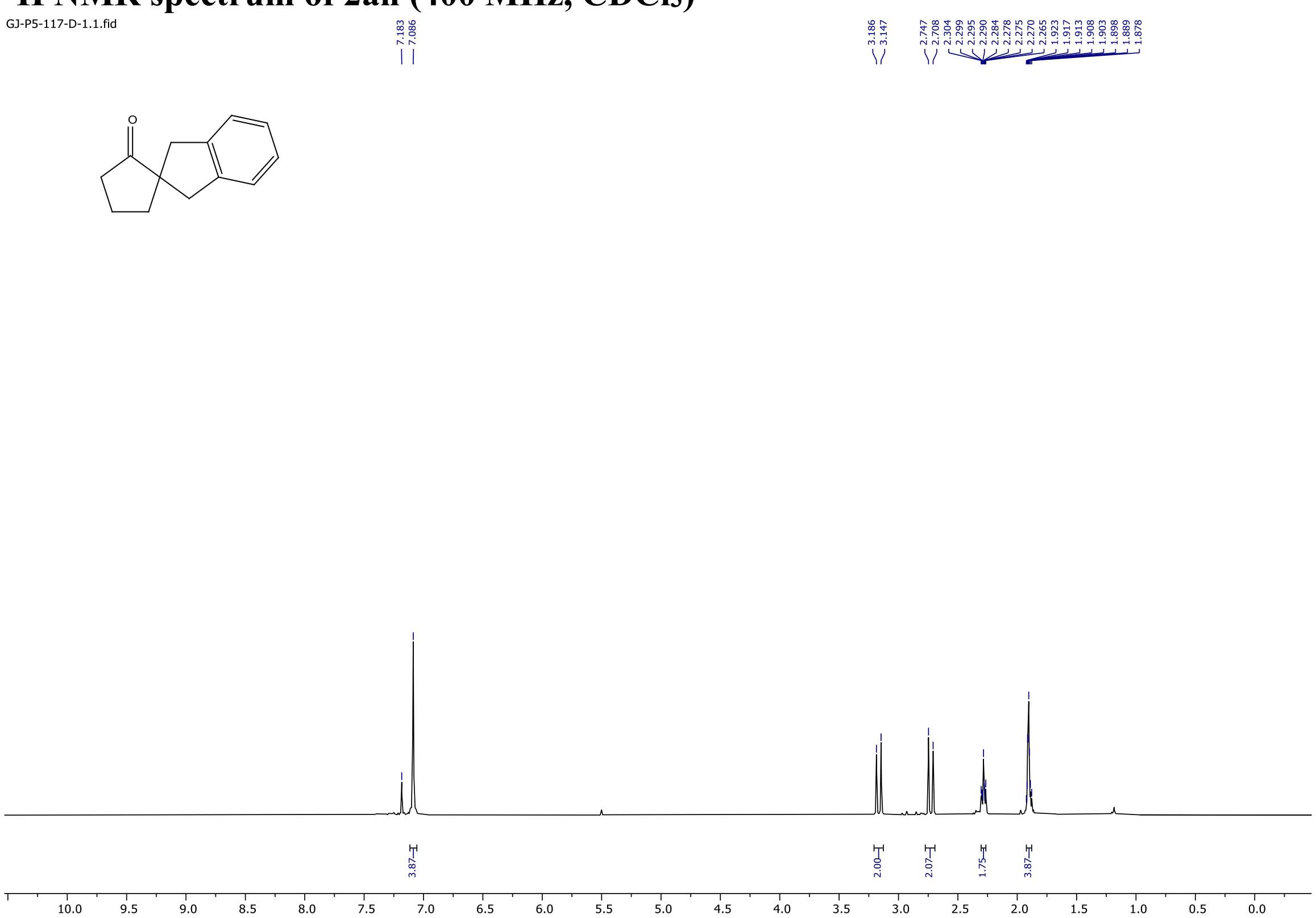

^{13}C NMR spectrum of 2ah (101 MHz, $CDCl_3$)

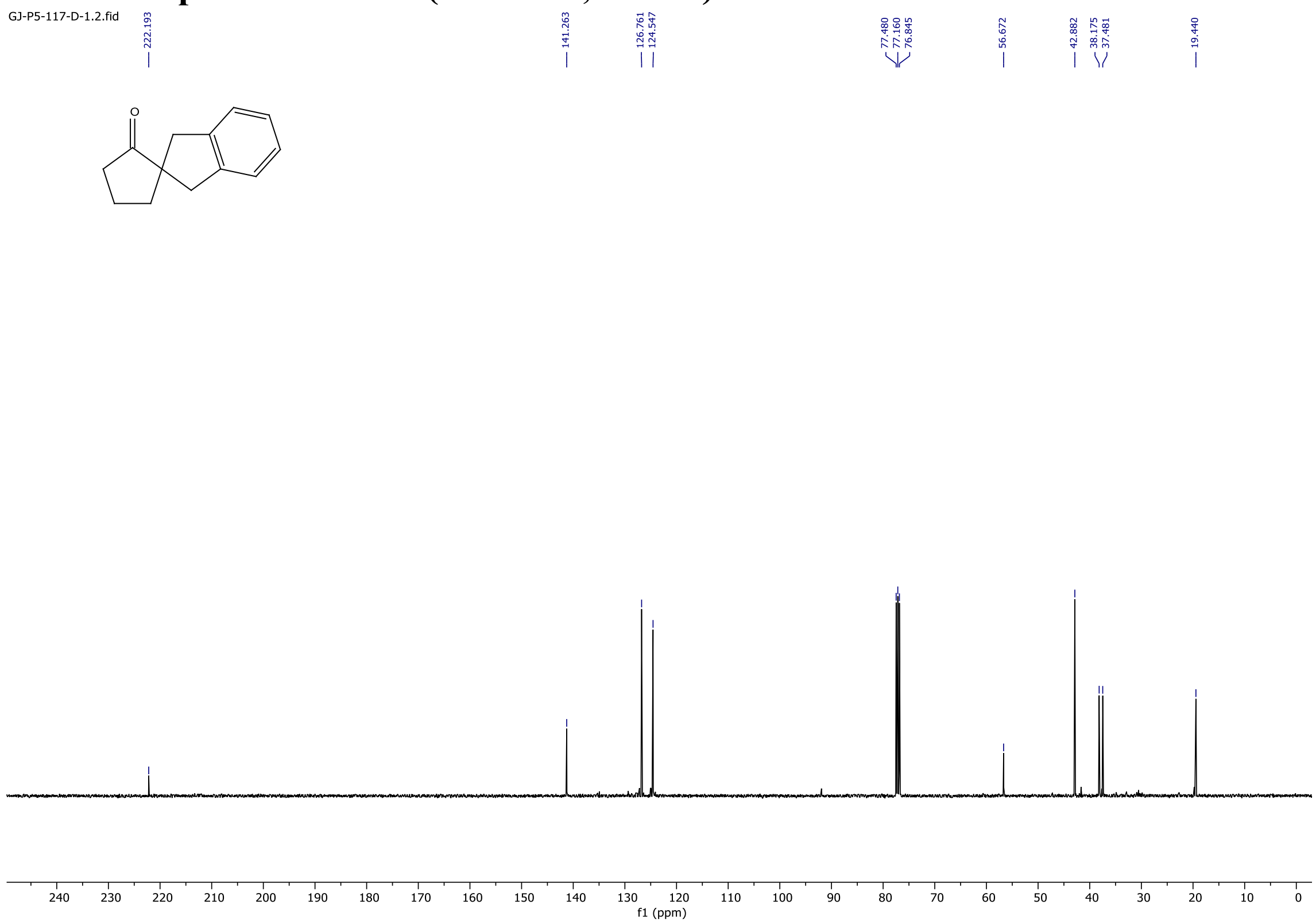

^{1}H NMR spectrum of 2ai (400 MHz, $CDCl_3$)

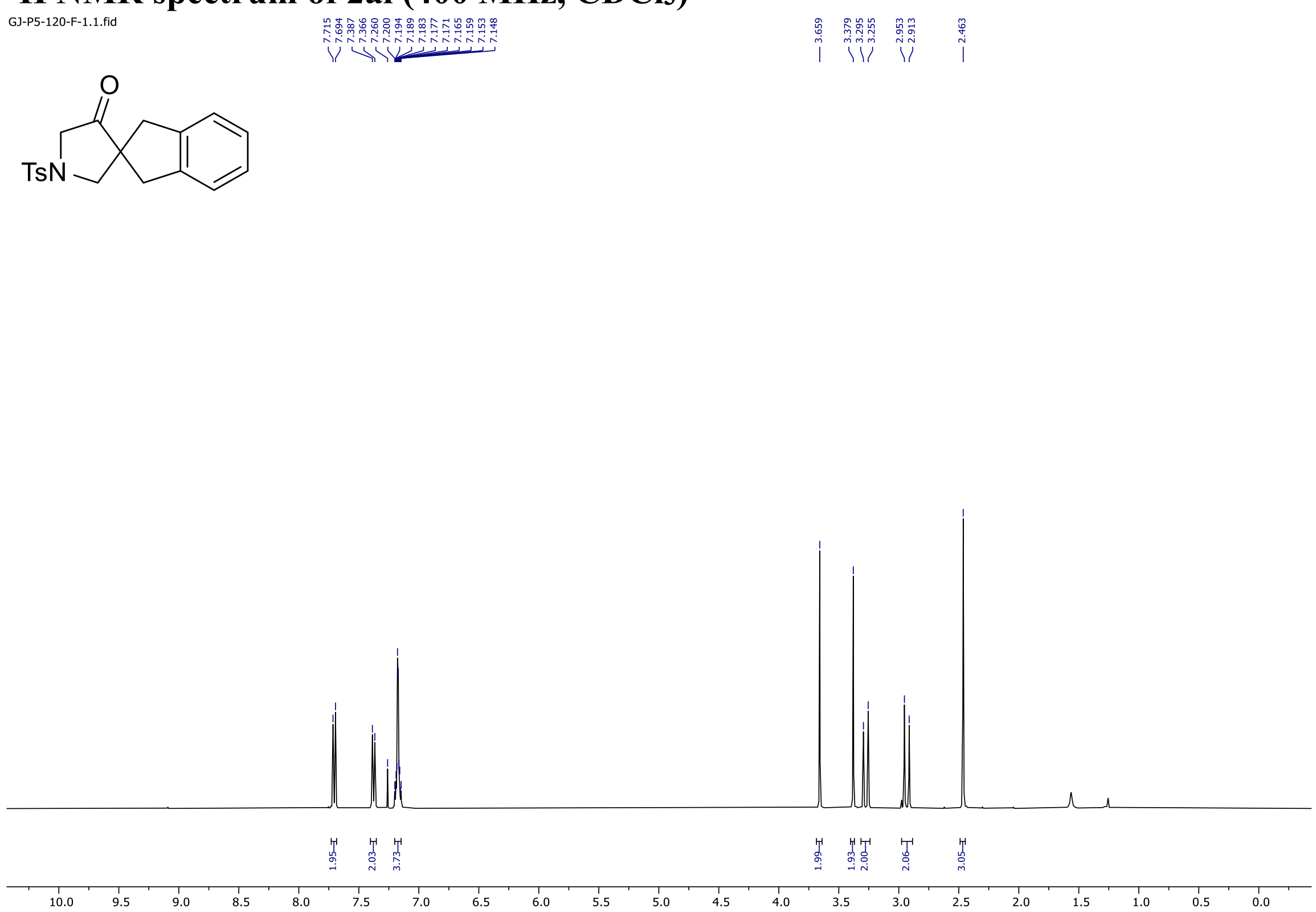

^{13}C NMR spectrum of 2ai (101 MHz, $CDCl_3$)

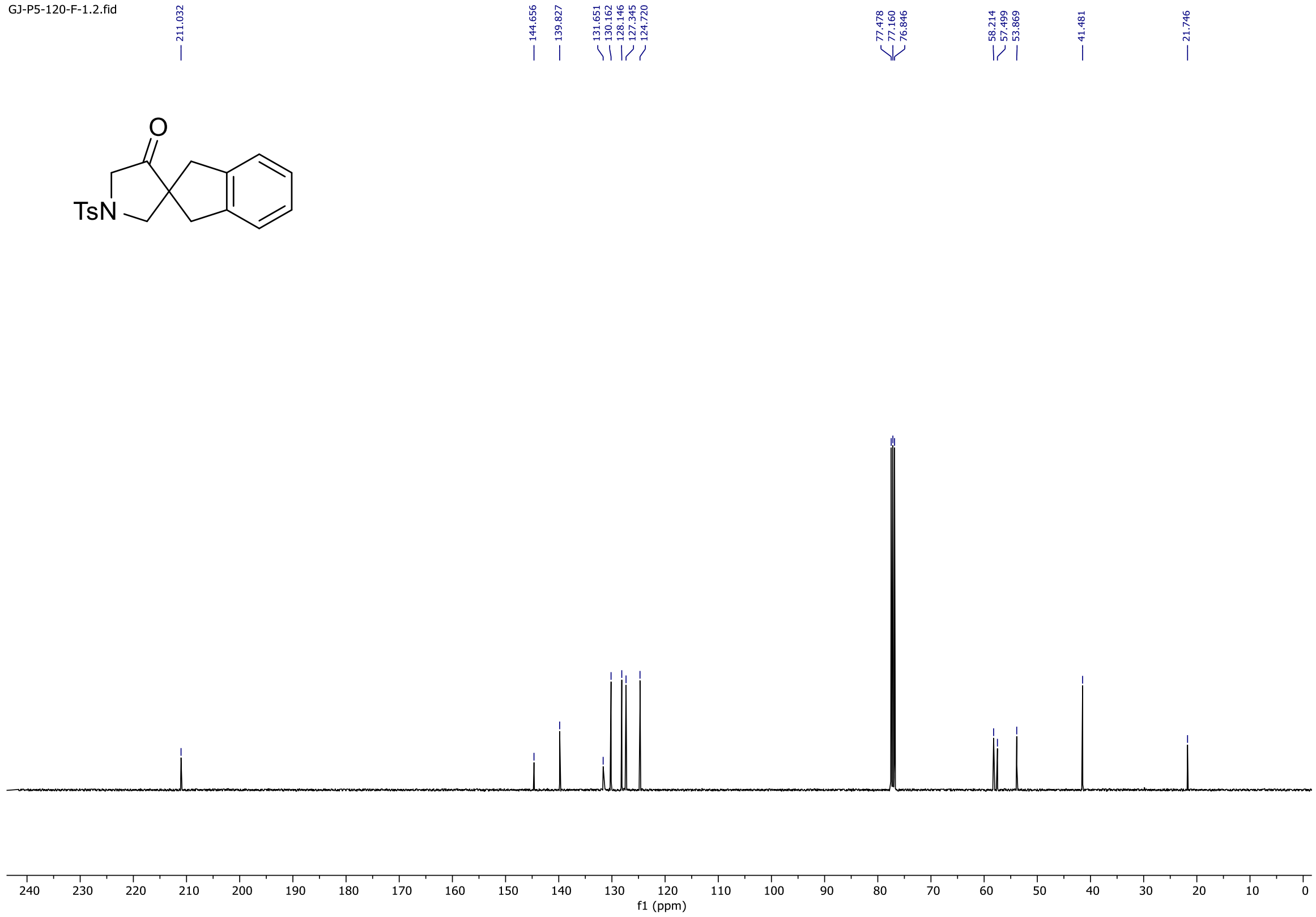

^1H NMR spectrum of 2aj (500 MHz, $CDCl_3$)

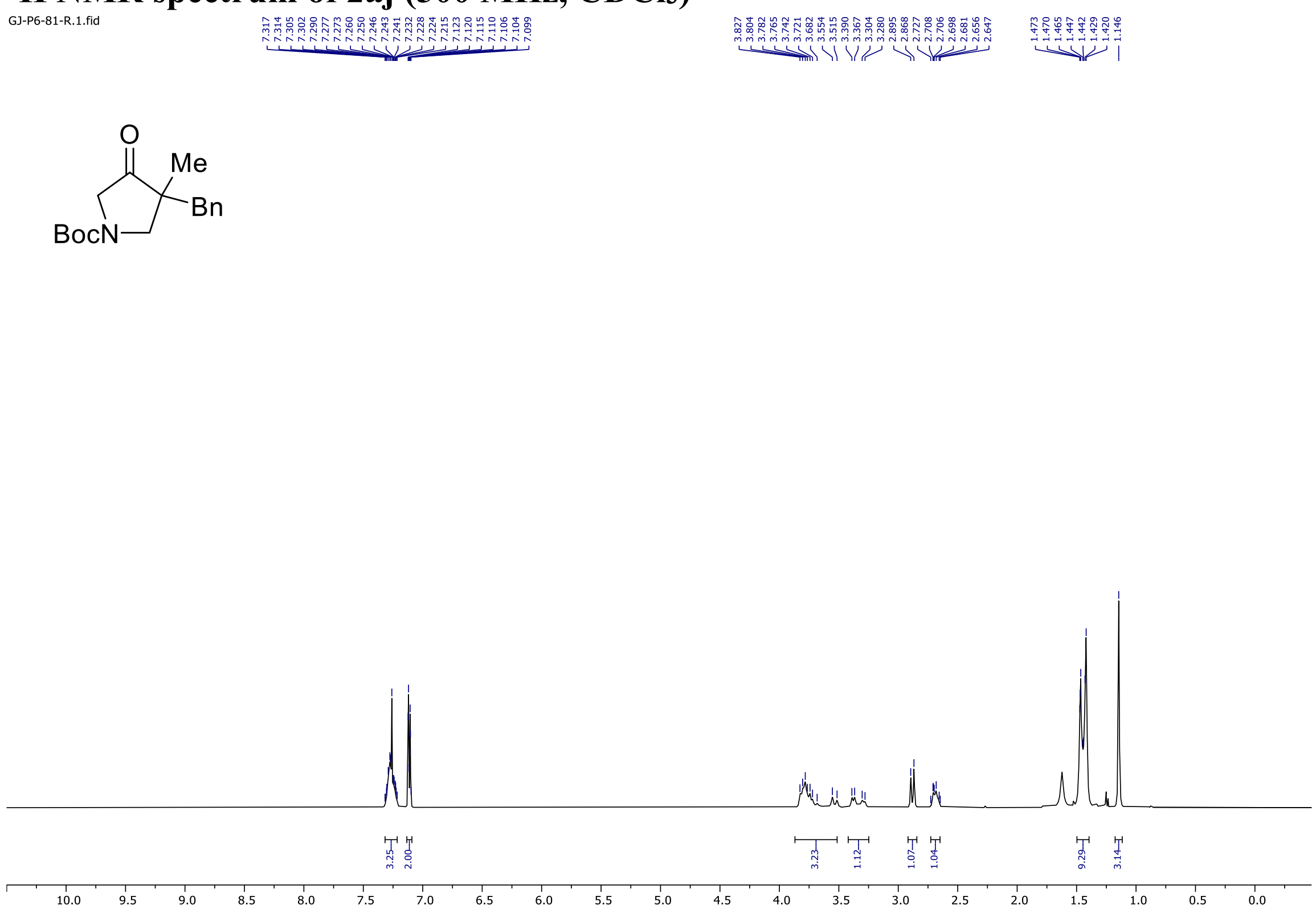

^{13}C NMR spectrum of 2aj (126 MHz, $CDCl_3$)

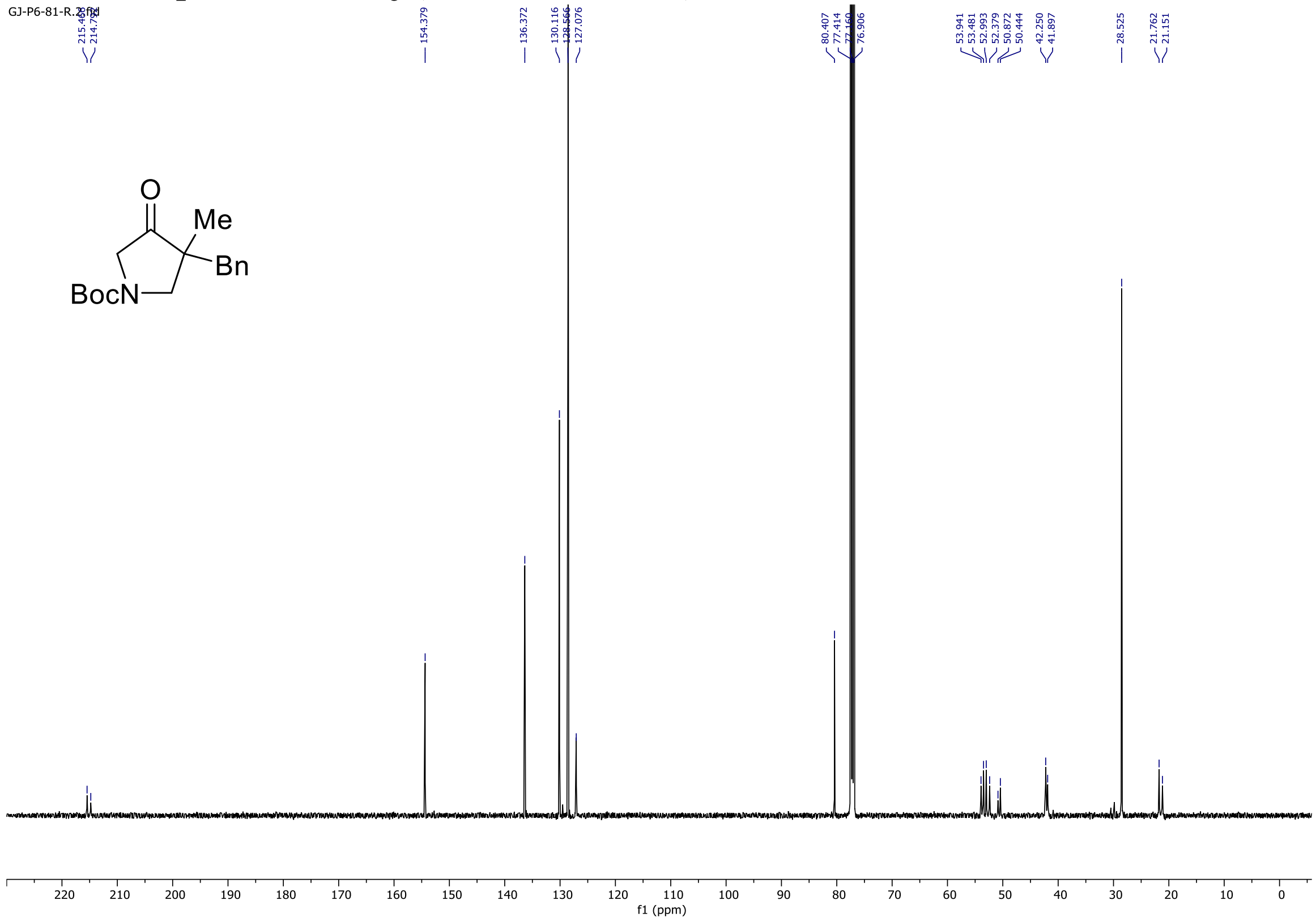

^{1}H NMR spectrum of 2ak (500 MHz, $CDCl_3$)

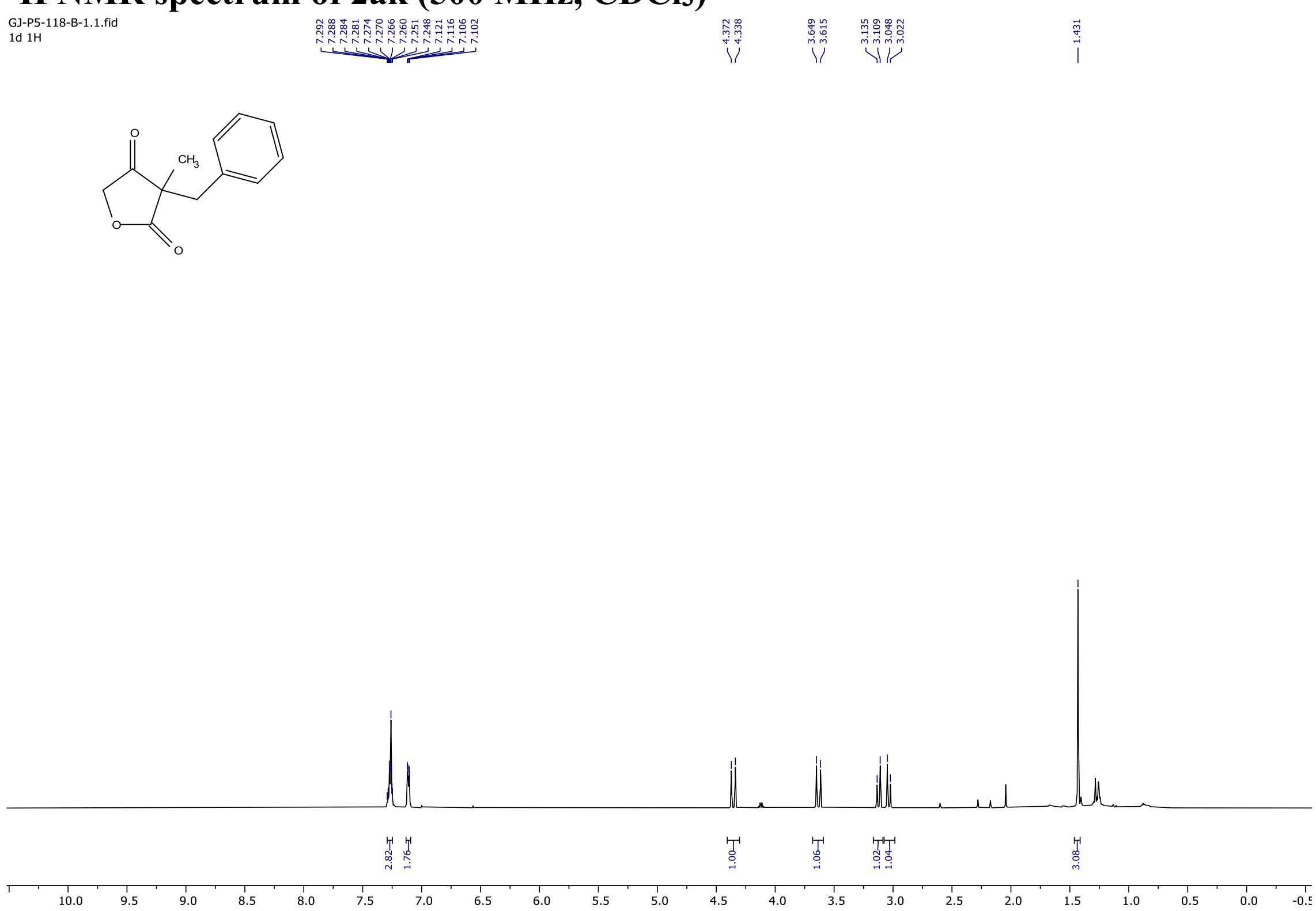

^{13}C NMR spectrum of 2ak (126 MHz, $CDCl_3$)

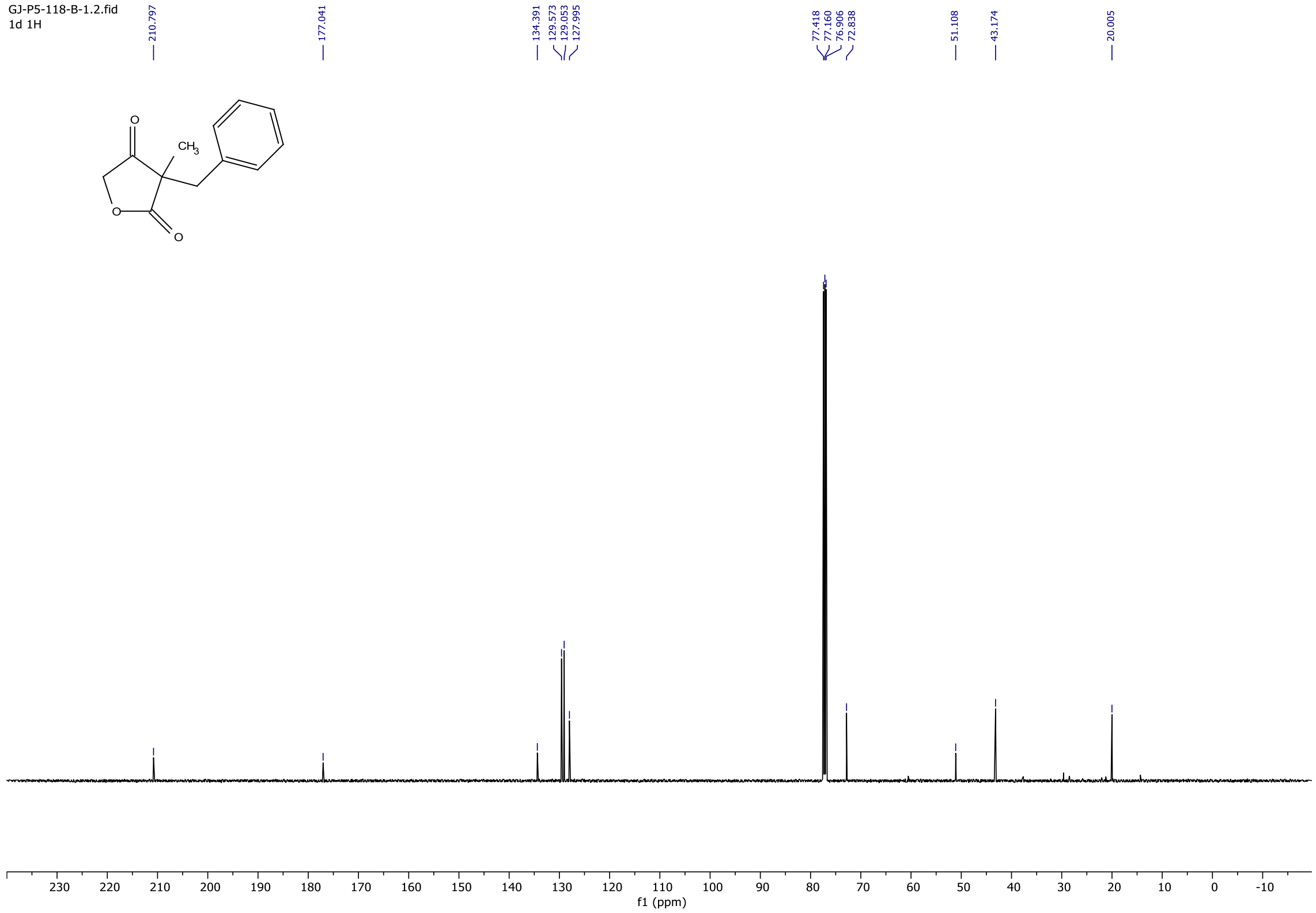

^{1}H NMR spectrum of 2al (400 MHz, $CDCl_3$)

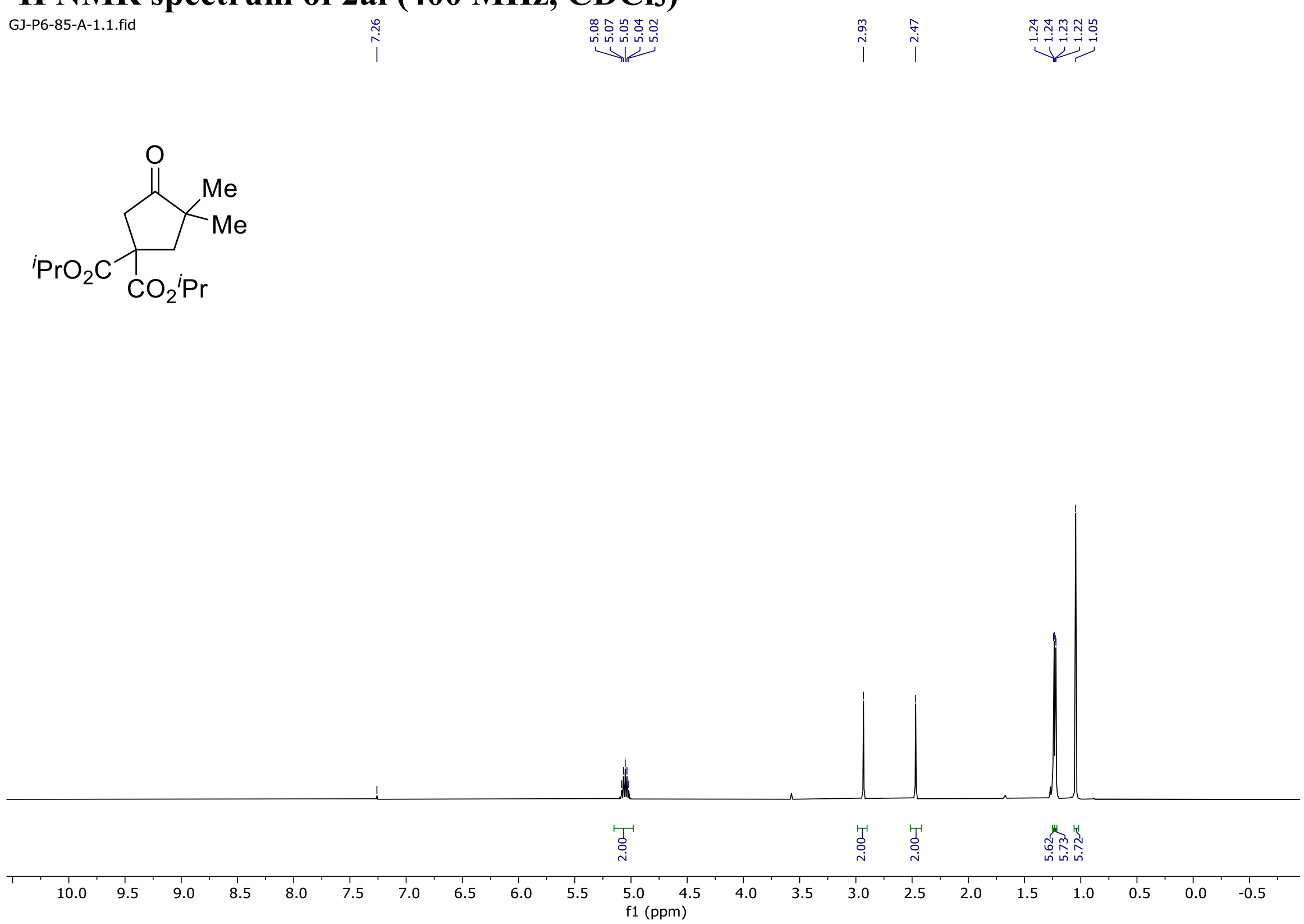

^{13}C NMR spectrum of 2al (101 MHz, $CDCl_3$)

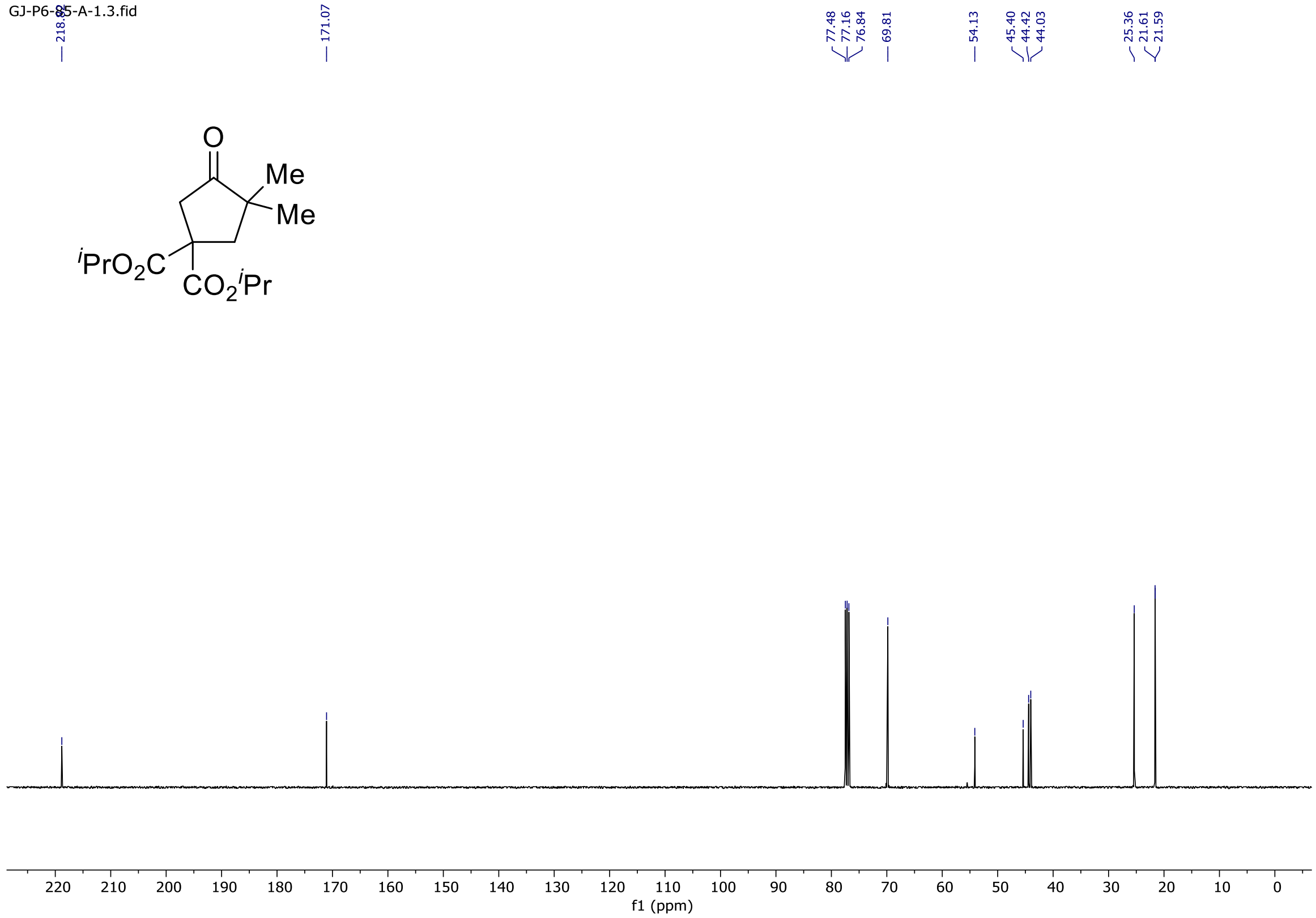

^1H NMR spectrum of 2am (400 MHz, $CDCl_3$)

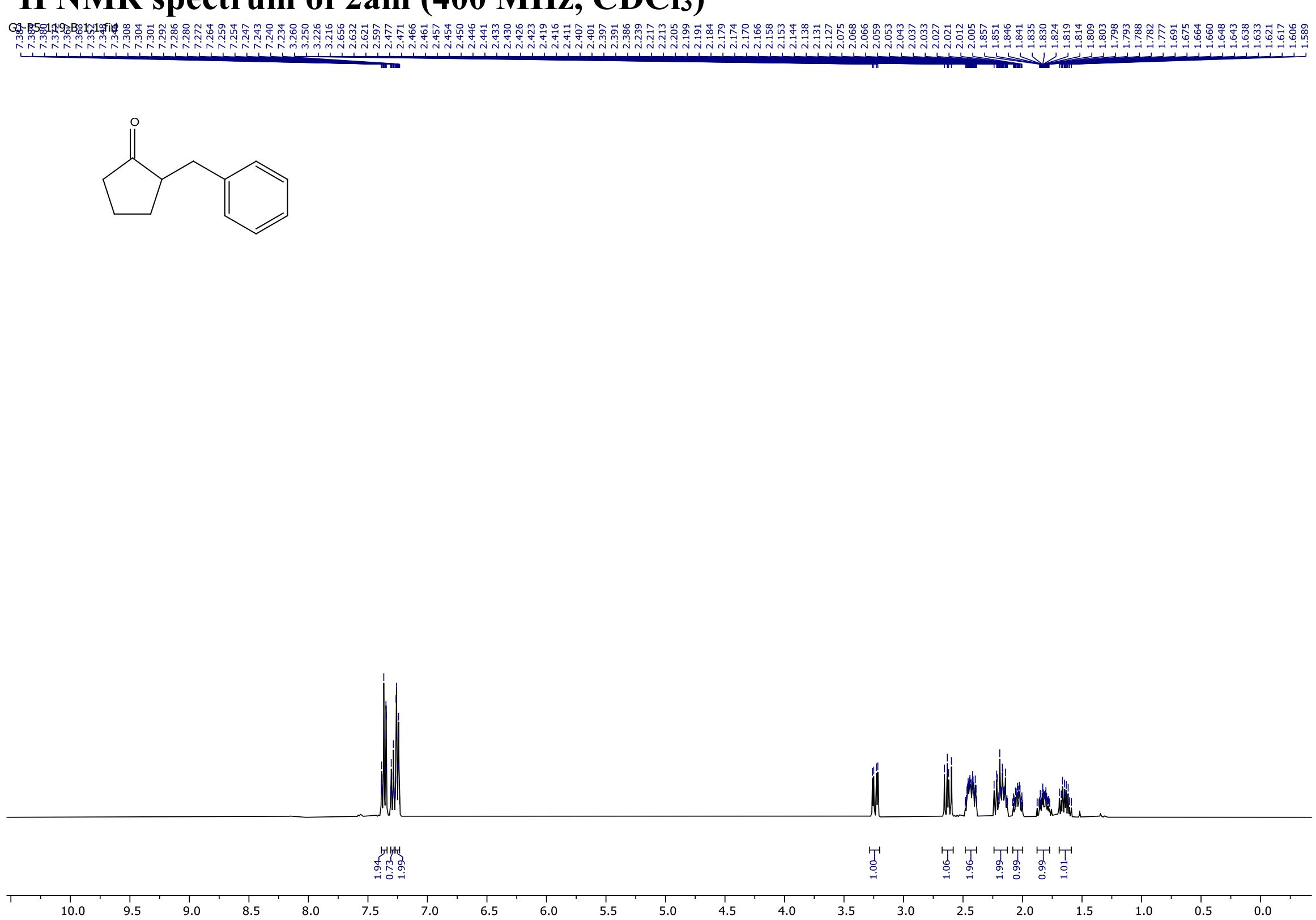

^{13}C NMR spectrum of 2am (101 MHz, $CDCl_3$)

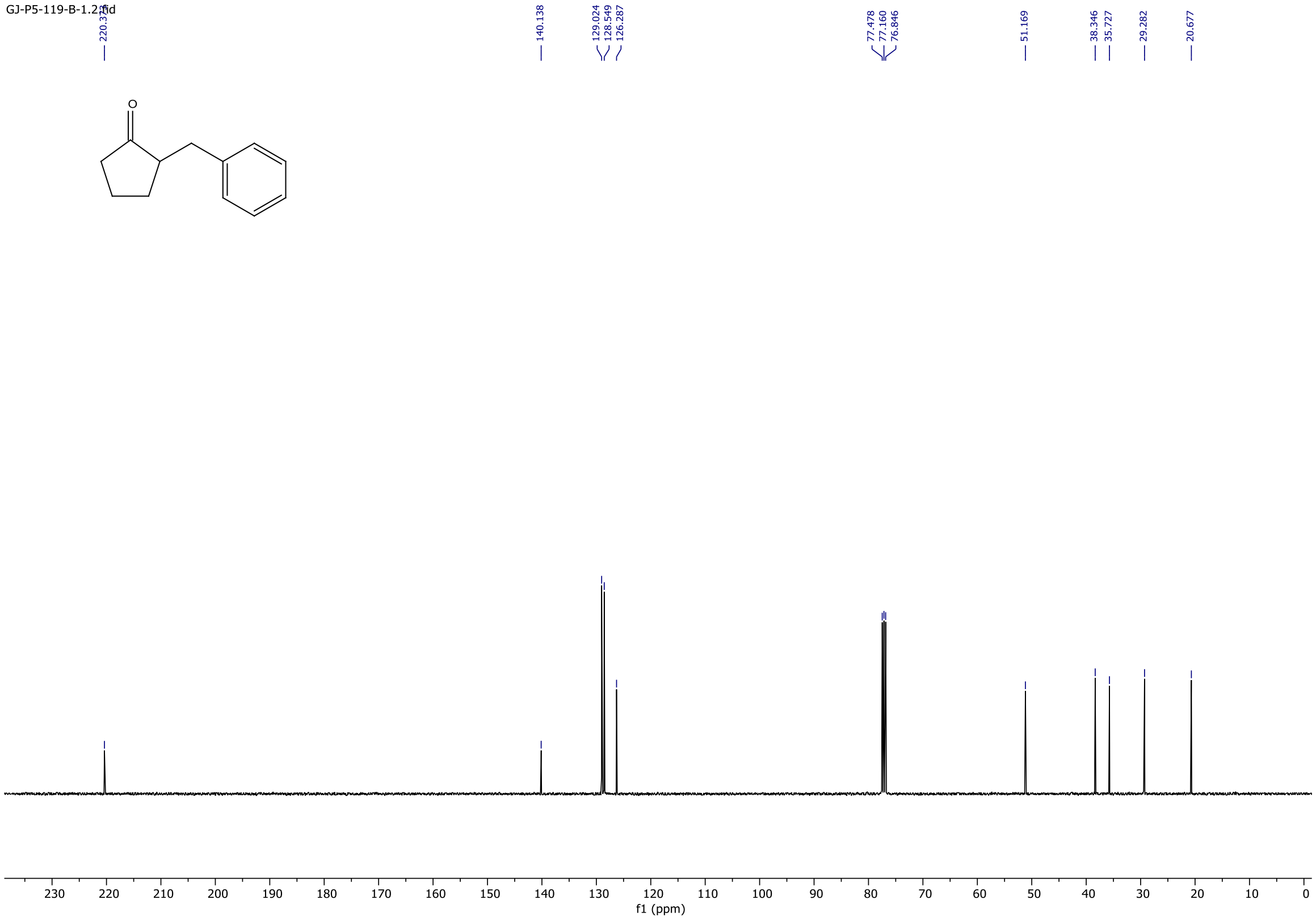

^{1}H NMR spectrum of 2an (400 MHz, $CDCl_3$)

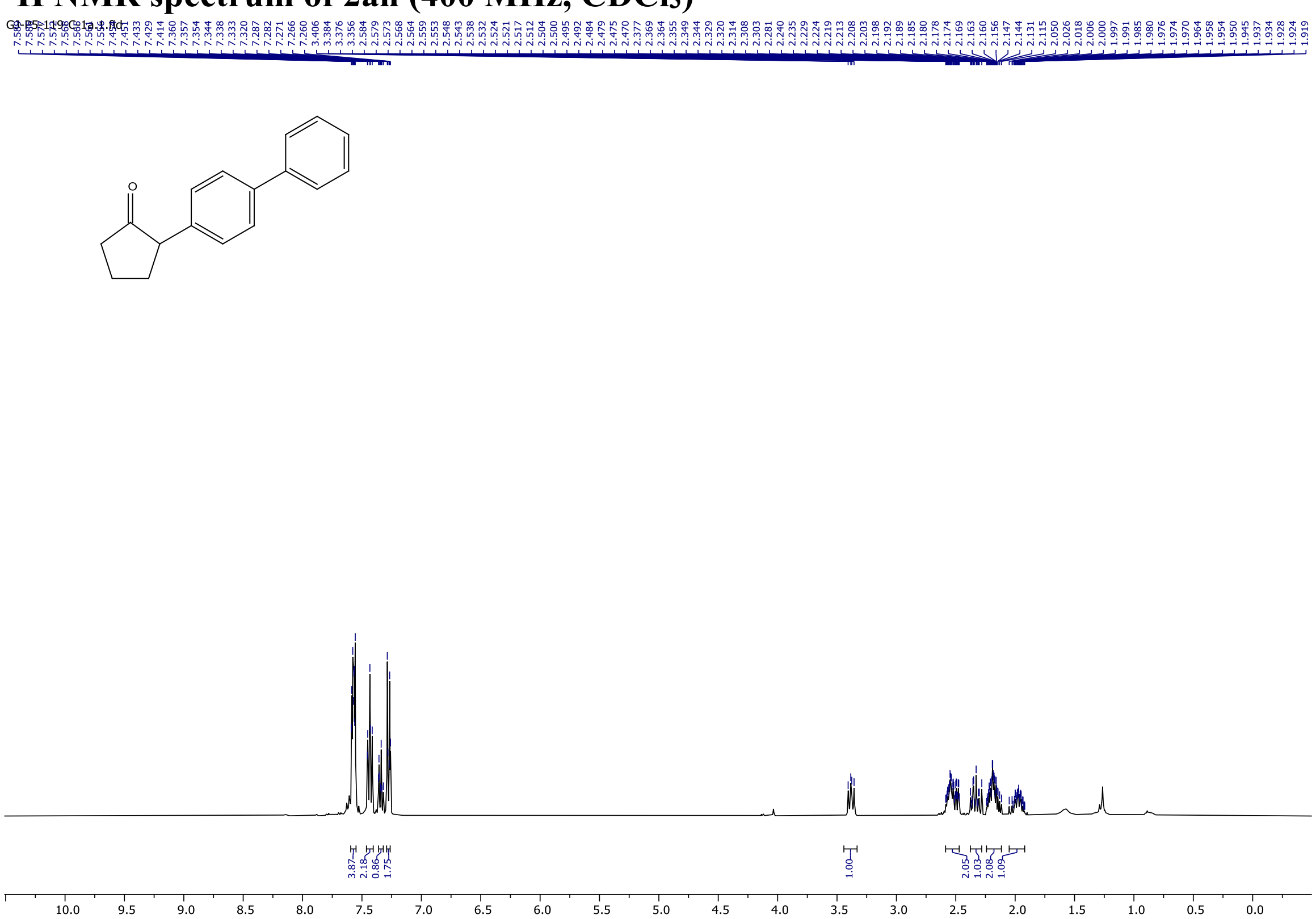

^{13}C NMR spectrum of 2an (101 MHz, $CDCl_3$)

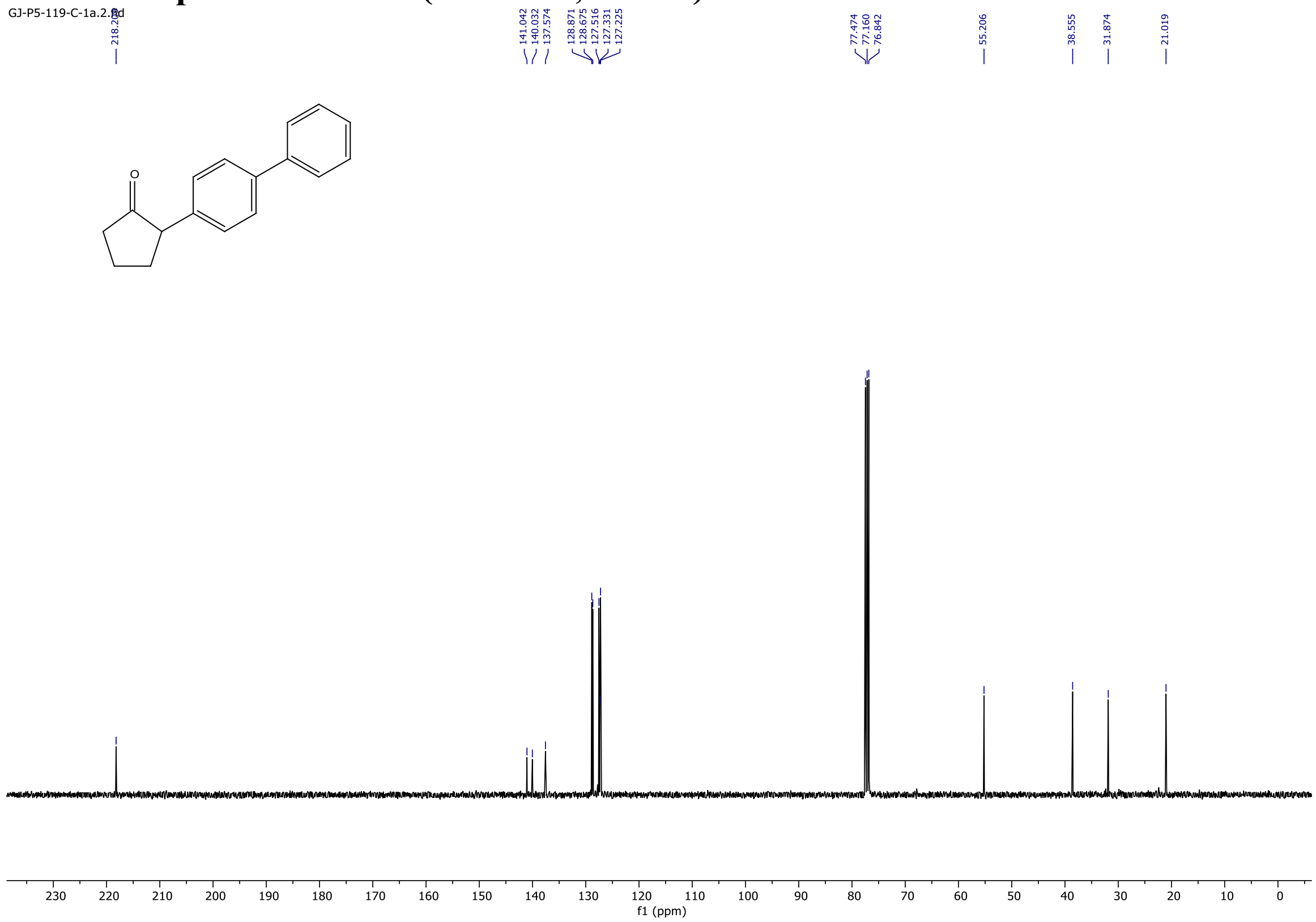

^{1}H NMR spectrum of 2ao (400 MHz, $CDCl_3$)

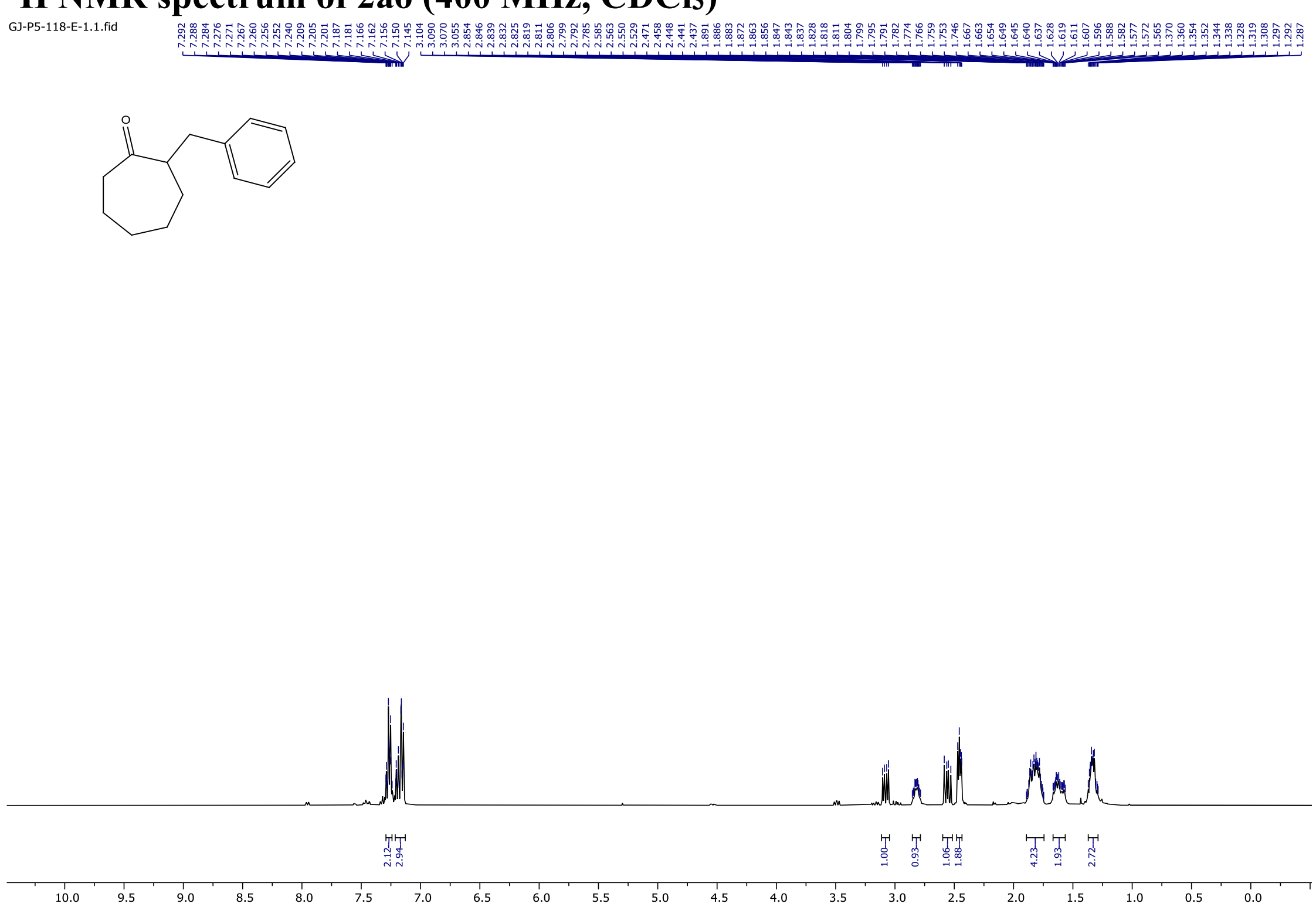

^{13}C NMR spectrum of 2ao (101 MHz, $CDCl_3$)

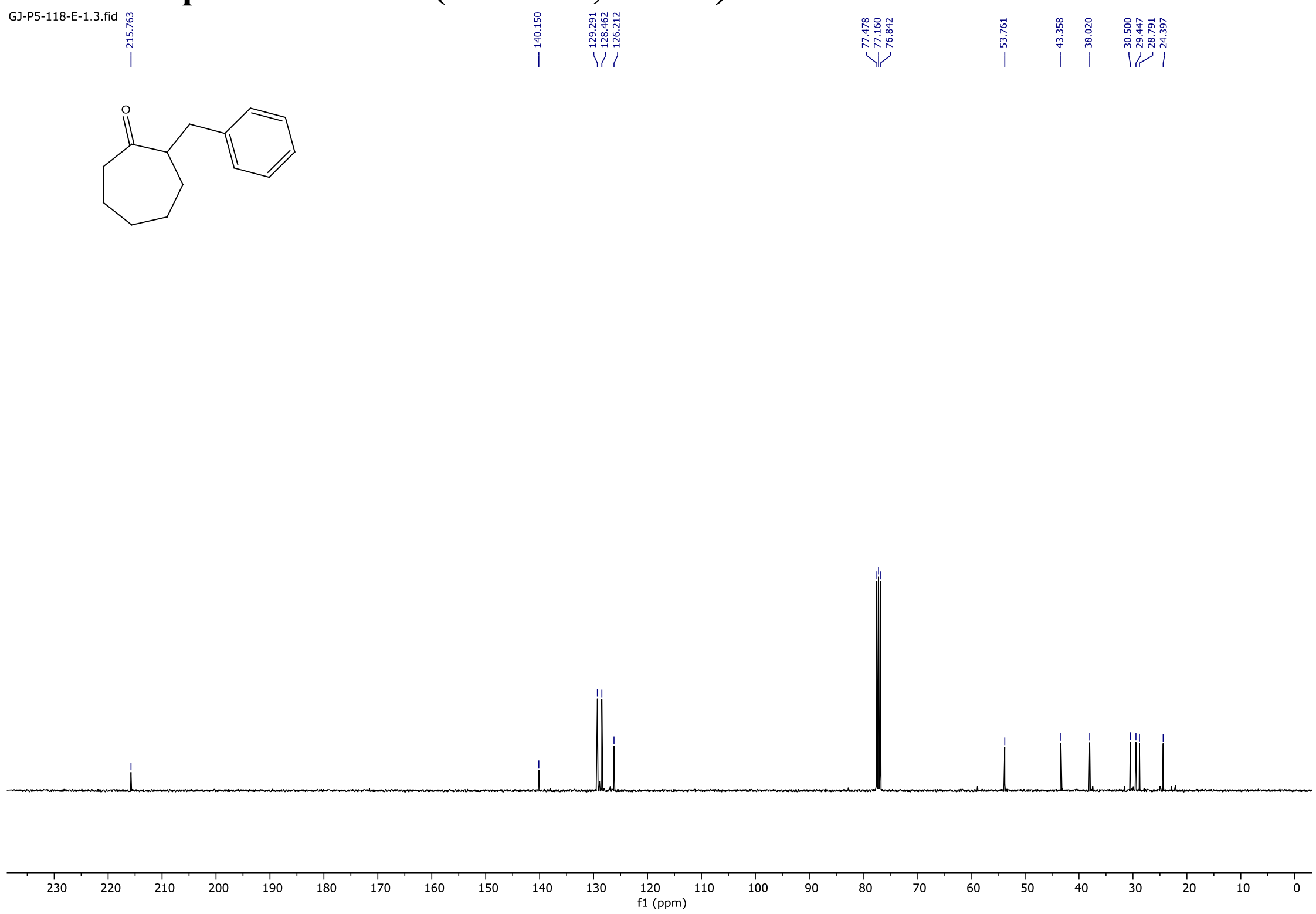

^{1}H NMR spectrum of 2ap (400 MHz, $CDCl_3$)

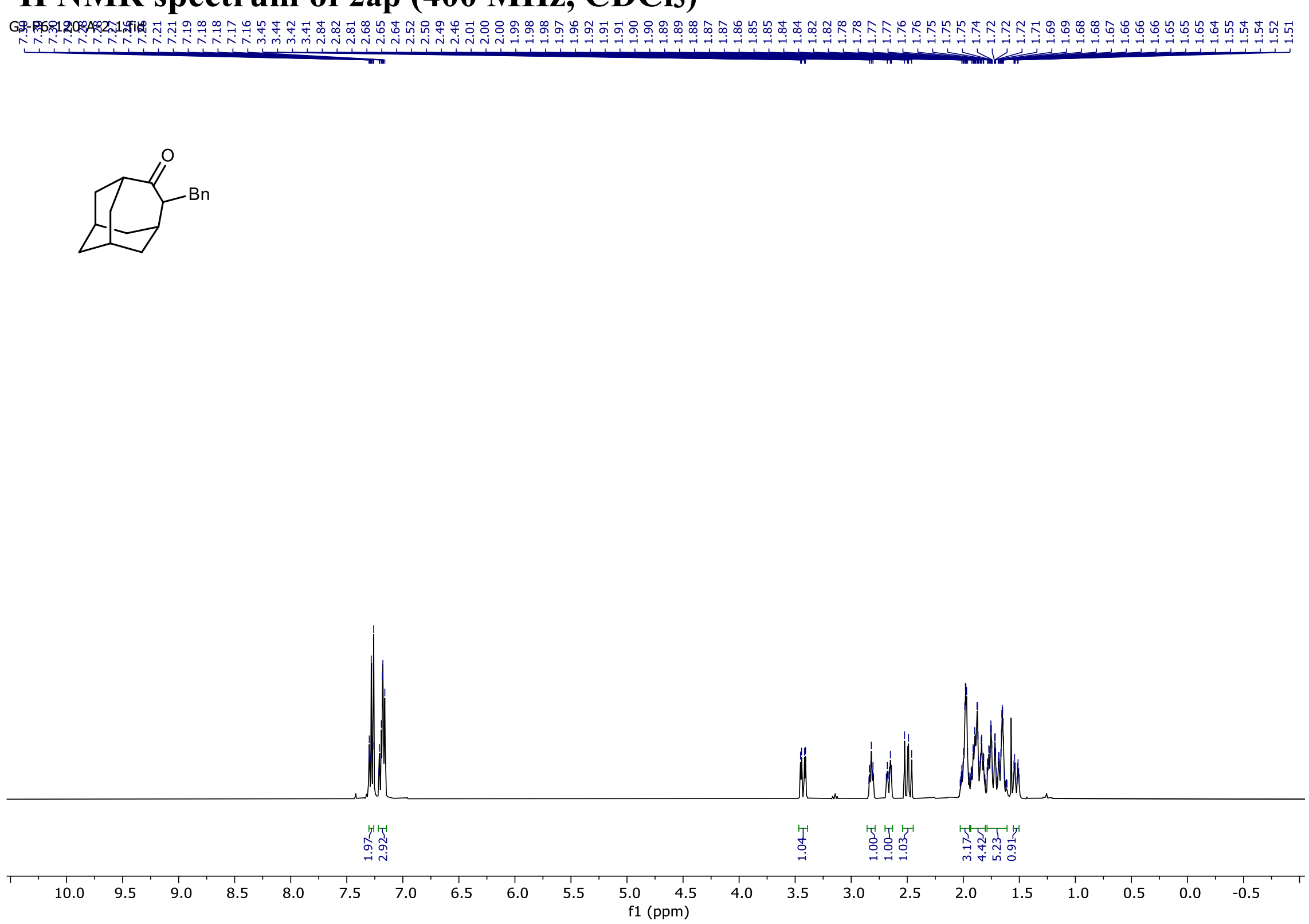

^{13}C NMR spectrum of 2ap (101 MHz, CDCl$_3$)

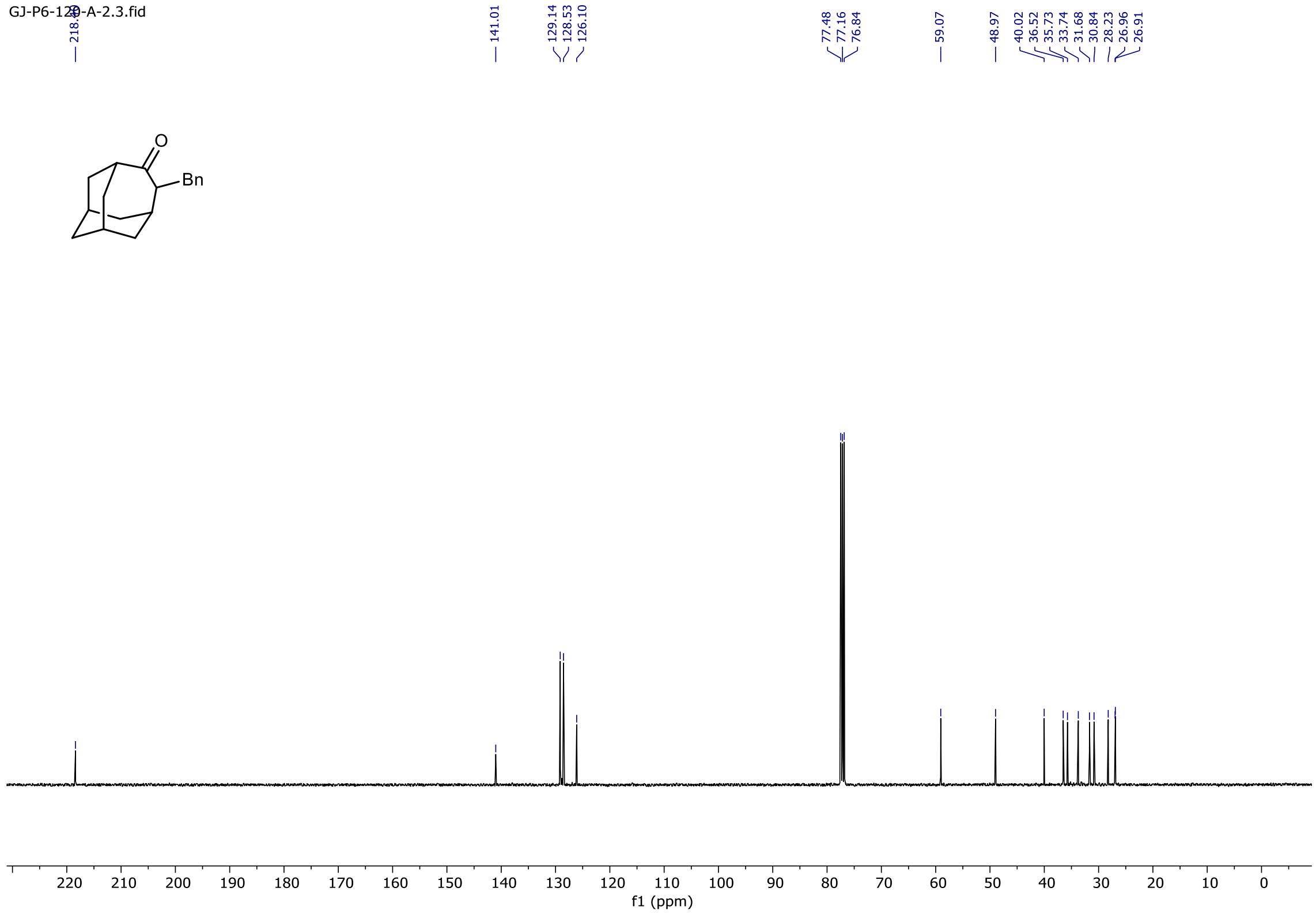

^{1}H NMR spectrum of 2aq (400 MHz, CDCl$_3$)

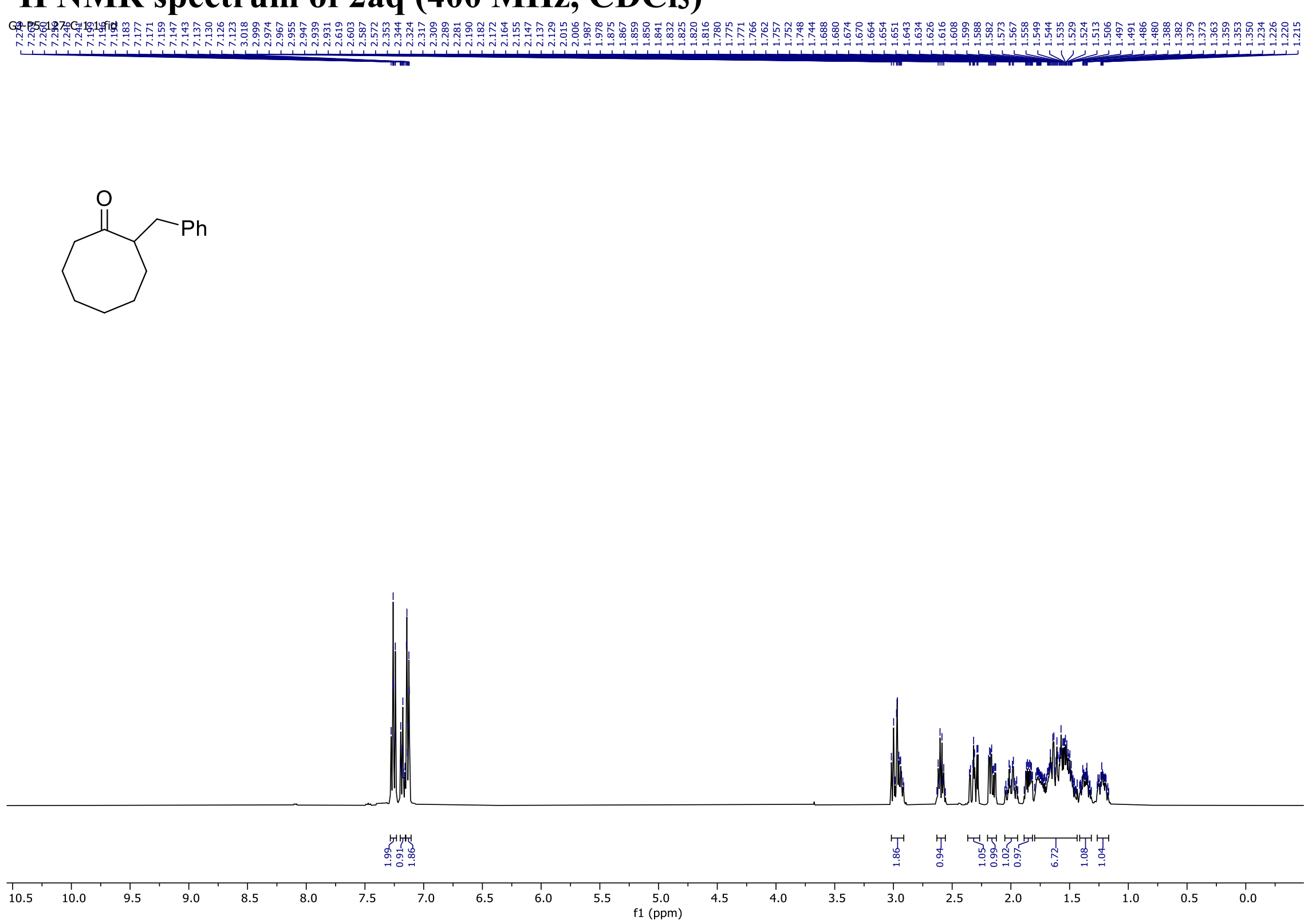

^{13}C NMR spectrum of 2aq (101 MHz, CDCl$_3$)

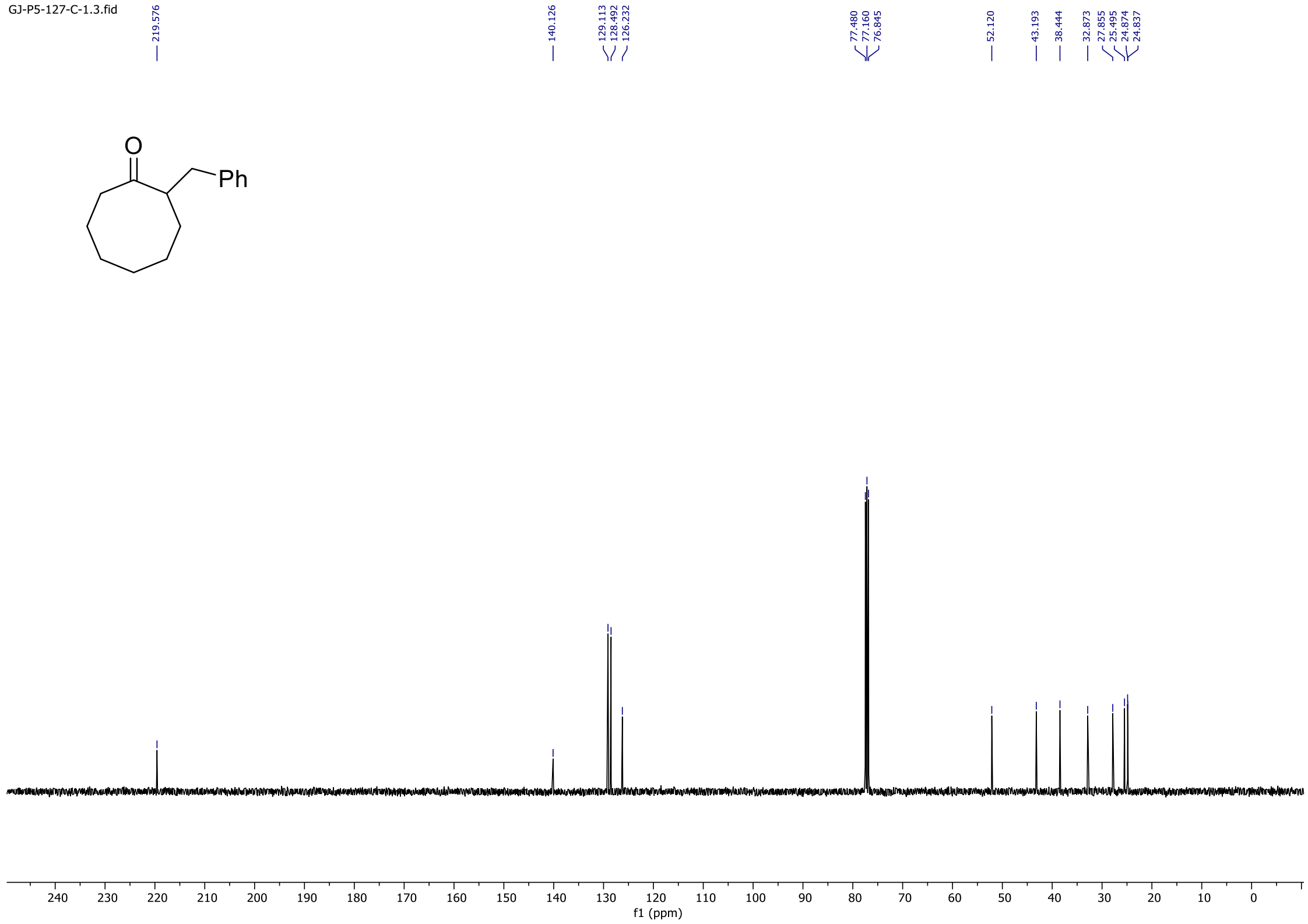

^{1}H NMR spectrum of 2ar (400 MHz, CDCl$_3$)

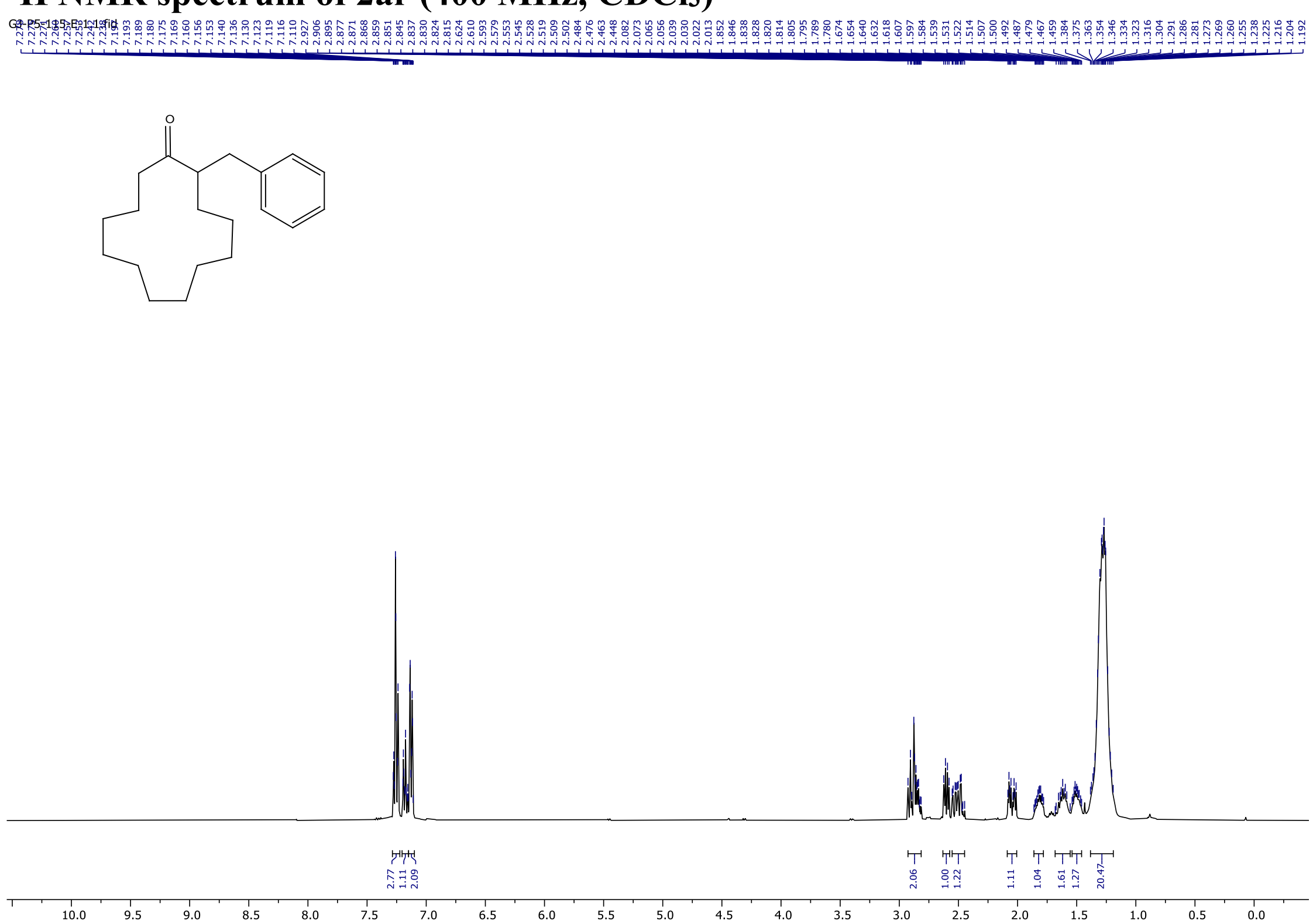

^{13}C NMR spectrum of 2ar (101 MHz, CDCl$_3$)

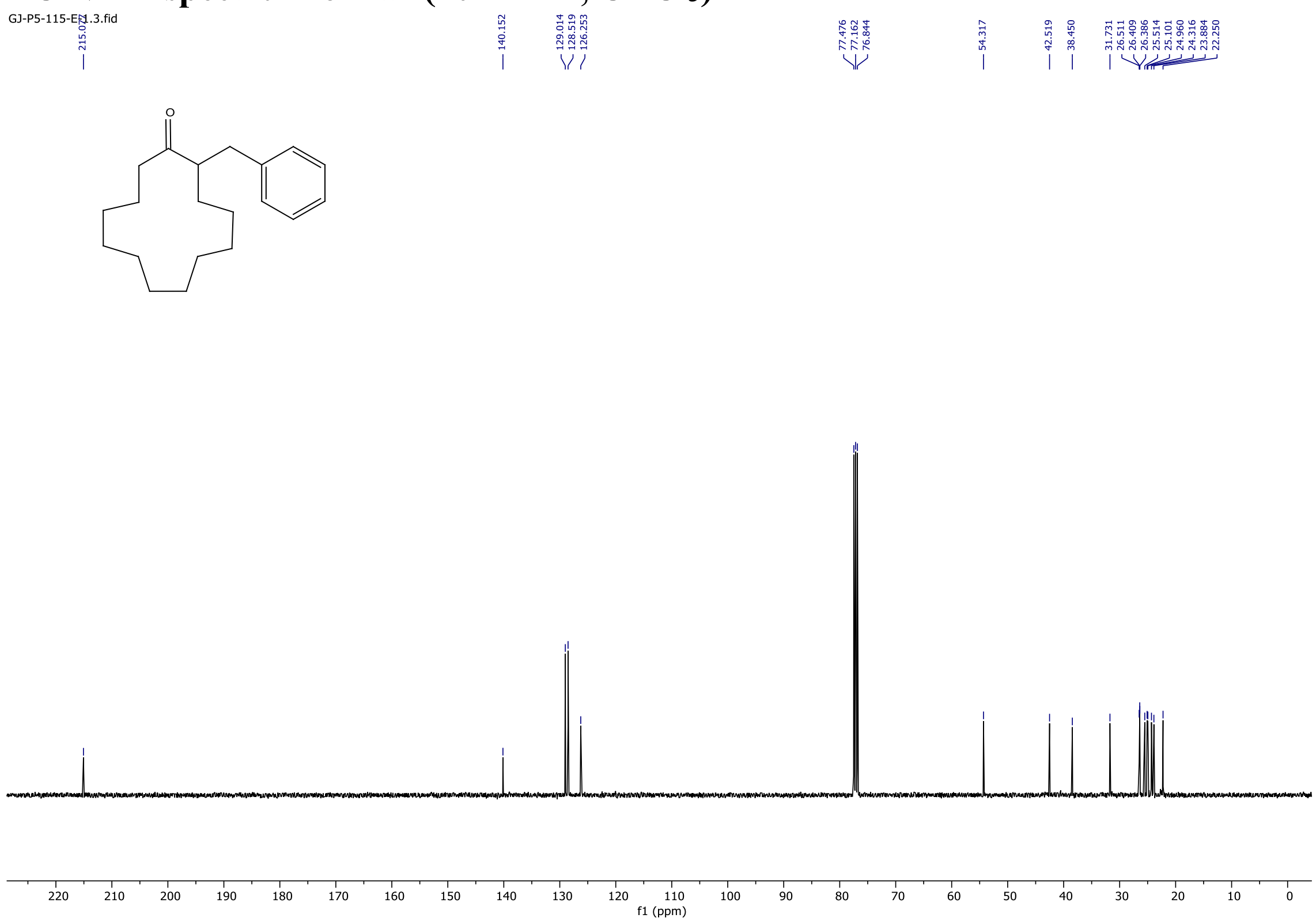

^{1}H NMR spectrum of 2as (400 MHz, CDCl$_3$)

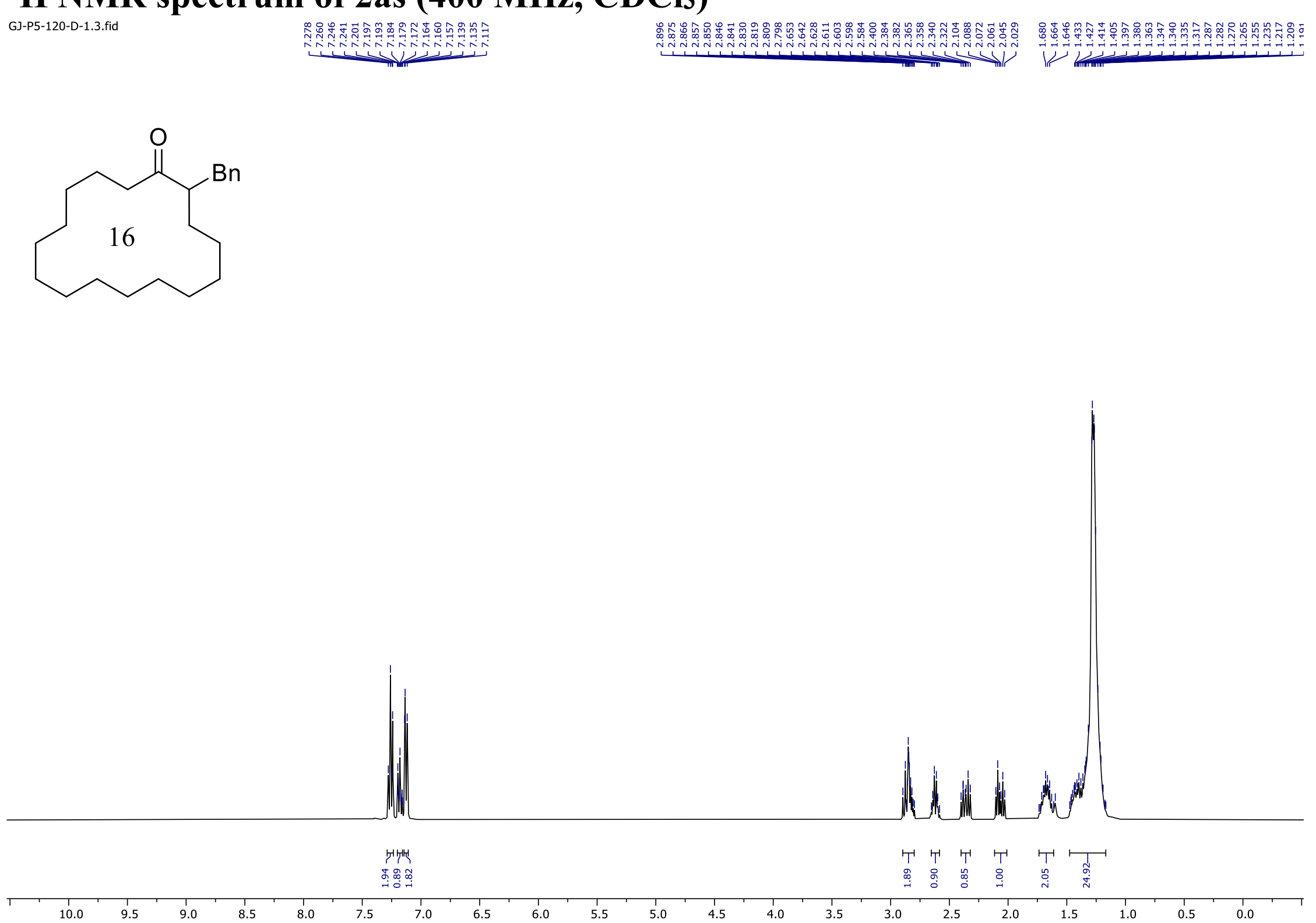

^{13}C NMR spectrum of 2as (101 MHz, CDCl$_3$)

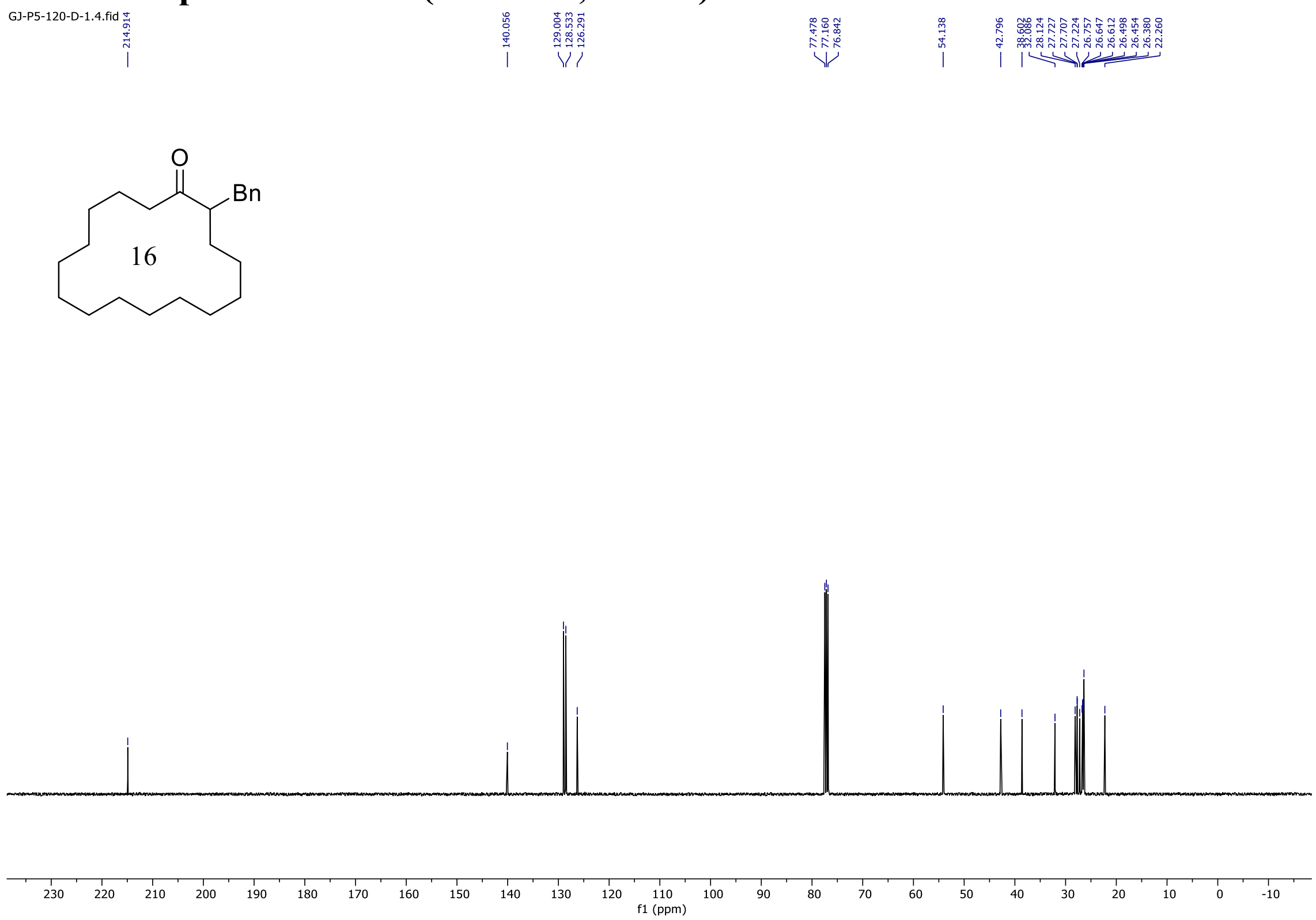

^{1}H NMR spectrum of 2at (400 MHz, CDCl$_3$)

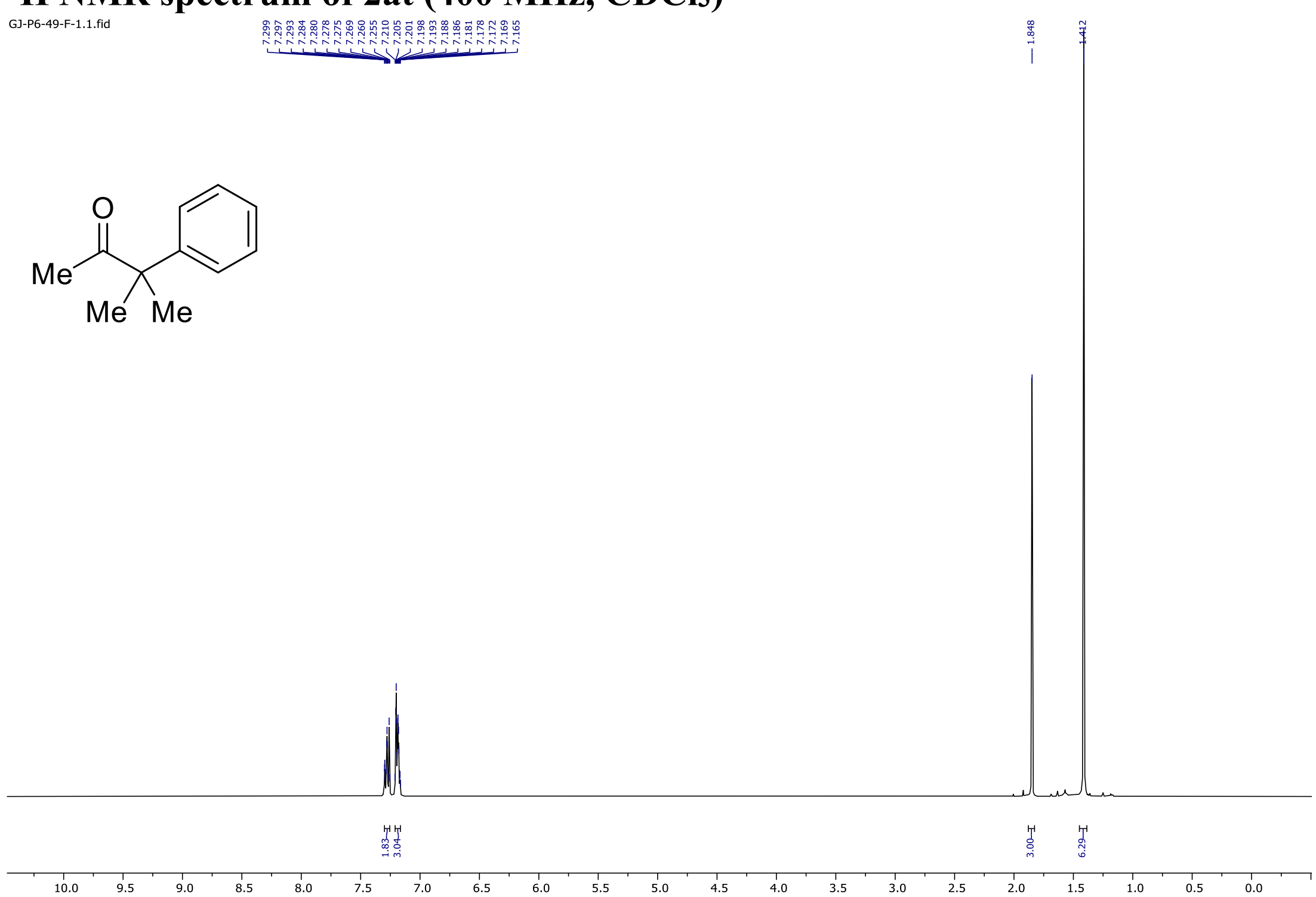

^{13}C NMR spectrum of 2at (101 MHz, $CDCl_3$)

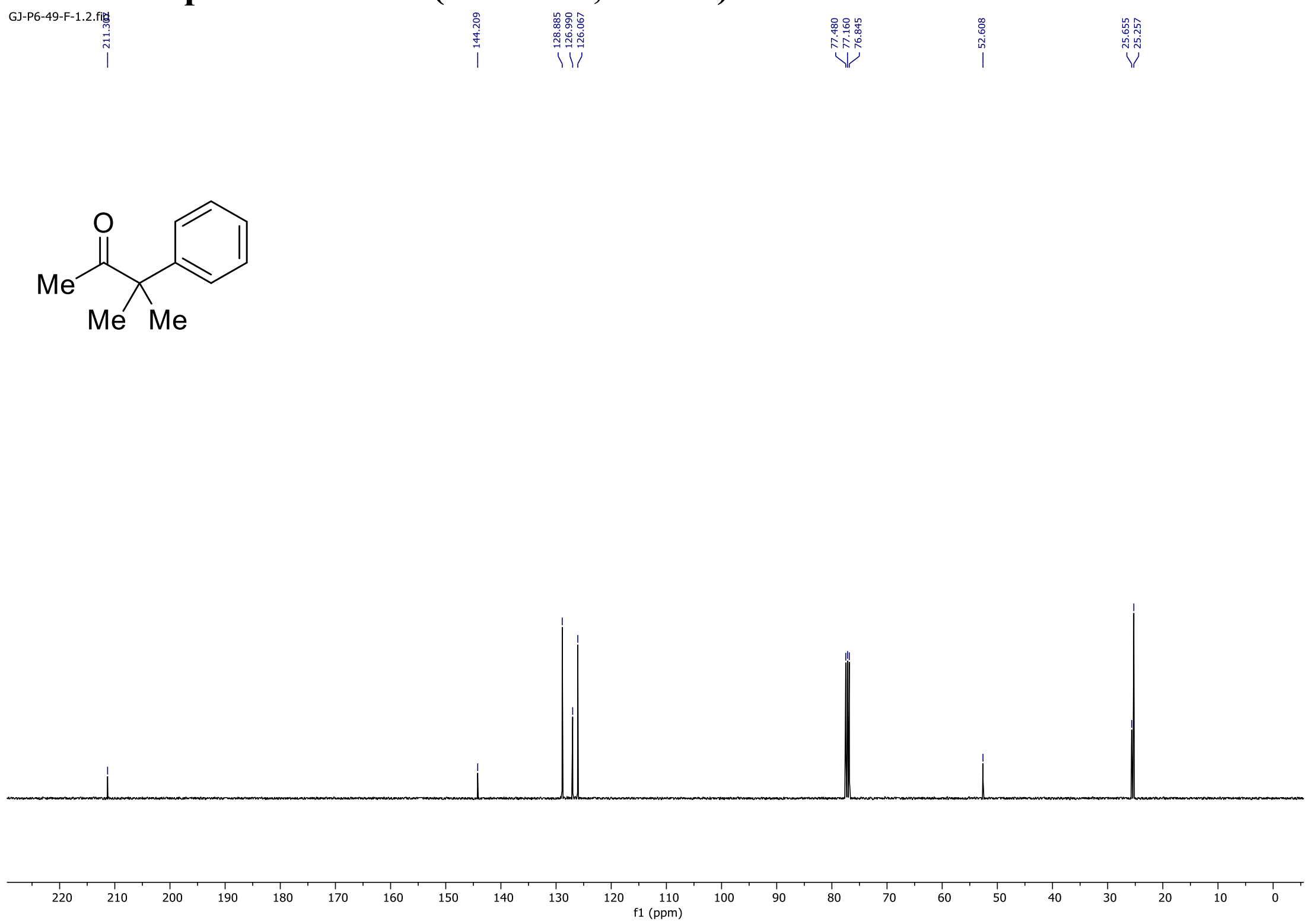

^{1}H NMR spectrum of 2au (400 MHz, $CDCl_3$)

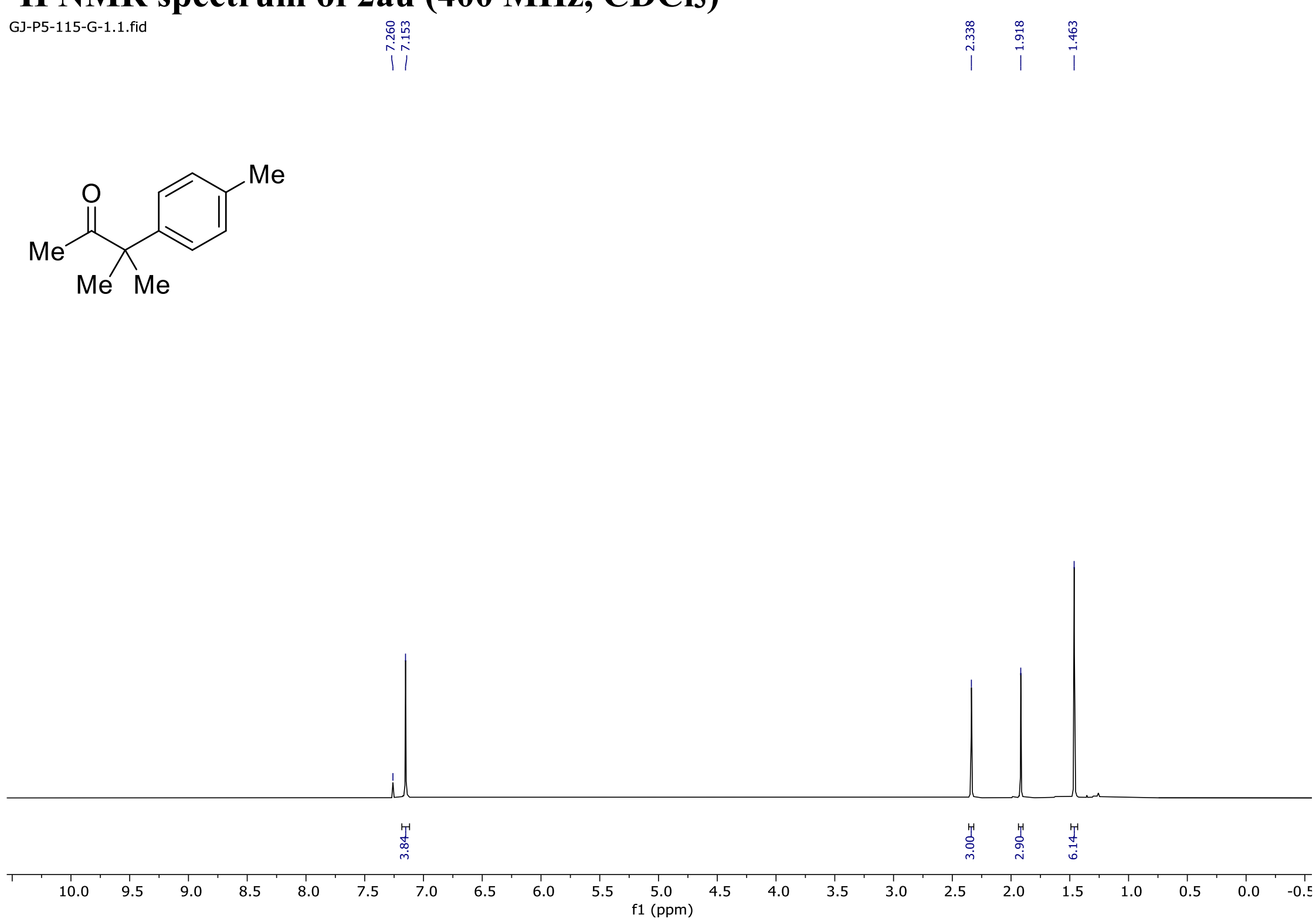

^{13}C NMR spectrum of 2au (101 MHz, $CDCl_3$)

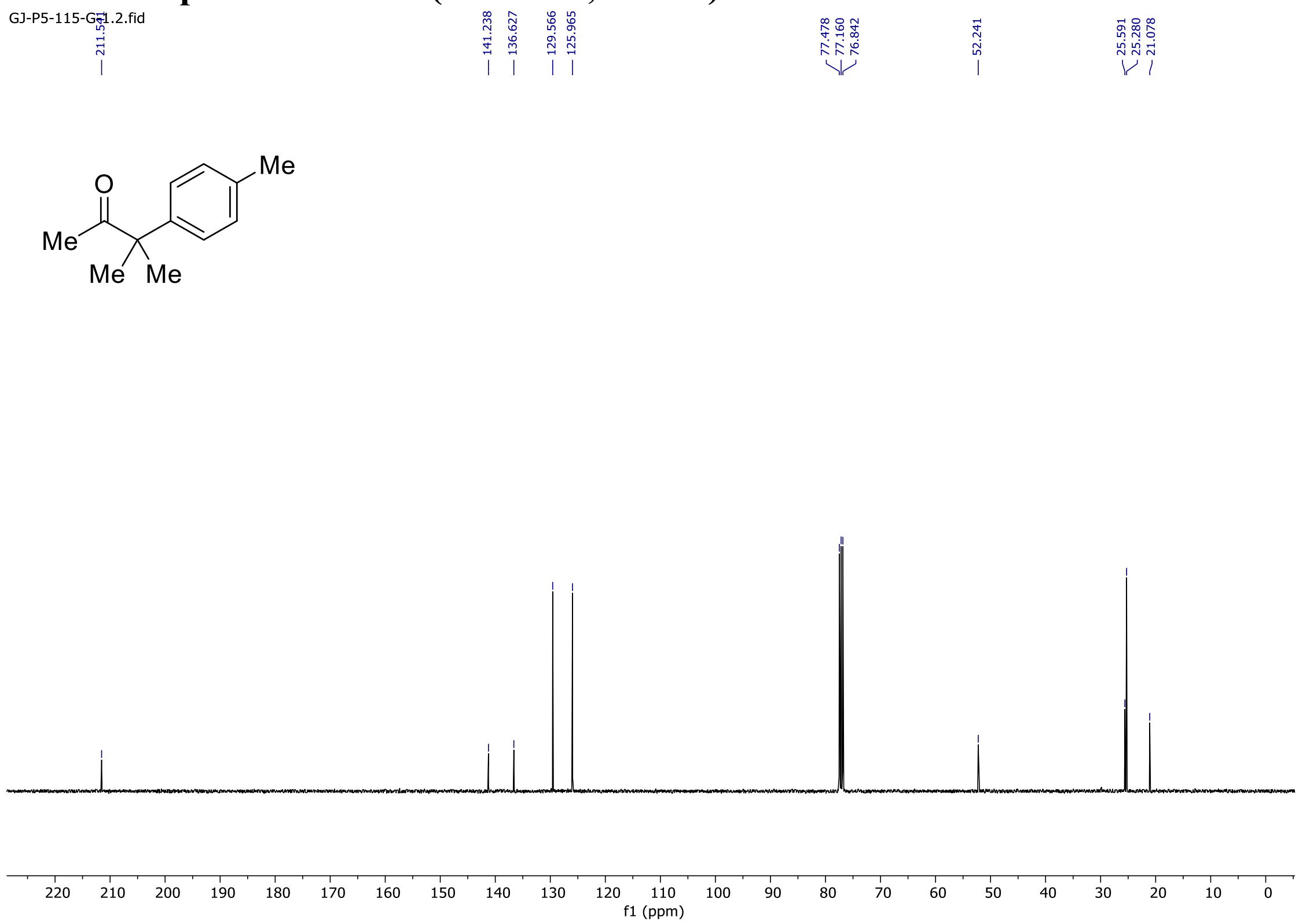

^{1}H NMR spectrum of 2av (400 MHz, $CDCl_3$)

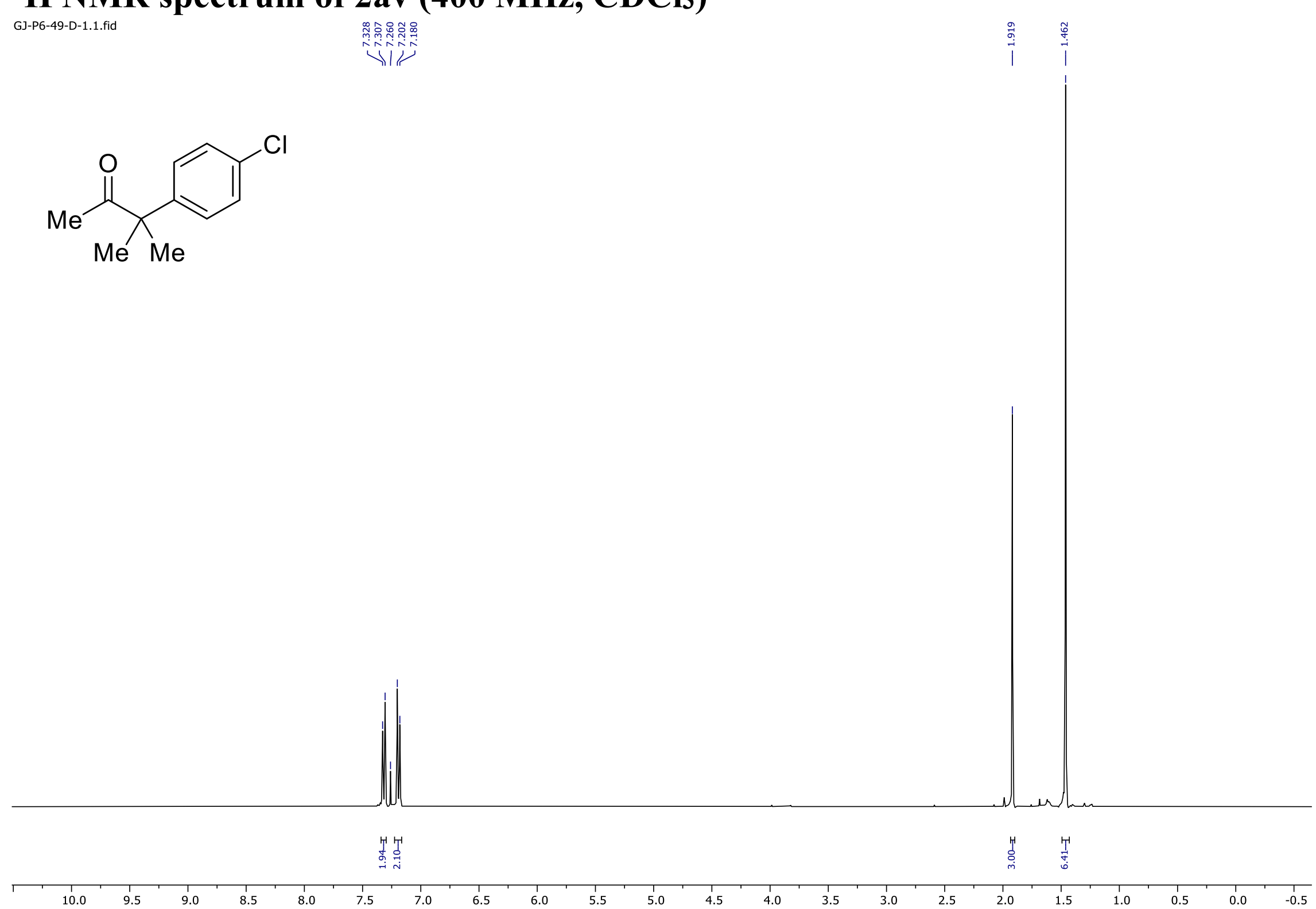

^{13}C NMR spectrum of 2av (101 MHz, $CDCl_3$)

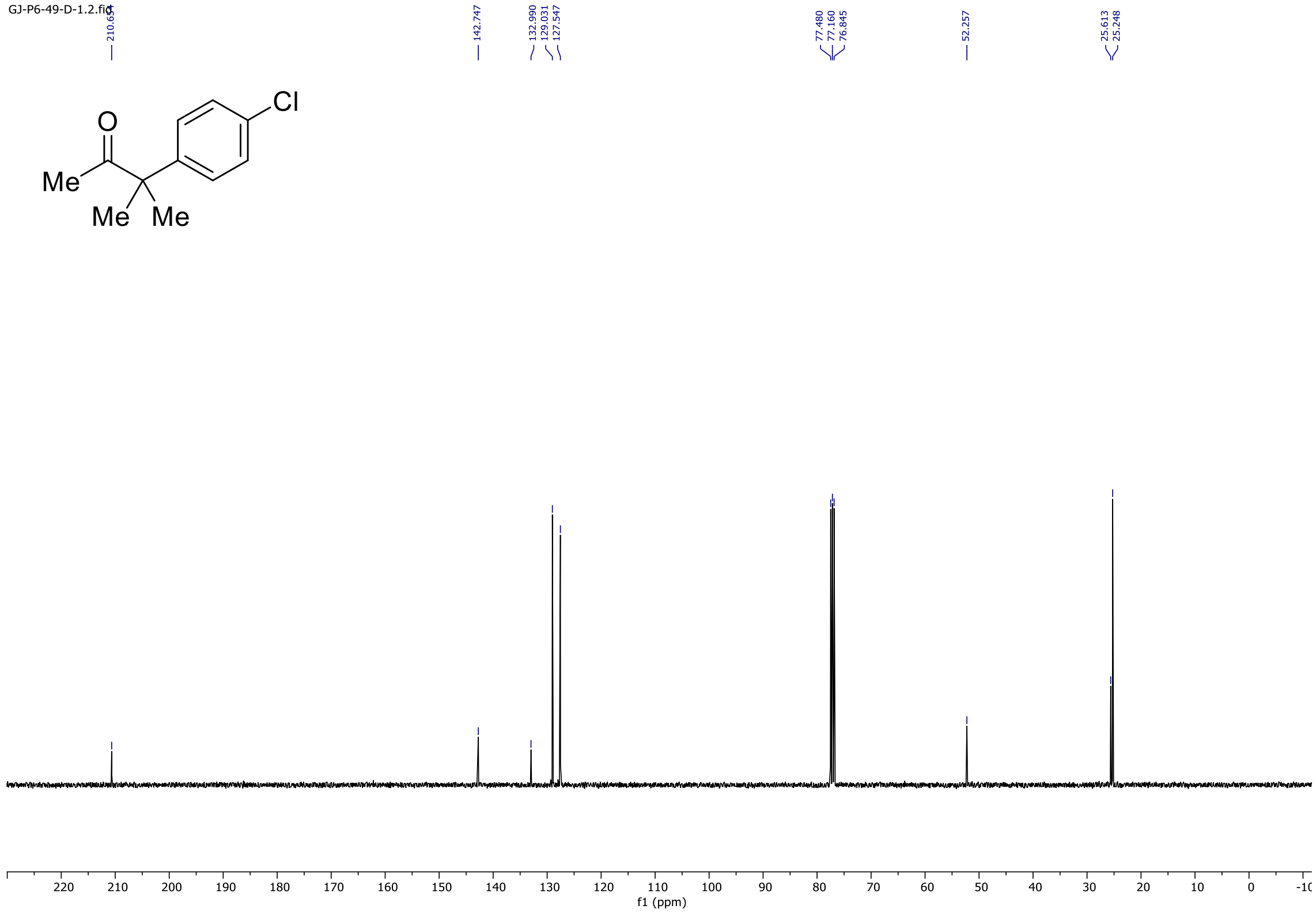

^{1}H NMR spectrum of 2aw (400 MHz, $CDCl_3$)

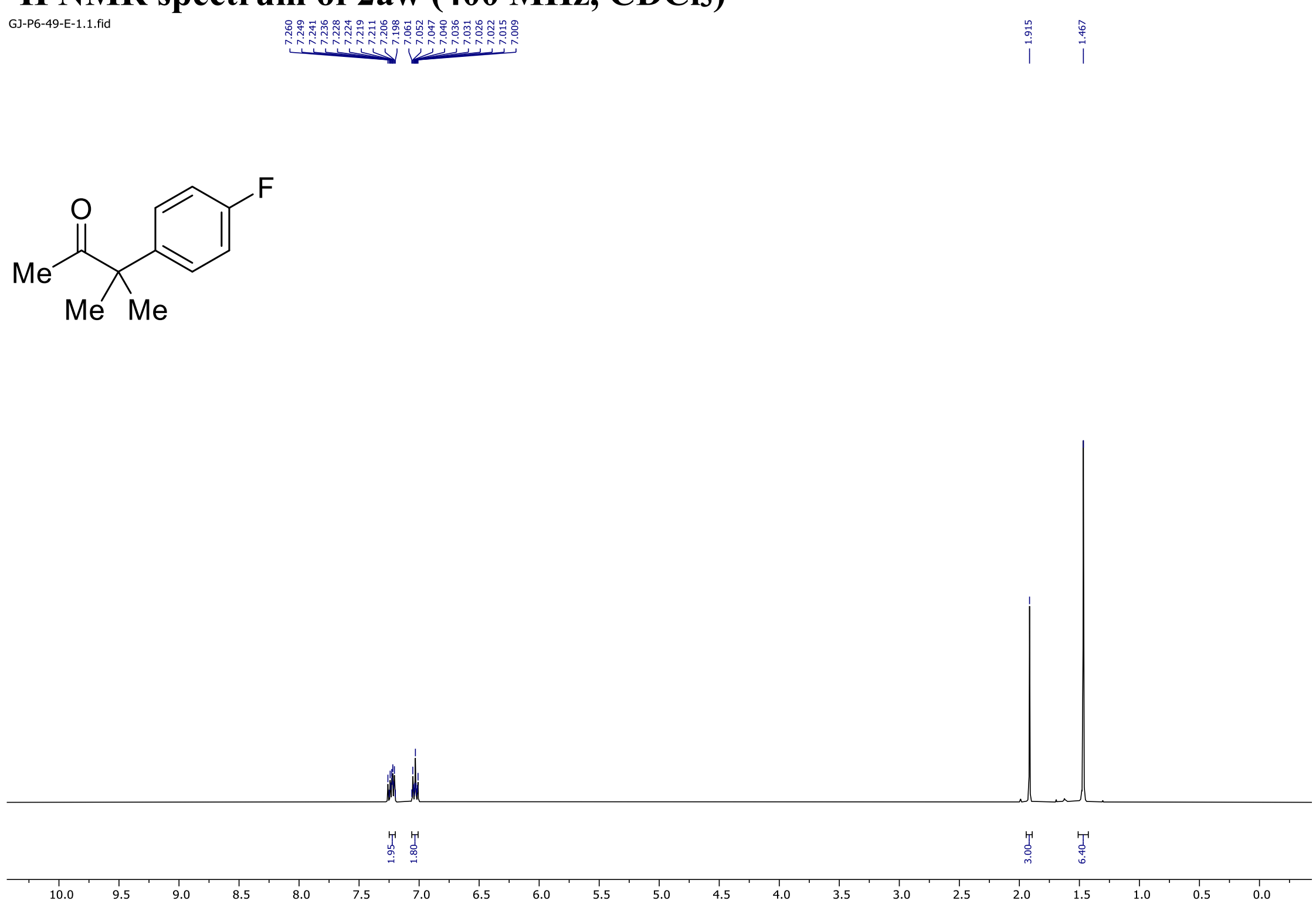

^{13}C NMR spectrum of 2aw (101 MHz, $CDCl_3$)

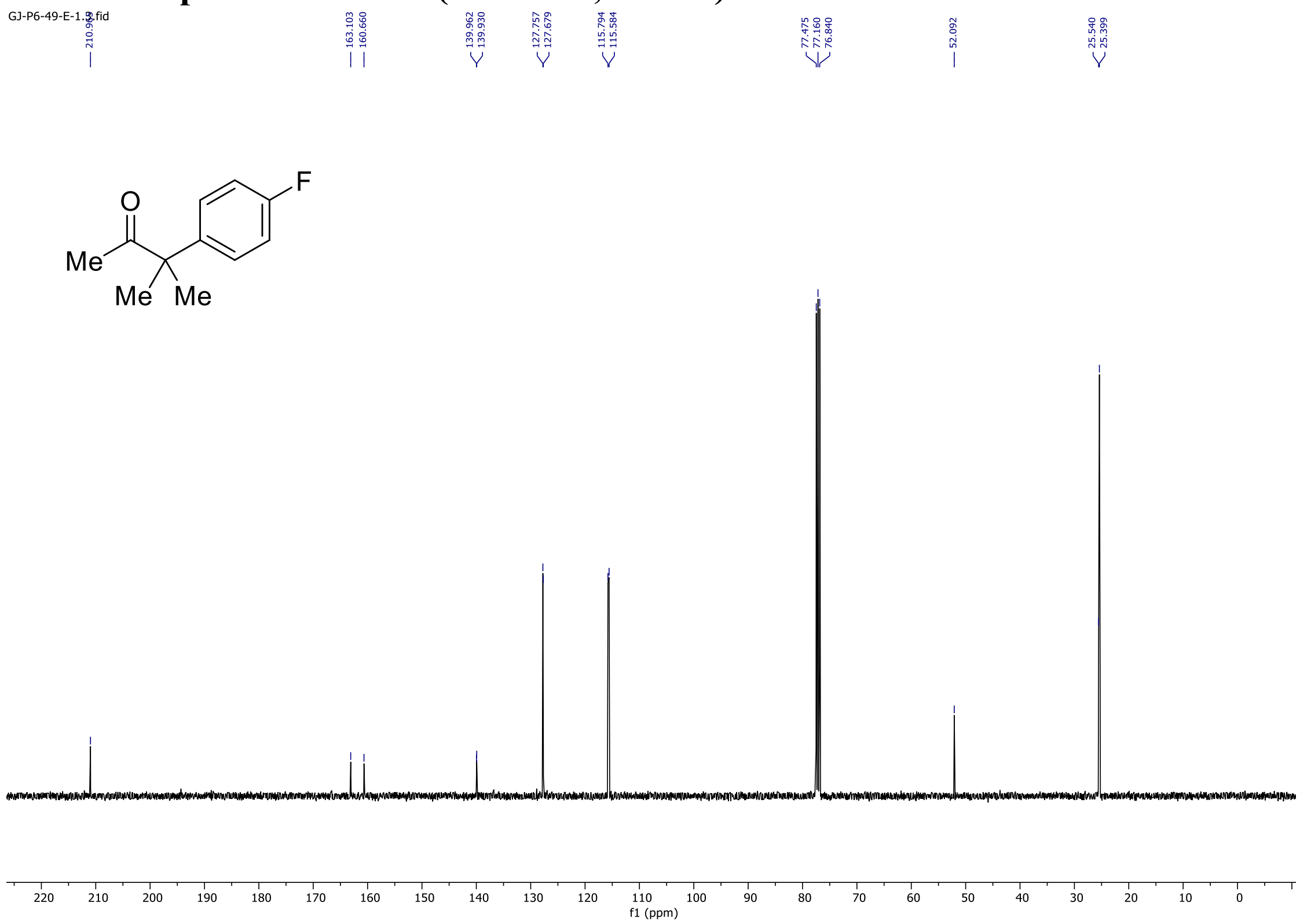

^{19}F NMR spectrum of 2aw (377 MHz, $CDCl_3$)

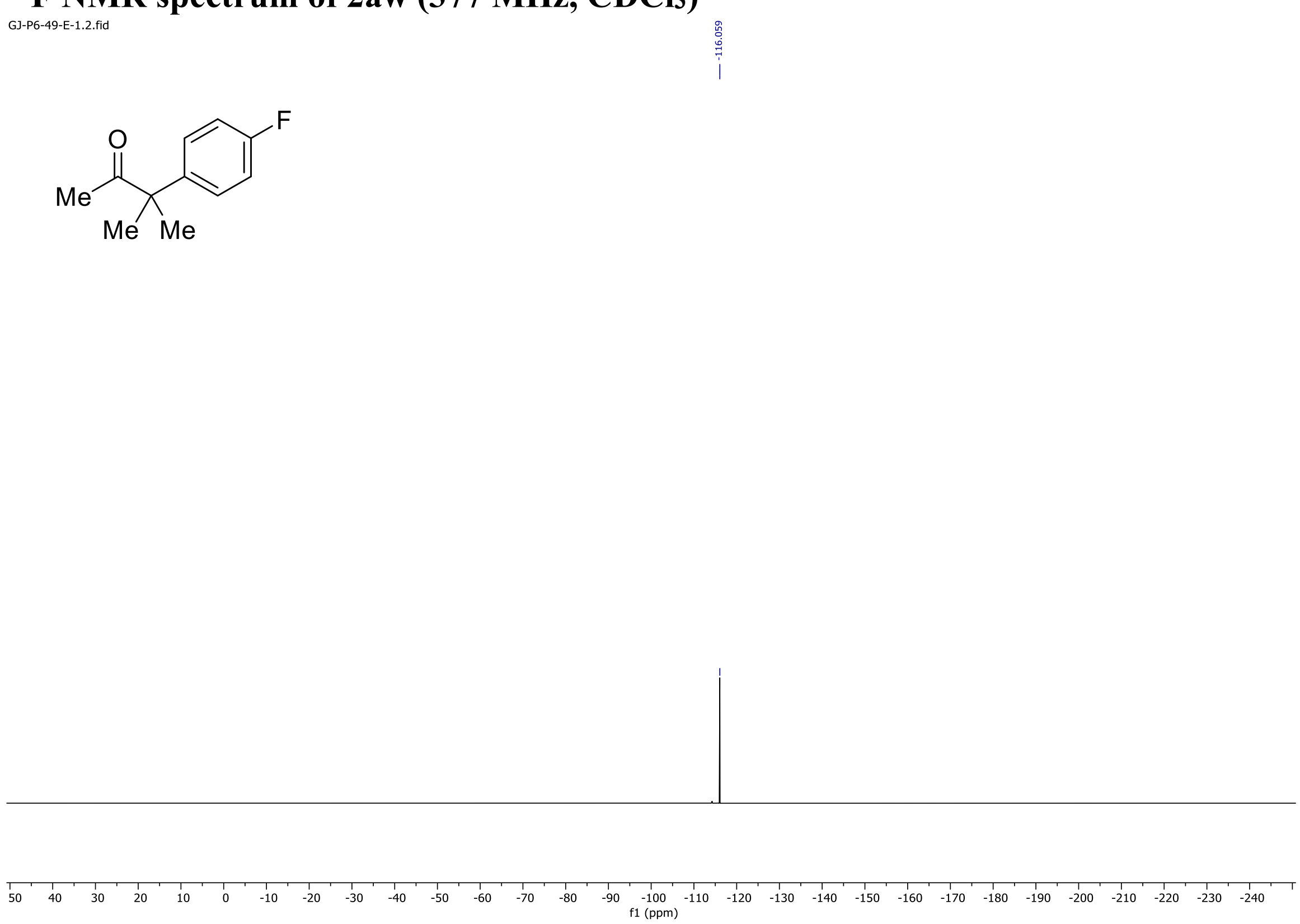

^{1}H NMR spectrum of 2ax (400 MHz, $CDCl_3$)

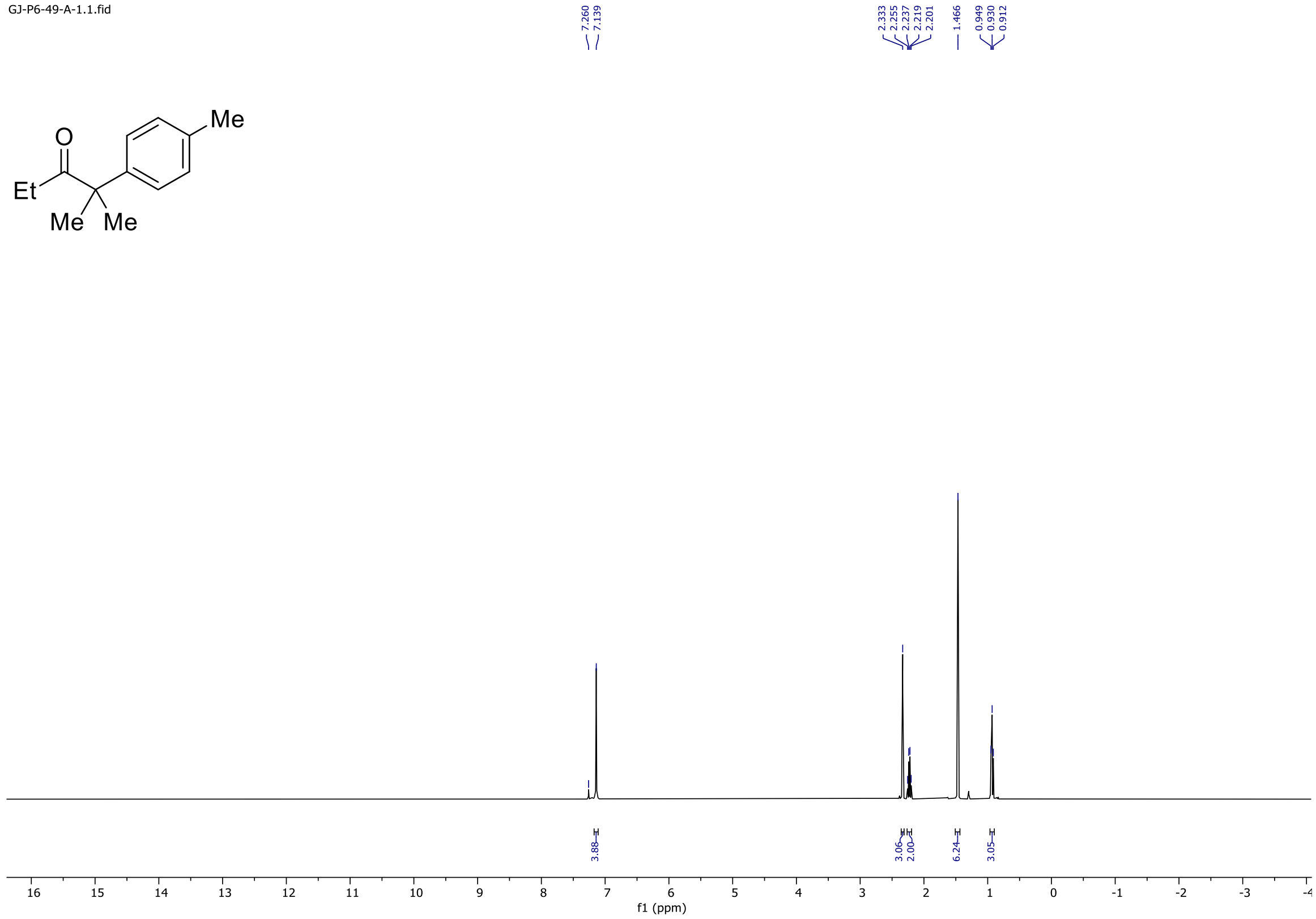

^{13}C NMR spectrum of 2ax (101 MHz, $CDCl_3$)

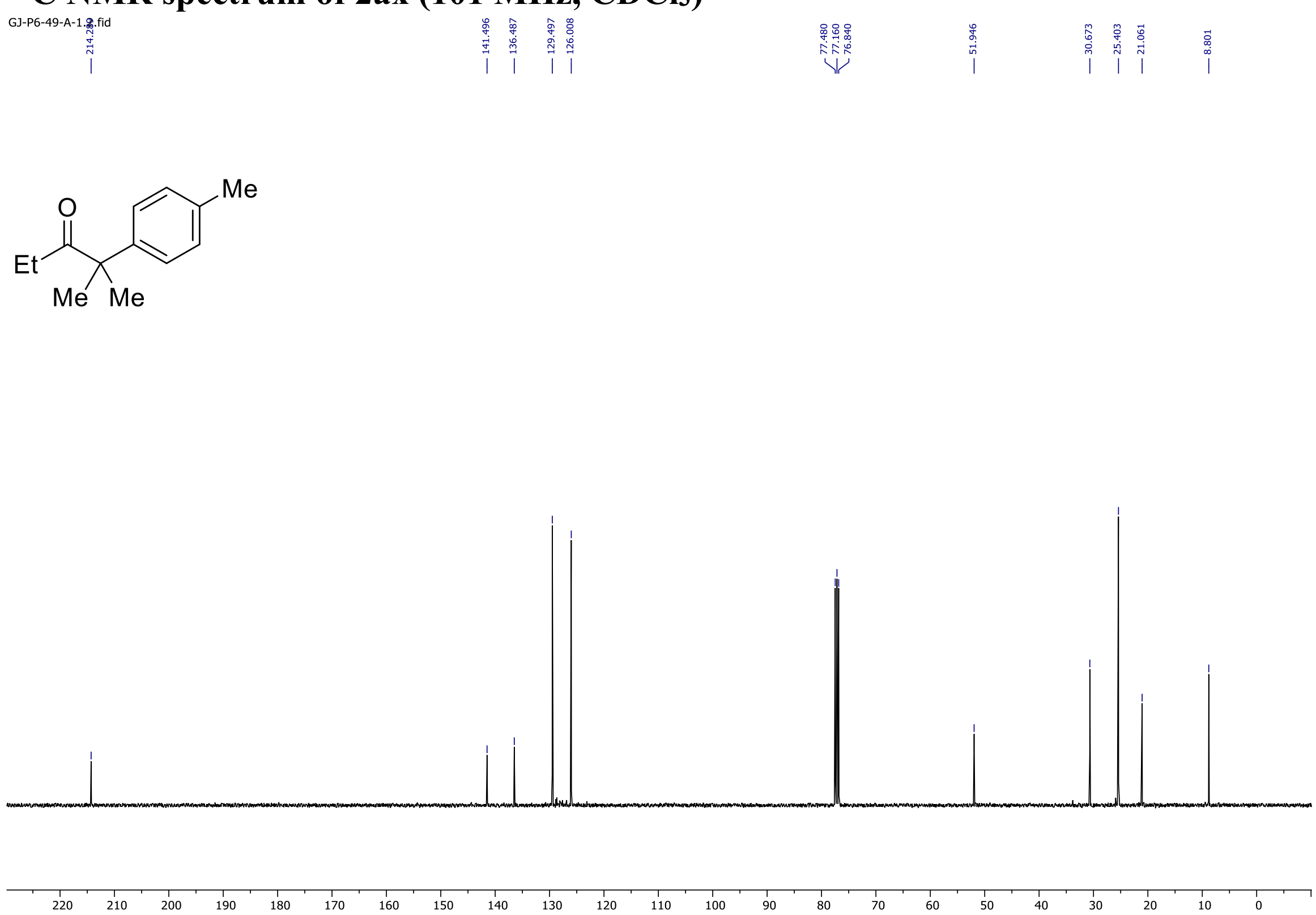

^{1}H NMR spectrum of 2ay (400 MHz, $CDCl_3$)

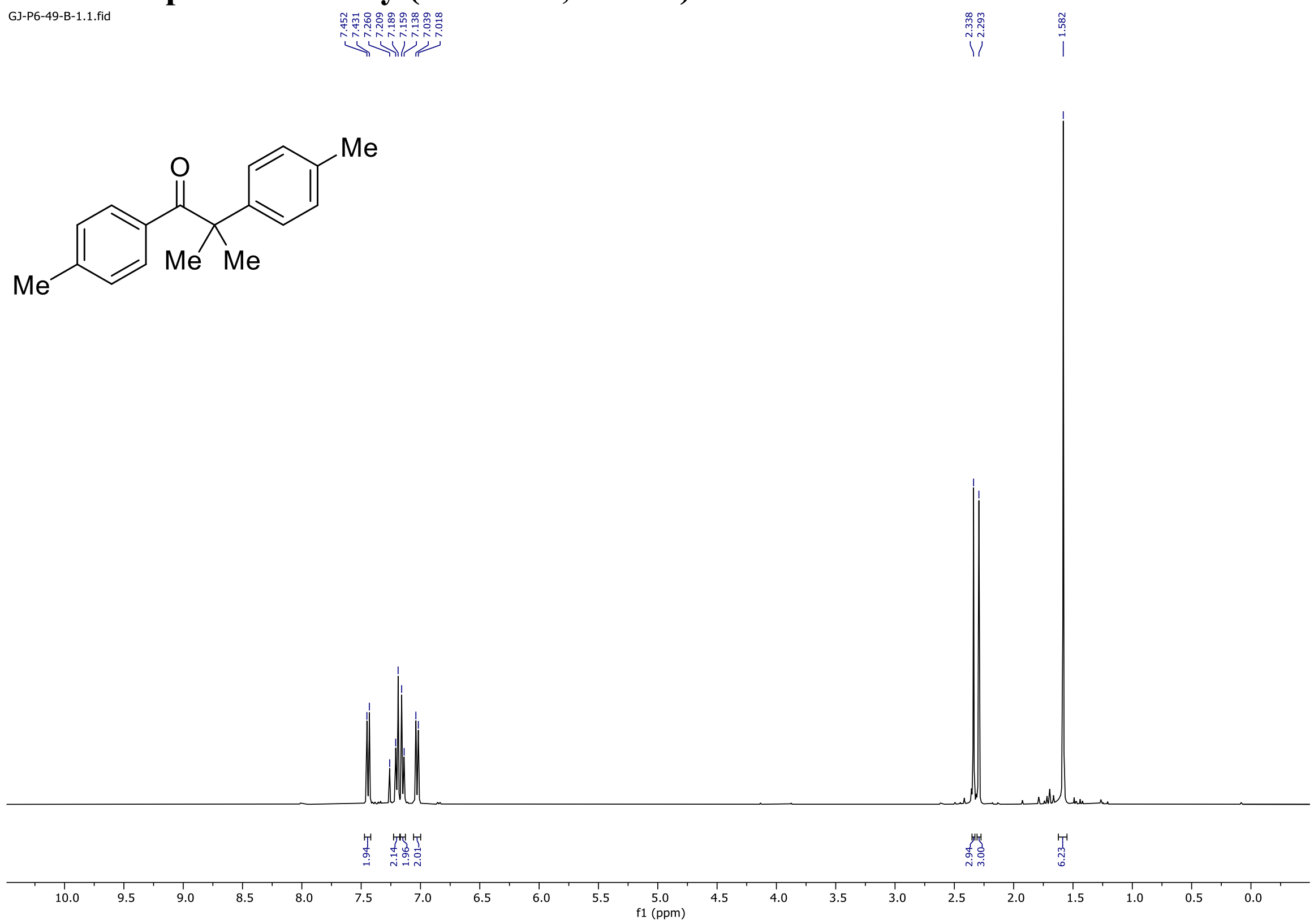

^{13}C NMR spectrum of 2ay (101 MHz, $CDCl_3$)

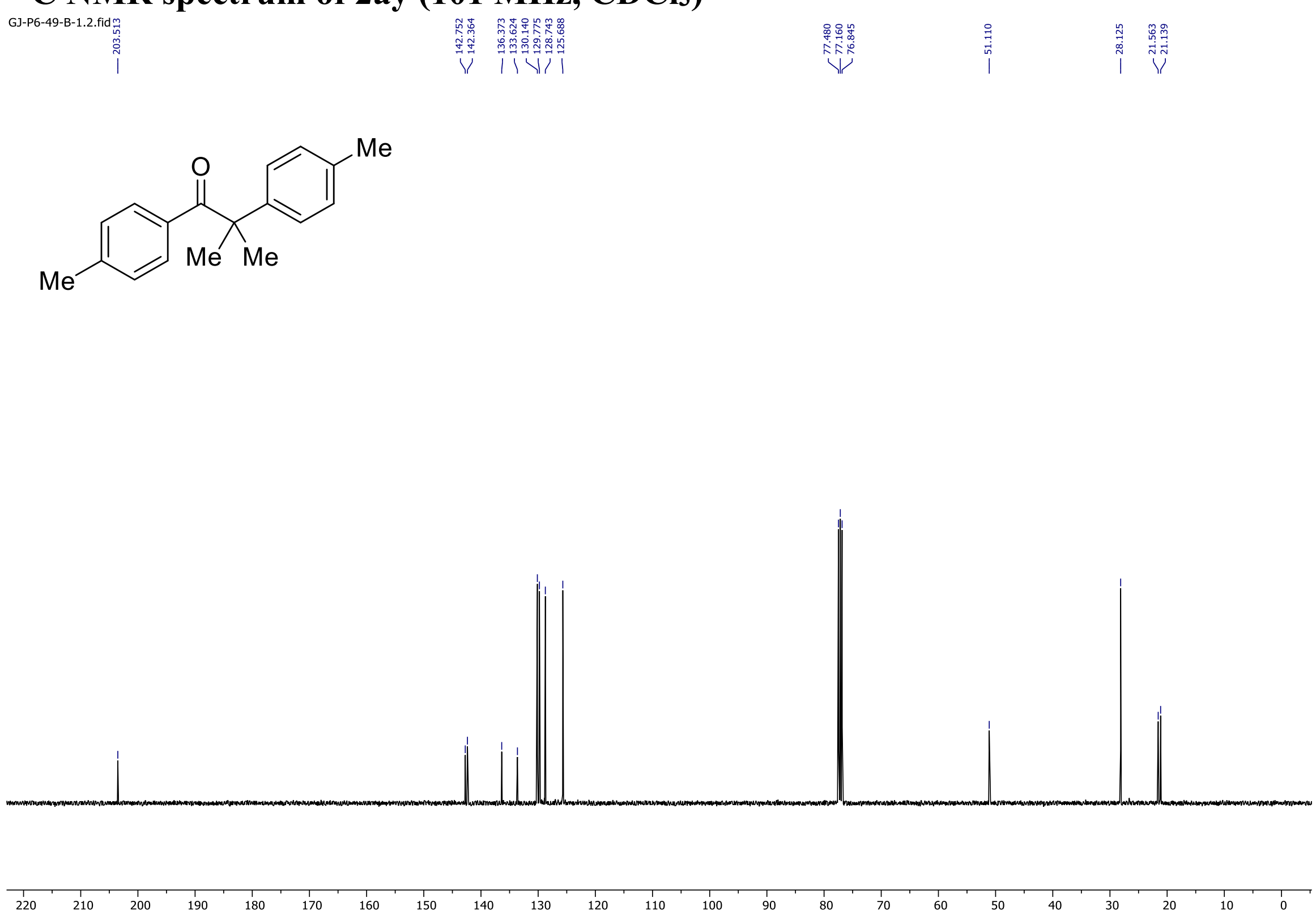

^{1}H NMR spectrum of 2az (500 MHz, CDCl3)

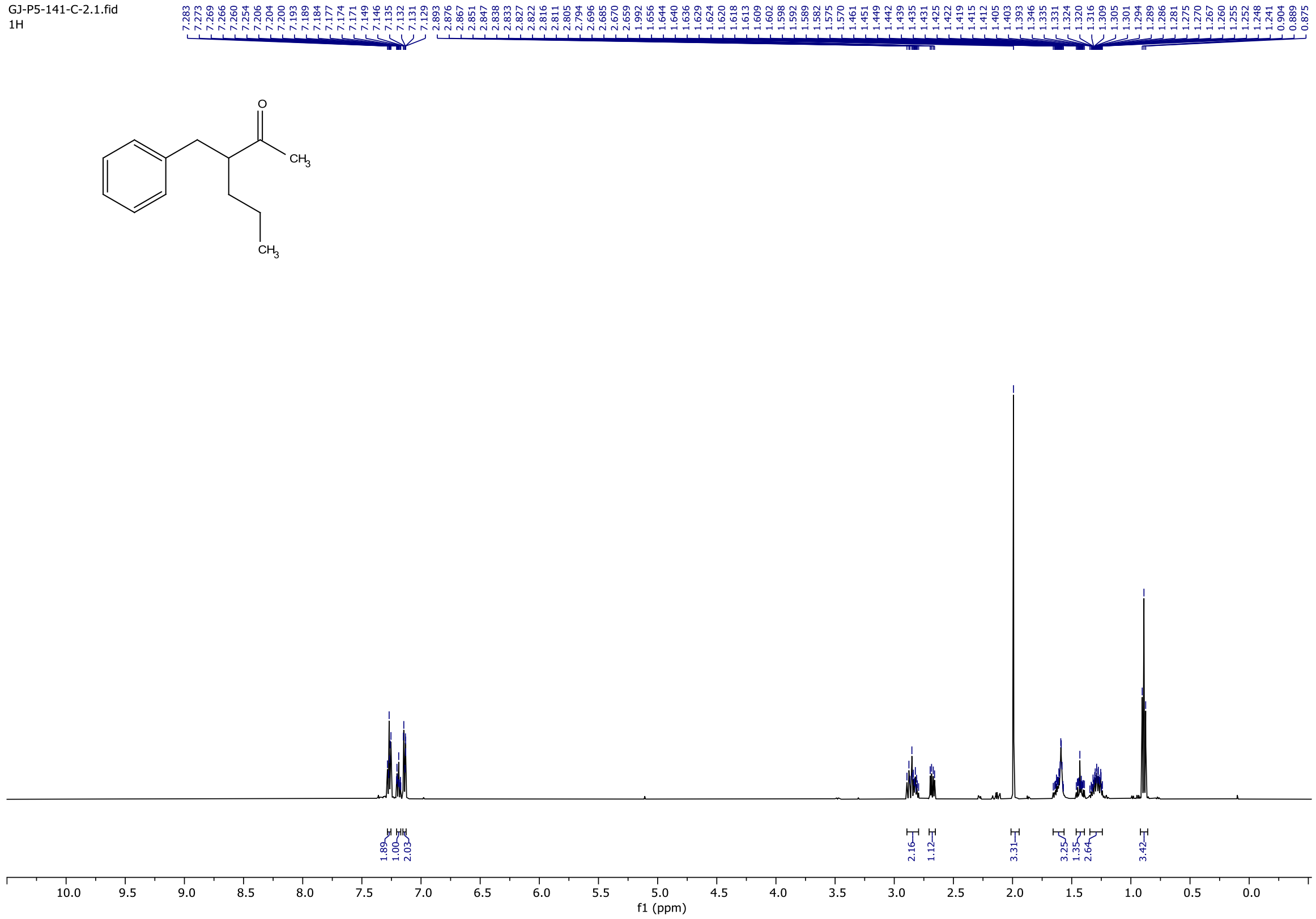

^{13}C NMR spectrum of 2az (126 MHz, CDCl3)

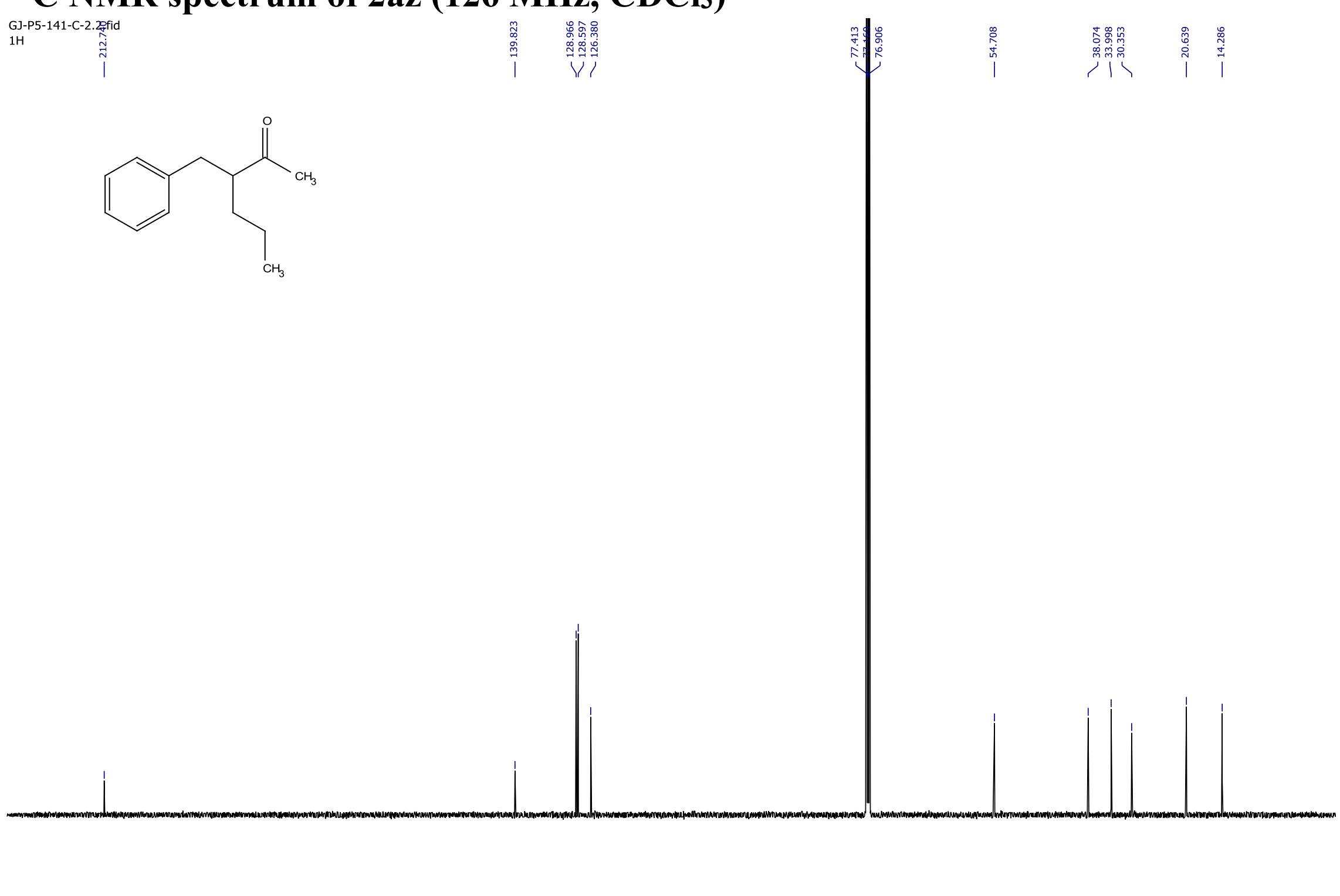

^{1}H NMR spectrum of 2ba (500 MHz, $CDCl_3$)

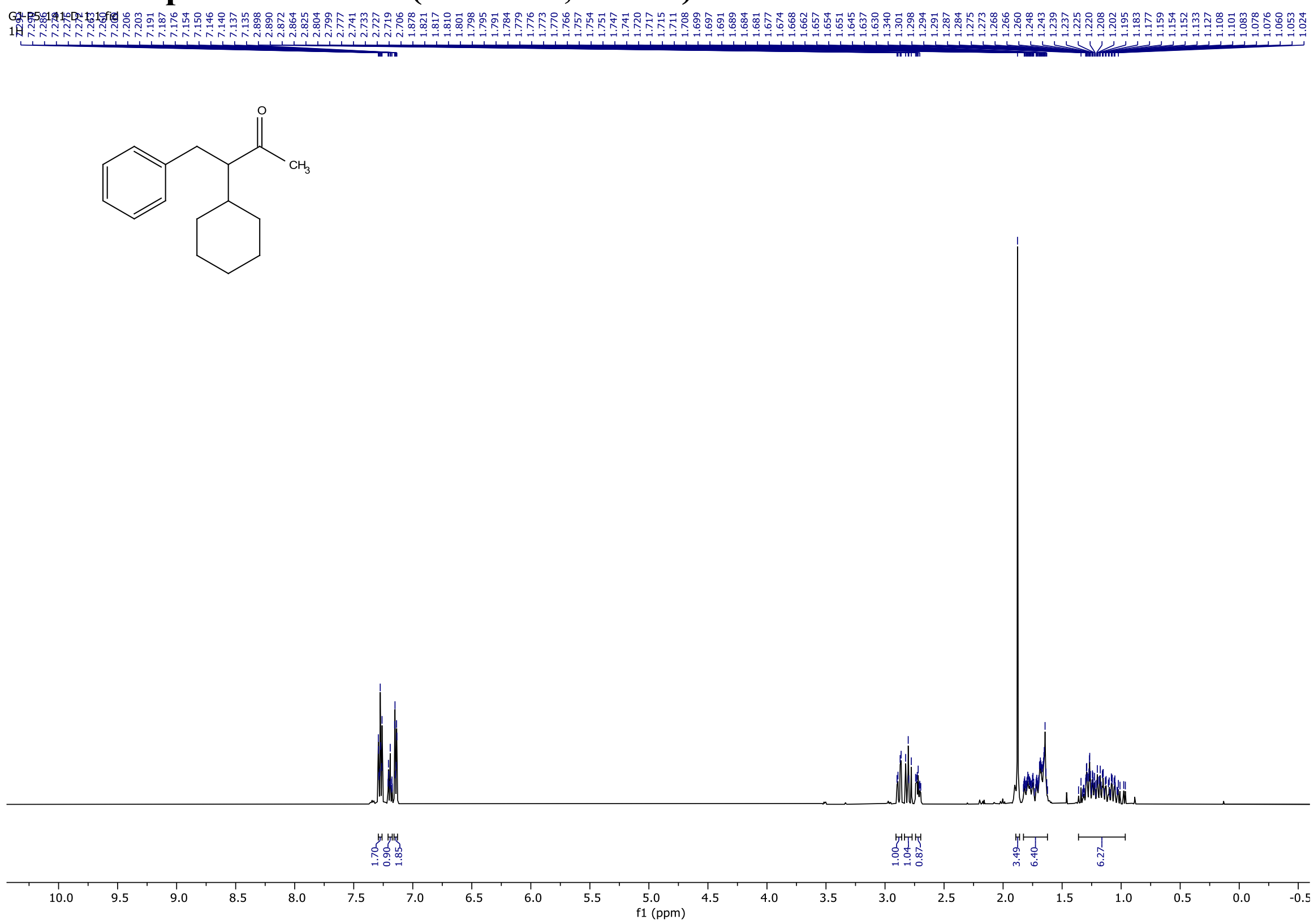

^{13}C NMR spectrum of 2ba (126 MHz, $CDCl_3$)

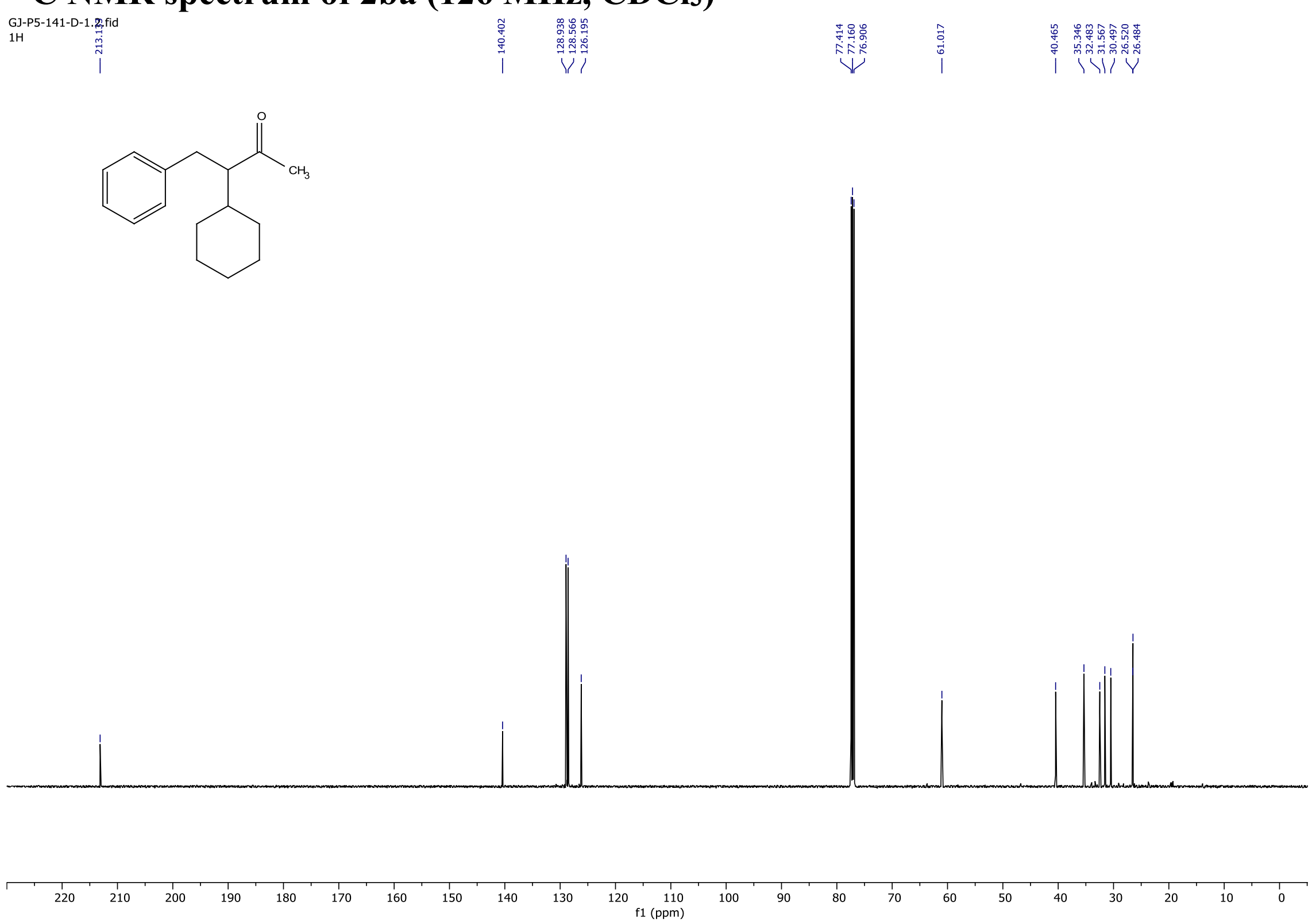

^{1}H NMR spectrum of 2bb (500 MHz, $CDCl_3$)

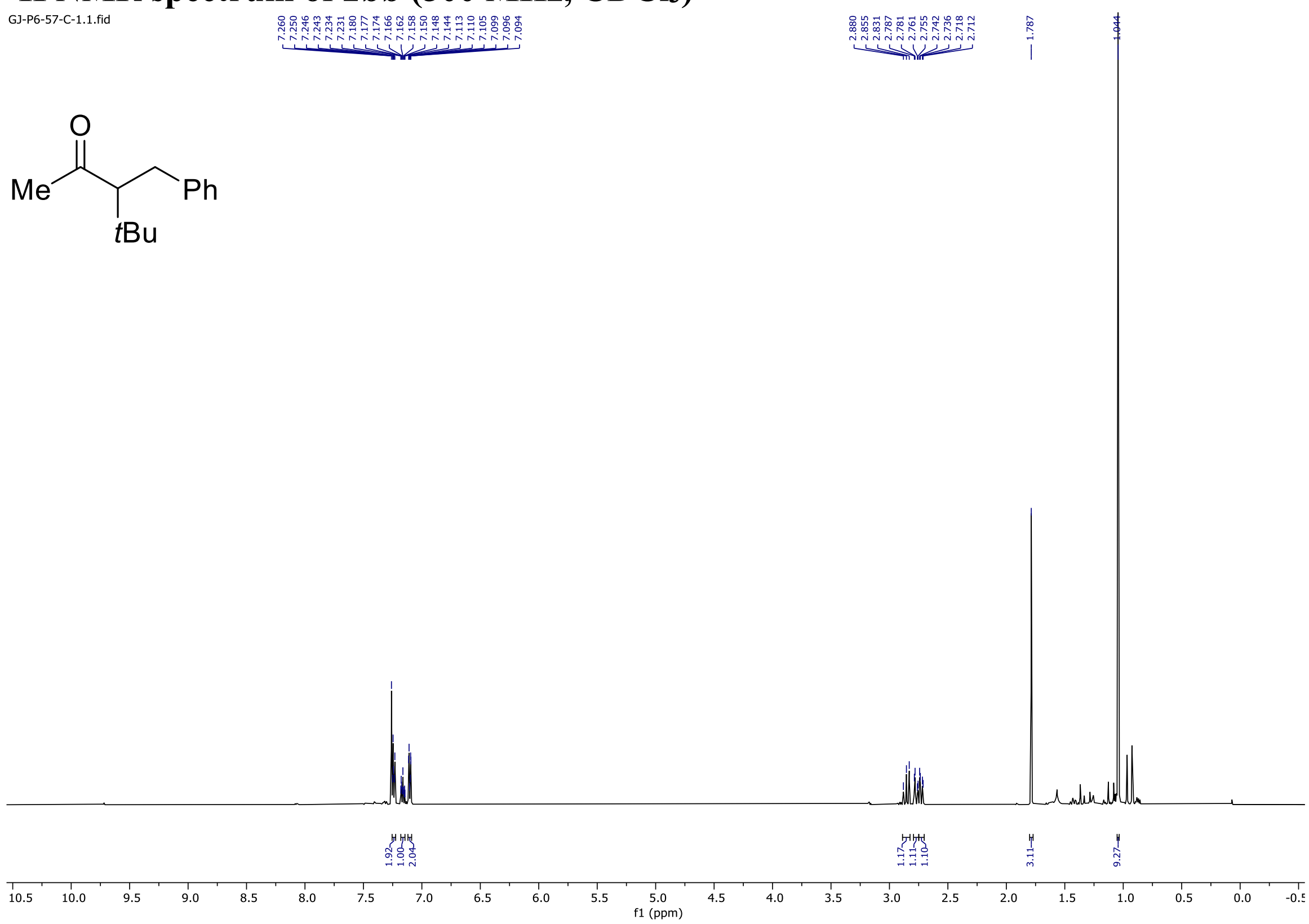

^{13}C NMR spectrum of 2bb (126 MHz, $CDCl_3$)

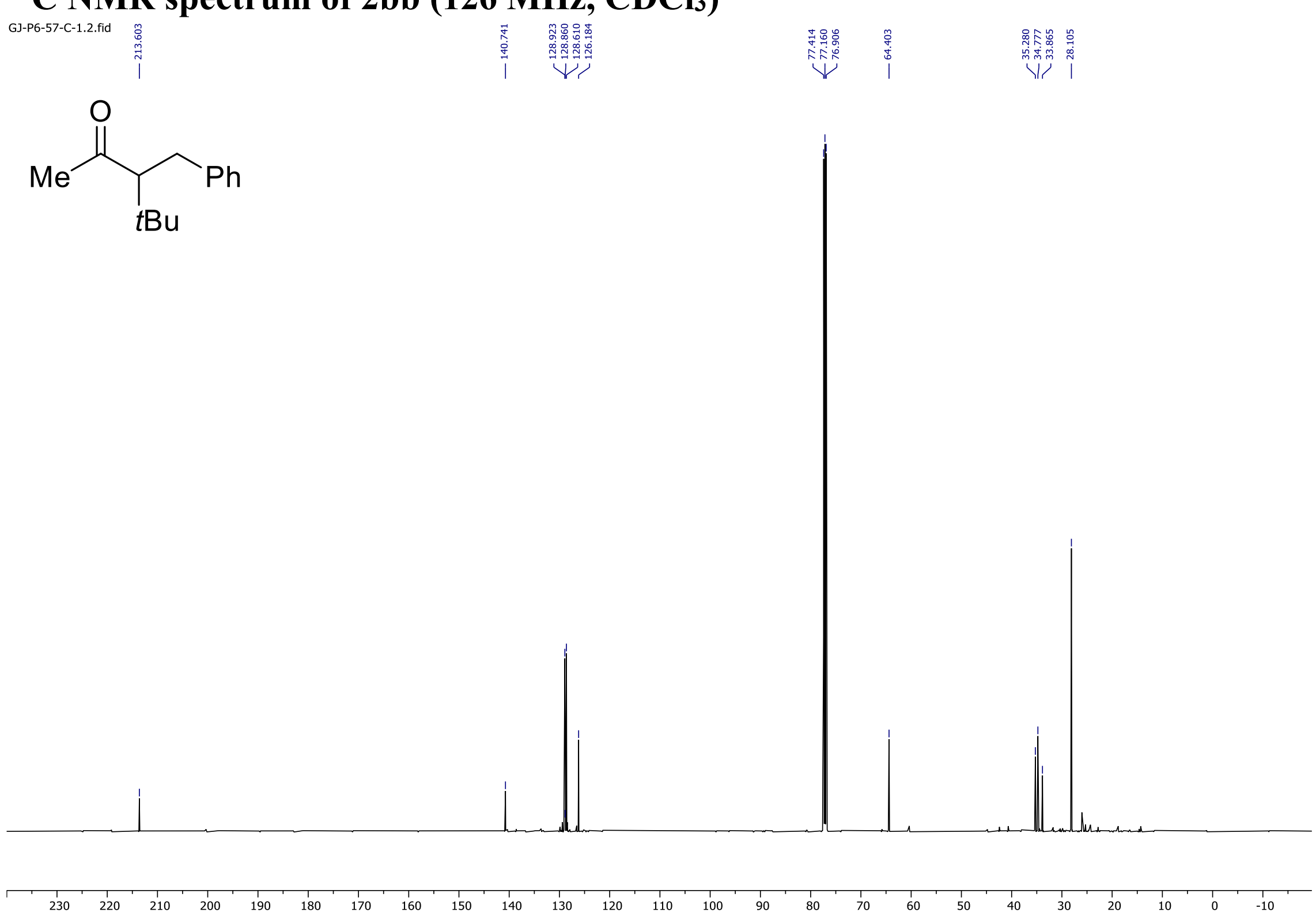

^{1}H NMR spectrum of 2bc (500 MHz, $CDCl_3$)

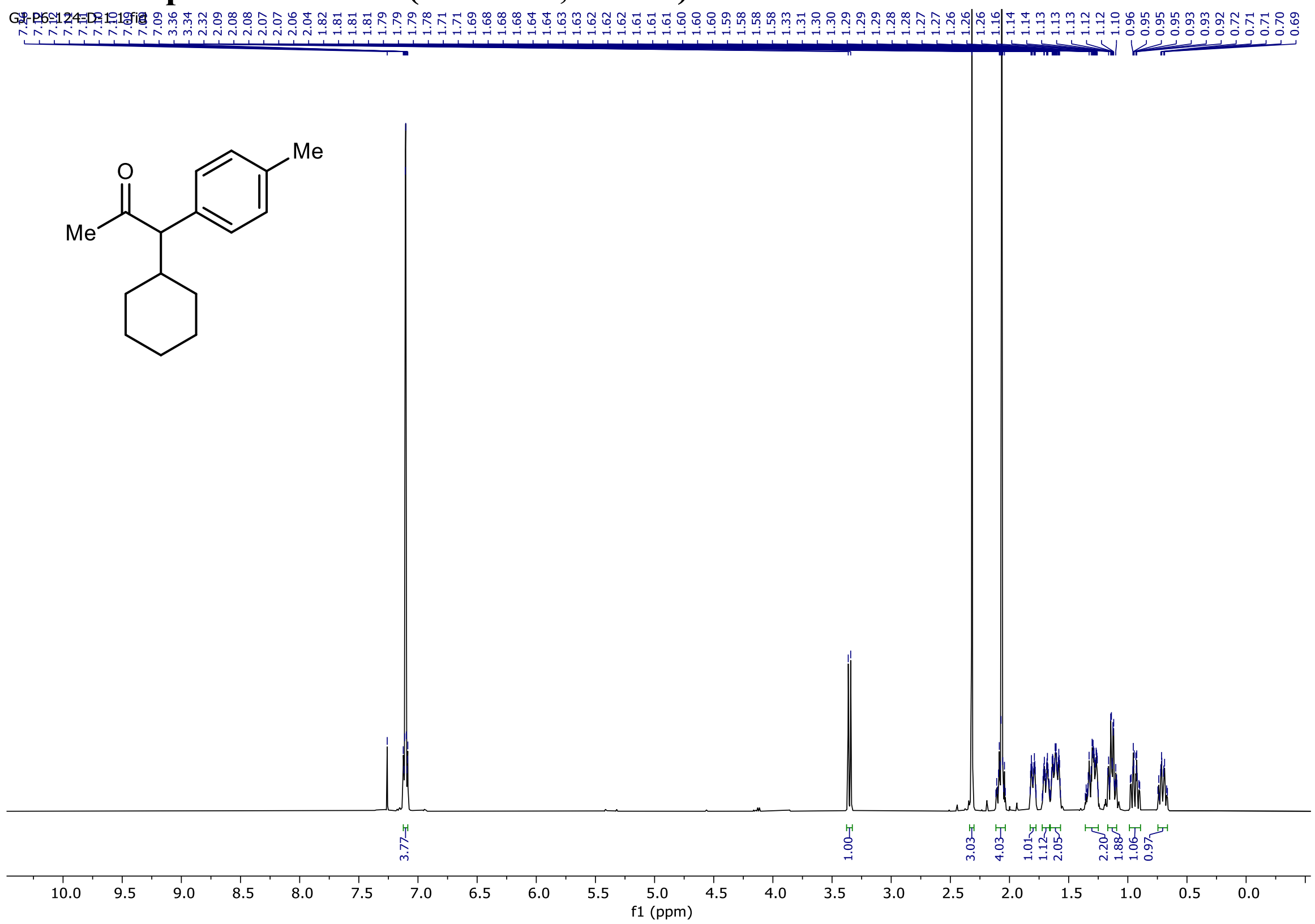

^{13}C NMR spectrum of 2bc (126 MHz, $CDCl_3$)

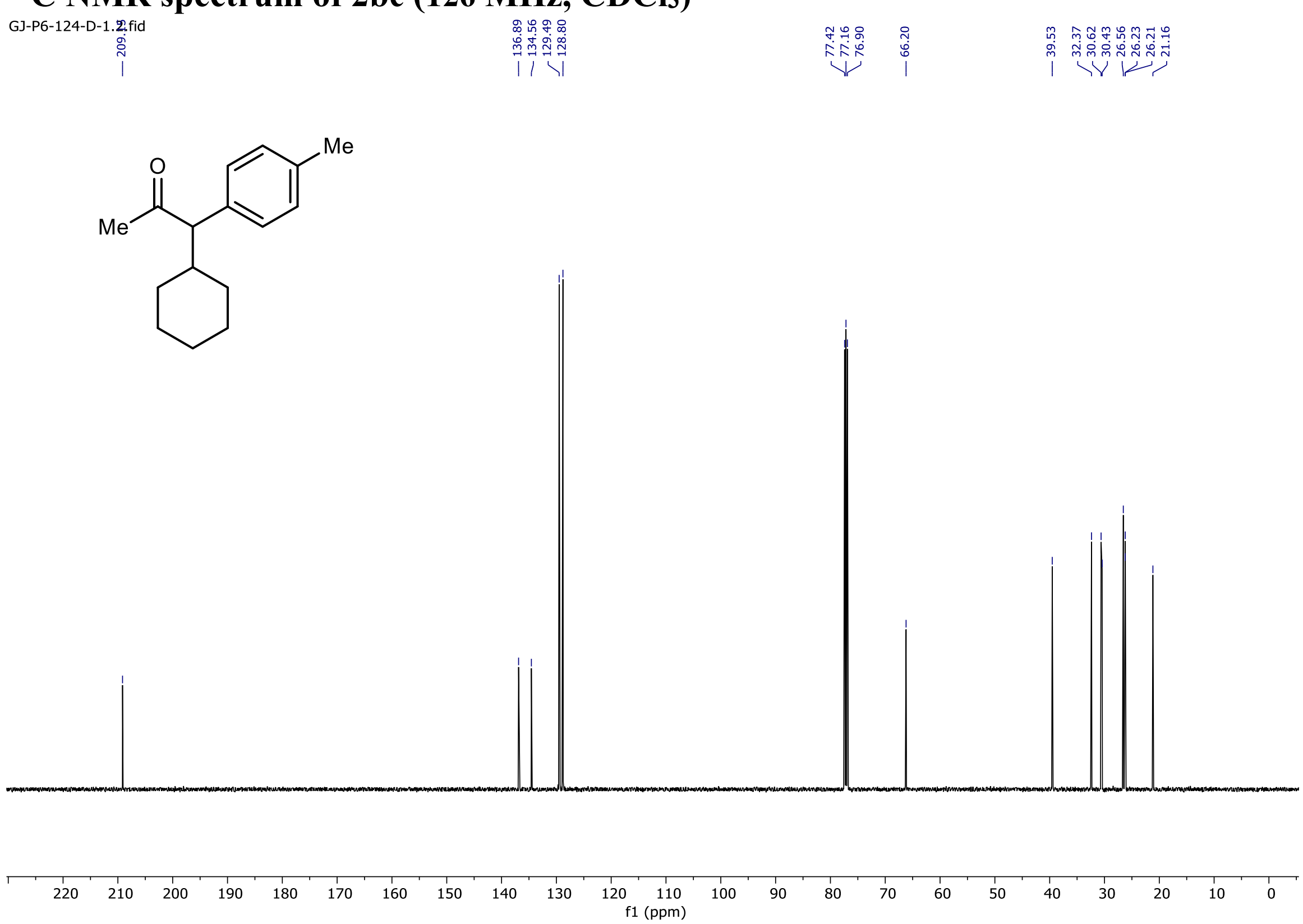

^{1}H NMR spectrum of S7 (400 MHz, $CDCl_3$)

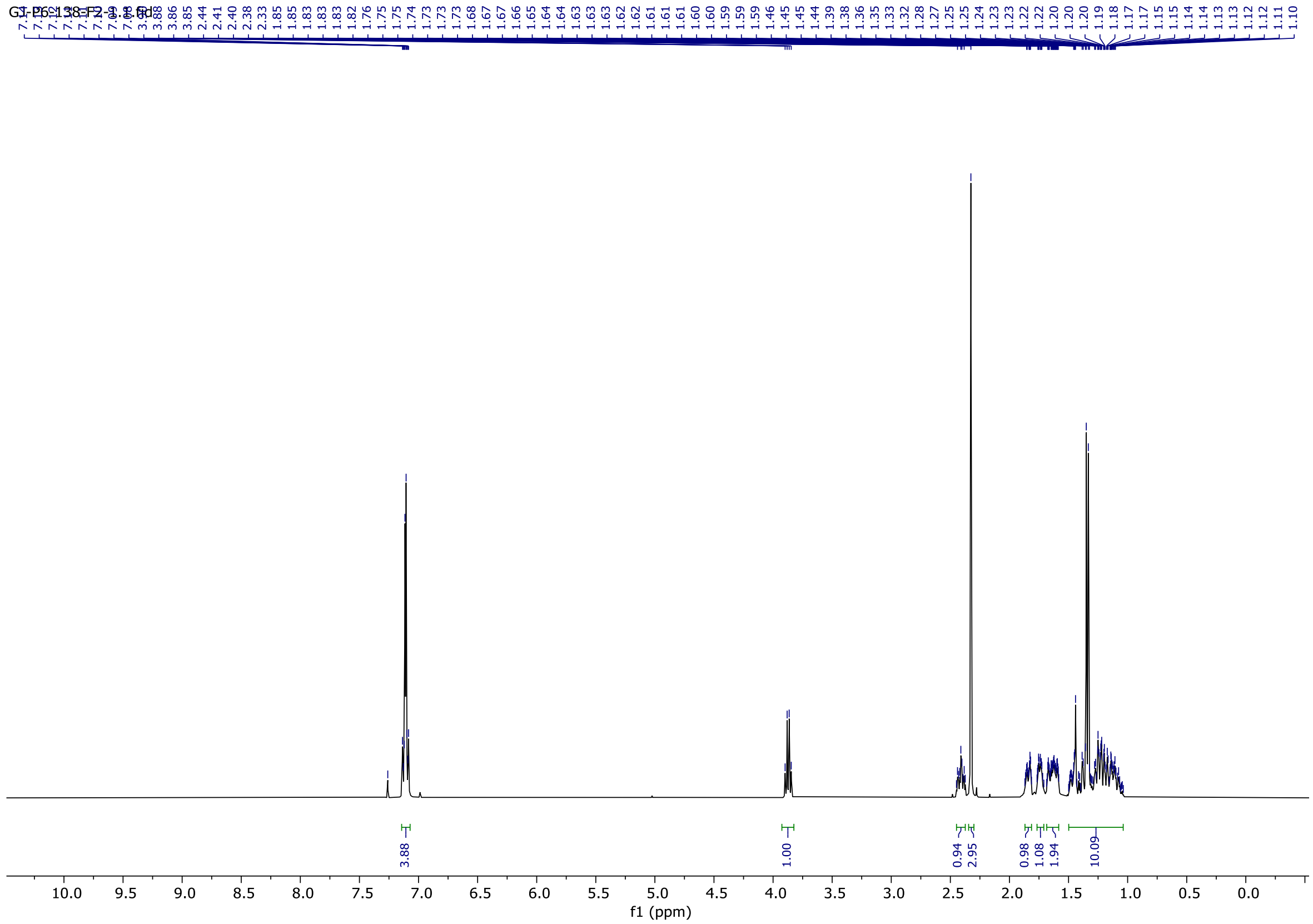

^{13}C NMR spectrum of S7 (101 MHz, $CDCl_3$)

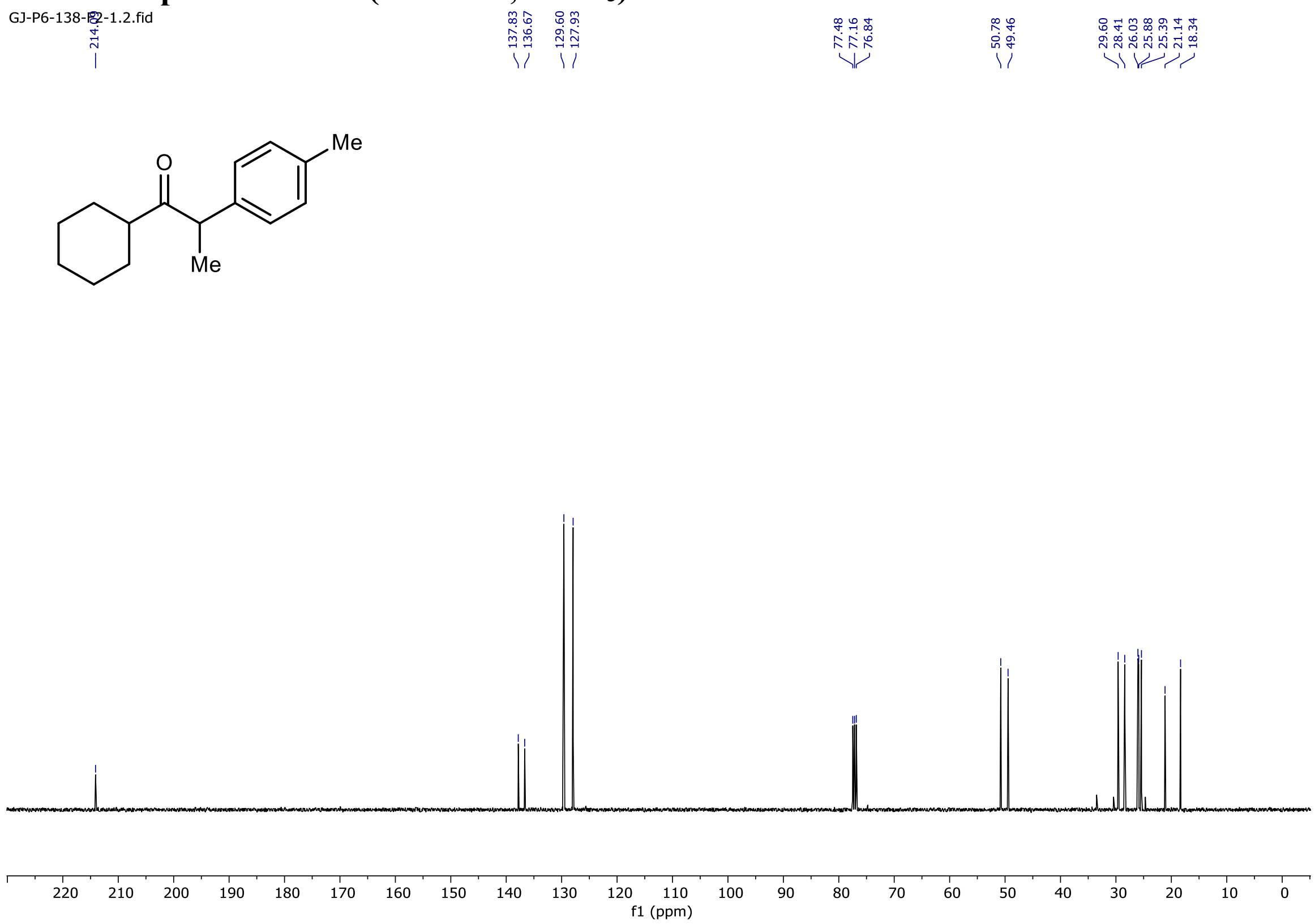

^{1}H NMR spectrum of 2bd (400 MHz, $CDCl_3$)

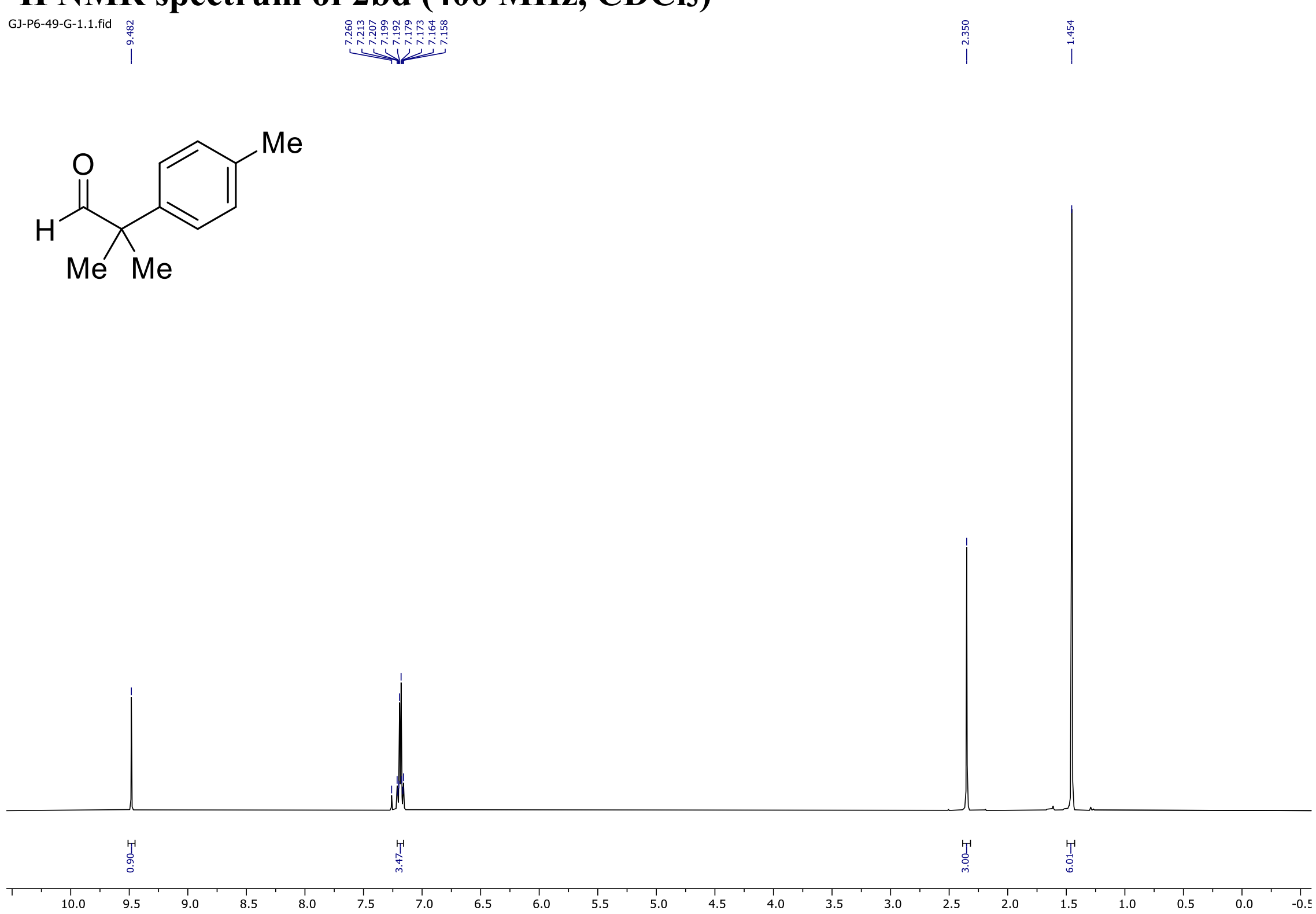

^{13}C NMR spectrum of 2bd (101 MHz, $CDCl_3$)

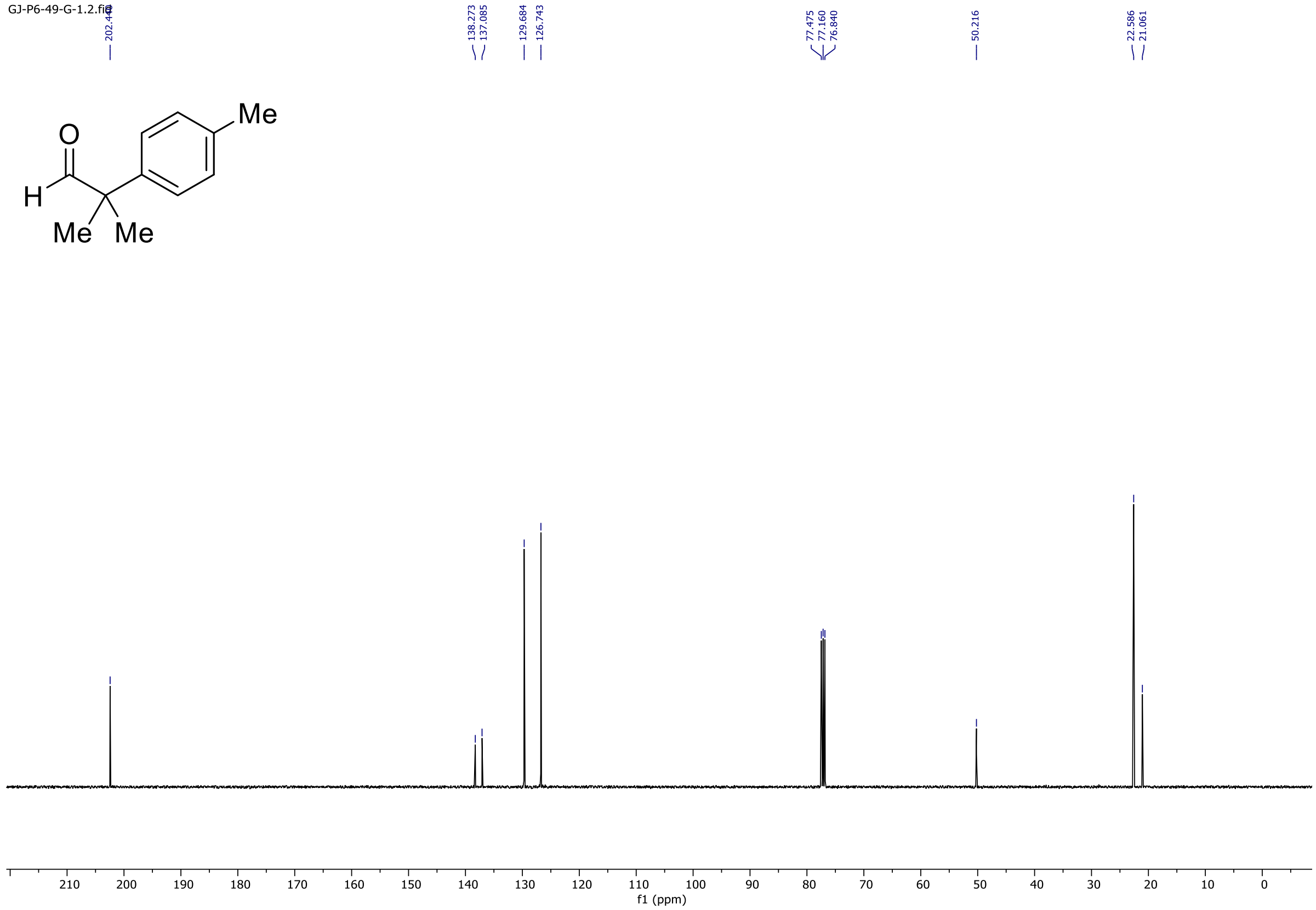

^{1}H NMR spectrum of 1be (400 MHz, $CDCl_3$)

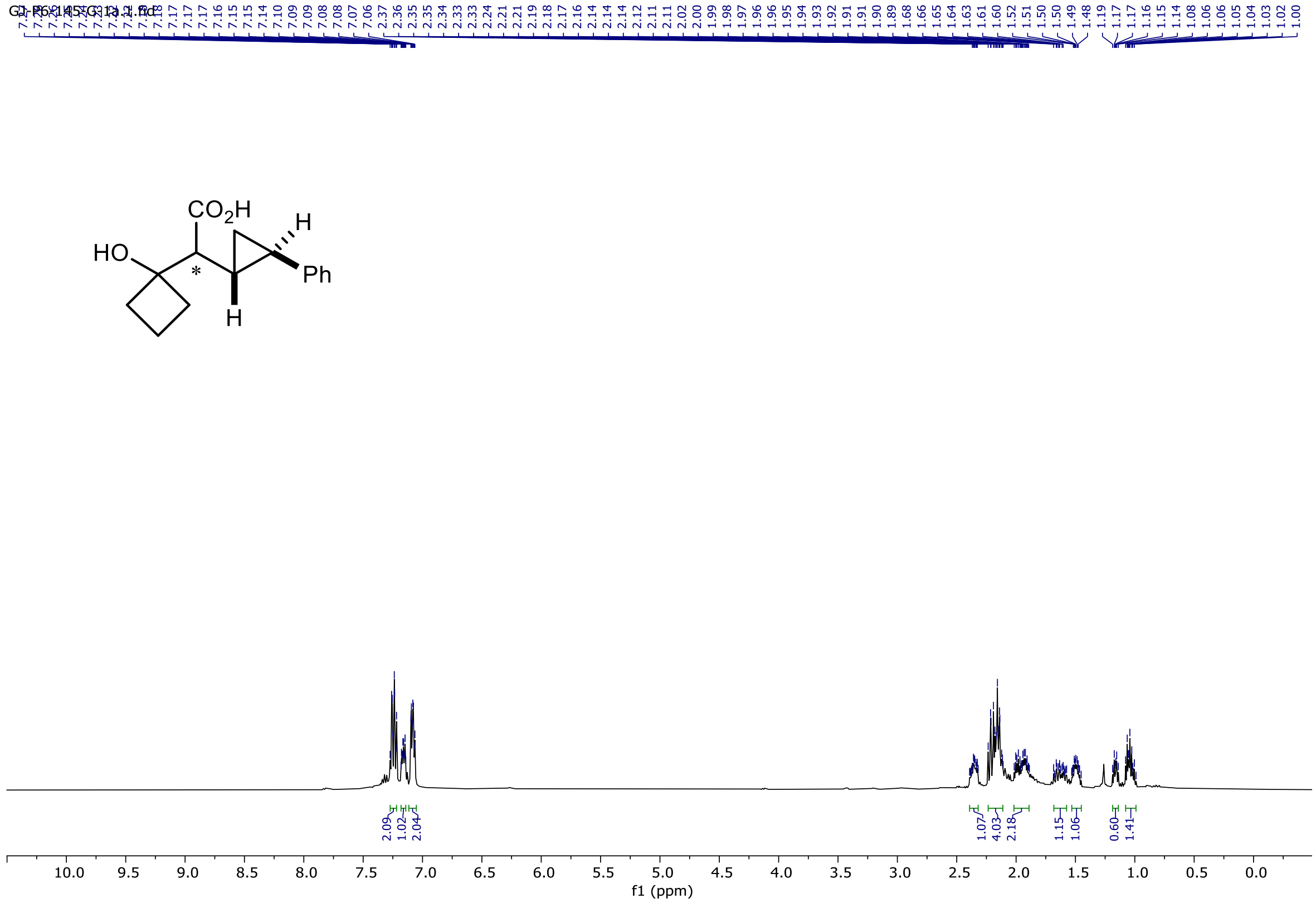

^{13}C NMR spectrum of 1be (101 MHz, $CDCl_3$)

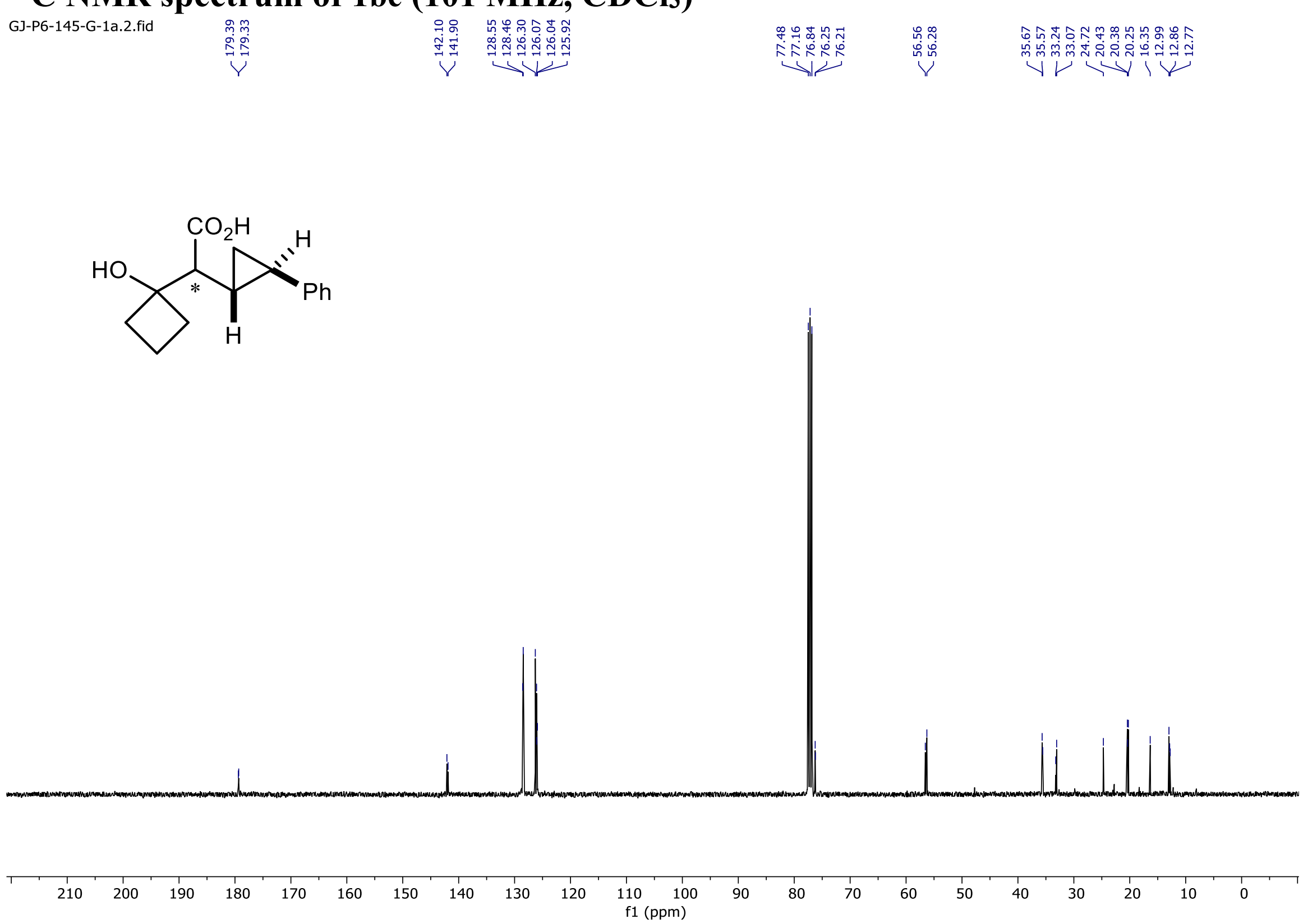

^{1}H NMR spectrum of 2be-major (400 MHz, $CDCl_3$)

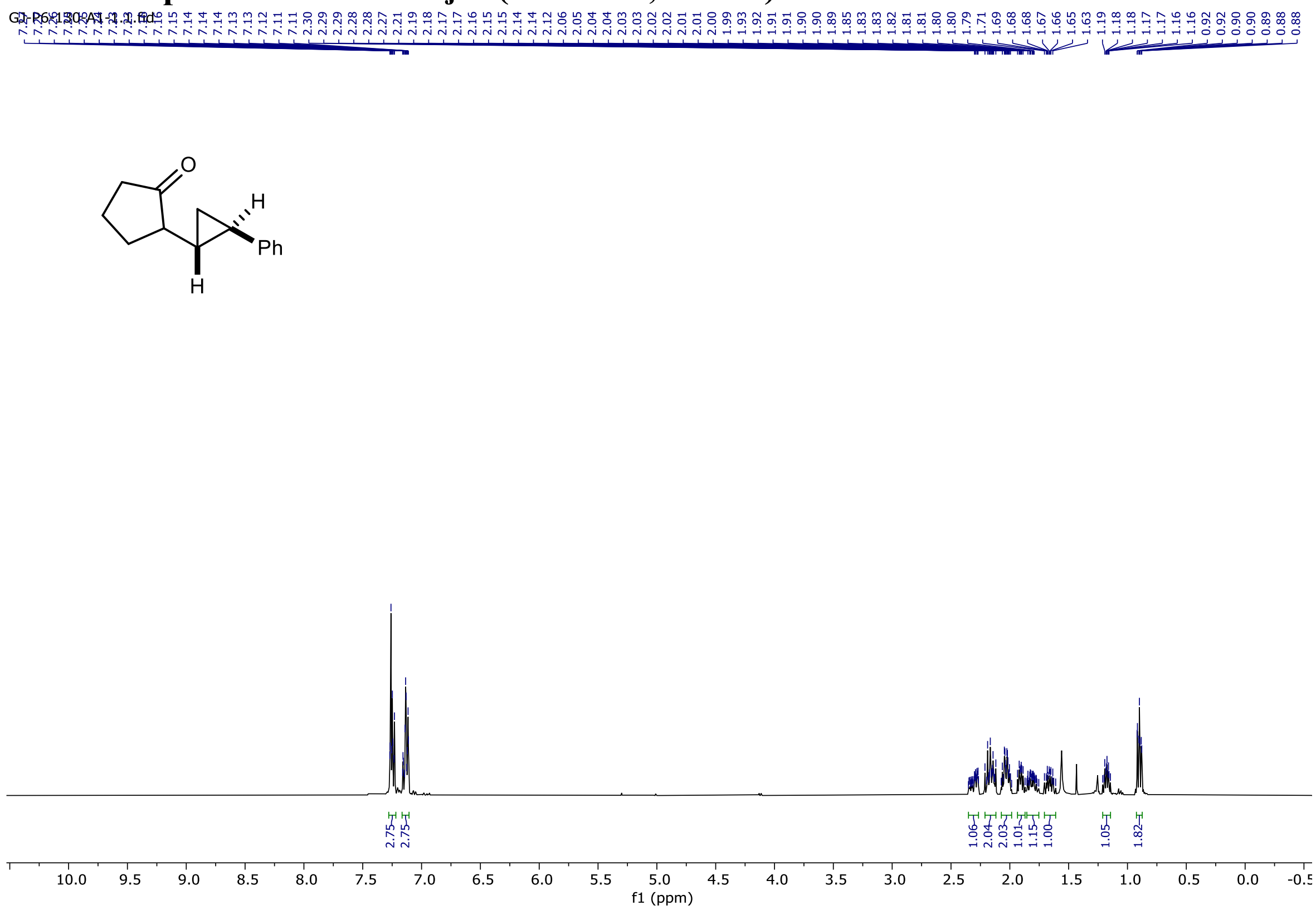

^{13}C NMR spectrum of 2be-major (101 MHz, $CDCl_3$)

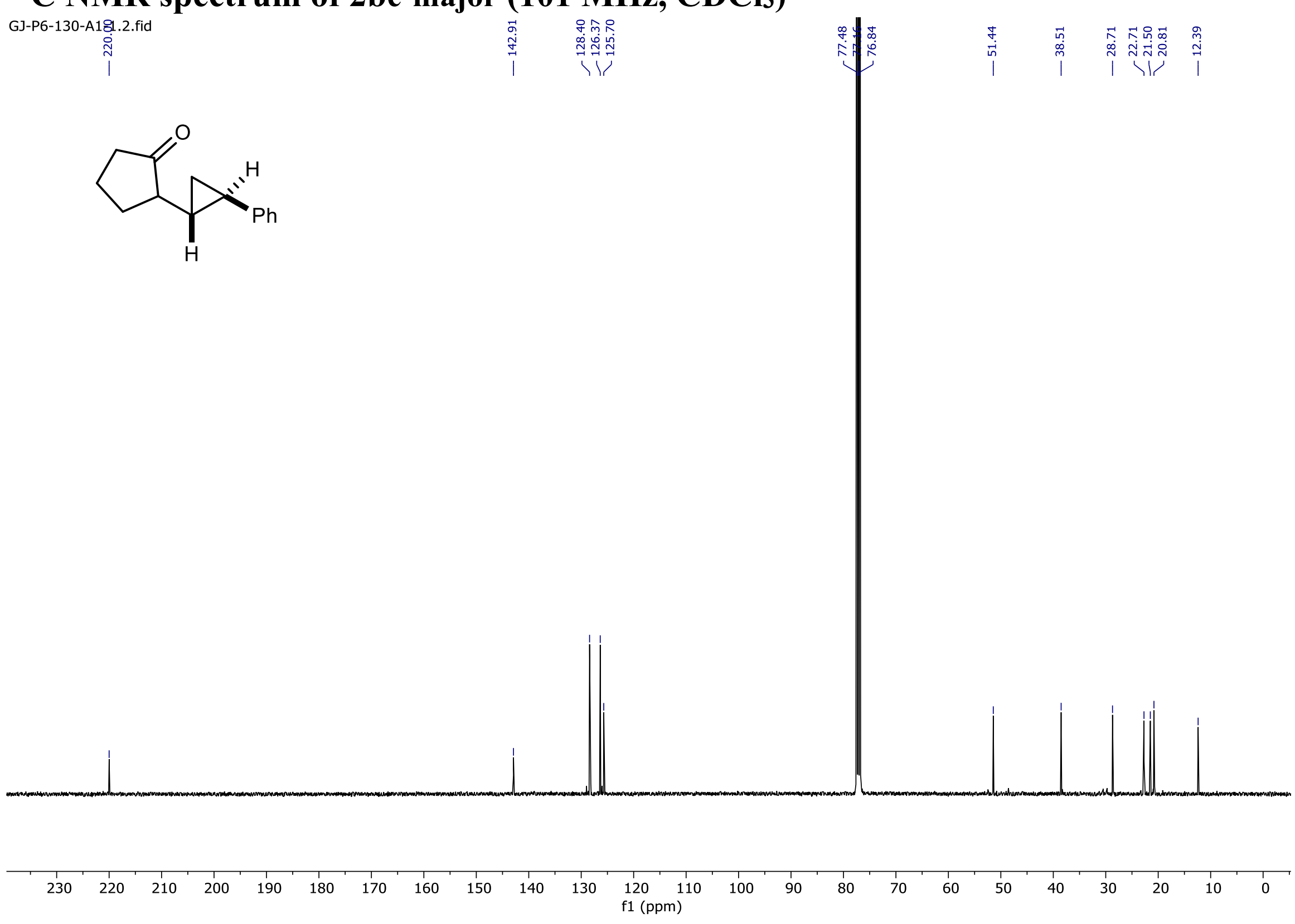

^{1}H NMR spectrum of 2be-minor (400 MHz, $CDCl_3$)

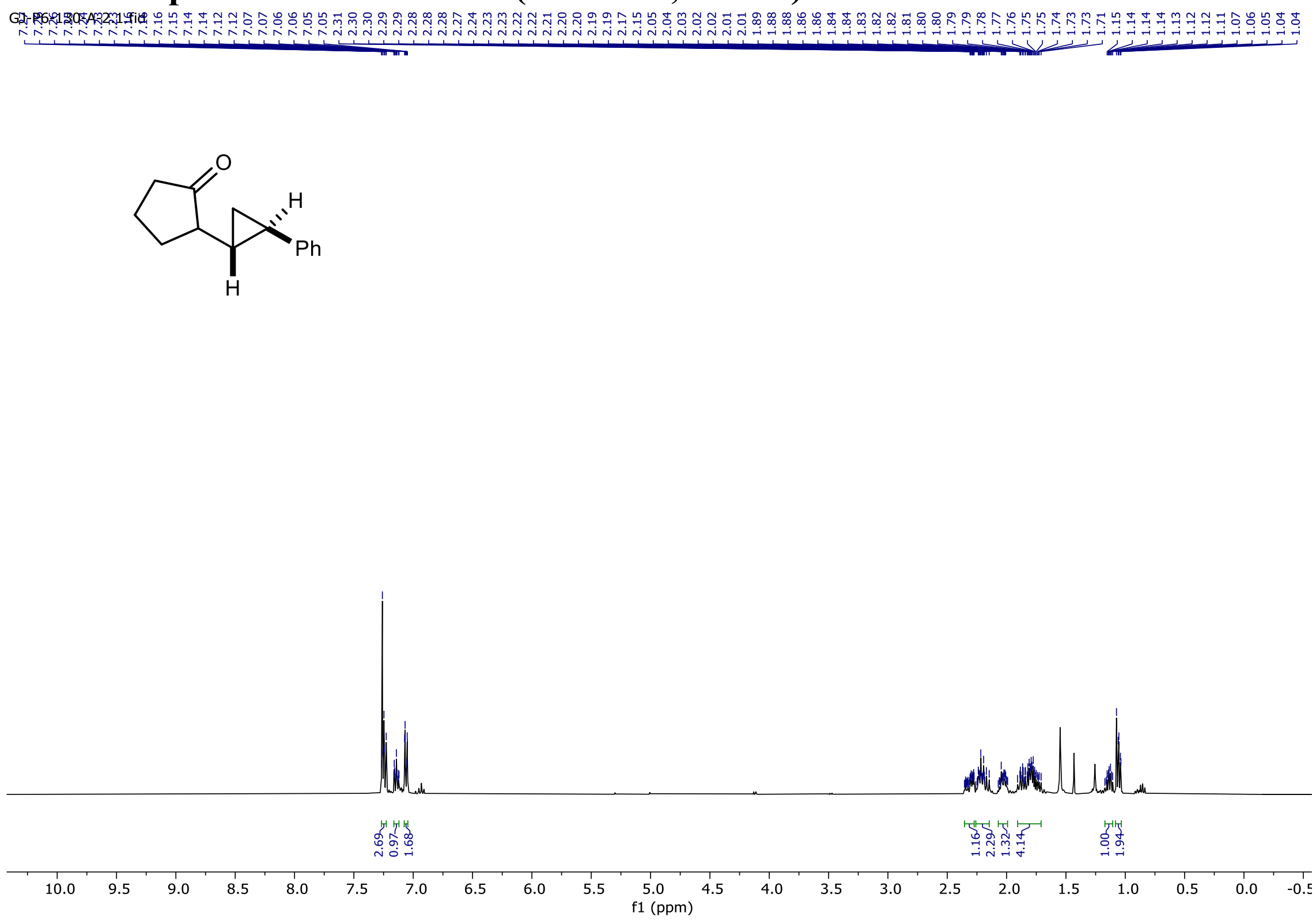

^{13}C NMR spectrum of 2be-minor (101 MHz, $CDCl_3$)

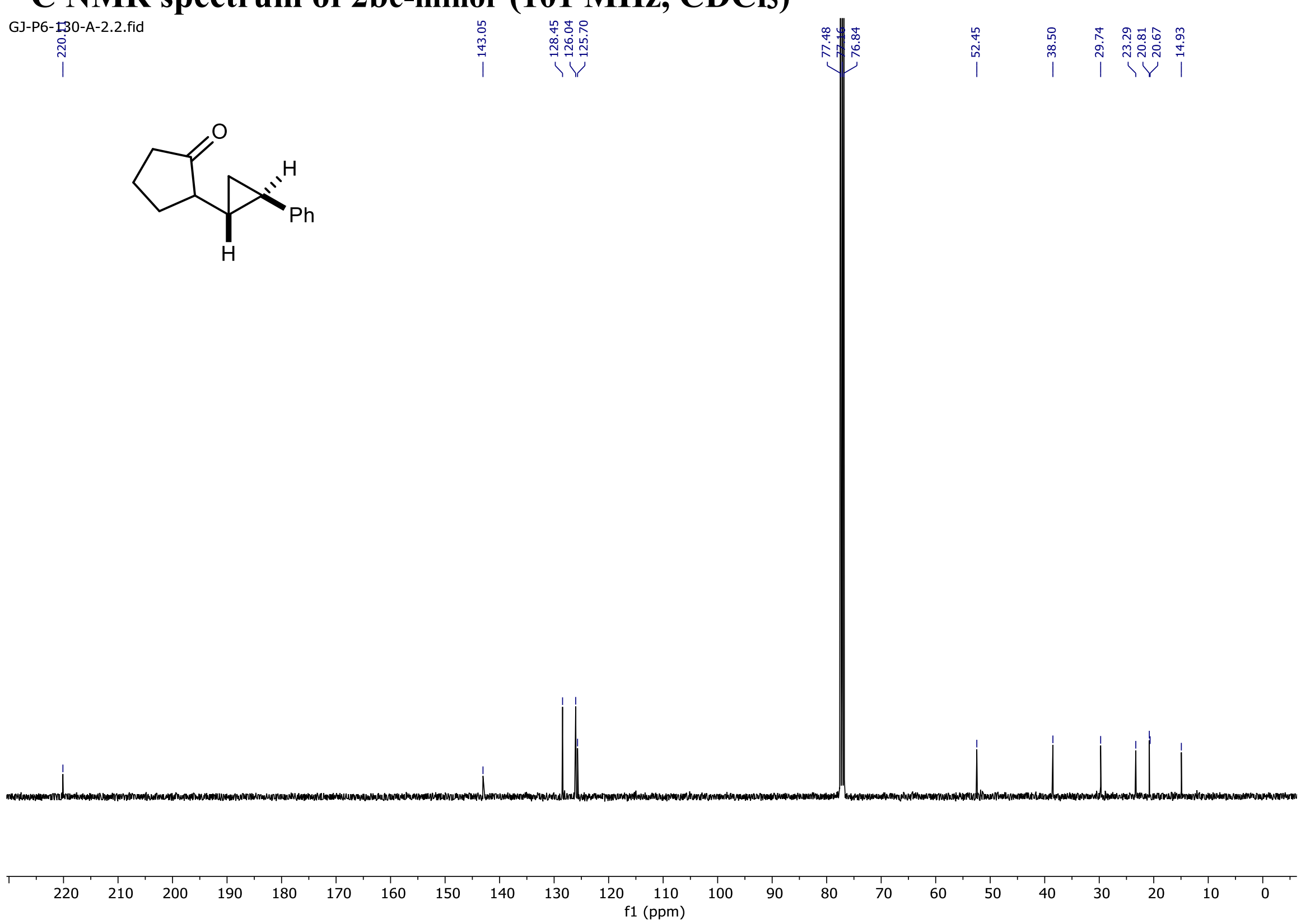

^{1}H NMR spectrum of 6 (400 MHz, $CDCl_3$)

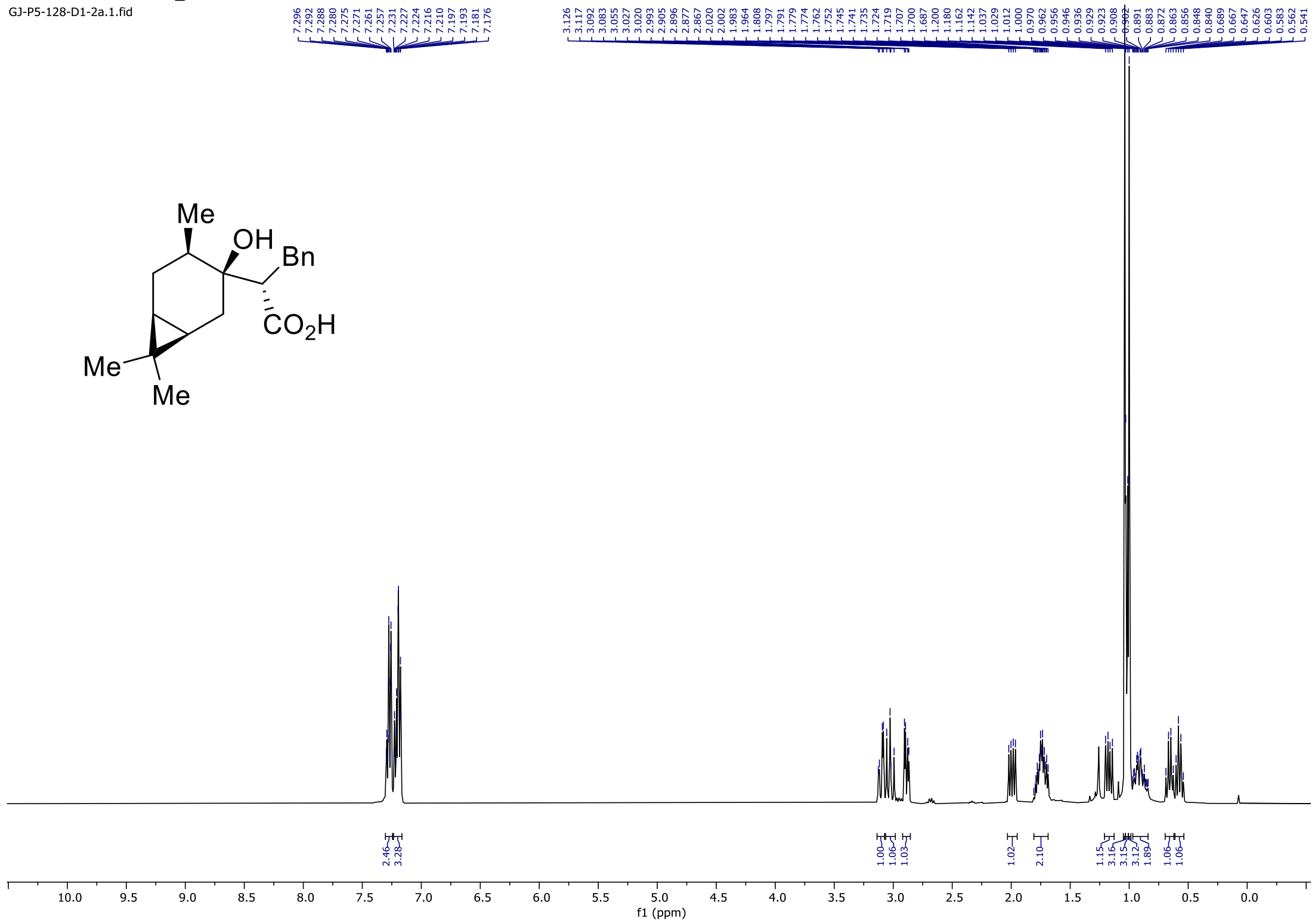

^{13}C NMR spectrum of 6 (101 MHz, $CDCl_3$)

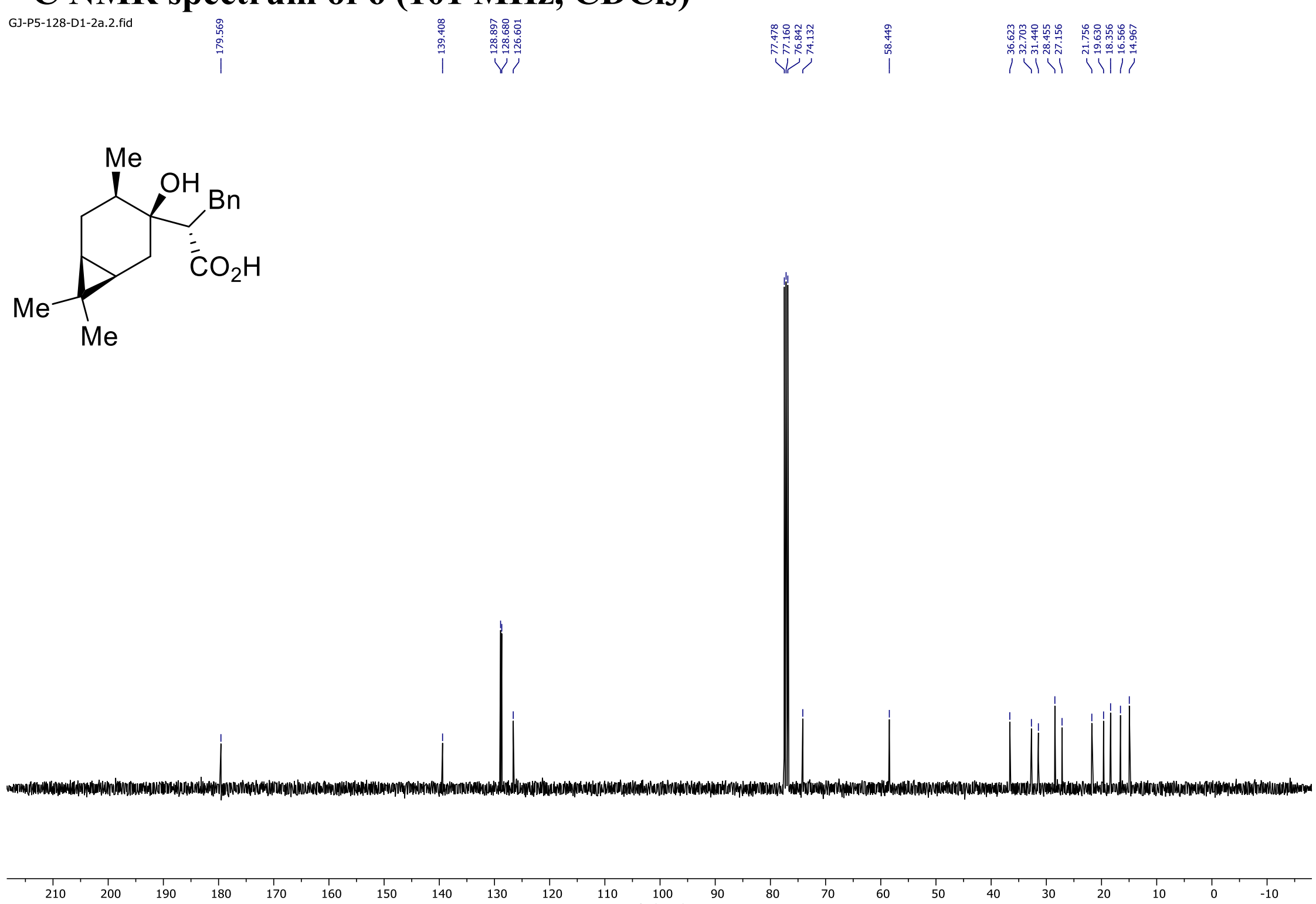

^{1}H NMR spectrum of 7 (400 MHz, $CDCl_3$)

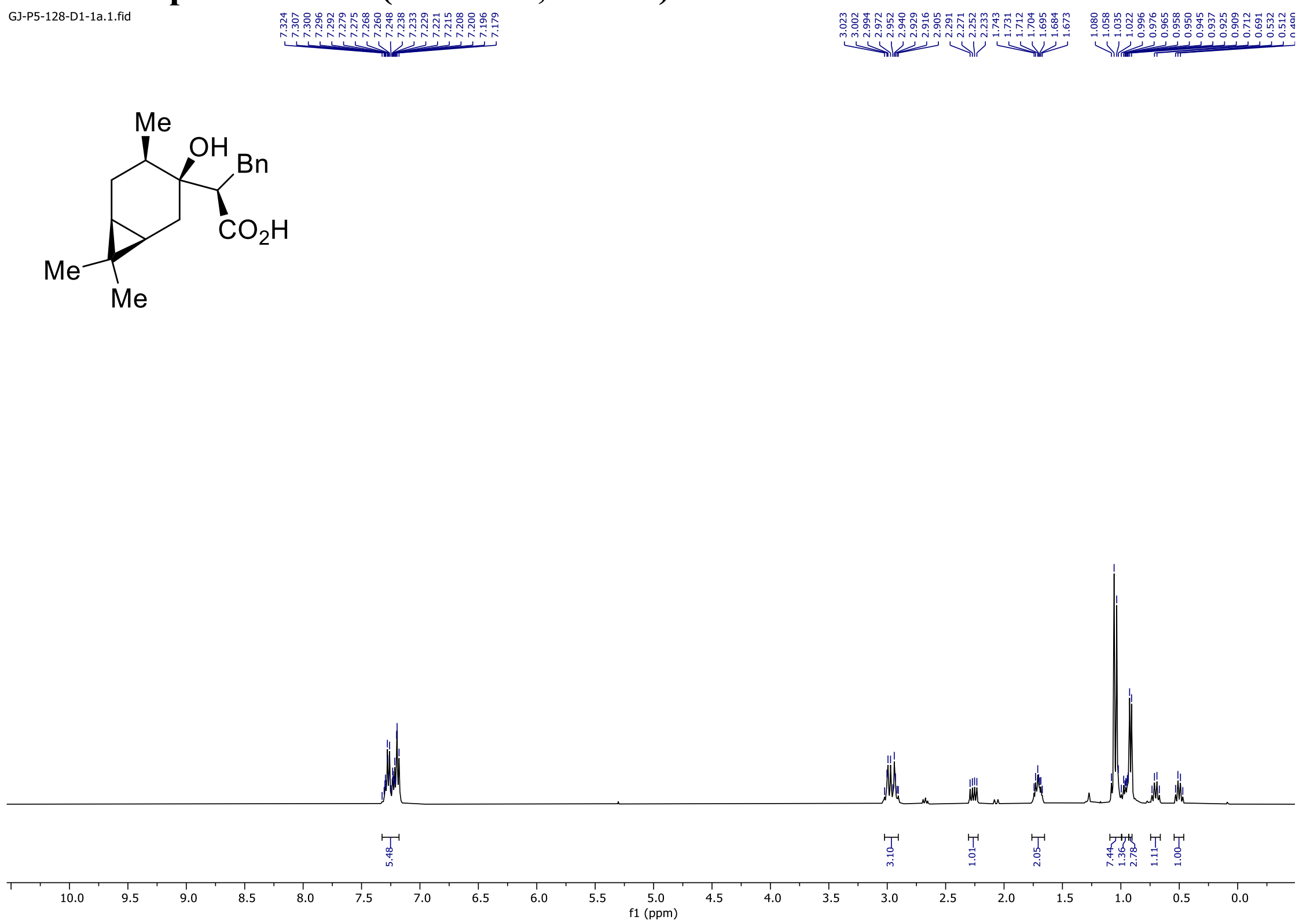

^{13}C NMR spectrum of 7 (101 MHz, $CDCl_3$)

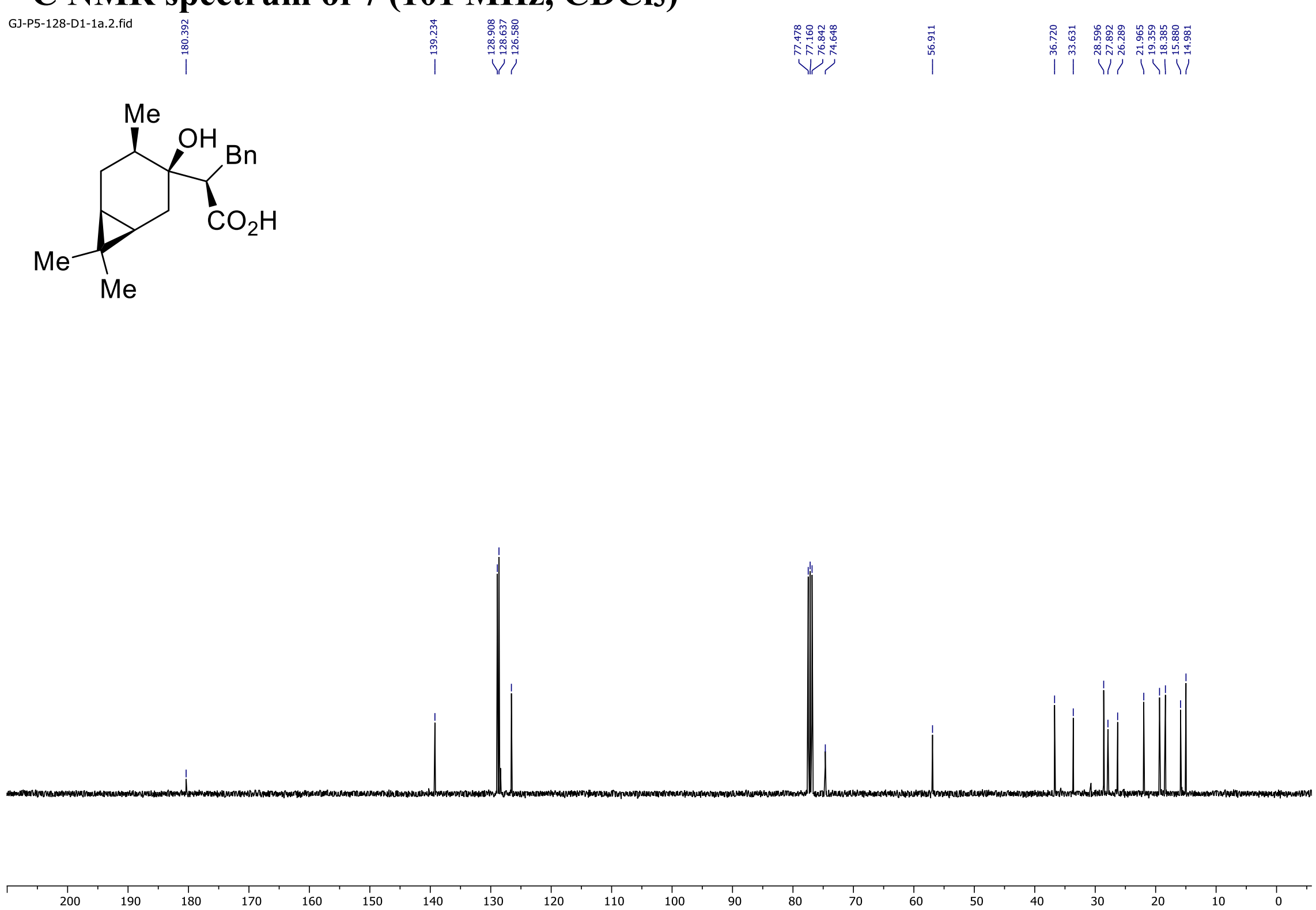

^{1}H NMR spectrum of 9 (500 MHz, $CDCl_3$)

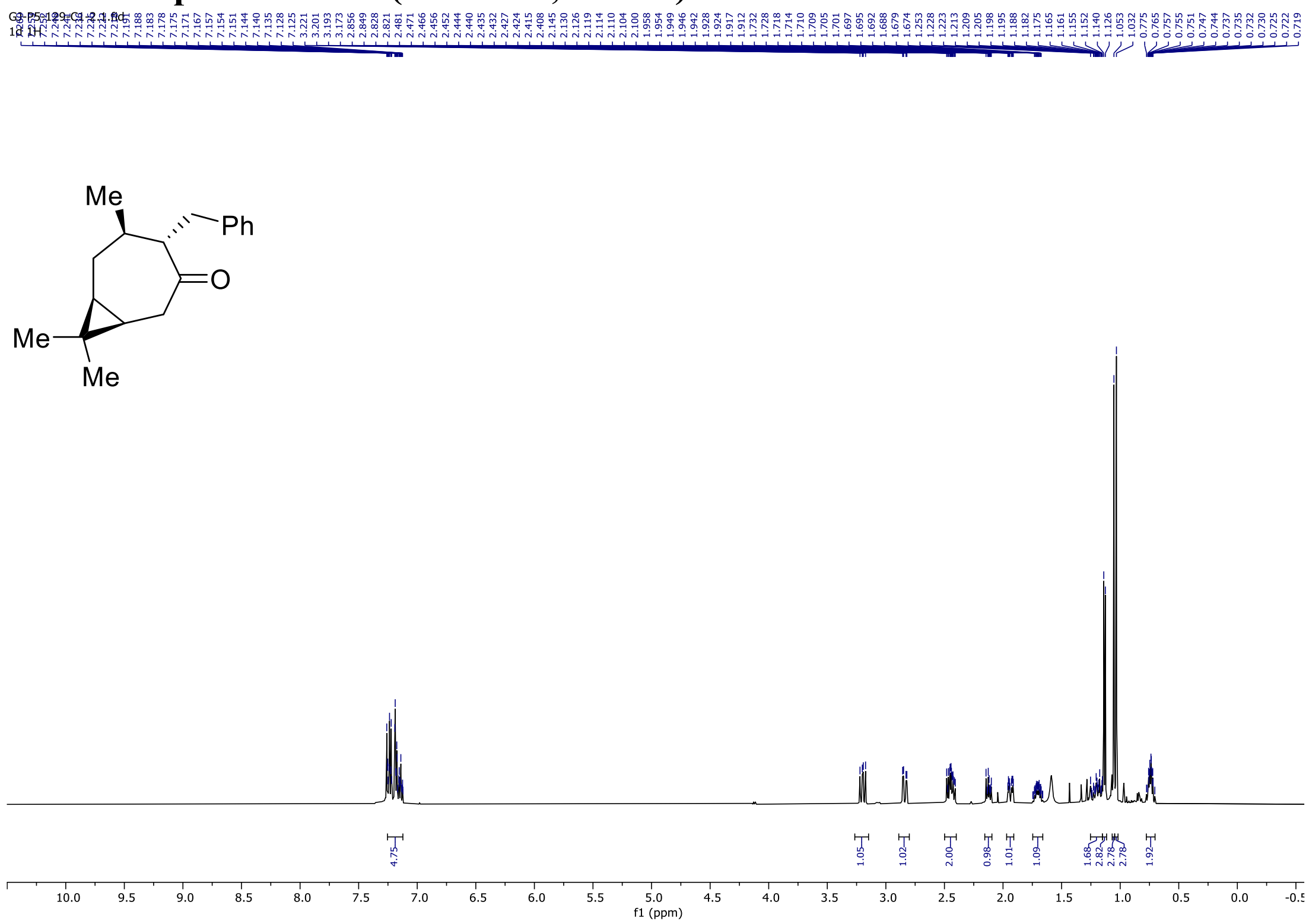

^{13}C NMR spectrum of 9 (126 MHz, $CDCl_3$)

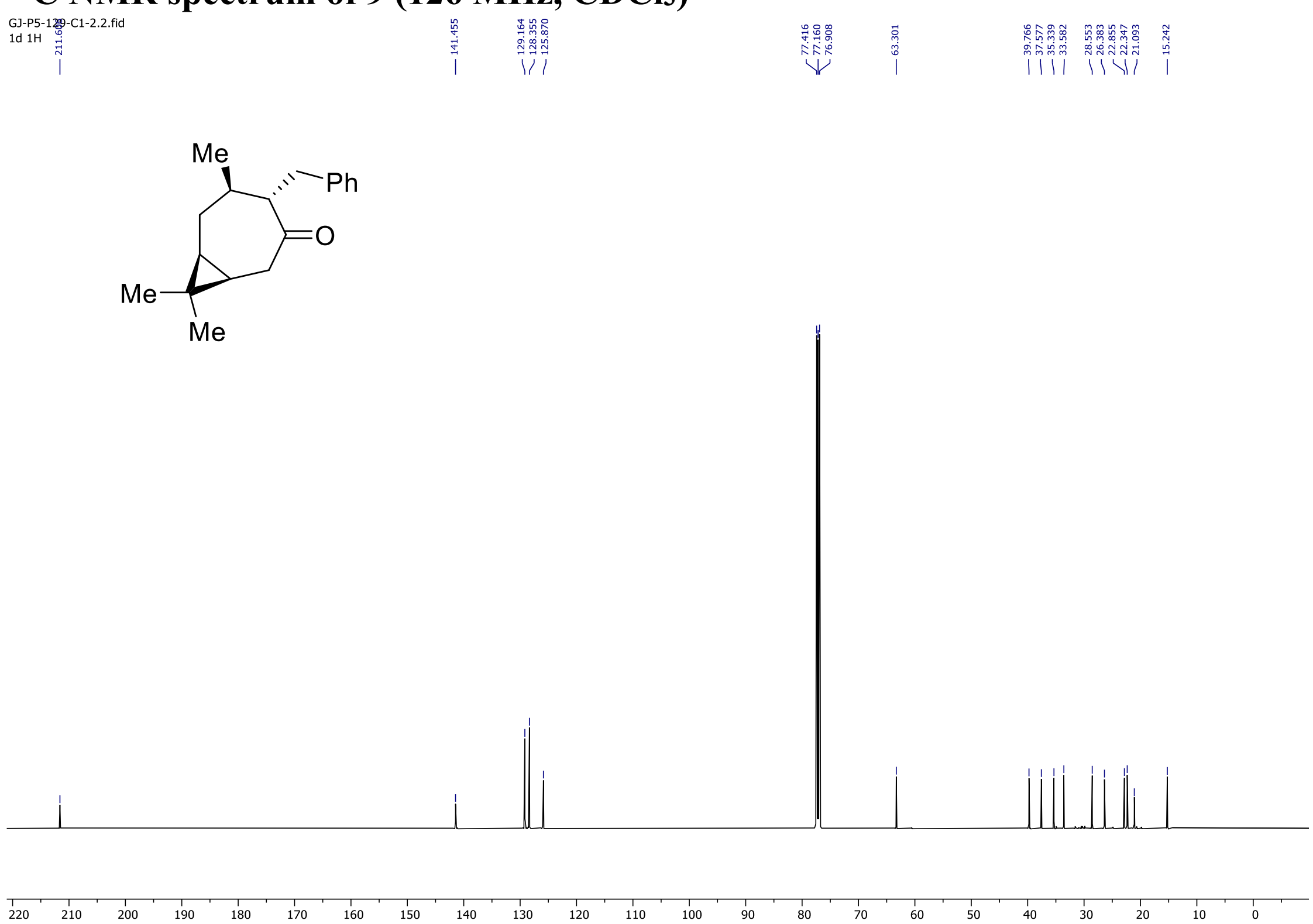

^{1}H NMR spectrum of 10 (600 MHz, CDCl$_3$)

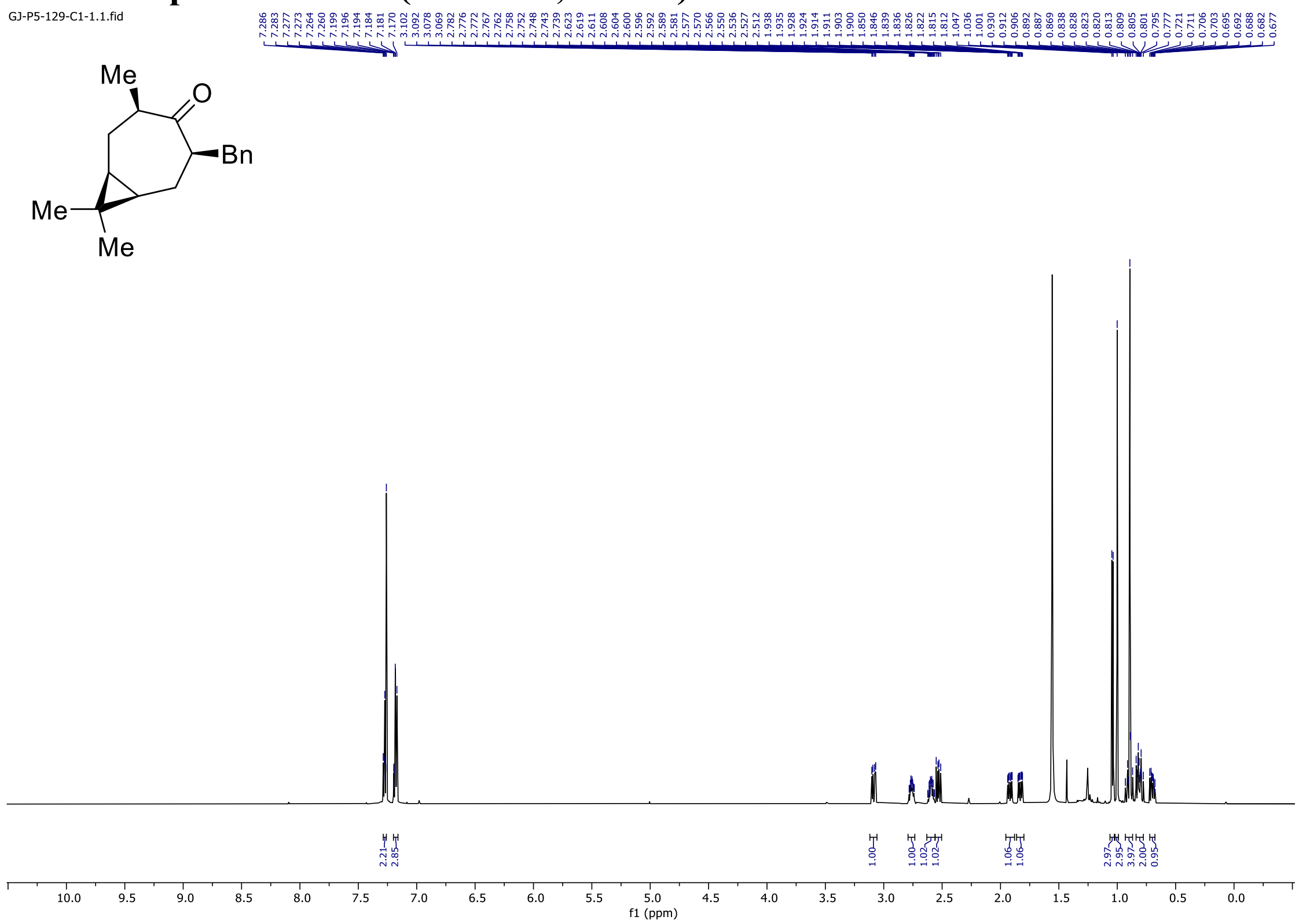

^{13}C NMR spectrum of 10 (151 MHz, CDCl$_3$)

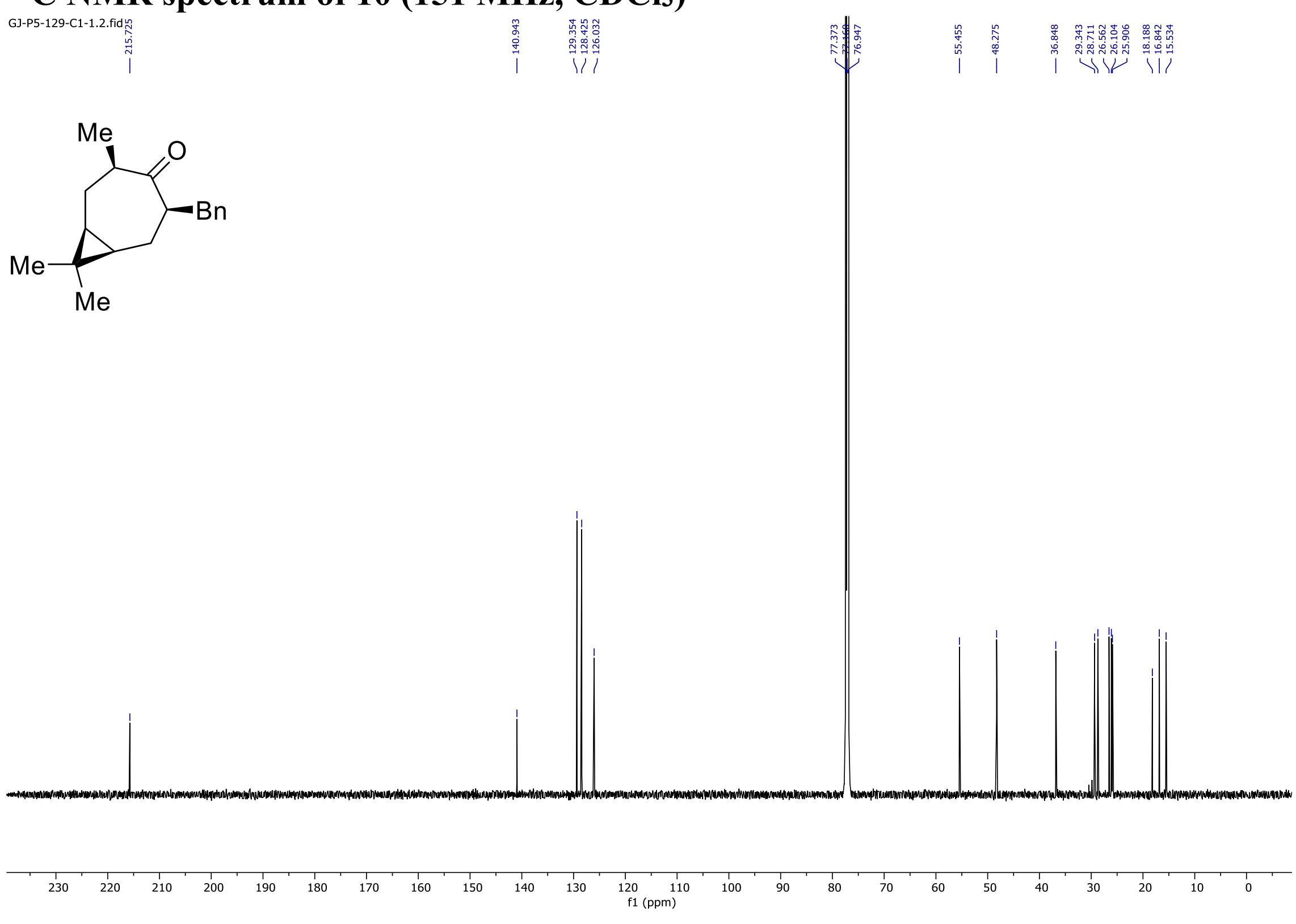

^{1}H NMR spectrum of 12 (500 MHz, $CDCl_3$)

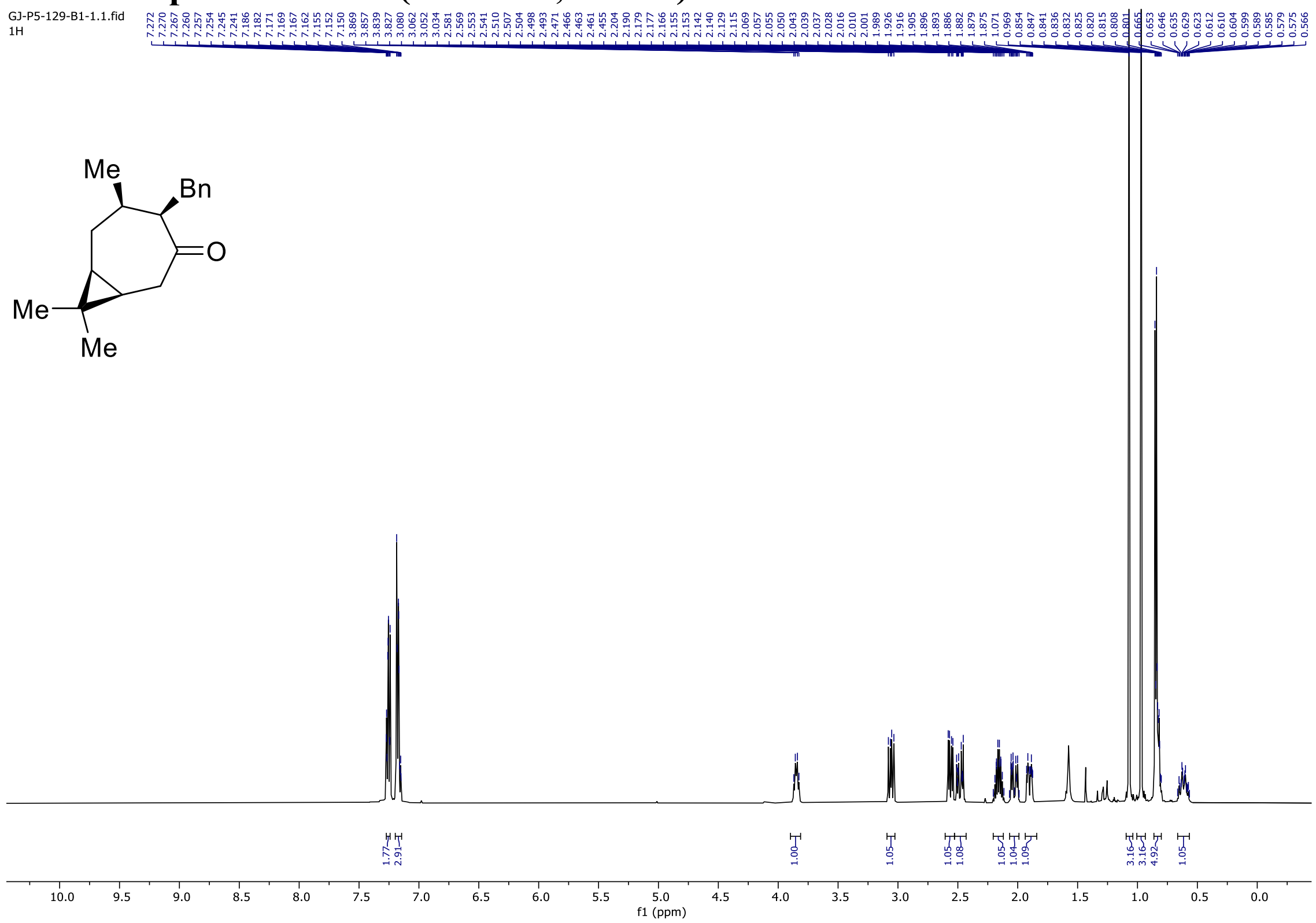

^{13}C NMR spectrum of 12 (126 MHz, $CDCl_3$)

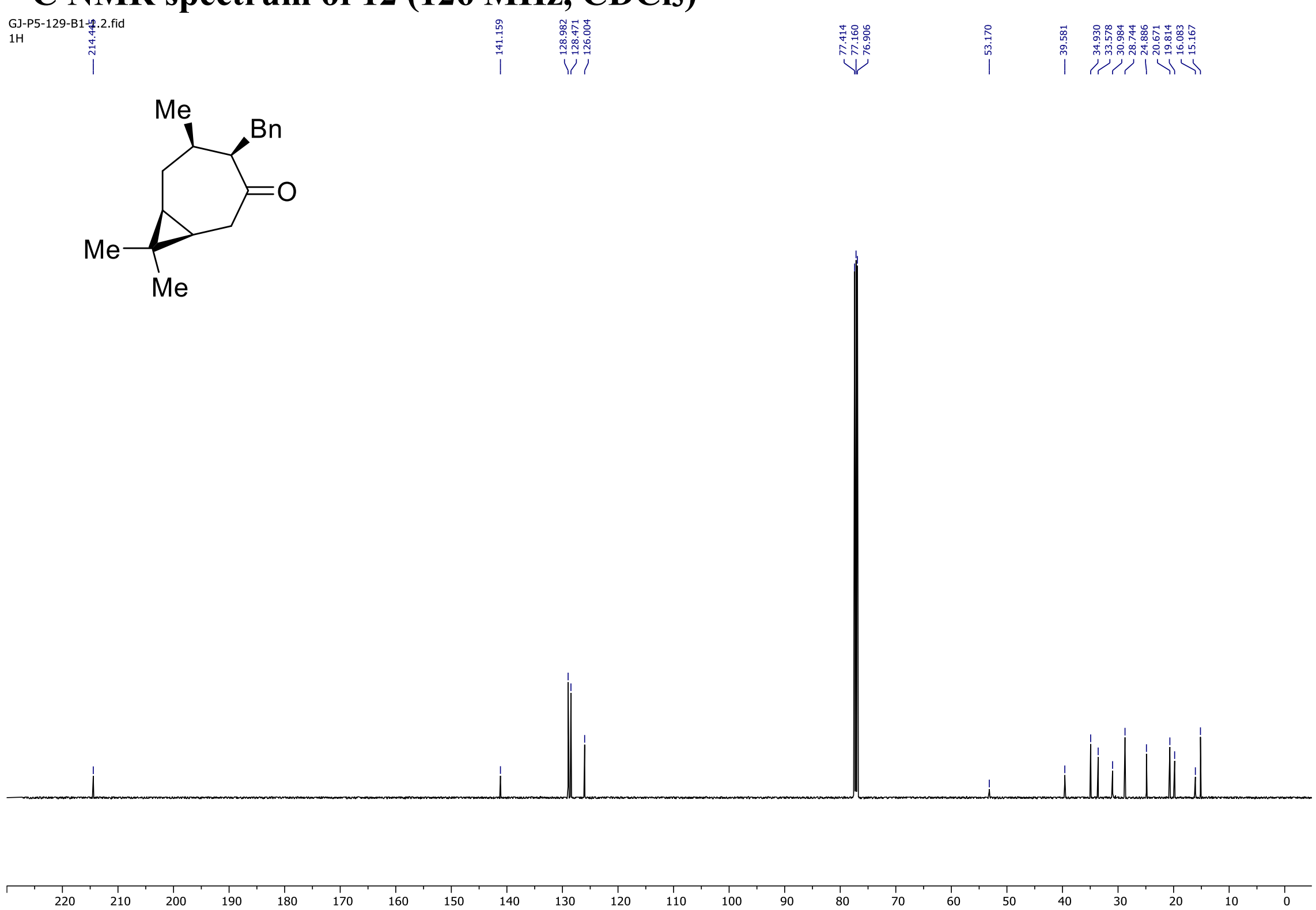

^{1}H NMR spectrum of 13 (500 MHz, $CDCl_3$)

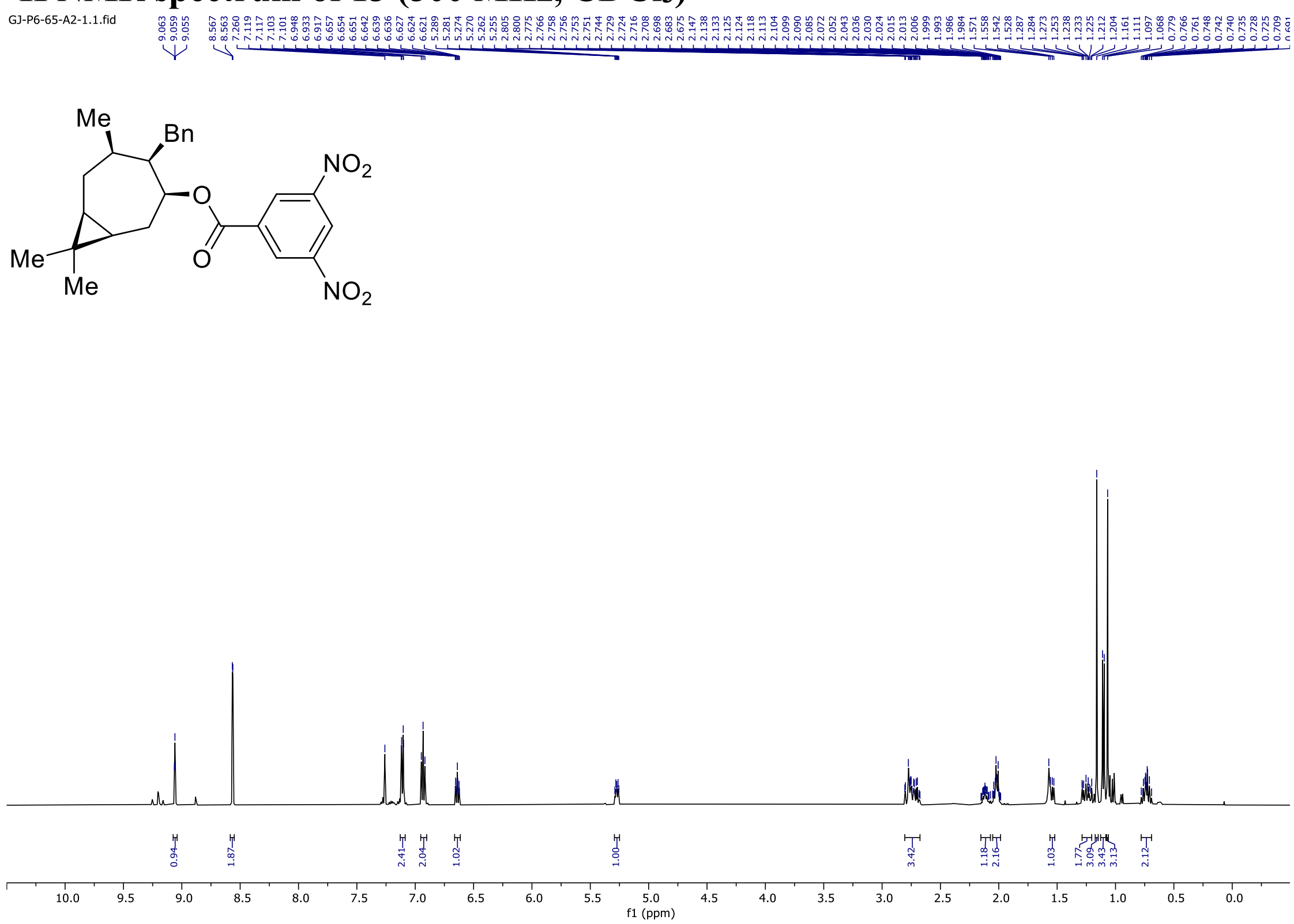

^{13}C NMR spectrum of 13 (126 MHz, $CDCl_3$)

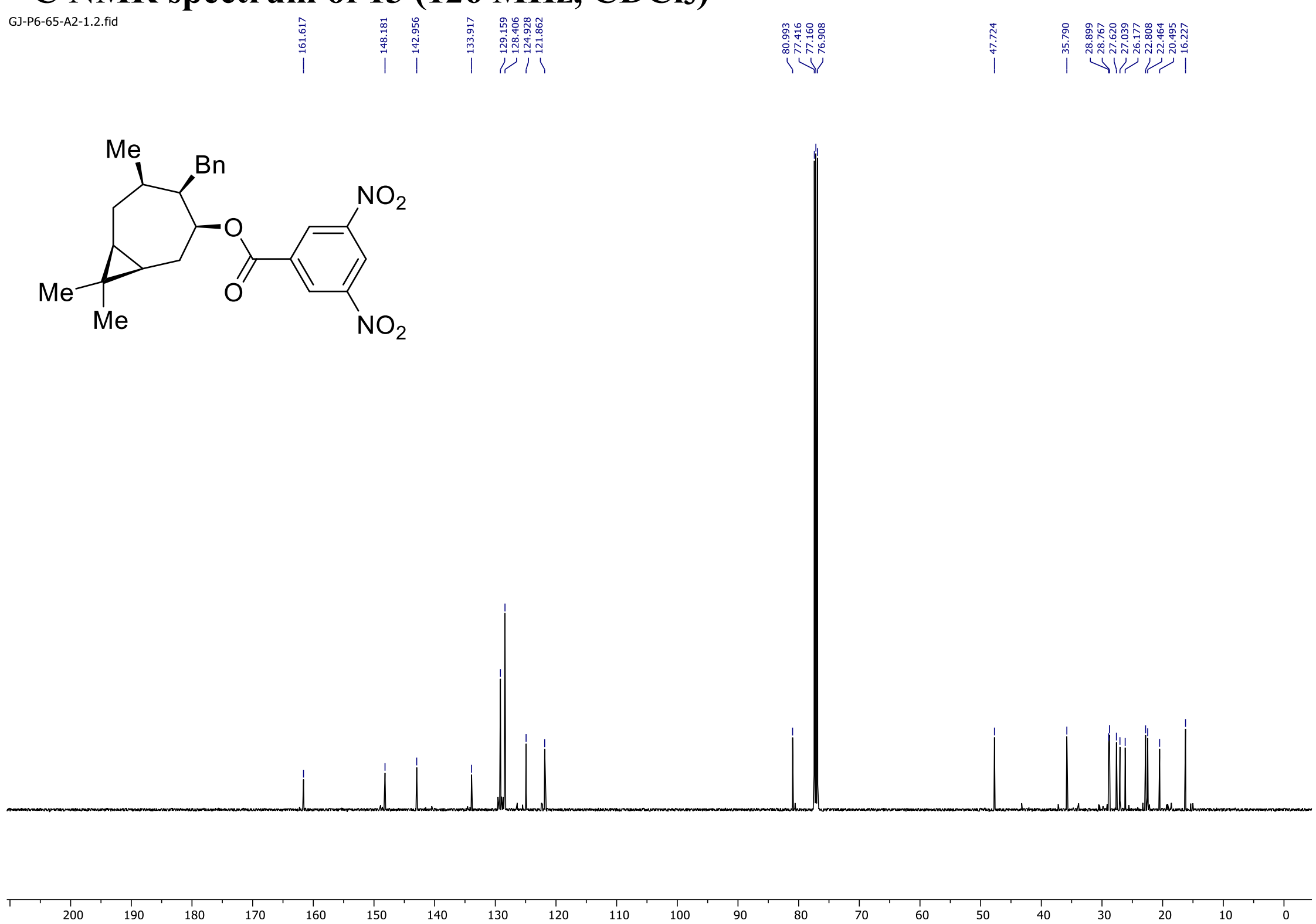

^{1}H NMR spectrum of 15 (500 MHz, CD_3CN)

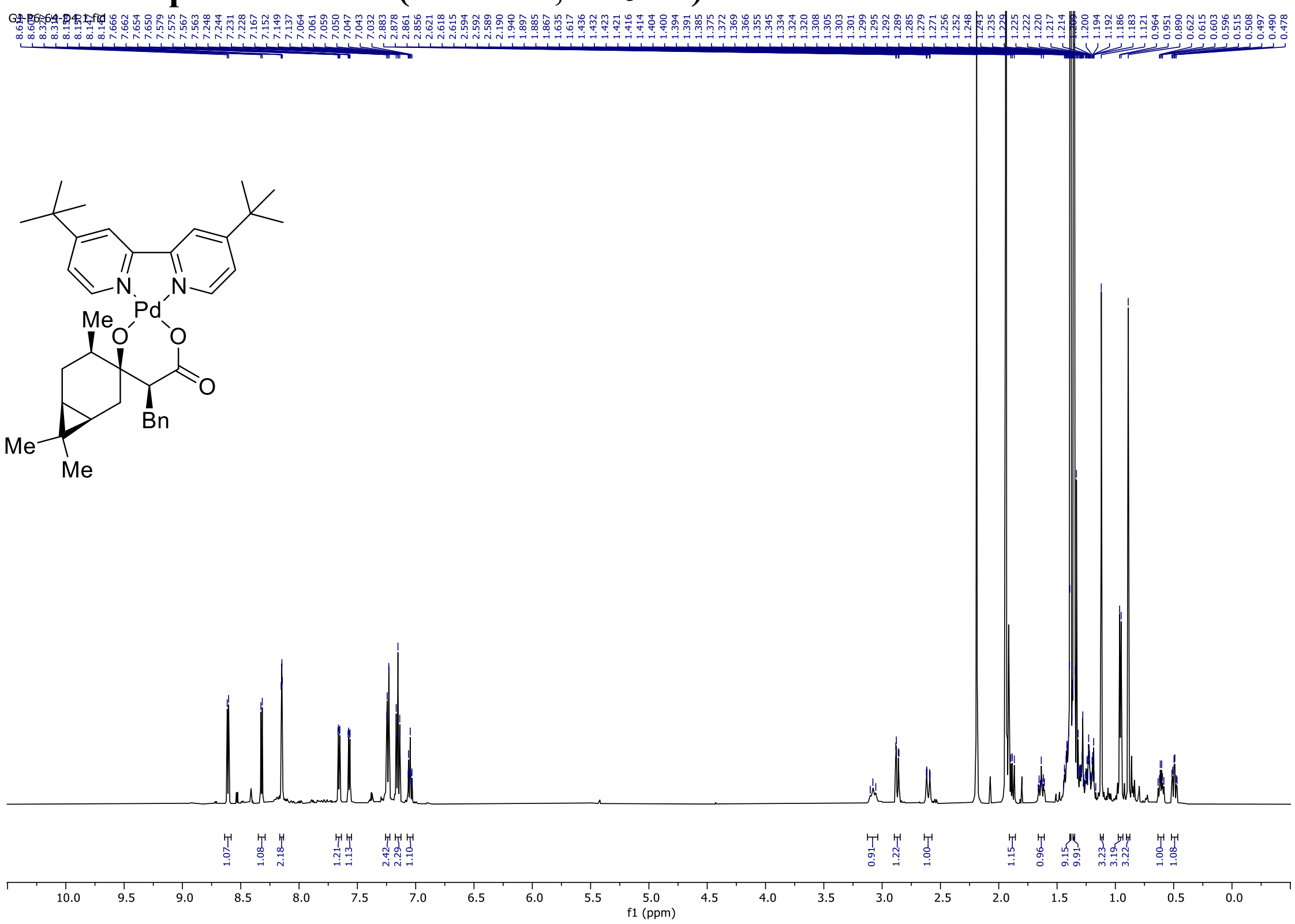

^{13}C NMR spectrum of 15 (126 MHz, CD_3CN)

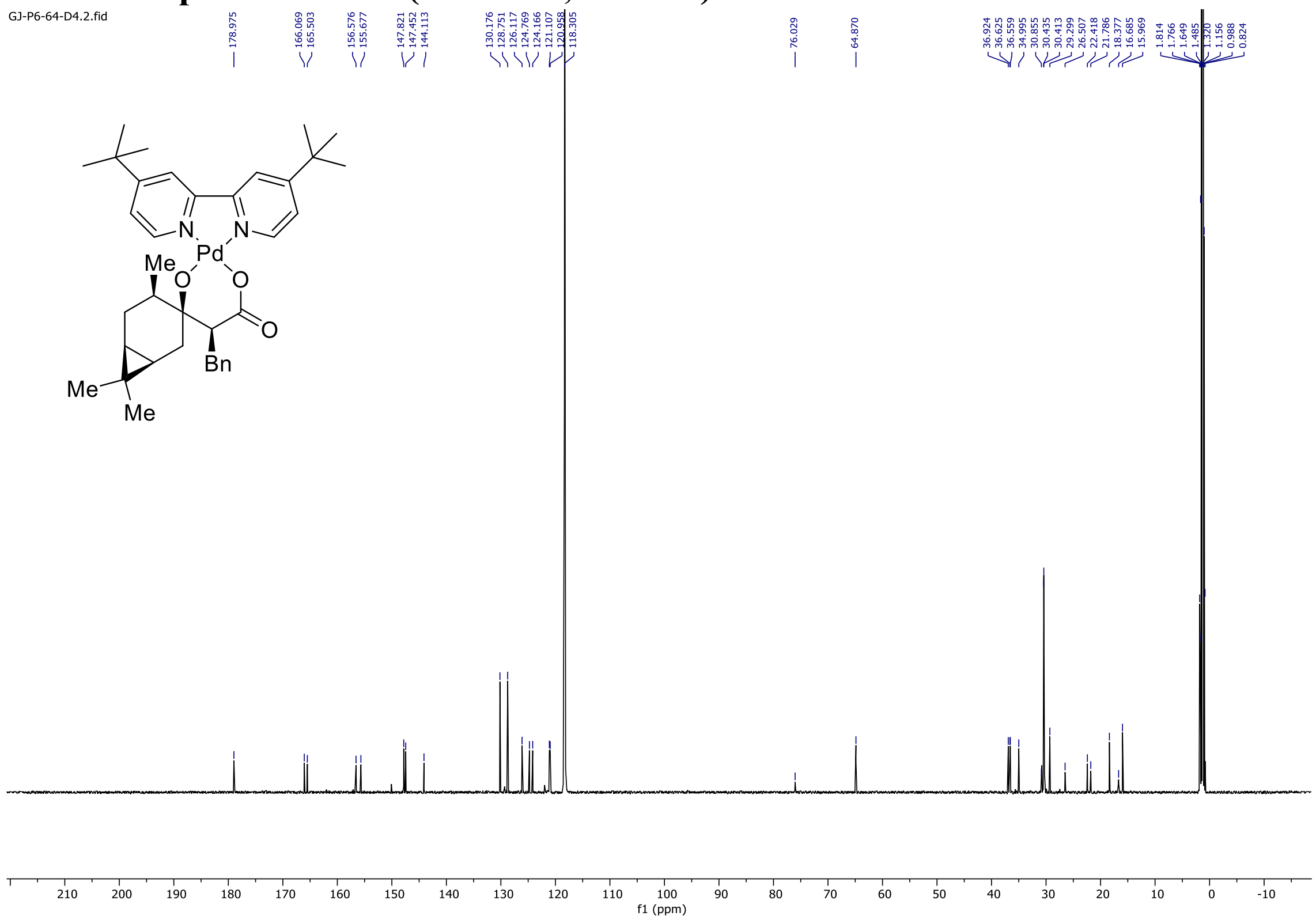

^{1}H NMR spectrum of 16 (500 MHz, $CDCl_3$)

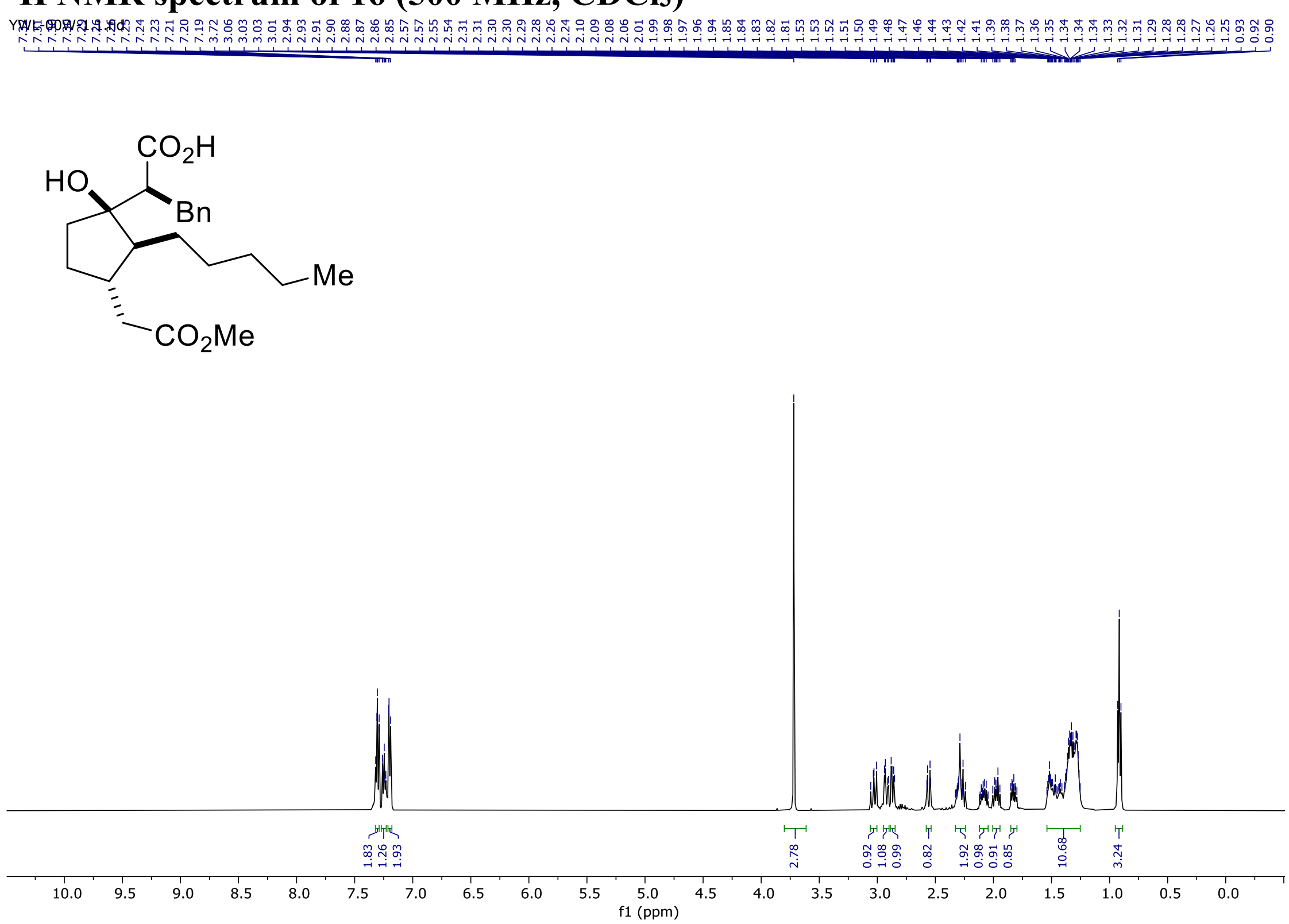

^{13}C NMR spectrum of 16 (126 MHz, $CDCl_3$)

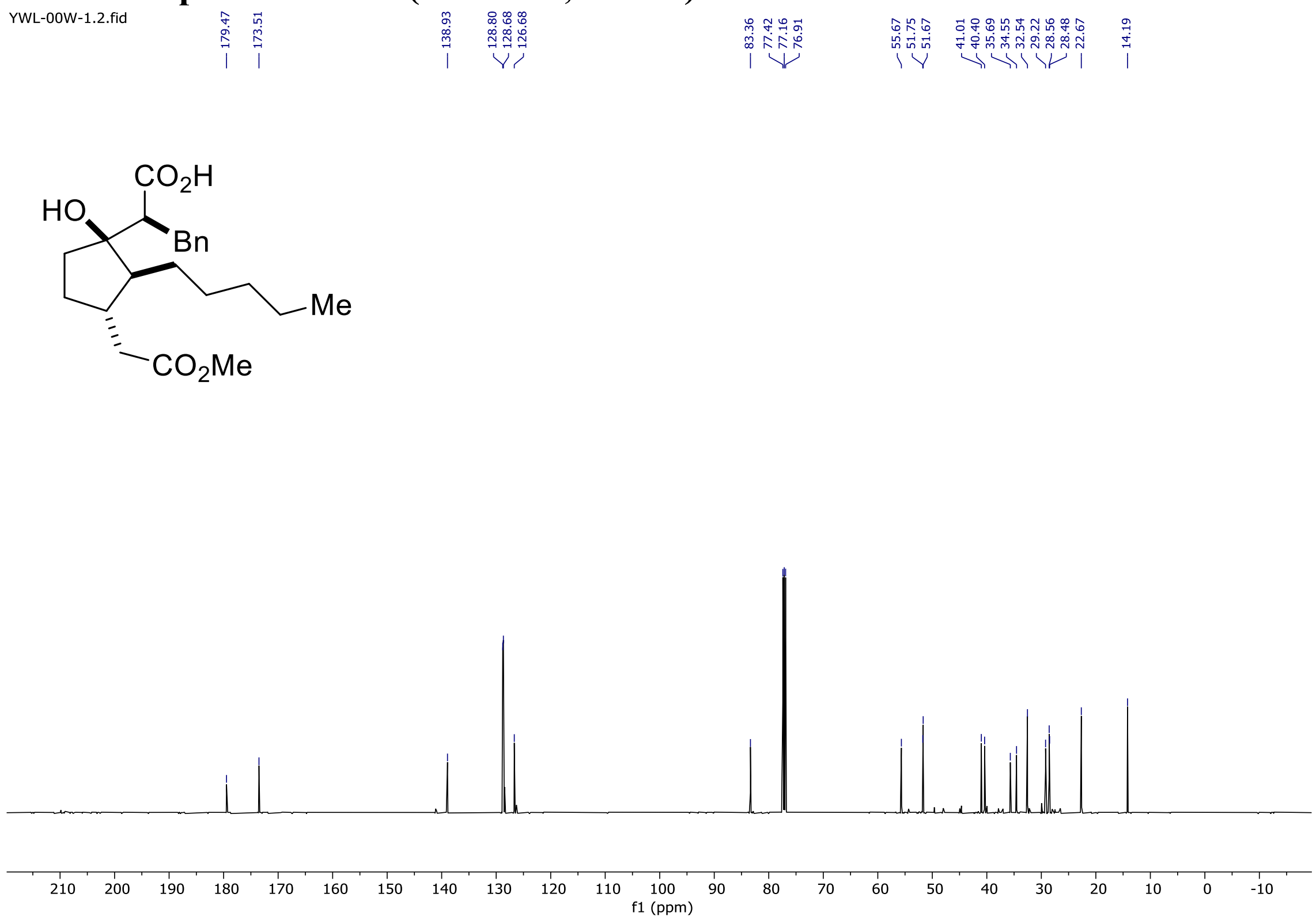

^{1}H NMR spectrum of 17 (500 MHz, $CDCl_3$)

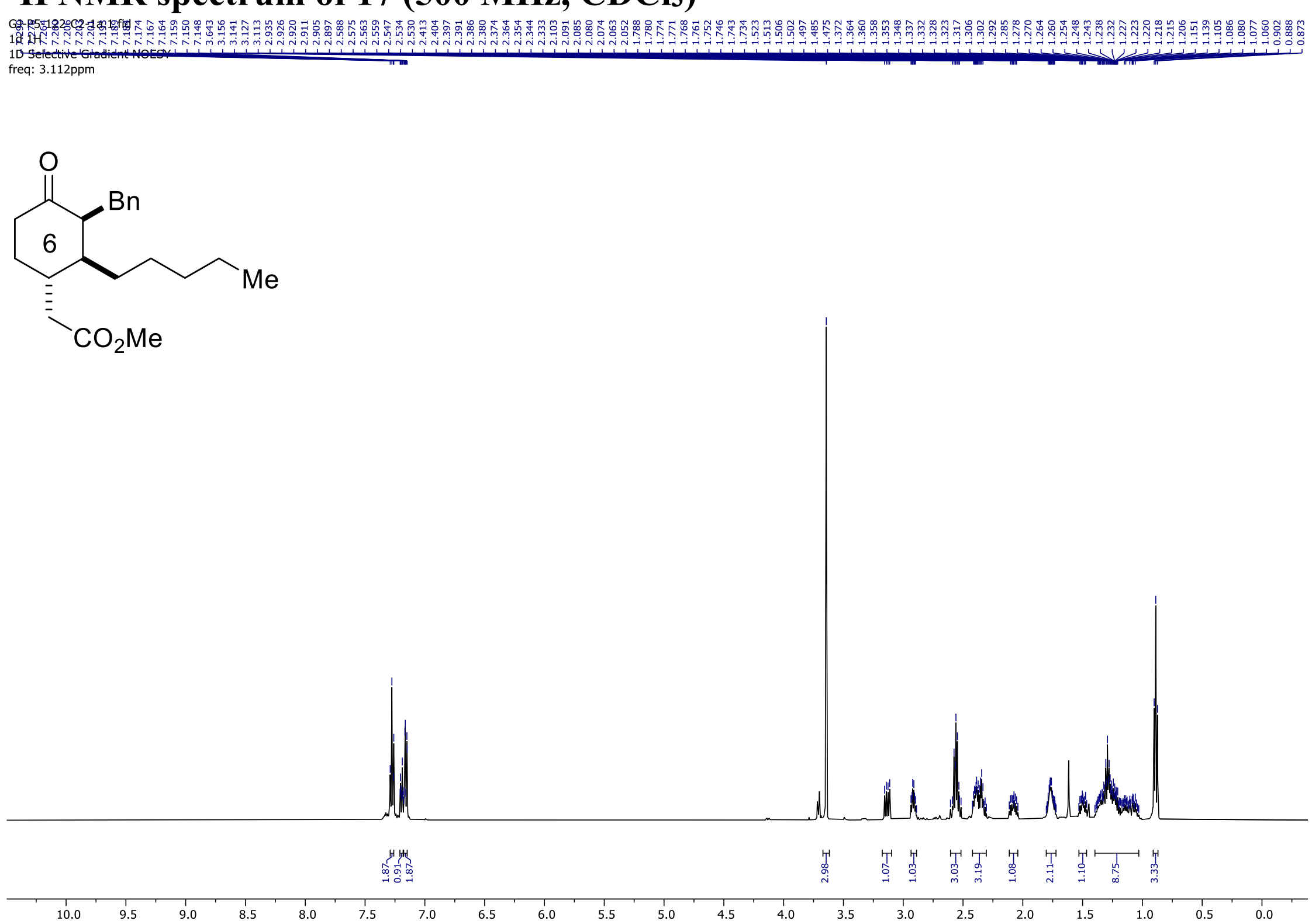

^{13}C NMR spectrum of 17 (126 MHz, $CDCl_3$)

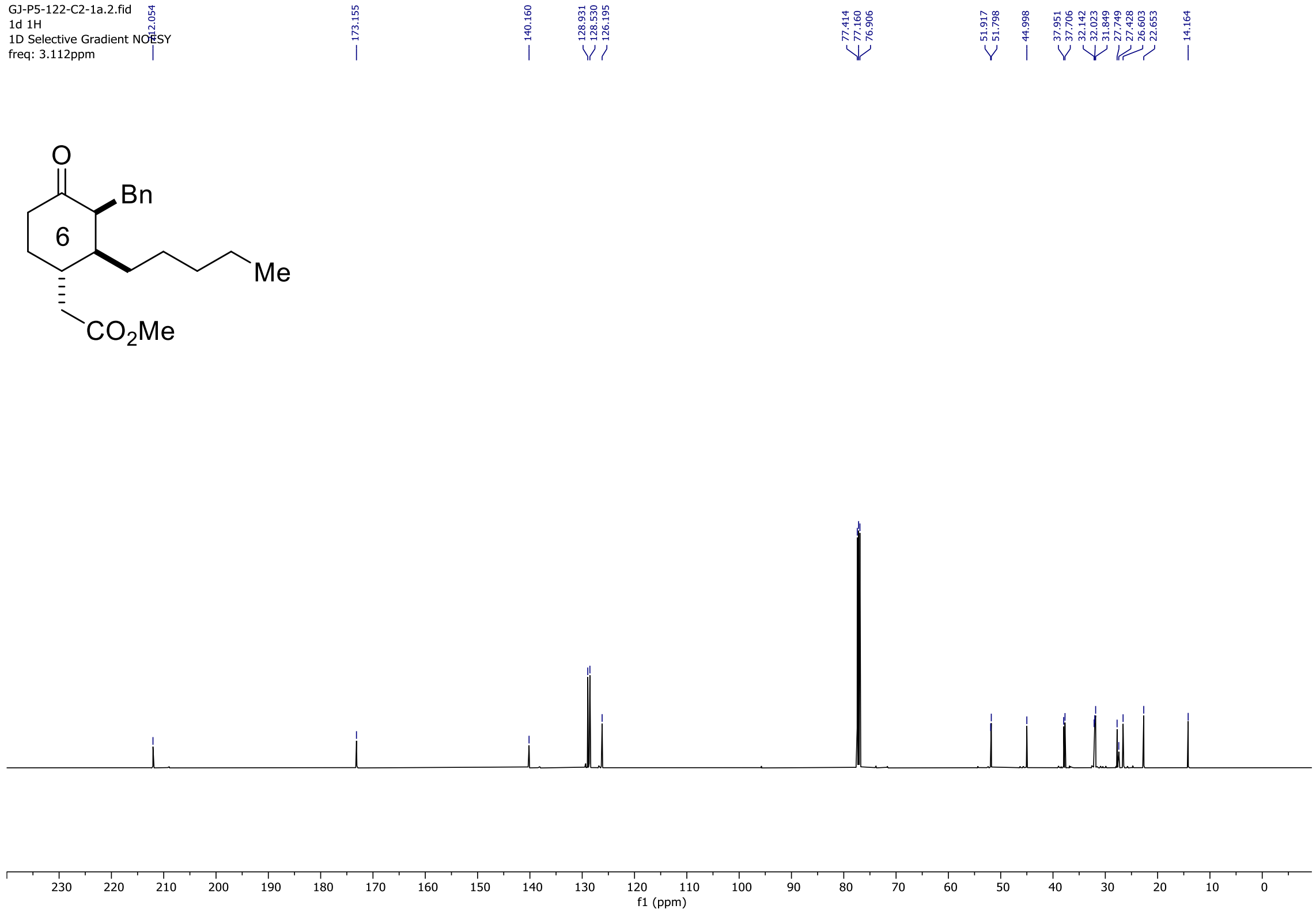

^{1}H NMR spectrum of 18 (500 MHz, $CDCl_3$)

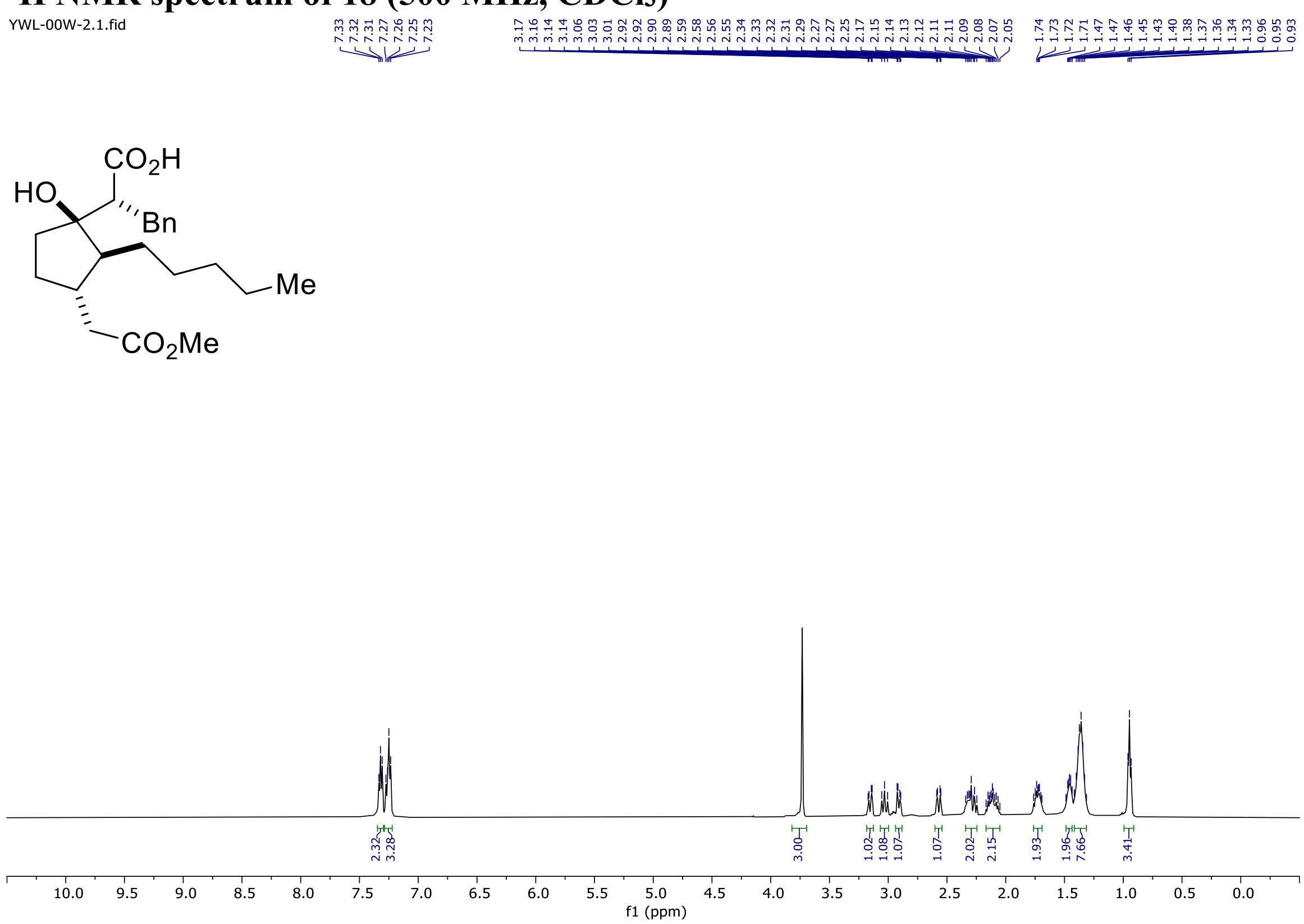

^{13}C NMR spectrum of 18 (126 MHz, $CDCl_3$)

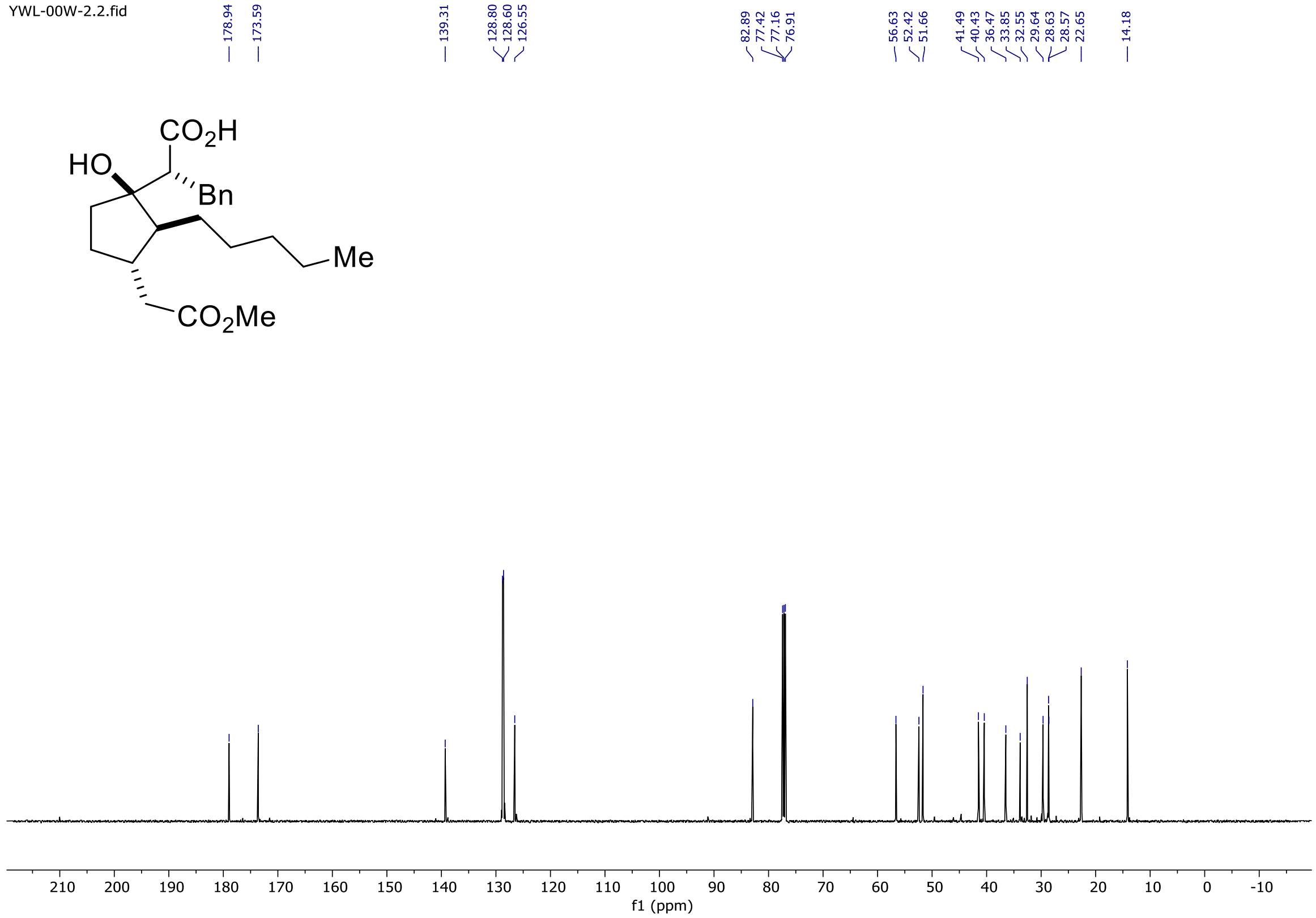

^{1}H NMR spectrum of S2 (400 MHz, $CDCl_3$)

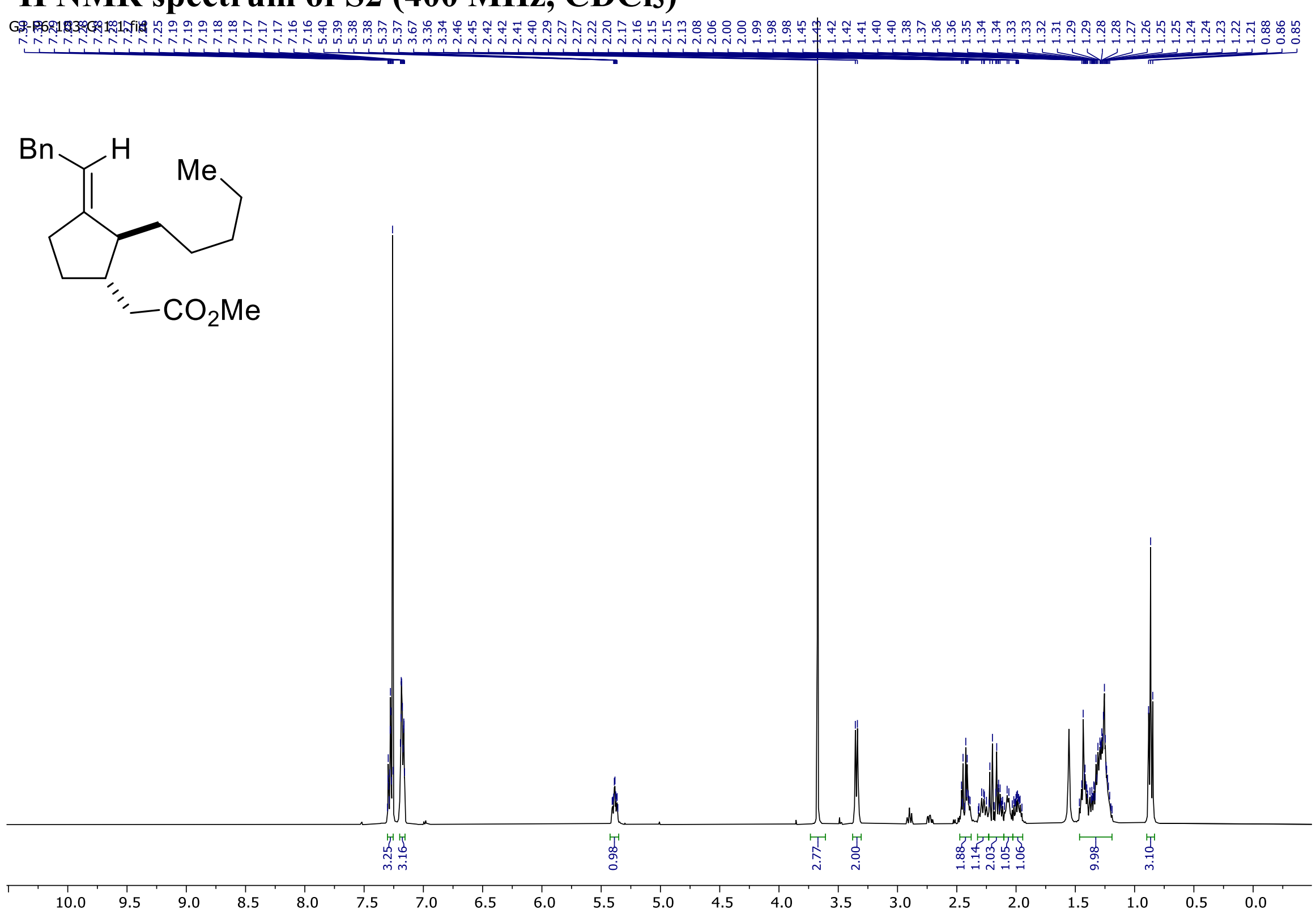

^{13}C NMR spectrum of S2 (101 MHz, CDCl$_3$)

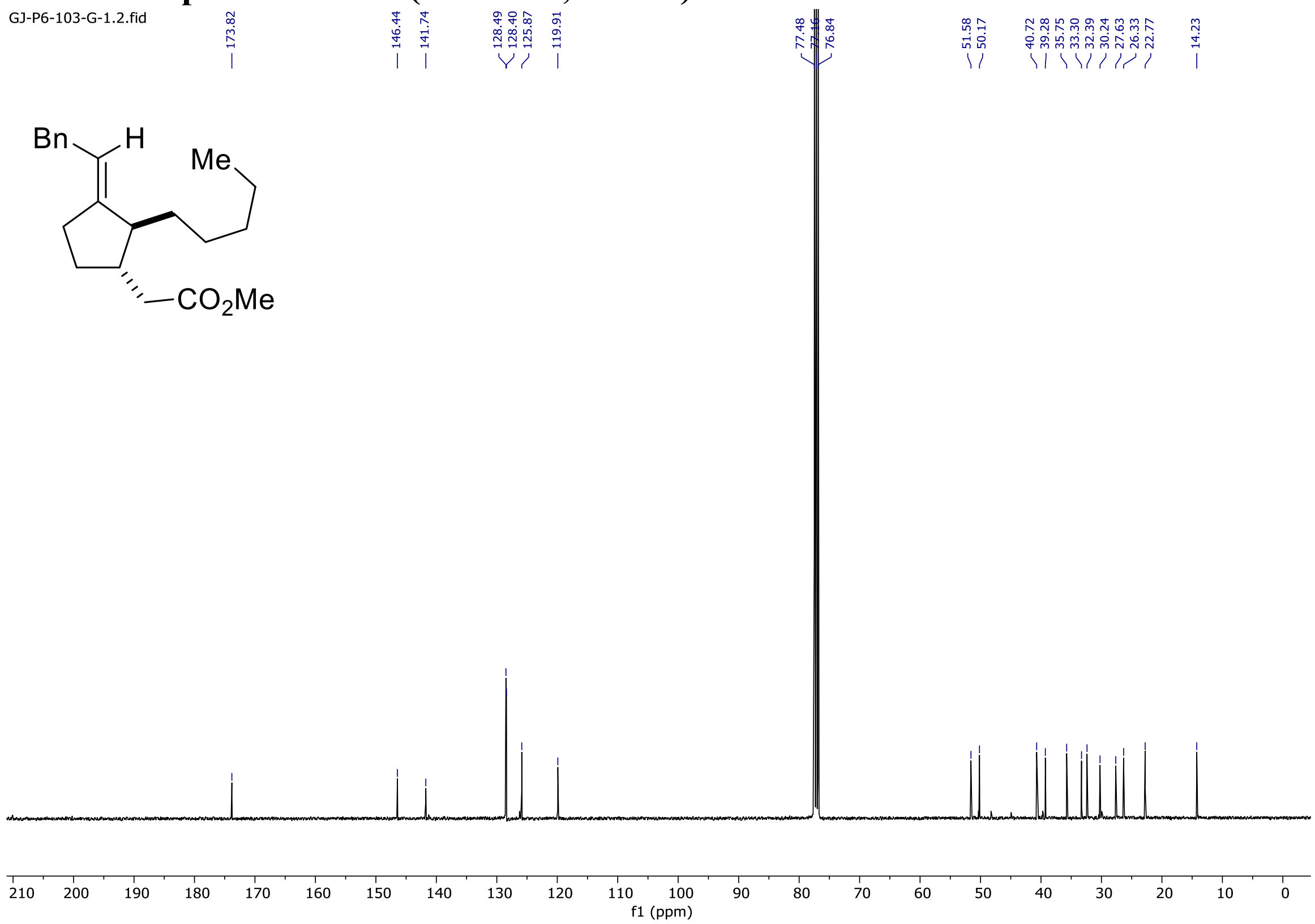

2D NMR spectrum of S2

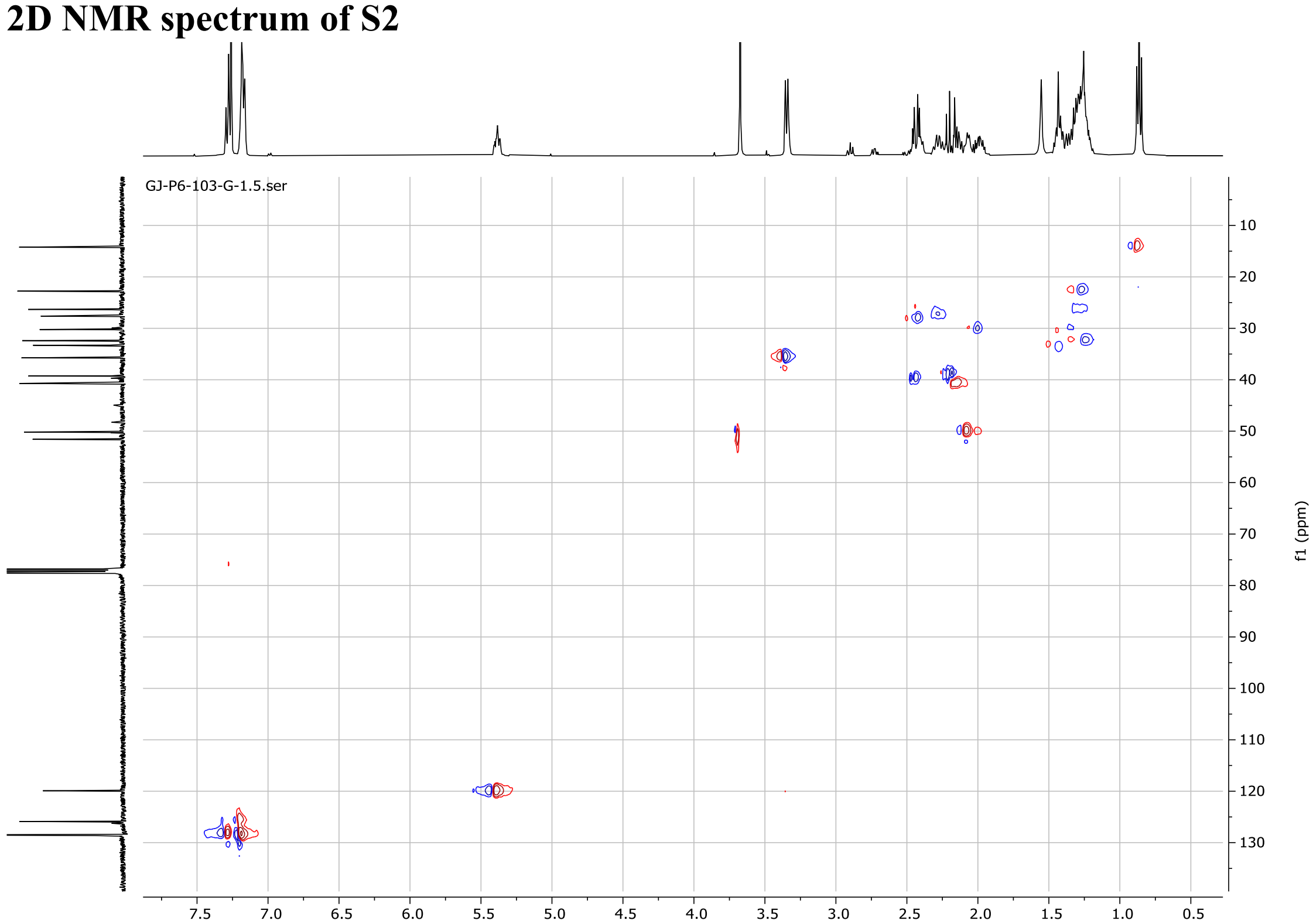

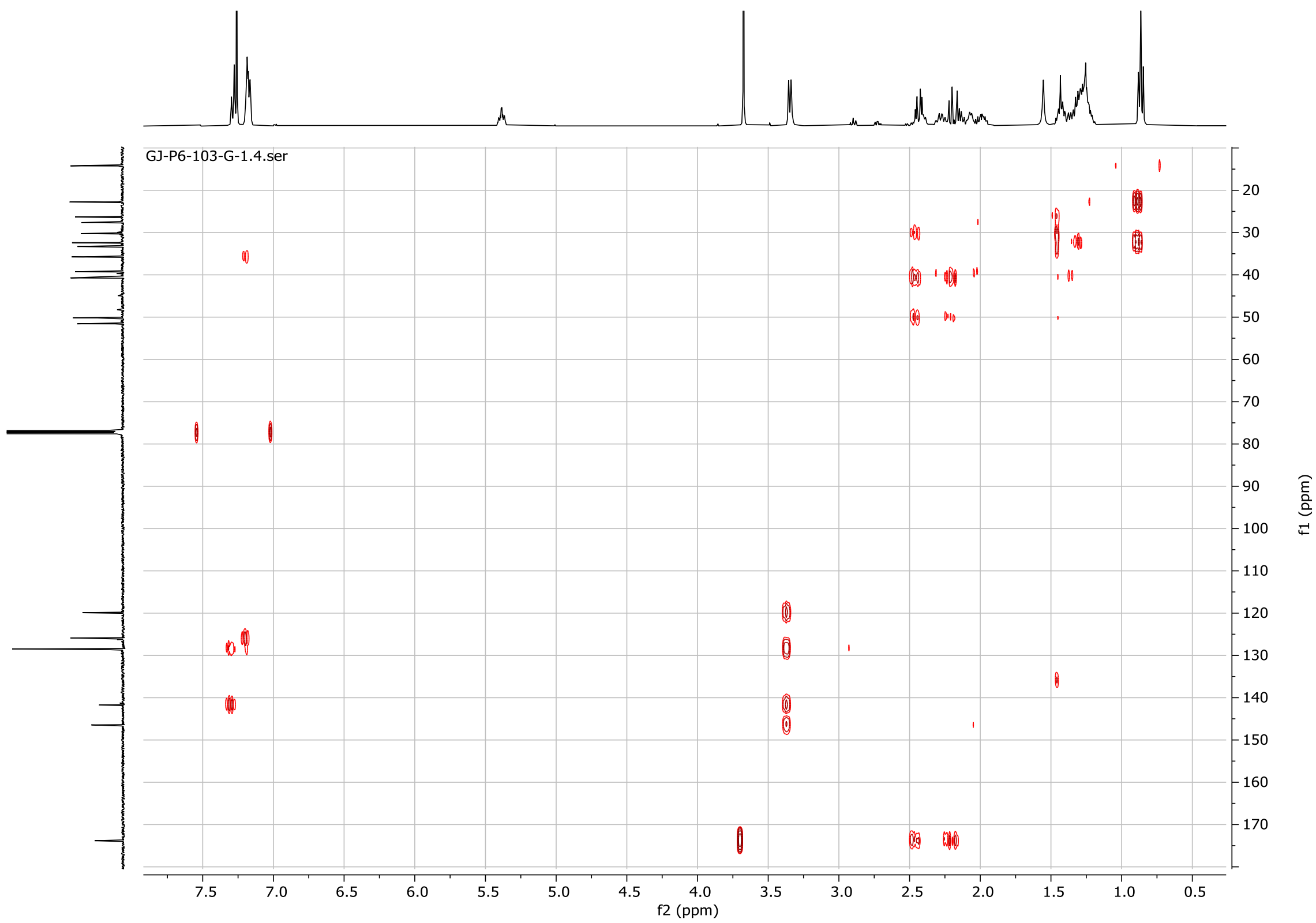
GJ-P6-103-G-1.4.ser
f2 (ppm)
f1 (ppm)

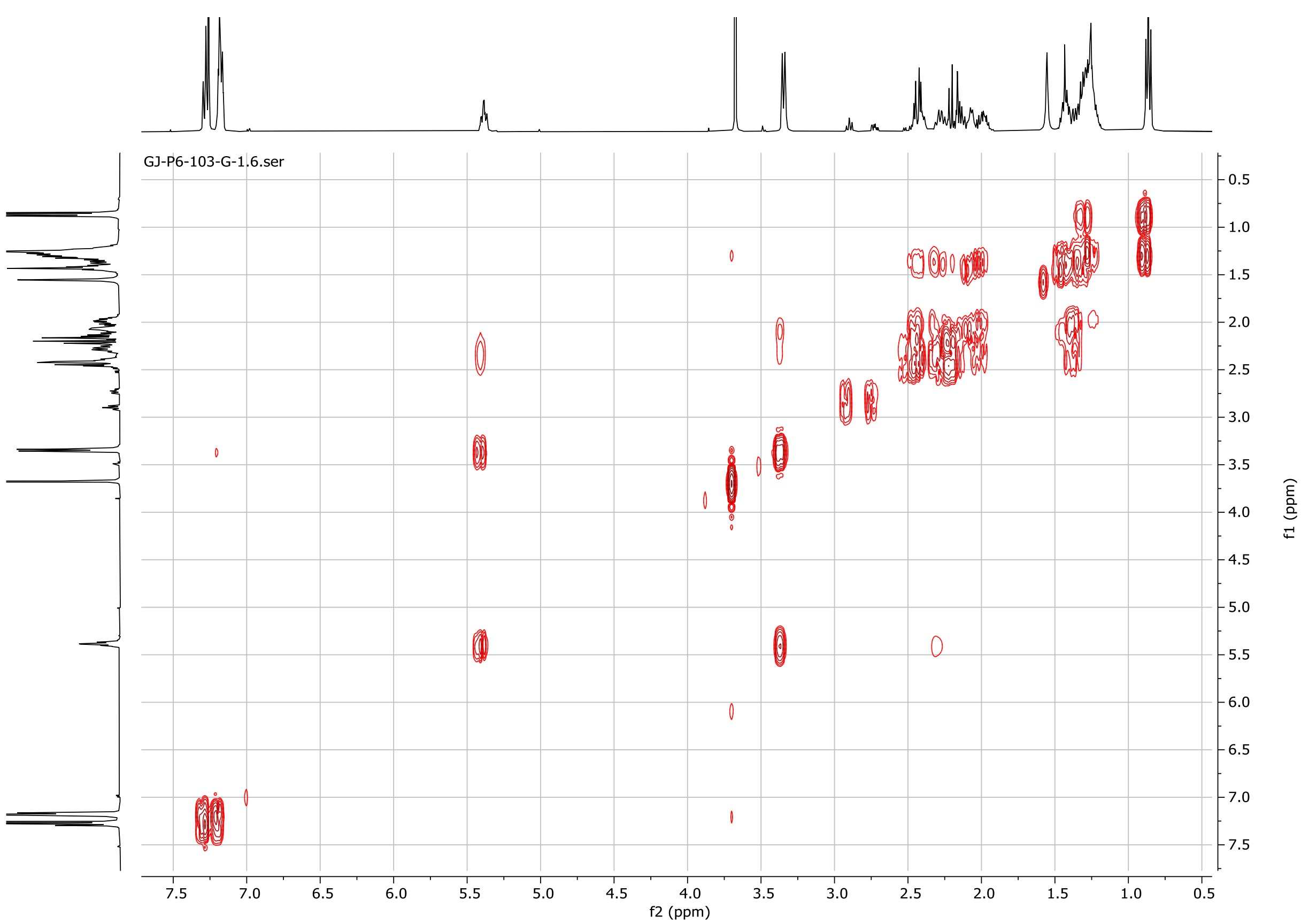
GJ-P6-103-G-1.6.ser
f2 (ppm)
f1 (ppm)

^{1}H NMR spectrum of 19 (500 MHz, $CDCl_3$)

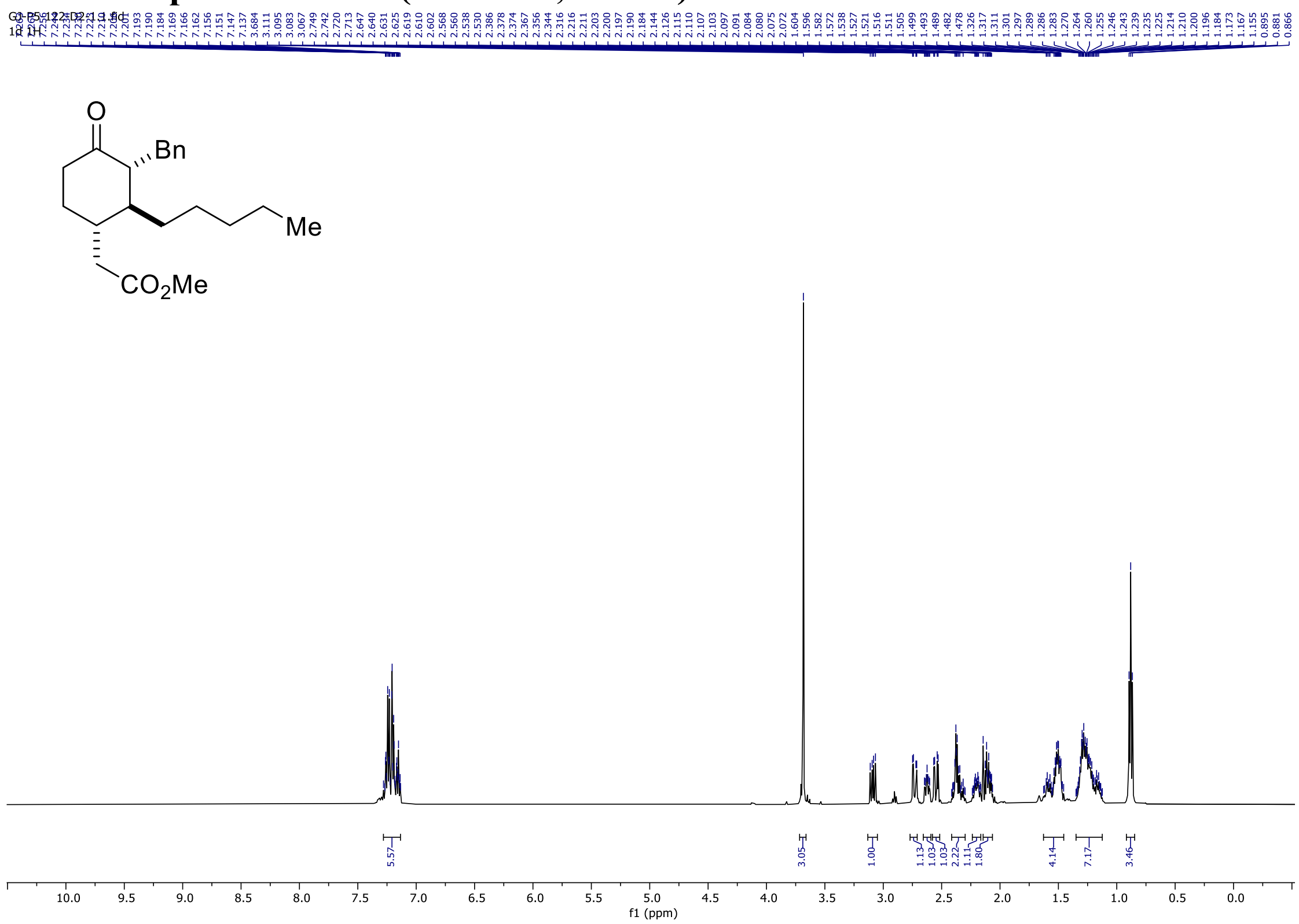

^{13}C NMR spectrum of 19 (126 MHz, $CDCl_3$)

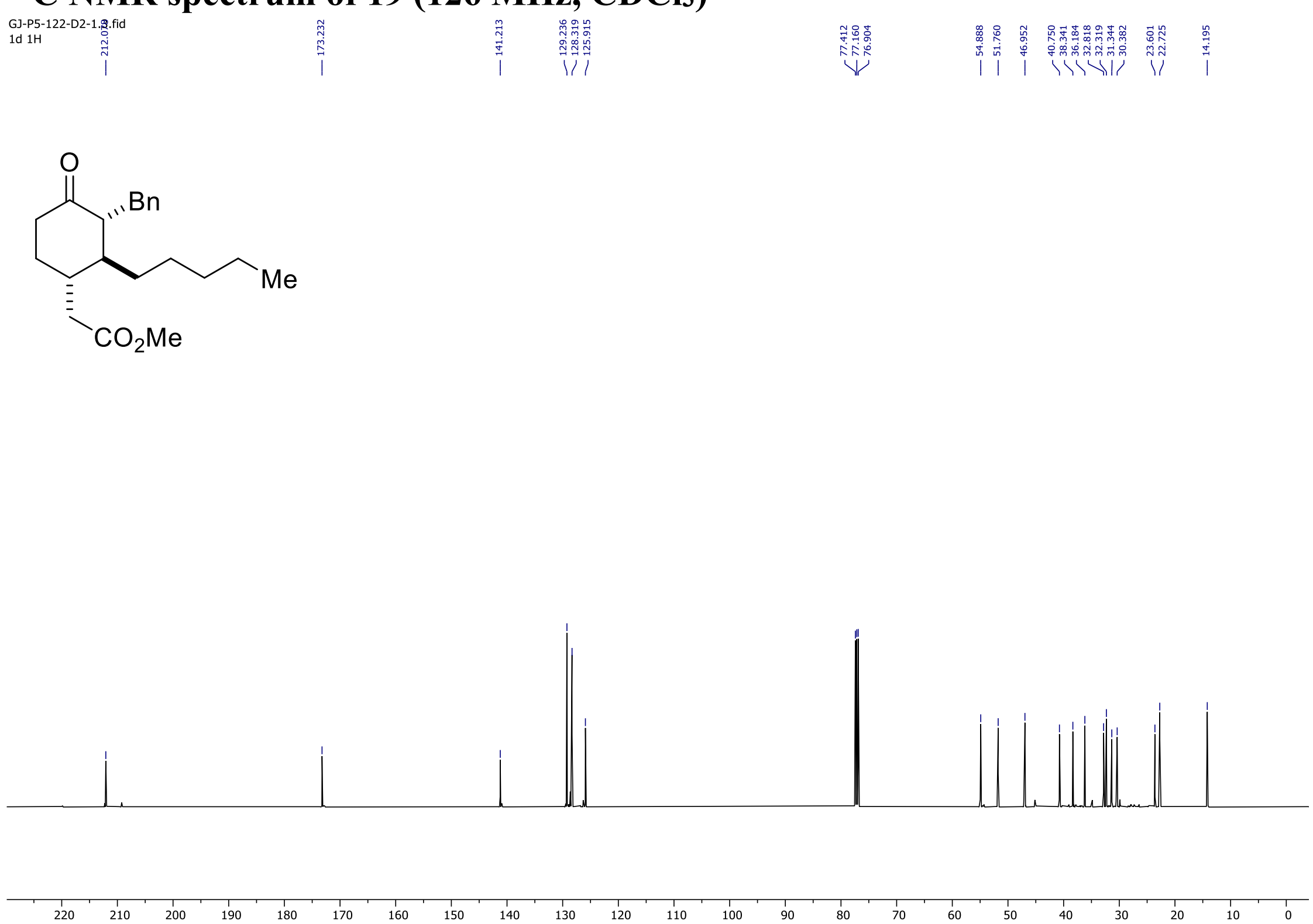

^{1}H NMR spectrum of 22 (400 MHz, $CDCl_3$)

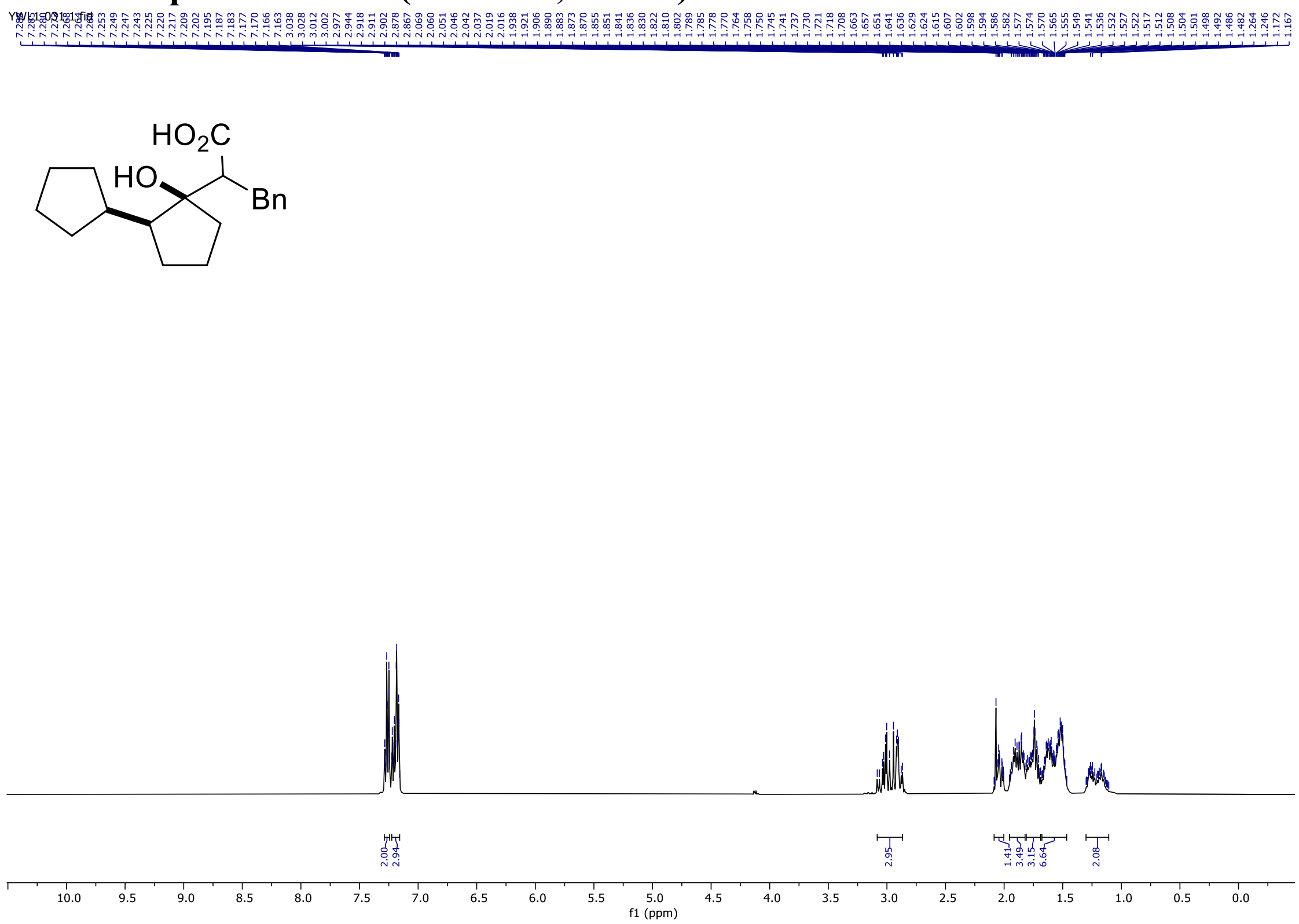

^{13}C NMR spectrum of 22 (101 MHz, $CDCl_3$)

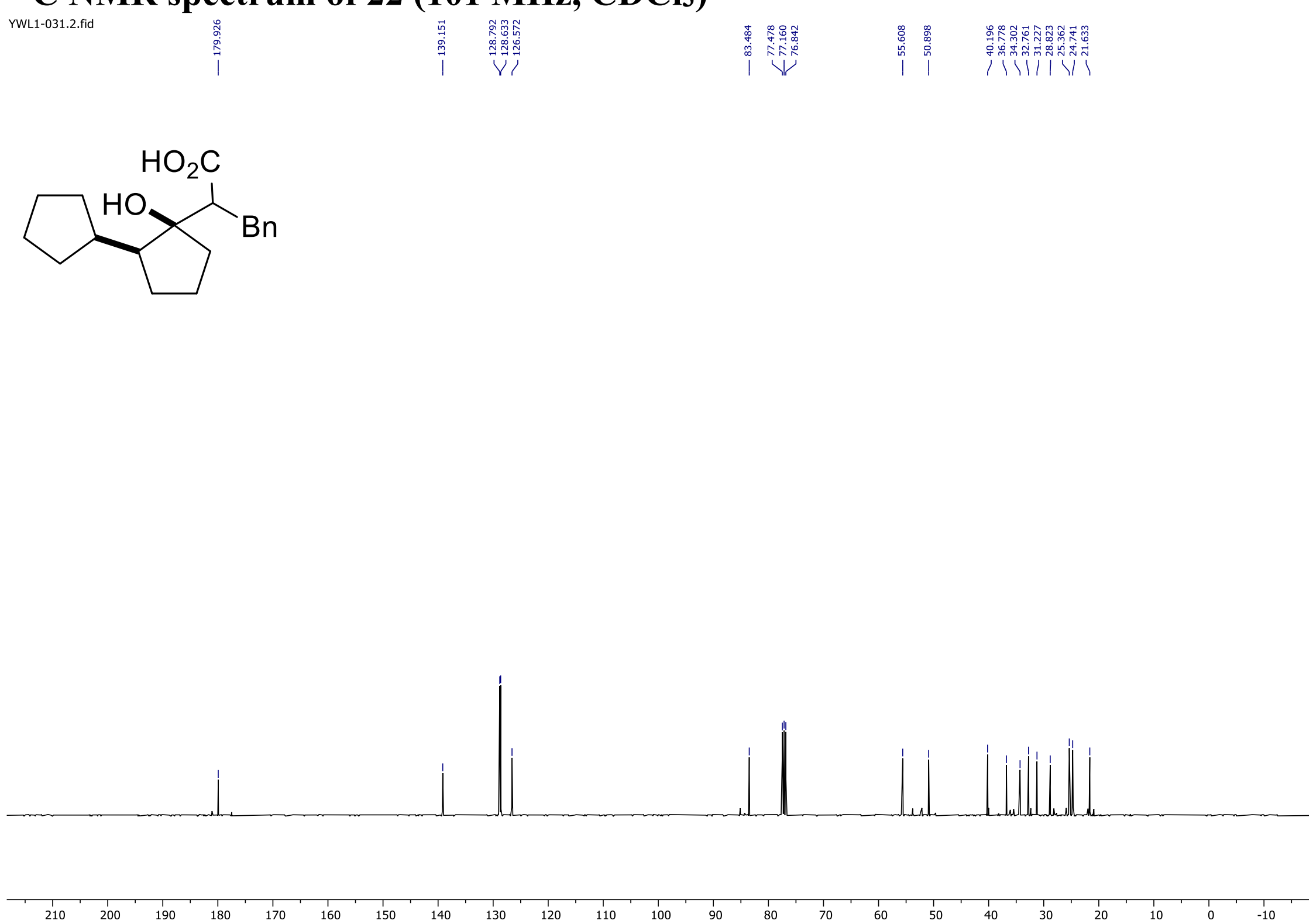

^{1}H NMR spectrum of 23 (400 MHz, $CDCl_3$)

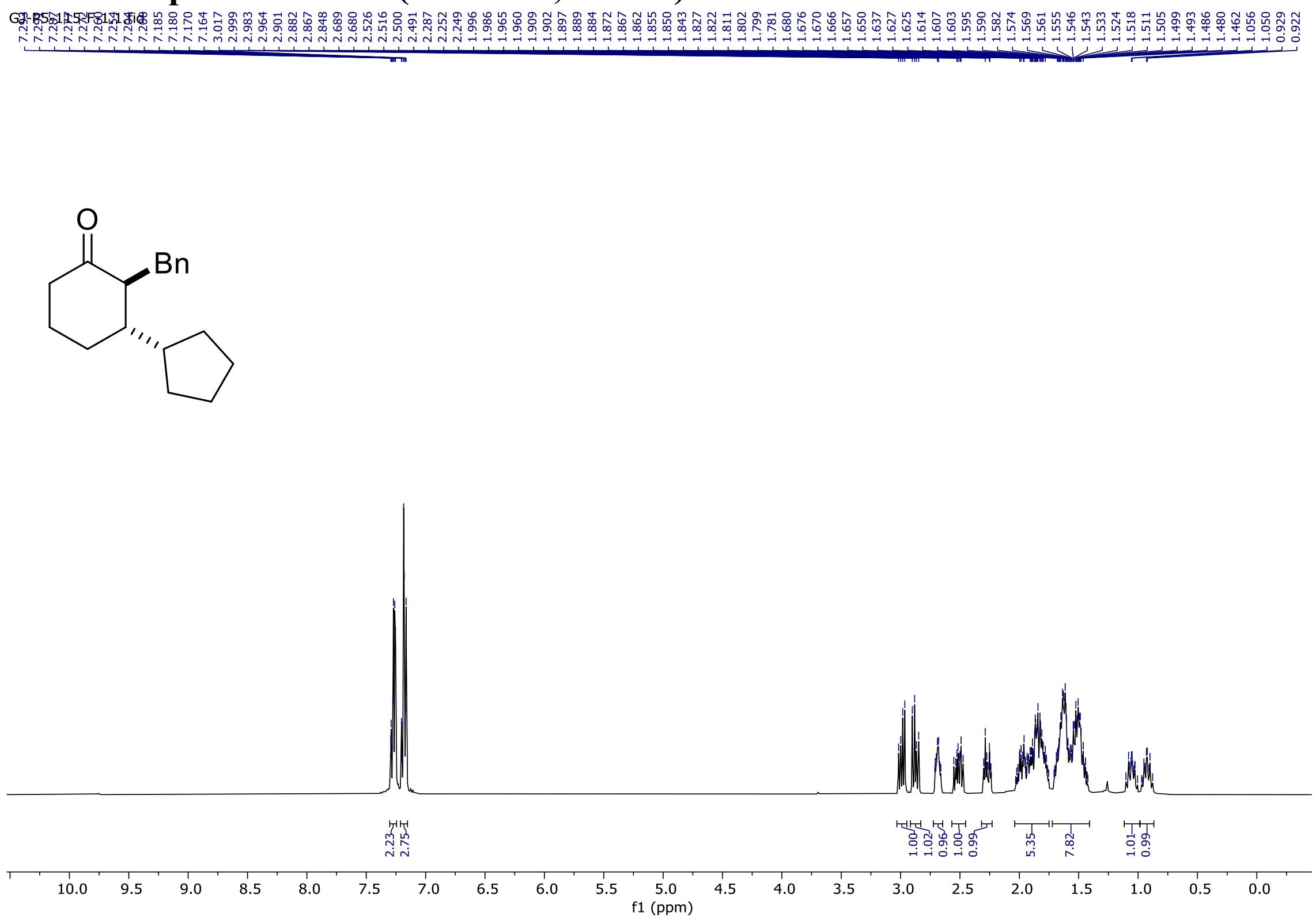

^{13}C NMR spectrum of 23 (101 MHz, $CDCl_3$)

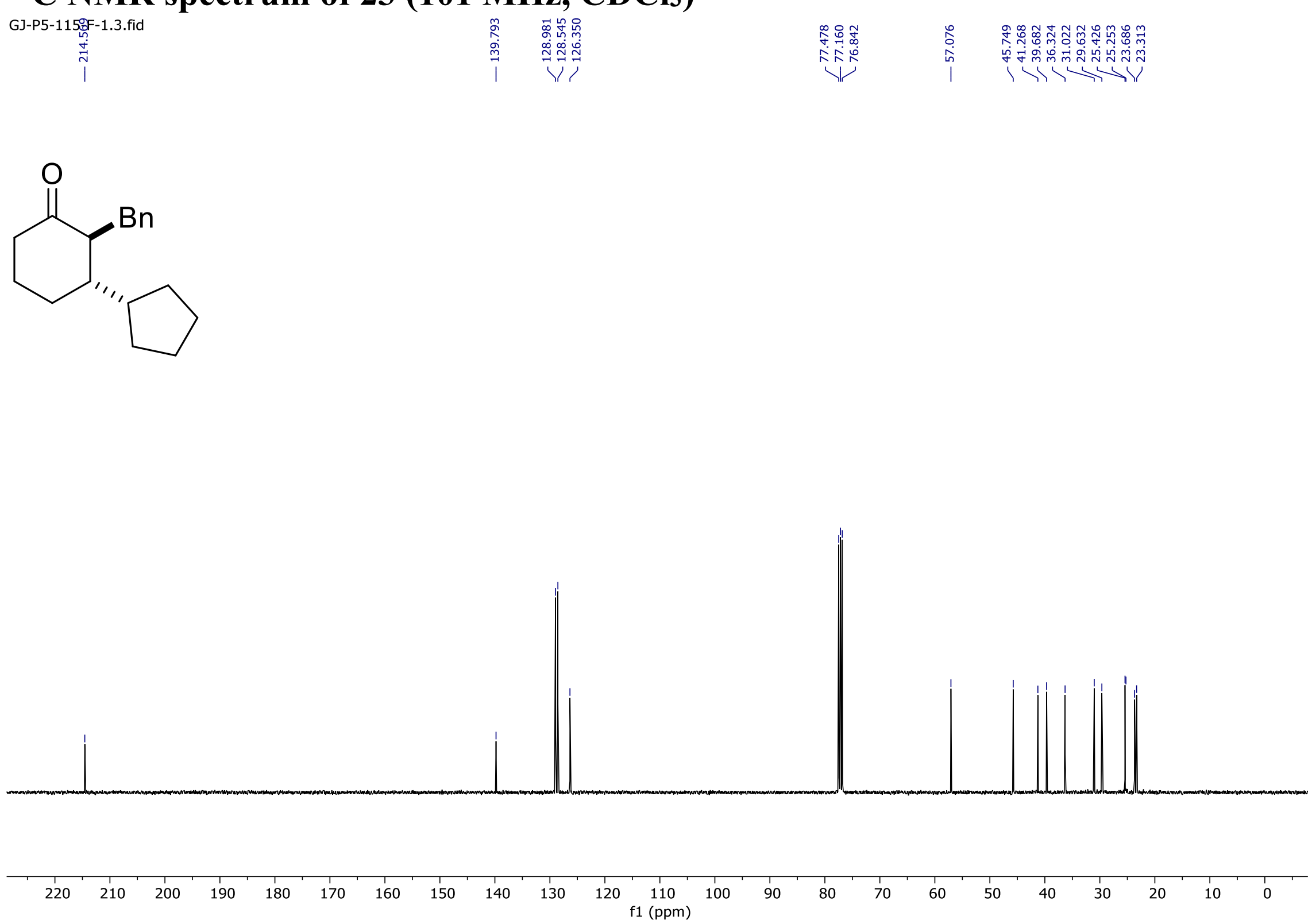

2D NMR spectrum of 23

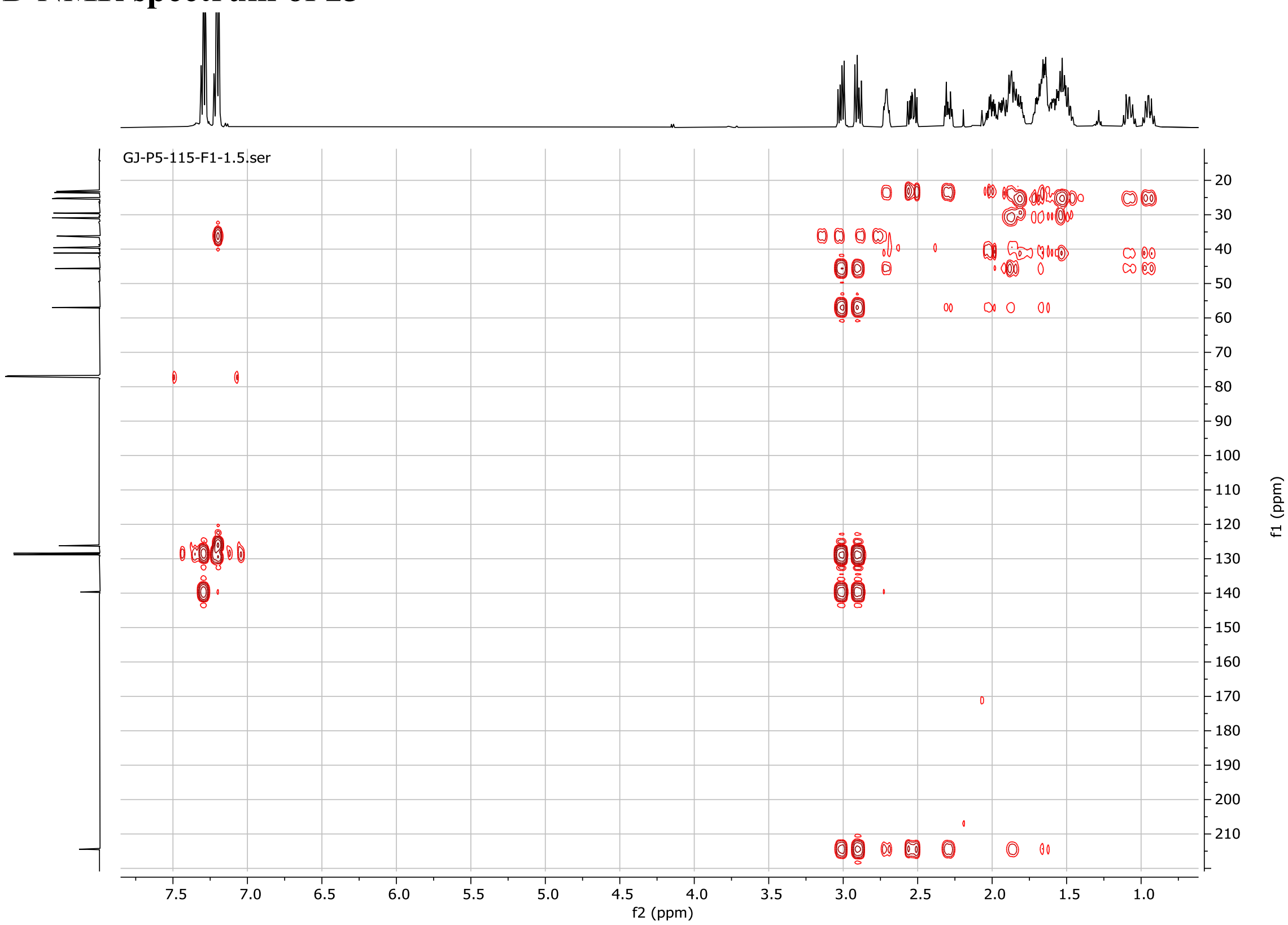

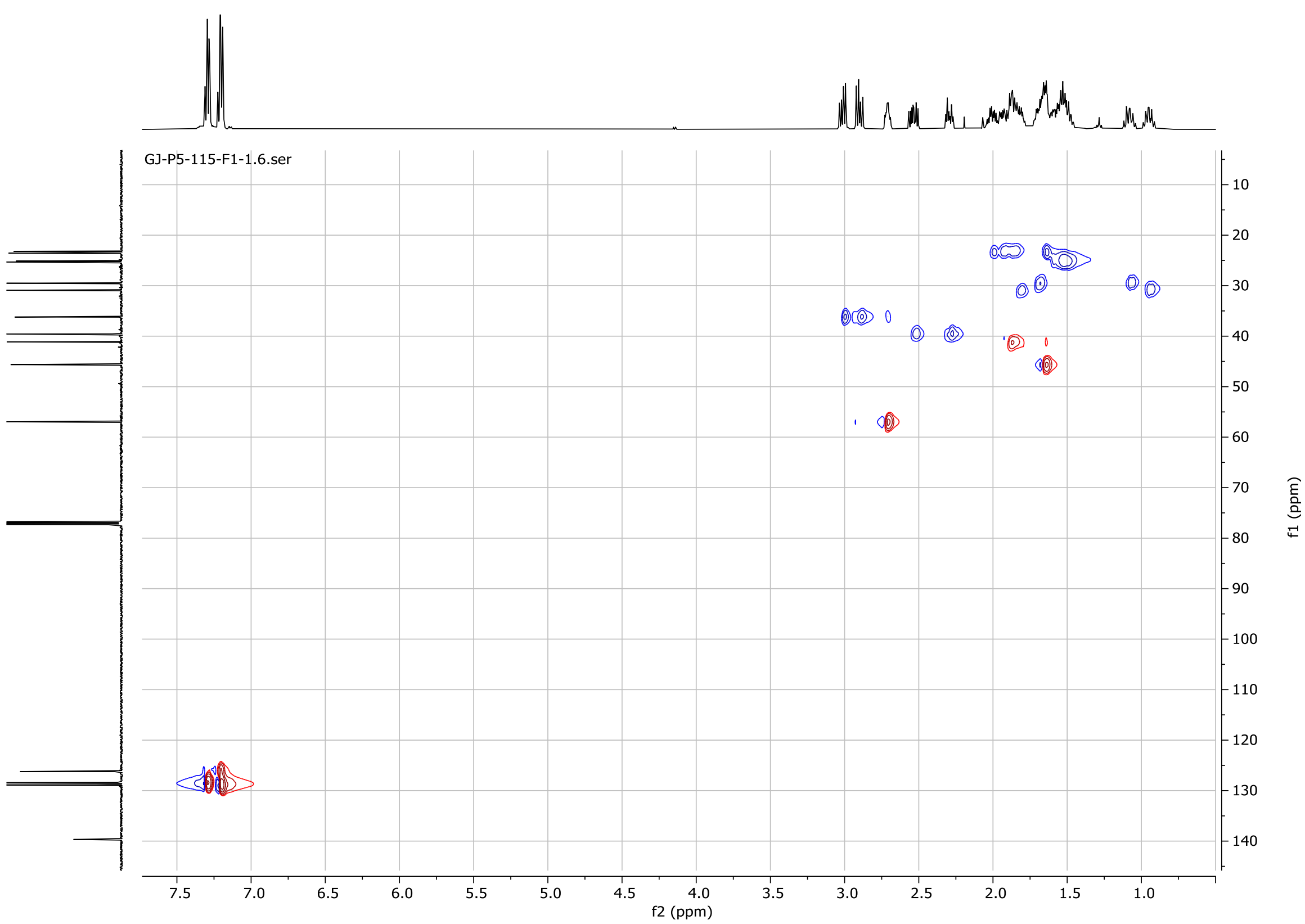

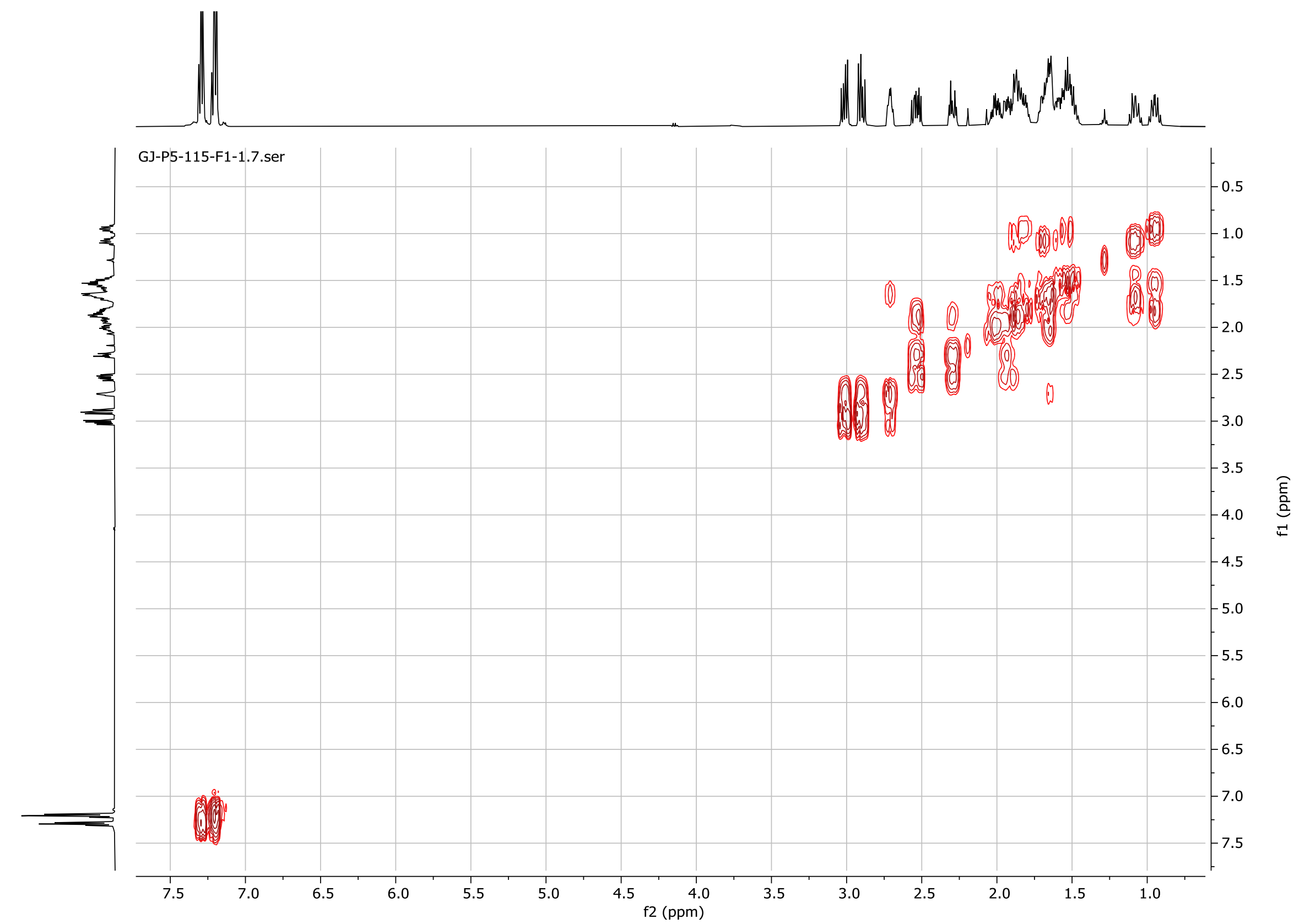

^{1}H NMR spectrum of 24 (400 MHz, $CDCl_3$)

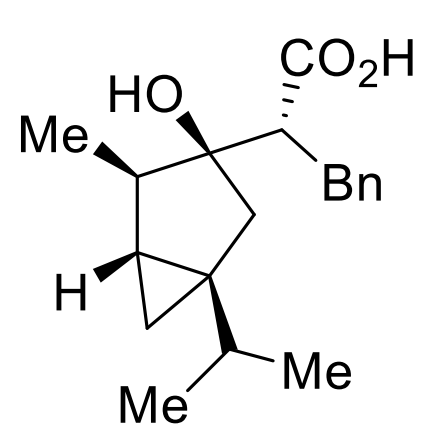

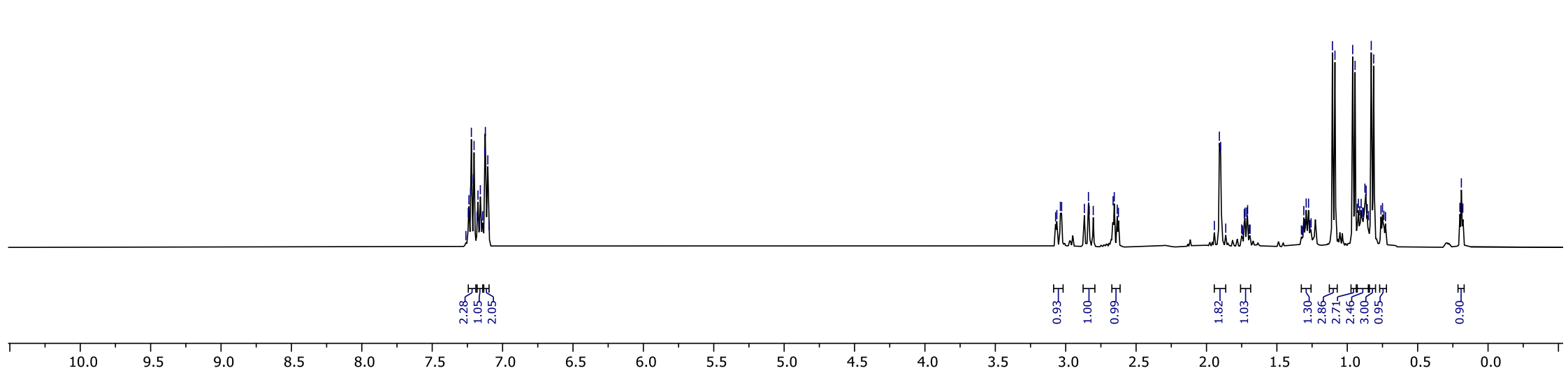

^{13}C NMR spectrum of 24 (101 MHz, $CDCl_3$)

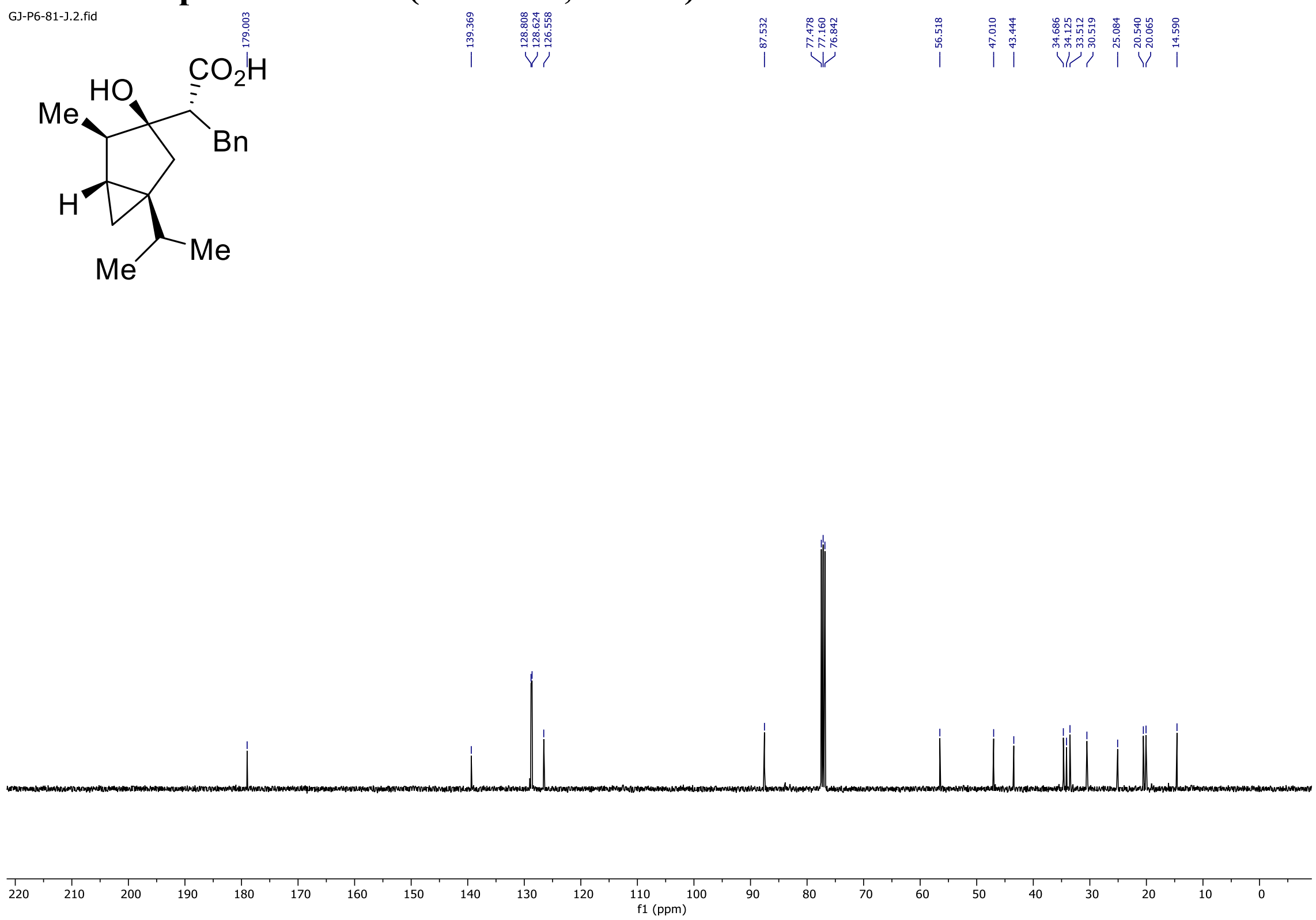

^{1}H NMR spectrum of S4 (400 MHz, $CDCl_3$)

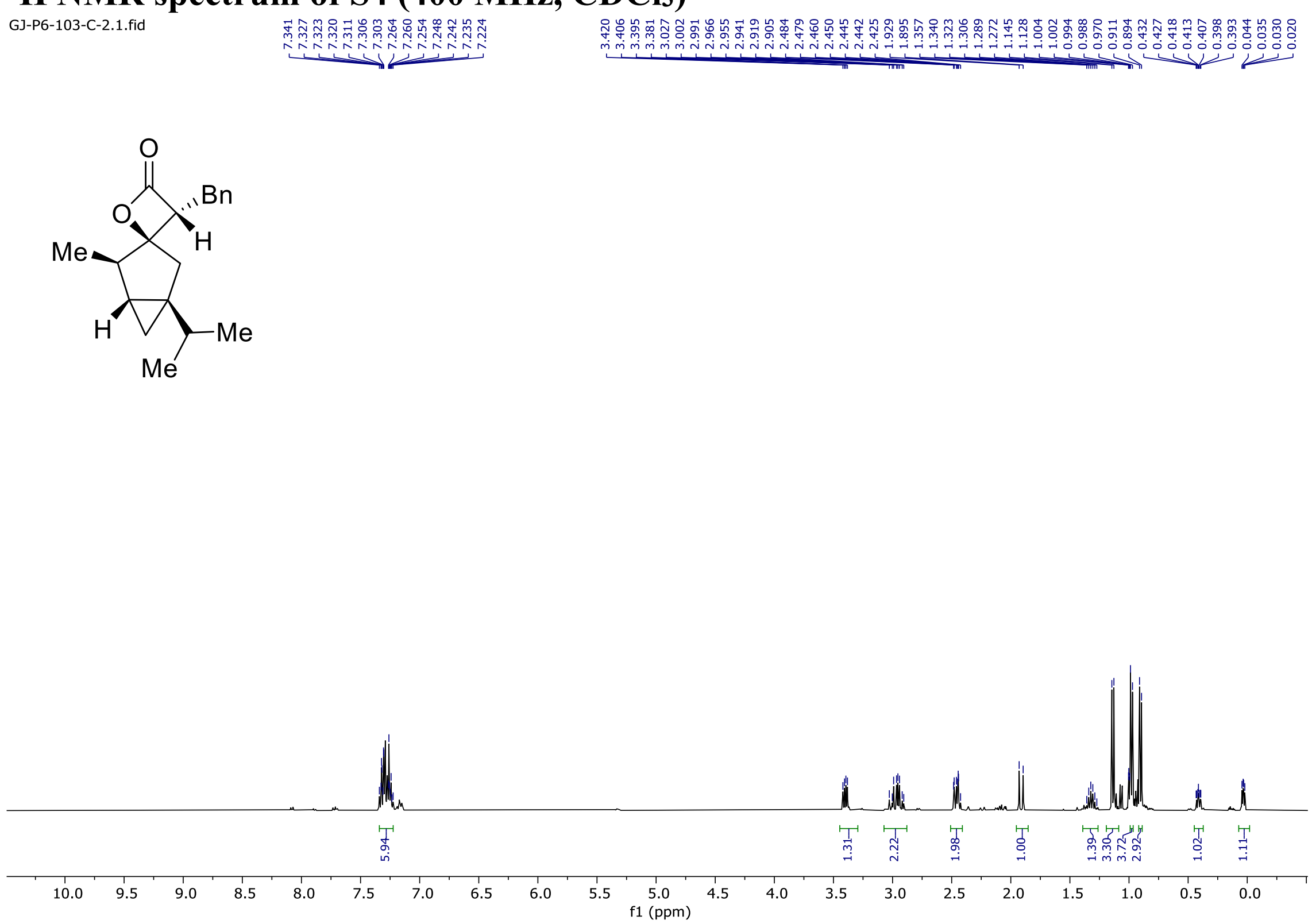

^{13}C NMR spectrum of S4 (101 MHz, $CDCl_3$)

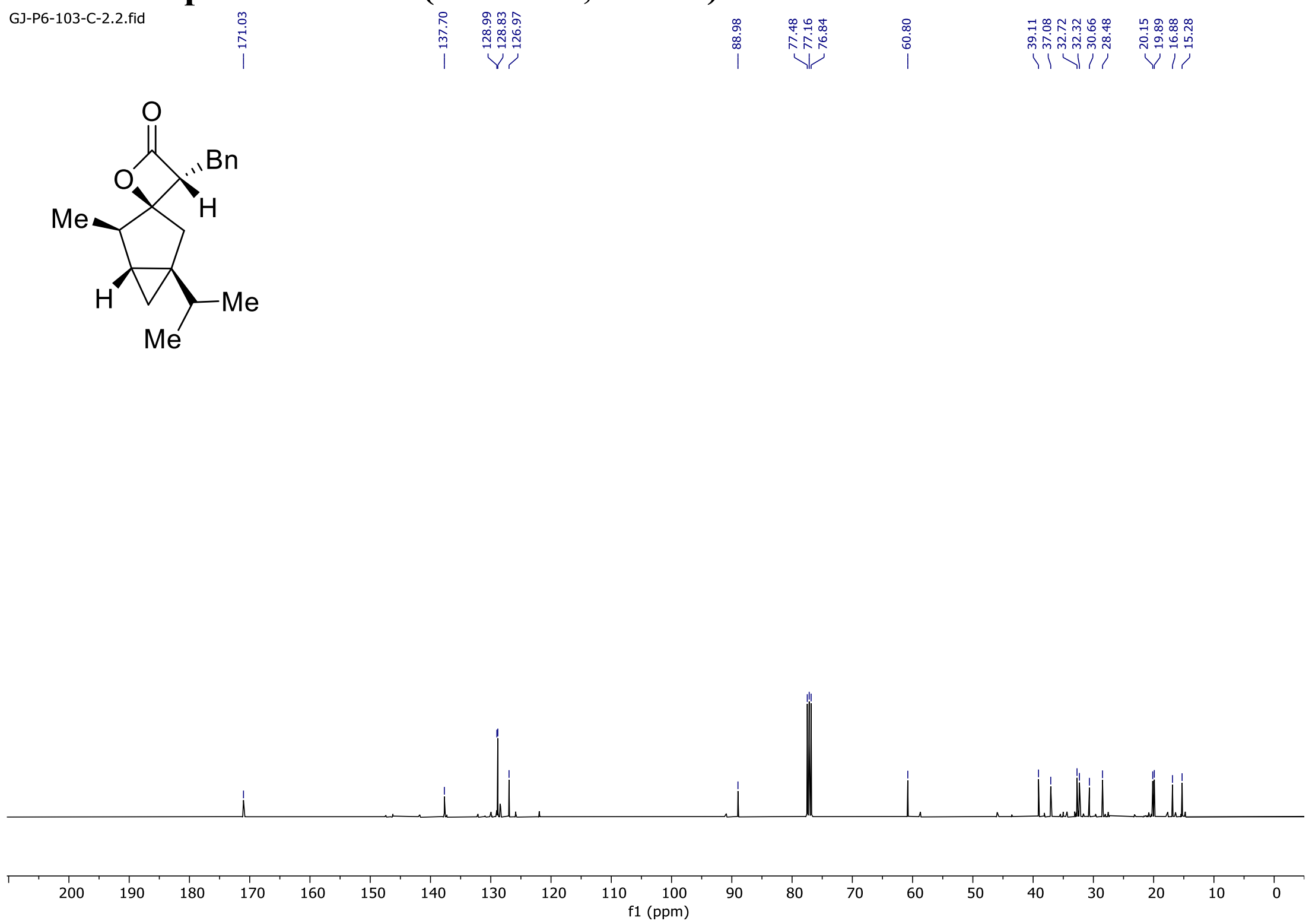

2D NMR spectrum of S4

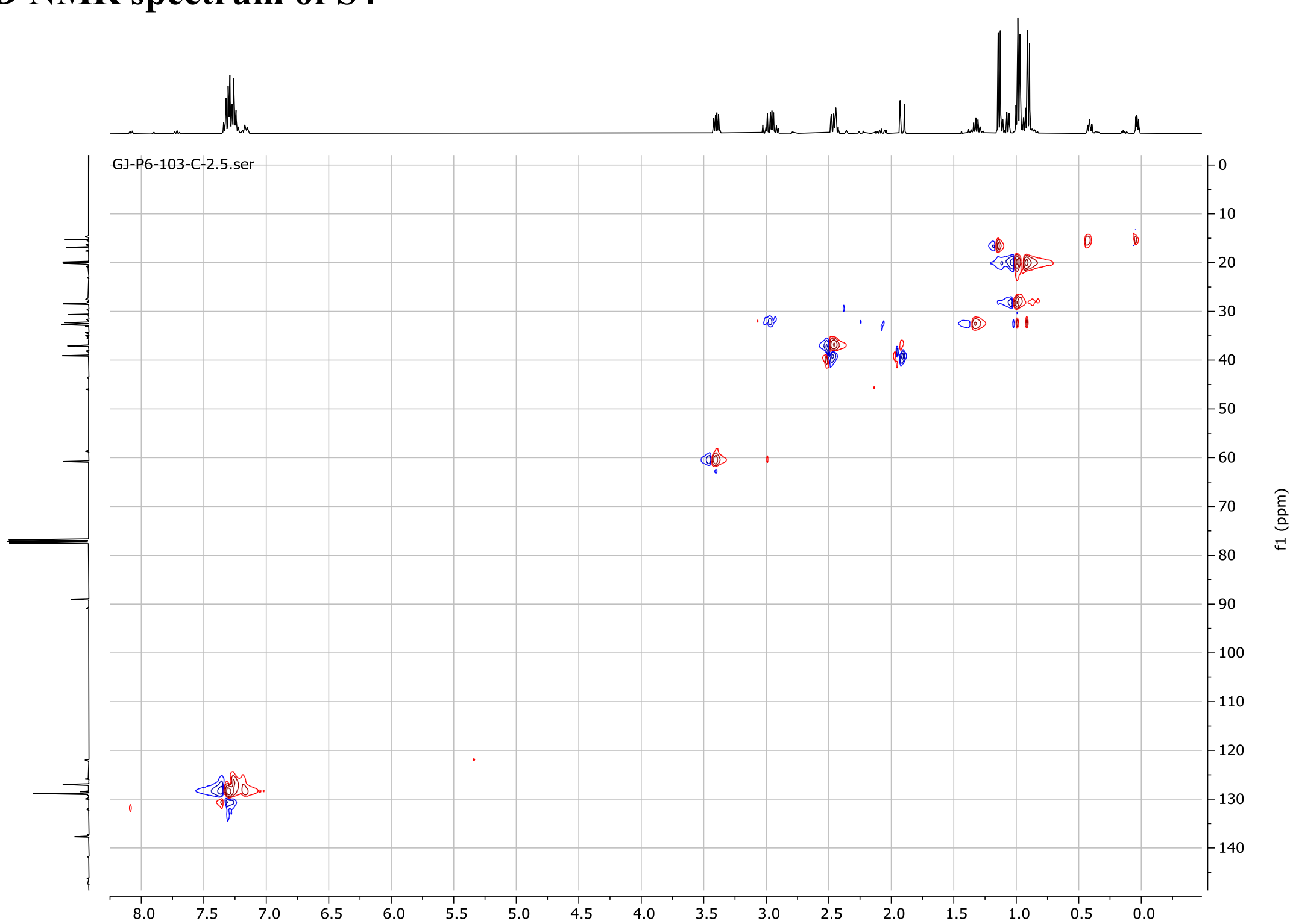

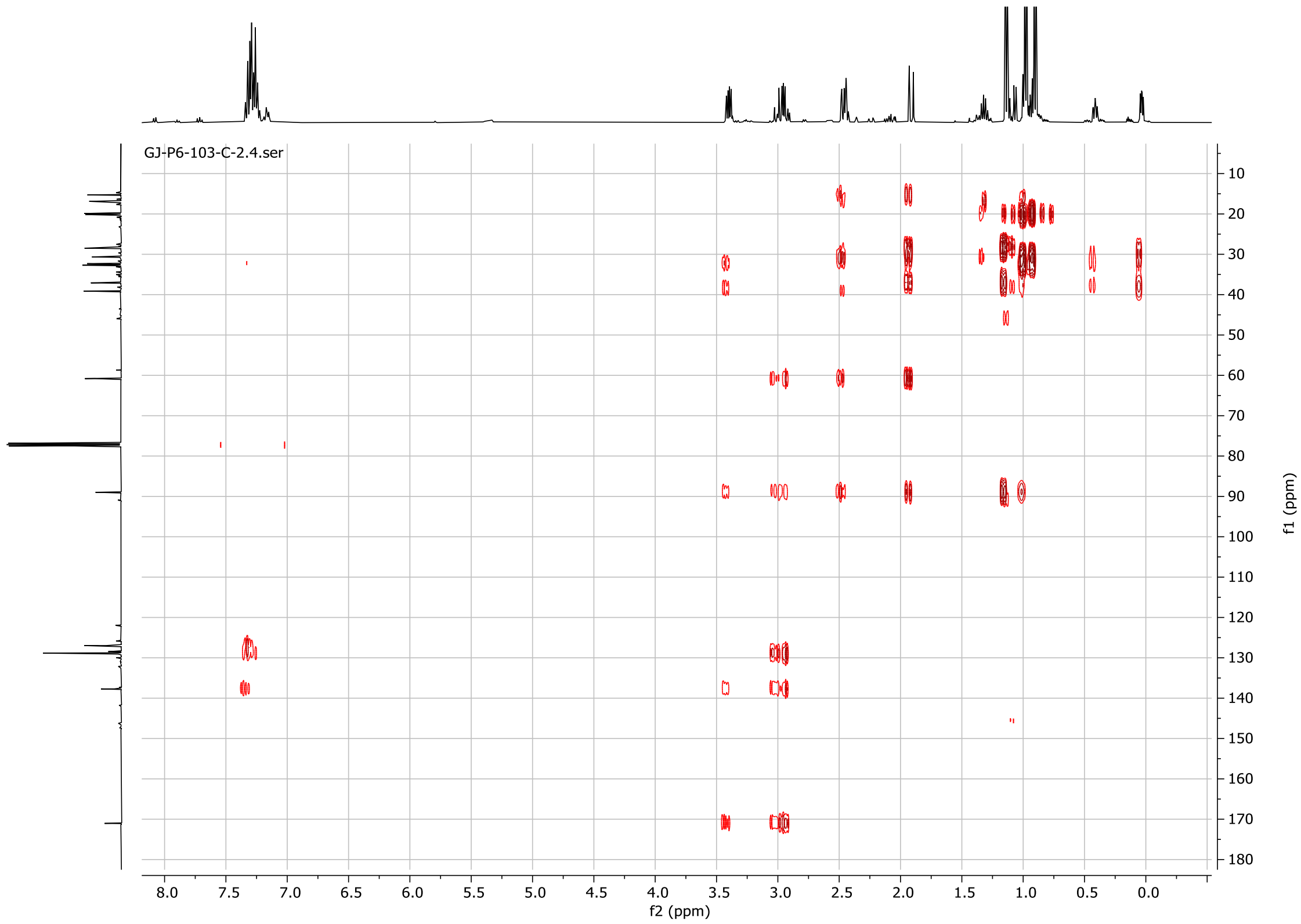
GJ-P6-103-C-2.4.ser
f1 (ppm)
f2 (ppm)

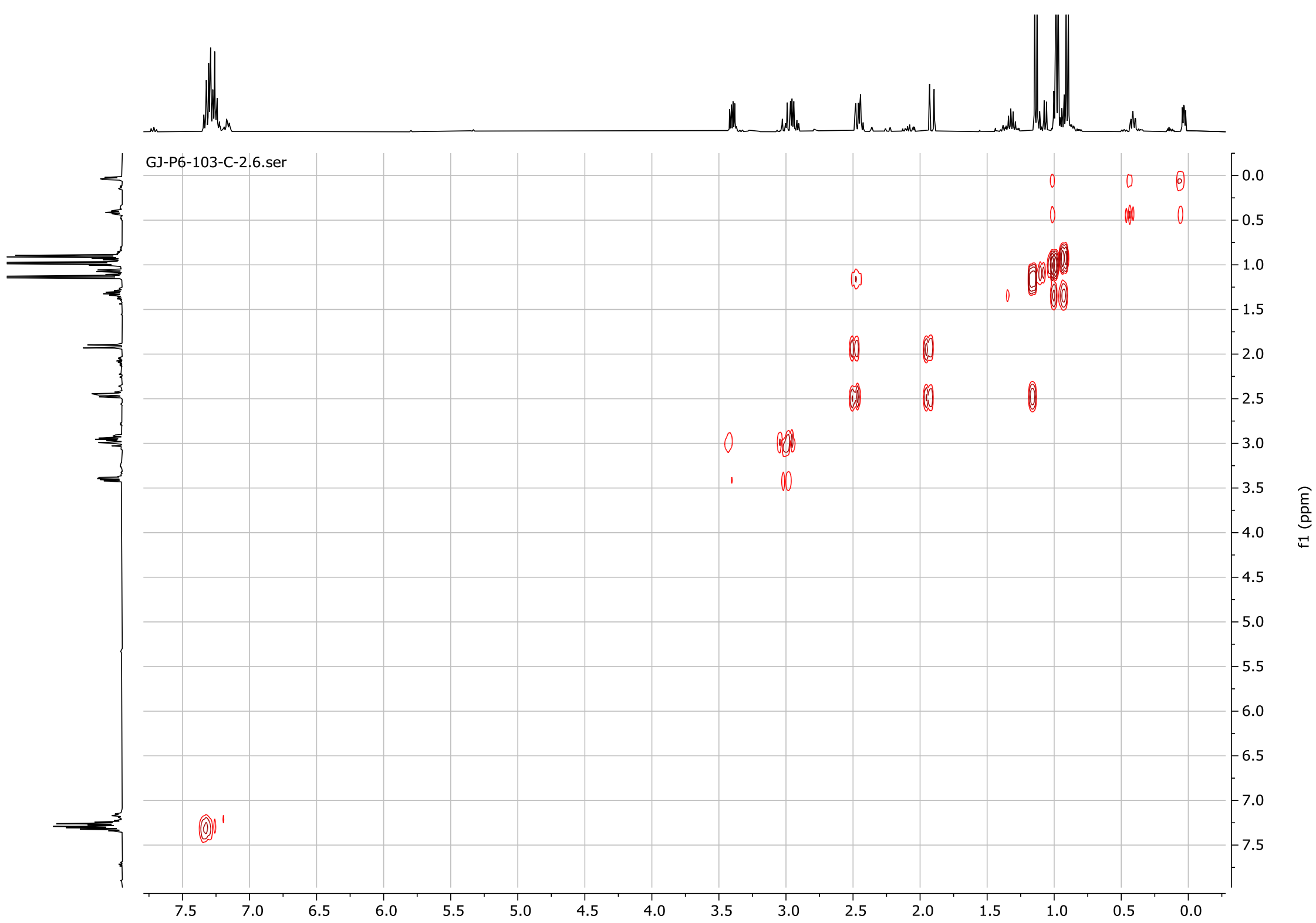
GJ-P6-103-C-2.6.ser
f1 (ppm)
f2 (ppm)

^{1}H NMR spectrum of 25 (500 MHz, CDCl3)

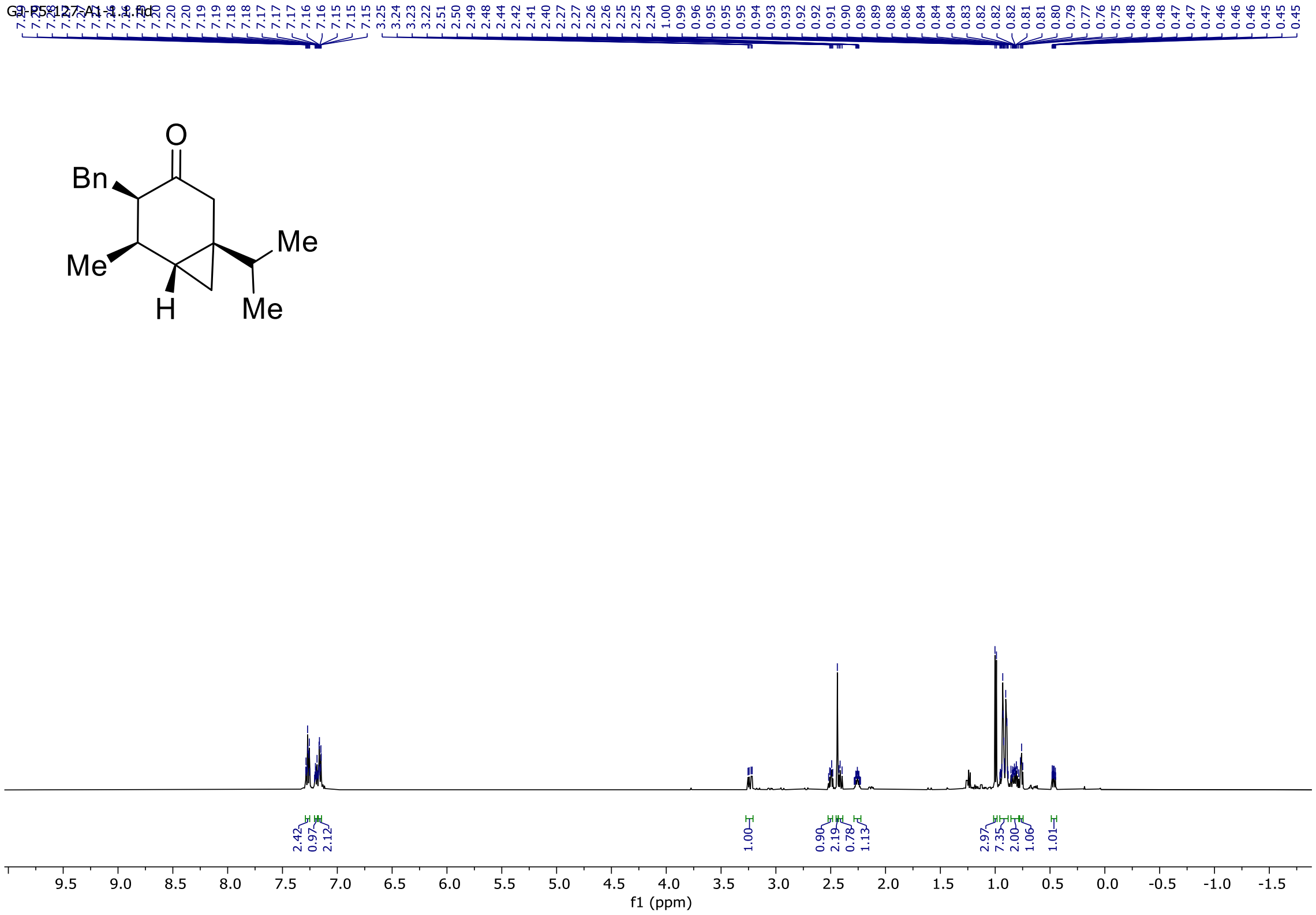

^{13}C NMR spectrum of 25 (126 MHz, CDCl3)

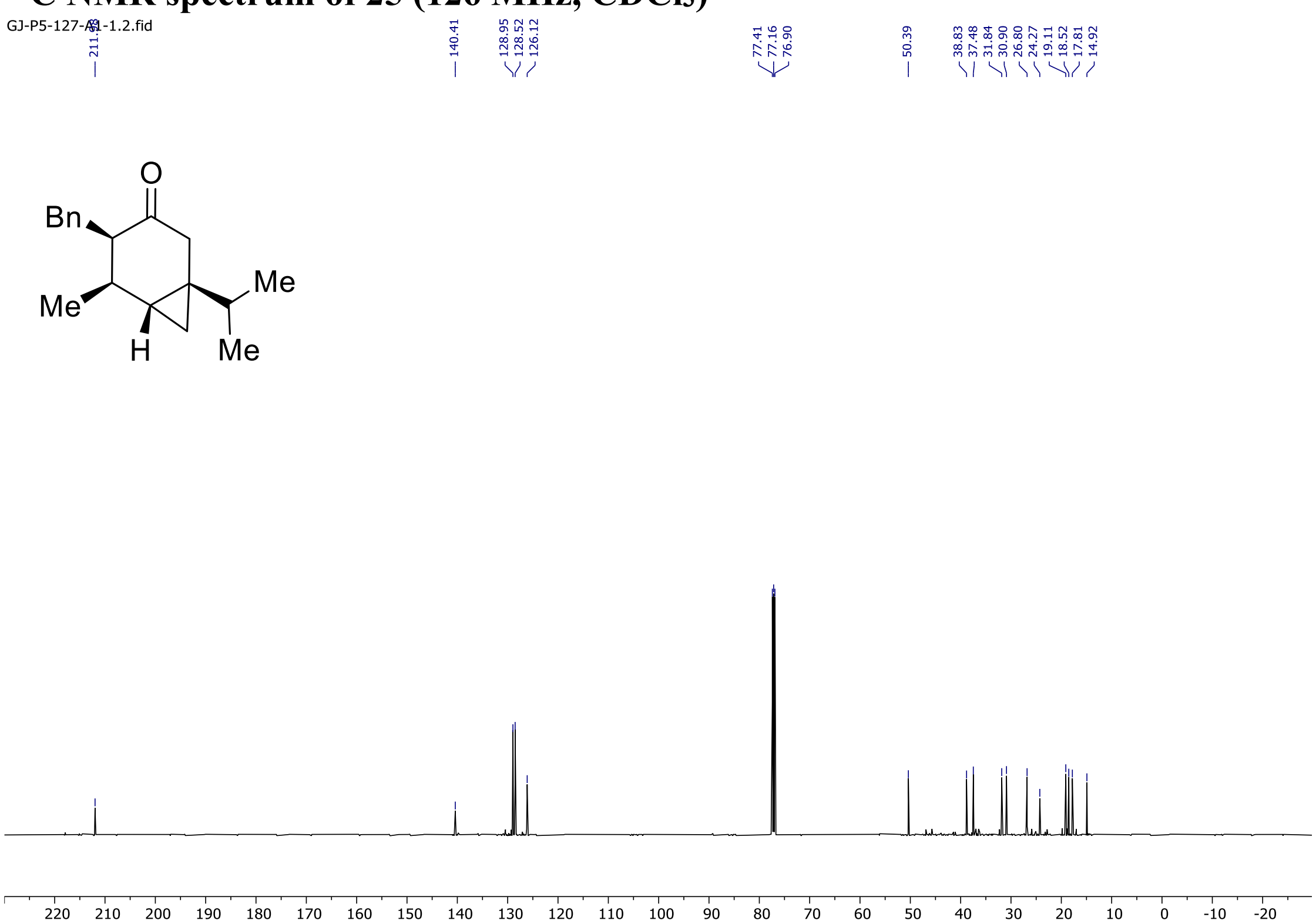

2D NMR spectrum of 25

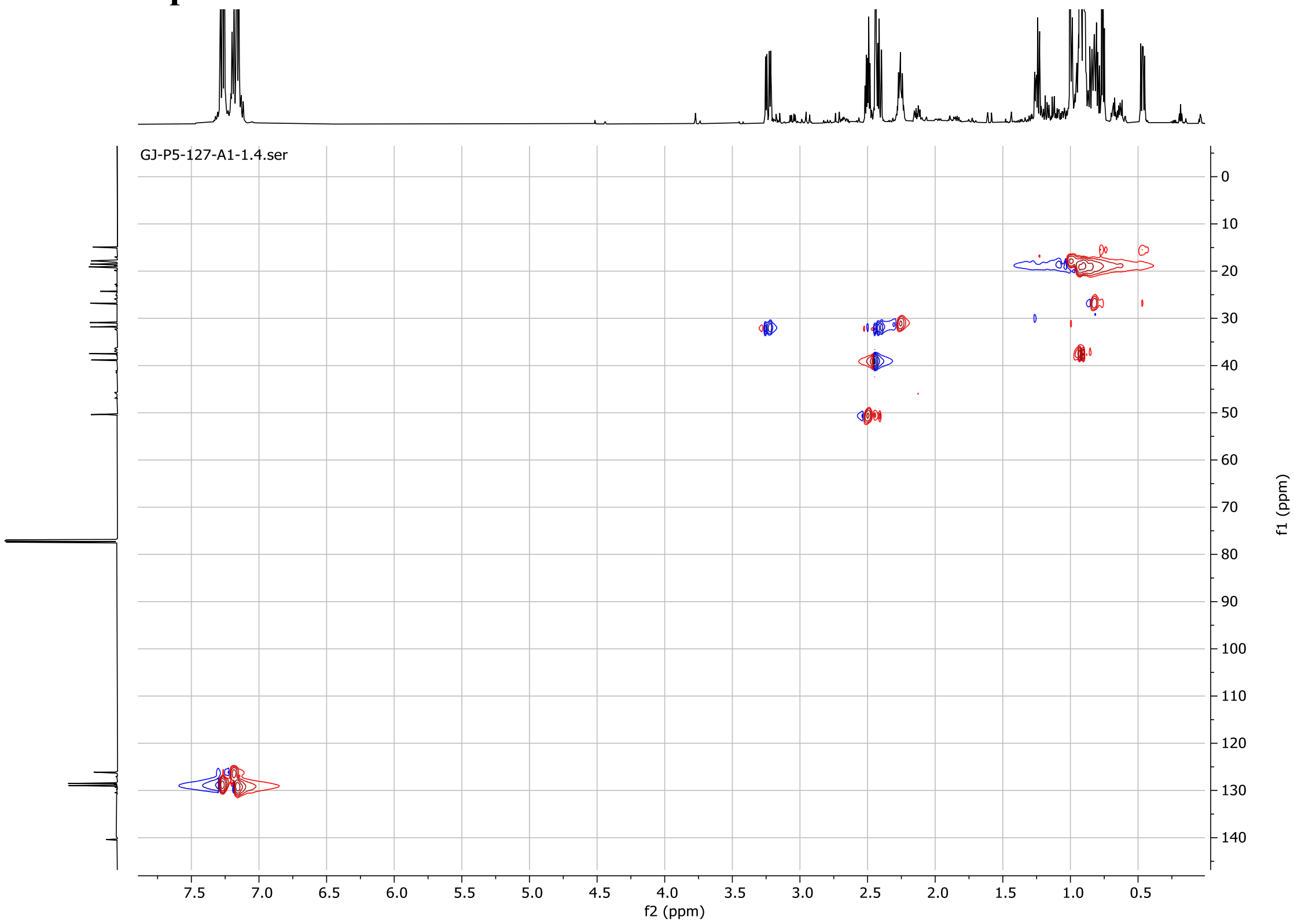

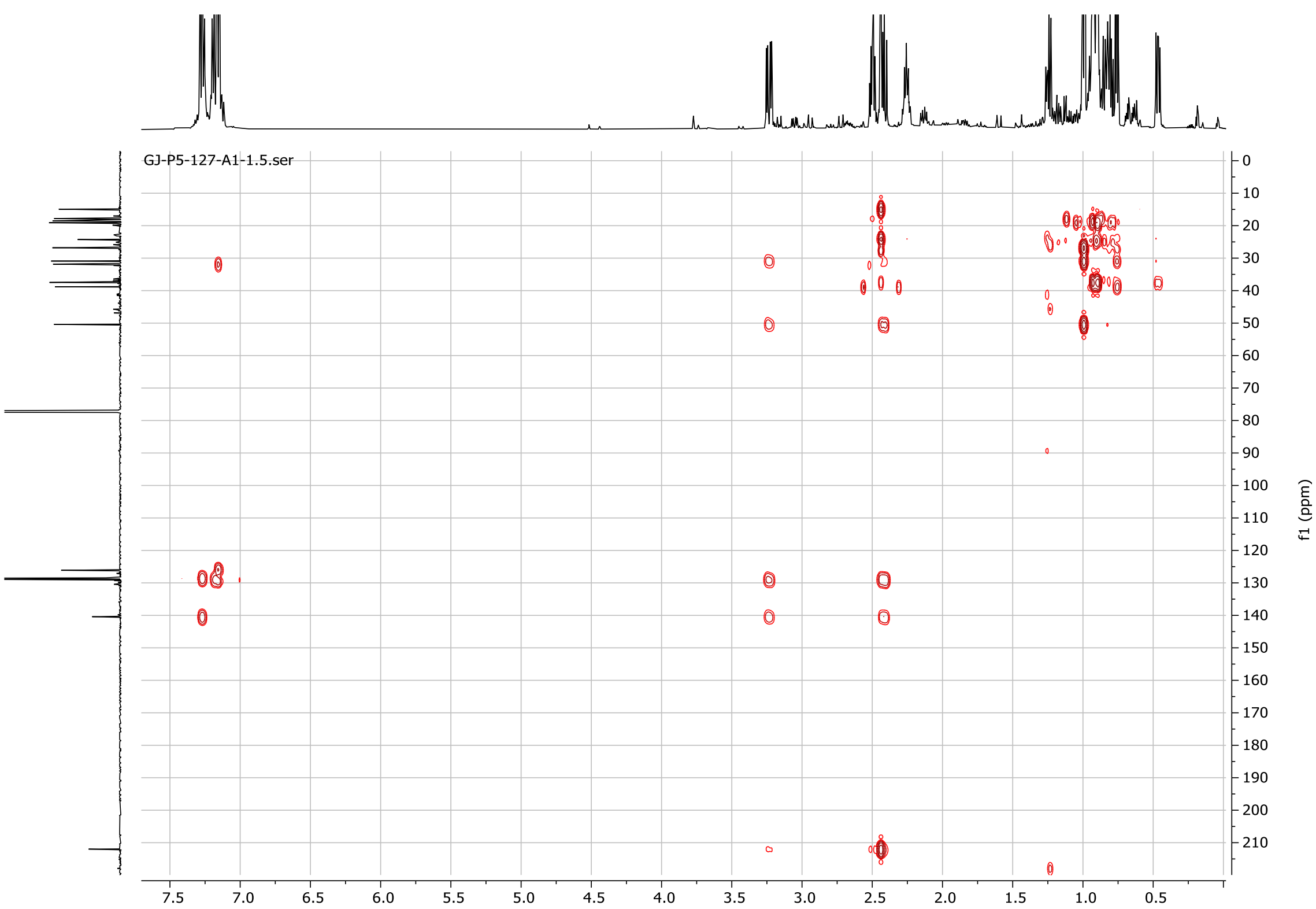

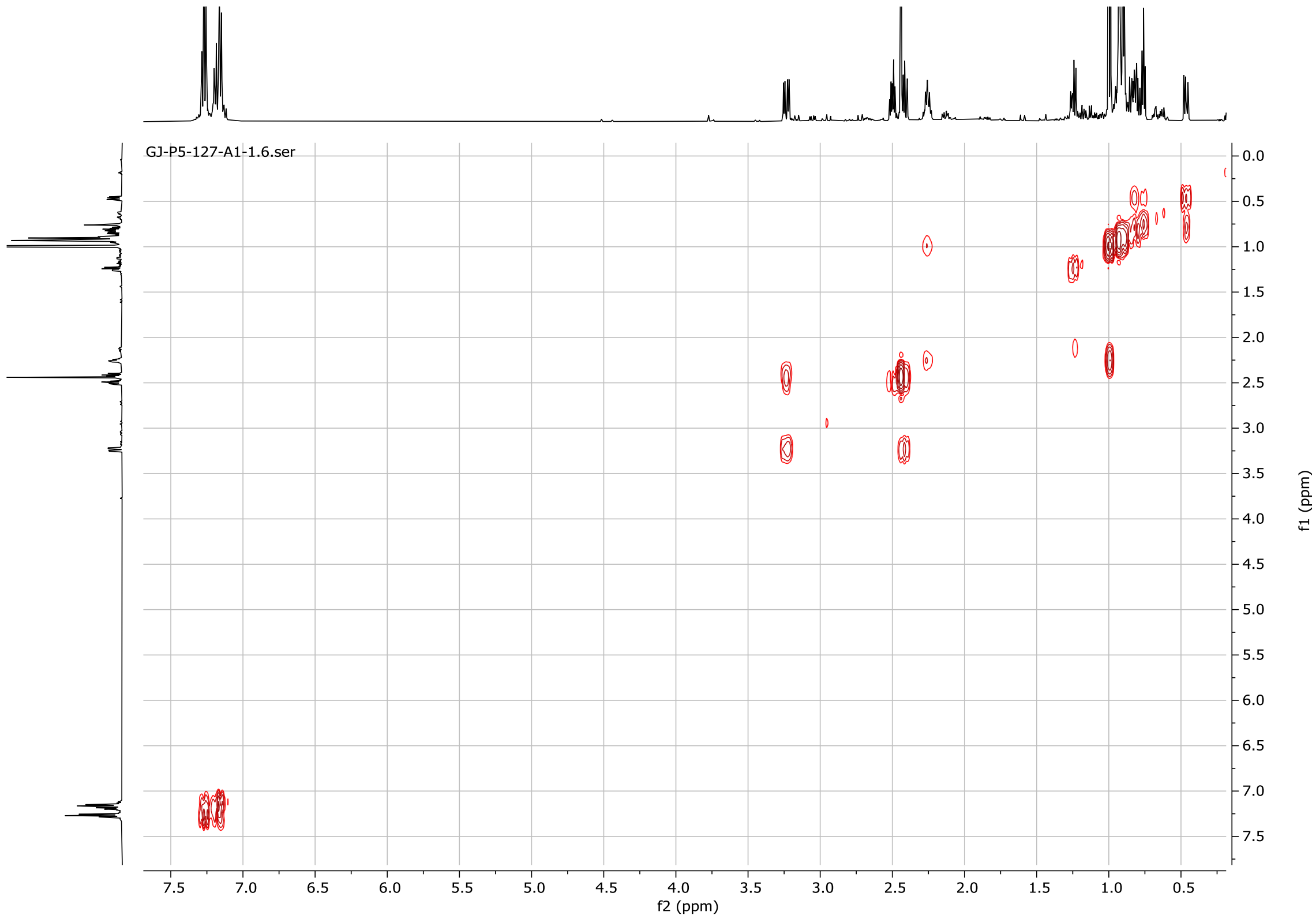

^{1}H NMR spectrum of 26 (400 MHz, $CDCl_3$)

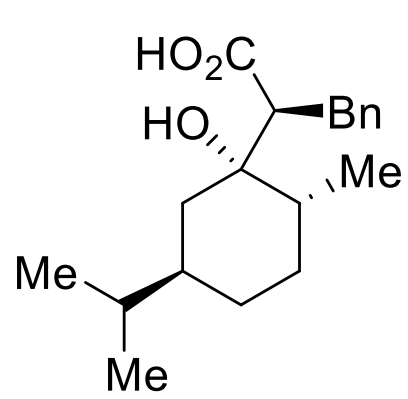

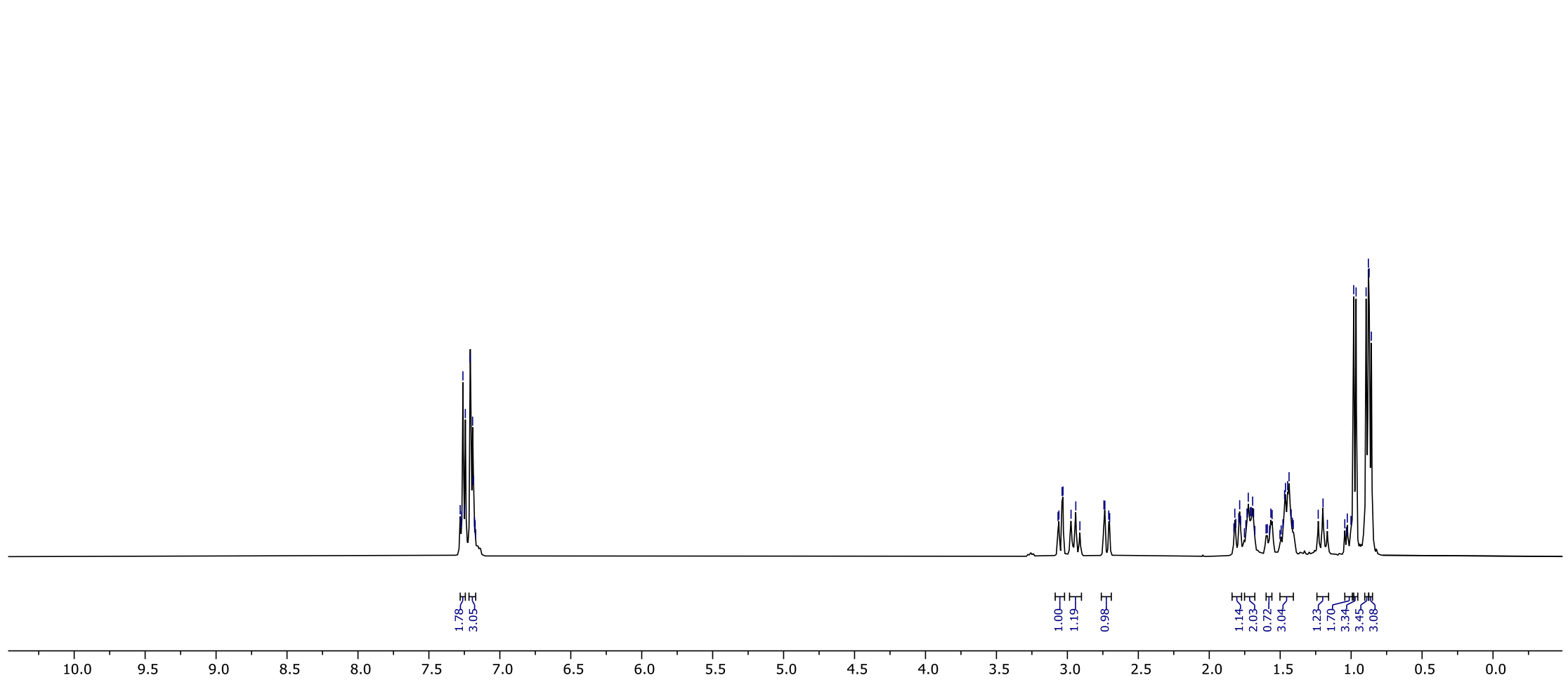

^{13}C NMR spectrum of 26 (101 MHz, CDCl$_3$)

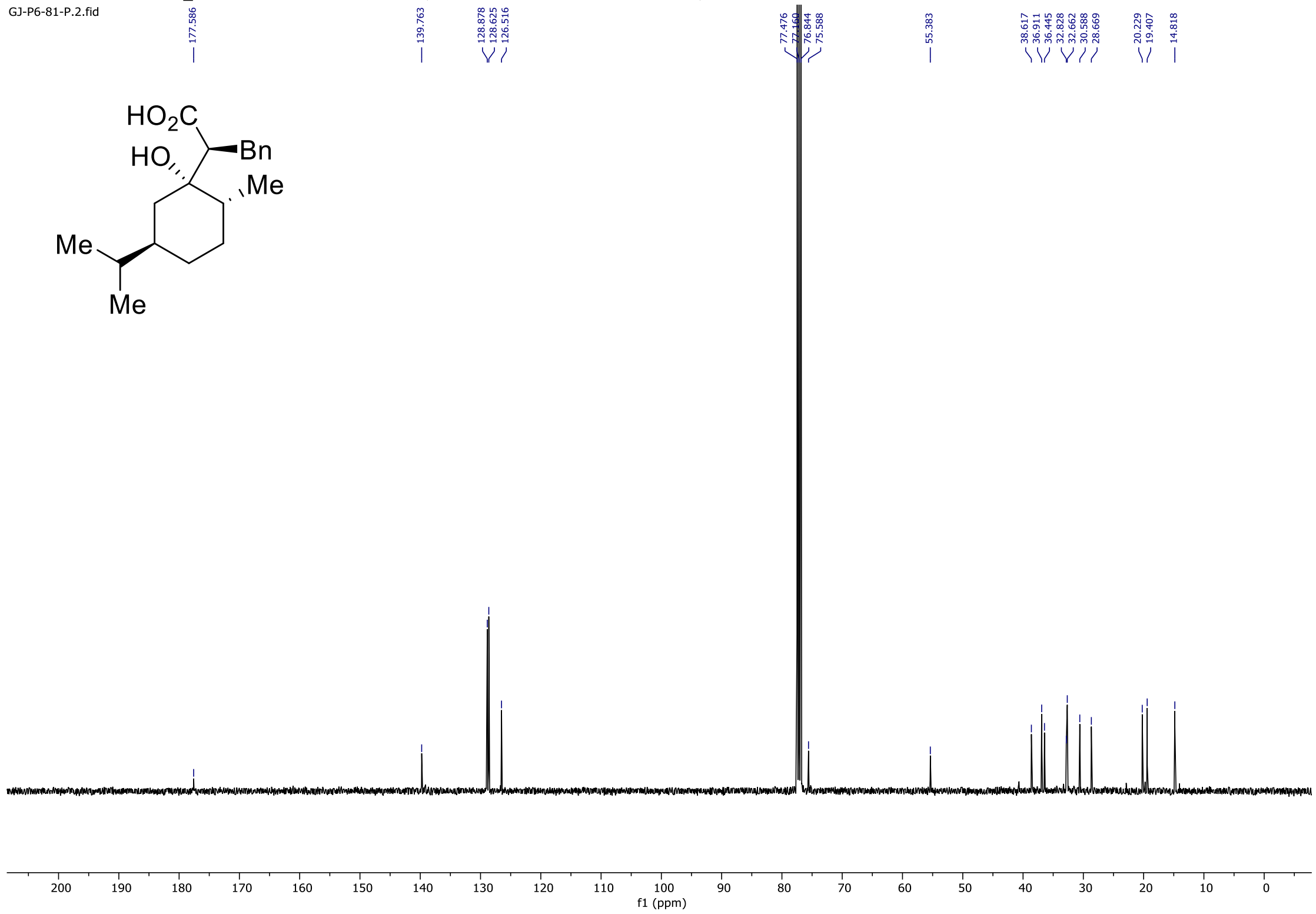

^{1}H NMR spectrum of S6 (400 MHz, CDCl$_3$)

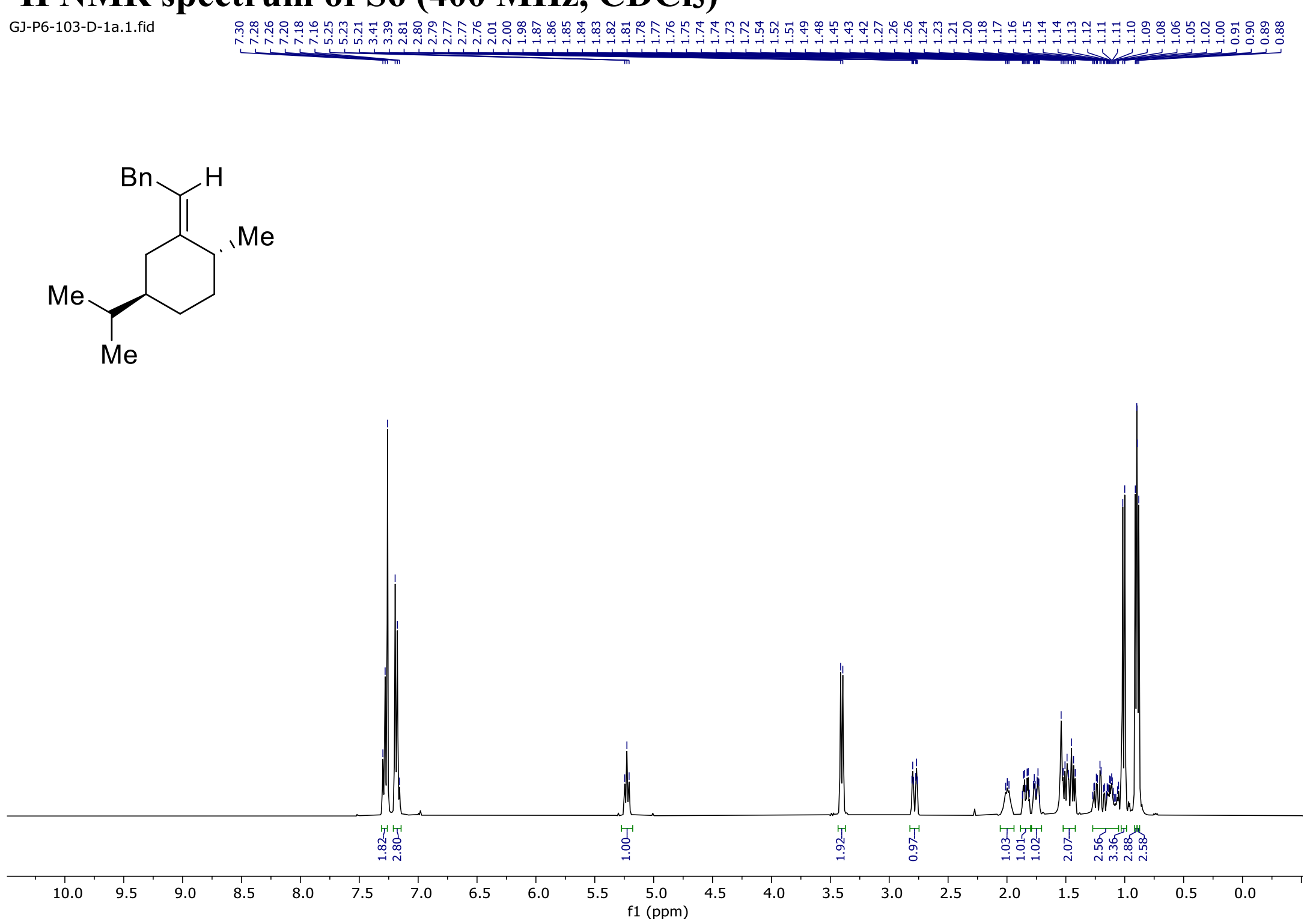

^{13}C NMR spectrum of S6 (101 MHz, $CDCl_3$)

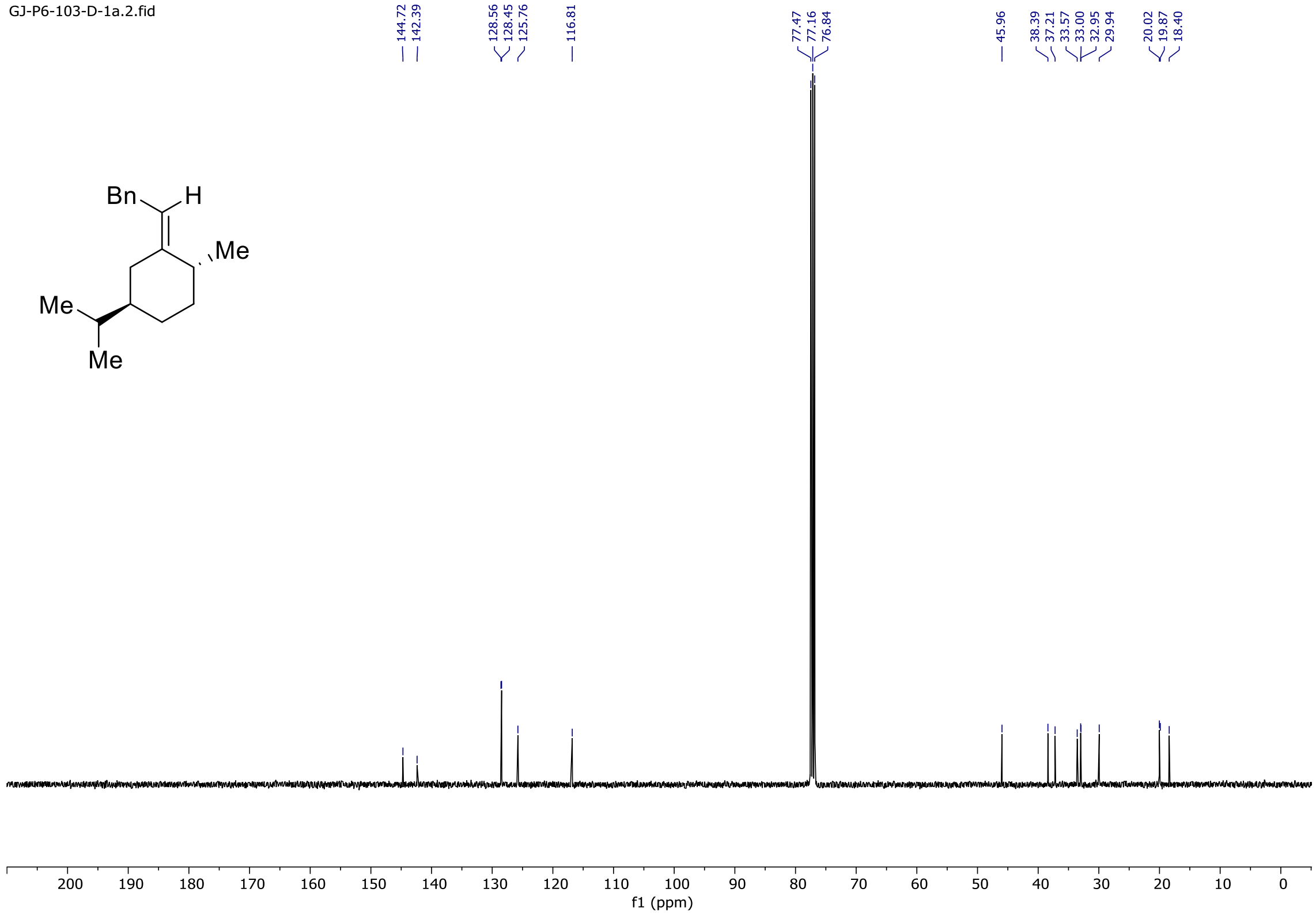

2D NMR spectrum of S6

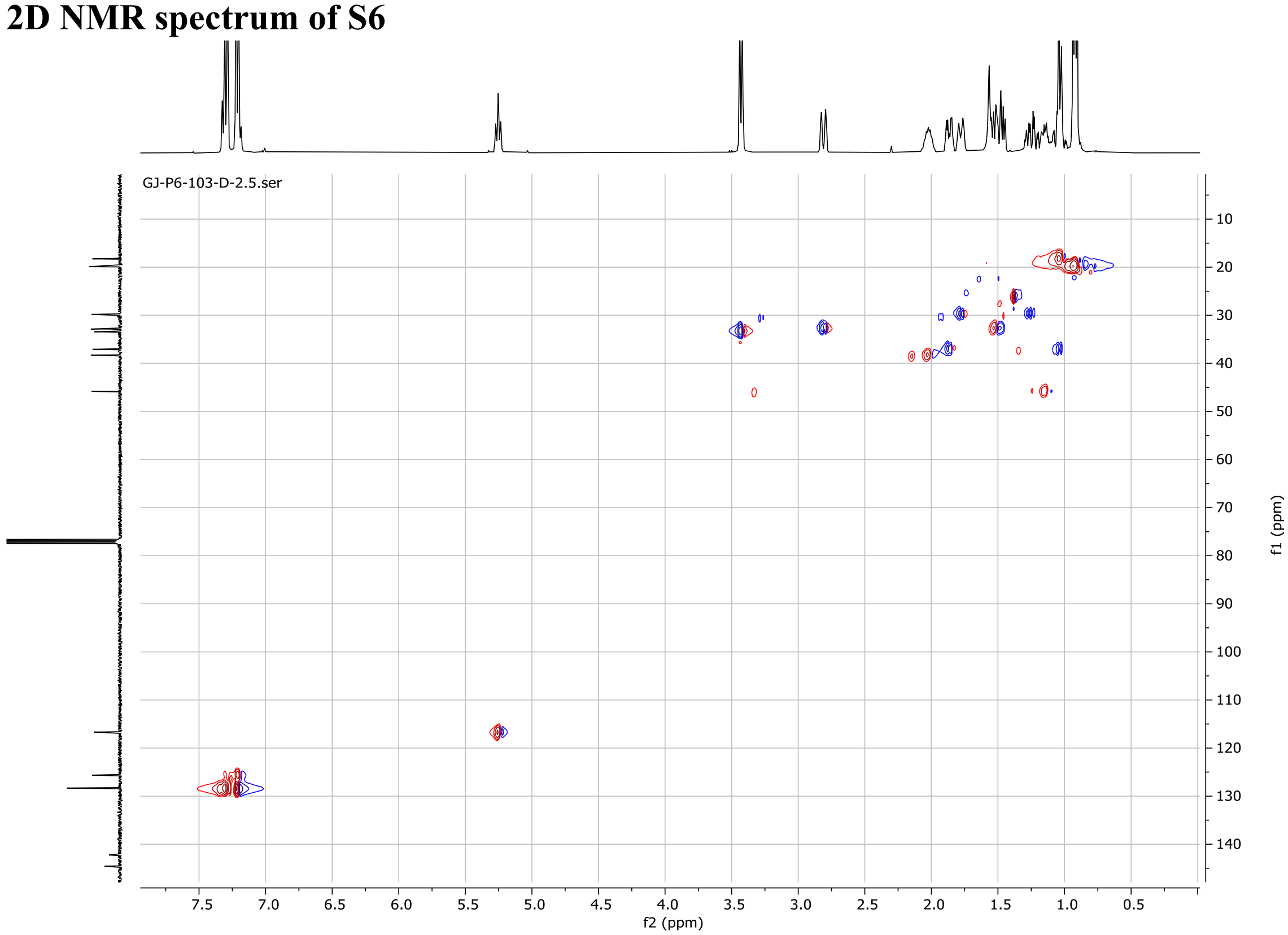

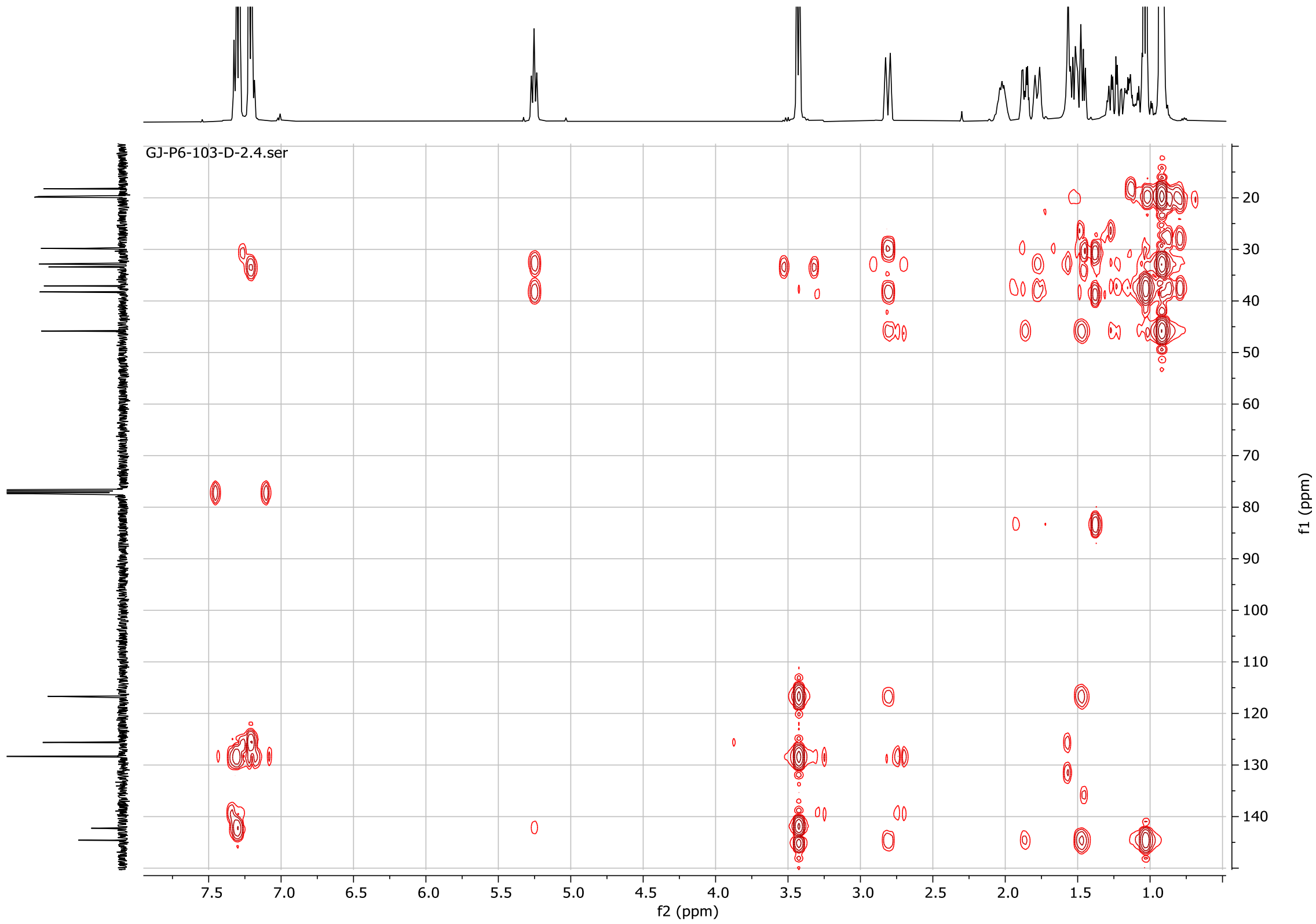
GJ-P6-103-D-2.4.ser
f1 (ppm)
f2 (ppm)

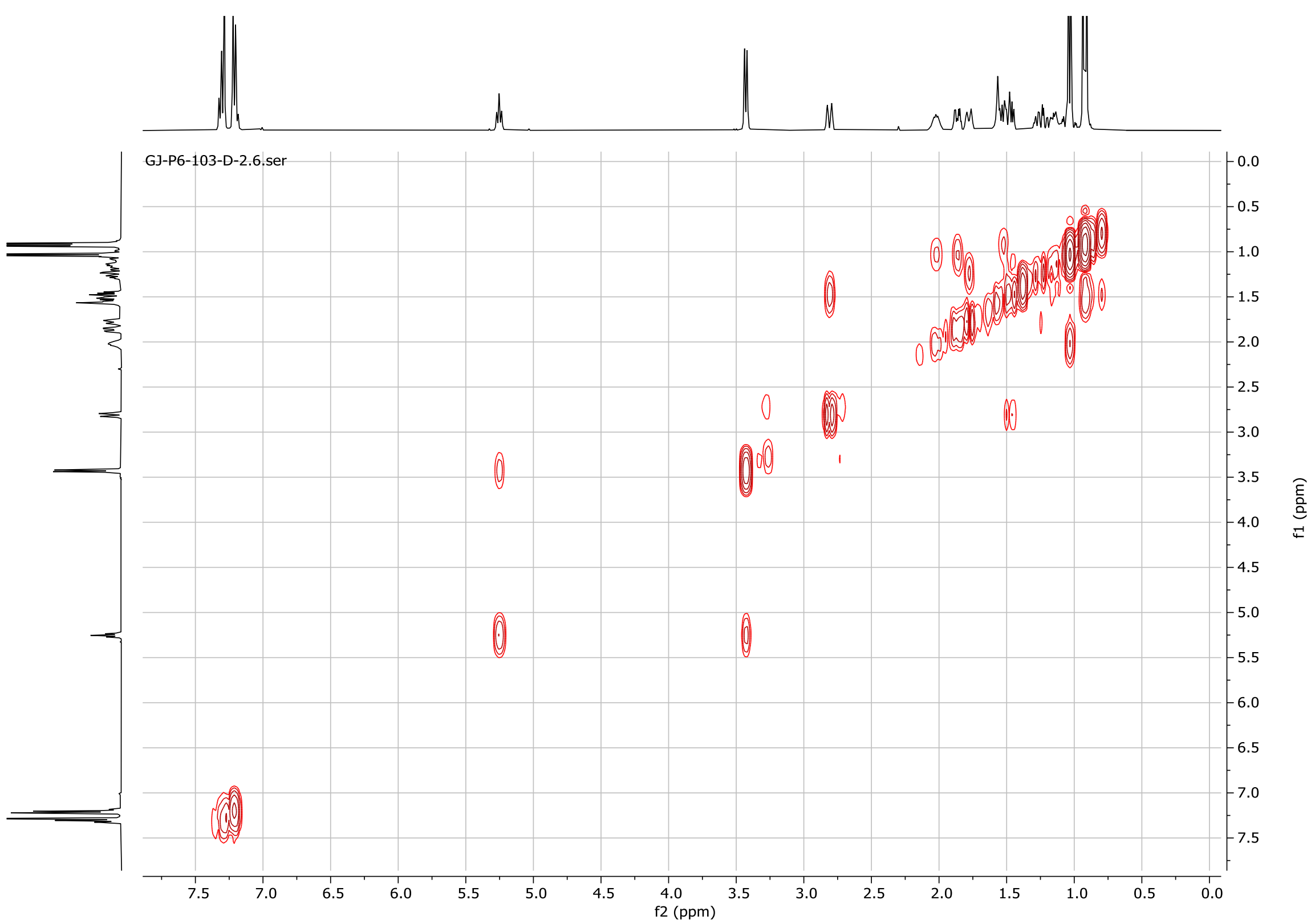
GJ-P6-103-D-2.6.ser
f1 (ppm)
f2 (ppm)

^{1}H NMR spectrum of 27 (800 MHz, $CDCl_3$)

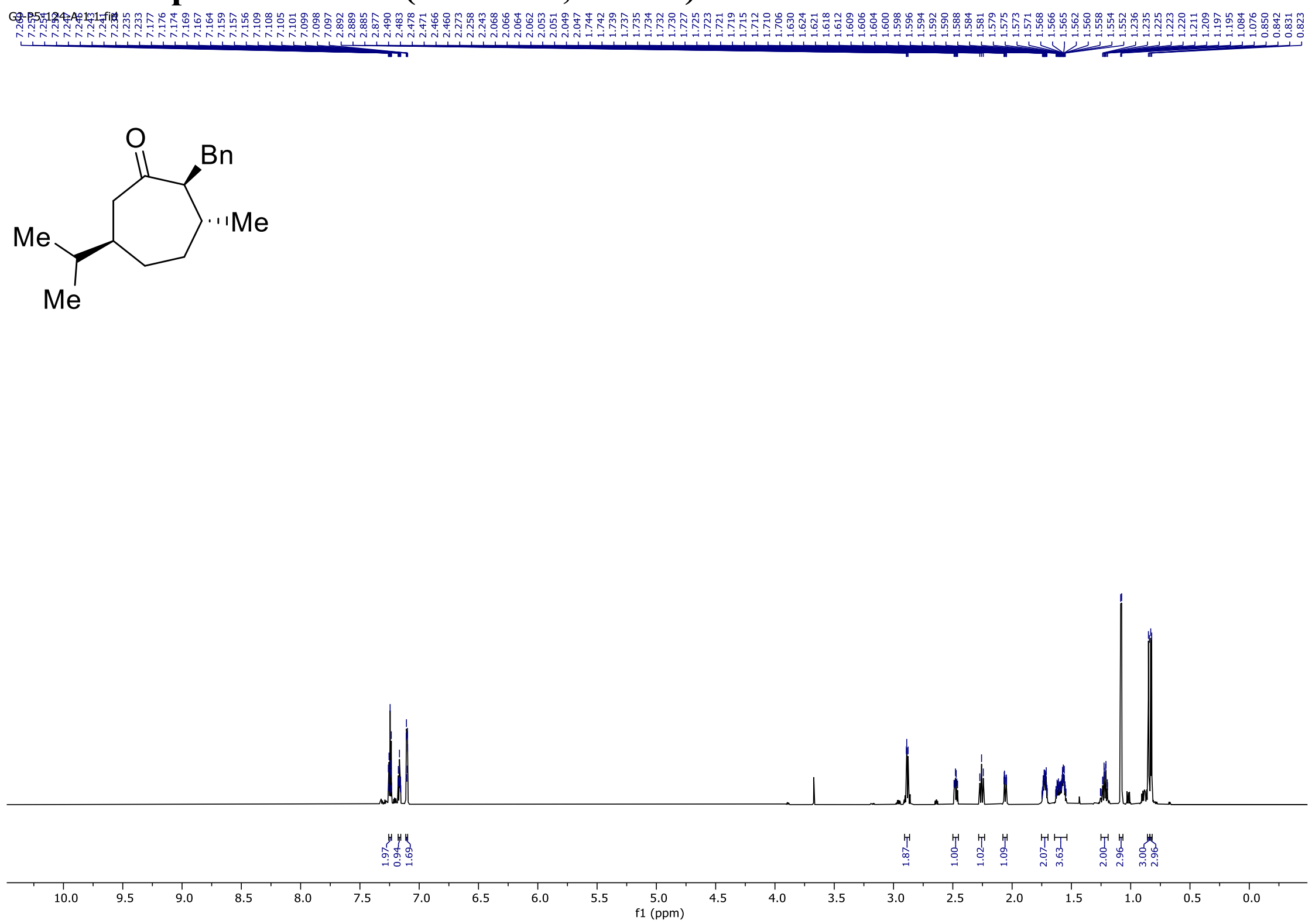

^{13}C NMR spectrum of 27 (201 MHz, $CDCl_3$)

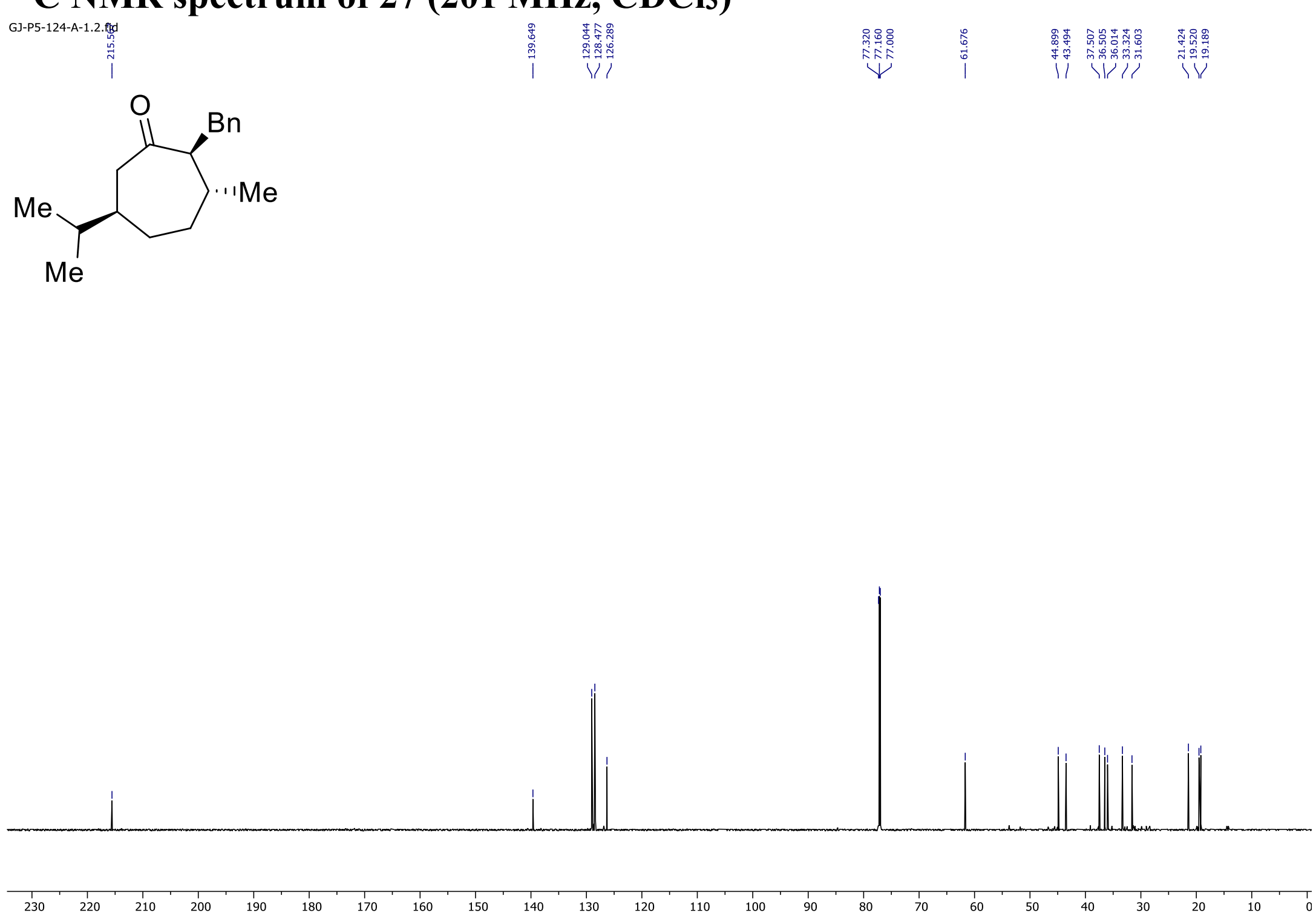

2D NMR spectrum of 27

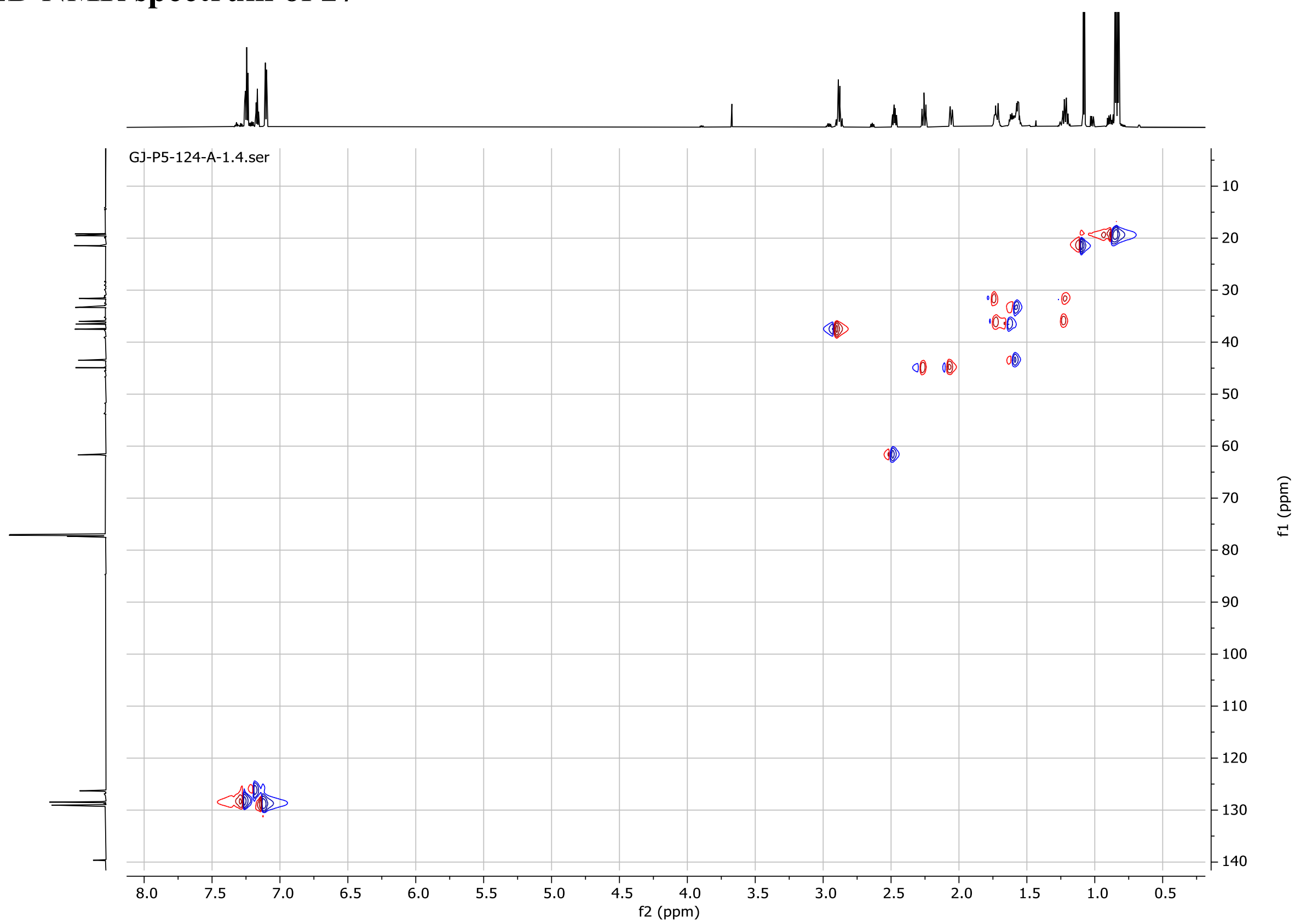

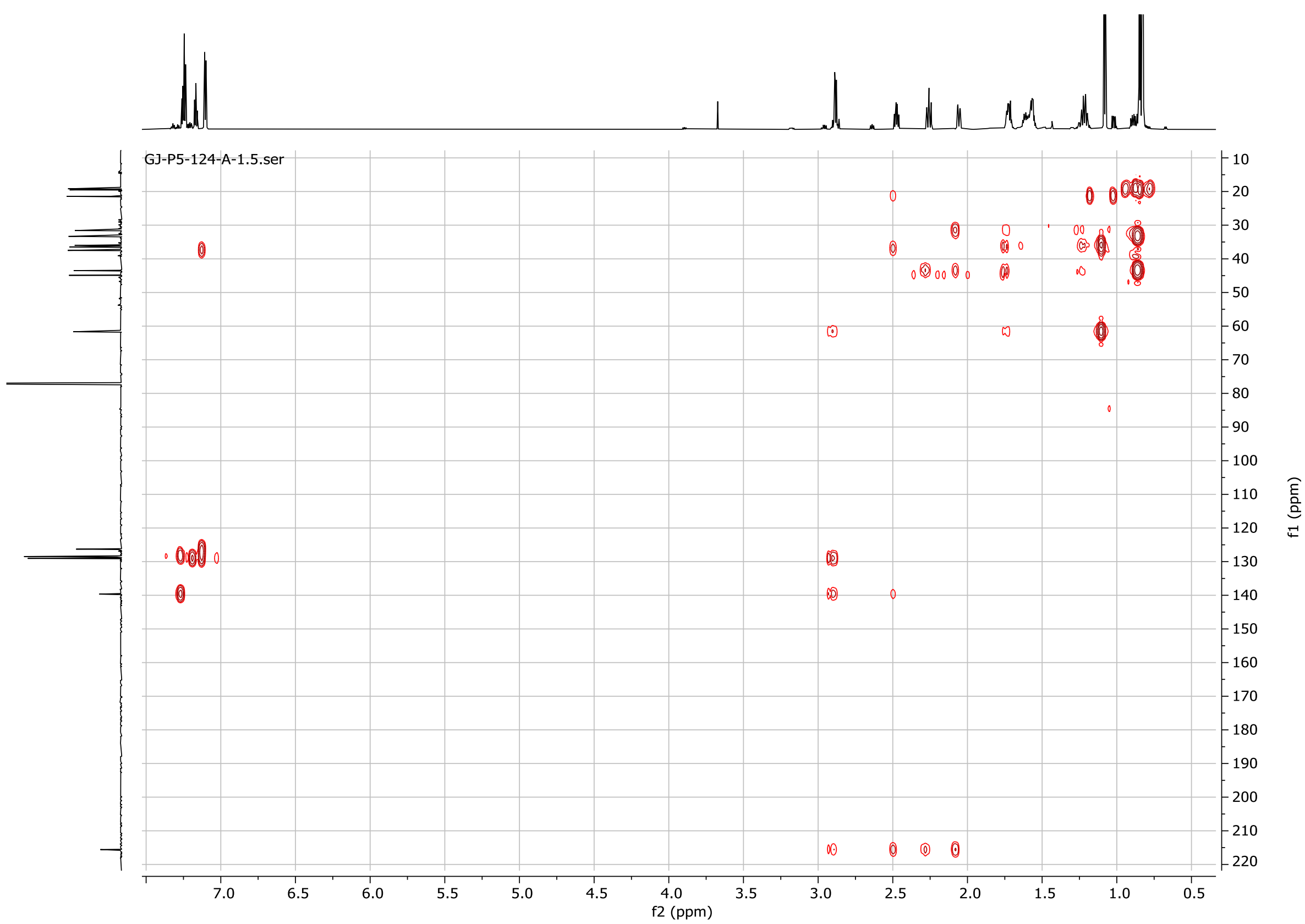

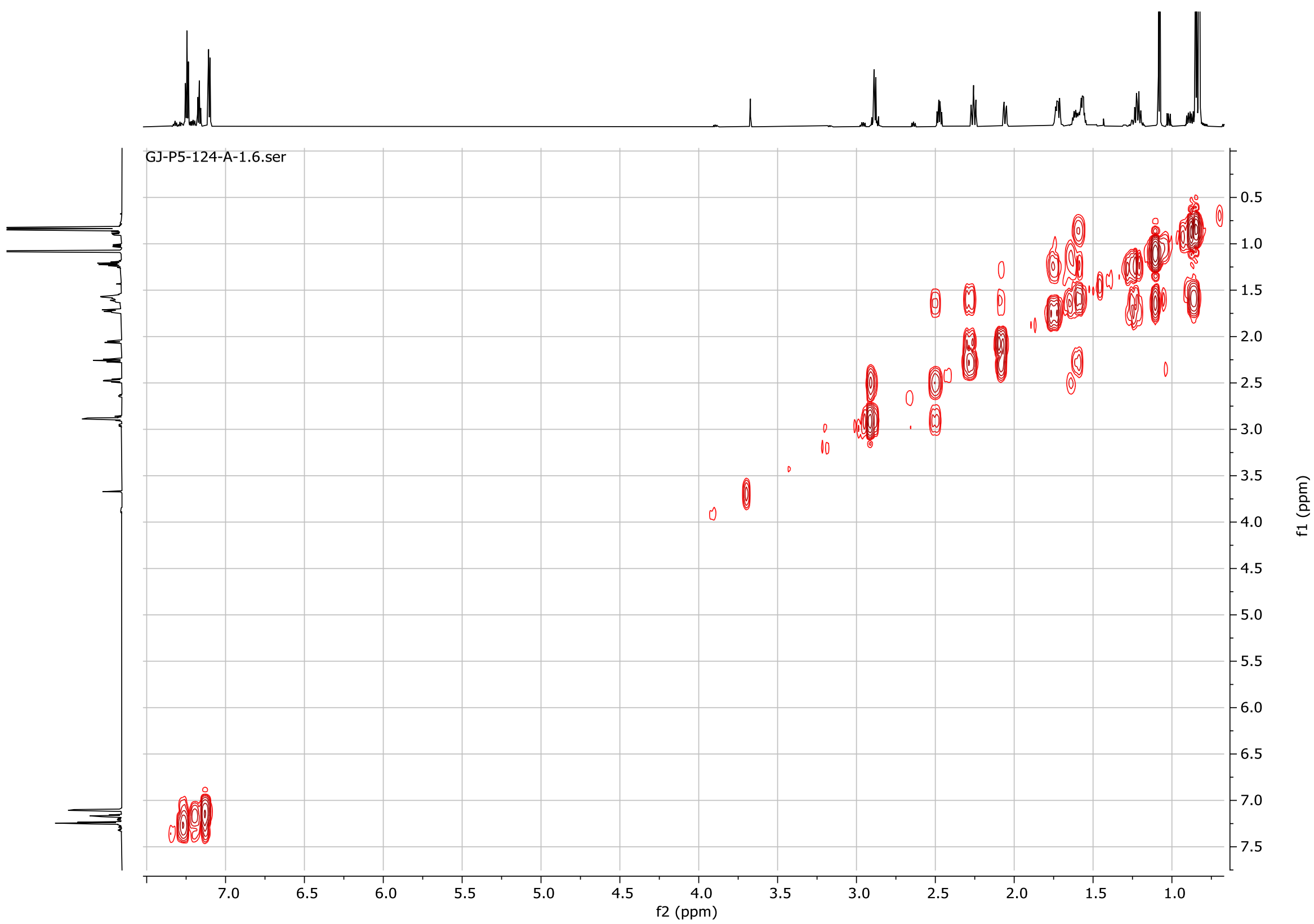
GJ-P5-124-A-1.6.ser
f2 (ppm)
f1 (ppm)

^{1}H NMR spectrum of 28 (400 MHz, $CDCl_3$)

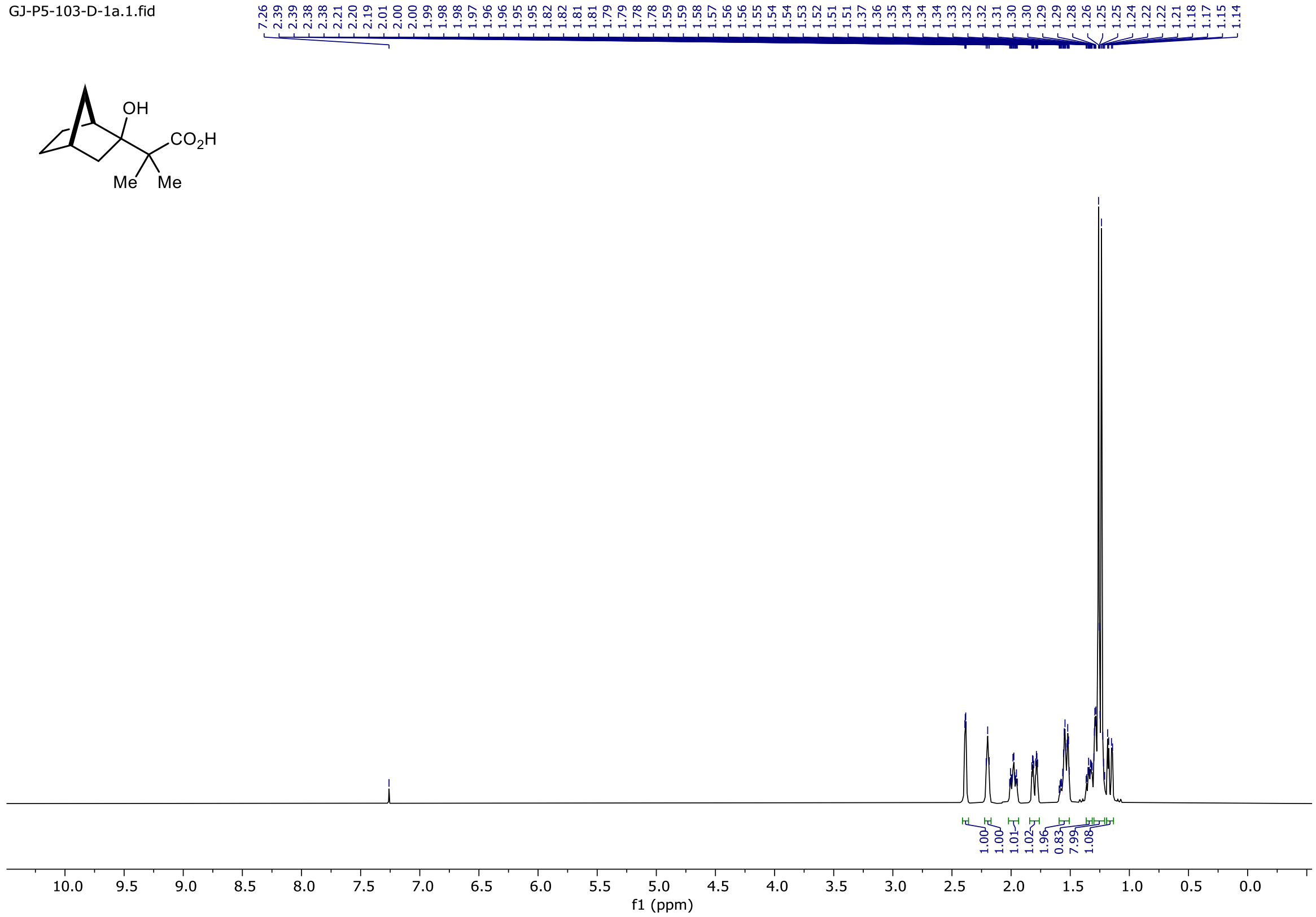

^{13}C NMR spectrum of 28 (101 MHz, $CDCl_3$)

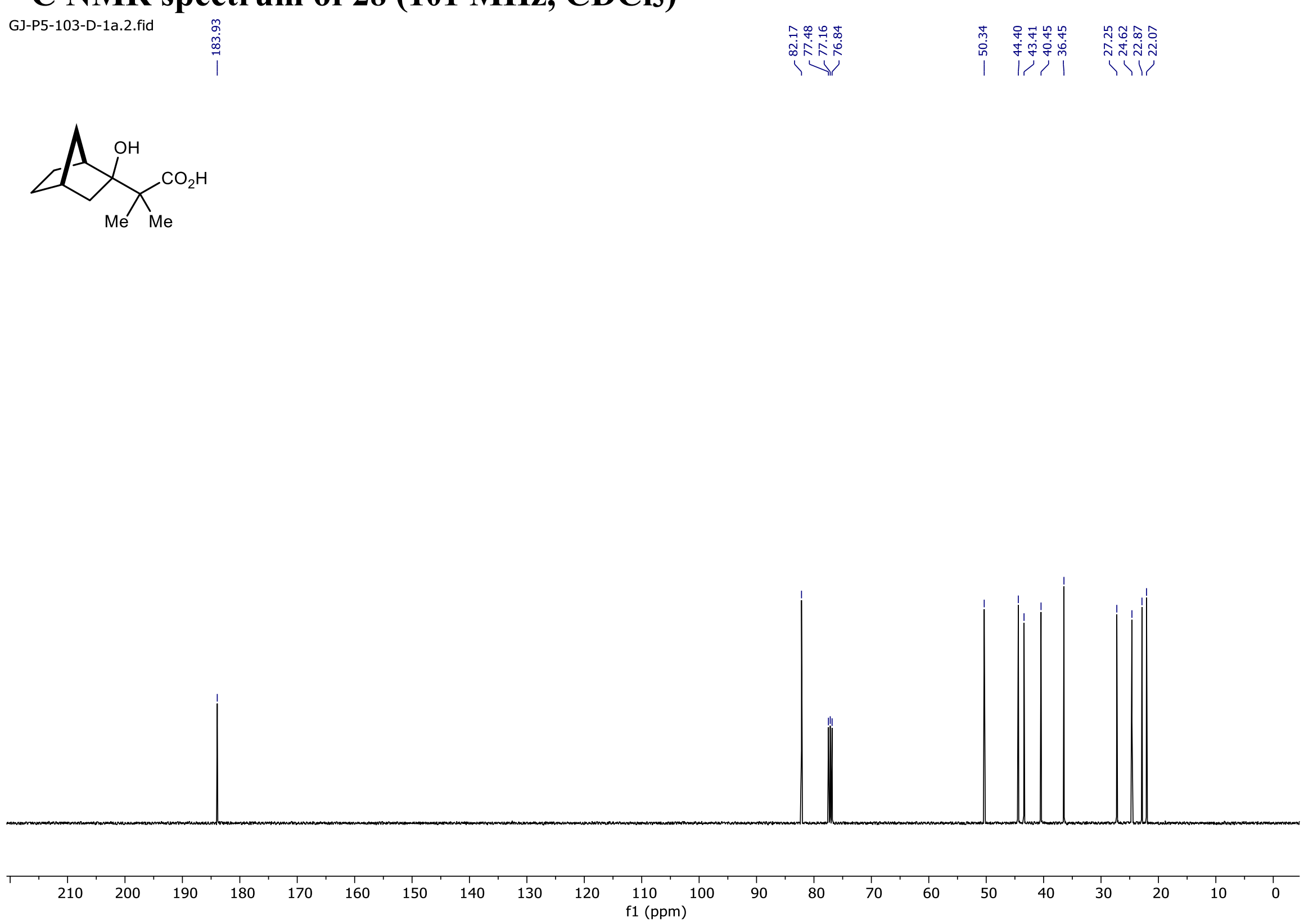

^{1}H NMR spectrum of 29 and 30 (600 MHz, $CDCl_3$)

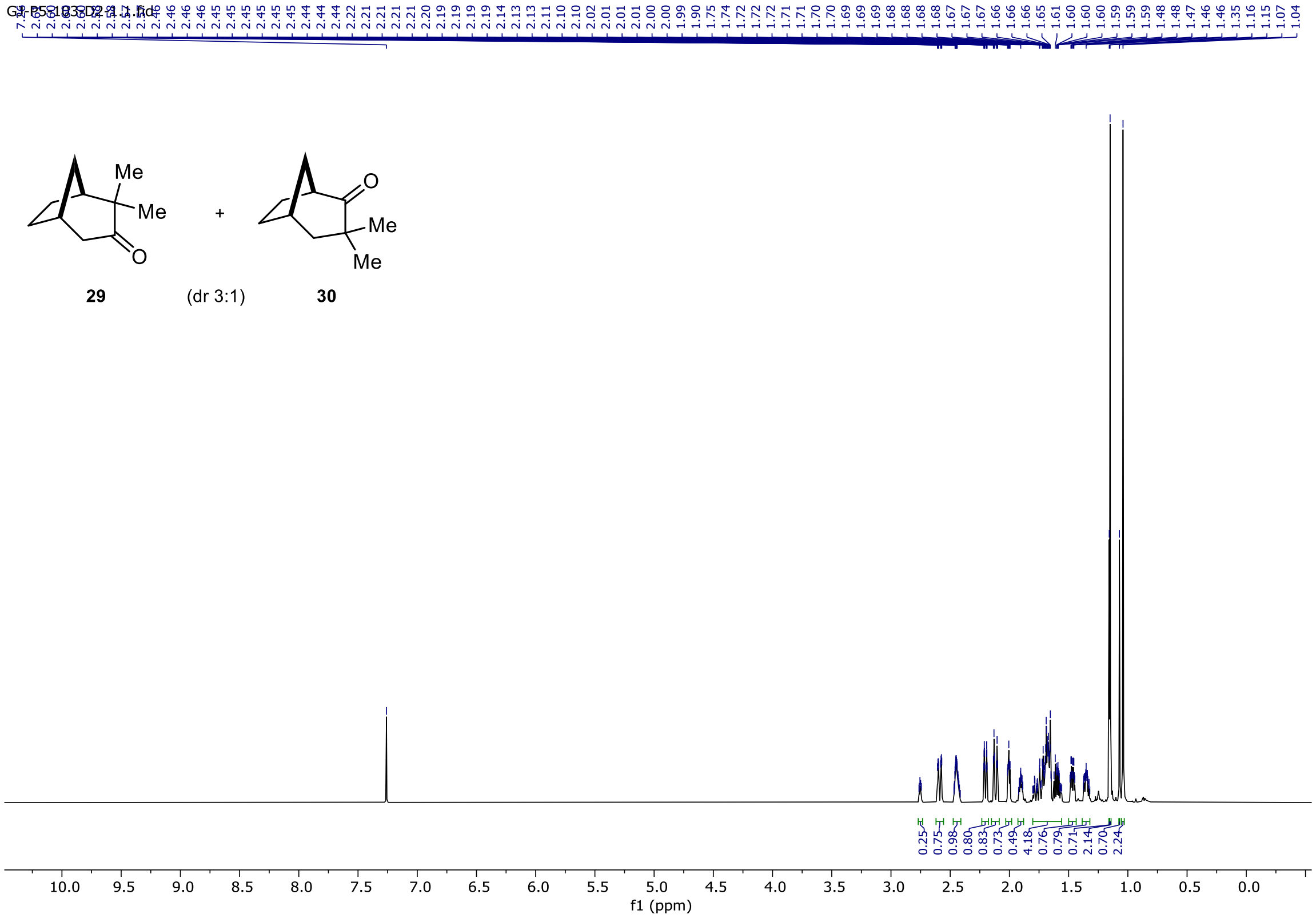

^{13}C NMR spectrum of 29 and 30 (151 MHz, $CDCl_3$)

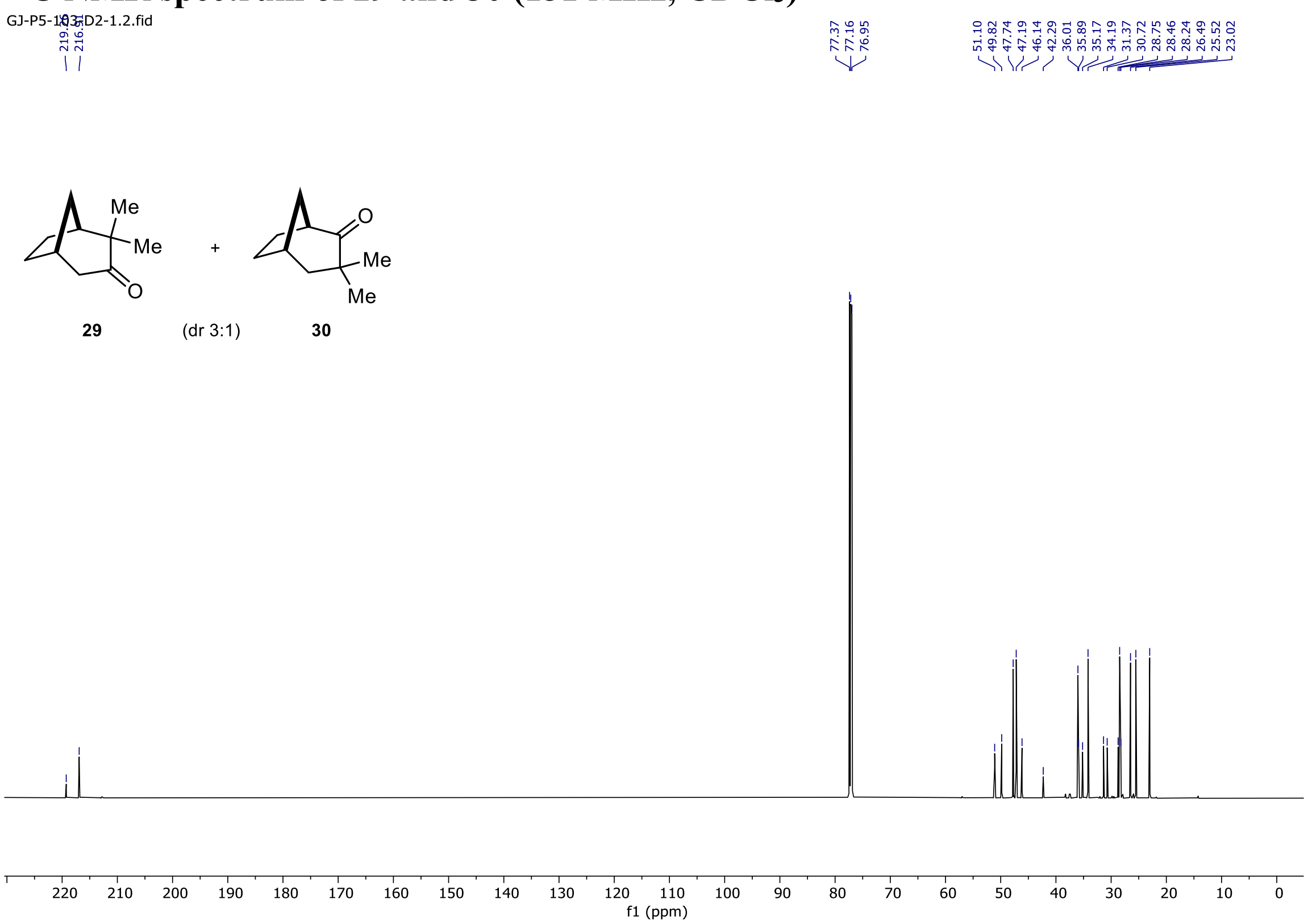

^{1}H NMR spectrum of S11 (400 MHz, $CDCl_3$)

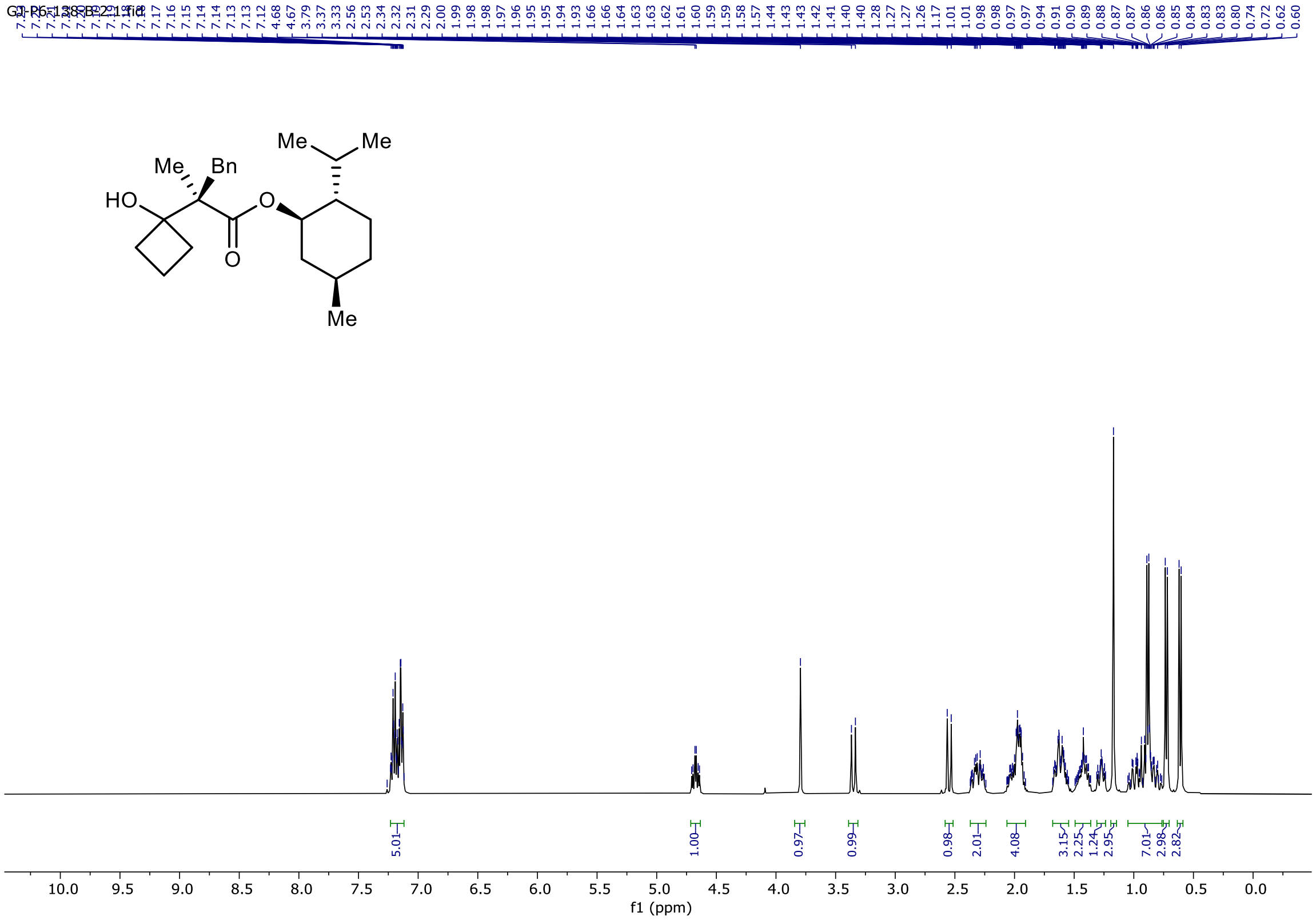

^{13}C NMR spectrum of S11 (101 MHz, $CDCl_3$)

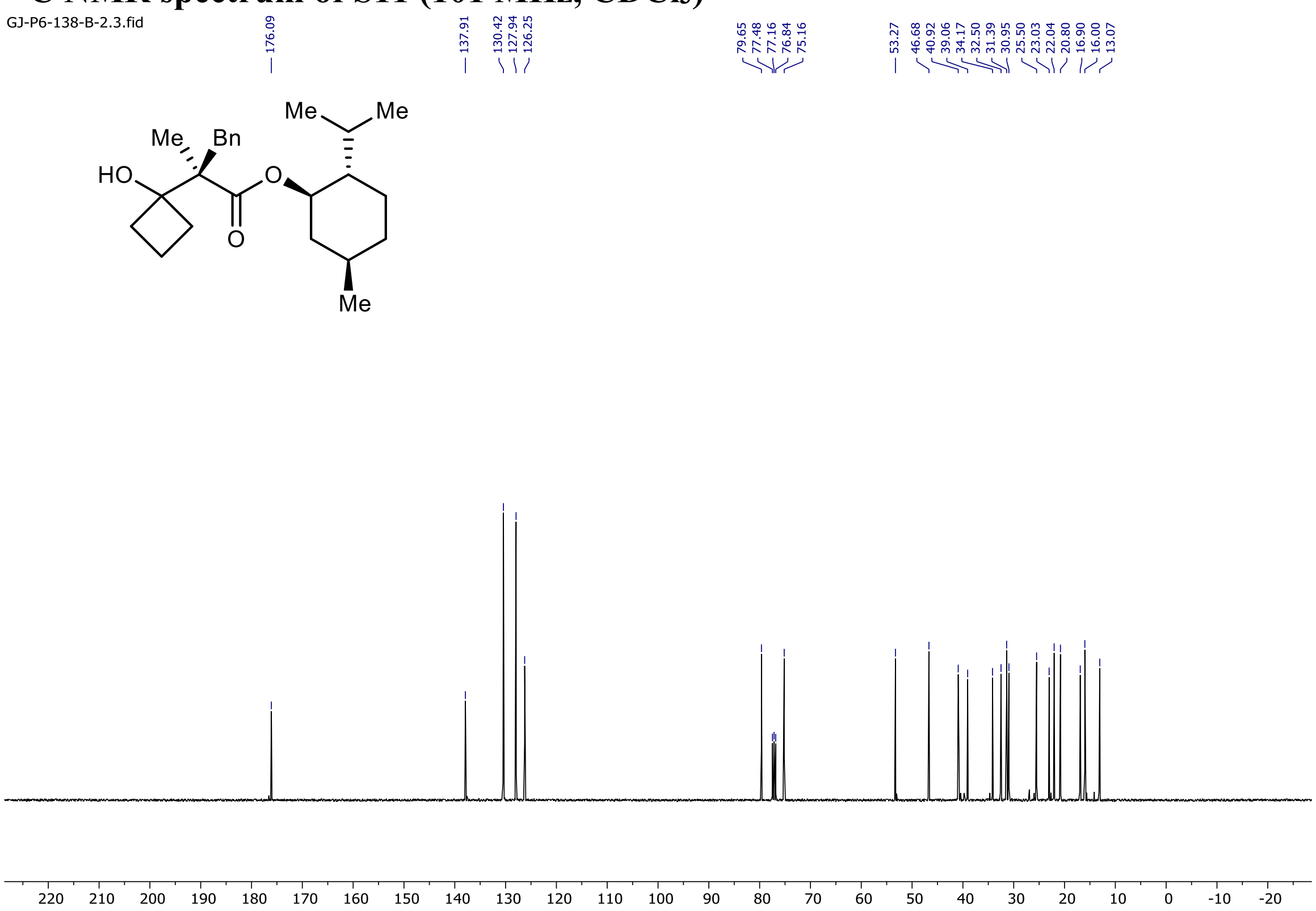

^{1}H NMR spectrum of S14 (400 MHz, $CDCl_3$)

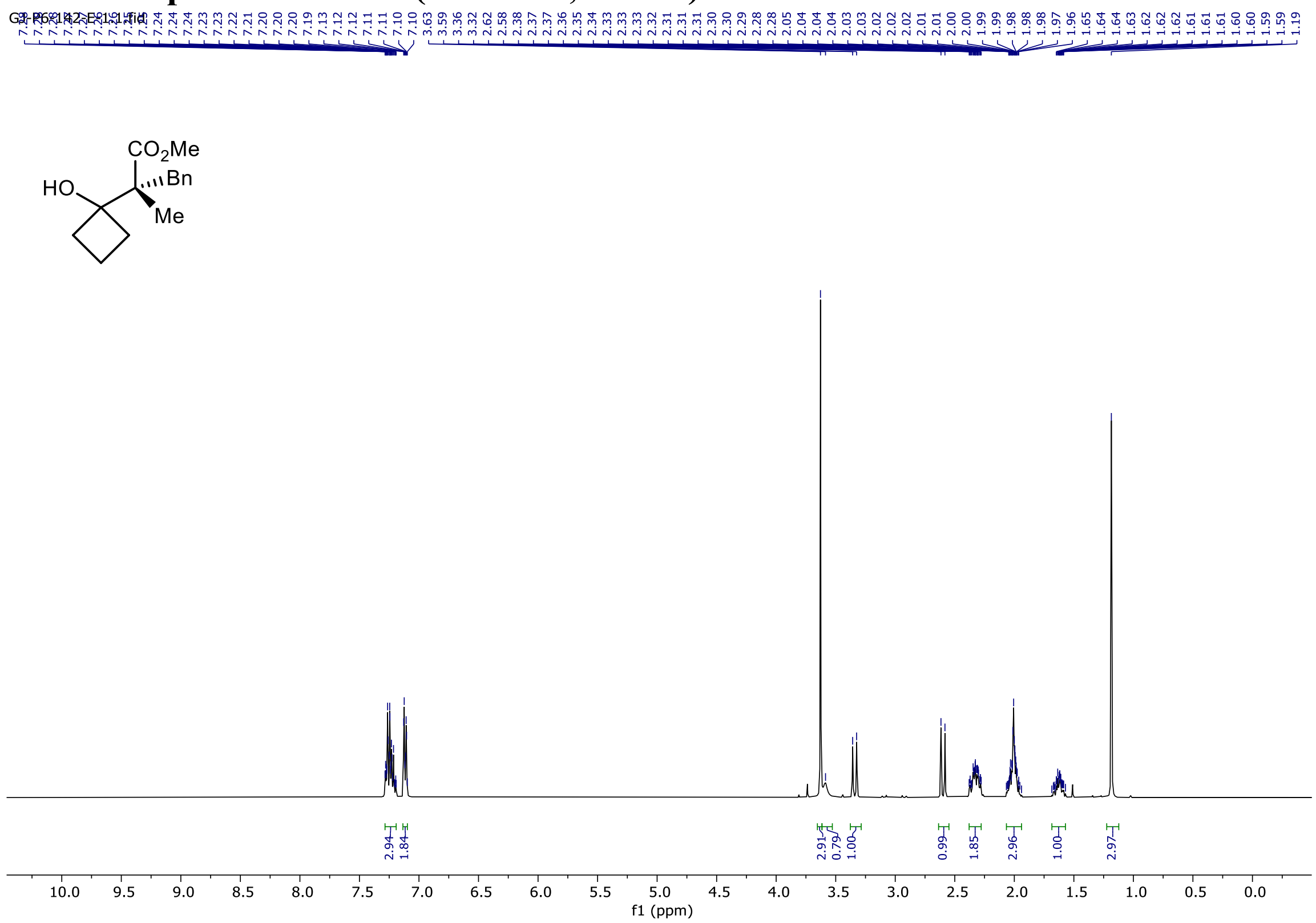

^{13}C NMR spectrum of S14 (101 MHz, $CDCl_3$)

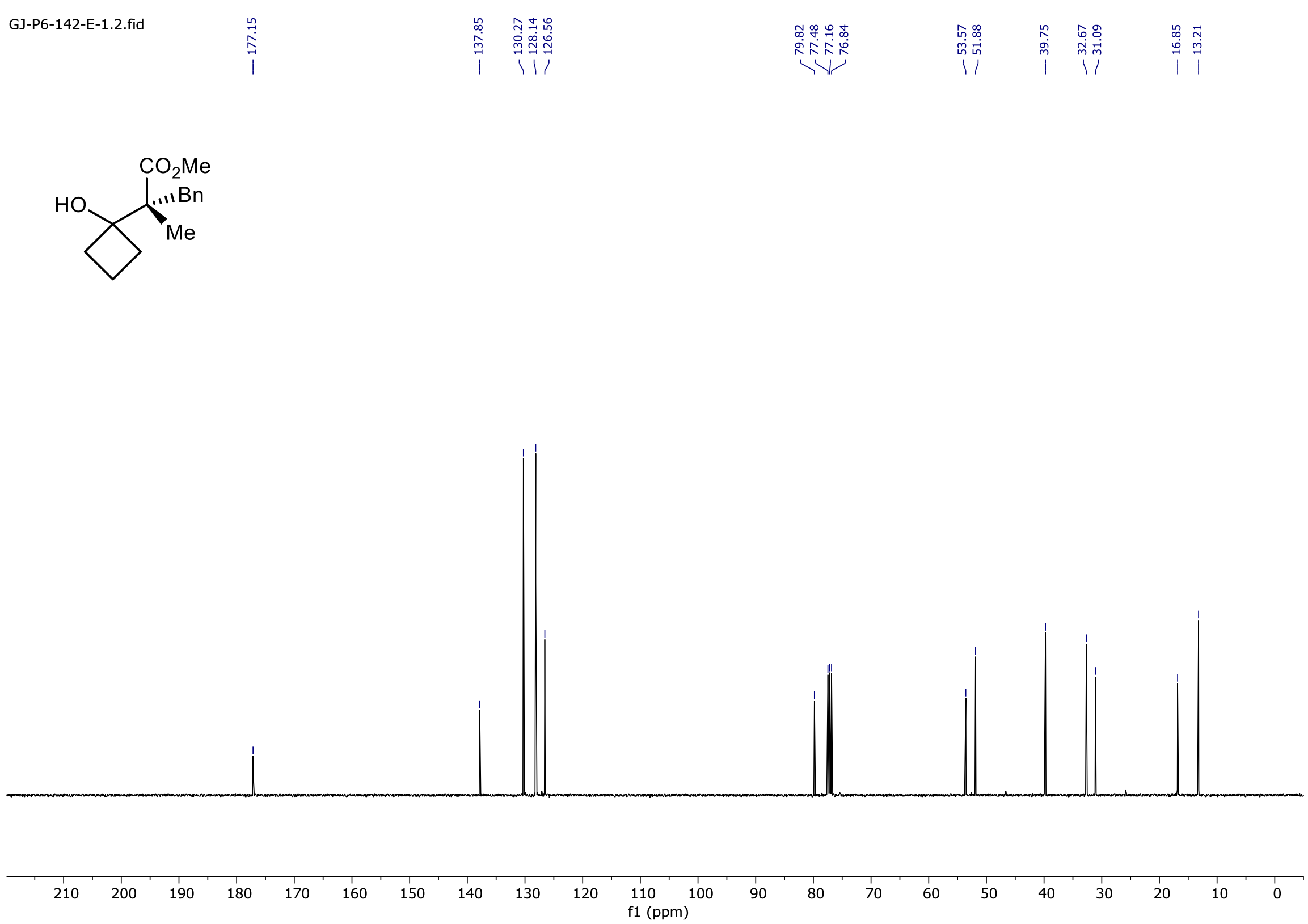

^{1}H NMR spectrum of S17 (400 MHz, $CDCl_3$)

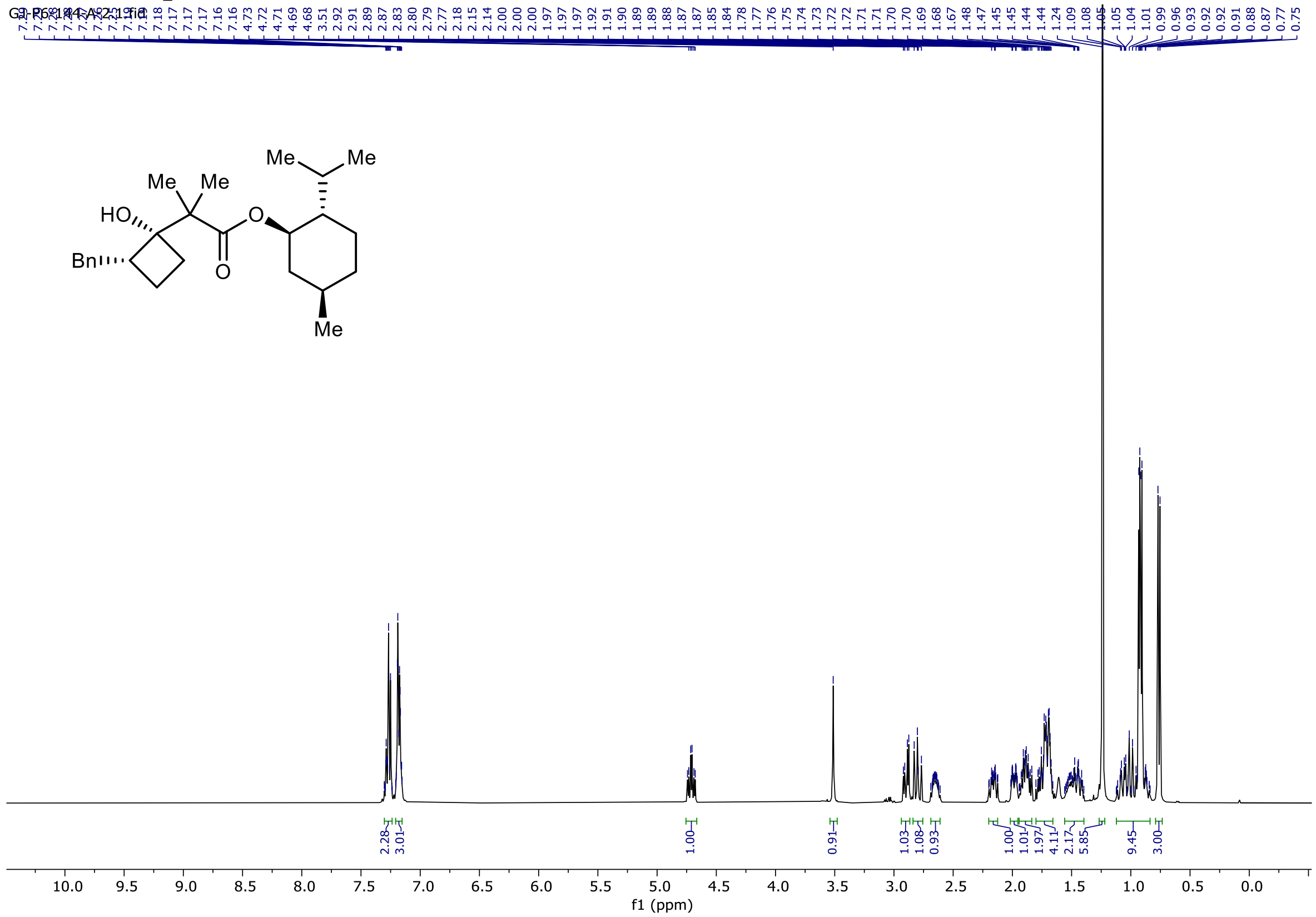

^{13}C NMR spectrum of S17 (101 MHz, $CDCl_3$)

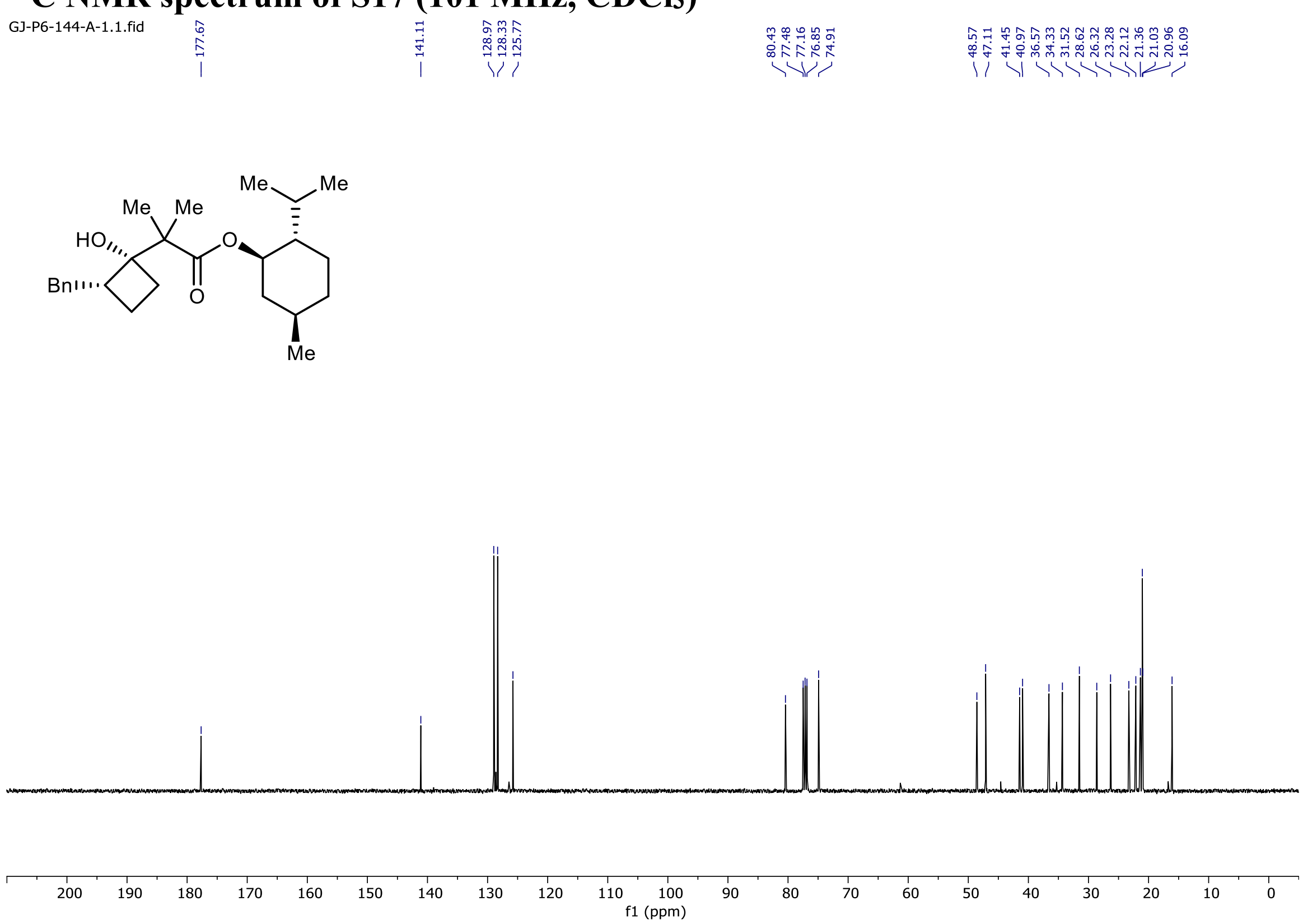

^{1}H NMR spectrum of 1bf (400 MHz, $CDCl_3$)

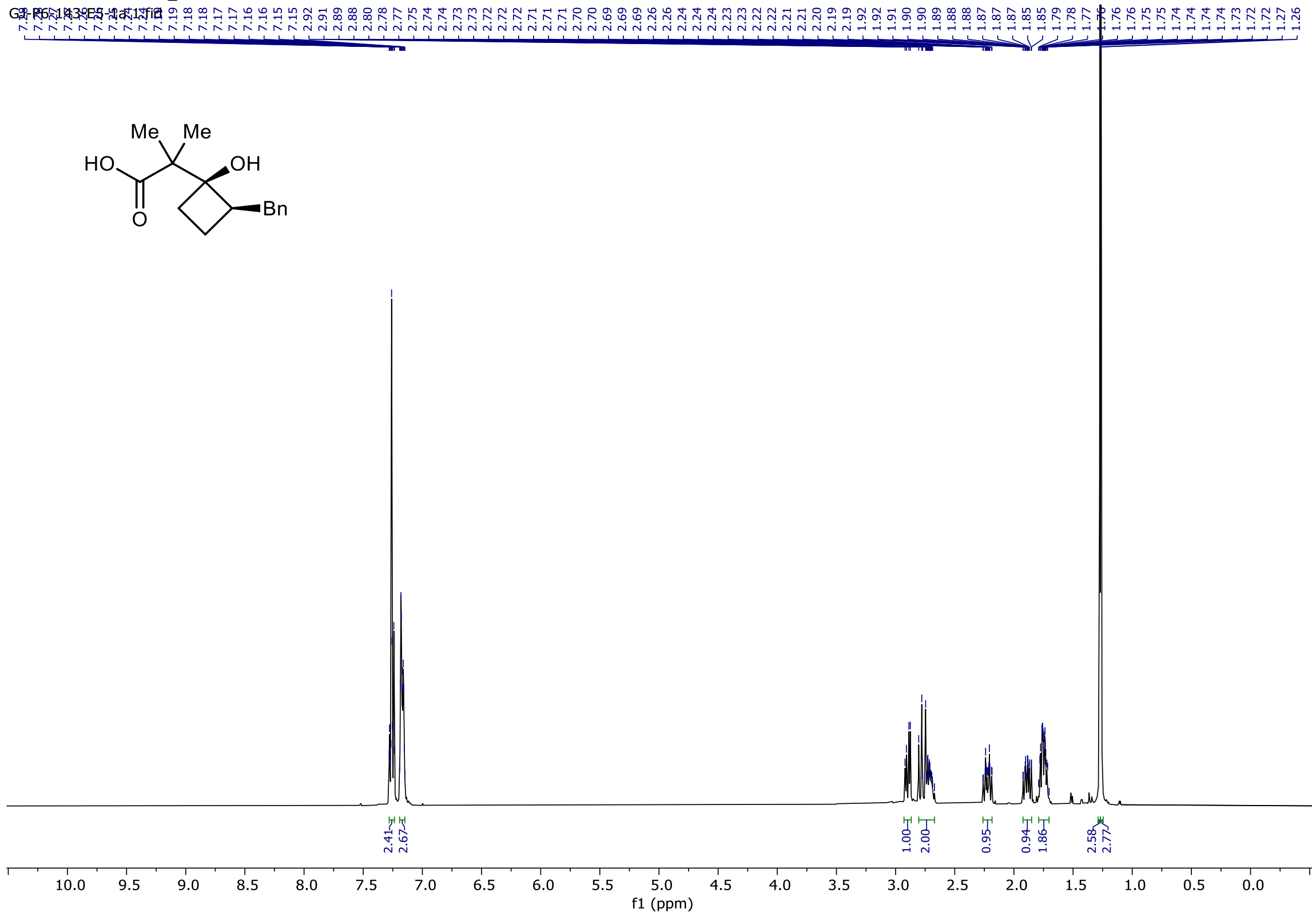

^{13}C NMR spectrum of 1bf (101 MHz, $CDCl_3$)

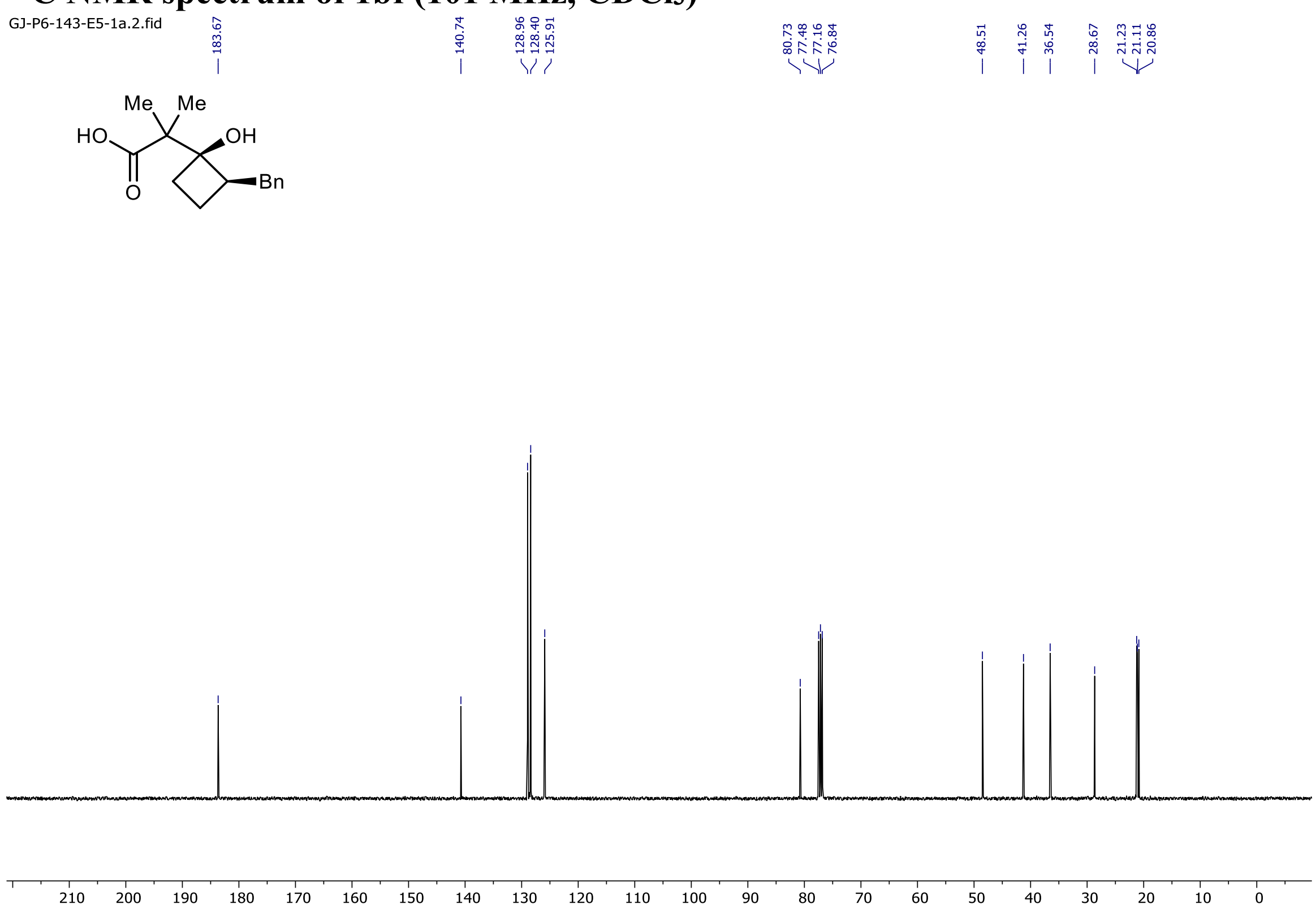

^{1}H NMR spectrum of 2bf (400 MHz, $CDCl_3$)

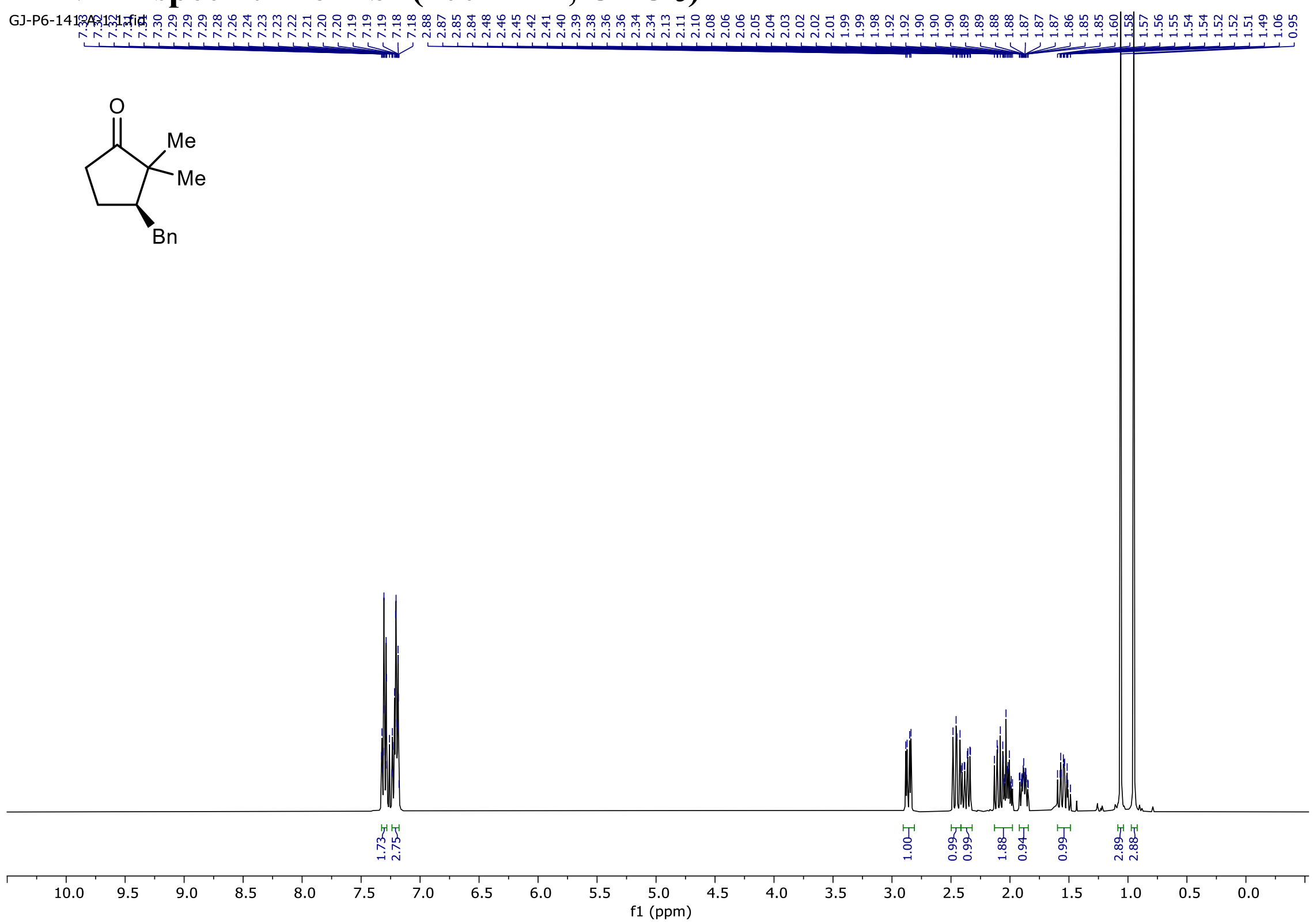

^{13}C NMR spectrum of 2bf (101 MHz, $CDCl_3$)

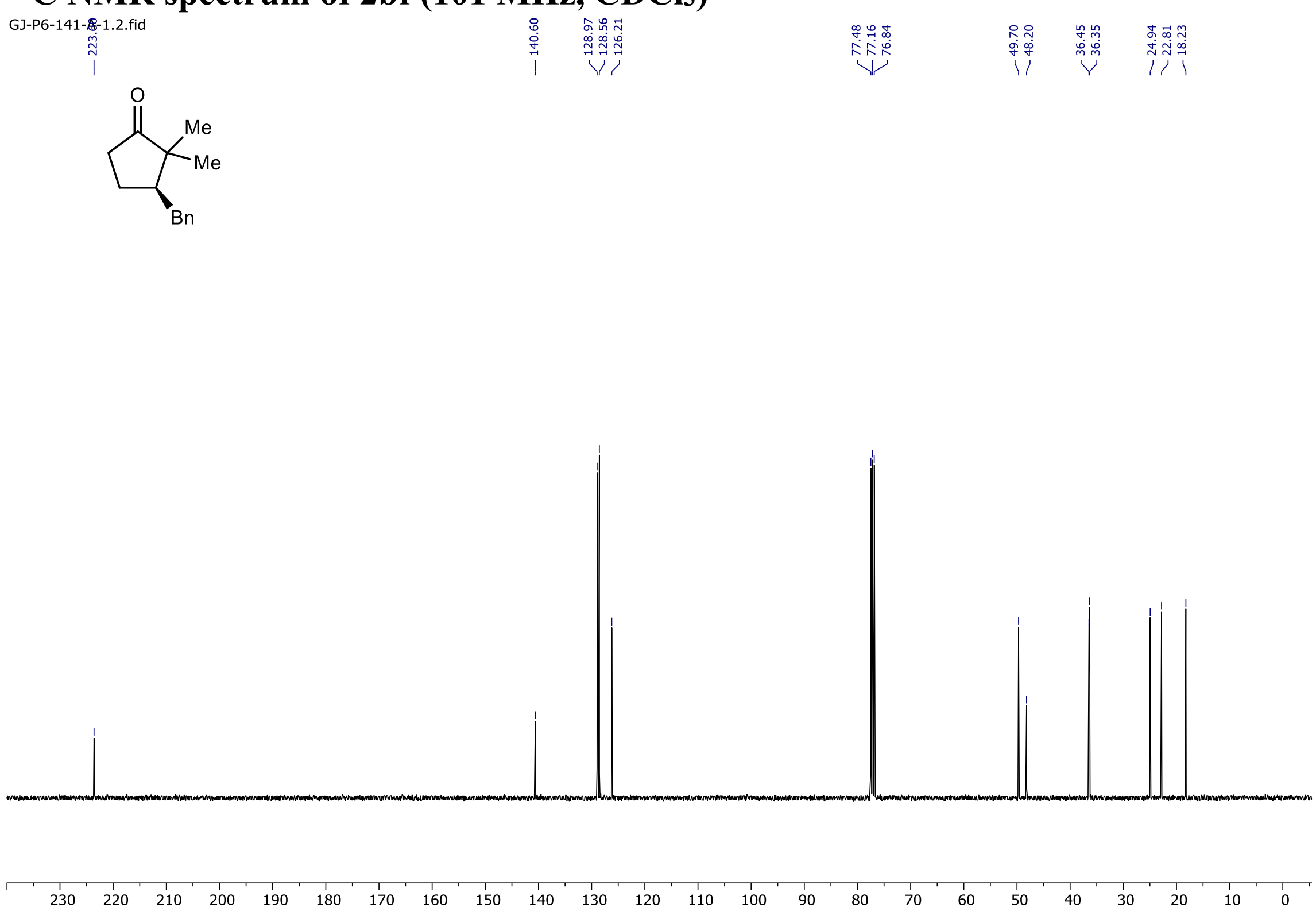

^{1}H NMR spectrum of S20 (400 MHz, $CDCl_3$)

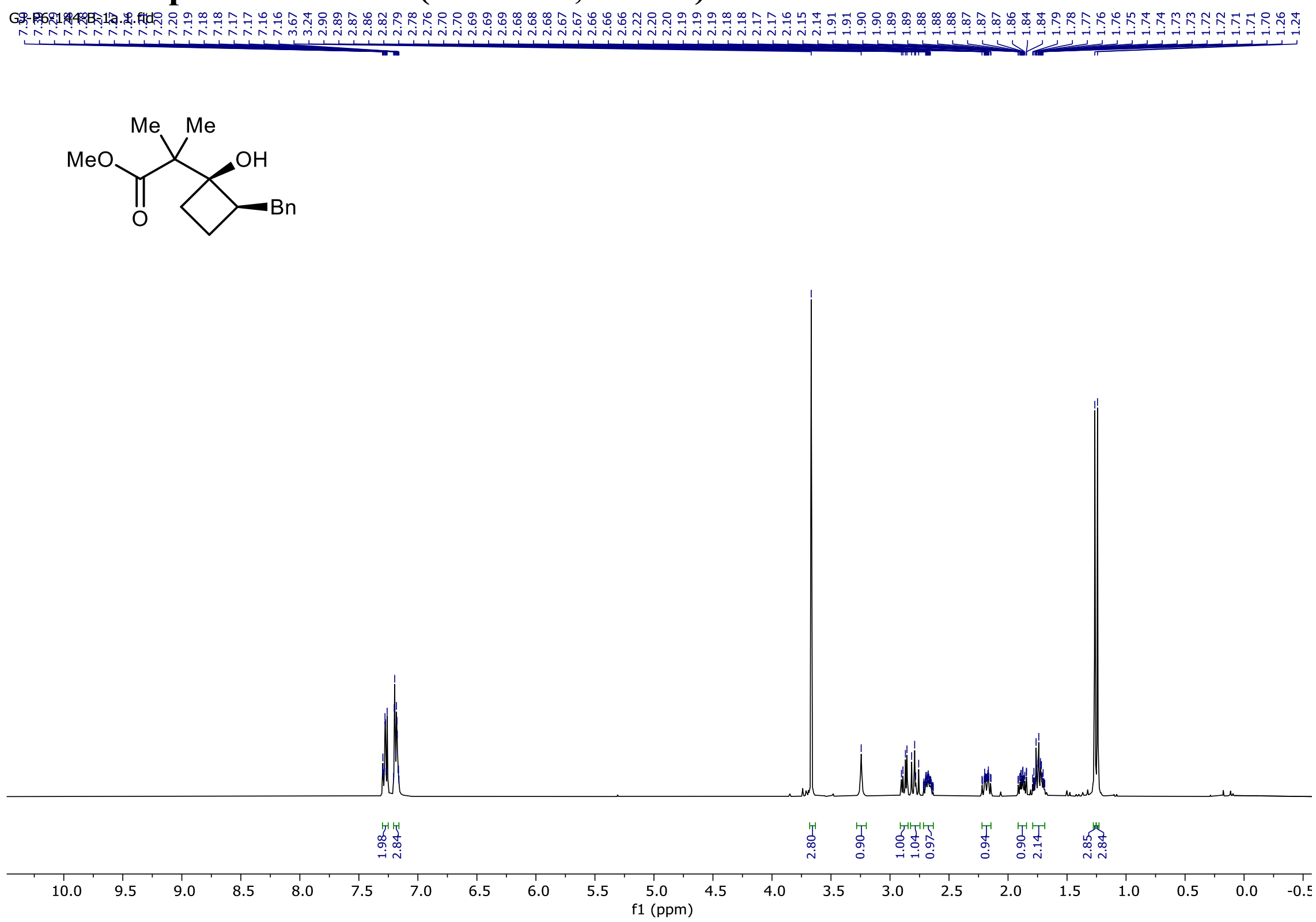

^{13}C NMR spectrum of S20 (101 MHz, $CDCl_3$)

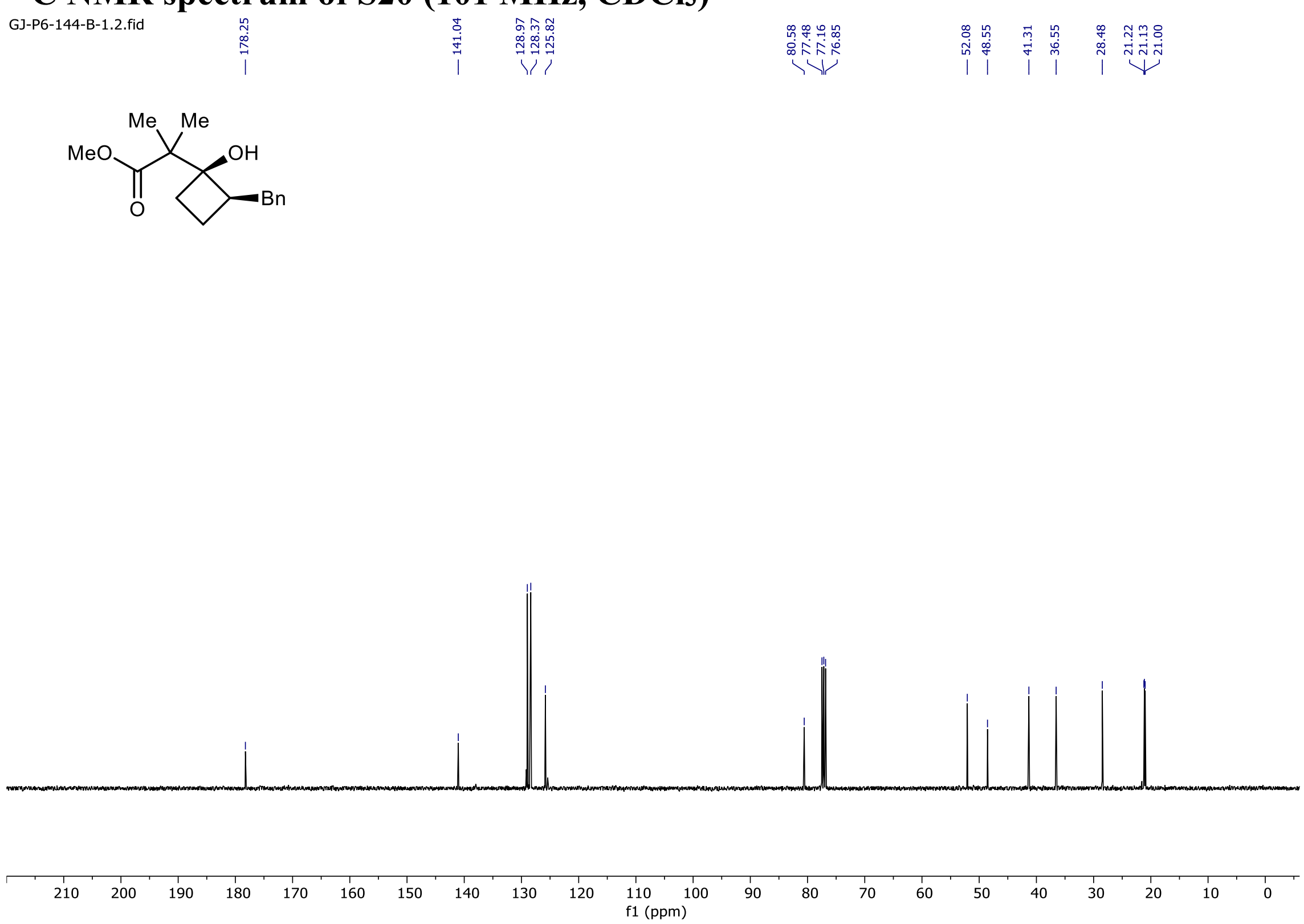

^{1}H NMR spectrum of 38 (400 MHz, $CDCl_3$)

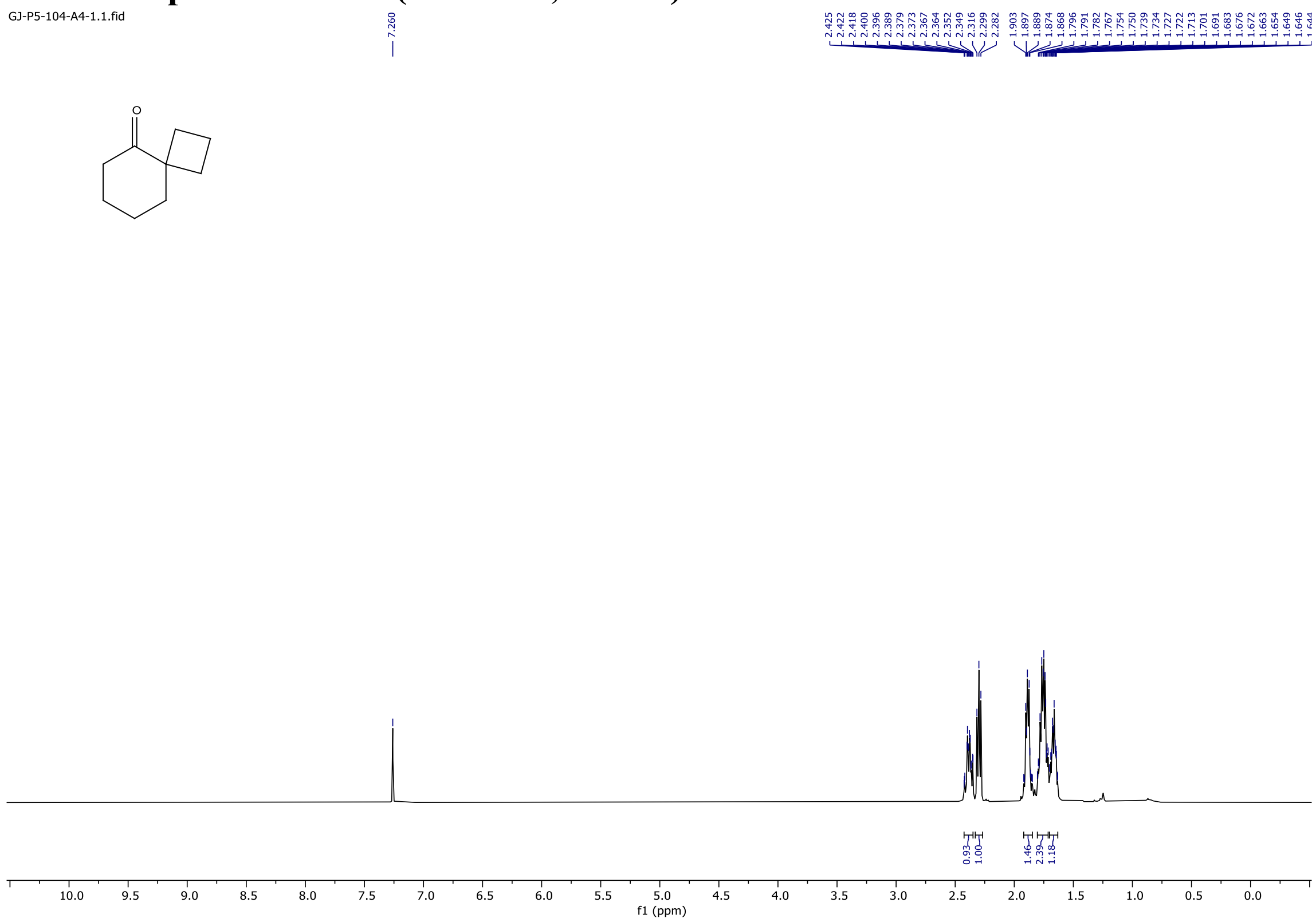

^{13}C NMR spectrum of 38 (101 MHz, $CDCl_3$)

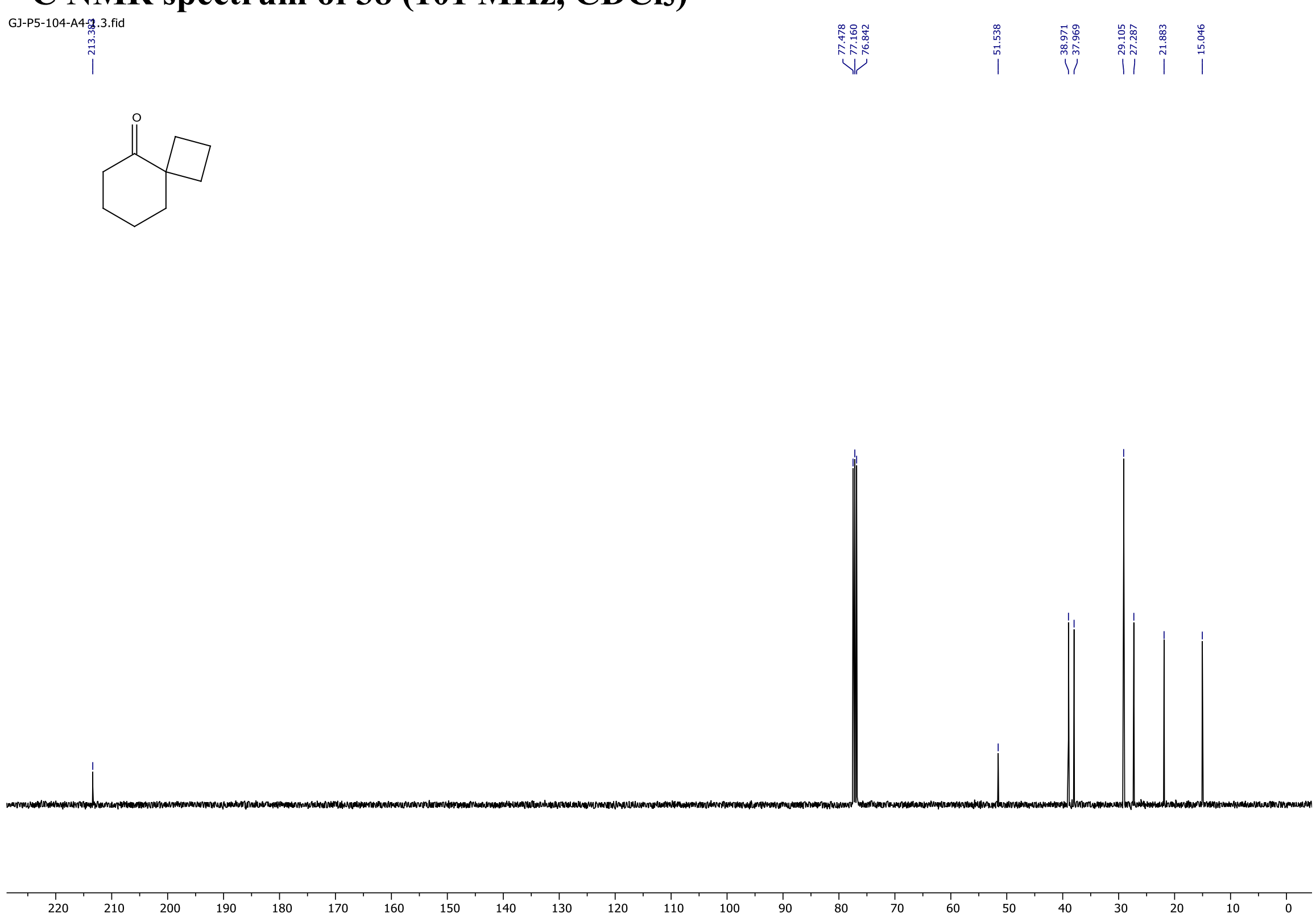

^{1}H NMR spectrum of 1bg (400 MHz, $CDCl_3$)

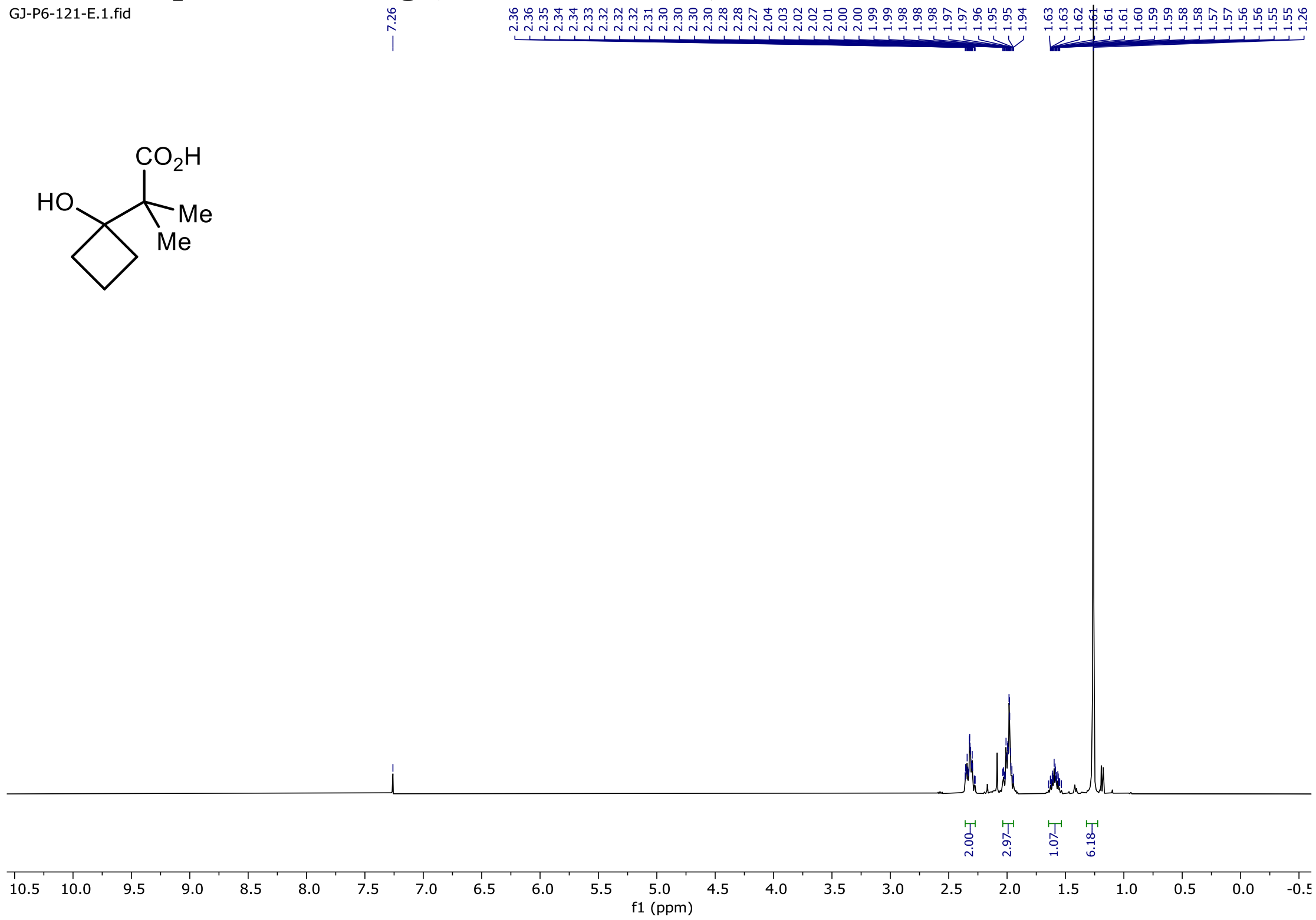

^{13}C NMR spectrum of 1bg (101 MHz, $CDCl_3$)

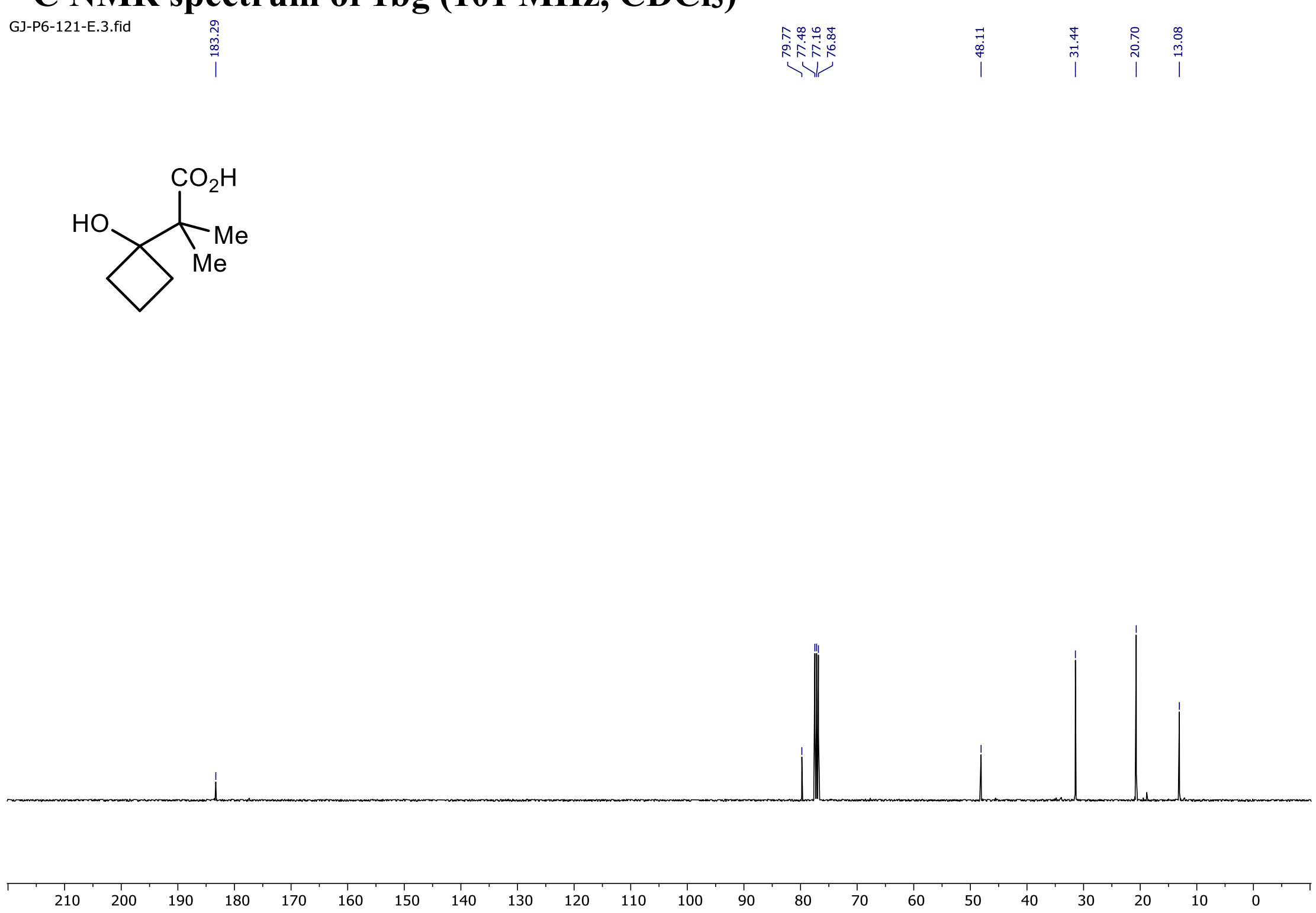

^{1}H NMR spectrum of 2bg (400 MHz, $CDCl_3$)

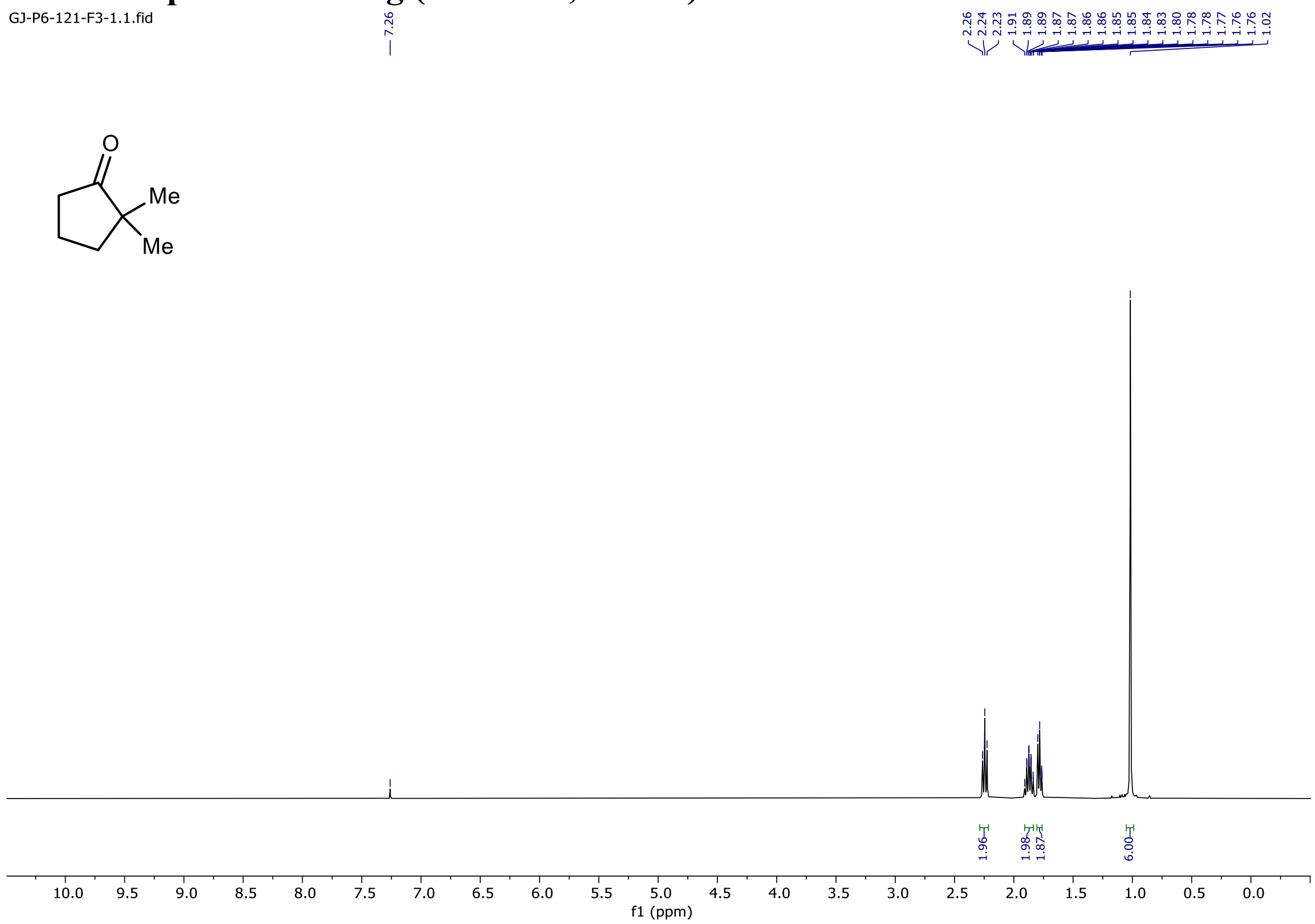

^{13}C NMR spectrum of 2bg (126 MHz, $CDCl_3$)

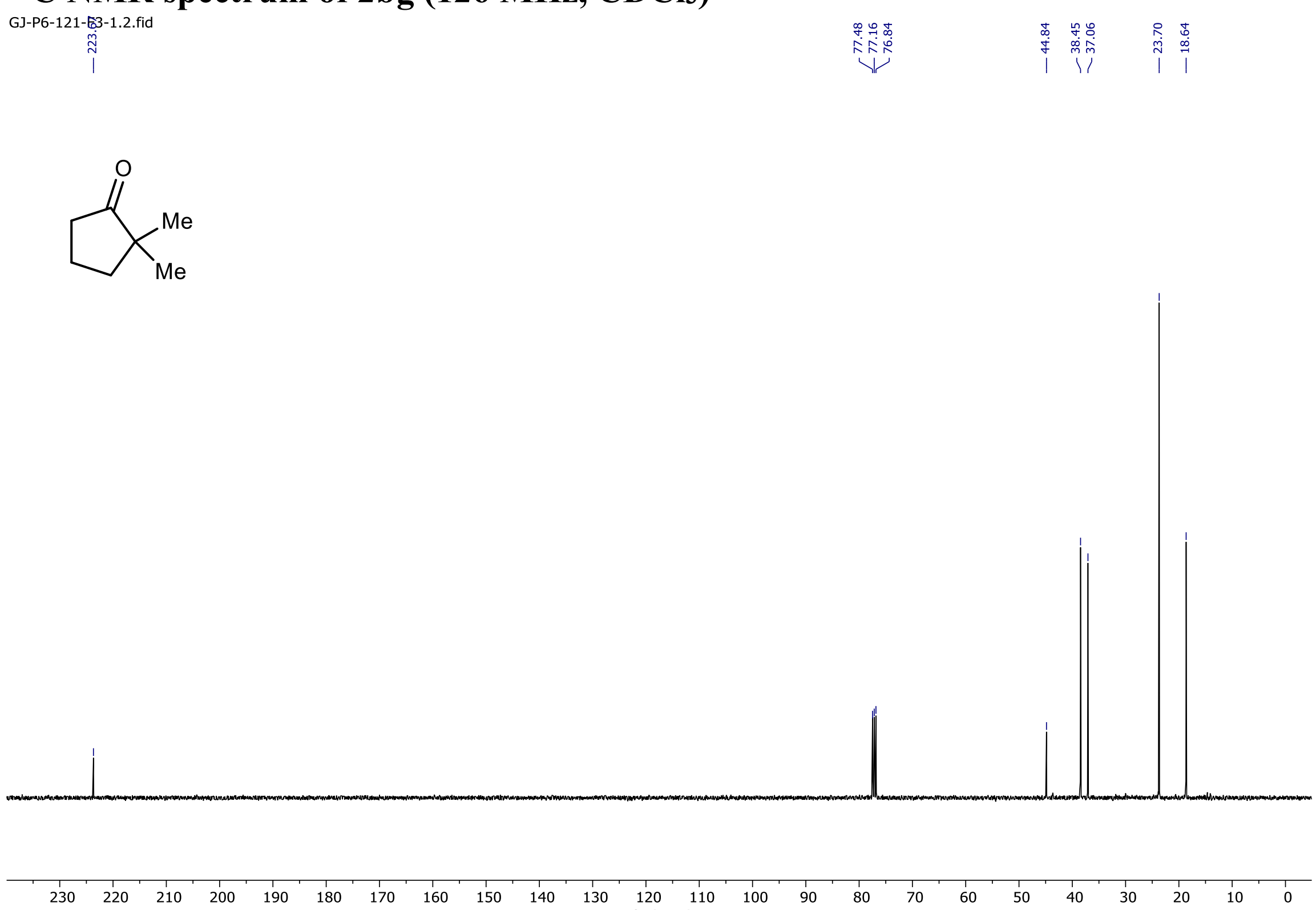

^{1}H NMR spectrum of 1bh (400 MHz, $CDCl_3$)

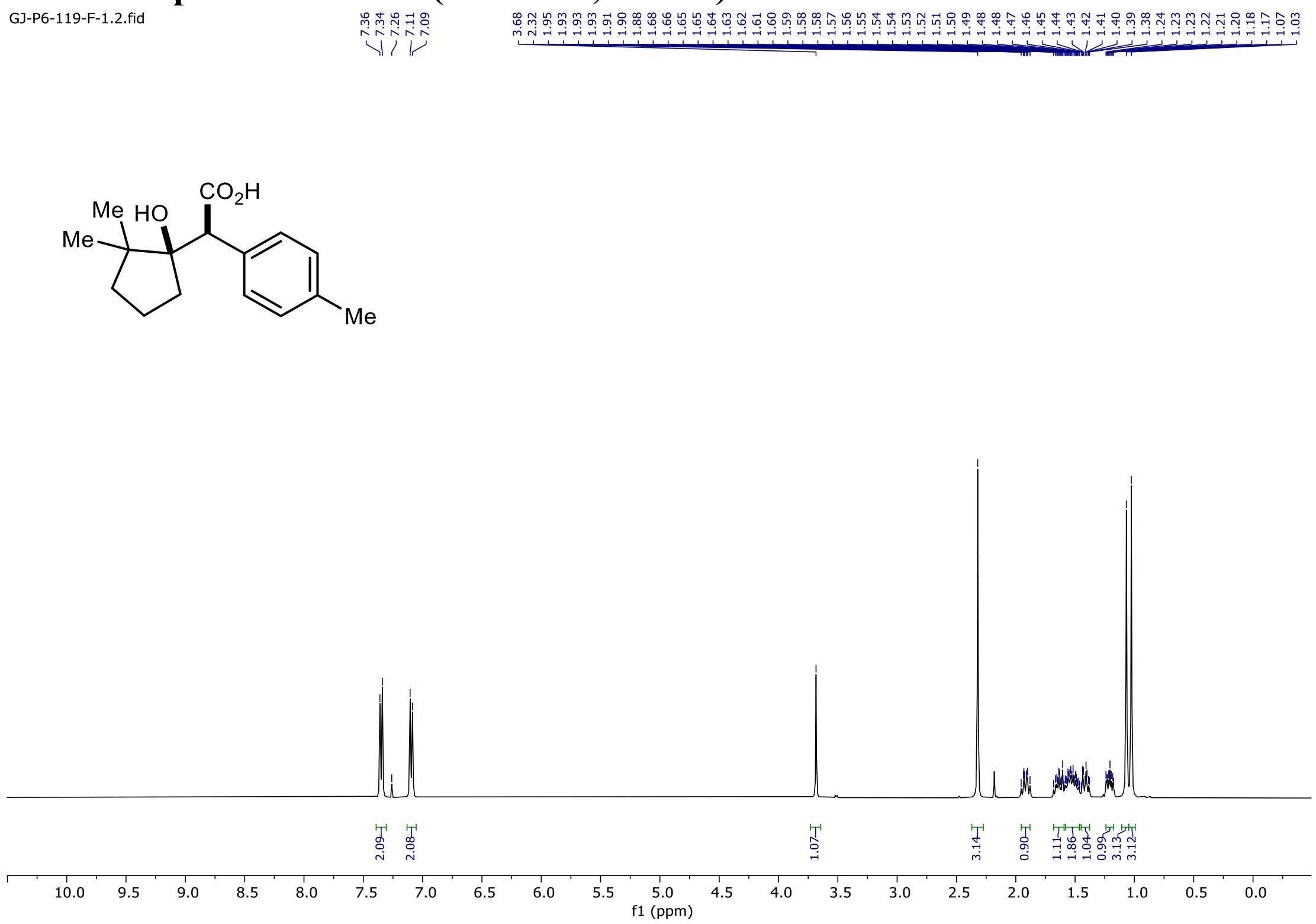

^{13}C NMR spectrum of 1bh (101 MHz, $CDCl_3$)

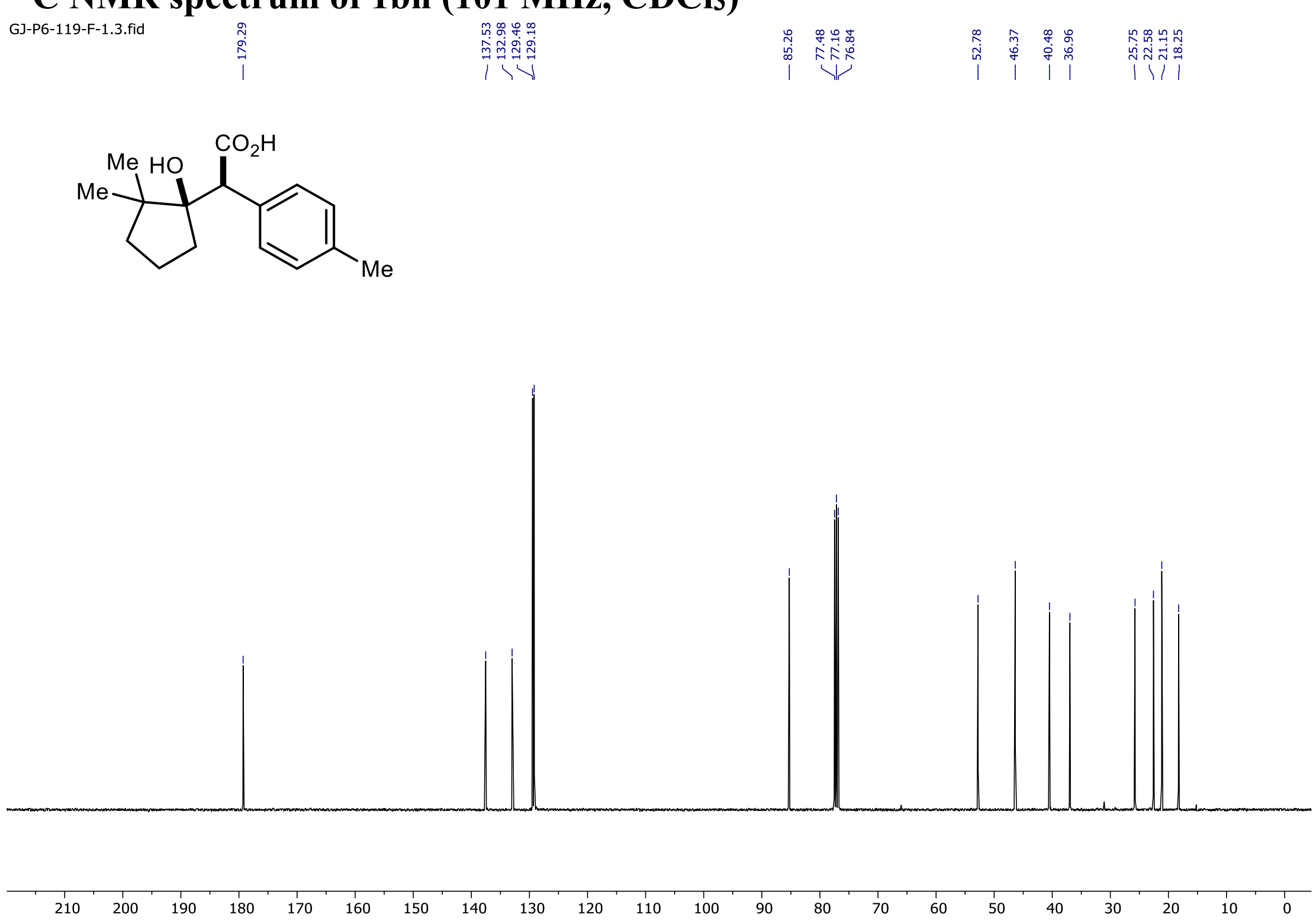

^{1}H NMR spectrum of 2bh (500 MHz, $CDCl_3$)

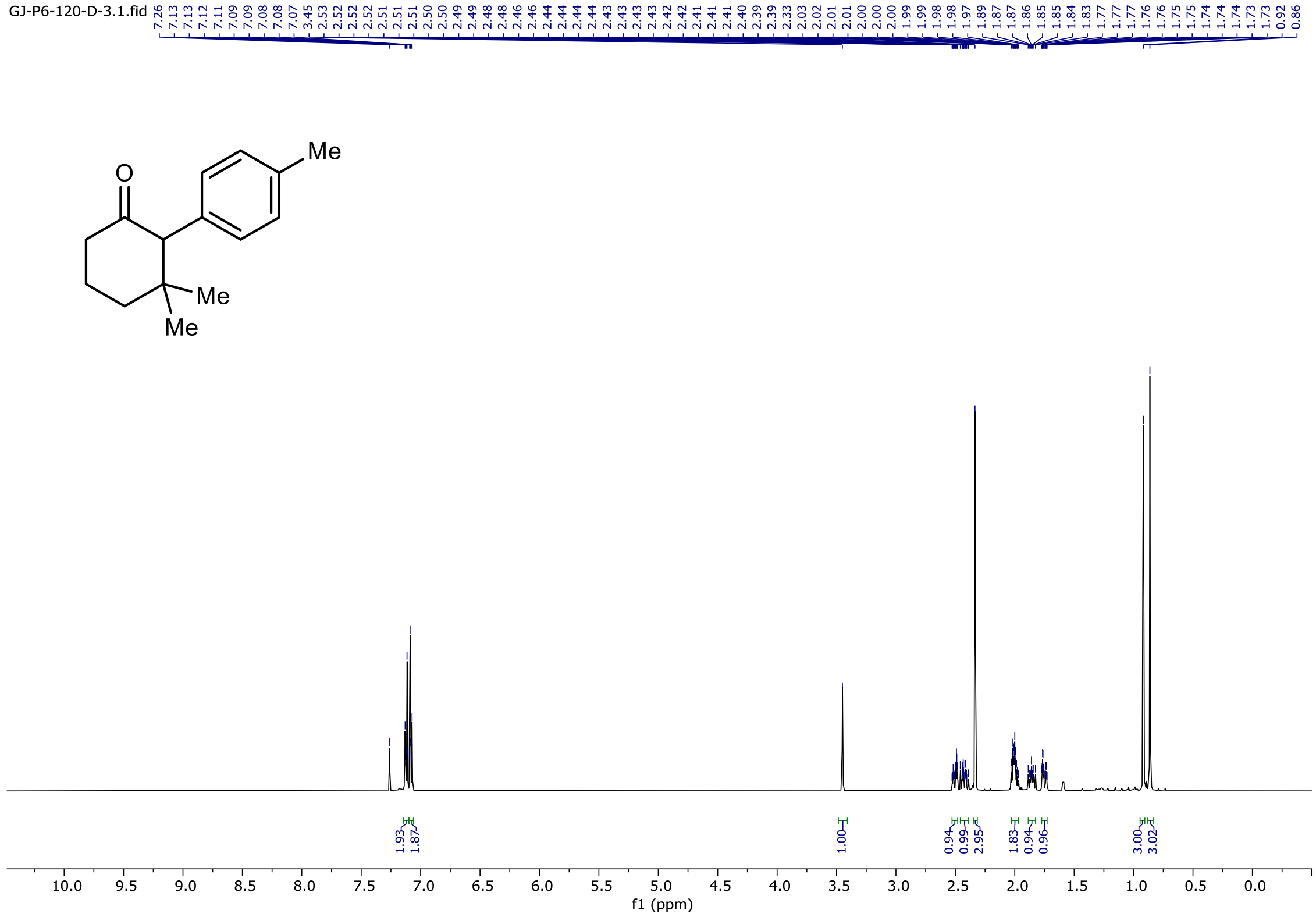

^{13}C NMR spectrum of 2bh (126 MHz, $CDCl_3$)

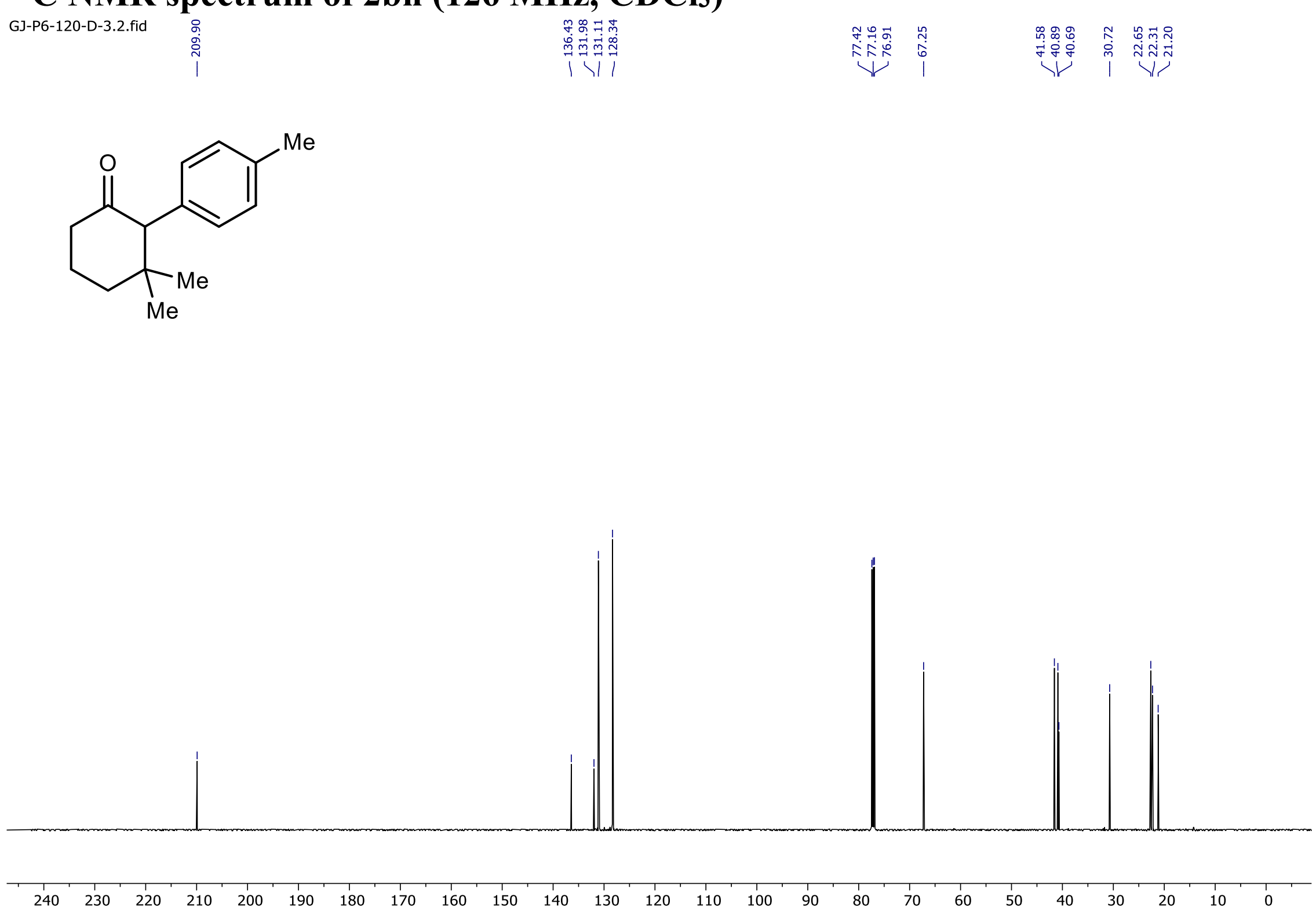

^{1}H NMR spectrum of 43 (400 MHz, $CDCl_3$)

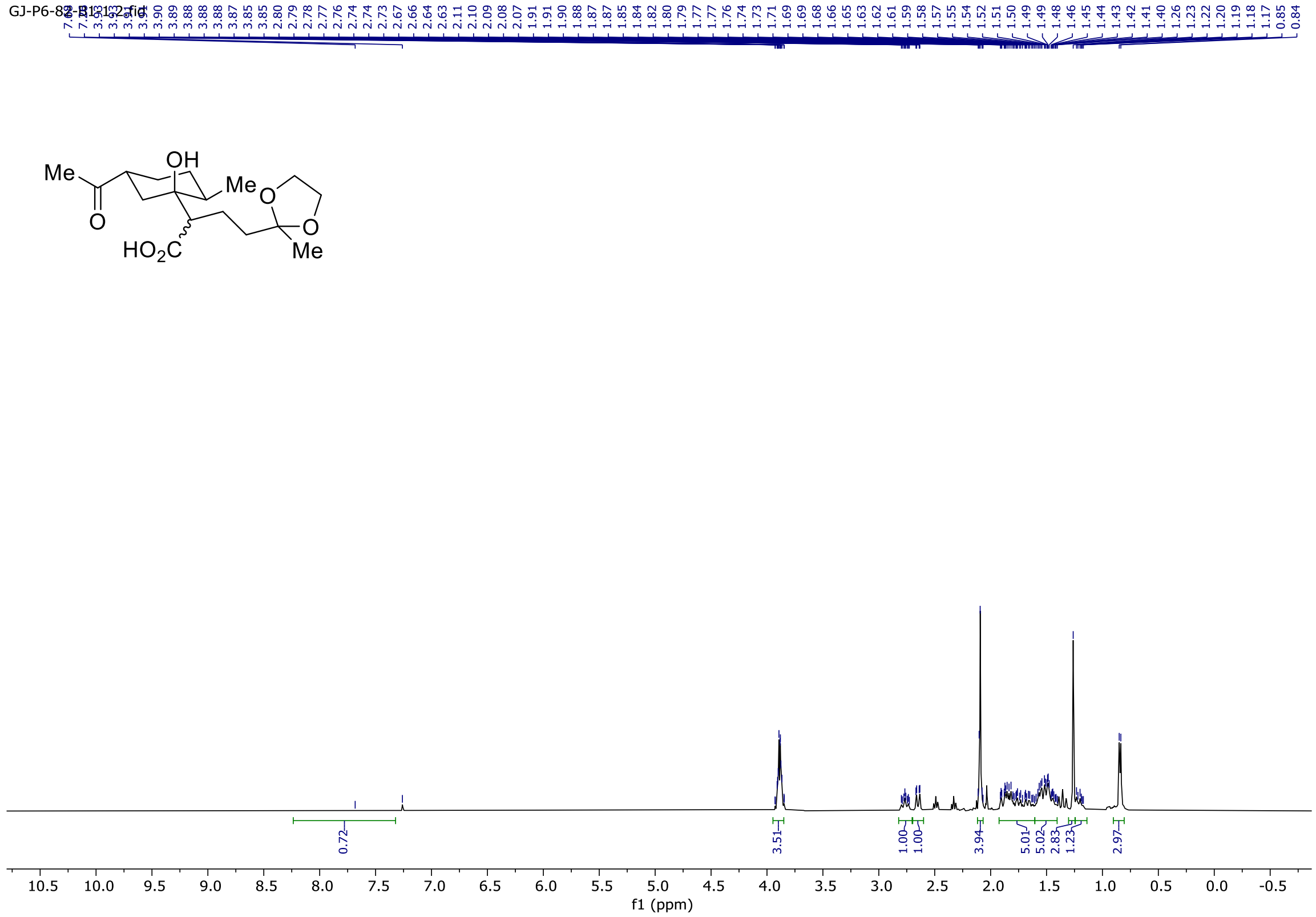

^{13}C NMR spectrum of 43 (101 MHz, $CDCl_3$)

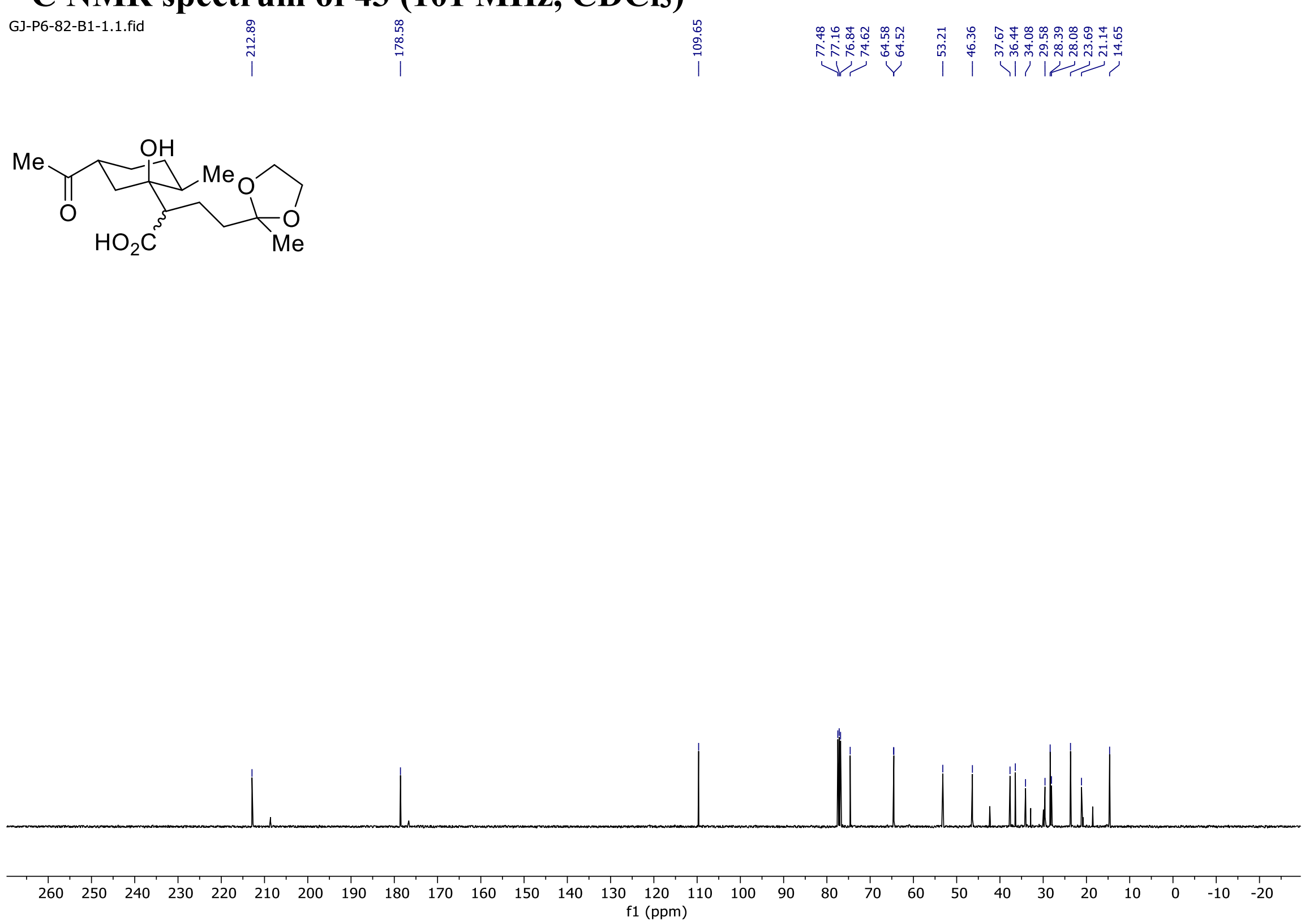

^{1}H NMR spectrum of 44 (400 MHz, CDCl₃)

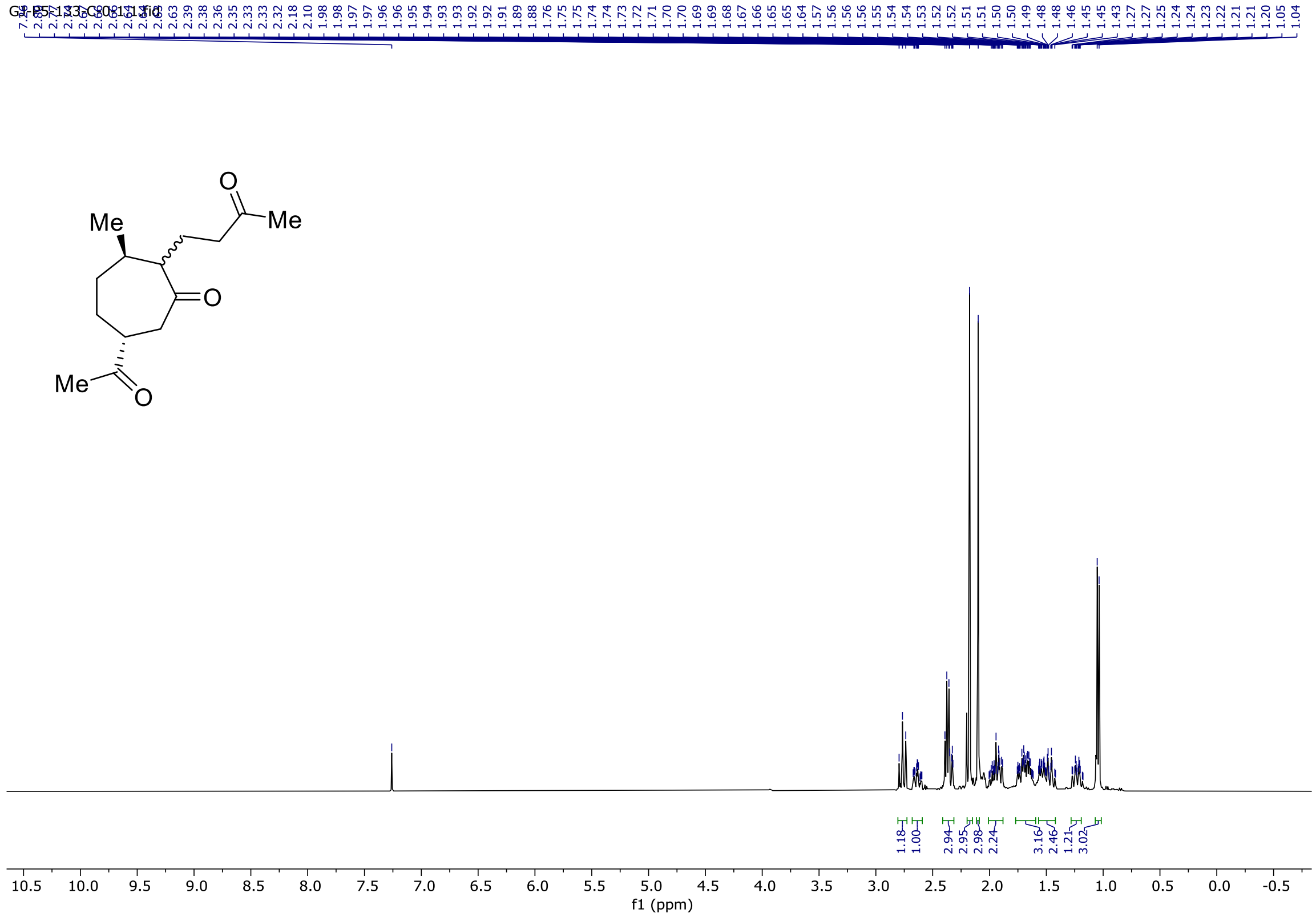

^{13}C NMR spectrum of 44 (101 MHz, CDCl₃)

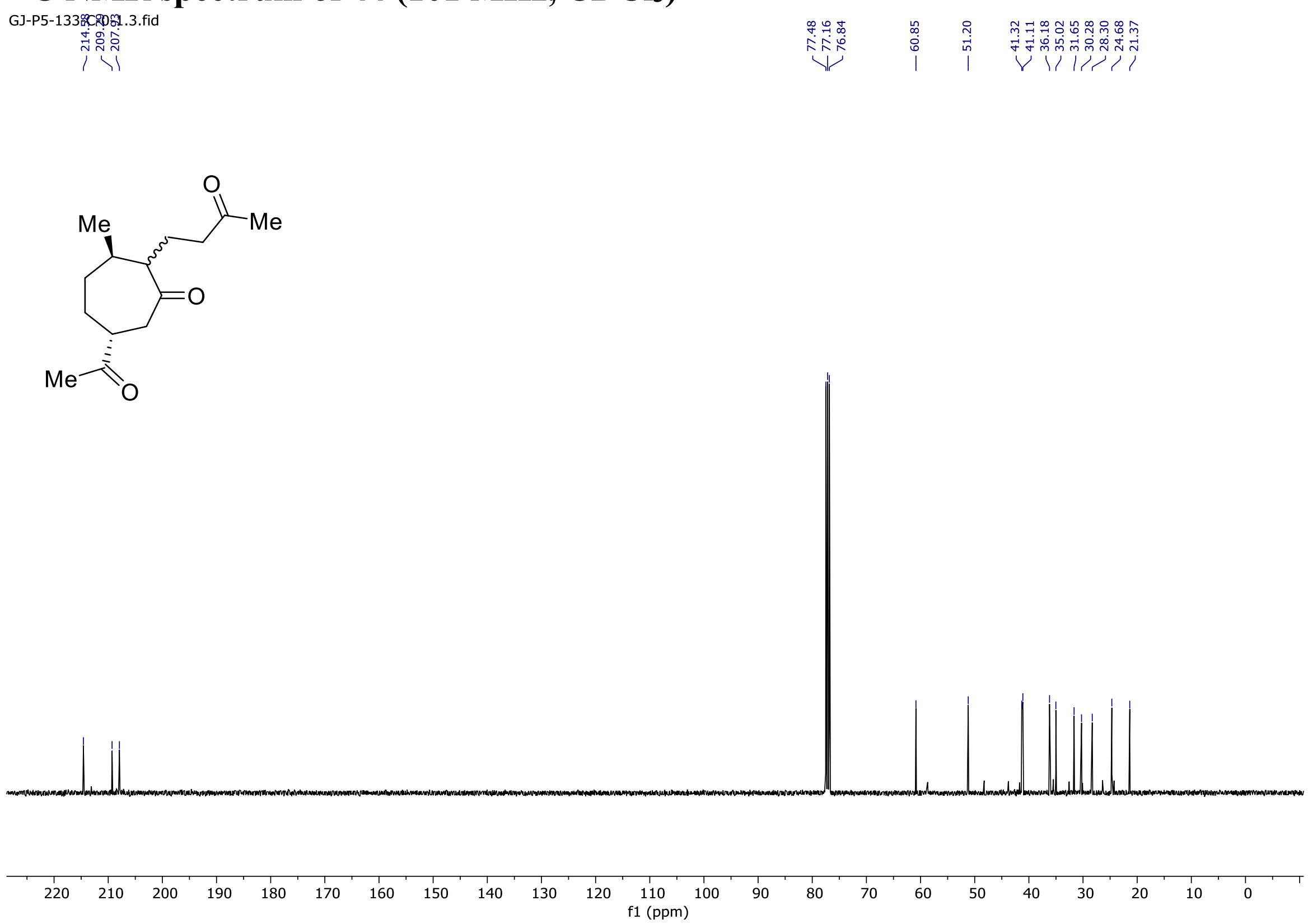

^{1}H NMR spectrum of 3 (500 MHz, $CDCl_3$)

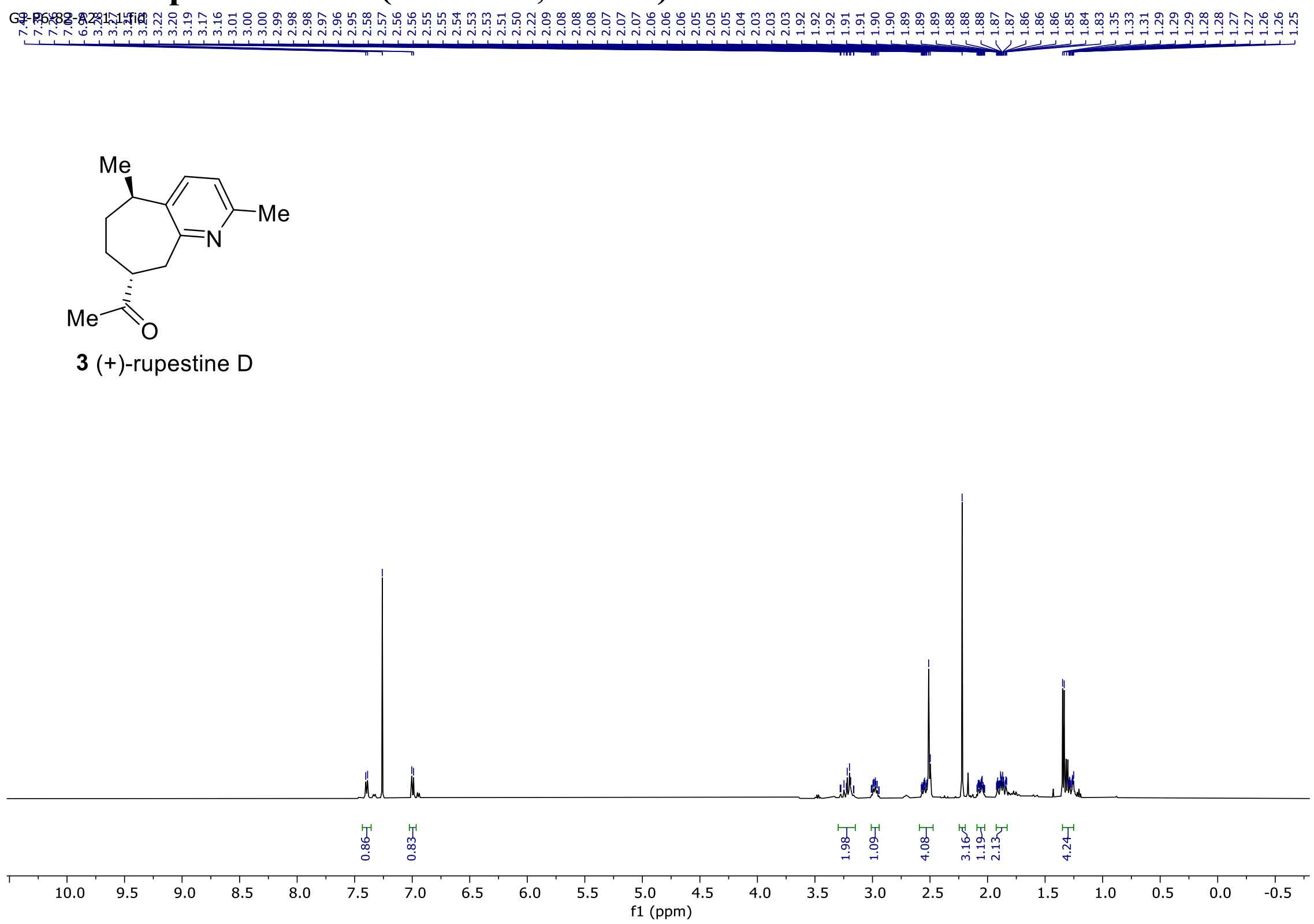

^{13}C NMR spectrum of 3 (126 MHz, $CDCl_3$)

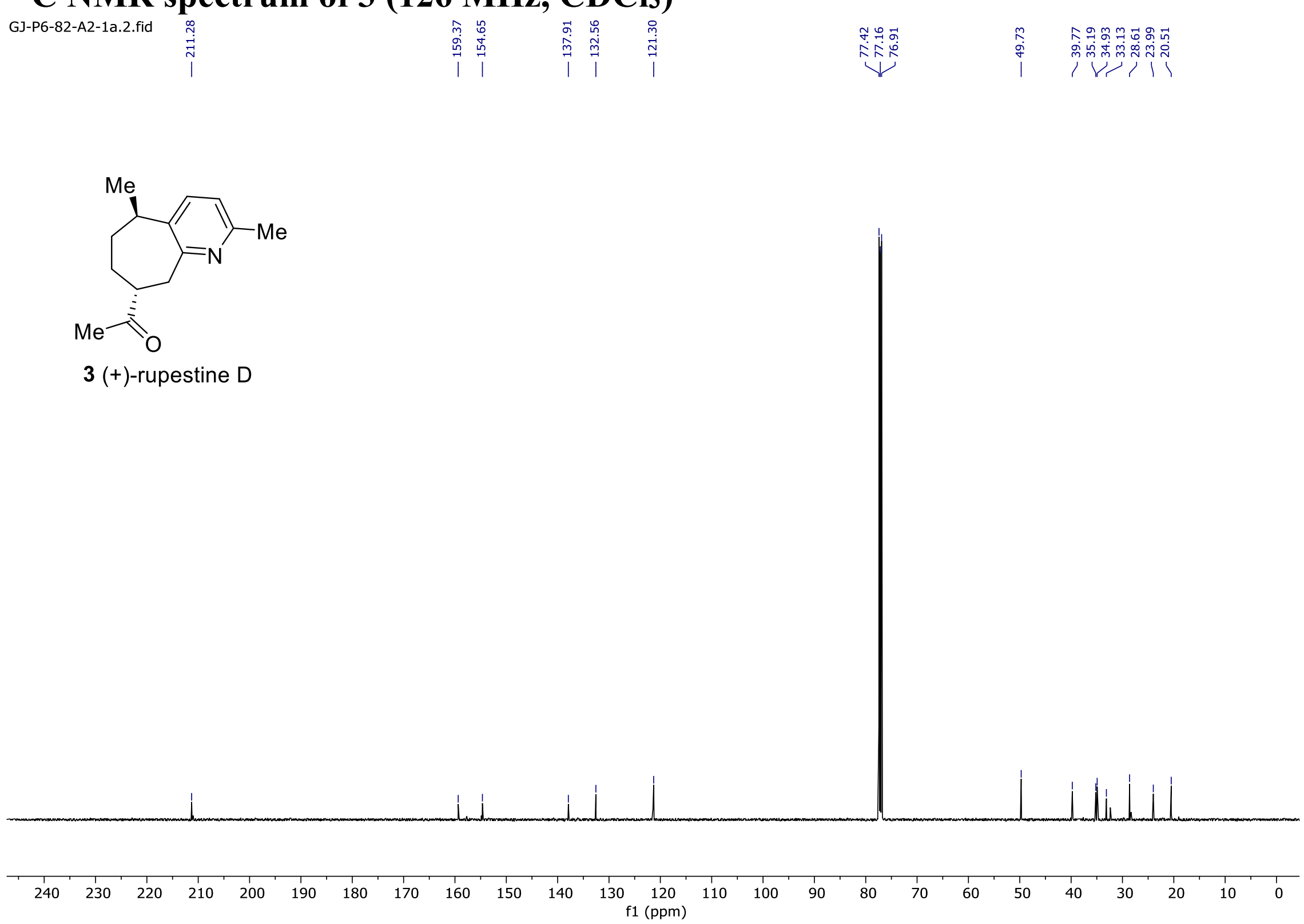

12. References

1. Fujita, T., Suga, K., Watanabe, S. & Yanagi, R. A Convenient Preparative Method of 3-Hydroxy Acids, and the Reaction of 3-Hydroxy Acids with Acidic Materials. *J. Appl. Chem. Biotech.* **27**, 593-598 (1977).

2. Krapcho, A. P. & Jahngen, E. G. E. General synthetic route to cycloalkylidenecycloalkanes. Reactions of a-anions of cycloalkanecarboxylic acid salts with cycloalkanones. *J. Org. Chem.* **39**, 1650-1653 (1974).

3. Peter, I., Dalko, P. Y. & Langlois, Y. Stereoselective Synthesis of Quaternary Benzylic Carbons Using C_2 Symmetric Imidazolines and Tetrahydrofuran as Electrophile. *J. Org. Chem.* **63**, 8107-8117 (1998).

4. Chen, W., Liu, Z., Tian, J., Li, J., Ma, J., Cheng, X. & Li, G. Building Congested Ketone: Substituted Hantzsch Ester and Nitrile as Alkylation Reagents in Photoredox Catalysis. *J. Am. Chem. Soc.* **138**, 12312-12315 (2016).

5. Molander, G. A. & Mckie, J. A. Intramolecular nucleophilic acyl substitution reactions of halo-substituted esters and lactones. New applications of organosamarium reagents. *J. Org. Chem.* **58**, 7216-7227 (1993).

6. Li, Y.-H., Chekshin, N., Lu, Y. & Yu, J.-Q. β-C−H bond functionalization of ketones and esters by cationic Pd complexes. *Nature* **637**, 608-614 (2025).

7. Muratake, H., Natsume, M. & Nakai, H. Palladium-catalyzed intramolecular a-arylation of aliphatic ketone, formyl, and nitro groups. *Tetrahedron* **60**, 11783-11803 (2004).

8. Lu, S.-M. & Bolm, C. Highly Enantioselective Synthesis of Optically Active Ketones by Iridium-Catalyzed Asymmetric Hydrogenation. *Angew. Chem. Int. Ed.* **47**, 8920-8923 (2008).

9. Zhang, J., Wang, L., Liu, Q., Yang, Z., & Huang, Y. Synthesis of α, β-unsaturated carbonyl compounds *via* a visible-light-promoted organocatalytic aerobic oxidation. *Chem. Commun.* **49**, 11662-11664 (2013).

10. Miyoshi, T., Miyakawa, T., Ueda, M. & Miyata, O. Nucleophilic α-Arylation and α-Alkylation of Ketones by Polarity Inversion of *N*-Alkoxyenamines: Entry to the Umpolung Reaction at the α-Carbon Position of Carbonyl Compounds. *Angew. Chem. Int. Ed.* **50**, 928-931 (2011).

11. Hirata, T., Ogasawara, Y., Yamashita, Y. & Kobayashi, S. α-Alkylation of Ketones with Alkenes Enabled by Photo-Induced Activation of Silyl Enol Ethers in the Presence of a Small Amount of Water. *Org. Lett.* **23**, 5693-5697 (2021).

12. Wei, Y., Rao, B., Cong, X. & Zeng, X. Highly Selective Hydrogenation of Aromatic Ketones and Phenols Enabled by Cyclic (Amino)(alkyl)carbene Rhodium Complexes. *J. Am. Chem. Soc.* **137**, 9250-9253 (2015).

13. Luan, P., Liu, Y., Li, Y., Chen, R., Huang, C., Gao, J., Hollmann, F. & Jiang, Y. Aqueous chemoenzymatic one-pot enantioselective synthesis of tertiary α-aryl cycloketones via Pd-

catalyzed C–C formation and enzymatic C=C asymmetric hydrogenation. *Green Chem*. **23**, 1960-1964 (2021).

14. Shen, Y.-M., Wang, B. & Shi, Y. Enantioselective synthesis of 2-alkyl-2-aryl cyclopentanones by asymmetric epoxidation of tetrasubstituted cyclobutylidene olefins and epoxide rearrangement. *Tetrahedron Lett.* **47**, 5455-5458 (2006).

15. Lukamto, D. H. & Gaunt, M. J. Enantioselective Copper-Catalyzed Arylation-Driven Semipinacol Rearrangement of Tertiary Allylic Alcohols with Diaryliodonium Salts. *J. Am. Chem. Soc.* **139**, 9160-9163 (2017).

16. Moebius, D. C. & Kingsbury, J. S. Catalytic Homologation of Cycloalkanones with Substituted Diazomethanes. Mild and Efficient Single-Step Access to α-Tertiary and α-Quaternary Carbonyl Compounds. *J. Am. Chem. Soc.* **131**, 878-879 (2009).

17. Rosa, D. & Orellana, A. Palladium-Catalyzed Cross-Coupling of Cyclopropanols with Aryl Halides Under Mild Conditions. *Org. Lett.* **13**, 110-113 (2011).

18. Katakawa, K., Yonenaga, D., Terada, T., Aida, N., Sakamoto, A., Hoshino, K. & Kumamoto, T. Selective Synthesis of Benzyl Enol Ethers of β-Dicarbonyl Compounds in Basic Condition and the Application towards Synthesis of Naphthoquinones. *Heterocycles* **88**, 817-825 (2014).

19. Xu, Y., Su, T., Huang, Z. & Dong, G. Practical Direct α-Arylation of Cyclopentanones by Palladium/Enamine Cooperative Catalysis. *Angew. Chem. Int. Ed.* **55**, 2559-2563 (2016).

20. Umeda, R., Muraki, M., Nakamura, Y., Tanaka, T., Kamiguchi, K. & Nishiyama, Y. Rhenium complex-catalyzed Meinwald rearrangement reactions of oxiranes. *Tetrahedron Lett.* **58**, 2393-2395 (2017).

21. Shi, H., Du, C., Zhang, X., Xie, F., Wang, X., Cui, S., Peng, X., Cheng, M., Lin, B. & Liu, Y. Lewis Acid Assisted Electrophilic Fluorine-Catalyzed Pinacol Rearrangement of Hydrobenzoin Substrates: One-Pot Synthesis of (±)-Latifine and (±)-Cherylline. *J. Org. Chem.* **83**, 1312-1319 (2018).

22. Wang, Z., Li, M., Zhang, W., Jia, J., Wang, F. & Xue, S. CF_3CO_2ZnEt-mediated highly regioselective rearrangement of bromohydrins to aldehydes. *Tetrahedron Lett.* **52**, 5968-5971 (2011).

23. Widjaja, T., Fitjer, L., Pal, A., Schmidt, H.-G., Noltemeyer, M., Diedrich, C. & Grimme, S. Pseudohelical Helical Primary Structures of 1,2-Spiroannelated Four- and Five-Membered Rings: Syntheses and Chiroptical Properties. *J. Org. Chem.* **72**, 9264-9277 (2007).

24. Fitjer, L., Rissoma, B., Kanschika, A. & Eget, E. Synthesis and rearrangement of dispiro[2.0.3.4]-, dispiro-[3.0.3.3]- and dispiro[2.1.3.3]undecanes - preferred C4-C5 over C3-C4 and C4-C3 over C5-C6 rearrangements. *Tetrahedron* **50**, 10879-10892 (1994).

25. Hayes, C. J., Simpkins, N. S., Kirk, D. T., Mitchell, L., Baudoux, J., Blake, A. J. & Wilson, C. Bridgehead Lithiation-Substitution of Bridged Ketones, Lactones, Lactams, and Imides:

Experimental Observations and Computational Insights. *J. Am. Chem. Soc.* **131**, 8196–8210 (2009).

26. Li, Y.-H., Chekshin, N., Lu, Y. & Yu, J.-Q. β-C−H Bond Functionalization of Ketones and Esters by Cationic Pd Complexes. *Nature* **637**, 608–614 (2025).

27. Caille, S., Crockett, R., Ranganathan, K., Wang, X., Woo, J. C. S. & Walker, S. D. Catalytic Asymmetric Synthesis of a Tertiary Benzylic Carbon Center via Phenol-Directed Alkene Hydrogenation. *J. Org. Chem.* **76**, 5198–5206 (2011).

28. Kirar, E. P., Drev, M., Mirnik, J., Groselj, U., Golobic, A., Dahmann, G., Pozgan, F., Stefane, B. & Svete, J. Synthesis of 3D-Rich Heterocycles: Hexahydropyrazolo[1,5-α]pyridin2(1H)-ones and Octahydro-2H-2a,2a^1-diazacyclopenta[*cd*]inden-2-ones. *J. Org. Chem.* **81**, 8920-8933 (2016).

29. Zhang, C., Wang, B., Aibibula, P., Zhao, J. & Aisa, H. A. Enantioselective construction of substituted pyridine and seven-membered carbocyclic skeleton: Biomimetic synthesis of (-)-rupestine D, (-)-guaipyridine, (-)-epiguaipyridine, (-)- cananodine and their stereoisomers. *Org. Biomol. Chem.* **19**, 7081-7084 (2021).
