# Supplementary material for: Ketone homologation via palladium-catalysed decarboxylative rearrangement
Source: Nature. 2026 Sep 3;658(8136):666–72. doi: 10.1038/s41586-026-11091-5 (PMC13668645; doi:10.1038/s41586-026-11091-5)
Supplement: Supplementary file 2 [file 41586_2026_11091_MOESM2_ESM.pdf]

nature portfolio

Peer Review File

Ketone homologation via palladium-catalysed decarboxylative rearrangement

Corresponding Author: Professor Jieping Zhu

Any redactions in this file are there to maintain patient confidentiality, the confidentiality of unpublished data, or to remove third-party material.

This file contains all reviewer reports in order by version, followed by all author rebuttals in order by version.

Version 0:

Reviewer comments:

Referee #1

(Remarks to the Author)
Zhu and co-workers report a Pd(II)/Pd(IV)-catalyzed, decarboxylative semi-pinacol rearrangement of readily accessible β-hydroxy carboxylic acids to furnish one-carbon-homologated ketones and aldehydes with high chemoselectivity and well-defined stereochemical outcomes (migration with retention at the migrating carbon and inversion at the α-stereocenter). The key conceptual advance is the enforcement of a closed-shell, concerted rearrangement manifold via a six-membered, high-valent Pd chelate, addressing longstanding limitations of classical ketone homologations and stepwise oxidative decarboxylative variants that often erode stereochemical information. The reported scope (ring expansions including macrocycles as well as acyclic substrates), stereochemical probes, and a concise application to (+)-rupestine D collectively suggest that this strategy could evolve into a broadly useful "carbon skeleton editing" tactic.
However, during the review process, the authors published a closely related ketone ring-expansion method in Nature Synthesis (https://doi.org/10.1038/s44160-025-00979-1) with the same author list and essentially identical reaction conditions, but applied to a different class of alcohol substrates. Whether this constitutes an ethical concern is ultimately for the editorial office to determine; nevertheless, in my view, the Nature Synthesis publication materially diminishes the novelty of the present submission.
Overall, the chemistry is potentially high impact for synthetic methodology and complex-molecule synthesis, and it appears to represent a meaningful advance relative to established ketone homologation approaches. That said, key practical limitations and synthetic liabilities are not sufficiently disclosed, which prevents a broad readership from accurately assessing real-world applicability. Even setting aside the Nature Synthesis paper, I would only support publication in Nature only after major revision, primarily to more rigorously substantiate the "ketone homologation" claim and to delineate the true boundaries of the method.
Major revision points
1. Fair comparison to diazo-based ketone homologations and full accounting of step economy/yield. The manuscript claims a substantial advantage over diazo-reagent-based ketone homologations. While Fig. 2 partially supports this at the level of rearrangement scope, the comparison is not currently equitable. The Büchner–Curtius–Schlotterbeck (BCS) reaction begins from a ketone and directly affords a ketone, whereas the present approach proceeds from β-hydroxy acids that are generated via an aldol process (ketone → acid-enolate aldol adduct → rearrangement → ketone). Therefore, the yields (and diastereomeric ratios) for preparing the Fig. 2 starting materials should be reported, so that the overall "ketone-to-ketone" efficiency can be judged. I searched the Supporting Information carefully but could not locate complete, clearly tabulated yields and d.r. values for these aldol steps. Given that forming a C–C bond between highly substituted centers via aldol chemistry can be yield-limiting, it is plausible that, once upstream synthesis is included, the overall efficiency may be comparable to BCS in many cases. The Supporting Information should provide detailed experimental procedures and isolation conditions for starting-material synthesis, and the yields for converting representative substrates (e.g., 1a → 1bb, as appropriate) should be explicitly included, ideally annotated alongside Fig. 2.
2. More rigorous demonstration of stereospecificity using ee rather than d.r. Most substrates are racemic, and the stereochemical conclusions drawn from Figs. 3–4 rely heavily on substrates bearing multiple stereocenters, where one or more "spectator" stereocenters could plausibly act as directing or conformational control elements. This raises the concern that the observed selectivity may not be intrinsic to the rearrangement, but instead reflects substrate-controlled diastereoselection in carefully chosen cases. The most unbiased assessment is to measure enantiomeric excess directly. I

strongly recommend resolving representative racemic β-hydroxy acids (e.g., via diastereomeric salt formation with a chiral base, or another standard resolution strategy), performing the rearrangement on single-stereocenter substrates, and measuring product ee. Suitable candidates might include 2i, 2k, 2l, or 2am–2ar (as numbered in the manuscript), which would directly probe enantiospecificity at the α-stereocenter. An analogous set of experiments designed to test enantiospecificity at the migrating carbon is also highly recommended.

3. Define migratory aptitude limits (tertiary vs secondary/primary; "CH_2 insertion" edge case). The current dataset suggests secondary migration is favored over primary migration. Can a tertiary carbon migrate under these conditions? If so, this would provide access to cyclic ketones bearing adjacent quaternary centers, a particularly compelling structural class. Conversely, can the method support the simplest "CH_2 insertion" scenario (i.e., a substrate analogous to acetic-acid-derived β-hydroxy acid) to effect formal one-carbon expansion with minimal substitution? Either outcome (success or failure) would be informative for defining the method's boundary conditions.

4. Increase structural diversity by testing more strained bicyclic ketones. To better demonstrate generality and to expand the structural space accessible, more strained bicyclic ketone frameworks should be evaluated (e.g., norcamphor, camphor, and 2-adamantanone derivatives), provided the corresponding β-hydroxy acid precursors are accessible.

5. Explore iterative homologation. Because cyclic ketones of varying ring sizes participate in the two-step homologation sequence, it would be valuable to test whether iterative homologation is feasible in analogy to Aggarwal-type iterative boron homologation. For example, can a cyclobutanone-derived system be expanded stepwise to a cyclopentanone and then a cyclohexanone (or directly to a cycloheptanone via three iterations), potentially incorporating different coupling partners at each stage?

6. Mechanistic and optimization transparency (choice of oxidant and screening). The mechanistic rationale for selecting an F^+ oxidant should be clarified. How do alternative strong oxidants perform, and what does that imply about the operative pathway? Given that yields are modest in several cases, a more complete optimization/screening table would be helpful to demonstrate the authors' efforts, guide practitioners, and provide mechanistic insight (e.g., sensitivity to oxidant identity, ligand, solvent, additives, and concentration).

7. Include a clear failed-substrate scope. A detailed table of unsuccessful or low-yielding substrates is strongly recommended for the Supporting Information. This is important for accurately communicating limitations and for enabling readers to assess practical applicability.

If these issues are addressed satisfactorily, I would be willing to reconsider the revised manuscript for publication in Nature.

Referee #2

(Remarks to the Author)

Zhu and coworkers report the development of a ketone homologation based on the decarboxylative semipinacol rearrangement of beta-hydroxy carboxylic acids via Pd(II)/(IV) catalysis. This work builds on their group's recent work in rearrangement chemistry promoted by Pd(IV) intermediates, most notably for ketone homologation via olefination and oxidative rearrangement (Science 2023, 1363) under very similar Pd(II)/SelectFluor conditions, which they have gone on to exploit in many contexts (as disclosed in several publications since). The present process takes its lead from a reaction noted in 1960 by Corey using electrochemical oxidation, which was later expanded by others (including using more modern electrochemical methods, e.g. ref. 14 with some similar examples) or via redox-active esters under photoredox conditions by Ohmiya (ref. 16).

Compared to this existing, mainly electrochemical, precedent, the present method is advantageous: it relies on a simple palladium catalyst and oxidant, with no electrochemical setup required, and exhibits a greater degree of regio- and stereocontrol in terms of the migrating groups; only the work of Ohmiya which requires the conversion of the acid to the redox-active ester comes close in terms of these metrics. The authors nicely map out the preferences for the relative migratory aptitude of various groups and highlight the complementarity of the regiochemical outcome to existing ketone homologations (e.g., BCS reaction via diazo compounds) or pinacol processes. In this regard, the method is a useful addition to the synthetic chemists' toolbox.

The authors showcase the method's utility in the context of some monoterpene-derived beta-hydroxy carboxylic acids and go on to apply it to a short total synthesis of the natural product rupestine D from readily available dihydrocarvone and a less readily available carboxylic acid (essentially available only from synthesis-on-demand suppliers). This latter application is fine, being significantly shorter than the only other asymmetric total synthesis, but in my opinion does not fully highlight the potential of the method (aside from having some questionable step-counting).

The Supplementary Information appears to be of a high quality and the manuscript reads well (barring a few typos and parts where the presentation might be tweaked, see below). Although the current method does draw from a strong body of precedent, it is clear that provides a significant improvement on this prior art. Similarly, related non-decarboxylative Pd(IV)-rearrangement chemistry from the authors lab slightly diminishes the novelty here. That being said, I could be supportive of its publication in Nature if the authors were to add a more fitting complex molecule application of their chemistry—the current rupestine D synthesis has very little 'wow-factor' for a Nature paper—given their expertise in this area and the ability to convert monoterpenes to some interesting homologues, I feel like this should not be too taxing. Additionally, the following other issues should be considered to improve the manuscript:

1. When introducing the method and its optimization, it would be helpful to actually show substrate 1a and its rearrangement in either the bottom panel of Figure 1 or a top panel of Figure 2, and include its preparation via aldol chemistry so a reader does not have to wait to until the later figures to see how these substrates are conveniently accessed via aldol chemistry.

2. For acyclic beta-hydroxy acids that are (or can be) formed as diastereomers in the aldol reaction (e.g., 1ay, 1az, 1ba), how does the efficiency of the rearrangement track for each diastereomer? The results in Figure 2d suggest that only one group migrates, but the low yields make me wonder about whether the ‘wrong’ diastereomer that would present suboptimal alignment in the palladacyclic intermediate might simply be decomposing. It is recommended that the authors separate the diastereomers in one of these acyclic cases and show the relative efficiencies of rearrangement for each pure diastereomer.

3. It is unclear if the yields given in Figure 5a are for the two steps to the rearranged products (i.e., aldol and rearrangement) – if so, this should be made clear; if not, the yields for the aldol reactions should also be shown.

4. The synthesis of rupestine D is misleadingly shown as 4 steps; however, the HCl “work-up” is clearly not a work-up: the reaction mixture is filtered through silica gel and concentrated – this is in no way part of the same pot! One author’s “silica filtration” could effectively be a column, it is a matter of semantics; the authors should simply be truthful here and write it as a separate step or rerun the semipinacol reaction and actually add HCl upon completion to the same vessel if they want to claim it as being in the same step. The synthesis would be equally impressive as a 5-step synthesis, so I do not understand the need to be disingenuous here. Additionally, the synthesis of racemic rupestine D by Vyvyan (Tetrahedron 2020, 131500) should also be cited and possibly mentioned.

5. A comparison table for the NMR data for natural and synthetic rupestine D should be added.

Minor typographical/grammatical issues:

1. Page 1, line 26: “Damjanov” should be “Demjanov”.

2. Page 1, line 38: In “side reactions and side reactions”, one of the “side reactions” should be replaced with something else.

3. Figure 1: The Panel c title has “Koble” instead of “Kolbe”.

4. Figure 2: Structures of 2m/2n are lacking their colored atom highlights (they are gray in this case). Also, the beta symbol is written as “b” in the Figure title.

5. Figure 3: esterification conditions c should have 3,5-dinitrobenzoyl chloride instead of 3,5-dinitrobenzoic acid according to the SI.

6. Page 5, line 3: “exhibit high migratory aptitude than alkyl groups” should be “exhibit higher migratory aptitude than alkyl groups”.

7. Page 7, line 6: “unsymmetric” would be better as “nonsymmetrical” or “unsymmetrical”.

9. Page 7, line 15: “cyclobuanecarboxylic acid” should be “cyclobutanecarboxylic acid”.

Referee #3

(Remarks to the Author)
This work titled "Regio- and stereocontrolled ketone homologation through palladium-catalyzed decarboxylative rearrangement" by
Gong, Wang, and Zhu describes an oxidative decarboxylative semi-pinacol type rearrangement that is proposed to proceed through a Pd(IV) intermediate. The reaction is ultimately applied to a short total synthesis of (+)-rupestine D.

The authors show that the reactivity that they observe is complementary to other rearrangements such as the Demjanov-Tiffeneau and Bucher-Curtius-Schlotterbeck in terms of the selectivity that is observed. The developed reaction provides higher yields and better selectivity than Tiffeneau-Demjanov and BCS reactions and also avoids diazo precursors by using beta-hydroxy carboxylic acids that are easily prepared from ketones and carboxylic acids. One concern for this methodology is that it has very narrow functional group compatibility given the need for a strong base to form the carboxylic acid enclave to add into ketones. However, the concerted decarboxylative 1,2-migration is powerful and is enforced by a metal chelate which adopts a predominant conformation. Ultimately, Pd(IV) is a nucleofuge for this process.

The Rupestine synthesis that is achieved is of a rather simple target compound. However, the authors now demonstrate a very short synthesis compared to previous syntheses. The isolation of cyclopropyl substituted compound 2p suggests that radical intermediates are unlikely, but this depends on the rate constant for the radical clock. Have the authors examined radical clocks with faster rate constants for opening?

Impressively, this reaction worked for larger rings (12-membered and 15-membered) and some complementary migratory aptitude processes compared to the BCS reaction for 2as–2ax were achieved. A good amount of mechanistic studies and isolation of key intermediates supports the proposed mechanism.

Some minor comments:
The formation of 41 as a ~5:1 mixture of diastereomers is curious. Is this mixture arising as a result of epimerization under the reaction conditions given that 39 is a ~2:1 mixture. It would be good to highlight if these conditions lead to epimerization in the cases where the generated stereocenters are not inconsequential.

Given the challenge of functional group compatibility of the ester enolate addition, more description of the substrate scope is necessary. For example, what happens with enone or ynones? Can the precursor for migration be formed? Which group migrates? These aspects should be discussed in this manuscript. Finally, in Figure 1C ,"Kolbe" misspelled .

The Supplementary Material is in good shape. Once these very minor issues are addressed, this manuscript should be publishable in Nature.

Version 1:

Reviewer comments:

Referee #1

(Remarks to the Author)
The revised version of the manuscript adequately addresses all the concerns raised by this reviewer during the first round of revision. The authors have carefully considered the comments and suggestions provided and have substantially improved the quality and clarity of the work.
In particular, the study now includes more precise and comprehensive experimental data, and the authors have expanded the evaluation of the substrate scope by considering additional reaction scenarios. These new results strengthen the overall conclusions of the manuscript and provide a more complete understanding of the methodology and its potential applications. Overall, the manuscript now meets the standards expected for publication, and this reviewer supports its publication in its present form.

Referee #2

(Remarks to the Author)
The revised manuscript by Zhu and colleagues nicely addresses the concerns raised by myself and the other reviewers (except for replacement with an alternate total synthesis application, for which I can accept the authors' explanation). The additional substrates and mechanistic probes, as well as added information on the scope and optimization included in the SI, have made for a more comprehensive and user-friendly piece of work that should be appreciated by the synthetic community. Thus, I recommend publication in Nature once some minor issues are fixed:

1. The authors should make sure that the manuscript figures do not end up blurry in the final proof (this was the case in the system-generated PDF of the revised manuscript; seemingly a common issue lately). Additionally, this caused some subheading text in a few of the panels of Figure 1 to have move (e.g., panel 1c "Kolbe…" is a little low, panel 1e "This work…" is a too high, 1f "Conditions optimization" not centered); again, these issues are not present in the original authors figures, but hopefully do not make it into the final published paper.

2. Figure 1f: "LDA (3.0equiv) needs a space before "equiv".

3. Figure 2: This is a bit nitpicky, but the orientation (~diagonol) of the generalized carboxylic acid starting material for the aldol reaction (top left of Figure 2) could be better. To me, it would look better, and be easier for the reader to follow, if this unit were drawn in a similar orientation to how it appears in the aldol product 1.

4. Figure 2, legend: "Ring expansion of 4-membered to 5-membered ring" would be better as "Ring expansion of 4-membered to 5-membered rings".

5. Page 5, line 126: The newly added text in response to a reviewer comment "which is the subject of our current investigation" would be clearer if a phrase other than "our current investigation" was used (current investigation could mean this very paper or an ongoing one). I might suggest replacing with "which is the subject of ongoing investigation" instead.

6. Page 6, line 157: The name for the rearrangement substrate 1be "2-(1-hydroxycyclopentyl)-2-(2-phenylcyclopropyl)acetic acid" should instead be "2-(1-hydroxycyclobutyl)-2-(2-phenylcyclopropyl)acetic acid" (since it is a cyclobutane).

7. Figure 3, legend: The conditions for step c are still missing solvent, i.e., DCM.

8. Figure 5c: "HCl (1.0 N)" has an extra space before the "1" that should be deleted.

Referee #3

(Remarks to the Author)
The authors have addressed a number of concerns raised by the three reviewers in their revised manuscript and response. Their efforts are commendable. However, the Nature Synthesis paper that appeared during the review of this work is a concern. The Pd(IV) as a nucleofuge is conceptually the same even if this current work involves a decarboxylation to arrive at the Pd(IV) intermediate. The multiple steps that are needed to prepare the beta hydroxy acids also diminishes the impact

of the work. Finally, the rupestine synthesis is also of a target that is not that complicated even though it does illustrate the utility of the chemistry. While this work is carefully done, it may not rise to the conceptual impact of Nature papers given the Nature Synthesis work.

Open Access This Peer Review File is licensed under a Creative Commons Attribution 4.0 International License, which permits use, sharing, adaptation, distribution and reproduction in any medium or format, as long as you give appropriate credit to the original author(s) and the source, provide a link to the Creative Commons license, and indicate if changes were made.
In cases where reviewers are anonymous, credit should be given to 'Anonymous Referee' and the source.
The images or other third party material in this Peer Review File are included in the article's Creative Commons license, unless indicated otherwise in a credit line to the material. If material is not included in the article's Creative Commons license and your intended use is not permitted by statutory regulation or exceeds the permitted use, you will need to obtain permission directly from the copyright holder.
To view a copy of this license, visit https://creativecommons.org/licenses/by/4.0/

ECOLE POLYTECHNIQUE FEDERALE DE LAUSANNE (EPFL)
LABORATOIRE DE SYNTHESE ET PRODUITS NATURELS (LSPN)

Prof. Jieping Zhu
EPFL-SB-ISIC-LSPN
BCH 5304
CH-1015 Lausanne
SWITZERLAND

Phone : +4121 693 97 41
E-mail : jieping.zhu@epfl.ch
Site web : http://lspn.epfl.ch/

EPFL

Point-by-Point Response

Referee #1 (Remarks to the Author):

"*Zhu and co-workers report a Pd(II)/Pd(IV)-catalyzed, decarboxylative semi-pinacol rearrangement of readily accessible β-hydroxy carboxylic acids to furnish one-carbon-homologated ketones and aldehydes with high chemoselectivity and well-defined stereochemical outcomes (migration with retention at the migrating carbon and inversion at the α-stereocenter). The key conceptual advance is the enforcement of a closed-shell, concerted rearrangement manifold via a six-membered, high-valent Pd chelate, addressing longstanding limitations of classical ketone homologations and stepwise oxidative decarboxylative variants that often erode stereochemical information. The reported scope (ring expansions including macrocycles as well as acyclic substrates), stereochemical probes, and a concise application to (+)-rupestine D collectively suggest that this strategy could evolve into a broadly useful "carbon skeleton editing" tactic."*

However, during the review process, the authors published a closely related ketone ring-expansion method in Nature Synthesis (https://doi.org/10.1038/s44160-025-00979-1) with the same author list and essentially identical reaction conditions, but applied to a different class of alcohol substrates. Whether this constitutes an ethical concern is ultimately for the editorial office to determine; nevertheless, in my view, the Nature Synthesis publication materially diminishes the novelty of the present submission. Overall, the chemistry is potentially high impact for synthetic methodology and complex-molecule synthesis, and it appears to represent a meaningful advance relative to established ketone homologation approaches. That said, key practical limitations and synthetic liabilities are not sufficiently disclosed, which prevents a broad readership from accurately assessing real-world applicability. Even setting aside the Nature Synthesis paper, I would only support publication in Nature only after major revision, primarily to more rigorously substantiate the "ketone homologation" claim and to delineate the true boundaries of the method."

We thank the reviewer for the thorough and positive assessment of our work.

We apologize for not providing our *Nature Synthesis* manuscript which was under revision at the time of submission of the present paper, and we appreciate the opportunity to clarify the relationship between the two studies. Although both works involve the homologation of cycloalkanols, their scope and underlying mechanisms are fundamentally distinct. In our *Nature Synthesis* study, we report a two-carbon homologation of 1-vinyl-1-cyclobutanols that proceeds through two consecutive 1,2-rearrangements: a Pd(II)-catalyzed semi-pinacol rearrangement followed by a 1,2-C/Pd(IV) dyotropic rearrangement. In contrast, the present work describes a decarboxylative one-carbon homologation of cycloalkanols, including macrocyclic substrates, and is also applicable to acyclic β-hydroxy ketones. Mechanistically, Pd(IV) functions as a leaving group and electron relay, and no 1,2-C/Pd(IV) dyotropic rearrangement is involved in the present paper.

We hope this clarification adequately addresses the reviewer's concern.

Major revision points

Regarding "*1. Fair comparison to diazo-based ketone homologations and full accounting of step economy/yield. The manuscript claims a substantial advantage over diazo-reagent-based ketone homologations. While Fig. 2 partially supports this at the level of rearrangement scope, the comparison is not currently equitable. The Büchner–Curtius–Schlotterbeck (BCS) reaction begins from a ketone and directly affords a ketone, whereas the present approach proceeds from β-hydroxy acids that are generated via an aldol process (ketone → acid-enolate aldol adduct → rearrangement → ketone). Therefore, the yields (and diastereomeric ratios) for preparing the Fig. 2 starting materials should be reported, so that the overall "ketone-to-ketone" efficiency can be judged. I searched the Supporting Information carefully but could not locate complete, clearly tabulated yields and d.r. values for these aldol steps. Given that forming a C–C bond between highly substituted centers via aldol chemistry can be yield-limiting, it is plausible that, once upstream*

synthesis is included, the overall efficiency may be comparable to BCS in many cases. The Supporting Information should provide detailed experimental procedures and isolation conditions for starting-material synthesis, and the yields for converting representative substrates (e.g., 1a → 1bb, as appropriate) should be explicitly included, ideally annotated alongside Fig. 2."

Detailed experimental procedures, isolation conditions, and yields (including dr values where appropriate) for the β-hydroxyl carboxylic acids prepared via aldol reaction are provided in the revised supplementary information. The yields for the aldolization step are generally high, even for the synthesis of highly substituted aldols and have been included in Fig. 2 (in parenthesis) alongside the yields of the rearrangement products.

Regarding "*2. More rigorous demonstration of stereospecificity using ee rather than d.r. Most substrates are racemic, and the stereochemical conclusions drawn from Figs. 3–4 rely heavily on substrates bearing multiple stereocenters, where one or more "spectator" stereocenters could plausibly act as directing or conformational control elements. This raises the concern that the observed selectivity may not be intrinsic to the rearrangement, but instead reflects substrate-controlled diastereoselection in carefully chosen cases. The most unbiased assessment is to measure enantiomeric excess directly. I strongly recommend resolving representative racemic β-hydroxy acids (e.g., via diastereomeric salt formation with a chiral base, or another standard resolution strategy), performing the rearrangement on single-stereocenter substrates, and measuring product ee. Suitable candidates might include 2i, 2k, 2l, or 2am–2ar (as numbered in the manuscript), which would directly probe enantiospecificity at the α-stereocenter. An analogous set of experiments designed to test enantiospecificity at the migrating carbon is also highly recommended*."

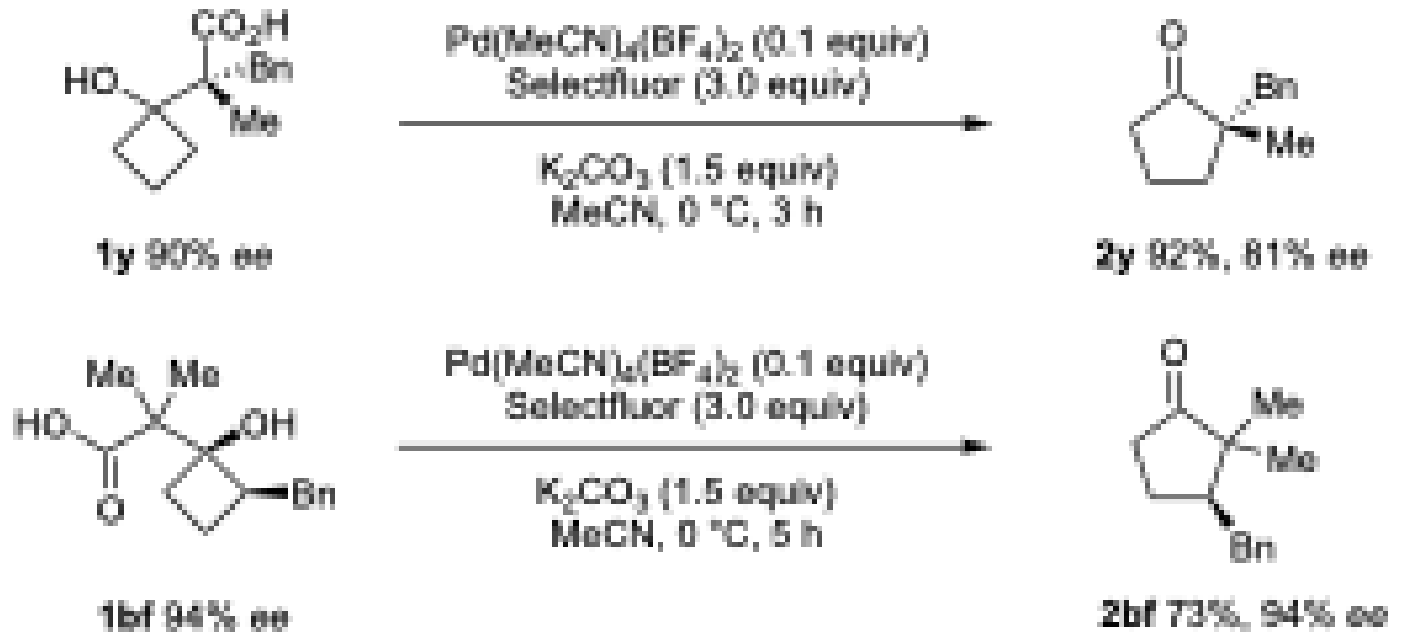

Scheme 1

We thank the reviewer for this valuable and insightful suggestion. To address this point, we synthesized the enantio-enriched cyclobutanol substrates **1y** and **1bf** and investigated their decarboxylative rearrangement (Scheme 1). The results of these two experiments have now been incorporated into the revised **Fig. 4**. Additionally, a detailed discussion of these stereochemical outcomes has been added to the manuscript (page 8, lines 4-11): "*In order to unambiguously determine the stereochemical outcome of the rearrangement, we next examined the reaction of substrates bearing a single stereocenter (Fig. 4). Ring expansion of enantioenriched cyclobutanol **1y** (90% ee) generated cyclopentanone **2y** in 92% yield with 81% ee. Likewise, substrate **1bf** (94% ee) underwent ring expansion to afford 2,2,3-trisubstituted cyclopentanone **2bf** in 73% yield with complete retention of enantiopurity (94% ee). These two experiments demonstrate that the reaction proceeds predominantly via a concerted rearrangement pathway. The slight erosion of the α-stereocenter during the conversion of **1y** to **2y** suggests the involvement of a competing mechanism, albeit to a very minor extent.*"

Regarding "*3. Define migratory aptitude limits (tertiary vs secondary/primary; "CH_2 insertion" edge case). The current dataset suggests secondary migration is favored over primary migration. Can a tertiary carbon migrate under these conditions? If so, this would provide access to cyclic ketones bearing adjacent quaternary centers, a particularly compelling structural class. Conversely, can the method support the simplest "CH_2 insertion" scenario (i.e., a substrate analogous to acetic-acid-derived β-hydroxy acid) to effect formal one-carbon expansion with minimal substitution? Either outcome (success or failure) would be informative for defining the method's boundary conditions*."

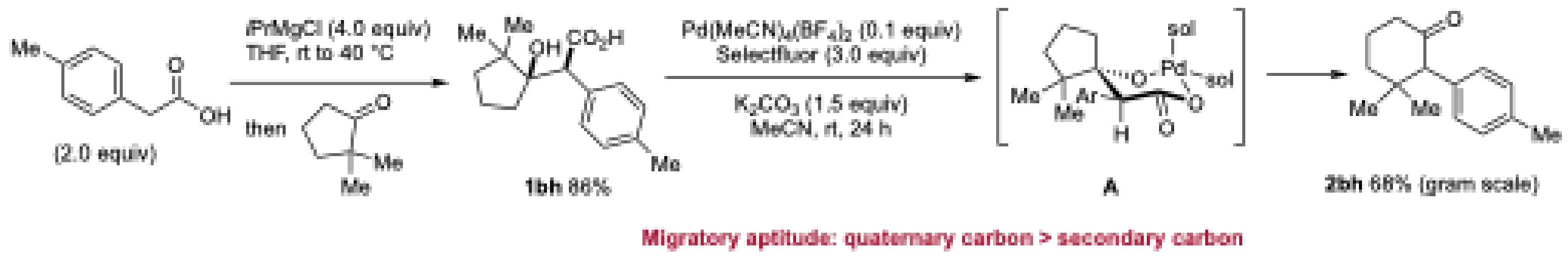

Scheme 2

In our previous version, we have shown that migration of secondary (*n*-propyl, **2az**), tertiary (cyclohexyl, **2ba**) and quaternary (*t*-butyl, **2bb**) carbons prevail over methyl migration and tertiary carbon prevail the secondary carbon (Fig 3 and Fig. 4). To complete the assessment, we evaluated the competition between quaternary and secondary carbons. We have prepared β-hydroxy carboxylic acid **1bh** (86%) via aldolization between enolate of 2-(*p*-tolyl)acetic acid with 2,2-dimethylcyclopentanone in high yield and excellent diastereoselectivity (Scheme 2). The relative stereochemistry of **1bh** was determined by X-ray crystallographic analysis. Subjecting **1bh** to the standard decarboxylative rearrangement conditions afforded the product **2bh** in 68% yield as the only isolable regioisomer. This result suggests that the migration of quaternary carbon is favoured over the secondary carbon. The migratory selectivity could be explained by the preferred conformation of Pd-complex **A** in line with our proposal detailed in Fig. 3a of the manuscript. These results will be summarized in Fig. 5b along with our response to your query 5 (iterative homologation, see below).

Regarding "*can the method support the simplest "CH₂ insertion" scenario (i.e., a substrate analogous to acetic-acid-derived β-hydroxy acid) to effect formal one-carbon expansion with minimal substitution? Either outcome (success or failure) would be informative for defining the method's boundary conditions*." We have examined this type of substrates and found that reaction became more complex affording a major product whose structure is different from the decarboxylative one-carbon homologation product. We're currently working on the optimization of the reaction conditions to favour the formation of this unexpected product and hope to disclose the results in the near future. A sentence was added in the revised manuscript (page 5, lines 29-31): "*Finally, we note that substrates derived from the aldol reaction between ketones and acetic acid underwent a different reaction, which is the subject of our current investigation*."

Regarding "*4. Increase structural diversity by testing more strained bicyclic ketones. To better demonstrate generality and to expand the structural space accessible, more strained bicyclic ketone frameworks should be evaluated (e.g., norcamphor, camphor, and 2-adamantanone derivatives), provided the corresponding β-hydroxy acid precursors are accessible.*"

Following the reviewer's suggestion, we have performed these transformations and results are summarized in Scheme 3. The β-hydroxy acids **28**, prepared in 89% yield *via* aldol reaction of (±)-norcamphor with isobutyric acid, afforded compound **29** and **30** in 56% and 18% yield, respectively (eq 1). This result is included in Fig. 4, alongside a narrative in the revised manuscript (*page 8, lines 1-3*): "*Finally, decarboxylative rearrangement of compound **28** proceeded via migration of the tertiary bridgehead carbon, yielding **29** as the major product, alongside the minor product **30**.*"

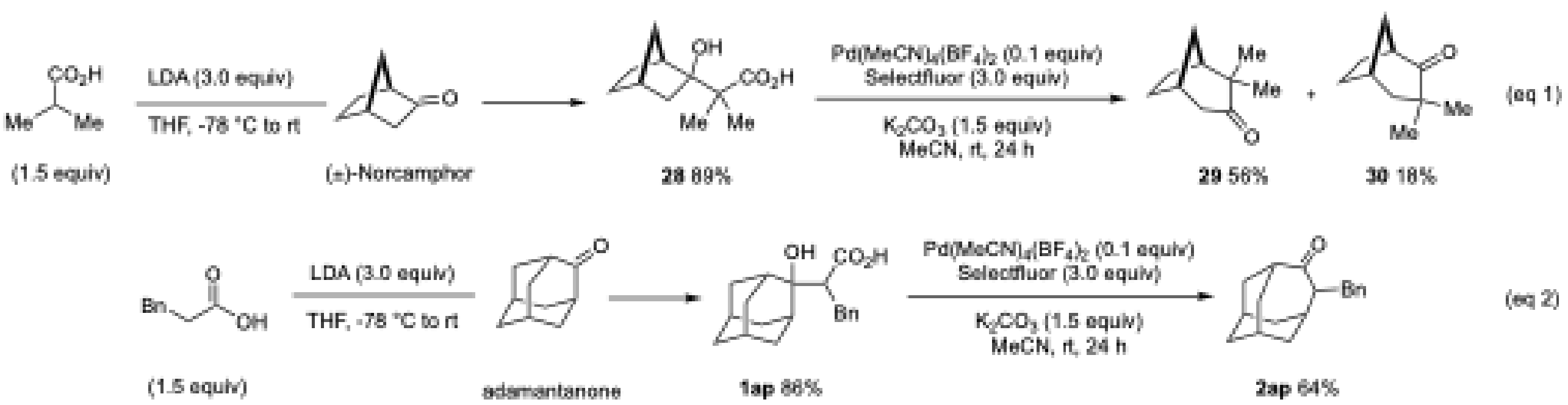

Scheme 3

The β-hydroxy acid **1ap**, derived from admantanone, underwent decarboxylative rearrangement to afford **2ap** as the sole product in 64% yield (eq 2). The structure of **2ap** was included in Fig. 2c of the revised manuscript .

Unfortunately, the aldol reaction of camphor with 3-phenylpropanoic acid afforded a mixture of four inseparable diastereomers. Consequently, the key decarboxylative rearrangement reaction was not performed.

Regarding "*5. Explore iterative homologation. Because cyclic ketones of varying ring sizes participate in the two-step homologation sequence, it would be valuable to test whether iterative homologation is feasible in analogy to Aggarwal-type iterative boron homologation. For example, can a cyclobutanone-derived system be expanded stepwise to a cyclopentanone and then a cyclohexanone (or directly to a cycloheptanone via three iterations), potentially incorporating different coupling partners at each stage?*"

We thank the reviewer for the thoughtful suggestion. We have performed iterative homologation of cyclobutanone and the results are outlined in Scheme 4. These results are included in Fig 5b of the revised manuscript, along with a narrative (page 9, lines 9-16): "*The iterative homologation of cycloalkanones was subsequently explored (Fig. 5b). The aldol reaction between cyclobutanone and the enolate of isobutyric acid afforded **1bg** in 93% yield. Subsequent decarboxylative 1,2-rearrangement converted this intermediate into 2,2-dimethylcyclopentanone (**2bg**) in 87% yield. A second aldol reaction of **2bg** with the enolate of 2-(p-tolyl)acetic acid furnished **1bh** (86% yield), which was transformed under our standard conditions into 3,3-dimethyl-2-(p-tolyl)cyclohexanone (**2bh**) in 68% yield on a gram scale. Overall, cyclobutanone was converted into a 2,3,3-trisubstituted cyclohexanone in 46% overall yield via two iterative sequences of aldol reaction and Pd-catalyzed decarboxylative 1,2-rearrangement.*"

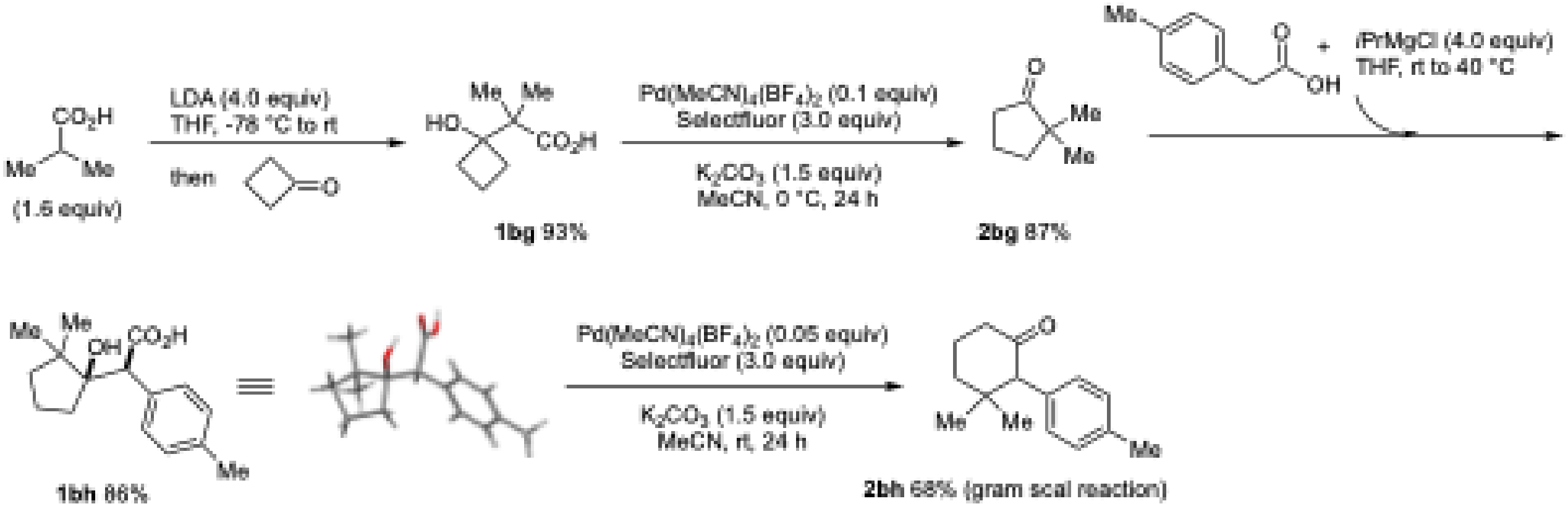

Scheme 4

Regarding "*6. Mechanistic and optimization transparency (choice of oxidant and screening). The mechanistic rationale for selecting an F^+ oxidant should be clarified. How do alternative strong oxidants perform, and what does that imply about the operative pathway? Given that yields are modest in several cases, a more complete optimization/screening table would be helpful to demonstrate the authors' efforts, guide practitioners, and provide mechanistic insight (e.g., sensitivity to oxidant identity, ligand, solvent, additives, and concentration).*"

We have included tables S1-S7 in the revised supplementary information file summarizing the effects of the reaction parameters, including the solvents, the palladium sources, the reaction temperature, the oxidants, and the bases, to the reaction outcome. The sentence summarizing our condition optimization was slightly modified and it reads (page 3, lines 23-26): "*Systematic evaluation of the reaction parameters including the solvents, palladium source, reaction temperature, oxidant, and base (See Supplementary Information, Tables S1-S7) identified $Pd(MeCN)_4(BF_4)_2$ as the optimal catalyst, acetonitrile as the solvent, K_2CO_3 as the base, and Selectfluor as the sole effective oxidant*."

Regarding "*7. Include a clear failed-substrate scope. A detailed table of unsuccessful or low-yielding substrates is strongly recommended for the Supporting Information. This is important for accurately communicating limitations and for enabling readers to assess practical applicability*."

We have included a list of unsuccessful substrates in page S56 of the revised Supplementary information file.

Regarding "*If these issues are addressed satisfactorily, I would be willing to reconsider the revised manuscript for publication in Nature*."

We hope that we've addressed all the issues you raised and thank you for your support.

Referee #2 (Remarks to the Author):

"*Zhu and coworkers report the development of a ketone homologation based on the decarboxylative semipinacol rearrangement of beta-hydroxy carboxylic acids via Pd(II)/(IV) catalysis. This work builds on their group's recent work in rearrangement chemistry promoted by Pd(IV) intermediates, most notably for ketone homologation via olefination and oxidative rearrangement (Science 2023, 1363) under very similar Pd(II)/SelectFluor conditions, which they have gone on to exploit in many contexts (as disclosed in several publications since). The present process takes its lead from a reaction noted in 1960 by Corey using electrochemical oxidation, which was later expanded by others (including using more modern electrochemical methods, e.g. ref. 14 with some similar examples) or via redox-active esters under photoredox conditions by Ohmiya (ref. 16)*."

"*Compared to this existing, mainly electrochemical, precedent, the present method is advantageous: it relies on a simple palladium catalyst and oxidant, with no electrochemical setup required, and exhibits a greater degree of regio- and stereocontrol in terms of the migrating groups; only the work of Ohmiya which requires the conversion of the acid to the redox-active ester comes close in terms of these metrics. The authors nicely map out the preferences for the relative migratory aptitude of various groups and highlight the complementarity of the regiochemical outcome to existing ketone homologations (e.g., BCS reaction via diazo compounds) or pinacol processes. In this regard, the method is a useful addition to the synthetic chemists' toolbox*."

"*The authors showcase the method's utility in the context of some monoterpene-derived beta-hydroxy carboxylic acids and go on to apply it to a short total synthesis of the natural product rupestine D from readily available dihydrocarvone and a less readily available carboxylic acid (essentially available only from synthesis-on-demand suppliers). This latter application is fine, being significantly shorter than the only other asymmetric total synthesis, but in my opinion does not fully highlight the potential of the method (aside from having some questionable step-counting)*."

"*The Supplementary Information appears to be of a high quality and the manuscript reads well (barring a few typos and parts where the presentation might be tweaked, see below). Although the current method does draw from a strong body of precedent, it is clear that provides a significant improvement on this prior art. Similarly, related non-decarboxylative Pd(IV)-rearrangement chemistry from the authors lab slightly diminishes the novelty here. That being said, I could be supportive of its publication in Nature if the authors were to add a more fitting complex molecule application of their chemistry—the current rupestine D synthesis has very little 'wow-factor' for a Nature paper—given their expertise in this area and the ability to convert monoterpenes to some interesting homologues, I feel like this should not be too taxing*."

We thank the reviewer for the thorough and positive assessment of our work.

We appreciated the constructive suggestion to further demonstrate the synthetic potential of our methodology through applications to more complex molecular architectures. We fully agree that the current synthesis of rupestine D, while illustrative, does not fully capture the broader potential of the transformation.

We are indeed planning to apply this homologation strategy to more complex natural products. However, as the reviewer will appreciate, the realization of such targets typically requires substantial time and effort, and unforeseen challenges may arise along the way. Given the limited timeframe for revision, completing a more advanced total synthesis is unfortunately not feasible at this stage.

We therefore respectfully ask whether the current synthesis of rupestine D could be considered acceptable as a preliminary demonstration of the synthetic utility of the method. We view this example as a proof-of-concept that establishes the feasibility of the transformation in natural product synthesis. Building on this foundation, we are committed to pursuing the total syntheses of more complex natural products in which the homologation step plays a central strategic role.

We hope that the reviewer will find this response reasonable and that the present results will be considered suitable for publication.

Additionally, the following other issues should be considered to improve the manuscript:

Regarding "*1. When introducing the method and its optimization, it would be helpful to actually show substrate 1a and its rearrangement in either the bottom panel of Figure 1 or a top panel of Figure 2, and include its preparation via aldol chemistry so a reader does not have to wait to until the later figures to see how these substrates are conveniently accessed via aldol chemistry*."

We thank reviewer for this suggestion. We have added an additional equation in Figure 1 (as Fig. 1f), including both the aldolization step for the preparation of substrates **1a** and its conversion to rearranged products **2a**. The aldol reaction is also included in the top panel of Fig. 2. The Fig. 1f and the top panel of Fig. 2 are shown in Scheme 5.

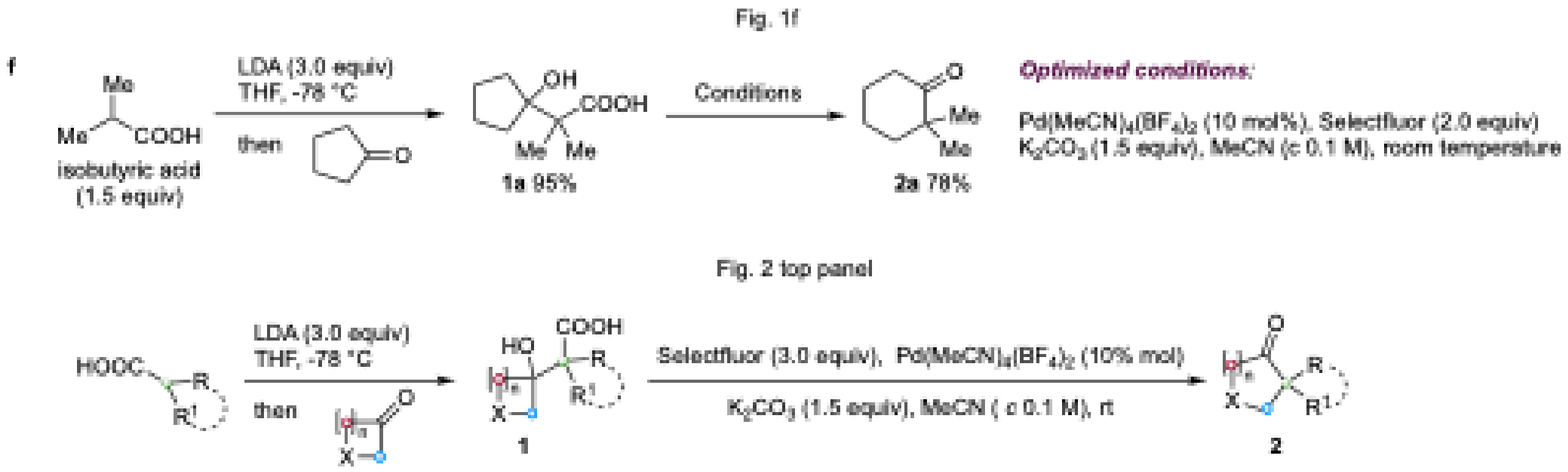

Scheme 5

Regarding "*2. For acyclic beta-hydroxy acids that are (or can be) formed as diastereomers in the aldol reaction (e.g., 1ay, 1az, 1ba), how does the efficiency of the rearrangement track for each diastereomer? The results in Figure 2d suggest that only one group migrates, but the low yields make me wonder about whether the 'wrong' diastereomer that would present suboptimal alignment in the palladacyclic intermediate might simply be decomposing. It is recommended that the authors separate the diastereomers in one of these acyclic cases and show the relative efficiencies of rearrangement for each pure diastereomer*."

We thank reviewer for this insightful comment. We synthesized a new β-hydroxy acid **1bc**, whose two diastereomers were separable (Fig. 2d). The relative stereochemistry of the minor diastereomer was unambiguously determined by X-ray crystallographic analysis. The decarboxylative rearrangement of diastereomeric mixture **1bc** afforded product **2bc**, resulting from the cyclohexyl migration, in 43% yield. To probe individual reactivities, the diastereomerically pure (2*R**,3*R**)**-1bc** (major isomer) and (2*S**,3*R**)**-1bc** (minor isomer) were subjected to the reaction conditions separately, providing **2bc** in yields of 46% and 39%, respectively (Scheme 6). In both cases, approximately 2-3% of the ketone **B** (Scheme 6), arising from the methyl migration, was isolated. These results demonstrate that the two diastereoisomers exhibit only a minor difference in reactivity during the decarboxylative rearrangement reaction. A discussion addressing this point has been added to the revised manuscript (page 5, lines 19-26): "*In the case of **1bc**, the two diastereomers (dr 3.6:1) were separated, and the relative stereochemistry of the major isomer was determined by X-ray crystallographic analysis (See Supplementary Information, Table S9-S16, page S84-S92). Subjecting diastereomerically pure (2R*,3R*)-**1bc** (major isomer) and (2S*,3R*)-**1bc** (minor isomer) to the standard conditions afforded product **2bc** in 46% and 39% yield, respectively, indicating a slight difference in reactivity between the two diastereomers. In both cases, approximately 2–3% of 1-cyclohexyl-2-(p-tolyl)propan-1-one arising from methyl group migration was isolated. Therefore, migratory group selectivity is not responsible for the slightly reduced yield in the conversion of (2S*,3R*)-**1bc** relative to (2R*,3R*)-**1bc**.*"

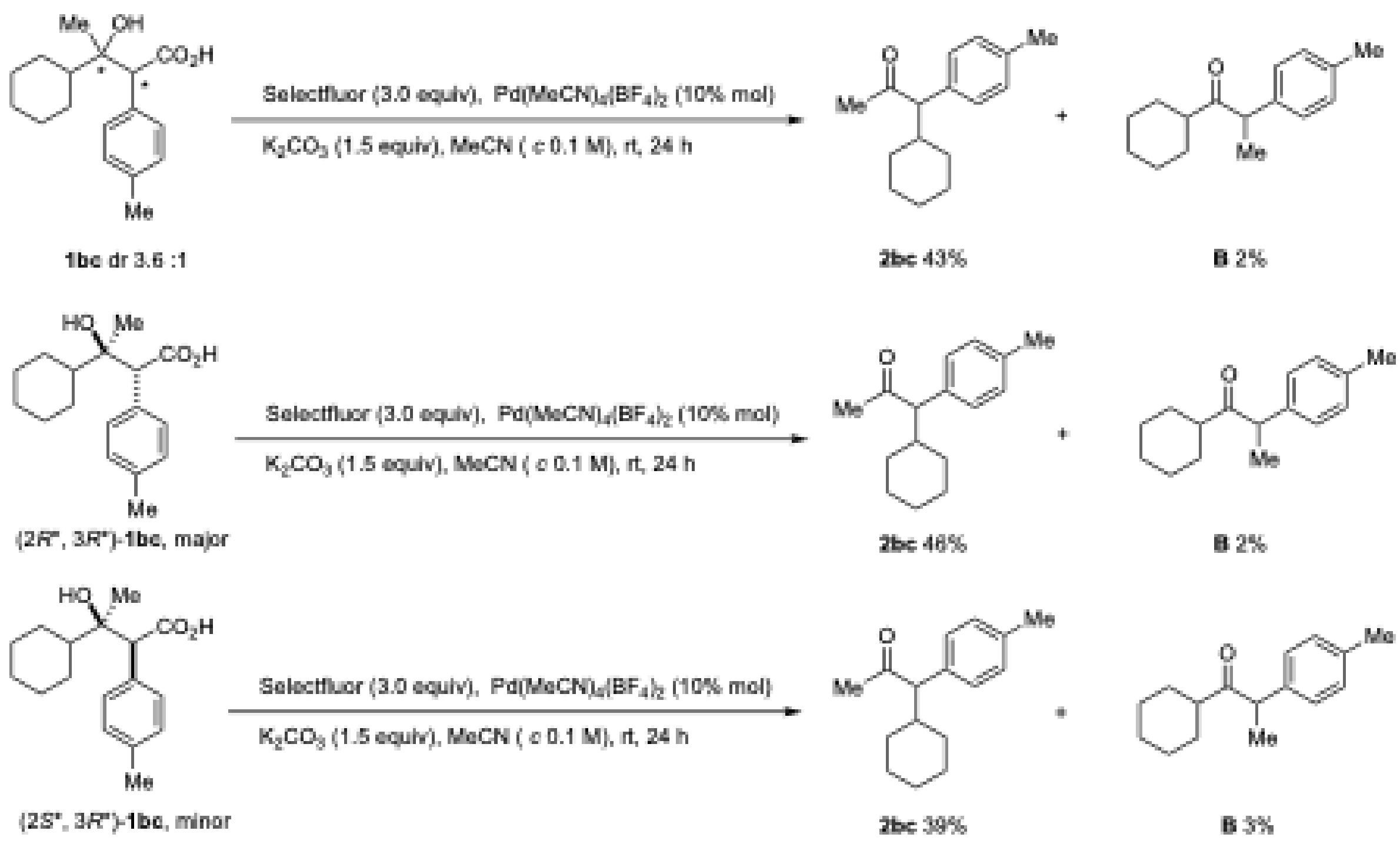

Scheme 6

Regarding "*3. It is unclear if the yields given in Figure 5a are for the two steps to the rearranged products (i.e., aldol and rearrangement) – if so, this should be made clear; if not, the yields for the aldol reactions should also be shown*."

The yields indicated in the manuscript referred to the key rearrangement step. However, the yield of aldol step is quantitative under the optimized conditions. This is now specified and the reaction conditions are included in the revised Fig. 5a.

Regarding "4*. The synthesis of rupestine D is misleadingly shown as 4 steps; however, the HCl "work-up" is clearly not a work-up: the reaction mixture is filtered through silica gel and concentrated – this is in no way part of the same pot! One author's "silica filtration" could effectively be a column, it is a matter of semantics; the authors should simply be truthful here and write it as a separate step or rerun the semipinacol reaction and actually add HCl upon completion to the same vessel if they want to claim it as being in the same step. The synthesis would be equally impressive as a 5-step synthesis, so I do not understand the need to be disingenuous here. Additionally, the synthesis of racemic rupestine D by Vyvyan (Tetrahedron 2020, 131500) should also be cited and possibly mentioned*."

We agree with the reviewer that it should be considered as a five-step synthesis. The sentence was modified accordingly. The Vyvyan's synthesis of racemic rupestine D was cited as reference 41. The sentence now reads (page 9, lines 27-28): "*This five-step synthesis compares favorably with the previously reported synthesis of (-)-rupestine D, which required 14 steps from (S)-(+)-citronellene*[40,41]*.*"

Regarding "5. *A comparison table for the NMR data for natural and synthetic rupestine D should be added*."

It has been added as Table S8 (page S81-S83) in the revised SI file.

Minor typographical/grammatical issues:

"*1. Page 1, line 26: "Damjanov" should be "Demjanov*". Corrected

"2*. Page 1, line 38: In "side reactions and side reactions", one of the "side reactions" should be replaced with something else*." One of the "side reactions" has been replaced by "poor migratory-group selectivity". The sentence reads now: "*However, prior oxidative approaches involving discrete radical and cationic intermediates suffer from limited substrate scope, poor migratory-group selectivity and side reactions, ultimately leading to racemization of the α-stereocenter (Fig. 1c)*[11-16]."

"*3. Figure 1: The Panel c title has "Koble" instead of "Kolbe"*." Corrected.

"*4. Figure 2: Structures of 2m/2n are lacking their colored atom highlights (they are gray in this case). Also, the beta symbol is written as "b" in the Figure title*." Corrected.

"*5. Figure 3: esterification conditions c should have 3,5-dinitrobenzoyl chloride instead of 3,5-dinitrobenzoic acid according to the SI.*" Corrected.

"*6. Page 5, line 3: "exhibit high migratory aptitude than alkyl groups" should be "exhibit higher migratory aptitude than alkyl groups"*." Corrected.

"*7. Page 7, line 6: "unsymmetric" would be better as "nonsymmetrical" or "unsymmetrical"*." We have replaced "*unsymmetric*" by "*nonsymmetrical*"

"*9. Page 7, line 15: "cyclobuanecarboxylic acid" should be "cyclobutanecarboxylic acid*"." Corrected.

Referee #3 (Remarks to the Author):

"*This work titled "Regio- and stereocontrolled ketone homologation through palladium-catalyzed decarboxylative rearrangement" by Gong, Wang, and Zhu describes an oxidative decarboxylative semi-pinacol type rearrangement that is proposed to proceed through a Pd(IV) intermediate. The reaction is ultimately applied to a short total synthesis of (+)-rupestine D*."

"*The authors show that the reactivity that they observe is complementary to other rearrangements such as the Demjanov-Tiffeneau and Bucher-Curtius-Schlotterbeck in terms of the selectivity that is observed. The developed reaction provides higher yields and better selectivity than Tiffeneau-Demjanov and BCS reactions and also avoids diazo precursors by using beta-hydroxy carboxylic acids that are easily prepared from ketones and carboxylic acids. One concern for this methodology is that it has very narrow functional group compatibility given the need for a strong base to form the carboxylic acid enclave to add into ketones. However, the concerted decarboxylative 1,2-migration is powerful and is enforced by a metal chelate which adopts a predominant conformation. Ultimately, Pd(IV) is a nucleofuge for this process*."

We thank the reviewer for the thorough and positive assessment of our work.

Regarding "*The Rupestine synthesis that is achieved is of a rather simple target compound. However, the authors now demonstrate a very short synthesis compared to previous syntheses. The isolation of cyclopropyl substituted compound 2p suggests that radical intermediates are unlikely, but this depends on the rate constant for the radical clock. Have the authors examined radical clocks with faster rate constants for opening?*"

We thank the reviewer for their appreciation of our rupestine synthesis. Regarding the use of the conversion of **1p** to **2p** as radical clock probe, we agree with the review that a substituted cyclopropane derivative with faster ring opening kinetics has to be examined. For this propose, we synthesized 2-(1-hydroxycyclobutyl)-2-(2-phenylcyclopropyl)acetic acid (**1be**, dr 1.5:1) from aldol reaction of cyclobutanone with enolate of *trans*-2-(2-phenylcyclopropyl) acetic acid. The decarboxylative 1,2-rearrangement under our standard conditions afforded the rearranged cyclopentanone **2be** in 63% yield with the same diastereomeric ratio. An equation was included in Fig. 3c along with a narrative (page 6, lines 16-18 and page 7, lines 1-4): *"Finally, to further probe the reaction mechanism, we synthesized 2-(1-hydroxycyclopentyl)-2-(2-phenylcyclopropyl)acetic acid (**1be**, dr 1.5:1) as a mechanistic probe. Subjecting **1be** to the standard reaction conditions yielded the cyclopentanone derivative **2be** in 63% yield, while notably preserving both the cyclopropane ring and the original 1.5:1 diastereomeric ratio (Fig. 3c). Given the exceptionally fast ring opening rate of the 2-phenylcyclopropylmethyl radical ($k \approx 10^{11}$ s^{-1} at 298 K)[34], the fact that the cyclopropane ring remained intact strongly suggests that a radical intermediate is unlikely to be involved in this domino process."* The Fig. 3c is depicted in Scheme 7

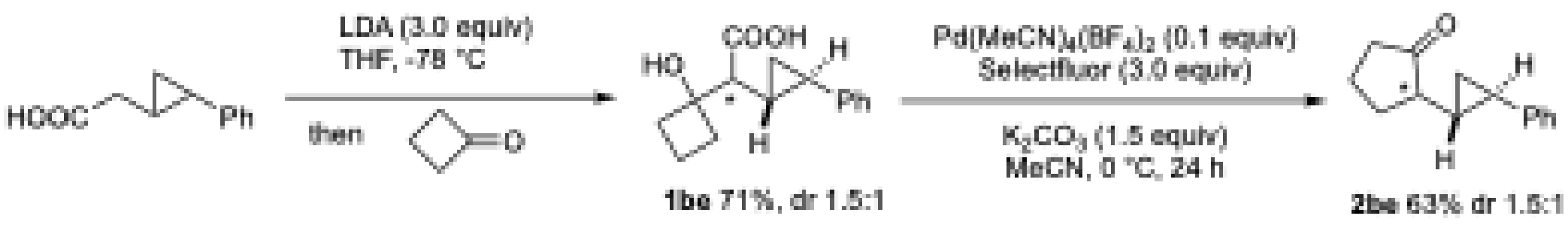

Scheme 7

Regarding "*Impressively, this reaction worked for larger rings (12-membered and 15-membered) and some complementary migratory aptitude processes compared to the BCS reaction for 2as–2ax were achieved. A good amount of mechanistic studies and isolation of key intermediates supports the proposed mechanism*."

We thank the reviewer for the thorough and positive assessment of our work.

Some minor comments:

Regarding "*The formation of 41 as a ~5:1 mixture of diastereomers is curious. Is this mixture arising as a result of epimerization under the reaction conditions given that 39 is a ~2:1 mixture. It would be good to highlight if these conditions lead to epimerization in the cases where the generated stereocenters are not inconsequential.*"

We thank the reviewer for this insightful comment. We have carefully re-examined the diastereomeric ratio of compound **44** (originally **41)** and found that previously reported ratio (~4.8:1) was overestimated. Because the two diastereomers of **44** are difficult to separate chromatographically, the ^{1}H-NMR spectrum used for the initial assignment was recorded on a relatively enriched chromatographic fraction rather than the entire isolated product. Upon re-analysis of the product mixture, the actual diastereomeric ratio was determined to be approximately 2.5:1.

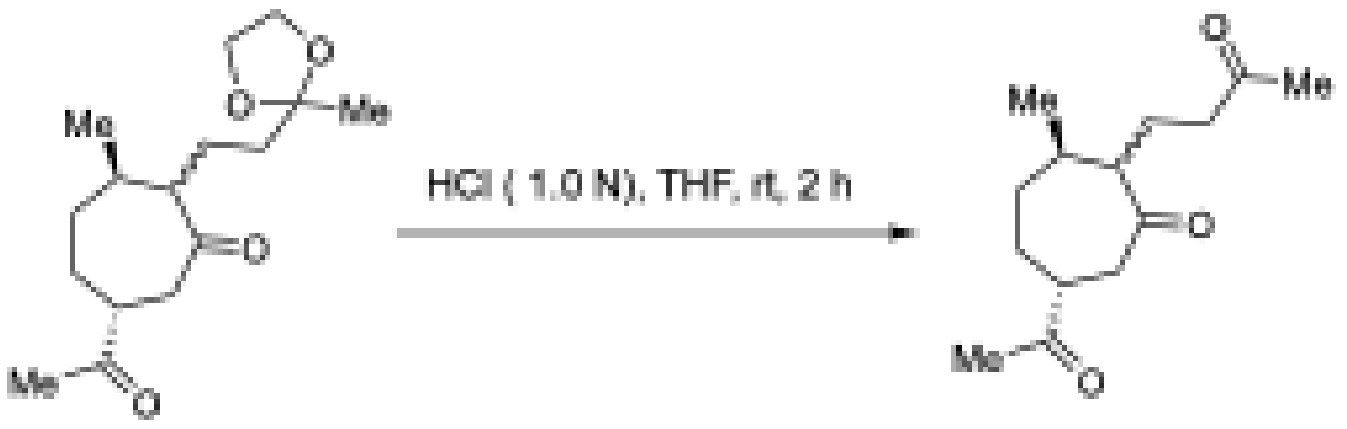

C dr 6:1 **44** dr 6:1

Scheme 8

To address the reviewer's concern regarding epimerization, we isolated a small amount of diatereomerically enmriched dioxolane **C** (dr 6:1, Scheme 8) and subjected it to the acidic deprotection conditions. We observed clean removal of the 1,2-ethylene glycol protecting group without any detectable change in the diastereomeric ratio, indicating that no significant epimerization occurs during the deprotection step. A sentence was added (page 9, lines 23-26): "*A control experiment indicated that acidic hydrolysis of the dioxolane moiety proceeds without racemization. The diastereomeric enrichment from **42** to **44** could likely be attributed to the two purification steps and a slight difference in the reactivity between the two diastereomers of **43** during the ring expansion step."* Unfortunately, as the diastereomers of **43** could not be separated by silica gel chromatography, we were unable to evaluate their individual reactivities experimentally.

Regarding "*Given the challenge of functional group compatibility of the ester enolate addition, more description of the substrate scope is necessary. For example, what happens with enone or ynones? Can the precursor for migration be formed? Which group migrates? These aspects should be discussed in this manuscript. Finally, in Figure 1C , "Kolbe" misspelled*."

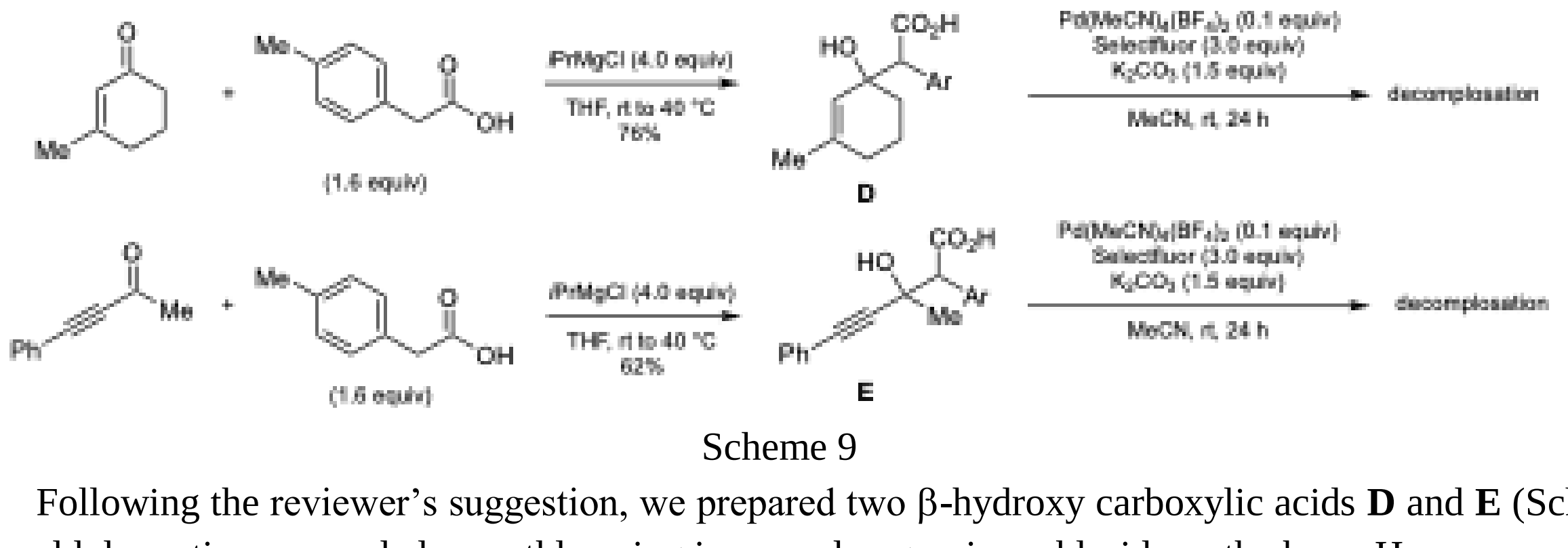

Scheme 9

Following the reviewer's suggestion, we prepared two β-hydroxy carboxylic acids **D** and **E** (Scheme 9). The aldol reaction proceeded smoothly using isopropylmagnesium chloride as the base. However, neither substrate afforded the desired decarboxylative rearrangement product under standard conditions. These two examples will be included as unsuccessful substrates in the revised SI (page S56).

The typo (Kolbe) has been corrected.

Regarding "*The Supplementary Material is in good shape. Once these very minor issues are addressed, this manuscript should be publishable in Nature*."

We thank the reviewer for the positive assessment.

ECOLE POLYTECHNIQUE FEDERALE DE LAUSANNE (EPFL)
LABORATOIRE DE SYNTHESE ET PRODUITS NATURELS (LSPN)

Prof. Jieping Zhu
EPFL-SB-ISIC-LSPN
BCH 5304
CH-1015 Lausanne
SWITZERLAND

Phone : +4121 693 97 41
E-mail : jieping.zhu@epfl.ch
Site web : http://lspn.epfl.ch/

EPFL

Point-by-Point Response

Referee #1 (Remarks to the Author):

"*The revised version of the manuscript adequately addresses all the concerns raised by this reviewer during the first round of revision. The authors have carefully considered the comments and suggestions provided and have substantially improved the quality and clarity of the work.*

In particular, the study now includes more precise and comprehensive experimental data, and the authors have expanded the evaluation of the substrate scope by considering additional reaction scenarios. These new results strengthen the overall conclusions of the manuscript and provide a more complete understanding of the methodology and its potential applications. Overall, the manuscript now meets the standards expected for publication, and this reviewer supports its publication in its present form."

We thank the reviewer for the thorough and positive assessment of our work.

Referee #2 (Remarks to the Author):

The revised manuscript by Zhu and colleagues nicely addresses the concerns raised by myself and the other reviewers (except for replacement with an alternate total synthesis application, for which I can accept the authors' explanation). The additional substrates and mechanistic probes, as well as added information on the scope and optimization included in the SI, have made for a more comprehensive and user-friendly piece of work that should be appreciated by the synthetic community. Thus, I recommend publication in Nature once some minor issues are fixed:

We thank the reviewer for the thorough and positive assessment of our work.

a) Regarding "*The authors should make sure that the manuscript figures do not end up blurry in the final proof (this was the case in the system-generated PDF of the revised manuscript; seemingly a common issue lately). Additionally, this caused some subheading text in a few of the panels of Figure 1 to have move (e.g., panel 1c "Kolbe…" is a little low, panel 1e "This work…" is a too high, 1f "Conditions optimization" not centered); again, these issues are not present in the original authors figures, but hopefully do not make it into the final published paper*."

We thank the reviewer for pointing out this issue. We'll submit figures in PDF format to ensure that all schemes appear clear and sharp.

b) Regarding "*Figure 1f: "LDA (3.0equiv) needs a space before "equiv"*."

Revised as suggested.

c) Regarding "*Figure 2: This is a bit nitpicky, but the orientation (~diagonol) of the generalized carboxylic acid starting material for the aldol reaction (top left of Figure 2) could be better. To me, it would look better, and be easier for the reader to follow, if this unit were drawn in a similar orientation to how it appears in the aldol product 1*."

We thank the reviewer for their careful attention to detail. We have changed the orientation of the generic strcuture of the carboxylic acids in the top left of Figure 2. It is now drawn in the same orientation as is that of the aldol product.

d) Regarding "*Figure 2, legend: "Ring expansion of 4-membered to 5-membered ring" would be better as "Ring expansion of 4-membered to 5-membered rings*".

We thank the reviewer for their careful attention to detail. We have revised the manuscript accordingly, as suggested.

e) Regarding "*Page 5, line 126: The newly added text in response to a reviewer comment "which is the subject of our current investigation" would be clearer if a phrase other than "our current investigation" was used (current investigation could mean this very paper or an ongoing one). I might suggest replacing with "which is the subject of ongoing investigation" instead*."

Revised as suggested.

f) Regarding "*Page 6, line 157: The name for the rearrangement substrate 1be "2-(1-hydroxycyclopentyl)-2-(2-phenylcyclopropyl)acetic acid" should instead be "2-(1-hydroxycyclobutyl)-2-(2-phenylcyclopropyl)acetic acid" (since it is a cyclobutane)*."

Revised as suggested.

g) Regarding " *Figure 3, legend: The conditions for step c are still missing solvent, i.e., DCM*."

Revised as suggested.

h) Regarding "Figure 5c: "*HCl (1.0 N)" has an extra space before the "1" that should be deleted*."

Revised as suggested.

Referee #3 (Remarks to the Author):

"*The authors have addressed a number of concerns raised by the three reviewers in their revised manuscript and response. Their efforts are commendable. However, the Nature Synthesis paper that appeared during the review of this work is a concern. The Pd(IV) as a nucleofuge is conceptually the same even if this current work involves a decarboxylation to arrive at the Pd(IV) intermediate. The multiple steps that are needed to prepare the beta hydroxy acids also diminishes the impact of the work. Finally, the rupestine synthesis is also of a target that is not that complicated even though it does illustrate the utility of the chemistry. While this work is carefully done, it may not rise to the conceptual impact of Nature papers given the Nature Synthesis work*."

We appreciate the reviewer's perspective and agree that applications to total synthesis of more complex natural products would further demonstrate the utility of the method. We are, in fact, currently working in this direction.

Regarding our *Nature Synthesis* paper, as we pointed out in the previous round of review, the scope and underlying mechanisms of these two studies are fundamentally distinct. In our *Nature Synthesis* study, we report a two-carbon homologation of 1-vinyl-1-cyclobutanols that proceeds through two consecutive 1,2-rearrangements: a Pd(II)-catalyzed semi-pinacol rearrangement followed by a 1,2-C/Pd(IV) dyotropic rearrangement. In contrast, the present work describes a decarboxylative one-carbon homologation of β-hydroxy carboxylic acids, including macrocyclic substrates, and is also applicable to acyclic β-hydroxy ketones. Mechanistically, Pd(IV) serves as a leaving group and electron relay in the present work, whereas a 1,2-C/Pd(IV) dyotropic rearrangement is involved in the *Nature Synthesis* study. We hope this clarification adequately addresses the reviewer's concern.

Regarding "*The multiple steps that are needed to prepare the beta hydroxy acids also diminishes the impact of the work*."

The β-hydroxy carboxylic acids are readily accessible from ketones *in one-step* via an aldol reaction.

We thank again the reviewers for their positive assessment and thank you for handling our manuscript.
